# Automated Assembly of Starch and Glycogen Polysaccharides

**DOI:** 10.1021/jacs.1c02188

**Published:** 2021-06-11

**Authors:** Yuntao Zhu, Martina Delbianco, Peter H. Seeberger

**Affiliations:** † Max Planck Institute for Colloids and Interfaces, Am Mühlenberg 1, 14476 Potsdam, Germany; ‡ Institute for Chemistry and Biochemistry, Freie Universität Berlin, Arnimallee 22, 14195 Berlin, Germany

## Abstract

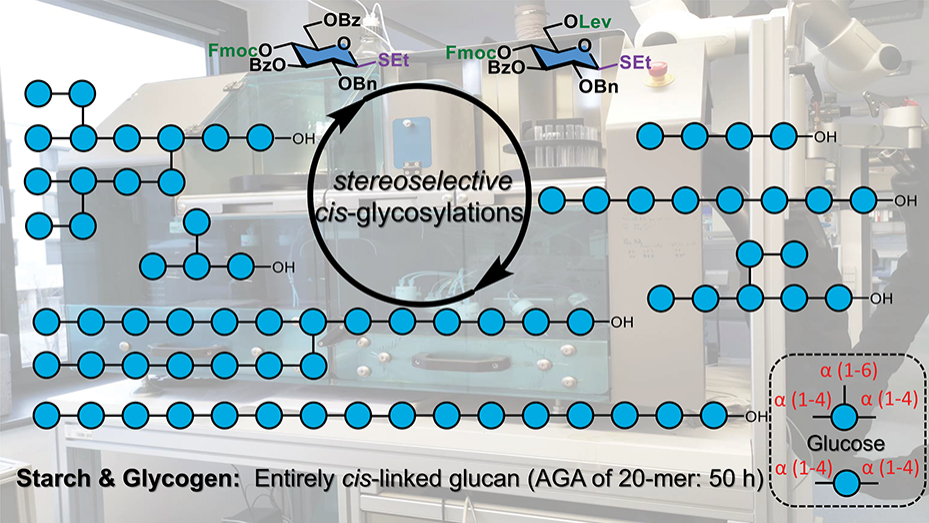

Polysaccharides are Nature’s most abundant biomaterials essential for plant cell wall construction and energy storage. Seemingly minor structural differences result in entirely different functions: cellulose, a β (1–4) linked glucose polymer, forms fibrils that can support large trees, while amylose, an α (1–4) linked glucose polymer forms soft hollow fibers used for energy storage. A detailed understanding of polysaccharide structures requires pure materials that cannot be isolated from natural sources. Automated Glycan Assembly provides quick access to *trans*-linked glycans analogues of cellulose, but the stereoselective installation of multiple *cis*-glycosidic linkages present in amylose has not been possible to date. Here, we identify thioglycoside building blocks with different protecting group patterns that, in concert with temperature and solvent control, achieve excellent stereoselectivity during the synthesis of linear and branched α-glucan polymers with up to 20 *cis*-glycosidic linkages. The molecules prepared with the new method will serve as probes to understand the biosynthesis and the structure of α-glucans.

## Introduction

**Figure 1:**
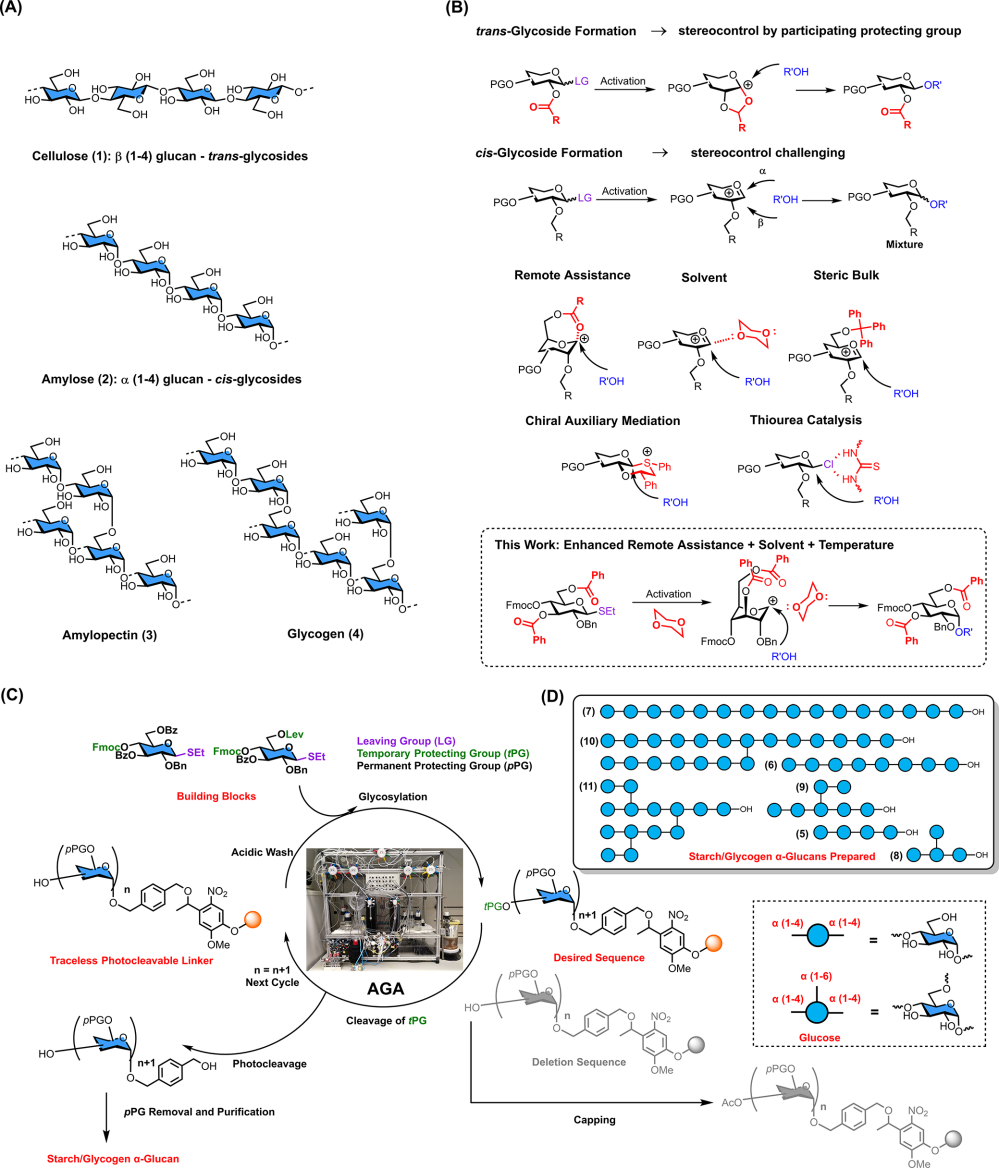
Chemical structure, synthetic strategies, and Automated Glycan Assembly of starch and glycogen. (A) Chemical structure of β (1–4) linked polyglucoside cellulose (**1**), linear α (1–4) linked polyglucoside amylose (**2**), α (1–4) linked polyglucoside with α (1–6) branched α (1–4) linked polyglucoside side chains, amylopectin (**3**), and highly branched glycogen (**4**). (B) Participating neighboring protecting group ensures *trans*-glycoside construction, stereocontrolled *cis*-glycosidic bond formation is challenging: uncontrolled glycosylations result in anomeric mixtures. Typical solutions: Solvent assistance,^19^ bulky protecting group assistance,^50^ ester remote assistance,^26^ chiral-auxiliary-mediation,^18^ macrocyclic bis-thiourea catalysis.^27^ Strategy developed in this work for α (1–4) glucan AGA with simple chemistry. (C) Schematic view of Automated Glycan Assembly and the building blocks used to prepare *cis*-linked polyglucosides. (D) Synthetic amylose, and amylopectin oligo- and polysaccharides (**5**–**11**) prepared by AGA.

Polysaccharides are built from monosaccharide units connected through glycosidic bonds with different regio- and stereochemistry. Glucose-based polymers are the most abundant biomaterials on earth, but differ greatly in structure and function depending on whether they are β (1–4) linked as in cellulose, or α (1–4) linked as in starch and glycogen (Figure 1A). Cellulose (**1**) is a linear polymer that unlike starch does not coil or branch, but adopts an extended and stiff rodlike conformation.^1,2^ Hydrogen bonding between parallel chains form microfibrils with high tensile strength that are part of the polysaccharide matrix in the cell wall.^1−5^ Starch is a mixture of about one-quarter strictly linear amylose (**2**) and about three-quarters α (1–6) branched amylopectin (**3**).^6,7^ Glycogen (**4**), the polymer that stores energy in the form of glucose in animals, is highly branched.^8^ Amylose exists in a disordered amorphous conformation or can adopt two different helical conformations that can host other molecules.^6,7,9−12^ Amylopectin is linear and does not crystallize as well as the long linear chains of amylose.^6,7,10,13^

The molecular level understanding of cellulose structure formation has benefitted from synthetic glycans and single molecule glycan imaging.^14,15^ In order to understand the factors contributing to the structure of amylose, amylopectin, and glycogen, pure oligo- and polysaccharides containing exclusively *cis*-glycosidic linkages are needed. The stereocontrolled synthesis of linear or branched oligosaccharides has proven extremely challenging since glycosidic bond formation cannot rely on participating neighboring protecting groups as for *trans*-glycoside construction (Figure 1B). A host of methods to prepare *cis*-glycosides involving remote assistance, the use of chiral auxiliaries, or catalysts as well as substrate control combined with solvent and temperature effects has been developed (Figure 1B).^16−34^ Still, the synthesis of oligosaccharides with multiple *cis*-glycosidic linkages has been a major challenge to manual syntheses that relied on block couplings to prepare amylose and amylopectin sequences as long as 10-mers.^7,35,36^

Automated Glycan Assembly (AGA) has been used to prepare polysaccharides as large as 151-mers,^37^ a variety of biologically important complex glycans containing different monomers as well as natural and unnatural carbohydrate materials.^5,38−45^ AGA requires high yielding and completely selective glycosylation reactions as none of the intermediates, but only the products of the synthesis are purified.^46^ The separation of full-length oligosaccharide side-products that differ from the desired product only in the stereochemistry of one linkage is very difficult if not impossible. Therefore, very few oligosaccharides containing *cis*-glycosidic linkages have been prepared by AGA, exploiting building blocks with remote ester groups in the 3- and 6-positions.^47−49^

Here, we describe a simple strategy based on thioglycoside building blocks with protecting group patterns that, together with temperature and solvent control, allows for excellent *cis*-selectivity during AGA of α-glucans (Figure 1C). A series of glucans resembling linear or branched natural starch structures with an α (1–4) backbone and α (1–6) side chains were prepared to illustrate the power of the approach (Figure 1D). Branched 20-mer α-glucan **10**, the largest full *cis*-linked branched glycan ever made, was obtained within 50 h AGA time.

## Results and Discussion

### Building Block Design

Thioglycosides with temporary fluorenylmethoxycarbonyl (Fmoc) protecting groups have proven as useful building blocks for AGA (Table 1A).^40^ Thus, Fmoc protection to mask the C4 hydroxyl group required for chain elongation was selected. The orientation of C3 and C6 functional groups on the glucopyranose ring makes them suitable remote assistant groups to block the β-face during glycosylation (Figure 1B). Remote assistance of a 6-acetyl (Ac) protecting group is not sufficient to prepare α (1–4) glucan oligosaccharides with high *cis*-selectivity and good isolated yield.^49^ In search for building blocks that maximize *cis*-selectivity and yield during AGA, a comprehensive evaluation of different C3 and C6 ester groups and their influence on α (1–4) glucan formation was undertaken (see Table S1). An electron rich, nonparticipating benzyl (Bn) ether group was selected as a permanent protecting group on the C2 hydroxyl group to allow for *cis*-glycoside formation with good reactivity. Thioglycosides **13**–**25** carry a C3 benzyl ether as well as different esters at C6, while building blocks **26**–**30** carry ester protection groups both on C3 and C6 (see Table 1A). Additional building blocks with ester at C3 but benzyl protection at C6-OH were also shown (Table S1, compound **110**–**112**) as comparison. Thioglycosides **12** and **31**–**32**, substituted with a fluorine or an ether at C6, respectively, were prepared for comparison. All building blocks were prepared from a single precursor using stratified methods in order to minimize experimental work (see Figure S1).

**Table 1:**
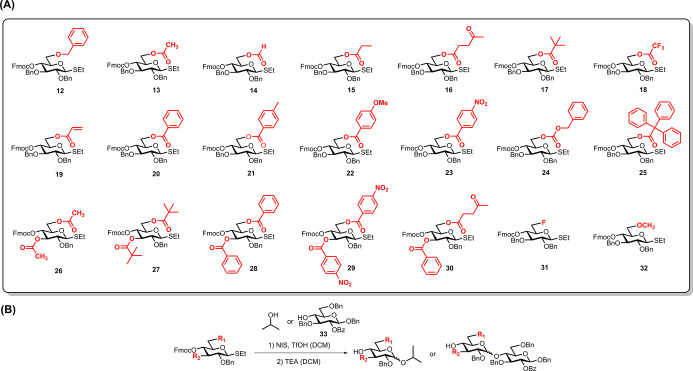
Glucose Building Blocks and Solution Phase Glycosylations^a^

entry	building block	acceptor	ratio (α/β)	yield (α + β)	entry	building block	acceptor	ratio (α/β)	yield (α + β)
1	**12**	*i*-PrOH	**1:2.9**	93%	15	**12**	**33**	1.5:1	83%
2	**13**	*i*-PrOH	**2.3:1**	95%	16	**13**	**33**	3.8:1	75%
3	**17**	*i*-PrOH	2.5:1	92%	17	**17**	**33**	5.5:1	69%
4	**20**	*i*-PrOH	1.7:1	93%	18	**20**	**33**	6.8:1	72%
5	**22**	*i*-PrOH	2.2:1	90%	19	**22**	**33**	5.6:1	68%
6	**23**	*i*-PrOH	3.5:1	89%	20	**23**	**33**	6.2:1	67%
7	**25**	*i*-PrOH	1.9:1	90%	21	**25**	**33**	7.5:1	44%
8	**26**	*i*-PrOH	**4.5:1**	84%	22	**26**	**33**	9.8:1	78%
9	**27**	*i*-PrOH	**6.4:1**	88%	23	**27**	**33**	>10:1	62%
10	**28**	*i*-PrOH	**4.8:1**	87%	24	**28**	**33**	**>10:1**	**83%**
11	**29**	*i*-PrOH	**7.1:1**	74%	25	**29**	**33**	>10:1	64%
12	**30**	*i*-PrOH	2.4:1	76%	26	**30**	**33**	**>10:1**	**80%**
13	**31**	*i*-PrOH	1:1.1	96%	27	**31**	**33**	3.9:1	75%
14	**32**	*i*-PrOH	1:2.6	94%	28	**32**	**33**	2.7:1	81%

a (A) The glucose thioglycoside building blocks were prepared from a common precursor as described in Figure S1. (B) Glycosylations using isopropanol and monosaccharide as nucleophiles. The ratio of α/β anomers were quantified by NMR, yield represented the isolated yield of α- and β-anomers (complete results in Table S1).

### Solution-Phase Glycosylation

The differentially protected thioglycoside building blocks were used to glycosylate different nucleophiles to screen the influence of protecting groups on α-selectivity (Table 1B and Table S1). Initially, all building blocks were activated with *N*-iodosuccinimide (NIS)–triflic acid (TfOH) in DCM for 5 min at −15 °C followed by 60 min at 0 °C to glycosylate isopropanol (*i*-PrOH) as a model for a secondary hydroxyl group. Glycosylations involving **12**–**25** (entries 1–7 in Table 1B, and entries 1–14 in Table S1) as well as **31**–**32** (entries 13 and 14) suggest a relationship between electronic and steric properties of the C6 protecting groups and the α/β ratio of the glycoside products. An electron withdrawing group (EWG) on C6 improves *cis*-selectivity. Thioglycosides **12** (entry 1) and **32** (entry 14) that carry nonparticipating electron donating groups (EDGs), are less α-selective than building blocks **13**–**25** (entries 2–7, and entries 2–14 in Table S1) and **31** (entry 13). Esterification on 6-OH(entries 2–7, and entries 2–14 in Table S1) resulted in higher stereoselectivity than deoxyfluorination (entry 13), even though ester groups are less electron withdrawing than fluorine. These results further supported the ester remote assistance hypothesis during glycosylation.^16,26,49^ The argument whether the ester group really stabilized the intermediate by forming a covalent glycosyl dioxolenium ion during glycosylations has not been settled, although this intermediate can be detected under extreme conditions.^51−54^ We observed that 6-OH esterification alone does not ensure excellent selectivity during α (1–4) glycosylations. Selectivities for the glycosylation of isopropanol ranged from 1.5–3:1 (α/β) as changes in the electronic and steric properties of ester groups had little effect (entries 2–7, and entries 2–14 in Table S1). Additional experiments employing 3-OH esterified, 6-OH benzylated building blocks (entries 22–24 in Table S1) agreed with our previous findings that C3 esters are an EWG that can provide remote assistance, although usually less helpful than C6 esters.^49^ To increase the selectivity further, 3,6-disubstituted building blocks **26**–**30** were tested (entries 8–12). All of these compounds improved *cis*-glycoside formation.

With initial information concerning the selectivity of different thioglycoside building blocks in hand, we next investigated the influence of the reaction temperature on the product ratio (Table S2). Reactions at a constant temperature revealed that glycosylations at higher temperatures gave better stereoselectivity, as previously reported.^28^ However, activation temperatures above 0 °C may result in lower yields (Table S2, entry 3 vs 4). Solvent is another important factor to control the stereochemistry during glycosylation. Ethers are the most common alpha directing solvents.^16,19,28,49^ We tested our system in several different ethers (Table S3, entries 1–5). Although most of the selected ethers were helpful for producing *cis*-glycoside, the poor solubility of our building blocks and NIS makes these solvents not compatible with AGA. Dioxane is a good choice for use in the AGA system, but a balance between the solubility of the activator and the melting point of the reaction mixture needs to be maintained. For AGA we dissolved the activator in a DCM–dioxane mixture (2:1), and mixed it with the same volume of building block DCM solution (DCM–dioxane 5:1 during reaction).^39^ We tested several typical building blocks in a 5:1 DCM–dioxane mixture and proved that this condition can be used to increase the *cis*-product formation(Table S3, entries 1 and 6–14).

Next, the influence of the nucleophile on the outcome of the glycosylation was explored, by analyzing the glycosylation of the secondary hydroxyl group at the C4 position of monosaccharide **33** (entries 15–28).^55^ EWG and ester remote assistance contribute both to the formation of *cis*-linked disaccharide. Higher *cis-*selectivity was obtained with the more sterically hindered monosaccharide-acceptor compared to isopropanol, in particular when building blocks with bulky pivaloyl (Piv) **17** (entry 3 vs 17), triphenylacetyl **25** (entry 7 vs 21) protecting groups or disubstituted thioglycosides **27**–**30** (entries 9–12 vs 23–26) were used. For sterically more demanding nucleophiles, the protecting group pattern on the glycosylating agent might have an important impact on the stereochemical outcome of the glycosylation. Bulky protecting groups had been used in the solution phase synthesis to introduce *cis*-linked glucose residues, and an α-glucan decamer was prepared recently.^50,56^ Overall, selectivities exceeding 10:1 (α/β) were achieved when all factors favoring *cis*-glycoside formation were combined using building blocks **27**–**30** carrying 3,6-diesters for AGA.

### Glycosylations on Solid Support

Using the optimized solution-phase conditions, glycosylations were carried out on a Merrifield resin functionalized with the 5-aminopentanol photolabile linker (**35**) (Table 2). The coupling cycle involved an acidic wash with trimethylsilyl trifluoromethanesulfonate (TMSOTf), followed by NIS-TfOH induced glycosylation (−20 °C for 5 min → 0 °C for 60 min), capping of any unreacted nucleophile with acetic anhydride (Ac_2_O) and methanesulfonic acid (MsOH), and cleavage of the temporary Fmoc protective group with triethylamine (TEA). After completion of each synthesis, the product was released from the resin by irradiation with UV light. The α (1–4) glucose pentasaccharide containing a β-glycosidic linkage between the first unit and the amino pentanol linker served as a model to study the *cis*-selectivity on both C4 secondary and C6 primary hydroxyl groups.^49^

**Table 2:**
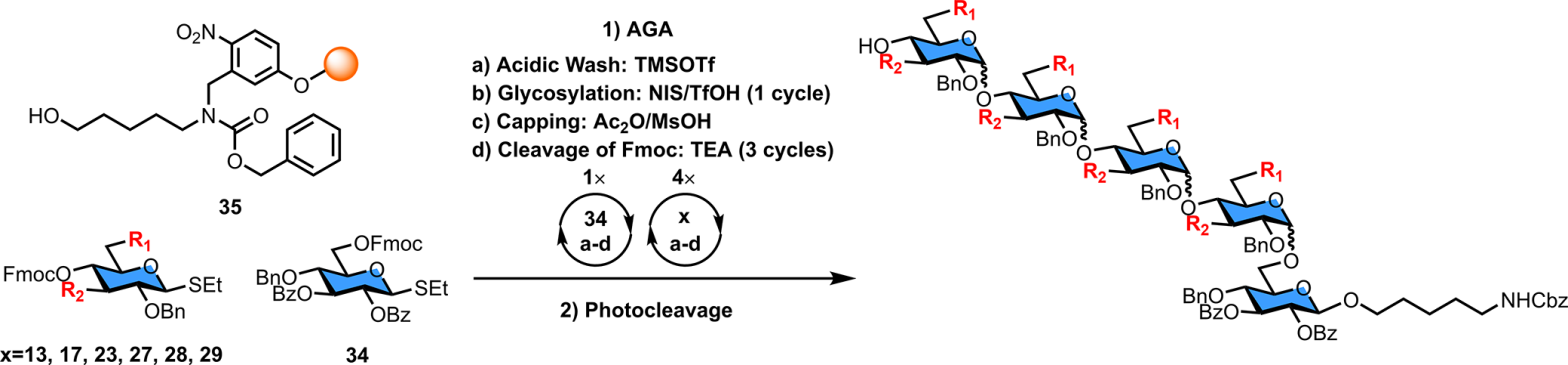
Screening of Glycosylation Conditions by AGA

entry	building block	*R*_1_	*R*_2_	product	selectivity	yield
**1**	**13**	OAc	OBn	**36**	0.35	40%
**2**	**17**	OPiv	OBn	**37**	0.93	4%
**3**	**23**	O(*p-*NO_2_Bz)	OBn	**38**	0.68^a^	14%
**4**	**27**	OPiv	OPiv	**39**	>0.95	3%
**5**	**28**	**OBz**	**OBz**	**40**	**0.74**	**17%**
**6**	**29**	O(*p-*NO_2_Bz)	O(*p-*NO_2_Bz)	**41**	0.45^a^	19%

a The *p*-nitrobenzoyl group present in building blocks **23** and **29** is not stable to UV irradiation. Therefore, the data was calculated based on deprotected products.

The products **36**–**41** were characterized by analytical HPLC and were purified in protected form to remove any deletion sequences. All pentamer products were collected. The isolated yield of the combined pentamers is reported independently of the stereochemistry of the linkages. The selectivity is calculated as the ratio of the amount of desired product containing four α-linkages to all pentamer products, as measured by NMR spectroscopy (for details, see Supporting Information). For protected glucans in this study, the chemical shift of α-linked anomeric proton is usually > 4.8 ppm and anomeric carbon < 100 ppm; for β-linked anomeric proton, the chemical shift is usually < 4.7 ppm and anomeric carbon > 100 ppm.

In search for a suitable building block for α-glucan AGA, both yield and *cis*-selectivity were taken into consideration. The results for thioglycoside **13** were in good agreement with our earlier findings (Table 2, entry 1).^49^ Despite the promising yield obtained with monoacetylated **13**, only 35% of the pentamer products had the desired stereochemistry (entry 1). The pivaloyl group at 6-OH (**17** and **27**) resulted in good *cis*-selectivity on solid phase (entries 2 and 4). However, bulky esters at C3 or C6 lowered the reactivity of **17** and **27** both as glycosylating agents and nucleophiles to render them unsuitable for the assembly of longer oligosaccharides. Building blocks **23** and **28** showed similar yields and *cis*-selectivity (entries 3 and 5). The *trans*-linked side-products arose mainly from the glycosylation of primary hydroxyl group of **34**. Di(*p*-nitrobenzoyl) protected building block **29** showed a slightly lower *cis*-selectivity (entry 6). Additionally, the *p*-nitrobenzoyl group was not stable under UV irradiation and complicated purification and characterization of the protected oligomers. 3,6-Dibenzoylated thioglycoside building block **28** emerged as the best choice considering selectivity and yield (entry 5). Similar good *cis*-selectivity was observed recently with galactose building blocks bearing multiple benzoyl (Bz) groups.^57^

### AGA of Starch and Glycogen α-Glucans

**Figure 2:**
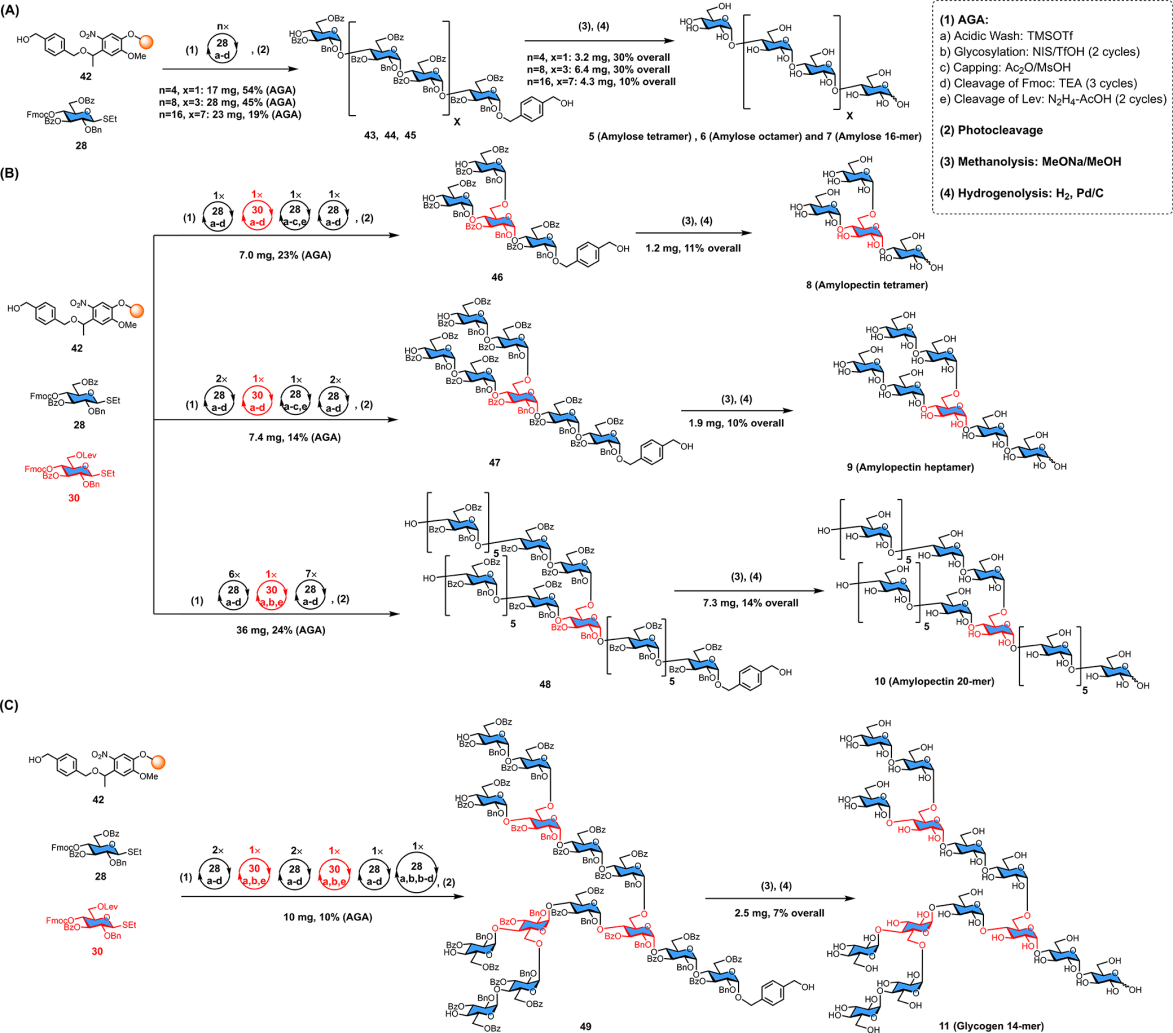
AGA of amylose, amylopectin, and glycogen α-glucans. (A) AGA of amylose oligosaccharides **5**–**7**. (B) AGA of amylopectin polysaccharides **8**–**10**. (C) AGA of glycogen oligosaccharide **11**.

**Figure 3:**
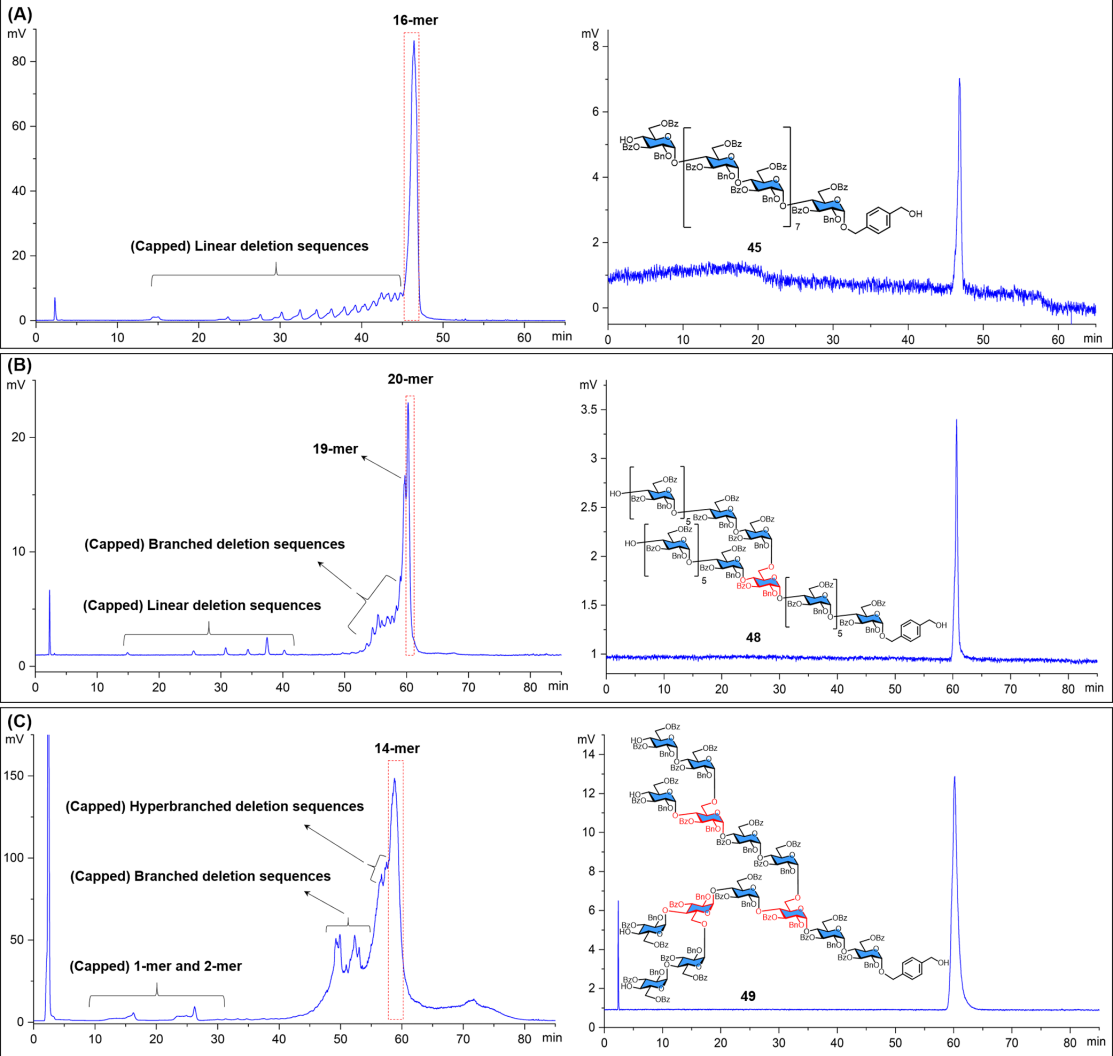
HPLC analysis of protected 16-mer **45**, 20-mer **48,** and 14-mer **49**. Left side, crude mixture after AGA; right side, target compound after purification. (A) 16-mer **45**; (B) 20-mer **48**; (C) 14-mer **49**. Signal was collected through ELSD.

On the basis of the preliminary AGA results described above, the reaction conditions were optimized in the context of the synthesis of a series of α-glucans (Figure 2). The 3,6-di-*O*-benzyolated thioglycoside **28** carrying a temporary 4-OFmoc protecting group was employed to assemble the α (1–4) glycan backbone. α (1–6) Glycan branching was installed using building block **30** with benzoate ester at C3 and a 4-OFmoc and a 6-OLev temporary protecting groups. A double glycosylation cycle (−20 °C for 5 min → 0 °C for 60 min, twice) with thioglycoside **28** was used for each elongation to increase yields and minimize the formation of deletion sequences. Traceless linker (**42**)^42^ was cleaved by photolysis and removed after global deprotection to release the natural starch/glycogen α-glucan. Linear amylose oligomer α (1–4) glucan, including tetramer **5**, octamer **6**, and 16-mer **7**, were prepared with good yield and selectivity (Figure 2A, see Supporting Information for full characterization). The HPLC analysis of the crude protected 16-mer **45** after AGA displayed the (partially capped) deletion sequences as the record of glycan elongation (Figure 3A). No *trans*-glycosylic linkages were observed by NMR spectroscopy, indicating that even the first glycosylation between **28** and linker **42** proceeded with excellent *cis*-selectivity.

To extend the AGA scope to natural amylopectin and glycogen structures, the selective installation of α (1–6) branching points within the α (1–4) backbone had to be developed. Glycosylation of the C6 primary hydroxyl group of **34** using building block **28** did not result in satisfactory *cis*-selectivity (Table 2). Two synthetic strategies were tested in the context of the syntheses of **8**–**11**, as the order of Fmoc and Lev cleavage may influence yield and selectivity. In Strategy A, after incorporation of building block **30**, the Fmoc group was cleaved and one glycosylation was performed in the presence of the levaloyl group. Then, Lev was cleaved and the C6 hydroxyl group was glycosylated (Figure S2A). After the branching point was introduced, α (1–4) elongation of backbone and side chain was achieved simultaneously. Tetramer **8** and heptamer **9** were prepared using this strategy (Figure 2B). Protected heptamer **47** was fully analyzed using HPLC and HSQC NMR to evaluate the *cis*-selectivity of Strategy A. After AGA, the crude product was analyzed with HPLC and all the detectable side-products were assigned according to MALDI-TOF MS (Figure S2B). Interestingly, most of the deletion sequences were capped by benzoyl groups that may result from the acetylated deletion sequences (capping step) through the ester exchange reaction. The MS signal of the desired heptamer was only observed for the main peak (red square) and its surrounding area, together with the signal for some deletion sequences (black square). The main product proved to be the desired fully *cis*-linked heptamer **47** by HSQC NMR (Figure S2C). The mixture of deletion sequences was also analyzed by HSQC NMR showing no evidence of β linkage (Figure S2D). These results strongly suggest that Strategy A is a highly selective method for the installation of α (1–6) linkages in our synthesis. Glycosylation at C4-OH may increase the glycan bulk for the subsequent glycosylation of the C6 hydroxyl group, thereby promoting *cis*-selectivity.

Strategy B was tested for trimer **50** (Figure S3) which not only allowed for the formation of different patterns during glycan chain elongation, but also gave a much higher yield compared to Strategy A and greatly simplified the synthesis of longer amylopectins. After glycosylation with building block **30**, cleavage of the levulinic ester was followed by glycosylation on C6-OH in the presence of 4-OFmoc (Figure S3A). After Fmoc cleavage, the C4 hydroxyl groups were glycosylated. Only one main fraction was observed by HPLC analysis of the crude product (Figure S3B) that proved to be the desired *cis*-linked trimer by HSQC NMR analysis (Figure S3C). The higher selectivity observed for this reaction, compared to the glycosylation of **34**, could result from the electron withdrawing 4-OFmoc in **30**, instead of an electron donating benzyl ether in **34** that renders it less nucleophilic.

Amylopectin 20-mer **10** was assembled using Strategy B (Figure 2B). After HPLC purification (Figure 3B), we obtained the protected 20-mer in 24% yield. The major side products are (partially capped) deletion sequences formed during chain extension after the α (1–6) branching point was installed. The simultaneous elongation of backbone and side chain on large glycan may not be as efficient using the standard glycosylation condition.

The assembly of a more challenging, hyperbranched glycogen model **11** was undertaken. Up to the secondary branching points, the assembly followed the procedures established above. Two secondary branching points was introduced with one double glycosylation, but in order to add four glucose units in one pot at the end, two double glycosylation cycles were required. The HPLC analysis of the crude product after AGA indicated that the deletion sequences were mainly caused by incomplete multiple-point glycosylation (Figure 3C).

Since the end-stage deletion sequences partially overlap with the product peak, three HPLC purifications were required for **48** and **49** (see Supporting Information, Method B3 in AGA part). The first purification yielded pure fraction (I) (purity >90% indicated by MS) and fraction (II) (purity >50%). The second purification of fraction (II) yielded fraction (III) (purity >90%), before the third purification of combined fractions (I) and (III) gave the product in high purity. After deprotection, both **10** and **11** were obtained in good yield and stereochemical purity, as confirmed by HSQC NMR (see Supporting Information for full characterization). The entire AGA process to access the protected 20-mer **48** took about 50 h and for protected 14-mer **49** took only 30 h.

### Comparison of Synthetic α-Glucans and Natural Starch/Glycogen

**Figure 4:**
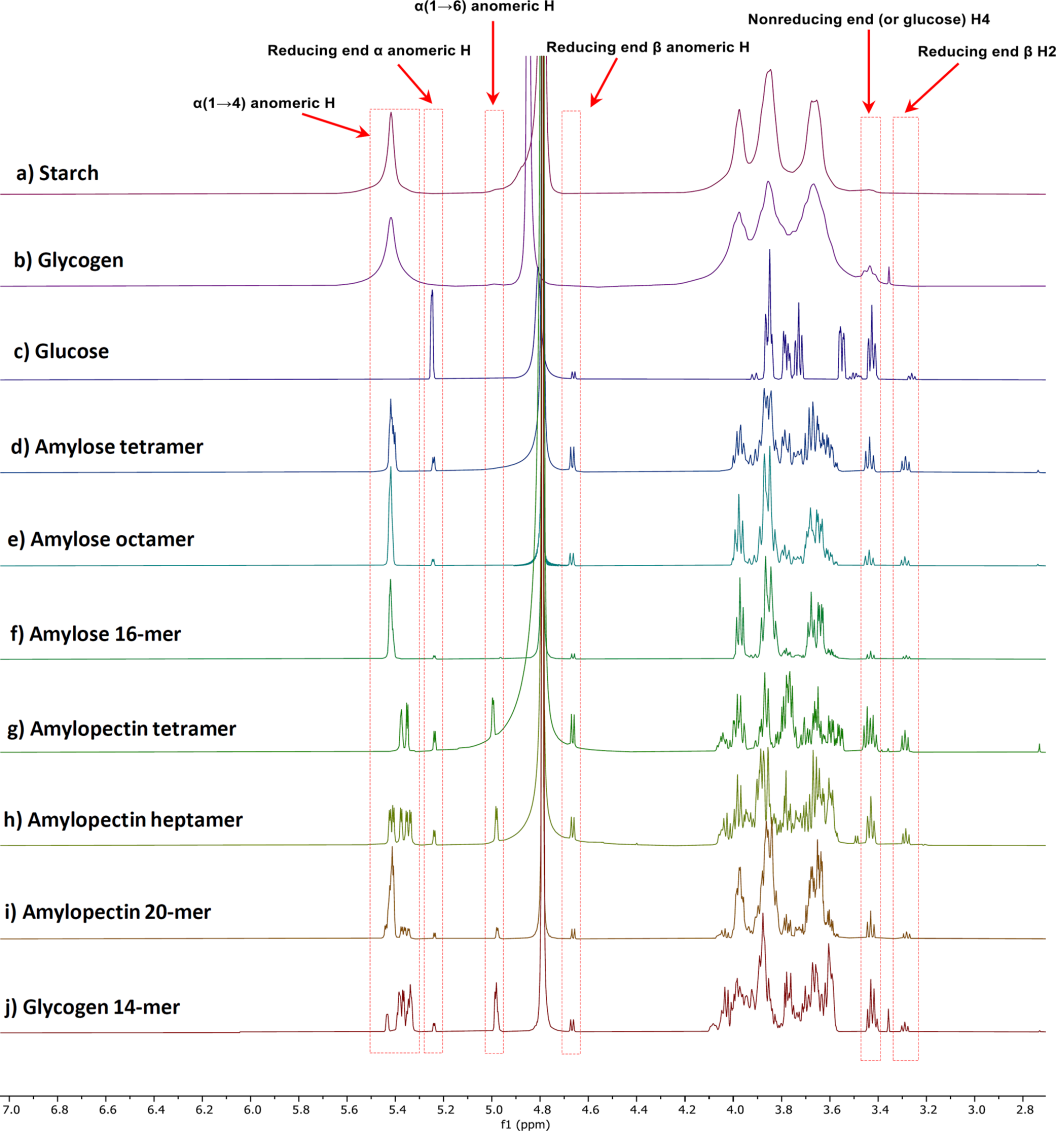
Comparison of ^1^H NMR spectra of natural starch (a), glycogen (b), glucose (c), and synthetic α-glucans (d–j).

The properties of the synthetic α-glucan were compared to natural potato starch and glycogen from bovine liver. All characteristic signals in the NMR spectra of the synthetic glucans exactly match those of the natural compounds (Figure 4). Except for glucose, all compounds show signals from 5.30 to 5.50 ppm that represent the α (1–4) anomeric protons of the essential starch/glycogen linkages. Signals at 5.24, 4.67, and 3.29 ppm represent the α, β anomeric protons and the β H2 at the reducing end that are not observed in natural starch and glycogen. The signals at 4.99–4.96 ppm in starch and glycogen represent the α (1–6) anomeric protons of the glycan branching points. The signals at 3.45–3.39 ppm represent the C4 protons of the nonreducing end. These signals are indicative of the “degree of branching” of starch/glycogen polysaccharides with strong signals (compared to the signals of reducing end protons) for glycogen and 14-mer **11**. In contrast to the broad signals for heterogeneous natural polysaccharides, all homogeneous synthetic compounds show sharp NMR signals. Our data highlight the importance of synthetic compounds as standard to characterize naturally sourced compounds.

**Figure 5:**
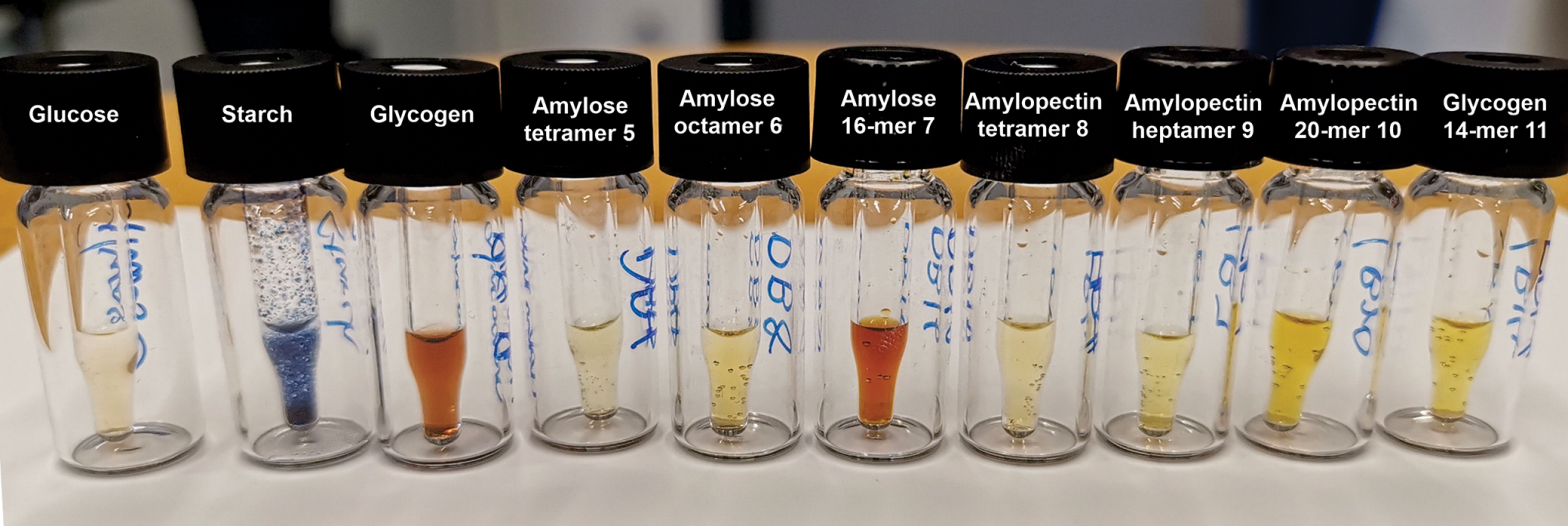
I_2_–KI staining of glucose, natural starch/glycogen and synthetic α-glucans.

The iodine–starch test is a simple, but characteristic detection method for α (1–4) glucans.^9^ Although the details of this interaction was not entirely understood, an iodine–iodide complex is known to embed into the helix formed by α (1–4) glucan.^58,59^ With increasing glucan chain length, more complex is bound, causing a color change from yellow, orange, red-brown, to purple and dark blue. Amylose tetramer **5**, amylopectin tetramer **8**, and heptamer **9** show a light yellow color similar to glucose. Amylose octamer **6**, amylopectin 20-mer **10**, and glycogen 14-mer **11** result in a deep yellow color, indicative of weak interactions between iodide complex and glucan. Amylose 16-mer **7** gave a red-brown color similar to glycogen indicating that the α (1–4) glucan chain has the required length to strongly interact with the iodine–iodide complex. The long α (1–4) glucan backbone of natural starch is poorly soluble at room temperature and has a very strong bonding with the complex, indicated by the dark blue color (Figure 5). Our results match literature reports that six continuous α (1–4) glucose residues are needed to form the repeating unit of helical structure, which can assemble with iodine complex nicely.^60,61^

## Conclusions

On the basis of a comprehensive study of 21 differentially protected thioglycoside building blocks, glycosylation conditions that result in high yielding *cis*-selective glycosidic bond formation were developed. With amylose and amylopectin oligo- and polysaccharides as targets, 3,6-dibenzoylated glucose thioglycoside **28** provided the perfect balance between *cis*-selectivity and reactivity during AGA required to prepare long α (1–4) glycans. Differentially protected thioglycoside **30** was the key to α (1–6) branching with excellent *cis*-selectivity and good yield using AGA. The 20-mer amylopectin polysaccharide **10** is the largest entirely *cis*-linked branched carbohydrate assembled by chemical synthesis to date. NMR studies and iodine-stain tests, confirmed that the synthetic glucans share common structural properties with natural starch and glycogen. This simple and efficient AGA method provides convenient access to large, well-defined *cis*-linked starch and glycogen polysaccharides for biological and material science investigations. Future improvements on the instrumentation hardware and the chemistry will shorten assembly times. Increased glycosylation efficiencies will save building blocks, reduce deletion sequences, and facilitate HPLC purification.

## Supporting Information Available

Experimental procedures, additional data, and spectral data for all reactions and compounds (PDF)

## ABBREVIATIONS

AGA: automated glycan assembly
Ac: acetyl
Fmoc: fluorenylmethoxycarbonyl
Bn: benzyl
Bz: benzoyl
NIS: *N*-iodosuccinimide
TfOH: triflic acid
DCM: dichloromethane
*i*-PrOH: isopropanol
EWG: electron withdrawing group
EDG: electron donating group
Piv: pivaloyl
TMSOTf: trimethylsilyl trifluoromethanesulfonate
Ac_2_O: acetic anhydride
MsOH: methanesulfonic acid
TEA: trimethylamine
Lev: levaloyl
HPLC: high-performance liquid chromatography
HRMS: high-resolution mass spectrometry
MALDI-TOF MS: matrix-assisted laser desorption ionization–time-of-flight mass spectrometry
ELSD: vaporative light scattering detector
