# Supplementary material for: Automated Assembly of Starch and Glycogen Polysaccharides
Source: J Am Chem Soc. 2021 Jun 11;143(26):9758–68. doi: 10.1021/jacs.1c02188 (PMC8267850; doi:10.1021/jacs.1c02188)
Supplement: Supplementary file 1 — ja1c02188_si_001.pdf [file ja1c02188_si_001.pdf]

Electronic Supplementary Information

Automated Assembly of Starch and Glycogen Polysaccharides

Yuntao Zhu [1], Martina Delbianco [1], Peter H. Seeberger [1, 2, *]

[1] Max Planck Institute for Colloids and Interfaces, Am Mühlenberg 1, 14476 Potsdam, Germany

[2] Institute for Chemistry and Biochemistry, Freie Universität Berlin, Arnimallee 22, 14195 Berlin, Germany.

Contents

Supporting Figures and Tables 6

1. General Materials and Methods 11

2. Synthesis of Building Blocks 12

2.1 Synthesis of 12 12

2.2 Synthesis of 13 15

2.3 Synthesis of 14 21

2.4 Synthesis of 15 27

2.5 Synthesis of 16 33

2.6 Synthesis of 17 39

2.7 Synthesis of 18 51

2.8 Synthesis of 19 57

2.9 Synthesis of 20 60

2.10 Synthesis of 21 66

2.11 Synthesis of 22 69

2.12 Synthesis of 23 72

2.13 Synthesis of 24 81

2.14 Synthesis of 25 84

2.15 Synthesis of 26 87

2.16 Synthesis of 27 102

2.17 Synthesis of 28 105

2.18 Synthesis of 29 108

2.19 Synthesis of 30 111

2.20 Synthesis of 31 117

2.21 Synthesis of 32 125

2.22 Synthesis of 110 140

2.23 Synthesis of 111 152

2.24 Synthesis of 112 155

3. General Methods for Solution-phase Glycosylation Study 158

3.1 Method A: Glycosylation with Isopropanol as Nucleophile 158

3.2 Method B: Glycosylation with Monosaccharide as Nucleophile 159

4. Characterization of Solution-phase Glycosylation Products 160

4.1 Isopropyl 2,3,6-tri-*O*-benzyl-D-glucopyranoside, 74 160
4.2 Isopropyl 2,3-di-*O*-benzyl-6-*O*-acetyl-D-glucopyranoside, 75 165
4.3 Isopropyl 2,3-di-*O*-benzyl-6-*O*-formyl-D-glucopyranoside, 76 170
4.4 Isopropyl 2,3-di-*O*-benzyl-6-*O*-propionyl-D-glucopyranoside, 77 175
4.5 Isopropyl 2,3-di-*O*-benzyl-6-*O*-levulinoyl-D-glucopyranoside, 78 180
4.6 Isopropyl 2,3-di-*O*-benzyl-6-*O*-pivaloyl-D-glucopyranoside, 79 185
4.7 Isopropyl 2,3-di-*O*-benzyl-D-glucopyranoside, 80 190
4.8 Isopropyl 2,3-di-*O*-benzyl-6-*O*-acryl-D-glucopyranoside, 81 195
4.9 Isopropyl 2,3-di-*O*-benzyl-6-*O*-benzoyl-D-glucopyranoside, 82 200
4.10 Isopropyl 2,3-di-*O*-benzyl-6-*O*-(4-methylbenzoyl)-D-glucopyranoside, 83 205
4.11 Isopropyl 2,3-di-*O*-benzyl-6-*O*-(4-methoxybenzoyl)-D-glucopyranoside, 84 210
4.12 Isopropyl 2,3-di-*O*-benzyl-6-*O*-(4-nitrobenzoyl)-D-glucopyranoside, 85 215
4.13 Isopropyl 2,3-di-*O*-benzyl-6-*O*-carboxybenzyl-D-glucopyranoside, 86 220
4.14 Isopropyl 2,3-di-*O*-benzyl-6-*O*-triphenylacetyl-D-glucopyranoside, 87 225
4.15 Isopropyl 2-*O*-benzyl-3,6-di-*O*-acetyl-D-glucopyranoside, 88 230
4.16 Isopropyl 2-*O*-benzyl-3,6-di-*O*-pivaloyl-D-glucopyranoside, 89 235
4.17 Isopropyl 2-*O*-benzyl-3,6-di-*O*-benzoyl-D-glucopyranoside, 90 240
4.18 Isopropyl 2-*O*-benzyl-3,6-di-*O*-(4-nitrobenzoyl)-D-glucopyranoside, 91 245
4.19 Isopropyl 2-*O*-benzyl-3-*O*-benzoyl-6-*O*-levulinoyl-D-glucopyranoside, 92 250
4.20 Isopropyl 2,3-di-*O*-benzyl-6-deoxy-6-fluoro-D-glucopyranoside, 93 255
4.21 Isopropyl 2,3-di-*O*-benzyl-6-*O*-methyl-D-glucopyranoside, 94 261
4.22 Benzyl 2,3,6-tri-*O*-benzyl-D-glucopyranosyl-(1→4)-2-*O*-benzoyl-3,6-di-*O*-benzyl-β-D-glucopyranoside, 95 266
4.23 Benzyl 2,3-di-*O*-benzyl-6-*O*-acetyl-D-glucopyranosyl-(1→4)-2-*O*-benzoyl-3,6-di-*O*-benzyl-β-D-glucopyranoside, 96 271
4.24 Benzyl 2,3-di-*O*-benzyl-6-*O*-pivaloyl-D-glucopyranosyl-(1→4)-2-*O*-benzoyl-3,6-di-*O*-benzyl-β-D-glucopyranoside, 97 276
4.25 Benzyl 2,3-di-*O*-benzyl-6-*O*-benzoyl-D-glucopyranosyl-(1→4)-2-*O*-benzoyl-3,6-di-*O*-benzyl-β-D-glucopyranoside, 98 281
4.26 Benzyl 2,3-di-*O*-benzyl-6-*O*-(4-methoxybenzoyl)-D-glucopyranosyl-(1→4)-2-*O*-benzoyl-3,6-di-*O*-benzyl-β-D-glucopyranoside, 99 286
4.27 Benzyl 2,3-di-*O*-benzyl-6-*O*-(4-nitrobenzoyl)-D-glucopyranosyl-(1→4)-2-*O*-benzoyl-3,6-di-*O*-benzyl-β-D-glucopyranoside, 100 291
4.28 Benzyl 2,3-di-*O*-benzyl-6-*O*-triphenylacetyl-D-glucopyranosyl-(1→4)-2-*O*-benzoyl-3,6-di-*O*-benzyl-β-D-glucopyranoside, 101 296

4.29 Benzyl 2-*O*-benzyl-3,6-di-*O*-acetyl-D-glucopyranosyl-(1→4)-2-*O*-benzoyl-3,6-di-*O*-benzyl-β-D-glucopyranoside, 102 301

4.30 Benzyl 2-*O*-benzyl-3,6-di-*O*-pivaloyl-D-glucopyranosyl-(1→4)-2-*O*-benzoyl-3,6-di-*O*-benzyl-β-D-glucopyranoside, 103 306

4.31 Benzyl 2-*O*-benzyl-3,6-di-*O*-benzoyl-D-glucopyranosyl-(1→4)-2-*O*-benzoyl-3,6-di-*O*-benzyl-β-D-glucopyranoside, 104 311

4.32 Benzyl 2-*O*-benzyl-3,6-di-*O*-(4-nitrobenzoyl)-D-glucopyranosyl-(1→4)-2-*O*-benzoyl-3,6-di-*O*-benzyl-β-D-glucopyranoside, 105 316

4.33 Benzyl 2-*O*-benzyl-3-*O*-benzoyl-6-*O*-levulinoyl-D-glucopyranosyl-(1→4)-2-*O*-benzoyl-3,6-di-*O*-benzyl-β-D-glucopyranoside, 106 321

4.34 Benzyl 2,3-di-*O*-benzyl-6-deoxy-6-fluoro-D-glucopyranosyl-(1→4)-2-*O*-benzoyl-3,6-di-*O*-benzyl-β-D-glucopyranoside, 107 326

4.35 Benzyl 2,3-di-*O*-benzyl-6-*O*-methyl-D-glucopyranosyl-(1→4)-2-*O*-benzoyl-3,6-di-*O*-benzyl-β-D-glucopyranoside, 108 332

4.36 Isopropyl 2,6-di-*O*-benzyl-3-*O*-acetyl-D-glucopyranoside, 117 337

4.37 Isopropyl 2,6-di-*O*-benzyl-3-*O*-benzoyl-D-glucopyranoside, 118 342

4.38 Isopropyl 2,6-di-*O*-benzyl-3-*O*-pivaloyl-D-glucopyranoside, 119 347

4.39 Benzyl 2,6-di-*O*-benzyl-3-*O*-acetyl-D-glucopyranosyl-(1→4)-2-*O*-benzoyl-3,6-di-*O*-benzyl-β-D-glucopyranoside, 120 352

4.40 Benzyl 2,6-di-*O*-benzyl-3-*O*-benzoyl-D-glucopyranosyl-(1→4)-2-*O*-benzoyl-3,6-di-*O*-benzyl-β-D-glucopyranoside, 121 357

4.41 Benzyl 2,6-di-*O*-benzyl-3-*O*-pivaloyl-D-glucopyranosyl-(1→4)-2-*O*-benzoyl-3,6-di-*O*-benzyl-β-D-glucopyranoside, 122 362

5. Temperature and Solvent Control Study 367

5.1 Temperature control study 367

5.2 Glycosylation study in different ethers 371

5.3 Glycosylation study in anhydrous DCM-dioxane (5:1) 374

6. Automated Glycan Assembly 384

6.1 General Materials and Methods 385

6.2 Solid-phase Glycosylation Study 391

6.2.1 Synthesis of pentasaccharide 36 391

6.2.2 Synthesis of pentasaccharide 37 394

6.2.3 Synthesis of pentasaccharide 38 399

6.2.4 Synthesis of pentasaccharide 39 404

6.2.5 Synthesis of pentasaccharide 40 407

6.2.6 Synthesis of pentasaccharide 41 ... 410
7. Synthesis of Starch/Glycogen α-Glucan ... 413
7.1 Synthesis of amylose tetramer 5 ... 413
7.2 Synthesis of amylose octamer 6 ... 425
7.3 Synthesis of amylose 16-mer 7 ... 437
7.4 Synthesis of trimer 50 ... 449
7.5 Synthesis of amylopectin tetramer 8 ... 455
7.6 Synthesis of amylopectin heptamer 9 ... 467
7.7 Synthesis of amylopectin 20-mer 10 ... 481
7.8 Synthesis of glycogen 14-mer 11 ... 493
8. I_2-KI Stain Test ... 504
9. References ... 505

Supporting Figures and Tables

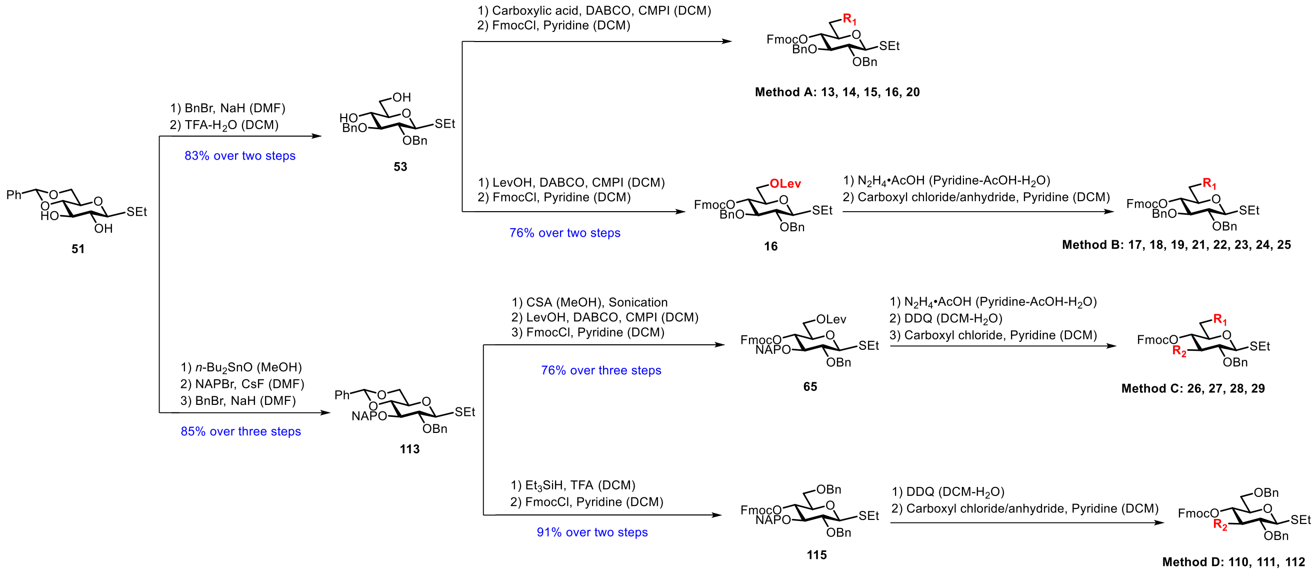

Figure S1 | General strategies for building block synthesis.

Table S1 | Solution phase glycosylations for all building blocks

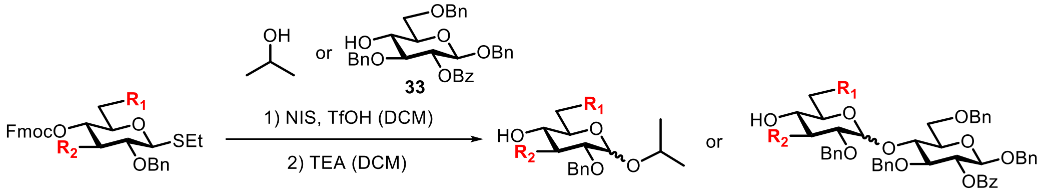

Entry	Building Block	R_1	R_2	Acceptor	Temperature	Ratio (α:β)	Yield (α+β)
1	**12**	OBn	OBn	*i*-PrOH	-15 °C → 0 °C	**1 : 2.9**	93%
2	**13**	OAc	OBn	*i*-PrOH	-15 °C → 0 °C	**2.3 : 1**	95%
3	**14**	OCHO	OBn	*i*-PrOH	-15 °C → 0 °C	2.0 : 1	95%
4	**15**	O(CO)C_2H_5	OBn	*i*-PrOH	-15 °C → 0 °C	2.2 : 1	94%
5	**16**	OLev	OBn	*i*-PrOH	-15 °C → 0 °C	1.6 : 1	93%
6	**17**	OPiv	OBn	*i*-PrOH	-15 °C → 0 °C	2.5 : 1	92%
7	**18**	O(CO)CF_3	OBn	*i*-PrOH	-15 °C → 0 °C	2.4 : 1	84%*
8	**19**	O(CO)C_2H_3	OBn	*i*-PrOH	-15 °C → 0 °C	2.0 : 1	62%
9	**20**	OBz	OBn	*i*-PrOH	-15 °C → 0 °C	1.7 : 1	93%
10	**21**	O(*p*-MeBz)	OBn	*i*-PrOH	-15 °C → 0 °C	2.1 : 1	92%
11	**22**	O(*p*-OMeBz)	OBn	*i*-PrOH	-15 °C → 0 °C	2.2 : 1	90%
12	**23**	O(*p*-NO_2Bz)	OBn	*i*-PrOH	-15 °C → 0 °C	3.5 : 1	89%
13	**24**	OCbz	OBn	*i*-PrOH	-15 °C → 0 °C	1.3 : 1	92%

14	**25**	O(CO)Ph_3	OBn	*i*-PrOH	-15 °C → 0 °C	1.9 : 1	90%
15	**26**	OAc	OAc	*i*-PrOH	-15 °C → 0 °C	**4.5 : 1**	84%
16	**27**	OPiv	OPiv	*i*-PrOH	-15 °C → 0 °C	**6.4 : 1**	88%
17	**28**	OBz	OBz	*i*-PrOH	-15 °C → 0 °C	4.8 : 1	87%
18	**29**	O(*p*-NO_2Bz)	O(*p*-NO_2Bz)	*i*-PrOH	-15 °C → 0 °C	**7.1 : 1**	74%
19	**30**	OLev	OBz	*i*-PrOH	-15 °C → 0 °C	2.4 : 1	76%
20	**31**	F	OBn	*i*-PrOH	-15 °C → 0 °C	1 : 1.1	96%
21	**32**	OMe	OBn	*i*-PrOH	-15 °C → 0 °C	1 : 2.6	94%
22	**110**	OBn	OAc	*i*-PrOH	-15 °C → 0 °C	1 : 1.4	79%
23	**111**	OBn	OBz	*i*-PrOH	-15 °C → 0 °C	1 : 1.1	95%
24	**112**	OBn	OPiv	*i*-PrOH	-15 °C → 0 °C	1 : 1.3	73%
25	**12**	OBn	OBn	**33**	-15 °C → 0 °C	1.5 : 1	83%
26	**13**	OAc	OBn	**33**	-15 °C → 0 °C	3.8 : 1	75%
27	**17**	OPiv	OBn	**33**	-15 °C → 0 °C	5.5 : 1	69%
28	**20**	OBz	OBn	**33**	-15 °C → 0 °C	6.8 : 1	72%
29	**22**	O(*p*-OMeBz)	OBn	**33**	-15 °C → 0 °C	5.6 : 1	68%
30	**23**	O(*p*-NO_2Bz)	OBn	**33**	-15 °C → 0 °C	6.2 : 1	67%
31	**25**	O(CO)Ph_3	OBn	**33**	-15 °C → 0 °C	7.5 : 1	44%
32	**26**	OAc	OAc	**33**	-15 °C → 0 °C	9.8 : 1	78%
33	**27**	OPiv	OPiv	**33**	-15 °C → 0 °C	>10 : 1	62%
34	**28**	**OBz**	**OBz**	**33**	-15 °C →0 °C	**>10 : 1**	**83%**
35	**29**	O(*p*-NO_2Bz)	O(*p*-NO_2Bz)	**33**	-15 °C →0 °C	>10 : 1	64%
36	**30**	**OLev**	**OBz**	**33**	-15 °C →0 °C	**>10 : 1**	**80%**
37	**31**	F	OBn	**33**	-15 °C →0 °C	3.9 : 1	75%
38	**32**	OMe	OBn	**33**	-15 °C →0 °C	2.7 : 1	81%
39	**110**	OBn	OAc	**33**	-15 °C → 0 °C	2.9 : 1	65%
40	**111**	OBn	OBz	**33**	-15 °C → 0 °C	4.9 : 1	64%
41	**112**	OBn	OPiv	**33**	-15 °C → 0 °C	4.7 : 1	65%

Glycosylations using isopropanol and monosaccharides as nucleophiles in DCM, -15 °C for 5 min then 0 °C for 60 min. The ratio of α/β anomers were quantified by NMR, yield represented the isolated yield of α and β anomers. *For building block **18**, trifluoroacetyl group was hydrolysed during workup, the data was calculated from C6-OH compound.

Table S2 | Solution phase temperature control study

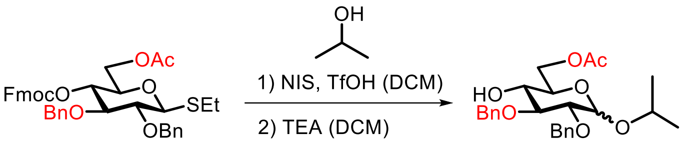

13 75

Entry	Building Block	R_1	R_2	Acceptor	Temperature	Ratio (α:β)	Yield (α+β)
1	**13**	OAc	OBn	*i*-PrOH	-45 °C (60 min)	1.3 : 1	17%
2	**13**	OAc	OBn	*i*-PrOH	-20 °C (60 min)	1.8 : 1	84%
3	**13**	OAc	OBn	*i*-PrOH	0 °C (60 min)	2.9 : 1	90%
4	**13**	OAc	OBn	*i*-PrOH	-15 °C (5 min) → 0 °C (60 min)	**2.3 : 1**	95%

Table S3 | Solution phase solvent control study

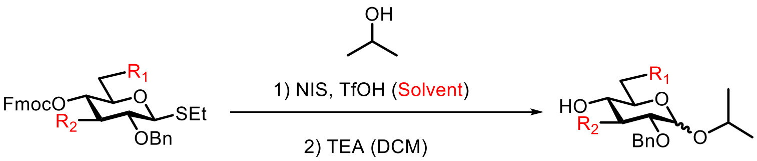

12,13,20,111,28 74,75,82,118,90

Entry	Building Block	R_1	R_2	Acceptor	Temperature	Solvent	Ratio (α:β)	Yield (α+β)
1	**13**	OAc	OBn	*i*-PrOH	-15 °C (5 min) → 0 °C (60 min)	DCM	2.3 : 1	95%
2	**13**	OAc	OBn	*i*-PrOH	-15 °C (5 min) → 0 °C (60 min)	Ethyl ether[a]	4.1 : 1	87%
3	**13**	OAc	OBn	*i*-PrOH	-15 °C (5 min) → 0 °C (60 min)	THF	2.2 : 1	83%
4	**13**	OAc	OBn	*i*-PrOH	-15 °C (5 min) → 0 °C (60 min)	Dioxane[b]	4.3 : 1	92%
5	**13**	OAc	OBn	*i*-PrOH	-15 °C (5 min) → 0 °C (60 min)	MTBE[a,c]	5.0 : 1	70%
6	**13**	OAc	OBn	*i*-PrOH	-15 °C (5 min) → 0 °C (60 min)	DCM-Dioxane[d]	2.8 : 1	90%
7	**12**	OBn	OBn	*i*-PrOH	-15 °C (5 min) → 0 °C (60 min)	DCM	1 : 2.9	93%
8	**12**	OBn	OBn	*i*-PrOH	-15 °C (5 min) → 0 °C (60 min)	DCM-Dioxane[d]	1 : 1.4	92%
9	**20**	OBz	OBn	*i*-PrOH	-15 °C (5 min) → 0 °C (60 min)	DCM	1.7 : 1	93%
10	**20**	OBz	OBn	*i*-PrOH	-15 °C (5 min) → 0 °C (60 min)	DCM-Dioxane[d]	2.2 : 1	94%
11	**111**	OBn	OBz	*i*-PrOH	-15 °C (5 min) → 0 °C (60 min)	DCM	1 : 1.1	95%
12	**111**	OBn	OBz	*i*-PrOH	-15 °C (5 min) → 0 °C (60 min)	DCM-Dioxane[d]	2.2 : 1	93%
13	**28**	OBz	OBz	*i*-PrOH	-15 °C (5 min) → 0 °C (60 min)	DCM	4.8 : 1	87%
14	**28**	OBz	OBz	*i*-PrOH	-15 °C (5 min) → 0 °C (60 min)	DCM-Dioxane[d]	**5.1 : 1**	84%

[a] Poor solubility of building block and NIS mixture. [b] Freezing during reaction. [c] MTBE : Methyl *tert*-butyl ether. [d] DCM : dioxane = 5 : 1.

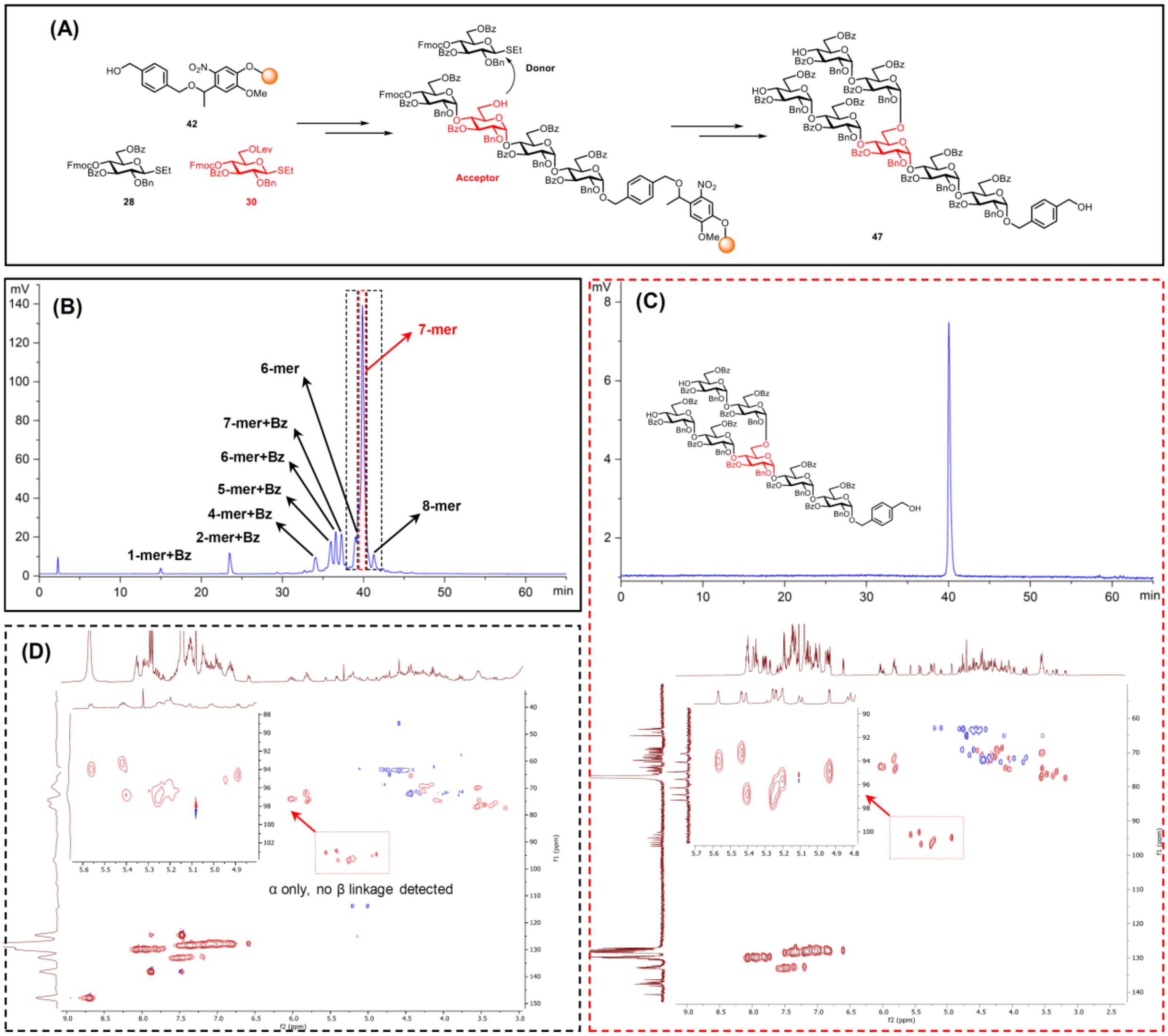

Figure S2 | Analysis of α (1-6) linkage incorporation during assembly of compound 47 (Strategy A). (A) Donor and acceptor for α (1-6) linkage incorporation during AGA. (B) HPLC analysis of crude **47** after AGA. The detectable side products are labelled. Desired heptamer **47** labelled in red square, and the side products close to **47** labelled in black square. (C) HPLC and HSQC NMR spectrums of pure **47**. (D) HSQC NMR spectrum of side products closed to **47** shows no β linkage is detectable.

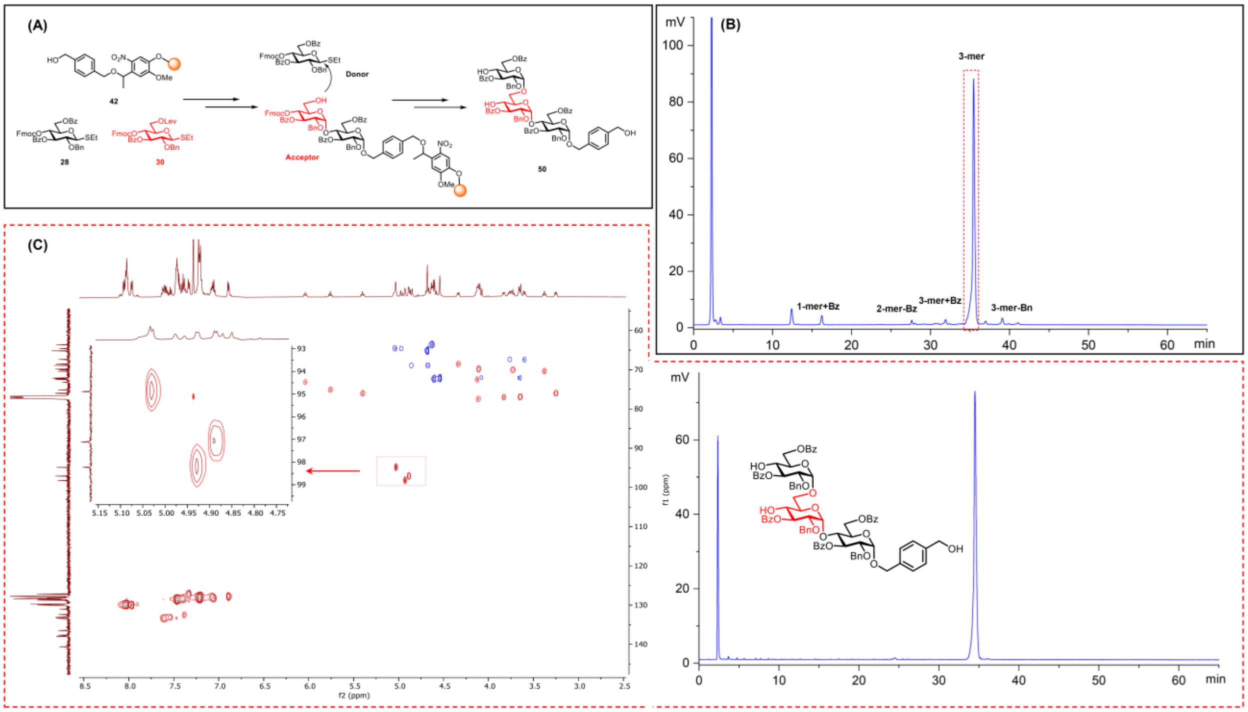

Figure S3 | Analysis of α (1-6) linkage incorporation during assembly of compound 50 (Strategy B). (A) Donor and acceptor for α (1-6) linkage incorporation during AGA. (B) HPLC analysis of crude **50** after AGA. Only trace amount of side products are detected and labelled. Desired trimer **50** labelled in red square. (C) HPLC and HSQC NMR spectrums of pure **50**.

1. General Materials and Methods

All chemicals used were reagent grade and used as supplied unless otherwise noted. The automated syntheses were performed on a home-built synthesizer developed at the Max Planck Institute of Colloids and Interfaces. Analytical thin-layer chromatography (TLC) was performed on Merck silica gel 60 F254 plates (0.25 mm). Compounds were visualized by UV irradiation or dipping the plate in a *p*-anisaldehyde (PAA) solution. Flash column chromatography was carried out by using forced flow of the indicated solvent on Fluka Kieselgel 60 M (0.04 – 0.063 mm). Analysis and purification by normal and reverse phase HPLC was performed by using an Agilent 1200 series. Products were lyophilized using a Christ Alpha 2-4 LD plus freeze dryer. ^{1}H, ^{13}C and HSQC NMR spectra were recorded on a Varian 400-MR (400 MHz), Varian 600-MR (600 MHz), or Bruker Biospin AVANCE700 (700 MHz) spectrometer. Spectra were recorded in $CDCl_3$ by using the solvent residual peak chemical shift as the internal standard ($CDCl_3$: 7.26 ppm ^{1}H, 77.0 ppm ^{13}C) or in D_2O using the solvent as the internal standard in ^{1}H NMR (D_2O: 4.79 ppm ^{1}H). High resolution mass spectra were obtained using a 6210 ESI-TOF mass spectrometer (Agilent) and a MALDI-TOF autoflex™ (Bruker). MALDI and ESI mass spectra were run on IonSpec Ultima instruments. IR spectra were recorded on a Perkin-Elmer 1600 FTIR spectrometer. Optical rotations were measured by using a Perkin-Elmer 241 and Unipol L1000 polarimeter.

2. Synthesis of Building Blocks

2.1 Synthesis of 12

Ethyl 2,3,6-tri-*O*-benzyl-4-*O*-(9-fluorenylmethoxycarbonyl)-1-thio-β-D-glucopyranoside, 12

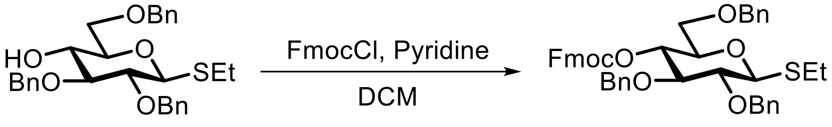

52 **12**

52 was prepared according to previously established procedures. [1]

Ethyl 2,3,6-tri-*O*-benzyl-1-thio-β-D-glucopyranoside **52** (940 mg, 1.9 mmol) was dissolved in anhydrous DCM (8 mL) and pyridine (2 mL) was added. The solution was cooled with an ice bath for 30 min, and fluorenylmethyloxycarbonyl chloride (FmocCl, 1.23 g, 4.7 mmol) was added slowly. The reaction was warmed to room temperature and stirred for 6 h. Upon completion, DCM (50 mL) was added and the organic phase was washed with aqueous citric acid (0.5 M, 50 mL). After extracting the water phase with DCM (20 mL), the organic layers were combined and dried over Na_2SO_4, filtered, and evaporated. The resulting crude product was purified by column chromatography (Hexane : EtOAc = 10:1→5:1) to give **12** as a white solid (1.12 g, 82%). ^{1}H NMR (400 MHz, $CDCl_3$) δ 7.79 (d, *J* = 7.6 Hz, 2H), 7.60 (dd, *J* = 7.6 Hz, 1H), 7.55 (dd, *J* = 7.6 Hz, 1H), 7.47 – 7.20 (m, 19H), 4.98 – 4.84 (m, 3H), 4.75 (appt, *J* = 10.6 Hz, 2H), 4.55 (m, 3H), 4.33 (qd, *J* = 10.4, 7.2 Hz, 2H), 4.15 (appt, *J* = 7.3 Hz, 1H), 3.77 (appt, *J* = 9.1 Hz, 1H), 3.73 – 3.63 (m, 3H), 3.53 (dd, *J* = 9.8, 8.8 Hz, 1H), 2.81 (qq, *J* = 12.7, 7.4 Hz, 2H), 1.38 (t, *J* = 7.4 Hz, 3H); ^{13}C NMR (101 MHz, $CDCl_3$) δ 154.55, 143.40, 143.27, 141.38, 141.34, 138.11, 138.02, 137.82, 128.56, 128.53, 128.43, 128.10, 128.01, 127.83, 127.79, 127.72, 127.30, 127.27, 125.25, 125.15, 120.18, 120.16, 85.26, 83.92, 81.43, 75.73, 75.71, 73.68, 70.17, 69.93, 46.75, 25.25, 15.31; $[\alpha]_D^{25}$ -4.39 (c = 1, $CHCl_3$); IR (neat) ν_{max} = 1753, 1257, 738 cm^{-1}; m/z (HRMS$^+$) [M + Na]$^+$ 739.2705 ($C_{44}H_{44}O_7SNa^+$ requires 739.2700).

^{1}H NMR of 12 (400 MHz, $CDCl_3$)

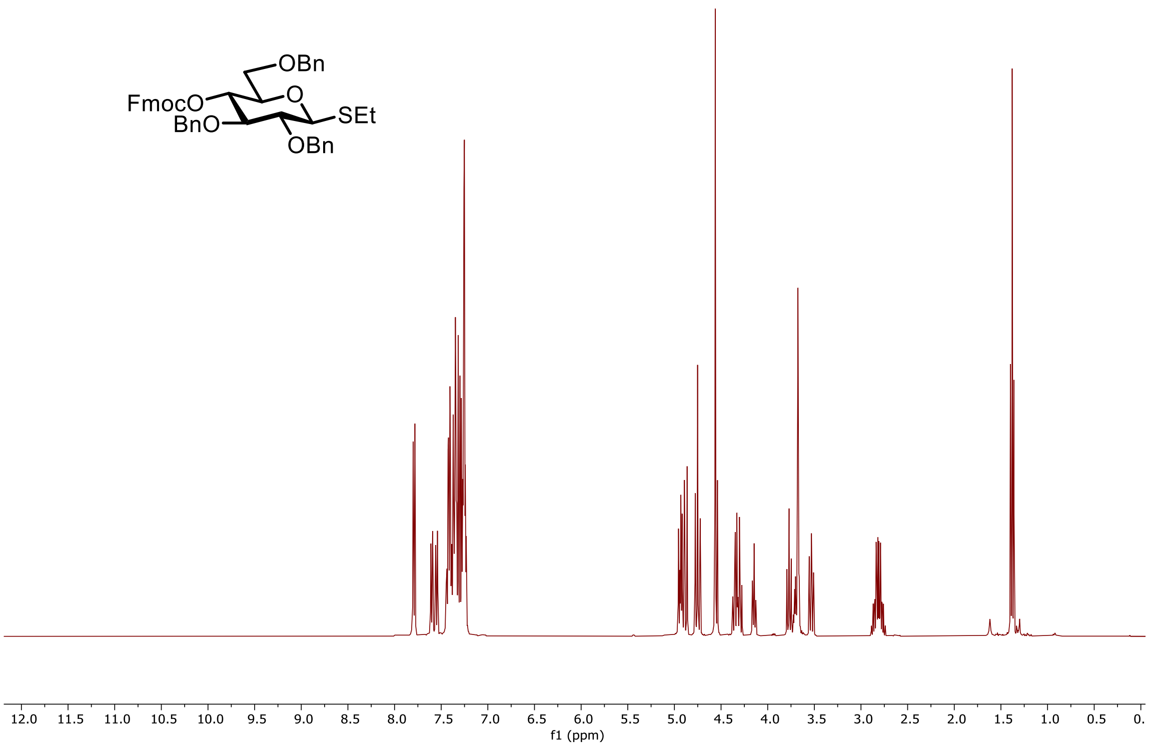

^{13}C NMR of 12 (101 MHz, $CDCl_3$)

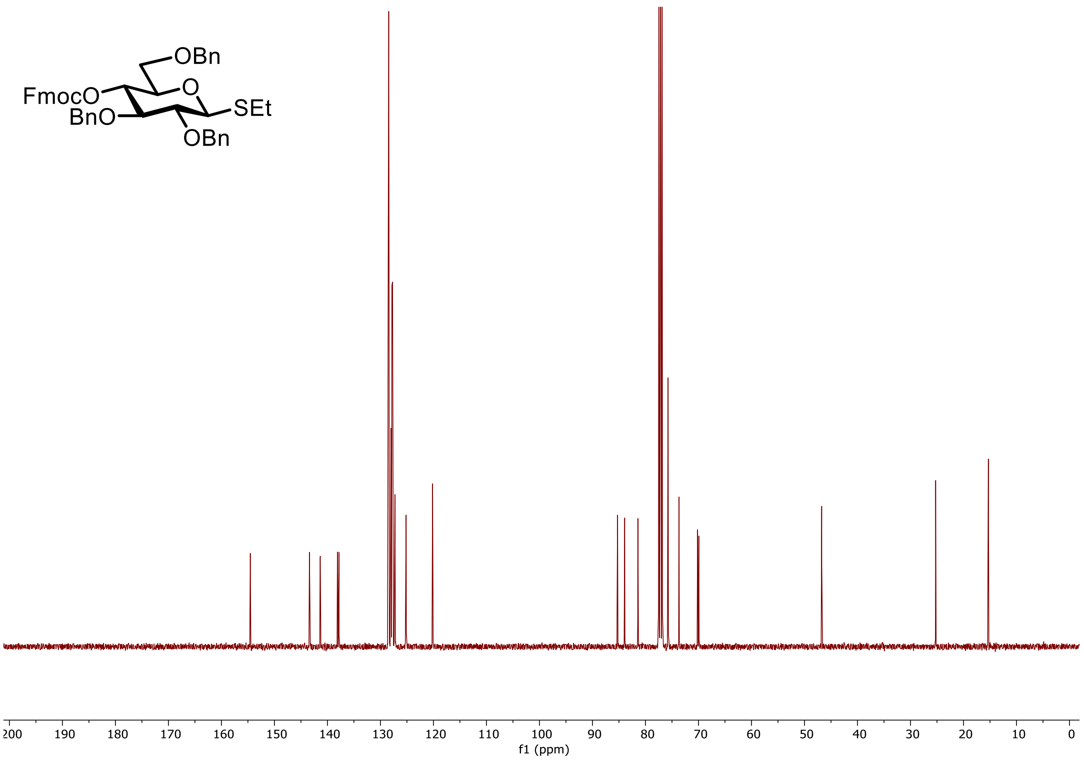

COSY NMR of 12 ($CDCl_3$)

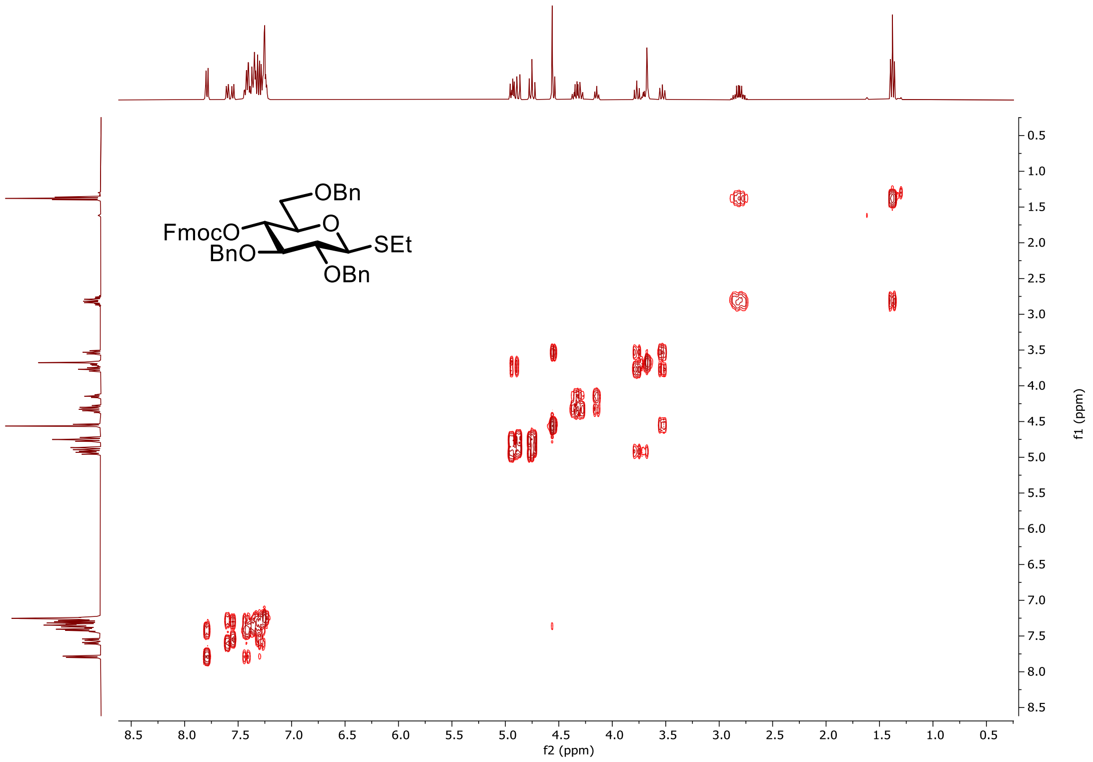

HSQC NMR of 12 ($CDCl_3$)

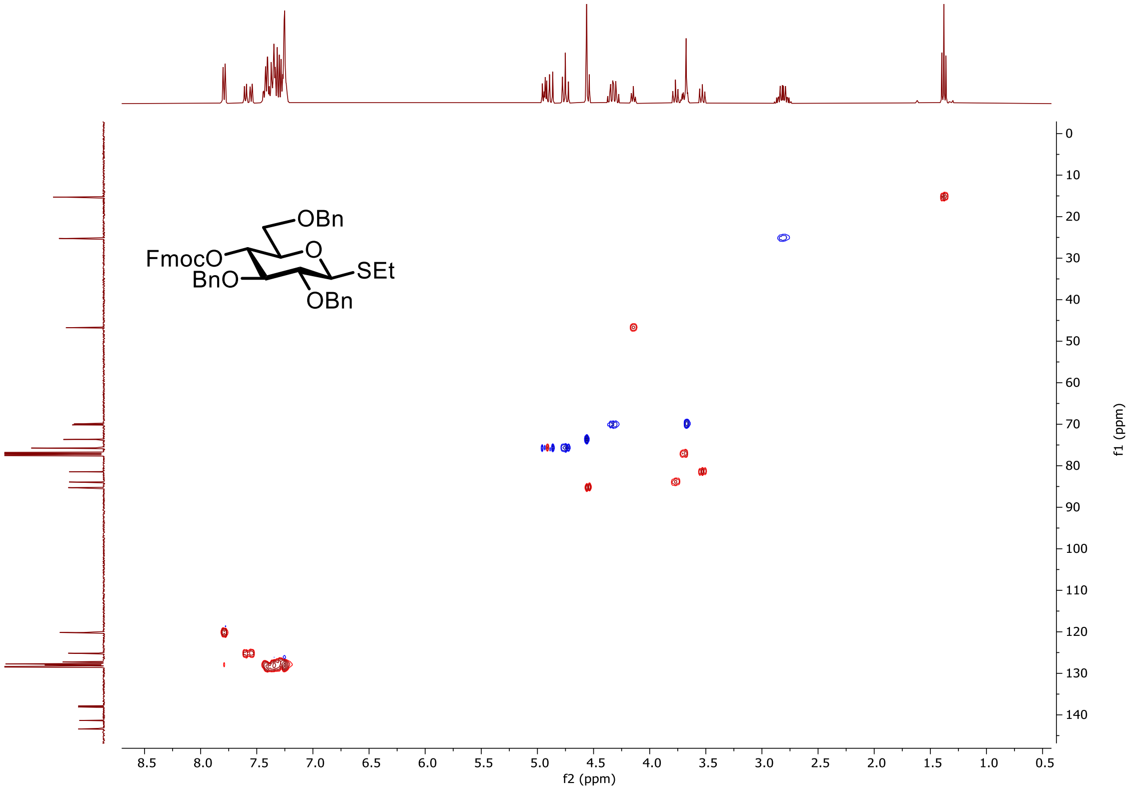

2.2 Synthesis of 13

Ethyl 2,3-di-*O*-benzyl-6-*O*-acetyl-1-thio-β-D-glucopyranoside, 54

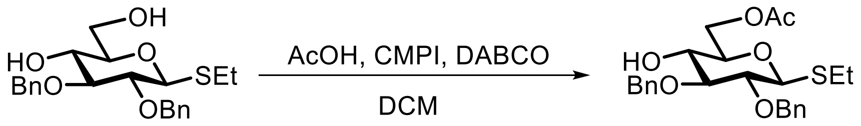

53 **54**

53 was prepared according to previously established procedures. [2]

Ethyl 2,3-di-*O*-benzyl-1-thio-β-D-glucopyranoside **53** (850 mg, 2.1 mmol) was dissolved in anhydrous DCM (20 mL), then 2-chloro-1-methylpyridium iodide (CMPI, 1.33 g, 5.2 mmol) and 1,4-diazabicyclo[2.2.2]octane (DABCO, 942 mg, 8.4 mmol) were added at room temperature. The reaction mixture was cooled to -15 °C, and acetic acid (AcOH, 132 μL, 2.3 mmol) was added slowly. After 3 h, DCM (20 mL) was added and the organic phase was washed with aqueous saturated $NaHCO_3$ (50 mL), and the water phase was extracted with DCM (20 mL). The obtained organic layers were combined and dried over Na_2SO_4, filtered, and evaporated. The resulting crude product was purified by column chromatography (Hexane : EtOAc = 6:1→2:1) to give **54** as a white solid (570 mg, 61%). ^{1}H NMR (400 MHz, $CDCl_3$) δ 7.44 – 7.28 (m, 10H), 4.95 (dd, *J* = 10.8, 2.8 Hz, 2H), 4.76 (appt, *J* = 11.2 Hz, 2H), 4.49 (d, *J* = 9.6 Hz, 1H), 4.37 (dd, *J* = 12.1, 4.8 Hz, 1H), 4.30 (dd, *J* = 12.1, 2.1 Hz, 1H), 3.57 – 3.37 (m, 4H), 2.86 – 2.69 (m, 2H), 2.66 – 2.52 (br. s, 1H), 2.09 (s, 3H), 1.34 (t, *J* = 7.4 Hz, 3H); ^{13}C NMR (101 MHz, $CDCl_3$) δ 171.60, 138.37, 137.82, 128.72, 128.50, 128.43, 128.08, 128.04, 85.71, 85.41, 81.31, 77.48, 75.58, 75.52, 70.05, 63.60, 25.34, 20.96, 15.18; $[\alpha]_D^{25}$ -47.99 (c = 1, $CHCl_3$); IR (neat) ν_{max} = 1742, 1293, 1060 cm^{-1}; m/z (HRMS$^+$) $[M + Na]^+$ 469.1658 ($C_{24}H_{30}O_6SNa^+$ requires 469.1655).

^{1}H NMR of 54 (400 MHz, $CDCl_3$)

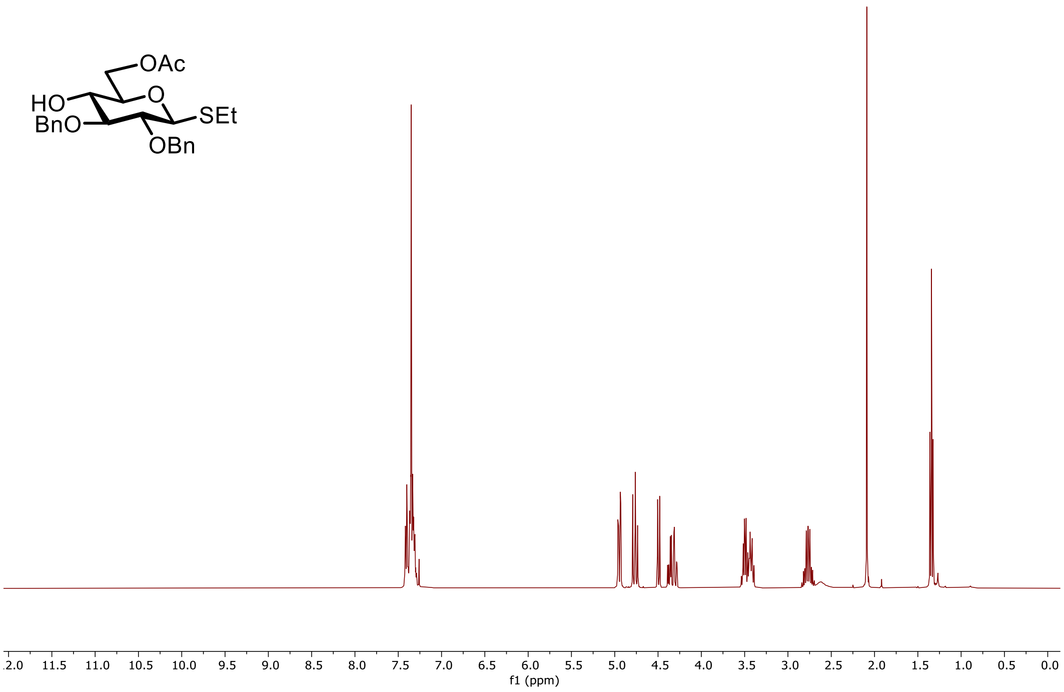

^{13}C NMR of 54 (101 MHz, $CDCl_3$)

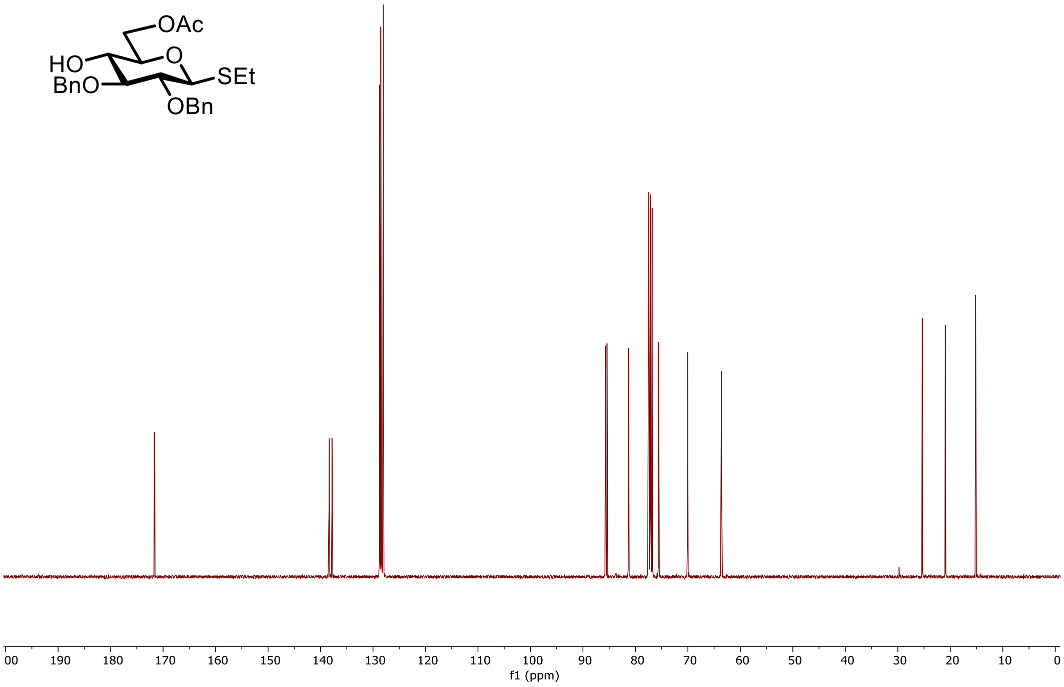

COSY NMR of 54 ($CDCl_3$)

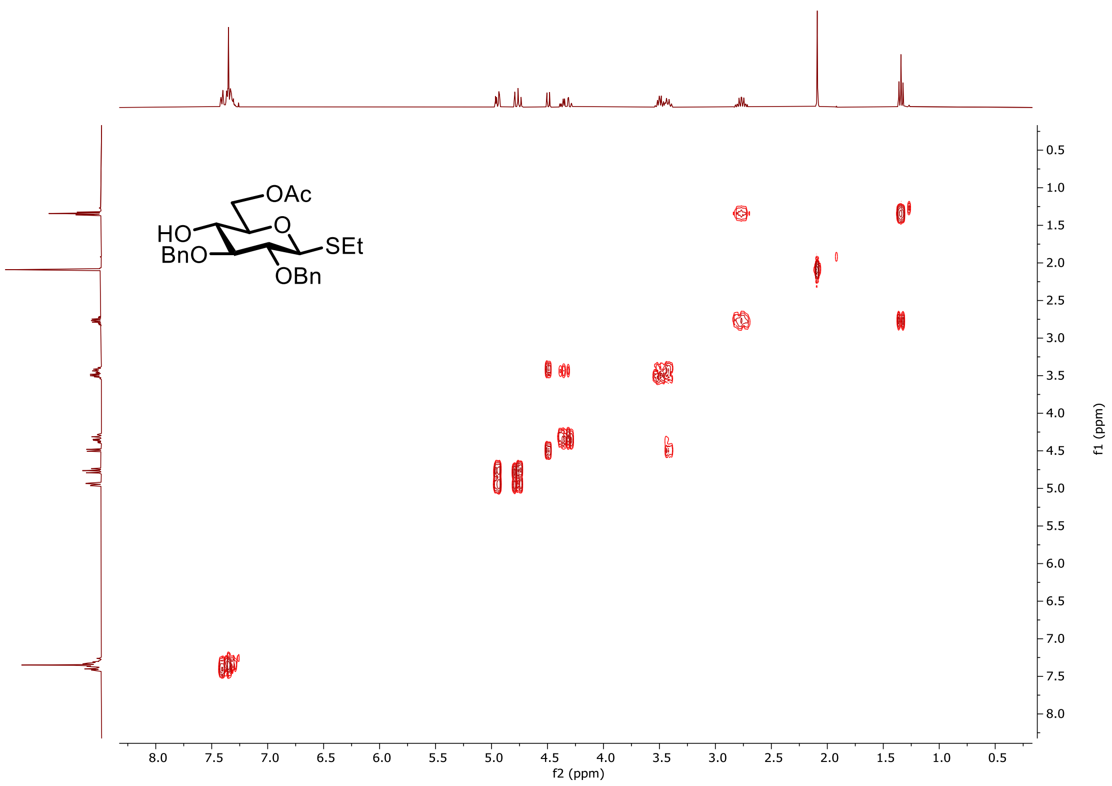

HSQC NMR of 54 ($CDCl_3$)

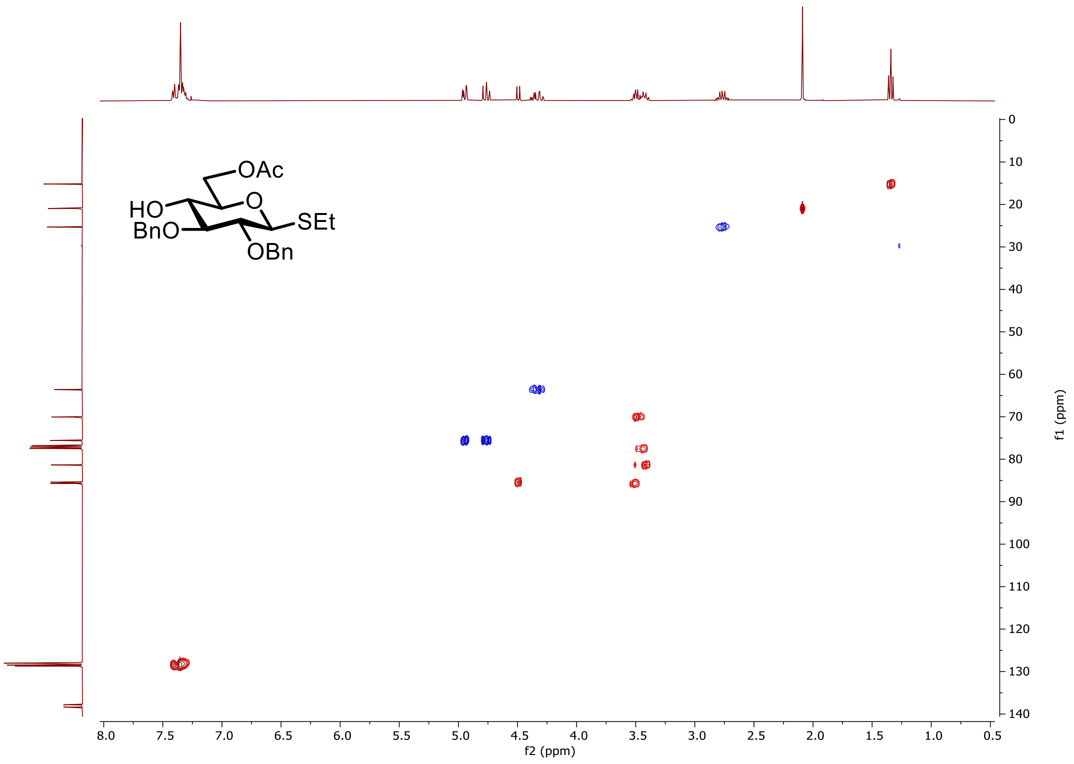

Ethyl 2,3-di-*O*-benzyl-4-*O*-(9-fluorenylmethoxycarbonyl)-6-*O*-acetyl-1-thio-β-D-glucopyranoside, 13

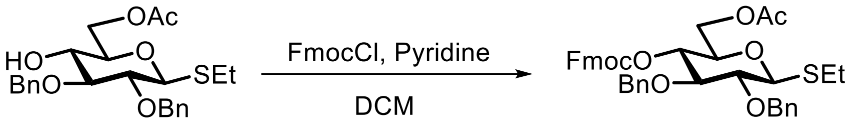

54 **13**

Ethyl 2,3-di-*O*-benzyl-6-*O*-acetyl-1-thio-β-D-glucopyranoside **54** (570 mg, 1.3 mmol) was dissolved in anhydrous DCM (6 mL) and pyridine (1.5 mL) was added. The solution was cooled with an ice bath for 30 min, and fluorenylmethyloxycarbonyl chloride (FmocCl, 828 mg, 3.2 mmol) was added slowly. The reaction was warmed to room temperature and stirred for 6 h. Upon completion, DCM (20 mL) was added and the organic phase was washed with aqueous citric acid (0.5 M, 20 mL). After extracting the water phase with DCM (10 mL), the organic layers were combined and dried over Na_2SO_4, filtered, and evaporated. The resulting crude product was purified by column chromatography (Hexane : EtOAc = 8:1→4:1) to give **13** as a white solid (735 mg, 86%). ^{1}H NMR (400 MHz, $CDCl_3$) δ 7.84 – 7.76 (m, 2H), 7.64 (m, 1H), 7.60 (m, 1H), 7.47 – 7.27 (m, 9H), 7.25 (s, 5H), 5.00 – 4.92 (m, 2H), 4.88 (d, *J* = 11.2 Hz, 1H), 4.77 (d, *J* = 10.2 Hz, 1H), 4.72 (d, *J* = 11.2 Hz, 1H), 4.58 – 4.46 (m, 2H), 4.41 – 4.29 (m, 2H), 4.28 – 4.17 (m, 2H), 3.76 (appt, *J* = 9.1 Hz, 1H), 3.70 (ddd, *J* = 10.1, 5.4, 2.6 Hz, 1H), 3.55 (dd, *J* = 9.8, 8.8 Hz, 1H), 2.94 – 2.69 (m, 2H), 2.11 (s, 3H), 1.39 (t, *J* = 7.4 Hz, 3H); ^{13}C NMR (101 MHz, $CDCl_3$) δ 170.76, 154.39, 143.30, 143.16, 141.32, 141.30, 137.89, 137.69, 128.51, 128.46, 128.37, 128.08, 127.98, 127.96, 127.78, 127.75, 127.26, 125.16, 125.02, 120.14, 120.13, 85.41, 83.71, 81.24, 75.69, 75.67, 75.45, 74.47, 70.28, 62.67, 46.71, 25.28, 20.87, 15.18; $[\alpha]_D^{25}$ -2.62 (c = 1, $CHCl_3$); IR (neat) ν_{max} = 1748, 1257, 740 cm^{-1}; m/z (HRMS$^+$) [M + Na]$^+$ 691.2340 ($C_{39}H_{40}O_8SNa^+$ requires 691.2336).

^{1}H NMR of 13 (400 MHz, $CDCl_3$)

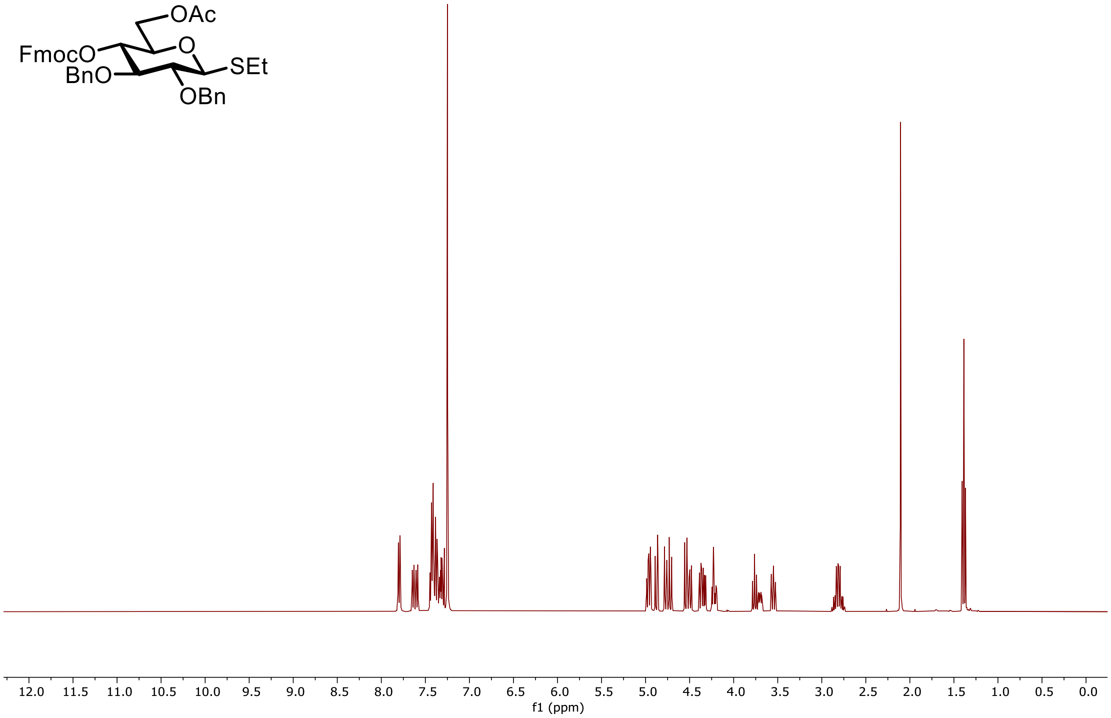

^{13}C NMR of 13 (101 MHz, $CDCl_3$)

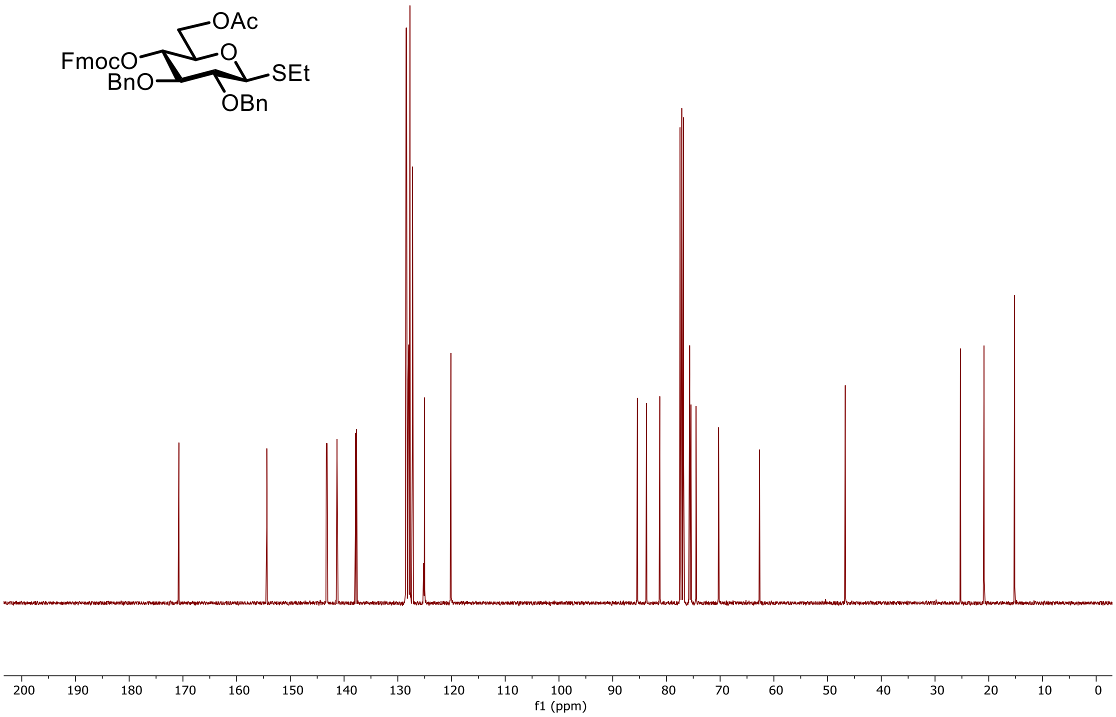

COSY NMR of 13 ($CDCl_3$)

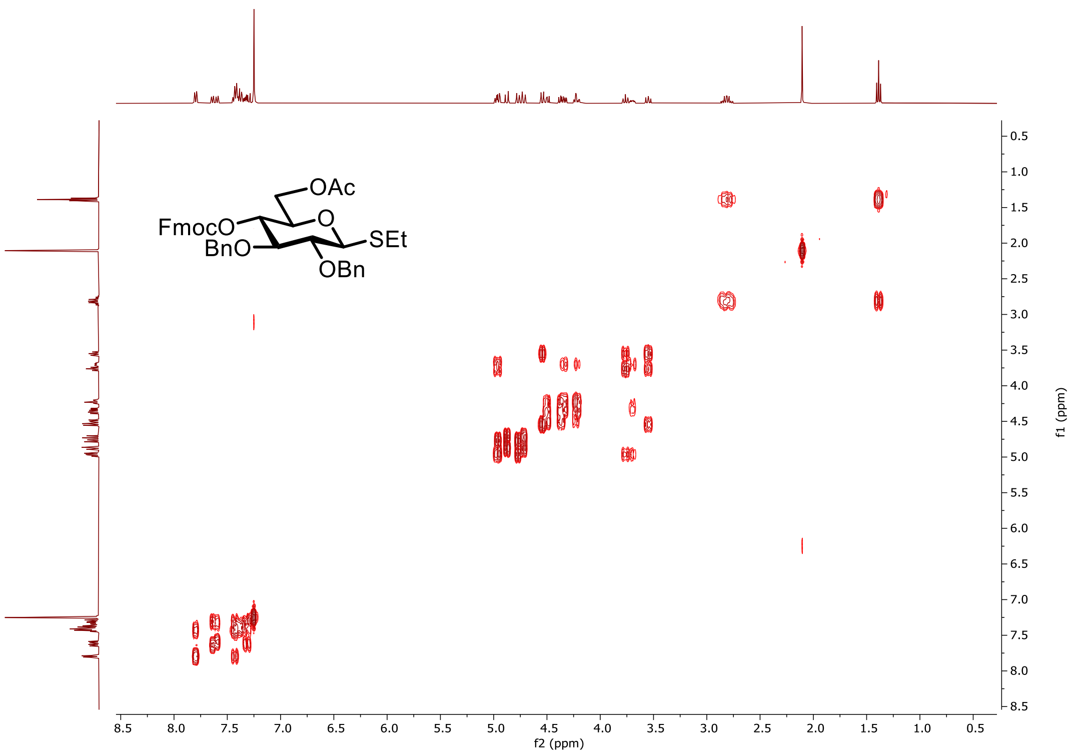

HSQC NMR of 13 ($CDCl_3$)

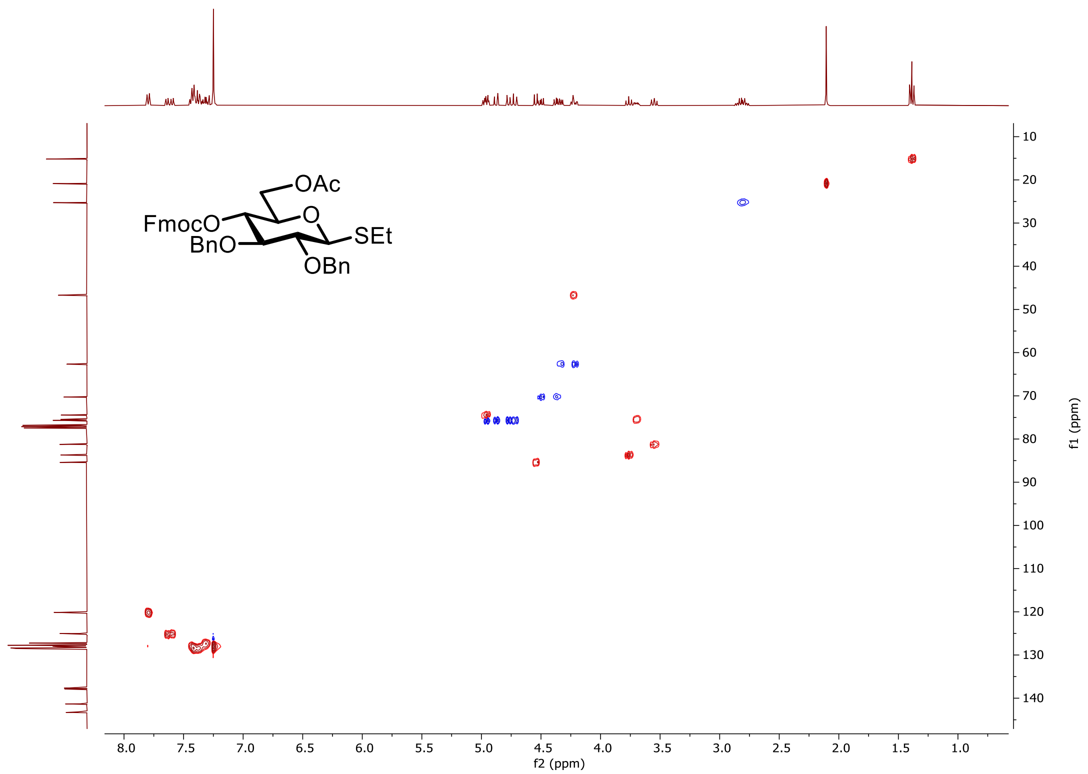

2.3 Synthesis of 14

Ethyl 2,3-di-*O*-benzyl-6-*O*-formyl-1-thio-β-D-glucopyranoside, 55

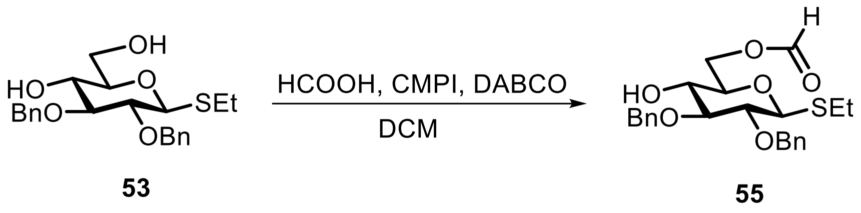

53 was prepared according to previously established procedures. [2]

Ethyl 2,3-di-*O*-benzyl-1-thio-β-D-glucopyranoside **53** (500 mg, 1.2 mmol) was dissolved in anhydrous DCM (10 mL), then 2-chloro-1-methylpyridium iodide (CMPI, 792 mg, 3.1 mmol) and 1,4-diazabicyclo[2.2.2]octane (DABCO, 561 mg, 5.0 mmol) were added at room temperature. The reaction mixture was cooled to -15 °C, and formic acid (52 μL, 1.3 mmol) was added slowly. After 3 h, DCM (10 mL) was added and the organic phase was washed with aqueous saturated $NaHCO_3$ (20 mL), and the water phase was extracted with DCM (20 mL). The obtained organic layers were combined and dried over Na_2SO_4, filtered, and evaporated. The resulting crude product was purified by column chromatography (Hexane : EtOAc = 6:1→2:1) to give **55** as a white solid (330 mg, 62%). ^{1}H NMR (400 MHz, $CDCl_3$) δ 8.11 (s, 1H), 7.40 (m, 10H), 4.99 (dd, *J* = 10.8, 6.4 Hz, 2H), 4.77 (d, *J* = 10.8 Hz, 2H), 4.53 (d, *J* = 9.5 Hz, 1H), 4.43 (m, 2H), 3.57 – 3.41 (m, 4H), 2.89 – 2.70 (m, 2H), 2.55 (br. s, 1H), 1.37 (t, *J* = 7.4 Hz, 3H);^{13}C NMR (101 MHz, $CDCl_3$) δ 161.18, 138.33, 137.77, 128.78, 128.52, 128.44, 128.15, 128.06, 128.00, 85.71, 85.31, 81.35, 77.05, 75.50, 69.87, 63.07, 25.23, 15.16; $[\alpha]_D^{25}$ -44.51 (c = 1, $CHCl_3$); IR (neat) ν_{max} = 1725, 1060, 699 cm^{-1}; m/z ($HRMS^+$) $[M + Na]^+$ 455.1490 ($C_{23}H_{28}O_6SNa^+$ requires 455.1499).

^{1}H NMR of 55 (400 MHz, $CDCl_3$)

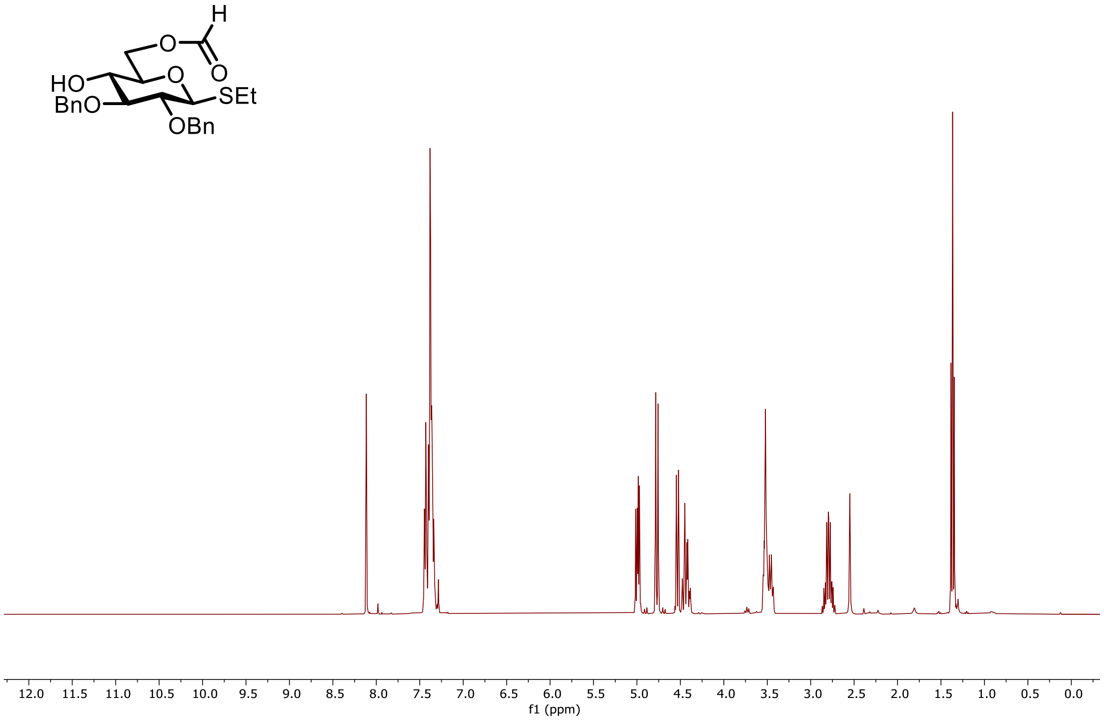

^{13}C NMR of 55 (101 MHz, $CDCl_3$)

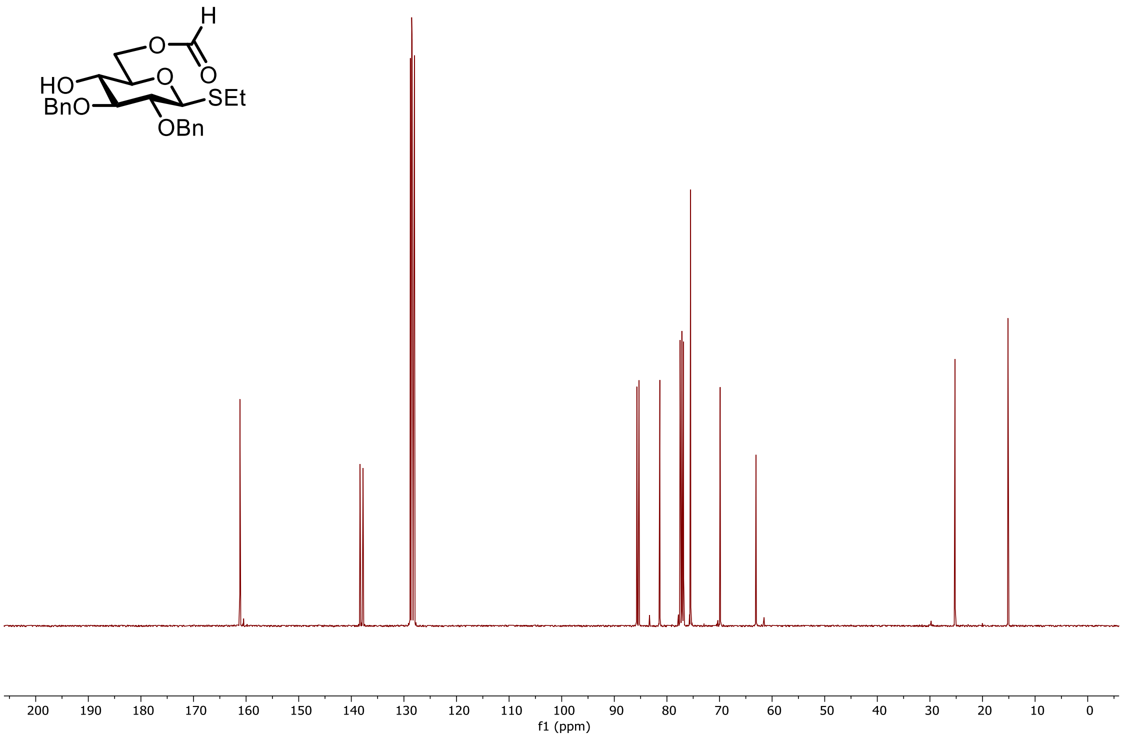

COSY NMR of 55 ($CDCl_3$)

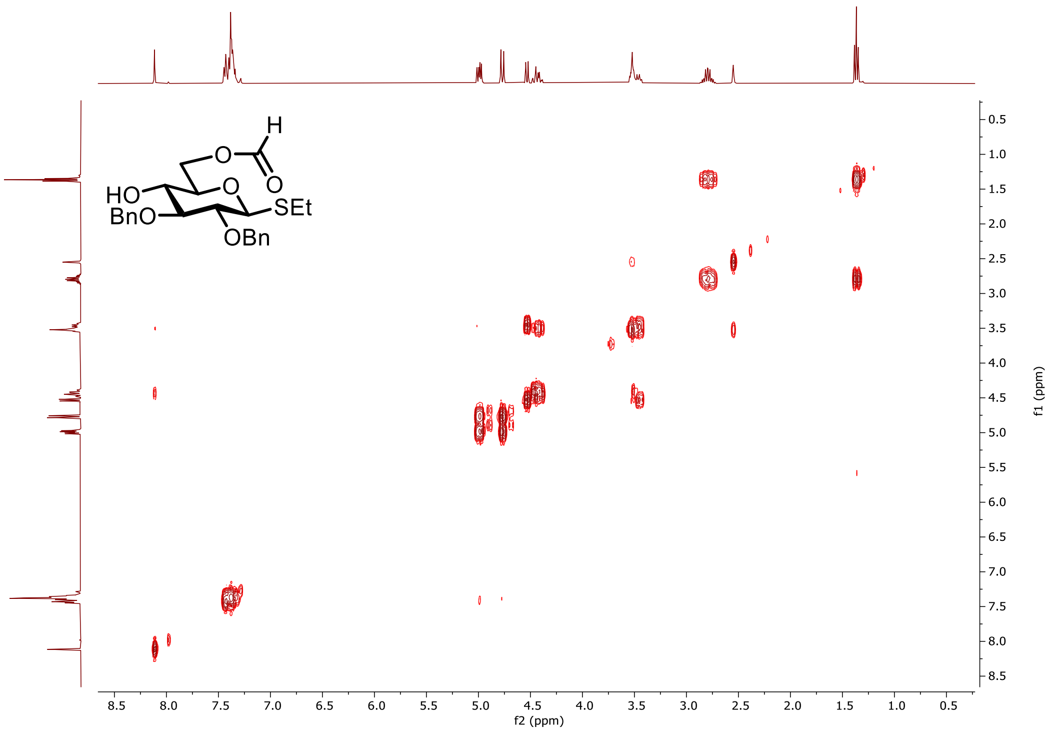

HSQC NMR of 55 ($CDCl_3$)

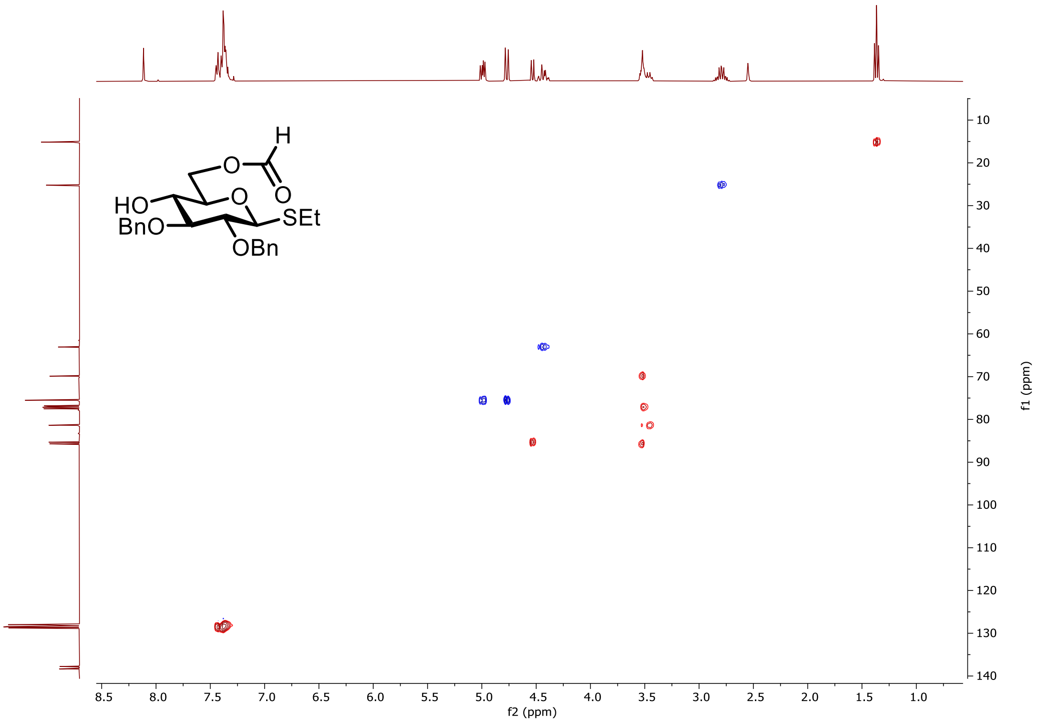

Ethyl 2,3-di-*O*-benzyl-4-*O*-(9-fluorenylmethoxycarbonyl)-6-*O*-formyl-1-thio-β-D-glucopyranoside, 14

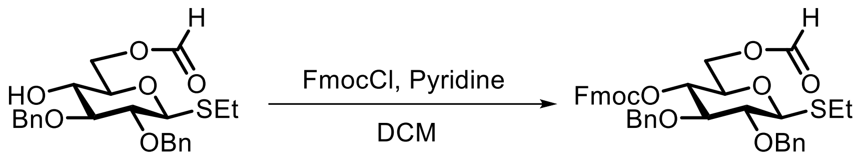

55 **14**

Ethyl 2,3-di-*O*-benzyl-6-*O*-formyl-1-thio-β-D-glucopyranoside **55** (330 mg, 0.76 mmol) was dissolved in anhydrous DCM (5 mL) and pyridine (1.2mL) was added. The solution was cooled with an ice bath for 30 min, and fluorenylmethyloxycarbonyl chloride (FmocCl, 493 mg, 1.9 mmol) was added slowly. The reaction was warmed to room temperature and stirred for 6 h. Upon completion, DCM (20 mL) was added and the organic phase was washed with aqueous citric acid (0.5 M, 20 mL). After extracting the water phase with DCM (10 mL), the organic layers were combined and dried over Na_2SO_4, filtered, and evaporated. The resulting crude product was purified by column chromatography (Hexane : EtOAc = 8:1→4:1) to give **14** as a white solid (375 mg, 75%). ^{1}H NMR (400 MHz, $CDCl_3$) δ 8.05 (s, 1H), 7.77 (d, *J* = 7.6 Hz, 2H), 7.66 – 7.52 (m, 2H), 7.46 – 7.25 (m, 9H), 7.23 (s, 5H), 4.98 – 4.81 (m, 3H), 4.72 (dd, *J* = 19.1, 10.7 Hz, 2H), 4.51 (m, 2H), 4.36 (dd, *J* = 10.5, 7.1 Hz, 1H), 4.33 – 4.28 (m, 2H), 4.20 (appt, *J* = 7.0 Hz, 1H), 3.78 – 3.65 (m, 2H), 3.55 – 3.46 (m, 1H), 2.88 – 2.68 (m, 2H), 1.35 (t, *J* = 7.5 Hz, 3H); ^{13}C NMR (101 MHz, $CDCl_3$) δ 160.52, 154.36, 143.17, 143.13, 141.31, 137.84, 137.64, 128.48, 128.42, 128.35, 128.05, 127.97, 127.96, 127.76, 127.72, 127.23, 125.09, 124.95, 120.14, 120.12, 85.27, 83.56, 81.13, 75.65, 75.61, 75.19, 74.50, 70.22, 62.11, 46.71, 25.10, 15.11; $[\alpha]_D^{25}$ -1.37 (c = 1, $CHCl_3$); IR (neat) ν_{max} = 1752, 1728, 1256, 740 cm^{-1}; m/z (HRMS$^+$) [M + Na]$^+$ 677.2203 ($C_{38}H_{38}O_8SNa^+$ requires 677.2180).

^{1}H NMR of 14 (400 MHz, $CDCl_3$)

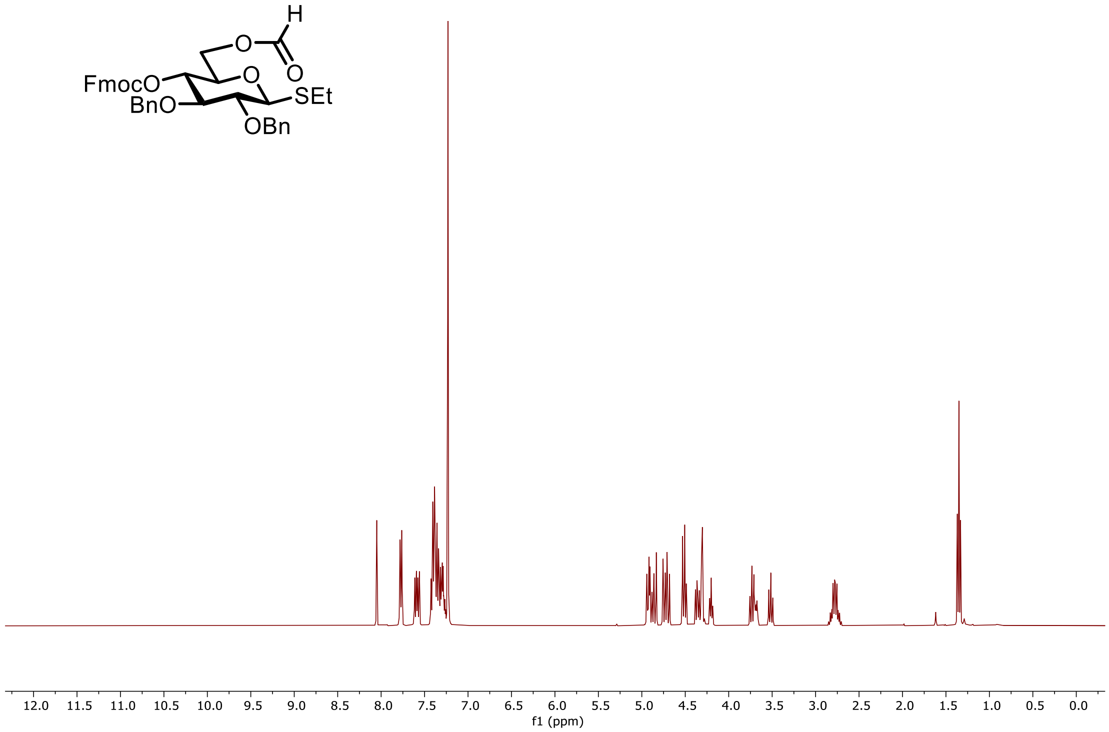

^{13}C NMR of 14 (101 MHz, $CDCl_3$)

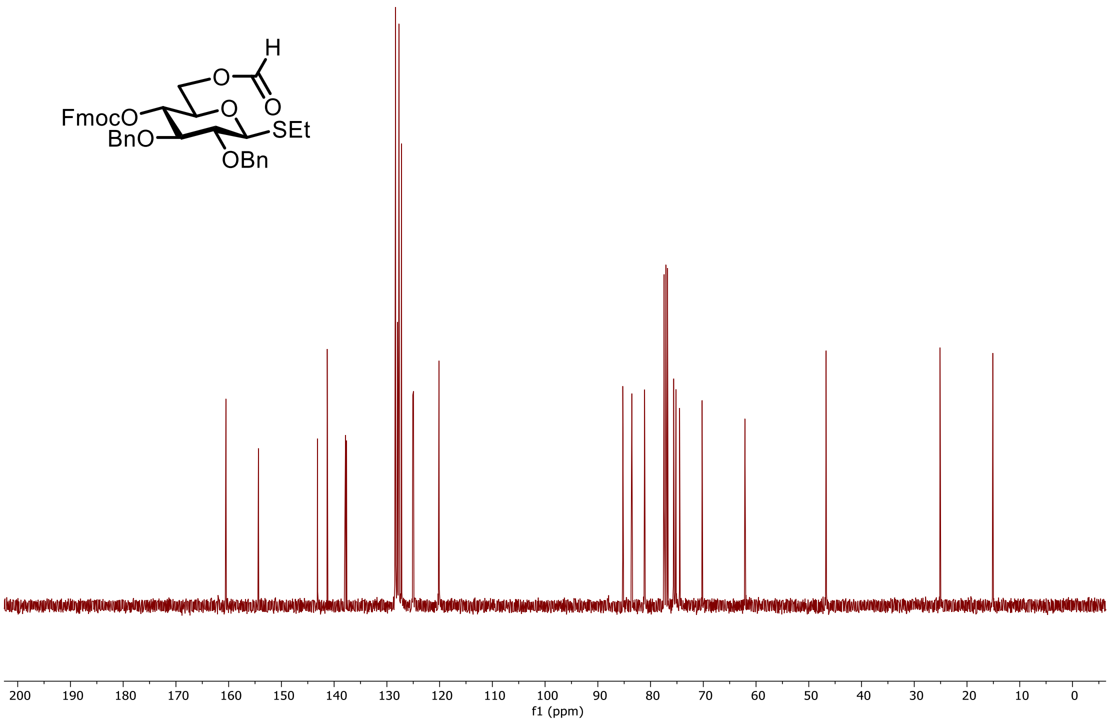

COSY NMR of 14 ($CDCl_3$)

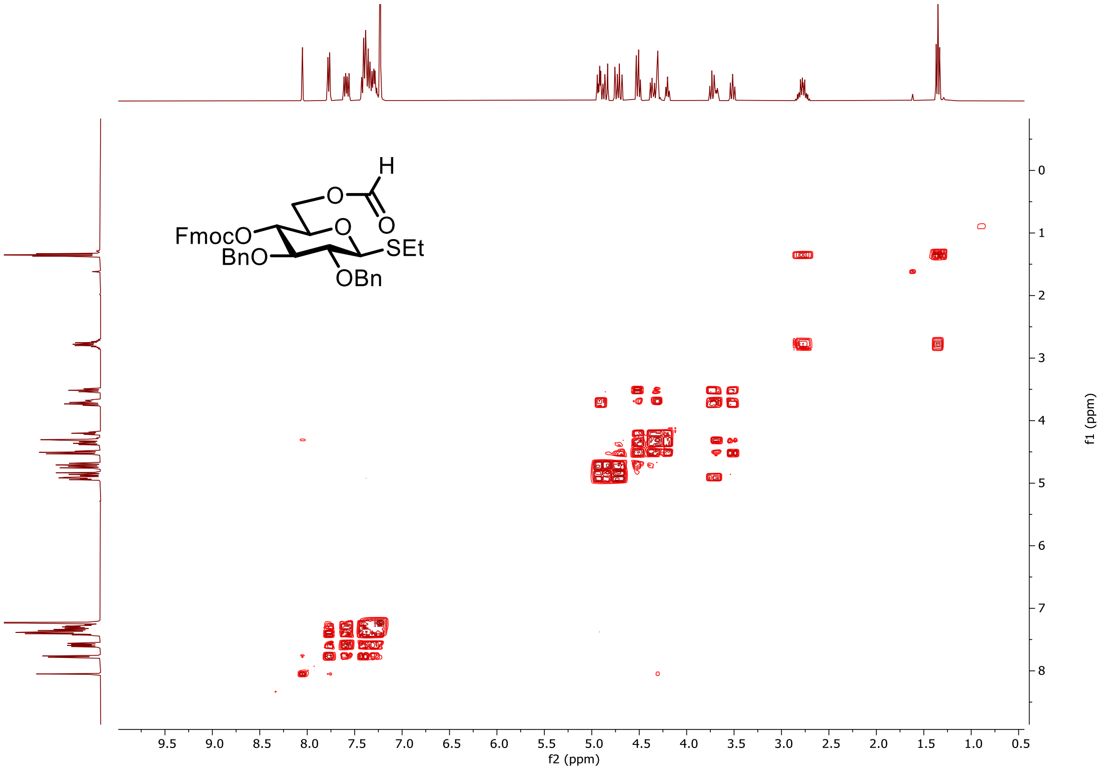

HSQC NMR of 14 ($CDCl_3$)

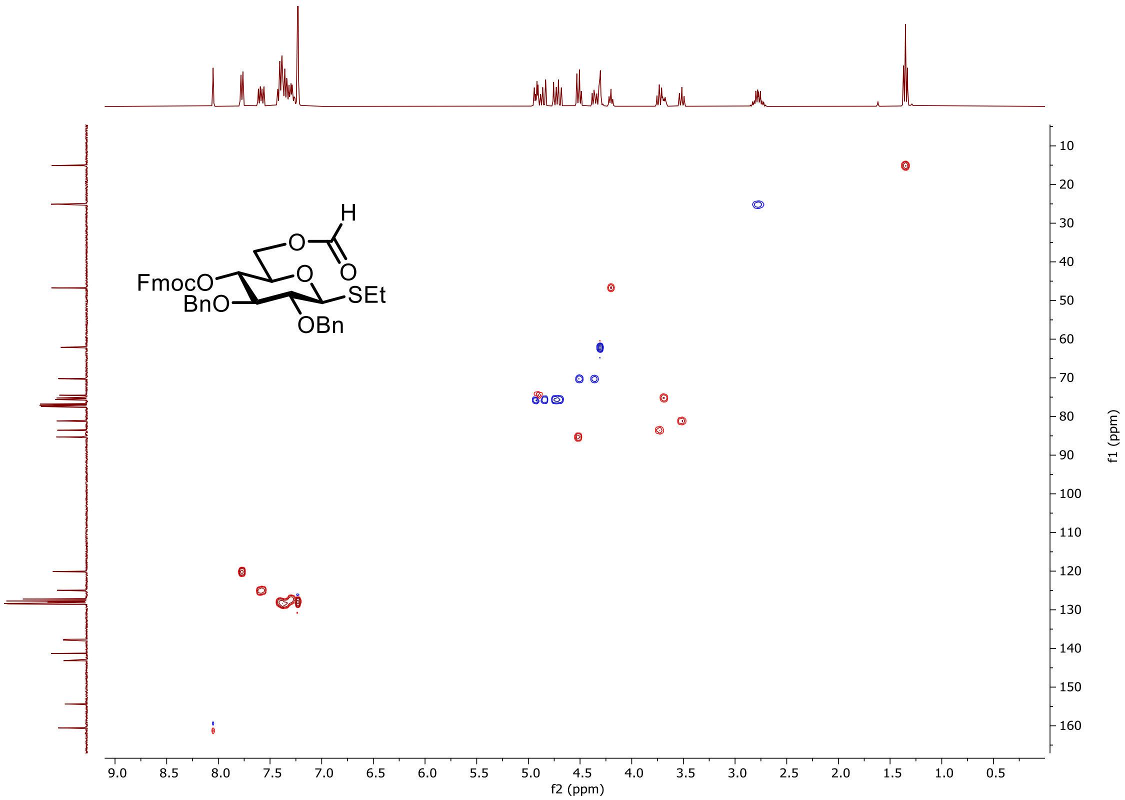

2.4 Synthesis of 15

Ethyl 2,3-di-*O*-benzyl-6-*O*-propionyl-1-thio-β-D-glucopyranoside, 56

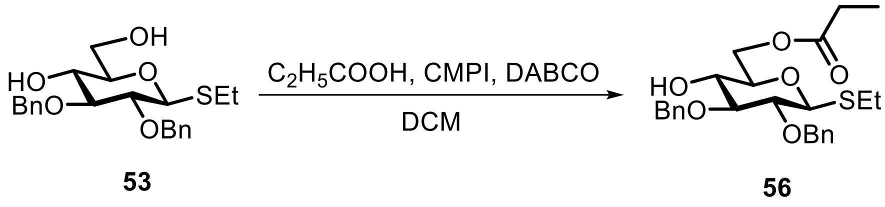

53 was prepared according to previously established procedures. [2]

Ethyl 2,3-di-*O*-benzyl-1-thio-β-D-glucopyranoside **53** (610 mg, 1.5 mmol) was dissolved in anhydrous DCM (12 mL), then 2-chloro-1-methylpyridium iodide (CMPI, 964 mg, 3.8 mmol) and 1,4-diazabicyclo[2.2.2]octane (DABCO, 673 mg, 6.0 mmol) were added at room temperature. The reaction mixture was cooled to -15 °C, and propionic acid (123 μL, 1.6 mmol) was added slowly. After 3 h, DCM (10 mL) was added and the organic phase was washed with aqueous saturated $NaHCO_3$ (20 mL), and the water phase was extracted with DCM (20 mL). The obtained organic layers were combined and dried over Na_2SO_4, filtered, and evaporated. The resulting crude product was purified by column chromatography (Hexane : EtOAc = 6:1→2:1) to give **56** as a white solid (599 mg, 87%). ^{1}H NMR (400 MHz, $CDCl_3$) δ 7.48 – 7.24 (m, 10H), 4.95 (d, *J* = 10.8 Hz, 2H), 4.84 (d, *J* = 11.3 Hz, 1H), 4.76 (d, *J* = 10.3 Hz, 1H), 4.51 (d, *J* = 9.7 Hz, 1H), 4.42 – 4.26 (m, 2H), 3.62 – 3.34 (m, 4H), 3.09 (br. s, 1H), 2.77 (dtt, *J* = 20.1, 12.6, 7.4 Hz, 2H), 2.38 (q, *J* = 7.6 Hz, 2H), 1.36 (t, *J* = 7.4 Hz, 3H), 1.16 (t, *J* = 7.6 Hz, 3H); ^{13}C NMR (101 MHz, $CDCl_3$) δ 174.94, 138.49, 137.94, 128.62, 128.45, 128.40, 127.95, 85.85, 85.24, 81.31, 77.62, 75.53, 75.45, 70.30, 63.67, 27.49, 25.19, 15.24, 9.12; $[\alpha]_D^{25}$ -52.34 (c = 1, $CHCl_3$); IR (neat) ν_{max} = 1738, 1062, 698 cm^{-1}; m/z ($HRMS^+$) $[M + Na]^+$ 483.1808 ($C_{25}H_{32}O_6SNa^+$ requires 483.1812).

^{1}H NMR of 56 (400 MHz, $CDCl_3$)

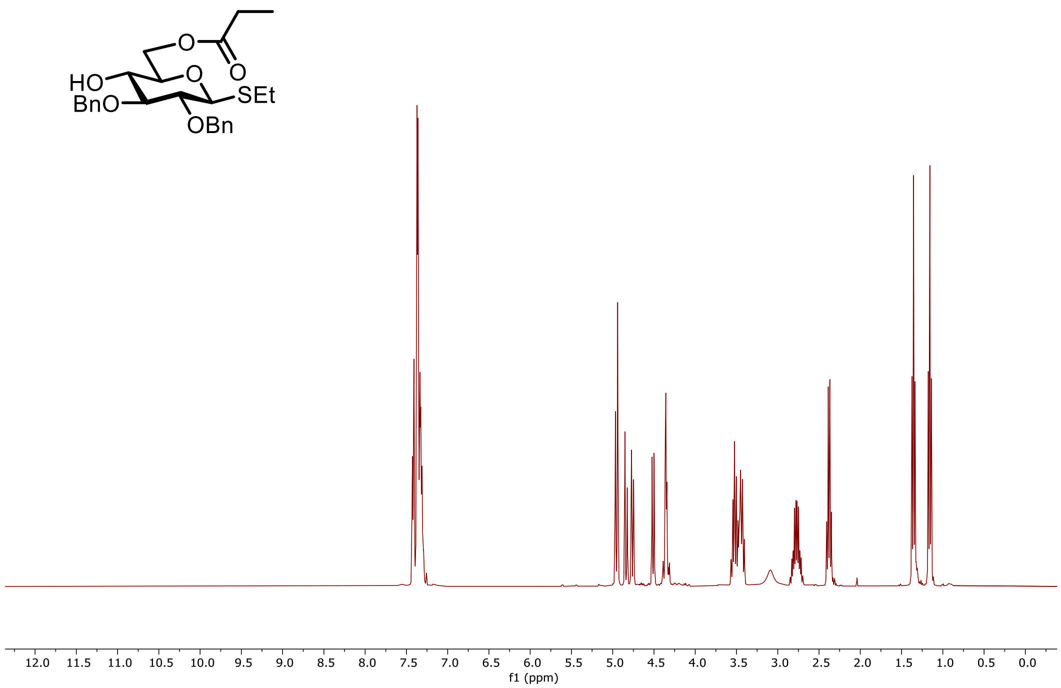

^{13}C NMR of 56 (101 MHz, $CDCl_3$)

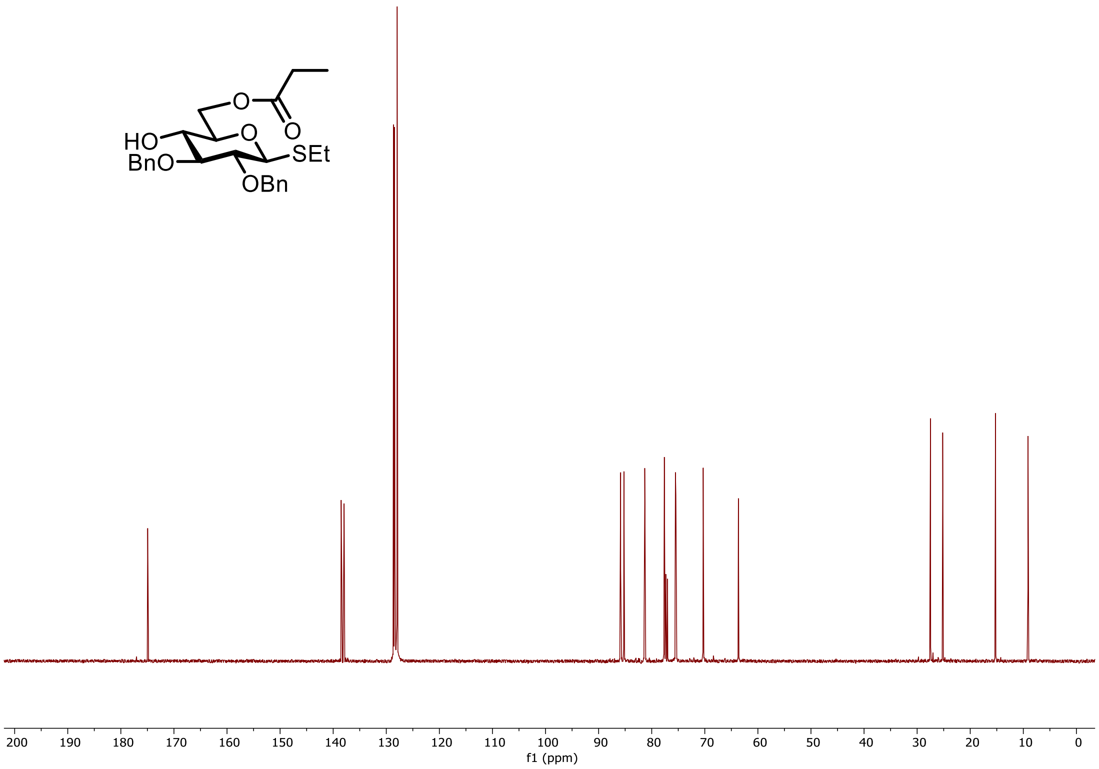

COSY NMR of 56 ($CDCl_3$)

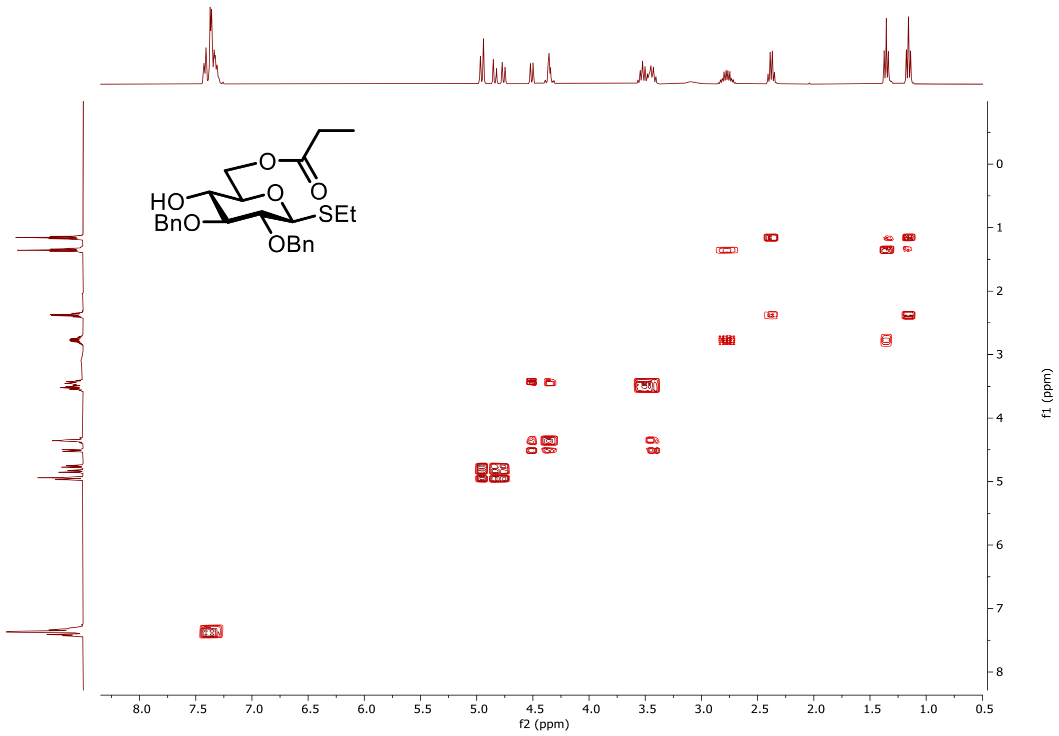

HSQC NMR of 56 ($CDCl_3$)

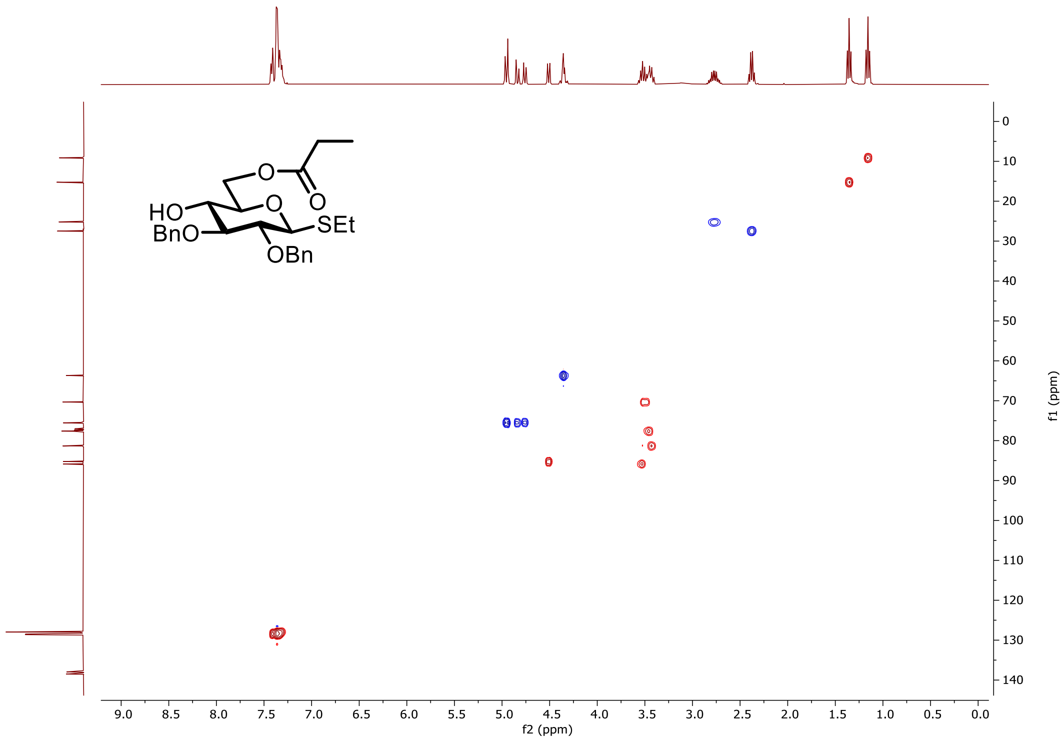

Ethyl 2,3-di-*O*-benzyl-4-*O*-(9-fluorenylmethoxycarbonyl)-6-*O*-propionyl-1-thio-β-D-glucopyranoside, 15

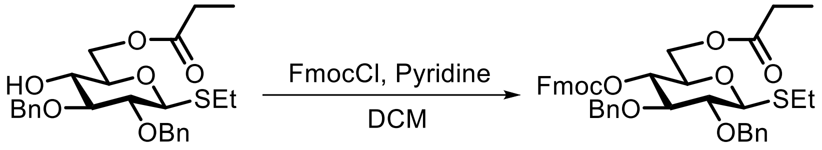

56 **15**

Ethyl 2,3-di-*O*-benzyl-6-*O*-propionyl-1-thio-β-D-glucopyranoside **56** (599 mg, 1.3 mmol) was dissolved in anhydrous DCM (6 mL) and pyridine (1.5 mL) was added. The solution was cooled with an ice bath for 30 min, and fluorenylmethyloxycarbonyl chloride (FmocCl, 982 mg, 3.8 mmol) was added slowly. The reaction was warmed to room temperature and stirred for 6 h. Upon completion, DCM (20 mL) was added and the organic phase was washed with aqueous citric acid (0.5 M, 20 mL). After extracting the water phase with DCM (10 mL), the organic layers were combined and dried over Na_2SO_4, filtered, and evaporated. The resulting crude product was purified by column chromatography (Hexane : EtOAc = 8:1→4:1) to give **15** as a white solid (806 mg, 91%). ^{1}H NMR (400 MHz, $CDCl_3$) δ 7.79 (d, *J* = 7.5 Hz, 2H), 7.64 (d, *J* = 7.6 Hz, 1H), 7.59 (d, *J* = 7.5 Hz, 1H), 7.47 – 7.28 (m, 9H), 7.25 (s, 5H), 4.97 (dd, *J* = 10.1, 8.7 Hz, 2H), 4.87 (d, *J* = 11.2 Hz, 1H), 4.74 (dd, *J* = 21.0, 10.7 Hz, 2H), 4.57 – 4.45 (m, 2H), 4.43 – 4.31 (m, 2H), 4.28 – 4.19 (m, 2H), 3.76 (appt, *J* = 9.1 Hz, 1H), 3.69 (ddd, *J* = 10.0, 5.3, 2.5 Hz, 1H), 3.55 (appt, *J* = 9.3 Hz, 1H), 2.80 (dddd, *J* = 20.1, 12.7, 7.4, 5.3 Hz, 2H), 2.39 (qd, *J* = 7.5, 2.4 Hz, 2H), 1.38 (t, *J* = 7.4 Hz, 3H), 1.18 (t, *J* = 7.6 Hz, 3H); ^{13}C NMR (101 MHz, $CDCl_3$) δ 174.10, 154.40, 143.34, 143.20, 141.33, 141.31, 137.93, 137.75, 128.50, 128.43, 128.37, 128.05, 127.97, 127.95, 127.76, 127.27, 125.16, 125.01, 120.15, 120.13, 85.32, 83.73, 81.27, 75.63, 75.56, 74.55, 70.26, 62.57, 46.74, 27.42, 25.18, 15.20, 9.07; $[\alpha]_D^{25}$ -0.35 (c = 1, $CHCl_3$); IR (neat) ν_{max} = 1750, 1256, 740 cm^{-1}; m/z (HRMS$^+$) $[M + Na]^+$ 705.2517 ($C_{40}H_{42}O_8SNa^+$ requires 705.2493).

^1H NMR of 15 (400 MHz, $CDCl_3$)

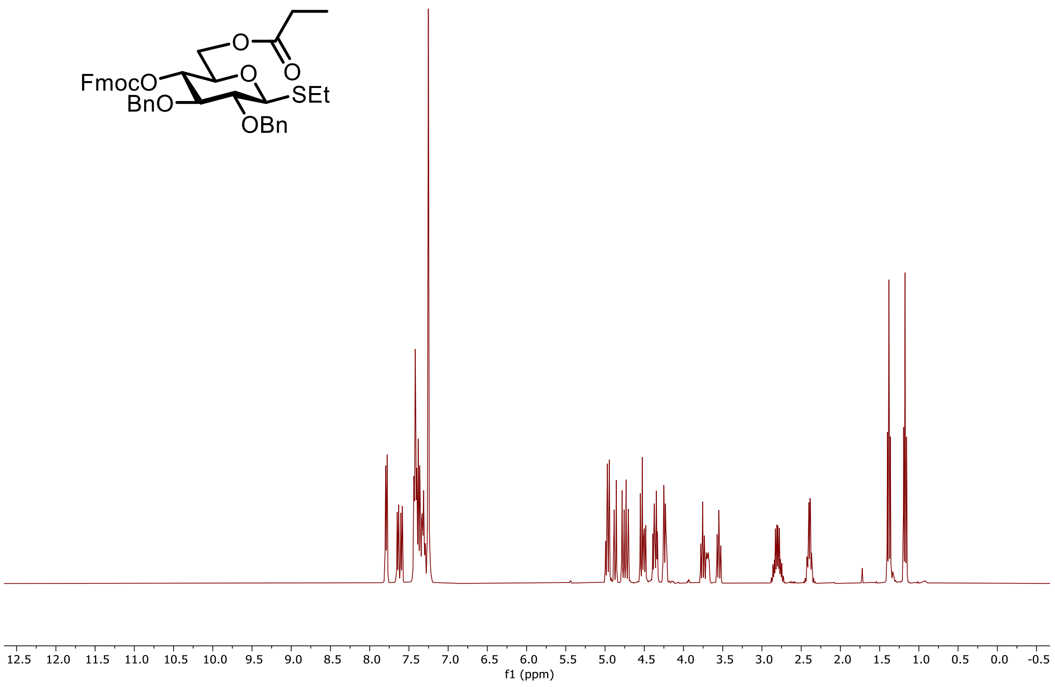

^{13}C NMR of 15 (101 MHz, $CDCl_3$)

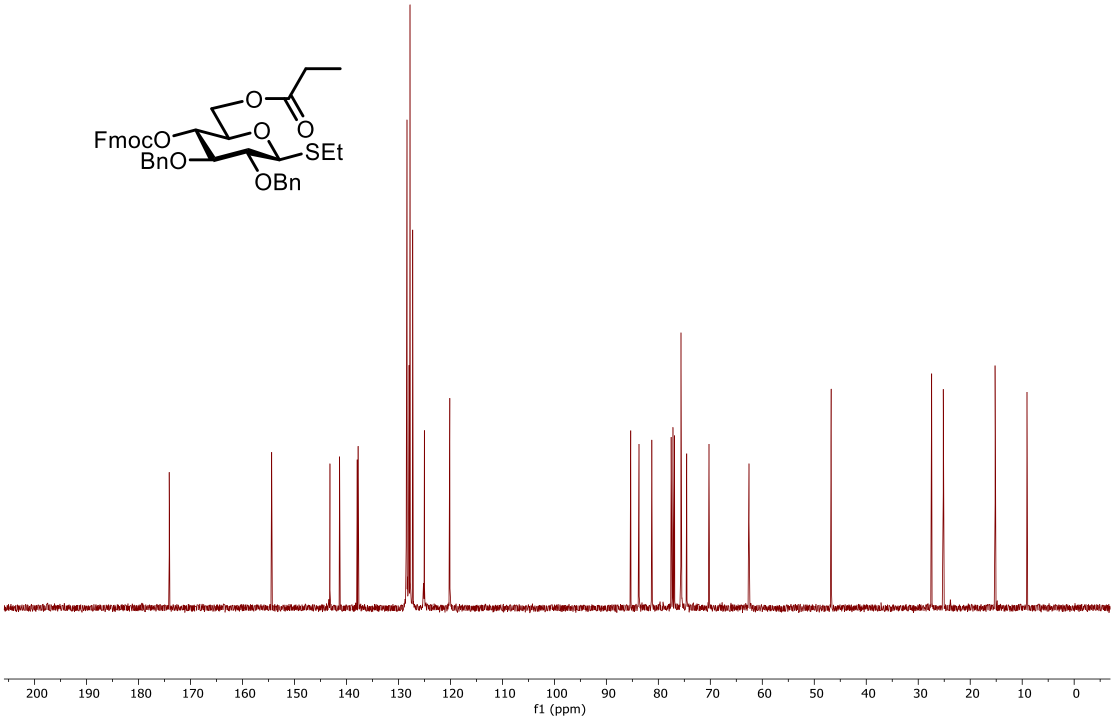

COSY NMR of 15 ($CDCl_3$)

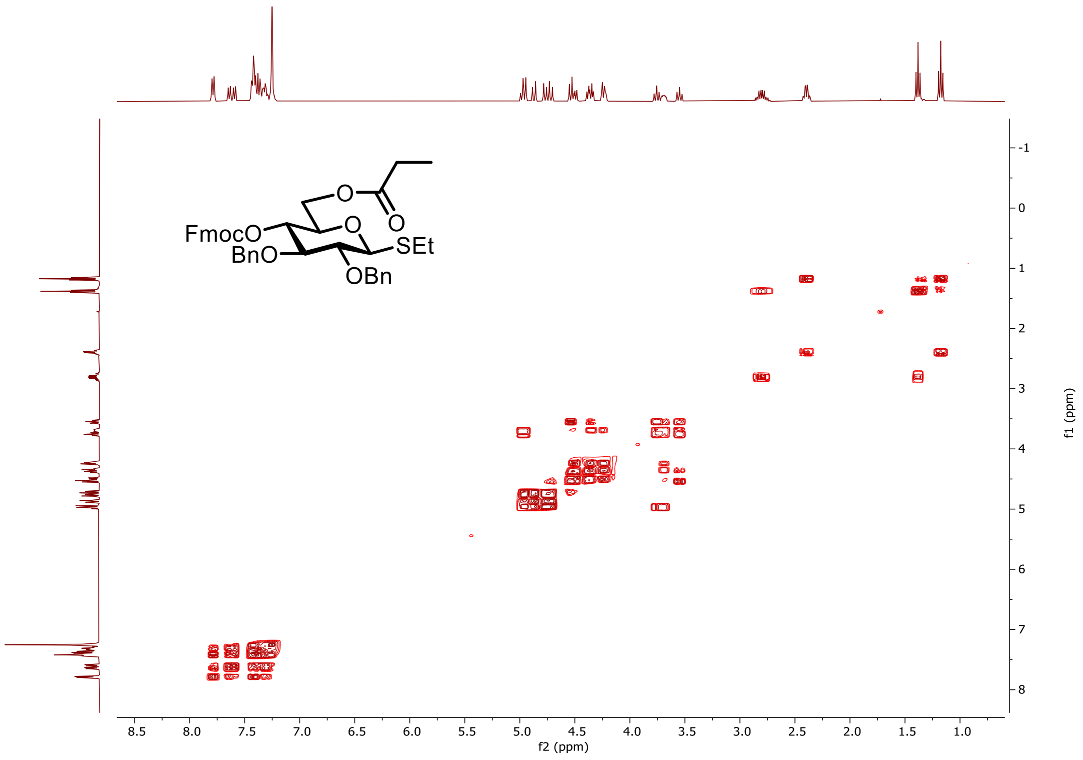

HSQC NMR of 15 ($CDCl_3$)

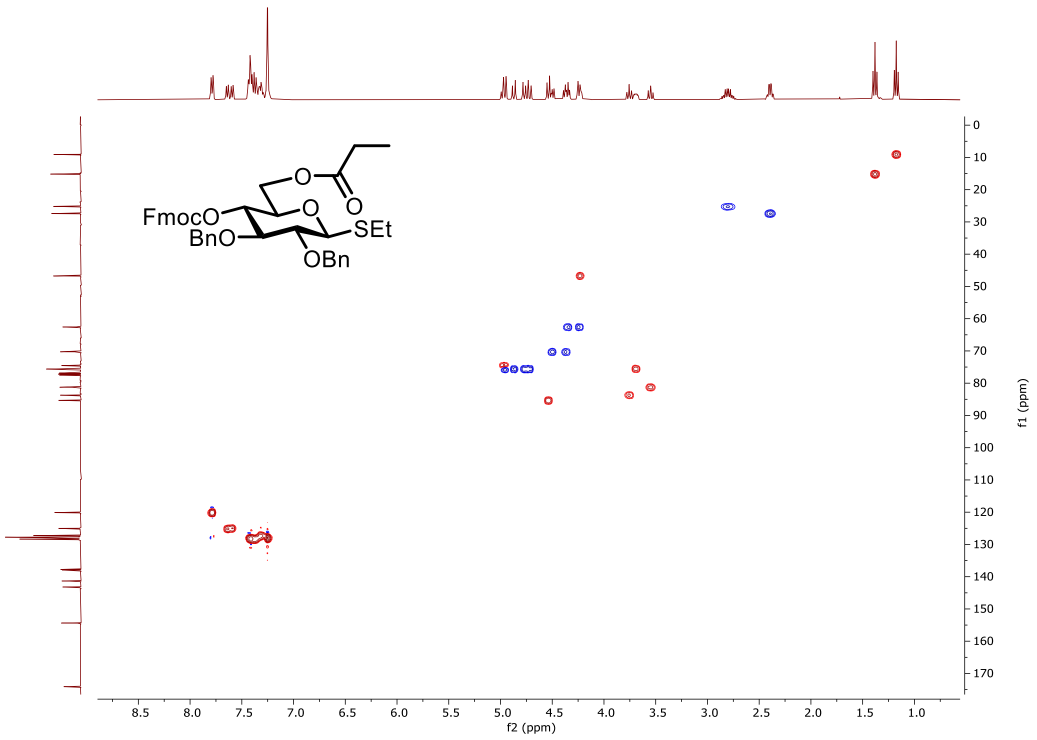

2.5 Synthesis of 16

Ethyl 2,3-di-*O*-benzyl-6-*O*-levulinoyl-1-thio-β-D-glucopyranoside, 57

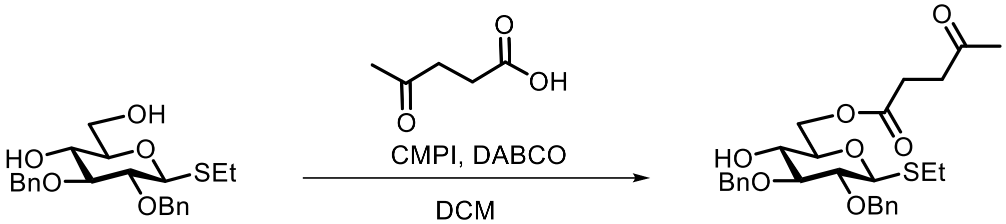

53 **57**

53 was prepared according to previously established procedures. [2]

Ethyl 2,3-di-*O*-benzyl-1-thio-β-D-glucopyranoside **53** (610 mg, 1.5 mmol) was dissolved in anhydrous DCM (12 mL), then 2-chloro-1-methylpyridium iodide (CMPI, 964 mg, 3.8 mmol) and 1,4-diazabicyclo[2.2.2]octane (DABCO, 673 mg, 6.0 mmol) were added at room temperature. The reaction mixture was cooled to -15 °C, and levulinic acid (LevOH, 192 mg, 1.6 mmol) was added slowly. After 3 h, DCM (10 mL) was added and the organic phase was washed with aqueous saturated $NaHCO_3$ (20 mL), and the water phase was extracted with DCM (20 mL). The obtained organic layers were combined and dried over Na_2SO_4, filtered, and evaporated. The resulting crude product was purified by column chromatography (Hexane : EtOAc = 6:1→1:1) to give **57** as a colorless oil (640 mg, 85%). ^{1}H NMR (400 MHz, $CDCl_3$) δ 7.43 – 7.24 (m, 10H), 4.93 (m, 2H), 4.81 (d, *J* = 11.4 Hz, 1H), 4.74 (d, *J* = 10.3 Hz, 1H), 4.48 (d, *J* = 9.7 Hz, 1H), 4.38 (dd, *J* = 12.1, 5.0 Hz, 1H), 4.31 (dd, *J* = 12.1, 2.2 Hz, 1H), 3.58 – 3.43 (m, 2H), 3.46 – 3.37 (m, 2H), 2.95 (br. s, 1H), 3.83 – 3.67 (m, 4H), 2.59 (t, *J* = 6.5 Hz, 2H), 2.17 (s, 3H), 1.33 (t, *J* = 7.4 Hz, 3H); ^{13}C NMR (101 MHz, $CDCl_3$) δ 206.86, 173.12, 138.50, 137.91, 128.59, 128.43, 128.36, 127.94, 127.92, 127.90, 85.77, 85.28, 81.29, 77.53, 75.48, 75.45, 70.15, 63.76, 37.95, 29.87, 27.89, 25.18, 15.17; $[\alpha]_D^{25}$ -32.32 (c = 1, $CHCl_3$); IR (neat) ν_{max} = 1737, 1718, 1060, 699 cm^{-1}; m/z (HRMS$^+$) [M + Na]$^+$ 525.1925 ($C_{27}H_{34}O_7SNa^+$ requires 525.1917).

^{1}H NMR of 57 (400 MHz, CDCl$_3$)

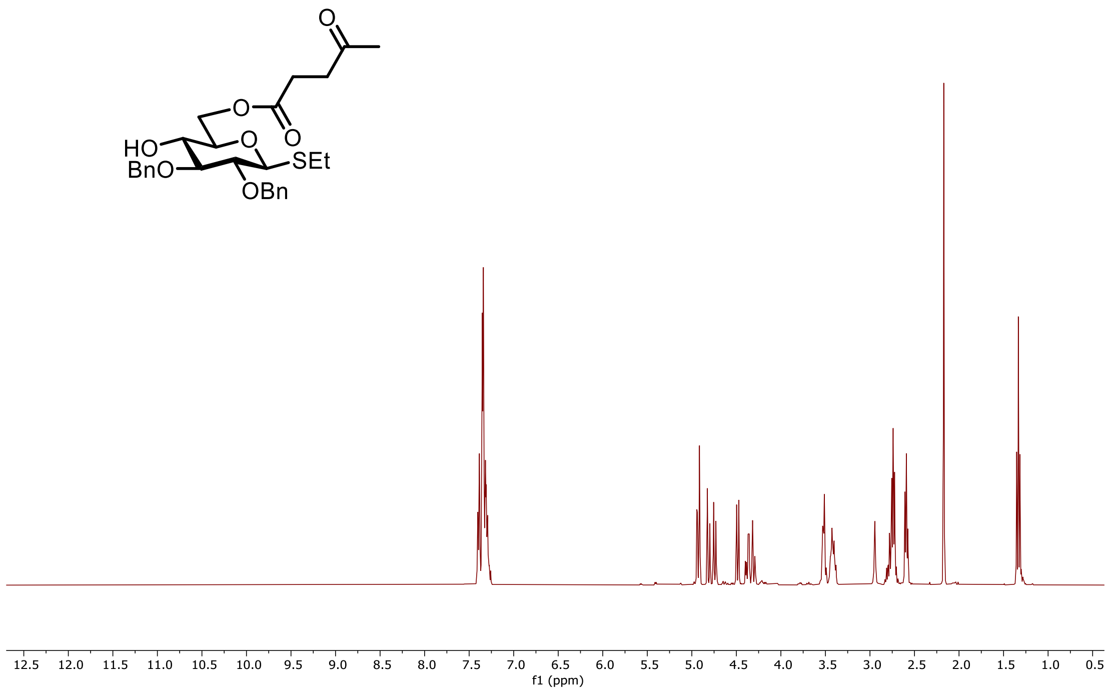

^{13}C NMR of 57 (101 MHz, CDCl$_3$)

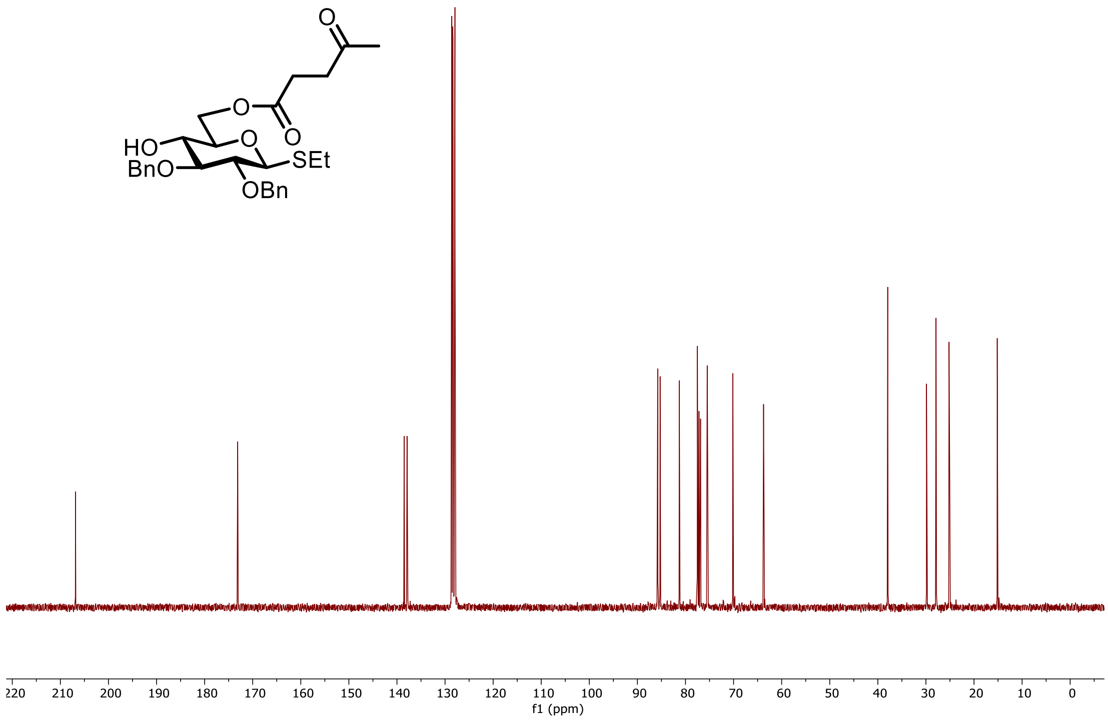

COSY NMR of 57 ($CDCl_3$)

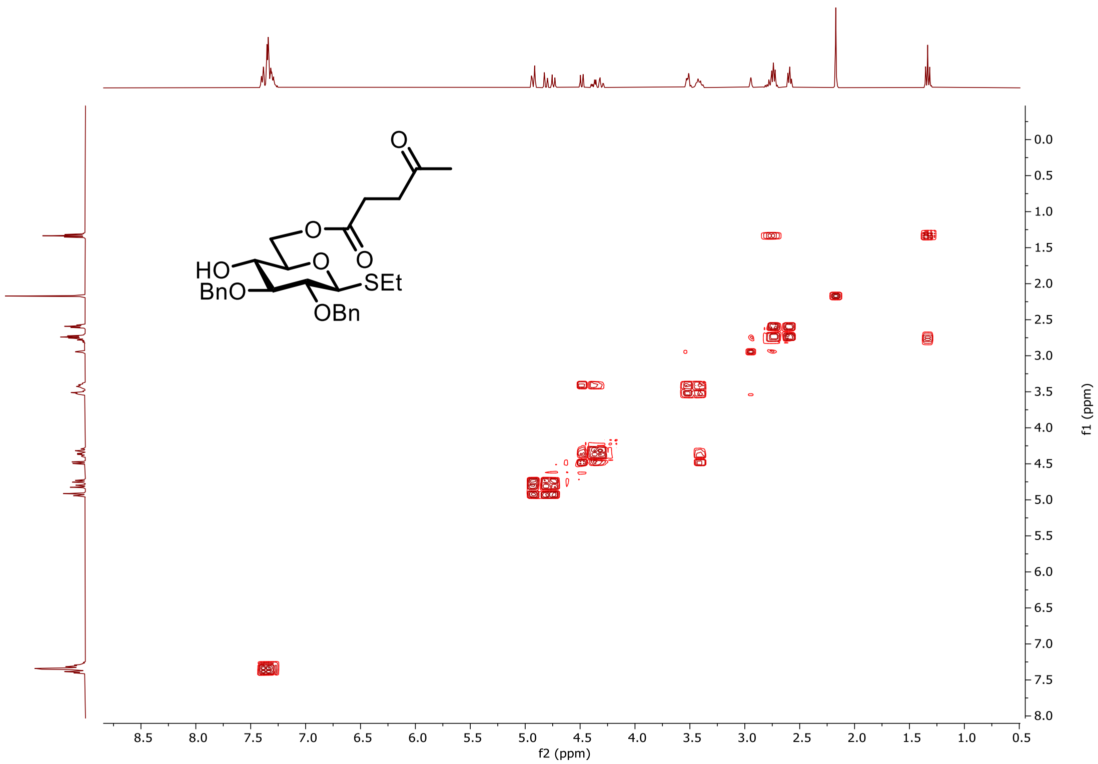

HSQC NMR of 57 ($CDCl_3$)

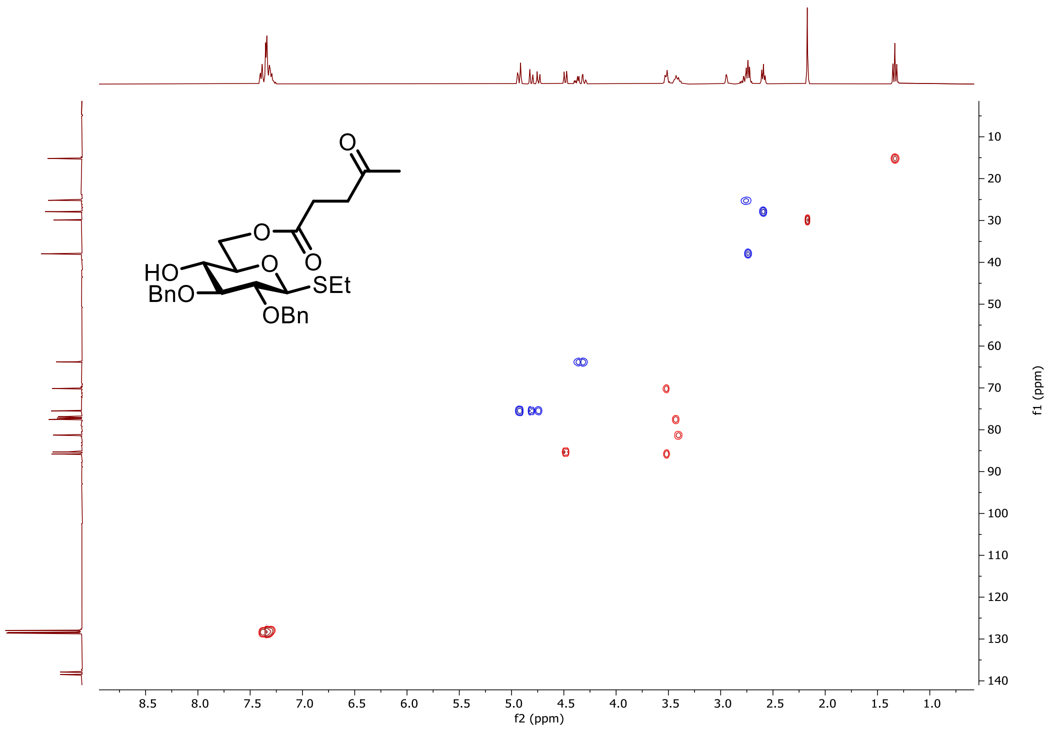

Ethyl 2,3-di-*O*-benzyl-4-*O*-(9-fluorenylmethoxycarbonyl)-6-*O*-levulinoyl-1-thio-β-D-glucopyranoside, 16

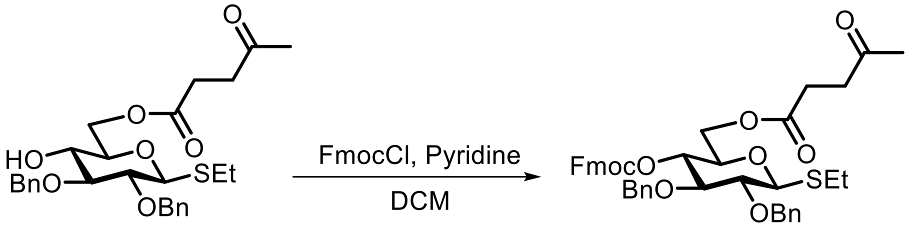

57 **16**

Ethyl 2,3-di-*O*-benzyl-6-*O*-levulinoyl-1-thio-β-D-glucopyranoside **57** (640 mg, 1.3 mmol) was dissolved in anhydrous DCM (6 mL) and pyridine (1.5 mL) was added. The solution was cooled with an ice bath for 30 min, and fluorenylmethyloxycarbonyl chloride (FmocCl, 821 mg, 3.2 mmol) was added slowly. The reaction was warmed to room temperature and stirred for 6 h. Upon completion, DCM (20 mL) was added and the organic phase was washed with aqueous citric acid (0.5 M, 20 mL). After extracting the water phase with DCM (10 mL), the organic layers were combined and dried over Na_2SO_4, filtered, and evaporated. The resulting crude product was purified by column chromatography (Hexane : EtOAc = 8:1→2:1) to give **16** as a white solid (830 mg, 90%). ^1H NMR (400 MHz, $CDCl_3$) δ 7.77 (d, *J* = 7.5 Hz, 2H), 7.59 (dd, *J* = 14.9, 7.5 Hz, 2H), 7.44 – 7.24 (m, 9H), 7.22 (s, 5H), 4.97 – 4.80 (m, 3H), 4.74 (d, *J* = 10.3, 1H), 4.69 (d, *J* = 11.2, 1H), 4.55 – 4.45 (m, 2H), 4.37 – 4.17 (m, 4H), 3.73 (appt, *J* = 9.1 Hz, 1H), 3.67 (ddd, *J* = 10.0, 5.3, 2.6 Hz, 1H), 3.51 (appt, *J* = 9.3 Hz, 1H), 2.87 – 2.53 (m, 6H), 2.17 (s, 3H), 1.36 (t, *J* = 7.4 Hz, 3H); ^{13}C NMR (101 MHz, $CDCl_3$) δ 206.40, 172.38, 154.38, 143.33, 143.15, 141.29, 141.26, 137.87, 137.70, 128.46, 128.39, 128.33, 128.01, 127.93, 127.91, 127.73, 127.23, 125.16, 125.00, 120.09, 120.07, 85.30, 83.67, 81.24, 75.61, 75.47, 74.51, 70.27, 62.89, 46.70, 37.87, 29.86, 27.87, 25.16, 15.16; $[\alpha]_D^{25}$ 0.54 (c = 1, $CHCl_3$); IR (neat) ν_{max} = 1749, 1719, 1256, 739 cm^{-1}; m/z ($HRMS^+$) $[M + Na]^+$ 747.2636 ($C_{42}H_{44}O_9SNa^+$ requires 747.2598).

^{1}H NMR of 16 (400 MHz, $CDCl_3$)

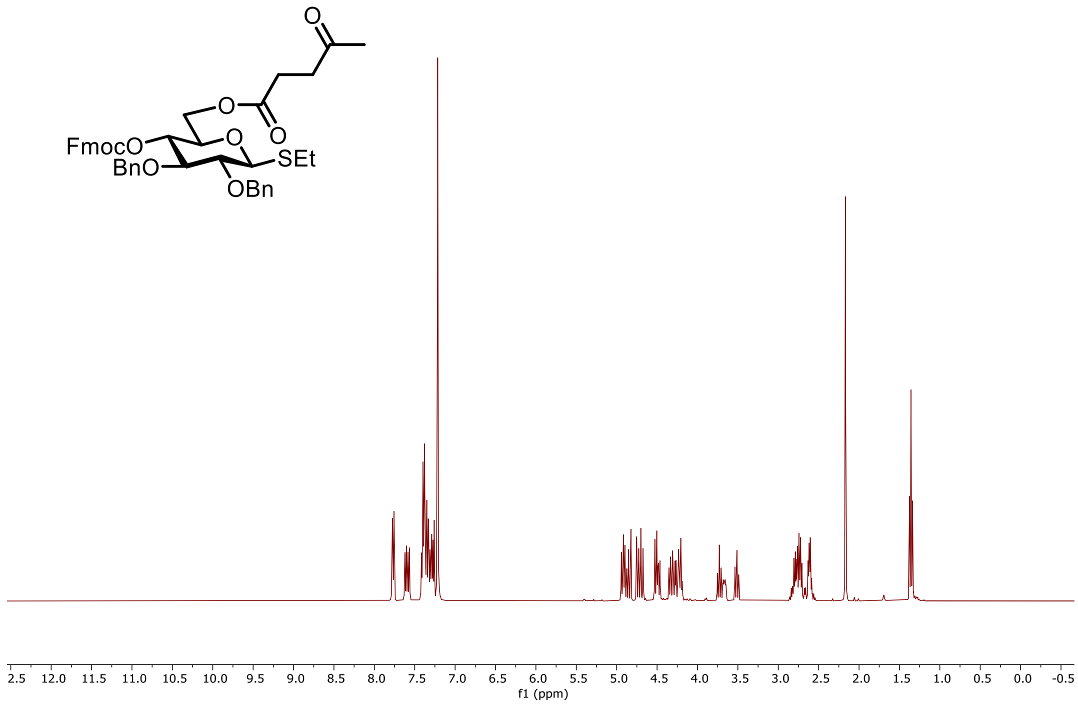

^{13}C NMR of 16 (101 MHz, $CDCl_3$)

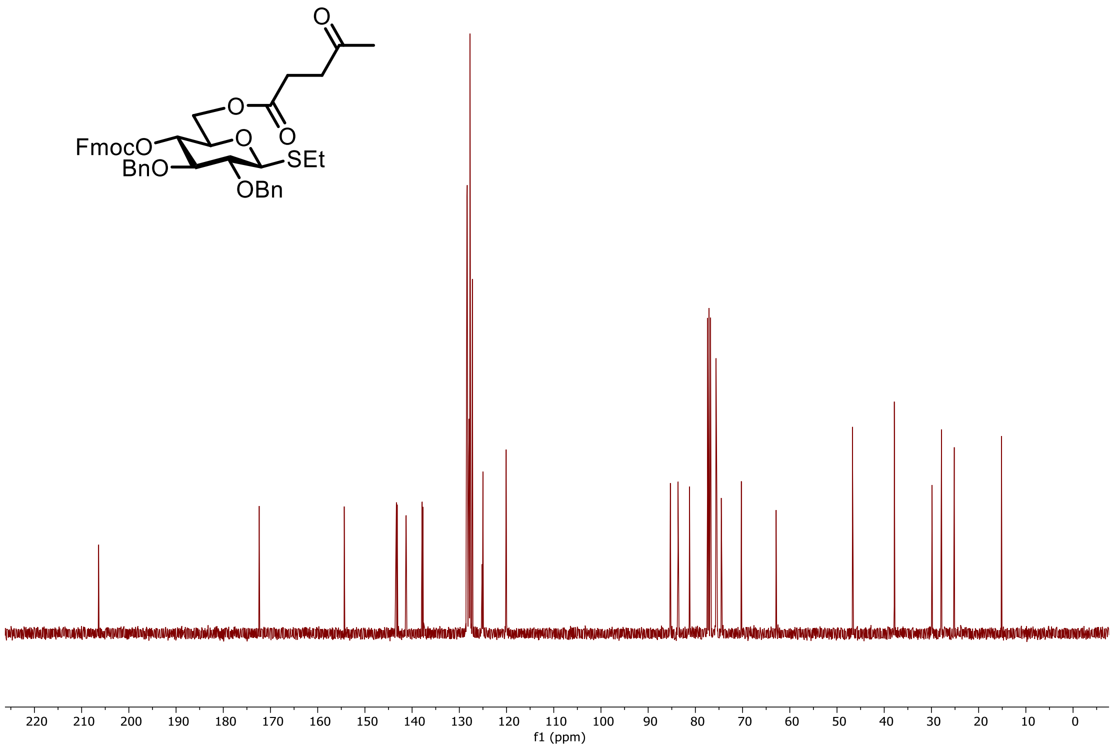

COSY NMR of 16 ($CDCl_3$)

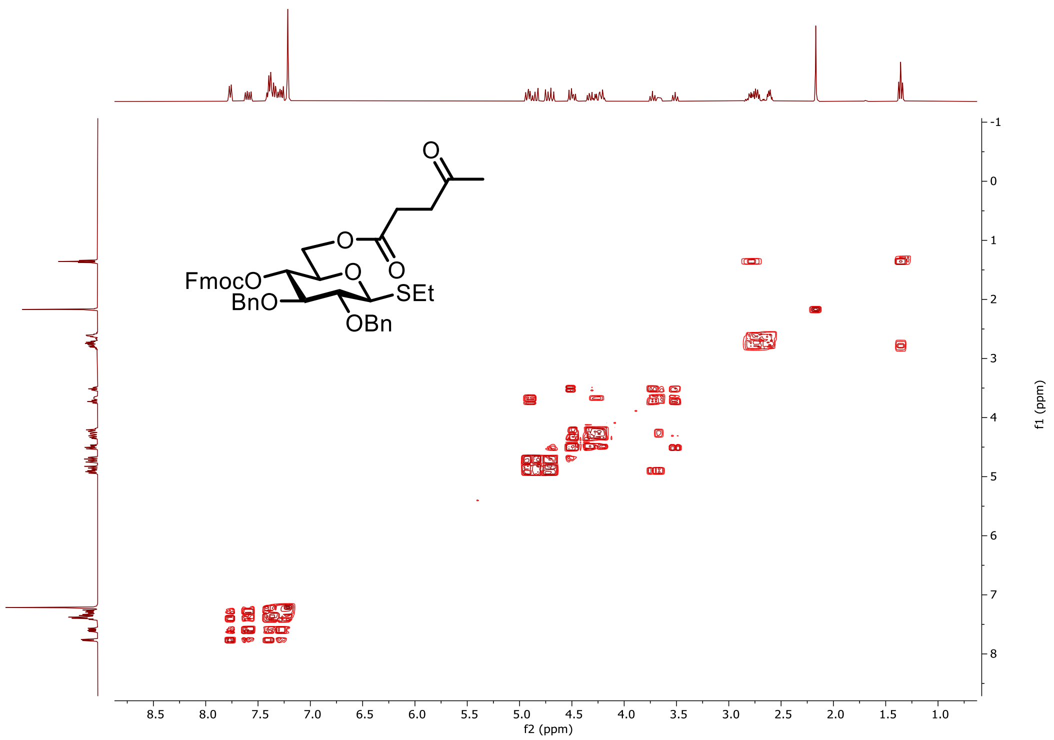

HSQC NMR of 16 ($CDCl_3$)

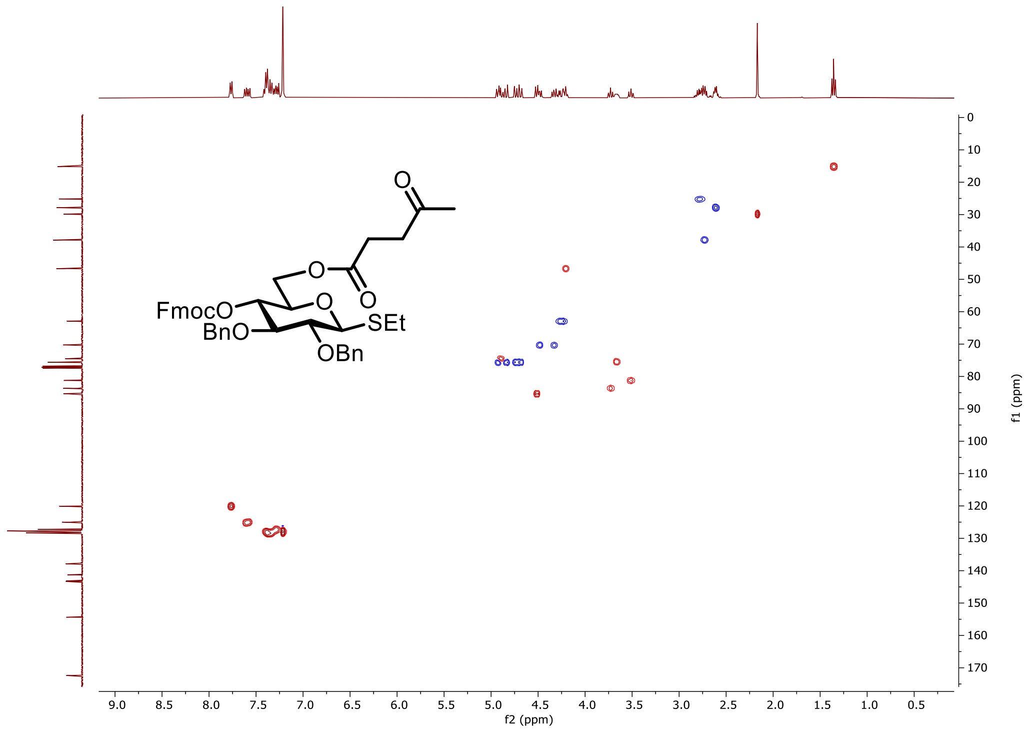

2.6 Synthesis of 17

Ethyl 2,3-di-*O*-benzyl-4-*O*-(9-fluorenylmethoxycarbonyl)-1-thio-β-D-glucopyranoside, 58

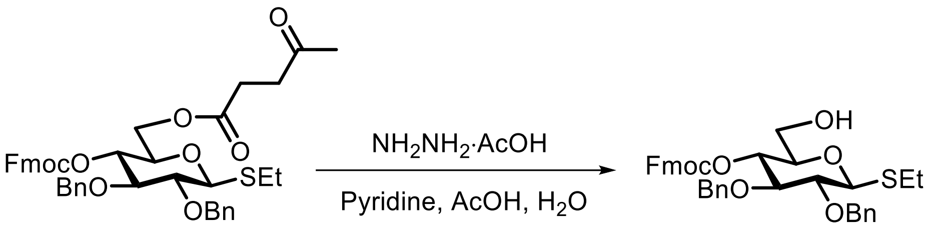

16 58

Ethyl 2,3-di-*O*-benzyl-4-*O*-(9-fluorenylmethoxycarbonyl)-6-*O*-levulinoyl-1-thio-β-D-glucopyranoside **16** (11.1 g, 15.3 mmol) was dissolved in a mixture of pyridine (80 mL) and acetic acid (20 mL) and H_2O (5 mL) were added. After a clear solution was obtained, hydrazine acetate (1.81 g, 19.5 mmol) was added. The reaction was kept at room temperature for 3 h. After it was completed, the reaction was quenched by adding acetone (5 mL). The solvent was removed by evaporation as much as possible, and DCM (200 mL) was added. The solution was washed with aqueous citric acid (0.5 M, 100 mL, 3 times), and the water layers were combined and extracted with DCM (50 mL). The obtained organic layers were also combined and dried over Na_2SO_4, filtered, and evaporated. The resulting crude product was purified by column chromatography (Hexane : EtOAc = 6:1→2:1) to give **58** as a white solid (9.40 g, 98%). ^{1}H NMR (400 MHz, $CDCl_3$) δ 7.81 – 7.74 (m, 2H), 7.62 – 7.54 (m, 2H), 7.47 – 7.19 (m, 14H), 4.96 – 4.81 (m, 3H), 4.74 (dd, *J* = 10.8, 9.0 Hz, 2H), 4.52 (dd, *J* = 10.1, 6.7 Hz, 2H), 4.40 (dd, *J* = 10.5, 7.0 Hz, 1H), 4.21 (appt, *J* = 7.0 Hz, 1H), 3.80 – 3.71 (m, 2H), 3.62 (dd, *J* = 12.5, 5.1 Hz, 1H), 3.55 – 3.44 (m, 2H), 2.88 – 2.69 (m, 2H), 2.31 (br. s, 1H), 1.35 (t, *J* = 7.4 Hz, 3H); ^{13}C NMR (101 MHz, $CDCl_3$) δ 154.87, 143.17, 143.07, 141.34, 141.31, 138.00, 137.72, 128.46, 128.40, 128.35, 128.01, 127.98, 127.72, 127.70, 127.23, 125.07, 124.95, 120.15, 120.13, 85.23, 83.58, 81.23, 77.97, 75.64, 75.56, 74.63, 70.18, 61.64, 46.77, 25.08, 15.15; $[\alpha]_D^{25}$ -15.59 (c = 1, $CHCl_3$); IR (neat) ν_{max} = 1752, 1256, 739 cm^{-1}; m/z (HRMS$^+$) [M + Na]$^+$ 649.2266 ($C_{37}H_{38}O_7SNa^+$ requires 649.2230).

^{1}H NMR of 58 (400 MHz, $CDCl_3$)

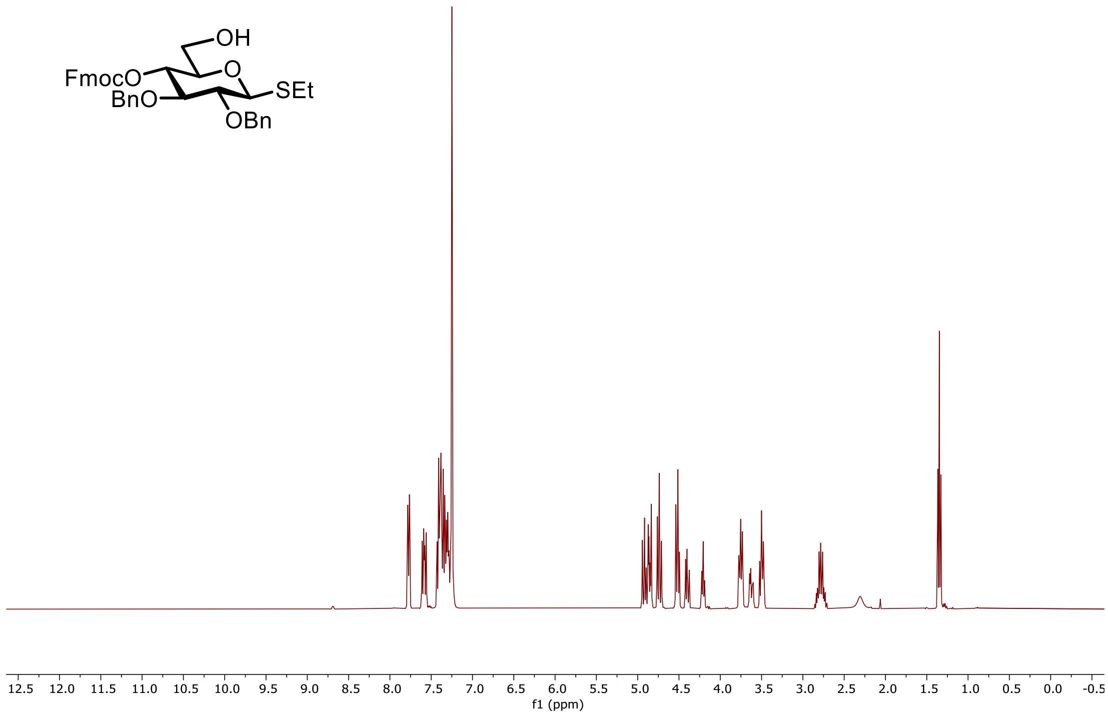

^{13}C NMR of 58 (101 MHz, $CDCl_3$)

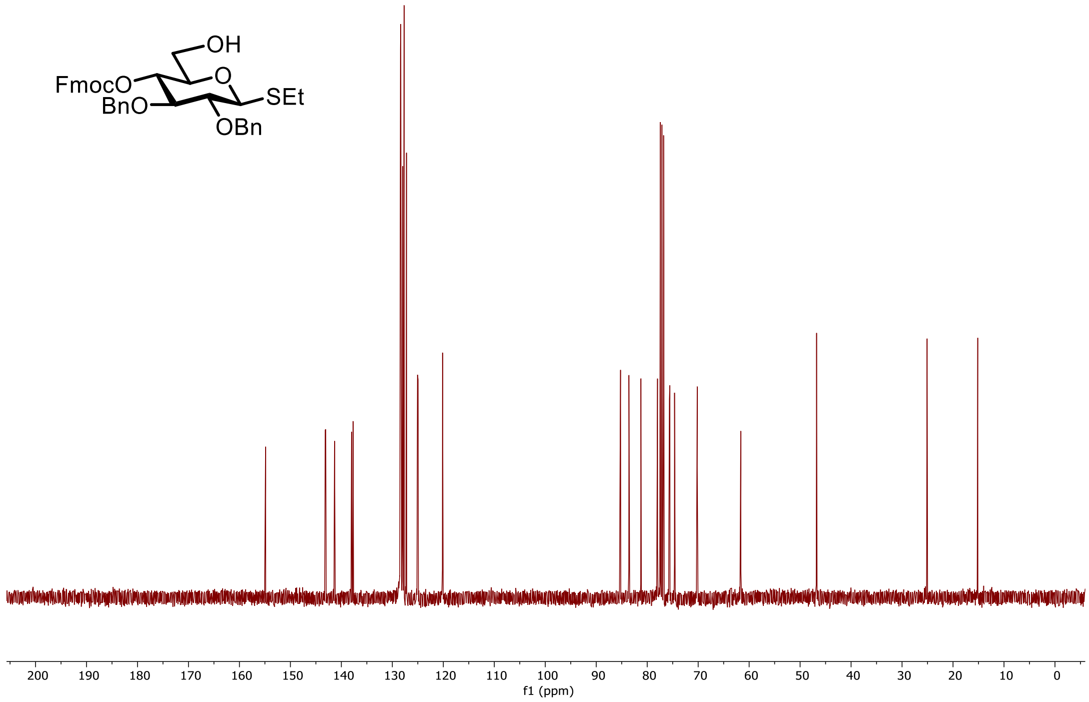

COSY NMR of 58 ($CDCl_3$)

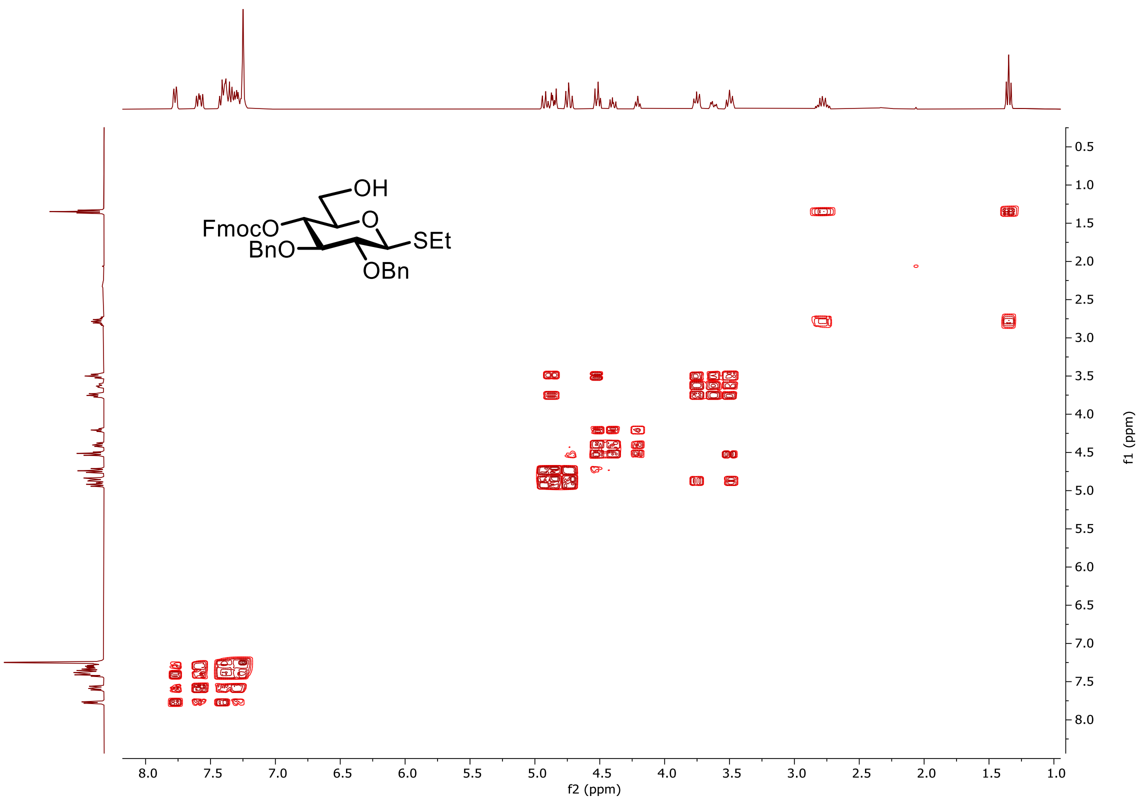

HSQC NMR of 58 ($CDCl_3$)

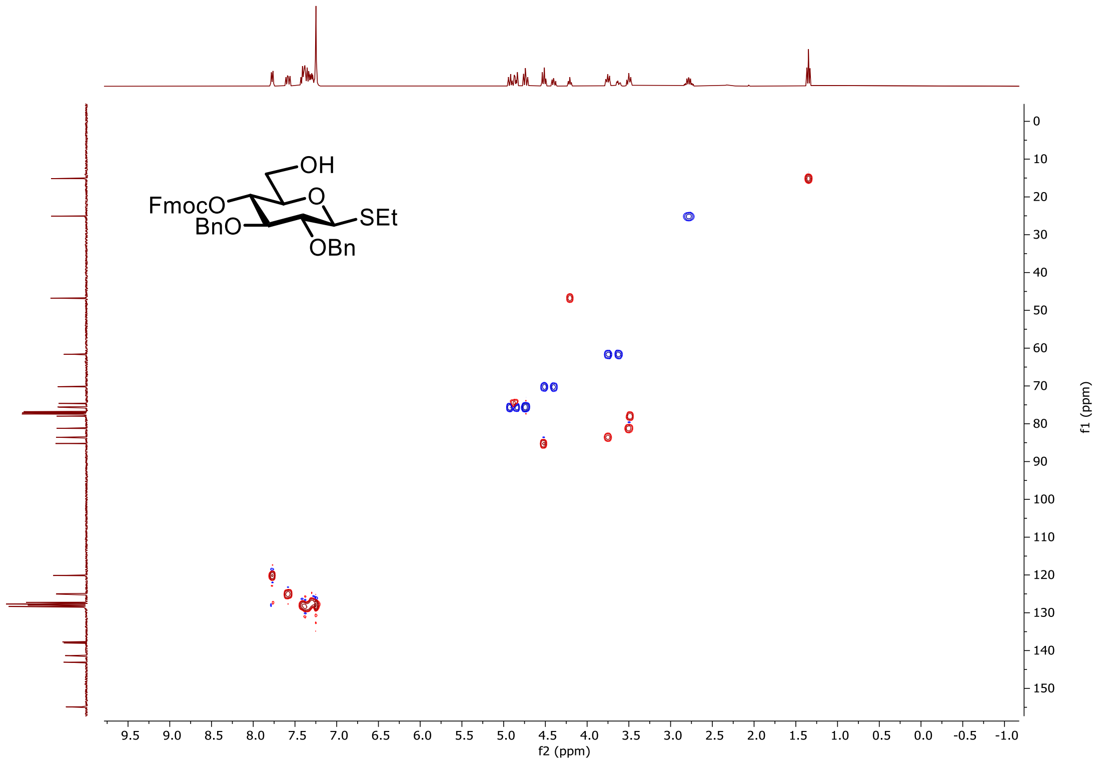

Ethyl 2,3-di-*O*-benzyl-4-*O*-(9-fluorenylmethoxycarbonyl)-6-*O*-pivaloyl-1-thio-β-D-glucopyranoside, 17

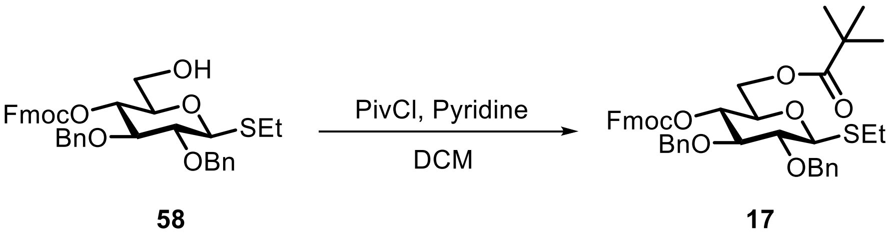

Ethyl 2,3-di-*O*-benzyl-4-*O*-(9-fluorenylmethoxycarbonyl)-1-thio-β-D-glucopyranoside **58** (1.00 g, 1.6 mmol) was dissolved in anhydrous DCM (20 mL) and pyridine (4 mL) was added. The solution was cooled with an ice bath for 30 min, and pivaloyl chloride (PivCl, 392 µL, 3.2 mmol) was added slowly. The reaction was warmed to room temperature and stirred for 16 h. Completion of the reaction was confirmed by TLC, MeOH (2 mL) was added. DCM (20 mL) was added 30 min later, and the organic phase was washed with aqueous citric acid (0.5 M, 20 mL). After extracting the water phase with DCM (10 mL), the organic layers were combined and dried over Na_2SO_4, filtered, and evaporated. The resulting crude product was purified by column chromatography (Hexane : EtOAc = 8:1→4:1) to give **17** as a white solid (900 mg, 79%). ^{1}H NMR (400 MHz, $CDCl_3$) δ 7.79 (d, *J* = 7.6 Hz, 2H), 7.67 – 7.61 (m, 1H), 7.61 – 7.57 (m, 1H), 7.46 – 7.28 (m, 9H), 7.27 – 7.22 (m, 5H), 4.99 – 4.89 (m, 2H), 4.87 (d, *J* = 11.2 Hz, 1H), 4.77 (d, *J* = 10.3 Hz, 1H), 4.72 (d, *J* = 11.2 Hz, 1H), 4.57 – 4.47 (m, 2H), 4.38 (dd, *J* = 10.4, 7.3 Hz, 1H), 4.31 (dd, *J* = 12.2, 2.5 Hz, 1H), 4.27 – 4.18 (m, 2H), 3.75 (appt, *J* = 9.1 Hz, 1H), 3.69 (ddd, *J* = 10.0, 5.6, 2.4 Hz, 1H), 3.55 (appt, *J* = 9.3 Hz, 1H), 2.90 – 2.69 (m, 2H), 1.38 (t, *J* = 7.5 Hz, 3H), 1.27 (s, 9H); ^{13}C NMR (101 MHz, $CDCl_3$) δ 178.07, 154.38, 143.31, 143.21, 141.31, 137.89, 137.76, 128.49, 128.41, 128.37, 128.04, 127.96, 127.81, 127.78, 127.27, 127.26, 125.16, 125.01, 120.14, 120.13, 84.92, 83.76, 81.30, 75.74, 75.69, 75.64, 74.63, 70.24, 62.81, 46.74, 38.87, 27.19, 24.81, 15.25; $[\alpha]_D^{25}$ 2.33 (c = 1, $CHCl_3$); IR (neat) ν_{max} = 1755, 1732, 1256, 742 cm^{-1}; m/z (HRMS$^+$) [M + Na]$^+$ 733.2870 ($C_{42}H_{46}O_8SNa^+$ requires 733.2806).

^{1}H NMR of 17 (400 MHz, CDCl$_3$)

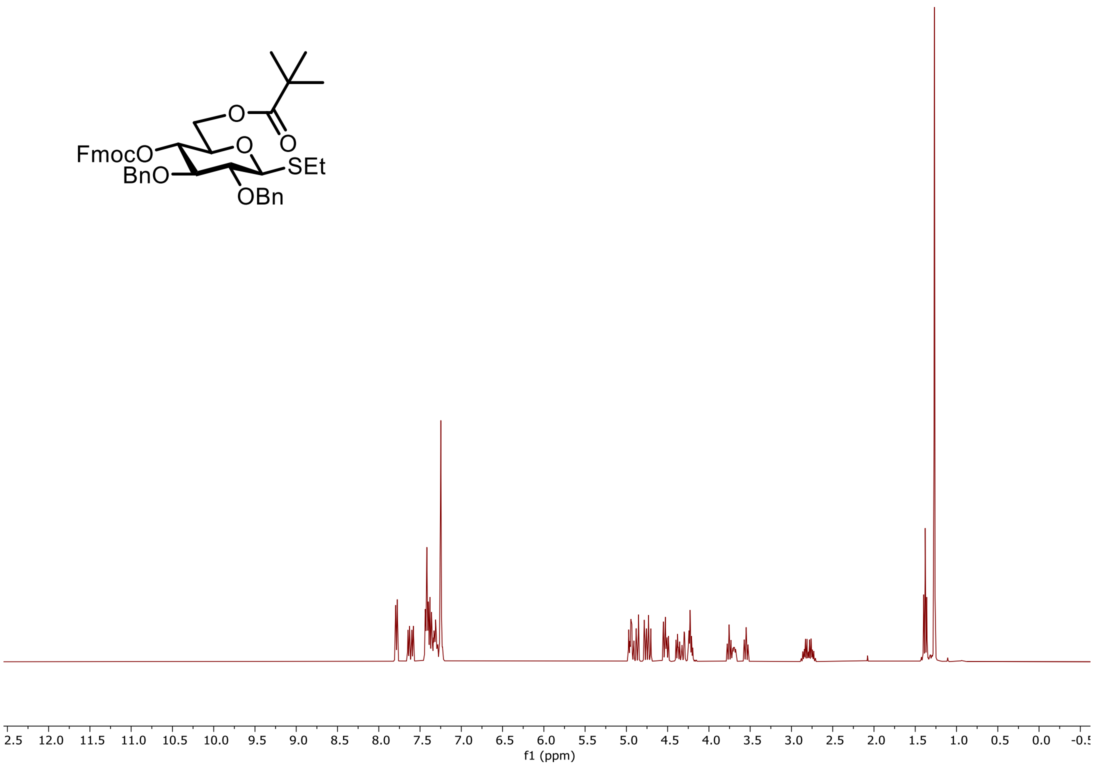

^{13}C NMR of 17 (101 MHz, CDCl$_3$)

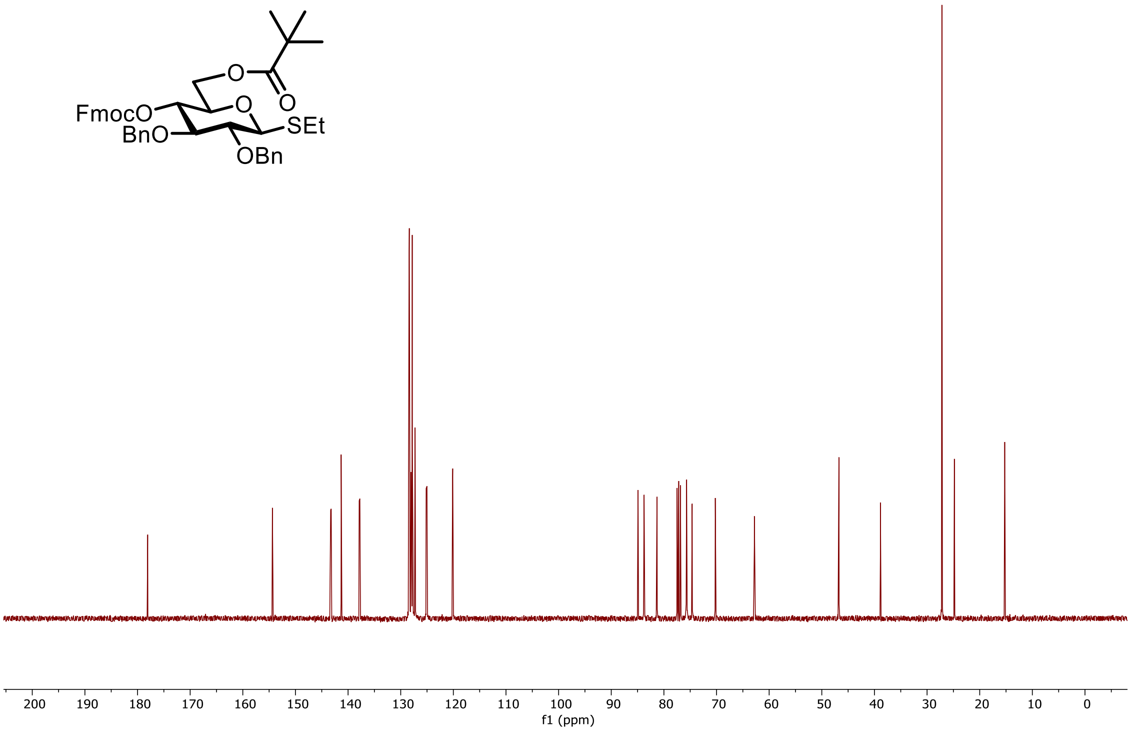

COSY NMR of 17 (CDCl₃)

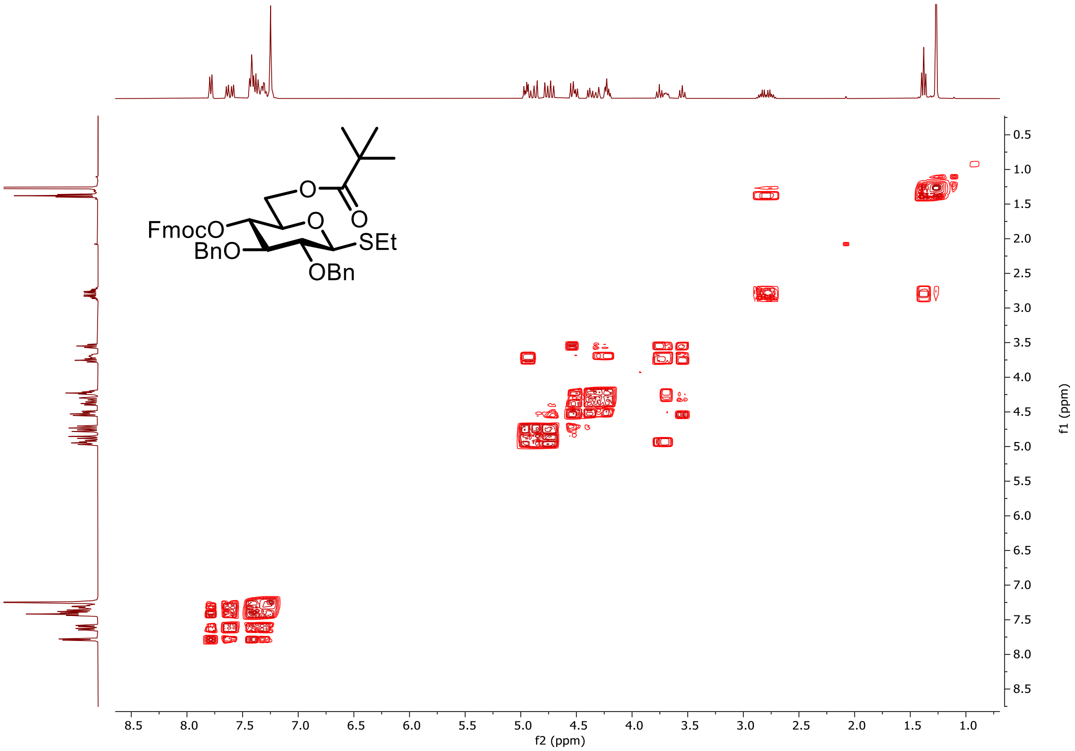

HSQC NMR of 17 (CDCl₃)

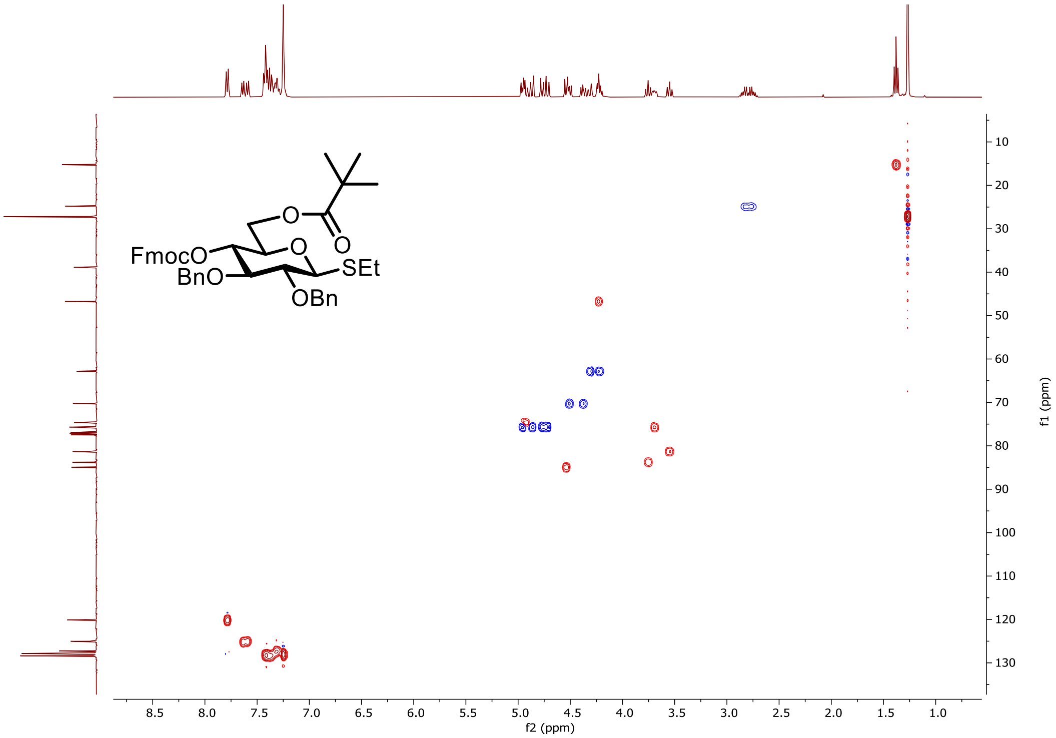

Dibutylphosphoryl 2,3-di-*O*-benzyl-4-*O*-(9-fluorenylmethoxycarbonyl)-6-*O*-pivaloyl-α-D-glucopyranoside, 59a

&

Dibutylphosphoryl 2,3-di-*O*-benzyl-4-*O*-(9-fluorenylmethoxycarbonyl)-6-*O*-pivaloyl-β-D-glucopyranoside, 59b

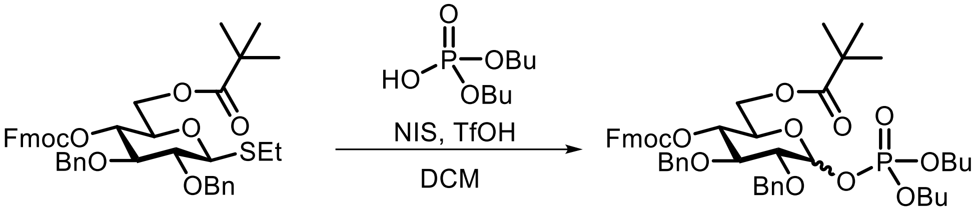

17 **59a + 59b**

Ethyl 2,3-di-*O*-benzyl-4-*O*-(9-fluorenylmethoxycarbonyl)-6-*O*-pivaloyl-1-thio-β-D-glucopyranoside **17** (328 mg, 0.46 mmol) was dissolved in anhydrous DCM (5 mL) and 4Å molecular sieve (500 mg) was added. After dibutyl phosphate (200 µL, 1.0 mmol) and *N*-iodosuccinimide (NIS, 126 mg, 0.56 mmol) were added, the reaction mixture was stirred at room temperature for 30 min and cooled to -15 °C under argon protection. Trifluoromethanesulfonic acid (TfOH, 8.1 µL, 92 µmol) was slowly added and the reaction was kept for 10 min at -15 °C, then warmed to 0 °C. 60 min later, saturated aq. $NaHCO_3$ solution (10 mL) was added to quench the reaction. DCM (20 mL) and saturated aq. $Na_2S_2O_3$ solution (10 mL) were added. After extraction, the organic layer was dried over Na_2SO_4, filtered, and evaporated. The resulting crude product was purified by column chromatography (Hexane : EtOAc = 4:1→2:1) to give the mixture of **59a** and **59b** as clear oil (382 mg, 96%). **59a**: ^{1}H NMR (400 MHz, $CDCl_3$) δ 7.80 (d, *J* = 7.5 Hz, 2H), 7.65 (d, *J* = 7.5 Hz, 1H), 7.60 (d, *J* = 7.5 Hz, 1H), 7.47 – 7.29 (m, 9H), 7.29 – 7.19 (m, 5H), 5.94 (dd, *J* = 7.2, 3.2 Hz, 1H), 5.01 (appt, *J* = 9.8 Hz, 1H), 4.91 (d, *J* = 11.2 Hz, 1H), 4.85 (d, *J* = 11.2 Hz, 1H), 4.72 (dd, *J* = 11.2, 8.7 Hz, 2H), 4.51 (dd, *J* = 10.5, 7.0 Hz, 1H), 4.36 (dd, *J* = 10.4, 7.3 Hz, 1H), 4.31 – 4.18 (m, 4H), 4.17 – 3.96 (m, 5H), 3.71 (dt, *J* = 9.6, 3.0 Hz, 1H), 1.77 – 1.59 (m, 4H), 1.52 – 1.28 (m, 4H), 1.25 (s, 9H), 0.99 (t, *J* = 7.4 Hz, 3H), 0.93 (t, *J* = 7.4 Hz, 3H); ^{13}C NMR (101 MHz, $CDCl_3$) δ 178.12, 154.25, 143.32, 143.19, 141.33, 141.30, 137.90, 137.44, 128.48, 128.33, 128.10, 128.02, 127.97, 127.92, 127.79, 127.26, 125.19, 125.02, 120.15, 120.13, 94.73 (d, *J* = 5.8 Hz), 79.14 (d, *J* = 7.1 Hz), 78.23, 75.58, 73.44, 73.20, 70.23, 69.42, 67.96 (d, *J* = 6.0 Hz), 67.70 (d, *J* = 5.9 Hz), 61.75, 46.71, 38.92, 32.29 (d, *J* = 7.0 Hz), 32.16 (d, *J* = 7.2 Hz), 27.16, 18.72, 18.62, 13.68, 13.66; $[\alpha]_D^{25}$ 43.50 (c = 1, $CHCl_3$); IR (neat) ν_{max} = 1756, 1734, 1255, 954, 739 cm^{-1}; m/z (HRMS$^+$) $[M + Na]^+$ 881.3774 ($C_{48}H_{59}O_{12}PNa^+$ requires 881.3636). **59b**: ^{1}H NMR (400 MHz, $CDCl_3$) δ 7.79 (d, *J* = 7.6 Hz, 2H), 7.64 (d, *J* = 7.5 Hz, 1H), 7.59 (d, *J* = 7.5 Hz, 1H), 7.46 – 7.26 (m, 9H), 7.21 (s, 5H), 5.28 (appt, *J* = 7.4 Hz, 1H), 5.05 – 4.90 (m, 2H), 4.88 – 4.78 (m, 2H), 4.68 (d, *J* = 11.2 Hz, 1H), 4.51 (dd, *J* = 10.4, 6.9 Hz, 1H), 4.41 – 4.30 (m, 2H), 4.22 (td, *J* = 7.3, 6.8, 4.5 Hz, 2H), 4.18 – 3.98 (m, 4H), 3.86 – 3.71 (m, 2H), 3.63 (appt, *J* = 8.5 Hz, 1H), 1.74 – 1.58 (m, 4H), 1.49 – 1.29 (m, 4H), 1.26 (s, 9H), 0.97 (t, *J* = 7.4 Hz, 3H), 0.91 (t, *J* = 7.4 Hz, 3H); ^{13}C NMR (101 MHz, $CDCl_3$) δ 178.00, 154.26, 143.28, 143.14, 141.31, 141.29, 137.80, 137.66, 128.43, 128.35, 127.98, 127.93, 127.87, 127.83, 127.73, 127.28, 125.17, 125.01, 120.15, 120.13, 98.55 (d, *J* = 6.1 Hz), 81.76

(d, *J* = 9.1 Hz), 81.49, 75.68, 75.05, 73.61, 72.38, 70.31, 67.84 (t, *J* = 4.9 Hz), 61.72, 46.70, 38.90, 32.20 (d, *J* = 5.9 Hz), 32.13(d, *J* = 5.9 Hz), 27.15, 18.68, 18.59, 13.67, 13.62; $[\alpha]_D^{25}$ 13.89 (c = 1, $CHCl_3$); IR (neat) ν_{max} = 1756, 1734, 1256, 1028, 739 cm^{-1}; m/z ($HRMS^+$) $[M + Na]^+$ 881.3774 ($C_{48}H_{59}O_{12}PNa^+$ requires 881.3636).

^{1}H NMR of 59a (400 MHz, $CDCl_3$)

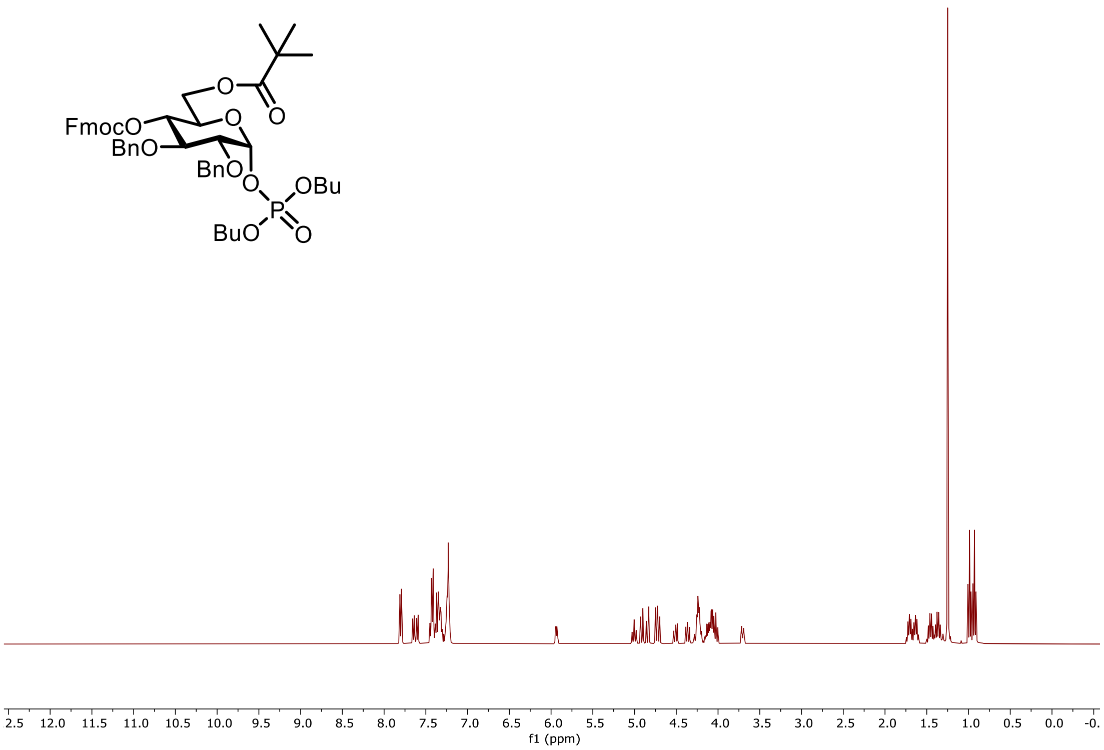

^{13}C NMR of 59a (101 MHz, $CDCl_3$)

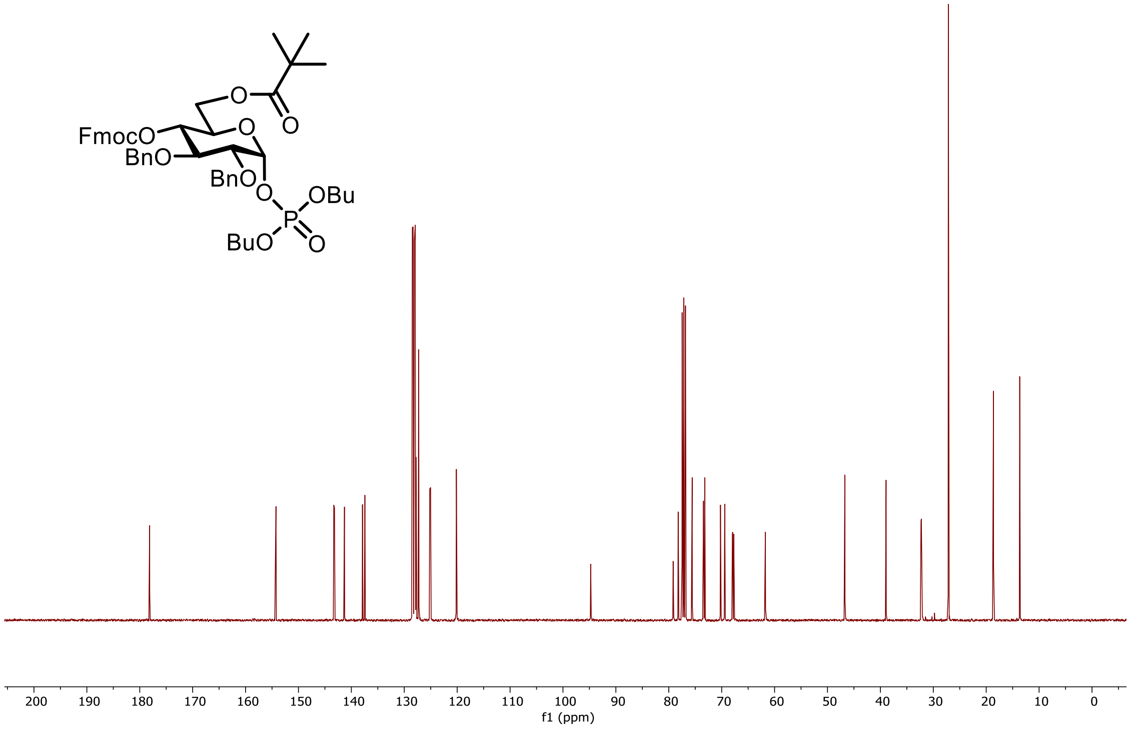

COSY NMR of 59a ($CDCl_3$)

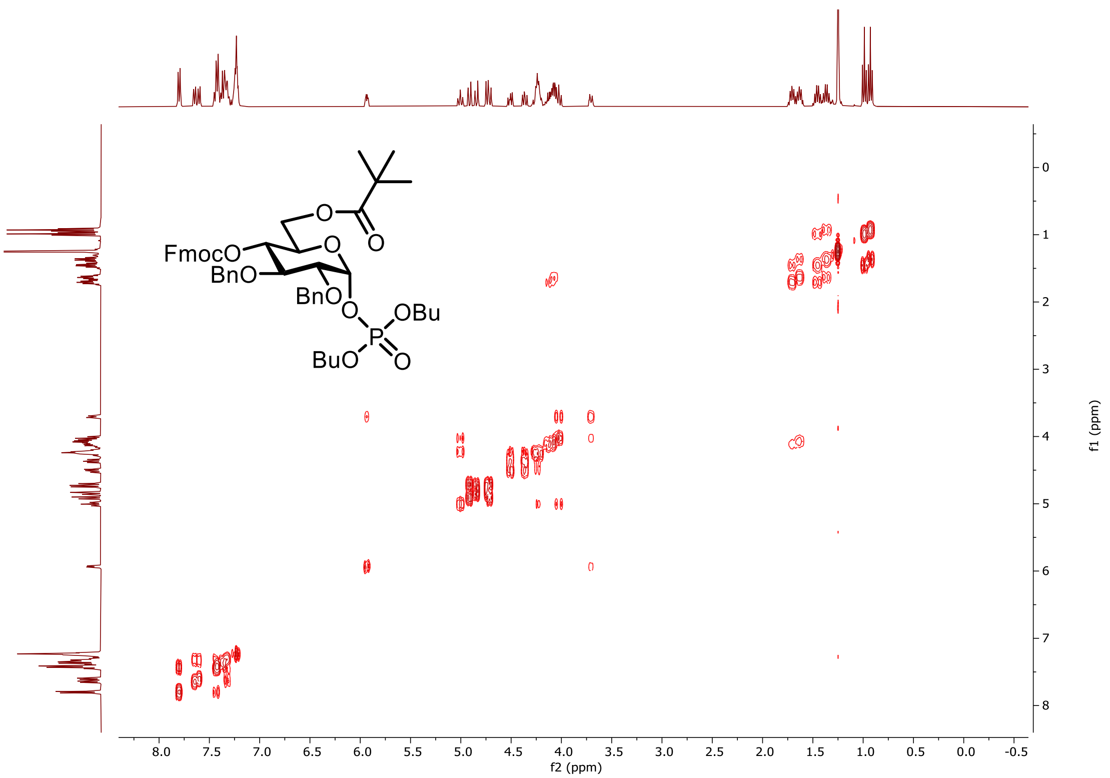

HSQC NMR of 59a ($CDCl_3$)

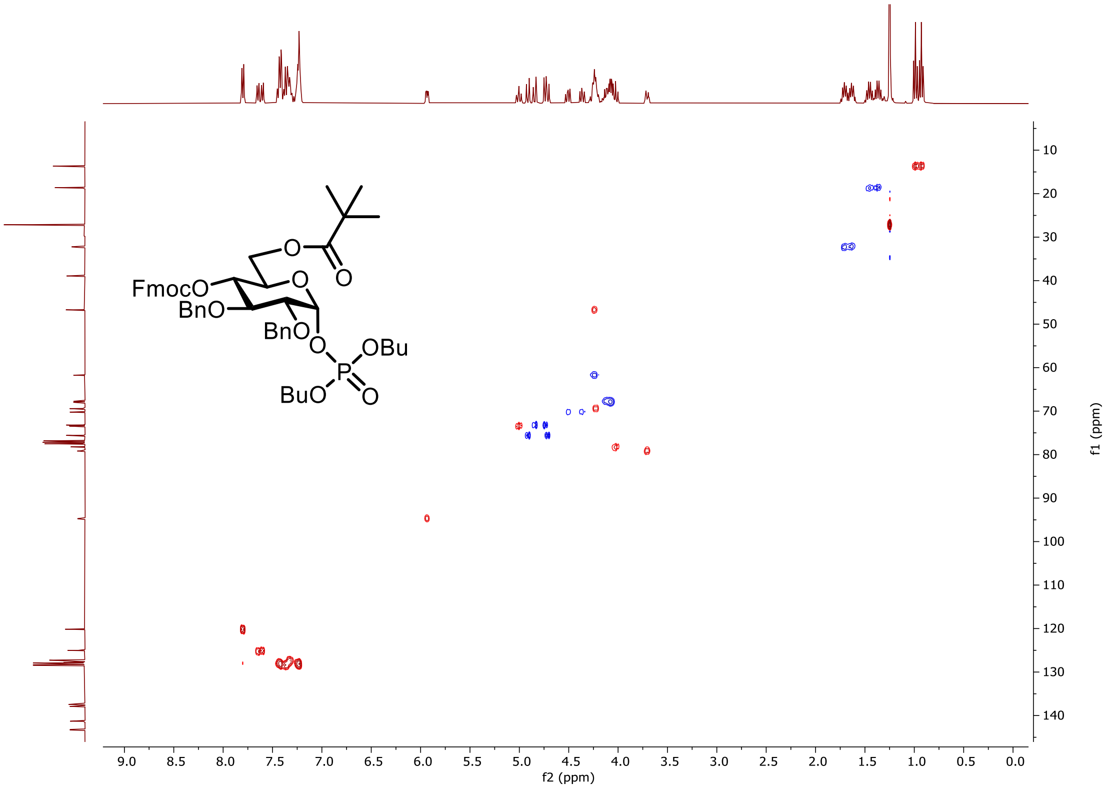

^{1}H NMR of 59b (400 MHz, $CDCl_3$)

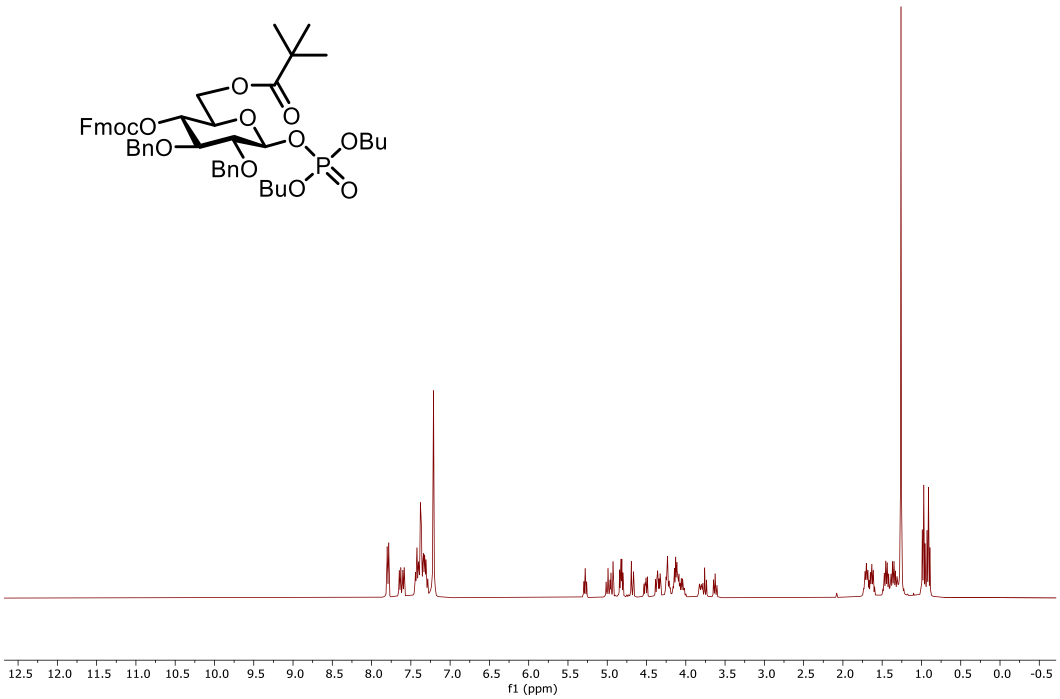

^{13}C NMR of 59b (101 MHz, $CDCl_3$)

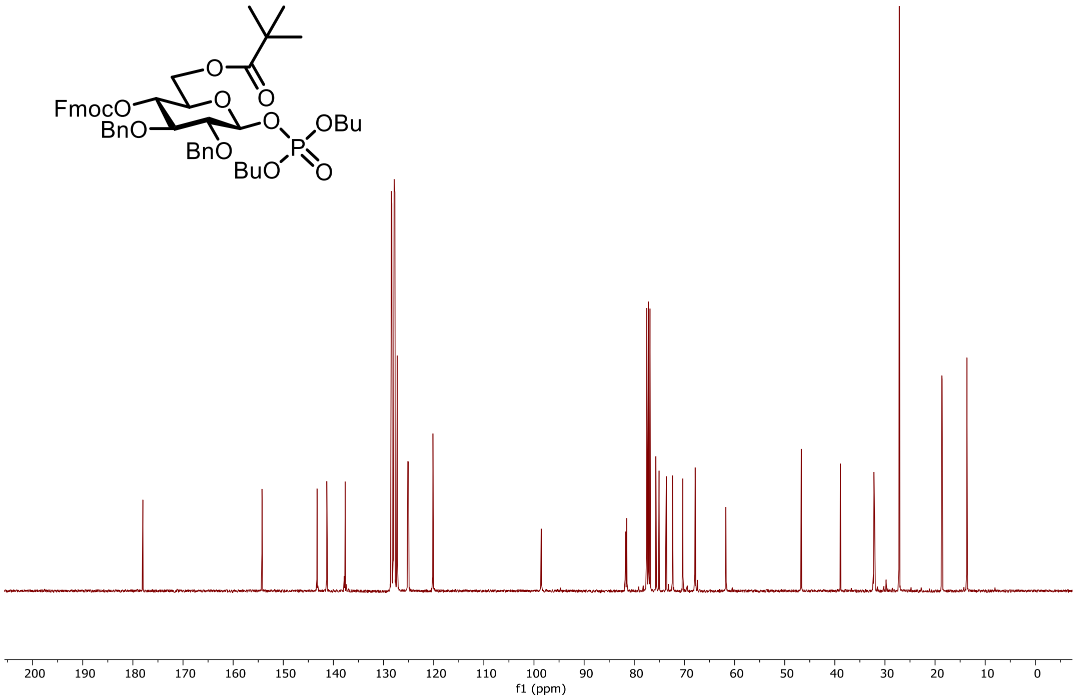

COSY NMR of 59b ($CDCl_3$)

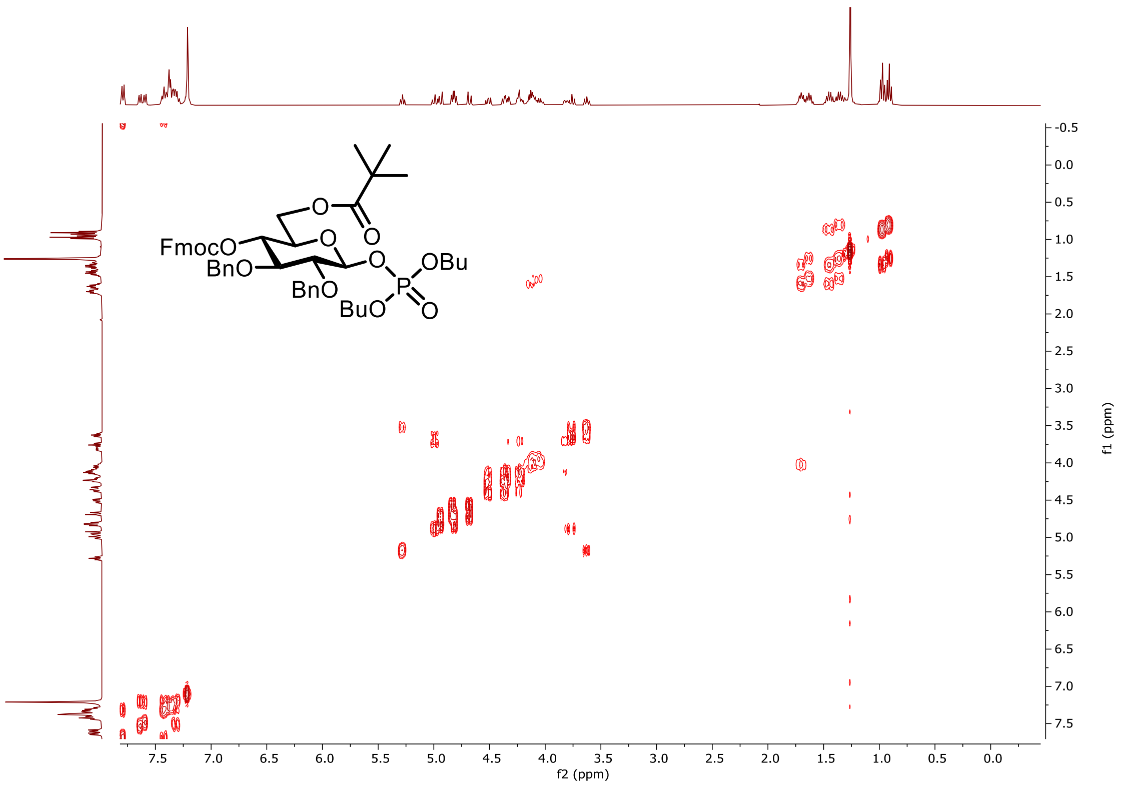

HSQC NMR of 59b ($CDCl_3$)

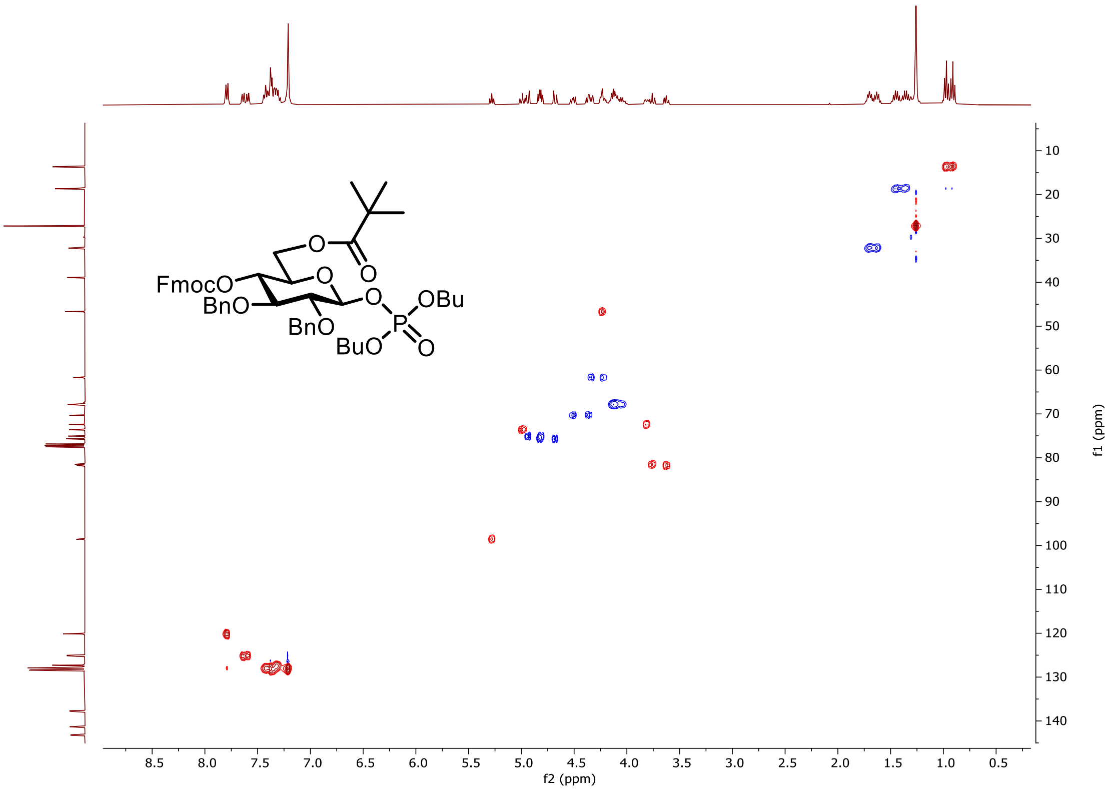

2.7 Synthesis of 18

Ethyl 2,3-di-*O*-benzyl-6-*O*-trifluoroacetyl-1-thio-β-D-glucopyranoside, 60

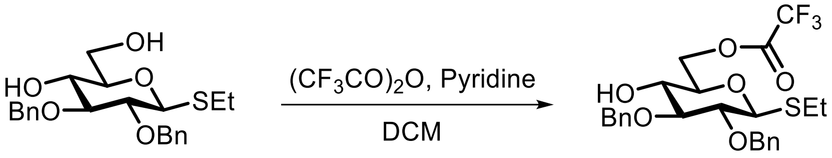

53 **60**

53 was prepared according to previously established procedures.[2]

Ethyl 2,3-di-*O*-benzyl-1-thio-β-D-glucopyranoside **53** (450 mg, 1.1 mmol) was dissolved in anhydrous DCM (10 mL) and pyridine (2 mL) was added. The solution was cooled to -15 °C, trifluoroacetic anhydride (172 μL, 1.2 mmol) was added dropwise. After 3 h, the system was warmed to room temperature, and the reaction solution was directly loaded on top and purified by column chromatography (Hexane : EtOAc = 8:1→2:1) to give **60** as a colorless oil (190 mg, 34%). ^{1}H NMR (400 MHz, $CDCl_3$) δ 7.46 – 7.32 (m, 10H), 5.00 (dd, *J* = 10.9, 8.4 Hz, 2H), 4.78 – 4.61 (m, 3H), 4.55 – 4.45 (m, 2H), 3.65 – 3.36 (m, 4H), 2.76 (qd, *J* = 12.8, 6.3 Hz, 2H), 2.38 (br. s, 1H), 1.34 (t, *J* = 7.4 Hz, 3H); ^{13}C NMR (101 MHz, $CDCl_3$) δ 157.29 (q, *J* = 42.5 Hz), 138.18, 137.67, 128.83, 128.52, 128.41, 128.28, 128.09, 127.98, 114.50 (q, *J* = 285.6 Hz), 85.62, 85.24, 81.31, 76.33, 75.46, 69.77, 66.89, 25.16, 15.11; $[\alpha]_D^{25}$ -25.32 (c = 1, $CHCl_3$); IR (neat) ν_{max} = 1790, 1223, 1167, 698 cm^{-1}; m/z (HRMS$^+$) [M + Na]$^+$ 523.1372 ($C_{24}H_{27}F_3O_6SNa^+$ requires 523.1373).

^{1}H NMR of 60 (400 MHz, $CDCl_3$)

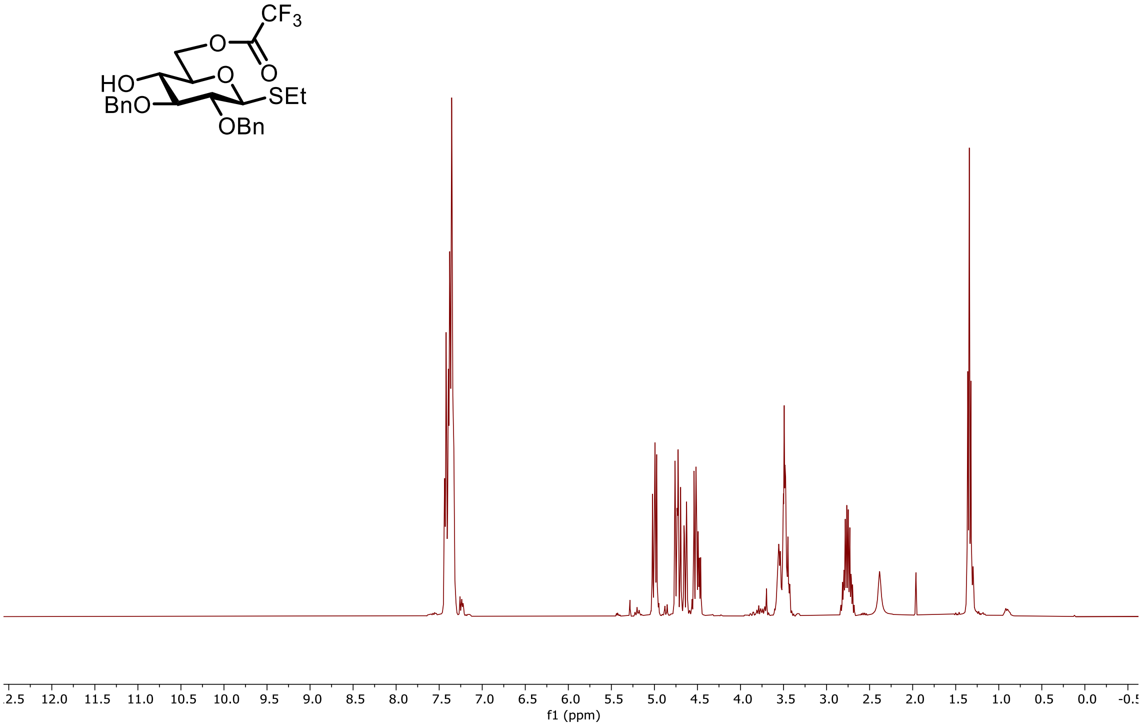

^{13}C NMR of 60 (101 MHz, $CDCl_3$)

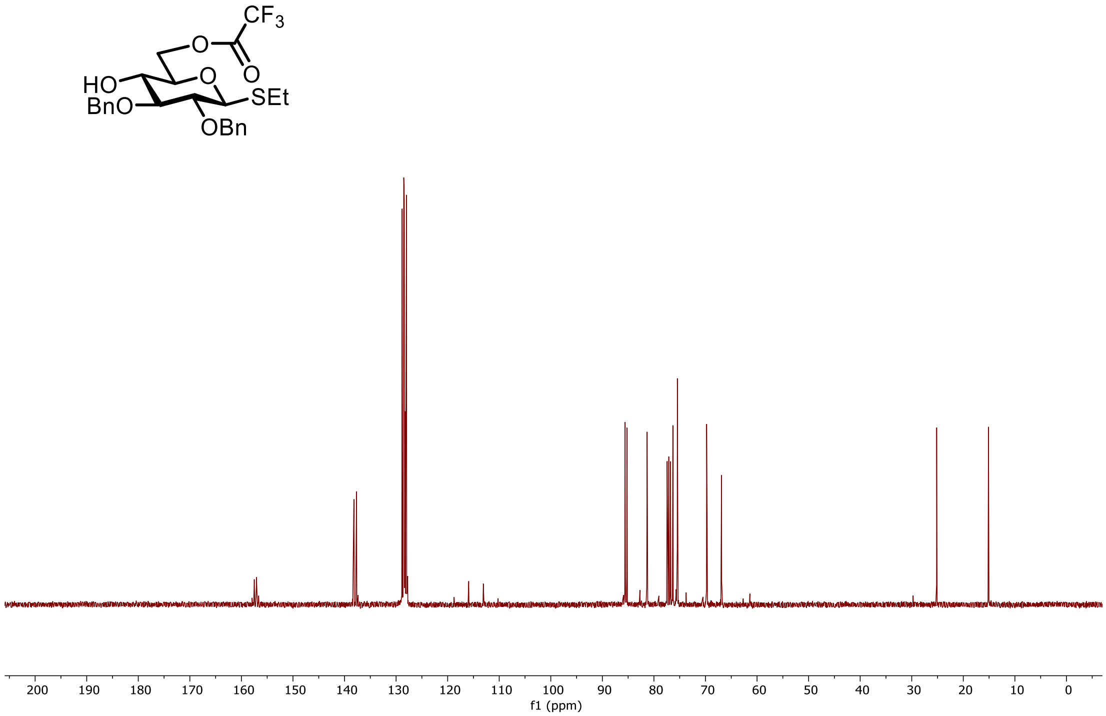

COSY NMR of 60 ($CDCl_3$)

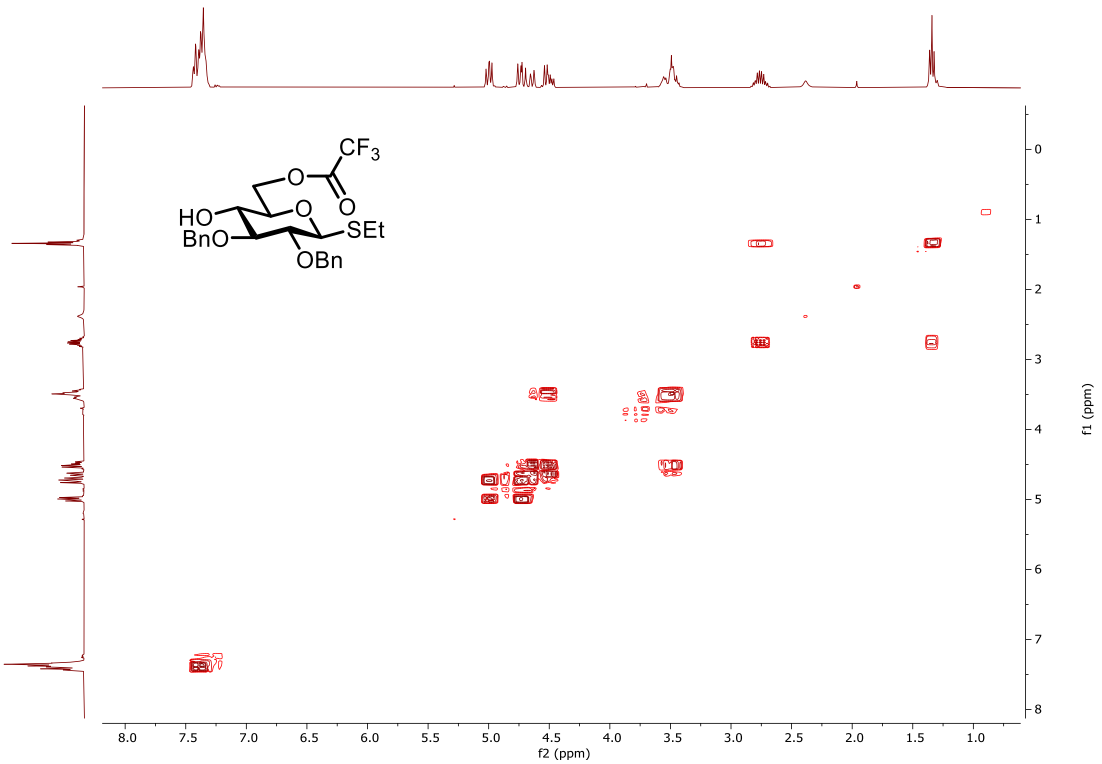

HSQC NMR of 60 ($CDCl_3$)

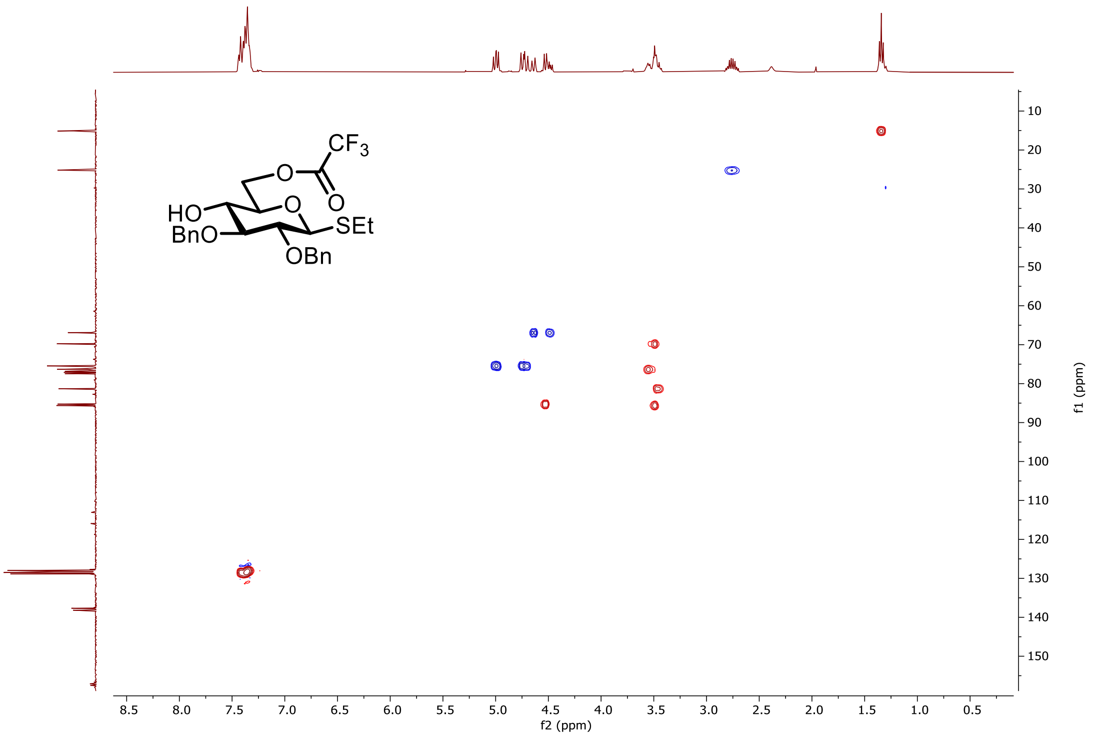

Ethyl 2,3-di-*O*-benzyl-4-*O*-(9-fluorenylmethoxycarbonyl)-6-*O*-trifluoroacetyl-1-thio-β-D-glucopyranoside, 18

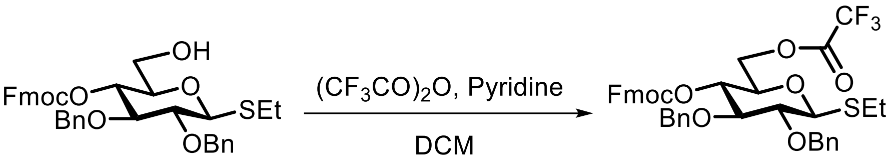

58 **18**

Ethyl 2,3-di-*O*-benzyl-4-*O*-(9-fluorenylmethoxycarbonyl)-1-thio-β-D-glucopyranoside **58** (1.20 g, 1.9 mmol) was dissolved in anhydrous DCM (20 mL) and pyridine (4 mL) was added. The solution was cooled with an ice bath for 30 min, trifluoroacetic anhydride (540 µL, 3.8 mmol) was added. The reaction was warmed to room temperature and stirred for 6 h. The reaction solution was directly loaded on top and purified by column chromatography (Hexane : EtOAc = 8:1→ Hexane : EtOAc : DCM =6:1:1) to give **18** as a white solid (1.10 g, 80%). ^{1}H NMR (400 MHz, $CDCl_3$) δ 7.79 (d, *J* = 7.6 Hz, 2H), 7.62 (d, *J* = 7.5 Hz, 1H), 7.58 (d, *J* = 7.5 Hz, 1H), 7.47 – 7.27 (m, 9H), 7.26 (s, 5H), 4.94 (d, *J* = 10.2 Hz, 1H), 4.90 – 4.80 (m, 2H), 4.73 (dd, *J* = 18.0, 10.7 Hz, 2H), 4.60 – 4.47 (m, 3H), 4.43 (dd, *J* = 10.6, 6.8 Hz, 1H), 4.35 (dd, *J* = 11.9, 2.5 Hz, 1H), 4.21 (appt, *J* = 6.8 Hz, 1H), 3.78 – 3.68 (m, 2H), 3.52 (appt, *J* = 9.3 Hz, 1H), 2.77 (qd, *J* = 12.8, 6.4 Hz, 2H), 1.36 (t, *J* = 7.4 Hz, 3H); ^{13}C NMR (101 MHz, $CDCl_3$) δ 157.11 (q, *J* = 42.9 Hz), 154.42, 143.11, 143.04, 141.38, 141.34, 137.77, 137.60, 128.50, 128.42, 128.39, 128.09, 128.01, 127.99, 127.83, 127.74, 127.25, 125.07, 124.89, 120.20, 120.16, 114.29 (q, *J* = 284.2 Hz), 85.28, 83.33, 81.03, 75.71, 74.73, 74.51, 70.20, 66.01, 46.79, 25.13, 15.12; $[\alpha]_D^{25}$ -4.48 (c = 1, $CHCl_3$); IR (neat) ν_{max} = 1791, 1753, 1257, 738 cm^{-1}; m/z (HRMS$^+$) [M + Na]$^+$ 745.2087 ($C_{39}H_{37}F_3O_8SNa^+$ requires 745.2053).

^{1}H NMR of 18 (400 MHz, $CDCl_3$)

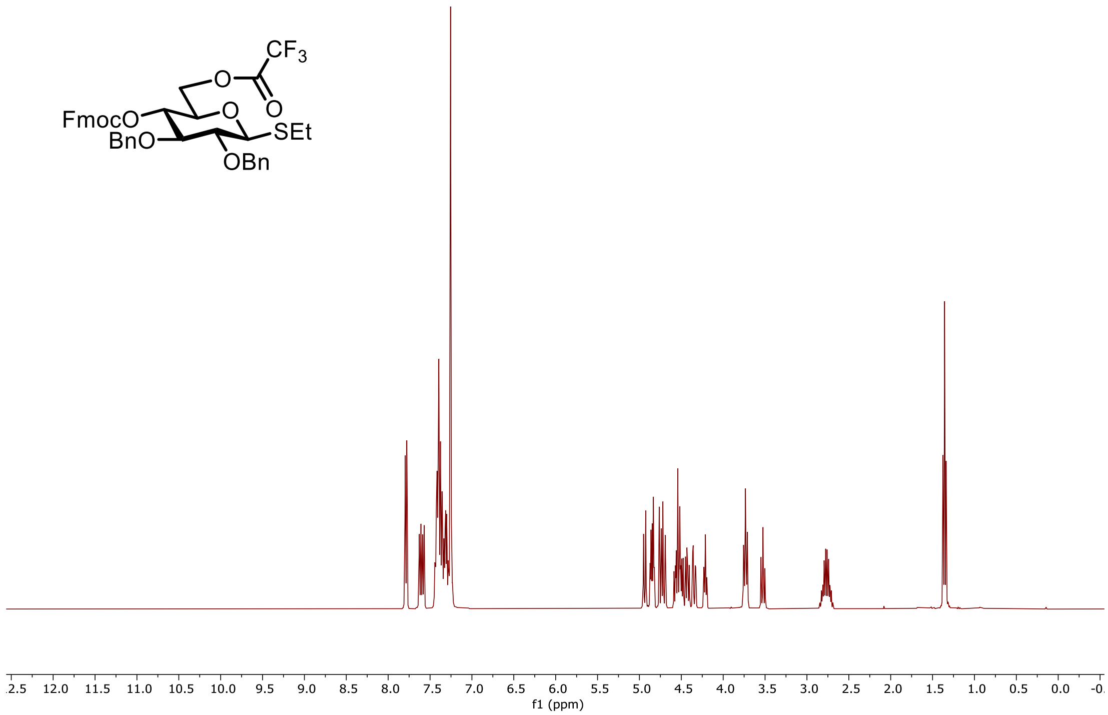

^{13}C NMR of 18 (101 MHz, $CDCl_3$)

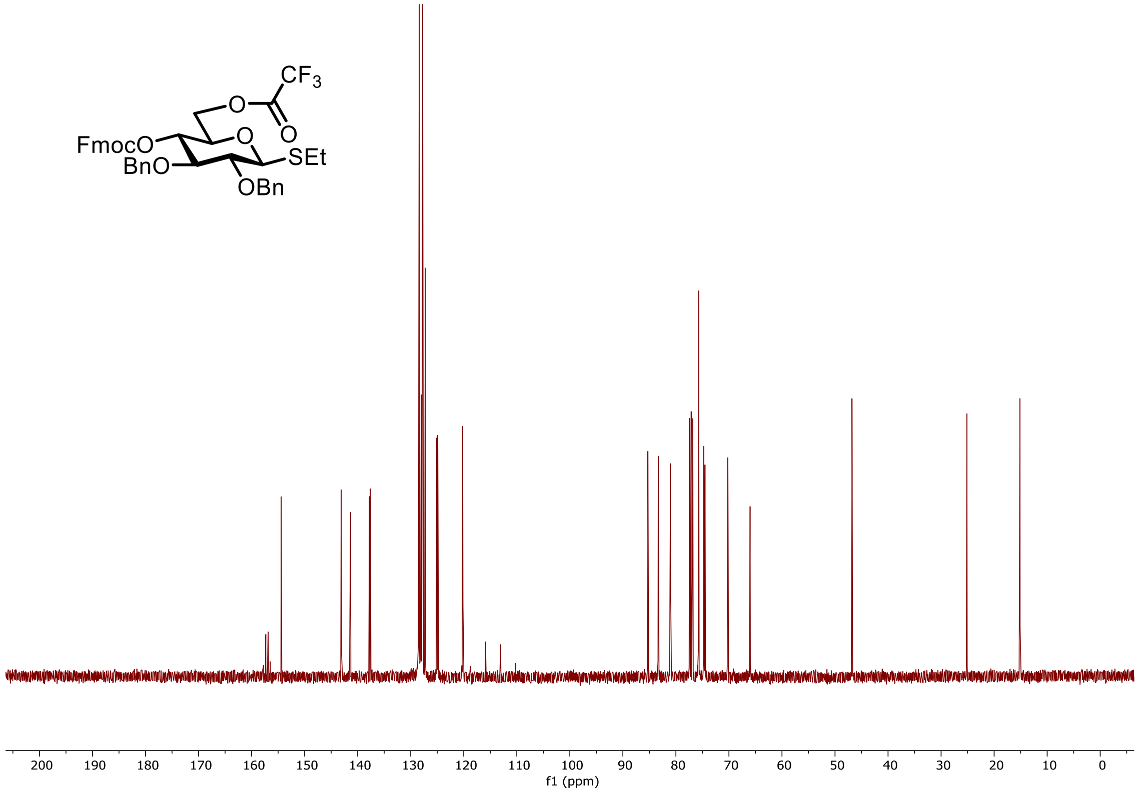

COSY NMR of 18 ($CDCl_3$)

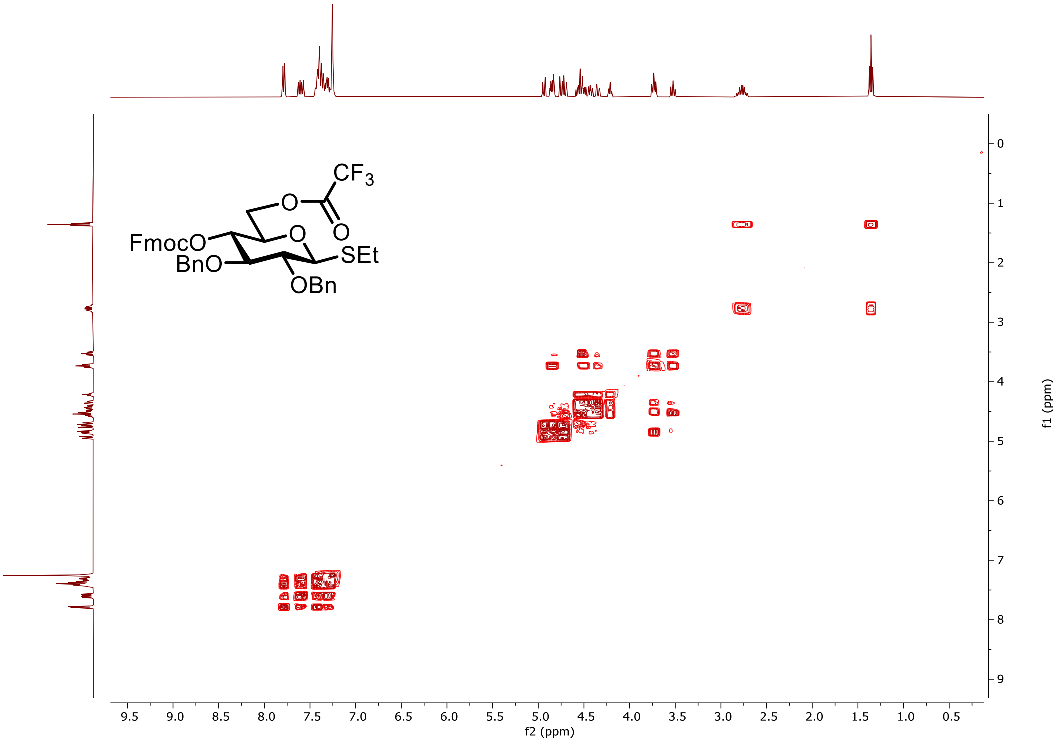

HSQC NMR of 18 ($CDCl_3$)

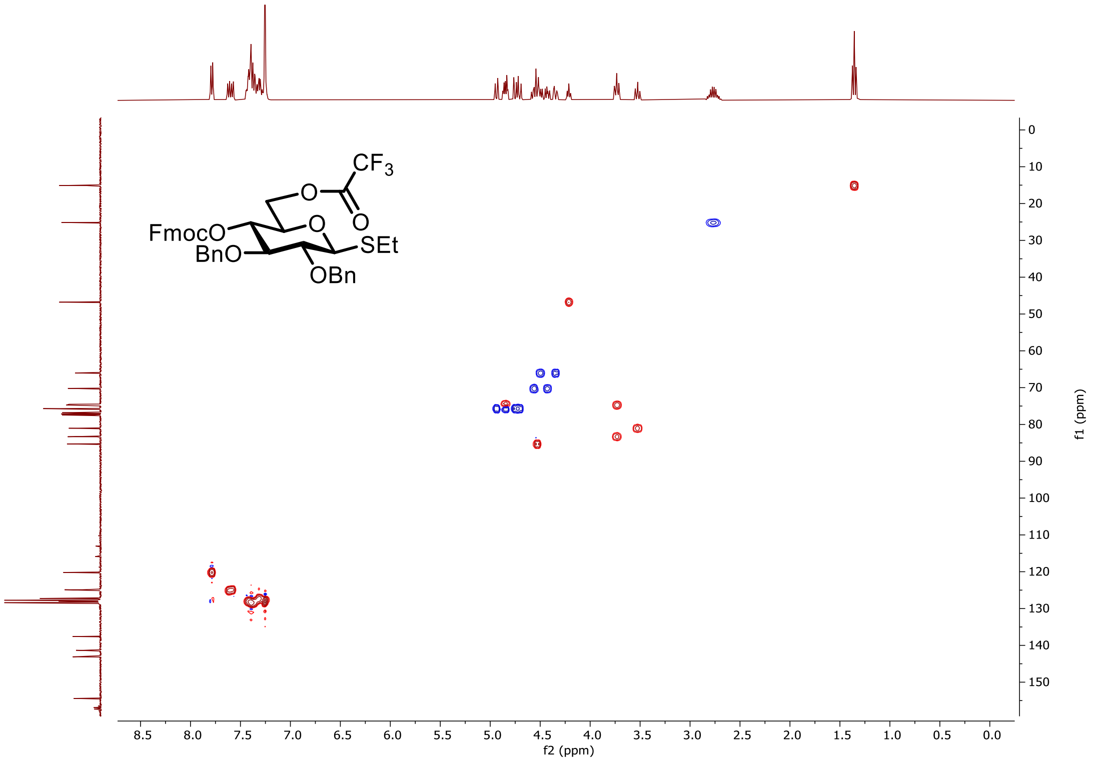

2.8 Synthesis of 19

Ethyl 2,3-di-*O*-benzyl-4-*O*-(9-fluorenylmethoxycarbonyl)-6-*O*-acryl-1-thio-β-D-glucopyranoside, 19

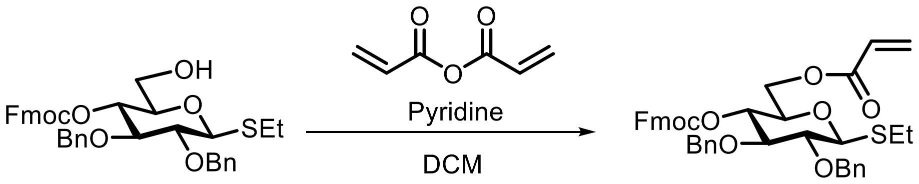

58 **19**

Acrylic acid (660 µL, 9.6 mmol) was dissolved in anhydrous THF (5 mL), the solution was cooled with an ice bath before *N,N'*-dicyclohexylcarbodiimide (DCC, 1.3 g, 6.4 mmol) was added. The reaction was allowed to warm to room temperature and kept stirring for 6 h. After filtration, the obtained acrylic anhydride was directly added into a solution made of ethyl 2,3-di-*O*-benzyl-4-*O*-(9-fluorenylmethoxycarbonyl)-1-thio-β-D-glucopyranoside **58** (900 mg, 1.4 mmol), anhydrous DCM (10 mL) and pyridine (3 mL). The reaction was stirred at room temperature for 16 h and its completion was confirmed by TLC (additional acrylic anhydride can be added if the reaction not was finished overnight). MeOH (2 mL) was added to quench the reaction and DCM (20 mL) was added 30 min later. The organic phase was washed with aqueous citric acid (0.5 M, 20 mL). After extracting the water phase with DCM (10 mL), the organic layers were combined and dried over Na_2SO_4, filtered, and evaporated. The resulting crude product was purified by column chromatography (Hexane : EtOAc = 8:1→Hexane : EtOAc : DCM =6:1:1) to give **8** as a white solid (763 mg, 80%). ^{1}H NMR (400 MHz, $CDCl_3$) δ 7.76 (d, *J* = 7.6 Hz, 2H), 7.60 (d, *J* = 7.5 Hz, 1H), 7.56 (d, *J* = 7.6 Hz, 1H), 7.44 – 7.24 (m, 9H), 7.22 (s, 5H), 6.43 (d, *J* = 17.3 Hz, 1H), 6.13 (dd, *J* = 17.3, 10.4 Hz, 1H), 5.83 (d, *J* = 10.4 Hz, 1H), 4.97 – 4.88 (m, 2H), 4.84 (d, *J* = 11.2 Hz, 1H), 4.74 (d, *J* = 10.3 Hz, 1H), 4.69 (d, *J* = 11.2 Hz, 1H), 4.52 (d, *J* = 9.8 Hz, 1H), 4.46 (dd, *J* = 10.5, 7.0 Hz, 1H), 4.38 – 4.25 (m, 3H), 4.19 (appt, *J* = 7.1 Hz, 1H), 3.78 – 3.67 (m, 2H), 3.52 (appt, *J* = 9.3 Hz, 1H), 2.86 – 2.67 (m, 2H), 1.34 (t, *J* = 7.4 Hz, 3H); ^{13}C NMR (101 MHz, $CDCl_3$) δ 165.65, 154.34, 143.22, 143.16, 141.28, 137.87, 137.67, 131.39, 128.45, 128.40, 128.32, 128.01, 127.95, 127.92, 127.72, 127.22, 125.11, 124.99, 120.08, 120.07, 85.27, 83.69, 81.24, 75.63, 75.44, 74.76, 70.23, 62.97, 46.70, 25.14, 15.15; $[\alpha]_D^{25}$ 2.30 (c = 1, $CHCl_3$); IR (neat) ν_{max} = 1753, 1730, 1256, 740 cm^{-1}; m/z (HRMS$^+$) [M + Na]$^+$ 703.2398 ($C_{40}H_{40}O_8SNa^+$ requires 703.2336).

^{1}H NMR of 19 (400 MHz, $CDCl_3$)

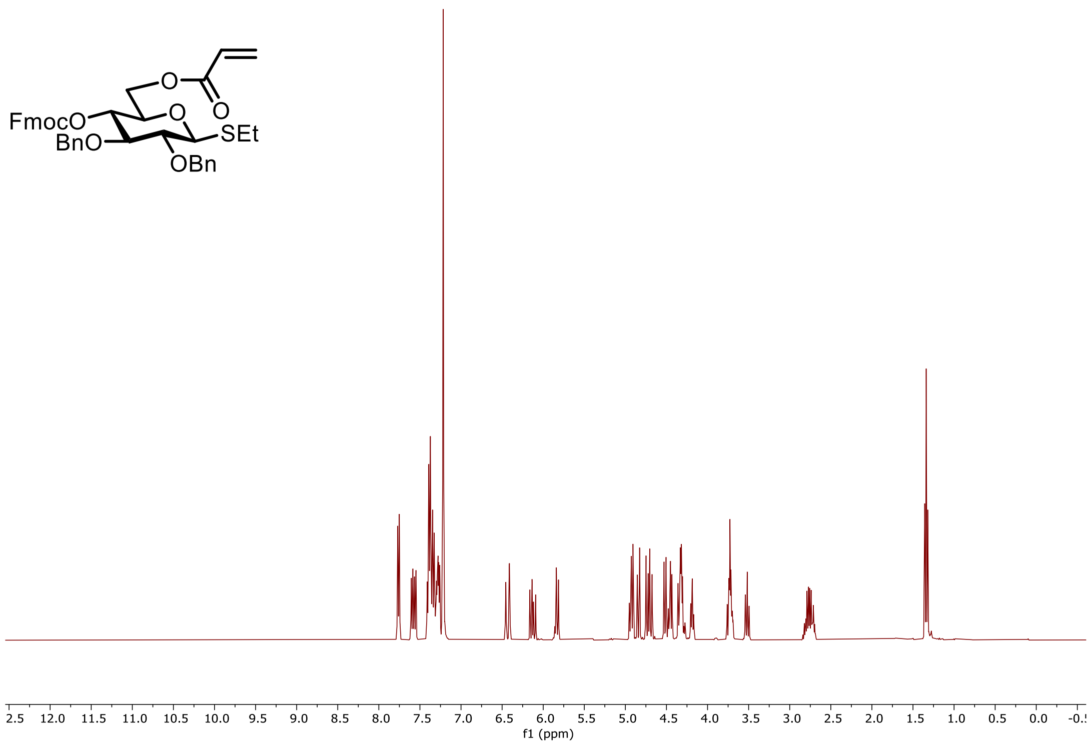

^{13}C NMR of 19 (101 MHz, $CDCl_3$)

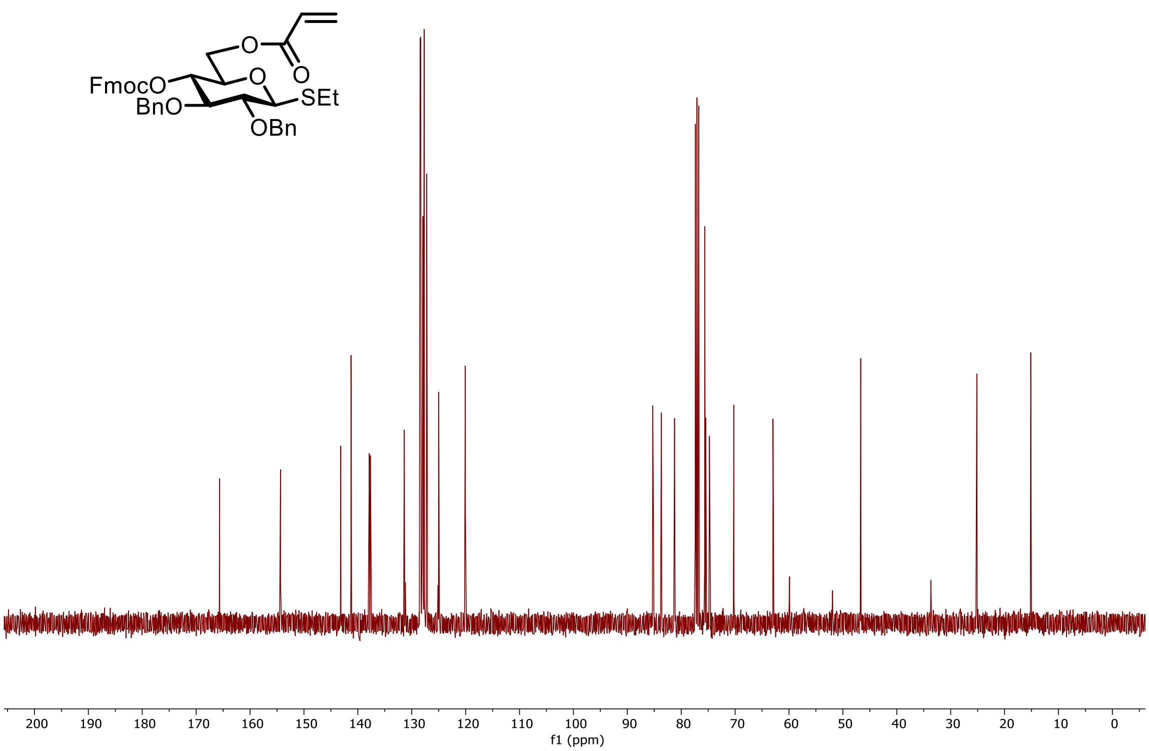

COSY NMR of 19 ($CDCl_3$)

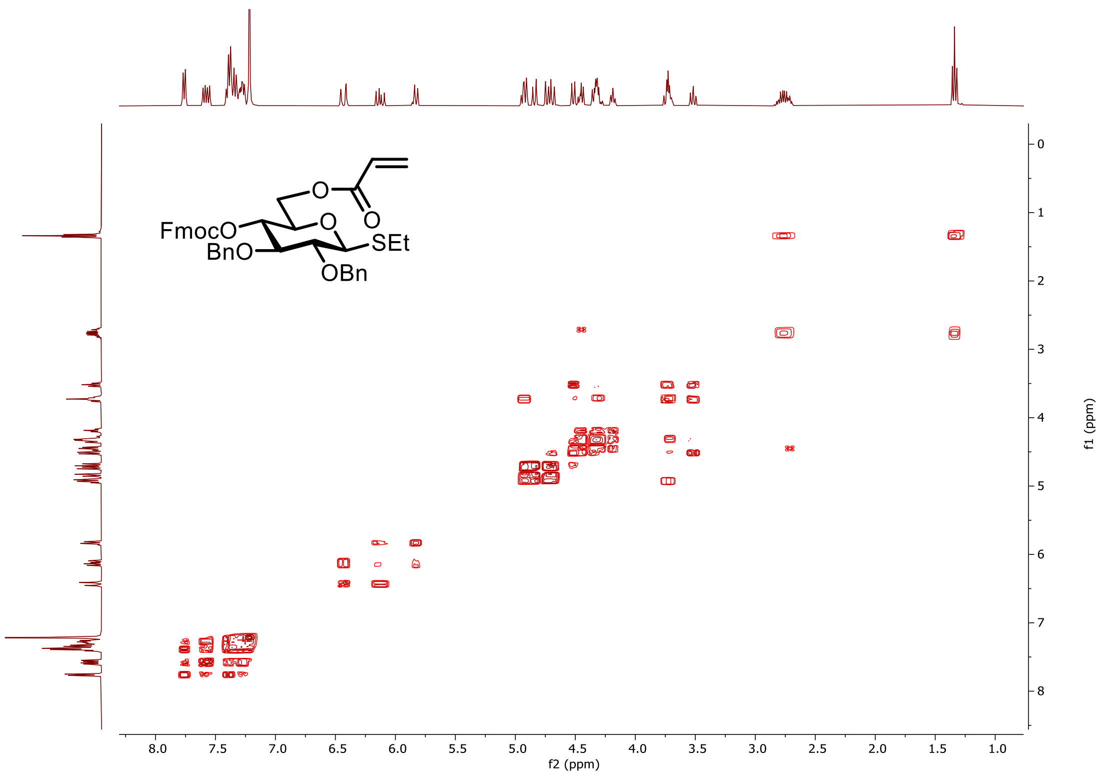

HSQC NMR of 19 ($CDCl_3$)

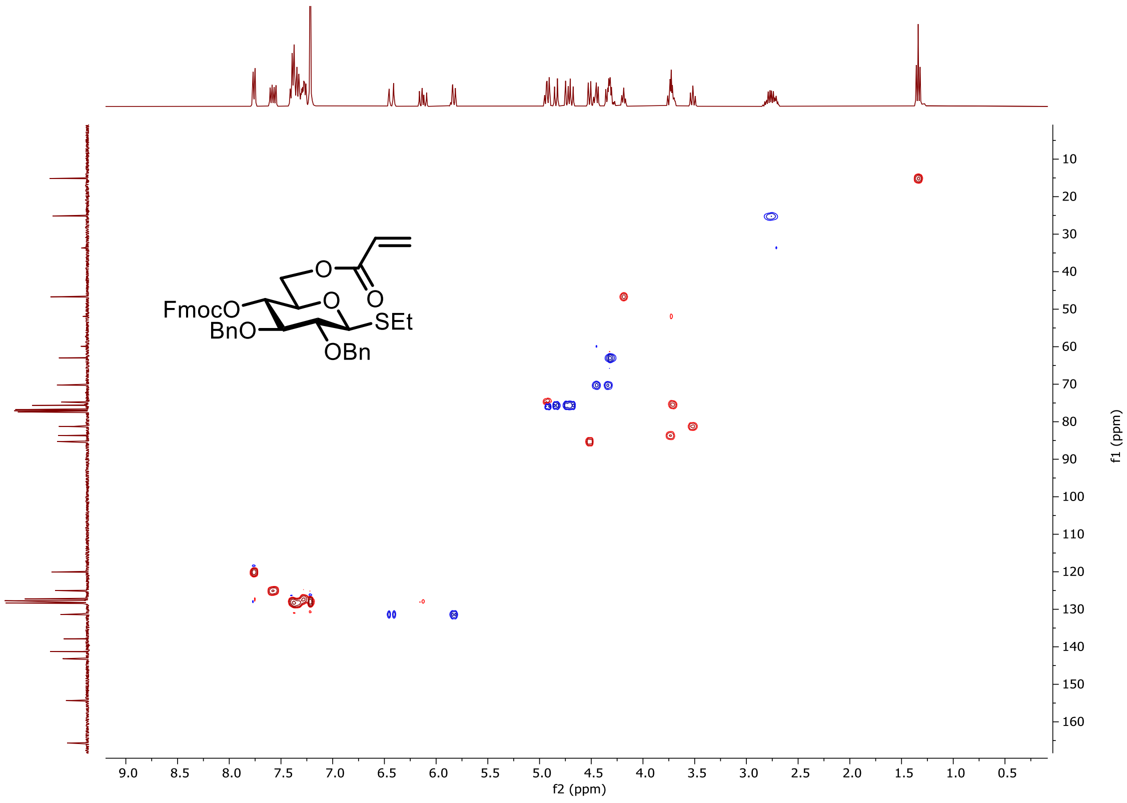

2.9 Synthesis of 20

Ethyl 2,3-di-*O*-benzyl-6-*O*-benzoyl-1-thio-β-D-glucopyranoside, 61

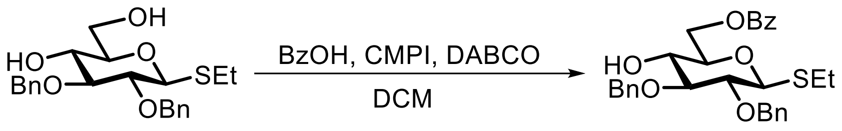

53 **61**

53 was prepared according to previously established procedures. [2]

Ethyl 2,3-di-*O*-benzyl-1-thio-β-D-glucopyranoside **53** (500 mg, 1.2 mmol) was dissolved in anhydrous DCM (15 mL), 2-chloro-1-methylpyridium iodide (CMPI, 792 mg, 3.1 mmol) and 1,4-diazabicyclo[2.2.2]octane (DABCO, 561 mg, 5.0 mmol) were added at room temperature. The reaction mixture was cooled to -15 °C, and benzoic acid (BzOH, 130 μL, 1.3 mmol) was added slowly. After 6 h, DCM (20 mL) was added and the organic phase was washed with aqueous saturated $NaHCO_3$ (50 mL), and the water phase was extracted with DCM (20 mL). The obtained organic layers were combined and dried over Na_2SO_4, filtered, and evaporated. The resulting crude product was purified by column chromatography (Hexane : EtOAc = 6:1→2:1) to give **61** as a white solid (220 mg, 35%). ^{1}H NMR (400 MHz, $CDCl_3$) δ 8.12 – 8.06 (m, 2H), 7.65 – 7.56 (m, 1H), 7.51 – 7.42 (m, 4H), 7.43 – 7.30 (m, 8H), 5.01 (d, *J* = 5.8 Hz, 1H), 4.99 (d, *J* = 4.7 Hz, 1H), 4.86 (d, *J* = 11.3 Hz, 1H), 4.80 (d, *J* = 10.2 Hz, 1H), 4.64 (appt, *J* = 2.6 Hz, 2H), 4.59 (d, *J* = 9.7 Hz, 1H), 3.69 – 3.56 (m, 3H), 3.50 (appt, *J* = 8.9 Hz, 1H), 2.90 – 2.70 (m, 2H), 1.36 (t, *J* = 7.4 Hz, 3H); ^{13}C NMR (101 MHz, $CDCl_3$) δ 166.95, 138.38, 137.88, 133.28, 129.86, 129.78, 128.73, 128.52, 128.44, 128.08, 128.04, 85.83, 85.25, 81.37, 77.76, 75.67, 75.53, 70.31, 64.16, 25.18, 15.26; $[\alpha]_D^{25}$ -31.59 (c = 1, $CHCl_3$); IR (neat) ν_{max} = 1721, 1276, 1063, 712 cm^{-1}; m/z ($HRMS^+$) $[M + Na]^+$ 531.1814 ($C_{29}H_{32}O_6SNa^+$ requires 531.1812).

^{1}H NMR of 61 (400 MHz, $CDCl_3$)

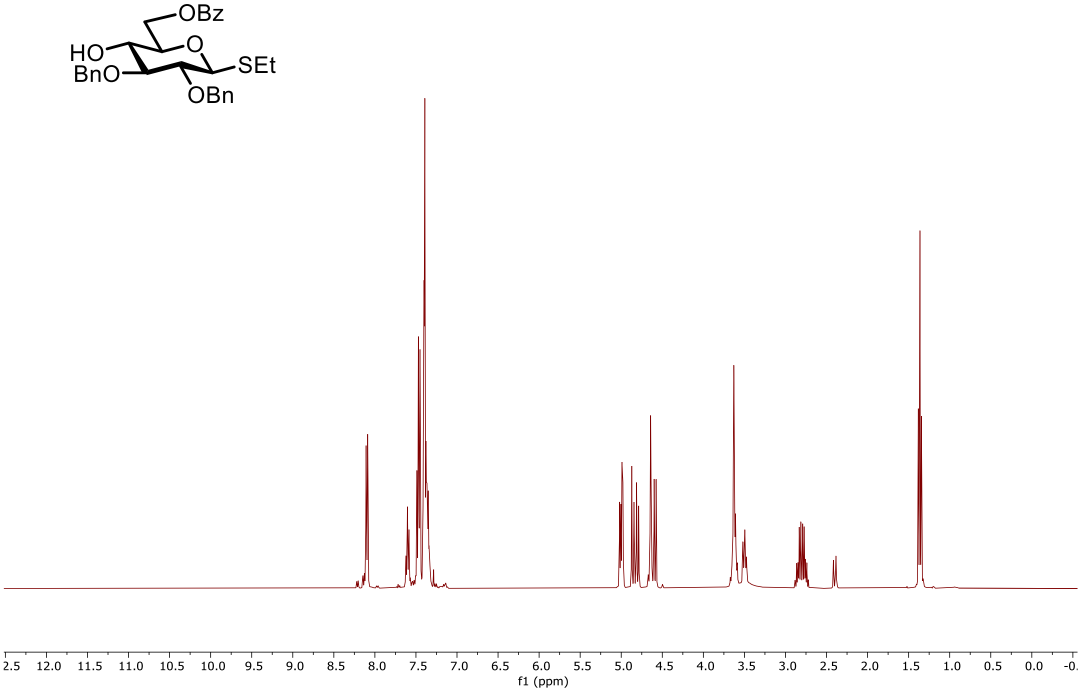

^{13}C NMR of 61 (101 MHz, $CDCl_3$)

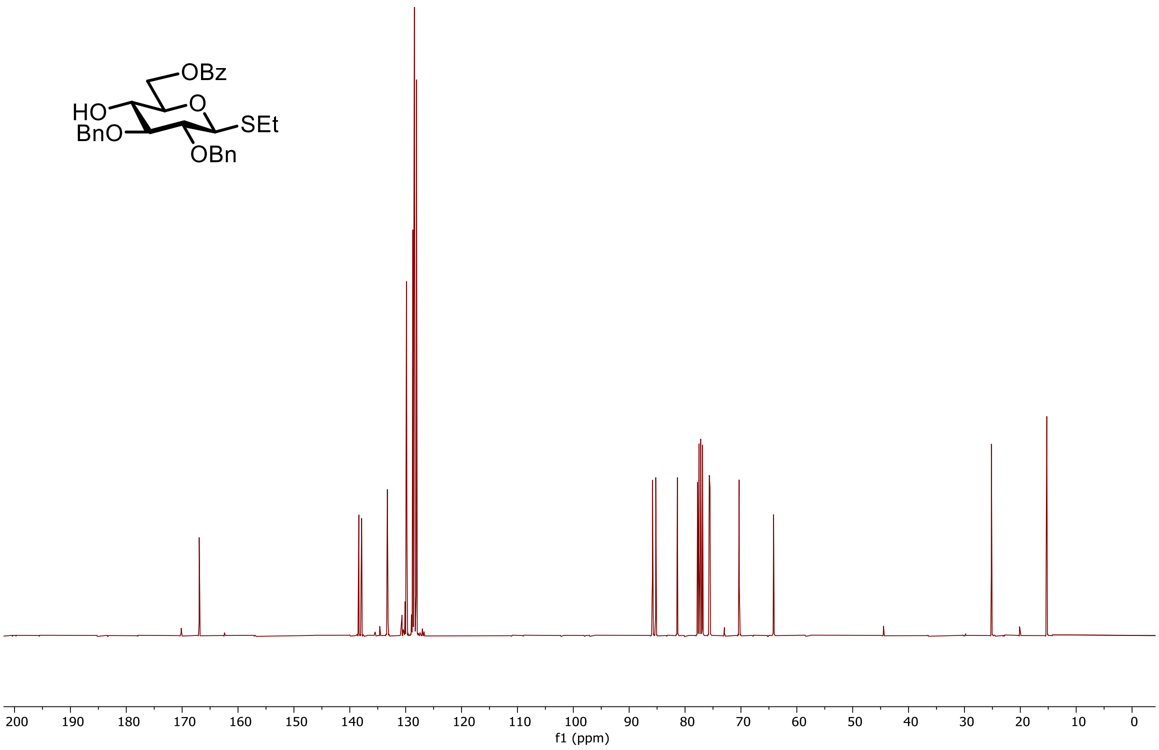

COSY NMR of 61 ($CDCl_3$)

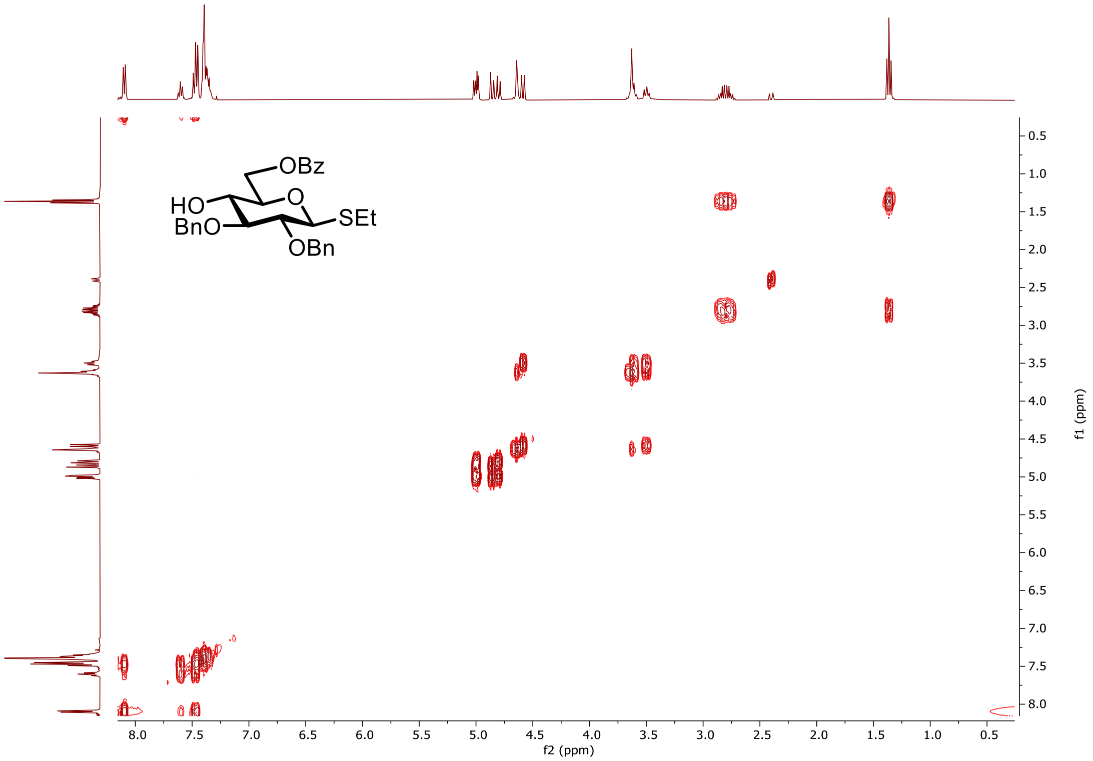

HSQC NMR of 61 ($CDCl_3$)

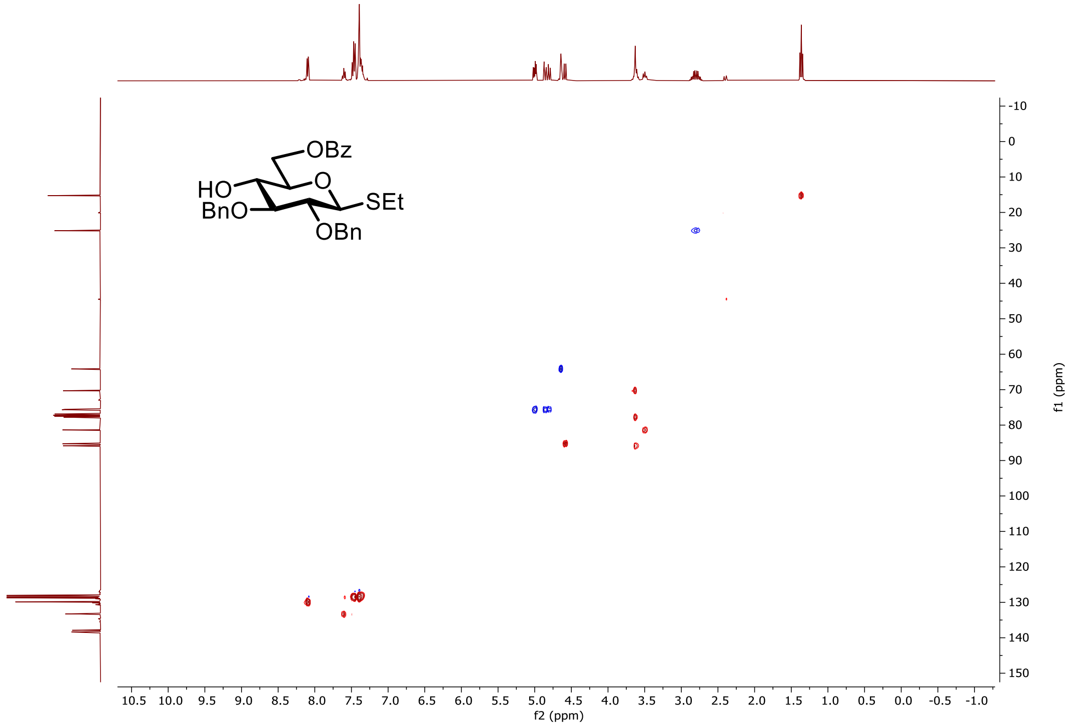

Ethyl 2,3-di-*O*-benzyl-4-*O*-(9-fluorenylmethoxycarbonyl)-6-*O*-benzoyl-1-thio-β-D-glucopyranoside, 20

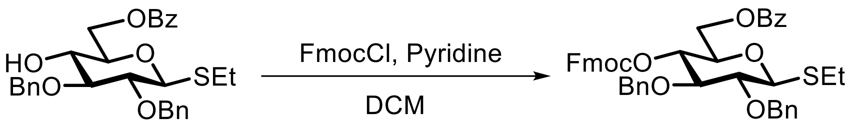

61 **20**

Ethyl 2,3-di-*O*-benzyl-6-*O*-benzoyl-1-thio-β-D-glucopyranoside **61** (220 mg, 0.43 mmol) was dissolved in anhydrous DCM (2 mL) and pyridine (0.5 mL) was added. The solution was cooled with an ice bath for 30 min, and fluorenylmethyloxycarbonyl chloride (FmocCl, 280 mg, 1.1 mmol) was added. The reaction was warmed to room temperature and stirred for 6 h. Upon completion, DCM (10 mL) was added and the organic phase was washed with aqueous citric acid (0.5 M, 10 mL). After extracting the water phase with DCM (10 mL), the organic layers were combined and dried over Na_2SO_4, filtered, and evaporated. The resulting crude product was purified by column chromatography (Hexane : EtOAc = 8:1→Hexane : EtOAc : DCM =6:1:1) to give **20** as a white solid (246 mg, 78%). ^{1}H NMR (400 MHz, $CDCl_3$) δ 8.11 (d, *J* = 7.7 Hz, 2H), 7.77 (d, *J* = 7.6 Hz, 2H), 7.64 – 7.52 (m, 3H), 7.49 – 7.32 (m, 9H), 7.33 – 7.19 (m, 7H), 5.05 (appt, *J* = 9.7 Hz, 1H), 4.96 (d, *J* = 10.2 Hz, 1H), 4.88 (d, *J* = 11.1 Hz, 1H), 4.75 (dd, *J* = 16.2, 10.7 Hz, 2H), 4.64 – 4.54 (m, 2H), 4.49 – 4.38 (m, 2H), 4.33 (dd, *J* = 10.5, 7.3 Hz, 1H), 4.17 (appt, *J* = 7.1 Hz, 1H), 3.88 – 3.75 (m, 2H), 3.58 (appt, *J* = 9.3 Hz, 1H), 2.77 (qt, *J* = 14.8, 6.4 Hz, 2H), 1.34 (t, *J* = 7.4 Hz, 3H); ^{13}C NMR (101 MHz, $CDCl_3$) δ 166.14, 154.42, 143.29, 143.12, 141.29, 141.27, 137.86, 137.71, 133.18, 129.82, 129.80, 128.50, 128.44, 128.42, 128.38, 128.06, 127.95, 127.93, 127.82, 127.80, 127.25, 125.18, 124.99, 120.11, 120.08, 85.23, 83.75, 81.34, 75.76, 75.69, 75.54, 74.96, 70.21, 63.45, 46.71, 25.11, 15.20; $[\alpha]_D^{25}$ 1.31 (c = 1, $CHCl_3$); IR (neat) ν_{max} = 1752, 1723, 1250, 738 cm^{-1}; m/z (HRMS$^+$) [M + Na]$^+$ 753.2517 ($C_{44}H_{42}O_8SNa^+$ requires 753.2493).

^{1}H NMR of 20 (400 MHz, CDCl$_3$)

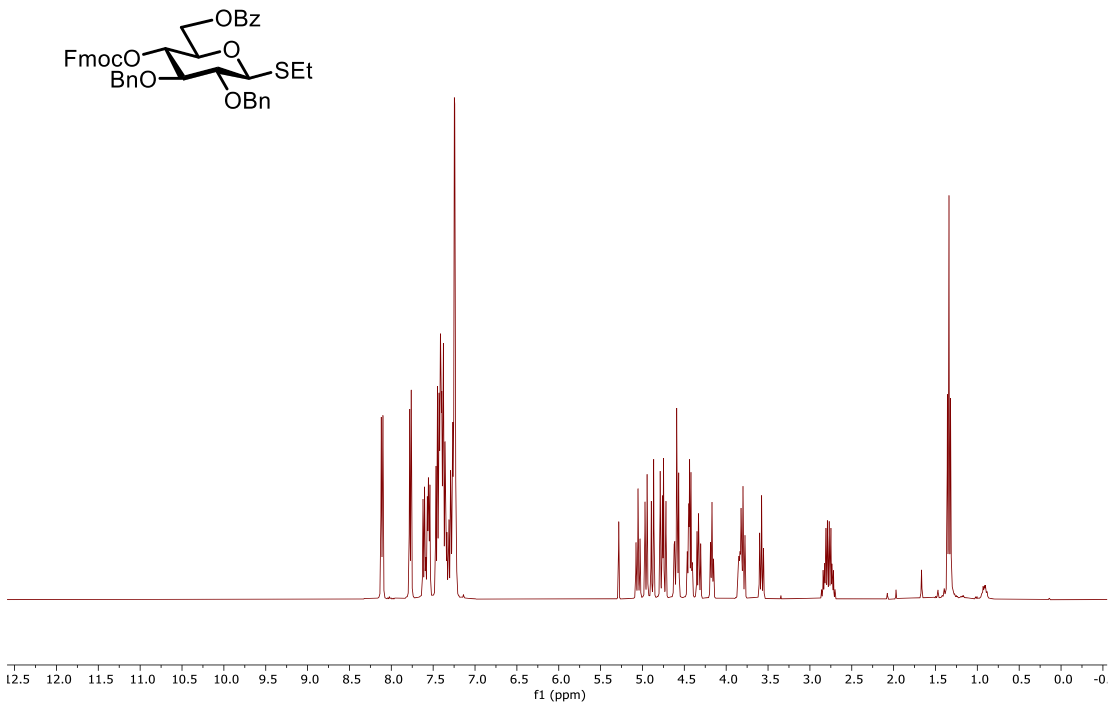

^{13}C NMR of 20 (101 MHz, CDCl$_3$)

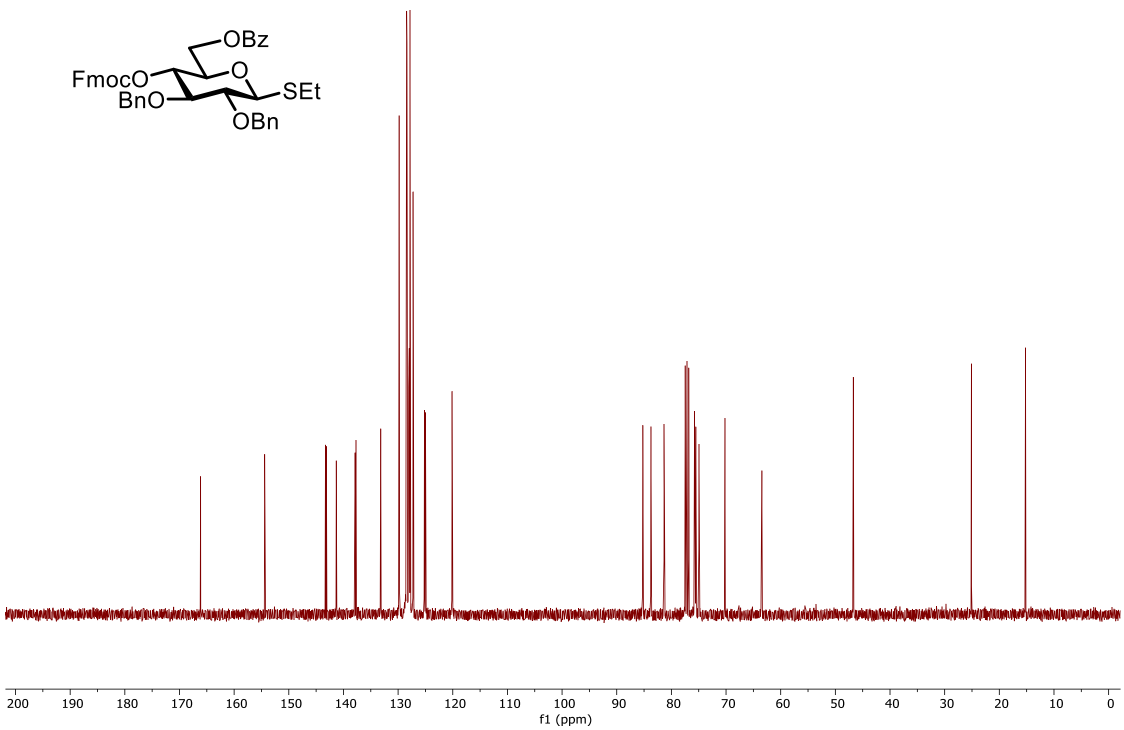

COSY NMR of 20 ($CDCl_3$)

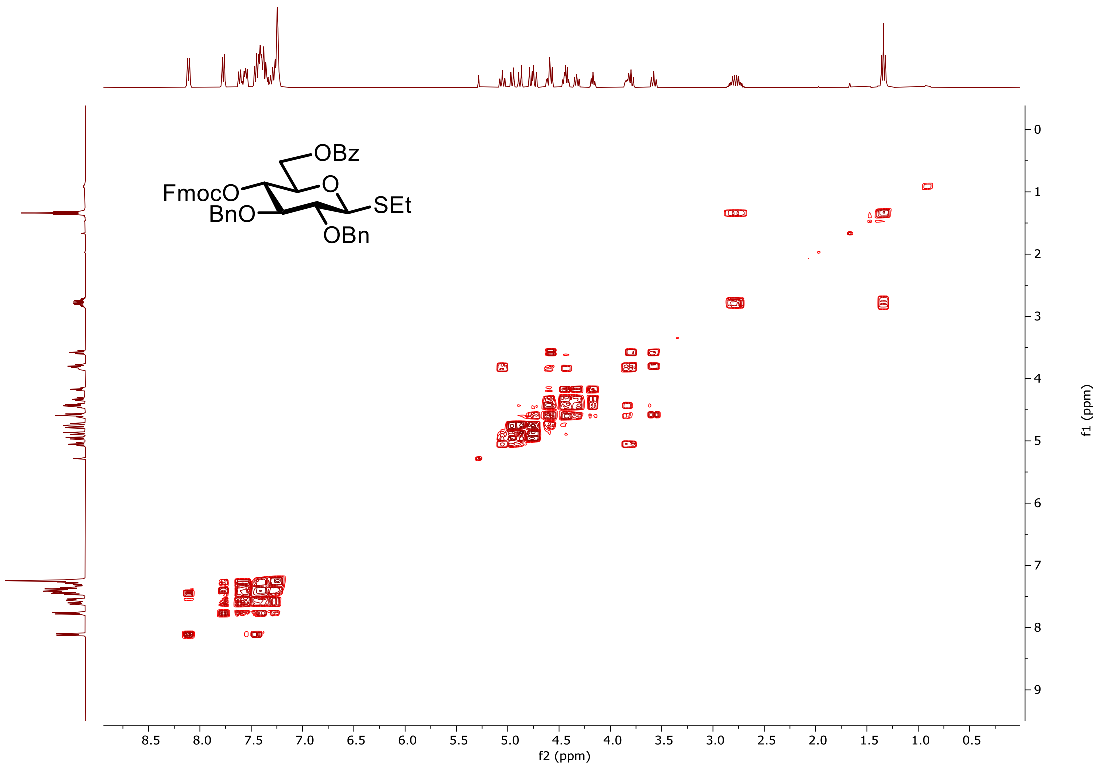

HSQC NMR of 20 ($CDCl_3$)

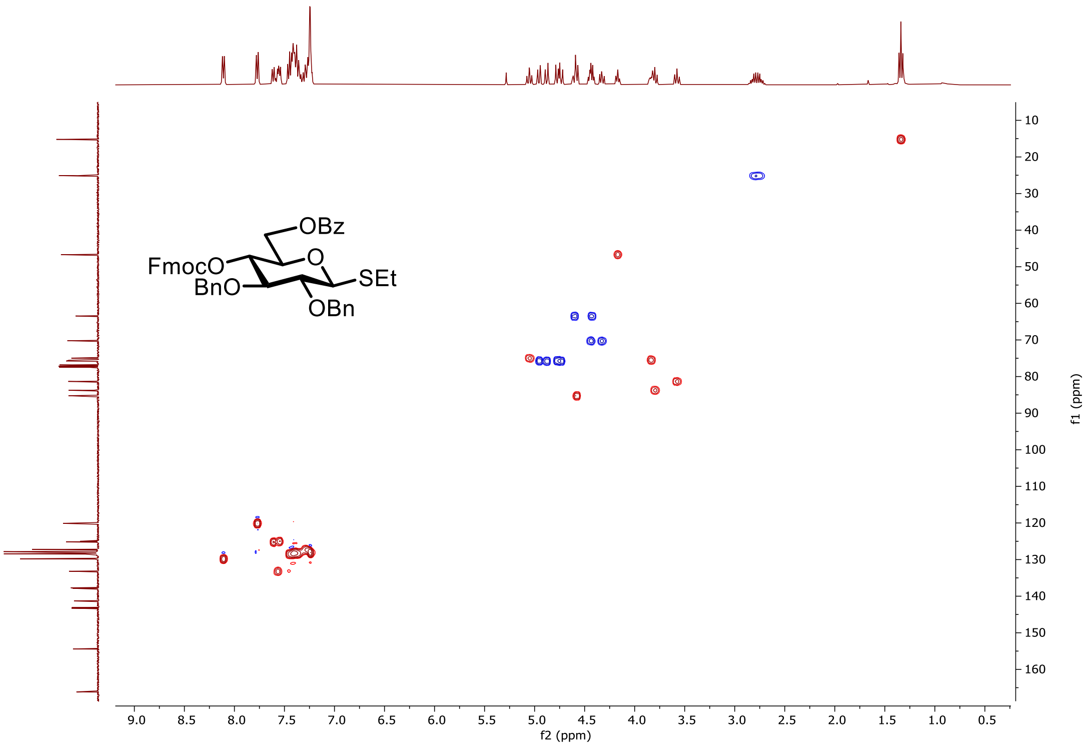

2.10 Synthesis of 21

Ethyl 2,3-di-*O*-benzyl-4-*O*-(9-fluorenylmethoxycarbonyl)-6-*O*-(4-methylbenzoyl)-1-thio-β-D-glucopyranoside, 21

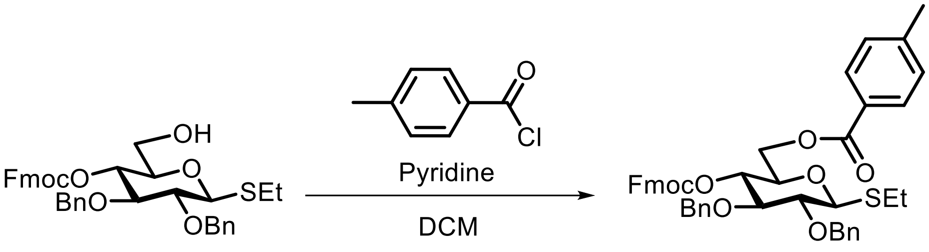

58 **21**

4-Methylbenzoic acid (440 mg, 3.2 mmol) was dissolved in anhydrous DCM (5 mL), the obtained solution was protected with argon and cooled with an ice bath for 30 min. After oxalyl chloride (360 µL, 4.2 mmol) was added, two drops of DMF were added to start the reaction. The reaction was allowed to warm to room temperature and kept stirring for 2 h. The solvent and remaining oxalyl chloride were removed under high vacuum, the crude product was coevaporated with toluene (5 mL × 3). The obtained 4-methylbenzoyl chloride was dissolved in anhydrous DCM (5 mL) and slowly added into an ice bath-precooled solution made of ethyl 2,3-di-*O*-benzyl-4-*O*-(9-fluorenylmethoxycarbonyl)-1-thio-β-D-glucopyranoside **58** (1.00 g, 1.6 mmol), anhydrous DCM (20 mL) and pyridine (5 mL). After 30 min, the reaction was warmed up and stirred at room temperature for 16 h. Its completion was confirmed by TLC (additional 4-methylbenzoyl chloride can be added if the reaction not finished overnight). MeOH (2 mL) was added to quench the reaction and DCM (20 mL) was added 30 min later. The organic phase was washed with aqueous citric acid (0.5 M, 20 mL). After extracting the water phase with DCM (20 mL), the organic layers were combined and dried over Na_2SO_4, filtered, and evaporated. The resulting crude product was purified by column chromatography (Hexane : EtOAc = 8:1→Hexane : EtOAc : DCM =6:1:1) to give **21** as a white solid (1.06 g, 89%). ^{1}H NMR (400 MHz, $CDCl_3$) δ 8.01 (d, *J* = 8.0 Hz, 2H), 7.78 (d, *J* = 7.6 Hz, 2H), 7.62 (d, *J* = 7.6 Hz, 1H), 7.56 (d, *J* = 7.5 Hz, 1H), 7.47 – 7.33 (m, 7H), 7.33 – 7.18 (m, 9H), 5.06 (appt, *J* = 9.7 Hz, 1H), 4.97 (d, *J* = 10.3 Hz, 1H), 4.89 (d, *J* = 11.2 Hz, 1H), 4.77 (dd, *J* = 14.8, 10.7 Hz, 2H), 4.59 (dd, *J* = 9.5, 2.5 Hz, 2H), 4.50 – 4.38 (m, 2H), 4.34 (dd, *J* = 10.5, 7.3 Hz, 1H), 4.17 (appt, *J* = 7.1 Hz, 1H), 3.89 – 3.76 (m, 2H), 3.59 (appt, *J* = 9.3 Hz, 1H), 2.90 – 2.69 (m, 2H), 2.41 (s, 3H), 1.35 (t, *J* = 7.5 Hz, 3H); ^{13}C NMR (101 MHz, $CDCl_3$) δ 166.19, 154.42, 143.86, 143.33, 143.17, 141.31, 141.28, 137.91, 137.76, 129.84, 129.15, 128.50, 128.44, 128.38, 128.05, 127.95, 127.83, 127.79, 127.26, 127.10, 125.19, 125.02, 120.11, 120.08, 85.22, 83.78, 81.37, 75.76, 75.69, 75.56, 75.12, 70.20, 63.41, 46.73, 25.10, 21.73, 15.23; $[\alpha]_D^{25}$ 0.68 (c = 1, $CHCl_3$); IR (neat) ν_{max} = 1754, 1721, 1255, 740 cm^{-1}; m/z ($HRMS^+$) $[M + Na]^+$ 767.2726 ($C_{45}H_{44}O_8SNa^+$ requires 767.2649).

^{1}H NMR of 21 (400 MHz, $CDCl_3$)

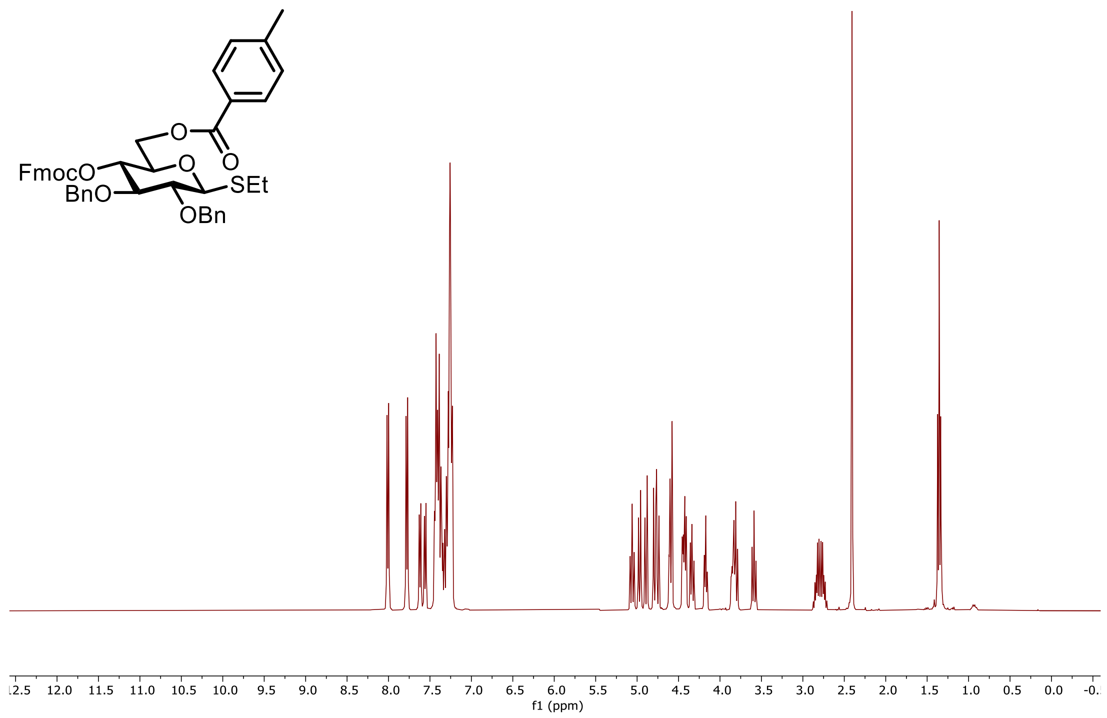

^{13}C NMR of 21 (101 MHz, $CDCl_3$)

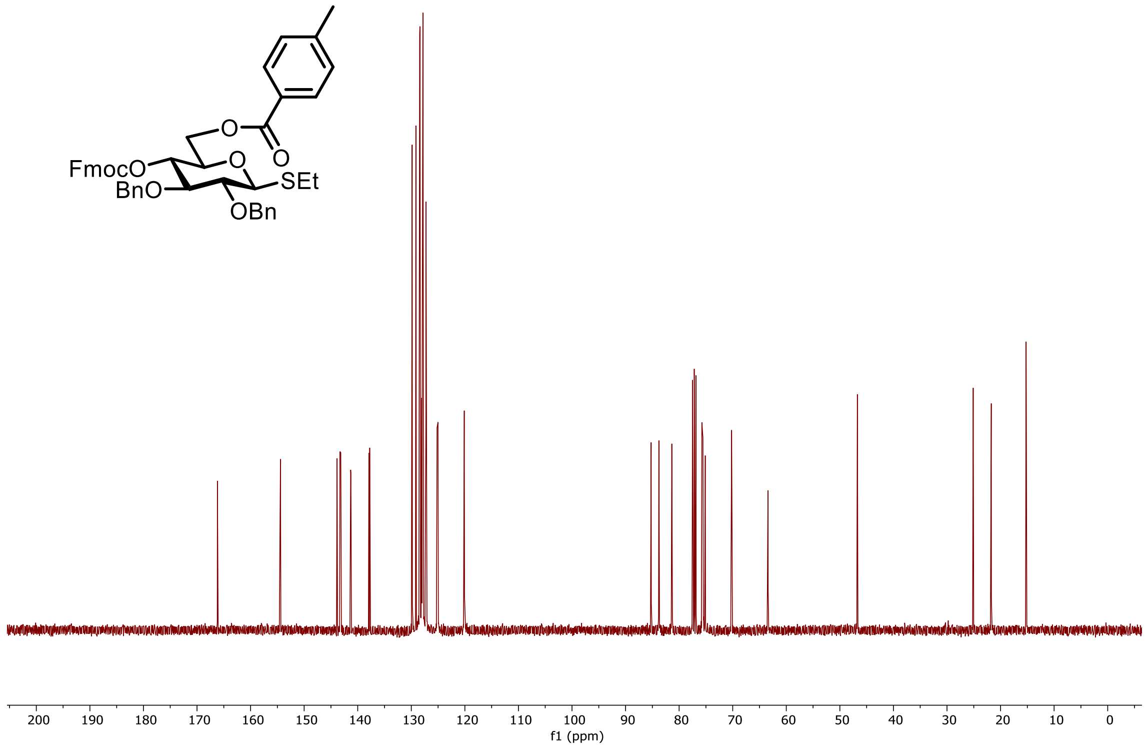

COSY NMR of 21 ($CDCl_3$)

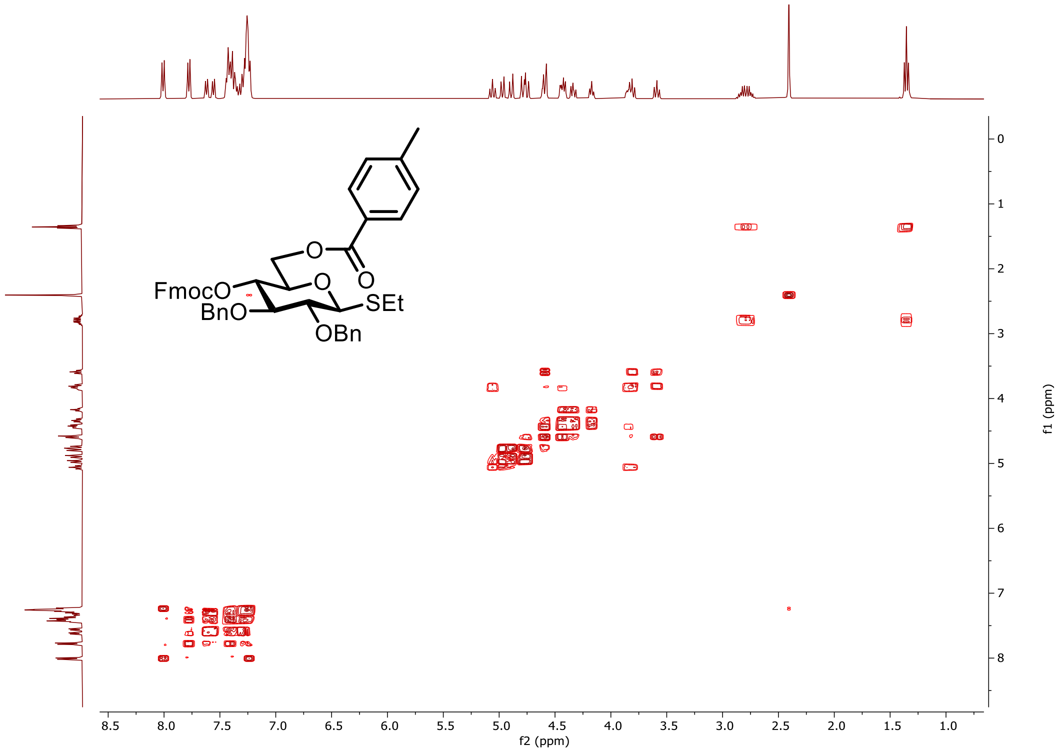

HSQC NMR of 21 ($CDCl_3$)

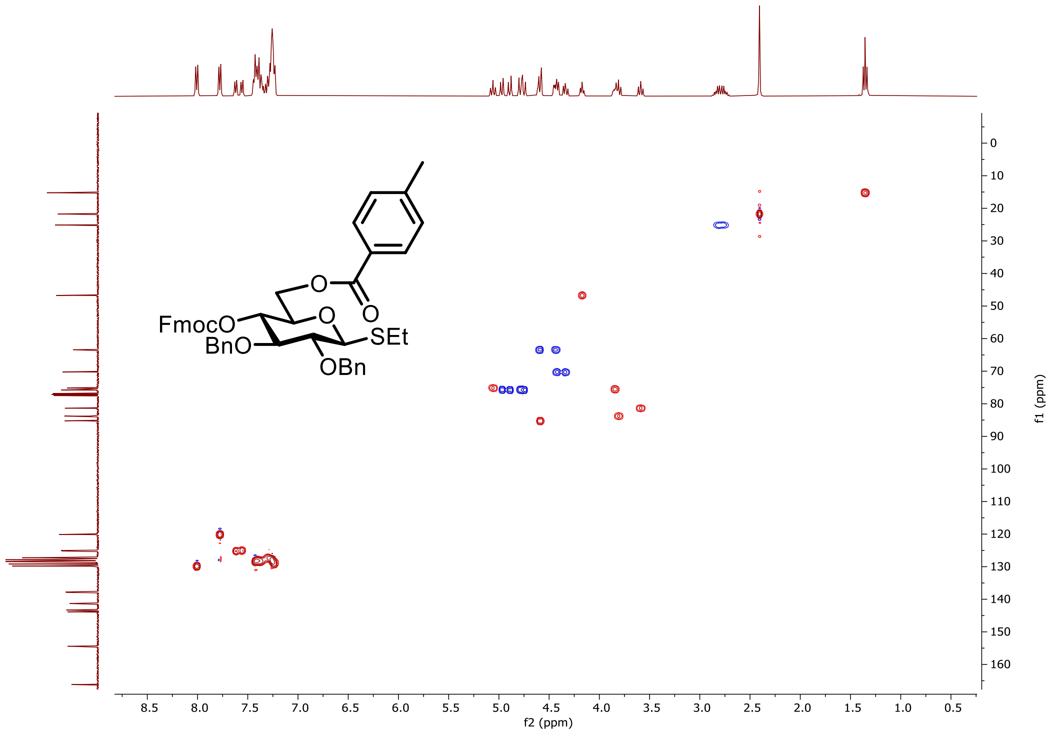

2.11 Synthesis of 22

Ethyl 2,3-di-*O*-benzyl-4-*O*-(9-fluorenylmethoxycarbonyl)-6-*O*-(4-methoxybenzoyl)-1-thio-β-D-glucopyranoside, 22

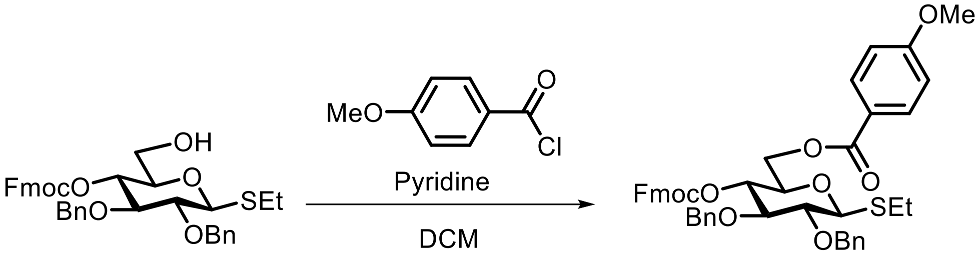

58 **22**

4-Methoxybenzoic acid (973 mg, 6.4 mmol) was dissolved in anhydrous DCM (10 mL), the obtained solution was protected with argon and cooled with an ice bath for 30 min. After oxalyl chloride (720 µL, 8.4 mmol) was added, two drops of DMF were added to start the reaction. The reaction was allowed to warm to room temperature and kept stirring for 2 h. The solvent and remaining oxalyl chloride were removed under high vacuum, the crude product was coevaporated with toluene (5 mL × 3). The obtained 4-methoxybenzoyl chloride was dissolved in anhydrous DCM (10 mL) and added into an ice bath-precooled solution made of ethyl 2,3-di-*O*-benzyl-4-*O*-(9-fluorenylmethoxycarbonyl)-1-thio-β-D-glucopyranoside **58** (1.00 g, 1.6 mmol), anhydrous DCM (30 mL) and pyridine (10 mL). After 30 min, the reaction was warmed up and stirred at room temperature for 16 h. Its completion was confirmed by TLC and MeOH (5 mL) was added to quench the reaction. DCM (30 mL) was added 30 min later, the organic phase was washed with aqueous citric acid (0.5 M, 30 mL). After extracting the water phase with DCM (30 mL), the organic layers were combined and dried over Na_2SO_4, filtered, and evaporated. The resulting crude product was purified by column chromatography (Hexane : EtOAc = 8:1→Hexane : EtOAc : DCM =6:1:1) to give **22** as a white solid (1.04 g, 85%). ^{1}H NMR (400 MHz, $CDCl_3$) δ 8.04 (d, *J* = 8.7 Hz, 2H), 7.76 (d, *J* = 7.5 Hz, 2H), 7.59 (d, *J* = 7.7 Hz, 1H), 7.54 (d, *J* = 7.5 Hz, 1H), 7.43 – 7.17 (m, 14H), 6.94 – 6.87 (m, 2H), 5.03 (appt, *J* = 9.7 Hz, 1H), 4.94 (d, *J* = 10.2 Hz, 1H), 4.87 (d, *J* = 11.2 Hz, 1H), 4.74 (dd, *J* = 15.7, 10.7 Hz, 2H), 4.60 – 4.53 (m, 2H), 4.44 – 4.34 (m, 2H), 4.31 (dd, *J* = 10.3, 7.4 Hz, 1H), 4.15 (appt, *J* = 7.2 Hz, 1H), 3.85 – 3.70 (m, 5H), 3.56 (appt, *J* = 9.3 Hz, 1H), 2.85 – 2.67 (m, 2H), 1.33 (t, *J* = 7.4 Hz, 3H); ^{13}C NMR (101 MHz, $CDCl_3$) δ 165.84, 163.50, 154.38, 143.29, 143.13, 141.28, 141.25, 137.87, 137.71, 131.83, 128.47, 128.41, 128.35, 128.03, 127.91, 127.80, 127.76, 127.22, 125.16, 124.99, 122.19, 120.08, 120.05, 113.64, 85.21, 83.77, 81.35, 75.74, 75.66, 75.58, 75.03, 70.22, 63.23, 55.42, 46.70, 25.09, 15.19; $[\alpha]_D^{25}$ 2.84 (c = 1, $CHCl_3$); IR (neat) ν_{max} = 1752, 1716, 1254, 1029, 740 cm^{-1}; m/z (HRMS$^+$) $[M + Na]^+$ 783.2700 ($C_{45}H_{44}O_9SNa^+$ requires 783.2598).

^{1}H NMR of 22 (400 MHz, $CDCl_3$)

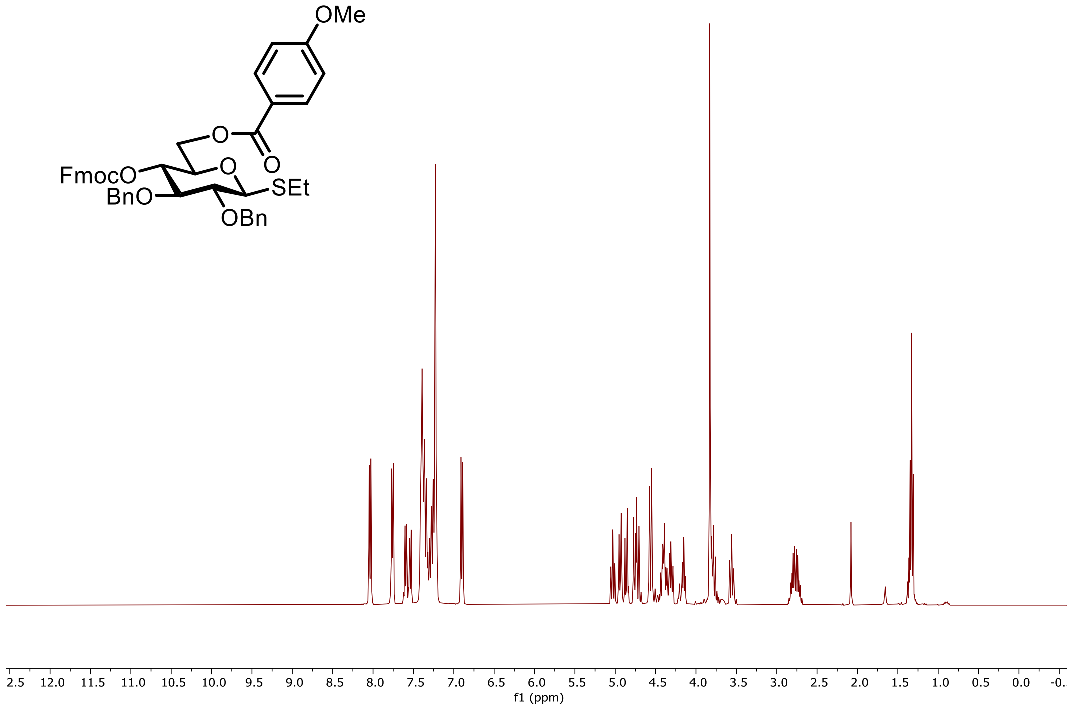

^{13}C NMR of 22 (101 MHz, $CDCl_3$)

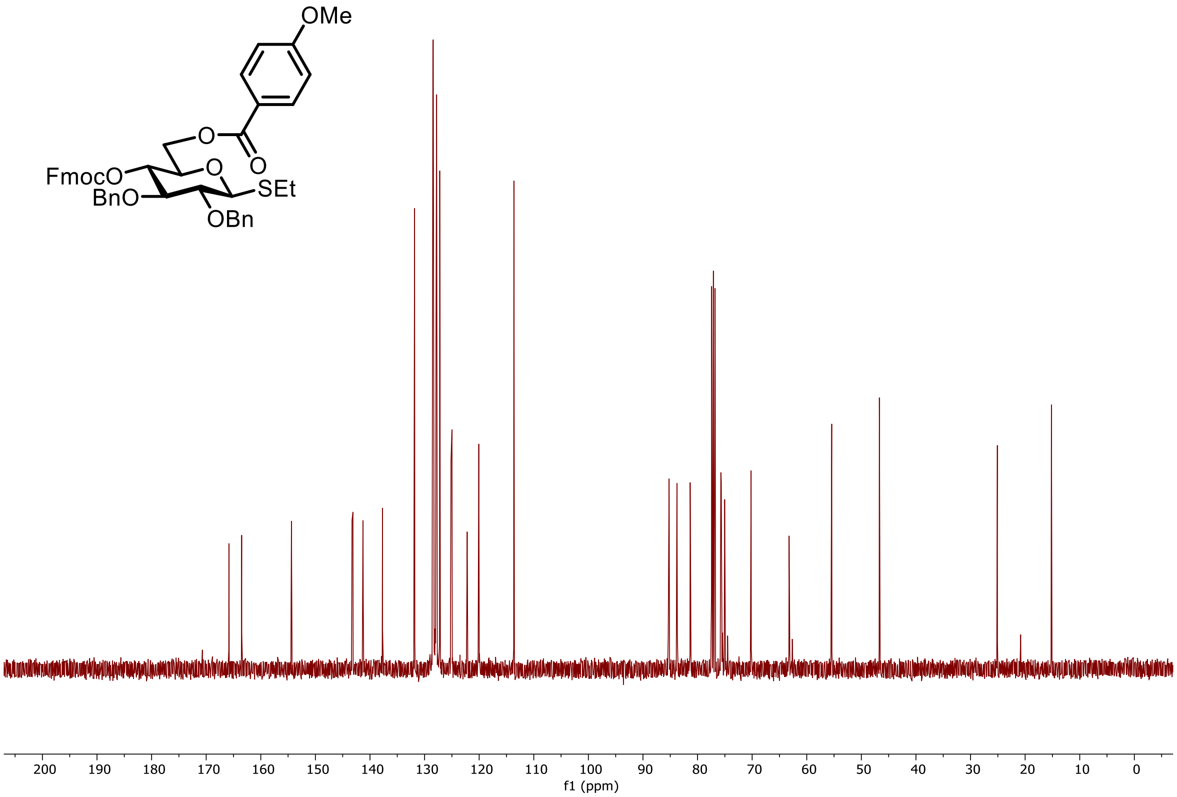

COSY NMR of 22 ($CDCl_3$)

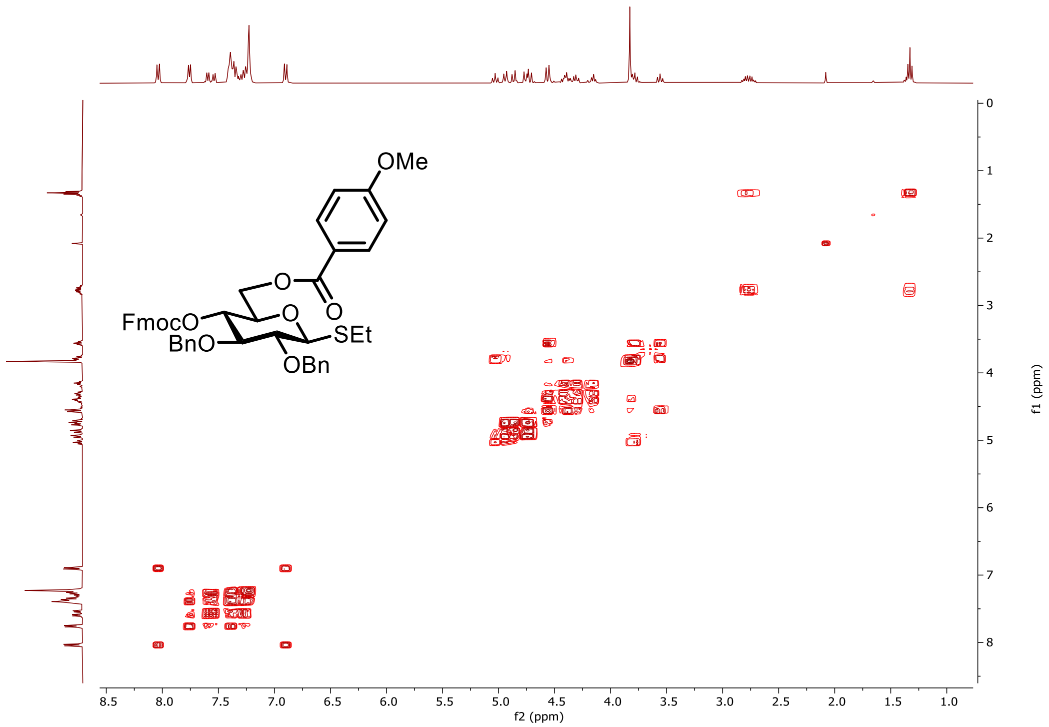

HSQC NMR of 22 ($CDCl_3$)

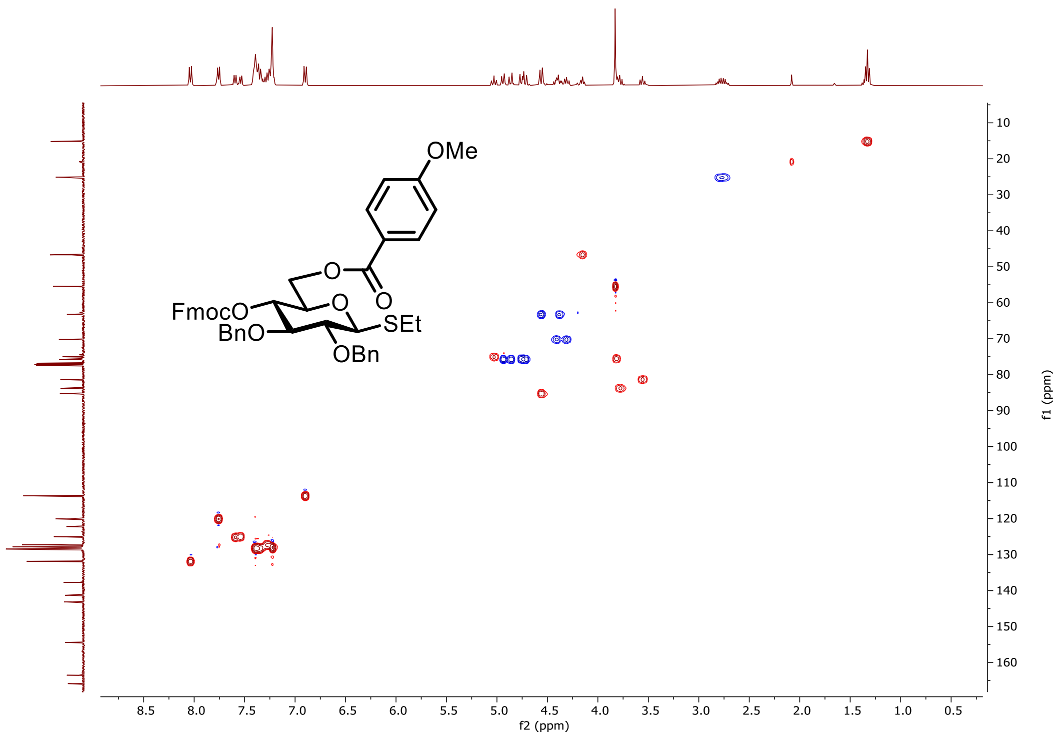

2.12 Synthesis of 23

Ethyl 2,3-di-*O*-benzyl-4-*O*-(9-fluorenylmethoxycarbonyl)-6-*O*-(4-nitrobenzoyl)-1-thio-β-D-glucopyranoside, 23

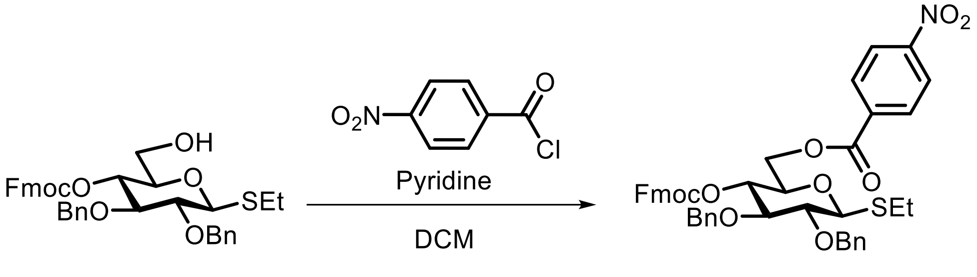

58 **23**

4-Nitrobenzoic acid (1.07 g, 6.4 mmol) was dissolved in anhydrous DCM (10 mL), the obtained solution was protected with argon and cooled with an ice bath for 30 min. After oxalyl chloride (720 µL, 8.4 mmol) was added, two drops of DMF were added to start the reaction. The reaction was allowed to warm to room temperature and kept stirring for 2 h. The solvent and remaining oxalyl chloride were removed under high vacuum, the crude product was coevaporated with toluene (5 mL × 3). The obtained 4-nitrobenzoyl chloride was dissolved in anhydrous DCM (10 mL) and added into an ice bath-precooled solution made of ethyl 2,3-di-*O*-benzyl-4-*O*-(9-fluorenylmethoxycarbonyl)-1-thio-β-D-glucopyranoside **58** (1.01 g, 1.6 mmol), anhydrous DCM (30 mL) and pyridine (10 mL). After 30 min, the reaction was warmed up and stirred at room temperature for 24 h. Its completion was confirmed by TLC and MeOH (5 mL) was added to quench the reaction. DCM (30 mL) was added 90 min later, the organic phase was washed with aqueous citric acid (0.5 M, 30 mL). After extracting the water phase with DCM (30 mL), the organic layers were combined and dried over Na_2SO_4, filtered, and evaporated. The resulting crude product was purified by column chromatography (Hexane : EtOAc = 8:1→Hexane : EtOAc : DCM =6:1:1) to give **23** as a white solid (1.07 g, 86%). ^{1}H NMR (400 MHz, $CDCl_3$) δ 8.22 (q, *J* = 9.0 Hz, 4H), 7.75 (d, *J* = 7.6 Hz, 2H), 7.55 (d, *J* = 7.6 Hz, 1H), 7.52 (d, *J* = 7.6 Hz, 1H), 7.42 – 7.29 (m, 6H), 7.29 – 7.17 (m, 8H), 5.03 (appt, *J* = 9.7 Hz, 1H), 4.92 (d, *J* = 10.2 Hz, 1H), 4.85 (d, *J* = 11.1 Hz, 1H), 4.74 (d, *J* = 10.2 Hz, 1H), 4.69 (d, *J* = 11.2 Hz, 1H), 4.63 – 4.51 (m, 2H), 4.47 – 4.36 (m, 2H), 4.32 (dd, *J* = 10.5, 7.0 Hz, 1H), 4.14 (appt, *J* = 7.1 Hz, 1H), 3.84 – 3.72 (m, 2H), 3.54 (appt, *J* = 9.3 Hz, 1H), 2.75 (dtt, *J* = 20.1, 12.7, 7.4 Hz, 2H), 1.31 (t, *J* = 7.4 Hz, 3H); ^{13}C NMR (101 MHz, $CDCl_3$) δ 164.29, 154.38, 150.58, 143.06, 143.03, 141.26, 141.25, 137.72, 137.56, 135.11, 130.87, 128.48, 128.40, 128.37, 128.07, 127.96, 127.82, 127.78, 127.19, 125.00, 124.90, 123.56, 120.13, 120.10, 85.42, 83.61, 81.22, 75.76, 75.70, 75.26, 74.42, 70.21, 63.84, 46.66, 25.15, 15.13; $[\alpha]_D^{25}$ 9.36 (c = 1, $CHCl_3$); IR (neat) ν_{max} = 1753, 1731, 1528, 1256 cm^{-1}; m/z (HRMS$^+$) [M + Na]$^+$ 798.2431 ($C_{44}H_{41}NO_{10}SNa^+$ requires 798.2343).

^{1}H NMR of 23 (400 MHz, $CDCl_3$)

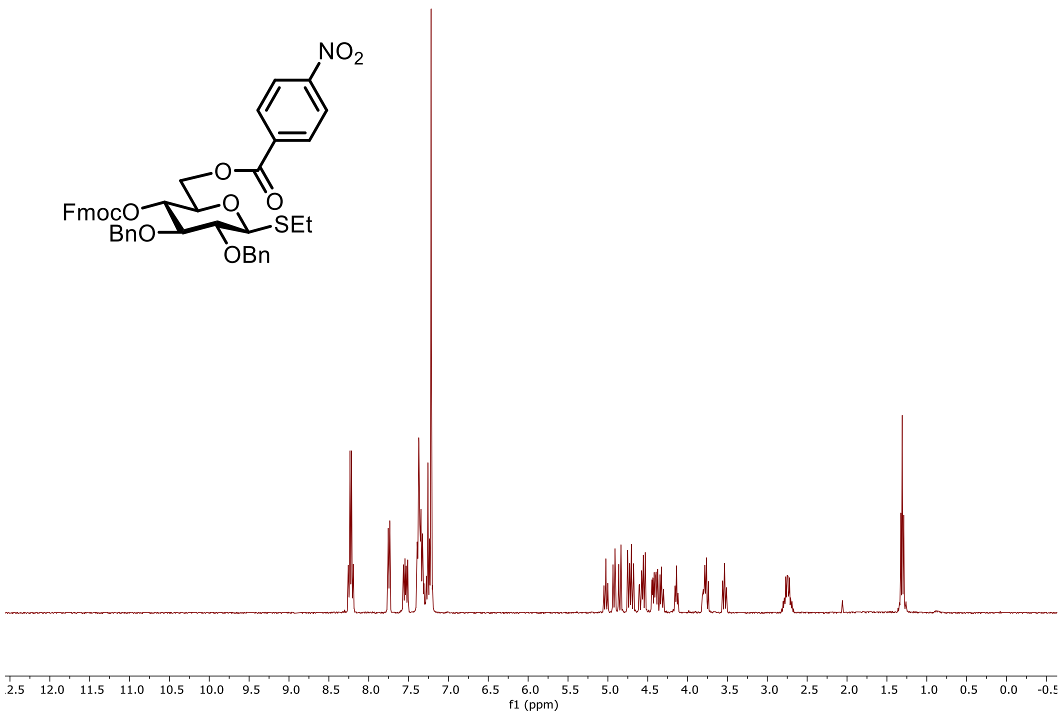

^{13}C NMR of 23 (101 MHz, $CDCl_3$)

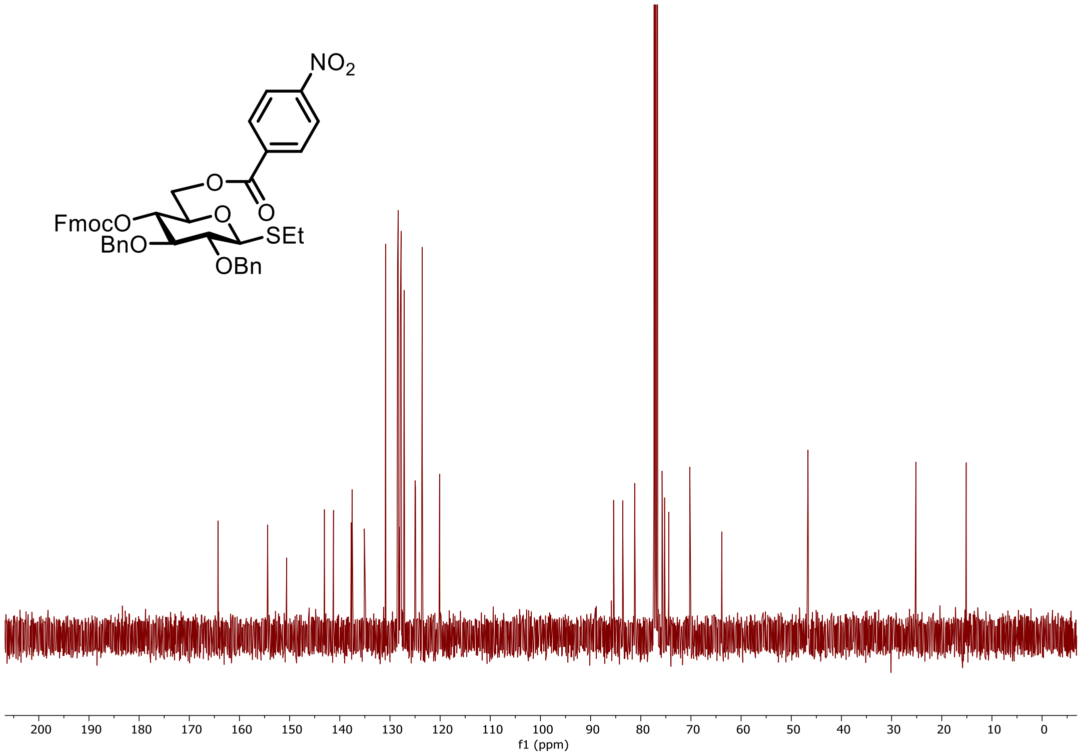

COSY NMR of 23 ($CDCl_3$)

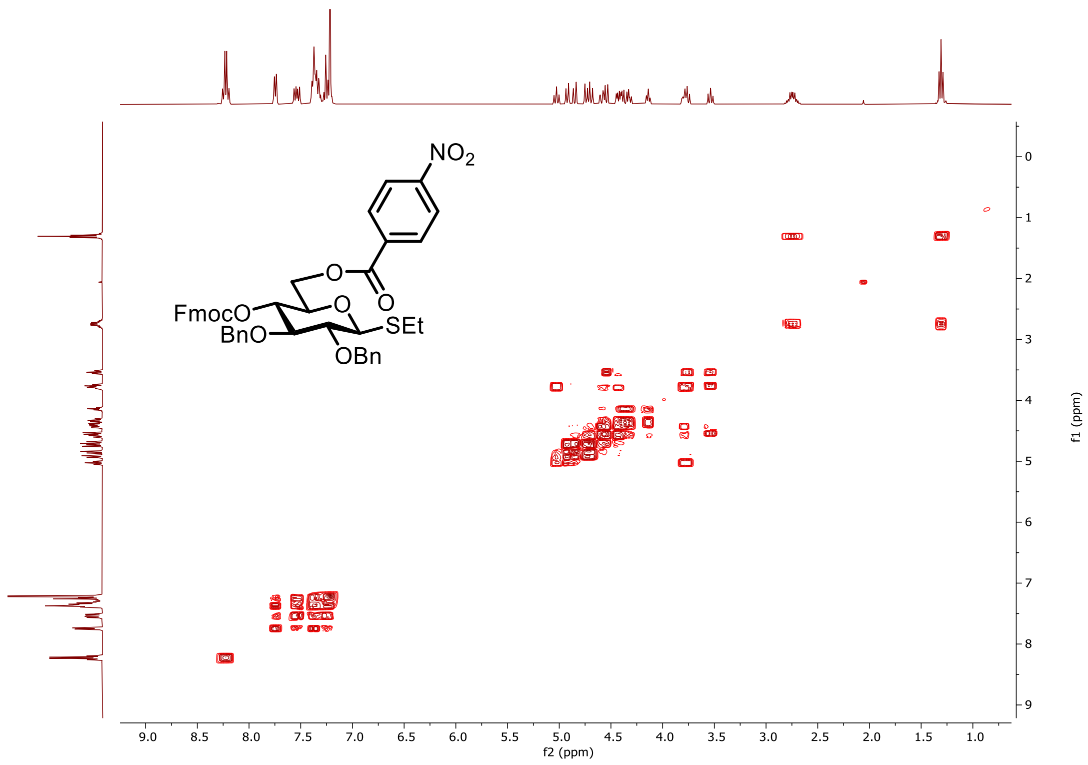

HSQC NMR of 23 ($CDCl_3$)

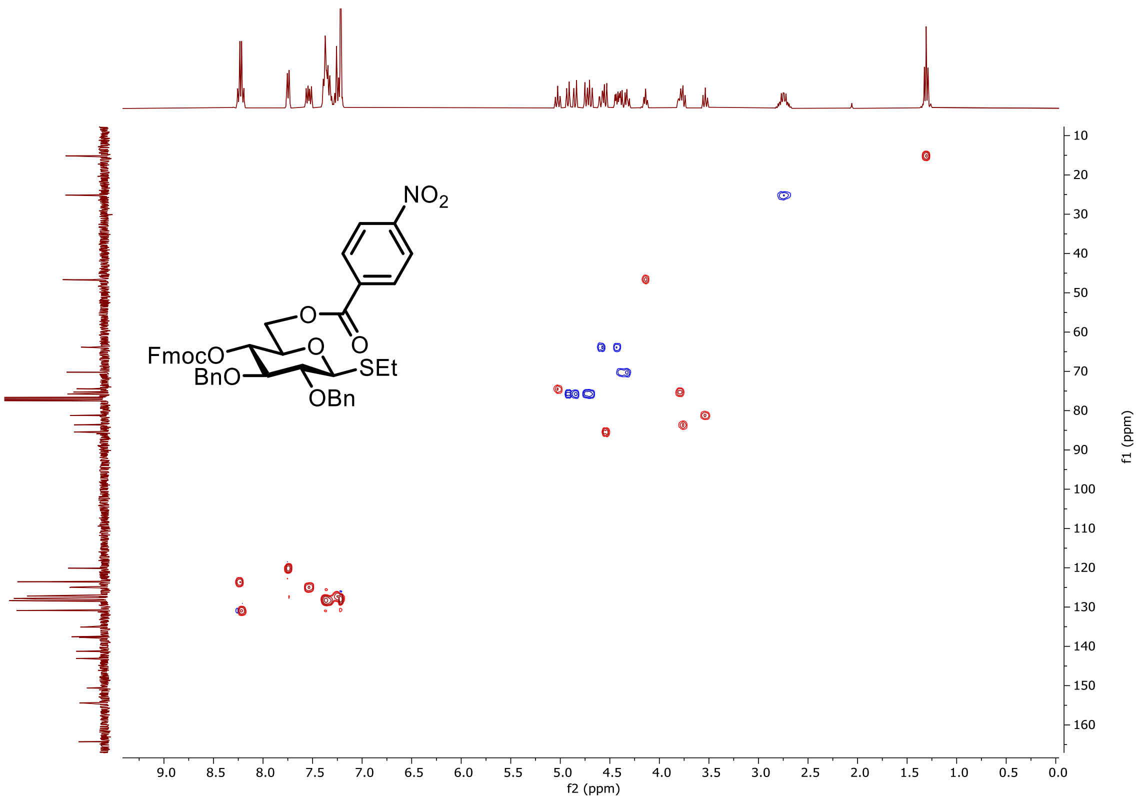

Dibutylphosphoryl 2,3-di-*O*-benzyl-4-*O*-(9-fluorenylmethoxycarbonyl)-6-*O*-(4-nitrobenzoyl)-α-D-glucopyranoside, 62a

&

Dibutylphosphoryl 2,3-di-*O*-benzyl-4-*O*-(9-fluorenylmethoxycarbonyl)-6-*O*-(4-nitrobenzoyl)-β-D-glucopyranoside, 62b

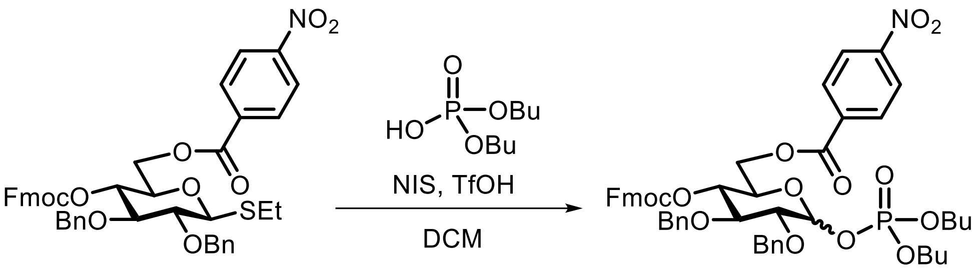

23 **62a + 62b**

Ethyl 2,3-di-*O*-benzyl-4-*O*-(9-fluorenylmethoxycarbonyl)-6-*O*-(4-nitrobenzoyl)-1-thio-β-D-glucopyranoside **23** (351 mg, 0.45 mmol) was dissolved in anhydrous DCM (5 mL) and 4Å molecular sieve (500 mg) was added. After dibutyl phosphate (200 µL, 1.0 mmol) and *N*-iodosuccinimide (NIS, 126 mg, 0.56 mmol) were added, the reaction mixture was stirred at room temperature for 30 min and cooled to -15 °C under argon protection. Trifluoromethanesulfonic acid (TfOH, 8.1 µL, 92 µmol) was slowly added and the reaction was kept for 10 min at -15 °C, then warmed to 0 °C. 60 min later, saturated aq. $NaHCO_3$ solution (10 mL) was added to quench the reaction. DCM (20 mL) and saturated aq. $Na_2S_2O_3$ solution (10 mL) were added. After extraction, the organic layer was dried over Na_2SO_4, filtered, and evaporated. The resulting crude product was purified by column chromatography (Hexane : EtOAc = 4:1→2:1) to give the mixture of **62a** and **62b** as clear oil (386 mg, 93%). **62a**: ^{1}H NMR (400 MHz, $CDCl_3$) δ 8.29 – 8.21 (m, 4H), 7.79 (d, *J* = 7.6 Hz, 2H), 7.61 (d, *J* = 7.5 Hz, 1H), 7.58 (d, *J* = 7.6 Hz, 1H), 7.46 – 7.20 (m, 14H), 5.90 (dd, *J* = 7.1, 3.2 Hz, 1H), 5.08 (appt, *J* = 9.9 Hz, 1H), 4.93 (d, *J* = 11.2 Hz, 1H), 4.83 (d, *J* = 11.3 Hz, 1H), 4.75 (d, *J* = 5.0 Hz, 1H), 4.72 (d, *J* = 4.9 Hz, 1H), 4.60 (dd, *J* = 12.5, 2.4 Hz, 1H), 4.48 – 4.33 (m, 4H), 4.20 (appt, *J* = 7.1 Hz, 1H), 4.14 – 4.02 (m, 5H), 3.73 (dt, *J* = 9.6, 3.1 Hz, 1H), 1.64 (ddd, *J* = 14.7, 12.2, 6.8 Hz, 4H), 1.40 (ddt, *J* = 19.6, 15.0, 7.4 Hz, 4H), 0.94 (dt, *J* = 11.8, 7.4 Hz, 6H); ^{13}C NMR (101 MHz, $CDCl_3$) δ 164.30, 154.41, 150.61, 143.11, 143.06, 141.32, 137.80, 137.33, 135.06, 130.97, 128.52, 128.37, 128.08, 128.02, 127.94, 127.87, 127.24, 125.08, 124.98, 123.61, 120.20, 120.16, 94.68 (d, *J* = 6.1 Hz), 78.92 (d, *J* = 7.3 Hz), 78.15, 75.70, 73.55, 73.19, 70.24, 69.09, 68.04 (d, *J* = 5.9 Hz), 67.78 (d, *J* = 5.9 Hz), 63.07, 46.70, 32.27 (d, *J* = 7.0 Hz), 32.16 (d, *J* = 7.0 Hz), 18.68, 18.62, 13.65; $[\alpha]_D^{25}$ 43.50 (c = 1, $CHCl_3$); IR (neat) ν_{max} = 1754, 1732, 1276, 1255, 955, 739 cm^{-1}; m/z (HRMS$^+$) [M + Na]$^+$ 946.3331 ($C_{50}H_{54}NO_{14}PNa^+$ requires 946.3174). **62b**: ^{1}H NMR (400 MHz, $CDCl_3$) δ 8.31 – 8.23 (m, 4H), 7.78 (d, *J* = 7.5 Hz, 2H), 7.59 (d, *J* = 7.4 Hz, 1H), 7.56 (d, *J* = 7.4 Hz, 1H), 7.45 – 7.16 (m, 14H), 5.30 (appt, *J* = 7.4 Hz, 1H), 5.07 (appt, *J* = 9.7 Hz, 1H), 4.91 (d, *J* = 11.1 Hz, 1H), 4.86 – 4.77 (m, 2H), 4.70 – 4.63 (m, 2H), 4.48 – 4.31 (m, 3H), 4.18 (appt, *J* = 6.9 Hz, 1H), 4.13 – 3.98 (m, 4H), 3.95 (ddt, *J* = 10.3, 4.6, 2.6 Hz, 1H), 3.79 (appt, *J* = 9.2 Hz, 1H), 3.64 (appt, *J* = 8.5 Hz, 1H), 1.60 (p, *J* = 6.8

Hz, 4H), 1.33 (ddd, *J* = 15.1, 7.5, 2.9 Hz, 4H), 0.87 (q, *J* = 7.5 Hz, 6H); ^{13}C NMR (101 MHz, $CDCl_3$) δ 164.19, 154.36, 150.64, 143.04, 143.01, 141.30, 137.62, 137.54, 134.97, 130.98, 128.45, 128.38, 128.02, 127.93, 127.89, 127.77, 127.23, 125.04, 124.93, 123.64, 120.19, 120.15, 98.53 (d, *J* = 6.1 Hz), 81.57 (d, *J* = 9.1 Hz), 81.34, 75.75, 75.06, 73.74, 72.00, 70.31, 67.95 (d, *J* = 5.9 Hz), 67.82 (d, *J* = 5.9 Hz), 63.09, 46.67, 32.16 (d, *J* = 7.4 Hz), 32.06 (d, *J* = 7.4 Hz), 18.58, 18.56, 13.58, 13.55; $[\alpha]_D^{25}$ 34.78 (c = 1, $CHCl_3$); IR (neat) ν_{max} = 1754, 1732, 1258, 1028, 739 cm^{-1}; m/z ($HRMS^+$) $[M + Na]^+$ 946.3331 ($C_{50}H_{54}NO_{14}PNa^+$ requires 946.3174).

^{1}H NMR of 62a (400 MHz, $CDCl_3$)

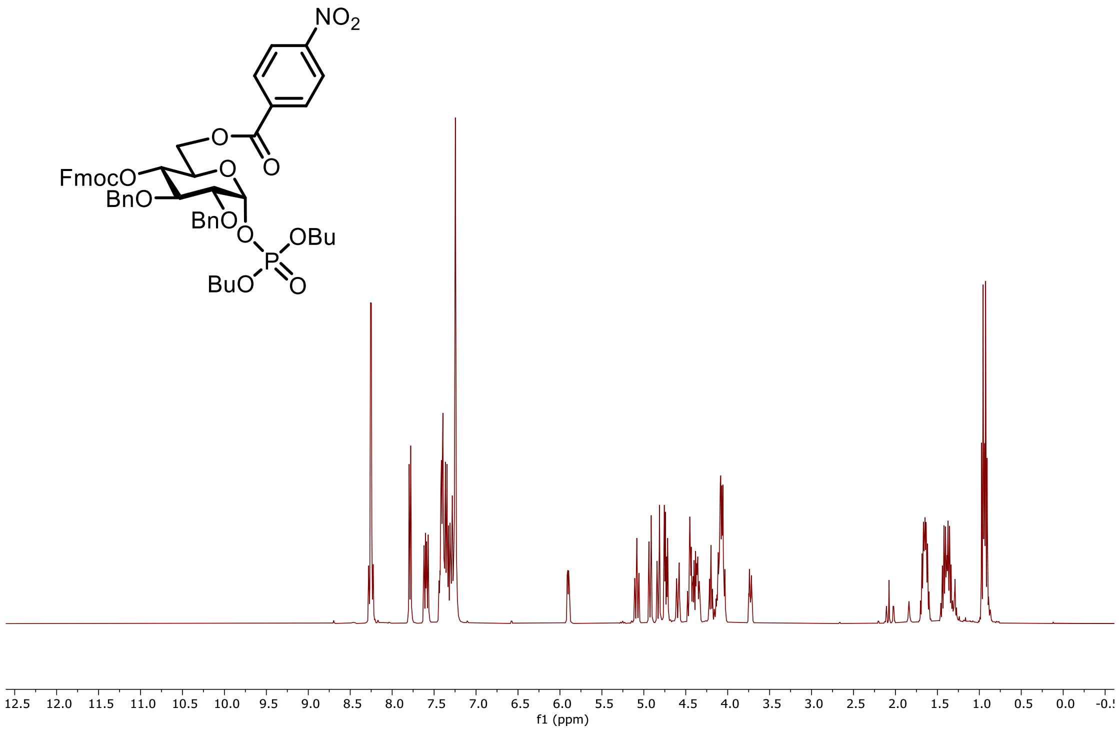

^{13}C NMR of 62a (101 MHz, $CDCl_3$)

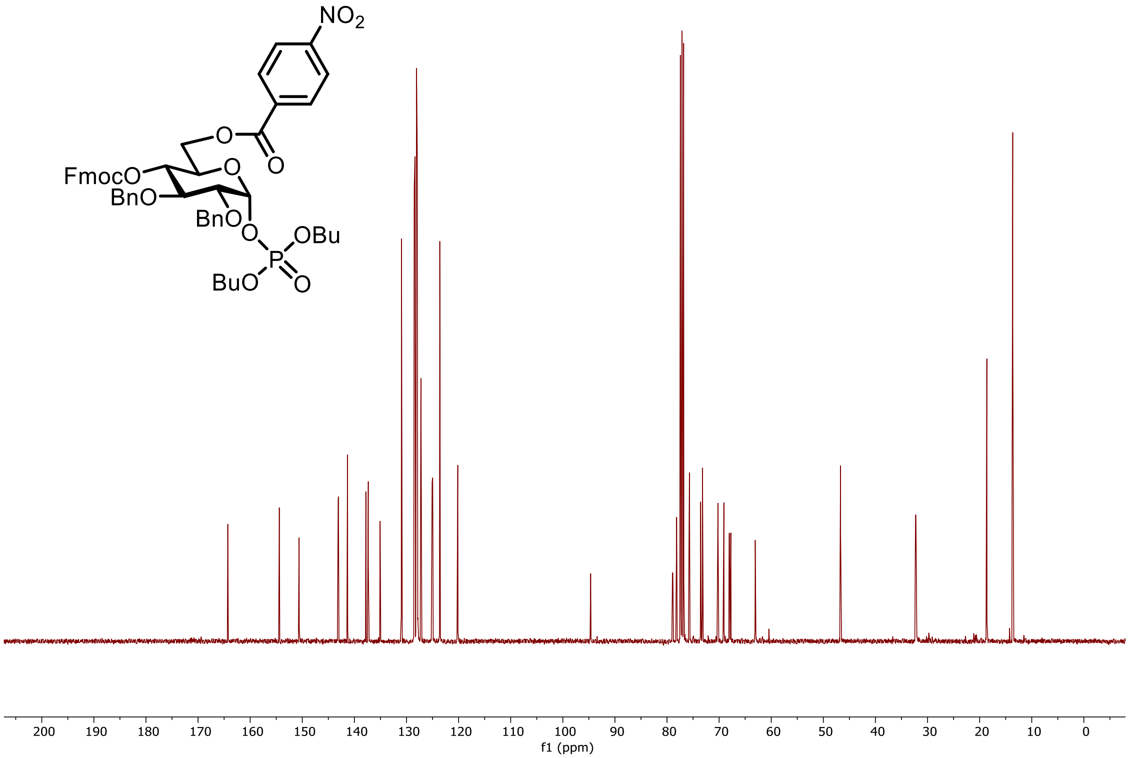

COSY NMR of 62a ($CDCl_3$)

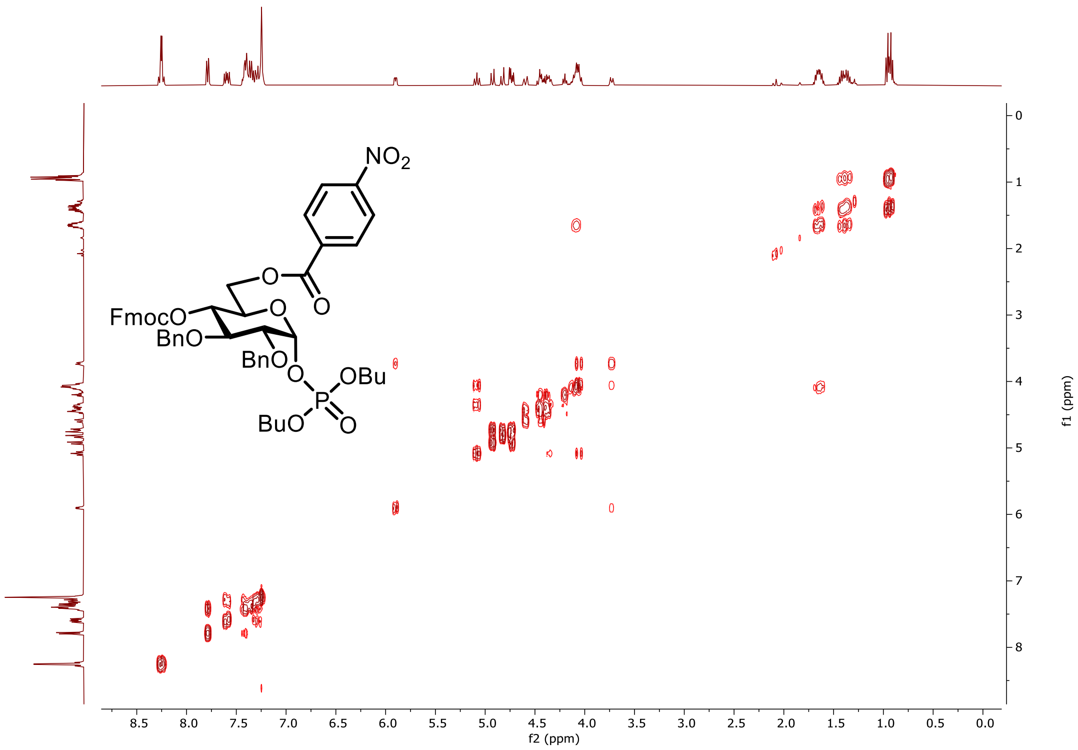

HSQC NMR of 62a ($CDCl_3$)

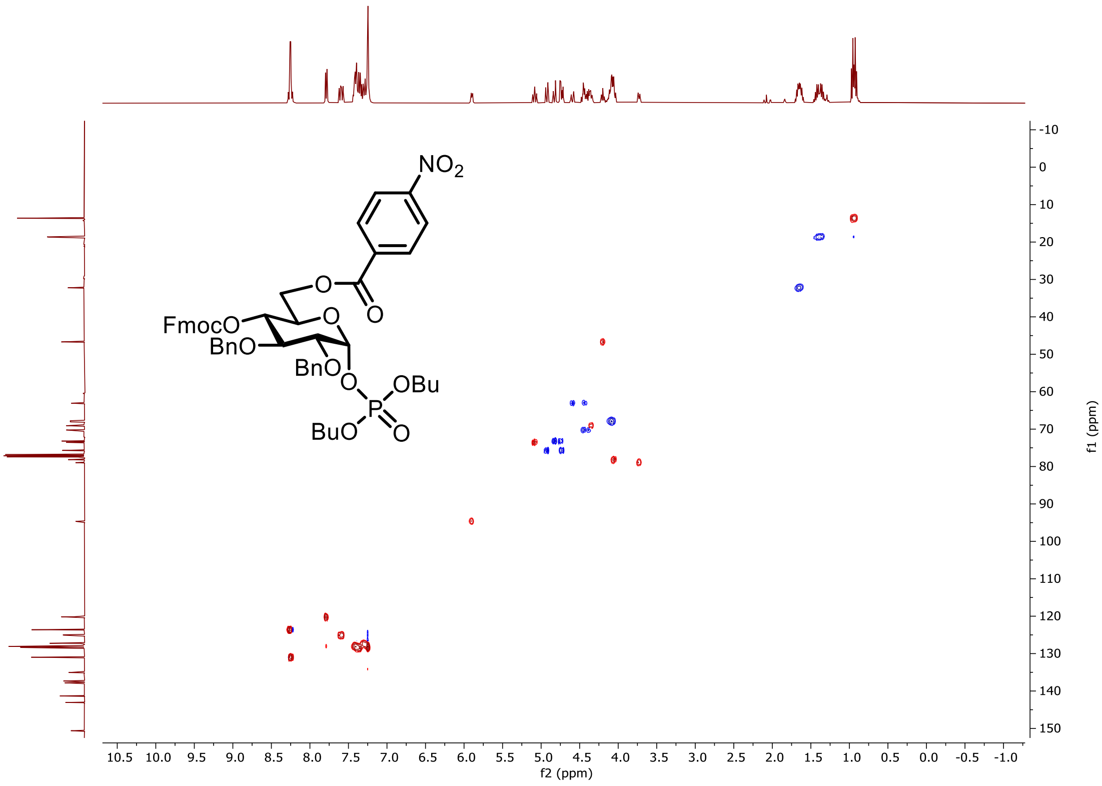

^{1}H NMR of 62b (400 MHz, $CDCl_3$)

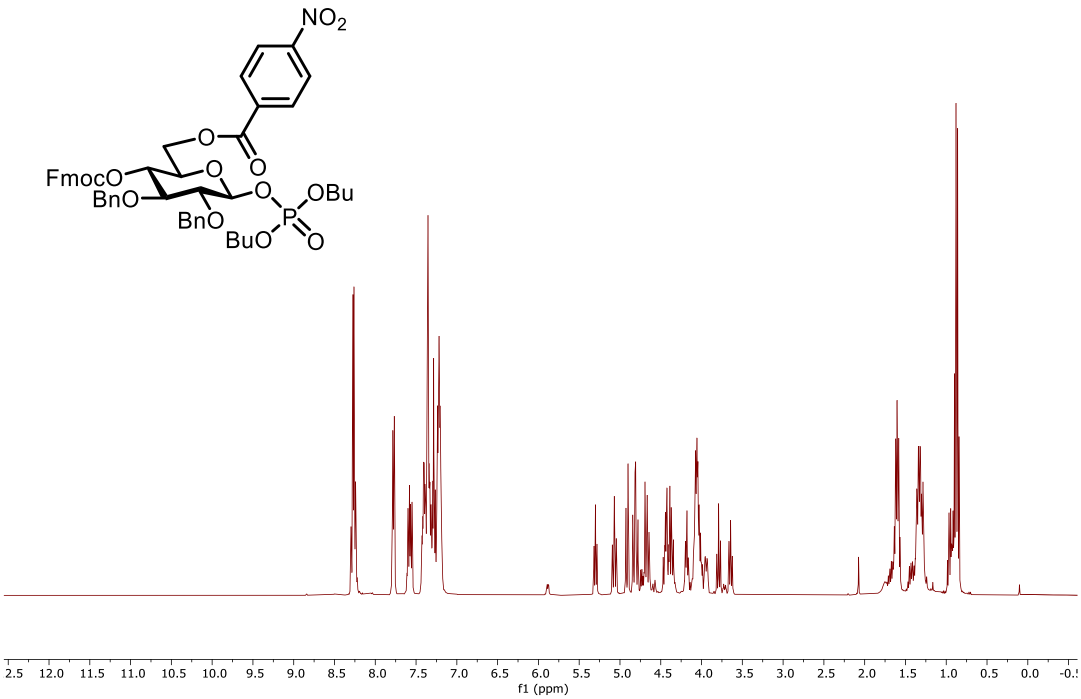

^{13}C NMR of 62b (101 MHz, $CDCl_3$)

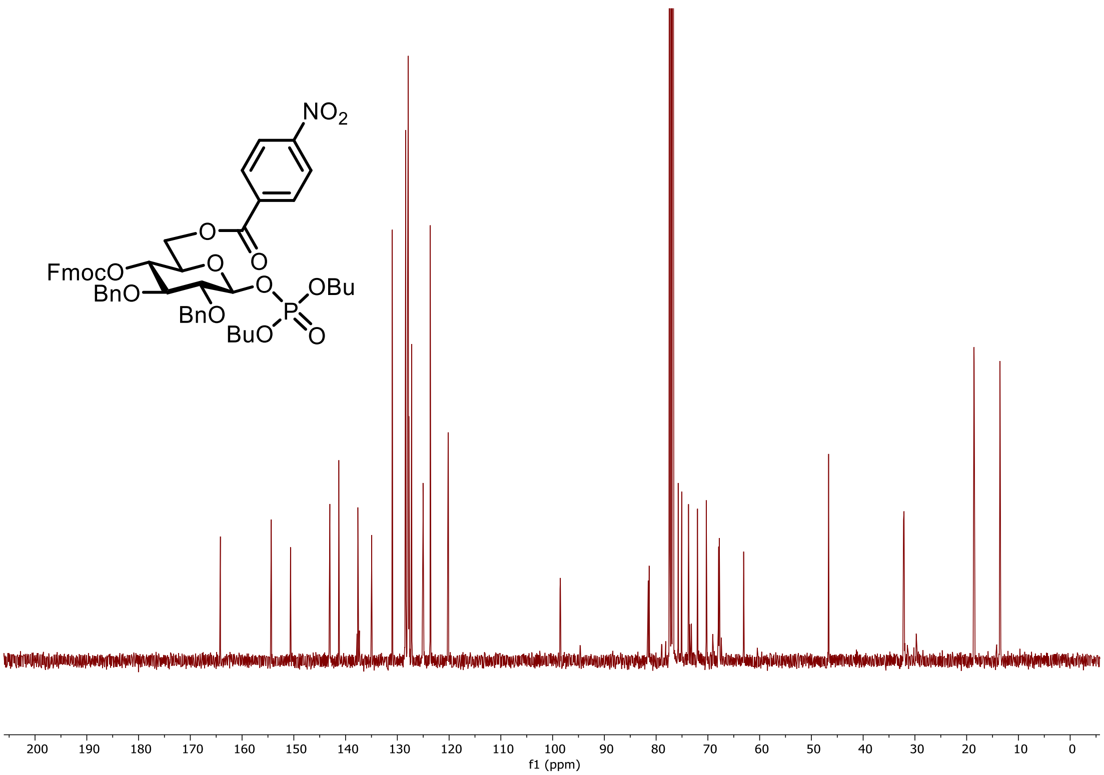

COSY NMR of 62b ($CDCl_3$)

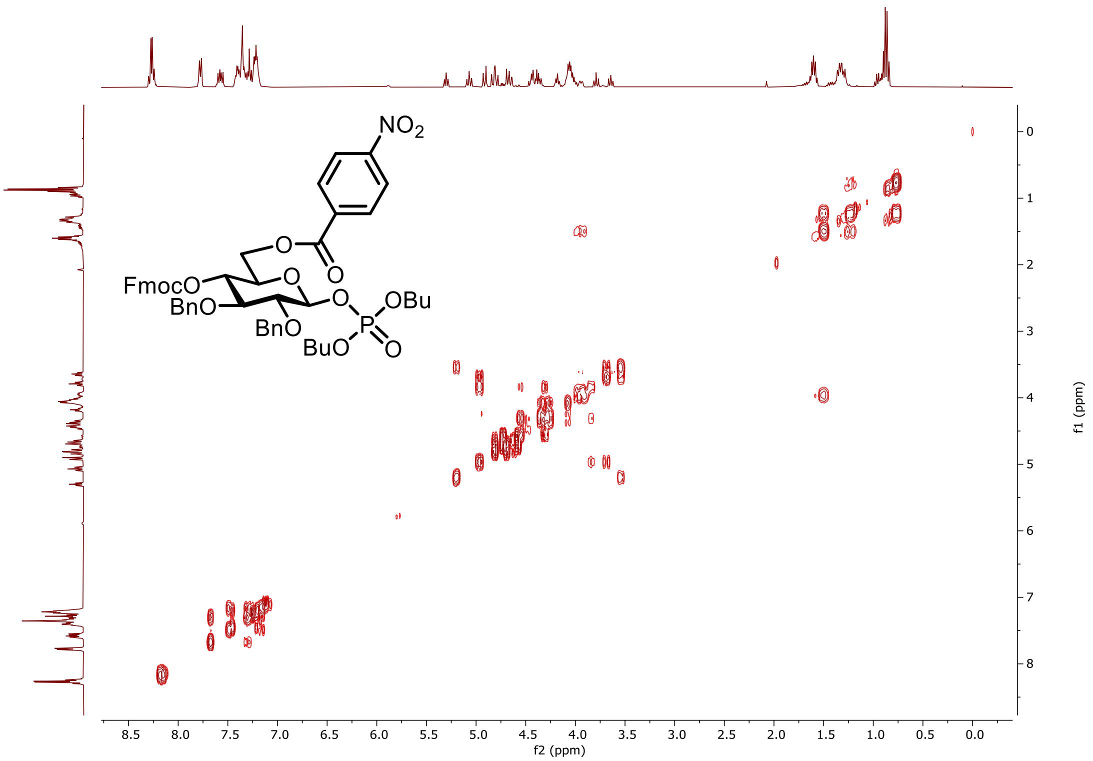

HSQC NMR of 62b ($CDCl_3$)

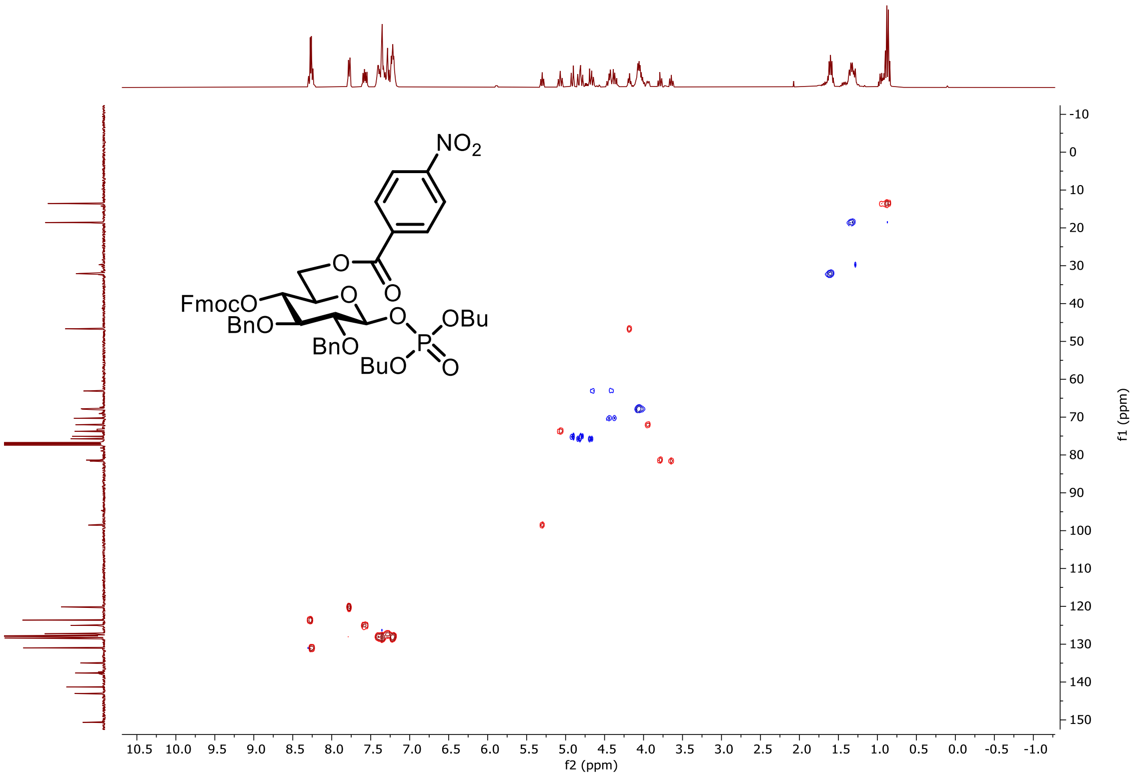

2.13 Synthesis of 24

Ethyl 2,3-di-*O*-benzyl-4-*O*-(9-fluorenylmethoxycarbonyl)-6-*O*-carboxybenzyl-1-thio-β-D-glucopyranoside, 24

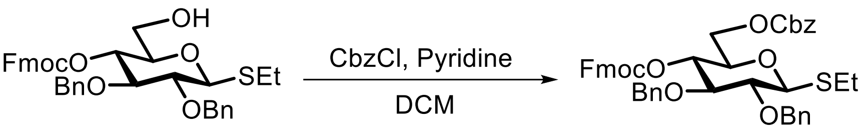

58 **24**

Ethyl 2,3-di-*O*-benzyl-4-*O*-(9-fluorenylmethoxycarbonyl)-1-thio-β-D-glucopyranoside **58** (1.10 g, 1.7 mmol) was dissolved in anhydrous DCM (20 mL) and pyridine (4 mL) was added. The solution was cooled with an ice bath for 30 min, and benzyl chloroformate (CbzCl, 626 mg, 3.7 mmol) was added slowly. The reaction was warmed to room temperature and stirred for 16 h. Completion of the reaction was confirmed by TLC, MeOH (2 mL) was added. DCM (20 mL) was added 30 min later, and the organic phase was washed with aqueous citric acid (0.5 M, 20 mL). After extracting the water phase with DCM (10 mL), the organic layers were combined and dried over Na_2SO_4, filtered, and evaporated. The resulting crude product was purified by column chromatography (Hexane : EtOAc = 8:1→4:1) to give **24** as a white solid (760 mg, 59%). ^{1}H NMR (400 MHz, $CDCl_3$) δ 7.76 (d, *J* = 7.6 Hz, 2H), 7.59 (d, *J* = 7.6 Hz, 1H), 7.54 (d, *J* = 7.5 Hz, 1H), 7.43 – 7.18 (m, 19H), 5.16 (d, *J* = 2.6 Hz, 2H), 4.95 – 4.81 (m, 3H), 4.71 (dd, *J* = 12.8, 10.7 Hz, 2H), 4.53 – 4.42 (m, 2H), 4.38 – 4.28 (m, 2H), 4.25 (dd, *J* = 11.8, 2.8 Hz, 1H), 4.18 (appt, *J* = 7.1 Hz, 1H), 3.78 – 3.66 (m, 2H), 3.50 (appt, *J* = 9.3 Hz, 1H), 2.75 (dtt, *J* = 20.2, 12.7, 7.4 Hz, 2H), 1.32 (t, *J* = 7.4 Hz, 3H); ^{13}C NMR (101 MHz, $CDCl_3$) δ 154.85, 154.39, 143.27, 143.14, 141.29, 141.27, 137.89, 137.66, 135.07, 128.58, 128.56, 128.45, 128.41, 128.35, 128.32, 128.01, 127.92, 127.71, 127.68, 127.21, 125.16, 125.00, 120.07, 120.05, 85.19, 83.58, 81.13, 75.63, 75.61, 75.38, 74.76, 70.25, 69.85, 66.51, 46.71, 25.08, 15.11; $[\alpha]_D^{25}$ -1.49 (c = 1, $CHCl_3$); IR (neat) ν_{max} = 1750, 1243, 738 cm^{-1}; m/z (HRMS$^+$) $[M + Na]^+$ 783.2689 ($C_{45}H_{44}O_9SNa^+$ requires 783.2598).

^{1}H NMR of 24 (400 MHz, $CDCl_3$)

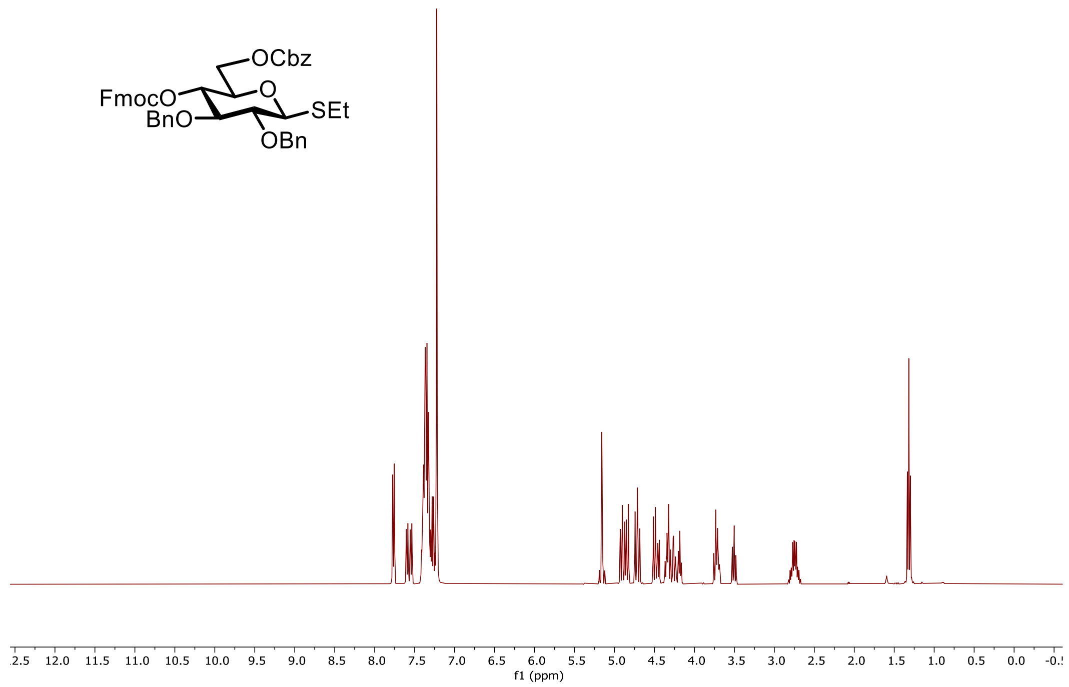

^{13}C NMR of 24 (101 MHz, $CDCl_3$)

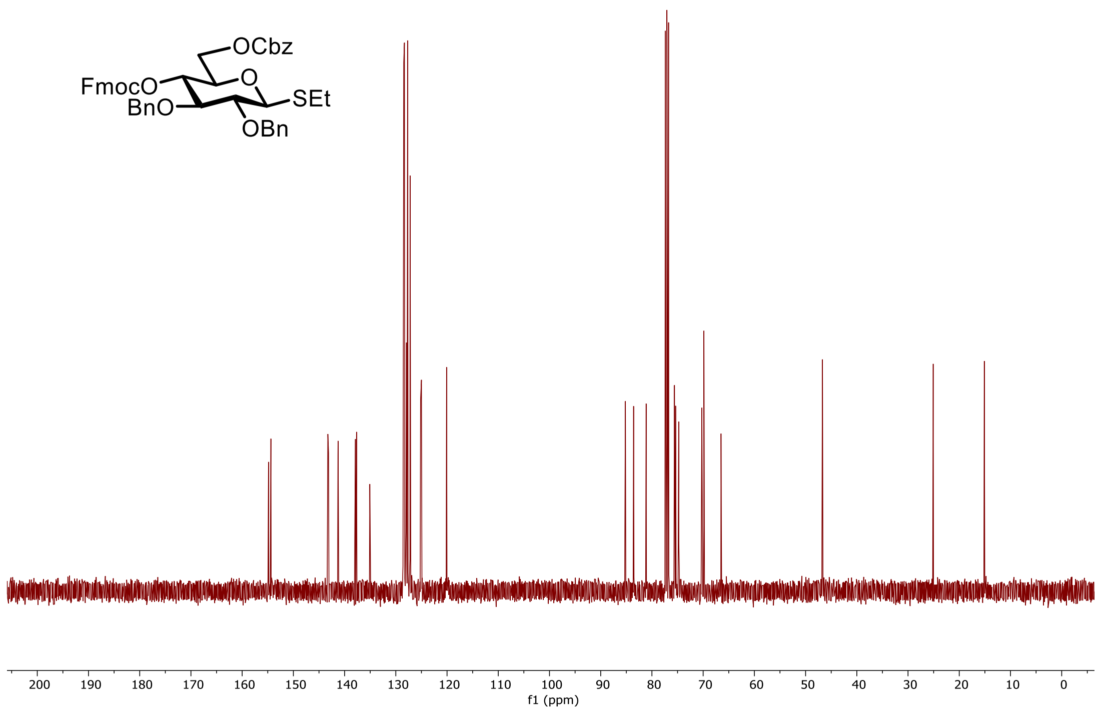

COSY NMR of 24 ($CDCl_3$)

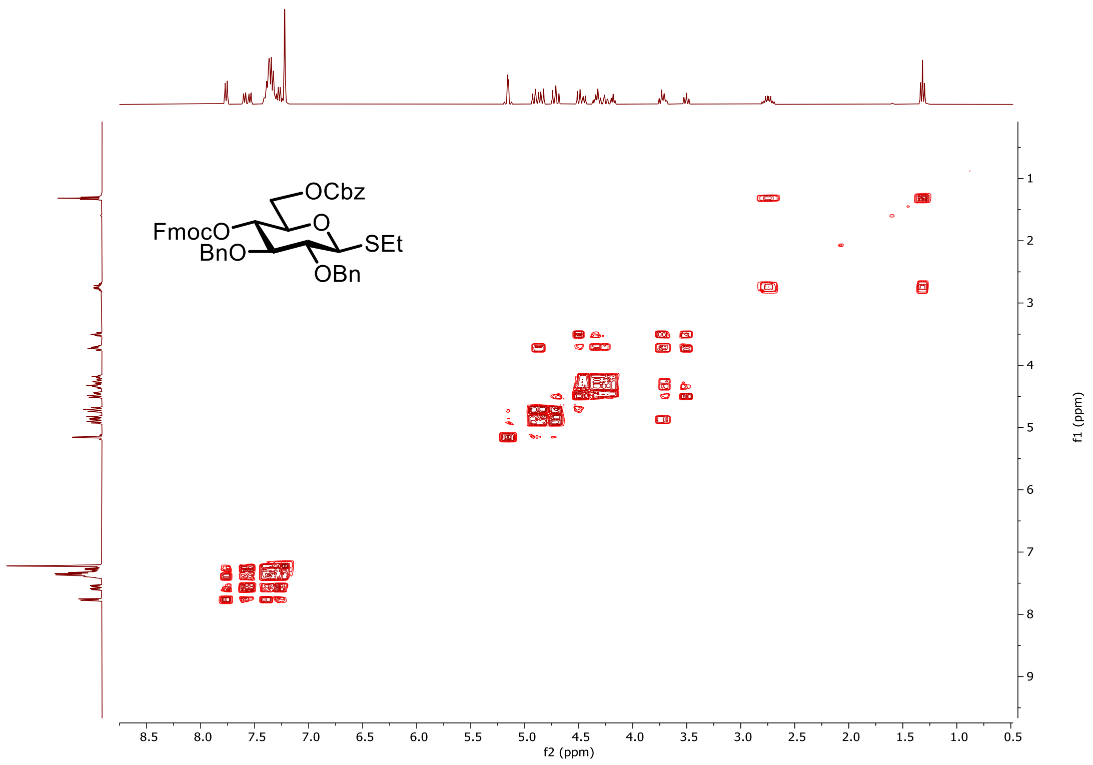

HSQC NMR of 24 ($CDCl_3$)

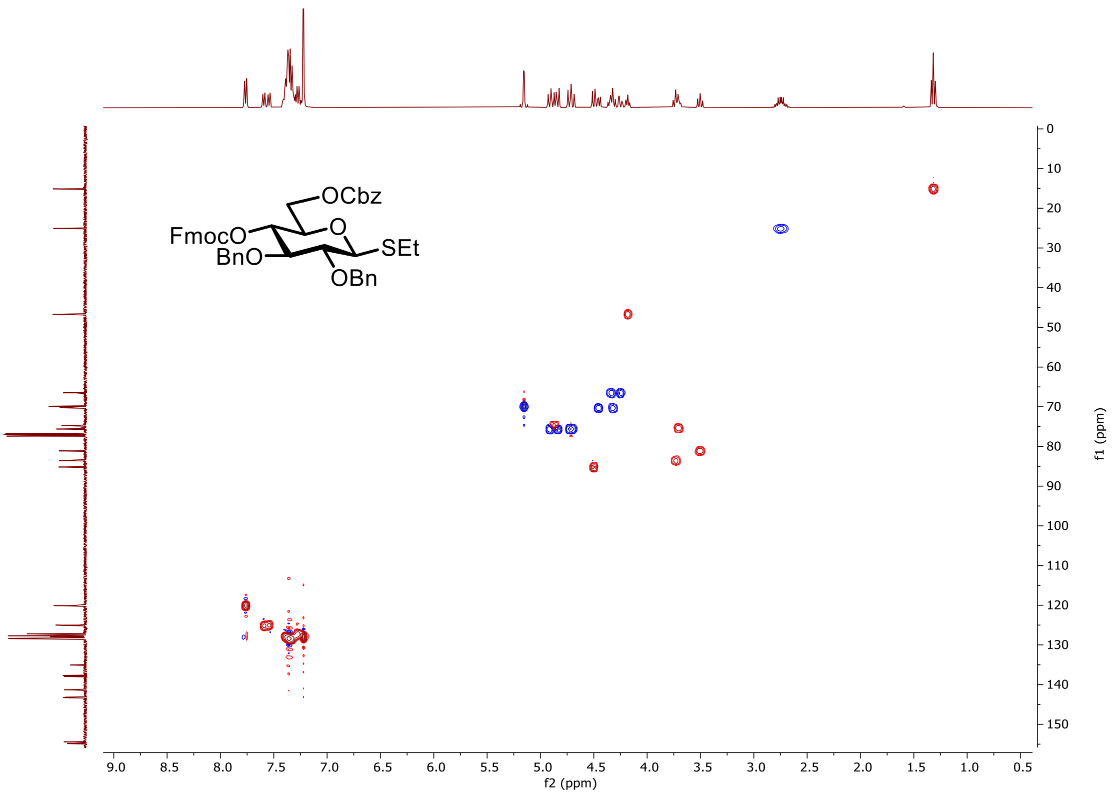

2.14 Synthesis of 25

Ethyl 2,3-di-*O*-benzyl-4-*O*-(9-fluorenylmethoxycarbonyl)-6-*O*-triphenylacetyl-1-thio-β-D-glucopyranoside, 25

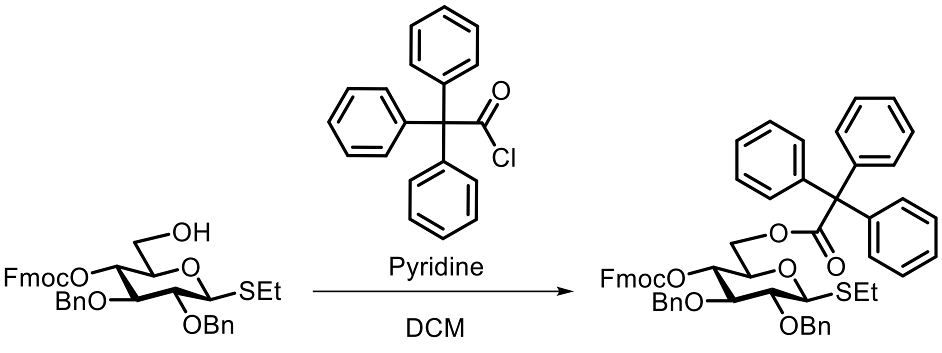

58 **25**

Triphenylacetic acid (1.85 g, 6.4 mmol) was dissolved in anhydrous DCM (10 mL), the obtained mixture was protected with argon and cooled with an ice bath for 30 min. After oxalyl chloride (720 µL, 8.4 mmol) was added, two drops of DMF were added to start the reaction. The reaction was allowed to warm to room temperature and kept stirring for 8 h. The solvent and remaining oxalyl chloride were removed under high vacuum, the crude product was coevaporated with toluene (10 mL × 3). The obtained triphenylacetyl chloride was dissolved in anhydrous DCM (10 mL) and slowly added into an ice bath-precooled solution made of ethyl 2,3-di-*O*-benzyl-4-*O*-(9-fluorenylmethoxycarbonyl)-1-thio-β-D-glucopyranoside **58** (1.01 g, 1.6 mmol), anhydrous DCM (40 mL) and pyridine (10 mL). After 30 min, the reaction was warmed up and stirred at room temperature for 24 h. On the next day, the reaction was cooled with an ice bath again, additional pyridine (10 mL) was added first, and then triphenylacetyl chloride was prepared from triphenylacetic acid (1.85 g, 6.4 mmol), added into the solution. After warmed to room temperature, the reaction was kept going for another 24 h. MeOH (10 mL) was added to quench the reaction and DCM (50 mL) was added 2 h later. The organic phase was washed with aqueous citric acid (0.5 M, 50 mL). After extracting the water phase with DCM (50 mL), the organic layers were combined and dried over Na_2SO_4, filtered, and evaporated. The resulting crude product was purified by column chromatography (Hexane : EtOAc = 10:1→Hexane : EtOAc : DCM =8:1:1) to give **25** as a white solid (1.12 g, 78%). ^{1}H NMR (400 MHz, $CDCl_3$) δ 7.78 (d, *J* = 7.6 Hz, 2H), 7.58 (dd, *J* = 10.1, 7.5 Hz, 2H), 7.45 – 7.17 (m, 29H), 4.90 (d, *J* = 10.2 Hz, 1H), 4.81 (d, *J* = 11.2 Hz, 1H), 4.76 – 4.63 (m, 3H), 4.55 – 4.42 (m, 2H), 4.39 (d, *J* = 9.7 Hz, 1H), 4.33 (dd, *J* = 10.5, 7.1 Hz, 1H), 4.26 – 4.14 (m, 2H), 3.67 (appt, *J* = 9.0 Hz, 1H), 3.58 (ddd, *J* = 9.6, 6.9, 2.1 Hz, 1H), 3.39 (appt, *J* = 9.3 Hz, 1H), 2.61 – 2.42 (m, 2H), 1.19 (t, *J* = 7.4 Hz, 3H); ^{13}C NMR (101 MHz, $CDCl_3$) δ 173.30, 154.30, 143.20, 142.73, 141.30, 137.87, 137.67, 130.43, 128.49, 128.45, 128.35, 128.05, 127.96, 127.76, 127.28, 126.97, 125.16, 125.02, 120.12, 84.59, 83.52, 80.98, 75.59, 75.51, 74.52, 70.22, 67.56, 63.95, 46.70, 24.49, 14.94; $[\alpha]_D^{25}$ 0.31 (c = 1, $CHCl_3$); IR (neat) ν_{max} = 1753, 1256, 742 cm^{-1}; m/z (HRMS$^+$) [M + K]$^+$ 935.3173 ($C_{57}H_{52}O_8SK^+$ requires 935.3014).

^{1}H NMR of 25 (400 MHz, CDCl$_3$)

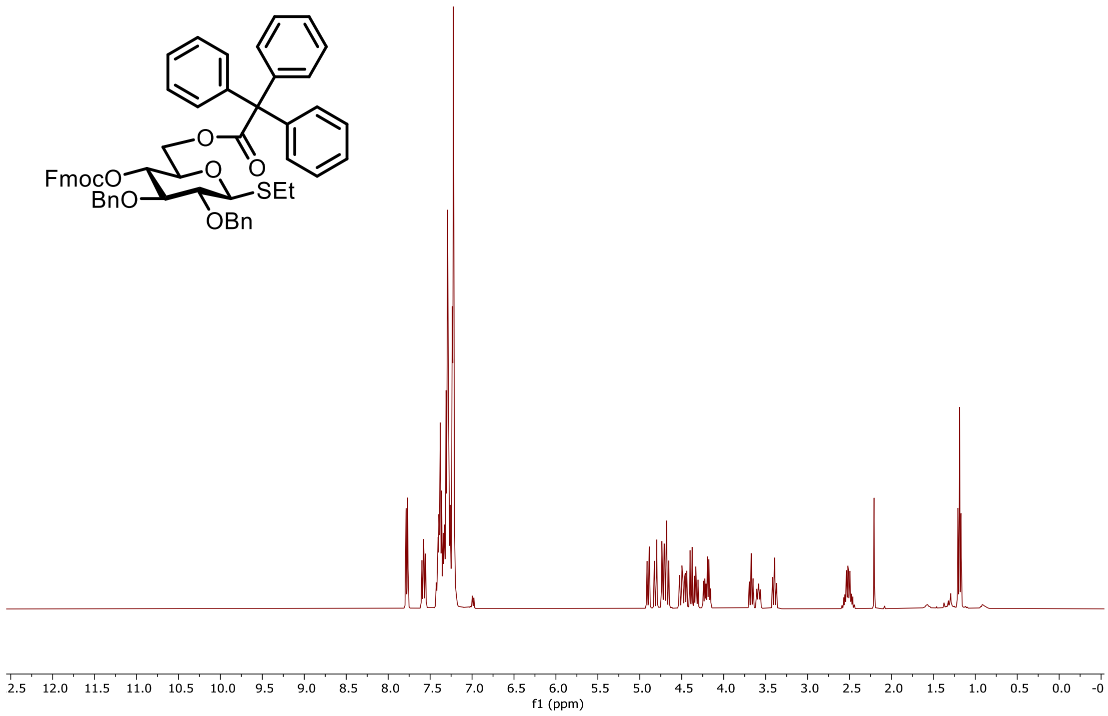

^{13}C NMR of 25 (101 MHz, CDCl$_3$)

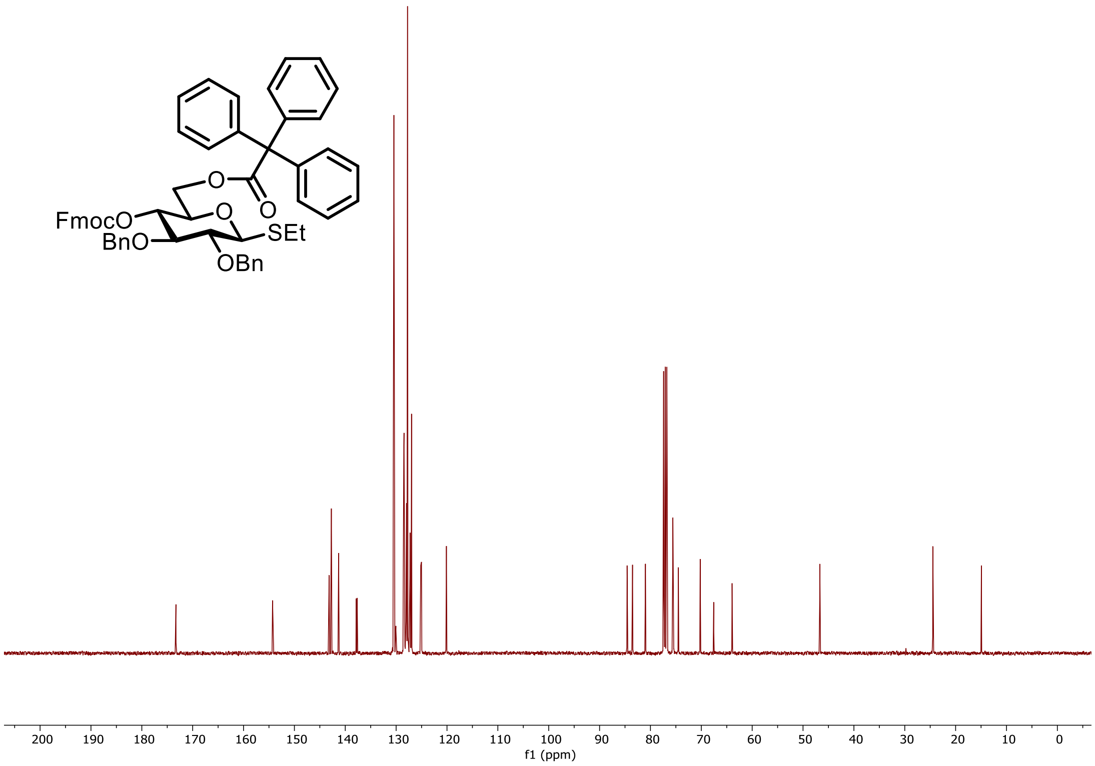

COSY NMR of 25 ($CDCl_3$)

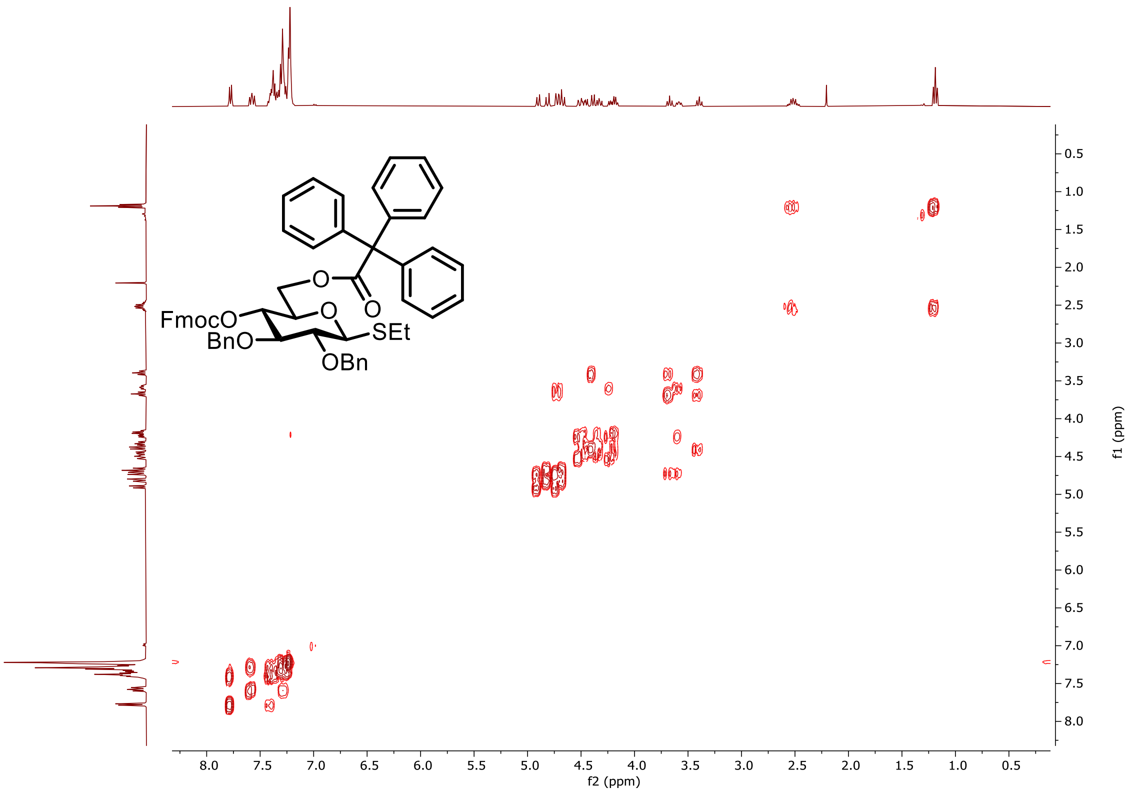

HSQC NMR of 25 ($CDCl_3$)

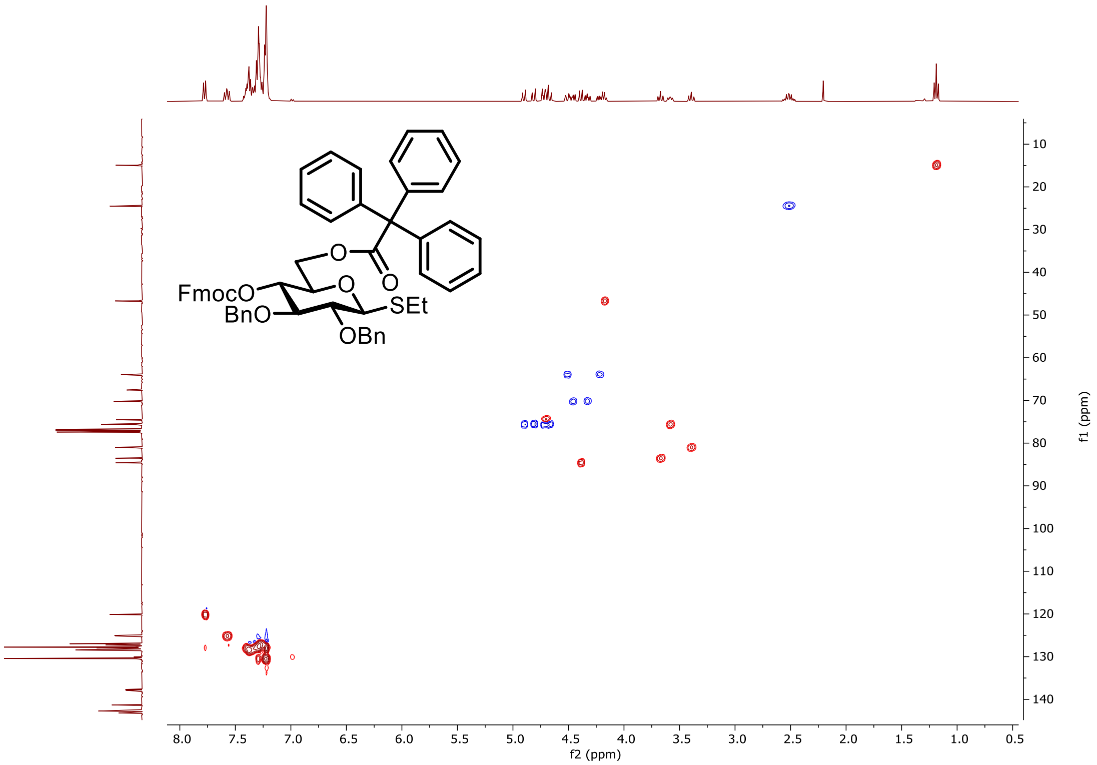

2.15 Synthesis of 26

Ethyl 2-*O*-benzyl-3-*O*-(2-naphthylmethyl)-6-*O*-levulinoyl-1-thio-β-D-glucopyranoside, 64

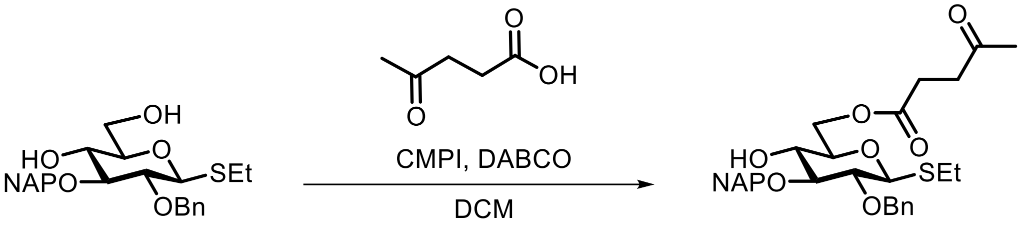

63 **64**

63 was prepared according to previously established procedures.[3]

Ethyl 2-*O*-benzyl-3-*O*-(2-naphthylmethyl)-1-thio-β-D-glucopyranoside **63** (1.81 g, 3.9 mmol) was dissolved in anhydrous DCM (40 mL), then 2-chloro-1-methylpyridium iodide (CMPI, 2.60 g, 10.2 mmol) and 1,4-diazabicyclo[2.2.2]octane (DABCO, 1.80 g, 16.0 mmol) were added at room temperature. The reaction mixture was cooled to -15 °C, and levulinic acid (LevOH, 512 mg, 4.4 mmol) was added slowly. After 3 h, DCM (30 mL) was added and the organic phase was washed with aqueous saturated $NaHCO_3$ (60 mL), and the water phase was extracted with DCM (50 mL). The obtained organic layers were combined and dried over Na_2SO_4, filtered, and evaporated. The resulting crude product was purified by column chromatography (Hexane : EtOAc = 3:1→2:1) to give **64** as a white solid (2.12 g, 97%). ^{1}H NMR (400 MHz, $CDCl_3$) δ 7.88 – 7.77 (m, 4H), 7.53 – 7.46 (m, 3H), 7.45 – 7.40 (m, 2H), 7.39 – 7.29 (m, 3H), 5.11 (d, *J* = 11.5 Hz, 1H), 5.03 – 4.94 (m, 2H), 4.79 (d, *J* = 10.2 Hz, 1H), 4.53 (d, *J* = 9.7 Hz, 1H), 4.45 (dd, *J* = 12.1, 5.0 Hz, 1H), 4.31 (dd, *J* = 12.1, 2.2 Hz, 1H), 3.65 – 3.54 (m, 2H), 3.51 – 3.42 (m, 2H), 2.89 – 2.70 (m, 4H), 2.63 (t, *J* = 6.5 Hz, 2H), 2.21 (s, 3H), 1.36 (t, *J* = 7.4 Hz, 3H); ^{13}C NMR (101 MHz, $CDCl_3$) δ 206.89, 173.25, 137.88, 135.83, 133.31, 133.04, 128.49, 128.41, 127.99, 127.77, 126.82, 126.24, 126.06, 125.87, 85.57, 85.38, 81.34, 77.55, 75.57, 75.54, 70.11, 63.70, 37.99, 29.93, 27.90, 25.27, 15.17; $[\alpha]_D^{25}$ -22.42 (c = 1, $CHCl_3$); IR (neat) ν_{max} = 1736, 1718, 1061, 753 cm^{-1}; m/z (HRMS$^+$) [M + Na]$^+$ 575.2087 ($C_{31}H_{36}O_7SNa^+$ requires 575.2074).

^{1}H NMR of 64 (400 MHz, $CDCl_3$)

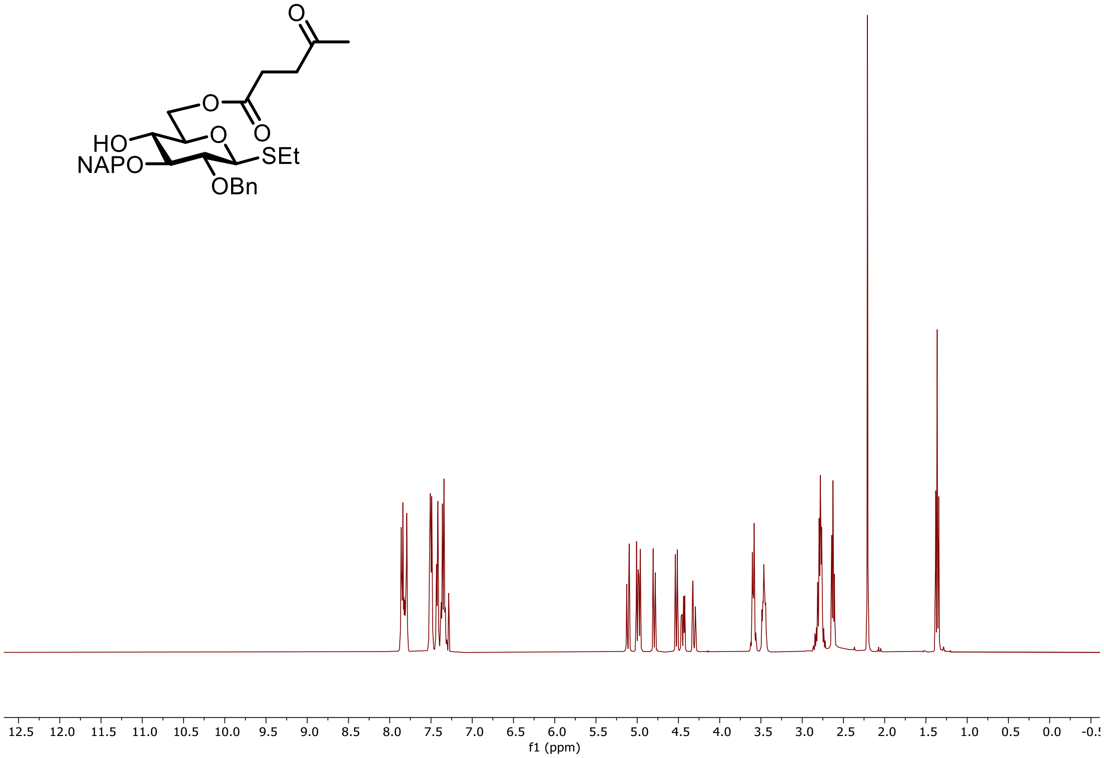

^{13}C NMR of 64 (101 MHz, $CDCl_3$)

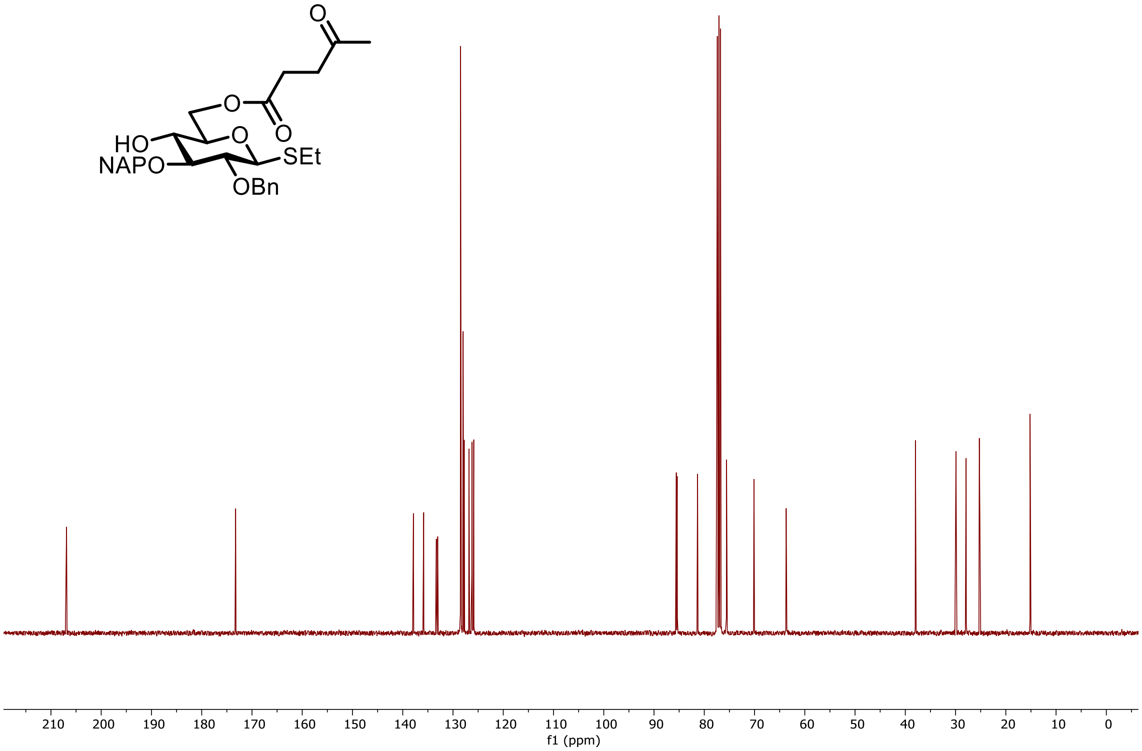

COSY NMR of 64 ($CDCl_3$)

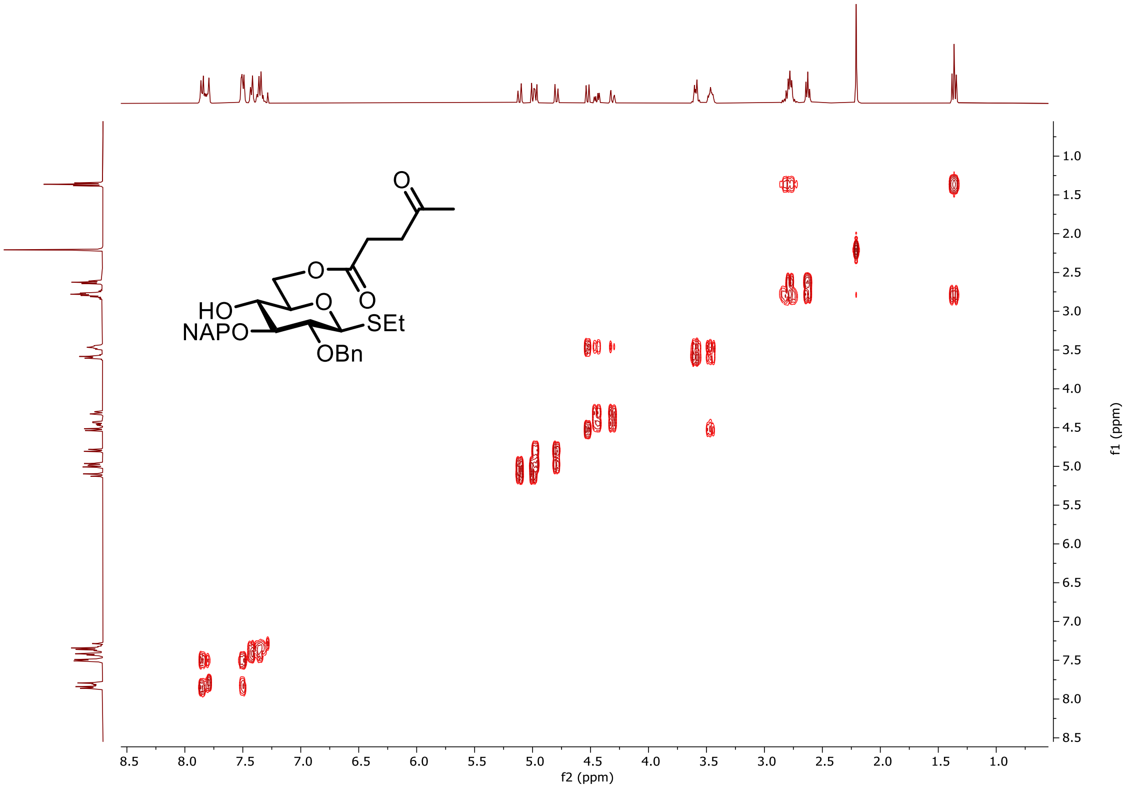

HSQC NMR of 64 ($CDCl_3$)

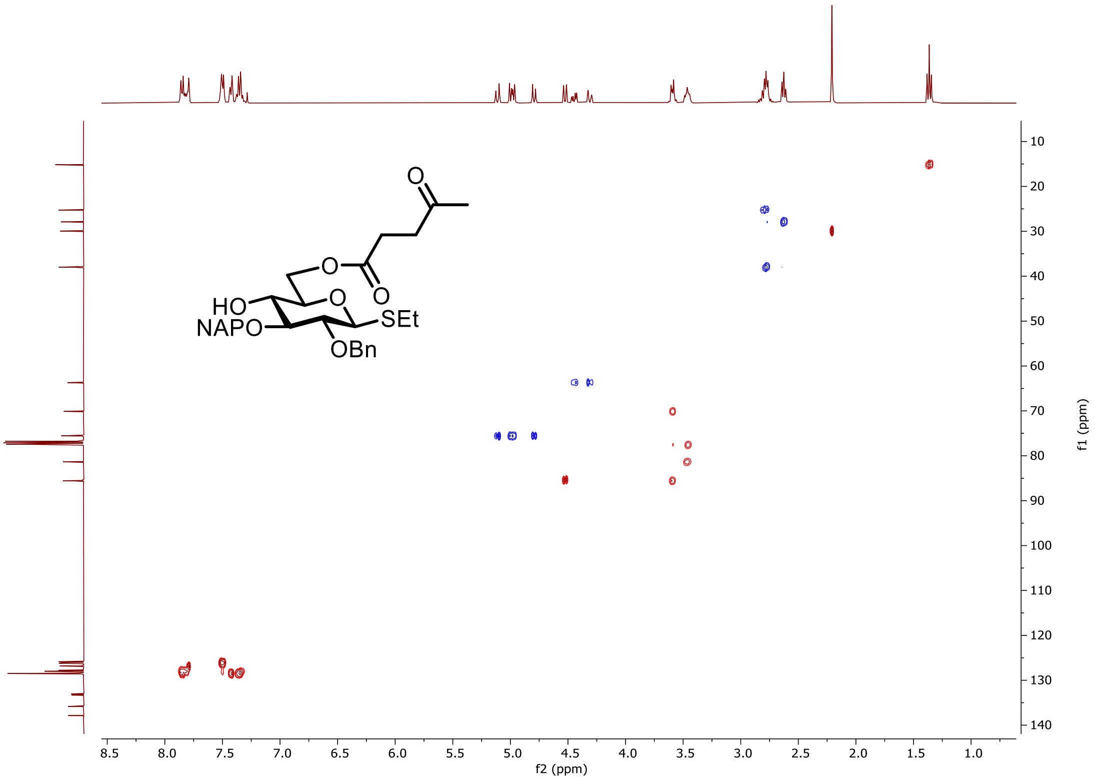

Ethyl 2-*O*-benzyl-3-*O*-(2-naphthylmethyl)-4-*O*-(9-fluorenylmethoxycarbonyl)-6-*O*-levulinoyl-1-thio-β-D-glucopyranoside, 65

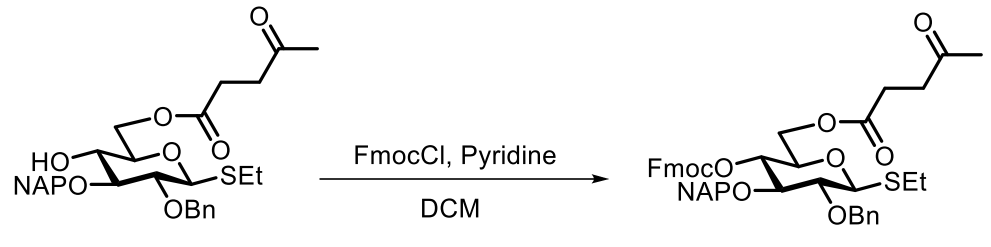

64 **65**

Ethyl 2-*O*-benzyl-3-*O*-(2-naphthylmethyl)-6-*O*-levulinoyl-1-thio-β-D-glucopyranoside **64** (2.12 g, 3.8 mmol) was dissolved in anhydrous DCM (20 mL) and pyridine (5 mL) was added. The solution was cooled with an ice bath for 30 min, and fluorenylmethyloxycarbonyl chloride (FmocCl, 2.50 g, 9.7 mmol) was added slowly. The reaction was warmed to room temperature and stirred for 6 h. Upon completion, DCM (40 mL) was added and the organic phase was washed with aqueous citric acid (0.5 M, 40 mL). After extracting the water phase with DCM (20 mL), the organic layers were combined and dried over Na_2SO_4, filtered, and evaporated. The resulting crude product was purified by column chromatography (Hexane : EtOAc : DCM =6:1:1→Hexane : EtOAc : DCM =3:1:1) to give **65** as a white solid (2.60 g, 88%). ^{1}H NMR (400 MHz, $CDCl_3$) δ 7.80 – 7.674 (m, 3H), 7.73 – 7.64 (m, 3H), 7.54 (dd, *J* = 7.4, 5.3 Hz, 2H), 7.49 – 7.32 (m, 10H), 7.31 – 7.22 (m, 2H), 5.06 – 4.92 (m, 3H), 4.86 (d, *J* = 12.1 Hz, 1H), 4.80 (d, *J* = 10.2 Hz, 1H), 4.56 (d, *J* = 9.8 Hz, 1H), 4.45 (dd, *J* = 10.4, 7.0 Hz, 1H), 4.36 – 4.21 (m, 3H), 4.13 (appt, *J* = 7.2 Hz, 1H), 3.81 (appt, *J* = 9.1 Hz, 1H), 3.70 (ddd, *J* = 10.0, 5.4, 2.6 Hz, 1H), 3.58 (appt, *J* = 9.3 Hz, 1H), 2.91 – 2.56 (m, 6H), 2.20 (s, 3H), 1.39 (t, *J* = 7.4 Hz, 3H); ^{13}C NMR (101 MHz, $CDCl_3$) δ 206.55, 172.46, 154.41, 143.24, 143.17, 141.29, 141.27, 137.72, 135.39, 133.21, 132.96, 128.52, 128.43, 128.13, 128.07, 127.93, 127.70, 127.24, 127.23, 126.51, 126.10, 125.93, 125.74, 125.14, 125.03, 120.09, 120.07, 85.36, 83.72, 81.28, 75.70, 75.46, 74.49, 70.21, 62.92, 46.63, 37.89, 29.93, 27.88, 25.24, 15.21; $[\alpha]_D^{25}$ 13.02 (c = 1, $CHCl_3$); IR (neat) ν_{max} = 1746, 1256, 739 cm^{-1}; m/z (HRMS$^+$) [M + Na]$^+$ 797.2847 ($C_{46}H_{46}O_9SNa^+$ requires 797.2755).

^{1}H NMR of 65 (400 MHz, CDCl$_3$)

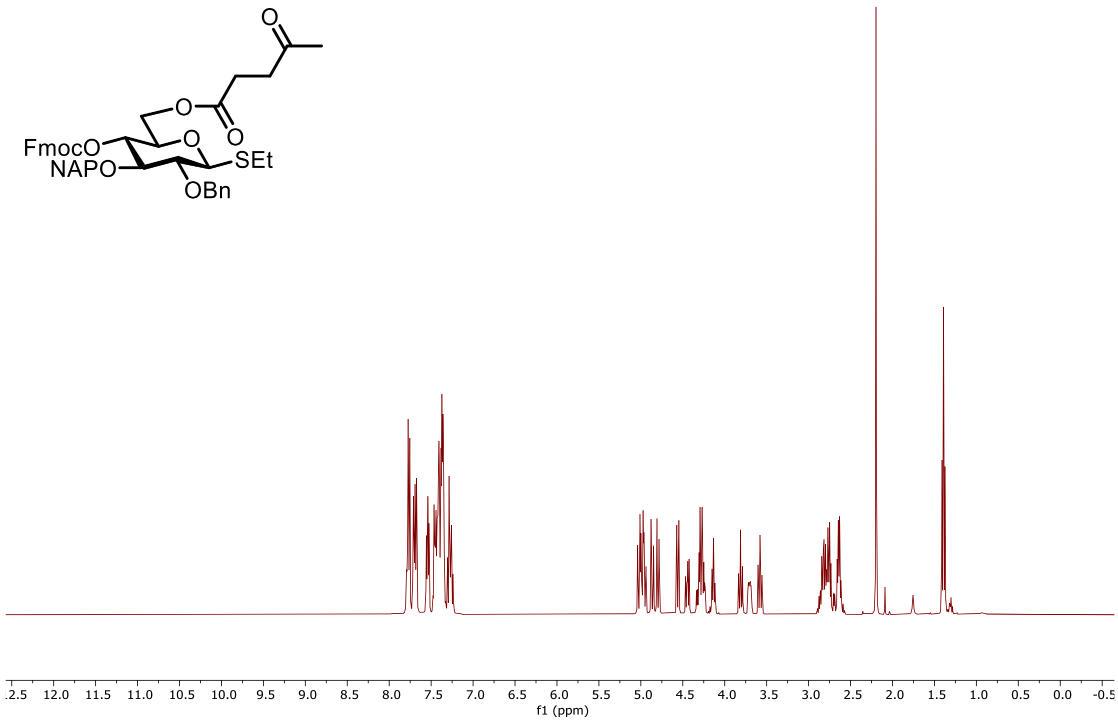

^{13}C NMR of 65 (101 MHz, CDCl$_3$)

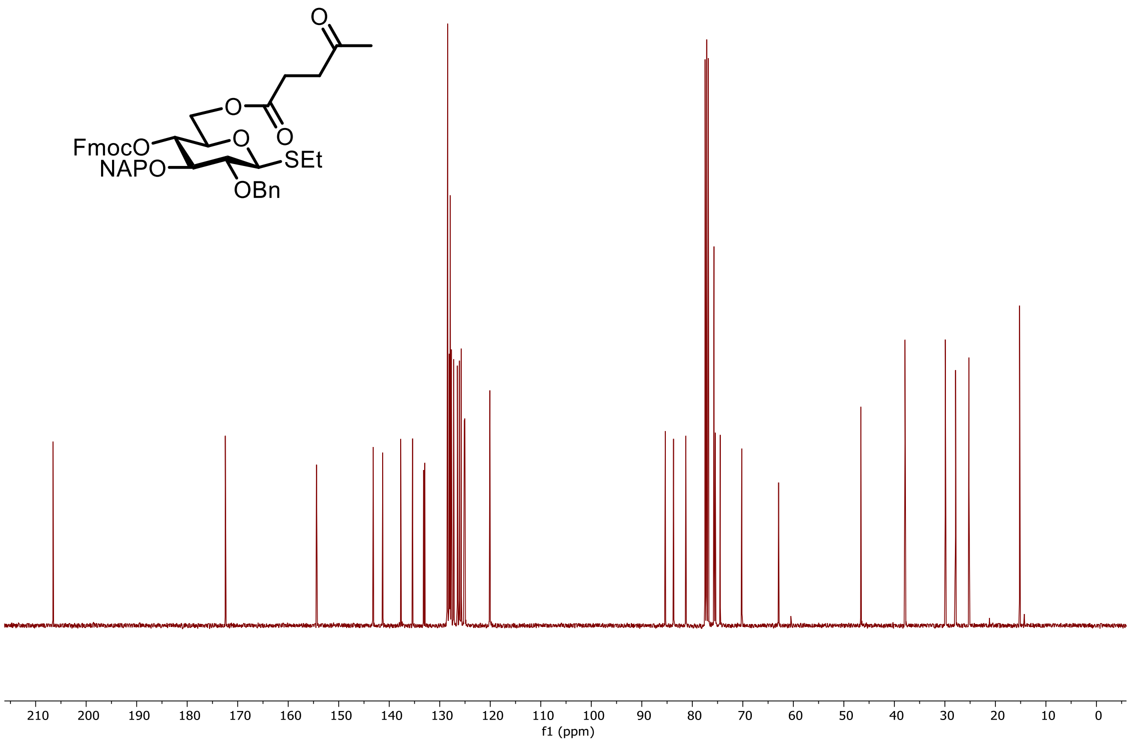

COSY NMR of 65 ($CDCl_3$)

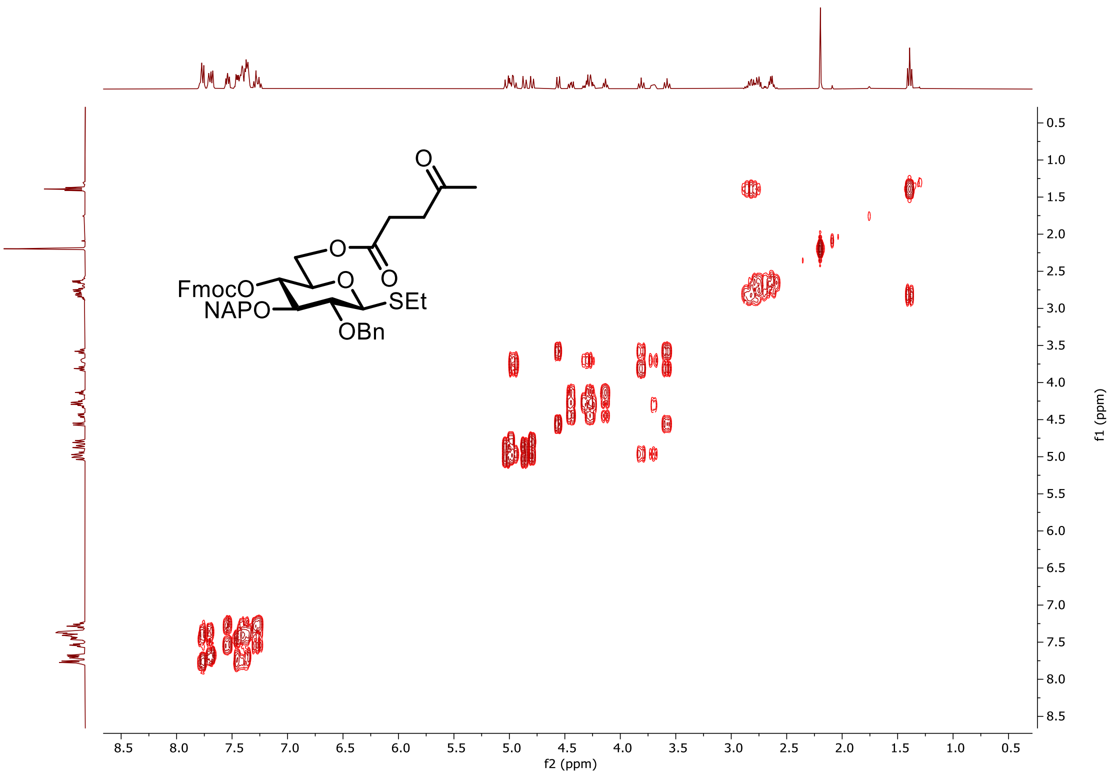

HSQC NMR of 65 ($CDCl_3$)

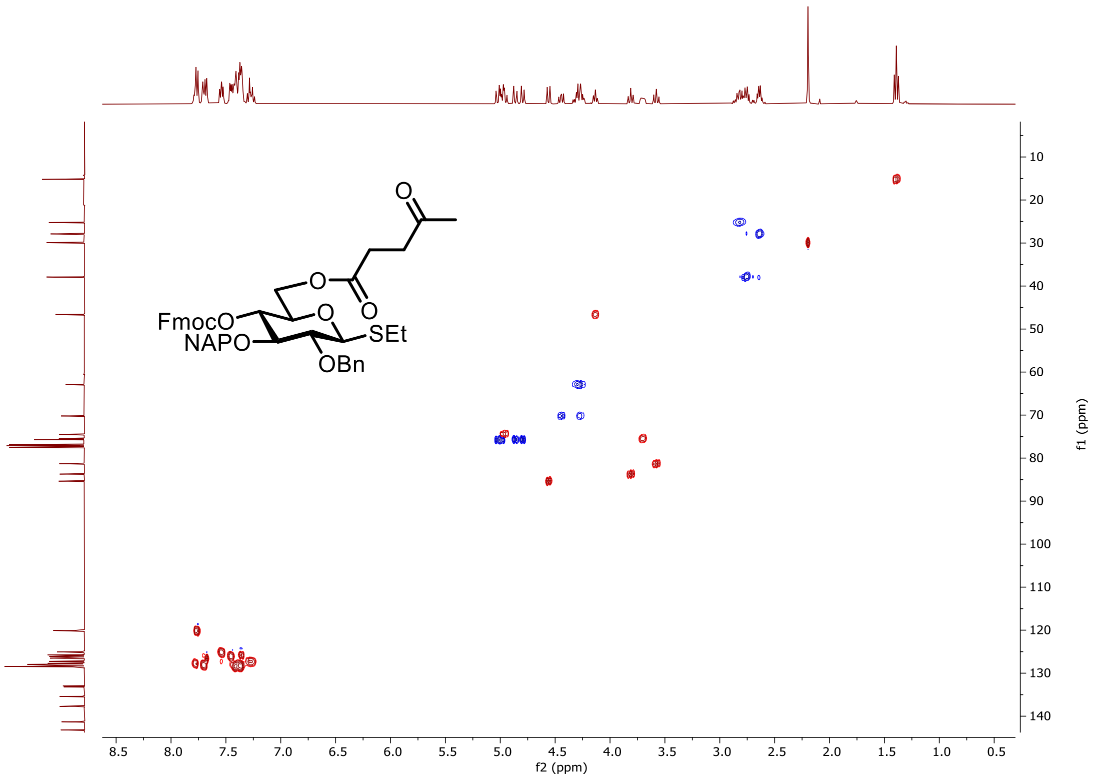

Ethyl 2-*O*-benzyl-3-*O*-(2-naphthylmethyl)-4-*O*-(9-fluorenylmethoxycarbonyl)-1-thio-β-D-glucopyranoside, 66

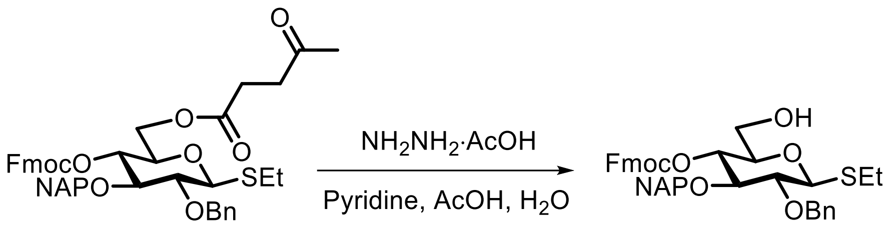

Ethyl 2-*O*-benzyl-3-*O*-(2-naphthylmethyl)-4-*O*-(9-fluorenylmethoxycarbonyl)-6-*O*-levulinoyl-1-thio-β-D-glucopyranoside **65** (2.60 g, 3.4 mmol) was dissolved in a mixture of pyridine (40 mL) and acetic acid (10 mL), H_2O (2.5 mL) was added. After a clear solution was obtained, hydrazine acetate (410 mg, 4.5 mmol) was added. The reaction was kept at room temperature for 3 h. After it was completed, the reaction was quenched by adding acetone (5 mL). The solvent was removed by evaporation as much as possible, and DCM (100 mL) was added. The solution was washed with aqueous citric acid (0.5 M, 80 mL, 3 times), and the water layers were combined and extracted with DCM (50 mL). The obtained organic layers were also combined and dried over Na_2SO_4, filtered, and evaporated. The resulting crude product was purified by column chromatography (Hexane : EtOAc = 6:1→2:1) to give **66** as a white solid (2.20 g, 95%). ^{1}H NMR (400 MHz, $CDCl_3$) δ 7.82 – 7.67 (m, 6H), 7.55 – 7.42 (m, 4H), 7.42 – 7.31 (m, 8H), 7.31 – 7.22 (m, 2H), 5.04 – 4.86 (m, 4H), 4.78 (d, *J* = 10.2 Hz, 1H), 4.55 (d, *J* = 9.8 Hz, 1H), 4.45 (dd, *J* = 10.5, 7.2 Hz, 1H), 4.29 (dd, *J* = 10.5, 6.8 Hz, 1H), 4.11 (appt, *J* = 7.0 Hz, 1H), 3.86 – 3.71 (m, 2H), 3.62 (dd, *J* = 12.6, 5.1 Hz, 1H), 3.58 – 3.42 (m, 2H), 2.89 – 2.70 (m, 2H), 1.99 (br. s, 1H), 1.36 (t, *J* = 7.4 Hz, 3H); ^{13}C NMR (101 MHz, $CDCl_3$) δ 154.87, 143.17, 142.93, 141.32, 141.27, 137.70, 135.51, 133.21, 132.94, 128.49, 128.41, 128.10, 128.03, 127.94, 127.68, 127.19, 126.40, 126.09, 125.90, 125.70, 125.02, 124.94, 120.11, 120.09, 85.26, 83.63, 81.25, 77.93, 75.70, 75.61, 74.57, 70.09, 61.62, 46.68, 25.13, 15.16; $[\alpha]_D^{25}$ 10.27 (c = 1, $CHCl_3$); IR (neat) ν_{max} = 1752, 1258, 740 cm^{-1}; m/z (HRMS$^+$) [M + Na]$^+$ 699.2445 ($C_{41}H_{40}O_7SNa^+$ requires 699.2387).

^{1}H NMR of 66 (400 MHz, $CDCl_3$)

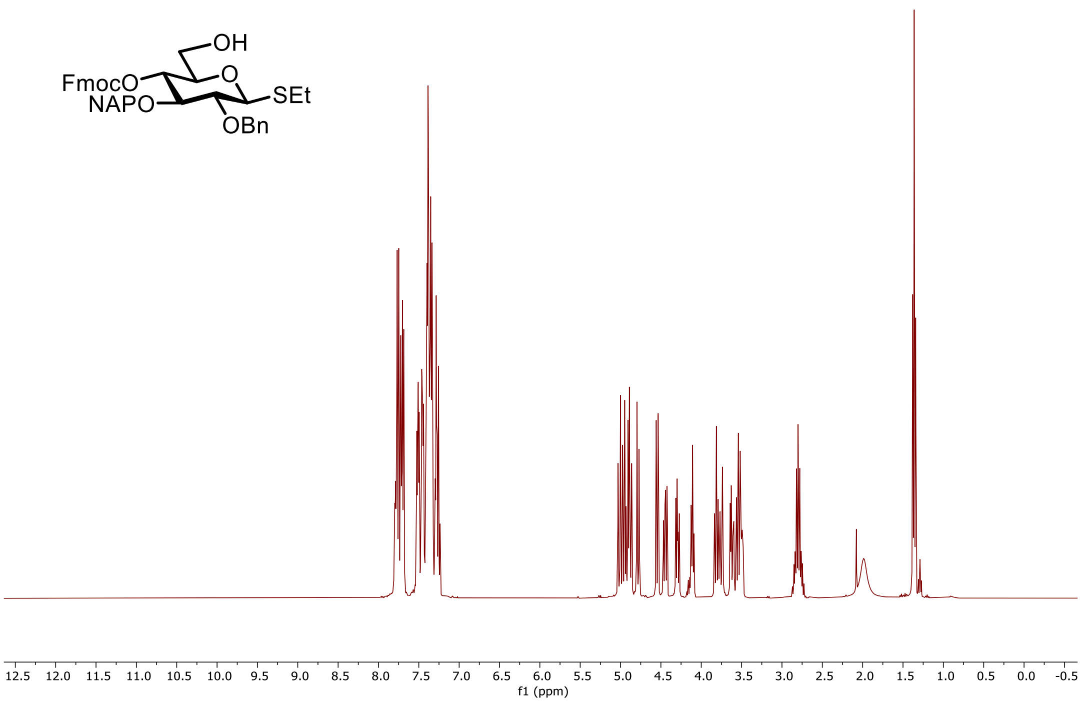

^{13}C NMR of 66 (101 MHz, $CDCl_3$)

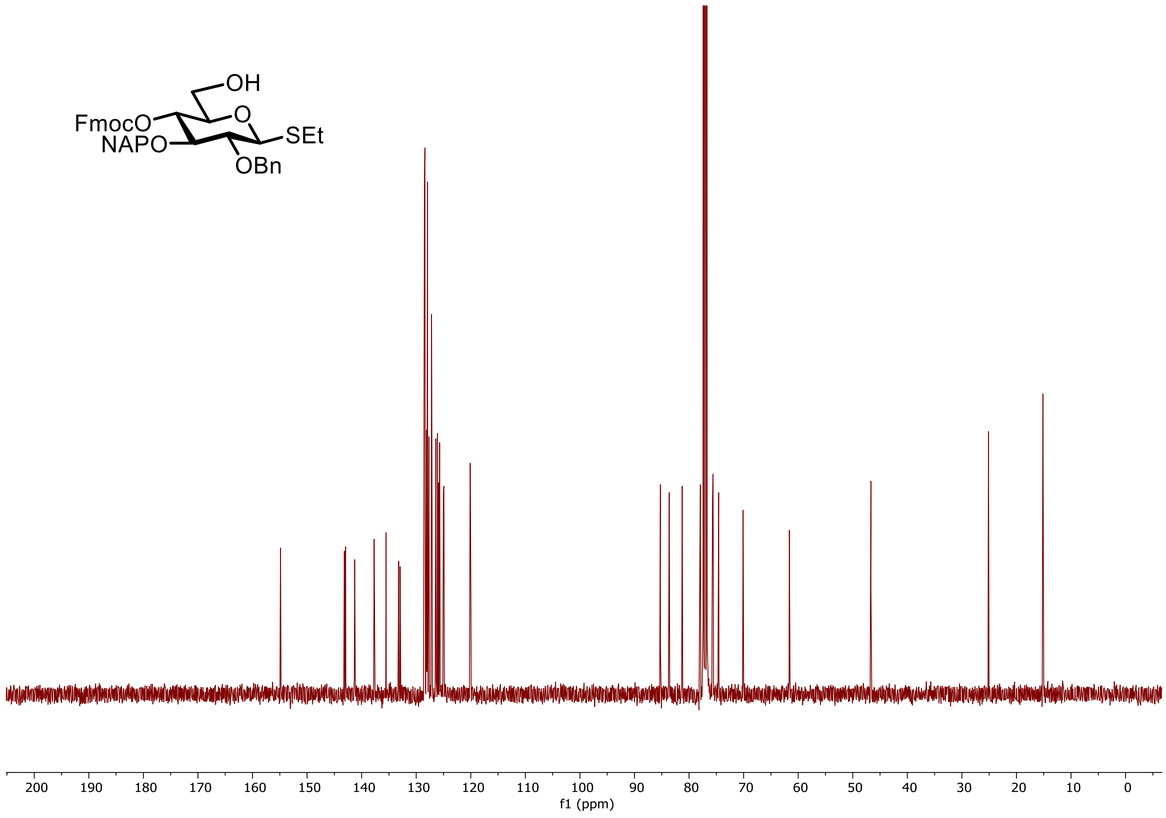

COSY NMR of 66 ($CDCl_3$)

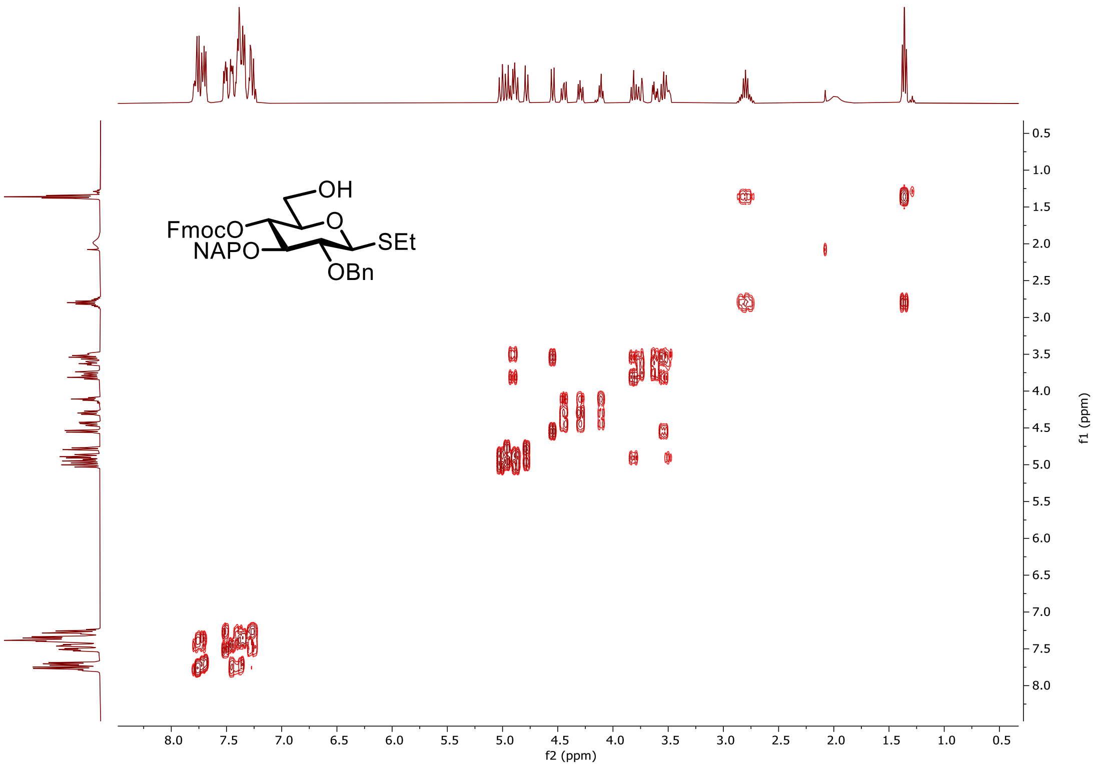

HSQC NMR of 66 ($CDCl_3$)

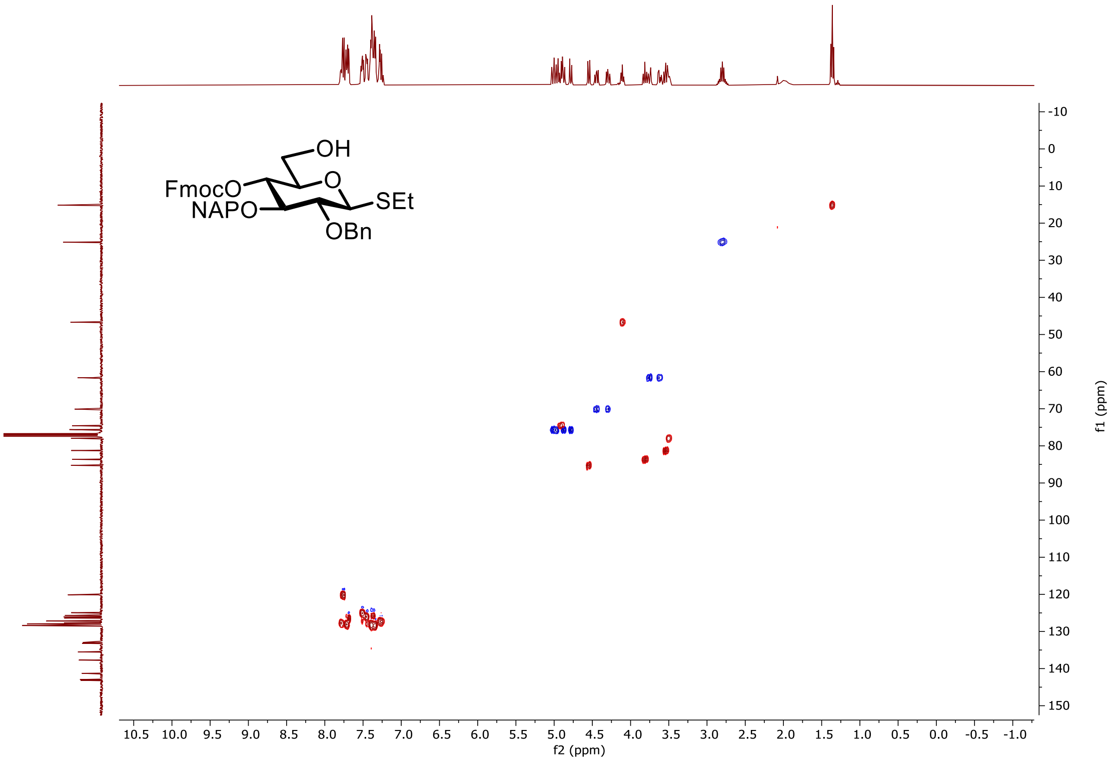

Ethyl 2-*O*-benzyl-4-*O*-(9-fluorenylmethoxycarbonyl)-1-thio-β-D-glucopyranoside, 67

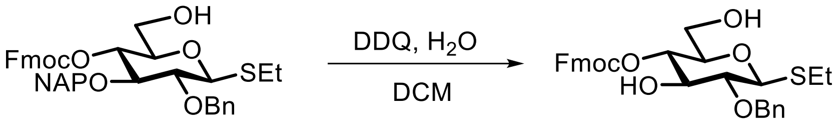

Ethyl 2-*O*-benzyl-3-*O*-(2-naphthylmethyl)-4-*O*-(9-fluorenylmethoxycarbonyl)-1-thio-β-D-glucopyranoside **66** (2.20 g, 3.2 mmol) was dissolved in DCM (50 mL) and water (2.5 mL) was added. 2,3-Dichloro-5,6-dicyano-1,4-benzoquinone (DDQ) (1.48 g, 6.6 mmol) was added, and reaction was stirred for 6 h at room temperature. DCM (50 mL) was added to dilute the reaction mixture and the solution was washed with $Na_2S_2O_3$ (aq) (5%, 100 mL).The water layer was extracted with DCM (50 mL), and the obtained organic layers were combined and dried over Na_2SO_4, filtered, and evaporated. The resulting crude product was purified by column chromatography (Hexane : EtOAc = 3:1→1:1) to give **67** as a white solid (1.65 g, 95%). ^{1}H NMR (400 MHz, $CDCl_3$) δ 7.79 (d, *J* = 7.6 Hz, 2H), 7.62 (dd, *J* = 7.6, 4.0 Hz, 2H), 7.49 – 7.31 (m, 9H), 5.00 (d, *J* = 10.9 Hz, 1H), 4.78 (appt, *J* = 9.7 Hz, 1H), 4.72 (d, *J* = 10.8 Hz, 1H), 4.56 – 4.47 (m, 3H), 4.28 (appt, *J* = 7.1 Hz, 1H), 3.85 (appt, *J* = 9.1 Hz, 1H), 3.77 (dd, *J* = 12.5, 2.4 Hz, 1H), 3.62 (dd, *J* = 12.6, 4.9 Hz, 1H), 3.50 (ddd, *J* = 10.4, 5.0, 2.4 Hz, 1H), 3.37 (appt, *J* = 9.2 Hz, 1H), 2.80 (p, *J* = 7.1 Hz, 2H), 2.13 (br. s, 2H), 1.36 (t, *J* = 7.4 Hz, 3H); ^{13}C NMR (101 MHz, $CDCl_3$) δ 155.22, 143.24, 142.97, 141.36, 141.33, 137.70, 128.70, 128.40, 128.28, 127.99, 127.98, 127.25, 125.09, 120.15, 120.14, 84.94, 81.19, 77.71, 75.92, 75.49, 74.22, 70.28, 61.56, 46.75, 25.24, 15.11; $[\alpha]_D^{25}$ 0.92 (c = 1, $CHCl_3$); IR (neat) ν_{max} = 1750, 1259, 741 cm^{-1}; m/z (HRMS$^+$) [M + Na]$^+$ 559.1775 ($C_{30}H_{32}O_7SNa^+$ requires 559.1761).

^{1}H NMR of 67 (400 MHz, $CDCl_3$)

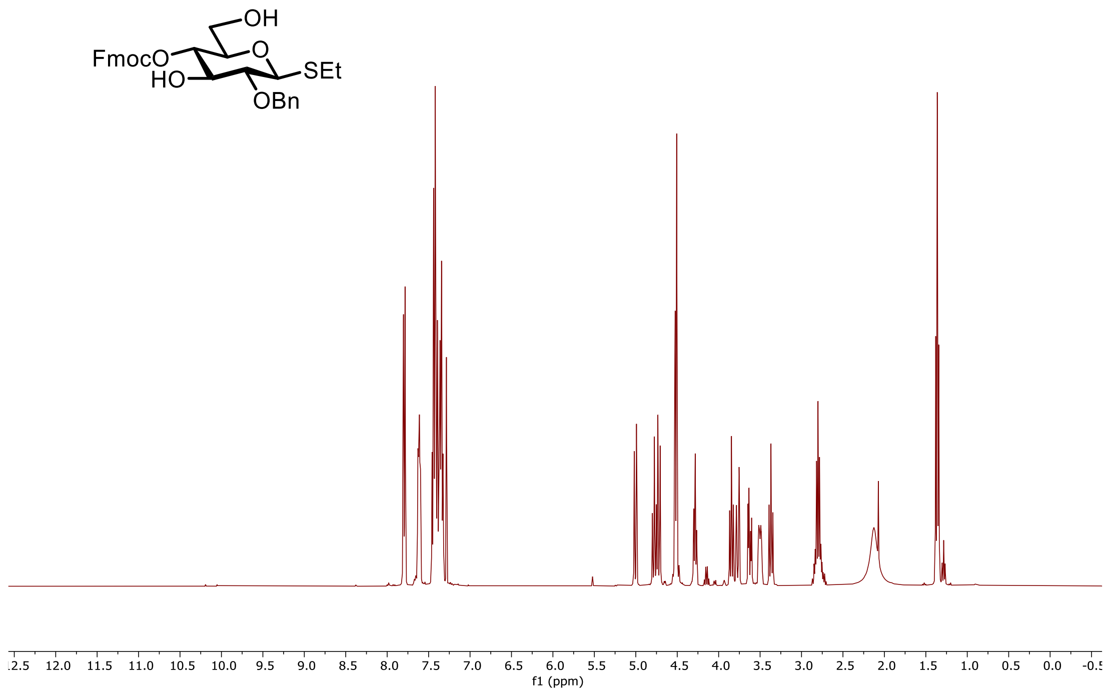

^{13}C NMR of 67 (101 MHz, $CDCl_3$)

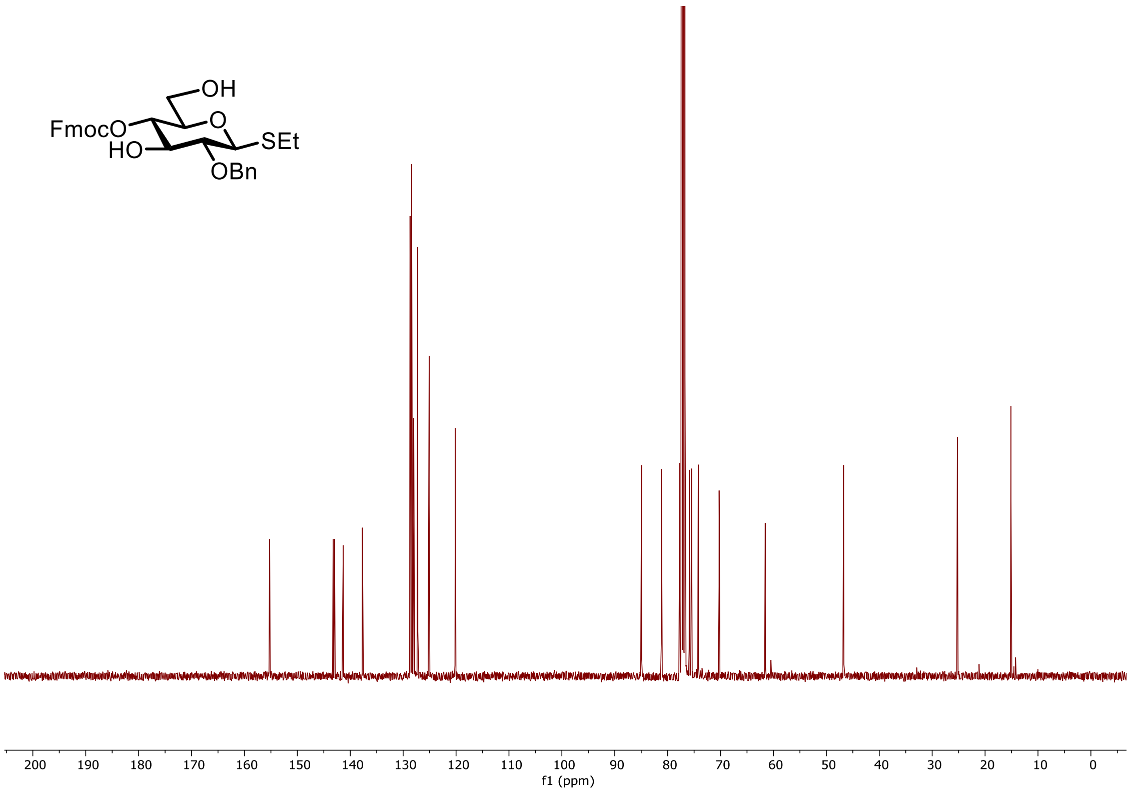

COSY NMR of 67 ($CDCl_3$)

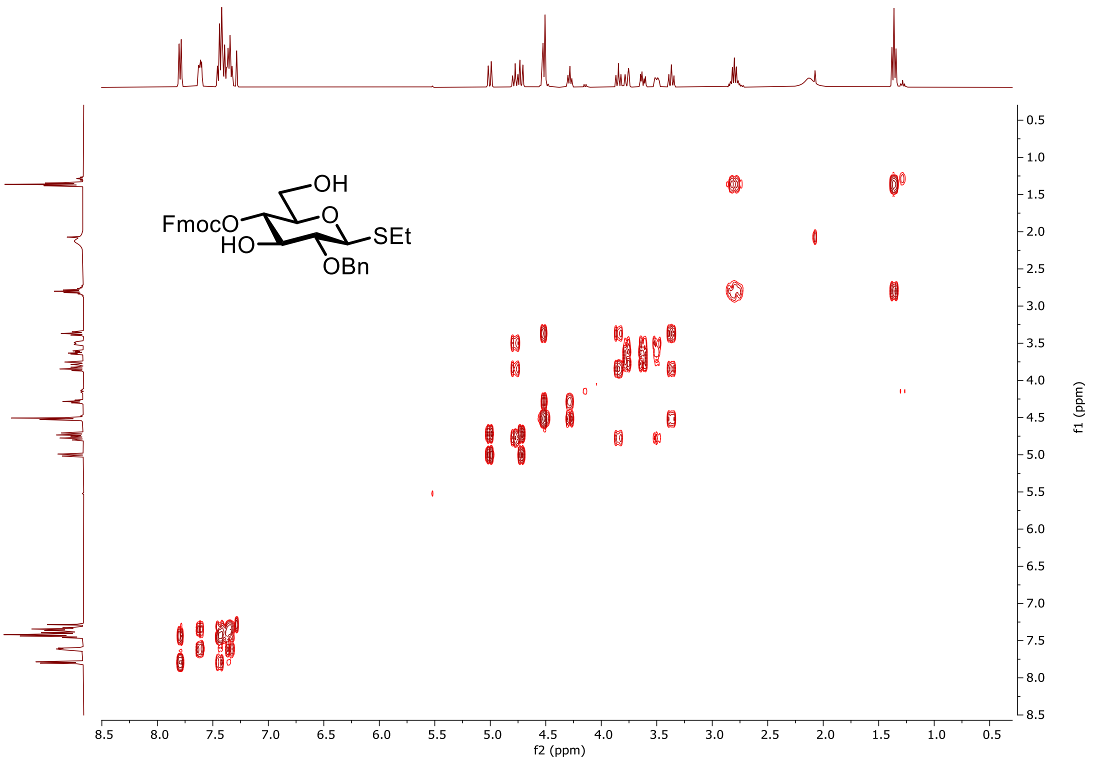

HSQC NMR of 67 ($CDCl_3$)

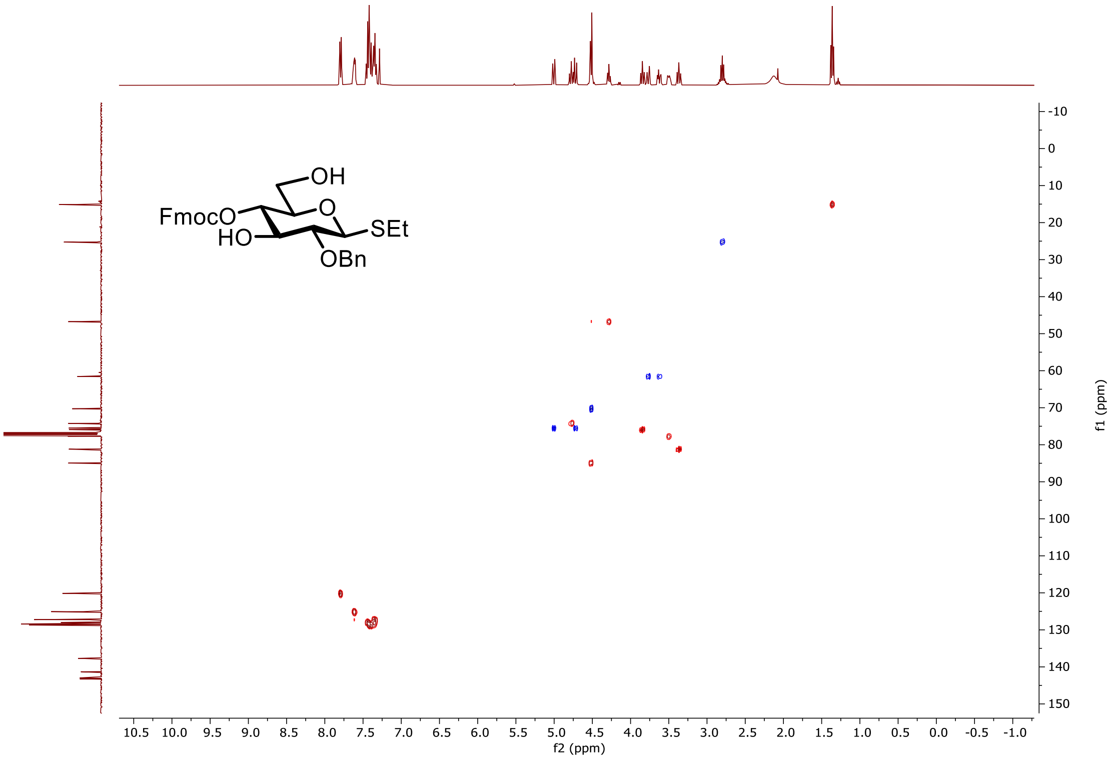

Ethyl 2-*O*-benzyl-3,6-di-*O*-acetyl-4-*O*-(9-fluorenylmethoxycarbonyl)-1-thio-β-D-glucopyranoside, 26

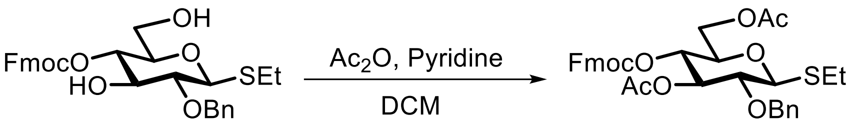

67 **26**

Ethyl 2-*O*-benzyl-4-*O*-(9-fluorenylmethoxycarbonyl)-1-thio-β-D-glucopyranoside **67** (200 mg, 0.37 mmol) was dissolved in anhydrous DCM (3 mL) and pyridine (1 mL) was added. The solution was cooled with an ice bath for 30 min, and acetic anhydride (Ac_2O, 142 µL, 1.5 mmol) was added slowly. The reaction was warmed to room temperature and stirred for 16 h. Completion of the reaction was confirmed by TLC, MeOH (0.2 mL) was added. DCM (10 mL) was added 30 min later, and the organic phase was washed with aqueous citric acid (0.5 M, 10 mL). After extracting the water phase with DCM (5 mL), the organic layers were combined and dried over Na_2SO_4, filtered, and evaporated. The resulting crude product was purified by column chromatography (Hexane : EtOAc = 8:1→4:1) to give **26** as a white solid (213 mg, 92%). ^{1}H NMR (400 MHz, $CDCl_3$) δ 7.79 (d, *J* = 7.5 Hz, 2H), 7.60 (t, *J* = 7.2 Hz, 2H), 7.43 (t, *J* = 7.5 Hz, 2H), 7.40 – 7.28 (m, 7H), 5.36 (appt, *J* = 9.3 Hz, 1H), 4.93 – 4.83 (m, 2H), 4.61 (d, *J* = 4.0 Hz, 1H), 4.58 (d, *J* = 2.5 Hz, 1H), 4.46 – 4.30 (m, 3H), 4.29 – 4.18 (m, 2H), 3.80 (ddd, *J* = 10.0, 5.1, 2.4 Hz, 1H), 3.52 (appt, *J* = 9.4 Hz, 1H), 2.81 (qd, *J* = 7.4, 3.8 Hz, 2H), 2.09 (s, 3H), 1.87 (s, 3H), 1.37 (t, *J* = 7.4 Hz, 3H); ^{13}C NMR (101 MHz, $CDCl_3$) δ 170.67, 169.96, 154.26, 143.18, 143.01, 141.26, 137.47, 128.50, 128.17, 128.02, 127.98, 127.34, 127.31, 125.19, 125.12, 120.12, 120.09, 85.49, 79.07, 75.27, 75.21, 75.10, 72.84, 70.60, 62.43, 46.51, 25.56, 20.82, 20.77, 15.10; $[\alpha]_D^{25}$ -7.07 (c = 1, $CHCl_3$); IR (neat) ν_{max} = 1750, 1262, 1223, 741 cm^{-1}; m/z ($HRMS^+$) $[M + K]^+$ 659.1758 ($C_{34}H_{36}O_9SK^+$ requires 659.1712).

^{1}H NMR of 26 (400 MHz, CDCl$_3$)

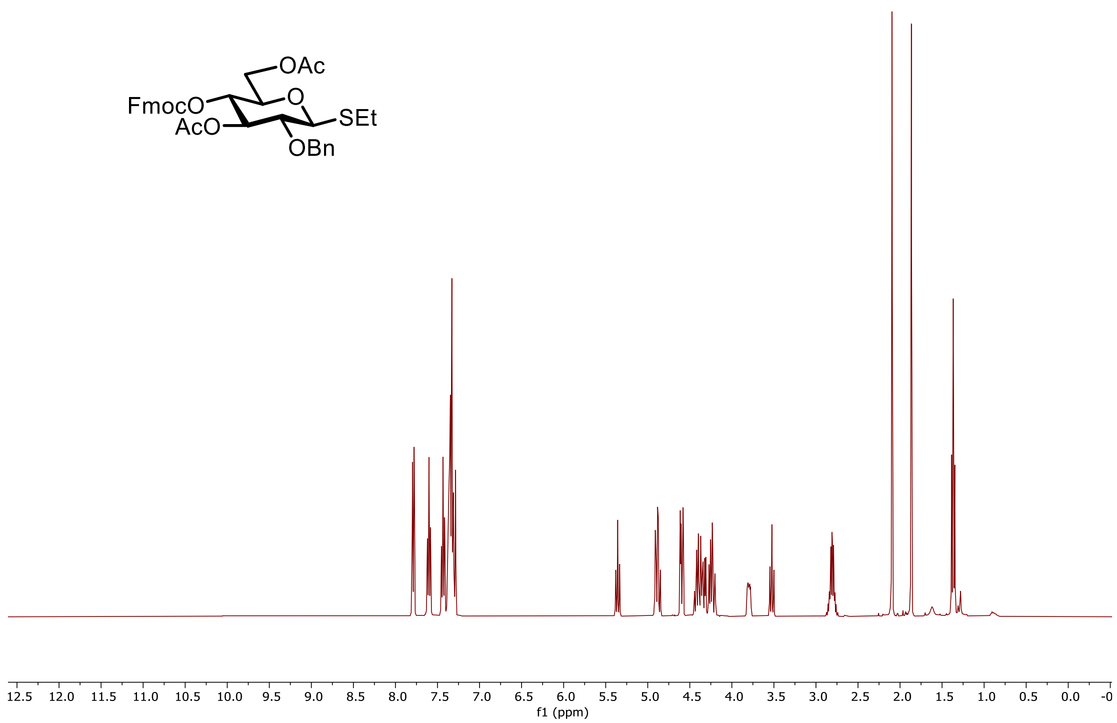

^{13}C NMR of 26 (101 MHz, CDCl$_3$)

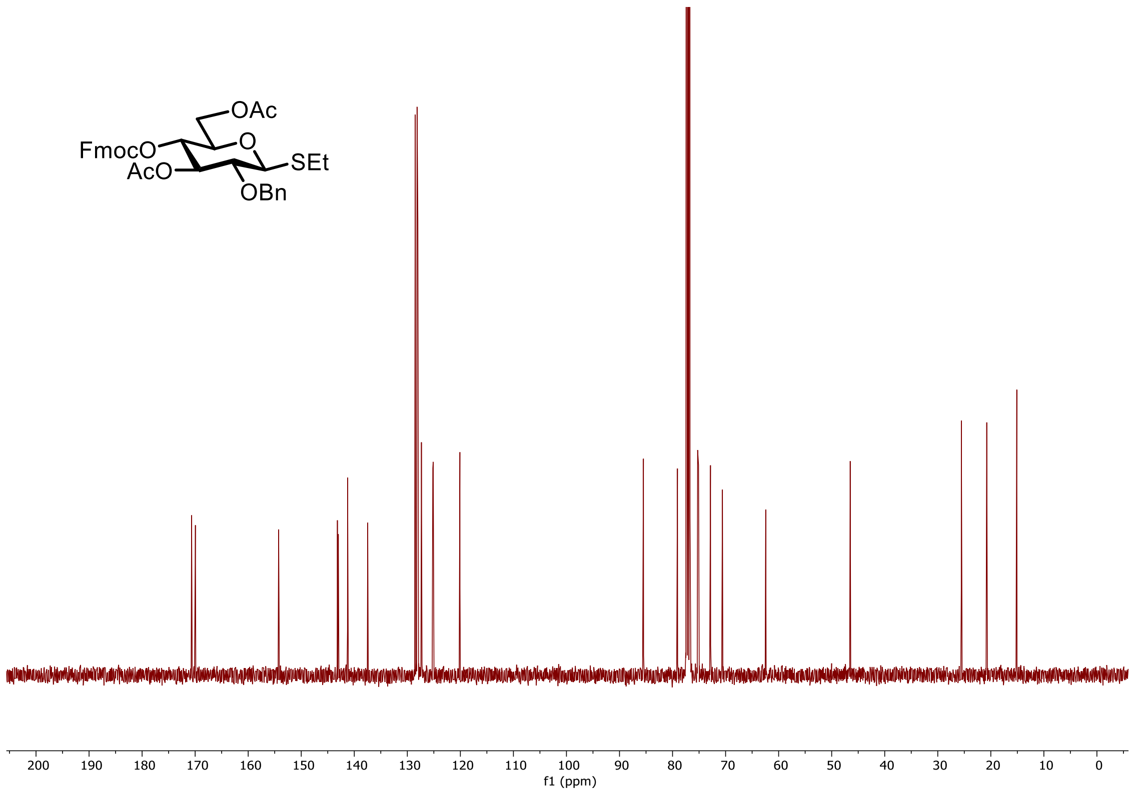

COSY NMR of 26 ($CDCl_3$)

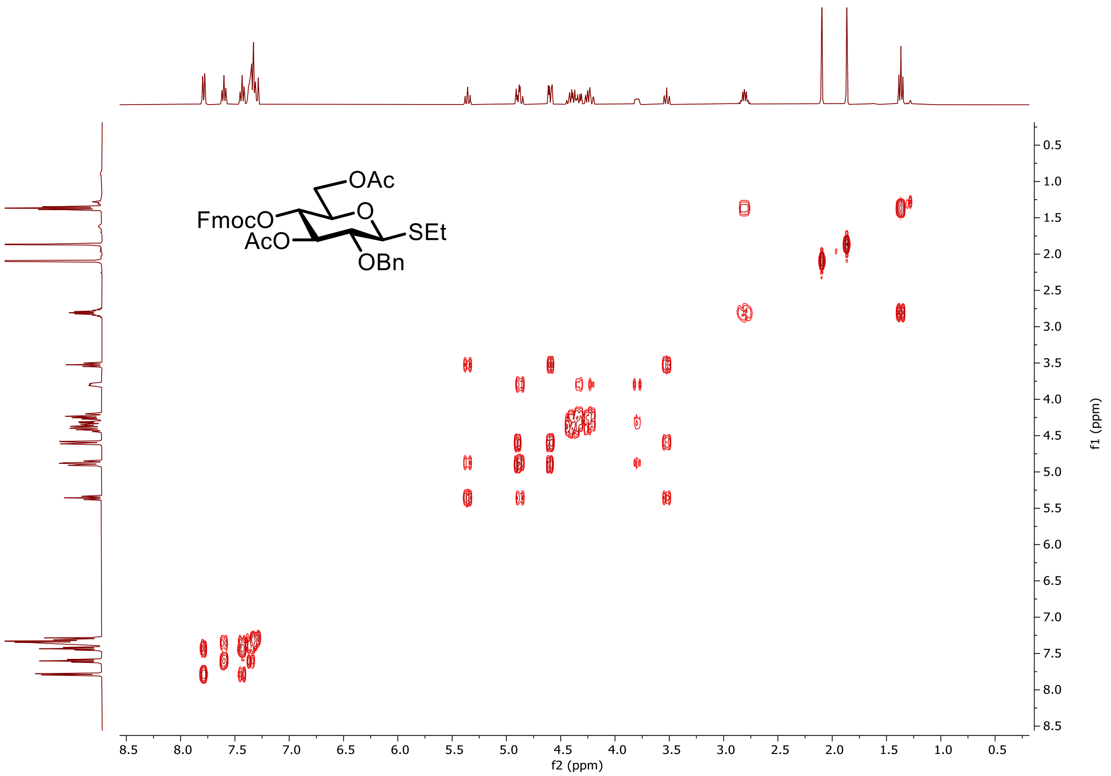

HSQC NMR of 26 ($CDCl_3$)

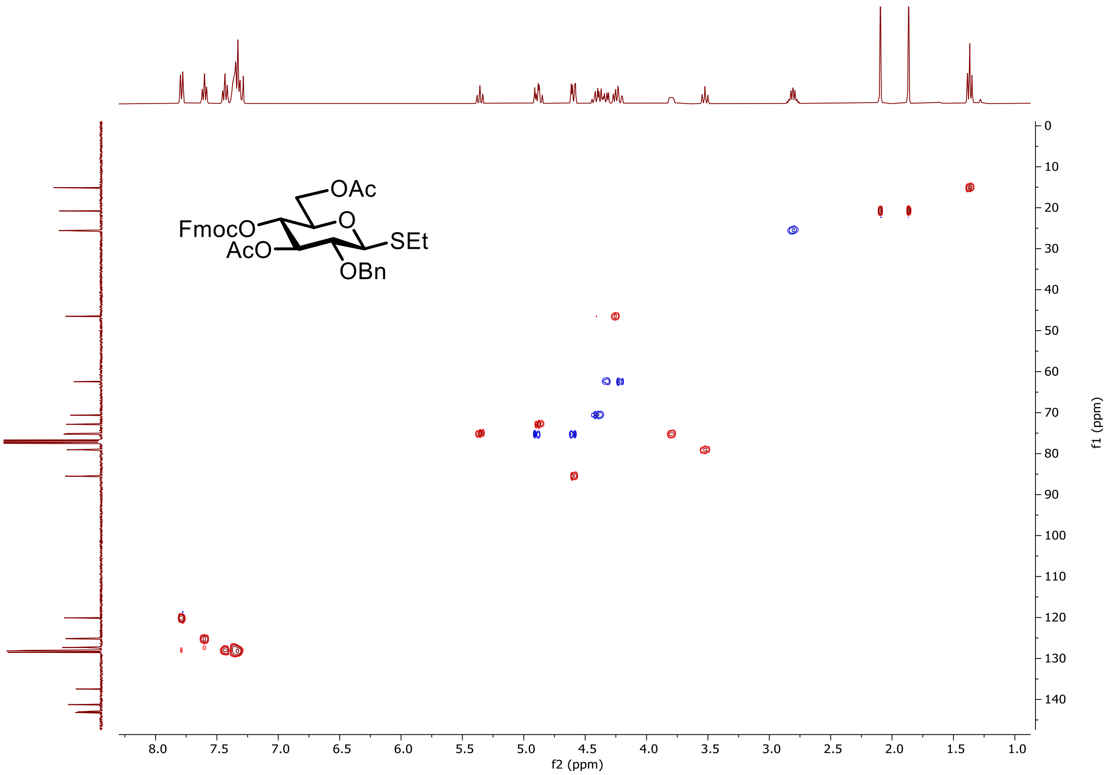

2.16 Synthesis of 27

Ethyl 2-*O*-benzyl-3,6-di-*O*-pivaloyl-4-*O*-(9-fluorenylmethoxycarbonyl)-1-thio-β-D-glucopyranoside, 27

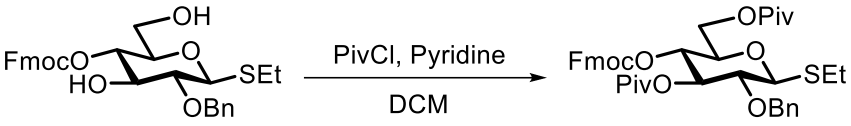

67 **27**

Ethyl 2-*O*-benzyl-4-*O*-(9-fluorenylmethoxycarbonyl)-1-thio-β-D-glucopyranoside **67** (200 mg, 0.37 mmol) was dissolved in anhydrous DCM (12 mL) and pyridine (4 mL) was added. The solution was cooled with an ice bath for 30 min, and pivaloyl chloride (PivCl, 550 μL, 4.5 mmol) was added slowly. The reaction was warmed to room temperature and stirred for 48 h. MeOH (1.0 mL) was added to quench the reaction and DCM (30 mL) was added 2 h later, and the organic phase was washed with aqueous citric acid (0.5 M, 30 mL). After extracting the water phase with DCM (15 mL), the organic layers were combined and dried over Na_2SO_4, filtered, and evaporated. The resulting crude product was purified by column chromatography (Hexane : EtOAc = 10:1→8:1) to give **27** as a clear oil (90 mg, 34%). ^{1}H NMR (400 MHz, $CDCl_3$) δ 7.79 (d, *J* = 7.5 Hz, 2H), 7.62 (dd, *J* = 10.5, 7.5 Hz, 2H), 7.44 (t, *J* = 7.5 Hz, 2H), 7.40 – 7.26 (m, 7H), 5.45 (appt, *J* = 9.3 Hz, 1H), 4.97 – 4.85 (m, 2H), 4.62 (dd, *J* = 13.3, 10.1 Hz, 2H), 4.44 (dd, *J* = 9.7, 6.7 Hz, 1H), 4.35 – 4.20 (m, 4H), 3.83 (ddd, *J* = 9.9, 5.6, 2.5 Hz, 1H), 3.55 (appt, *J* = 9.4 Hz, 1H), 2.80 (dtt, *J* = 20.2, 12.9, 7.4 Hz, 2H), 1.36 (t, *J* = 7.4 Hz, 3H), 1.26 (s, 9H), 1.11 (s, 9H); ^{13}C NMR (101 MHz, $CDCl_3$) δ 178.06, 177.20, 154.22, 143.24, 142.96, 141.29, 141.21, 137.46, 128.39, 128.03, 127.98, 127.82, 127.53, 127.39, 127.33, 125.37, 125.16, 120.13, 120.07, 84.96, 79.47, 75.56, 74.96, 74.85, 73.07, 70.60, 62.71, 46.54, 38.87, 38.83, 27.16, 26.99, 25.14, 15.17; $[\alpha]_D^{25}$ 11.71 (c = 1, $CHCl_3$); IR (neat) ν_{max} = 1753, 1737, 1258, 1149 cm^{-1}; m/z (HRMS$^+$) [M + Na]$^+$ 727.2983 ($C_{40}H_{48}O_9SNa^+$ requires 727.2911).

^{1}H NMR of 27 (400 MHz, $CDCl_3$)

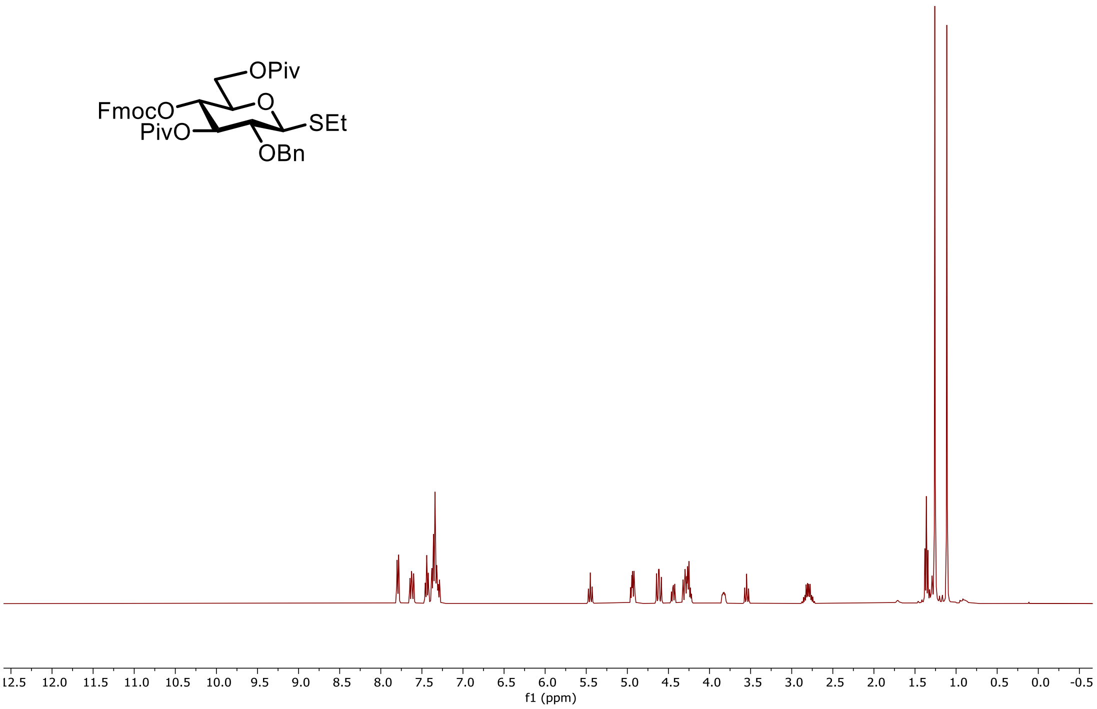

^{13}C NMR of 27 (101 MHz, $CDCl_3$)

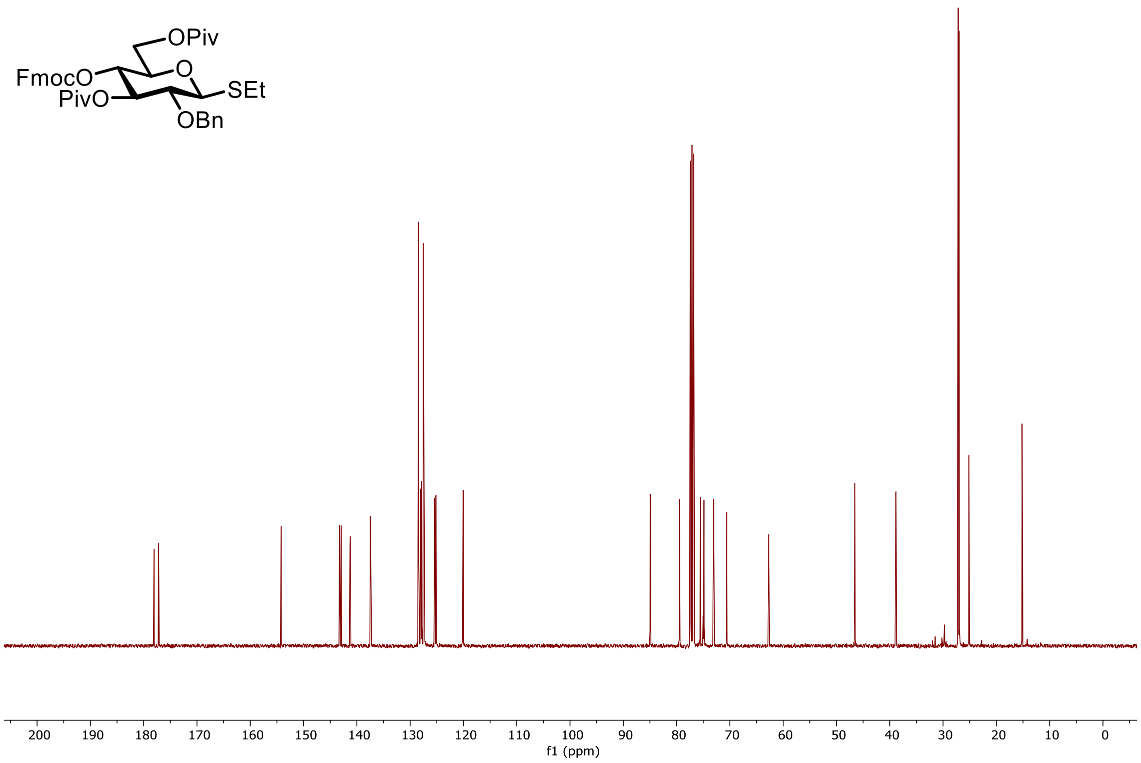

COSY NMR of 27 ($CDCl_3$)

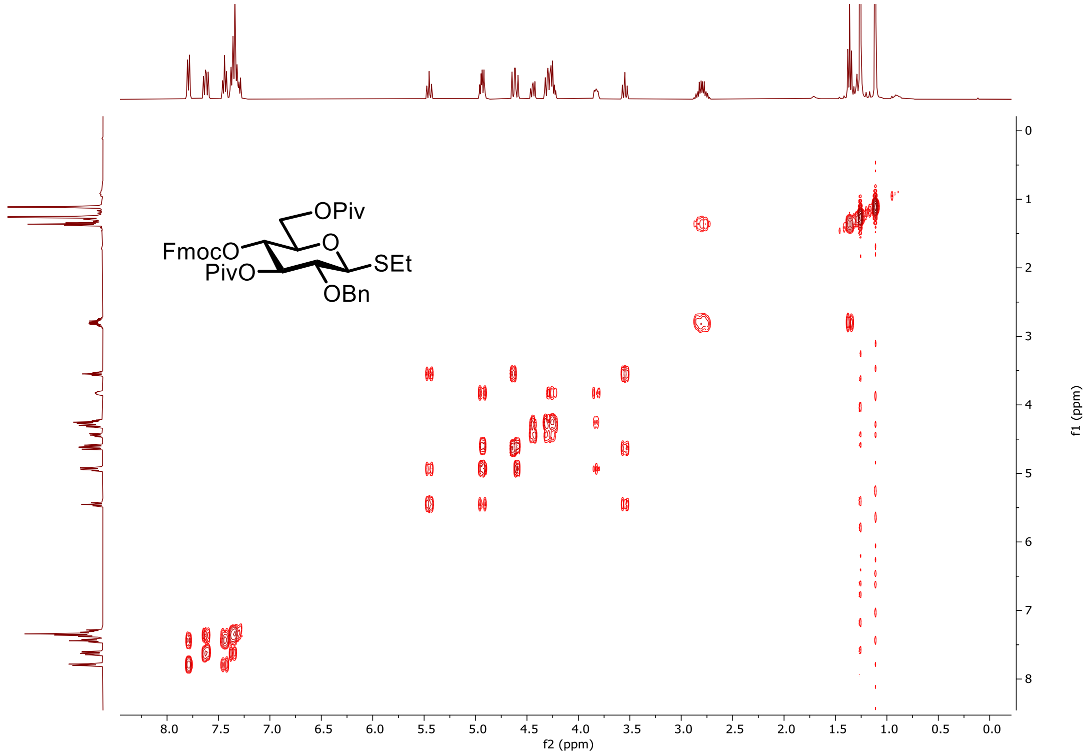

HSQC NMR of 27 ($CDCl_3$)

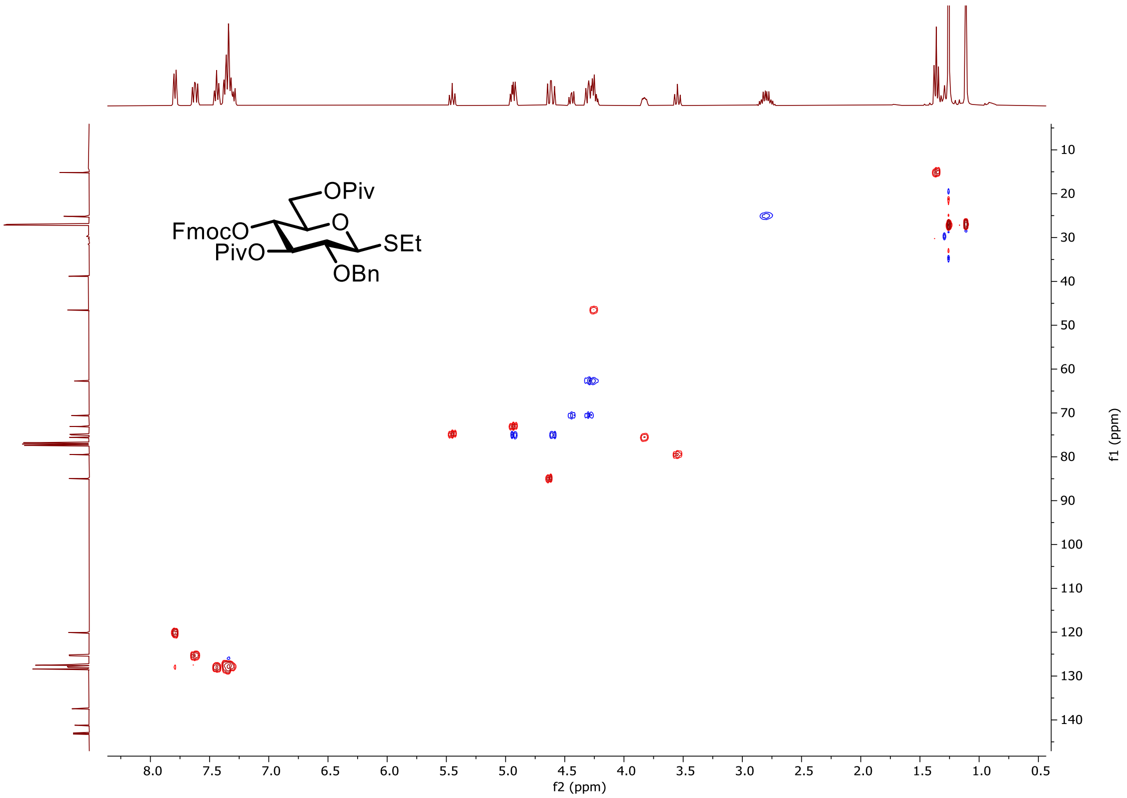

2.17 Synthesis of 28

Ethyl 2-*O*-benzyl-3,6-di-*O*-benzoyl-4-*O*-(9-fluorenylmethoxycarbonyl)-1-thio-β-D-glucopyranoside, 28

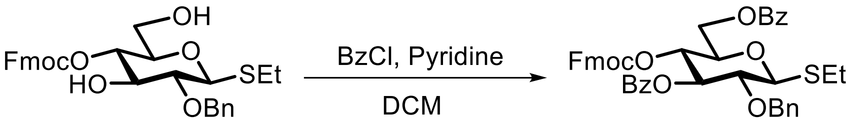

67 **28**

Ethyl 2-*O*-benzyl-4-*O*-(9-fluorenylmethoxycarbonyl)-1-thio-β-D-glucopyranoside **67** (200 mg, 0.37 mmol) was dissolved in anhydrous DCM (3 mL) and pyridine (1 mL) was added. The solution was cooled with an ice bath for 30 min, and benzoyl chloride (BzCl, 174 µL, 1.5 mmol) was added slowly. The reaction was warmed to room temperature and stirred for 24 h. After the reaction was finished, MeOH (0.2 mL) was added. DCM (10 mL) was added 2 h later, and the organic phase was washed with aqueous citric acid (0.5 M, 10 mL). After extracting the water phase with DCM (5 mL), the organic layers were combined and dried over Na_2SO_4, filtered, and evaporated. The resulting crude product was purified by column chromatography (Hexane : EtOAc = 8:1→Hexane : EtOAc : DCM =6:1:1) to give **28** as a white solid (215 mg, 78%). ^1H NMR (400 MHz, $CDCl_3$) δ 8.14 (d, *J* = 8.1 Hz, 2H), 8.01 (d, *J* = 8.1 Hz, 2H), 7.73 (dd, *J* = 7.6, 3.2 Hz, 2H), 7.65 – 7.56 (m, 1H), 7.55 – 7.32 (m, 9H), 7.32 – 7.13 (m, 7H), 5.74 (appt, *J* = 9.4 Hz, 1H), 5.19 (appt, *J* = 9.8 Hz, 1H), 4.88 (d, *J* = 10.8 Hz, 1H), 4.76 (d, *J* = 9.7 Hz, 1H), 4.69 – 4.59 (m, 2H), 4.54 (dd, *J* = 12.1, 5.4 Hz, 1H), 4.24 (dd, *J* = 10.5, 7.4 Hz, 1H), 4.15 (dd, *J* = 10.4, 7.5 Hz, 1H), 4.06 (ddd, *J* = 9.1, 5.4, 3.0 Hz, 1H), 3.98 (appt, *J* = 7.4 Hz, 1H), 3.74 (appt, *J* = 9.4 Hz, 1H), 2.84 (qd, *J* = 12.7, 6.5 Hz, 2H), 1.38 (t, *J* = 7.4 Hz, 3H); ^{13}C NMR (101 MHz, $CDCl_3$) δ 166.11, 165.61, 154.32, 143.17, 142.96, 141.16, 141.10, 137.09, 133.38, 133.29, 129.95, 129.86, 129.74, 129.28, 128.49, 128.45, 128.43, 128.34, 127.95, 127.86, 127.83, 127.23, 127.20, 125.18, 125.07, 119.96, 119.94, 85.45, 78.98, 75.78, 75.41, 75.21, 73.32, 70.45, 63.33, 46.44, 25.47, 15.21; $[\alpha]_D^{25}$ 4.14 (c = 1, $CHCl_3$); IR (neat) ν_{max} = 1753, 1725, 1265, 710 cm^{-1}; m/z ($HRMS^+$) $[M + Na]^+$ 767.2385 ($C_{44}H_{40}O_9SNa^+$ requires 767.2285).

^{1}H NMR of 28 (400 MHz, CDCl$_3$)

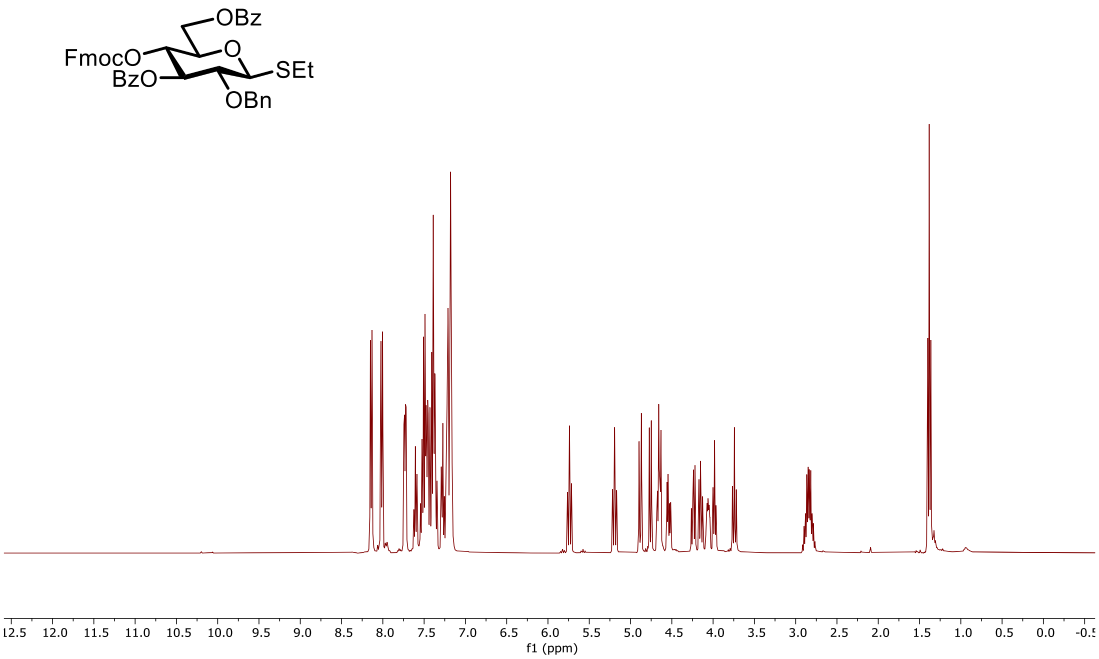

^{13}C NMR of 28 (101 MHz, CDCl$_3$)

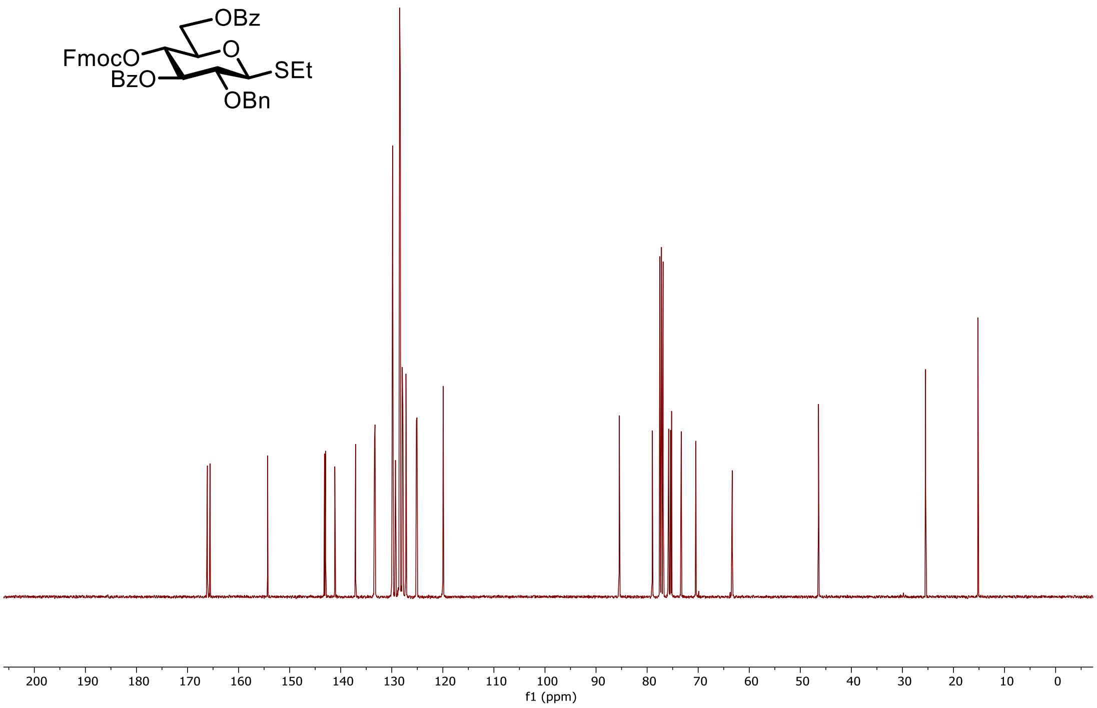

COSY NMR of 28 ($CDCl_3$)

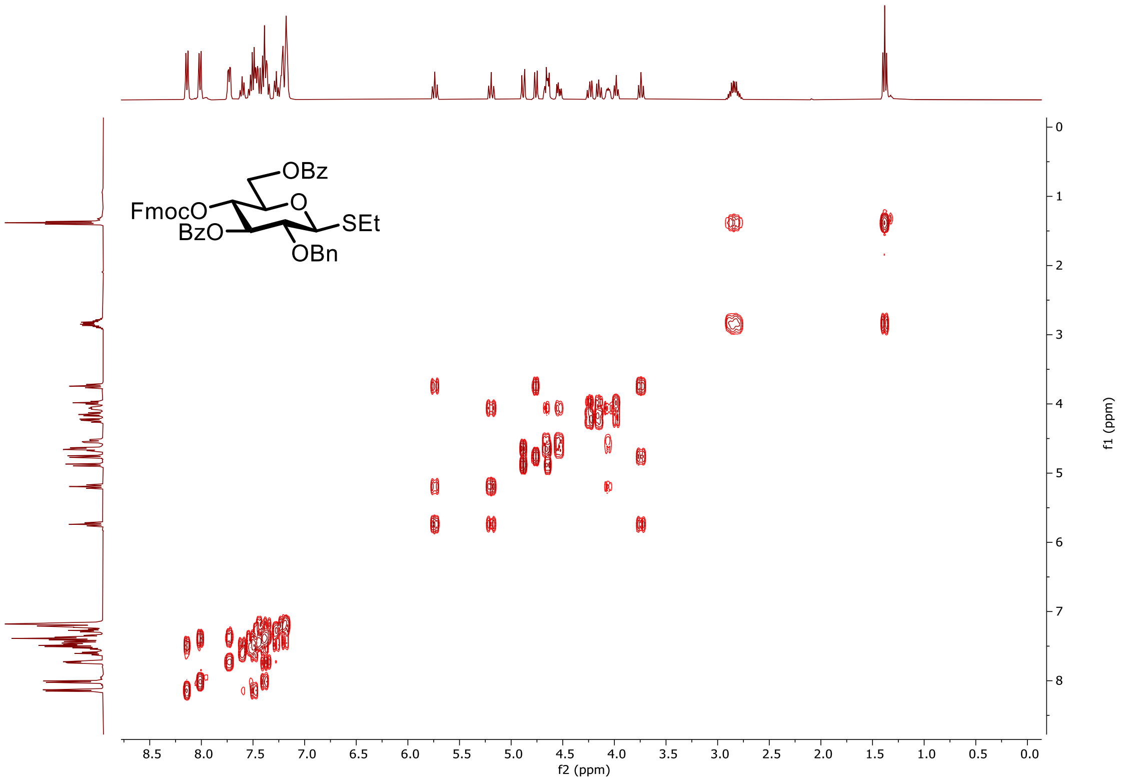

HSQC NMR of 28 ($CDCl_3$)

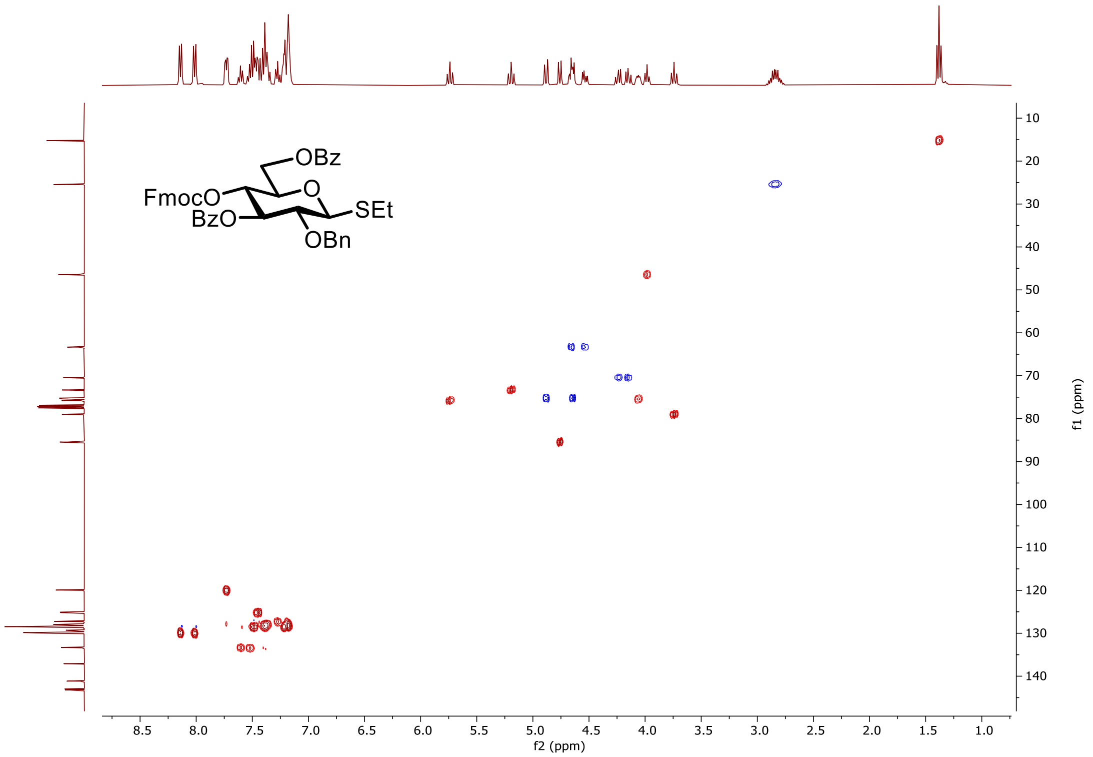

2.18 Synthesis of 29

Ethyl 2-*O*-benzyl-3,6-di-*O*-(4-nitrobenzoyl)-4-*O*-(9-fluorenylmethoxycarbonyl)-1-thio-β-D-glucopyranoside, 29

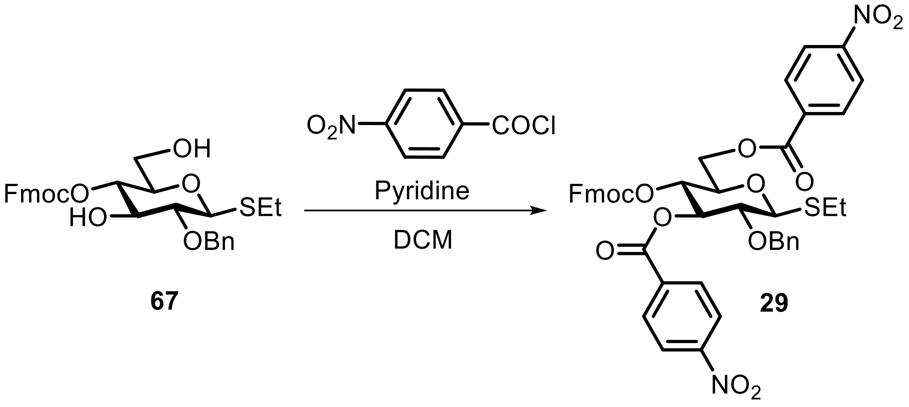

Ethyl 2-*O*-benzyl-4-*O*-(9-fluorenylmethoxycarbonyl)-1-thio-β-D-glucopyranoside **67** (200 mg, 0.37 mmol) was dissolved in anhydrous DCM (3 mL) and pyridine (1 mL) was added. The solution was cooled with an ice bath for 30 min, and 4-nitrobenzoyl chloride (278 mg, 1.5 mmol) was added slowly. The reaction was warmed to room temperature and stirred for 24 h. After the reaction was finished, MeOH (0.2 mL) was added. DCM (10 mL) was added 2 h later, and the organic phase was washed with aqueous citric acid (0.5 M, 10 mL). After extracting the water phase with DCM (5 mL), the organic layers were combined and dried over Na_2SO_4, filtered, and evaporated. The resulting crude product was purified by column chromatography (Hexane : EtOAc = 8:1→Hexane : EtOAc : DCM =6:1:1) to give **29** as a white solid (254 mg, 82%). ^{1}H NMR (400 MHz, $CDCl_3$) δ 8.32 (d, *J* = 8.7 Hz, 2H), 8.25 (d, *J* = 8.6 Hz, 2H), 8.13 (d, *J* = 8.5 Hz, 2H), 7.99 (d, *J* = 8.5 Hz, 2H), 7.71 (dd, *J* = 7.5, 5.4 Hz, 2H), 7.46 – 7.32 (m, 4H), 7.29 – 7.13 (m, 4H), 7.12 – 7.06 (m, 3H), 5.65 (appt, *J* = 9.4 Hz, 1H), 5.16 (appt, *J* = 9.8 Hz, 1H), 4.89 (d, *J* = 11.3 Hz, 1H), 4.77 – 4.64 (m, 2H), 4.60 – 4.50 (m, 2H), 4.18 (qd, *J* = 10.3, 7.4 Hz, 2H), 4.03 (ddd, *J* = 10.3, 5.0, 2.4 Hz, 1H), 3.94 (appt, *J* = 7.4 Hz, 1H), 3.70 (appt, *J* = 9.4 Hz, 1H), 2.84 (p, *J* = 7.4 Hz, 2H), 1.38 (t, *J* = 7.4 Hz, 3H); ^{13}C NMR (101 MHz, $CDCl_3$) δ 164.24, 163.68, 154.25, 150.68, 150.53, 142.78, 142.64, 141.09, 136.98, 134.95, 134.35, 130.93, 130.88, 128.36, 128.29, 128.02, 127.99, 127.95, 127.18, 124.89, 124.86, 123.67, 123.40, 120.13, 120.06, 85.72, 78.82, 76.51, 75.22, 75.09, 72.59, 70.60, 63.64, 46.34, 25.65, 15.16; $[\alpha]_D^{25}$ 31.56 (c = 1, $CHCl_3$); IR (neat) ν_{max} = 1731, 1526, 1264, 717 cm^{-1}; m/z (HRMS$^+$) $[M + K]^+$ 873.1883 ($C_{44}H_{38}N_2O_{13}SK^+$ requires 873.1732).

^{1}H NMR of 29 (400 MHz, $CDCl_3$)

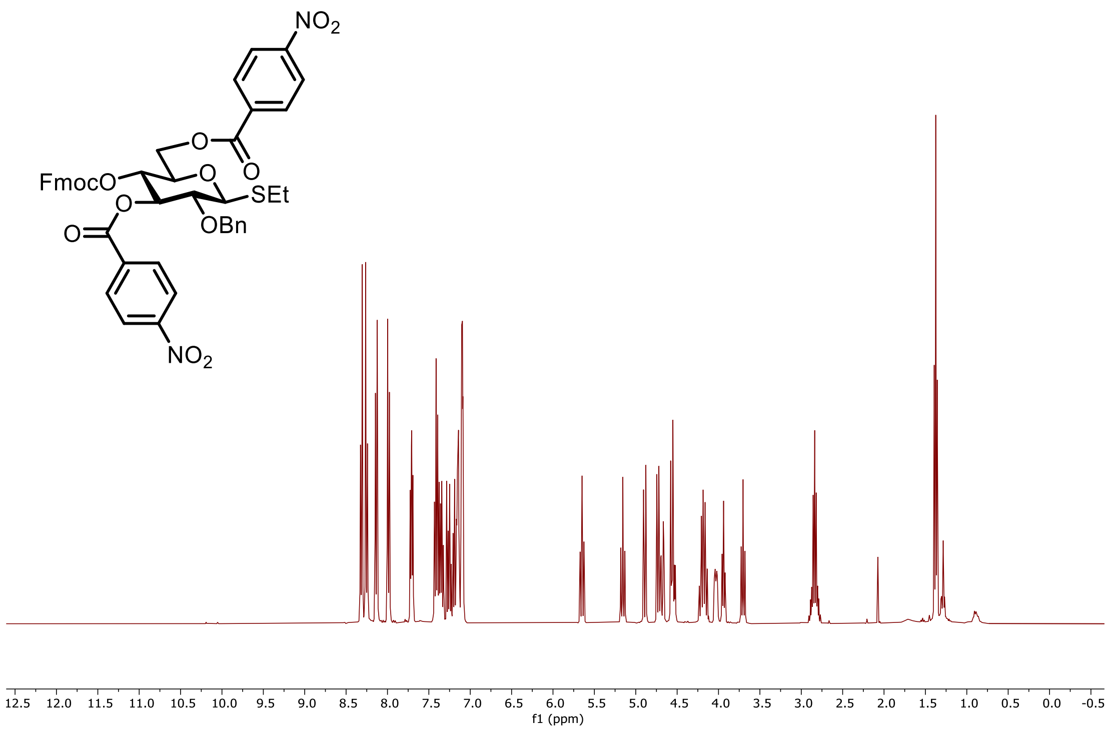

^{13}C NMR of 29 (101 MHz, $CDCl_3$)

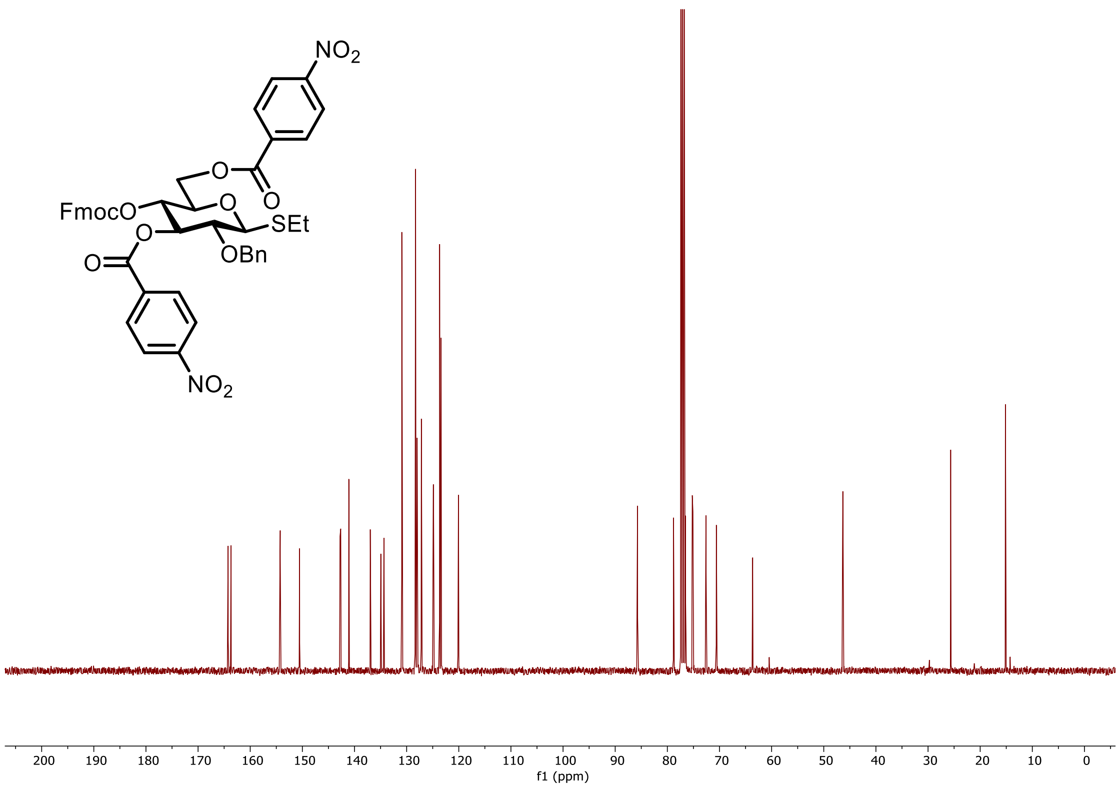

COSY NMR of 29 ($CDCl_3$)

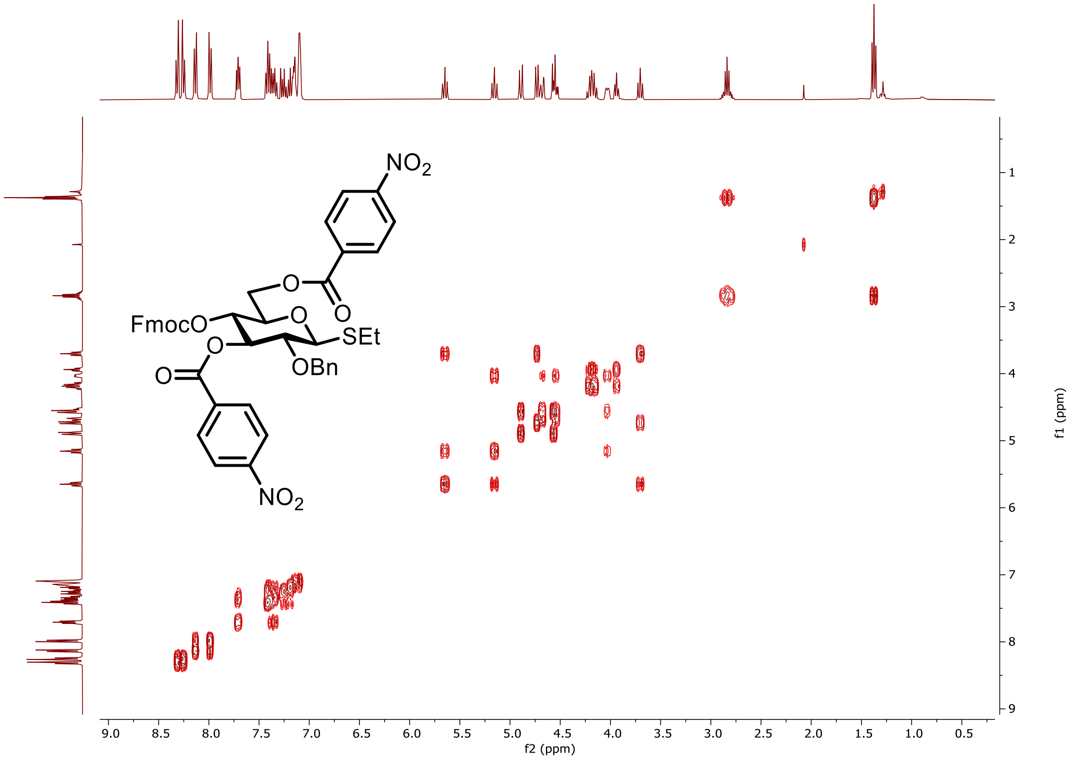

HSQC NMR of 29 ($CDCl_3$)

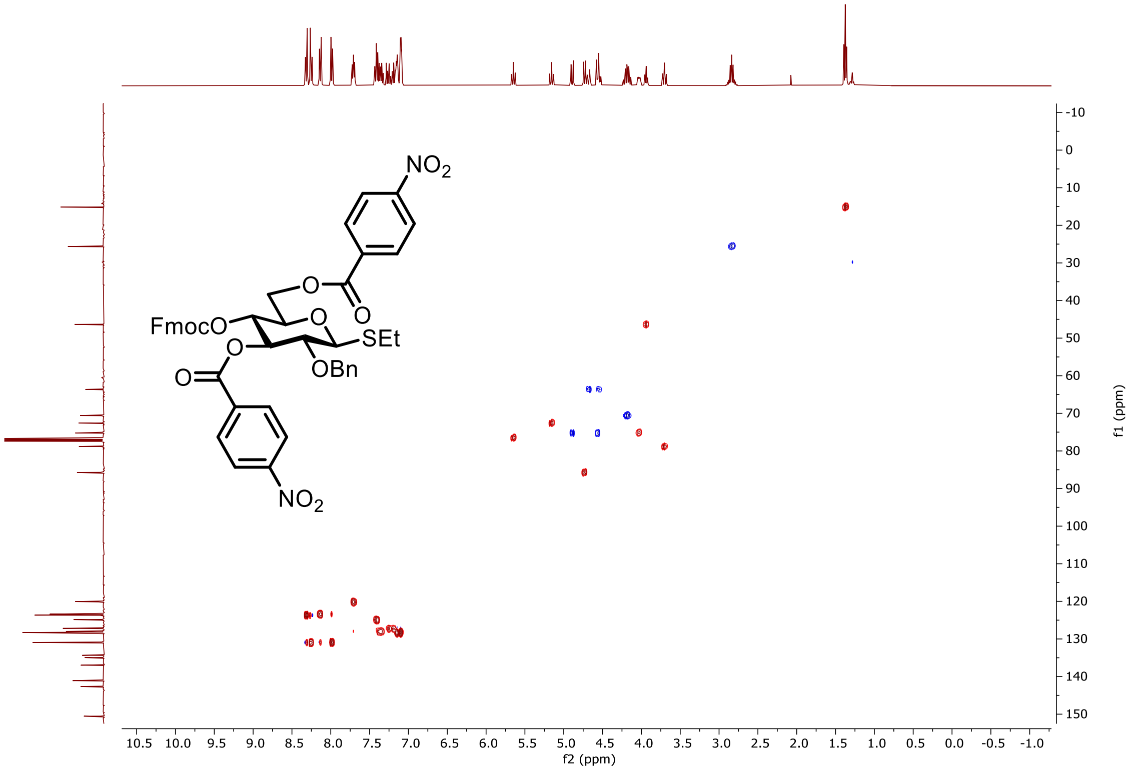

2.19 Synthesis of 30

Ethyl 2-*O*-benzyl-4-*O*-(9-fluorenylmethoxycarbonyl)-6-*O*-levulinoyl-1-thio-β-D-glucopyranoside, 68

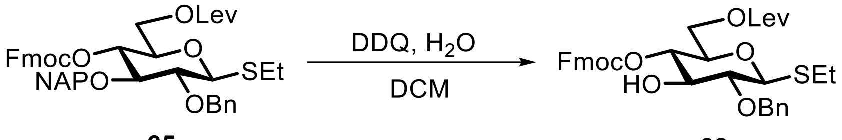

Ethyl 2-*O*-benzyl-3-*O*-(2-naphthylmethyl)-4-*O*-(9-fluorenylmethoxycarbonyl)-6-*O*-levulinoyl-1-thio-β-D-glucopyranoside **65** (5.02 g, 6.5 mmol) was dissolved in DCM (100 mL) and water (5.0 mL) was added. 2,3-Dichloro-5,6-dicyano-1,4-benzoquinone (DDQ) (2.91 g, 13.0 mmol) was added, and reaction was stirred for 6 h at room temperature. DCM (100 mL) was added to dilute the reaction mixture and the solution was washed with $Na_2S_2O_3$ (aq) (5%, 200 mL).The water layer was extracted with DCM (100 mL), and the obtained organic layers were combined and dried over Na_2SO_4, filtered, and evaporated. The resulting crude product was purified by column chromatography (Hexane : EtOAc = 3:1→1:1) to give **68** as a white solid (4.01 g, 97%). ^{1}H NMR (400 MHz, $CDCl_3$) δ 7.79 (dd, *J* = 7.4, 1.1 Hz, 2H), 7.63 (dd, *J* = 7.5, 3.9, 2H), 7.49 – 7.29 (m, 9H), 4.99 (d, *J* = 10.9 Hz, 1H), 4.84 – 4.70 (m, 2H), 4.53 – 4.43 (m, 3H), 4.33 – 4.26 (m, 2H), 4.23 (dd, *J* = 12.2, 2.6 Hz, 1H), 3.82 (appt, *J* = 9.1 Hz, 1H), 3.65 (ddd, *J* = 10.0, 5.3, 2.6 Hz, 1H), 3.39 (dd, *J* = 9.7, 8.7 Hz, 1H), 2.88 – 2.53 (m, 7H), 2.17 (s, 3H), 1.37 (t, *J* = 7.4 Hz, 3H); ^{13}C NMR (101 MHz, $CDCl_3$) δ 206.63, 172.44, 154.81, 143.27, 141.33, 141.30, 137.80, 128.66, 128.38, 128.22, 127.96, 127.28, 125.22, 125.19, 120.13, 120.12, 85.00, 81.22, 77.40, 76.09, 75.47, 75.25, 74.34, 70.33, 62.91, 46.71, 37.87, 29.88, 27.85, 25.28, 15.15; $[\alpha]_D^{25}$ 1.36 (c = 1, $CHCl_3$); IR (neat) ν_{max} = 1746, 1719, 1258, 1029, 742 cm^{-1}; m/z (HRMS$^+$) $[M + NH_4]^+$ 652.2786 ($C_{35}H_{42}NO_9S^+$ requires 652.2575).

^{1}H NMR of 68 (400 MHz, $CDCl_3$)

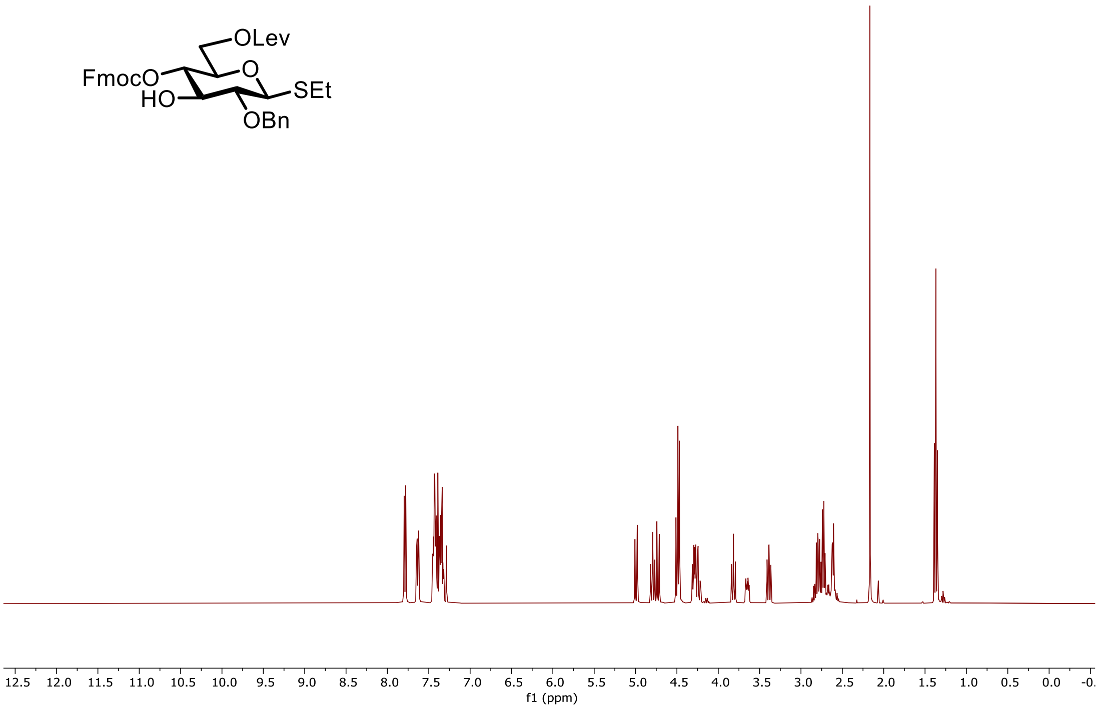

^{13}C NMR of 68 (101 MHz, $CDCl_3$)

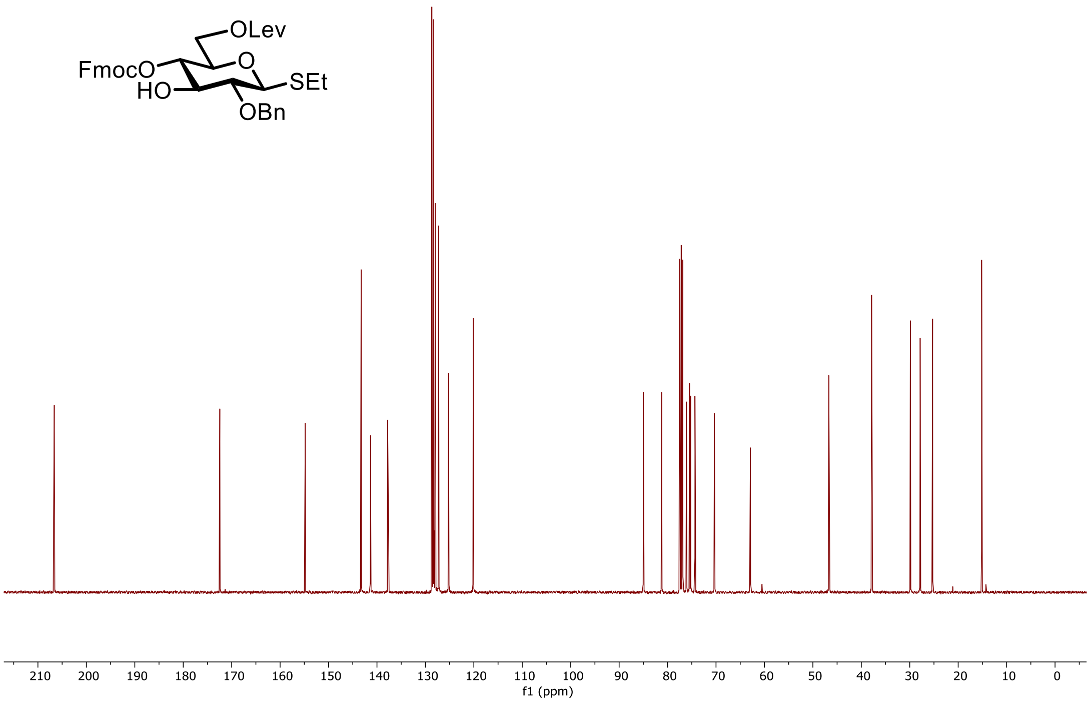

COSY NMR of 68 ($CDCl_3$)

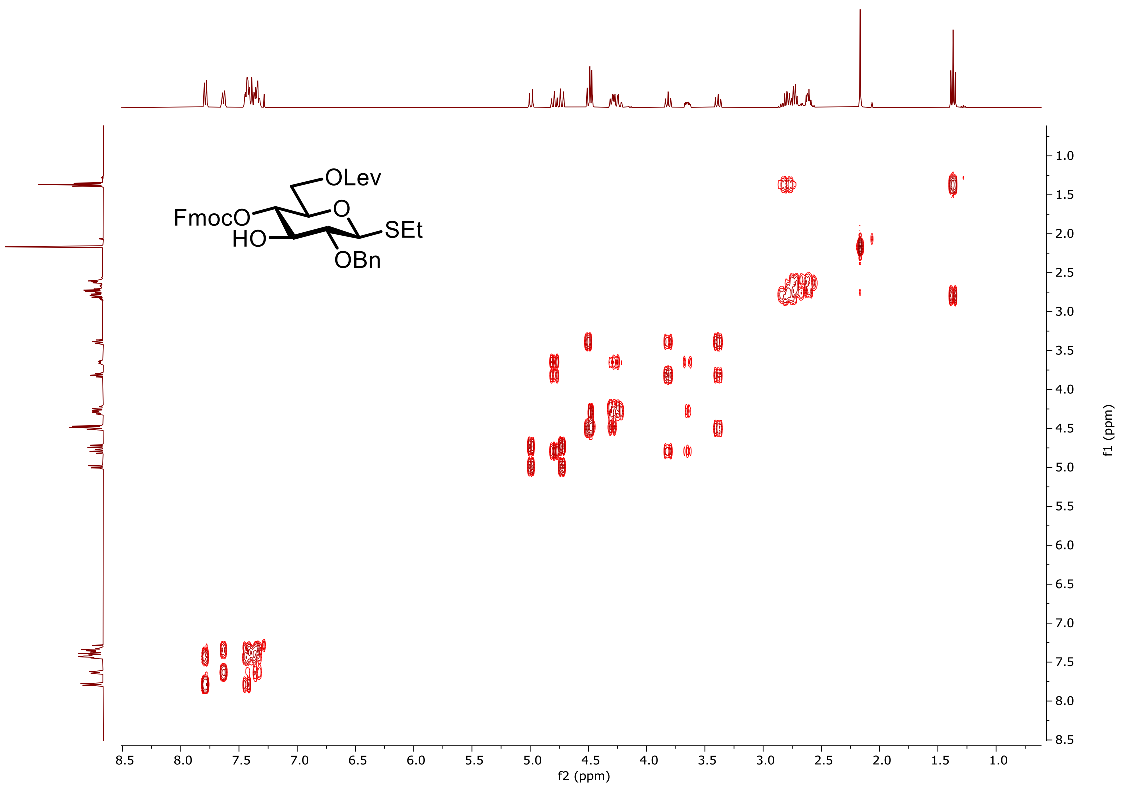

HSQC NMR of 68 ($CDCl_3$)

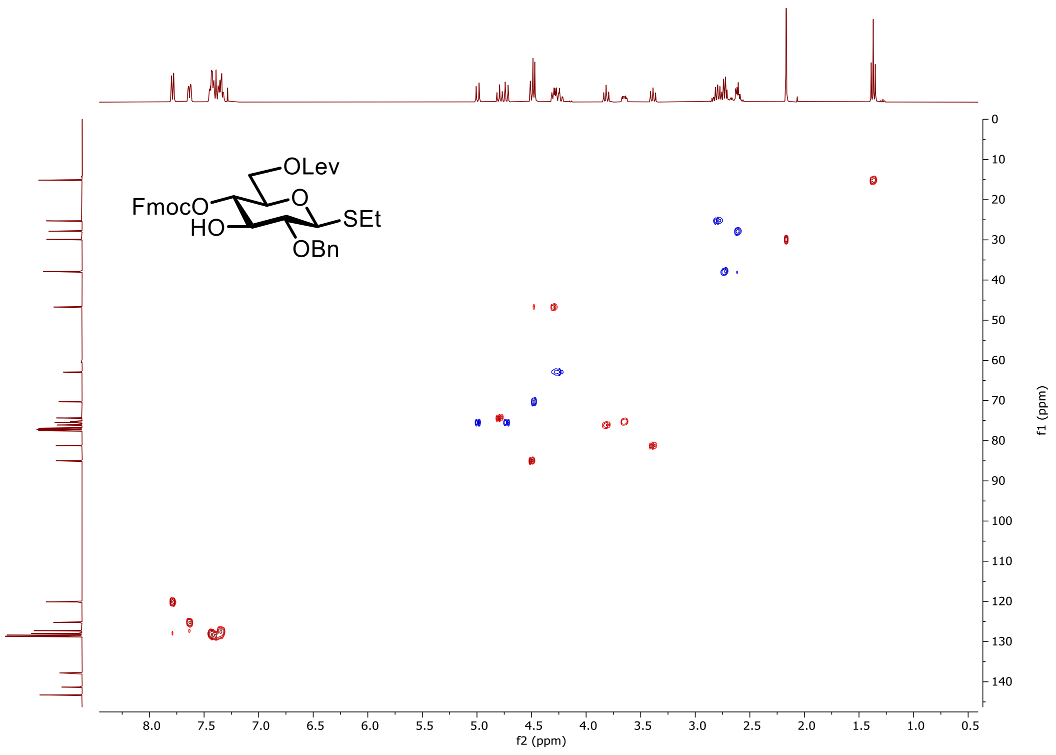

Ethyl 2-*O*-benzyl-3-*O*-benzoyl-4-*O*-(9-fluorenylmethoxycarbonyl)-6-*O*-levulinoyl-1-thio-β-D-glucopyranoside, 30

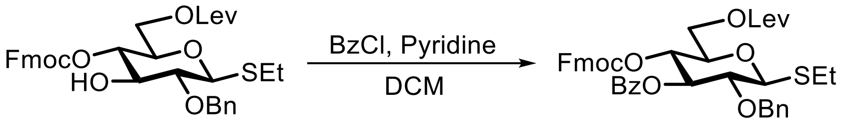

Ethyl 2-*O*-benzyl-4-*O*-(9-fluorenylmethoxycarbonyl)-6-*O*-levulinoyl-1-thio-β-D-glucopyranoside **68** (4.01 g, 6.3 mmol) was dissolved in anhydrous DCM (50 mL) and pyridine (15 mL) was added. The solution was cooled with an ice bath for 30 min, and benzoyl chloride (BzCl, 2.2 mL, 18.9 mmol) was added slowly. The reaction was warmed to room temperature and stirred for 24 h. After the reaction was finished, MeOH (5.0 mL) was added. DCM (200 mL) was added 2 h later, and the organic phase was washed with aqueous citric acid (0.5 M, 200 mL). After extracting the water phase with DCM (50 mL), the organic layers were combined and dried over Na_2SO_4, filtered, and evaporated. The resulting crude product was purified by column chromatography (Hexane : EtOAc = 8:1→Hexane : EtOAc : DCM =3:1:1) to give **30** as a white solid (4.26 g, 91%). ^{1}H NMR (400 MHz, $CDCl_3$) δ 7.99 – 7.93 (m, 2H), 7.72 (dd, *J* = 7.5, 2.6 Hz, 2H), 7.55 – 7.31 (m, 7H), 7.30 – 7.08 (m, 7H), 5.69 – 5.60 (m, 1H), 5.11 – 5.01 (m, 1H), 4.84 (d, *J* = 10.8 Hz, 1H), 4.68 (d, *J* = 9.7 Hz, 1H), 4.60 (d, *J* = 10.8 Hz, 1H), 4.39 – 4.14 (m, 4H), 4.00 (appt, *J* = 7.4 Hz, 1H), 3.88 (ddd, *J* = 10.0, 5.1, 2.6 Hz, 1H), 3.67 (appt, *J* = 9.4 Hz, 1H), 2.93 – 2.72 (m, 4H), 2.72 – 2.56 (m, 2H), 2.19 (s, 3H), 1.39 (t, *J* = 7.4 Hz, 3H); ^{13}C NMR (101 MHz, $CDCl_3$) δ 206.44, 172.41, 165.53, 154.23, 143.21, 142.98, 141.14, 141.07, 137.05, 133.32, 129.90, 129.25, 128.42, 128.37, 128.29, 127.89, 127.82, 127.80, 127.20, 127.19, 125.20, 125.05, 119.92, 85.48, 78.84, 75.73, 75.28, 75.14, 72.79, 70.51, 62.71, 46.42, 37.87, 29.90, 27.82, 25.48, 15.14; $[\alpha]_D^{25}$ -4.52 (c = 1, $CHCl_3$); IR (neat) ν_{max} = 1750, 1724, 1272, 712 cm^{-1}; m/z (HRMS$^+$) $[M + NH_4]^+$ 756.2946 ($C_{42}H_{46}NO_{10}S^+$ requires 756.2837).

^{1}H NMR of 30 (400 MHz, $CDCl_3$)

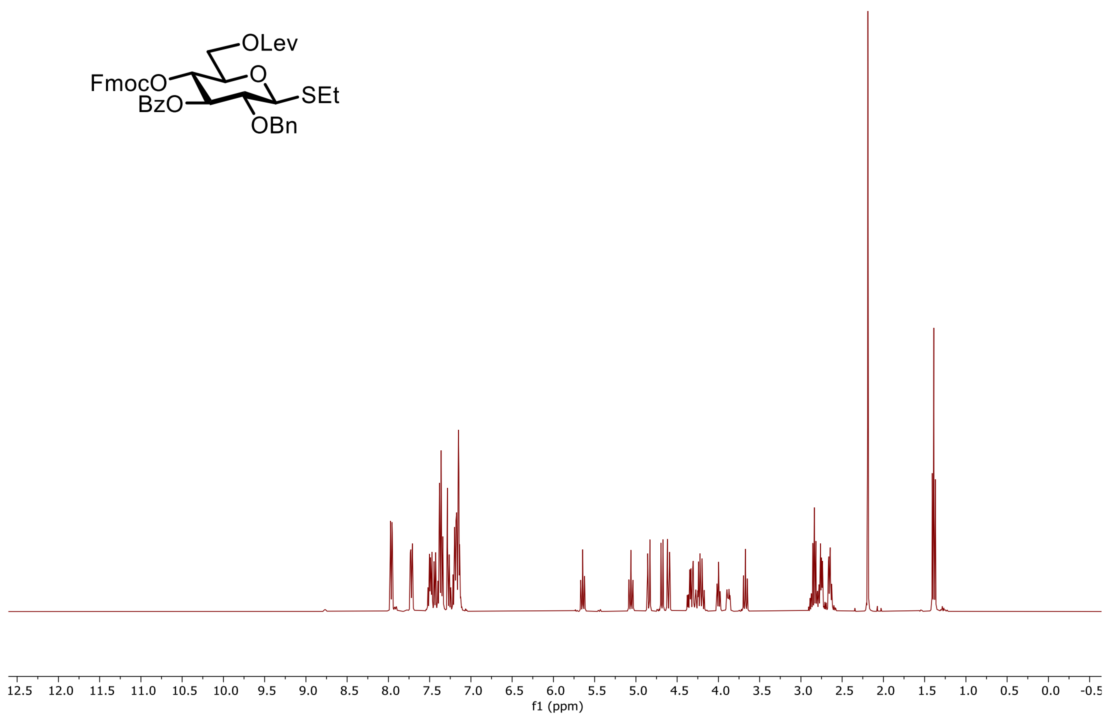

^{13}C NMR of 30 (101 MHz, $CDCl_3$)

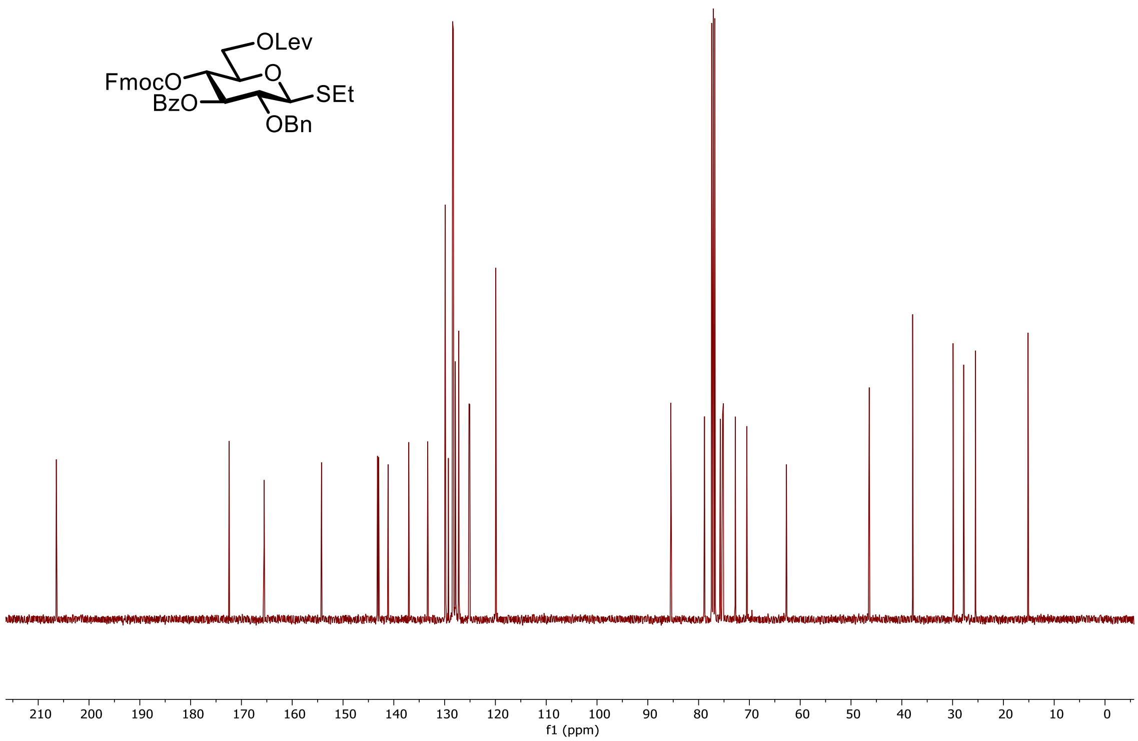

COSY NMR of 30 ($CDCl_3$)

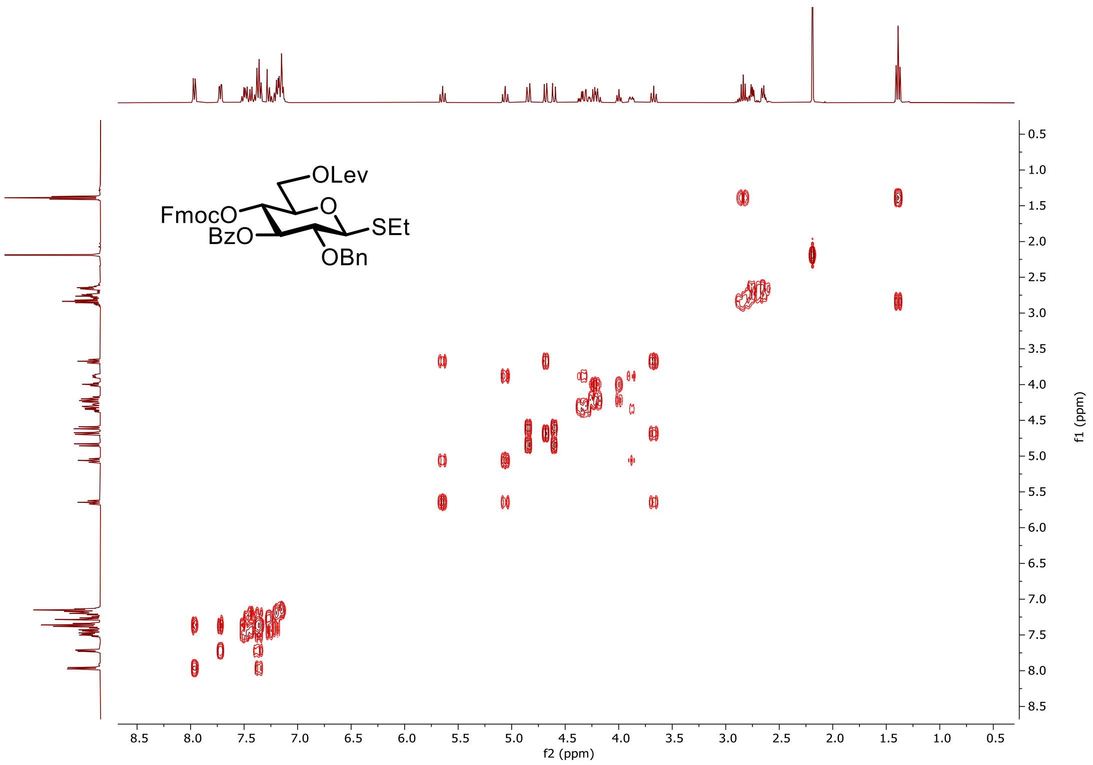

HSQC NMR of 30 ($CDCl_3$)

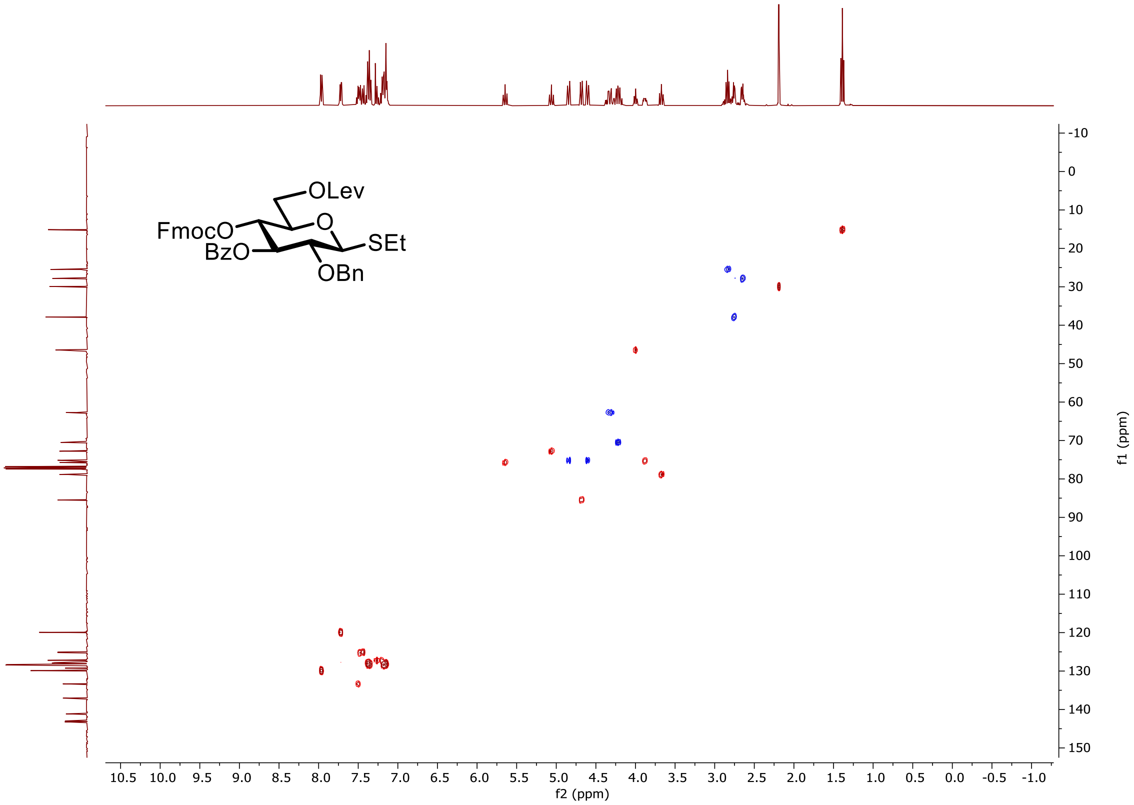

2.20 Synthesis of 31

Ethyl 2,3-di-*O*-benzyl-6-deoxy-6-fluoro-1-thio-β-D-glucopyranoside, 69

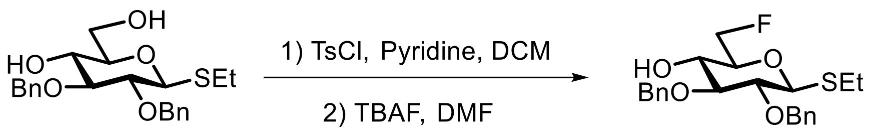

53 **69**

53 was prepared according to previously established procedures.[2]

Ethyl 2,3-di-*O*-benzyl-1-thio-β-D-glucopyranoside **53** (1.00 g, 2.5 mmol) was dissolved in anhydrous DCM (35 mL) and pyridine (5.0 mL) was added. The solution was cooled with an ice bath for 30 min, and 4-toluenesulfonyl chloride (TsCl, 614 mg, 3.2 mmol) was added slowly. The reaction was warmed to room temperature and stirred for 6 h. MeOH (1.0 mL) was added to quench the reaction, DCM (40 mL) was added 20 min later, and the organic phase was washed with aqueous citric acid (0.5 M, 200 mL). After extracting the water phase with DCM (50 mL), the organic layers were combined and dried over Na_2SO_4, filtered, and evaporated. The resulting crude product was directly loaded and purified quickly by column chromatography (Hexane : EtOAc = 8:1→2:1) to give the tosylated thioglycoside as a white solid (1.32 g, 94%). The obtained compound was dissolve in DMF (20 mL), and tetrabutylammonium fluoride (TBAF, 7 mL (1M in THF), 7.0 mmol) was added. The system was charged with argon and heated up to 65 °C for 18 h. On the next day, the reaction was diluted with water (100 mL) and extracted with ethyl acetate (100 mL). The organic layer was washed with water (50 mL) and dried over Na_2SO_4, filtered, and evaporated. The resulting crude product was purified by column chromatography (Hexane : EtOAc = 4:1→3:1) to give **69** as a white solid (670 mg, 66% over two steps). ^{1}H NMR (400 MHz, $CDCl_3$) δ 7.51 – 7.31 (m, 10H), 5.01 (dd, *J* = 10.9, 8.0 Hz, 2H), 4.81 – 4.69 (m, 2.5H), 4.69 – 4.59 (m, 1H), 4.57 – 4.49 (m, 1.5H), 3.62 – 3.41 (m, 4H), 2.81 (qq, *J* = 12.6, 7.4 Hz, 2H), 2.17 (br. s, 1H), 1.36 (t, *J* = 7.4 Hz, 3H); ^{13}C NMR (101 MHz, $CDCl_3$) δ 138.31, 137.74, 128.83, 128.52, 128.43, 128.20, 128.06, 127.98, 85.87, 85.23, 82.24 (d, *J* = 174.2 Hz), 81.38, 78.06 (d, *J* = 18.4 Hz), 75.46, 75.41, 68.90 (d, *J* = 7.4 Hz), 25.16, 15.11; ^{19}F NMR (564 MHz, $CDCl_3$) δ -233.37 (td, *J* = 47.5, 21.4 Hz); $[\alpha]_D^{25}$ -31.51 (c = 1, $CHCl_3$); IR (neat) ν_{max} = 3360, 1053, 693 cm^{-1}; m/z (HRMS$^+$) $[M + K]^+$ 445.1226 ($C_{22}H_{27}FO_4SK^+$ requires 445.1246).

^{1}H NMR of 69 (400 MHz, $CDCl_3$)

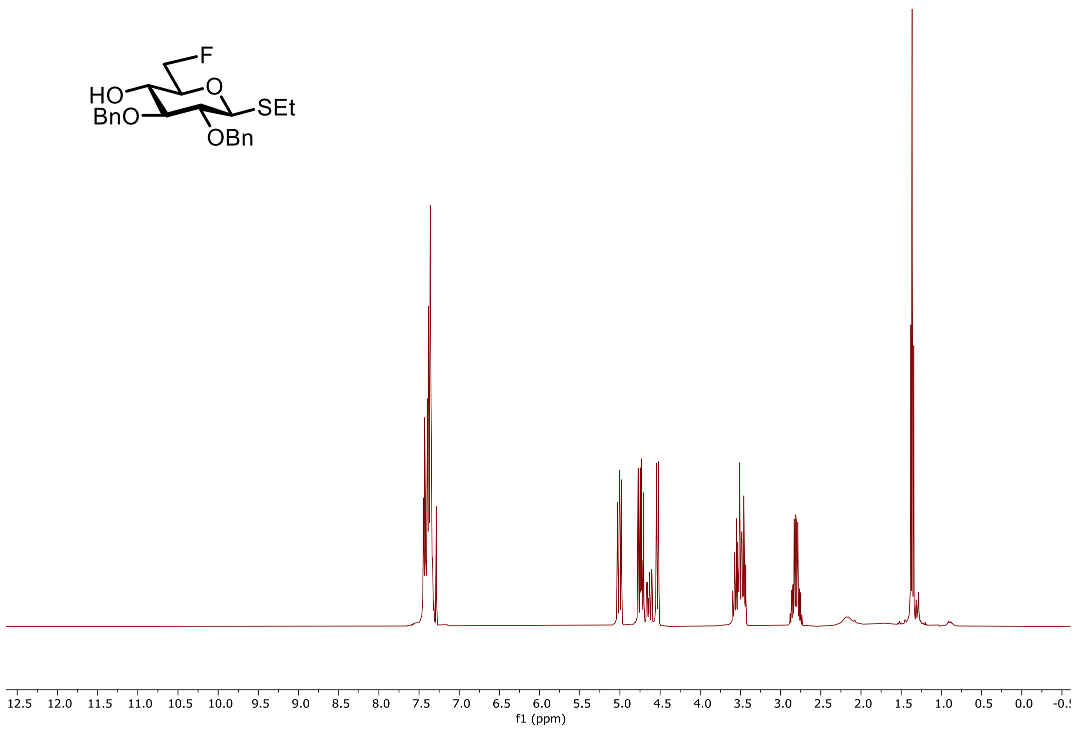

^{13}C NMR of 69 (101 MHz, $CDCl_3$)

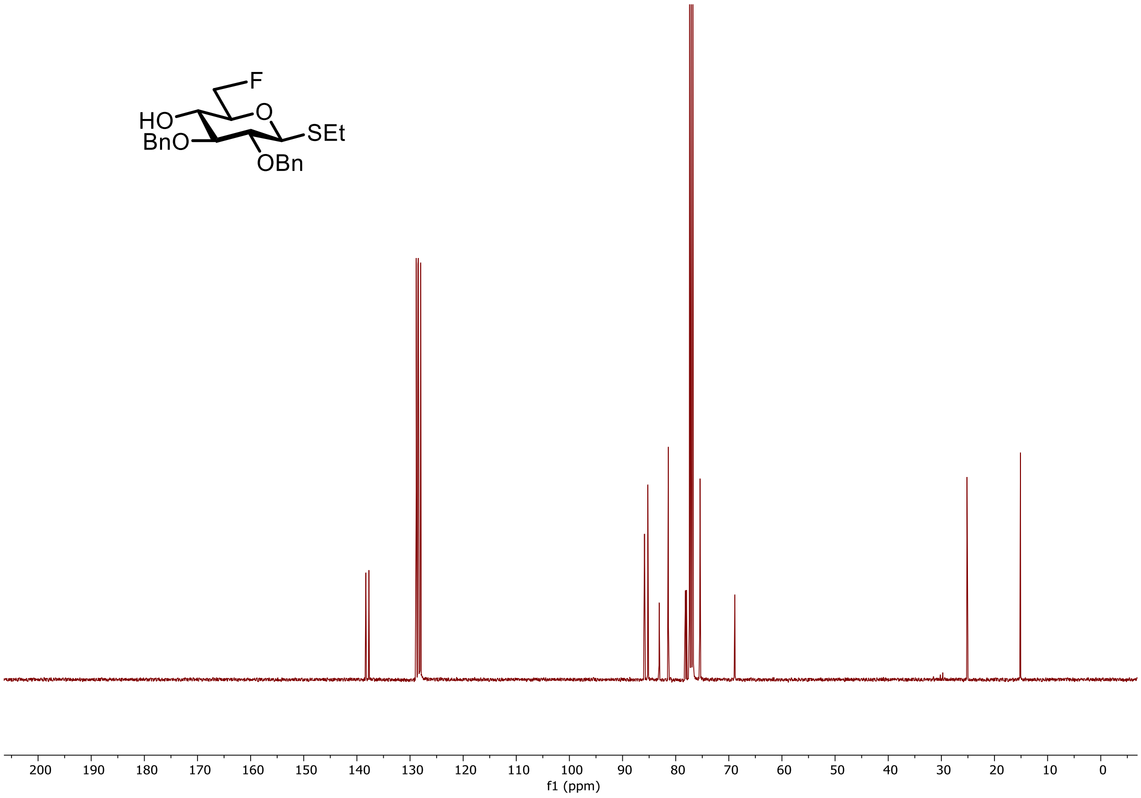

^{19}F NMR of 69 (564 MHz, $CDCl_3$)

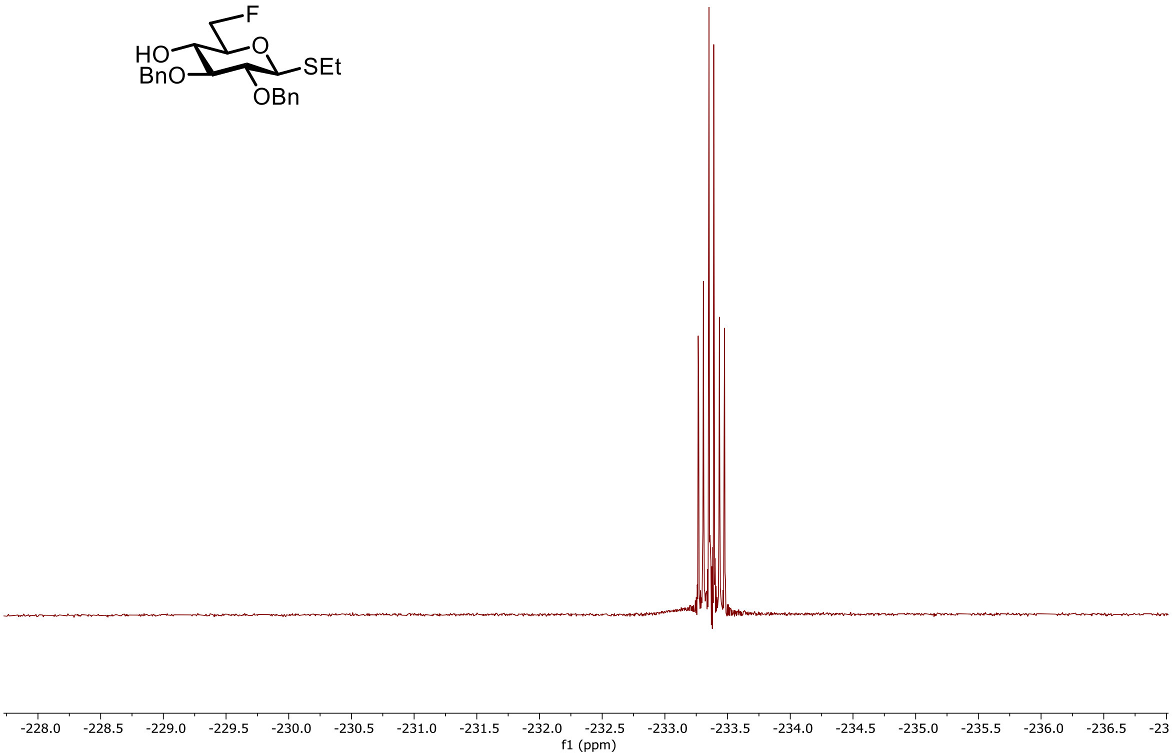

COSY NMR of 69 ($CDCl_3$)

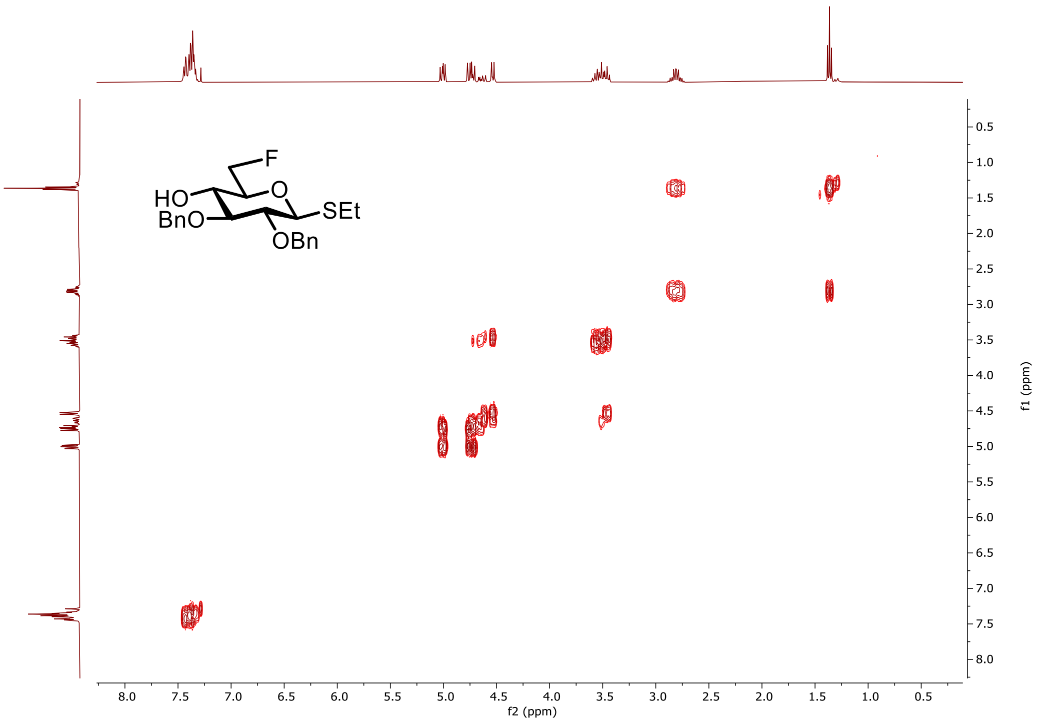

HSQC NMR of 69 ($CDCl_3$)

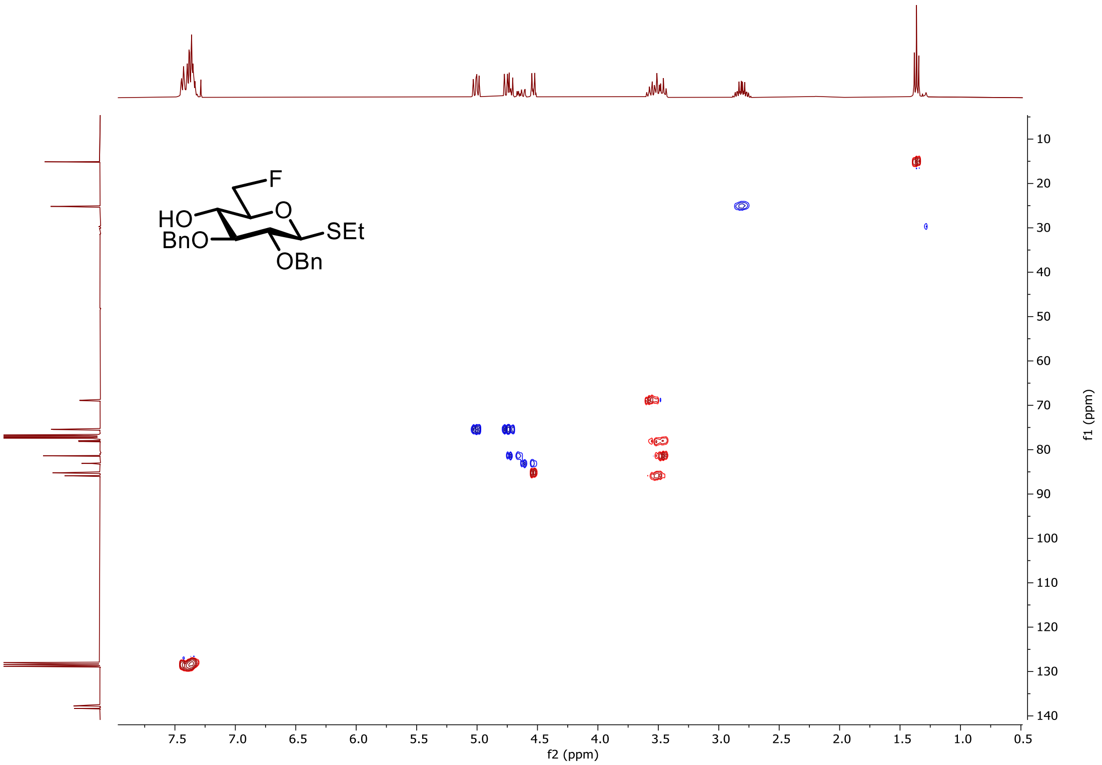

Ethyl 2,3-di-*O*-benzyl-4-*O*-(9-fluorenylmethoxycarbonyl)-6-deoxy-6-fluoro-1-thio-β-D-glucopyranoside, 31

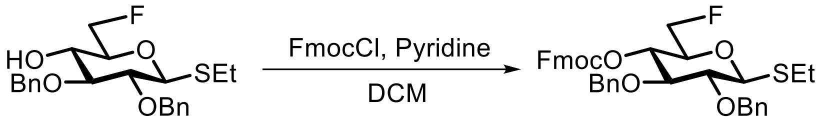

69 **31**

Ethyl 2,3-di-*O*-benzyl-6-deoxy-6-fluoro-1-thio-β-D-glucopyranoside **69** (670 mg, 1.6 mmol) was dissolved in anhydrous DCM (20 mL) and pyridine (5 mL) was added. The solution was cooled with an ice bath for 30 min, and fluorenylmethyloxycarbonyl chloride (FmocCl, 1.06 g, 4.1 mmol) was added slowly. The reaction was warmed to room temperature and stirred for 6 h. Upon completion, DCM (80 mL) was added and the organic phase was washed with aqueous citric acid (0.5 M, 80 mL). After extracting the water phase with DCM (40 mL), the organic layers were combined and dried over Na_2SO_4, filtered, and evaporated. The resulting crude product was purified by column chromatography (Hexane : EtOAc = 8:1→4:1) to give **31** as a white solid (800 mg, 79%). ^{1}H NMR (600 MHz, $CDCl_3$) δ 7.81 – 7.76 (m, 2H), 7.61 (d, *J* = 7.5 Hz, 1H), 7.58 (d, *J* = 7.5 Hz, 1H), 7.46 – 7.27 (m, 9H), 7.25 (s, 5H), 4.94 (d, *J* = 10.1 Hz, 1H), 4.90 – 4.83 (m, 2H), 4.75 (d, *J* = 10.1 Hz, 1H), 4.71 (d, *J* = 11.2 Hz, 1H), 4.57 – 4.48 (m, 3H), 4.44 (d, *J* = 3.9 Hz, 1H), 4.39 (ddd, *J* = 10.7, 7.0, 1.2 Hz, 1H), 4.21 (appt, *J* = 7.0 Hz, 1H), 3.76 (appt, *J* = 9.1 Hz, 1H), 3.69 (ddt, *J* = 20.2, 10.1, 3.7 Hz, 1H), 3.52 (appt, *J* = 9.2 Hz, 1H), 2.88 – 2.74 (m, 2H), 1.37 (t, *J* = 7.4 Hz, 3H); ^{13}C NMR (151 MHz, $CDCl_3$) δ 154.36, 143.17, 143.06, 141.34, 141.32, 137.88, 137.65, 128.48, 128.42, 128.36, 128.04, 127.98, 127.76, 127.72, 127.22, 125.08, 124.94, 120.15, 120.12, 85.21, 83.60, 81.70 (d, *J* = 174.8 Hz), 81.12, 76.34 (d, *J* = 19.5 Hz), 75.66, 75.63, 73.98 (d, *J* = 6.1 Hz), 70.17, 46.77, 25.09, 15.11; ^{19}F NMR (564 MHz, $CDCl_3$) δ -230.82 (td, *J* = 46.7, 20.2 Hz); $[\alpha]_D^{25}$ 1.10 (c = 1, $CHCl_3$); IR (neat) ν_{max} = 1753, 1452, 1251, 738 cm^{-1}; m/z (HRMS$^+$) [M + K]$^+$ 667.1961 ($C_{37}H_{37}FO_6SK^+$ requires 667.1926).

^{1}H NMR of 31 (600 MHz, $CDCl_3$)

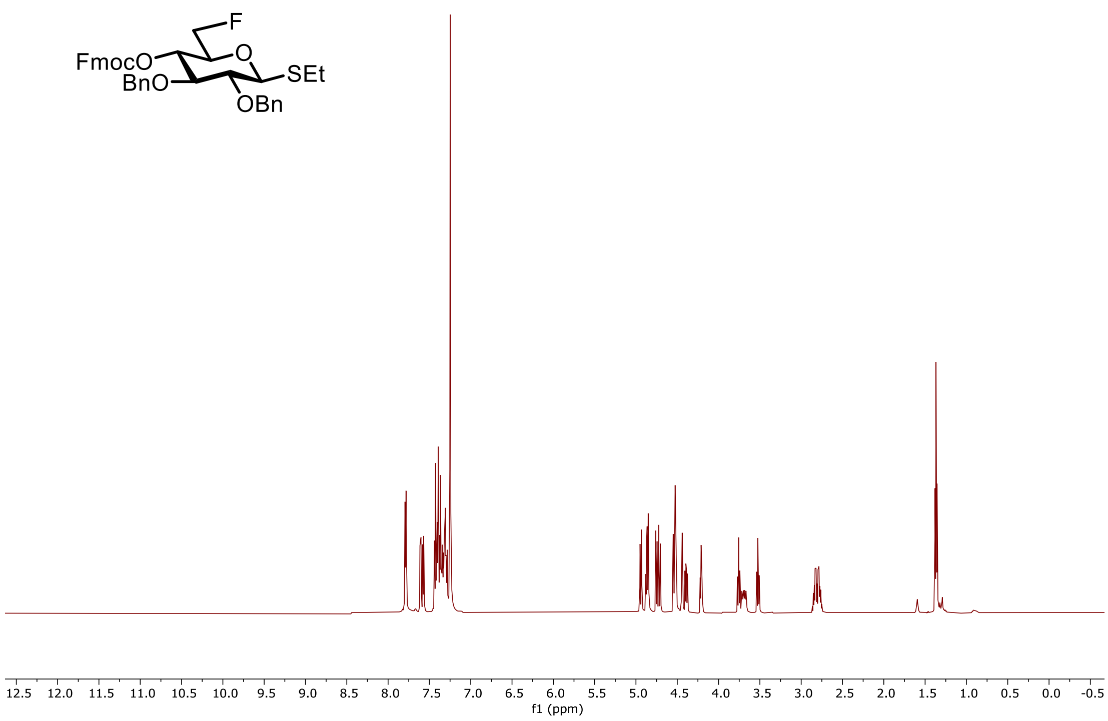

^{13}C NMR of 31 (151 MHz, $CDCl_3$)

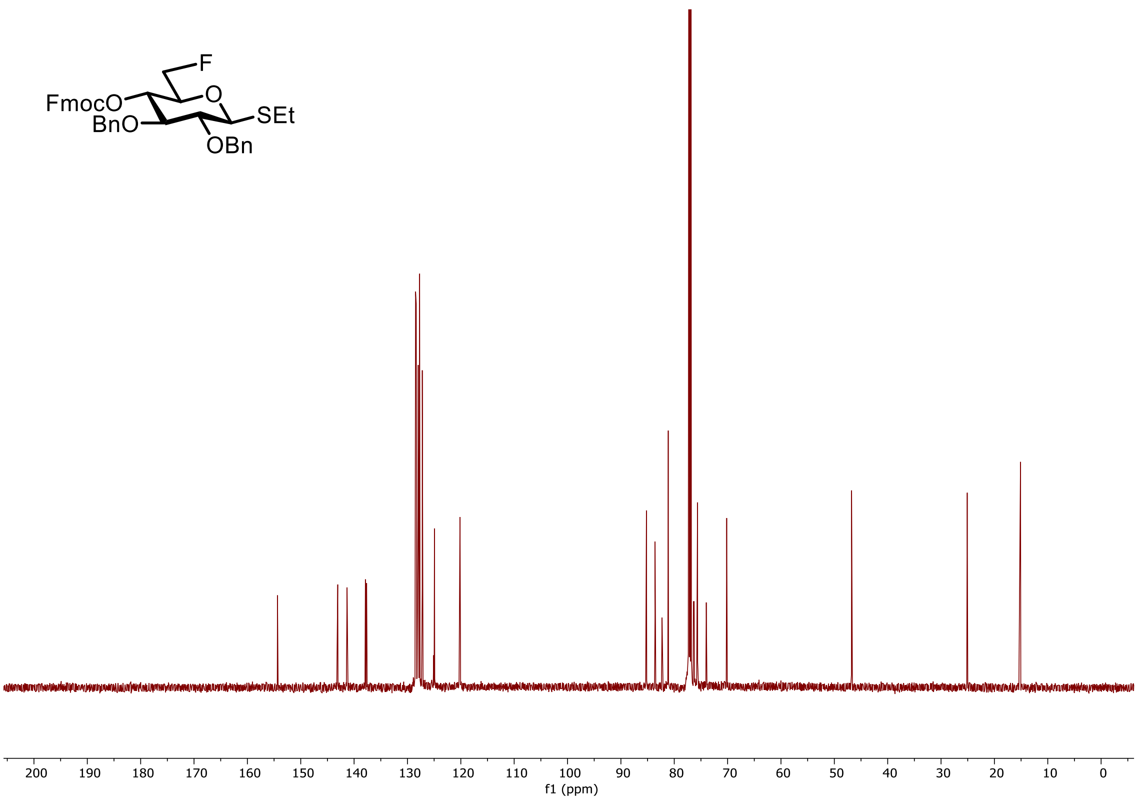

^{19}F NMR of 31 (564 MHz, CDCl$_3$)

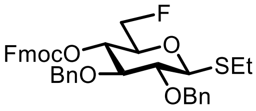

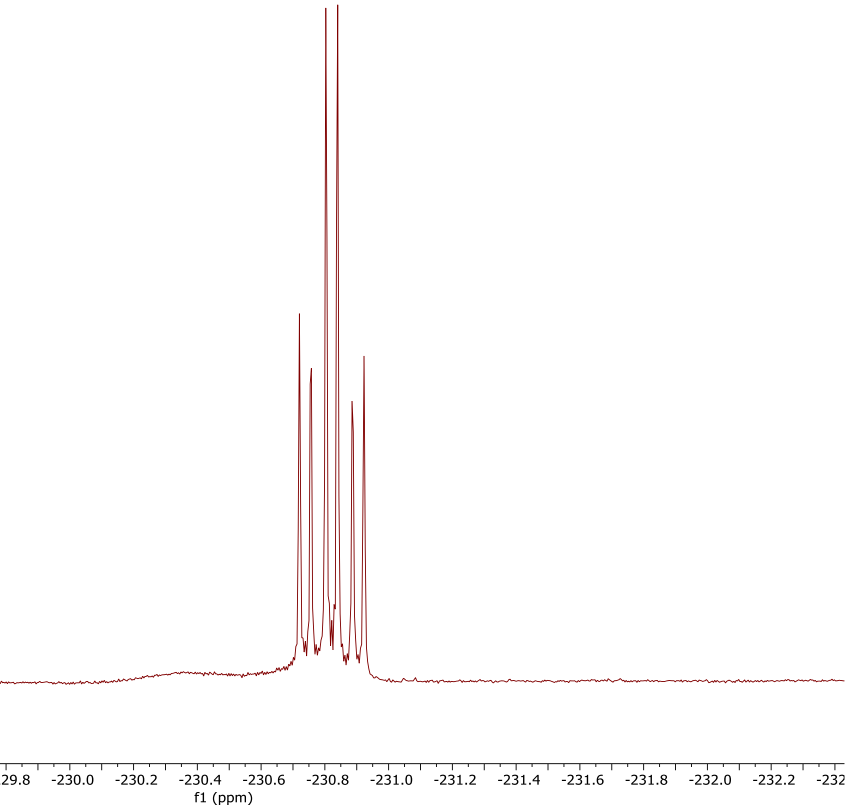

COSY NMR of 31 ($CDCl_3$)

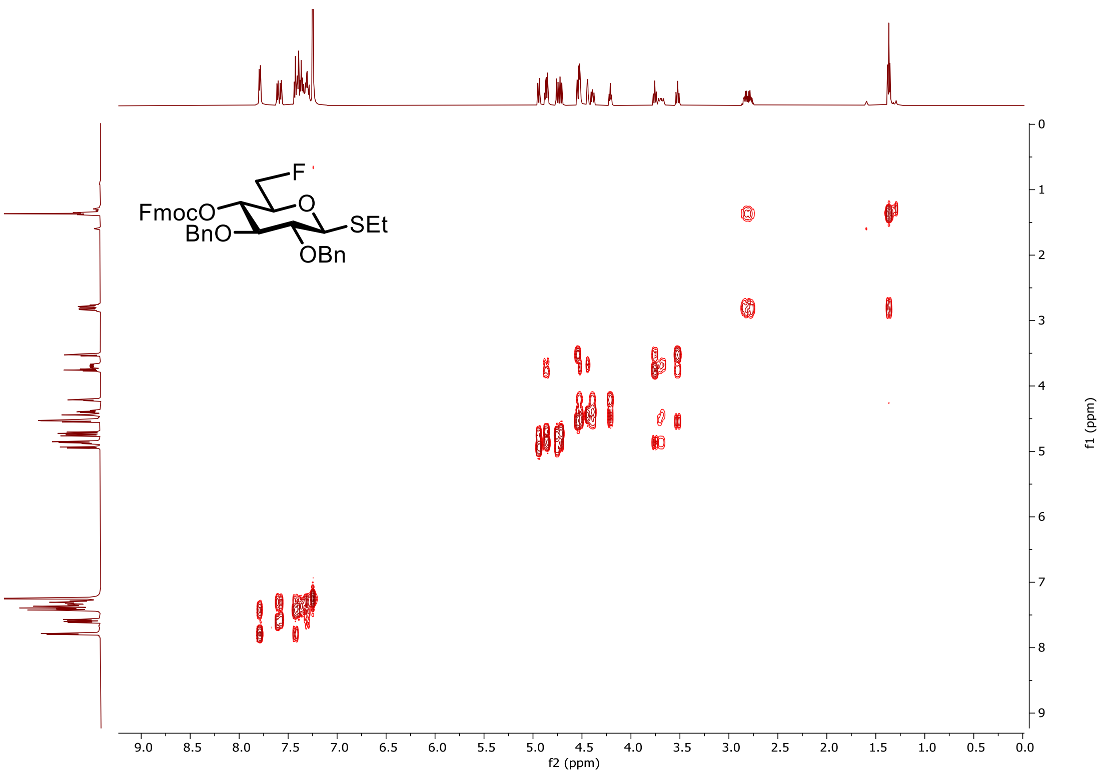

HSQC NMR of 31 ($CDCl_3$)

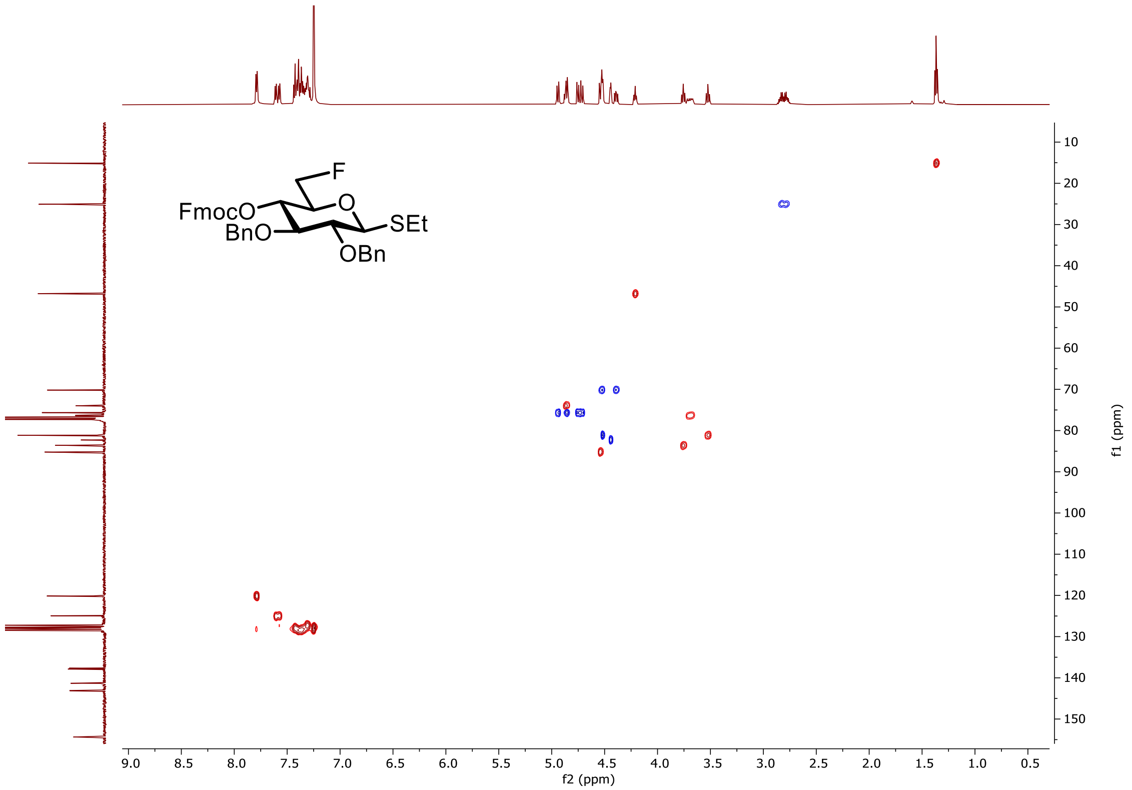

2.21 Synthesis of 32

Ethyl 2,3-di-*O*-benzyl-4,6-*O*-naphthylmethylene-1-thio-β-D-glucopyranoside, 70

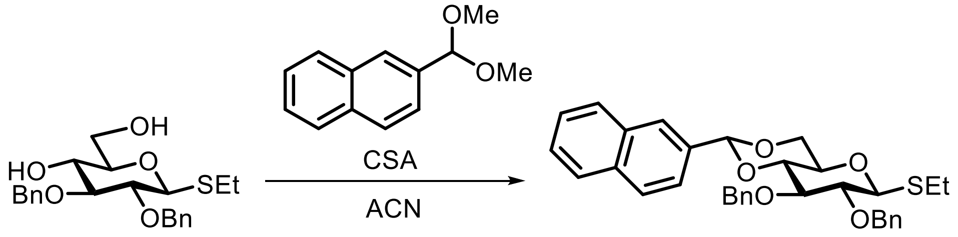

53 **70**

53 was prepared according to previously established procedures. [2]

Ethyl 2,3-di-*O*-benzyl-1-thio-β-D-glucopyranoside **53** (680 mg, 1.7 mmol) was dissolved in acetonitrile (10 mL), 2-(dimethoxymethyl)naphthalene (1.03 g, 5.1 mmol) and camphorsulfonic acid (CSA, 40 mg, 0.17 mmol) were added. The reaction was stirred for 2 h at room temperature, and TEA (0.1 mL) was added to stop the reaction. The solvent was evaporated and the resulting crude product was purified by column chromatography (Hexane : EtOAc = 1:0→6:1) to give **70** as a white solid (877 mg, 95%). ^{1}H NMR (600 MHz, $CDCl_3$) δ 8.00 (d, *J* = 1.6 Hz, 1H), 7.91 – 7.85 (m, 3H), 7.61 (dd, *J* = 8.5, 1.7 Hz, 1H), 7.56 – 7.49 (m, 2H), 7.44 – 7.27 (m, 10H), 5.76 (s, 1H), 5.00 (d, *J* = 11.3 Hz, 1H), 4.93 (d, *J* = 10.2 Hz, 1H), 4.86 (dd, *J* = 10.7, 2.3 Hz, 2H), 4.62 (d, *J* = 9.8 Hz, 1H), 4.44 (dd, *J* = 10.5, 5.0 Hz, 1H), 3.91 – 3.84 (m, 2H), 3.80 (appt, *J* = 9.3 Hz, 1H), 3.57 – 3.49 (m, 2H), 2.87 – 2.73 (m, 2H), 1.36 (t, *J* = 7.4 Hz, 3H); ^{13}C NMR (151 MHz, $CDCl_3$) δ 138.40, 137.96, 134.65, 133.61, 132.92, 128.41, 128.36, 128.12, 128.06, 127.92, 127.75, 127.72, 126.45, 126.21, 125.46, 123.68, 101.32, 85.90, 82.81, 81.72, 81.33, 76.02, 75.27, 70.28, 68.84, 25.23, 15.15; $[\alpha]_D^{25}$ -54.32 (c = 1, $CHCl_3$); IR (neat) ν_{max} = 2869, 1086, 697 cm^{-1}; m/z (HRMS$^+$) $[M + K]^+$ 581.1788 ($C_{33}H_{34}O_5SK^+$ requires 581.1759).

^{1}H NMR of 70 (600 MHz, $CDCl_3$)

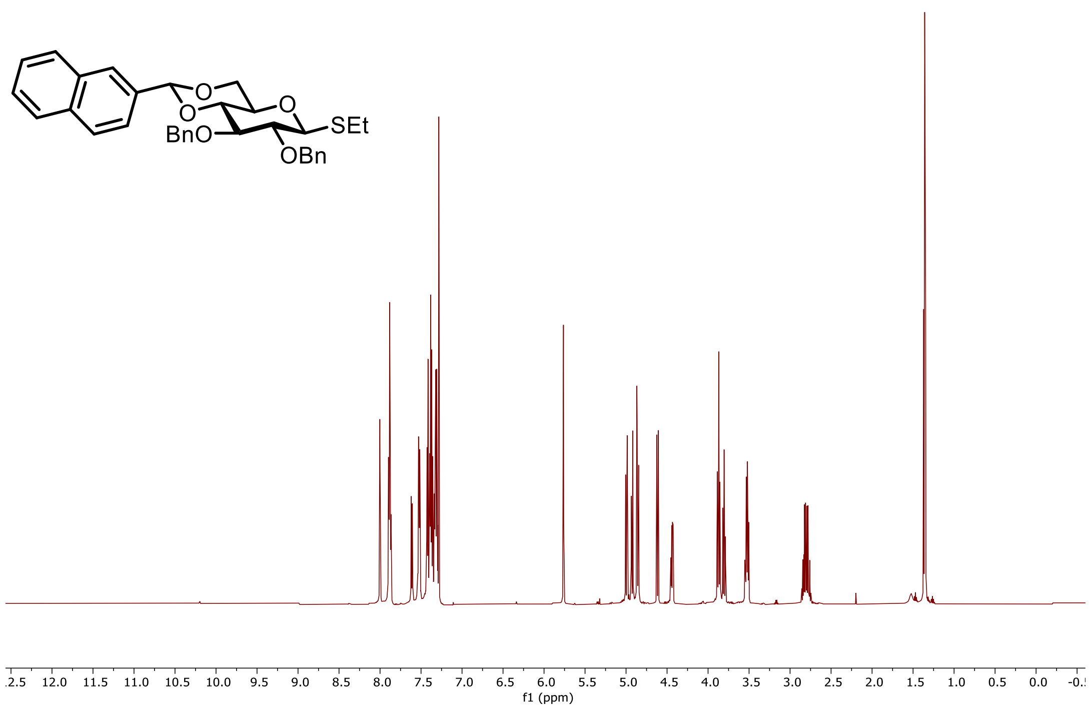

^{13}C NMR of 70 (151 MHz, $CDCl_3$)

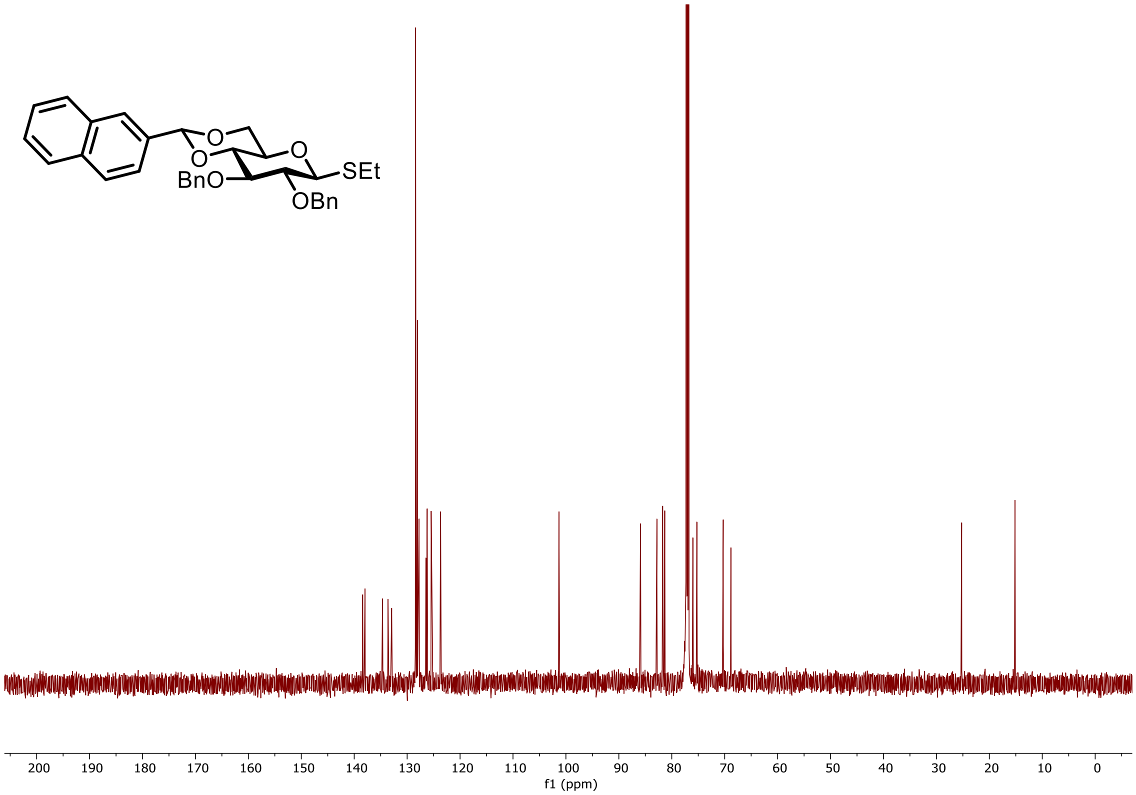

COSY NMR of 70 ($CDCl_3$)

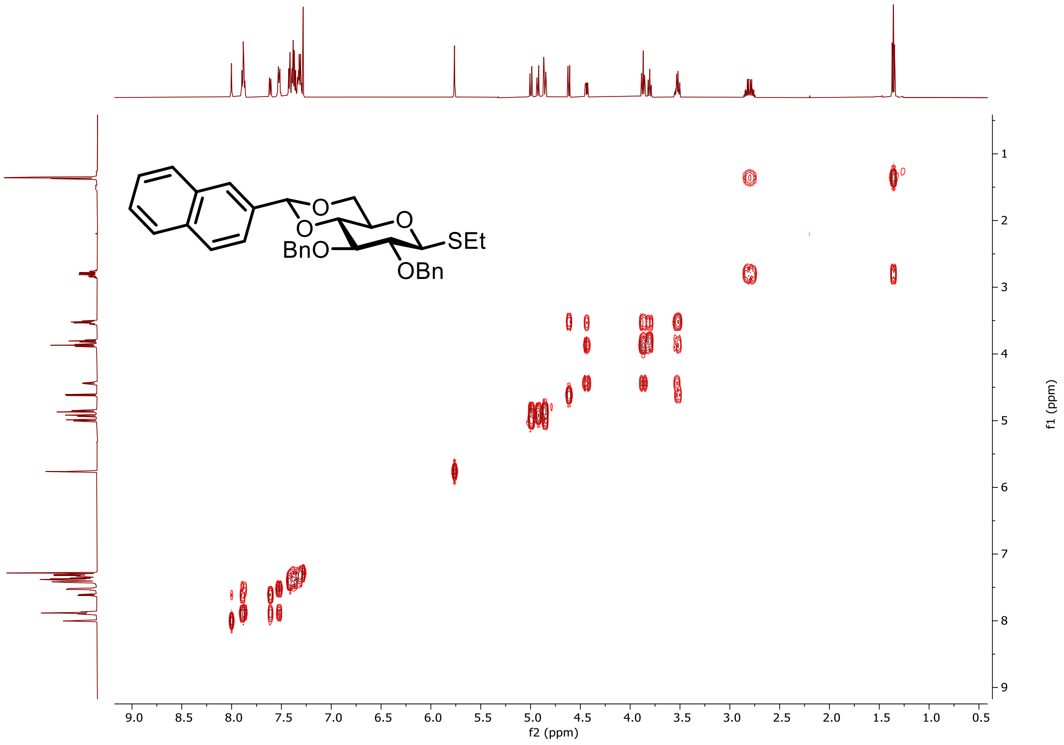

HSQC NMR of 70 ($CDCl_3$)

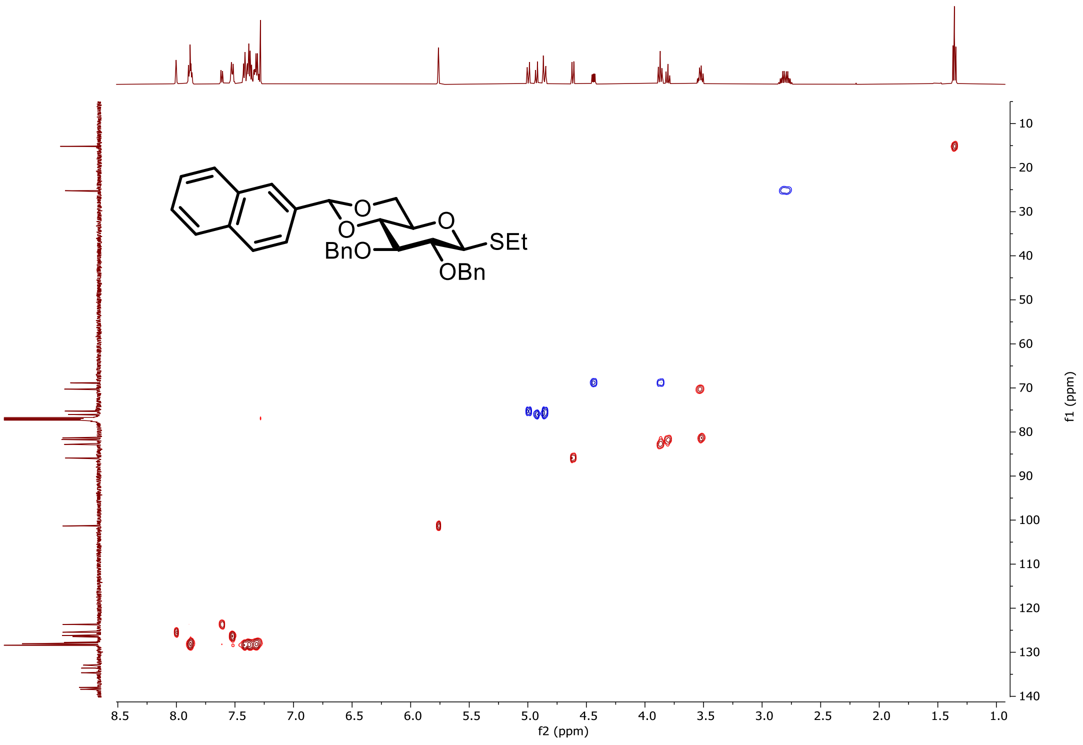

Ethyl 2,3-di-*O*-benzyl-4-*O*-(2-naphthylmethyl)-1-thio-β-D-glucopyranoside, 71

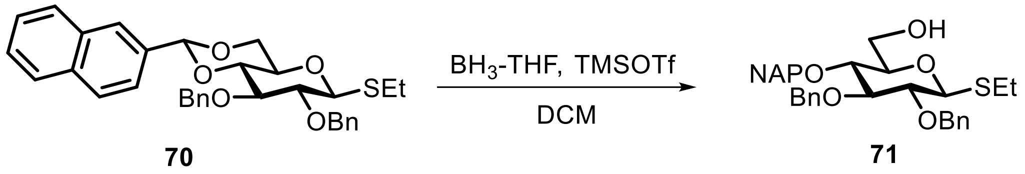

Ethyl 2,3-di-*O*-benzyl-4,6-*O*-naphthylmethylene-1-thio-β-D-glucopyranoside **70** (877 mg, 1.6 mmol) was dissolved in anhydrous DCM (10 mL), the system was charged with argon and BH_3-THF (3.2 mL (1M in THF), 3.2 mmol) was added. Trimethylsilyl trifluoromethanesulfonate (TMSOTf, 43 µL, 0.24 mmol) was slowly added and the reaction was stirred for 3 h at room temperature. Upon completion, the reaction was carefully quenched (ice bath) by addition of saturated aq. $NaHCO_3$ solution (10 mL). The water layer was extracted with DCM (5 mL) and the organic layers were combined and dried over Na_2SO_4, filtered, and evaporated. The resulting crude product was purified by column chromatography (Hexane : EtOAc = 8:1→3:1) to give **71** as a white solid (645 mg, 74%). ^{1}H NMR (600 MHz, $CDCl_3$) δ 7.87 – 7.77 (m, 3H), 7.72 (s, 1H), 7.54 – 7.47 (m, 2H), 7.45 – 7.38 (m, 3H), 7.37 – 7.30 (m, 8H), 5.04 (d, *J* = 11.1 Hz, 1H), 4.99 (d, *J* = 11.0 Hz, 1H), 4.95 (d, *J* = 10.1 Hz, 1H), 4.92 (d, *J* = 11.0 Hz, 1H), 4.85 (d, *J* = 11.1 Hz, 1H), 4.79 (d, *J* = 10.2 Hz, 1H), 4.55 (d, *J* = 9.8 Hz, 1H), 3.94 (dd, *J* = 12.0, 2.7 Hz, 1H), 3.80 – 3.72 (m, 2H), 3.67 (appt, *J* = 9.4 Hz, 1H), 3.50 – 3.41 (m, 2H), 2.79 (dddd, *J* = 20.1, 12.6, 7.5, 5.2 Hz, 2H), 1.85 (br. s, 1H), 1.36 (t, *J* = 7.4 Hz, 3H); ^{13}C NMR (151 MHz, $CDCl_3$) δ 138.43, 137.89, 135.33, 133.26, 133.04, 128.51, 128.46, 128.34, 127.96, 127.94, 127.80, 127.75, 127.72, 126.87, 126.18, 126.05, 125.96, 86.50, 85.30, 81.80, 79.30, 77.71, 75.82, 75.62, 75.24, 62.23, 25.25, 15.20; $[\alpha]_D^{25}$ -13.10 (c = 1, $CHCl_3$); IR (neat) ν_{max} = 2907, 1081, 1067, 696 cm^{-1}; m/z (HRMS$^+$) [M + Na]$^+$ 567.2191 ($C_{33}H_{36}O_5SNa^+$ requires 567.2176).

^{1}H NMR of 71 (600 MHz, $CDCl_3$)

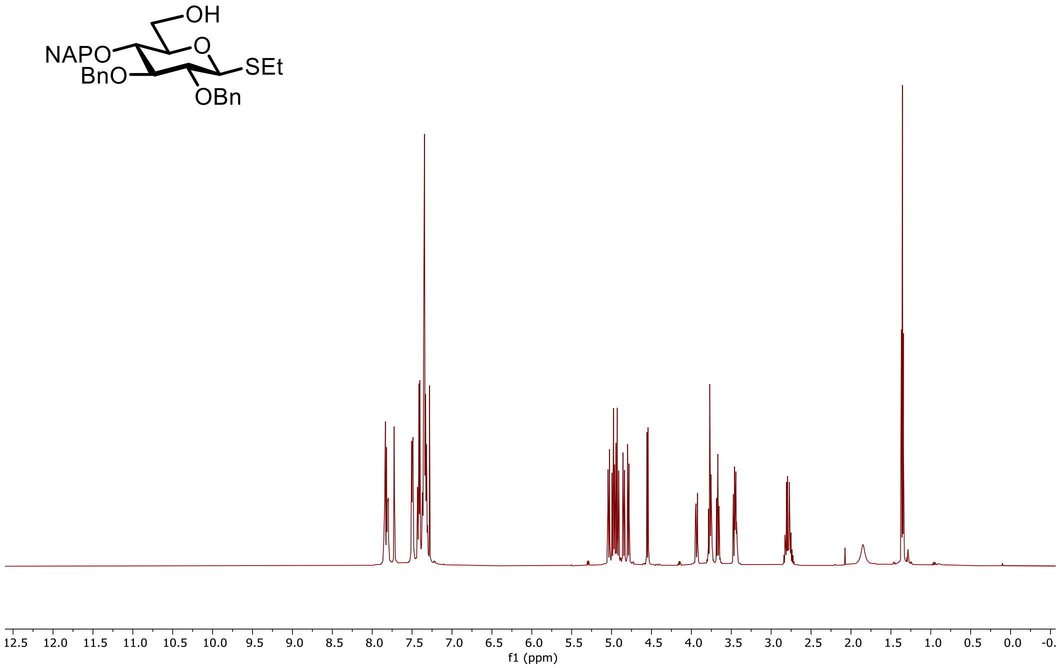

^{13}C NMR of 71 (151 MHz, $CDCl_3$)

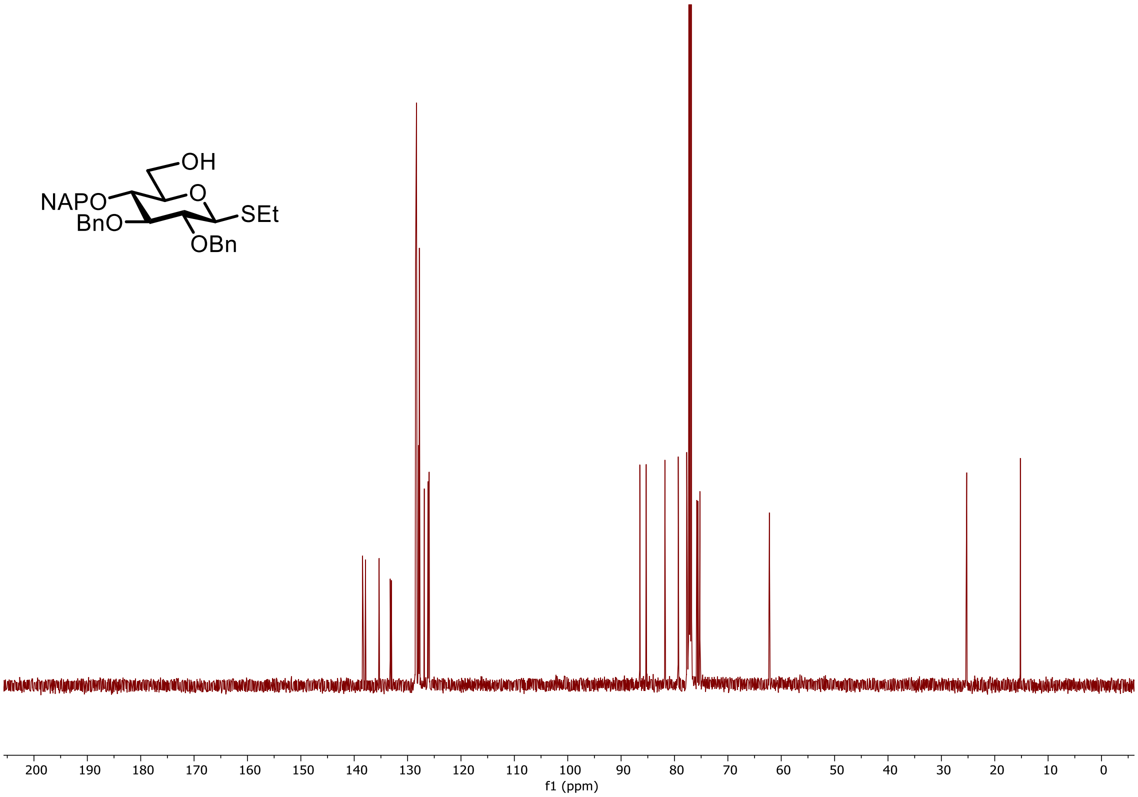

COSY NMR of 71 ($CDCl_3$)

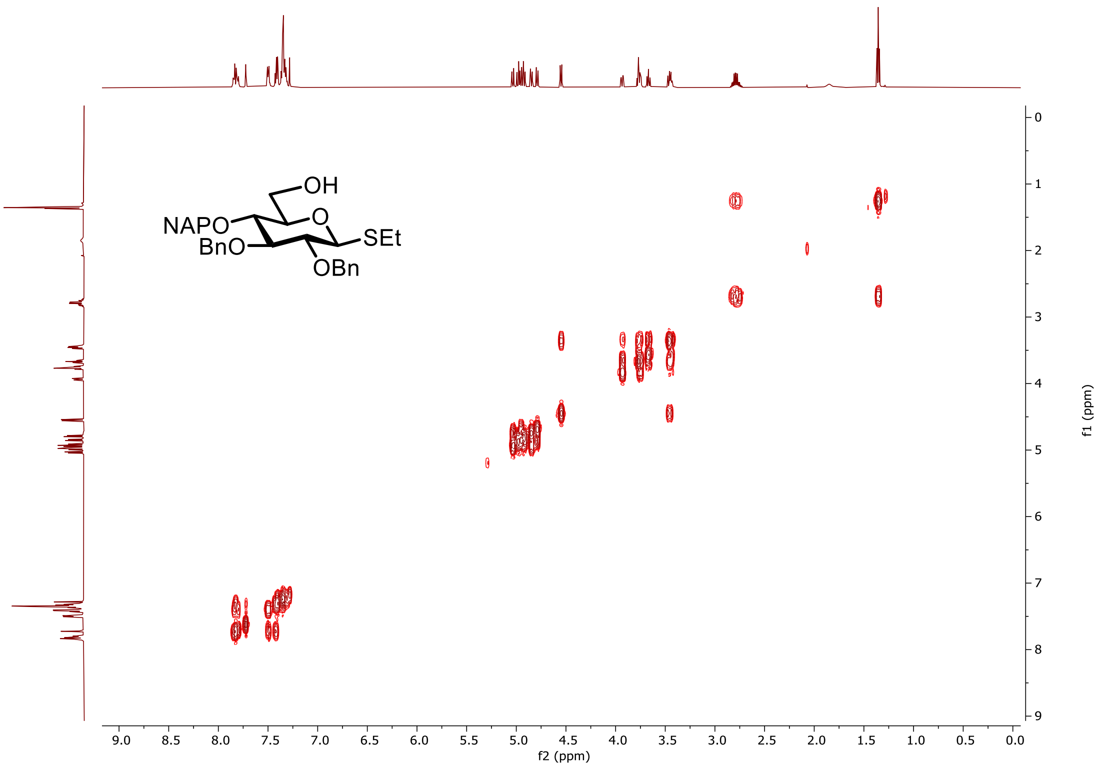

HSQC NMR of 71 ($CDCl_3$)

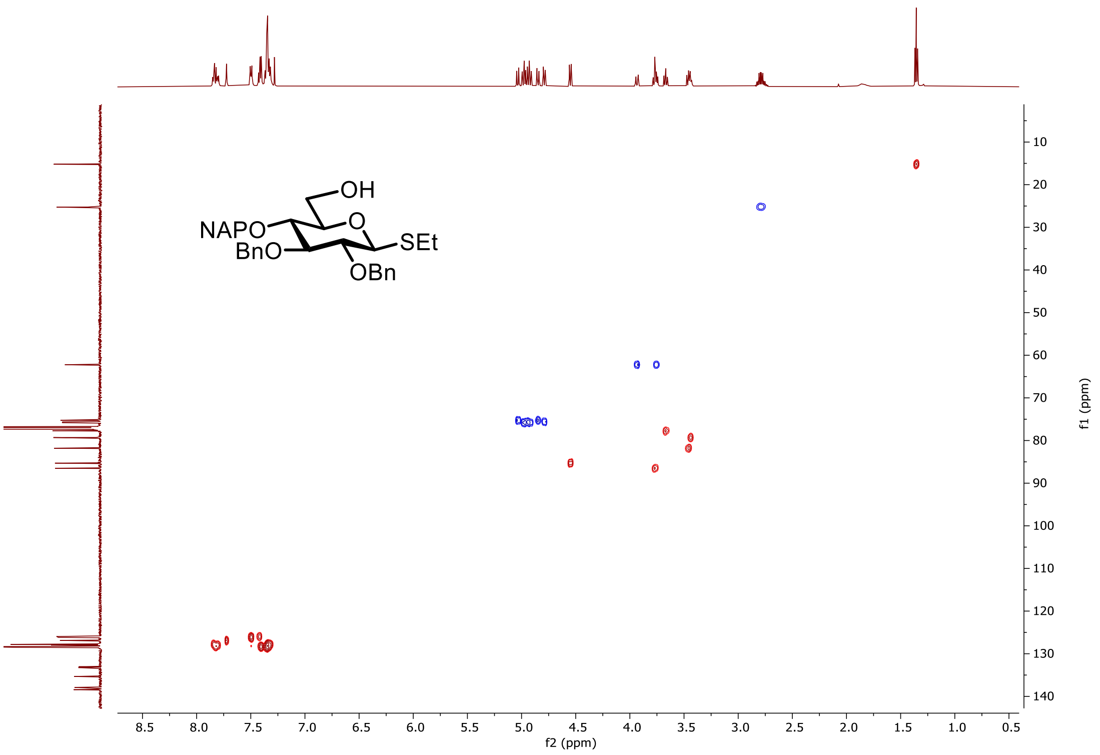

Ethyl 2,3-di-*O*-benzyl-4-*O*-(2-naphthylmethyl)-6-*O*-methyl-1-thio-β-D-glucopyranoside, 72

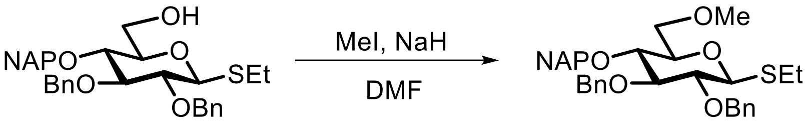

Ethyl 2,3-di-*O*-benzyl-4-*O*-(2-naphthylmethyl)-1-thio-β-D-glucopyranoside **71** (645 mg, 1.2 mmol) was dissolved in DMF (15 mL), sodium hydride (60% dispersion in mineral oil, 190 mg, 4.8 mmol) was added. The system was cooled under an ice bath and stirred for 30 min. After this time, iodomethane (224 µL, 3.6 mmol) was added and the reaction mixture was allowed to warm to room temperature and kept for 3 h. Upon completion, the reaction was carefully quenched (ice bath) by addition of ethanol (1.0 mL), after that, water (50 mL) was added. The mixture was extracted with ethyl acetate (50 mL) and the obtained organic layer was washed with water (50 mL), dried over Na_2SO_4, filtered, and evaporated. The resulting crude product was purified by column chromatography (Hexane : EtOAc = 10:1→6:1) to give **72** as a white solid (640 mg, 95%). ^{1}H NMR (600 MHz, $CDCl_3$) δ 7.89 – 7.78 (m, 3H), 7.73 (s, 1H), 7.54 – 7.47 (m, 2H), 7.44 – 7.39 (m, 3H), 7.38 – 7.29 (m, 8H), 5.03 (d, *J* = 11.1 Hz, 1H), 4.99 (d, *J* = 11.1 Hz, 1H), 4.96 (d, *J* = 10.3 Hz, 1H), 4.92 (d, *J* = 11.1 Hz, 1H), 4.82 (d, *J* = 11.1 Hz, 1H), 4.79 (d, *J* = 10.2 Hz, 1H), 4.51 (d, *J* = 9.8 Hz, 1H), 3.77 – 3.67 (m, 3H), 3.64 (dd, *J* = 10.8, 4.6 Hz, 1H), 3.53 – 3.45 (m, 2H), 3.39 (s, 3H), 2.87 – 2.73 (m, 2H), 1.36 (t, *J* = 7.4 Hz, 3H); ^{13}C NMR (151 MHz, $CDCl_3$) δ 138.59, 138.01, 135.63, 133.30, 133.02, 128.47, 128.44, 128.38, 128.24, 127.95, 127.89, 127.78, 127.70, 127.68, 126.72, 126.15, 125.98, 86.68, 85.22, 81.84, 79.00, 77.89, 75.77, 75.55, 75.17, 71.46, 59.38, 25.12, 15.07; $[\alpha]_D^{25}$ -13.68 (c = 1, $CHCl_3$); IR (neat) ν_{max} = 2873, 1455, 1083, 698 cm^{-1}; m/z (HRMS$^+$) $[M + Na]^+$ 581.2351 ($C_{34}H_{38}O_5SNa^+$ requires 581.2332).

^{1}H NMR of 72 (600 MHz, $CDCl_3$)

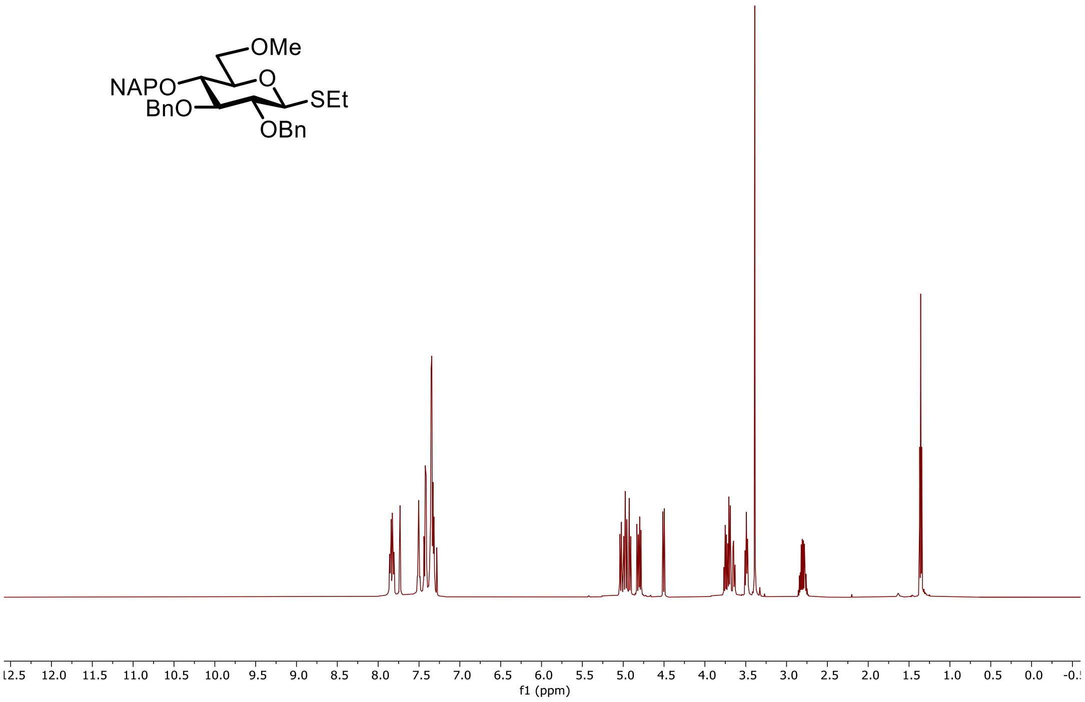

^{13}C NMR of 72 (151 MHz, $CDCl_3$)

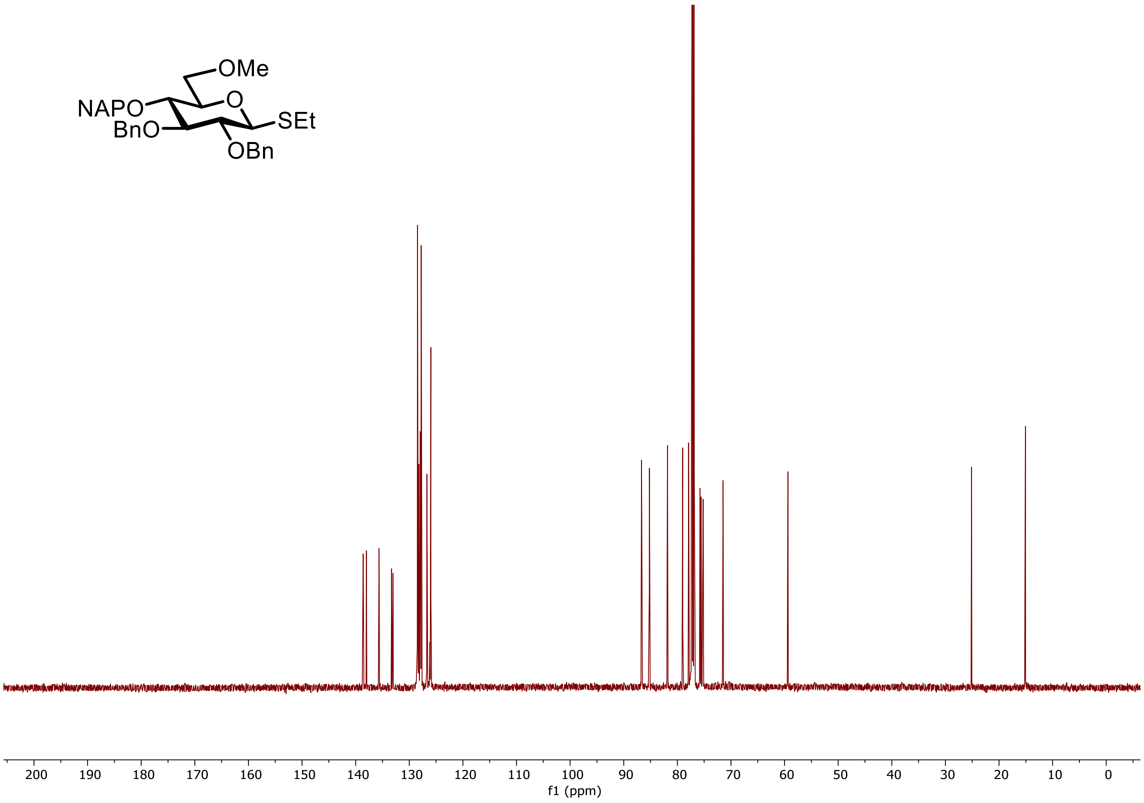

COSY NMR of 72 ($CDCl_3$)

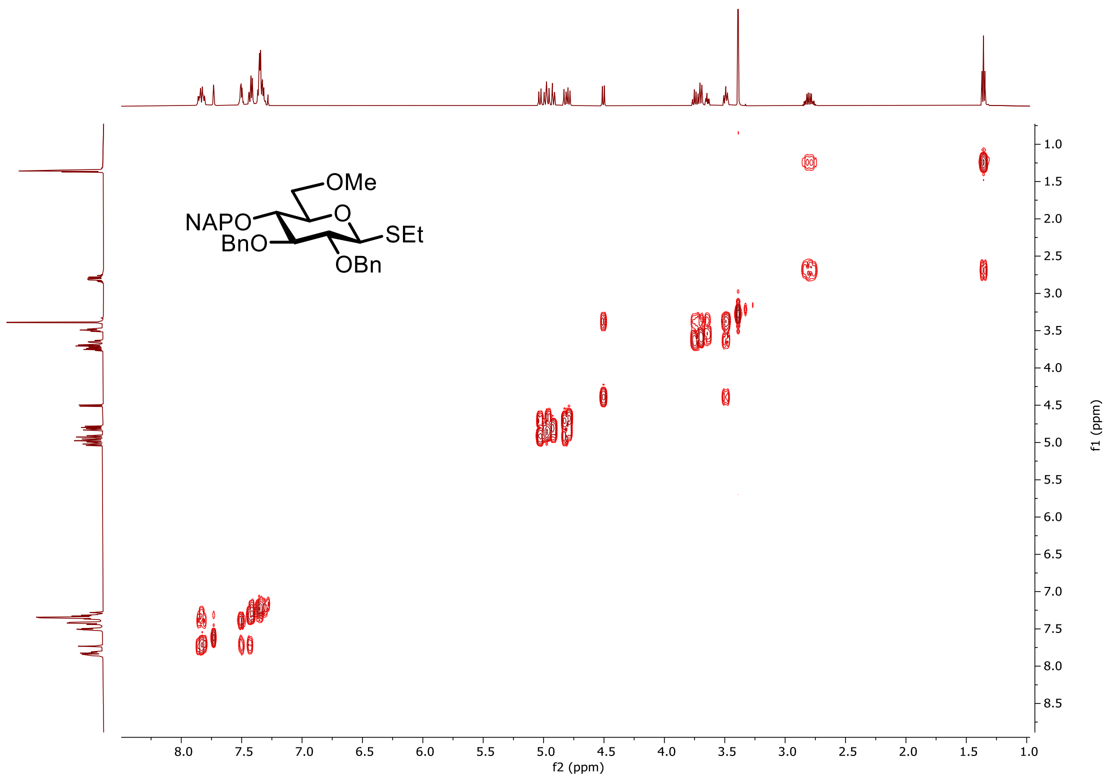

HSQC NMR of 72 ($CDCl_3$)

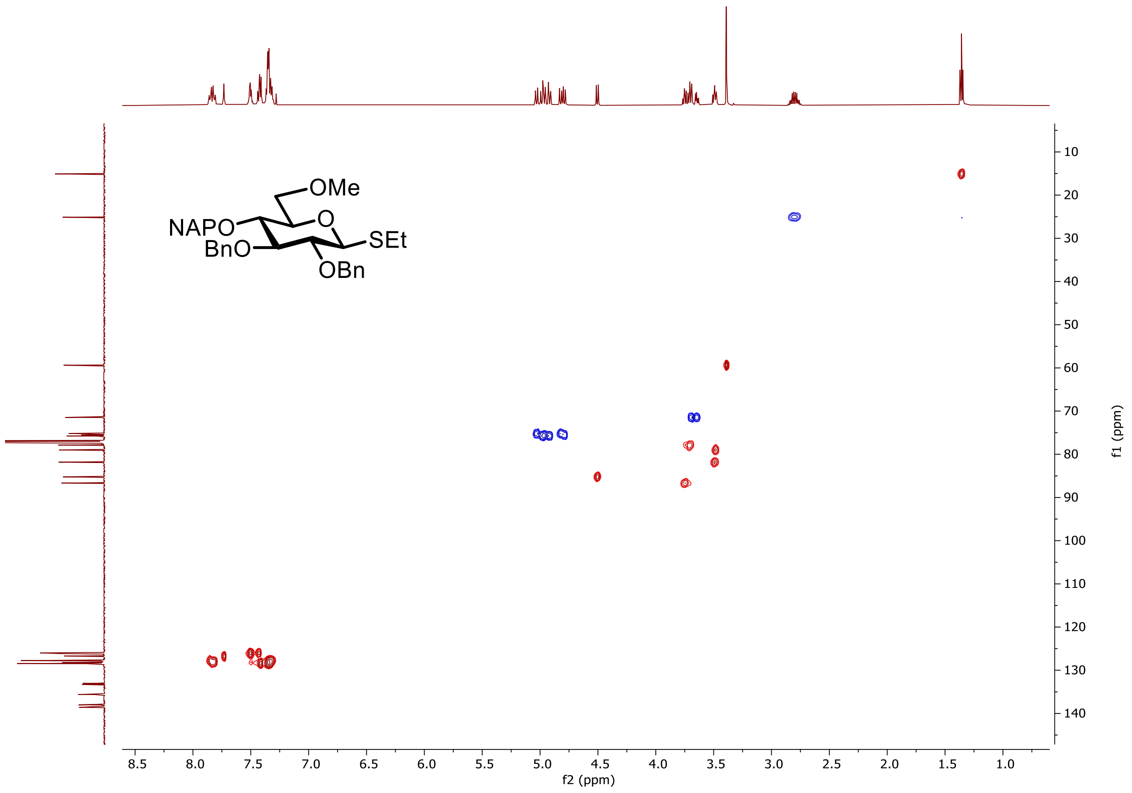

Ethyl 2,3-di-*O*-benzyl-6-*O*-methyl-1-thio-β-D-glucopyranoside, 73

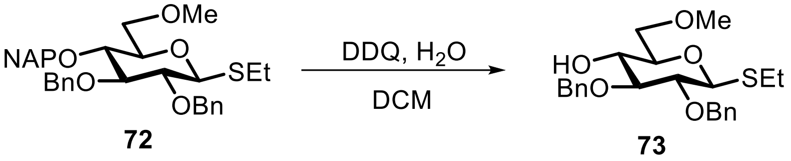

Ethyl 2,3-di-*O*-benzyl-4-*O*-(2-naphthylmethyl)-6-*O*-methyl-1-thio-β-D-glucopyranoside **72** (640 mg, 1.1 mmol) was dissolved in DCM (20 mL) and water (1.0 mL) was added. 2,3-Dichloro-5,6-dicyano-1,4-benzoquinone (DDQ) (520 mg, 2.3 mmol) was added, and reaction was stirred for 6 h at room temperature. DCM (20 mL) was added to dilute the reaction mixture and the solution was washed with $Na_2S_2O_3$ (aq) (5%, 40 mL).The water layer was extracted with DCM (20 mL), and the obtained organic layers were combined and dried over Na_2SO_4, filtered, and evaporated. The resulting crude product was purified by column chromatography (Hexane : EtOAc = 6:1→2:1) to give **73** as a clear oil (433 mg, 94%). ^{1}H NMR (600 MHz, $CDCl_3$) δ 7.44 – 7.30 (m, 10H), 4.96 (dd, *J* = 10.8, 6.5 Hz, 2H), 4.81 (d, *J* = 11.4 Hz, 1H), 4.76 (d, *J* = 10.3 Hz, 1H), 4.51 (d, *J* = 9.7 Hz, 1H), 3.71 – 3.59 (m, 3H), 3.53 (appt, *J* = 8.8 Hz, 1H), 3.46 – 3.39 (m, 5H), 2.85 – 2.71 (m, 2H), 2.60 (br. s, 1H), 1.35 (t, *J* = 7.4 Hz, 3H); ^{13}C NMR (151 MHz, $CDCl_3$) δ 138.58, 137.93, 128.64, 128.45, 128.39, 127.95, 127.93, 86.00, 85.23, 81.29, 77.80, 75.47, 75.39, 72.95, 71.84, 59.58, 25.14, 15.09; $[\alpha]_D^{25}$ -37.03 (c = 1, $CHCl_3$); IR (neat) ν_{max} = 2873, 1455, 1056, 697 cm^{-1}; m/z (HRMS$^+$) $[M + Na]^+$ 441.1717 ($C_{23}H_{30}O_5SNa^+$ requires 441.1706).

^{1}H NMR of 73 (600 MHz, $CDCl_3$)

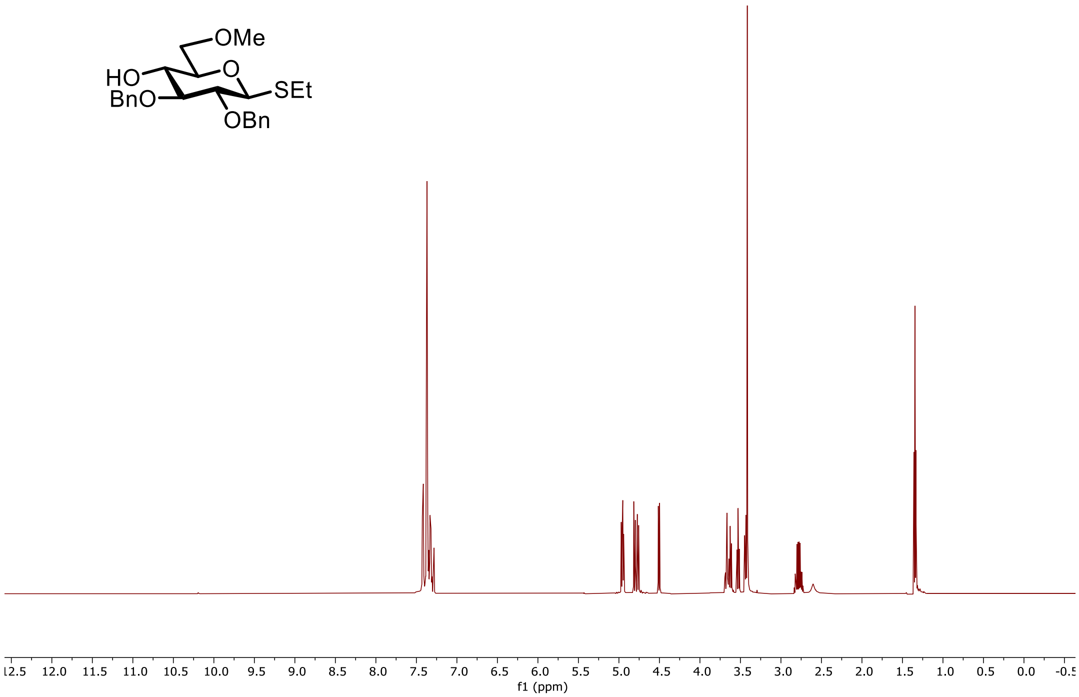

^{13}C NMR of 73 (151 MHz, $CDCl_3$)

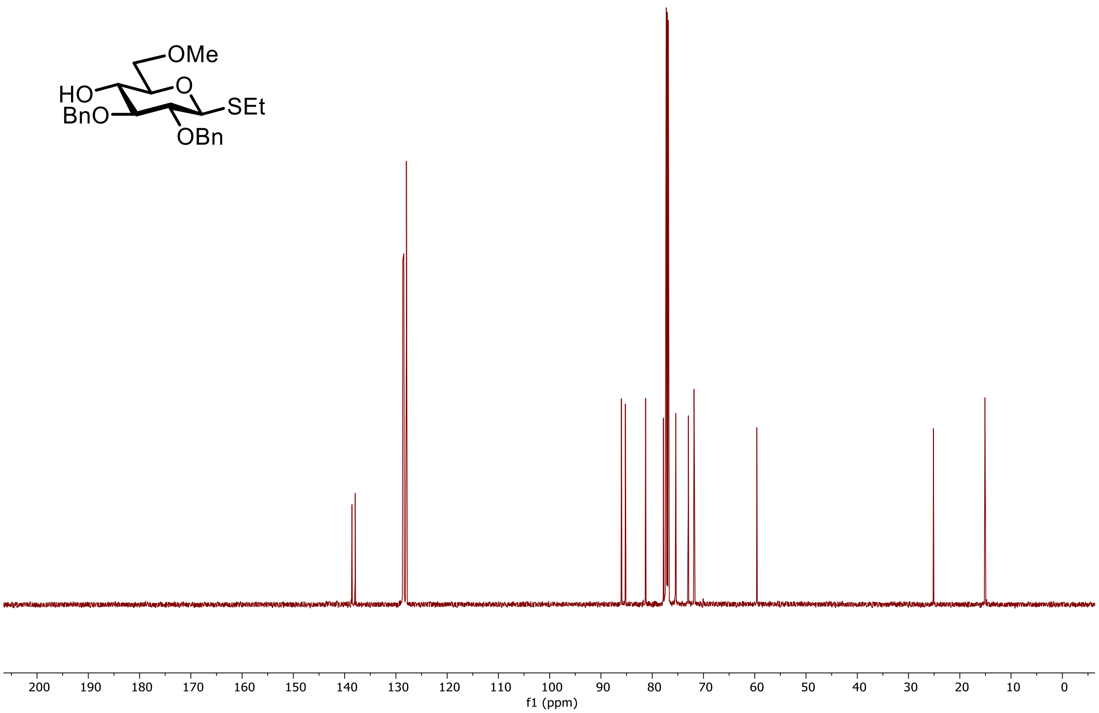

COSY NMR of 73 ($CDCl_3$)

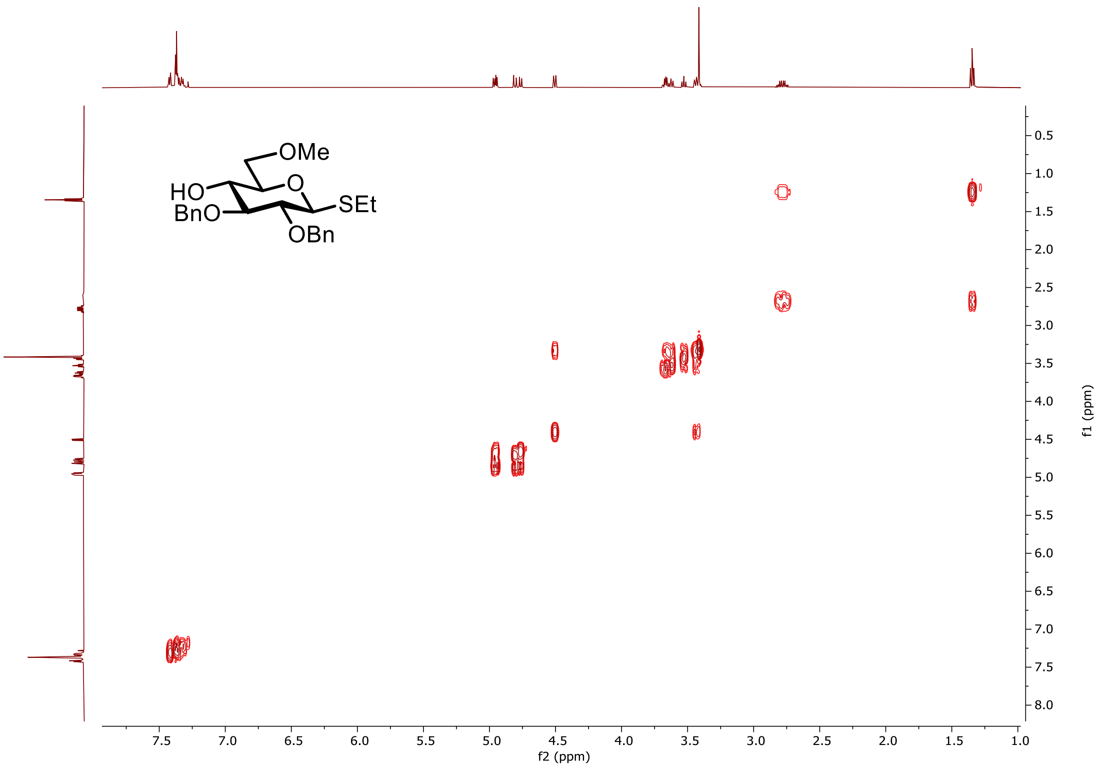

HSQC NMR of 73 ($CDCl_3$)

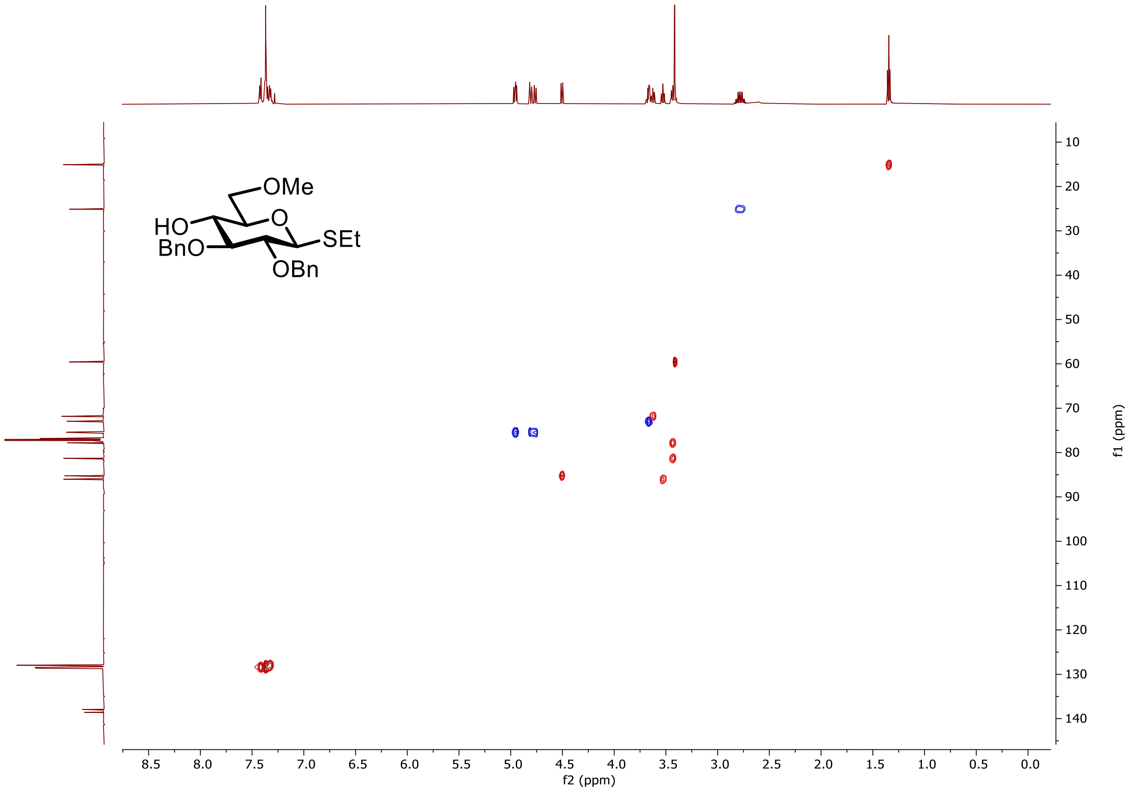

Ethyl 2,3-di-*O*-benzyl-4-*O*-(9-fluorenylmethoxycarbonyl)-6-*O*-methyl-1-thio-β-D-glucopyranoside, 32

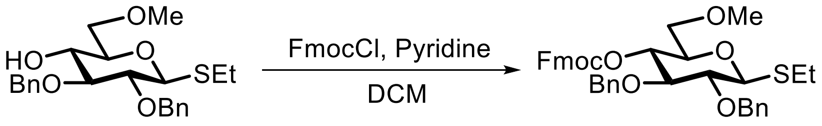

73 **32**

Ethyl 2,3-di-*O*-benzyl-6-*O*-methyl-1-thio-β-D-glucopyranoside **73** (433 mg, 1.0 mmol) was dissolved in anhydrous DCM (10 mL) and pyridine (2.5 mL) was added. The solution was cooled with an ice bath for 30 min, and fluorenylmethyloxycarbonyl chloride (FmocCl, 670 mg, 2.6 mmol) was added slowly. The reaction was warmed to room temperature and stirred for 6 h. Upon completion, DCM (40 mL) was added and the organic phase was washed with aqueous citric acid (0.5 M, 40 mL). After extracting the water phase with DCM (20 mL), the organic layers were combined and dried over Na_2SO_4, filtered, and evaporated. The resulting crude product was purified by column chromatography (Hexane : EtOAc = 8:1→4:1) to give **32** as a clear oil (584 mg, 88%). ^{1}H NMR (600 MHz, $CDCl_3$) δ 7.80 – 7.75 (m, 2H), 7.61 (d, *J* = 7.5 Hz, 1H), 7.58 (d, *J* = 7.5 Hz, 1H), 7.44 – 7.27 (m, 9H), 7.27 – 7.21 (m, 5H), 4.92 (d, *J* = 10.2 Hz, 1H), 4.89 – 4.83 (m, 3H), 4.73 (dd, *J* = 14.1, 10.8 Hz, 2H), 4.51 (d, *J* = 9.8 Hz, 1H), 4.47 (dd, *J* = 10.5, 7.3 Hz, 1H), 4.34 (dd, *J* = 10.5, 7.3 Hz, 1H), 4.21 (appt, *J* = 7.2 Hz, 1H), 3.74 (appt, *J* = 9.1 Hz, 1H), 3.62 (ddd, *J* = 9.6, 5.6, 3.2 Hz, 1H), 3.58 – 3.47 (m, 2H), 3.35 (s, 3H), 2.86 – 2.72 (m, 2H), 1.35 (t, *J* = 7.4 Hz, 3H); ^{13}C NMR (151 MHz, $CDCl_3$) δ 154.49, 143.29, 143.20, 141.31, 141.27, 138.07, 137.77, 128.43, 128.32, 127.95, 127.69, 127.19, 125.12, 125.09, 120.12, 85.19, 83.80, 81.33, 77.00, 75.59, 75.47, 71.99, 70.10, 59.54, 46.73, 25.13, 15.09; $[\alpha]_D^{25}$ 5.18 (c = 1, $CHCl_3$); IR (neat) ν_{max} = 1751, 1252, 1029, 738 cm^{-1}; m/z (HRMS$^+$) $[M + K]^+$ 679.2184 ($C_{38}H_{40}O_7SK^+$ requires 679.2126).

^{1}H NMR of 32 (600 MHz, $CDCl_3$)

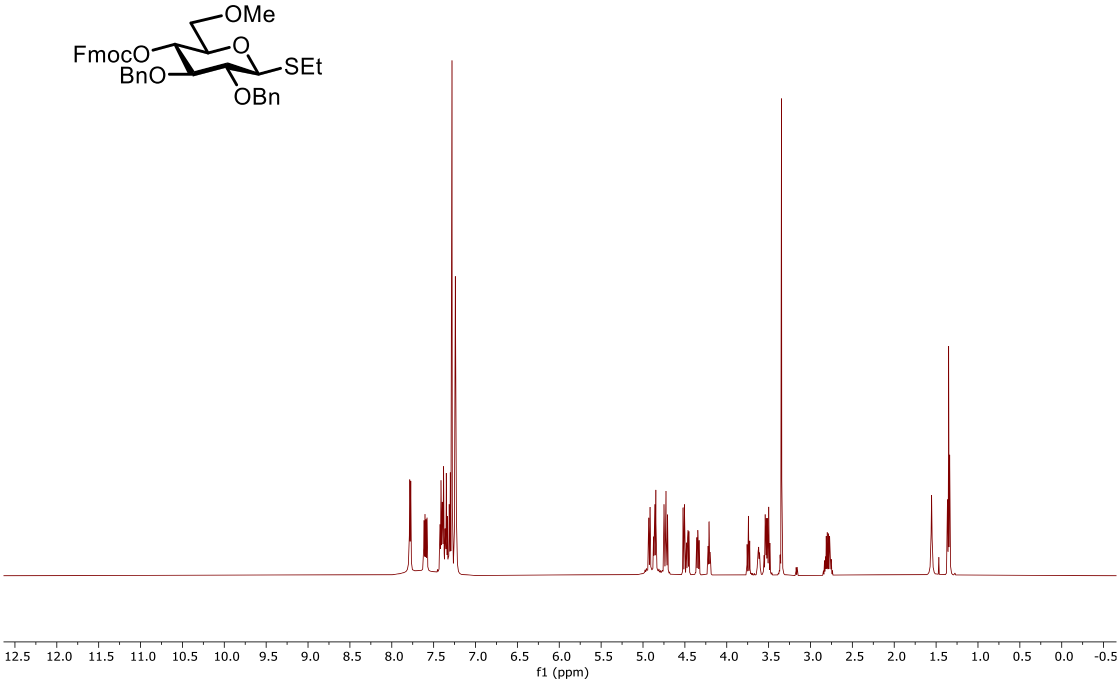

^{13}C NMR of 32 (151 MHz, $CDCl_3$)

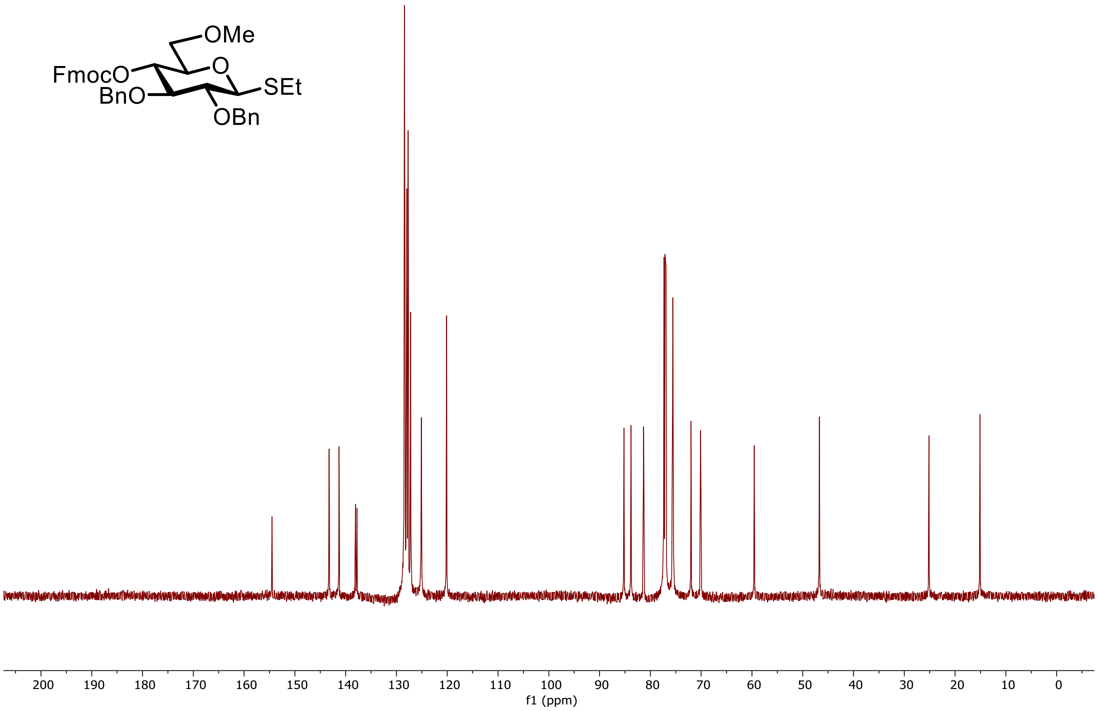

COSY NMR of 32 ($CDCl_3$)

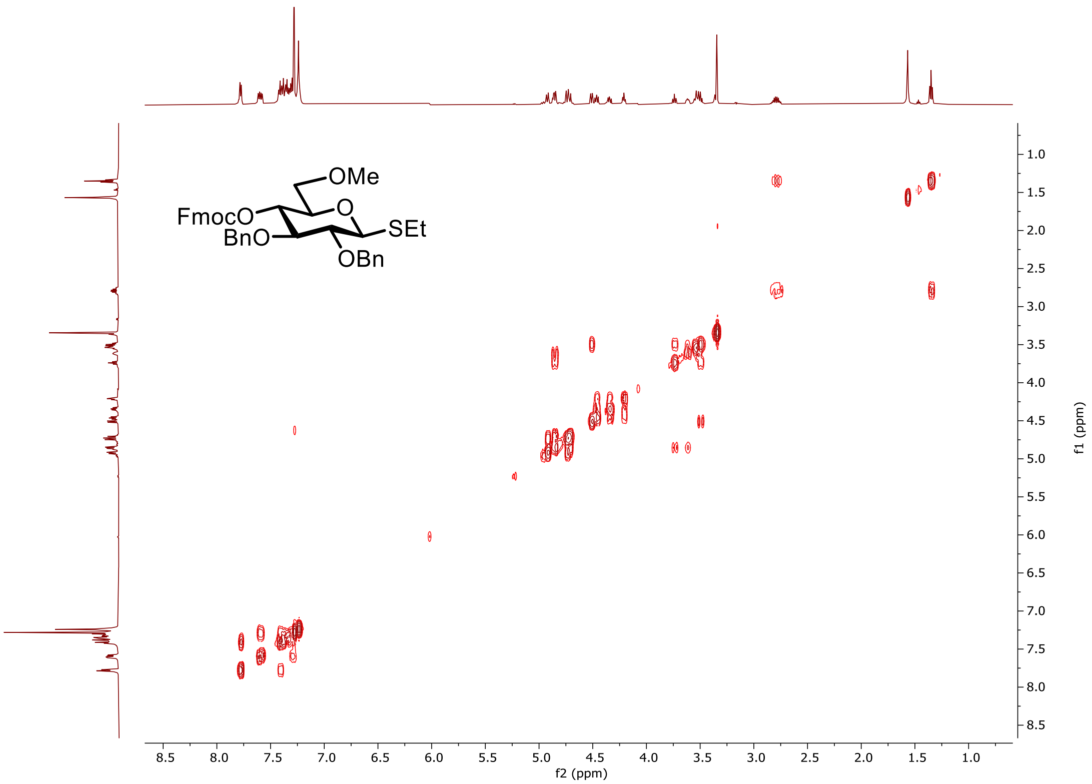

HSQC NMR of 32 ($CDCl_3$)

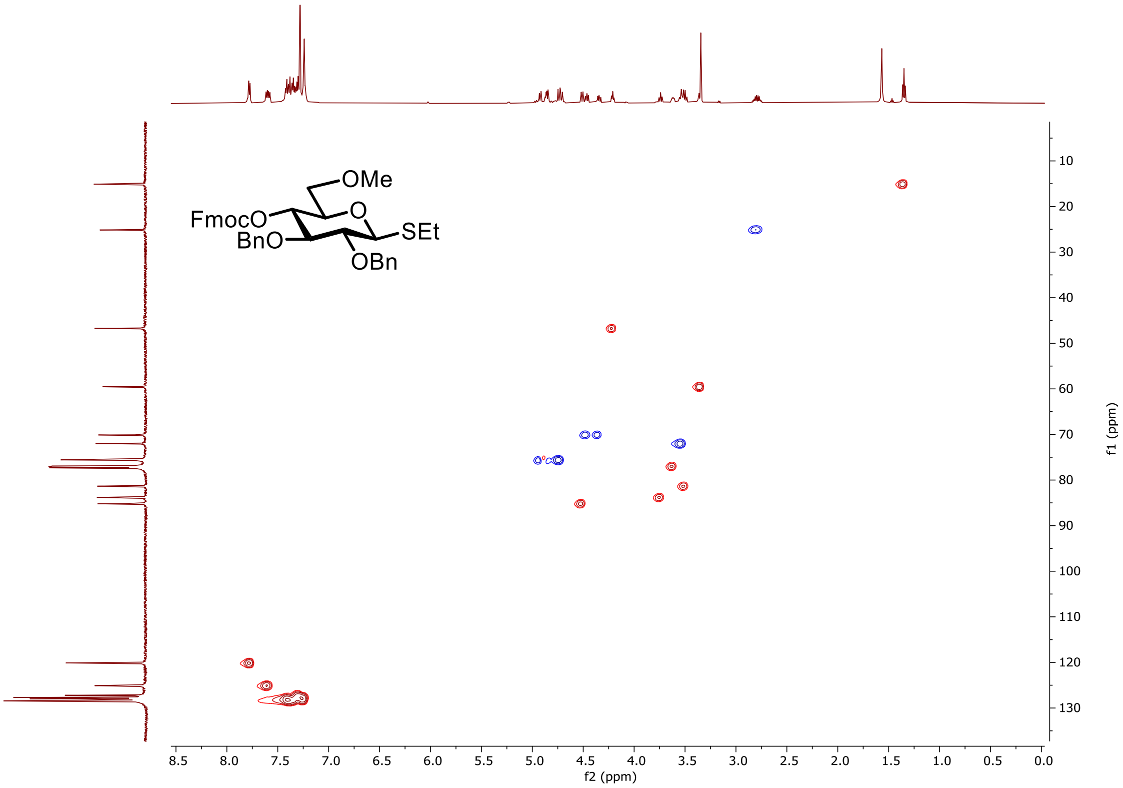

2.22 Synthesis of 110

Ethyl 2,6-di-*O*-benzyl-3-*O*-(2-naphthylmethyl)-1-thio-β-D-glucopyranoside, 114

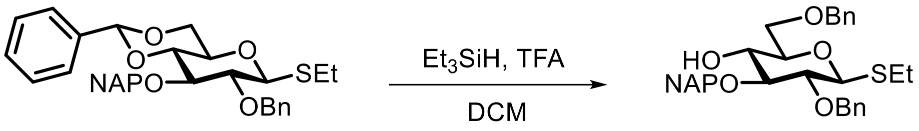

113 **114**

113 was prepared according to previously established procedures.[3]

Ethyl 2-*O*-benzyl-3-*O*-(2-naphthylmethyl)-4,6-*O*-phenylmethylene-1-thio-β-D-glucopyranoside **113** (3.00 g, 5.5 mmol) was dissolved in anhydrous DCM (70 mL), 4Å molecular sieves were added and the system was charged with nitrogen. Triethylsilane (8.8 mL, 55.3 mmol) was added and the mixture was stirred at room temperature for 30 min, and then cooled to 0°C. TFA (4.4 mL, 56.5 mmol) was added and the mixture was stirred for 1 h at 0°C. After the reaction was finished, molecular sieves were filtered and the organic phase was washed with saturated $NaHCO_3$ solution (aq) (150 mL, twice) and water (100 mL). The water layers were combined and extracted with DCM (50 mL). The obtained organic layers were combined and dried over Na_2SO_4, filtered, and evaporated. The resulting crude product was purified by column chromatography (Hexane : EtOAc = 6:1→4:1) to give **114** as a white solid (2.90 g, 97%). ^{1}H NMR (400 MHz, $CDCl_3$) δ 7.90 – 7.78 (m, 4H), 7.54 – 7.48 (m, 3H), 7.45 – 7.41 (m, 2H), 7.40 – 7.30 (m, 8H), 5.11 (d, *J* = 11.6 Hz, 1H), 5.03 – 4.96 (m, 2H), 4.80 (d, *J* = 10.2 Hz, 1H), 4.61 (d, *J* = 2.8 Hz, 2H), 4.54 (d, *J* = 9.7 Hz, 1H), 3.83 – 3.69 (m, 3H), 3.61 (appt, *J* = 8.8 Hz, 1H), 3.54 – 3.45 (m, 2H), 2.80 (qq, *J* = 12.6, 7.4 Hz, 2H), 2.62 (br. s, 1H), 1.37 (t, *J* = 7.4 Hz, 3H); ^{13}C NMR (101 MHz, $CDCl_3$) δ 137.96, 137.81, 135.97, 133.34, 133.05, 128.49, 128.45, 128.41, 128.00, 127.95, 127.84, 127.79, 127.77, 126.76, 126.20, 126.01, 125.88, 85.89, 85.20, 81.29, 77.83, 75.53, 75.50, 73.71, 72.26, 70.68, 25.14, 15.23; $[\alpha]_D^{25}$ -17.29 (c = 1, $CHCl_3$); IR (neat) ν_{max} =2896, 1059, 737 cm^{-1}; m/z (HRMS$^+$) [M + Na]$^+$ 567.2181 ($C_{33}H_{36}O_5SNa^+$ requires 567.2176).

^{1}H NMR of 114 (400 MHz, $CDCl_3$)

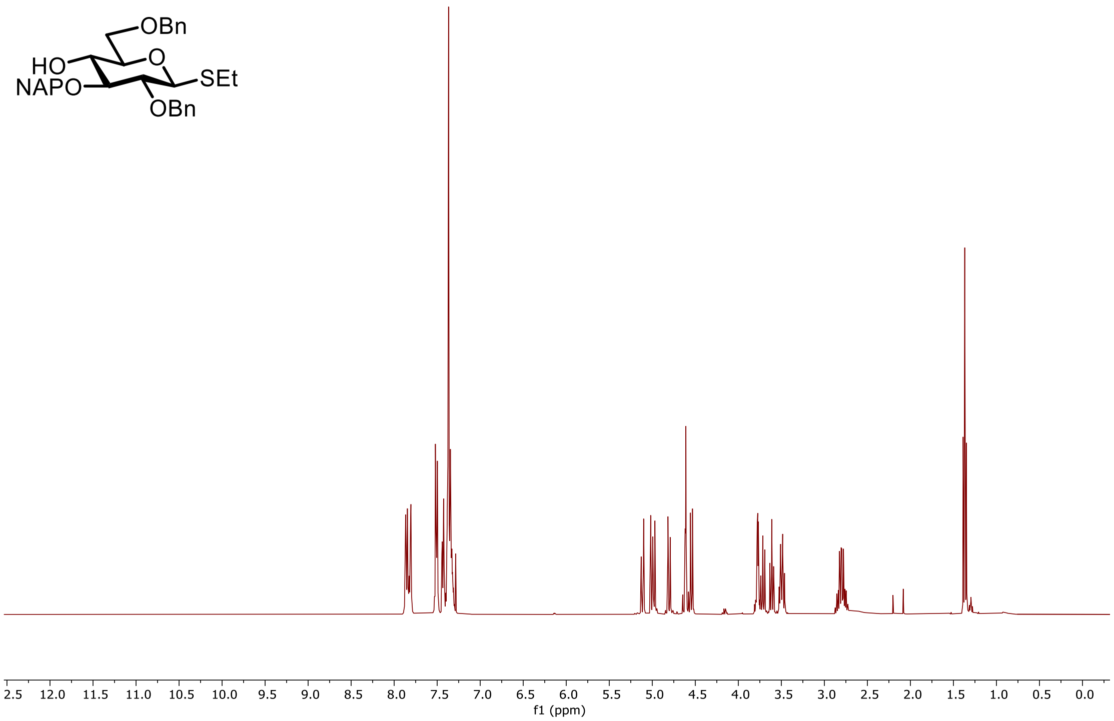

^{13}C NMR of 114 (101 MHz, $CDCl_3$)

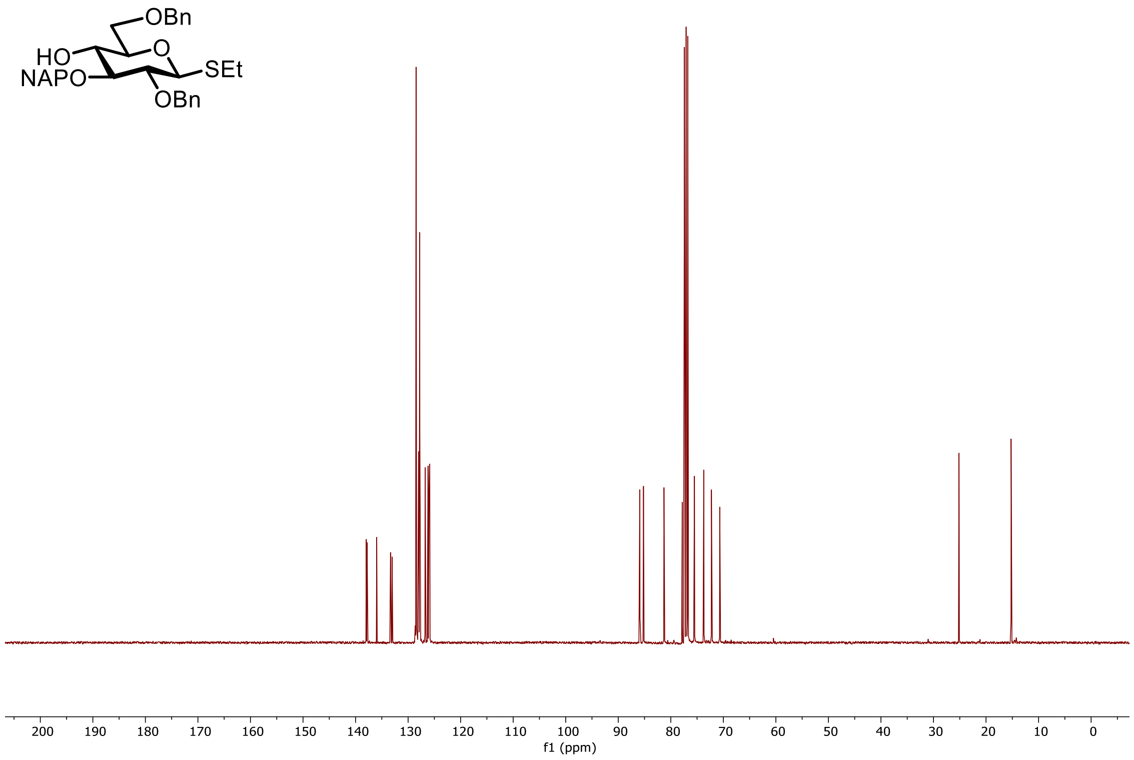

COSY NMR of 114 ($CDCl_3$)

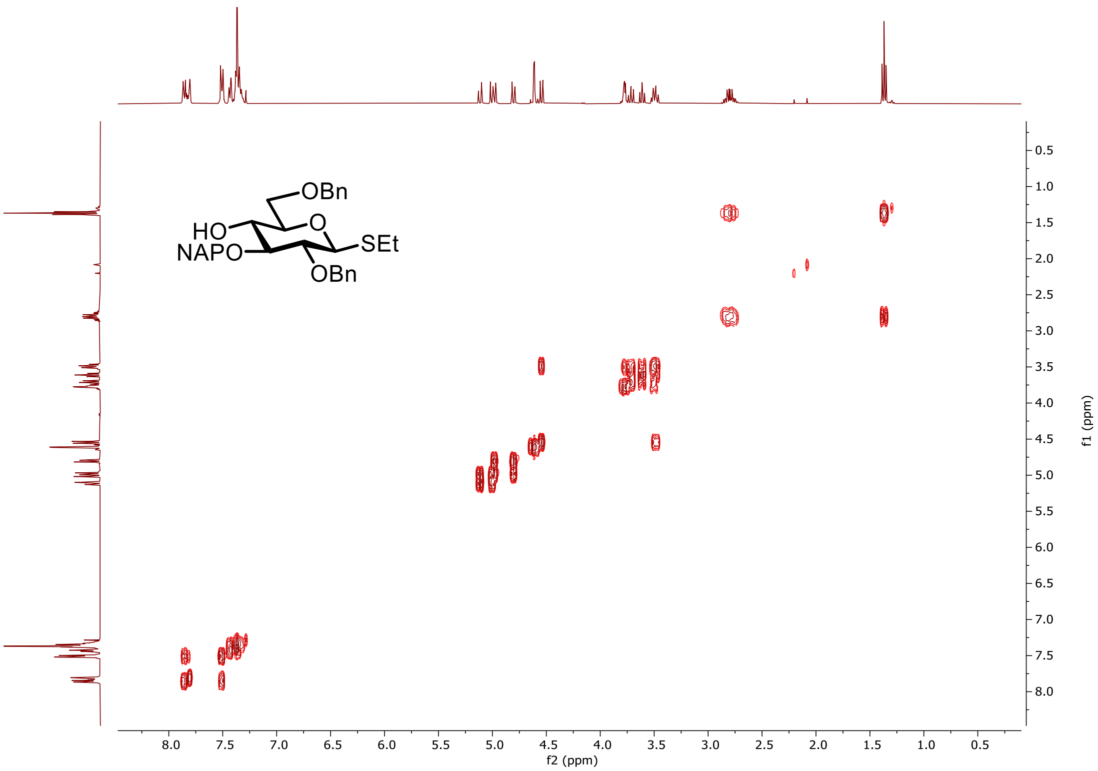

HSQC NMR of 114 ($CDCl_3$)

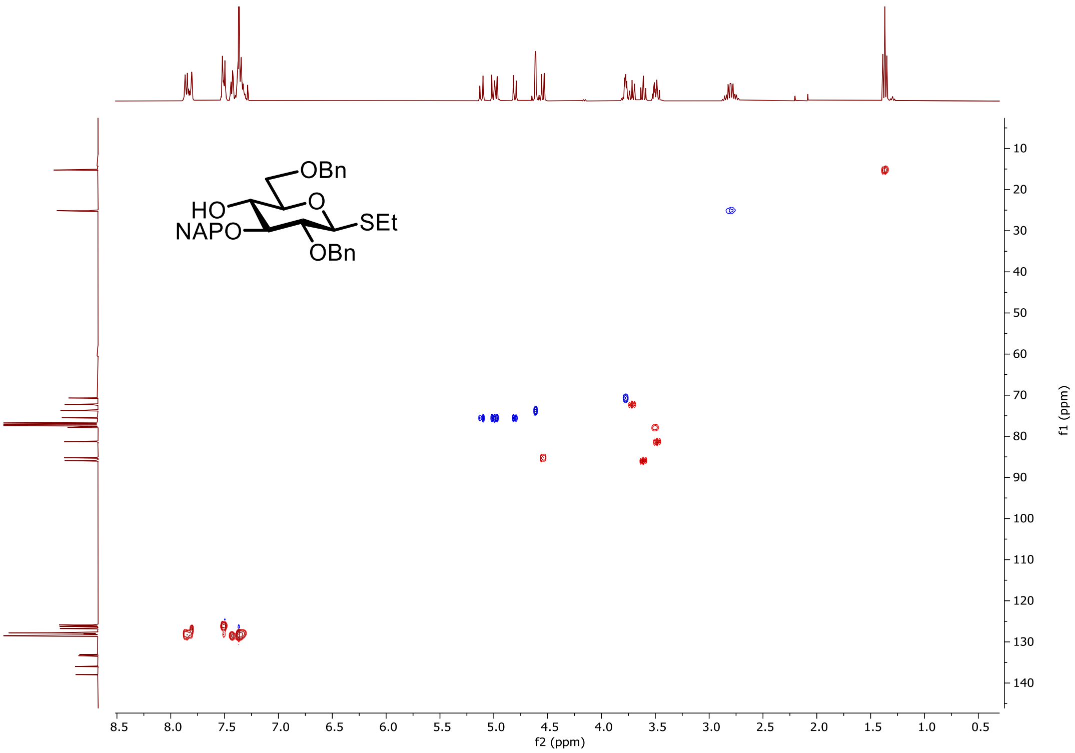

Ethyl 2,6-di-*O*-benzyl-3-*O*-(2-naphthylmethyl)-4-*O*-(9-fluorenylmethoxycarbonyl)-1-thio-β-D-glucopyranoside, 115

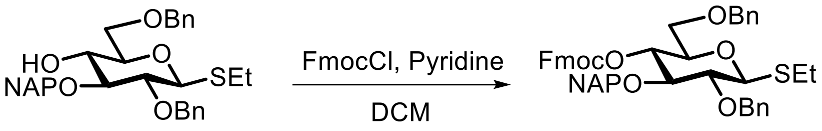

114 **115**

Ethyl 2,6-di-*O*-benzyl-3-*O*-(2-naphthylmethyl)-1-thio-β-D-glucopyranoside **114** (2.90 g, 5.3 mmol) was dissolved in anhydrous DCM (40 mL) and pyridine (10 mL) was added. The solution was cooled with an ice bath for 30 min, and fluorenylmethyloxycarbonyl chloride (FmocCl, 3.4 g, 13.1 mmol) was added slowly. The reaction was warmed to room temperature and stirred for 6 h. Upon completion, DCM (60 mL) was added and the organic phase was washed with aqueous citric acid (0.5 M, 100 mL). After extracting the water phase with DCM (40 mL), the organic layers were combined and dried over Na_2SO_4, filtered, and evaporated. The resulting crude product was purified by column chromatography (Hexane : EtOAc = 8:1→4:1) to give **115** as a white solid (3.81 g, 94%). ^{1}H NMR (600 MHz, $CDCl_3$) δ 7.80 – 7.74 (m, 3H), 7.73 – 7.68 (m, 3H), 7.49 (d, *J* = 7.5 Hz, 2H), 7.47 – 7.30 (m, 14H), 7.29 – 7.22 (m, 3H), 5.03 (d, *J* = 11.6 Hz, 1H), 5.00 – 4.92 (m, 2H), 4.89 (d, *J* = 11.5 Hz, 1H), 4.79 (d, *J* = 10.3 Hz, 1H), 4.60 – 4.52 (m, 3H), 4.28 (dd, *J* = 10.4, 7.3 Hz, 1H), 4.21 (dd, *J* = 10.4, 7.3 Hz, 1H), 4.04 (appt, *J* = 7.3 Hz, 1H), 3.82 (appt, *J* = 9.1 Hz, 1H), 3.74 – 3.64 (m, 3H), 3.57 (dd, *J* = 9.8, 8.8 Hz, 1H), 2.90 – 2.75 (m, 2H), 1.38 (t, *J* = 7.4 Hz, 3H); ^{13}C NMR (151 MHz, $CDCl_3$) δ 154.46, 143.23, 143.21, 141.27, 141.25, 137.95, 137.79, 135.57, 133.23, 132.95, 128.48, 128.42, 128.35, 128.09, 128.00, 127.94, 127.88, 127.67, 127.64, 127.16, 127.15, 126.44, 126.05, 125.86, 125.75, 125.11, 125.06, 120.05, 120.03, 85.20, 83.93, 81.40, 75.67, 75.61, 73.60, 70.01, 69.83, 46.61, 25.16, 15.22; $[\alpha]_D^{25}$ 10.49 (c = 1, $CHCl_3$); IR (neat) ν_{max} = 1750, 1451, 1250, 1029, 735 cm^{-1}; m/z (HRMS$^+$) [M + Na]$^+$ 789.2873 ($C_{48}H_{46}O_7SNa^+$ requires 789.2856).

^{1}H NMR of 115 (600 MHz, $CDCl_3$)

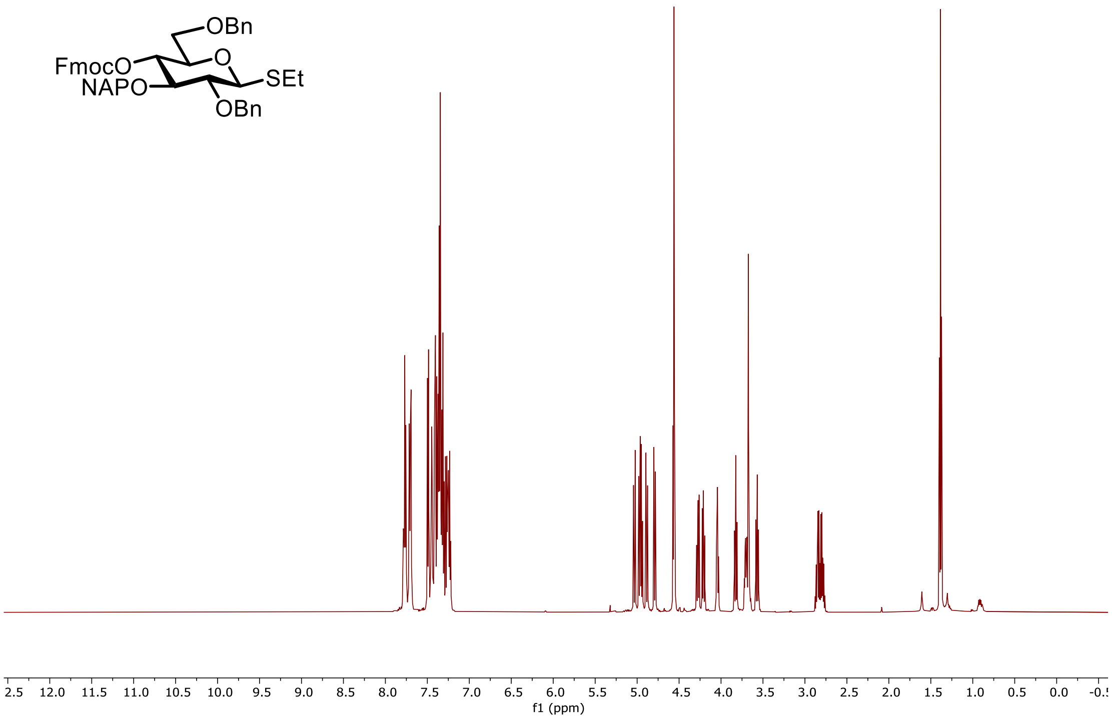

^{13}C NMR of 115 (151 MHz, $CDCl_3$)

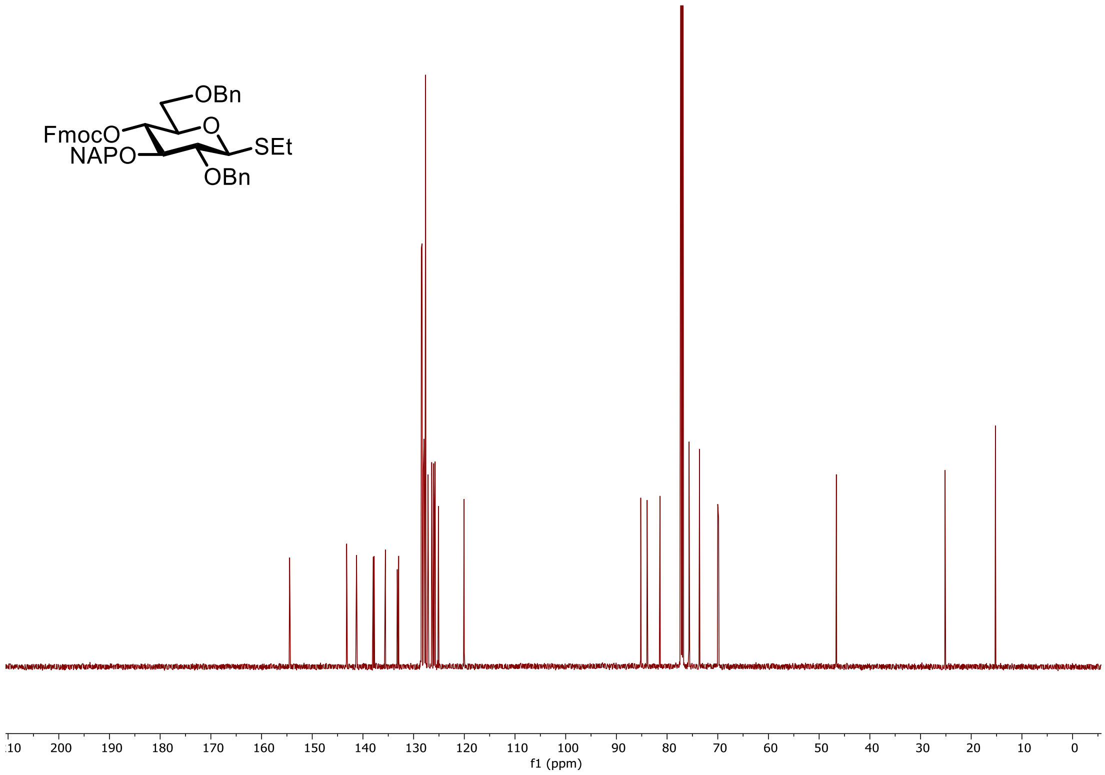

COSY NMR of 115 ($CDCl_3$)

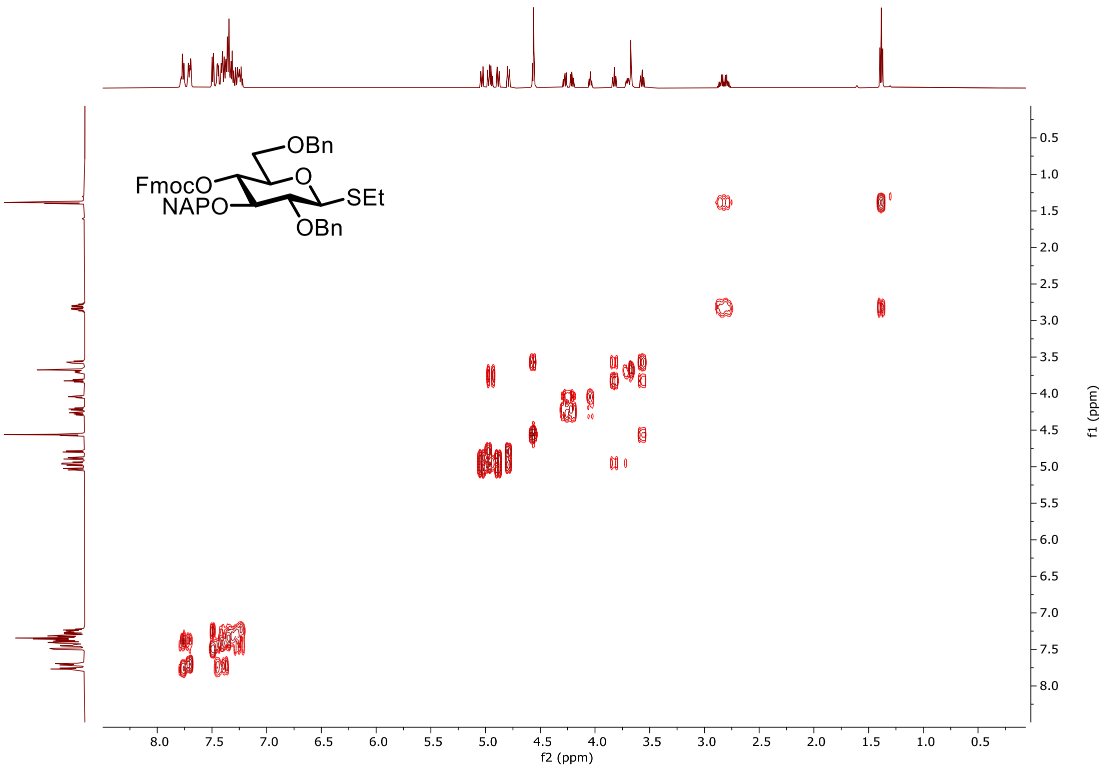

HSQC NMR of 115 ($CDCl_3$)

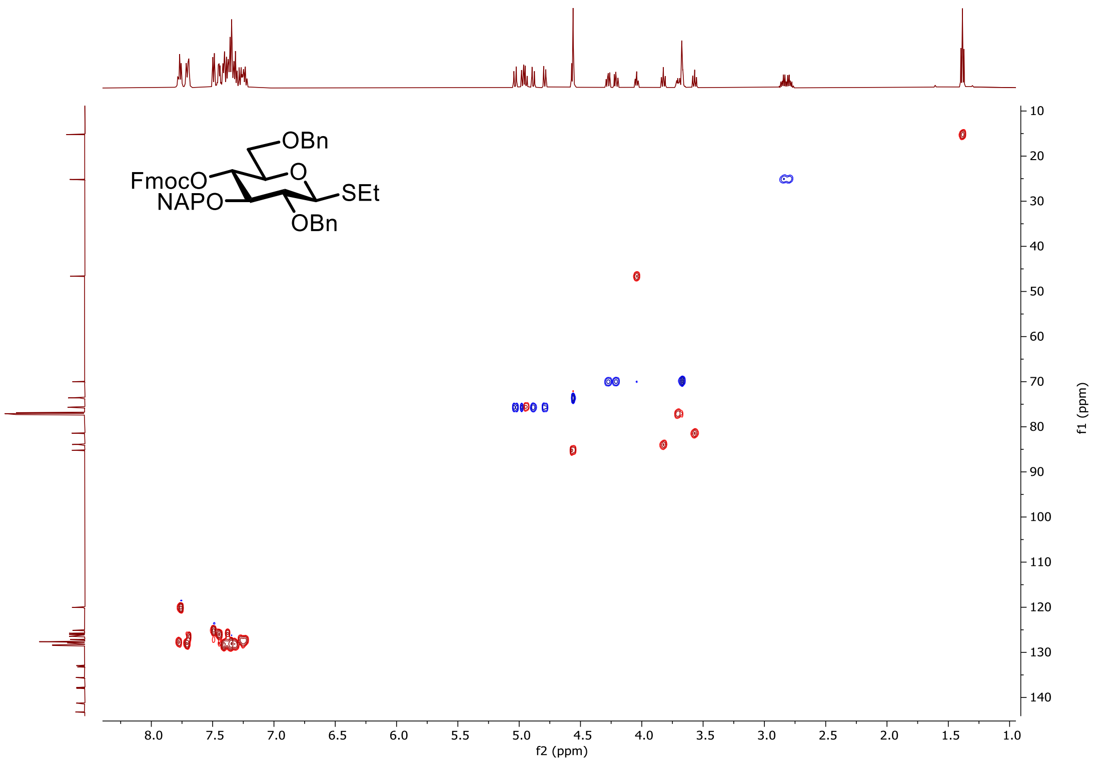

Ethyl 2,6-di-*O*-benzyl-4-*O*-(9-fluorenylmethoxycarbonyl)-1-thio-β-D-glucopyranoside, 116

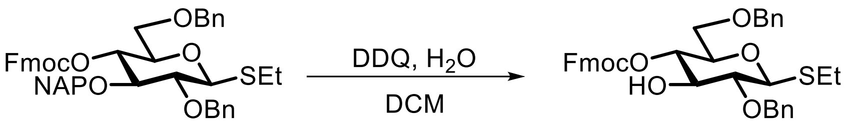

115 **116**

Ethyl 2,6-di-*O*-benzyl-3-*O*-(2-naphthylmethyl)-4-*O*-(9-fluorenylmethoxycarbonyl)-1-thio-β-D-glucopyranoside **115** (3.81 g, 5.0 mmol) was dissolved in DCM (40 mL) and water (2.0 mL) was added. 2,3-Dichloro-5,6-dicyano-1,4-benzoquinone (DDQ) (2.30 g, 10 mmol) was added, and reaction was stirred for 6 h at room temperature. DCM (50 mL) was added to dilute the reaction mixture and the solution was washed with $Na_2S_2O_3$ (aq) (5%, 80 mL).The water layer was extracted with DCM (40 mL), and the obtained organic layers were combined and dried over Na_2SO_4, filtered, and evaporated. The resulting crude product was purified by column chromatography (Hexane : EtOAc = 6:1→2:1) to give **116** as a white solid (2.52 g, 80%). ^{1}H NMR (600 MHz, $CDCl_3$) δ 7.82 – 7.76 (m, 2H), 7.60 (d, *J* = 7.5 Hz, 2H), 7.46 – 7.42 (m, 4H), 7.41 – 7.37 (m, 2H), 7.37 – 7.28 (m, 7H), 7.27 – 7.21 (m, 1H), 5.01 (d, *J* = 10.9 Hz, 1H), 4.82 (appt, *J* = 9.2 Hz, 1H), 4.73 (d, *J* = 10.9 Hz, 1H), 4.59 – 4.53 (m, 2H), 4.52 (d, *J* = 9.7 Hz, 1H), 4.46 (dd, *J* = 10.5, 7.3 Hz, 1H), 4.36 (dd, *J* = 10.5, 7.4 Hz, 1H), 4.23 (appt, *J* = 7.3 Hz, 1H), 3.84 (appt, *J* = 9.0 Hz, 1H), 3.71 – 3.61 (m, 3H), 3.40 (dd, *J* = 9.7, 8.7 Hz, 1H), 2.88 – 2.74 (m, 2H), 2.52 (s, 1H), 1.38 (t, *J* = 7.4 Hz, 3H); ^{13}C NMR (151 MHz, $CDCl_3$) δ 154.90, 143.30, 143.20, 141.32, 141.31, 137.91, 137.79, 128.67, 128.40, 128.35, 128.22, 127.94, 127.93, 127.65, 127.22, 127.20, 125.19, 125.16, 120.10, 84.84, 81.36, 76.94, 76.24, 75.45, 75.16, 73.58, 70.20, 69.48, 46.70, 25.24, 15.16; $[\alpha]_D^{25}$ 4.26 (c = 1, $CHCl_3$); IR (neat) ν_{max} = 2870, 1750, 1451, 1252, 737 cm^{-1}; m/z (HRMS$^+$) [M + Na]$^+$ 649.2254 ($C_{37}H_{38}O_7SNa^+$ requires 649.2230).

^{1}H NMR of 116 (600 MHz, CDCl$_3$)

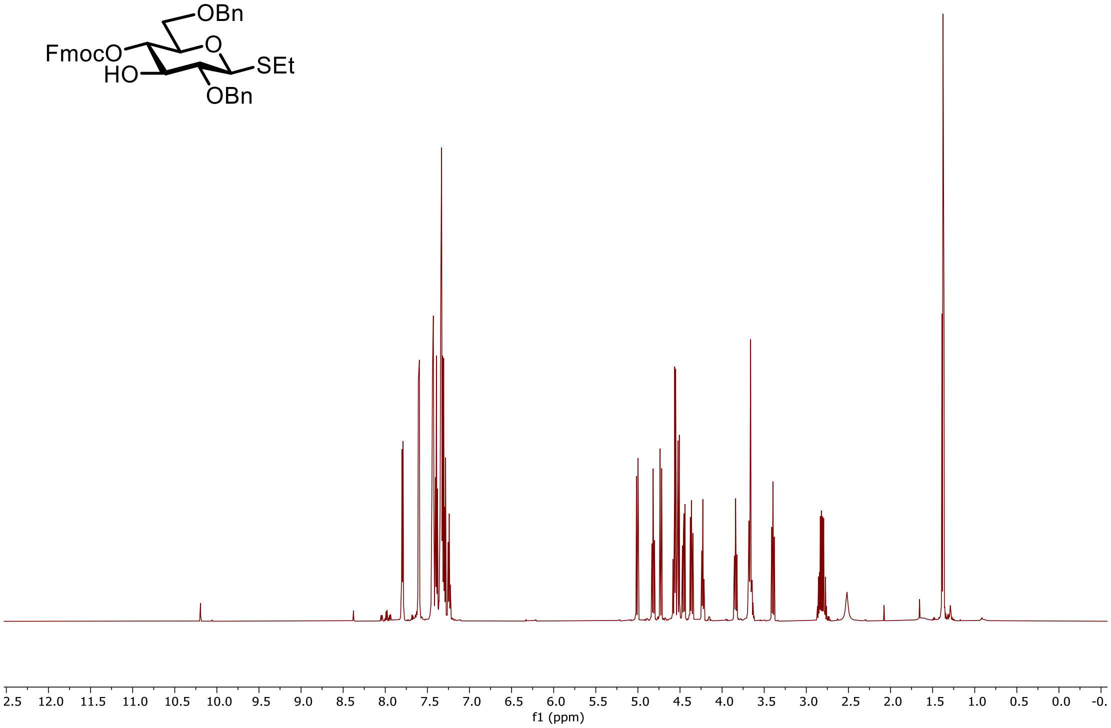

^{13}C NMR of 116 (151 MHz, CDCl$_3$)

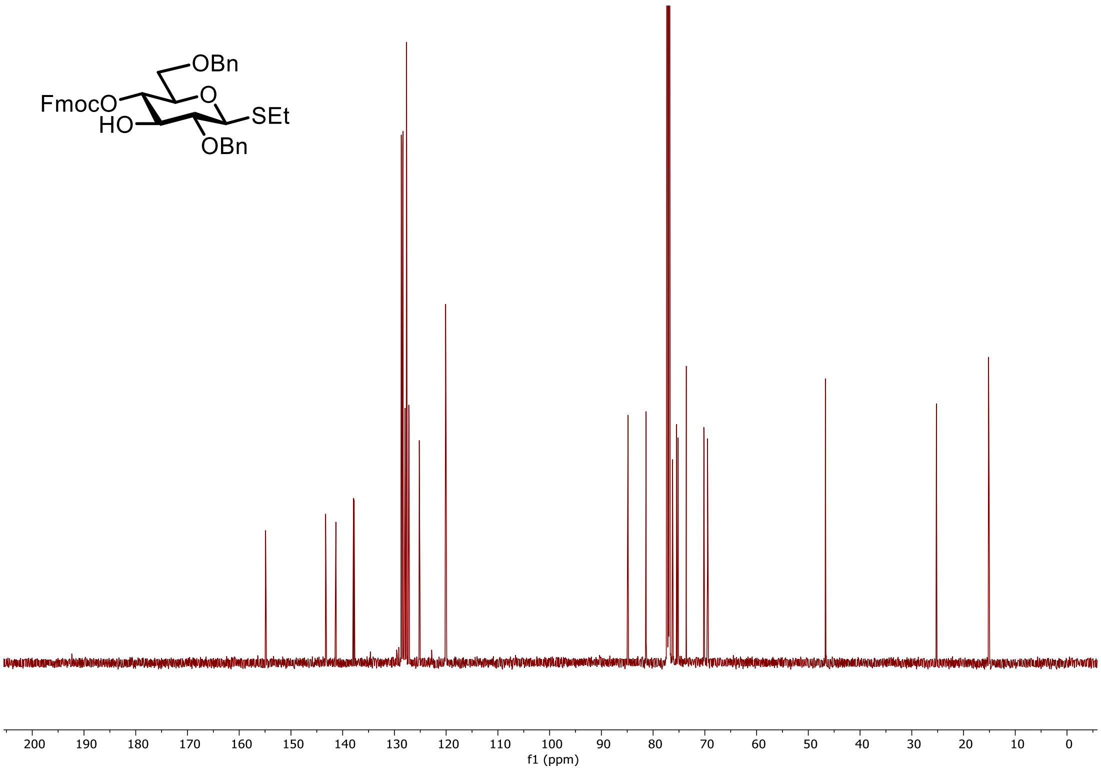

COSY NMR of 116 ($CDCl_3$)

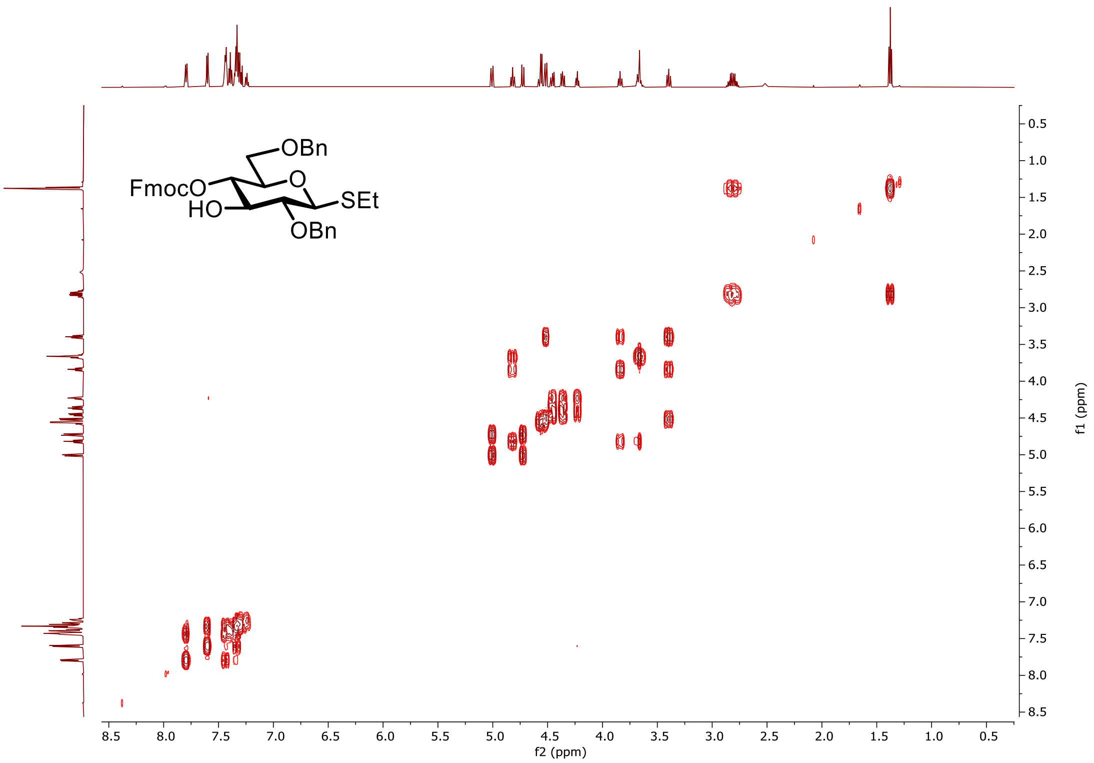

HSQC NMR of 116 ($CDCl_3$)

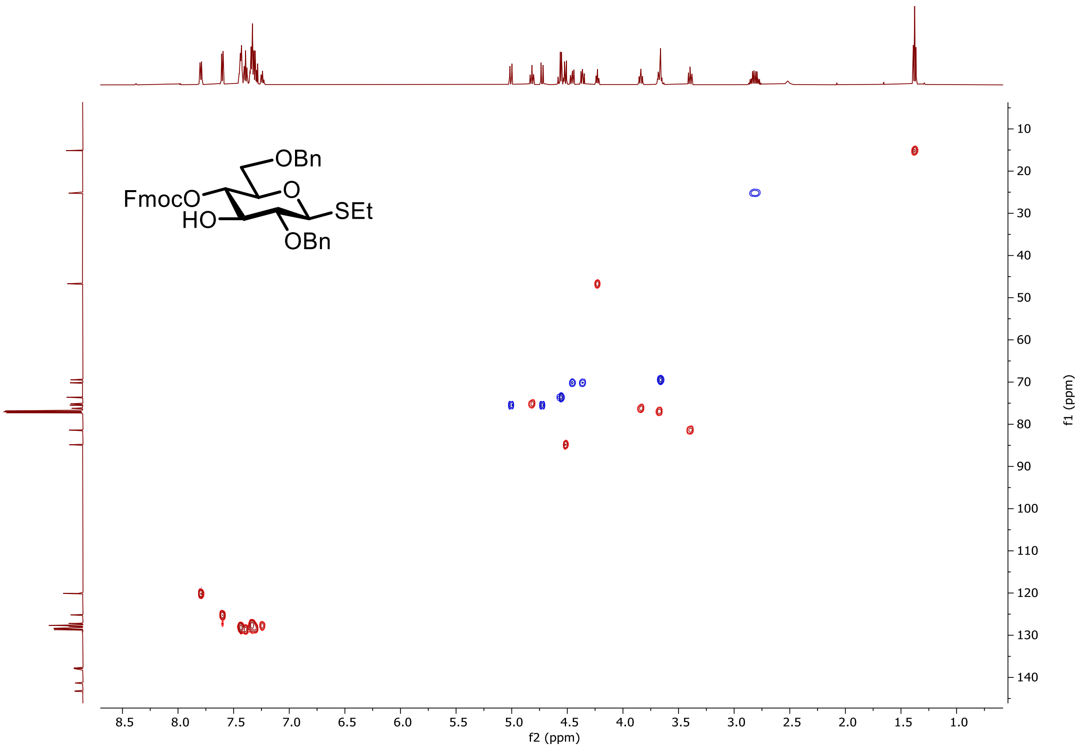

Ethyl 2,6-di-*O*-benzyl-3-*O*-acetyl-4-*O*-(9-fluorenylmethoxycarbonyl)-1-thio-β-D-glucopyranoside, 110

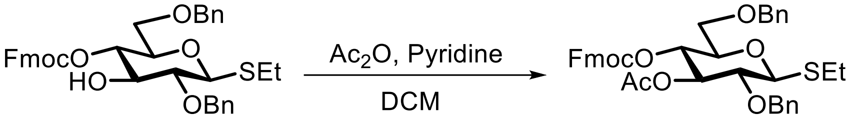

116 **110**

Ethyl 2,6-di-*O*-benzyl-4-*O*-(9-fluorenylmethoxycarbonyl)-1-thio-β-D-glucopyranoside **116** (626 mg, 1.0 mmol) was dissolved in anhydrous DCM (5 mL) and pyridine (1.5 mL) was added. The solution was cooled with an ice bath for 30 min, and acetic anhydride (Ac_2O, 280 µL, 3.0 mmol) was added slowly. The reaction was warmed to room temperature and stirred for 16 h. Completion of the reaction was confirmed by TLC, MeOH (0.5 mL) was added. DCM (20 mL) was added 30 min later, and the organic phase was washed with aqueous citric acid (0.5 M, 20 mL). After extracting the water phase with DCM (20 mL), the organic layers were combined and dried over Na_2SO_4, filtered, and evaporated. The resulting crude product was purified by column chromatography (Hexane : EtOAc = 8:1→4:1) to give **110** as a white solid (570 mg, 85%). ^{1}H NMR (700 MHz, $CDCl_3$) δ 7.79 (d, *J* = 7.9 Hz, 2H), 7.59 (d, *J* = 7.5 Hz, 1H), 7.57 (d, *J* = 7.5 Hz, 1H), 7.43 (appt, *J* = 7.5 Hz, 2H), 7.38 – 7.30 (m, 11H), 7.27 – 7.24 (m, 1H), 5.36 (appt, *J* = 9.3 Hz, 1H), 4.93 – 4.88 (m, 2H), 4.63 – 4.52 (m, 4H), 4.38 (dd, *J* = 10.3, 7.6 Hz, 1H), 4.24 (dd, *J* = 10.3, 7.6 Hz, 1H), 4.19 (appt, *J* = 7.5 Hz, 1H), 3.78 (ddd, *J* = 10.0, 5.1, 3.3 Hz, 1H), 3.71 – 3.64 (m, 2H), 3.53 (appt, *J* = 9.4 Hz, 1H), 2.88 – 2.76 (m, 2H), 1.89 (s, 3H), 1.37 (t, *J* = 7.4 Hz, 3H); ^{13}C NMR (176 MHz, $CDCl_3$) δ 169.98, 154.31, 143.24, 143.16, 141.27, 137.86, 137.62, 128.45, 128.33, 128.13, 127.95, 127.94, 127.92, 127.65, 127.64, 127.29, 127.24, 125.19, 125.18, 120.07, 120.05, 85.25, 79.27, 76.82, 75.31, 75.16, 73.71, 73.58, 70.34, 69.23, 46.56, 25.35, 20.78, 15.12; $[\alpha]_D^{25}$ -6.04 (c = 1, $CHCl_3$); IR (neat) ν_{max} = 1752, 1256, 1030, 737 cm^{-1}; m/z (HRMS$^+$) $[M + Na]^+$ 691.2341 ($C_{39}H_{40}O_8SNa^+$ requires 691.2336).

^{1}H NMR of 110 (700 MHz, $CDCl_3$)

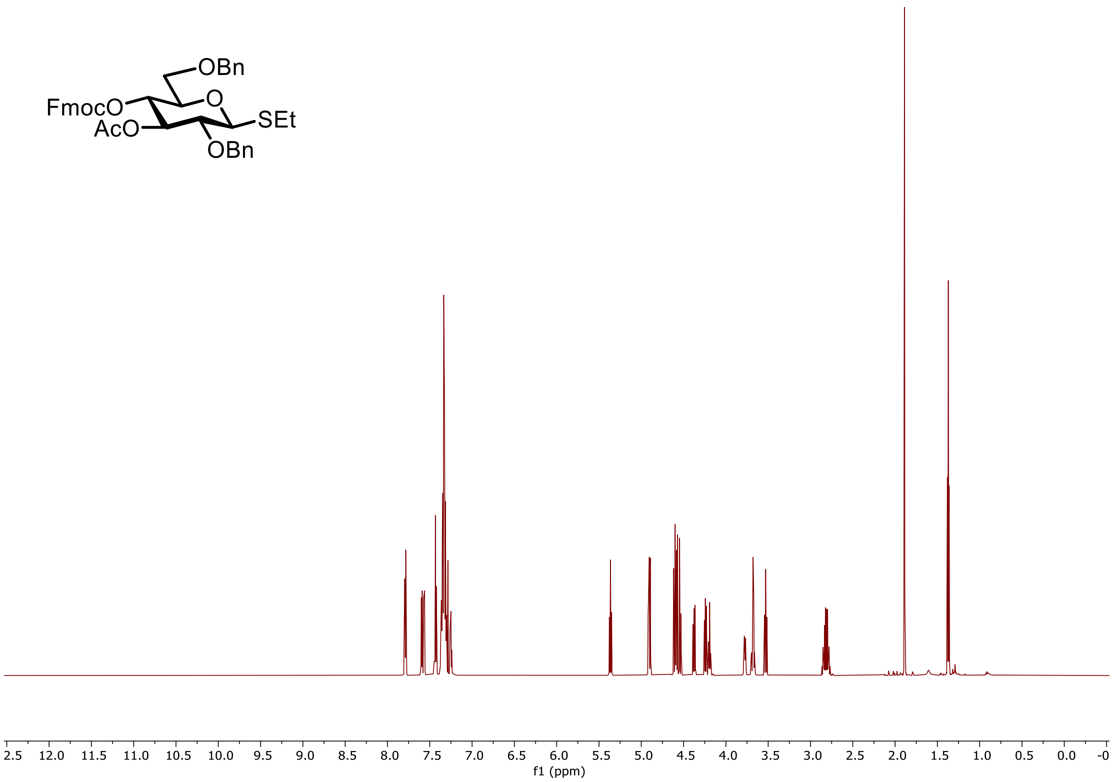

^{13}C NMR of 110 (176 MHz, $CDCl_3$)

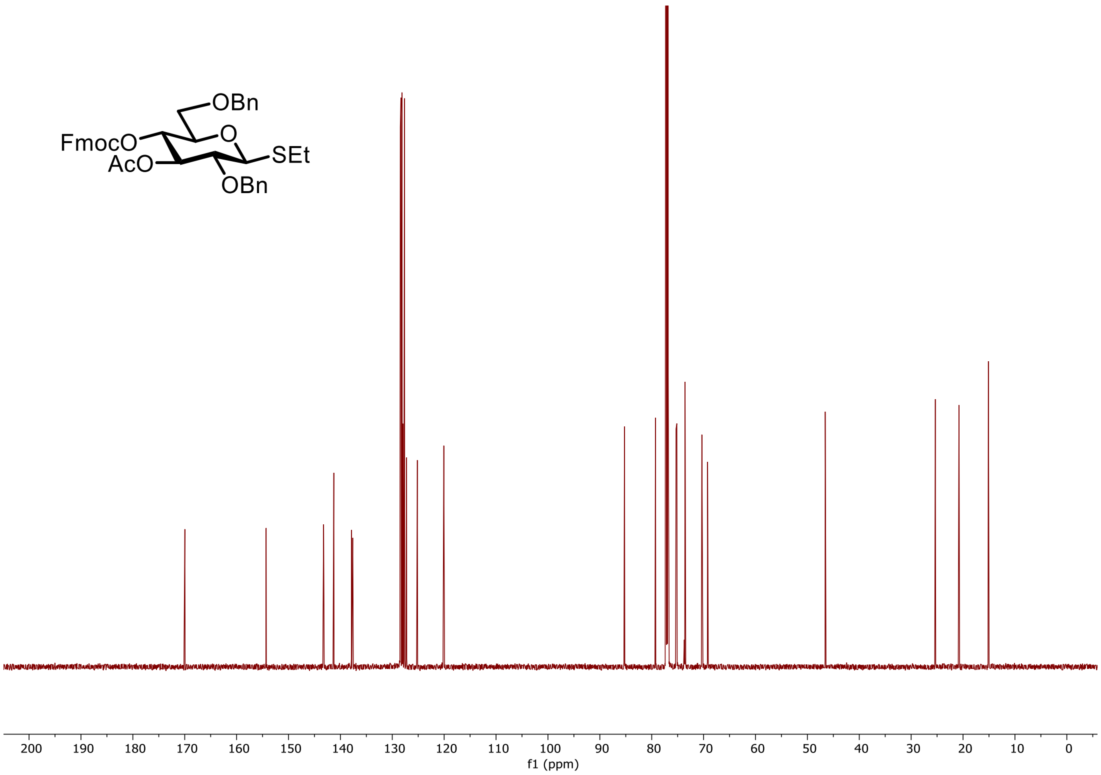

COSY NMR of 110 ($CDCl_3$)

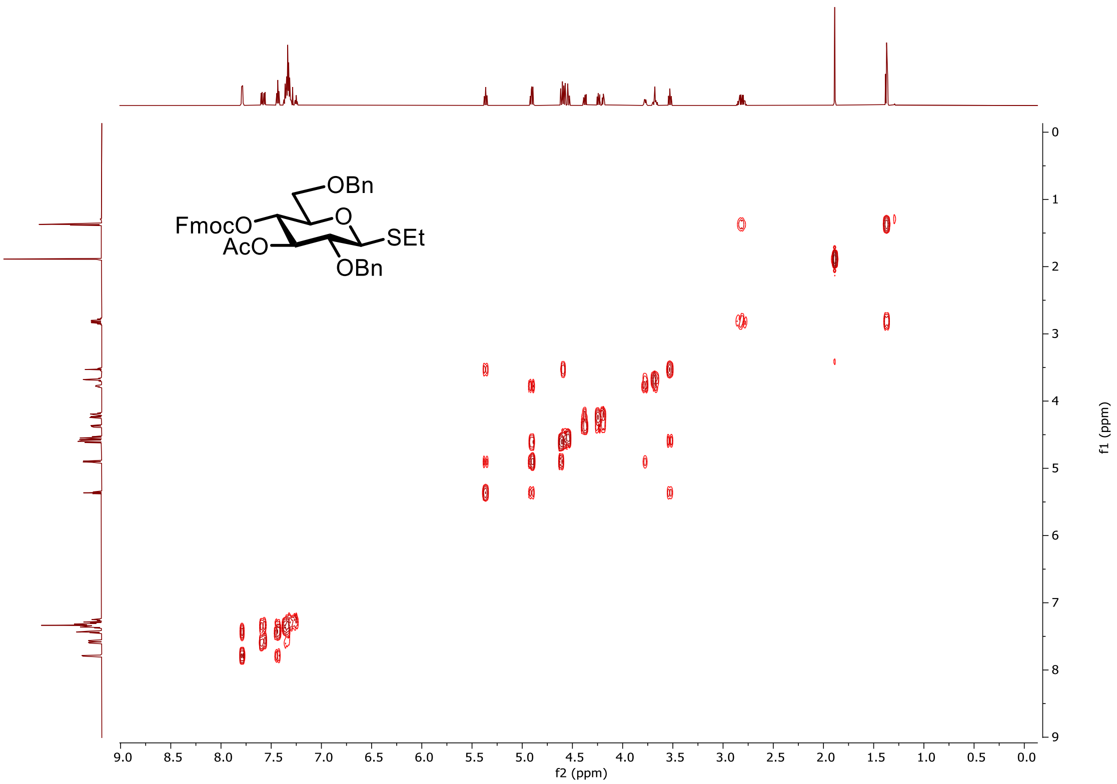

HSQC NMR of 110 ($CDCl_3$)

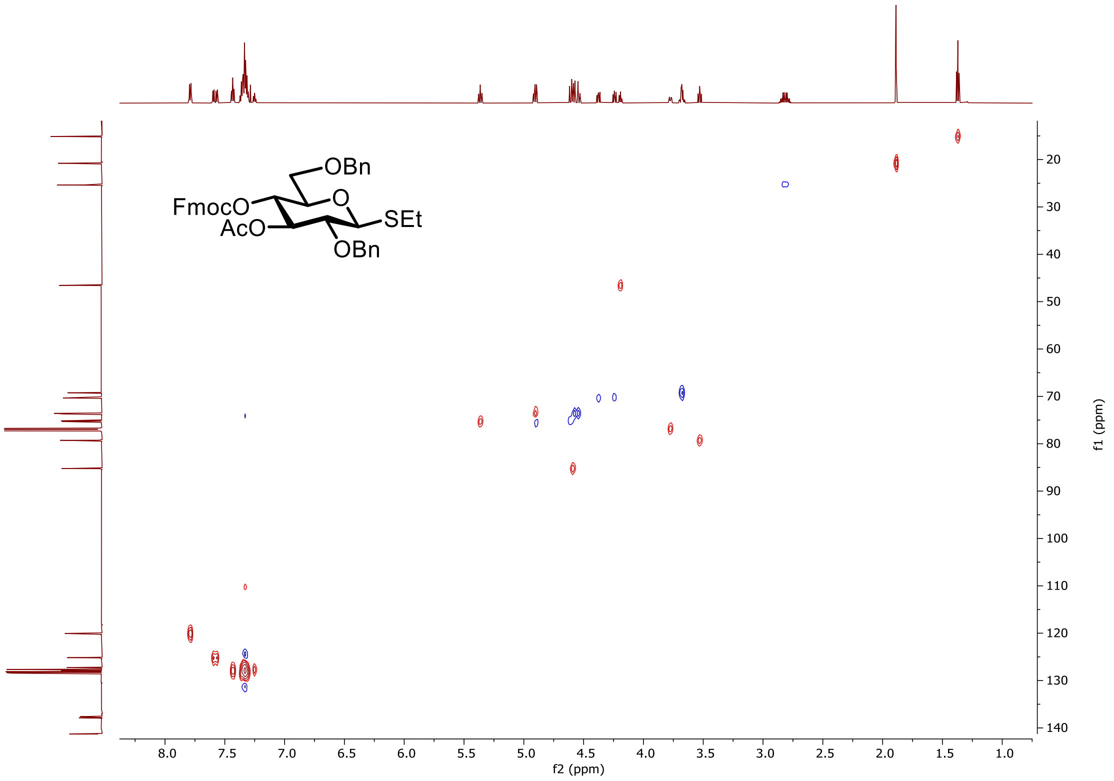

2.23 Synthesis of 111

Ethyl 2,6-di-*O*-benzyl-3-*O*-benzoyl-4-*O*-(9-fluorenylmethoxycarbonyl)-1-thio-β-D-glucopyranoside, 111

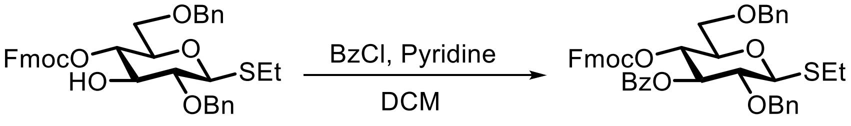

116 **111**

Ethyl 2,6-di-*O*-benzyl-4-*O*-(9-fluorenylmethoxycarbonyl)-1-thio-β-D-glucopyranoside **116** (626 mg, 1.0 mmol) was dissolved in anhydrous DCM (5 mL) and pyridine (1.5 mL) was added. The solution was cooled with an ice bath for 30 min, and benzoyl chloride (BzCl, 350 µL, 3.0 mmol) was added slowly. The reaction was warmed to room temperature and stirred for 16 h. Completion of the reaction was confirmed by TLC, MeOH (0.5 mL) was added. DCM (20 mL) was added 30 min later, and the organic phase was washed with aqueous citric acid (0.5 M, 20 mL). After extracting the water phase with DCM (20 mL), the organic layers were combined and dried over Na_2SO_4, filtered, and evaporated. The resulting crude product was purified by column chromatography (Hexane : EtOAc = 8:1→4:1) to give **111** as a white solid (700 mg, 96%). ^{1}H NMR (700 MHz, $CDCl_3$) δ 7.97 (d, *J* = 6.9 Hz, 2H), 7.75 – 7.70 (m, 2H), 7.53 – 7.48 (m, 1H), 7.46 (d, *J* = 7.5 Hz, 1H), 7.43 – 7.30 (m, 9H), 7.28 – 7.23 (m, 2H), 7.20 – 7.16 (m, 3H), 7.15 – 7.11 (m, 3H), 5.64 (appt, *J* = 9.4 Hz, 1H), 5.10 (appt, *J* = 9.8 Hz, 1H), 4.84 (d, *J* = 10.9 Hz, 1H), 4.67 (d, *J* = 9.7 Hz, 1H), 4.63 – 4.58 (m, 2H), 4.55 (d, *J* = 12.1 Hz, 1H), 4.22 (dd, *J* = 10.5, 7.3 Hz, 1H), 4.06 (dd, *J* = 10.5, 7.9 Hz, 1H), 3.94 (appt, *J* = 7.6 Hz, 1H), 3.85 (ddd, *J* = 10.0, 5.0, 3.1 Hz, 1H), 3.74 – 3.64 (m, 3H), 2.90 – 2.78 (m, 2H), 1.39 (t, *J* = 7.4 Hz, 3H); ^{13}C NMR (176 MHz, $CDCl_3$) δ 165.58, 154.26, 143.32, 142.99, 141.15, 141.08, 137.86, 137.16, 133.19, 129.89, 129.44, 128.38, 128.33, 128.23, 127.80, 127.78, 127.76, 127.68, 127.66, 127.13, 125.24, 125.02, 119.89, 85.32, 79.01, 76.91, 75.97, 75.06, 73.60, 73.53, 70.27, 69.16, 46.45, 25.37, 15.12; $[\alpha]_D^{25}$ -4.64 (c = 1, $CHCl_3$); IR (neat) ν_{max} = 2870, 1752, 1270, 1090, 739 cm^{-1}; m/z (HRMS$^+$) $[M + Na]^+$ 753.2481 ($C_{44}H_{42}O_8SNa^+$ requires 753.2493).

^{1}H NMR of 111 (700 MHz, $CDCl_3$)

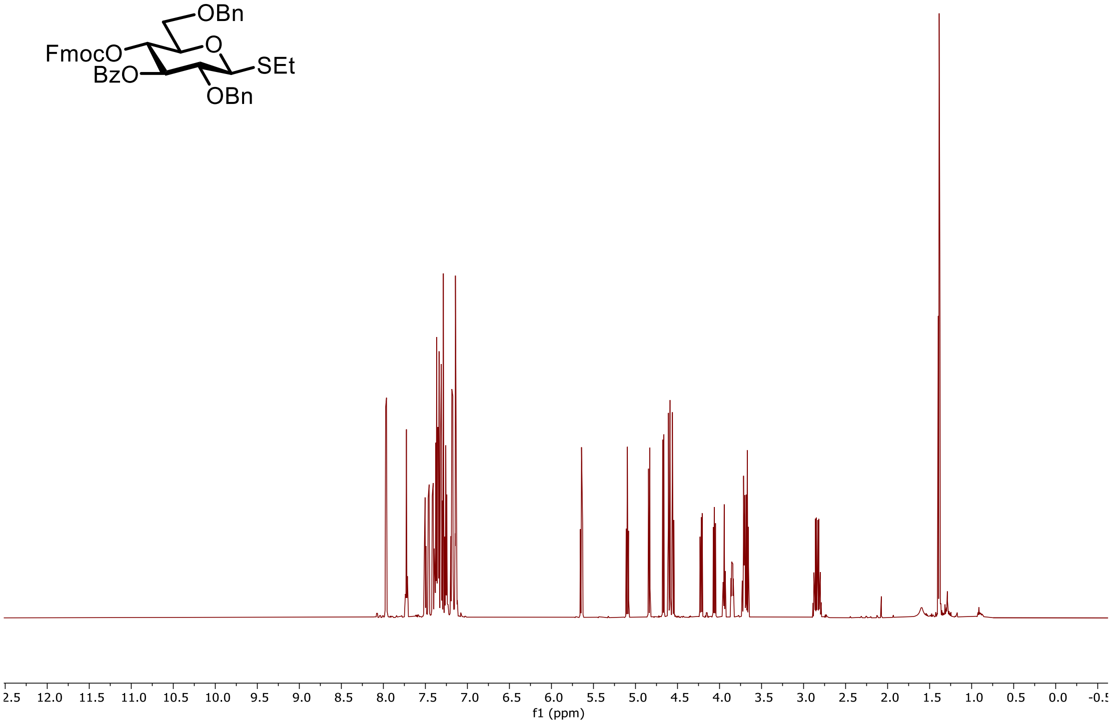

^{13}C NMR of 111 (176 MHz, $CDCl_3$)

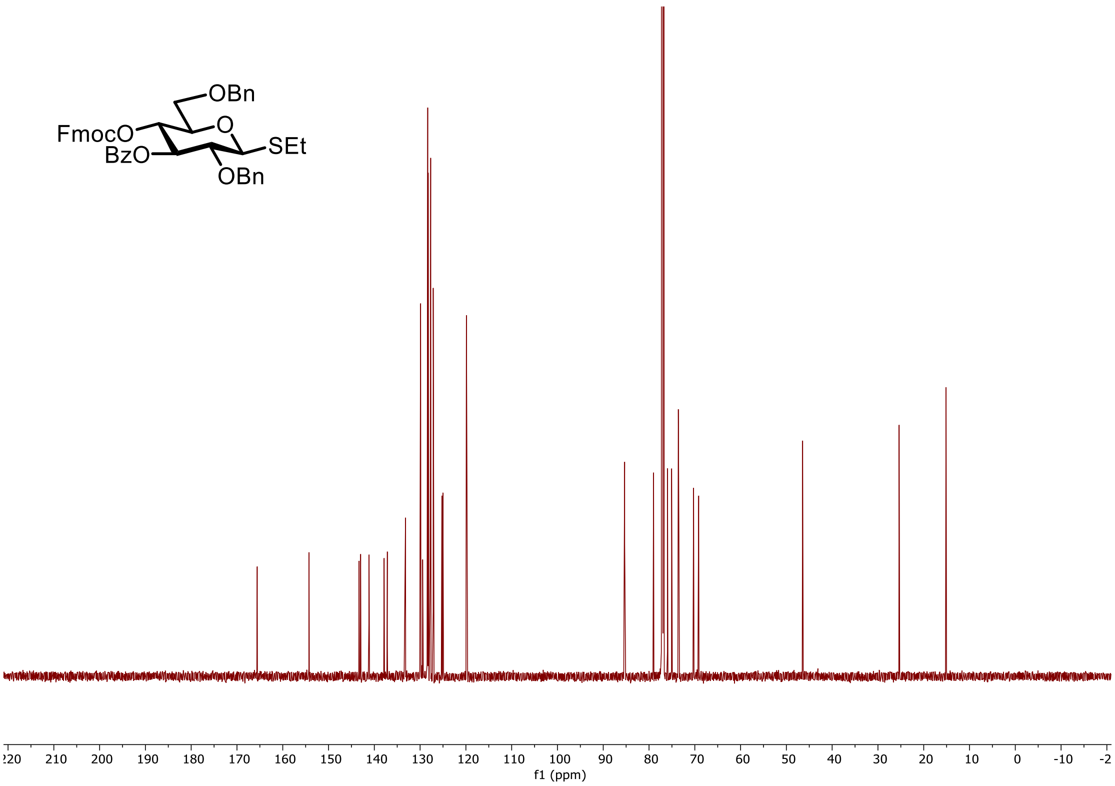

COSY NMR of 111 ($CDCl_3$)

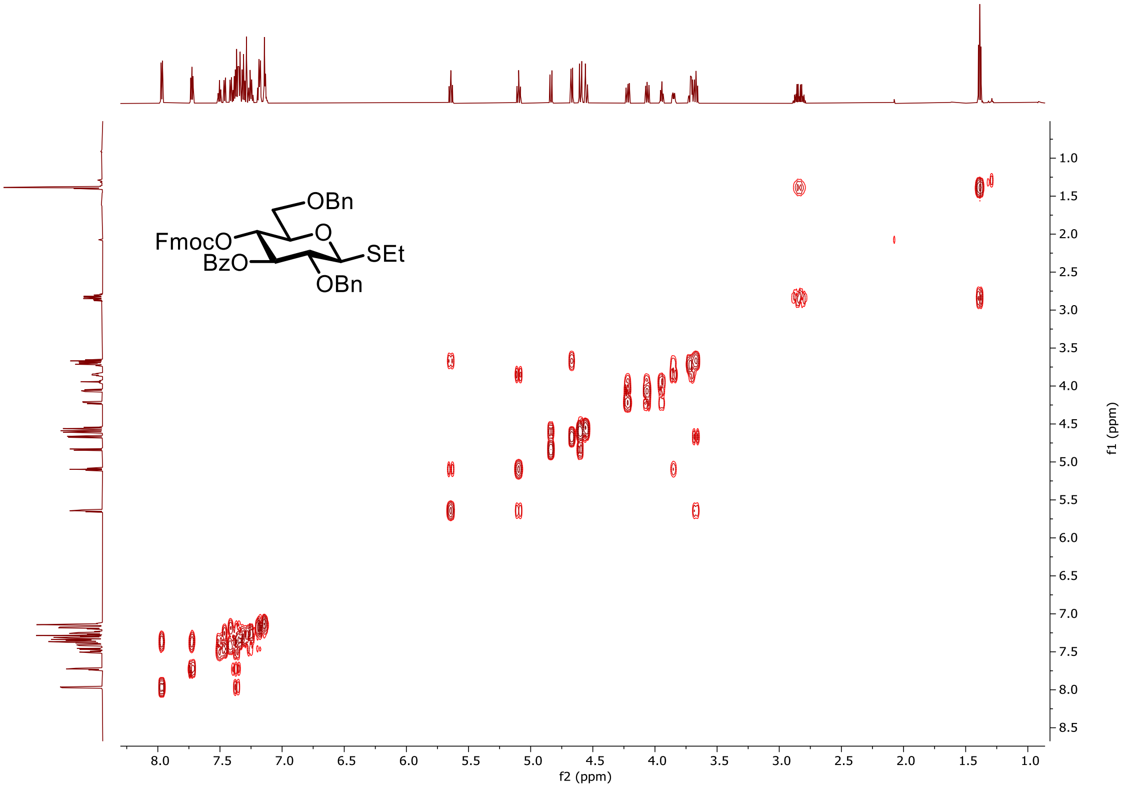

HSQC NMR of 111 ($CDCl_3$)

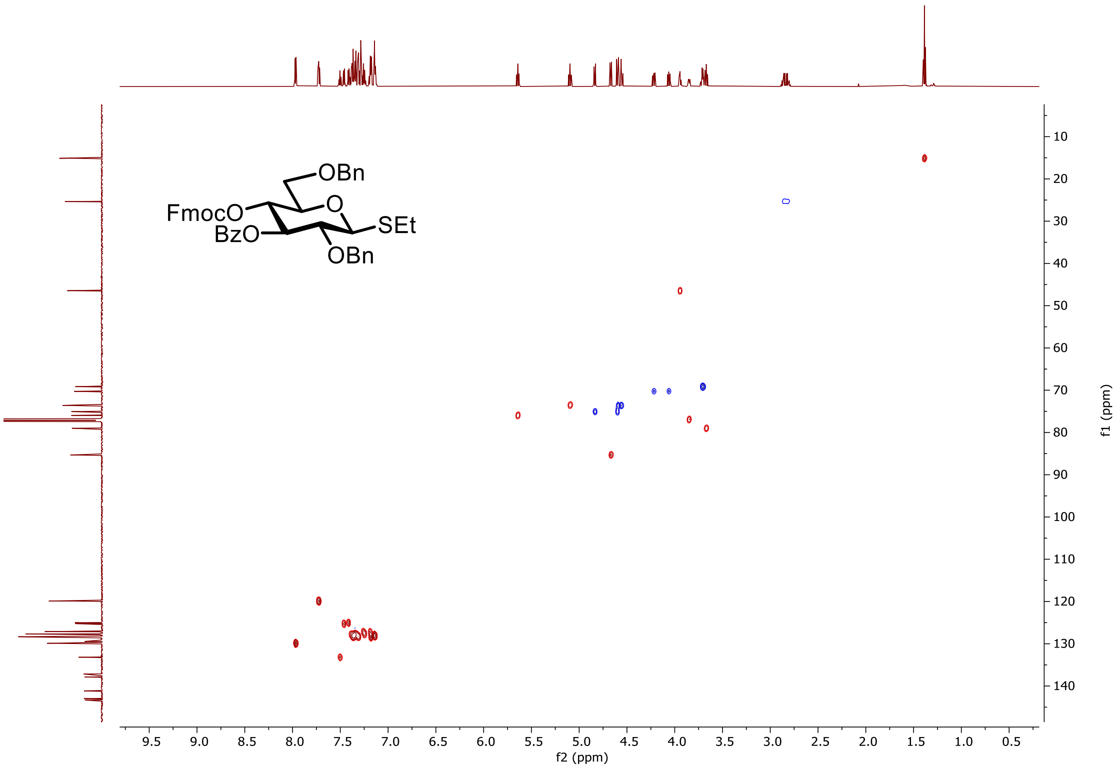

2.24 Synthesis of 112

Ethyl 2,6-di-*O*-benzyl-3-*O*-pivaloyl-4-*O*-(9-fluorenylmethoxycarbonyl)-1-thio-β-D-glucopyranoside, 112

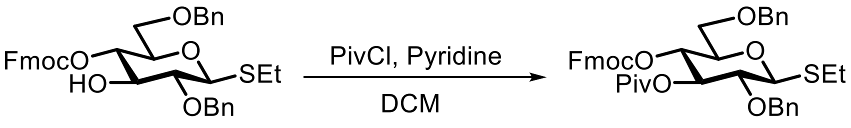

116 **112**

Ethyl 2,6-di-*O*-benzyl-4-*O*-(9-fluorenylmethoxycarbonyl)-1-thio-β-D-glucopyranoside **116** (626 mg, 1.0 mmol) was dissolved in anhydrous DCM (5 mL) and pyridine (1.5 mL) was added. The solution was cooled with an ice bath for 30 min, and pivaloyl chloride (PivCl, 367 µL, 3.0 mmol) was added slowly. The reaction was warmed to room temperature and stirred for 48 h, and MeOH (0.5 mL) was added. DCM (20 mL) was added 30 min later, and the organic phase was washed with aqueous citric acid (0.5 M, 20 mL). After extracting the water phase with DCM (20 mL), the organic layers were combined and dried over Na_2SO_4, filtered, and evaporated. The resulting crude product was purified by column chromatography (Hexane : EtOAc = 8:1→4:1) to give **112** as a white solid (170 mg, 24%). ^{1}H NMR (700 MHz, $CDCl_3$) δ 7.79 (dd, *J* = 7.6, 3.2 Hz, 2H), 7.61 (d, *J* = 7.5 Hz, 1H), 7.57 (d, *J* = 7.5 Hz, 1H), 7.43 (tt, *J* = 7.5, 1.4 Hz, 2H), 7.37 – 7.32 (m, 10H), 7.31 – 7.28 (m, 1H), 7.27 – 7.23 (m, 1H), 5.44 (appt, *J* = 9.4 Hz, 1H), 4.98 (appt, *J* = 9.8 Hz, 1H), 4.92 (d, *J* = 10.6 Hz, 1H), 4.63 – 4.54 (m, 4H), 4.32 – 4.24 (m, 2H), 4.17 (appt, *J* = 7.6 Hz, 1H), 3.79 (ddd, *J* = 10.0, 4.9, 3.6 Hz, 1H), 3.72 – 3.66 (m, 2H), 3.55 (appt, *J* = 9.4 Hz, 1H), 2.87 – 2.75 (m, 2H), 1.36 (t, *J* = 7.4 Hz, 3H), 1.12 (s, 9H); ^{13}C NMR (176 MHz, $CDCl_3$) δ 177.20, 154.29, 143.28, 143.09, 141.29, 141.21, 137.87, 137.56, 128.33, 127.96, 127.92, 127.74, 127.67, 127.54, 127.32, 127.22, 125.34, 125.20, 120.06, 120.01, 85.14, 79.56, 76.85, 75.00, 74.91, 73.82, 73.60, 70.36, 69.37, 46.56, 38.81, 27.00, 25.29, 15.11; $[\alpha]_D^{25}$ 3.23 (c = 1, $CHCl_3$); IR (neat) ν_{max} = 2872, 1751, 1254, 737 cm^{-1}; m/z (HRMS$^+$) $[M + Na]^+$ 733.2821 ($C_{42}H_{46}O_8SNa^+$ requires 733.2806).

^{1}H NMR of 112 (700 MHz, $CDCl_3$)

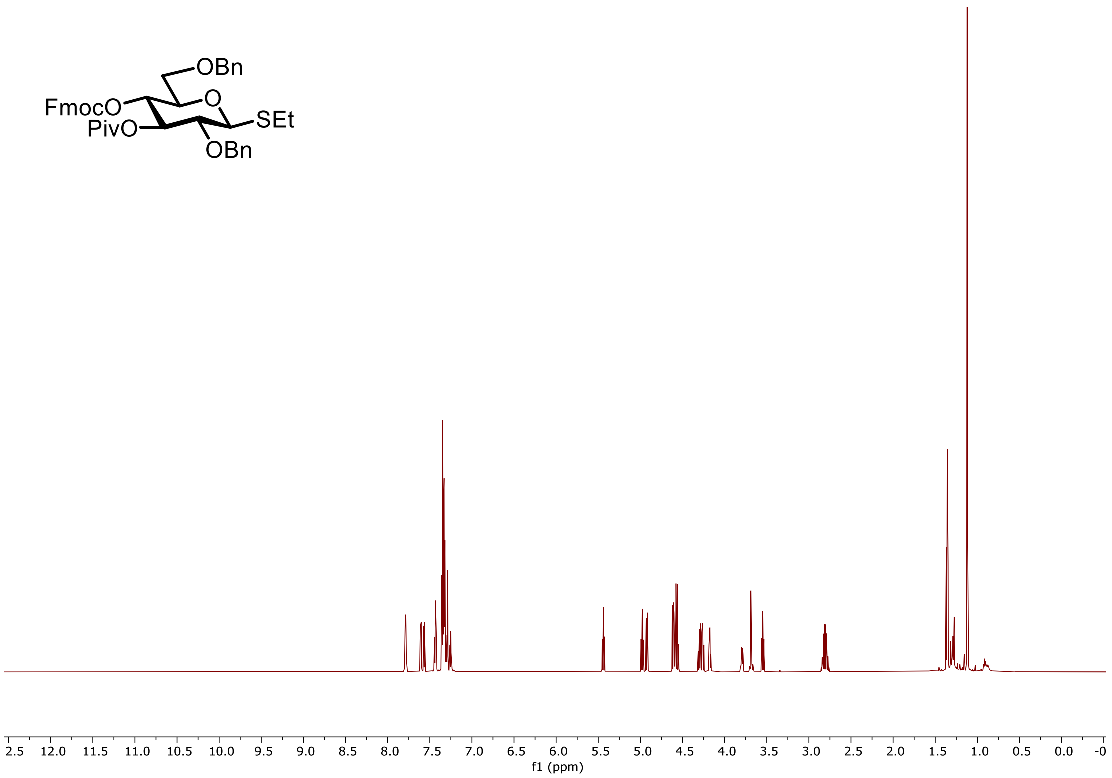

^{13}C NMR of 112 (176 MHz, $CDCl_3$)

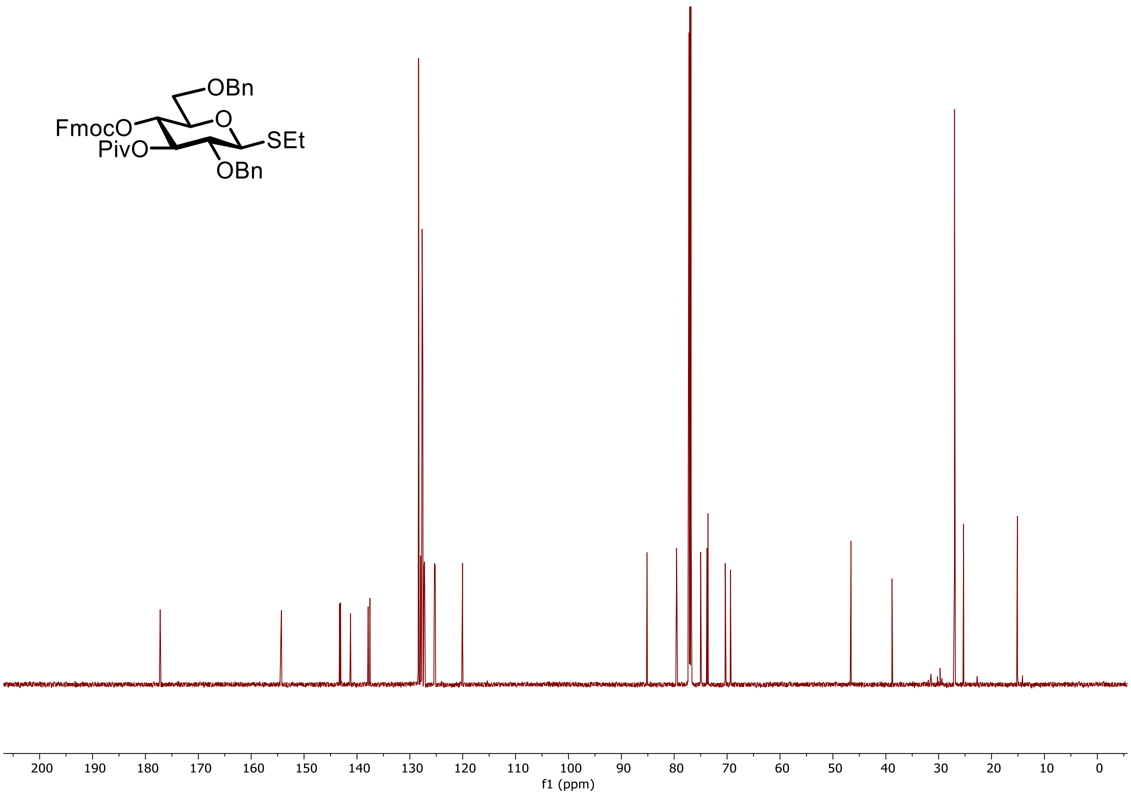

COSY NMR of 112 ($CDCl_3$)

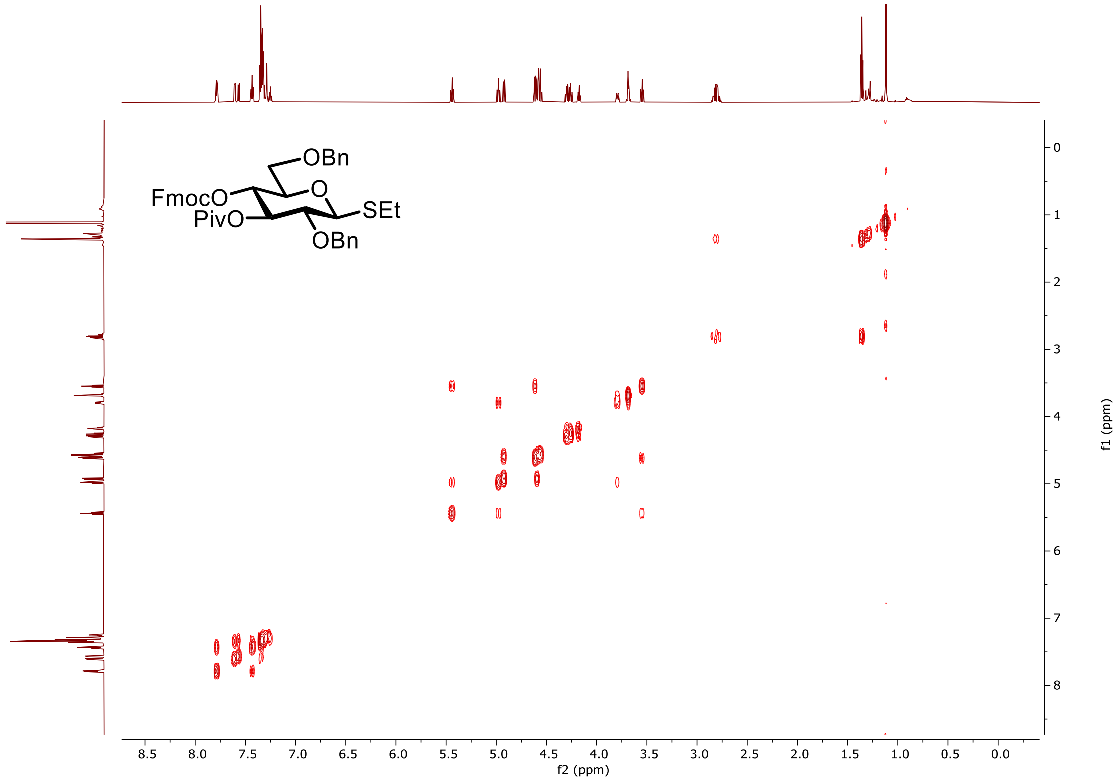

HSQC NMR of 112 ($CDCl_3$)

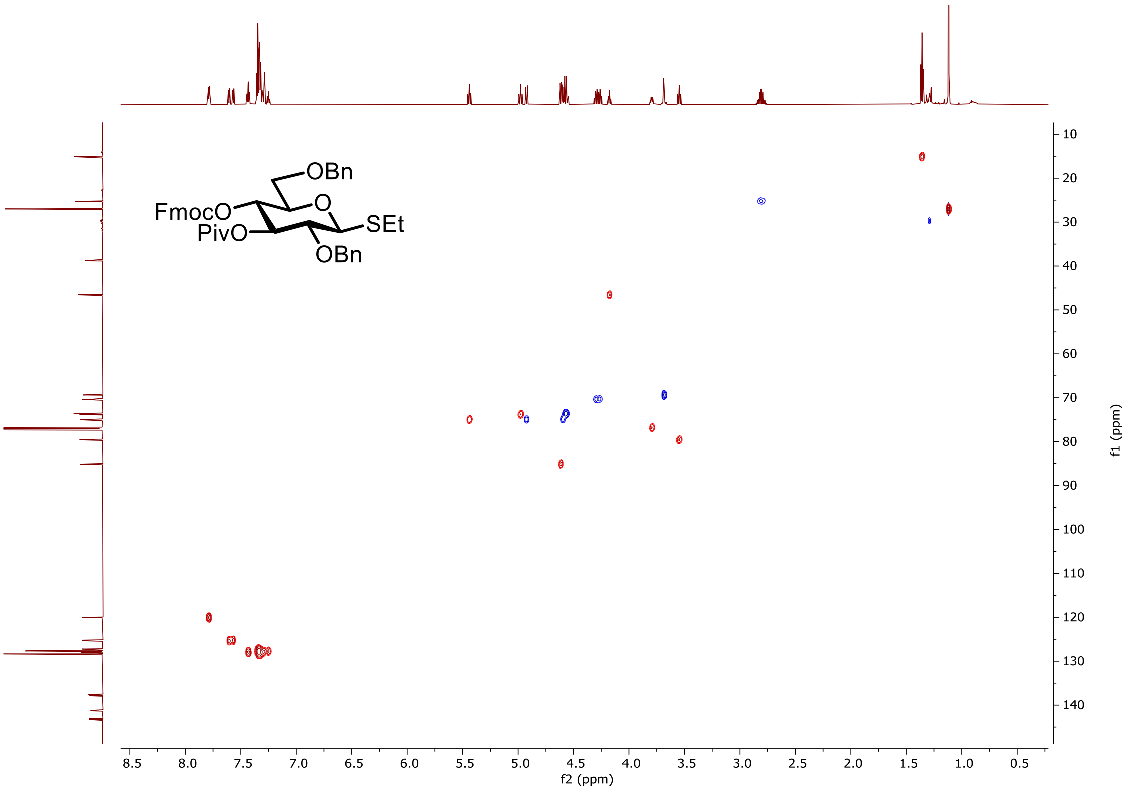

3. General Methods for Solution-phase Glycosylation Study

3.1 Method A: Glycosylation with Isopropanol as Nucleophile

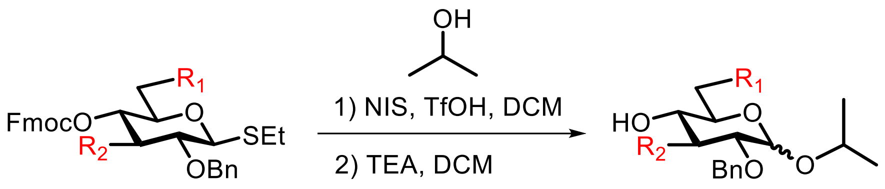

12-32 and **110-112** **74-94** and **117-119**

Thioglycoside (0.10 mmol) was dissolved in anhydrous DCM (1 mL) and 4Å molecular sieve (100 mg) was added. Next, isopropanol (15.3 µL, 0.20 mmol) and *N*-iodosuccinimide (NIS, 34 mg, 0.15 mmol) were added, the reaction mixture was stirred at room temperature for 30 min and cooled to -15 °C under argon protection. Trifluoromethanesulfonic acid (TfOH, 1.8 µL, 20 µmol) was slowly added and the reaction was left for 5 min at -15 °C, then warmed to 0 °C and kept for 60 min. Triethylamine (TEA, 0.2 mL) was added to quench the reaction and cleave the Fmoc protecting group. After 16 h, DCM (4 mL) was added to dilute the reaction and the organic phase was washed with aqueous citric acid (0.5 M, 2 mL), dried over Na_2SO_4, filtered, and evaporated. The resulting crude product was passed through a fast column (Hexane-EtOAc solvent system) to remove any non-carbohydrate impurity, the obtained mixture is directly used for NMR study to quantify the ratio of α/β anomers (**74-94** and **117-119**). After that, the mixture was carefully purified again by column chromatography (Toluene-EtOAc solvent system) to get the pure monosaccharide anomer (only the major products are characterized).

For the temperature gradient experiment, the entire glycosylation was performed at a specific temperature for 60 min. All the other conditions are same.

For the solvent control experiment, the glycosylation was performed in a specific anhydrous solvent (or solvent mixture) instead of pure DCM. All the other conditions are same.

3.2 Method B: Glycosylation with Monosaccharide as Nucleophile

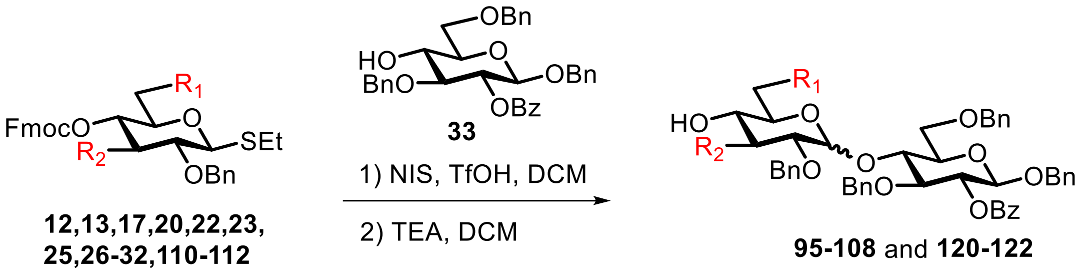

33 was prepared according to previously established procedures. [4]

Thioglycoside (0.10 mmol) was dissolved in anhydrous DCM (1 mL) and 4Å molecular sieve (100 mg) was added. Next, benzyl 2-*O*-benzoyl-3,6-di-*O*-benzyl-β-D-glucopyranoside **33** (66.6 mg, 0.12 mmol) and *N*-iodosuccinimide (NIS, 34 mg, 0.15 mmol) were added, the reaction mixture was stirred at room temperature for 30 min and cooled to -15 °C under argon protection. Trifluoromethanesulfonic acid (TfOH, 1.8 µL, 20 µmol) was slowly added and the reaction was left for 5 min at -15 °C, then warmed to 0 °C and kept for 60 min. Triethylamine (TEA, 0.2 mL) was added to quench the reaction and cleave the Fmoc protecting group. After 16 h, DCM (4 mL) was added to dilute the reaction and the organic phase was washed with aqueous citric acid (0.5 M, 2 mL), dried over Na_2SO_4, filtered, and evaporated. The resulting crude product was passed through a fast column (Hexane-EtOAc solvent system) to remove any non-carbohydrate impurity, the obtained mixture is directly used for NMR study to quantify the ratio of α/β anomers (**95-108** and **120-122**). After that, the mixture was carefully purified again by column chromatography (Toluene-EtOAc solvent system) to get the pure disaccharide anomer (only the major products are characterized).

4. Characterization of Solution-phase Glycosylation Products

4.1 Isopropyl 2,3,6-tri-*O*-benzyl-D-glucopyranoside, 74

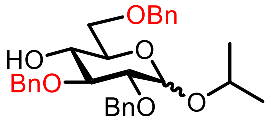

R_1=R_2=OBn

Total yield: 93% (45.8 mg). Ratio of anomer α : β = 1 : 2.9.

Spectrum data for isopropyl 2,3,6-tri-*O*-benzyl-β-D-glucopyranoside **74b**:

^{1}H NMR (400 MHz, $CDCl_3$) δ 7.43 – 7.27 (m, 15H), 5.01 (d, *J* = 10.9 Hz, 1H), 4.96 (d, *J* = 11.4 Hz, 1H), 4.76 (d, *J* = 6.3 Hz, 1H), 4.73 (d, *J* = 5.8 Hz, 1H), 4.62 (d, *J* = 2.7 Hz, 2H), 4.52 (d, *J* = 7.2 Hz, 1H), 4.05 (hept, *J* = 6.2 Hz, 1H), 3.80 (dd, *J* = 10.4, 3.9 Hz, 1H), 3.72 (dd, *J* = 10.3, 5.6 Hz, 1H), 3.60 (dd, *J* = 9.6, 8.1 Hz, 1H), 3.52 – 3.39 (m, 3H), 2.58 (br. s, 1H), 1.34 (d, *J* = 6.2 Hz, 3H), 1.28 (d, *J* = 6.1 Hz, 3H); ^{13}C NMR (101 MHz, $CDCl_3$) δ 138.67, 138.45, 137.99, 128.59, 128.45, 128.43, 128.27, 128.02, 127.87, 127.76, 127.73, 127.70, 102.24, 84.18, 81.73, 75.31, 74.79, 73.92, 73.65, 72.50, 71.71, 70.55, 23.75, 22.26; $[\alpha]_D^{25}$ -16.30 (c = 1, $CHCl_3$); IR (neat) ν_{max} = 2870, 1455, 1059, 697 cm^{-1}; m/z (HRMS$^+$) $[M + Na]^+$ 515.2399 ($C_{30}H_{36}O_6Na^+$ requires 515.2404).

^{1}H NMR of crude 74 (400 MHz, $CDCl_3$)

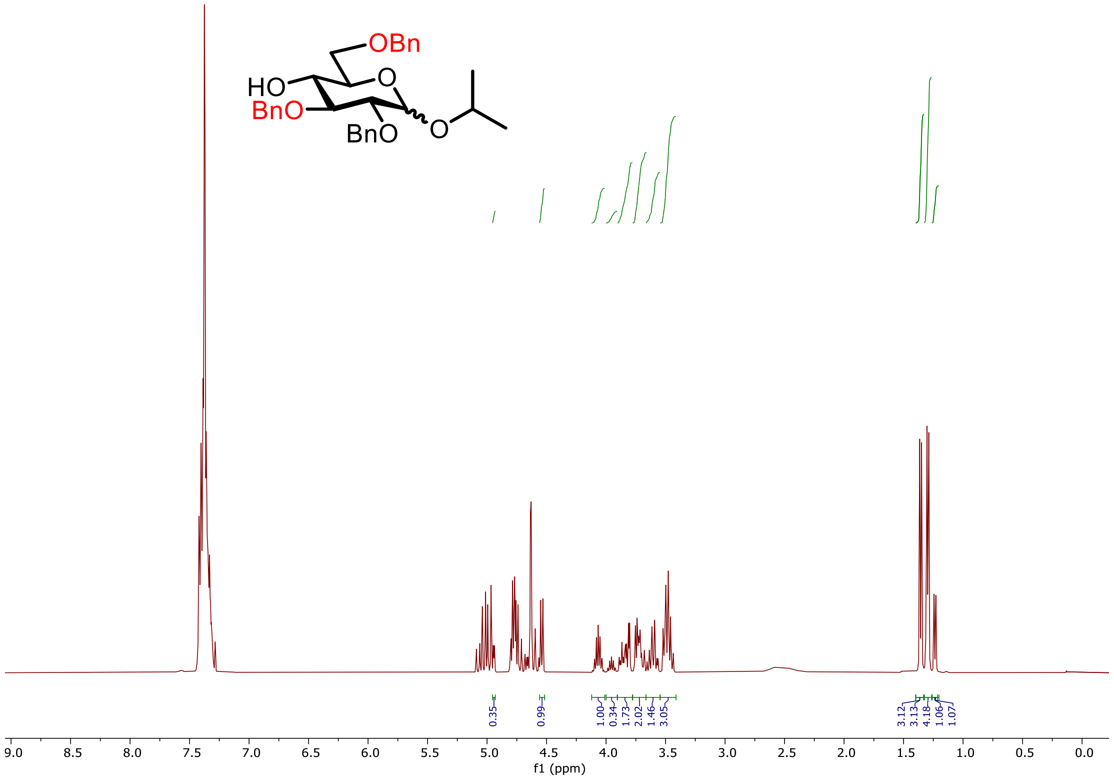

^{13}C NMR of crude 74 (101 MHz, $CDCl_3$)

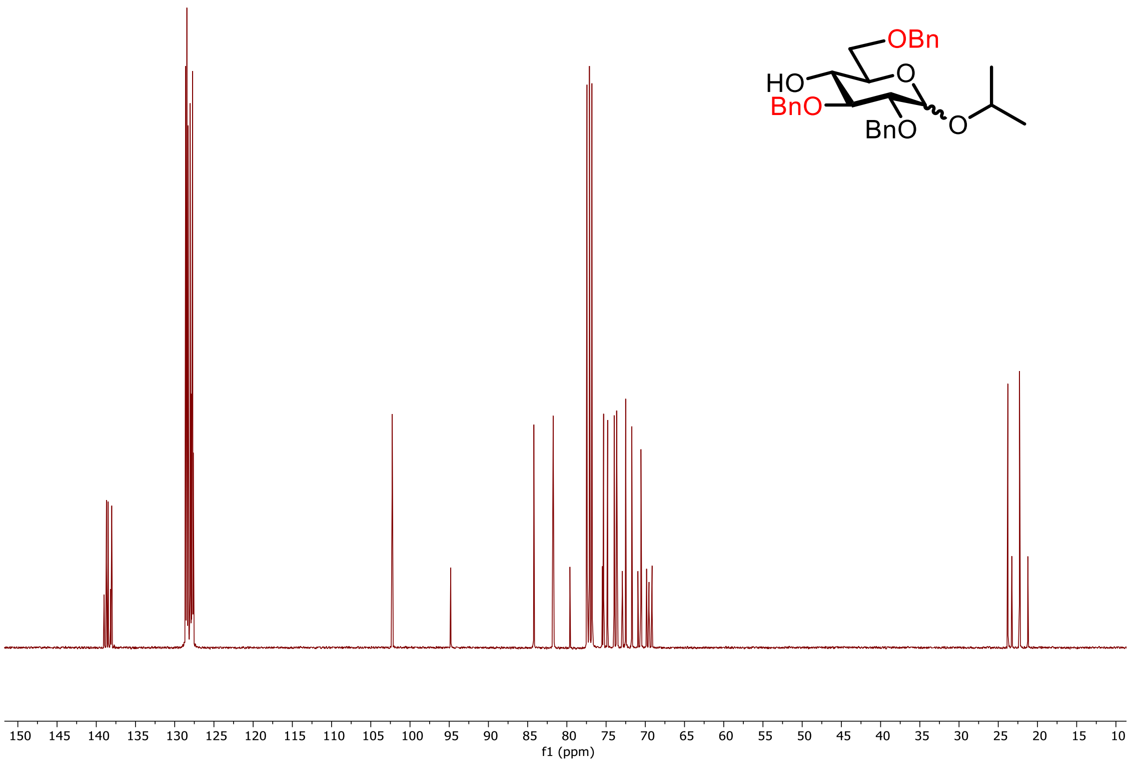

HSQC NMR of crude 74 ($CDCl_3$)

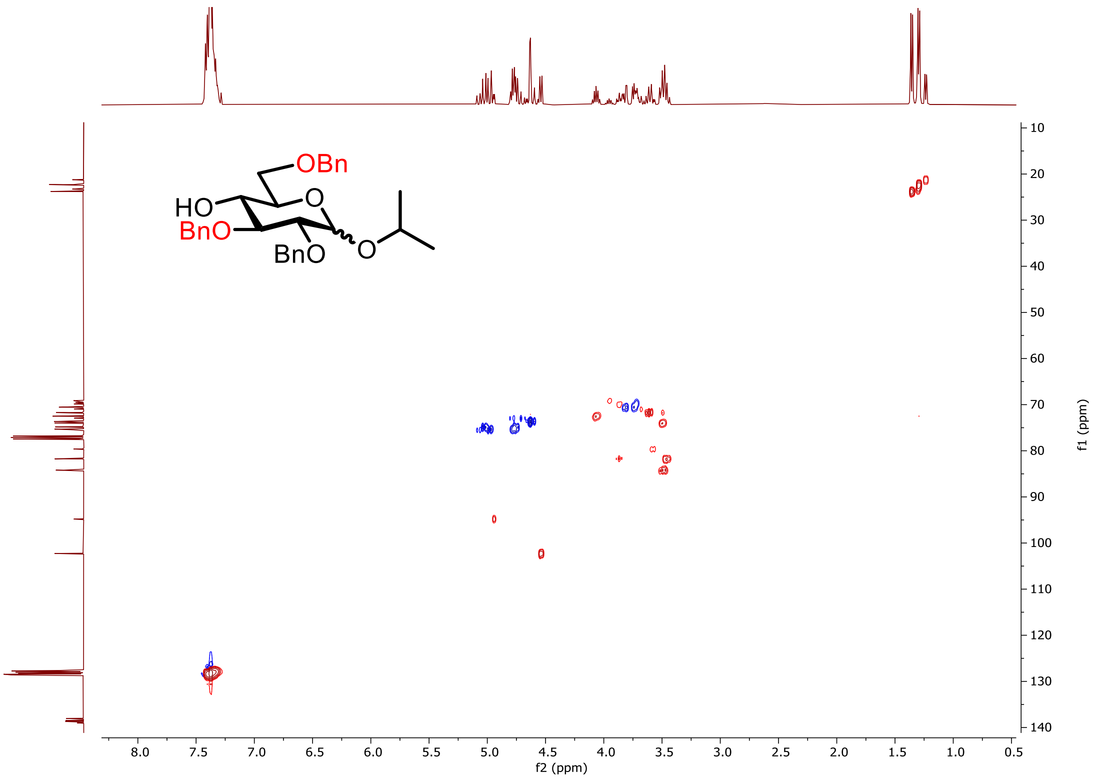

Coupled HSQC NMR of crude 74 ($CDCl_3$)

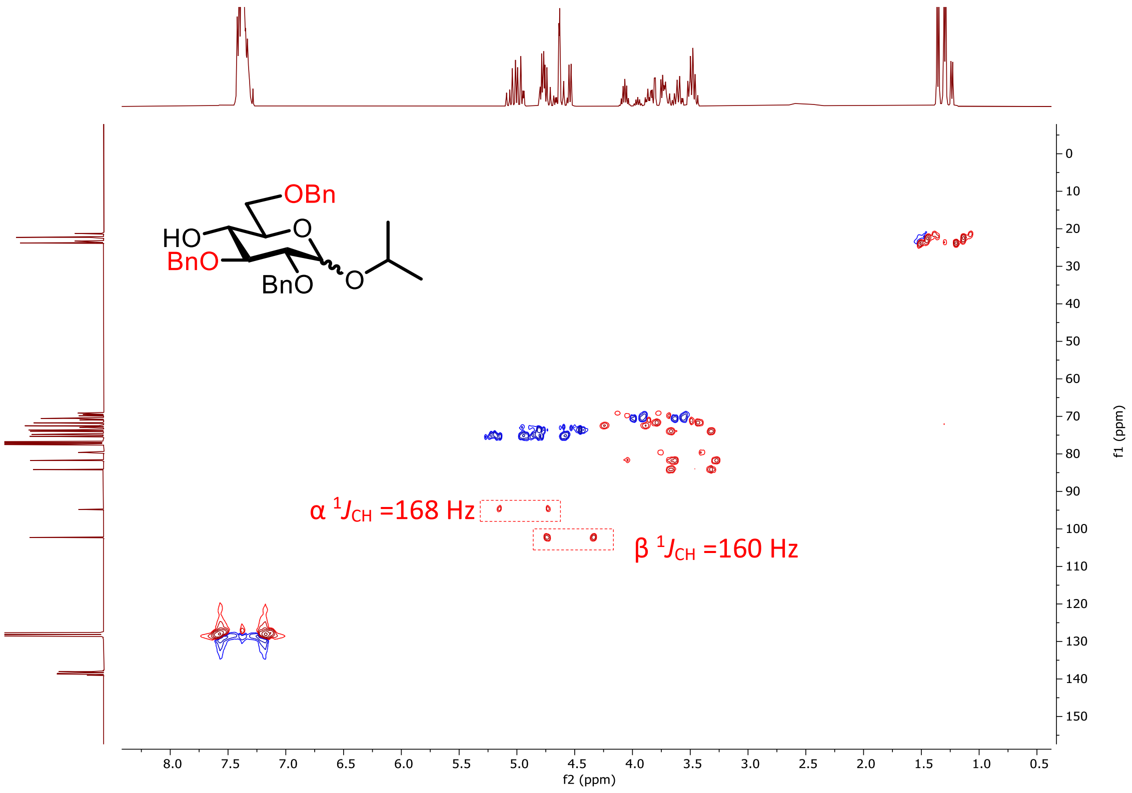

^{1}H NMR of 74b (400 MHz, $CDCl_3$)

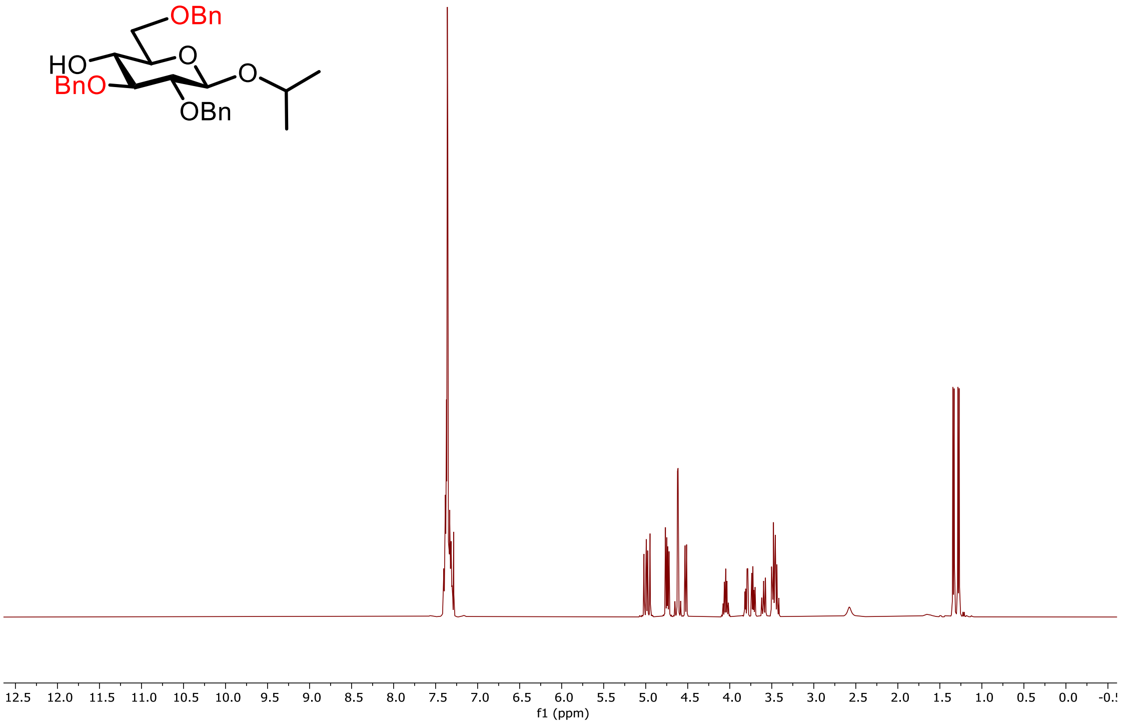

^{13}C NMR of 74b (101 MHz, $CDCl_3$)

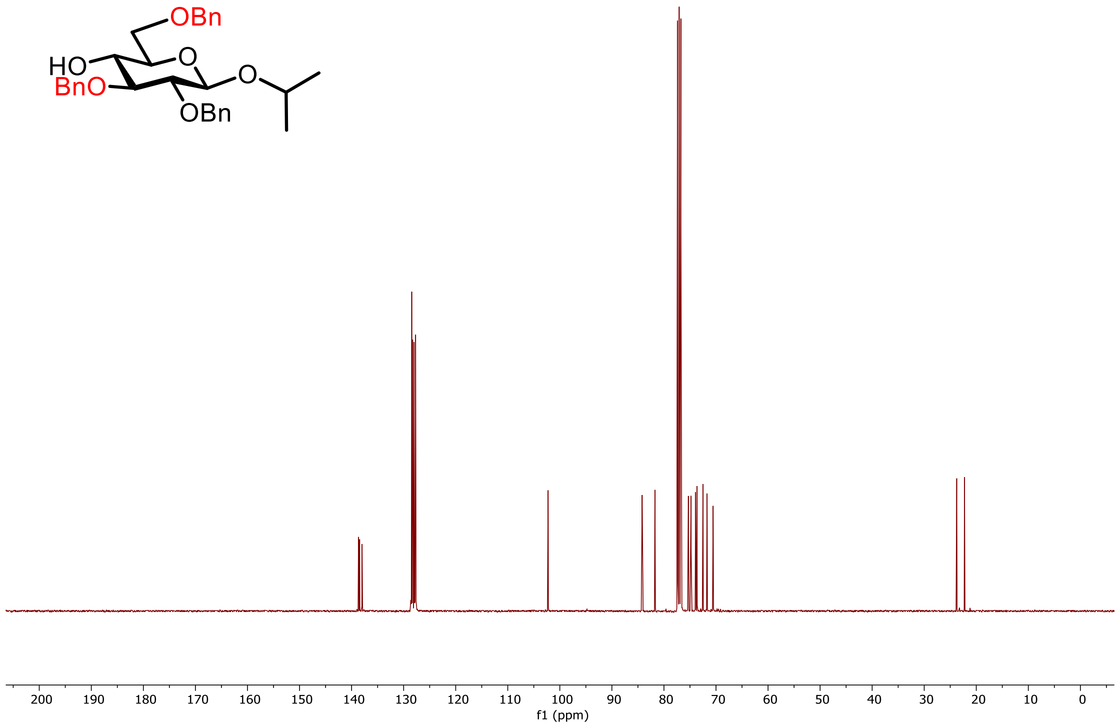

COSY NMR of 74b ($CDCl_3$)

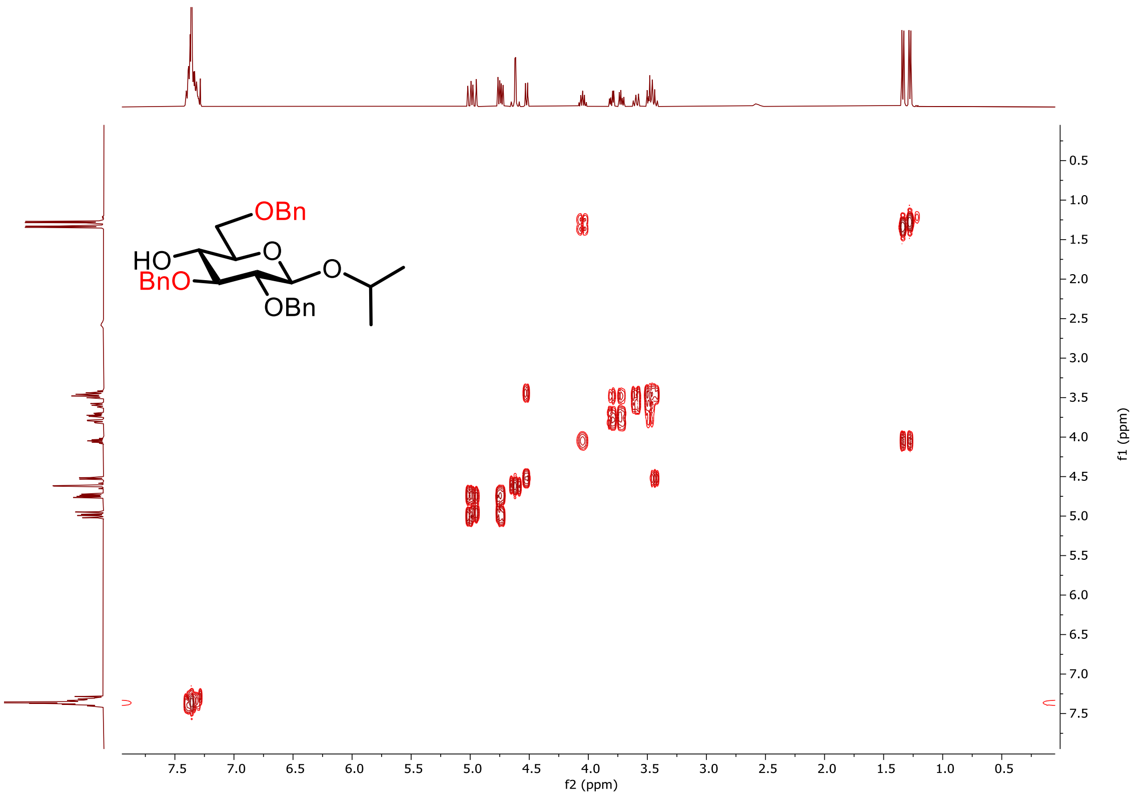

HSQC NMR of 74b ($CDCl_3$)

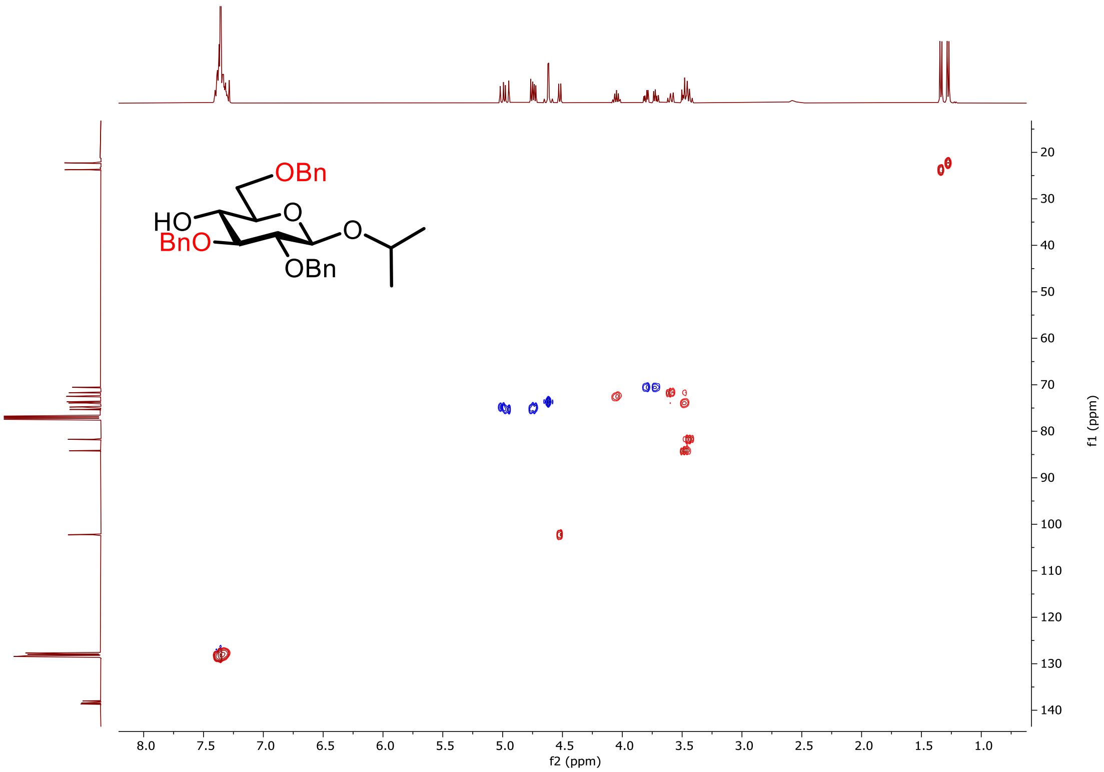

4.2 Isopropyl 2,3-di-*O*-benzyl-6-*O*-acetyl-D-glucopyranoside, 75

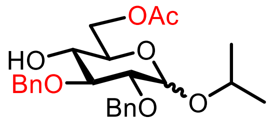

R$_1$=OAc; R$_2$=OBn

Total yield: 95% (42.2 mg). Ratio of anomer α : β = 2.3 : 1.

Spectrum data for isopropyl 2,3-di-*O*-benzyl-6-*O*-acetyl-α-D-glucopyranoside **75a**:

^{1}H NMR (400 MHz, $CDCl_3$) δ 7.44 – 7.27 (m, 10H), 5.04 (d, *J* = 11.2 Hz, 1H), 4.90 (d, *J* = 3.7 Hz, 1H), 4.77 (dd, *J* = 11.6, 2.5 Hz, 2H), 4.68 (d, *J* = 12.0 Hz, 1H), 4.46 (dd, *J* = 12.1, 4.6 Hz, 1H), 4.22 (dd, *J* = 12.1, 2.2 Hz, 1H), 3.97 – 3.80 (m, 3H), 3.52 (dd, *J* = 9.6, 3.7 Hz, 1H), 3.45 (dd, *J* = 10.0, 8.9 Hz, 1H), 2.56 (br. s, 1H), 2.10 (s, 3H), 1.28 (d, *J* = 6.3 Hz, 3H), 1.22 (d, *J* = 6.1 Hz, 3H); ^{13}C NMR (101 MHz, $CDCl_3$) δ 171.48, 138.76, 138.03, 128.64, 128.53, 128.19, 128.02, 127.92, 94.98, 81.26, 79.46, 75.61, 72.98, 70.05, 69.60, 69.27, 63.30, 23.16, 21.29, 20.89; $[\alpha]_D^{25}$ 26.82 (c = 1, $CHCl_3$); IR (neat) ν_{max} = 1742, 1242, 1058, 699 cm^{-1}; m/z (HRMS$^+$) $[M + Na]^+$ 467.2016 ($C_{25}H_{32}O_7Na^+$ requires 467.2040).

^{1}H NMR of crude 75 (400 MHz, $CDCl_3$)

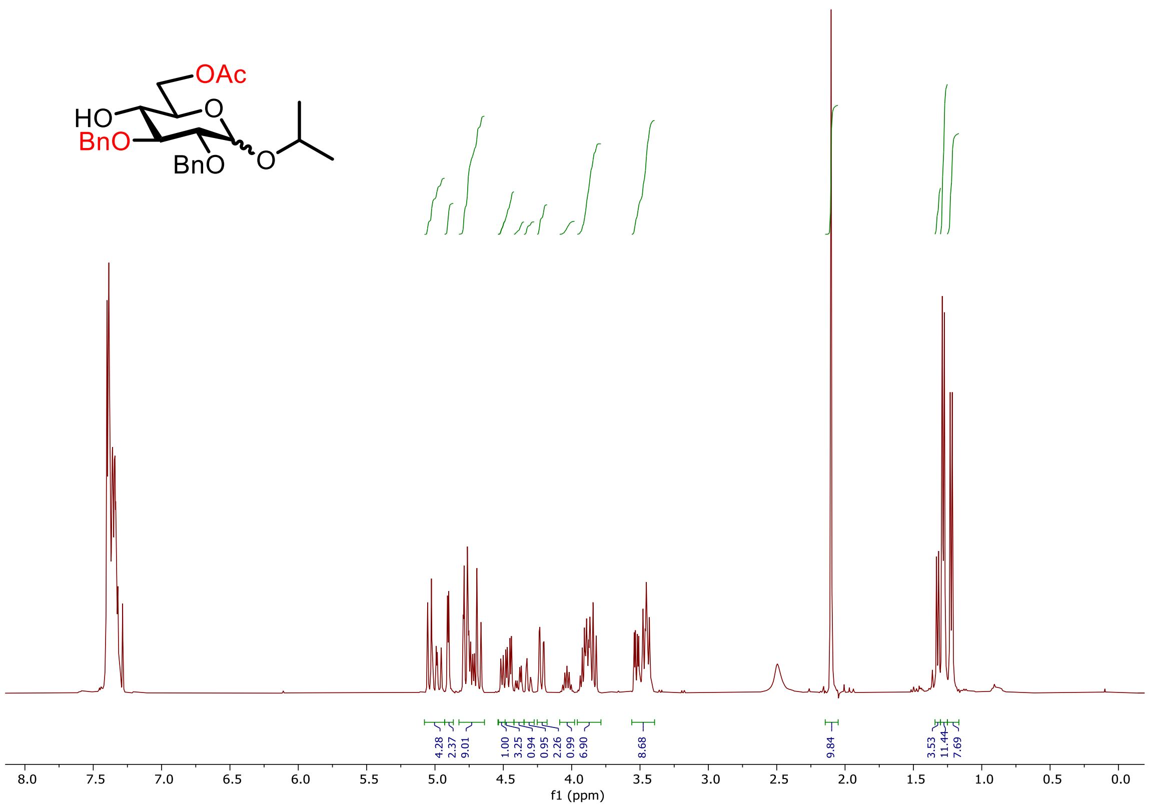

^{13}C NMR of crude 75 (101 MHz, $CDCl_3$)

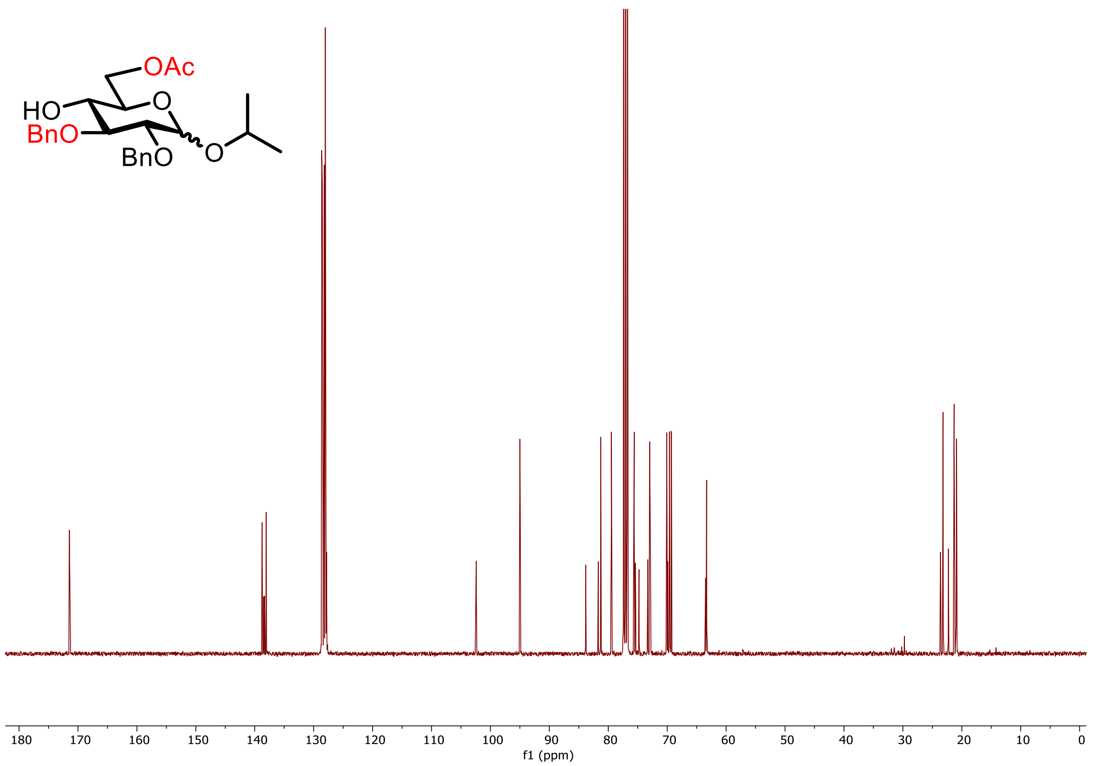

HSQC NMR of crude 75 ($CDCl_3$)

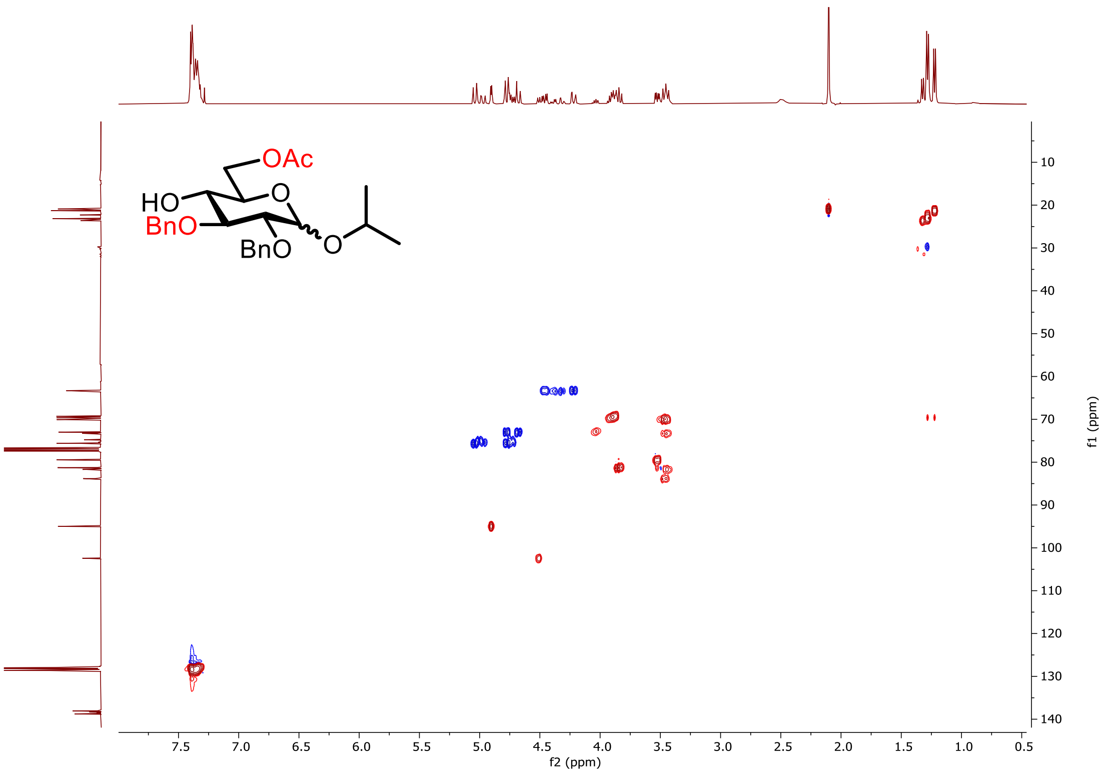

Coupled HSQC NMR of crude 75 ($CDCl_3$)

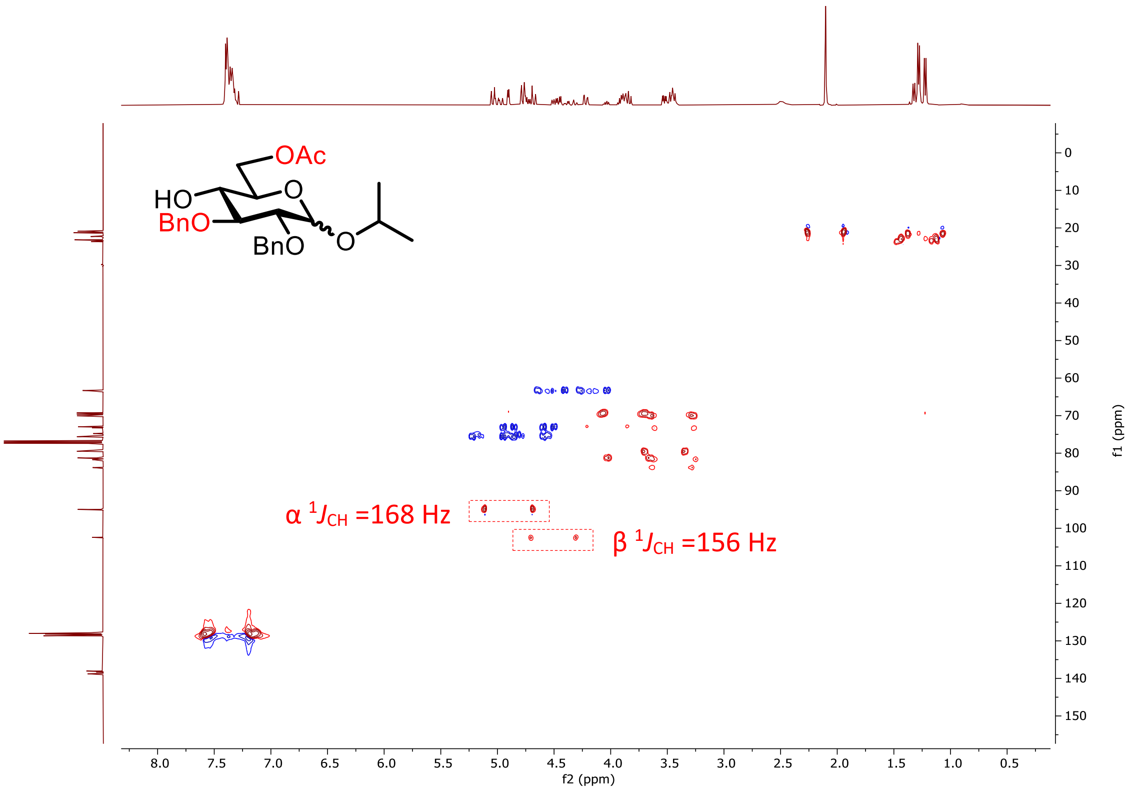

^{1}H NMR of 75a (400 MHz, $CDCl_3$)

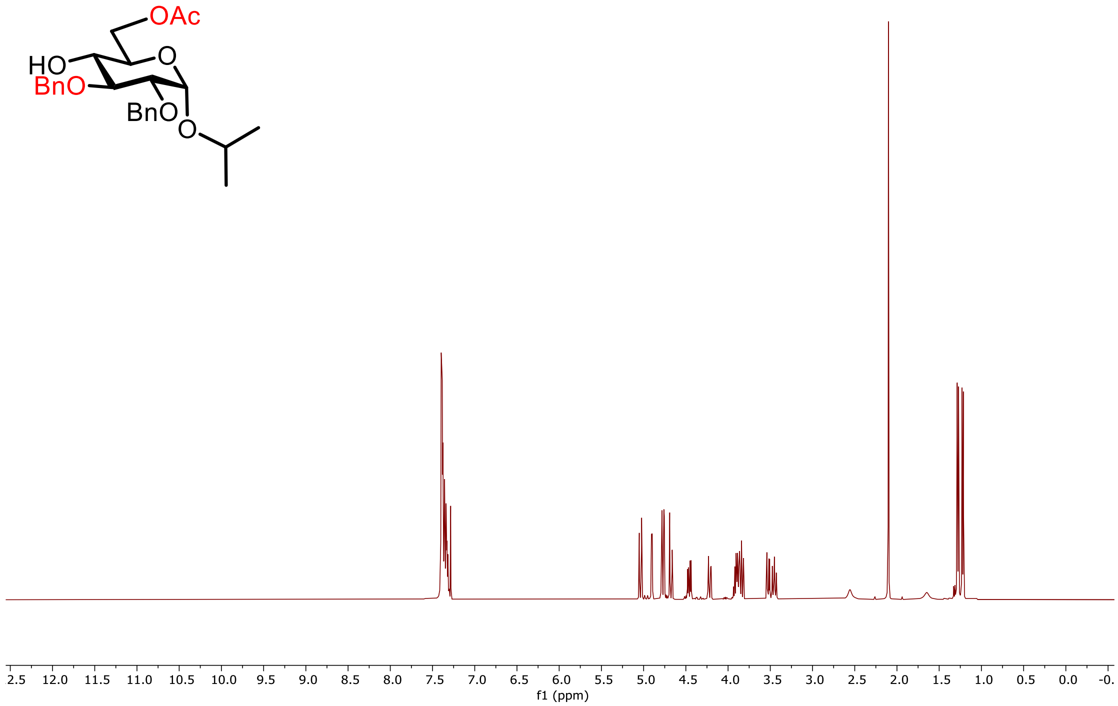

^{13}C NMR of 75a (101 MHz, $CDCl_3$)

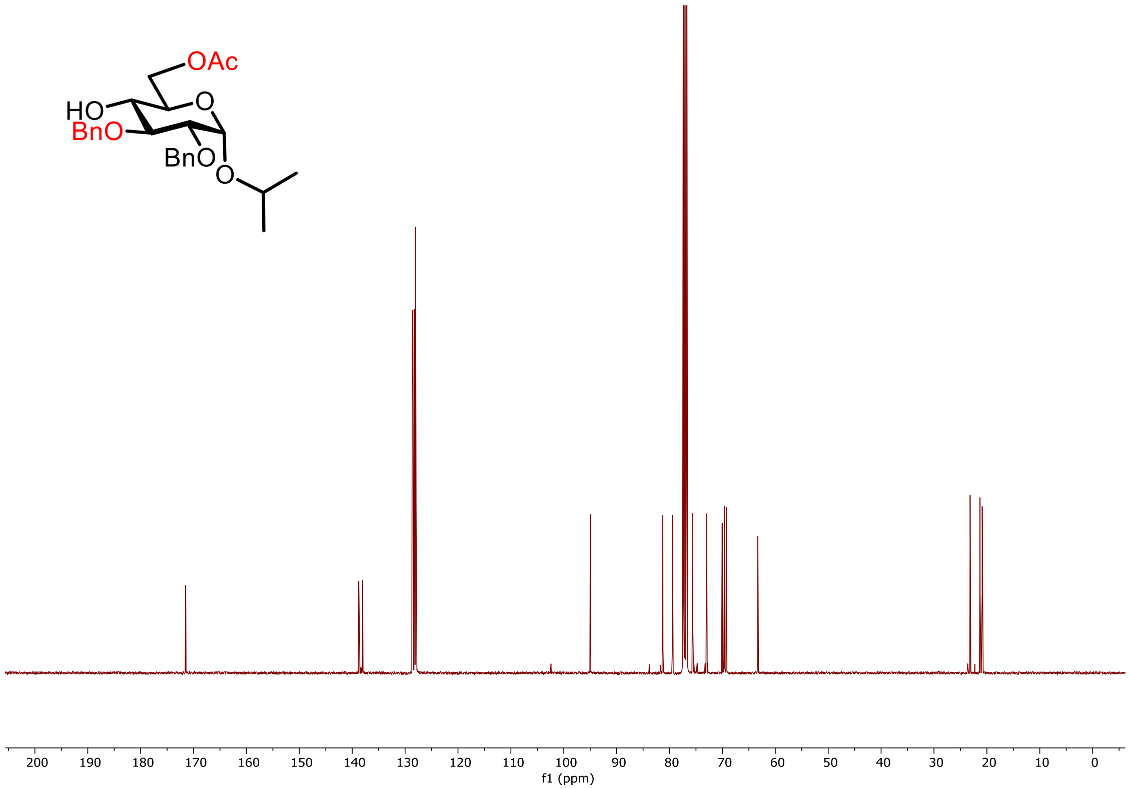

COSY NMR of 75a ($CDCl_3$)

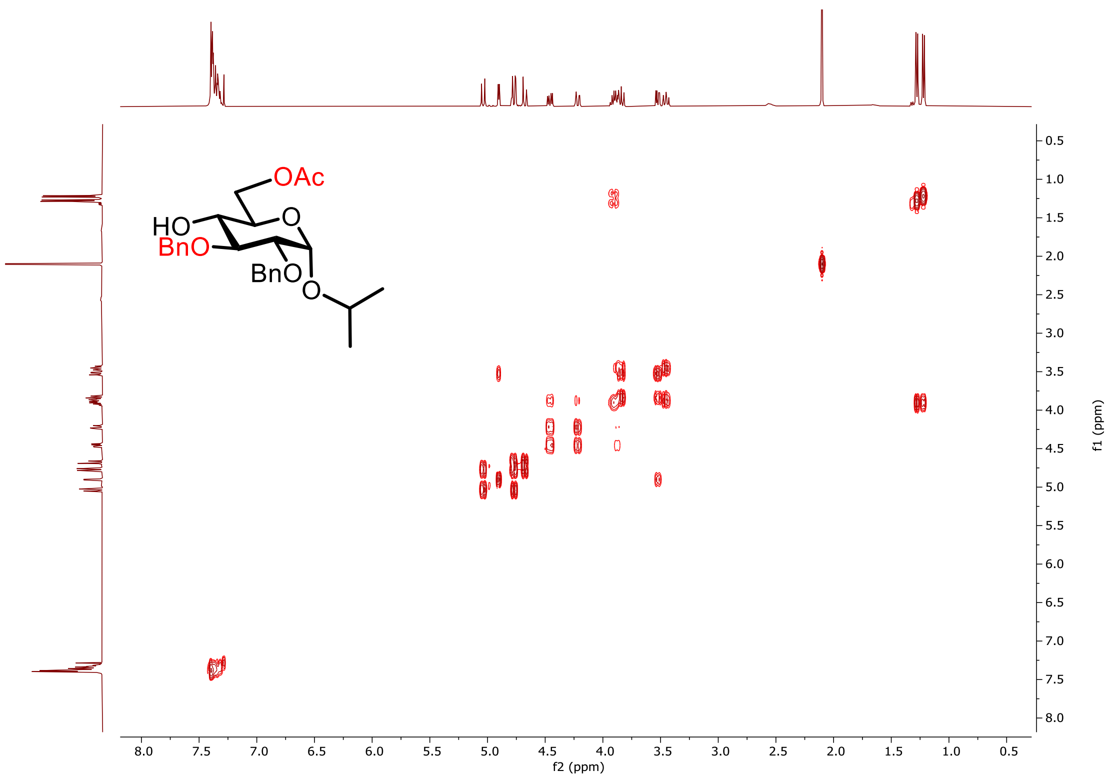

HSQC NMR of 75a ($CDCl_3$)

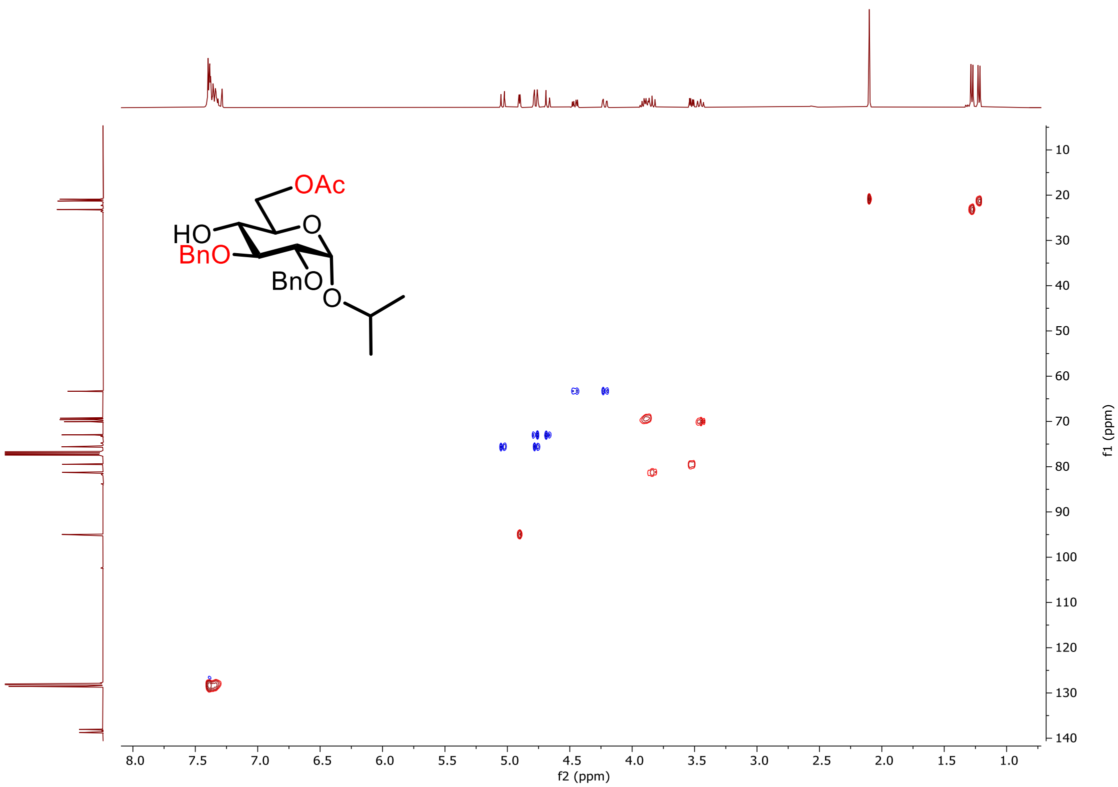

4.3 Isopropyl 2,3-di-*O*-benzyl-6-*O*-formyl-D-glucopyranoside, 76

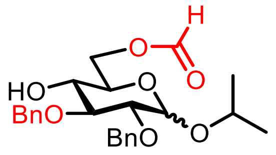

Total yield: 95% (40.9 mg). Ratio of anomer α : β = 2.0 : 1.

Spectrum data for isopropyl 2,3-di-*O*-benzyl-6-*O*-formyl-α-D-glucopyranoside **76a**: ^{1}H NMR (400 MHz, $CDCl_3$) δ 8.11 (s, 1H), 7.41 – 7.30 (m, 10H), 5.06 (d, *J* = 11.4 Hz, 1H), 4.90 (d, *J* = 3.7 Hz, 1H), 4.79 – 4.64 (m, 3H), 4.46 (ddd, *J* = 11.9, 5.0, 0.9 Hz, 1H), 4.36 (ddd, *J* = 12.0, 2.3, 0.8 Hz, 1H), 3.95 – 3.87 (m, 2H), 3.86 – 3.80 (m, 1H), 3.53 (dd, *J* = 9.5, 3.7 Hz, 1H), 3.47 (dd, *J* = 10.1, 8.8 Hz, 1H), 2.65 (br. s, 1H), 1.28 (d, *J* = 6.3 Hz, 3H), 1.22 (d, *J* = 6.1 Hz, 3H); ^{13}C NMR (101 MHz, $CDCl_3$) δ 161.04, 138.72, 137.94, 128.68, 128.54, 128.19, 128.05, 127.97, 127.96, 94.88, 81.26, 79.53, 75.48, 72.95, 69.98, 69.56, 68.86, 62.88, 23.19, 21.22; $[\alpha]_D^{25}$ 21.14 (c = 1, $CHCl_3$); IR (neat) ν_{max} = 2926, 1726, 1059, 689 cm^{-1}; m/z ($HRMS^+$) $[M + Na]^+$ 453.1857 ($C_{24}H_{30}O_7Na^+$ requires 453.1884).

^{1}H NMR of crude 76 (600 MHz, $CDCl_3$)

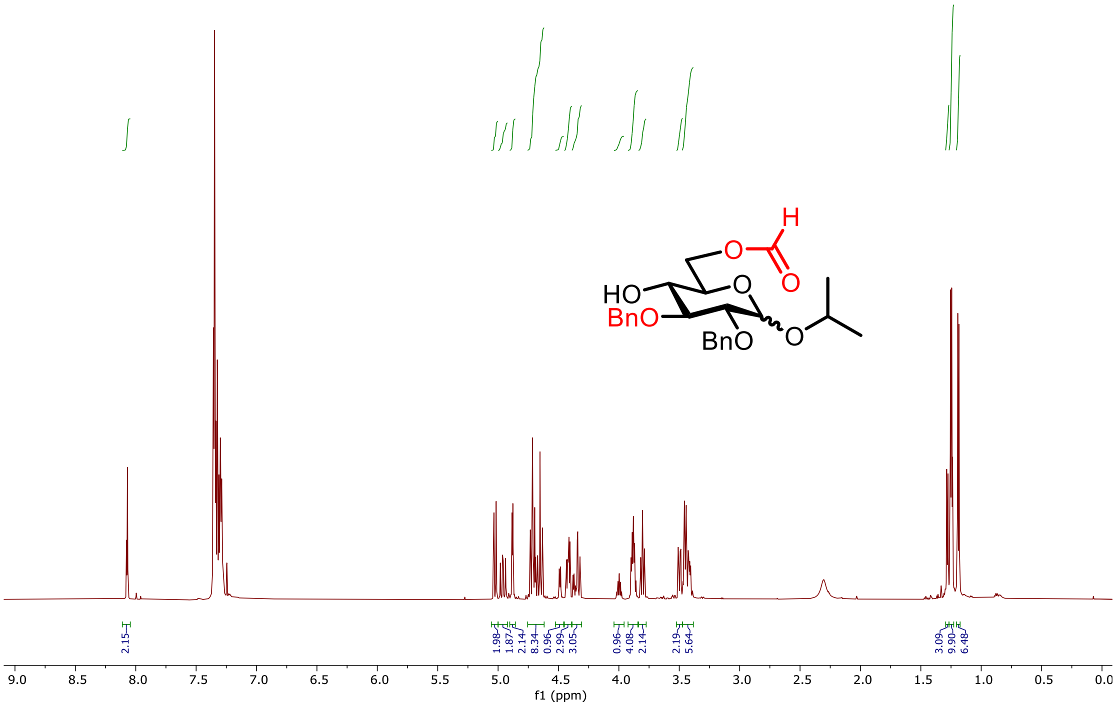

^{13}C NMR of crude 76 (151 MHz, $CDCl_3$)

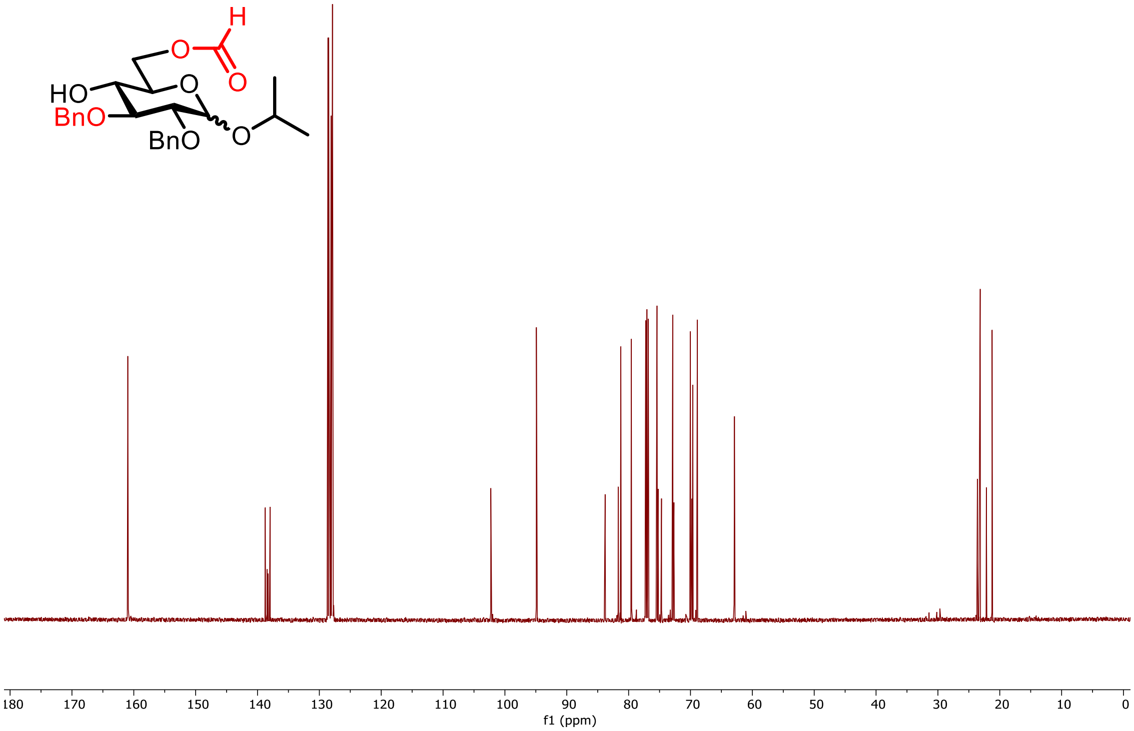

HSQC NMR of crude 76 ($CDCl_3$)

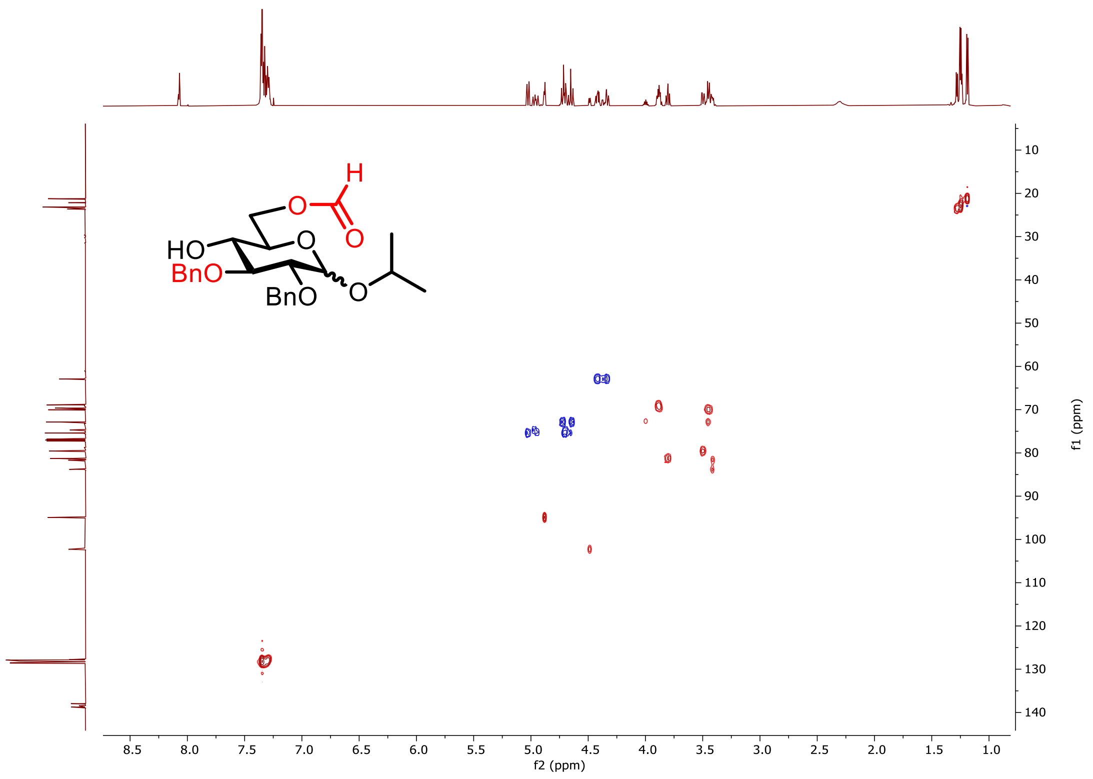

Coupled HSQC NMR of crude 76 ($CDCl_3$)

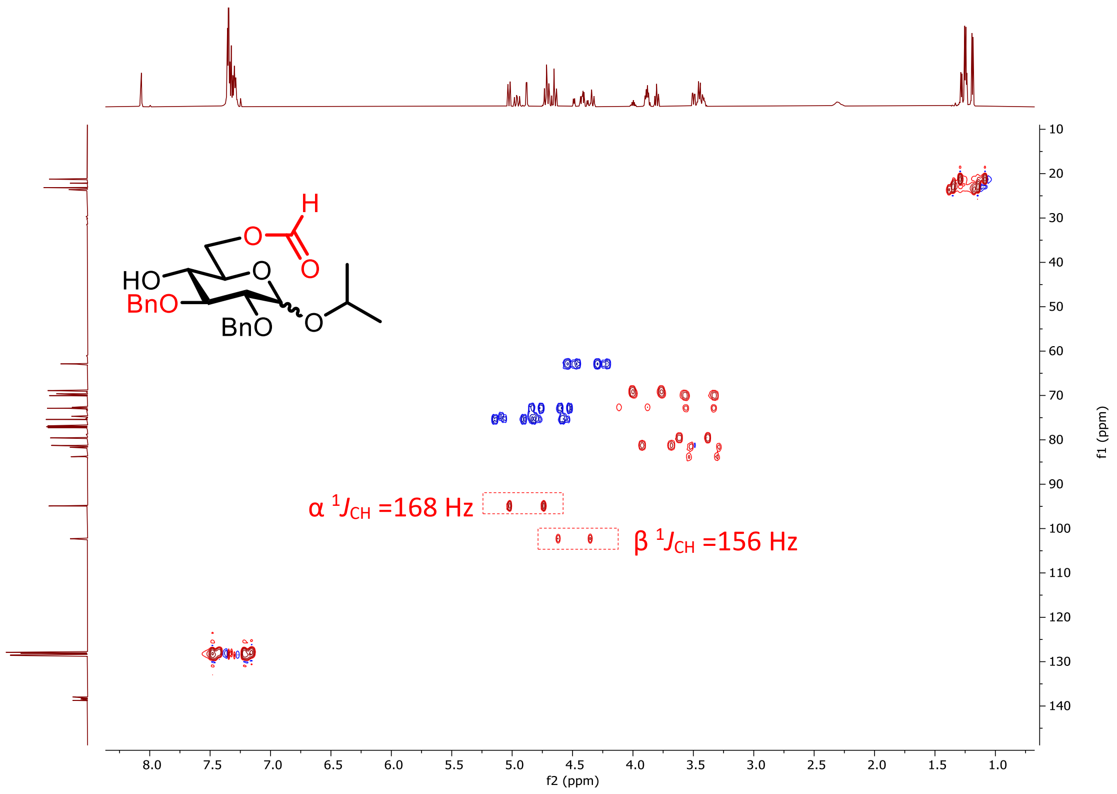

^{1}H NMR of 76a (400 MHz, $CDCl_3$)

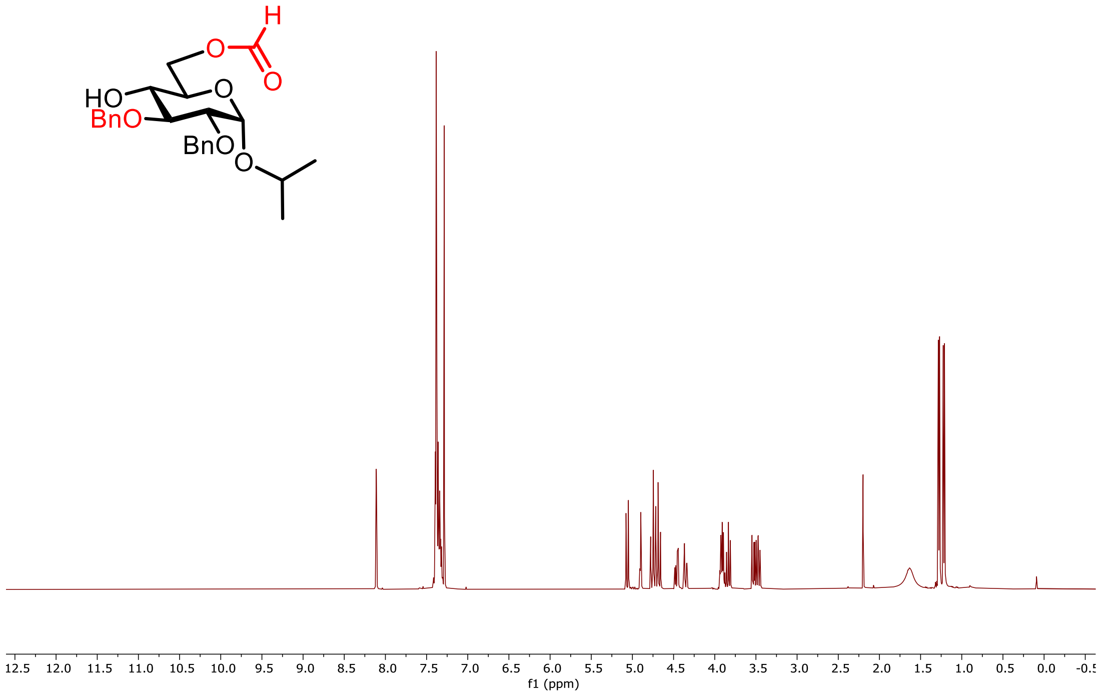

^{13}C NMR of 76a (101 MHz, $CDCl_3$)

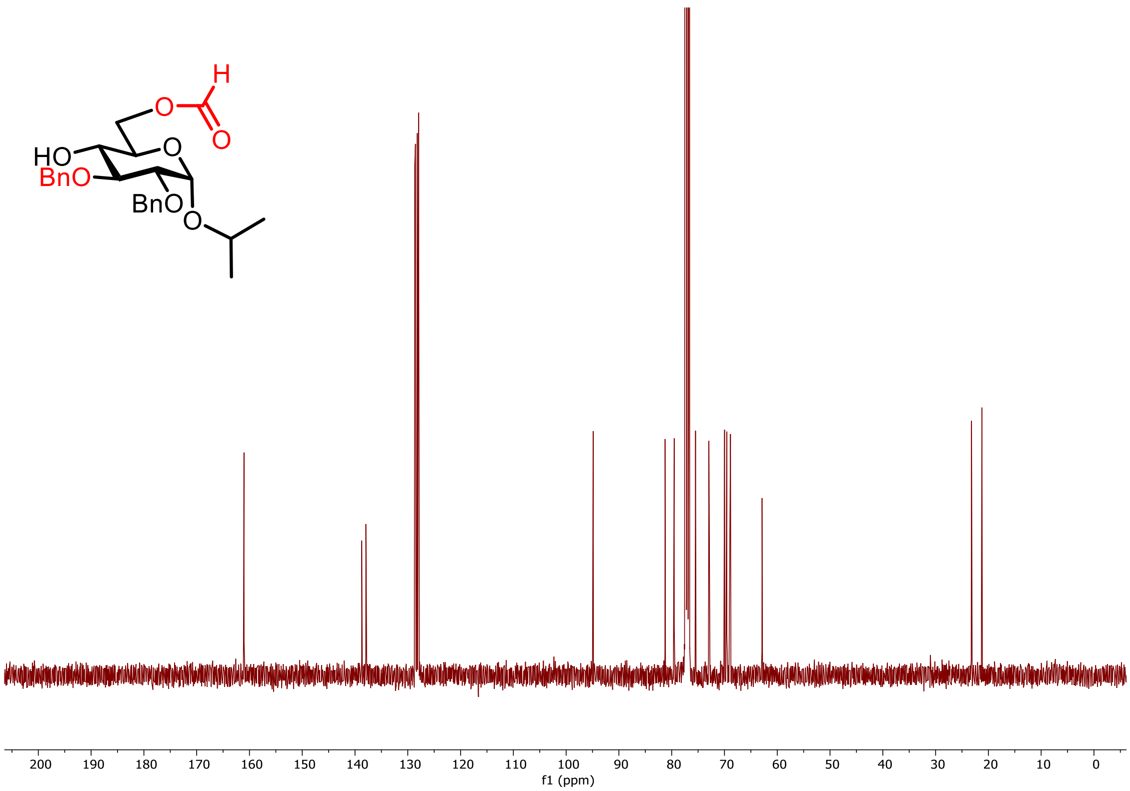

COSY NMR of 76a ($CDCl_3$)

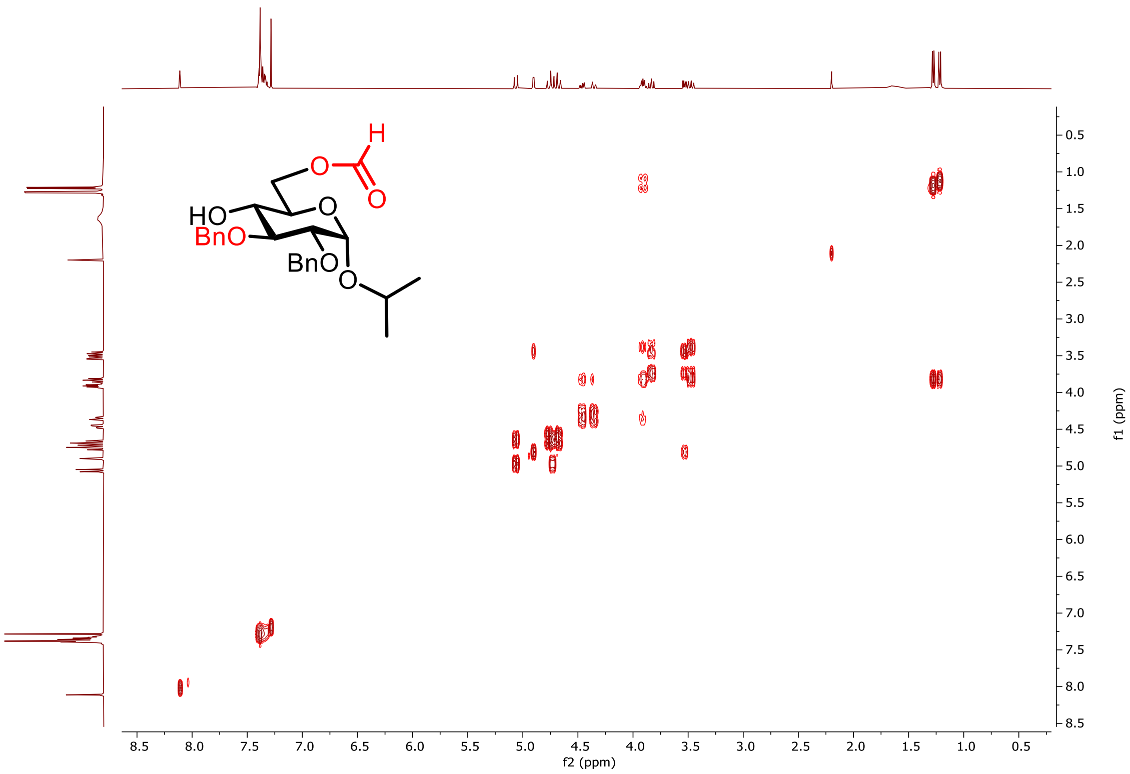

HSQC NMR of 76a ($CDCl_3$)

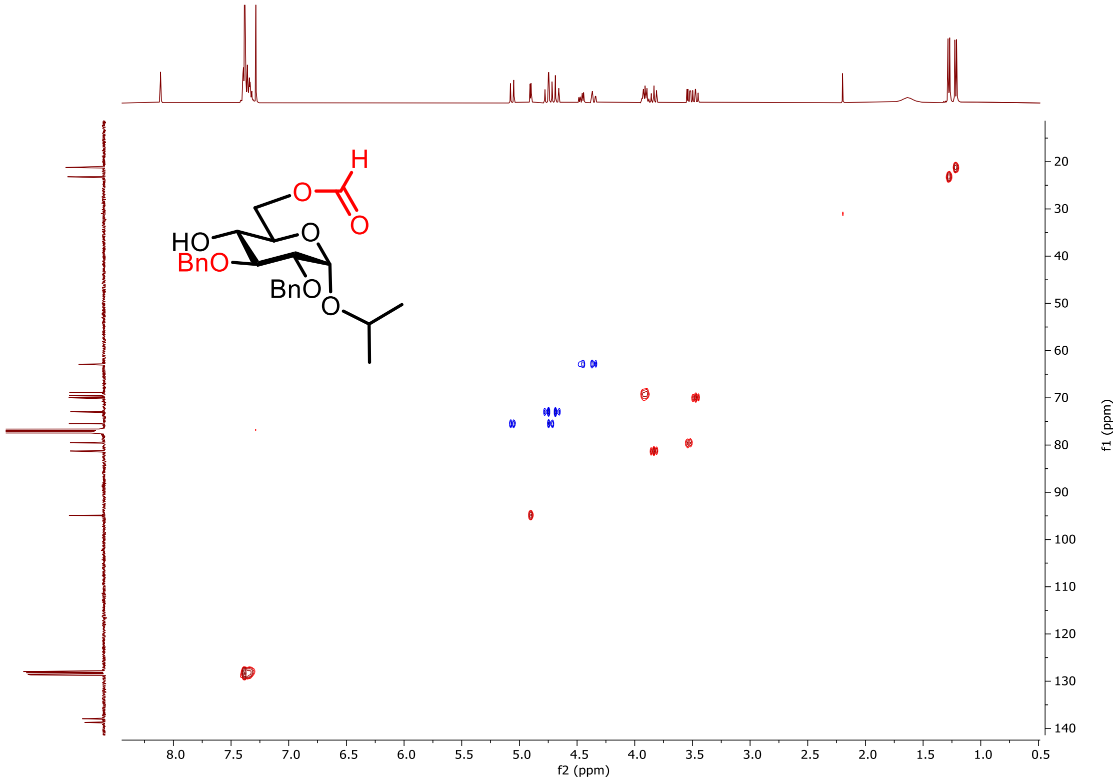

4.4 Isopropyl 2,3-di-*O*-benzyl-6-*O*-propionyl-D-glucopyranoside, 77

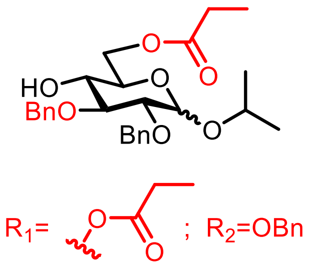

Total yield: 94% (43.1 mg). Ratio of anomer α : β = 2.2 : 1.

Spectrum data for isopropyl 2,3-di-*O*-benzyl-6-*O*-propionyl-α-D-glucopyranoside **77a**: ^{1}H NMR (400 MHz, $CDCl_3$) δ 7.45 – 7.27 (m, 10H), 5.03 (d, *J* = 11.2 Hz, 1H), 4.90 (d, *J* = 3.7 Hz, 1H), 4.81 – 4.72 (m, 2H), 4.67 (d, *J* = 11.9 Hz, 1H), 4.46 (dd, *J* = 12.1, 4.7 Hz, 1H), 4.23 (dd, *J* = 12.1, 2.2 Hz, 1H), 3.97 – 3.79 (m, 3H), 3.52 (dd, *J* = 9.6, 3.7 Hz, 1H), 3.44 (dd, *J* = 10.1, 8.8 Hz, 1H), 2.58 (br. s, 1H), 2.38 (q, *J* = 7.6 Hz, 2H), 1.28 (d, *J* = 6.3 Hz, 3H), 1.22 (d, *J* = 6.1 Hz, 3H), 1.15 (t, *J* = 7.6 Hz, 3H); ^{13}C NMR (101 MHz, $CDCl_3$) δ 174.92, 138.75, 138.04, 128.64, 128.52, 128.19, 128.05, 128.01, 127.92, 94.92, 81.27, 79.48, 75.63, 72.99, 70.12, 69.51, 69.36, 63.20, 27.42, 23.17, 21.26, 9.06; $[\alpha]_D^{25}$ 20.96 (c = 1, $CHCl_3$); IR (neat) ν_{max} = 2924, 1739, 1061, 689 cm^{-1}; m/z ($HRMS^+$) $[M + Na]^+$ 481.2171 ($C_{26}H_{34}O_7Na^+$ requires 481.2197).

^{1}H NMR of crude 77 (600 MHz, $CDCl_3$)

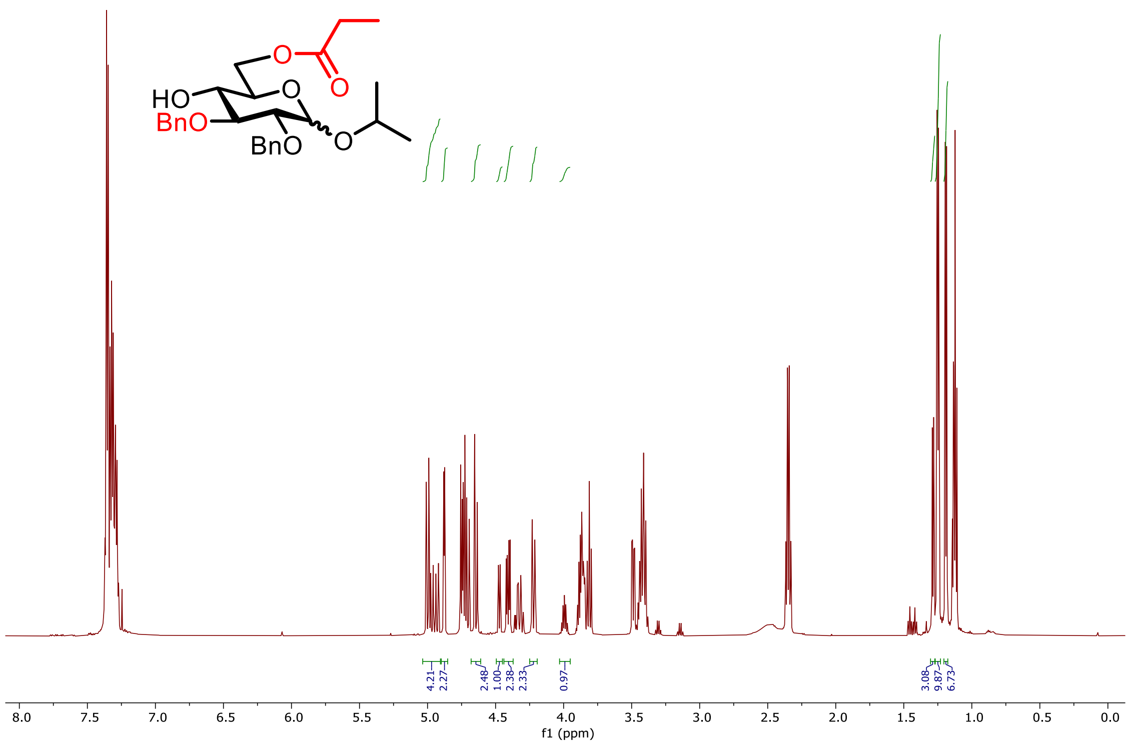

^{13}C NMR of crude 77 (151 MHz, $CDCl_3$)

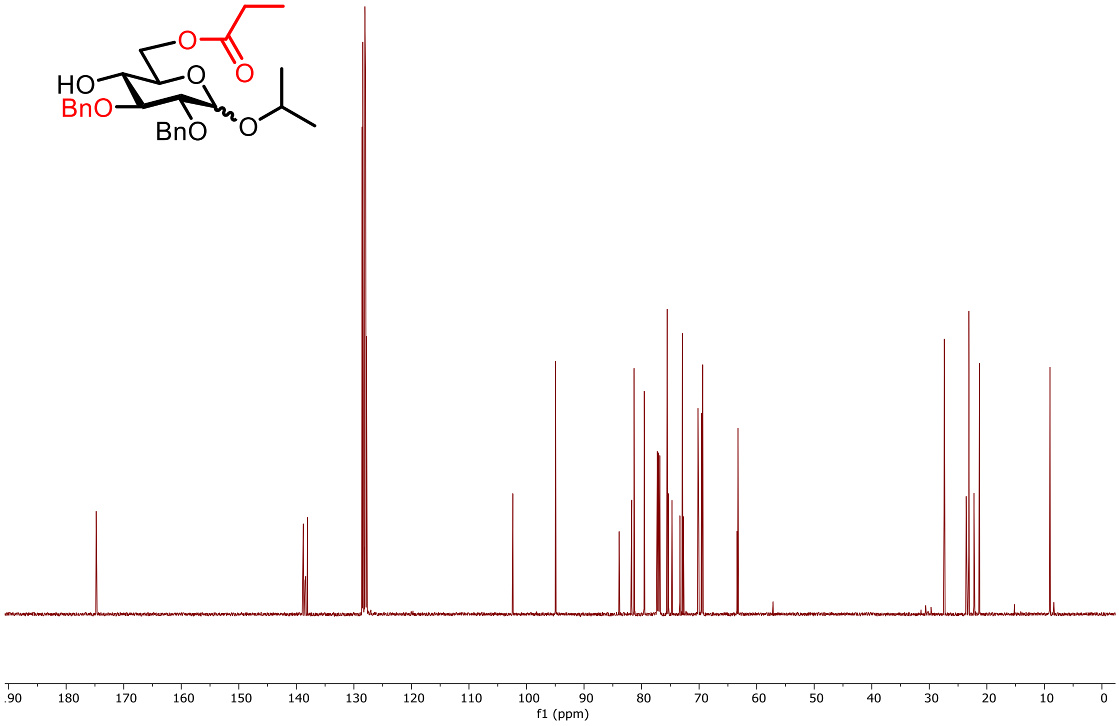

HSQC NMR of crude 77 ($CDCl_3$)

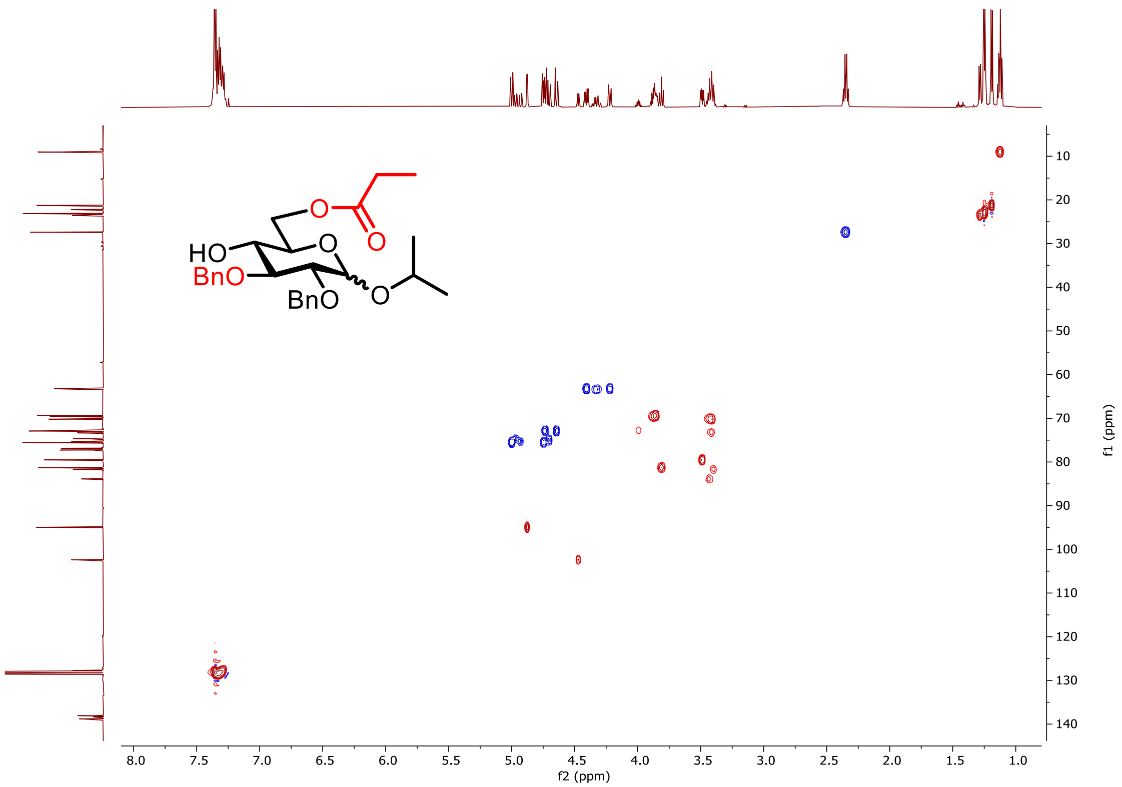

Coupled HSQC NMR of crude 77 ($CDCl_3$)

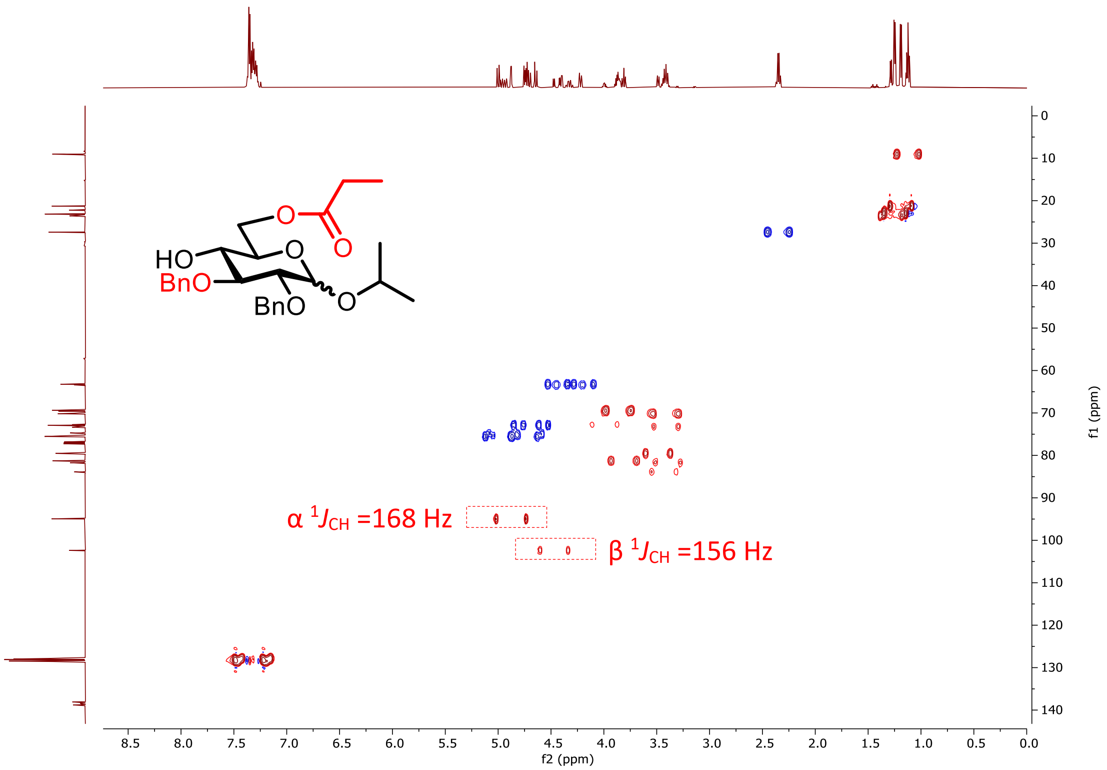

^{1}H NMR of 77a (400 MHz, $CDCl_3$)

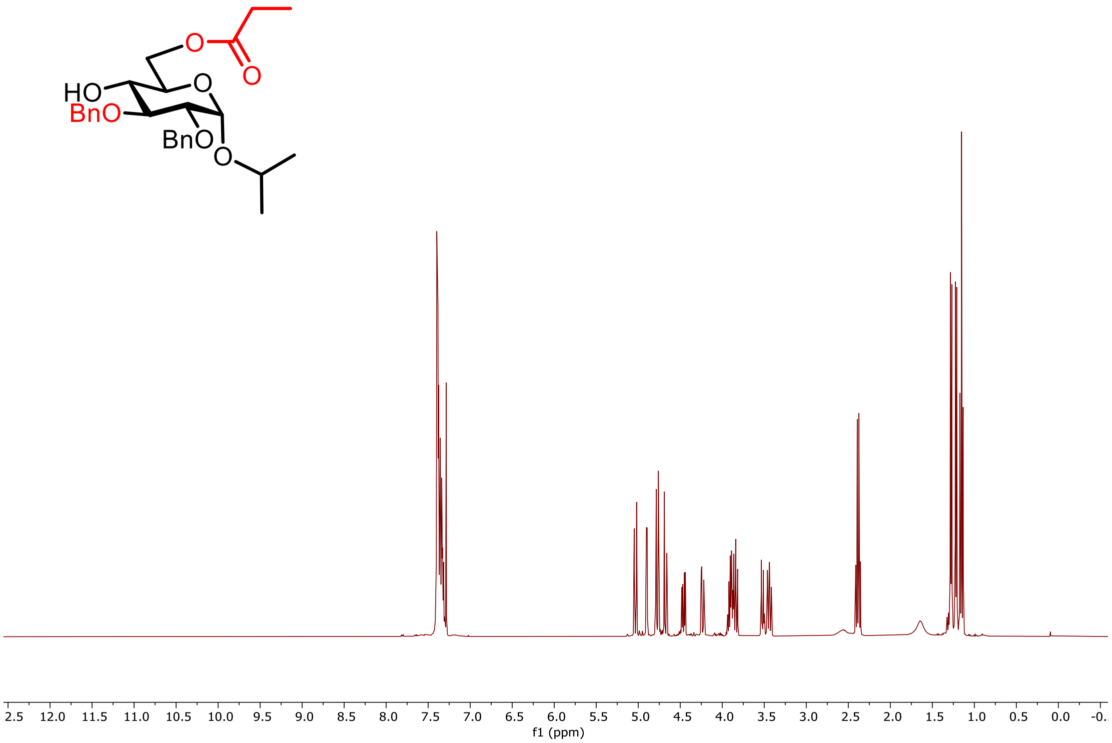

^{13}C NMR of 77a (101 MHz, $CDCl_3$)

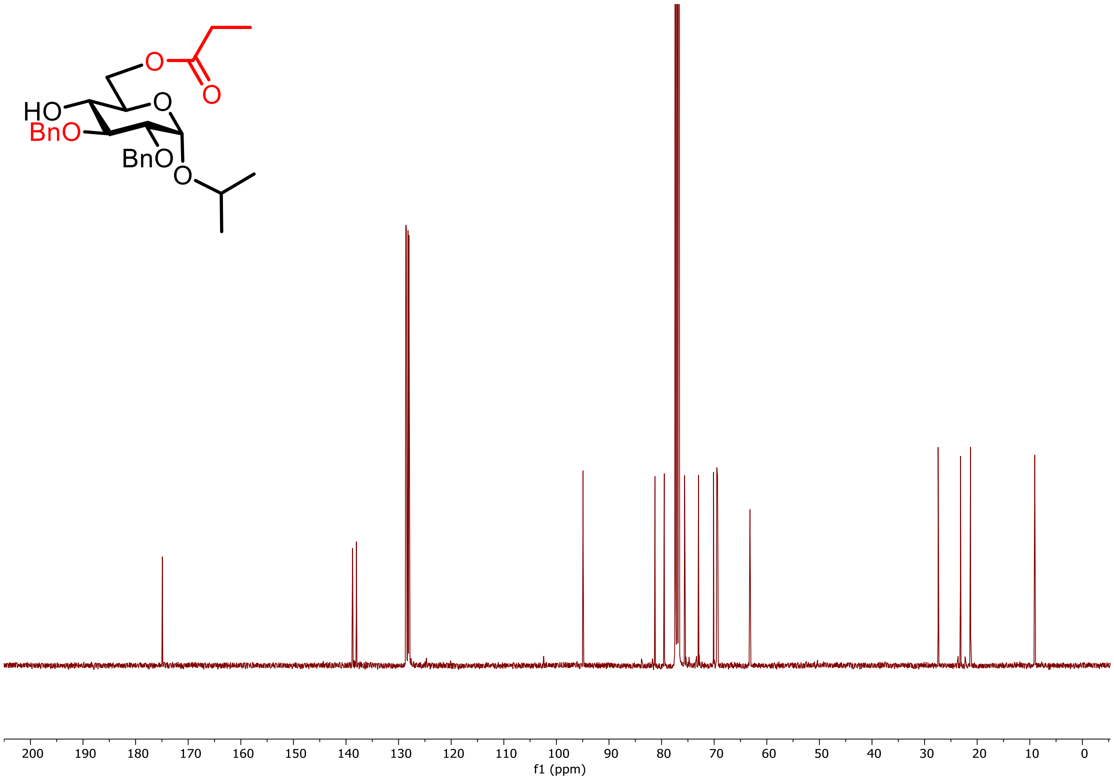

COSY NMR of 77a ($CDCl_3$)

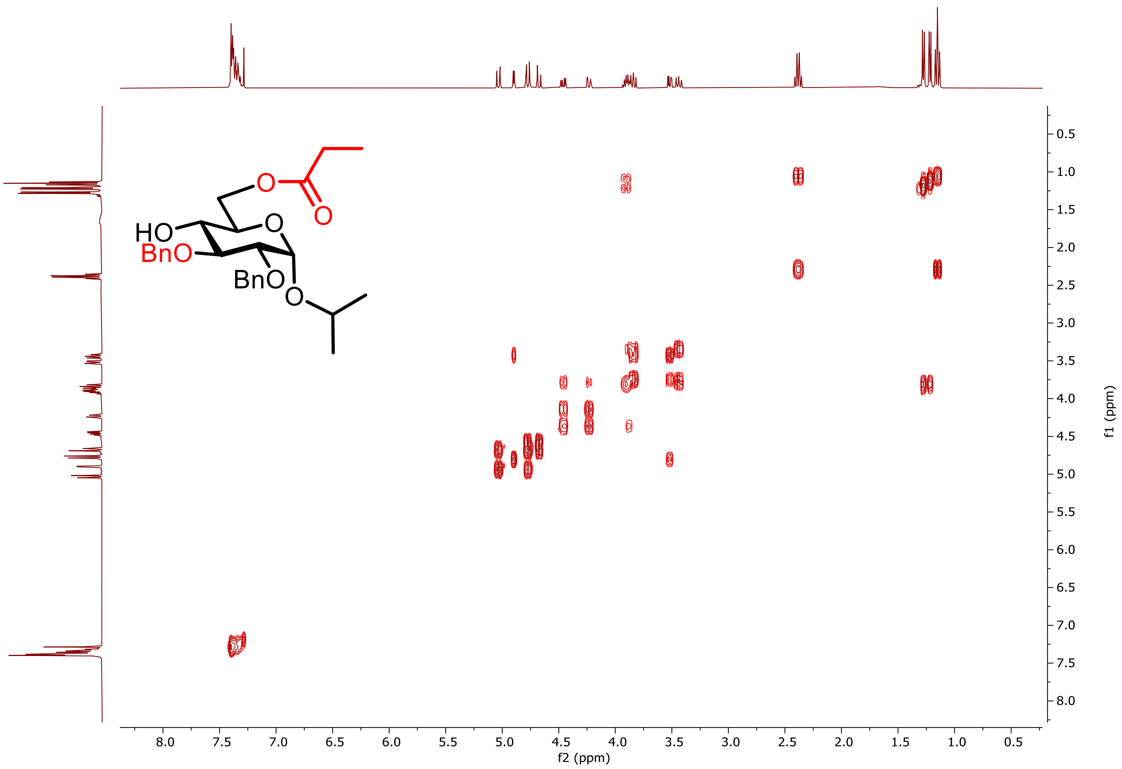

HSQC NMR of 77a ($CDCl_3$)

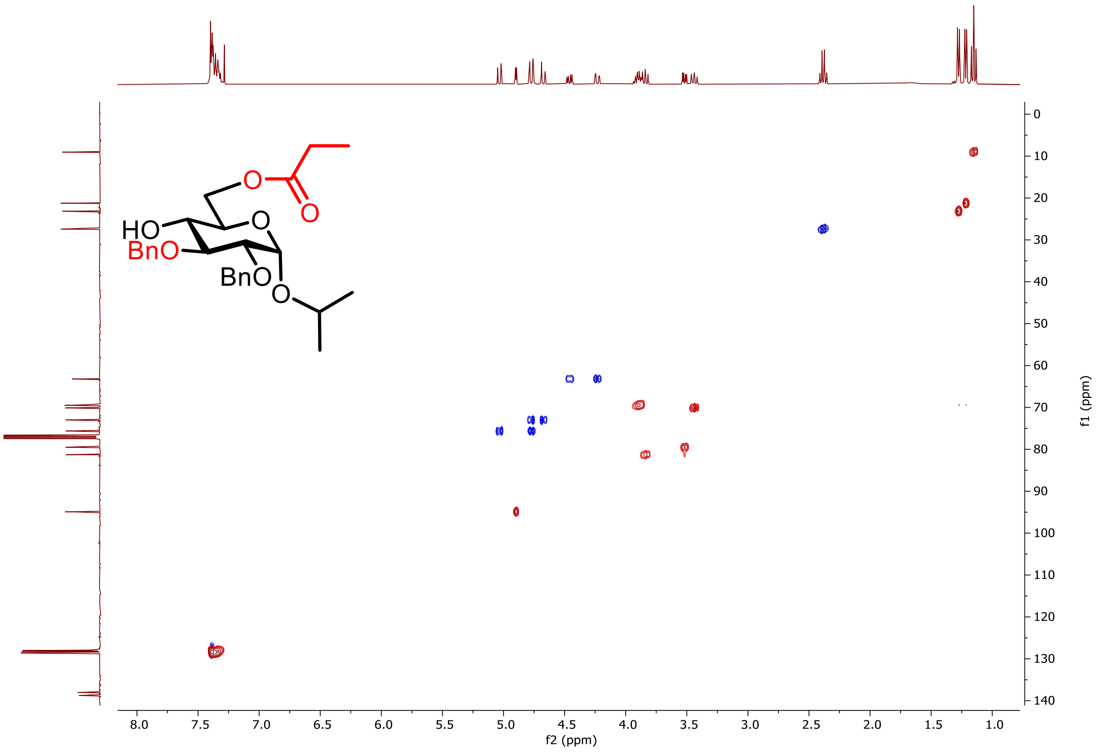

4.5 Isopropyl 2,3-di-*O*-benzyl-6-*O*-levulinoyl-D-glucopyranoside, 78

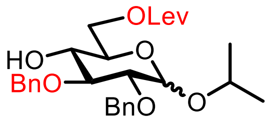

Total yield: 93% (46.5 mg). Ratio of anomer α : β = 1.6 : 1.

Spectrum data for isopropyl 2,3-di-*O*-benzyl-6-*O*-levulinoyl-α-D-glucopyranoside **78a**: ^{1}H NMR (400 MHz, $CDCl_3$) δ 7.41 – 7.29 (m, 10H), 5.03 (d, *J* = 11.2 Hz, 1H), 4.89 (d, *J* = 3.7 Hz, 1H), 4.77 (d, *J* = 11.6 Hz, 2H), 4.67 (d, *J* = 12.0 Hz, 1H), 4.48 (dd, *J* = 12.1, 4.7 Hz, 1H), 4.21 (dd, *J* = 12.1, 2.2 Hz, 1H), 3.97 – 3.79 (m, 3H), 3.52 (dd, *J* = 9.6, 3.7 Hz, 1H), 3.47 (appt, *J* = 9.5 Hz, 1H), 2.77 (dd, *J* = 7.2, 5.9 Hz, 2H), 2.62 (dd, *J* = 7.4, 6.3 Hz, 2H), 2.58 (br. s, 1H), 2.20 (s, 3H), 1.28 (d, *J* = 6.3 Hz, 3H), 1.22 (d, *J* = 6.1 Hz, 3H); ^{13}C NMR (101 MHz, $CDCl_3$) δ 206.64, 173.19, 138.82, 138.07, 128.61, 128.51, 128.19, 128.00, 127.87, 94.93, 81.27, 79.46, 75.57, 73.00, 70.09, 69.49, 69.28, 63.52, 37.92, 29.91, 27.79, 23.18, 21.25; $[\alpha]_D^{25}$ 15.13 (c = 0.25, $CHCl_3$); IR (neat) ν_{max} = 2926, 1719, 1058, 699 cm^{-1}; m/z ($HRMS^+$) $[M + Na]^+$ 523.2302 ($C_{28}H_{36}O_8Na^+$ requires 523.2302).

^{1}H NMR of crude 78 (600 MHz, $CDCl_3$)

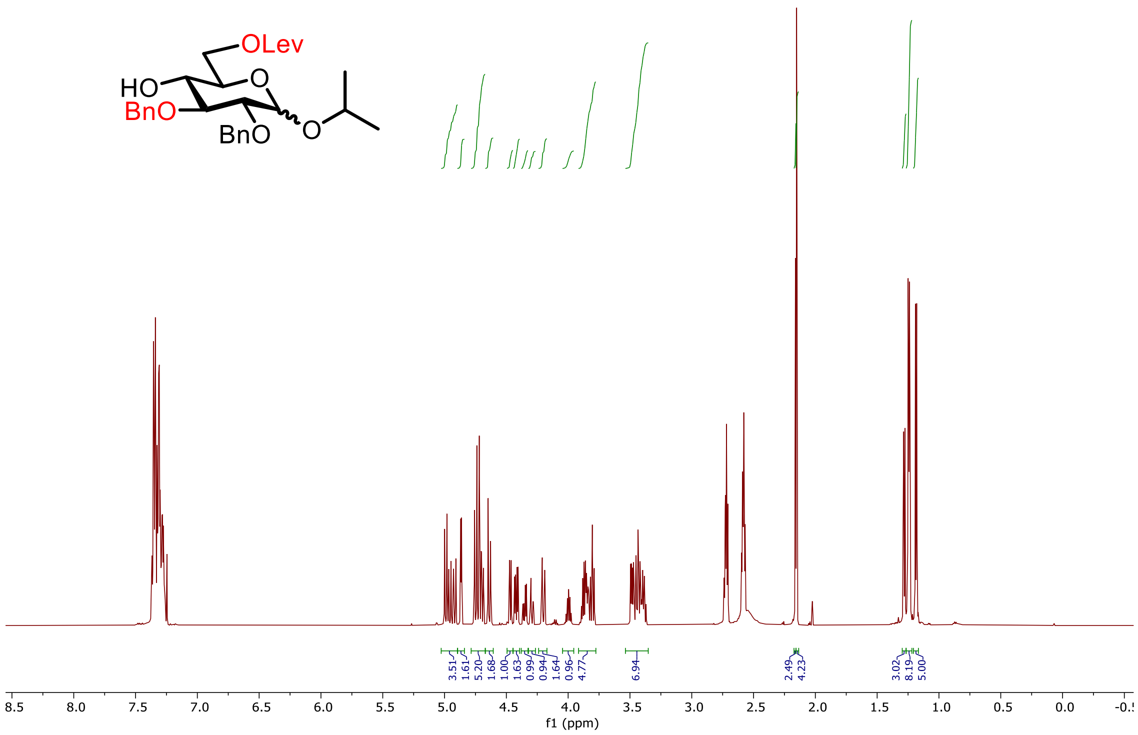

^{13}C NMR of crude 78 (151 MHz, $CDCl_3$)

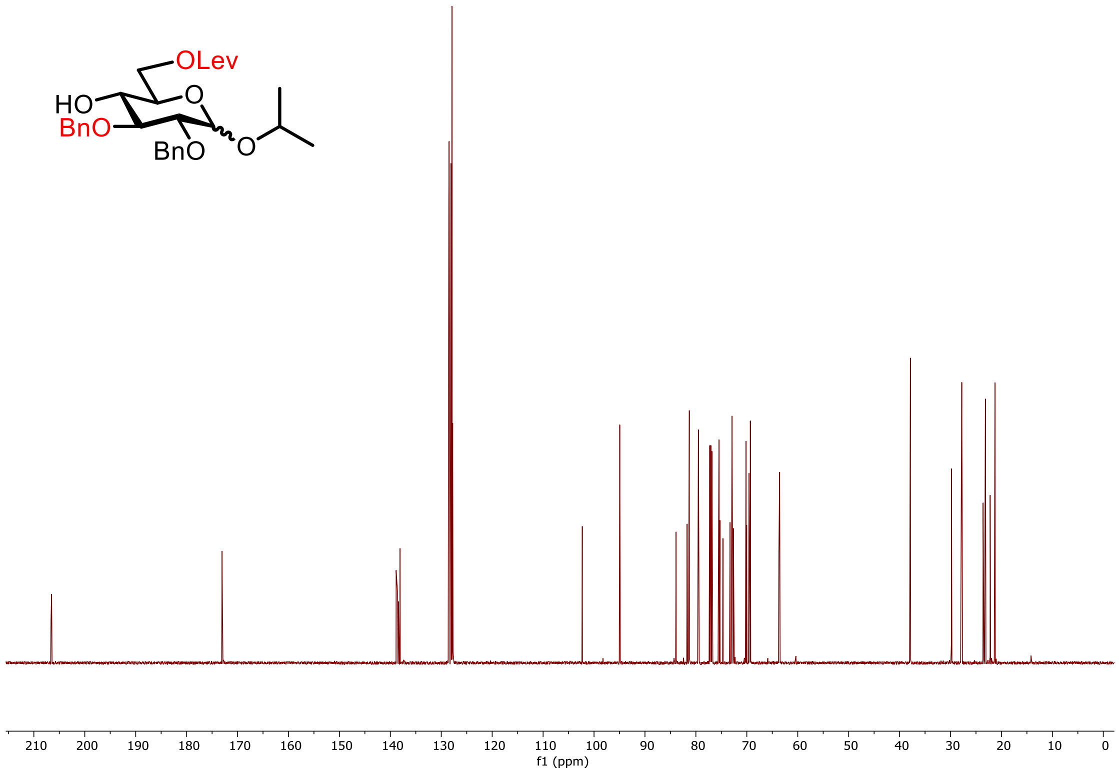

HSQC NMR of crude 78 ($CDCl_3$)

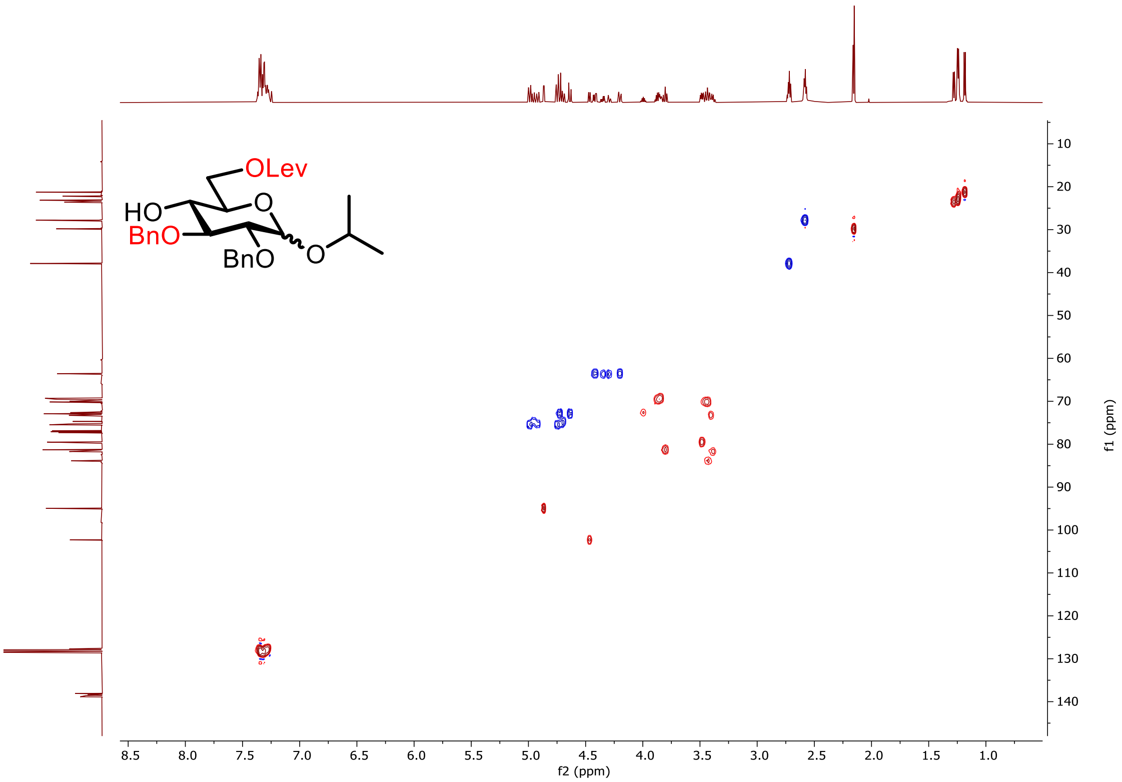

Coupled HSQC NMR of crude 78 ($CDCl_3$)

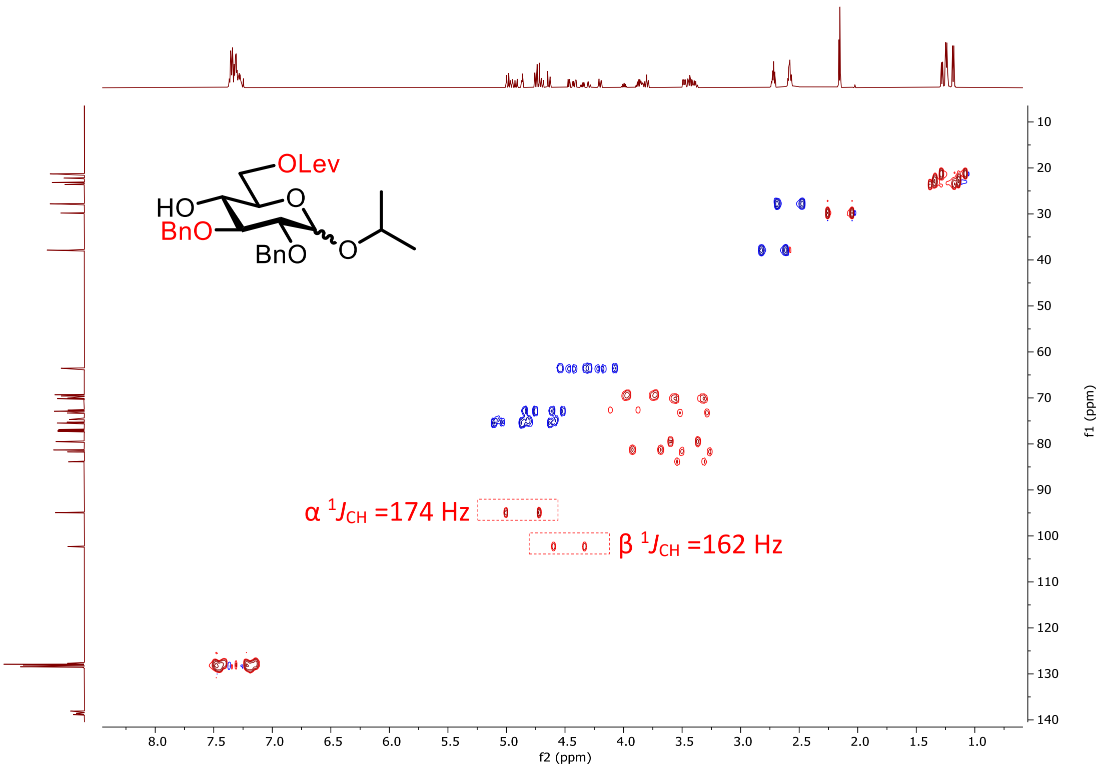

^{1}H NMR of 78a (400 MHz, $CDCl_3$)

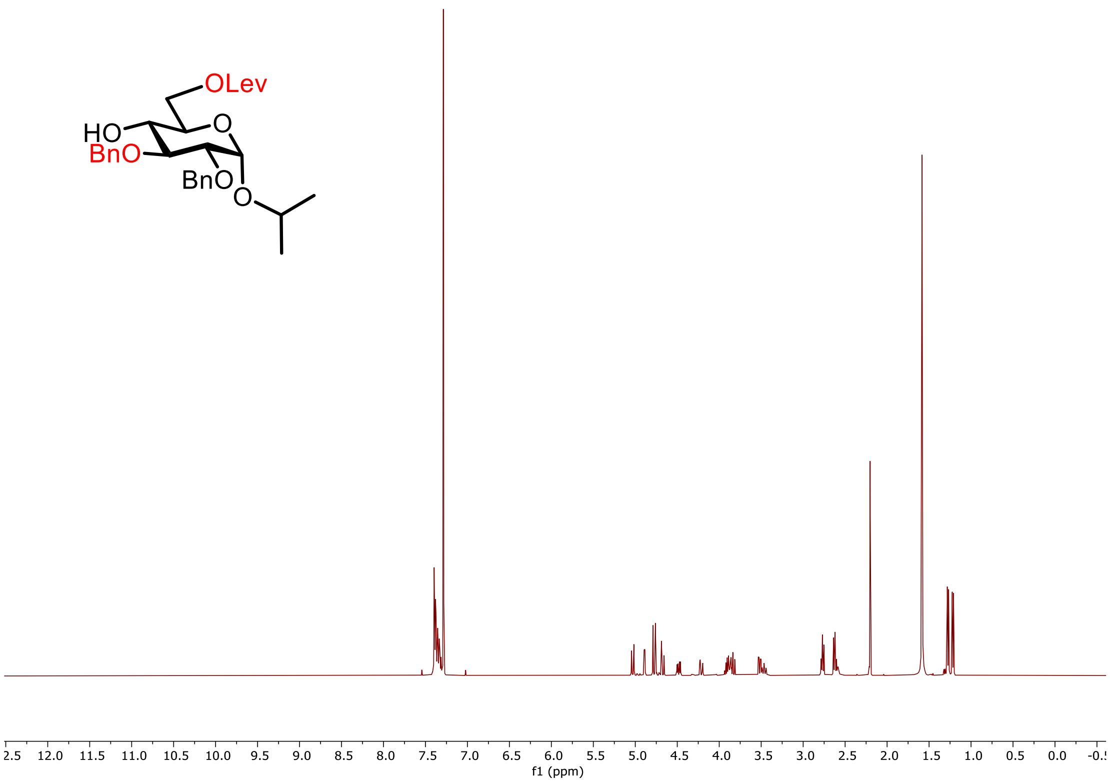

^{13}C NMR of 78a (101 MHz, $CDCl_3$)

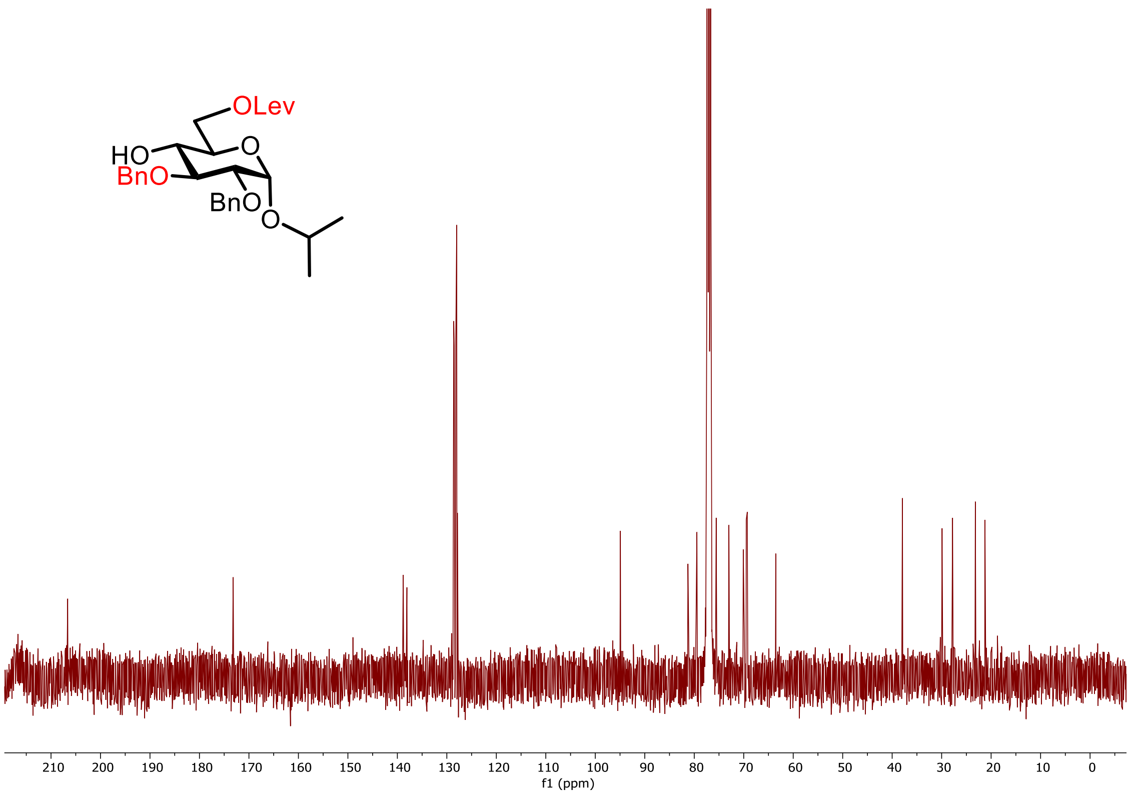

COSY NMR of 78a ($CDCl_3$)

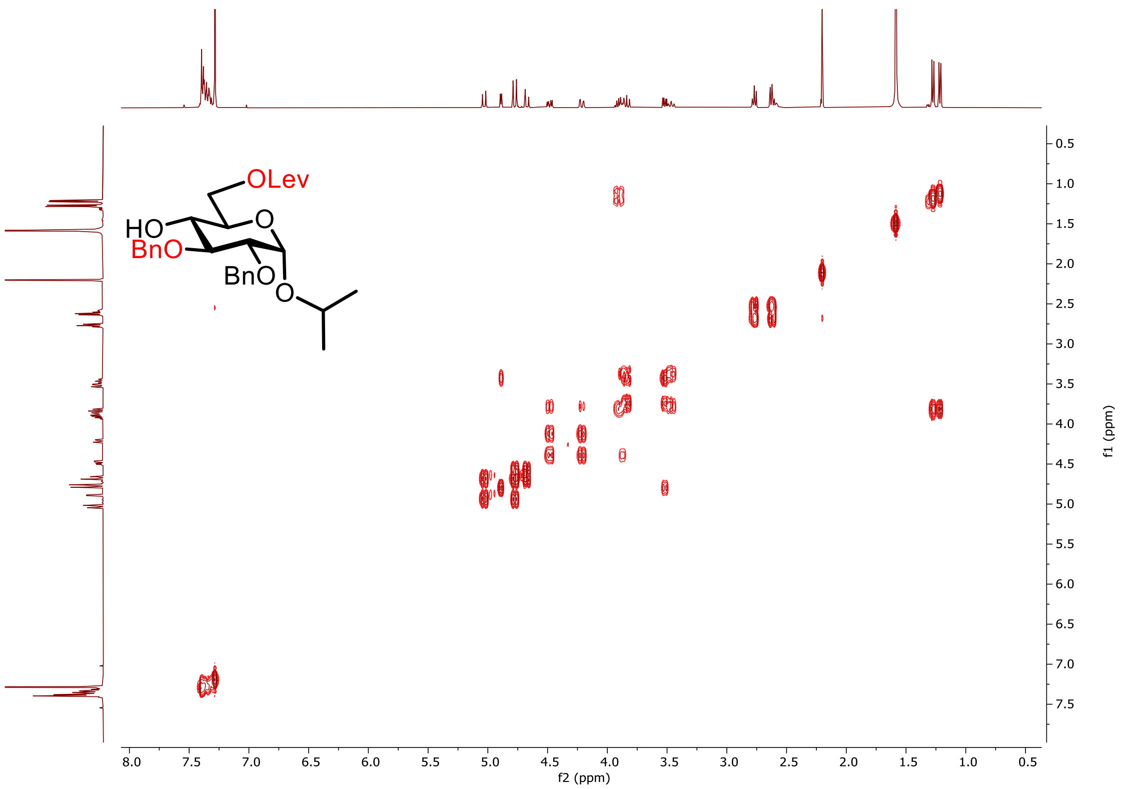

HSQC NMR of 78a ($CDCl_3$)

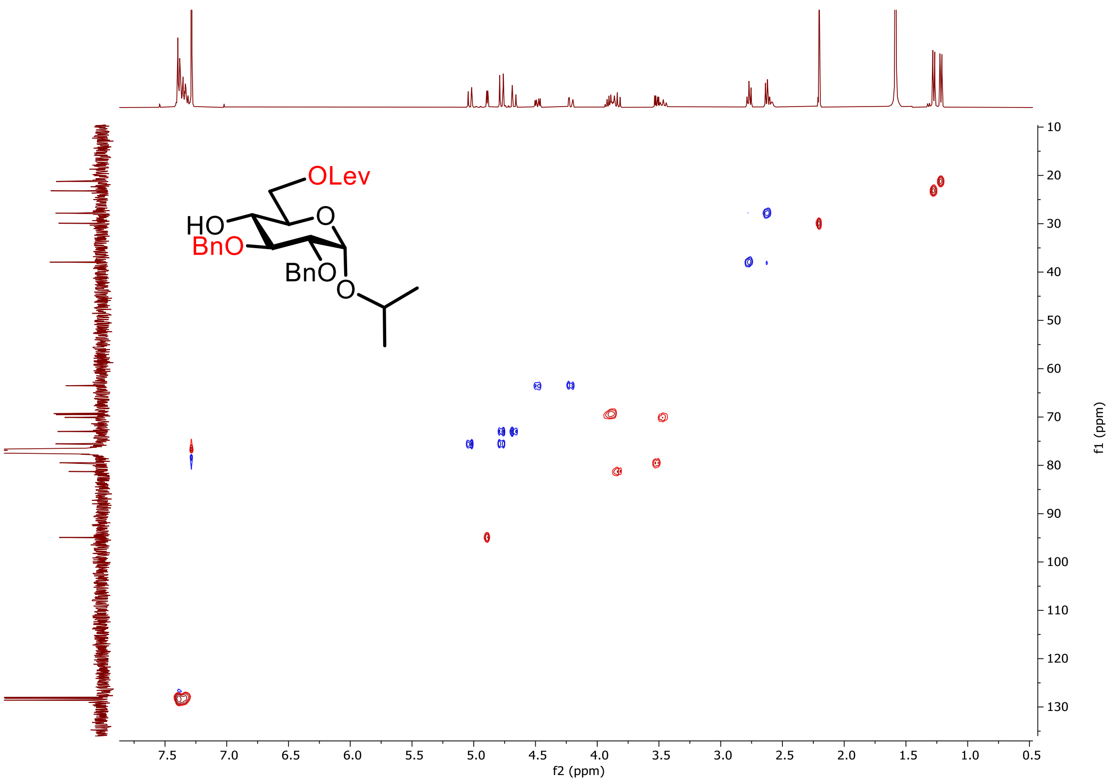

4.6 Isopropyl 2,3-di-*O*-benzyl-6-*O*-pivaloyl-D-glucopyranoside, 79

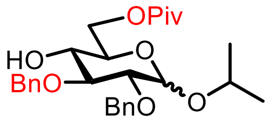

R_1=OPiv; R_2=OBn

Total yield: 92% (44.7 mg). Ratio of anomer α : β = 2.5 : 1.

Spectrum data for isopropyl 2,3-di-*O*-benzyl-6-*O*-pivaloyl-α-D-glucopyranoside **79a**: ^{1}H NMR (400 MHz, $CDCl_3$) δ 7.34 – 7.20 (m, 10H), 4.95 (d, J = 11.3 Hz, 1H), 4.80 (d, J = 3.7 Hz, 1H), 4.69 (d, J = 5.4 Hz, 1H), 4.66 (d, J = 4.8 Hz, 1H), 4.58 (d, J = 11.9 Hz, 1H), 4.26 – 4.18 (m, 2H), 3.88 – 3.79 (m, 2H), 3.74 (dd, J = 9.5, 8.8 Hz, 1H), 3.41 (dd, J = 9.6, 3.7 Hz, 1H), 3.31 (dd, J = 10.1, 8.8 Hz, 1H), 2.34 (br. s, 1H), 1.19 (d, J = 6.3 Hz, 3H), 1.14 – 1.10 (m, 12H); ^{13}C NMR (101 MHz, $CDCl_3$) δ 178.84, 138.74, 138.04, 128.65, 128.52, 128.16, 128.05, 128.01, 127.95, 94.62, 81.34, 79.65, 75.61, 72.97, 70.44, 69.48, 69.13, 63.60, 38.89, 27.21, 23.21, 21.16; $[\alpha]_D^{25}$ 26.62 (c = 0.5, $CHCl_3$); IR (neat) ν_{max} = 2974, 1731, 1061, 698 cm^{-1}; m/z (HRMS$^+$) $[M + Na]^+$ 509.2505 ($C_{28}H_{38}O_7Na^+$ requires 509.2510).

^{1}H NMR of crude 79 (600 MHz, $CDCl_3$)

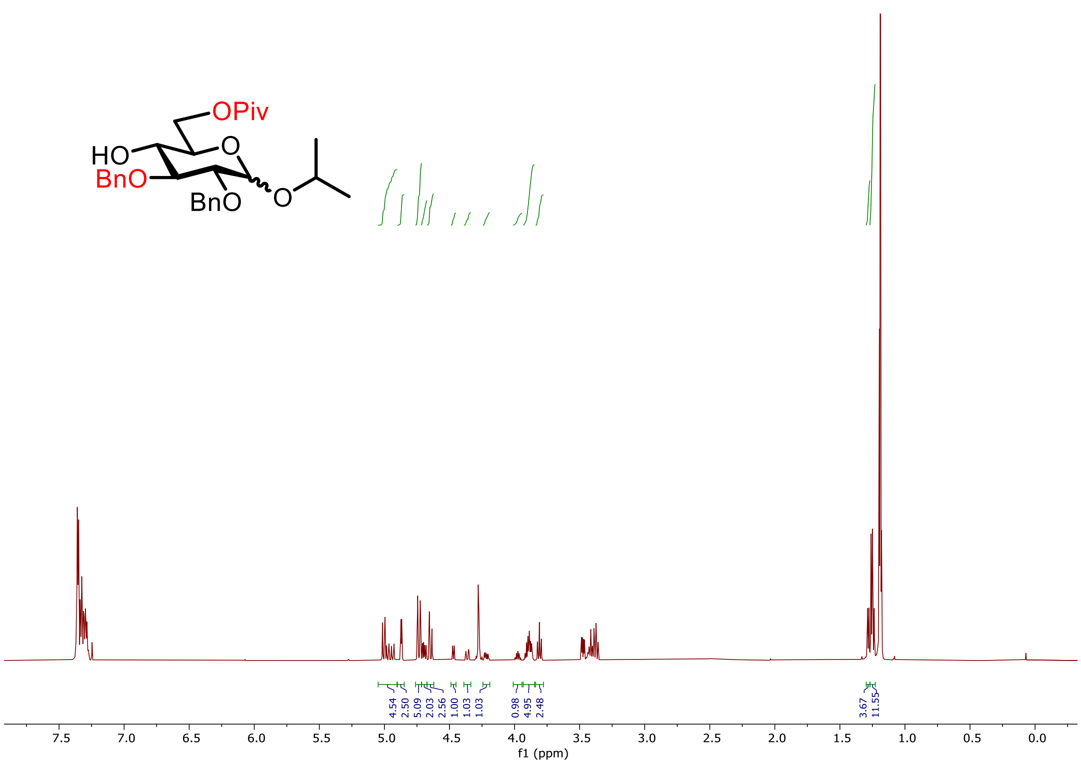

^{13}C NMR of crude 79 (151 MHz, $CDCl_3$)

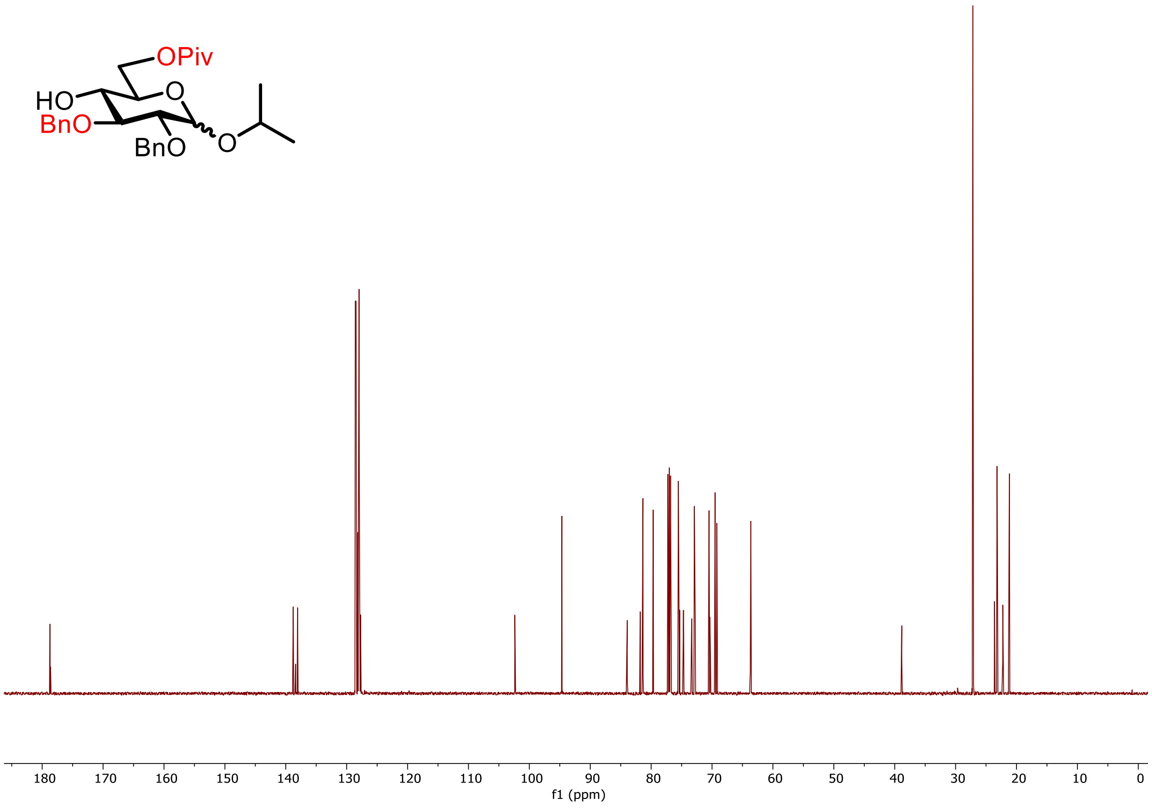

HSQC NMR of crude 79 ($CDCl_3$)

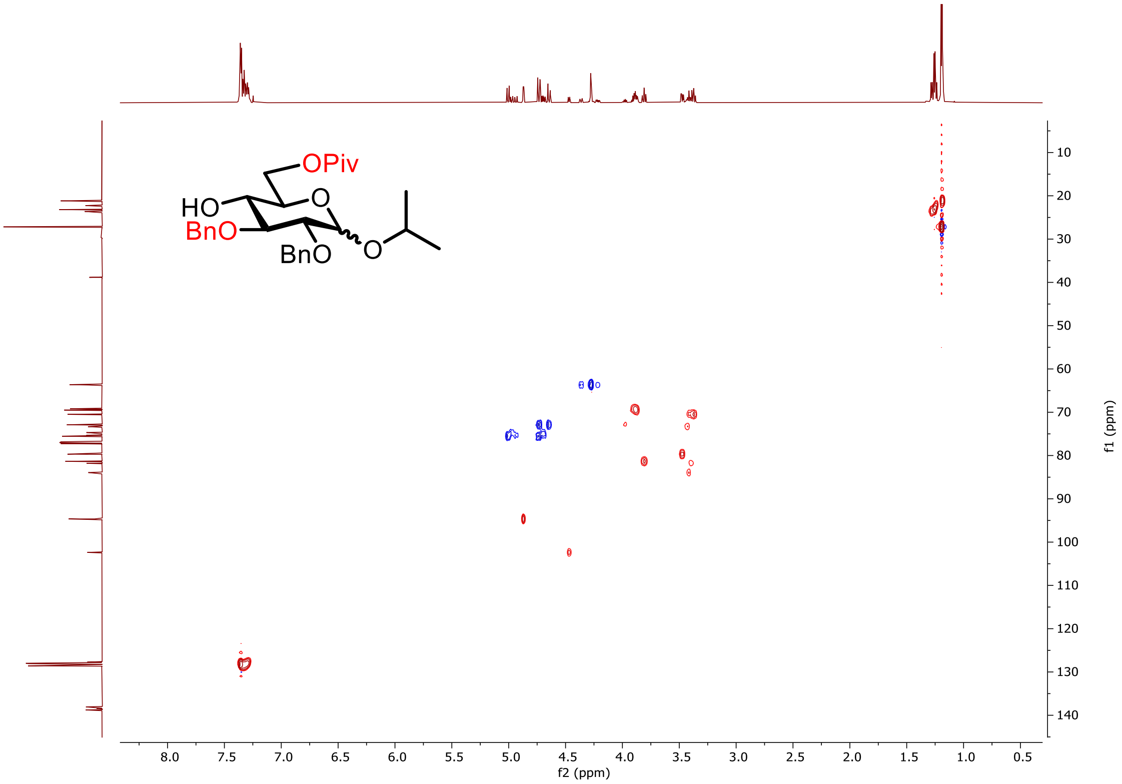

Coupled HSQC NMR of crude 79 ($CDCl_3$)

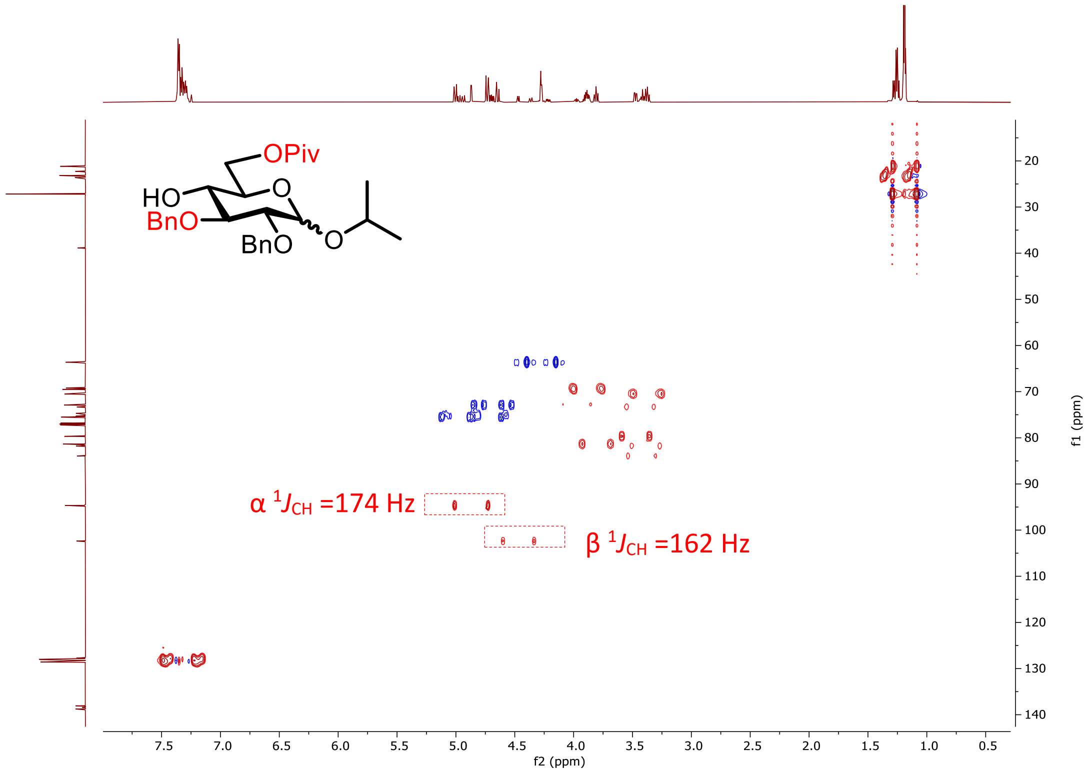

^{1}H NMR of 79a (400 MHz, $CDCl_3$)

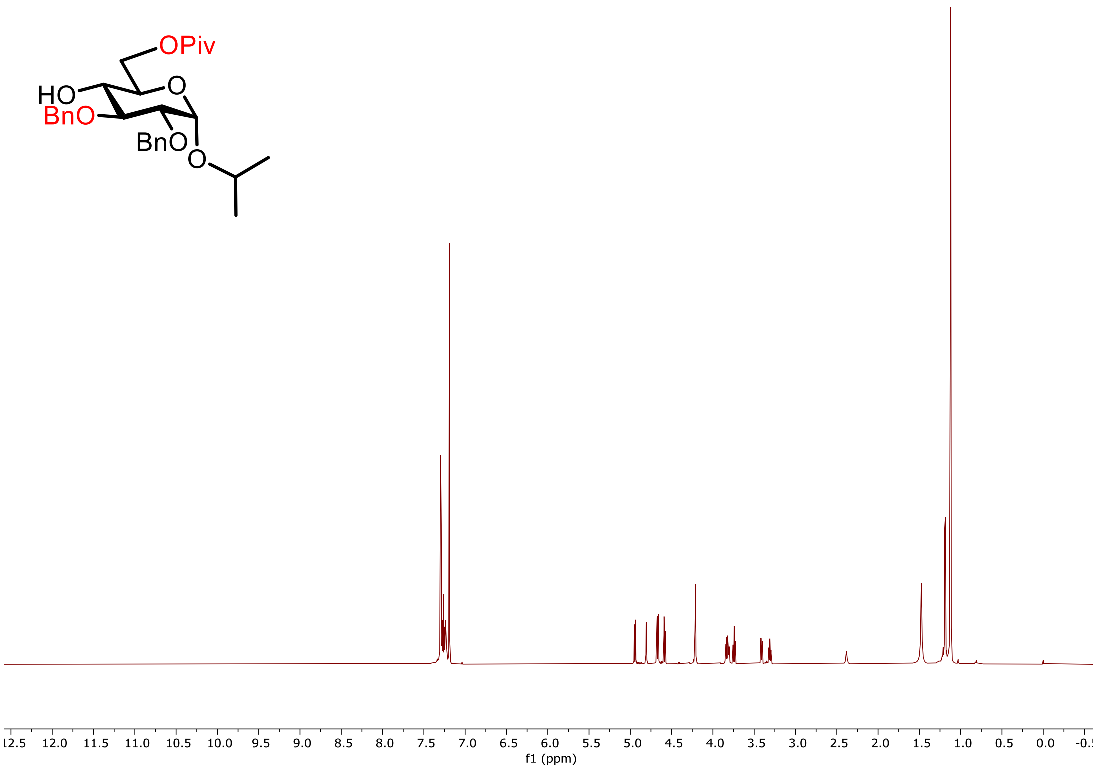

^{13}C NMR of 79a (101 MHz, $CDCl_3$)

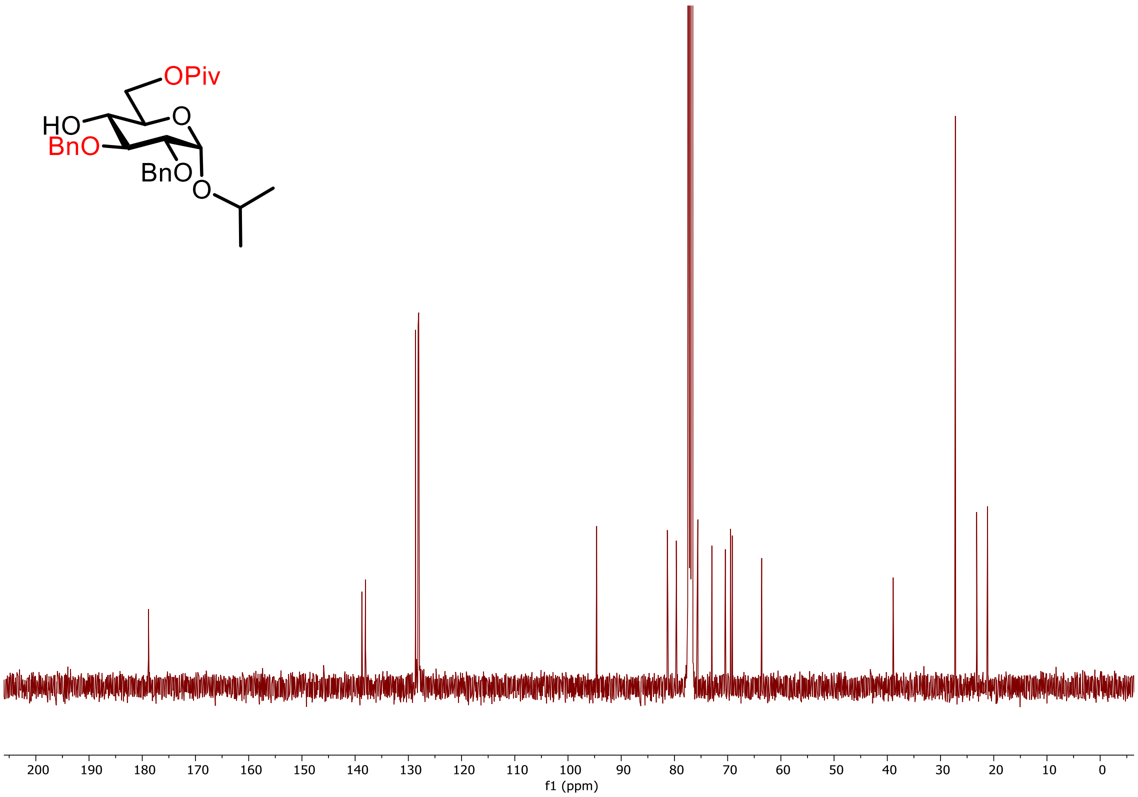

COSY NMR of 79a ($CDCl_3$)

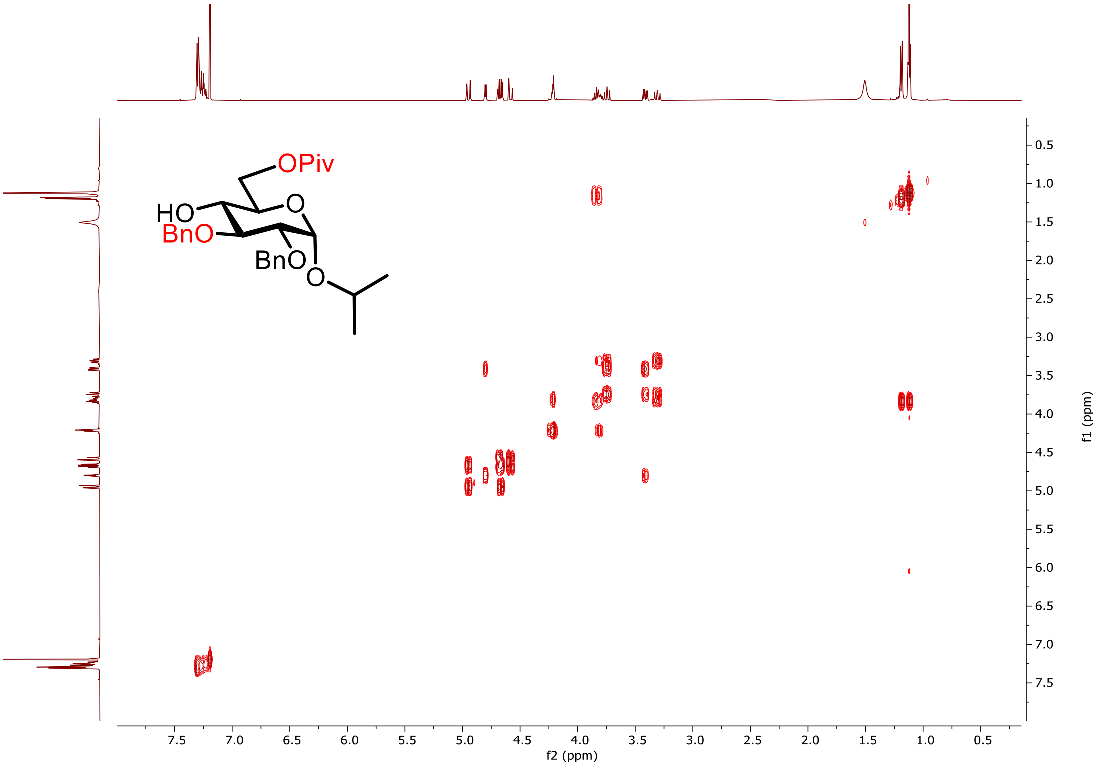

HSQC NMR of 79a ($CDCl_3$)

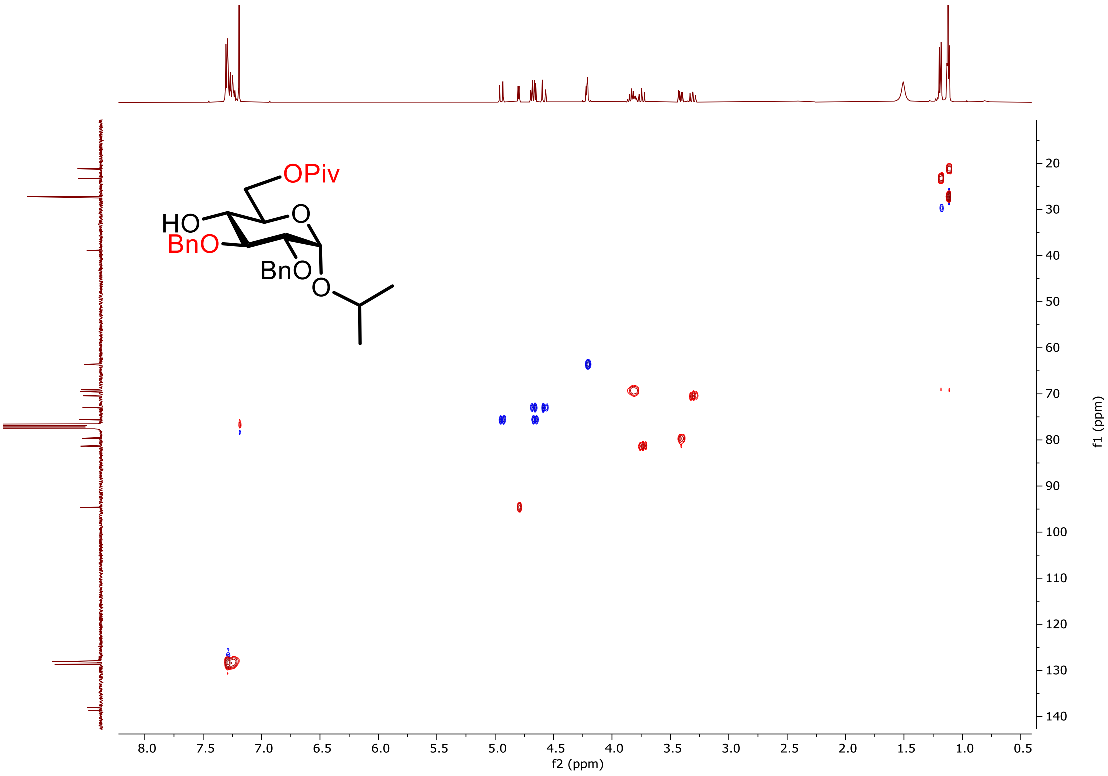

4.7 Isopropyl 2,3-di-*O*-benzyl-D-glucopyranoside, 80

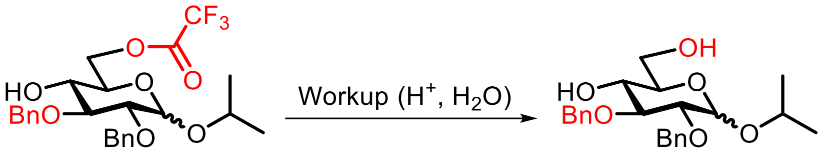

R$_1$=OCOCF$_3$; R$_2$=OBn R$_1$=OH; R$_2$=OBn

Isopropyl 2,3-di-*O*-benzyl-6-*O*-trifluoroacetyl-D-glucopyranoside is converted to isopropyl 2,3-di-*O*-benzyl-D-glucopyranoside during workup at acidic aqueous condition, which was used for further analysis.

Total yield: 84% (33.8 mg). Ratio of anomer α : β = 2.4 : 1.

Spectrum data for isopropyl 2,3-di-*O*-benzyl-α-D-glucopyranoside **80a**: ^{1}H NMR (400 MHz, $CDCl_3$) δ 7.43 – 7.28 (m, 10H), 5.07 (d, *J* = 11.4 Hz, 1H), 4.89 (d, *J* = 3.6 Hz, 1H), 4.76 (d, *J* = 11.9 Hz, 1H), 4.73 (d, *J* = 11.5 Hz, 1H), 4.68 (d, *J* = 11.9 Hz, 1H), 3.96 – 3.71 (m, 5H), 3.59 – 3.48 (m, 2H), 2.09 (br. s, 2H), 1.28 (d, *J* = 6.3 Hz, 3H), 1.22 (d, *J* = 6.1 Hz, 3H); ^{13}C NMR (101 MHz, $CDCl_3$) δ 138.85, 138.03, 128.65, 128.53, 128.19, 128.01, 127.89, 94.71, 81.45, 79.79, 75.38, 72.89, 70.64, 70.61, 69.21, 62.57, 23.25, 21.16; $[\alpha]_D^{25}$ 42.17 (c = 1, $CHCl_3$); IR (neat) ν_{max} = 3415, 2974, 1058, 1028, 698 cm^{-1}; m/z (HRMS$^+$) [M + Na]$^+$ 425.1909 ($C_{23}H_{30}O_6Na^+$ requires 425.1935).

^{1}H NMR of crude 80 (600 MHz, $CDCl_3$)

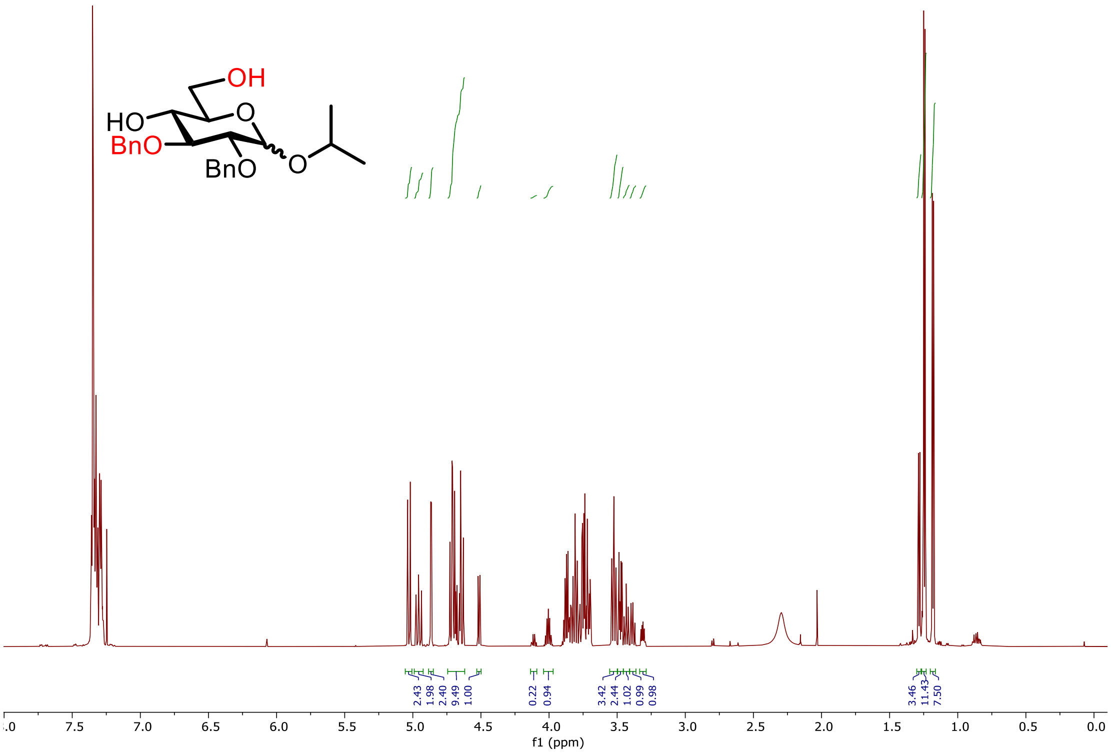

^{13}C NMR of crude 80 (151 MHz, $CDCl_3$)

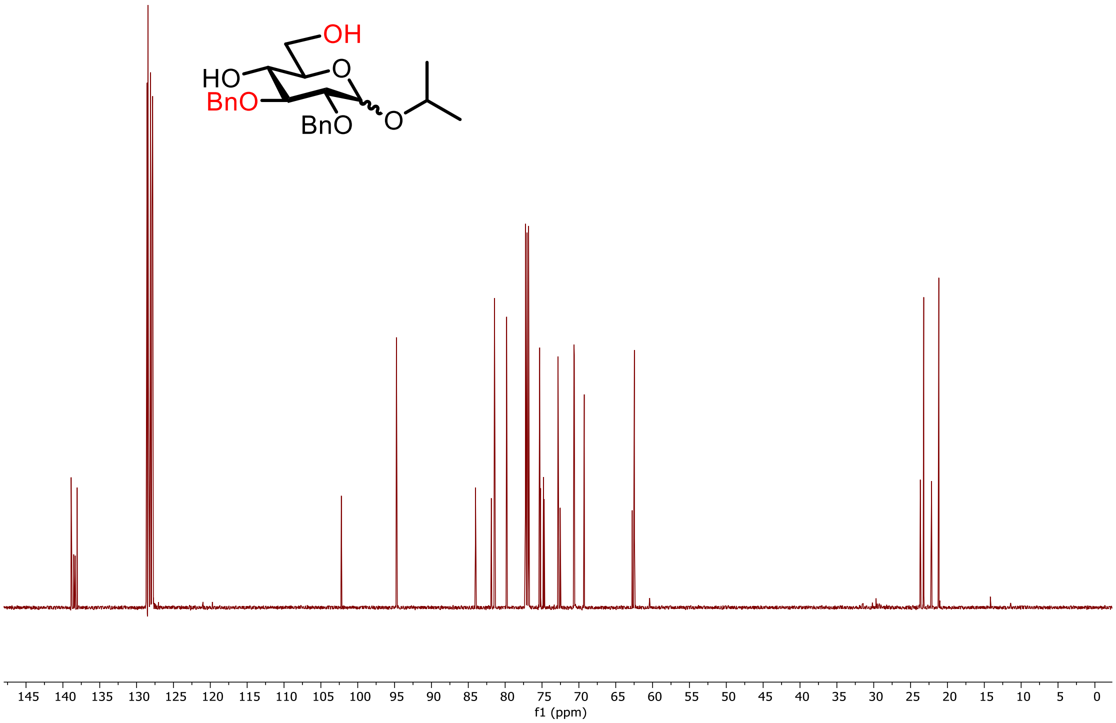

HSQC NMR of crude 80 ($CDCl_3$)

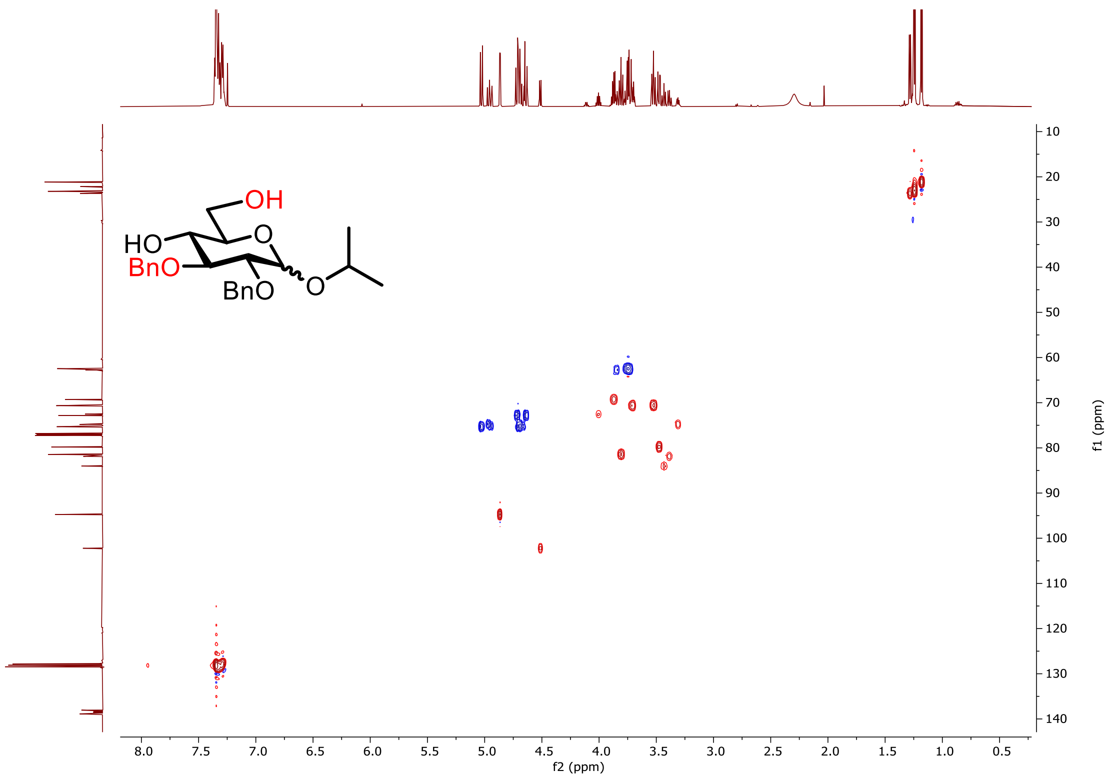

Coupled HSQC NMR of crude 80 ($CDCl_3$)

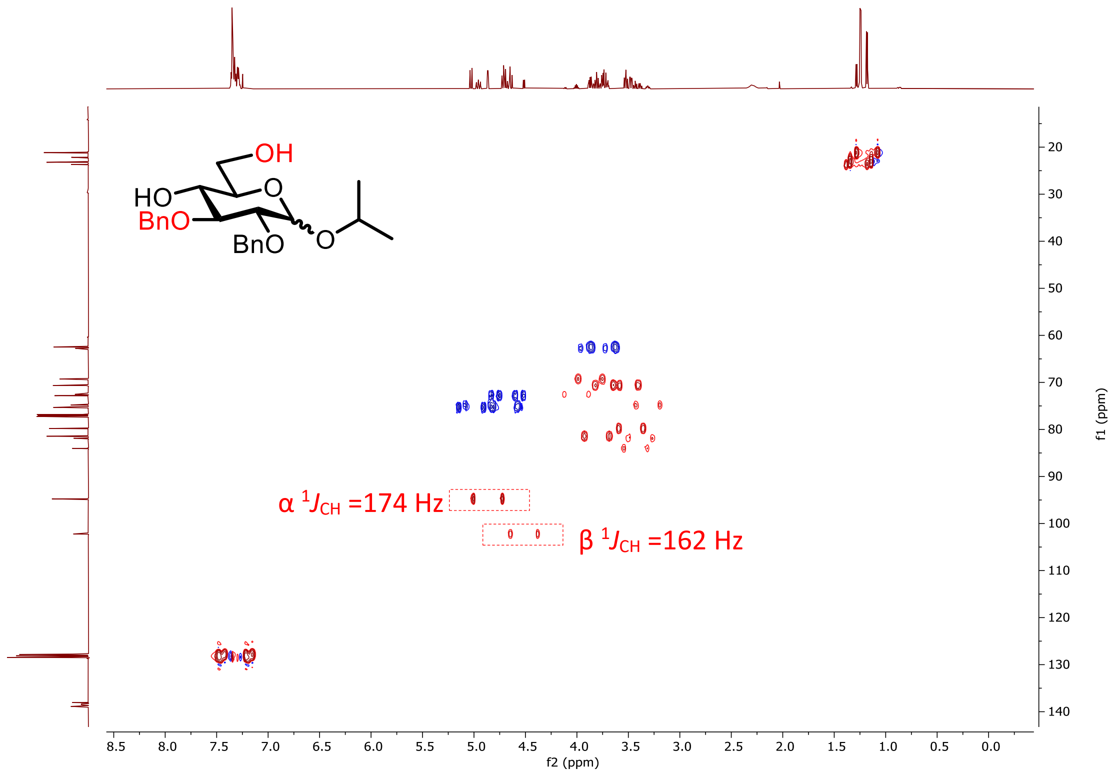

^{1}H NMR of 80a (400 MHz, $CDCl_3$)

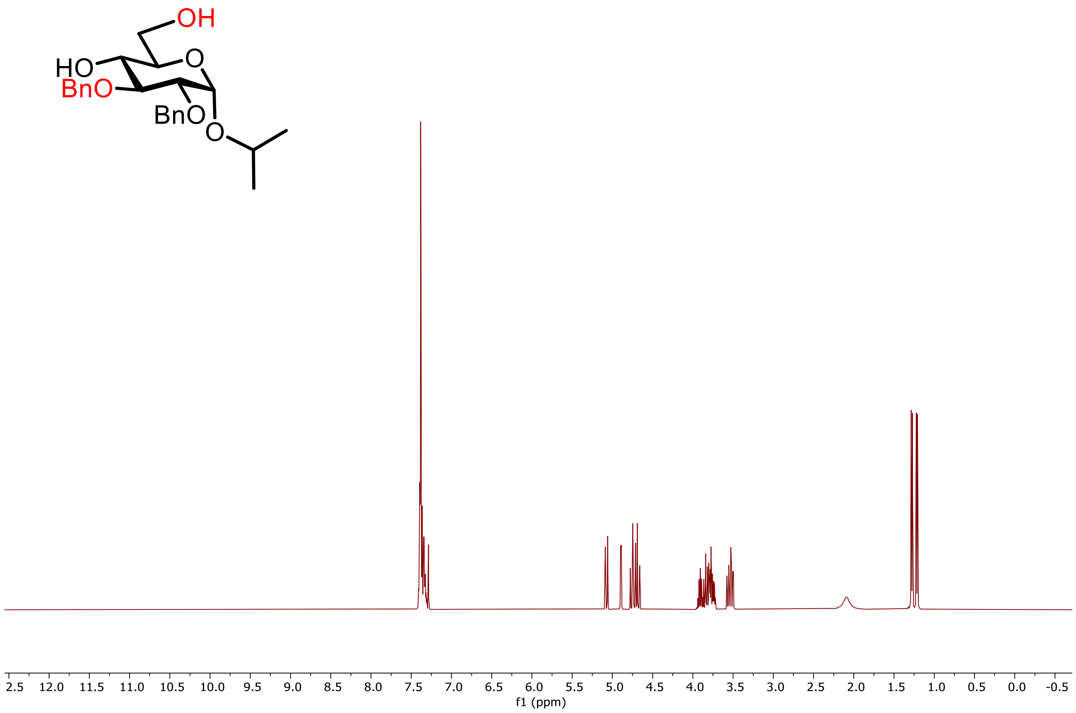

^{13}C NMR of 80a (101 MHz, $CDCl_3$)

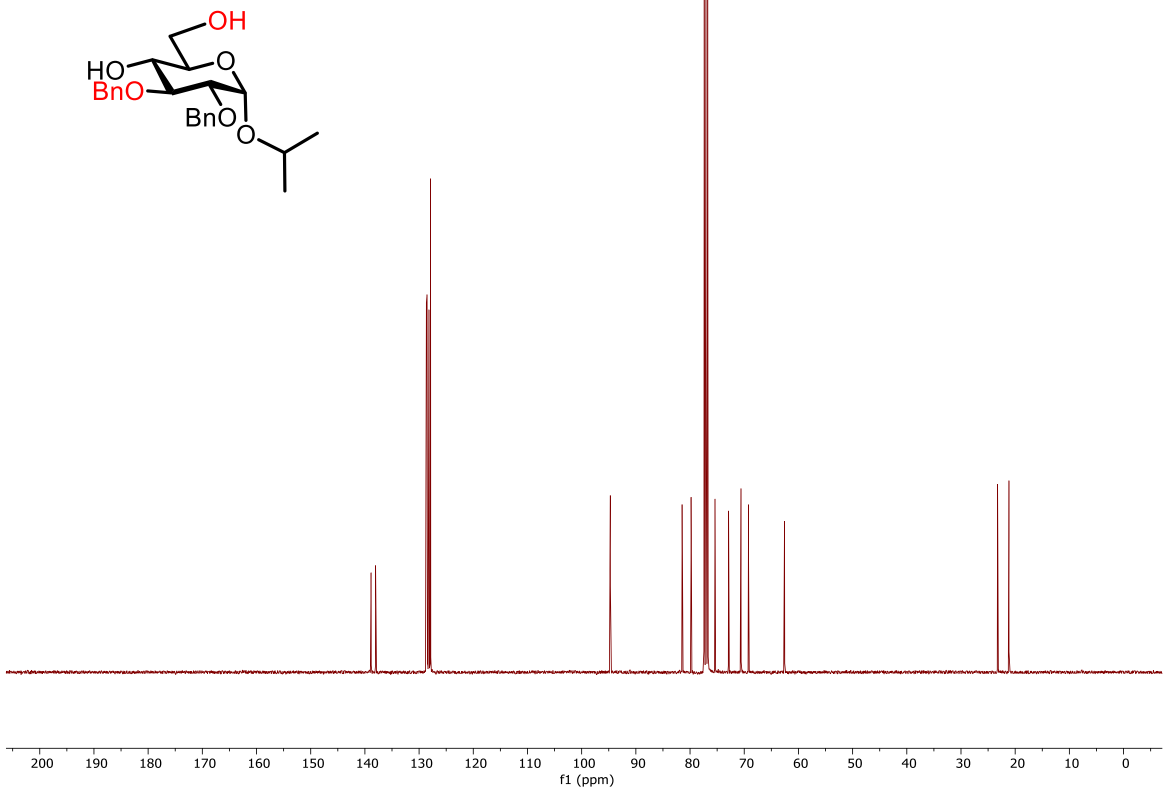

COSY NMR of 80a ($CDCl_3$)

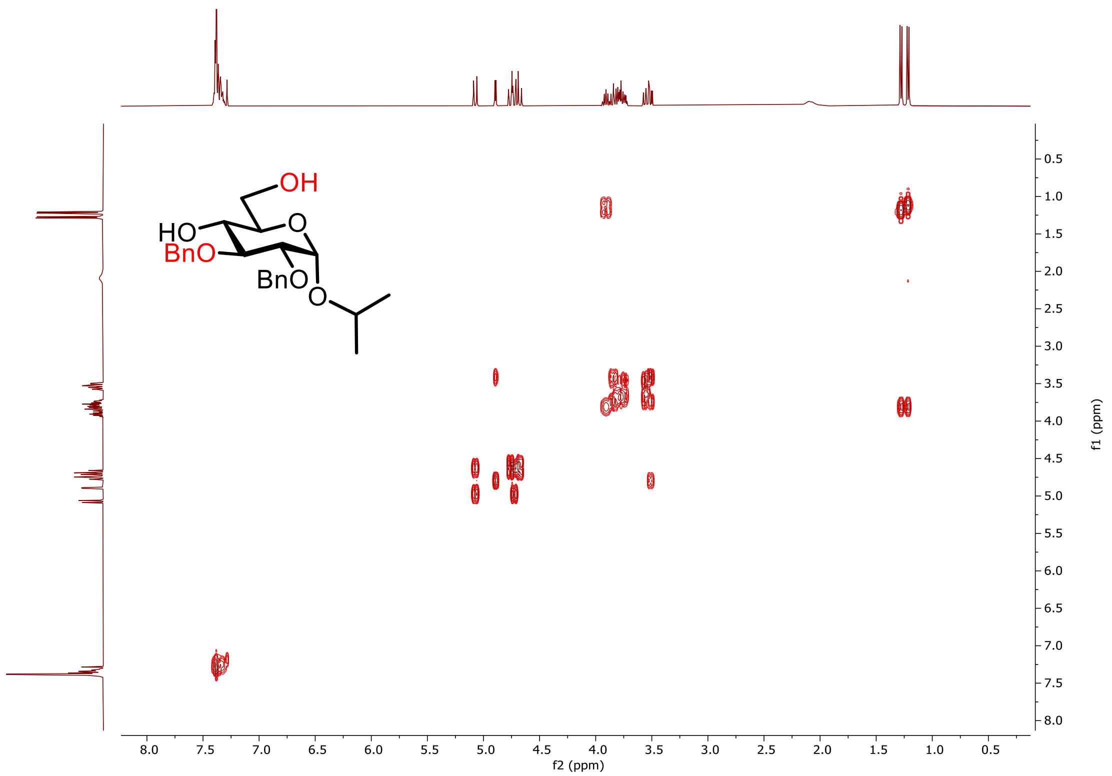

HSQC NMR of 80a ($CDCl_3$)

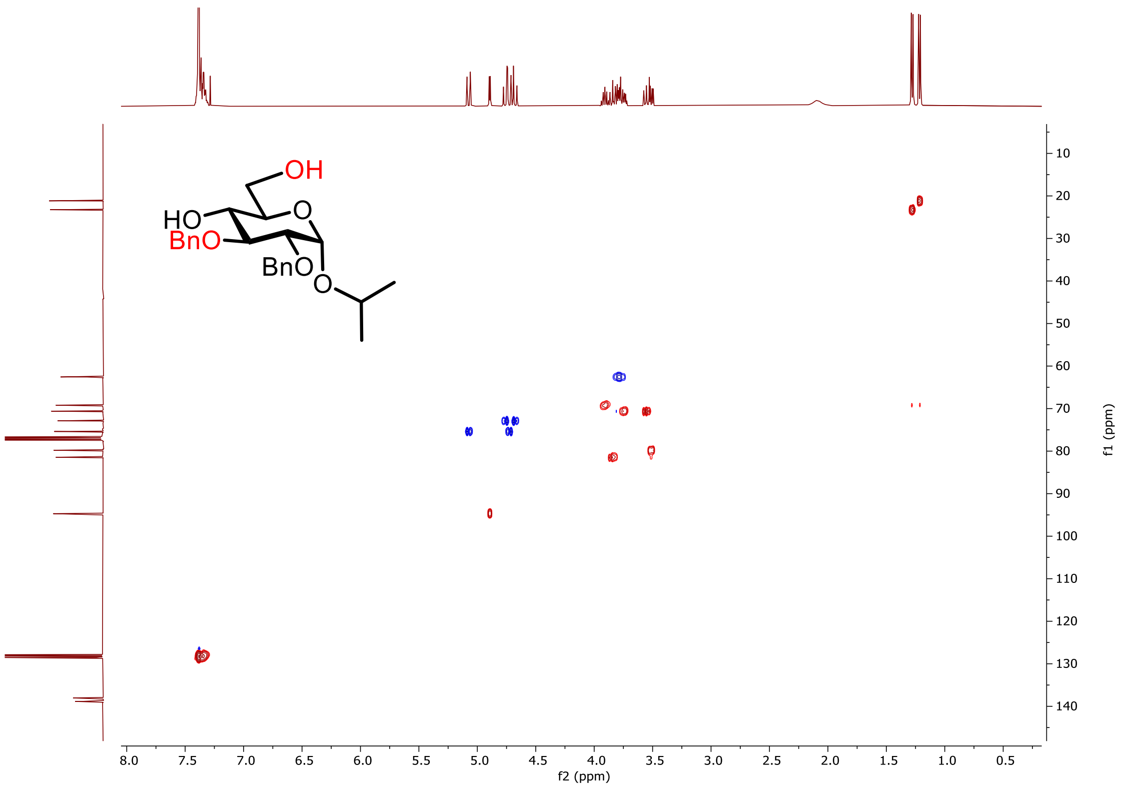

4.8 Isopropyl 2,3-di-*O*-benzyl-6-*O*-acryl-D-glucopyranoside, 81

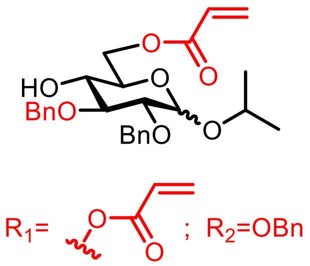

Total yield: 62% (28.3 mg). Ratio of anomer α : β = 2.0 : 1.

Spectrum data for isopropyl 2,3-di-*O*-benzyl-6-*O*-acryl-α-D-glucopyranoside **81a**: ^{1}H NMR (400 MHz, $CDCl_3$) δ 7.43 – 7.28 (m, 10H), 6.45 (dd, *J* = 17.3, 1.4 Hz, 1H), 6.16 (dd, *J* = 17.3, 10.4 Hz, 1H), 5.87 (dd, *J* = 10.4, 1.4 Hz, 1H), 5.04 (d, *J* = 11.2 Hz, 1H), 4.90 (d, *J* = 3.7 Hz, 1H), 4.80 – 4.74 (m, 2H), 4.67 (d, *J* = 12.0 Hz, 1H), 4.53 (dd, *J* = 12.1, 4.9 Hz, 1H), 4.33 (dd, *J* = 12.1, 2.2 Hz, 1H), 3.97 – 3.80 (m, 3H), 3.52 (dd, *J* = 9.6, 3.7 Hz, 1H), 3.47 (dd, *J* = 10.1, 8.8 Hz, 1H), 1.78 (br. s, 1H), 1.28 (d, *J* = 6.2 Hz, 3H), 1.22 (d, *J* = 6.1 Hz, 3H); ^{13}C NMR (101 MHz, $CDCl_3$) δ 166.49, 138.75, 138.04, 131.58, 128.63, 128.52, 128.17, 128.02, 128.00, 127.94, 127.92, 94.96, 81.29, 79.53, 75.61, 72.98, 70.12, 69.59, 69.35, 63.44, 23.17, 21.29; $[\alpha]_D^{25}$ 16.16 (c = 1, $CHCl_3$); IR (neat) ν_{max} = 2974, 1727, 1058, 689 cm^{-1}; m/z (HRMS$^+$) $[M + Na]^+$ 479.2024 ($C_{26}H_{32}O_7Na^+$ requires 479.2040).

^{1}H NMR of crude 81 (600 MHz, $CDCl_3$)

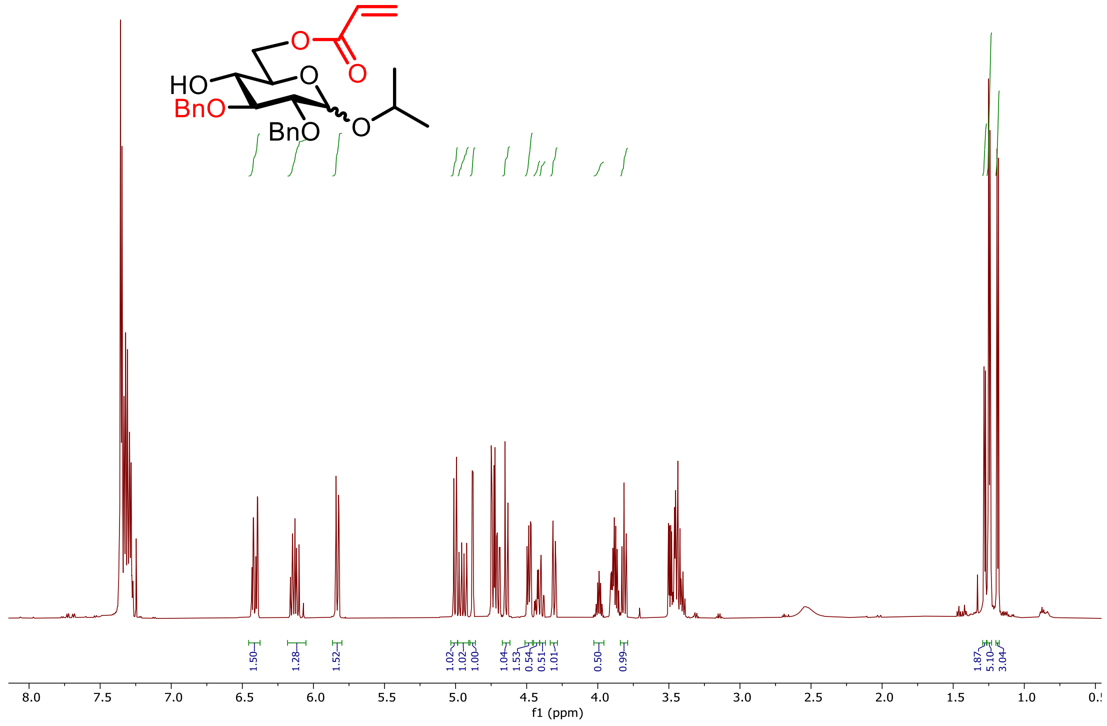

^{13}C NMR of crude 81 (151 MHz, $CDCl_3$)

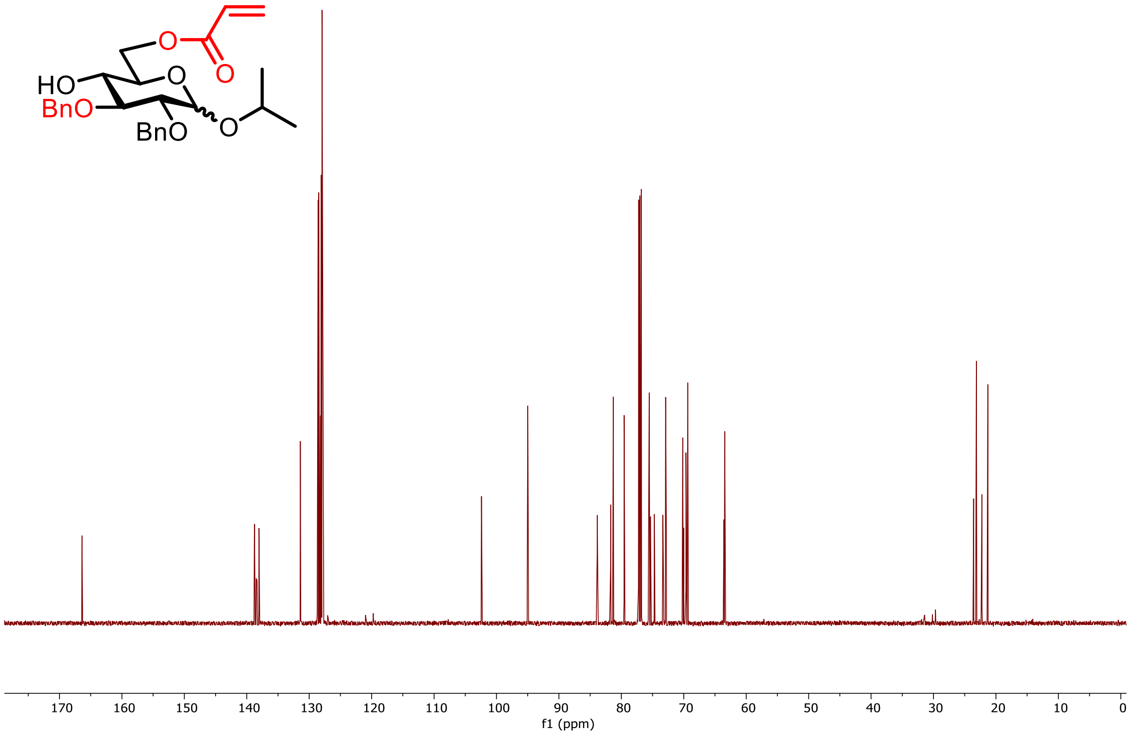

HSQC NMR of crude 81 ($CDCl_3$)

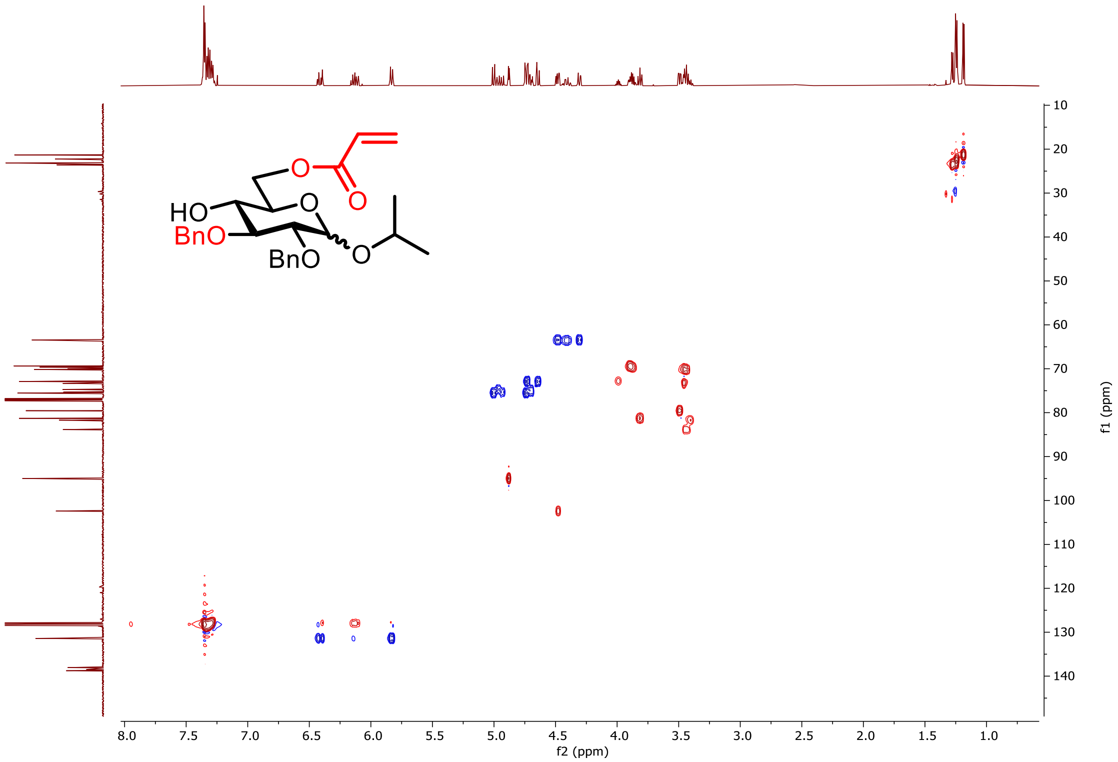

Coupled HSQC NMR of crude 81 ($CDCl_3$)

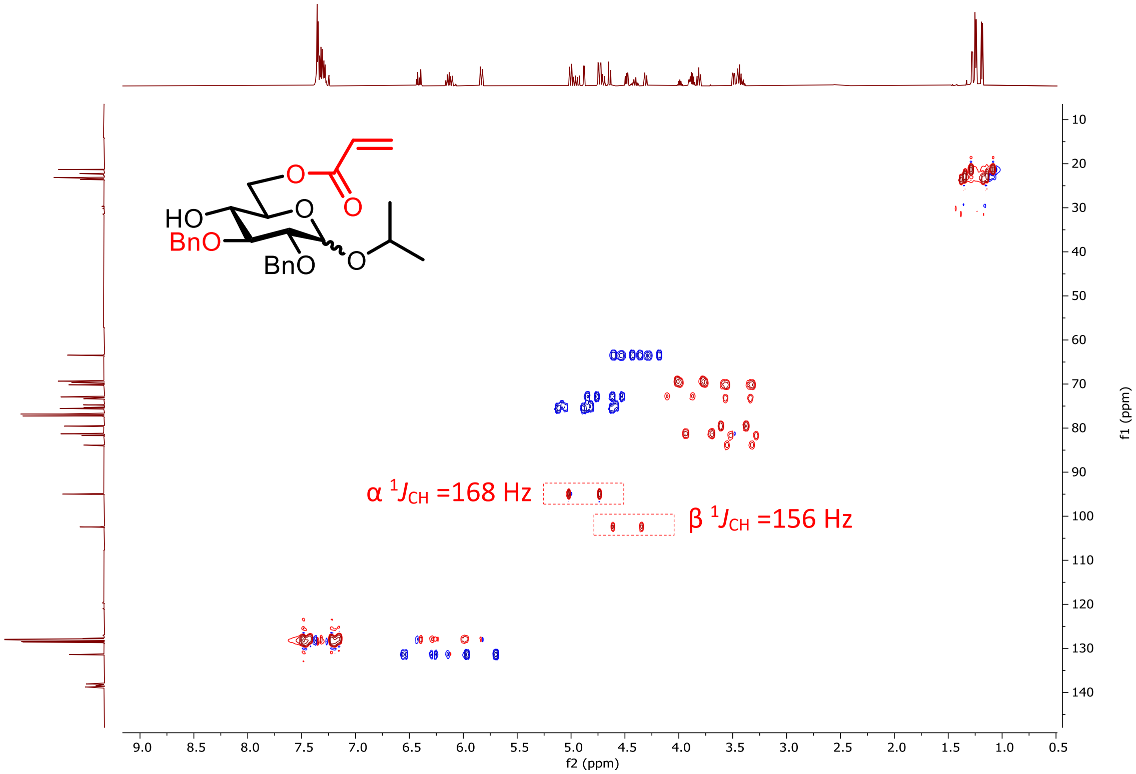

^{1}H NMR of 81a (400 MHz, $CDCl_3$)

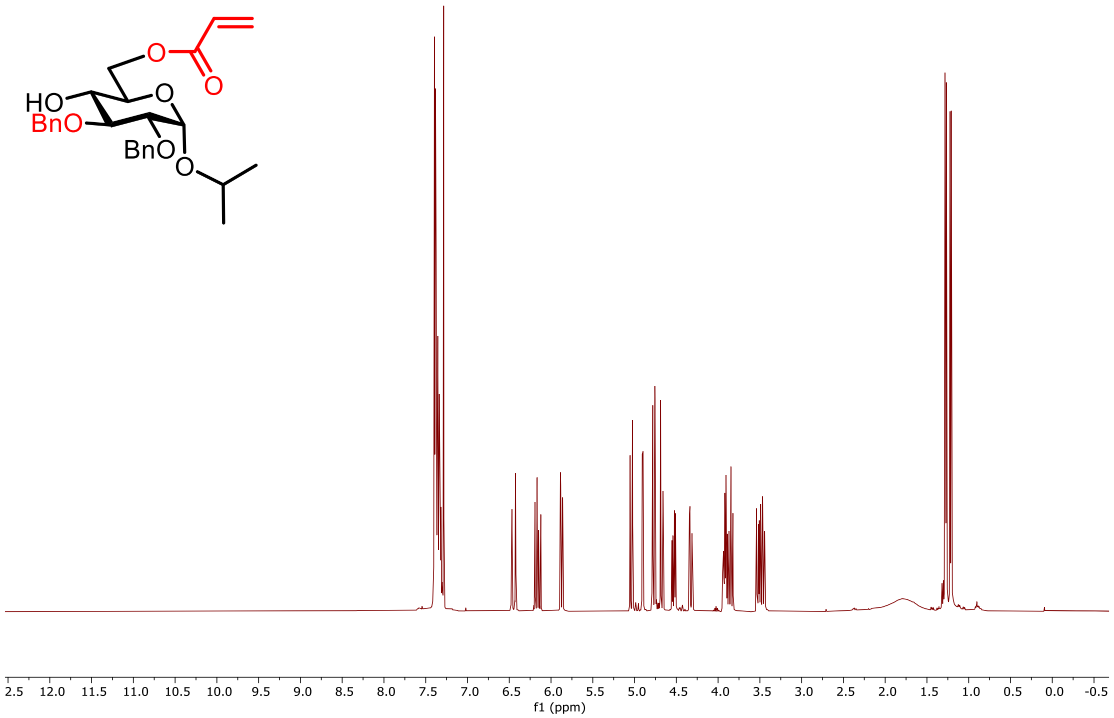

^{13}C NMR of 81a (101 MHz, $CDCl_3$)

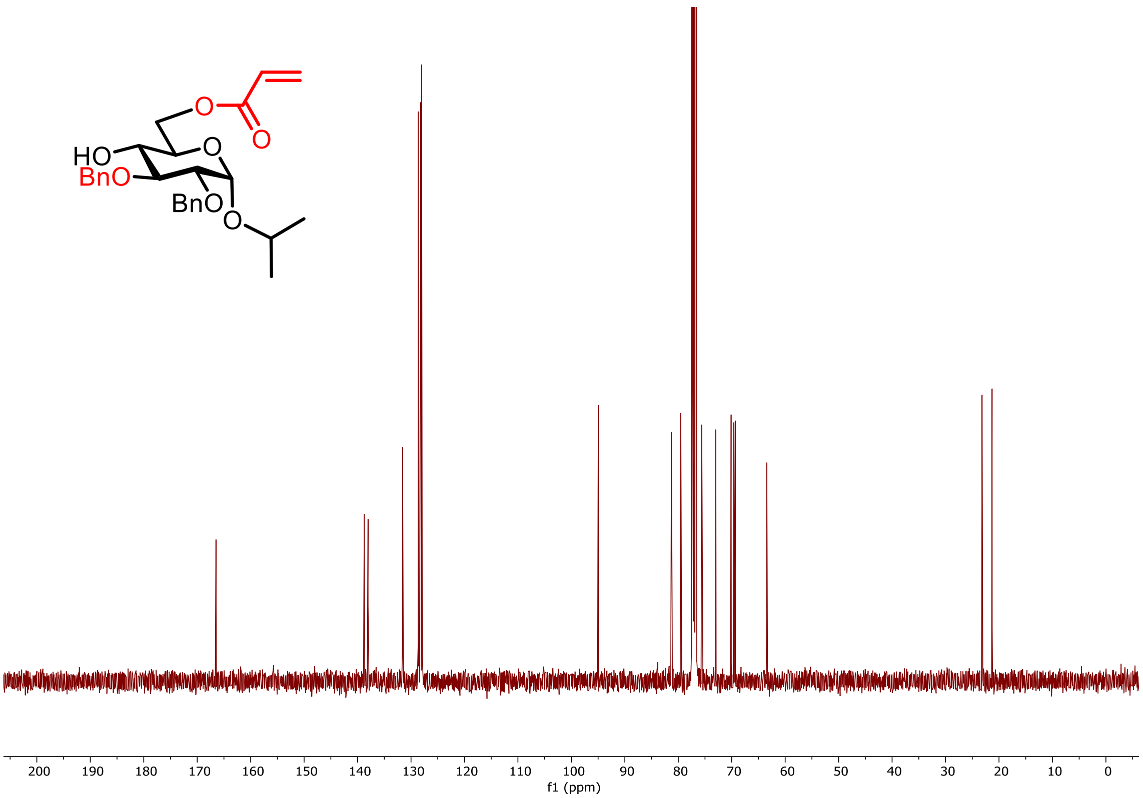

COSY NMR of 81a ($CDCl_3$)

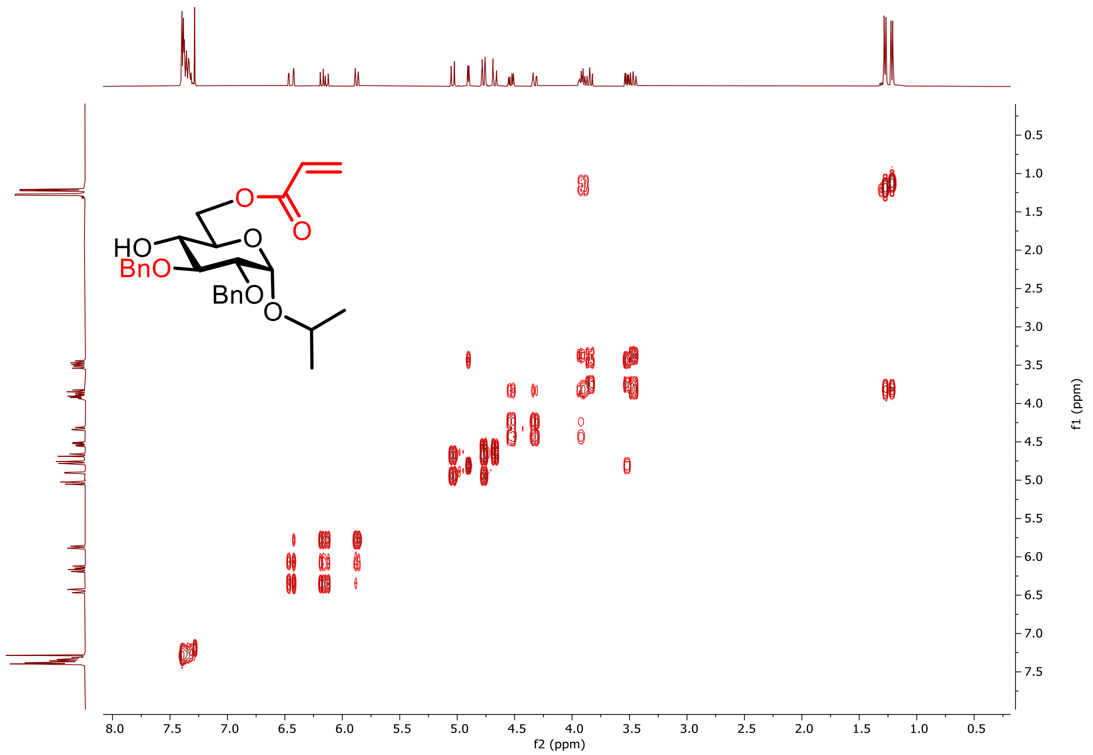

HSQC NMR of 81a ($CDCl_3$)

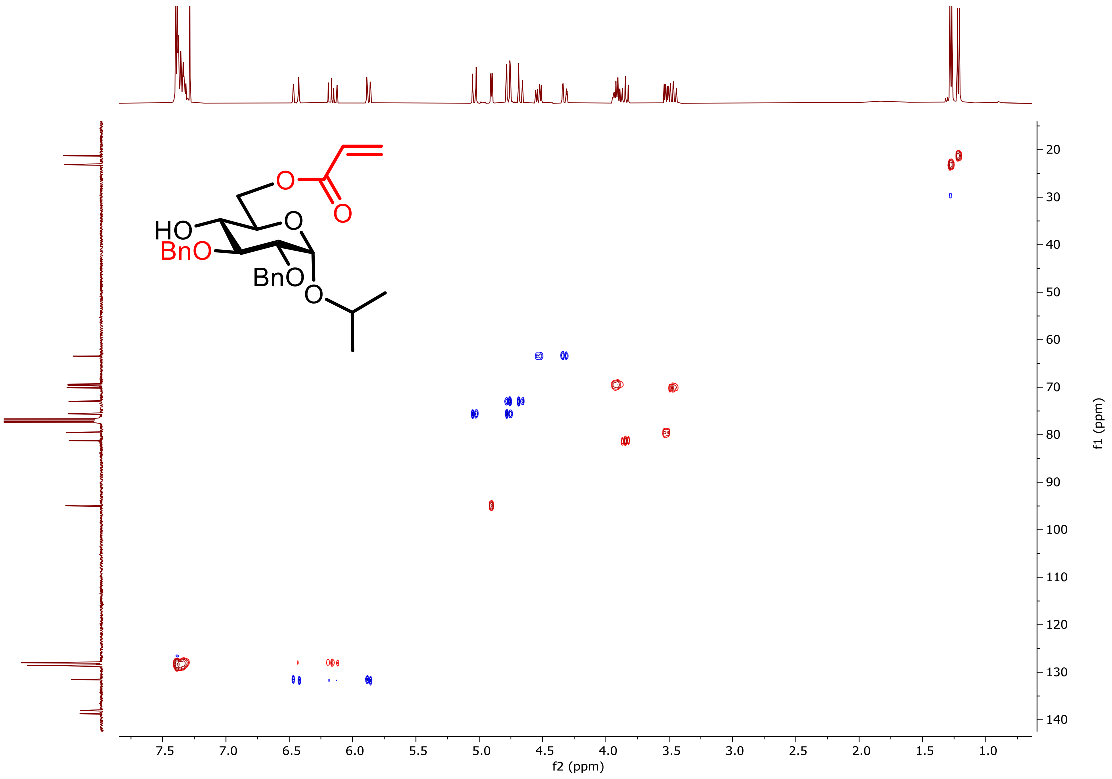

4.9 Isopropyl 2,3-di-*O*-benzyl-6-*O*-benzoyl-D-glucopyranoside, 82

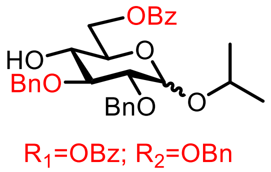

Total yield: 93% (47.1 mg). Ratio of anomer α : β = 1.7 : 1.

Spectrum data for isopropyl 2,3-di-*O*-benzyl-6-*O*-benzoyl-α-D-glucopyranoside **82a**: ^{1}H NMR (400 MHz, $CDCl_3$) δ 8.07 – 8.03 (m, 2H), 7.63 – 7.54 (m, 1H), 7.50 – 7.41 (m, 2H), 7.40 – 7.31 (m, 10H), 5.05 (d, *J* = 11.2 Hz, 1H), 4.92 (d, *J* = 3.7 Hz, 1H), 4.81 – 4.74 (m, 2H), 4.71 – 4.63 (m, 2H), 4.51 (dd, *J* = 12.0, 2.2 Hz, 1H), 4.02 (ddd, *J* = 10.2, 4.8, 2.1 Hz, 1H), 3.99 – 3.83 (m, 2H), 3.60 – 3.52 (m, 2H), 2.60 (br. s, 1H), 1.29 (d, *J* = 6.4 Hz, 3H), 1.21 (d, *J* = 6.1 Hz, 3H); ^{13}C NMR (101 MHz, $CDCl_3$) δ 166.87, 138.72, 138.04, 133.17, 129.78, 129.76, 128.65, 128.52, 128.38, 128.16, 128.06, 128.00, 127.94, 94.91, 81.37, 79.65, 75.66, 72.97, 70.26, 69.54, 69.46, 63.85, 23.22, 21.29; $[\alpha]_D^{25}$ 24.00 (c = 1, $CHCl_3$); IR (neat) ν_{max} = 2925, 1722, 1276, 1062, 712 cm^{-1}; m/z (HRMS$^+$) $[M + Na]^+$ 529.2198 ($C_{30}H_{34}O_7Na^+$ requires 529.2197).

^{1}H NMR of crude 82 (600 MHz, $CDCl_3$)

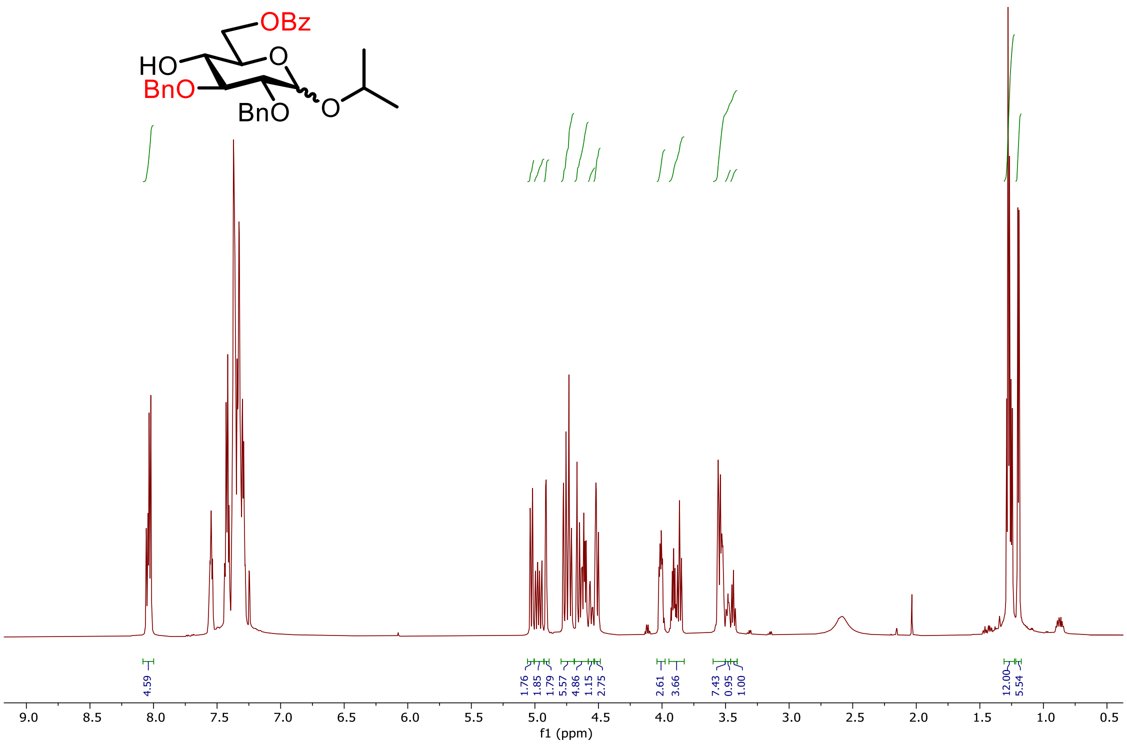

^{13}C NMR of crude 82 (151 MHz, $CDCl_3$)

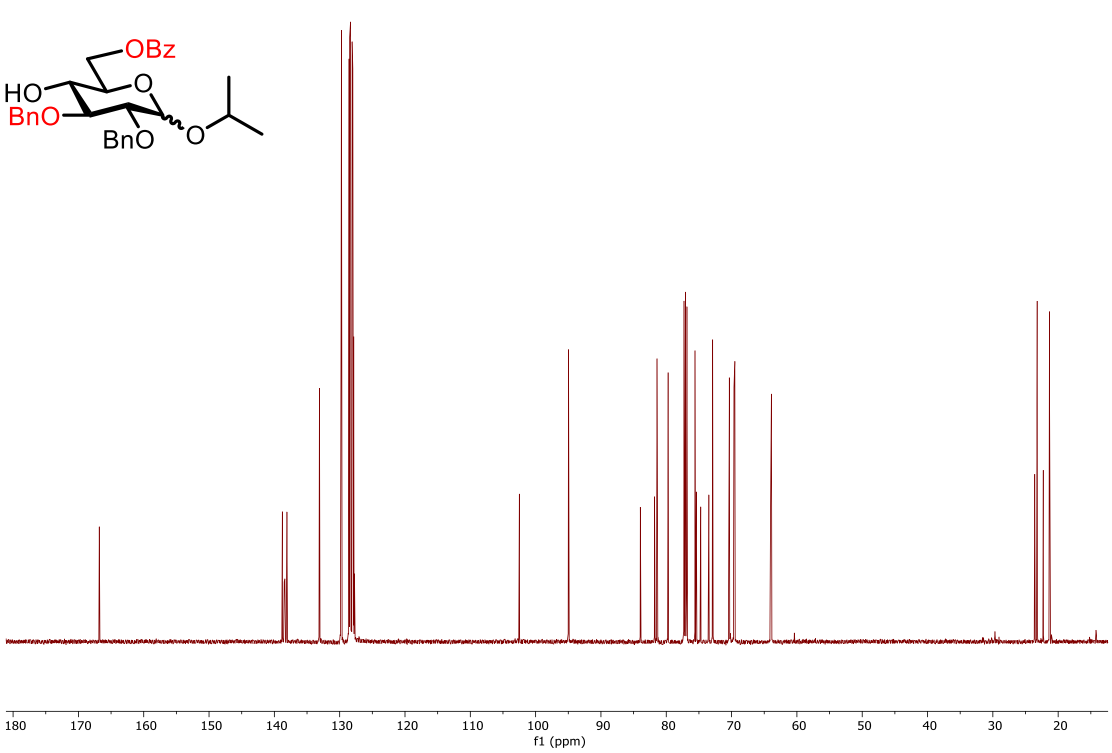

HSQC NMR of crude 82 ($CDCl_3$)

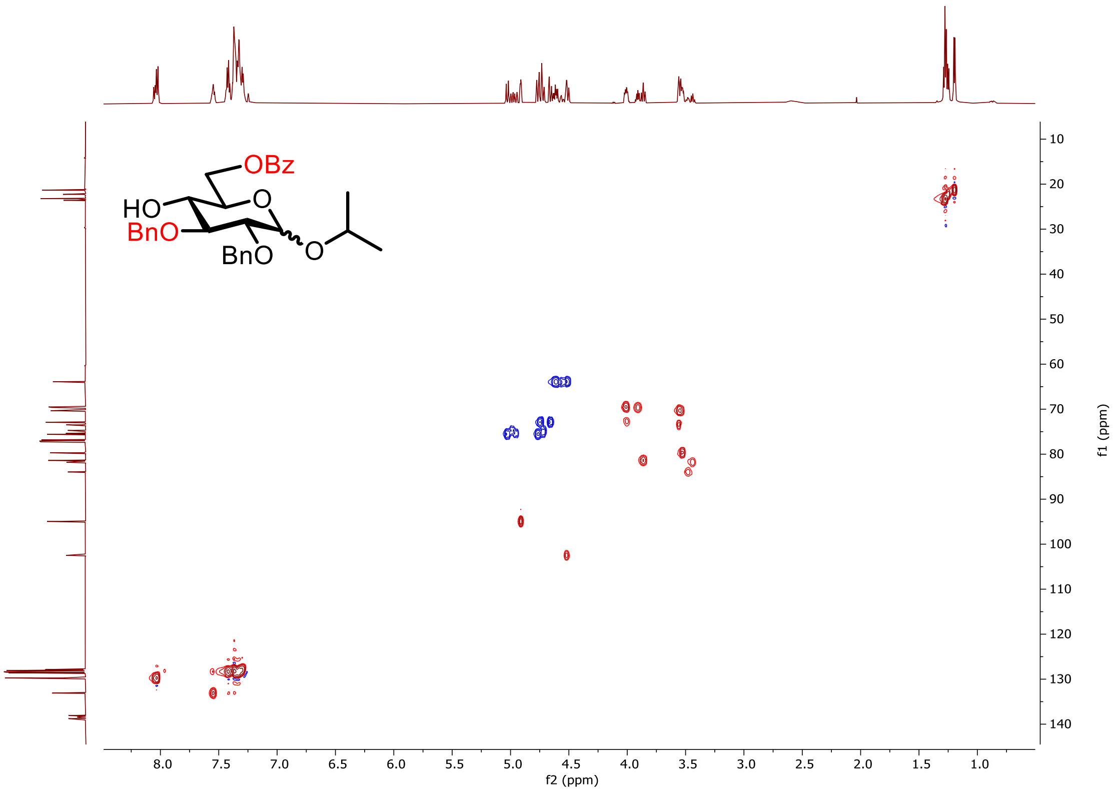

Coupled HSQC NMR of crude 82 ($CDCl_3$)

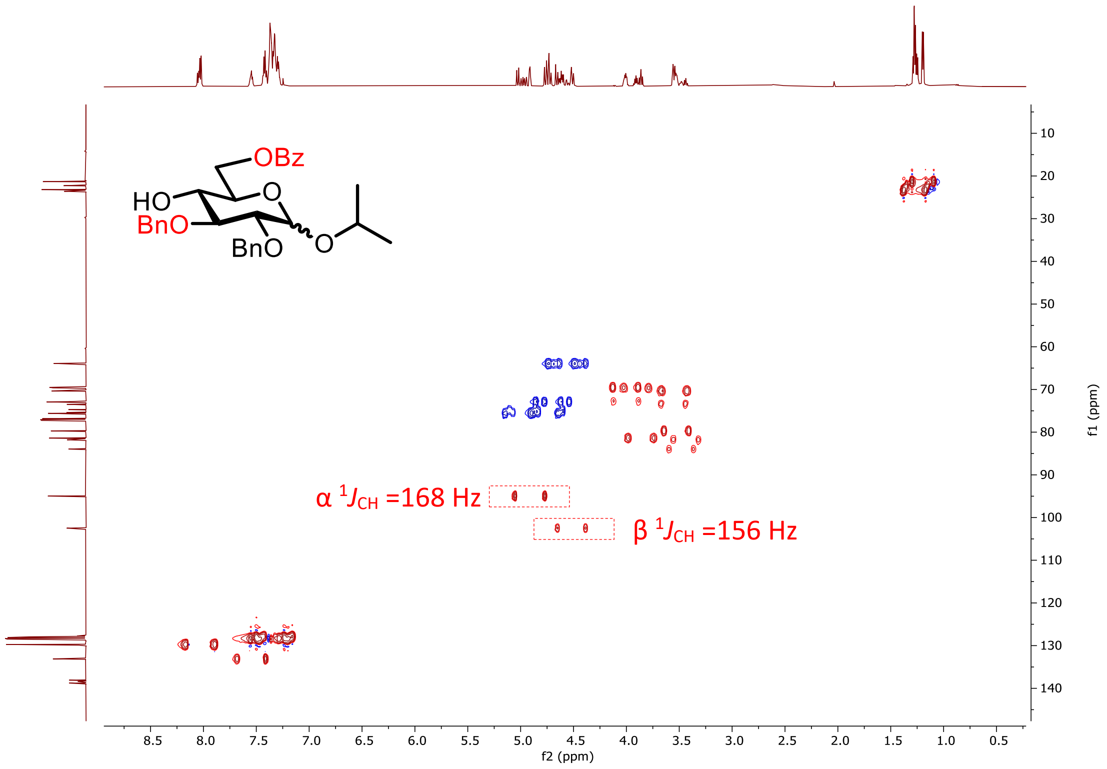

^{1}H NMR of 82a (400 MHz, $CDCl_3$)

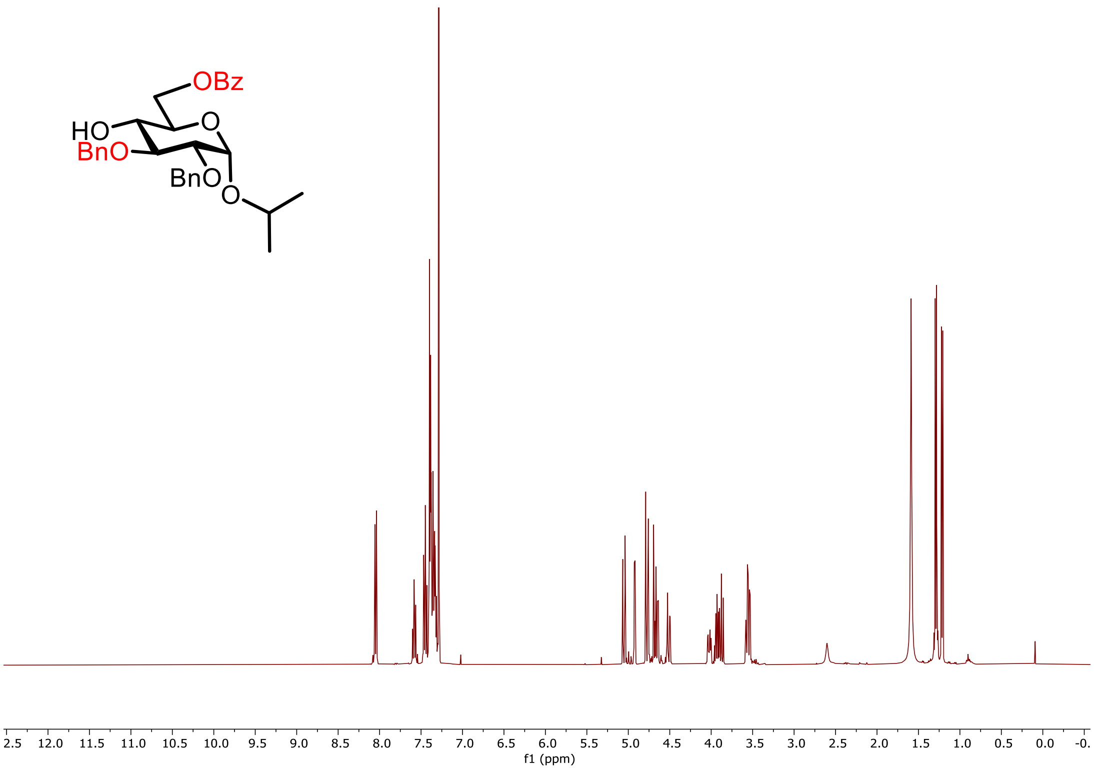

^{13}C NMR of 82a (101 MHz, $CDCl_3$)

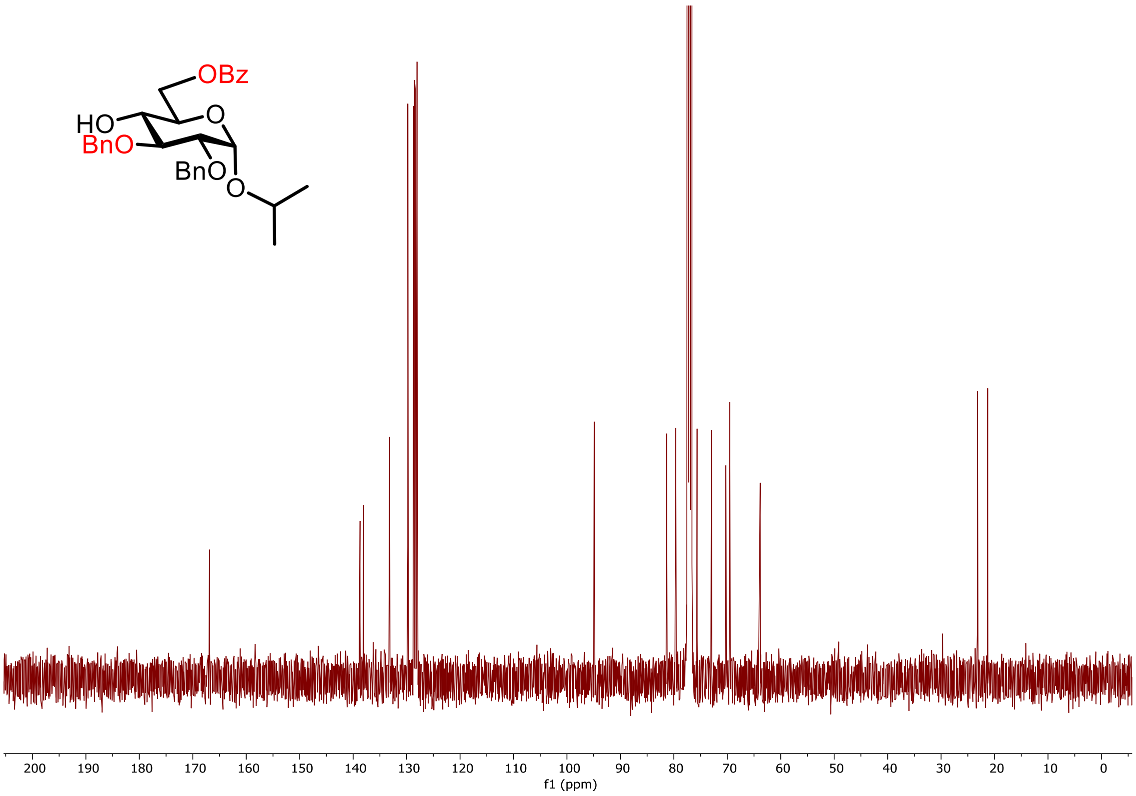

COSY NMR of 82a ($CDCl_3$)

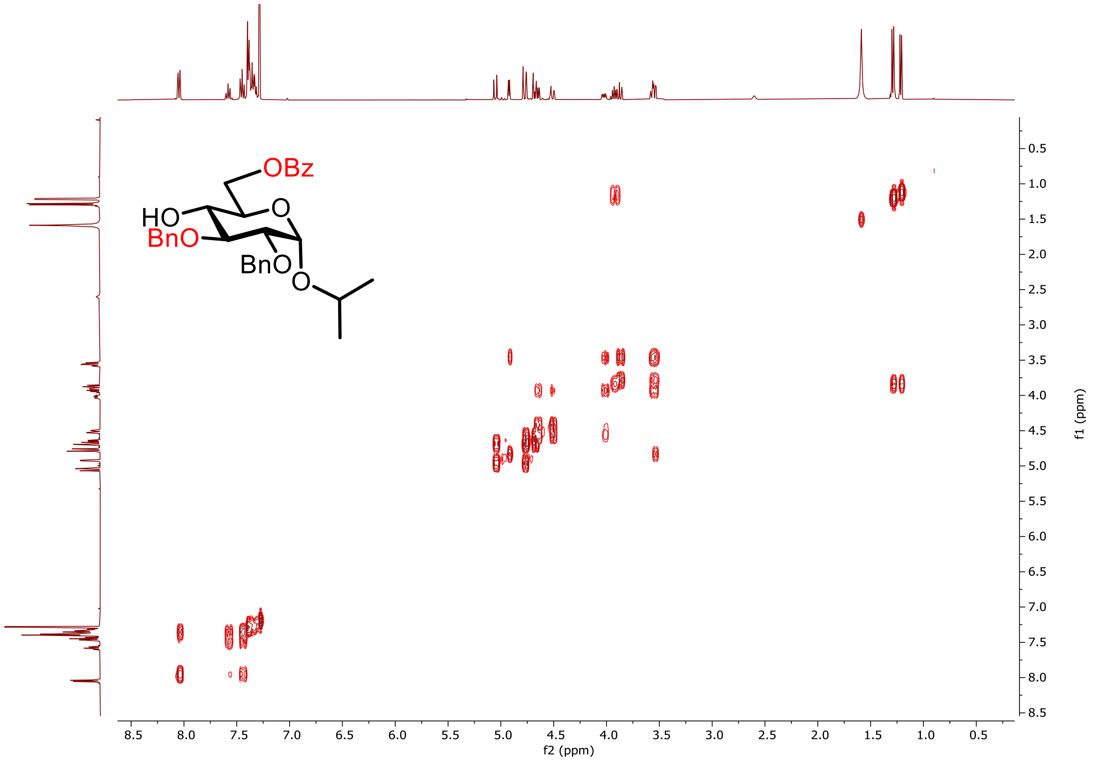

HSQC NMR of 82a ($CDCl_3$)

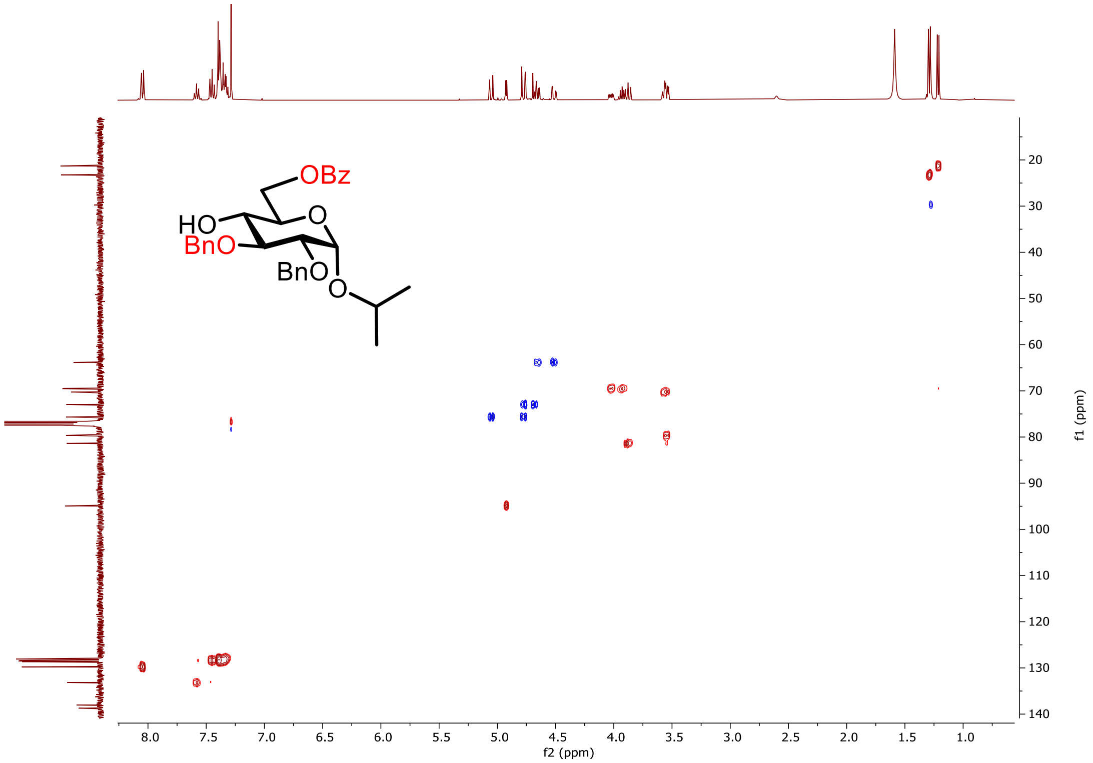

4.10 Isopropyl 2,3-di-*O*-benzyl-6-*O*-(4-methylbenzoyl)-D-glucopyranoside, 83

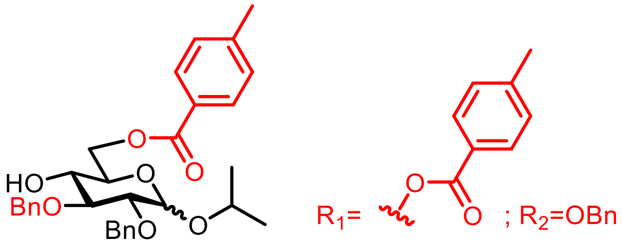

Total yield: 92% (47.9 mg). Ratio of anomer α : β = 2.1 : 1.

Spectrum data for isopropyl 2,3-di-*O*-benzyl-6-*O*-(4-methylbenzoyl)-α-D-glucopyranoside **83a**: ^{1}H NMR (400 MHz, $CDCl_3$) δ 7.96 – 7.91 (m, 2H), 7.45 – 7.29 (m, 10H), 7.24 (d, *J* = 8.0 Hz, 2H), 5.05 (d, *J* = 11.2 Hz, 1H), 4.92 (d, *J* = 3.7 Hz, 1H), 4.80 (d, *J* = 4.5 Hz, 1H), 4.77 (d, *J* = 5.2 Hz, 1H), 4.72 – 4.61 (m, 2H), 4.49 (dd, *J* = 12.1, 2.1 Hz, 1H), 4.02 (ddd, *J* = 10.0, 4.9, 2.1 Hz, 1H), 3.97 – 3.85 (m, 2H), 3.60 – 3.50 (m, 2H), 2.67 (s, 1H), 2.43 (s, 3H), 1.29 (d, *J* = 6.3 Hz, 3H), 1.22 (d, *J* = 6.1 Hz, 3H); ^{13}C NMR (101 MHz, $CDCl_3$) δ 166.99, 143.89, 138.76, 138.07, 129.81, 129.10, 128.64, 128.52, 128.16, 128.06, 127.99, 127.92, 127.02, 94.91, 81.38, 79.64, 75.66, 72.98, 70.29, 69.55, 69.51, 63.69, 23.22, 21.73, 21.30; $[\alpha]_D^{25}$ 33.55 (c = 1, $CHCl_3$); IR (neat) ν_{max} = 2923, 1718, 1276, 1060, 753 cm^{-1}; m/z (HRMS$^+$) [M + Na]$^+$ 543.2352 ($C_{31}H_{36}O_7Na^+$ requires 543.2353).

^{1}H NMR of crude 83 (600 MHz, $CDCl_3$)

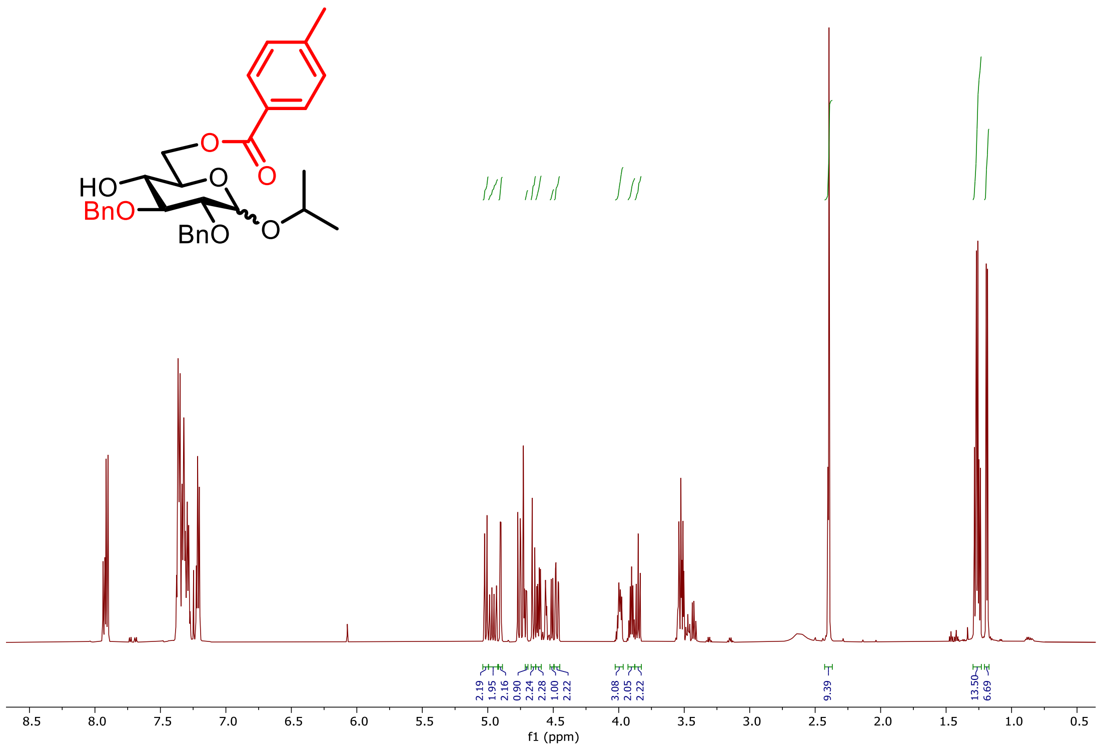

^{13}C NMR of crude 83 (151 MHz, $CDCl_3$)

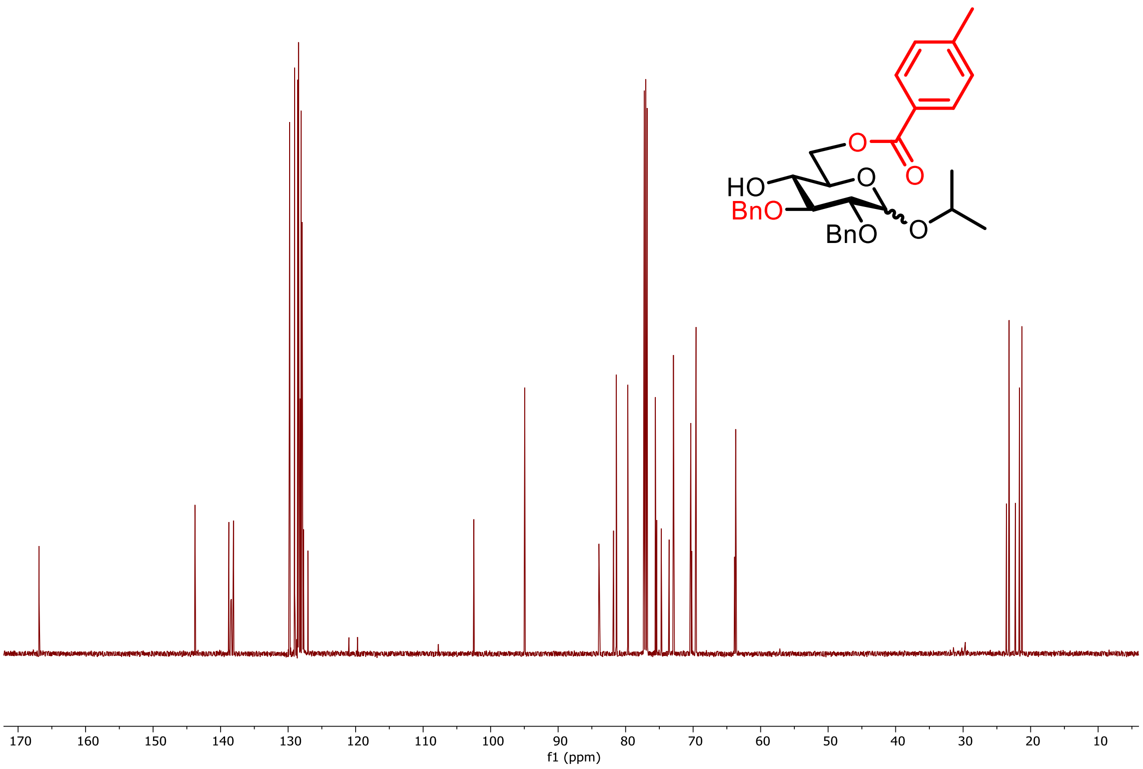

HSQC NMR of crude 83 ($CDCl_3$)

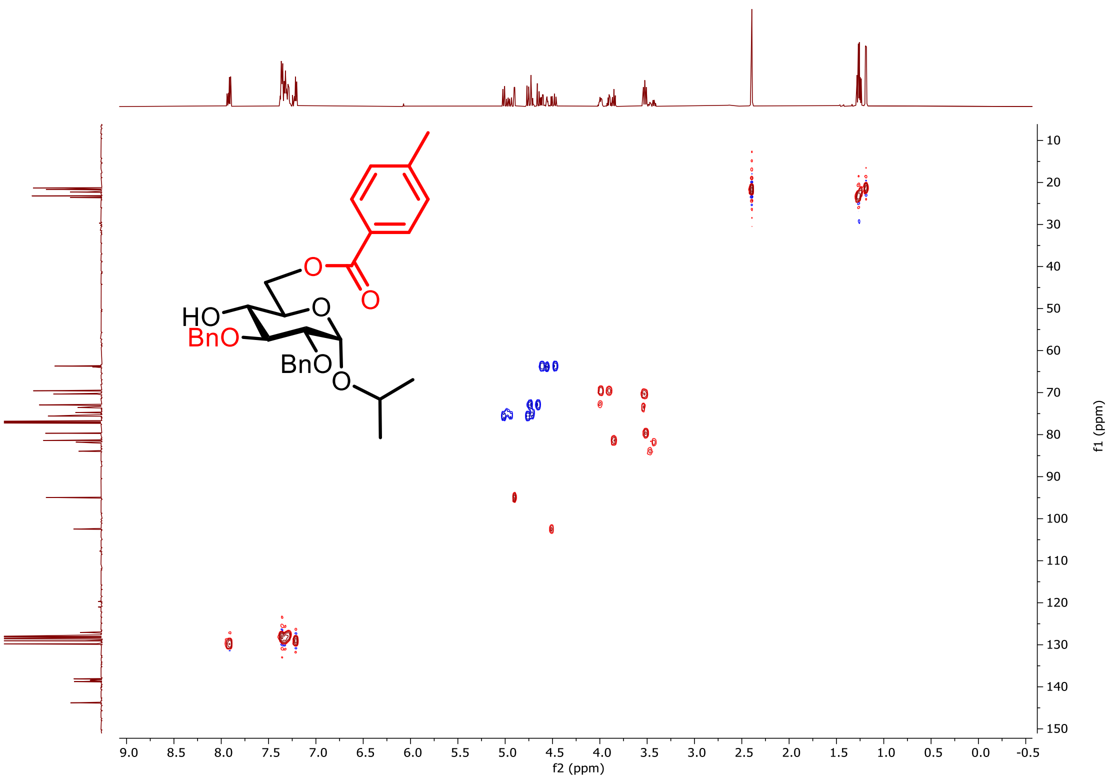

Coupled HSQC NMR of crude 83 ($CDCl_3$)

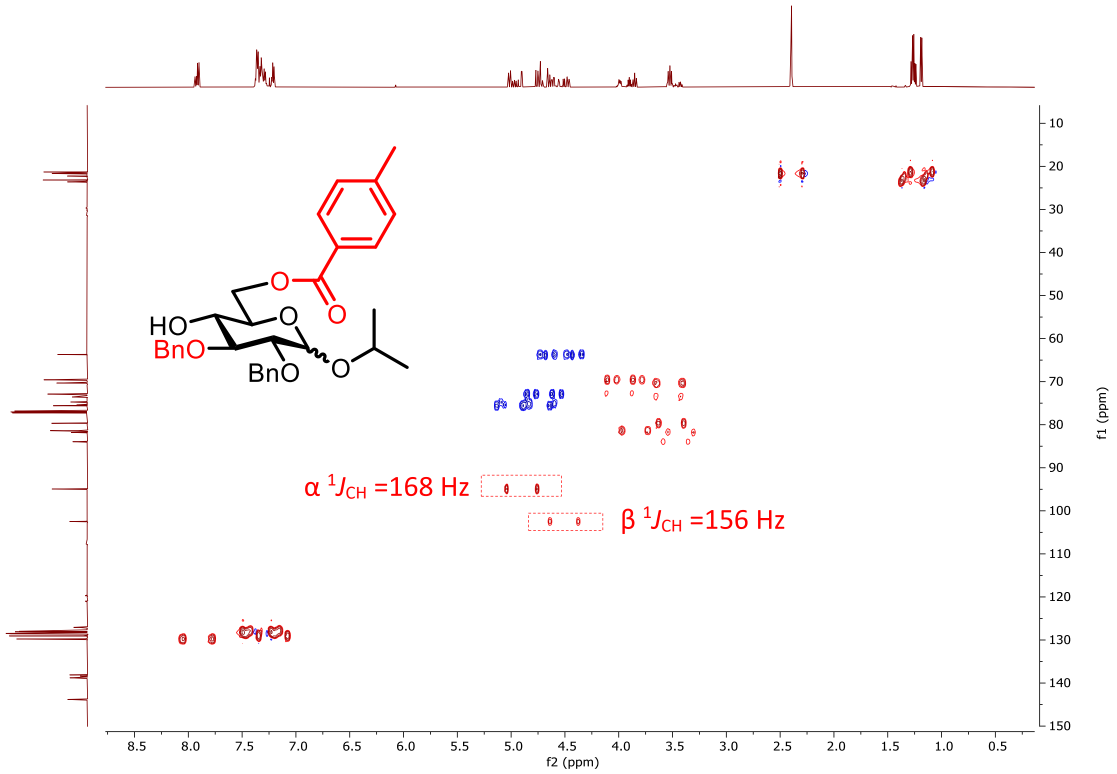

^{1}H NMR of 83a (400 MHz, $CDCl_3$)

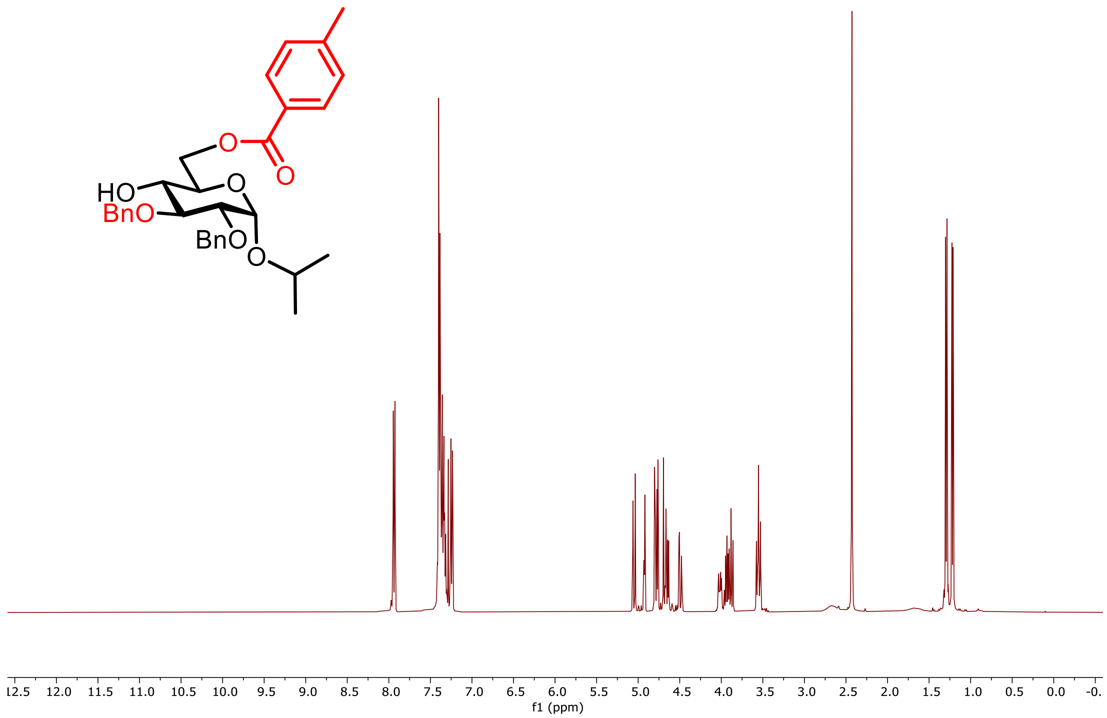

^{13}C NMR of 83a (101 MHz, $CDCl_3$)

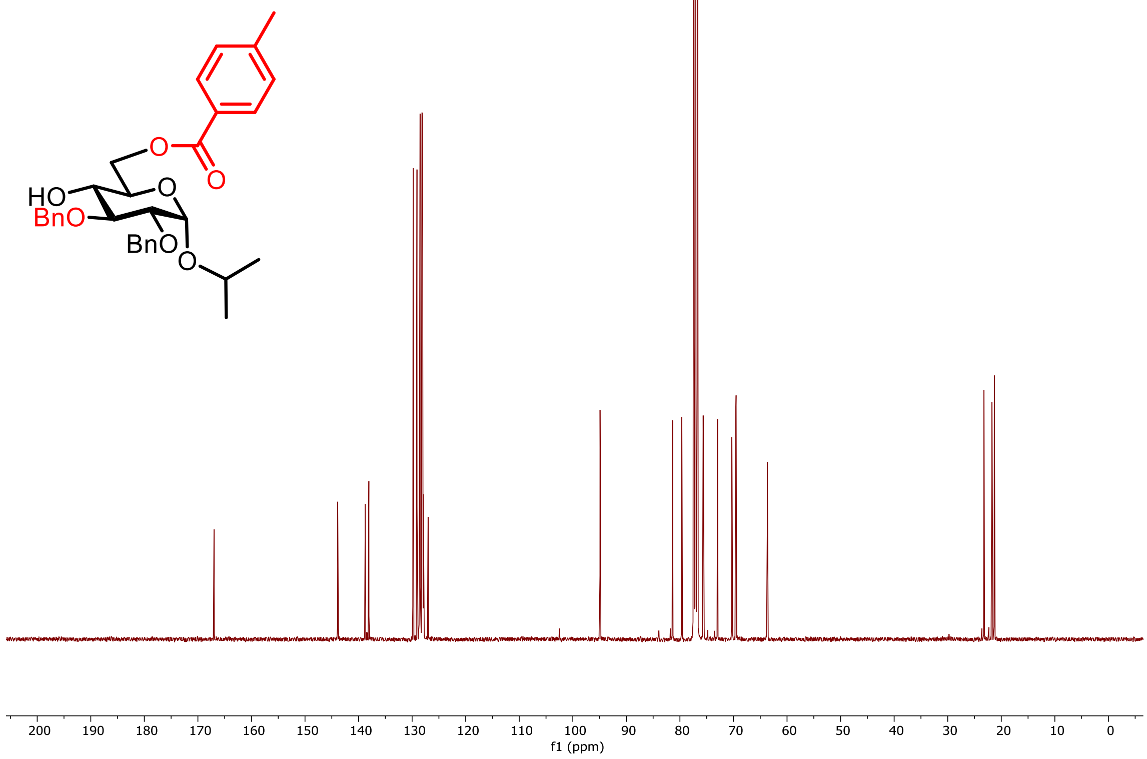

COSY NMR of 83a ($CDCl_3$)

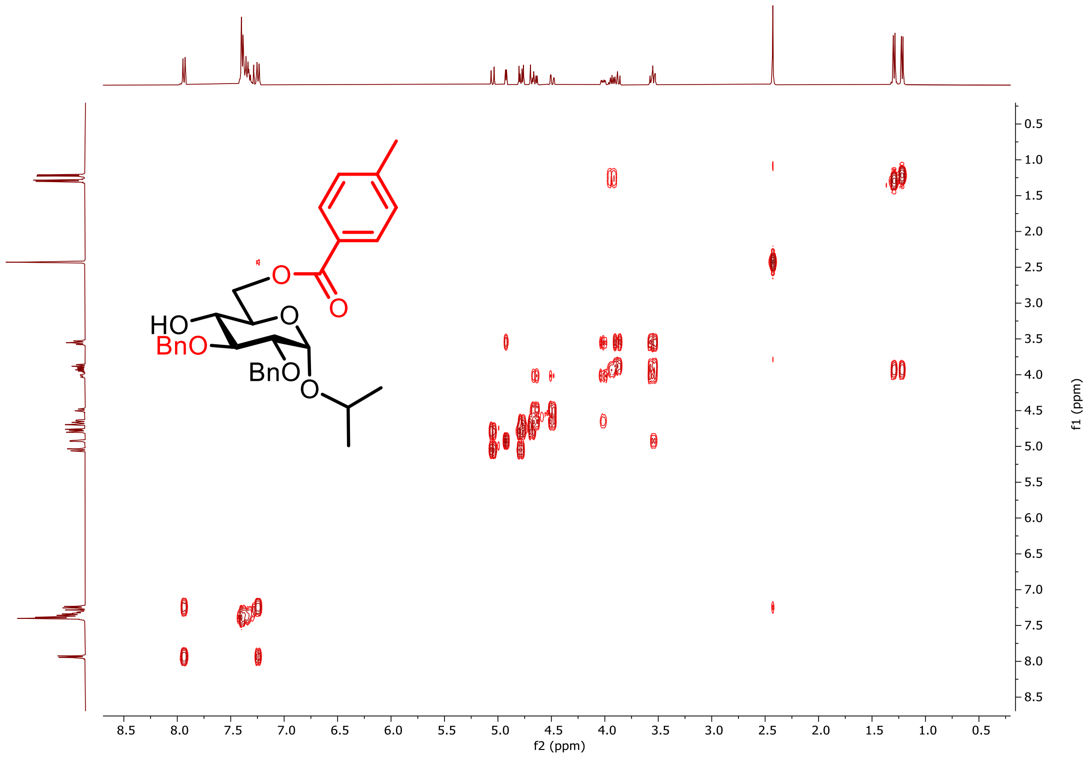

HSQC NMR of 83a ($CDCl_3$)

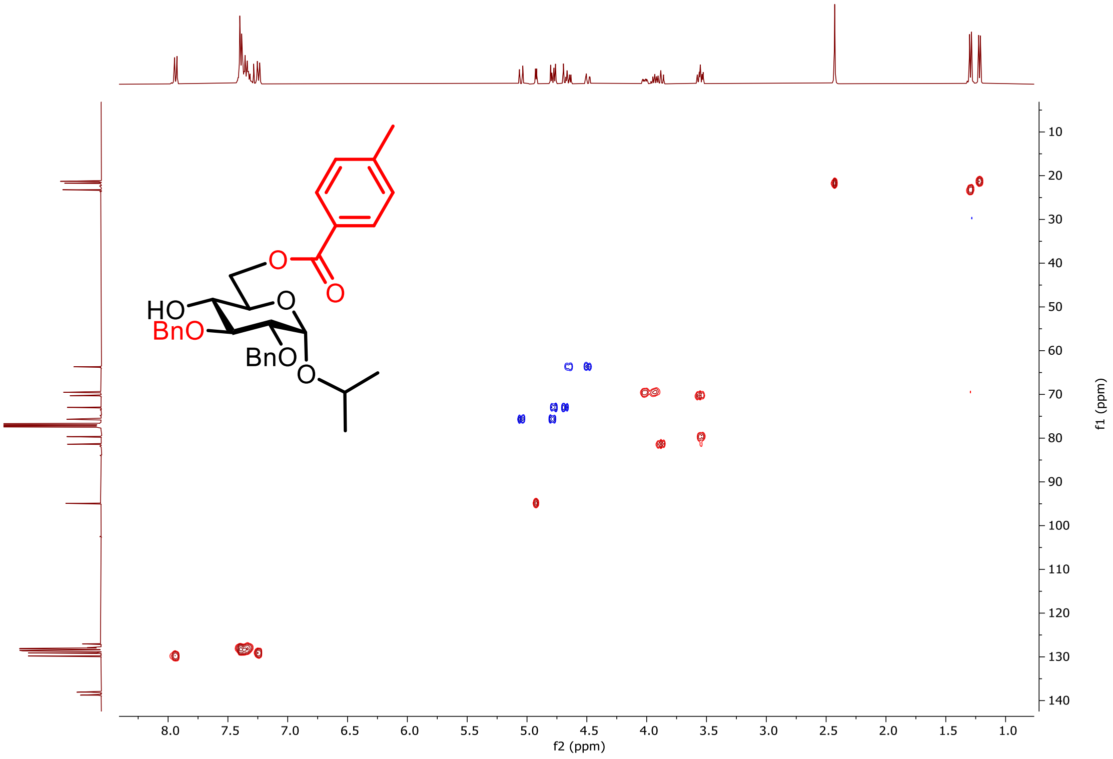

4.11 Isopropyl 2,3-di-*O*-benzyl-6-*O*-(4-methoxybenzoyl)-D-glucopyranoside, 84

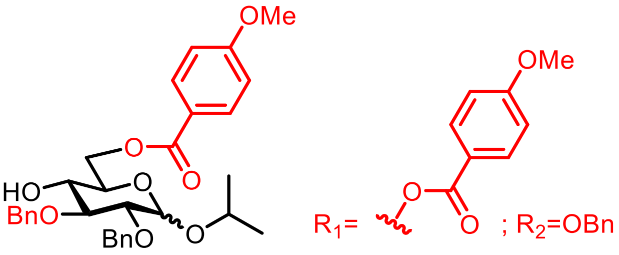

Total yield: 90% (48.3 mg). Ratio of anomer α : β = 2.2 : 1.

Spectrum data for isopropyl 2,3-di-*O*-benzyl-6-*O*-(4-methoxybenzoyl)-α-D-glucopyranoside **84a**: ^{1}H NMR (400 MHz, $CDCl_3$) δ 8.06 – 7.96 (m, 2H), 7.44 – 7.27 (m, 10H), 6.97 – 6.88 (m, 2H), 5.04 (d, *J* = 11.2 Hz, 1H), 4.92 (d, *J* = 3.7 Hz, 1H), 4.80 (d, *J* = 5.9 Hz, 1H), 4.77 (d, *J* = 6.5 Hz, 1H), 4.71 – 4.62 (m, 2H), 4.46 (dd, *J* = 12.1, 2.1 Hz, 1H), 4.00 (ddd, *J* = 10.0, 4.8, 2.1 Hz, 1H), 3.96 – 3.82 (m, 5H), 3.59 – 3.49 (m, 2H), 2.70 (br. s, 1H), 1.29 (d, *J* = 6.4 Hz, 3H), 1.22 (d, *J* = 6.1 Hz, 3H); ^{13}C NMR (101 MHz, $CDCl_3$) δ 166.71, 163.53, 138.76, 138.08, 131.86, 128.63, 128.51, 128.15, 128.06, 127.98, 127.90, 122.13, 113.61, 94.92, 81.34, 79.62, 75.67, 72.98, 70.28, 69.62, 69.49, 63.55, 55.47, 23.22, 21.29; $[\alpha]_D^{25}$ 32.33 (c = 0.5, $CHCl_3$); IR (neat) ν_{max} = 2928, 1715, 1258, 1061, 698 cm^{-1}; m/z (HRMS$^+$) $[M + Na]^+$ 559.2305 ($C_{31}H_{36}O_8Na^+$ requires 559.2302).

^{1}H NMR of crude 84 (600 MHz, $CDCl_3$)

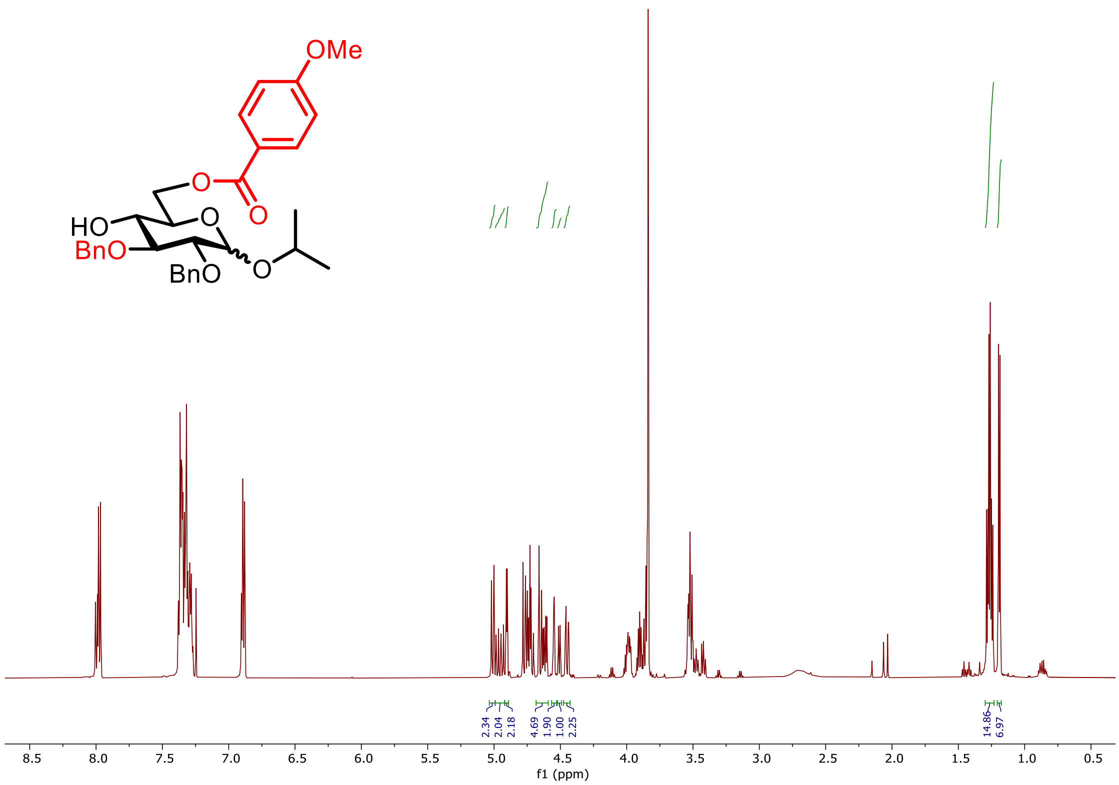

^{13}C NMR of crude 84 (151 MHz, $CDCl_3$)

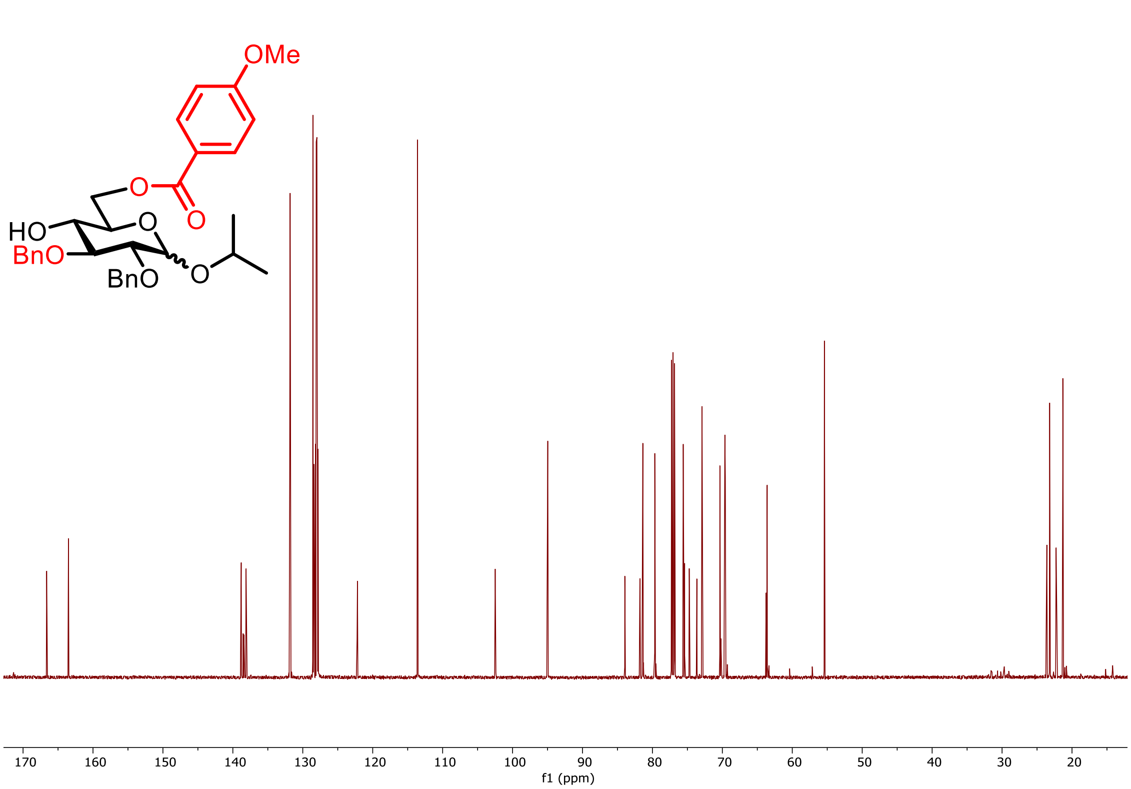

HSQC NMR of crude 84 ($CDCl_3$)

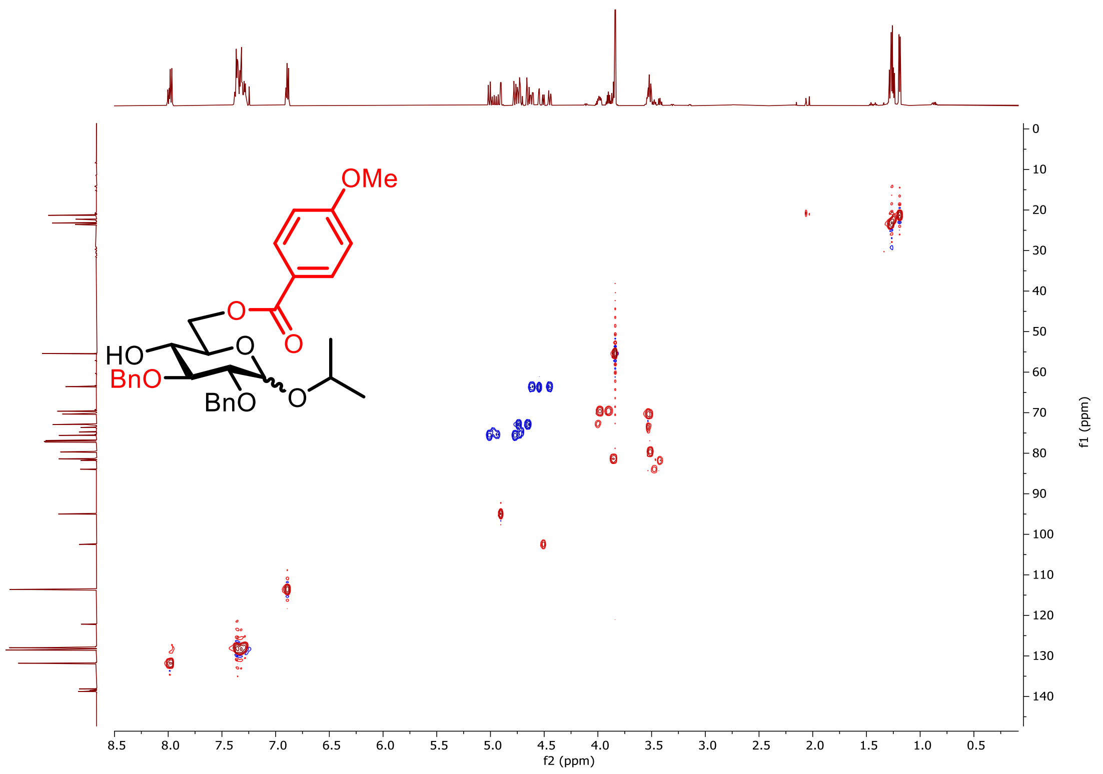

Coupled HSQC NMR of crude 84 ($CDCl_3$)

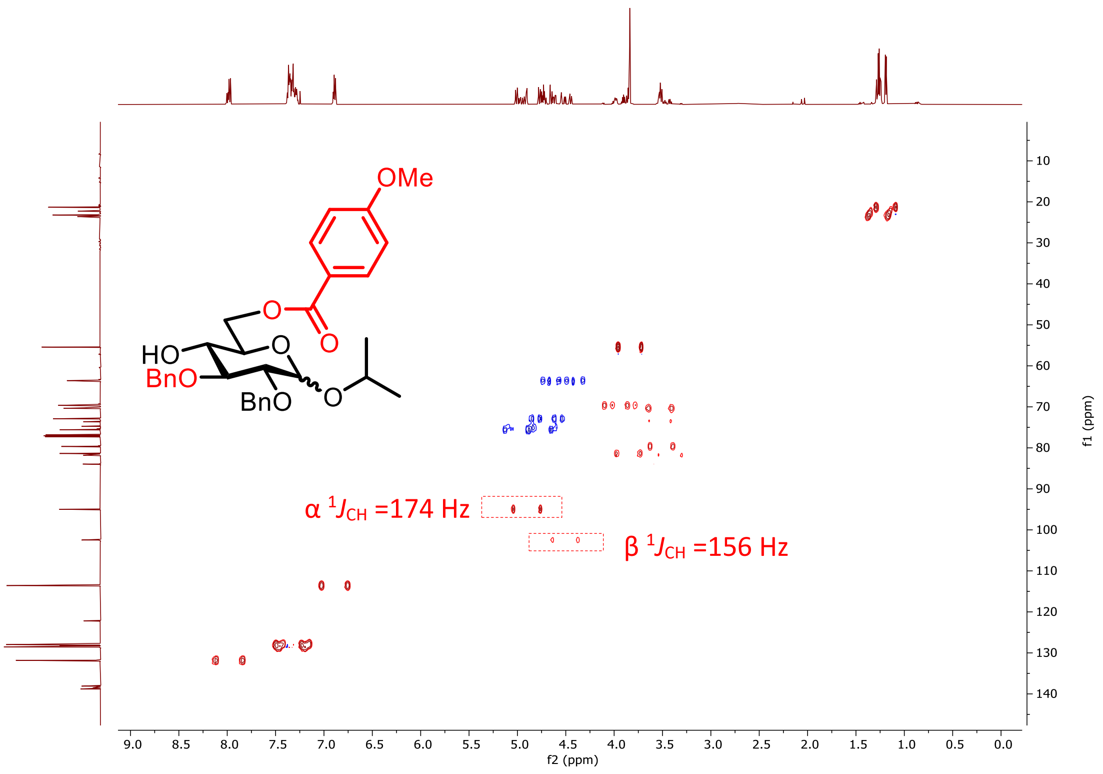

^{1}H NMR of 84a (400 MHz, $CDCl_3$)

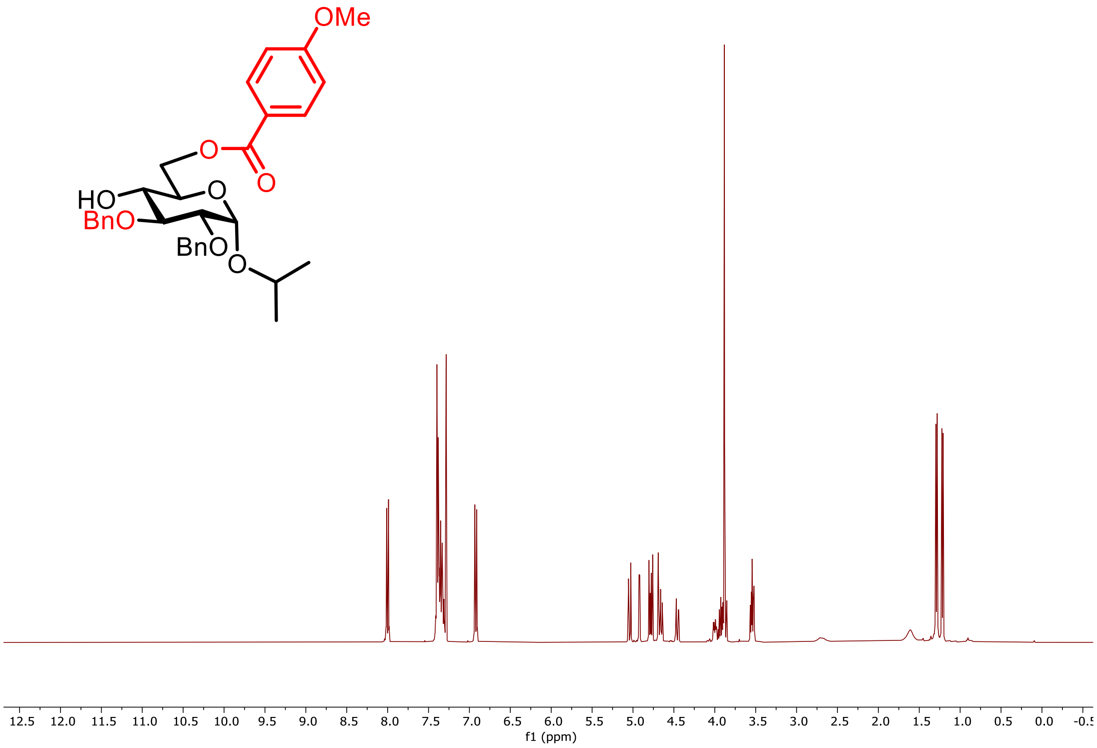

^{13}C NMR of 84a (101 MHz, $CDCl_3$)

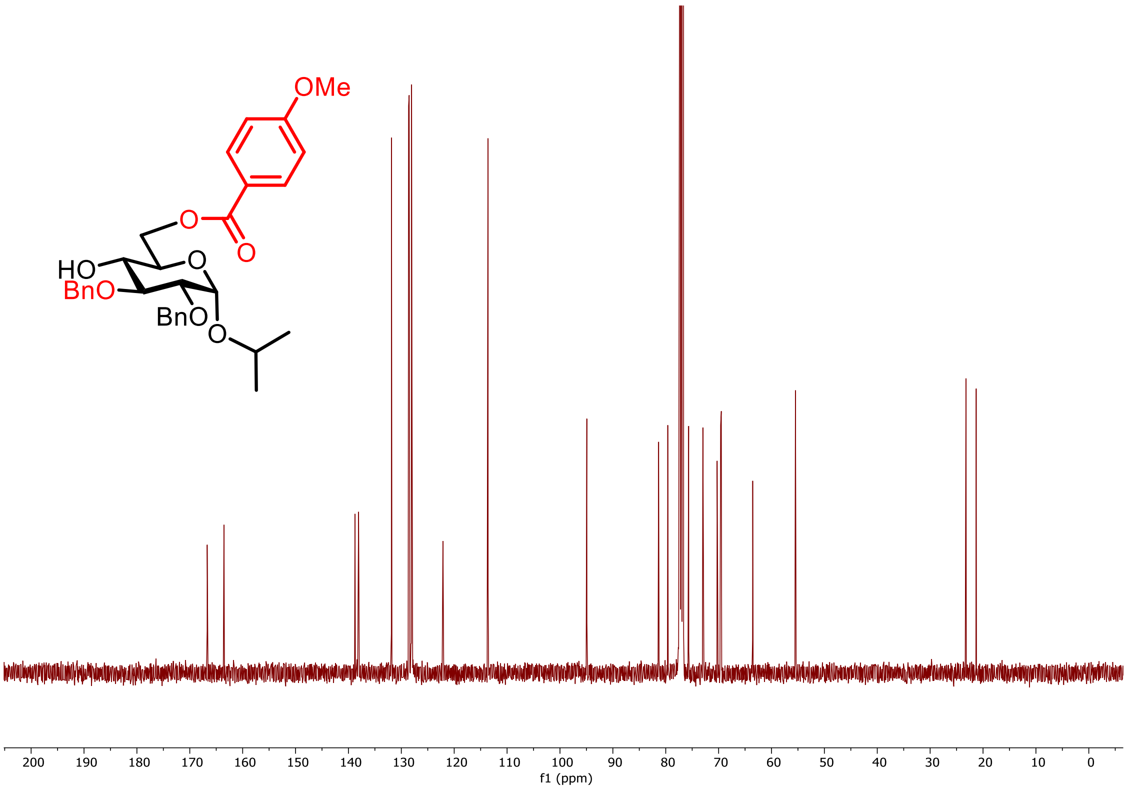

COSY NMR of 84a ($CDCl_3$)

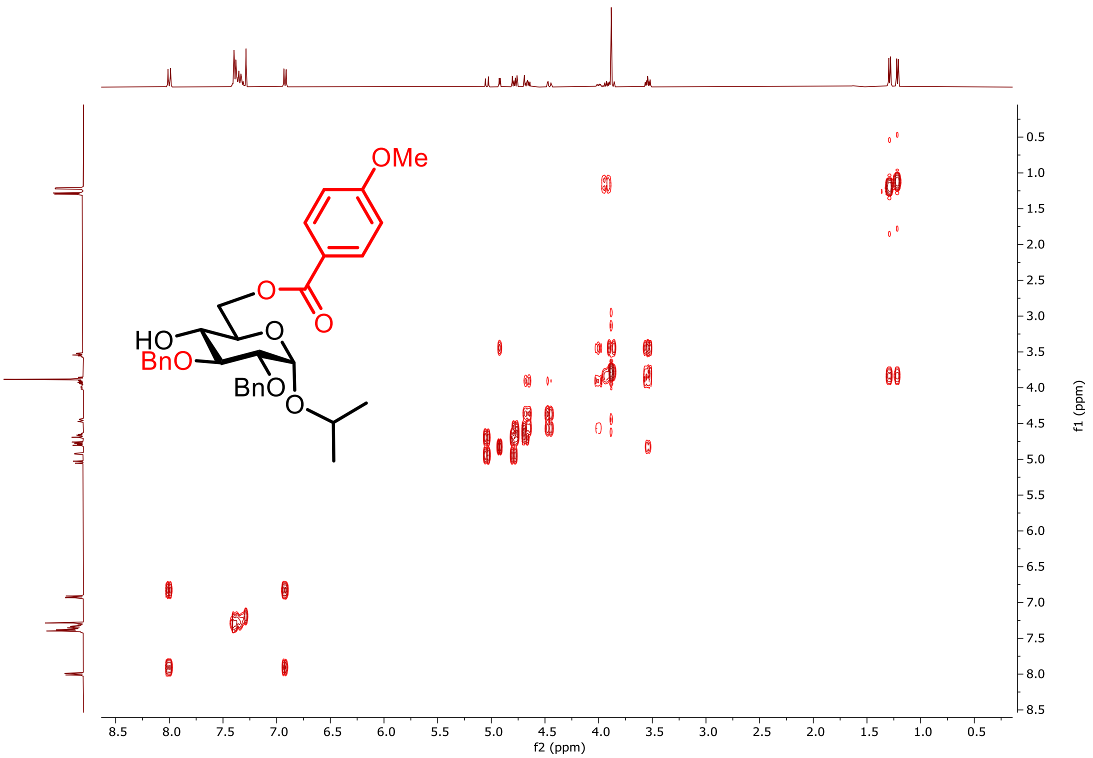

HSQC NMR of 84a ($CDCl_3$)

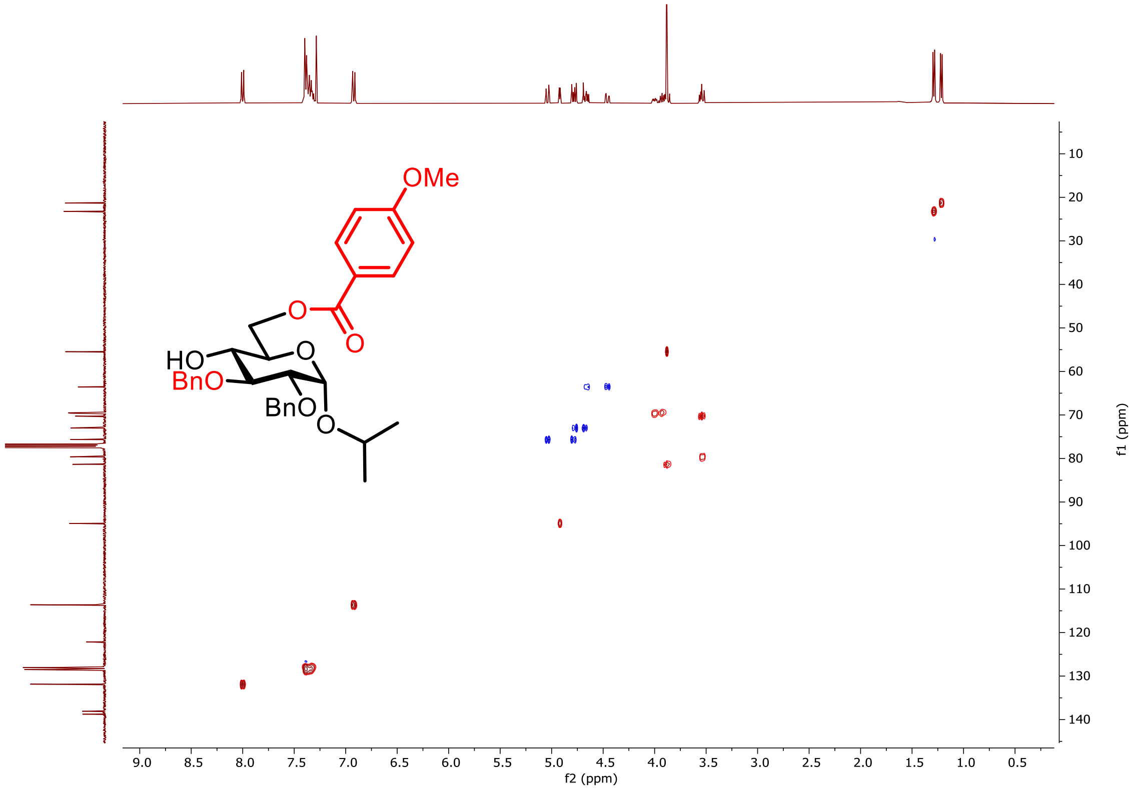

4.12 Isopropyl 2,3-di-*O*-benzyl-6-*O*-(4-nitrobenzoyl)-D-glucopyranoside, 85

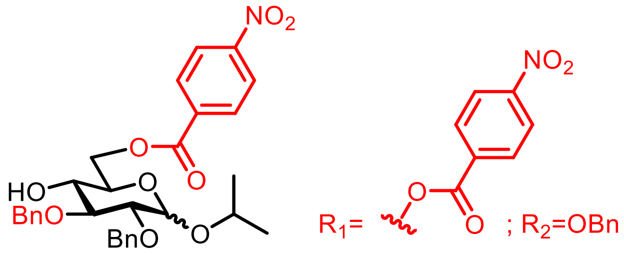

Total yield: 89% (49.1 mg). Ratio of anomer α : β = 3.5 : 1.

Spectrum data for isopropyl 2,3-di-*O*-benzyl-6-*O*-(4-nitrobenzoyl)-α-D-glucopyranoside **85a**: ^{1}H NMR (400 MHz, $CDCl_3$) δ 8.33 – 8.26 (m, 2H), 8.25 – 8.17 (m, 2H), 7.43 – 7.28 (m, 10H), 5.08 (d, *J* = 11.3 Hz, 1H), 4.93 (d, *J* = 3.7 Hz, 1H), 4.78 (d, *J* = 7.6 Hz, 1H), 4.75 (d, *J* = 6.9 Hz, 1H), 4.69 (d, *J* = 11.9 Hz, 1H), 4.66 – 4.59 (m, 2H), 4.05 (ddd, *J* = 10.1, 4.8, 2.7 Hz, 1H), 3.98 – 3.83 (m, 2H), 3.59 – 3.52 (m, 2H), 2.44 (br. s, 1H), 1.29 (d, *J* = 6.3 Hz, 3H), 1.23 (d, *J* = 6.1 Hz, 3H); ^{13}C NMR (101 MHz, $CDCl_3$) δ 164.76, 150.60, 138.62, 137.95, 135.27, 130.83, 128.72, 128.57, 128.15, 128.08, 128.06, 123.58, 95.03, 81.33, 79.63, 75.65, 72.92, 70.19, 69.85, 69.15, 64.79, 23.24, 21.38; $[\alpha]_D^{25}$ 41.82 (c = 1, $CHCl_3$); IR (neat) ν_{max} = 2923, 1727, 1528, 1275, 719 cm^{-1}; m/z ($HRMS^+$) $[M + Na]^+$ 574.2063 ($C_{30}H_{33}NO_9Na^+$ requires 574.2048).

^{1}H NMR of crude 85 (600 MHz, $CDCl_3$)

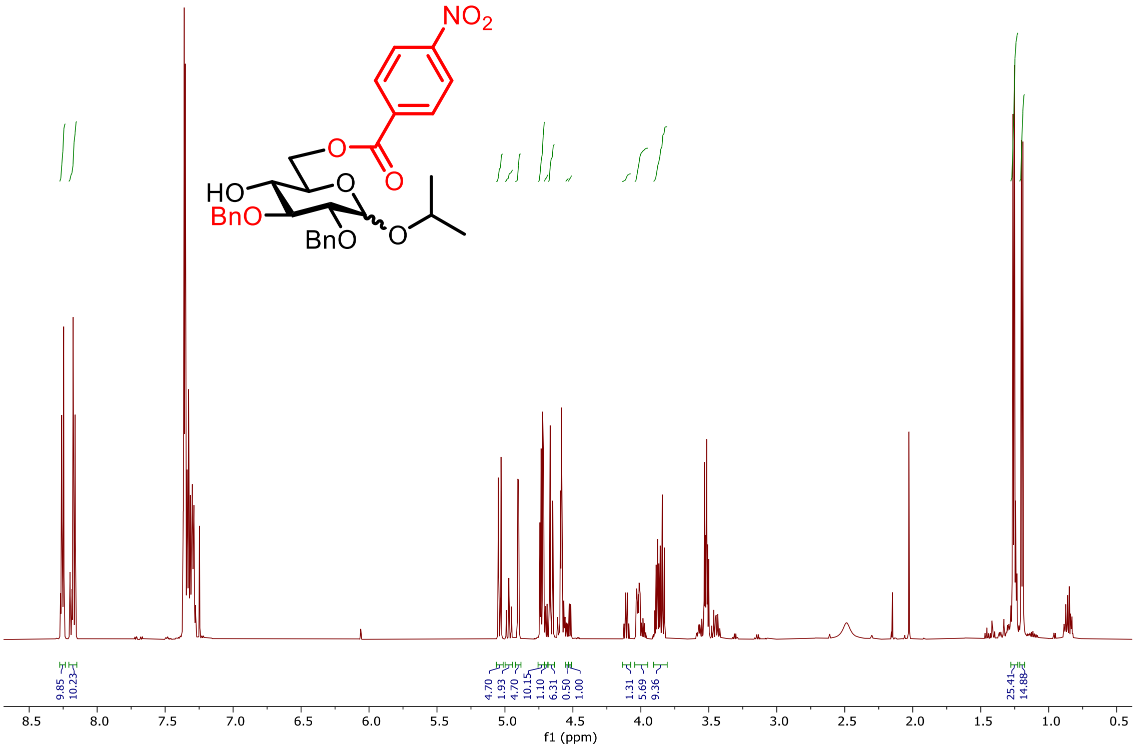

^{13}C NMR of crude 85 (151 MHz, $CDCl_3$)

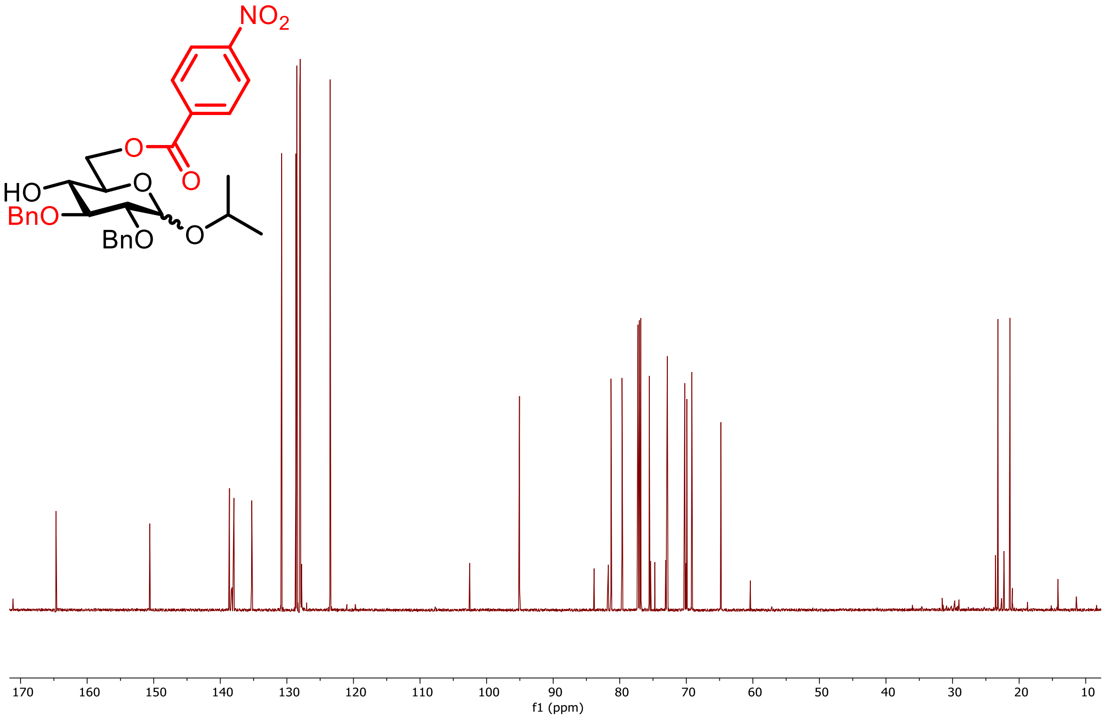

HSQC NMR of crude 85 ($CDCl_3$)

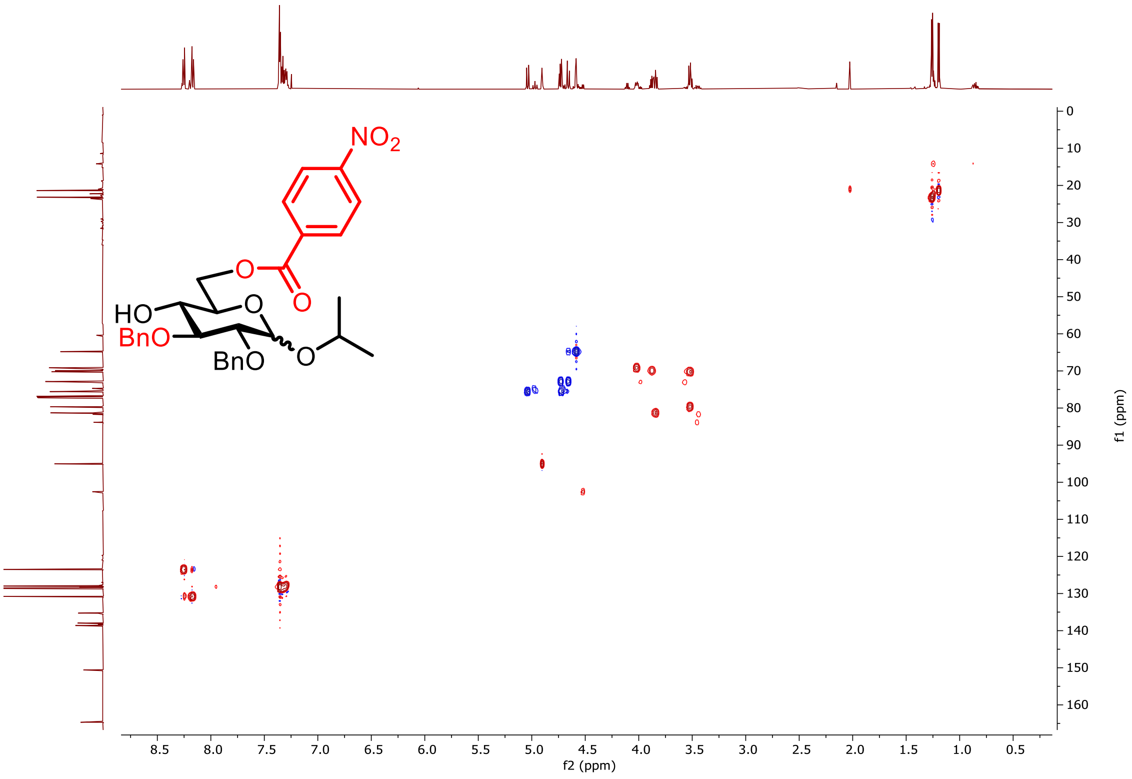

Coupled HSQC NMR of crude 85 ($CDCl_3$)

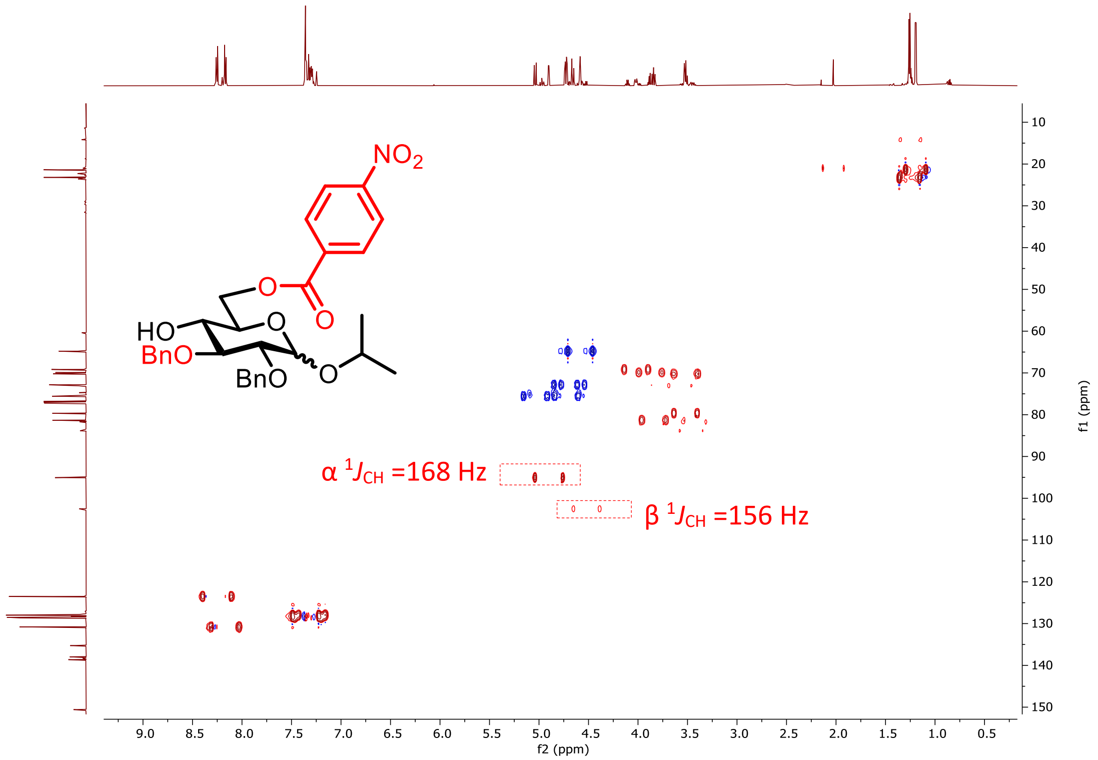

^{1}H NMR of 85a (400 MHz, CDCl$_3$)

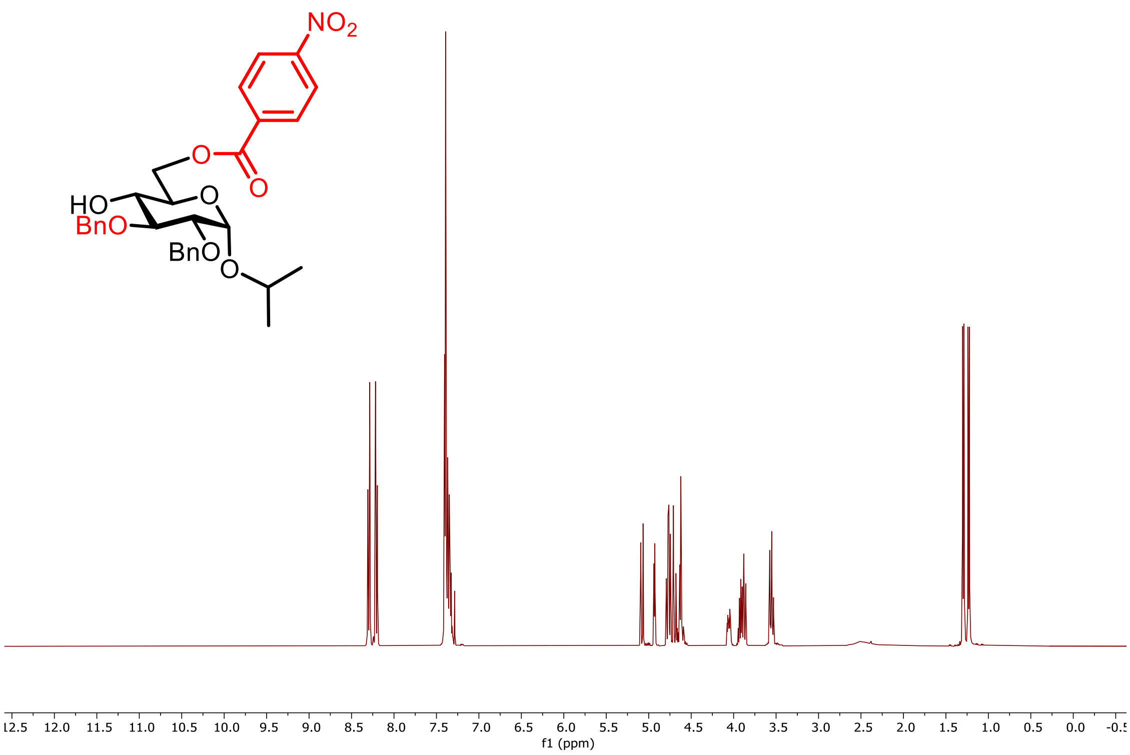

^{13}C NMR of 85a (101 MHz, CDCl$_3$)

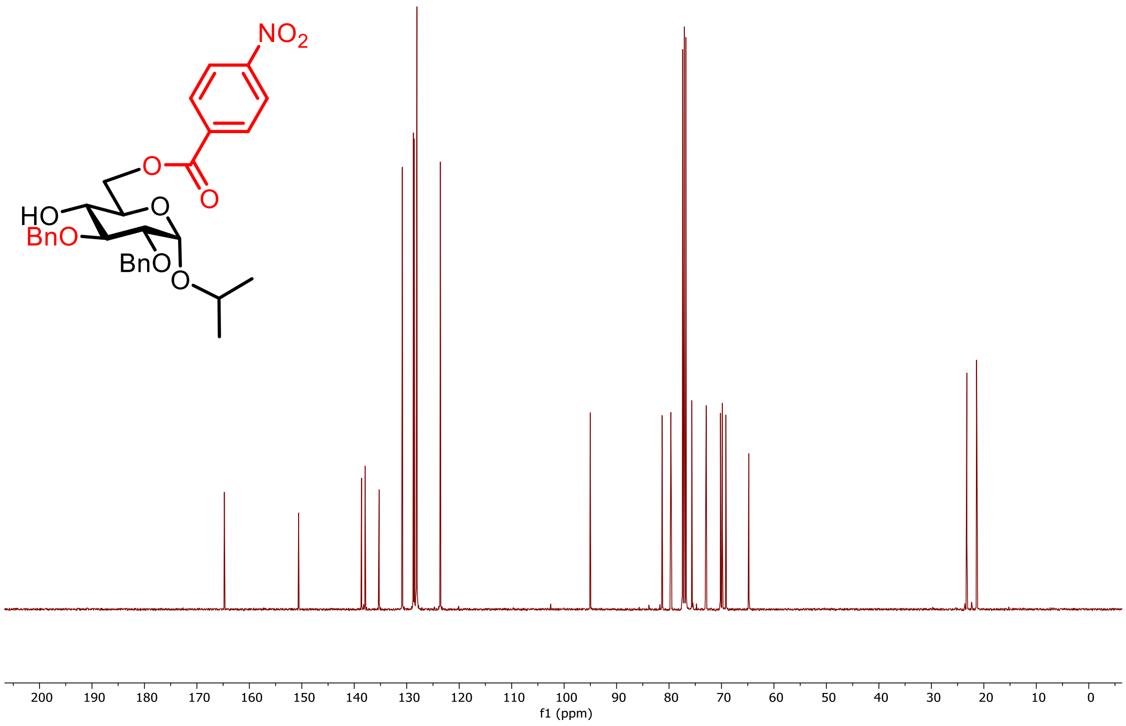

COSY NMR of 85a ($CDCl_3$)

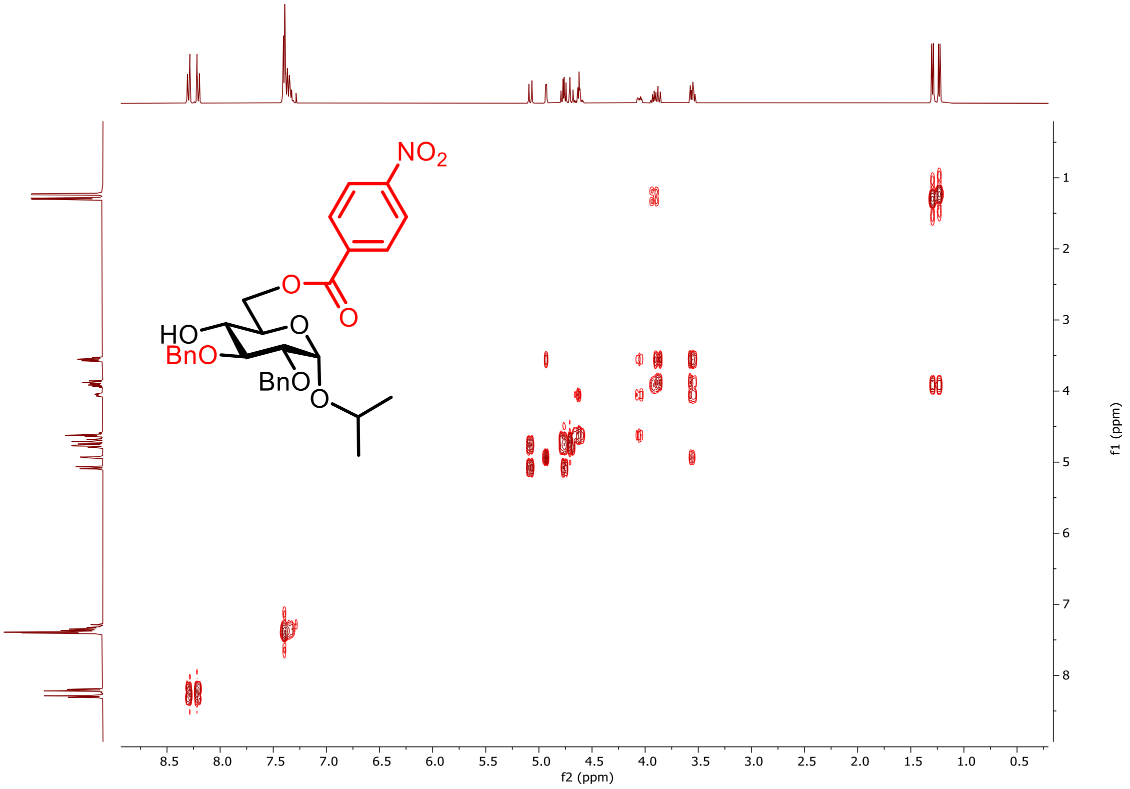

HSQC NMR of 85a ($CDCl_3$)

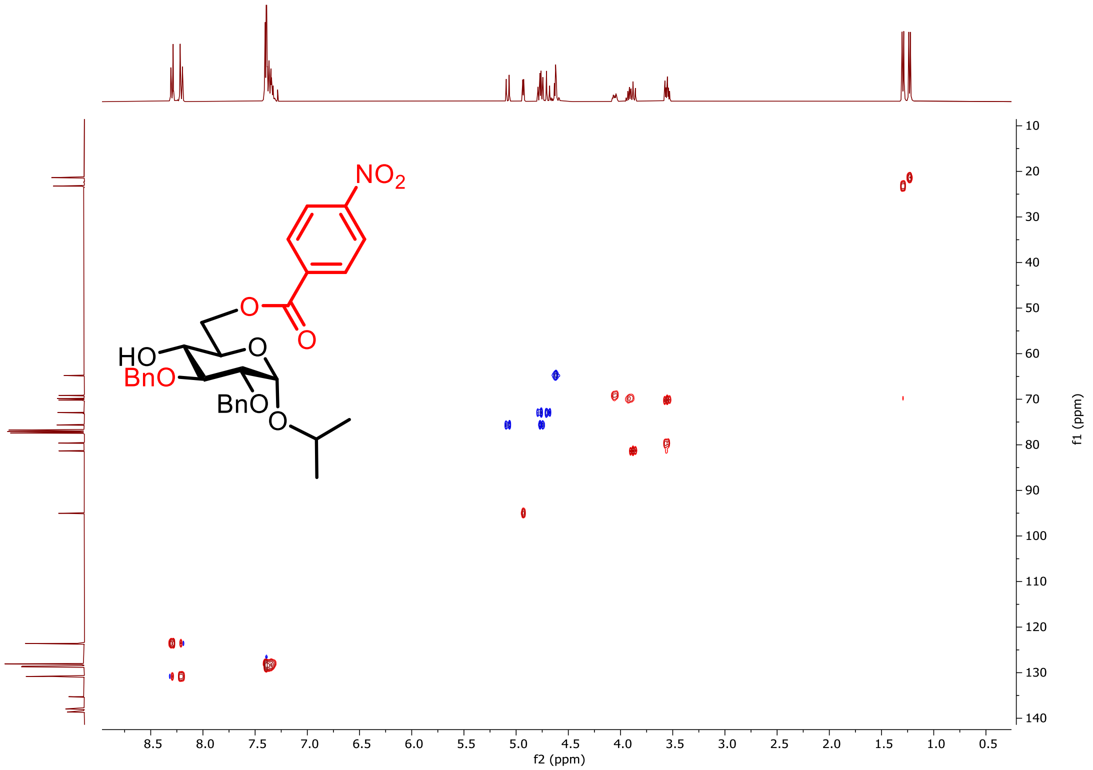

4.13 Isopropyl 2,3-di-*O*-benzyl-6-*O*-carboxybenzyl-D-glucopyranoside, 86

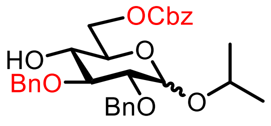

Total yield: 92% (49.4 mg). Ratio of anomer α : β = 1.3 : 1.

Spectrum data for isopropyl 2,3-di-*O*-benzyl-6-*O*-carboxybenzyl-α-D-glucopyranoside **86a**: ^{1}H NMR (400 MHz, $CDCl_3$) δ 7.43 – 7.30 (m, 15H), 5.17 (s, 2H), 5.05 (d, *J* = 11.4 Hz, 1H), 4.88 (d, *J* = 3.6 Hz, 1H), 4.76 (d, *J* = 8.2 Hz, 1H), 4.73 (d, *J* = 7.6 Hz, 1H), 4.66 (d, *J* = 11.9 Hz, 1H), 4.45 (dd, *J* = 11.7, 4.8 Hz, 1H), 4.34 (dd, *J* = 11.8, 2.3 Hz, 1H), 3.94 – 3.78 (m, 3H), 3.56 – 3.46 (m, 2H), 2.44 – 2.40 (br. s, 1H), 1.24 (d, *J* = 6.3 Hz, 3H), 1.19 (d, *J* = 6.1 Hz, 3H); ^{13}C NMR (101 MHz, $CDCl_3$) δ 155.44, 138.83, 138.00, 135.11, 128.62, 128.57, 128.51, 128.41, 128.18, 128.00, 127.89, 127.87, 94.83, 81.32, 79.48, 75.43, 72.93, 69.89, 69.80, 69.45, 69.08, 66.76, 23.16, 21.22; $[\alpha]_D^{25}$ 22.23 (c = 1, $CHCl_3$); IR (neat) ν_{max} = 2926, 1749, 1266, 1060, 698 cm^{-1}; m/z (HRMS$^+$) $[M + Na]^+$ 559.2314 ($C_{31}H_{36}O_8Na^+$ requires 559.2302).

^{1}H NMR of crude 86 (600 MHz, $CDCl_3$)

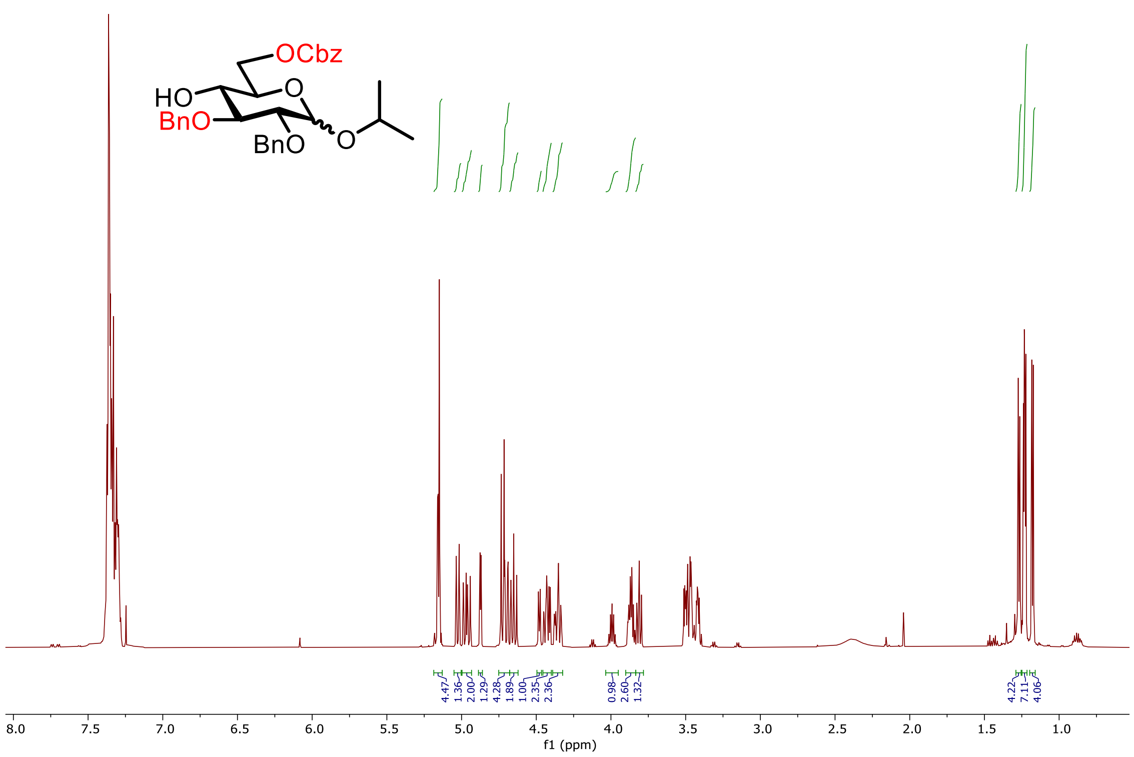

^{13}C NMR of crude 86 (151 MHz, $CDCl_3$)

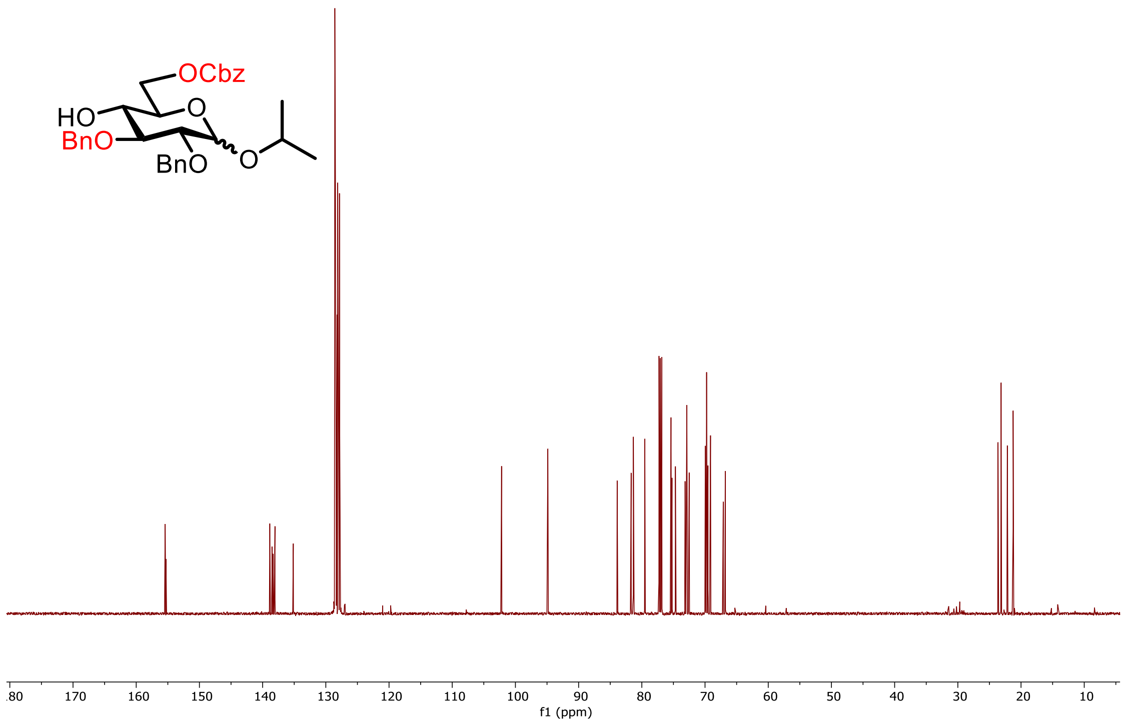

HSQC NMR of crude 86 ($CDCl_3$)

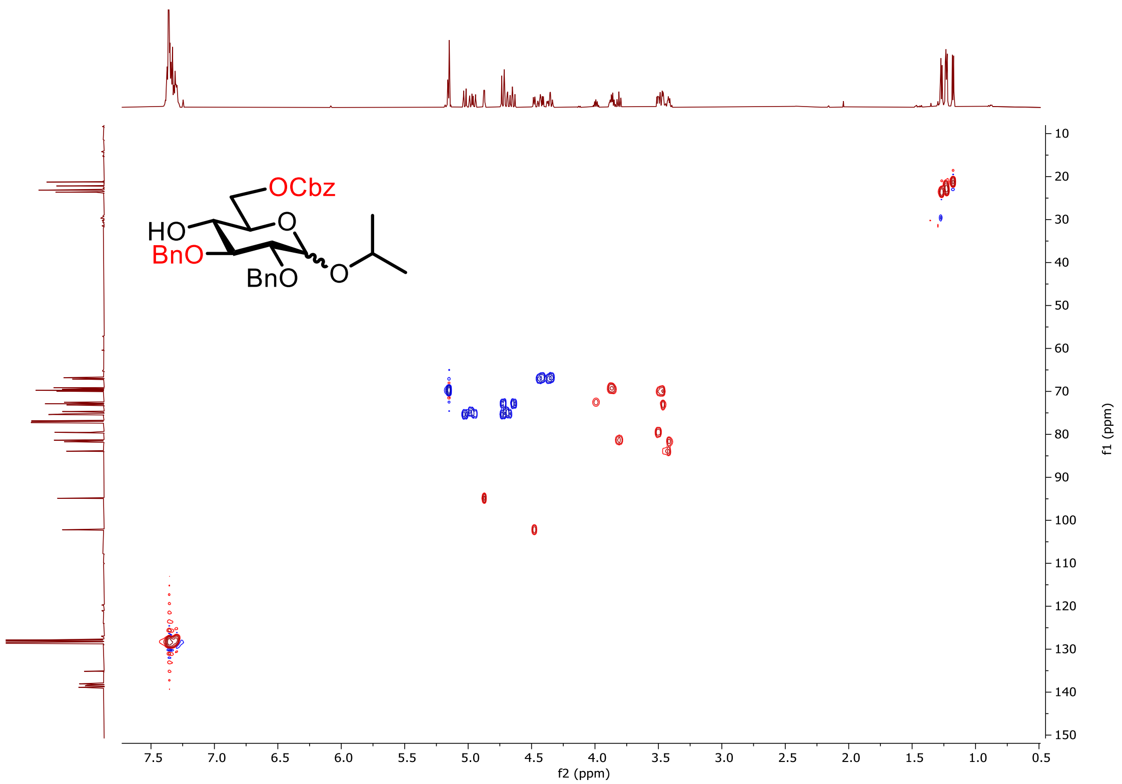

Coupled HSQC NMR of crude 86 ($CDCl_3$)

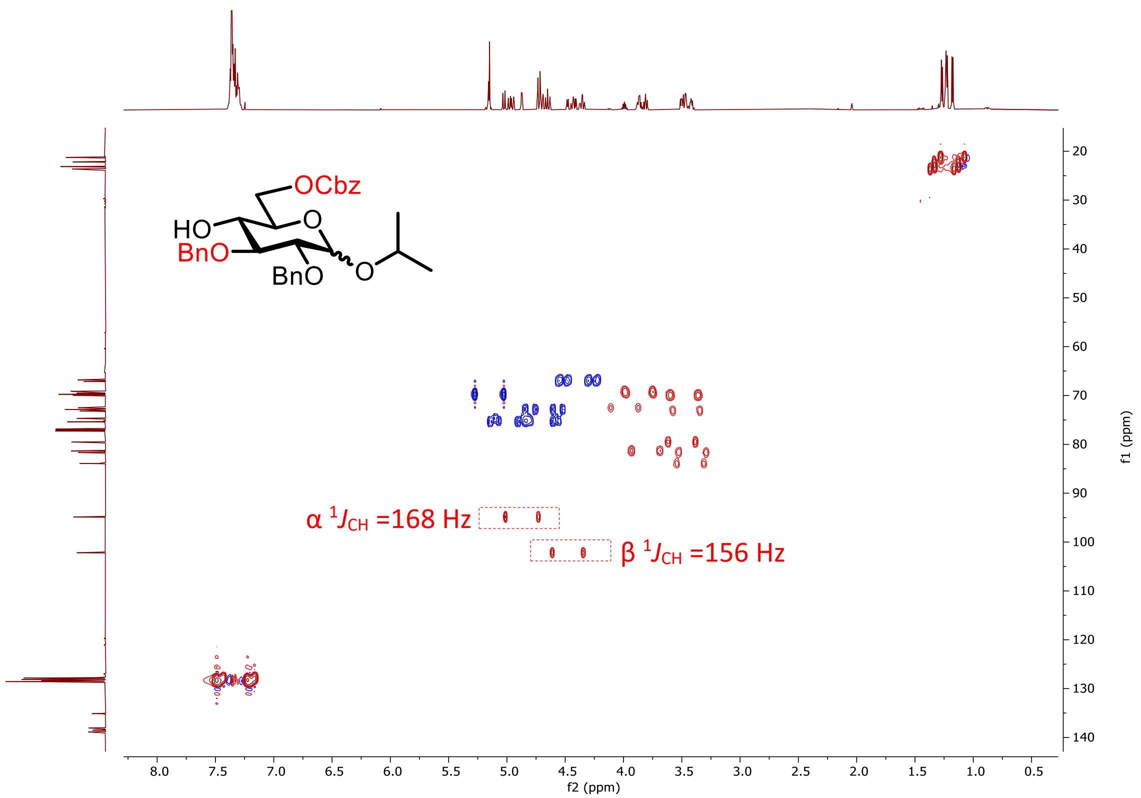

^{1}H NMR of 86a (400 MHz, $CDCl_3$)

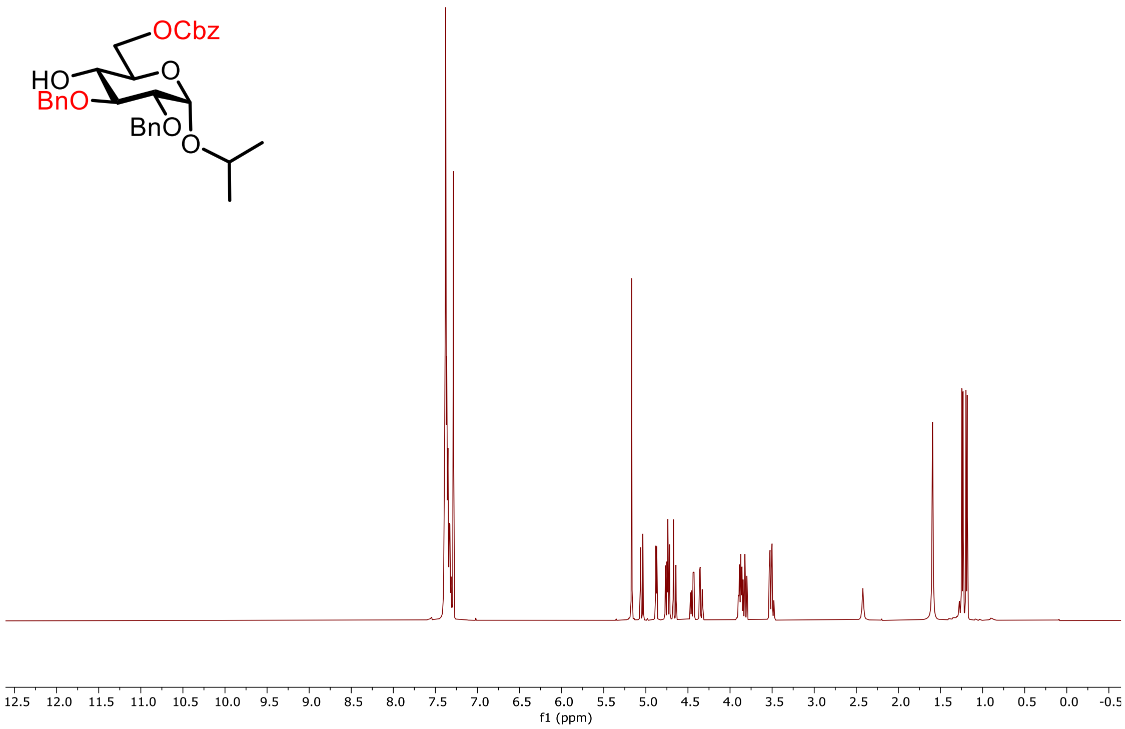

^{13}C NMR of 86a (101 MHz, $CDCl_3$)

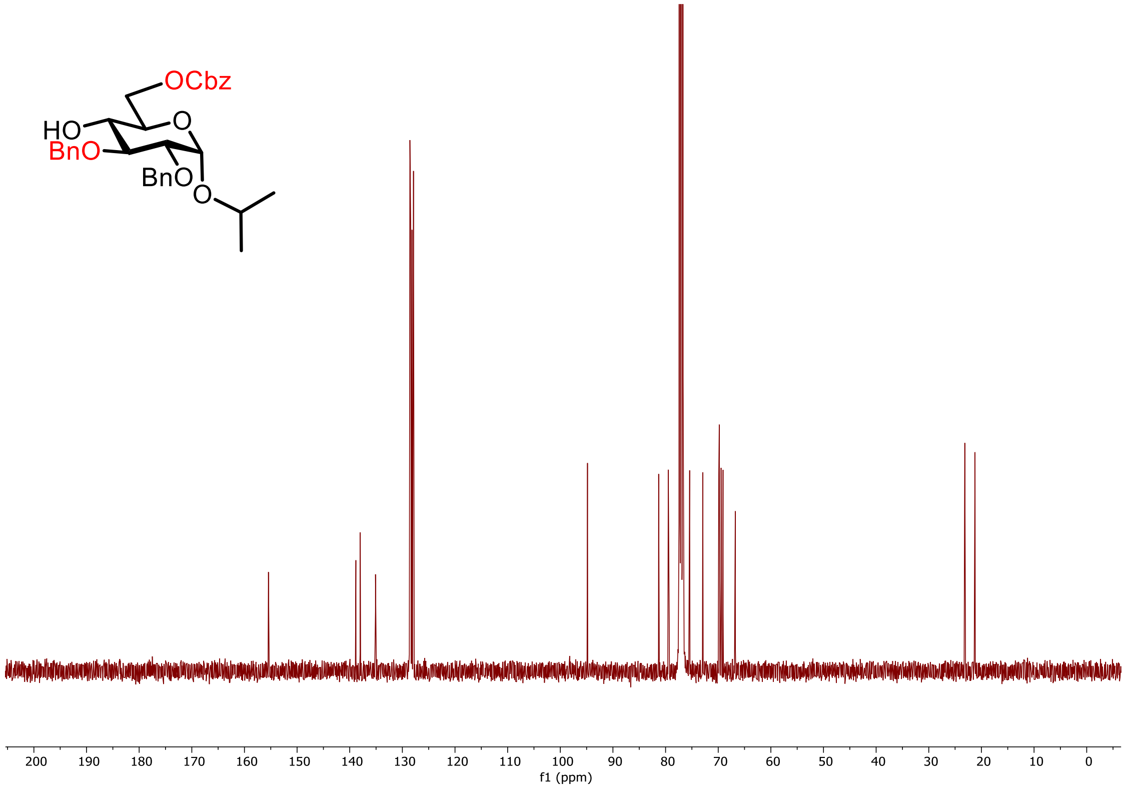

COSY NMR of 86a ($CDCl_3$)

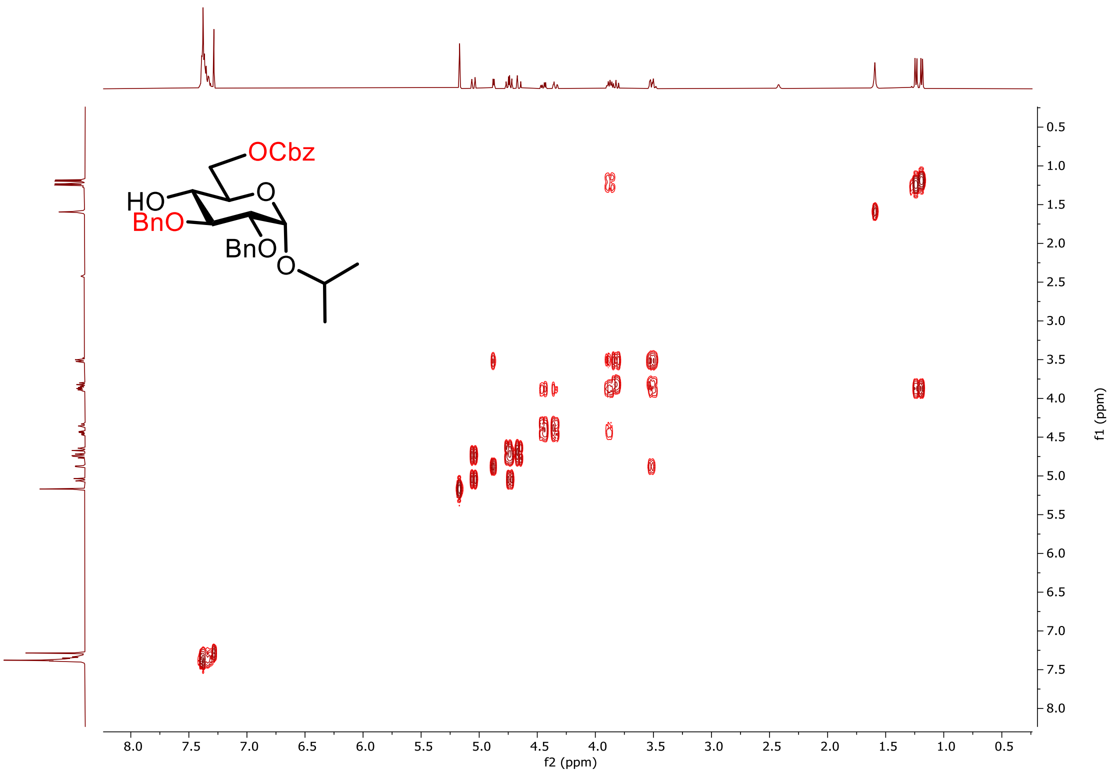

HSQC NMR of 86a ($CDCl_3$)

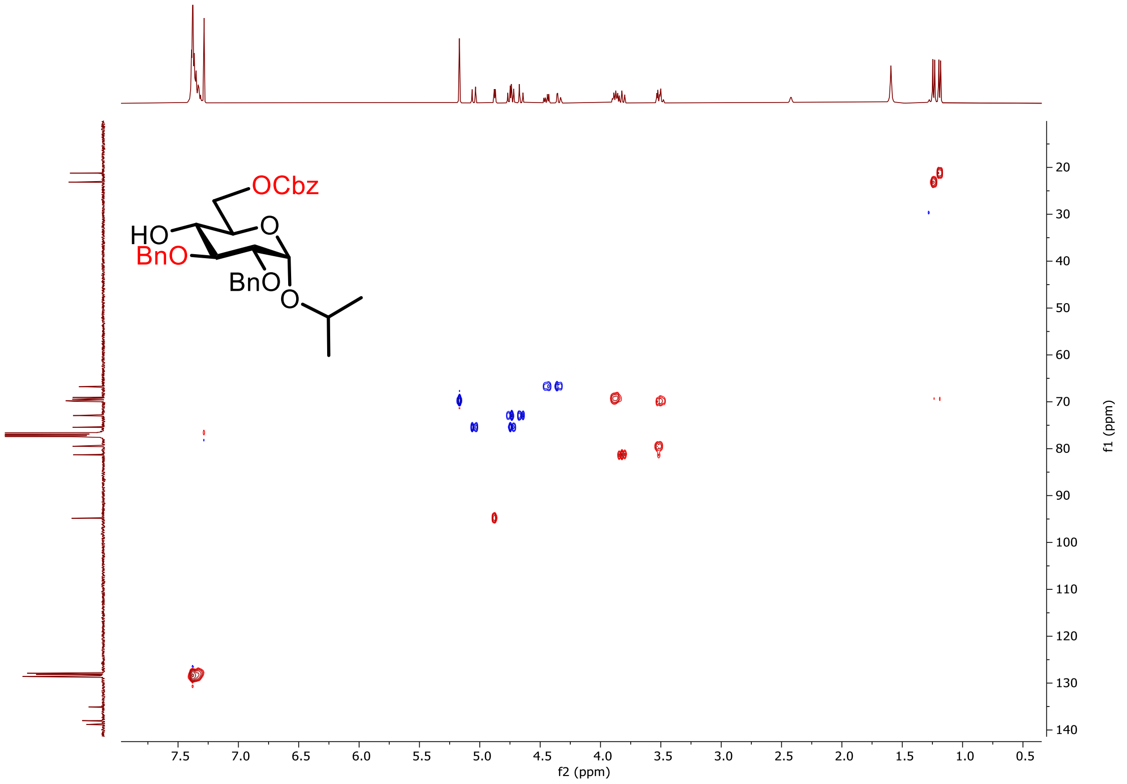

4.14 Isopropyl 2,3-di-*O*-benzyl-6-*O*-triphenylacetyl-D-glucopyranoside, 87

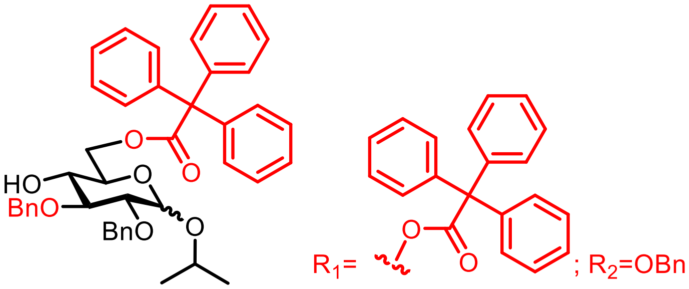

Total yield: 90% (60.5 mg). Ratio of anomer α : β = 1.9 : 1.

Spectrum data for isopropyl 2,3-di-*O*-benzyl-6-*O*-triphenylacetyl-α-D-glucopyranoside **87a**: ^{1}H NMR (400 MHz, $CDCl_3$) δ 7.44 – 7.30 (m, 10H), 7.30 – 7.23 (m, 9H), 7.23 – 7.15 (m, 6H), 5.01 (d, *J* = 11.5 Hz, 1H), 4.80 (d, *J* = 3.6 Hz, 1H), 4.77 – 4.63 (m, 3H), 4.45 (dd, *J* = 11.9, 2.4 Hz, 1H), 4.39 (dd, *J* = 11.9, 5.4 Hz, 1H), 3.83 – 3.71 (m, 2H), 3.65 (p, *J* = 6.2 Hz, 1H), 3.34 (dd, *J* = 9.6, 3.7 Hz, 1H), 3.12 (appt, *J* = 9.4 Hz, 1H), 2.23 (br. s, 1H), 1.11 (d, *J* = 6.0 Hz, 3H), 1.06 (d, *J* = 6.2 Hz, 3H); ^{13}C NMR (101 MHz, $CDCl_3$) δ 173.82, 142.81, 138.91, 138.07, 130.31, 128.60, 128.53, 128.14, 128.01, 127.83, 127.81, 127.69, 126.89, 94.18, 81.21, 79.19, 75.26, 72.73, 70.24, 68.97, 68.53, 67.58, 64.66, 23.06, 20.86; $[\alpha]_D^{25}$ 18.46 (c = 0.5, $CHCl_3$); IR (neat) ν_{max} = 2923, 1733, 1059, 699 cm^{-1}; m/z (HRMS$^+$) $[M + Na]^+$ 695.3045 ($C_{43}H_{44}O_7Na^+$ requires 695.2979).

^{1}H NMR of crude 87 (600 MHz, $CDCl_3$)

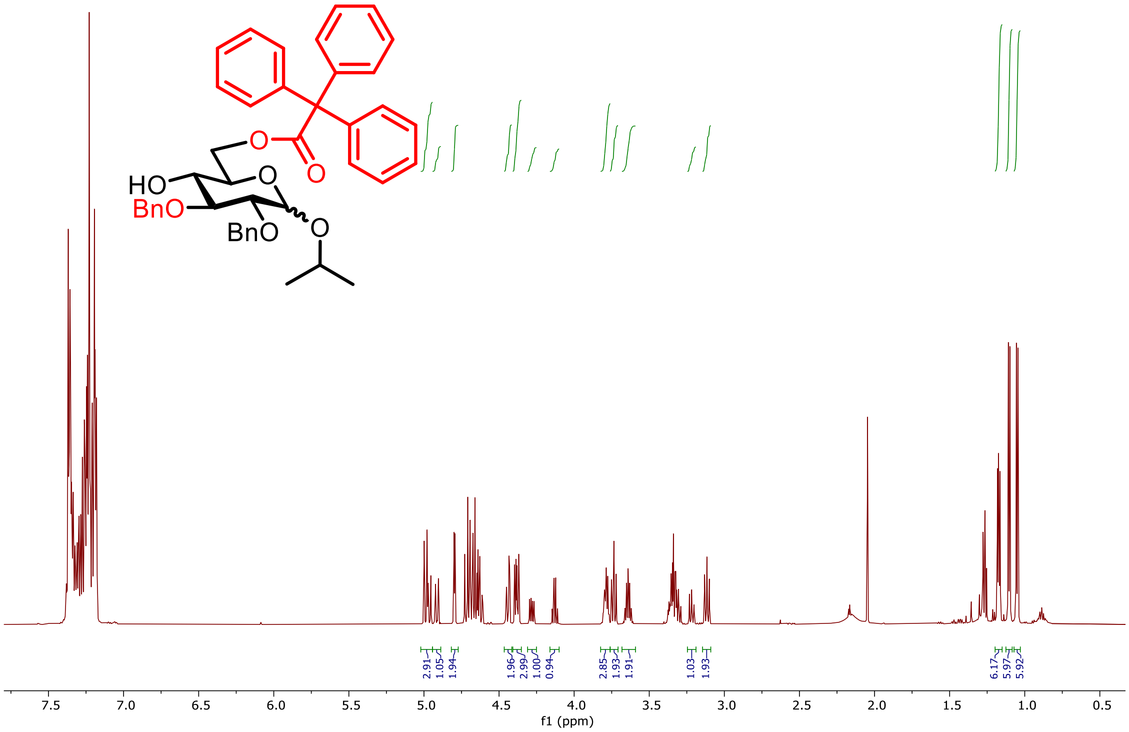

^{13}C NMR of crude 87 (151 MHz, $CDCl_3$)

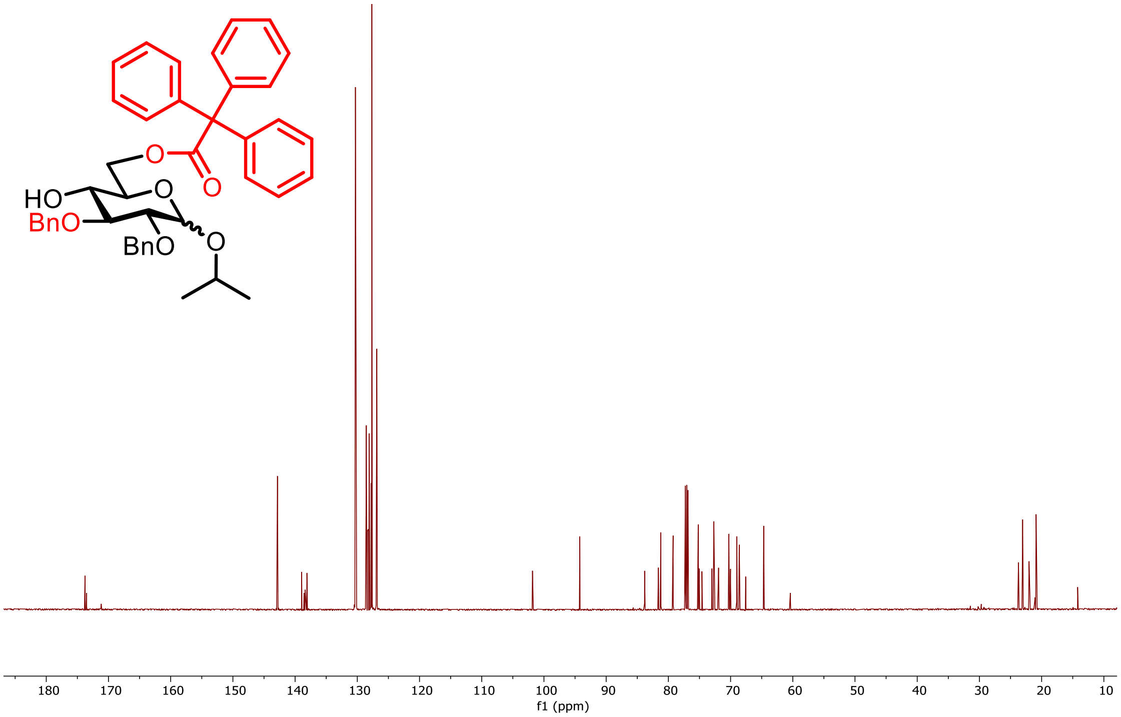

HSQC NMR of crude 87 ($CDCl_3$)

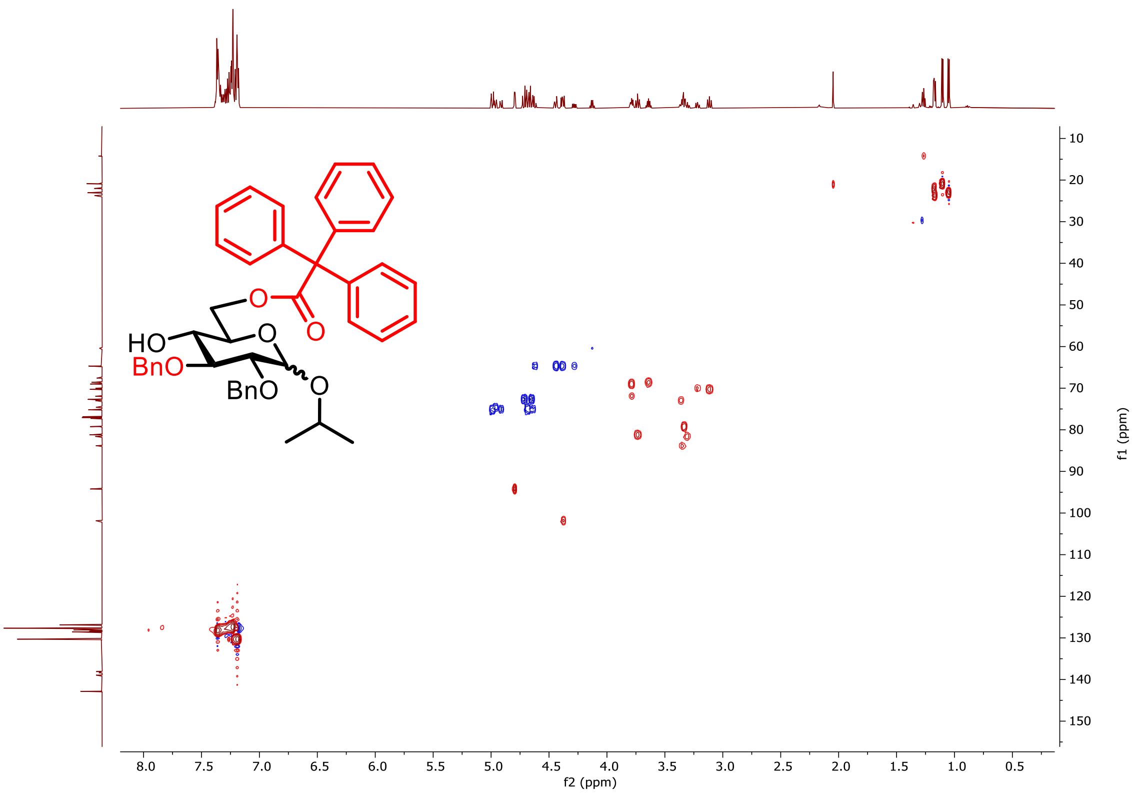

Coupled HSQC NMR of crude 87 ($CDCl_3$)

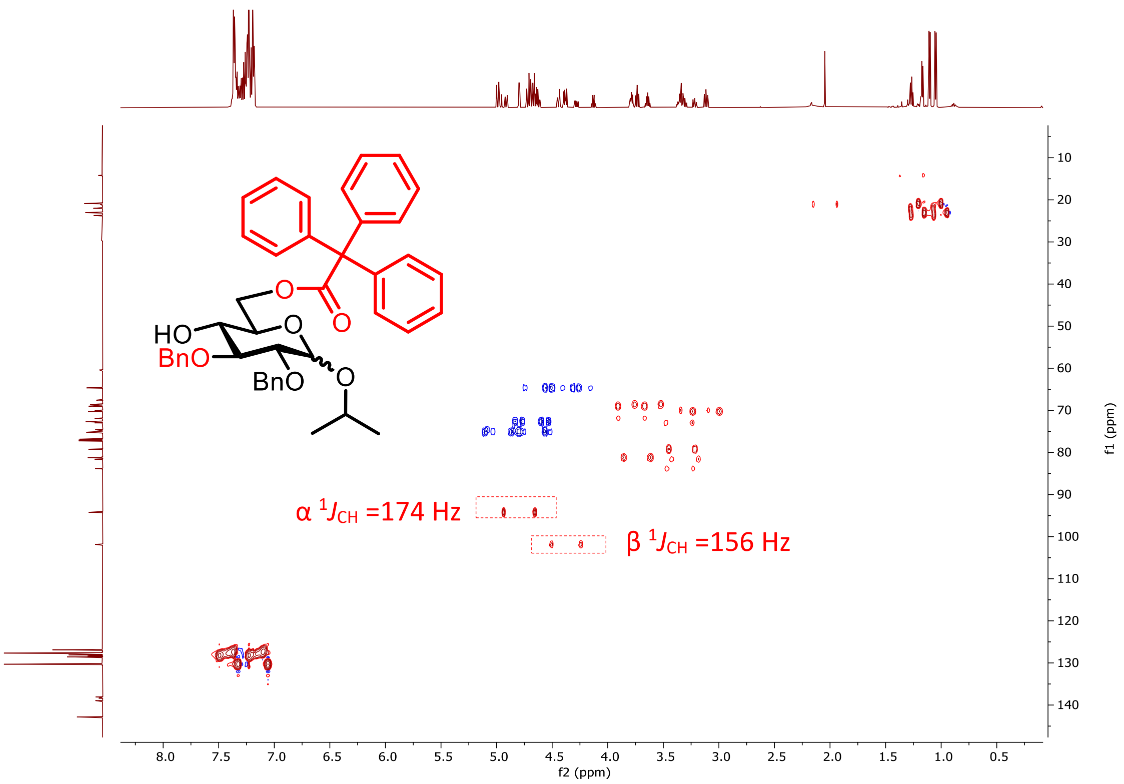

^{1}H NMR of 87a (400 MHz, $CDCl_3$)

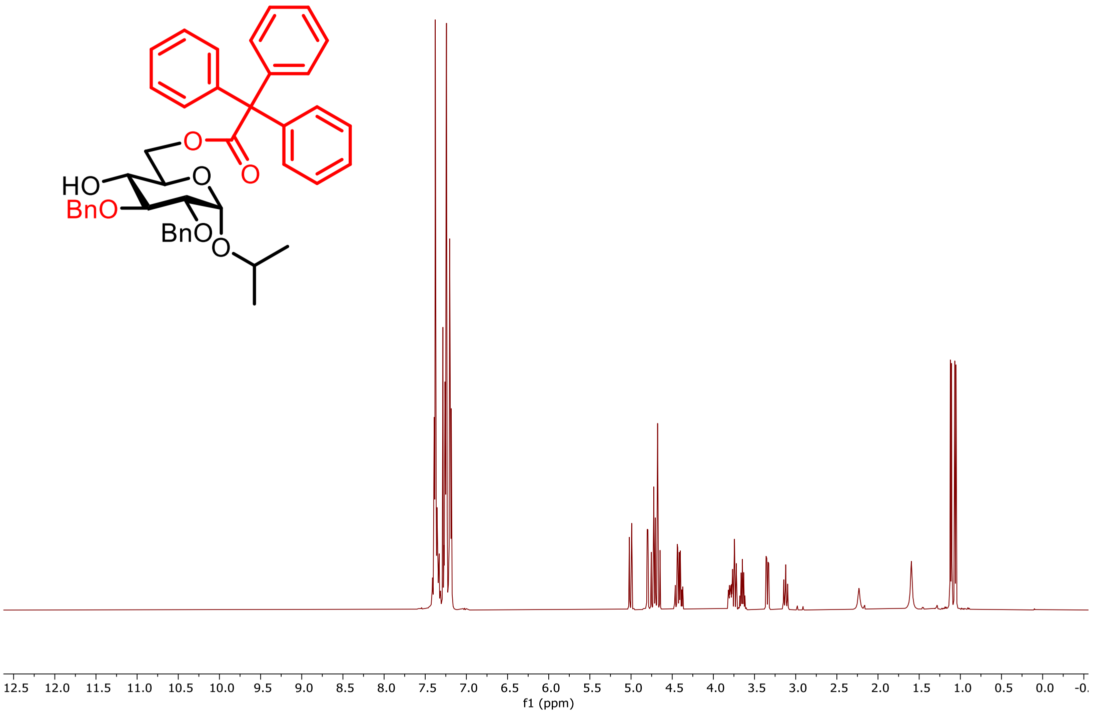

^{13}C NMR of 87a (101 MHz, $CDCl_3$)

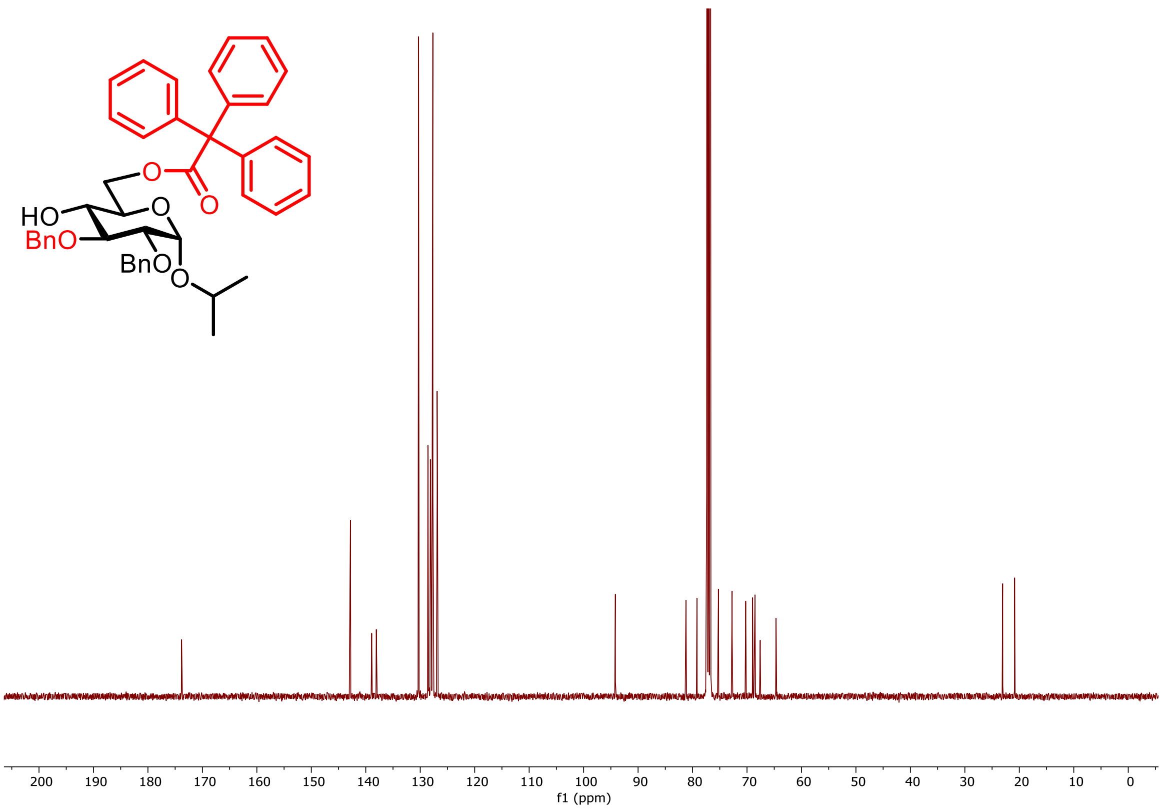

COSY NMR of 87a ($CDCl_3$)

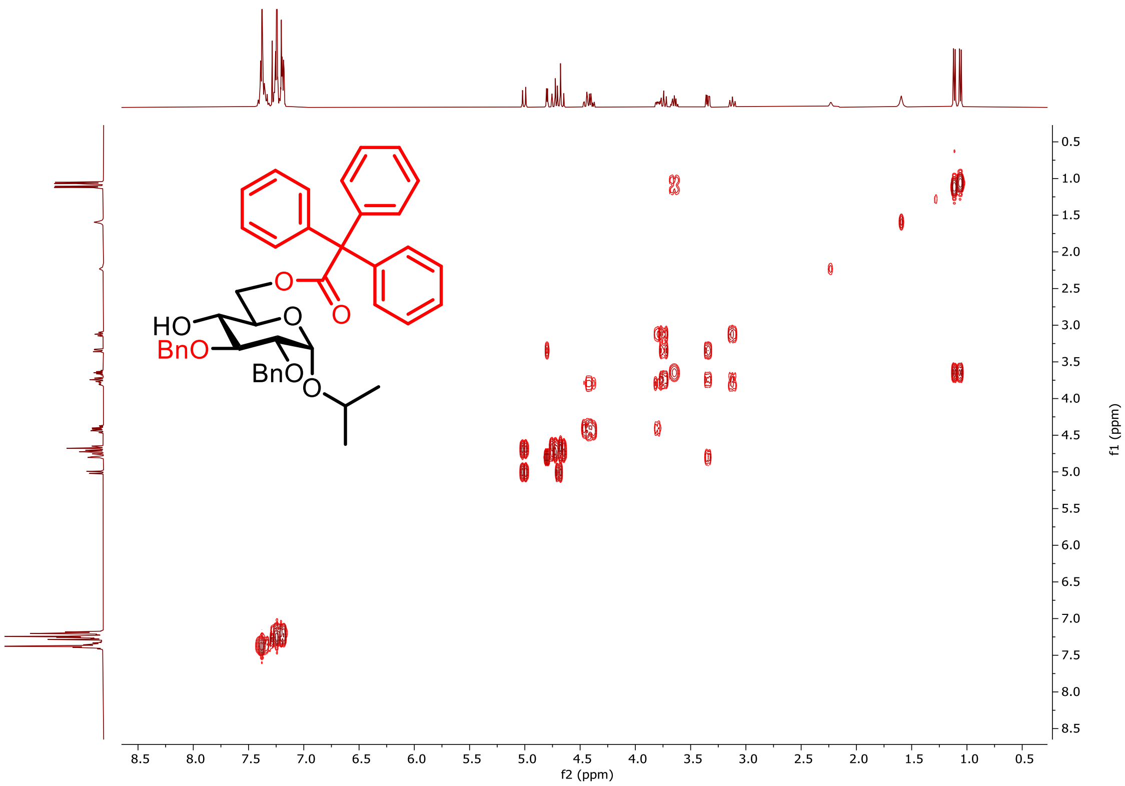

HSQC NMR of 87a ($CDCl_3$)

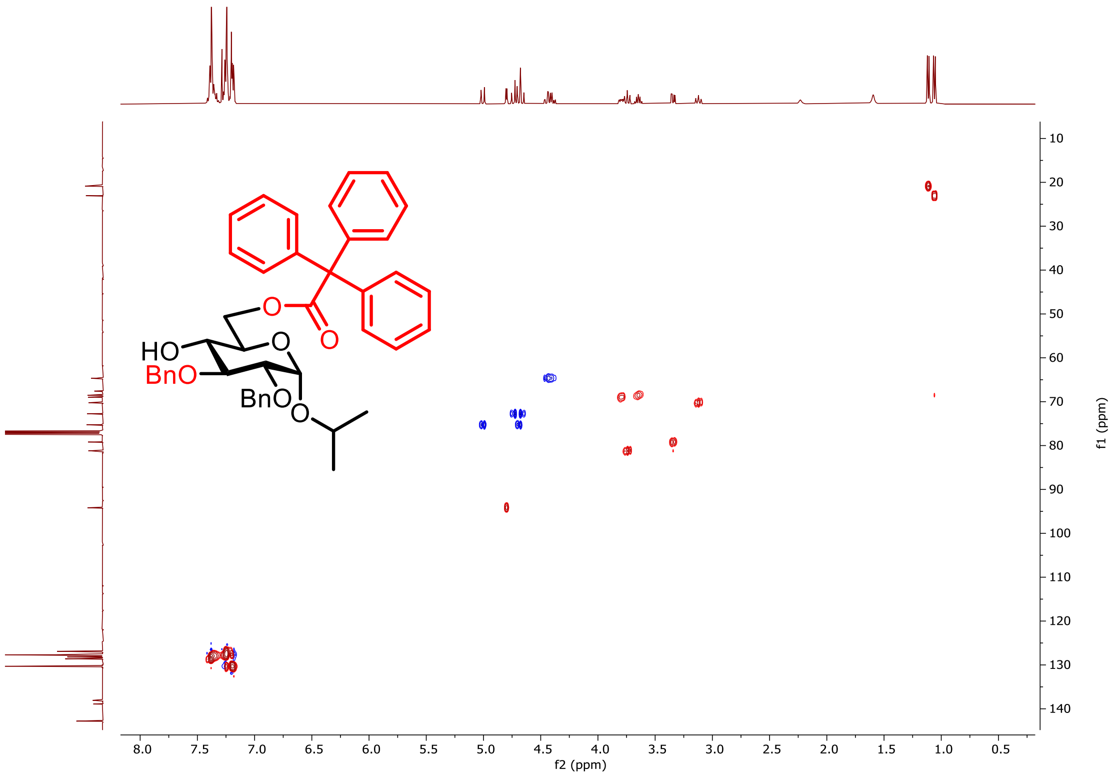

4.15 Isopropyl 2-*O*-benzyl-3,6-di-*O*-acetyl-D-glucopyranoside, 88

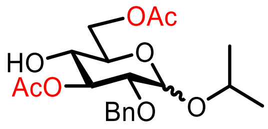

R1=R2=OAc

Total yield: 84% (33.3 mg). Ratio of anomer α : β = 4.5 : 1.

Spectrum data for isopropyl 2-*O*-benzyl-3,6-di-*O*-acetyl-α-D-glucopyranoside **88a**: ^{1}H NMR (400 MHz, $CDCl_3$) δ 7.41 – 7.31 (m, 5H), 5.25 (appt, *J* = 9.6 Hz, 1H), 4.92 (d, *J* = 3.7 Hz, 1H), 4.68 (d, *J* = 12.5 Hz, 1H), 4.62 (d, *J* = 12.3 Hz, 1H), 4.45 (dd, *J* = 12.1, 4.8 Hz, 1H), 4.27 (dd, *J* = 12.1, 2.2 Hz, 1H), 3.97 – 3.81 (m, 2H), 3.52 (dd, *J* = 9.9, 3.7 Hz, 1H), 3.47 (appt, *J* = 9.6 Hz, 1H), 3.13 – 2.92 (br. s, 1H), 2.14 (s, 3H), 2.12 (s, 3H), 1.27 (d, *J* = 6.3 Hz, 3H), 1.21 (d, *J* = 6.1 Hz, 3H); ^{13}C NMR (101 MHz, $CDCl_3$) δ 172.49, 171.38, 137.92, 128.50, 128.03, 127.91, 95.02, 76.32, 75.44, 72.76, 70.45, 69.93, 69.84, 63.17, 23.18, 21.46, 21.10, 20.89; $[\alpha]_D^{25}$ 61.44 (c = 1, $CHCl_3$); IR (neat) ν_{max} = 3464, 2975, 1741, 1229, 1027, 699 cm^{-1}; m/z (HRMS$^+$) [M + Na]$^+$ 419.1647 ($C_{20}H_{28}O_8Na^+$ requires 419.1676).

^{1}H NMR of crude 88 (600 MHz, $CDCl_3$)

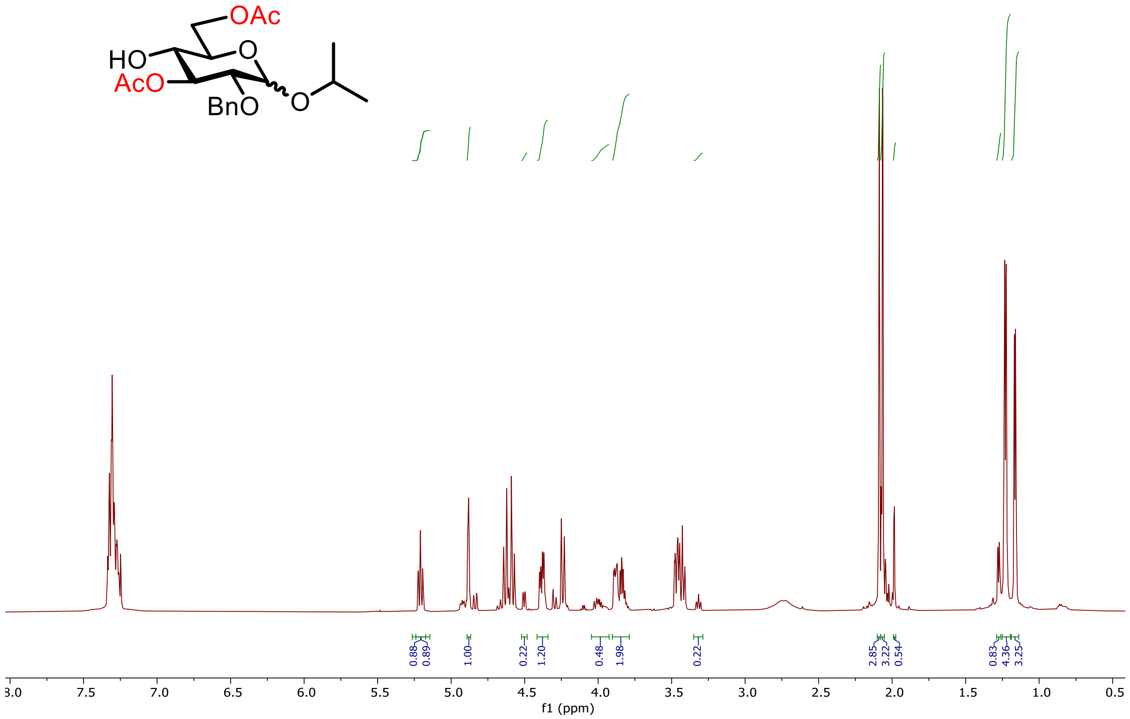

^{13}C NMR of crude 88 (151 MHz, $CDCl_3$)

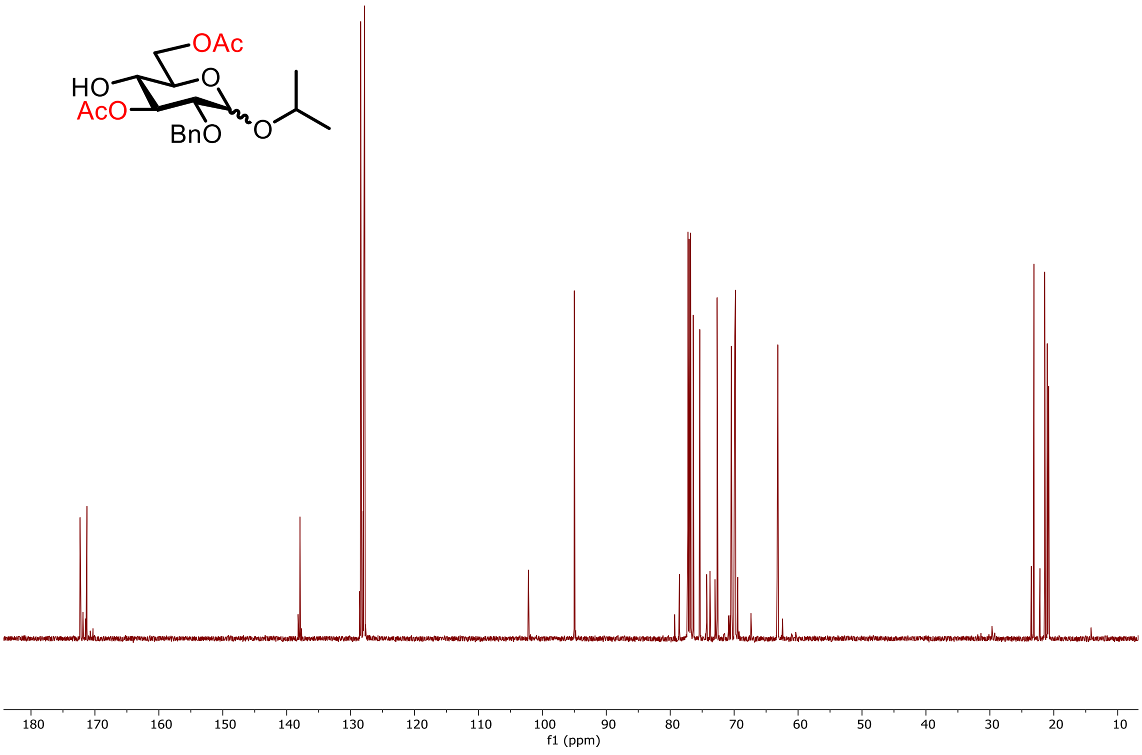

HSQC NMR of crude 88 ($CDCl_3$)

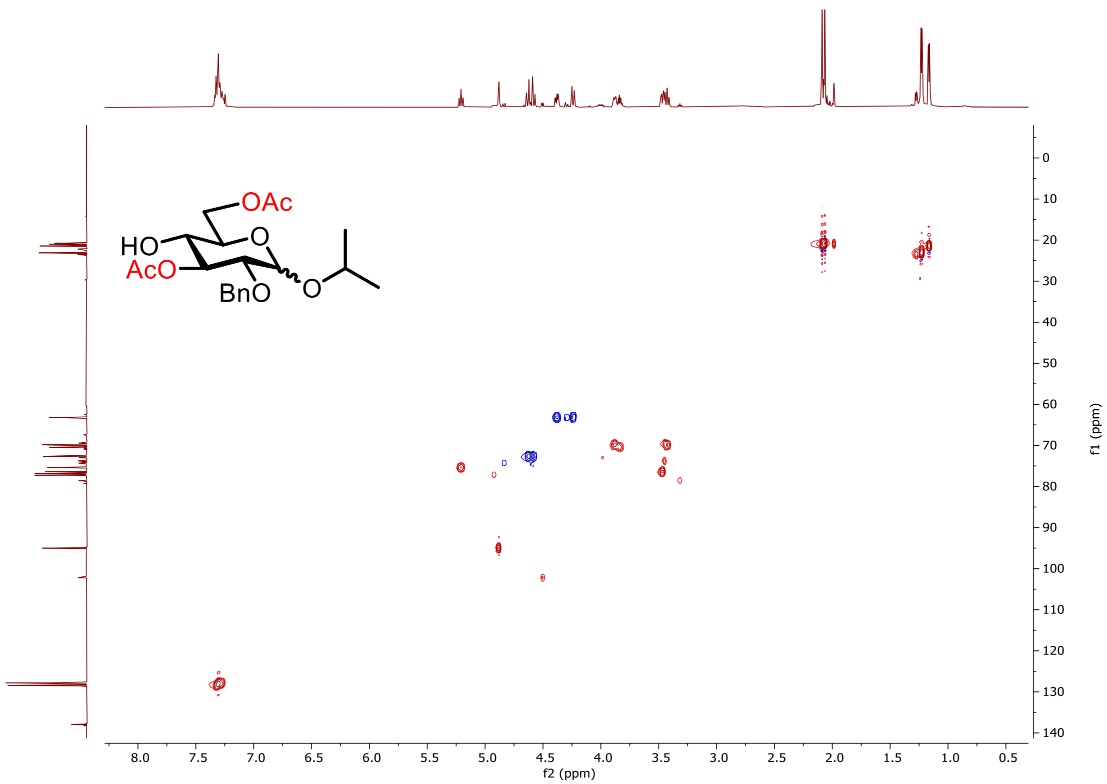

Coupled HSQC NMR of crude 88 ($CDCl_3$)

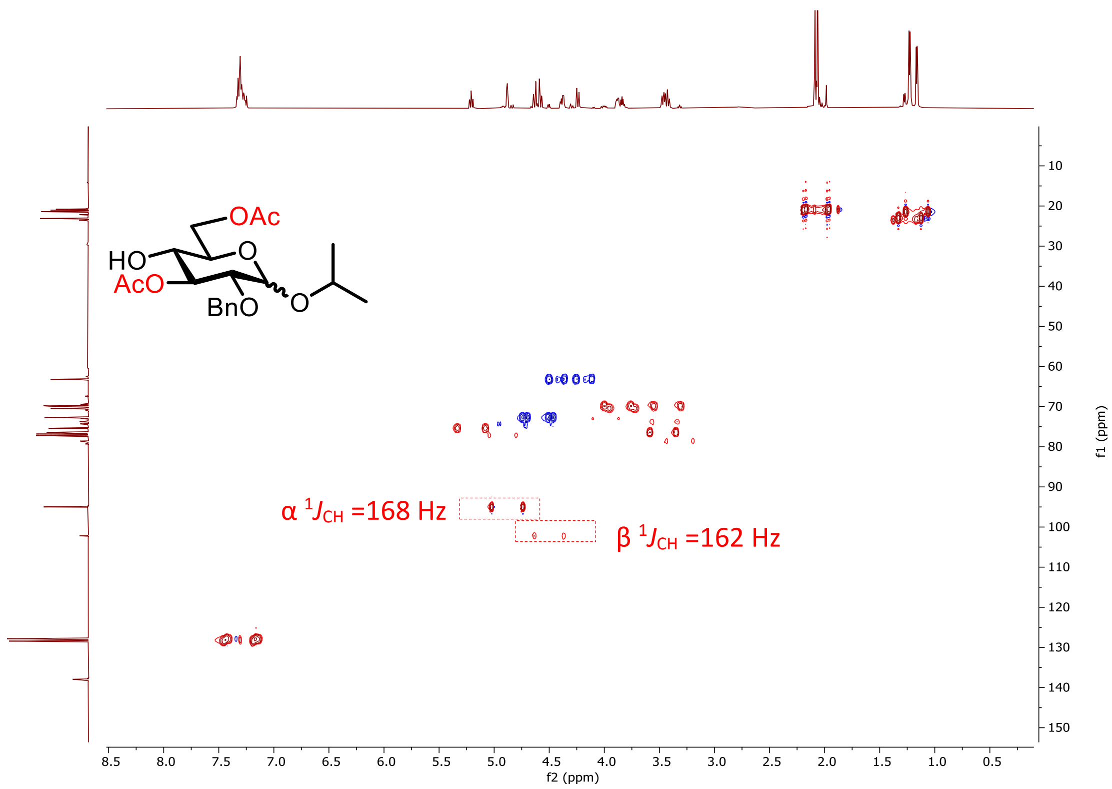

^{1}H NMR of 88a (400 MHz, $CDCl_3$)

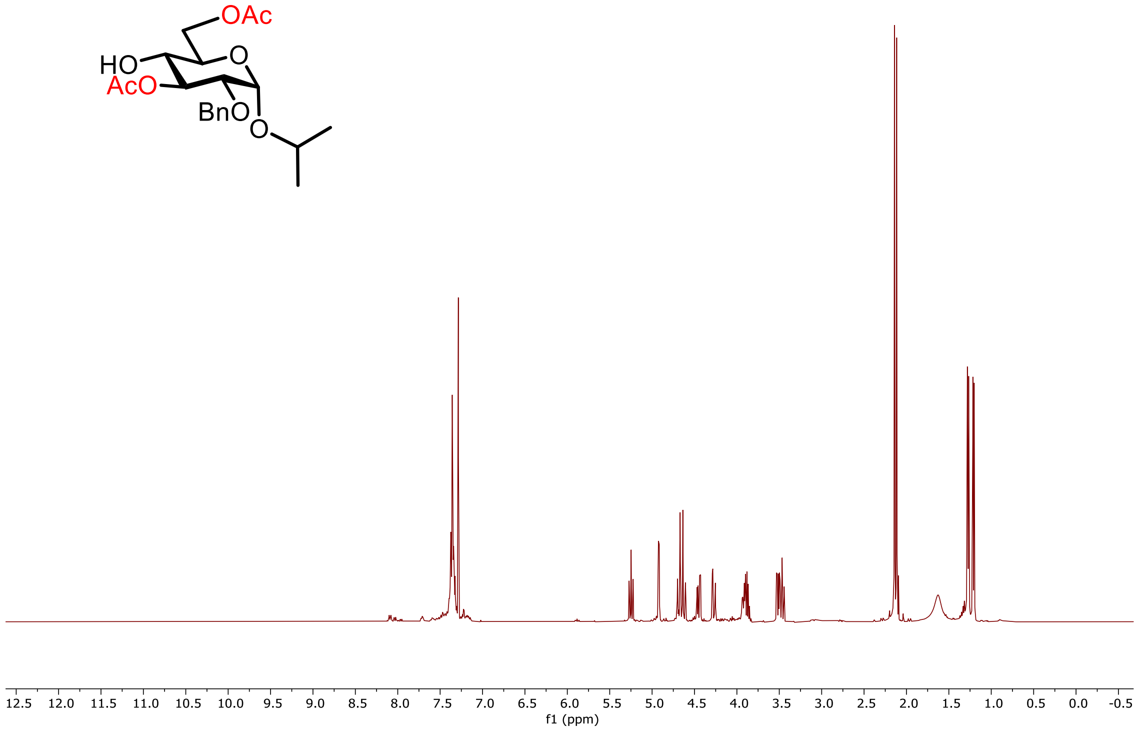

^{13}C NMR of 88a (101 MHz, $CDCl_3$)

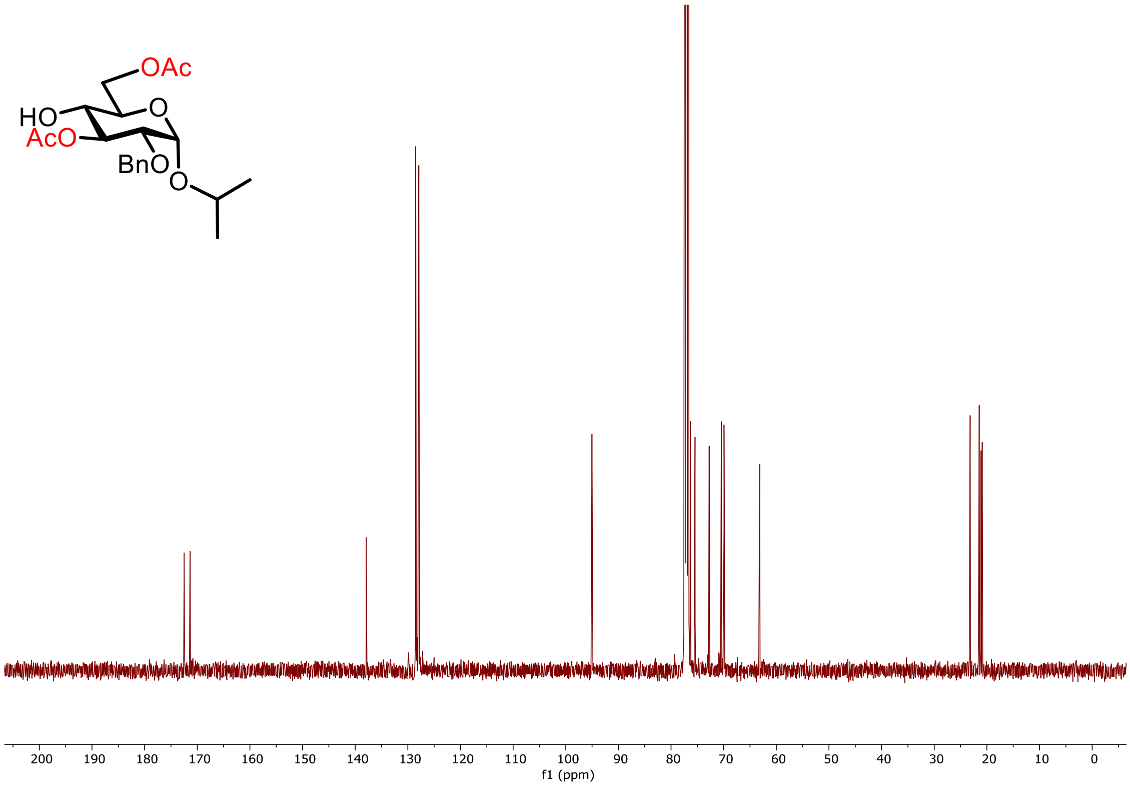

COSY NMR of 88a ($CDCl_3$)

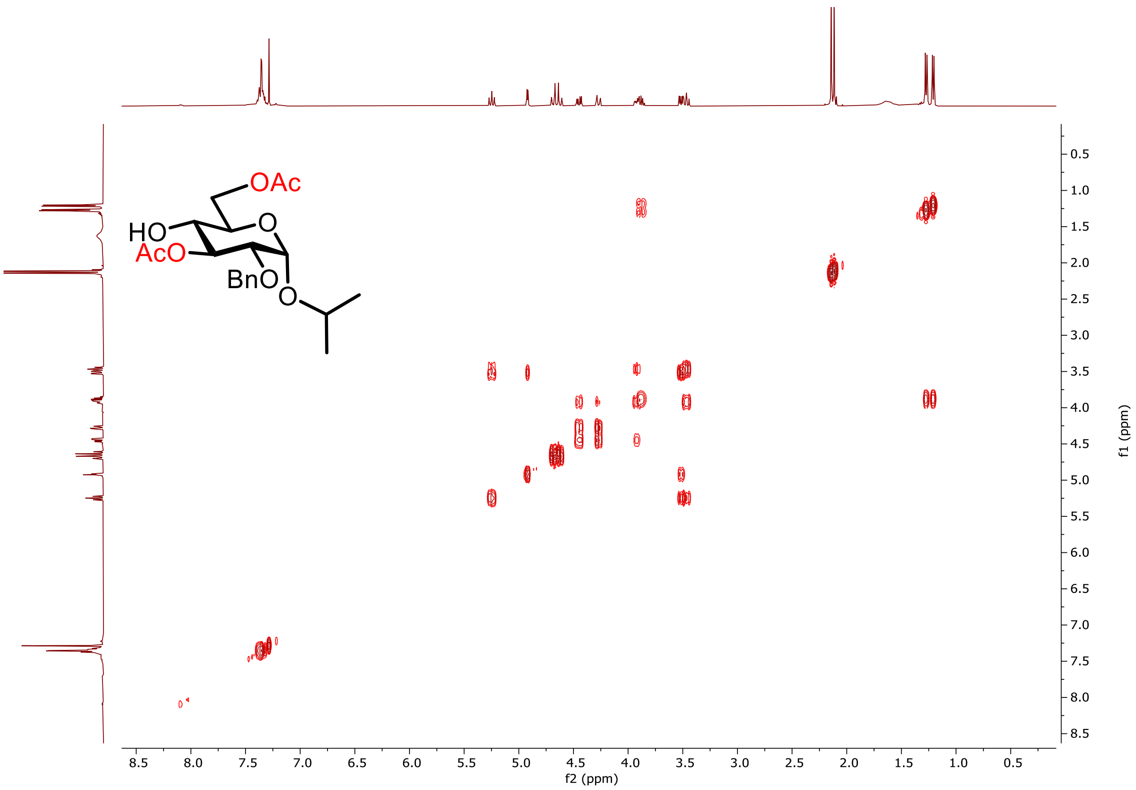

HSQC NMR of 88a ($CDCl_3$)

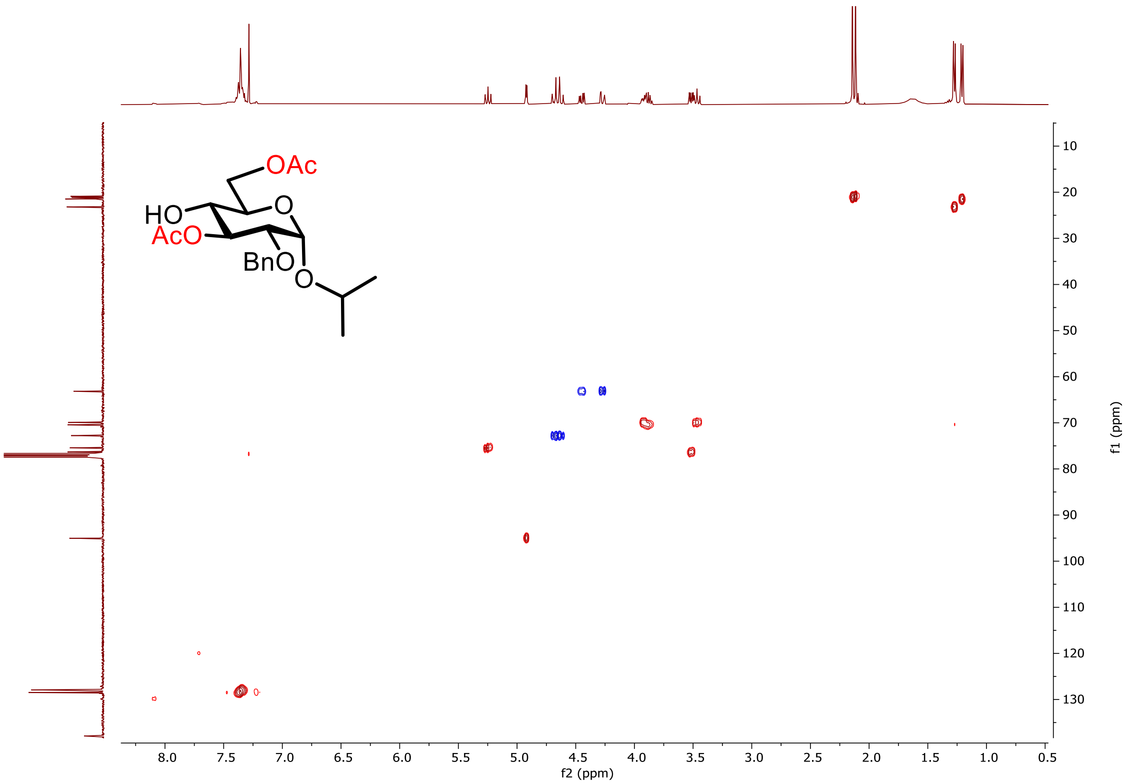

4.16 Isopropyl 2-*O*-benzyl-3,6-di-*O*-pivaloyl-D-glucopyranoside, 89

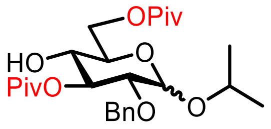

R_1=R_2=OPiv

Total yield: 88% (42.3 mg). Ratio of anomer α : β = 6.4 : 1.

Spectrum data for isopropyl 2-*O*-benzyl-3,6-di-*O*-pivaloyl-α-D-glucopyranoside **89a**: ^{1}H NMR (400 MHz, $CDCl_3$) δ 7.45 – 7.29 (m, 5H), 5.19 (appt, *J* = 9.5 Hz, 1H), 4.83 (d, *J* = 3.7 Hz, 1H), 4.75 (d, *J* = 12.0 Hz, 1H), 4.56 (d, *J* = 12.0 Hz, 1H), 4.39 (dd, *J* = 11.9, 2.2 Hz, 1H), 4.26 (dd, *J* = 11.9, 6.6 Hz, 1H), 3.98 – 3.84 (m, 2H), 3.53 (dd, *J* = 9.9, 3.7 Hz, 1H), 3.42 (appt, *J* = 9.5 Hz, 1H), 3.22 (br. s, 1H), 1.30 – 1.25 (m, 12H), 1.22 (s, 9H), 1.18 (d, *J* = 6.1 Hz, 3H); ^{13}C NMR (101 MHz, $CDCl_3$) δ 180.76, 178.55, 137.77, 128.53, 128.11, 94.59, 75.89, 73.23, 70.90, 70.22, 69.75, 63.60, 39.03, 38.86, 27.21, 23.23, 21.27; $[\alpha]_D^{25}$ 44.22 (c = 0.5, $CHCl_3$); IR (neat) ν_{max} = 3486, 2975, 1732, 1159, 1030, 699 cm^{-1}; m/z (HRMS$^+$) [M + Na]$^+$ 503.2604 ($C_{26}H_{40}O_8Na^+$ requires 503.2615).

^1H NMR of crude 89 (600 MHz, $CDCl_3$)

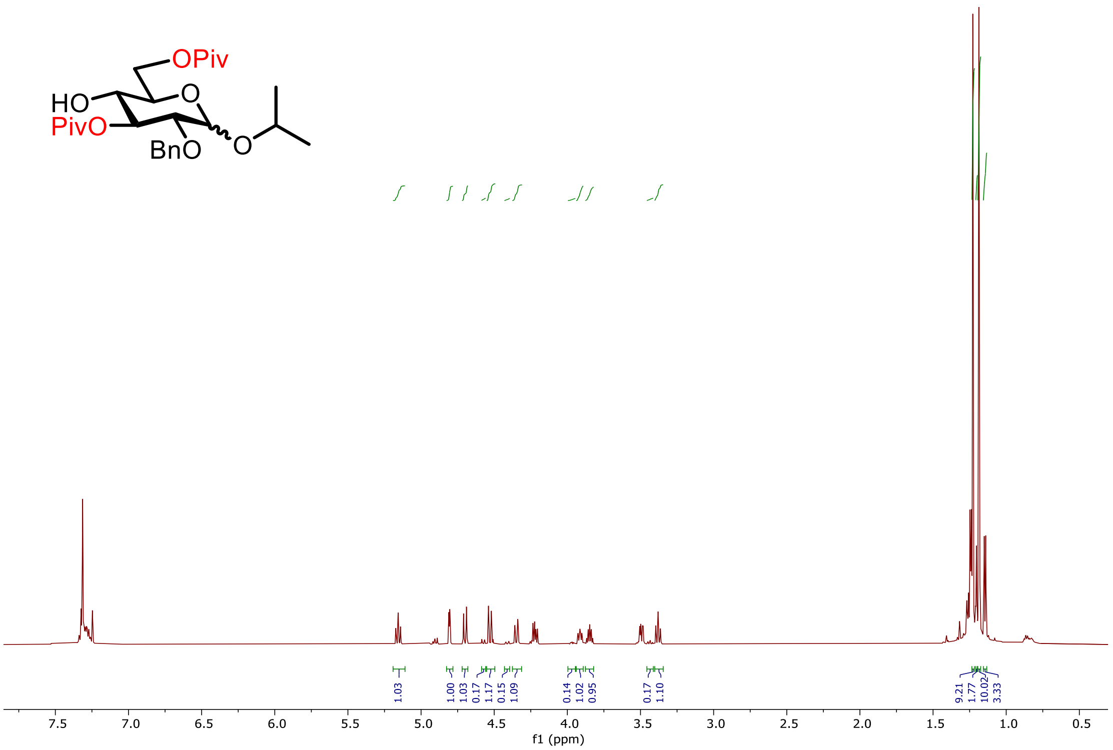

^{13}C NMR of crude 89 (151 MHz, $CDCl_3$)

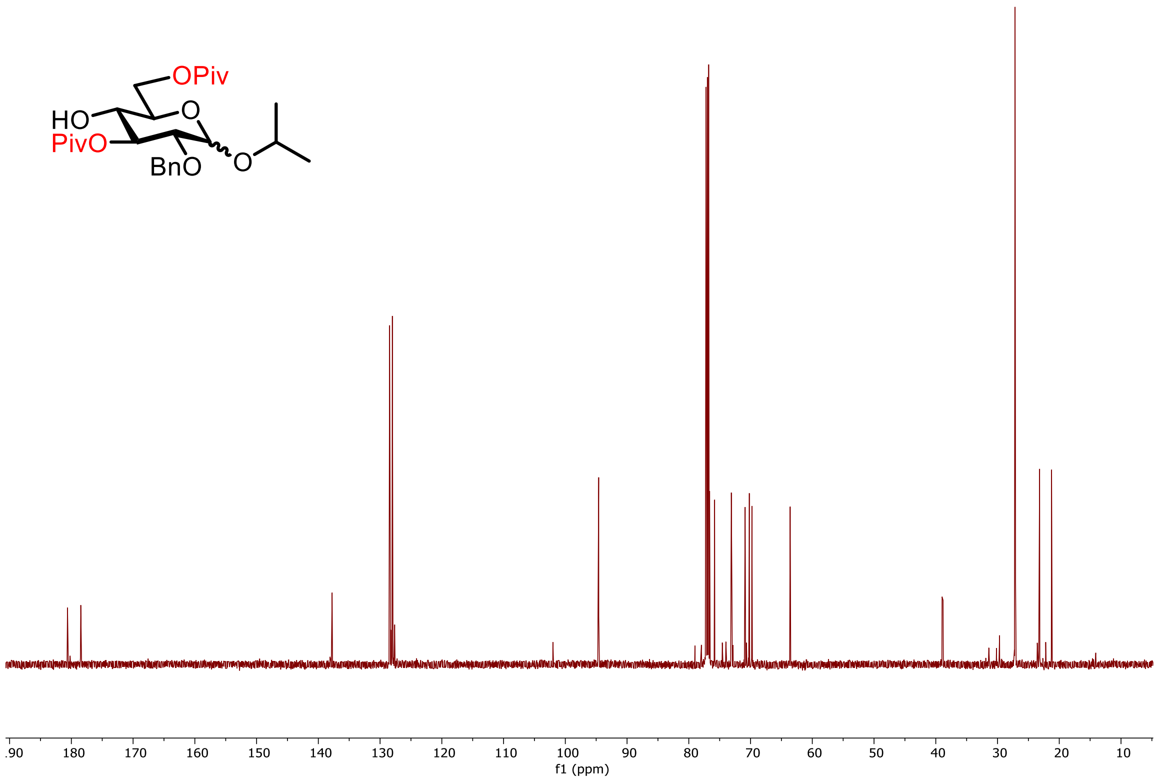

HSQC NMR of crude 89 ($CDCl_3$)

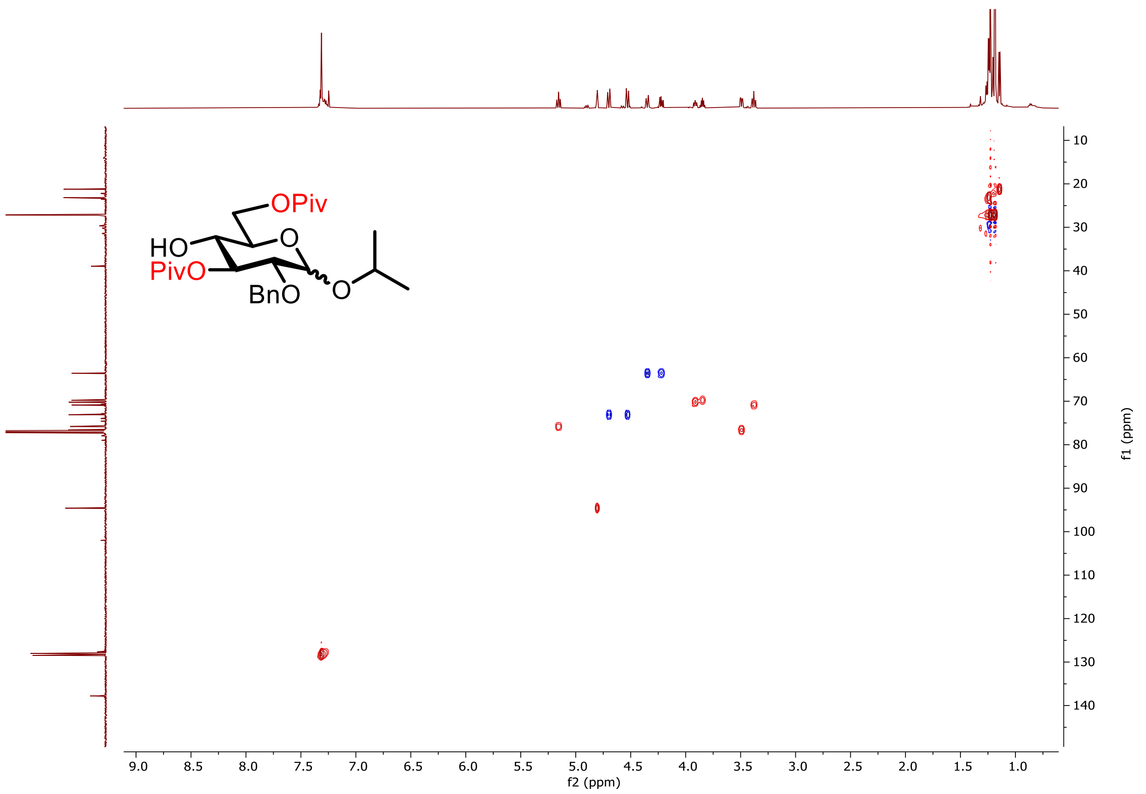

Coupled HSQC NMR of crude 89 ($CDCl_3$)

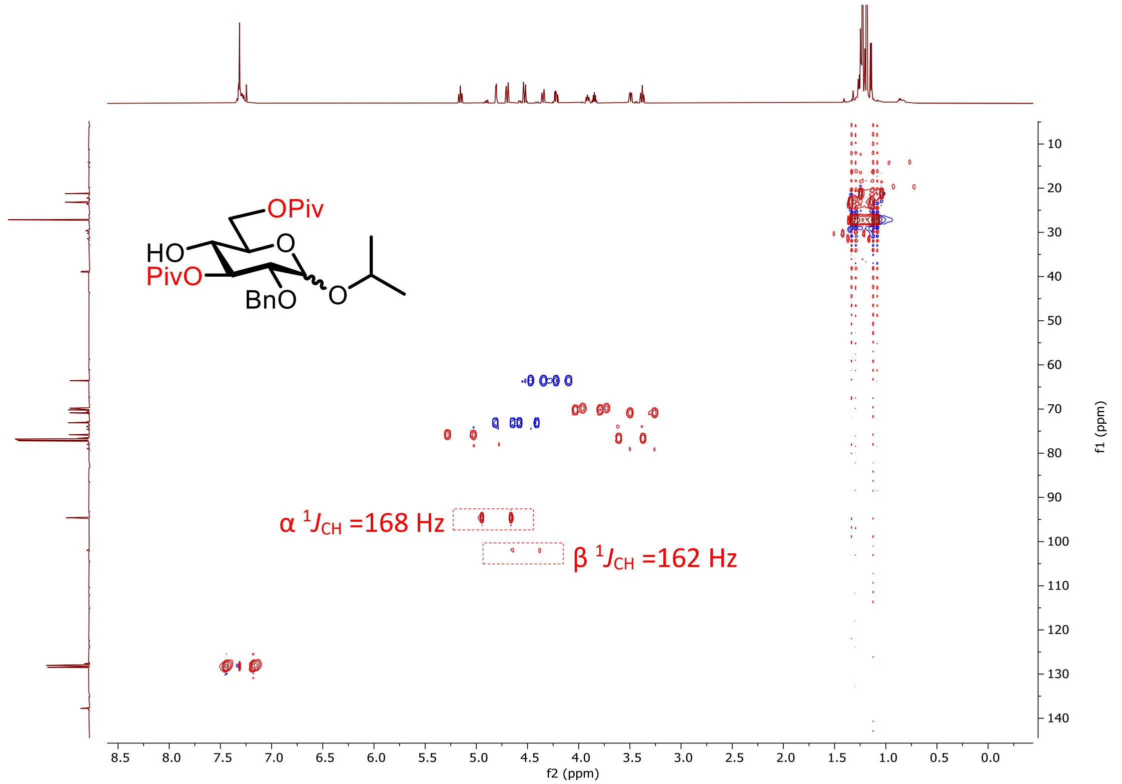

^{1}H NMR of 89a (400 MHz, $CDCl_3$)

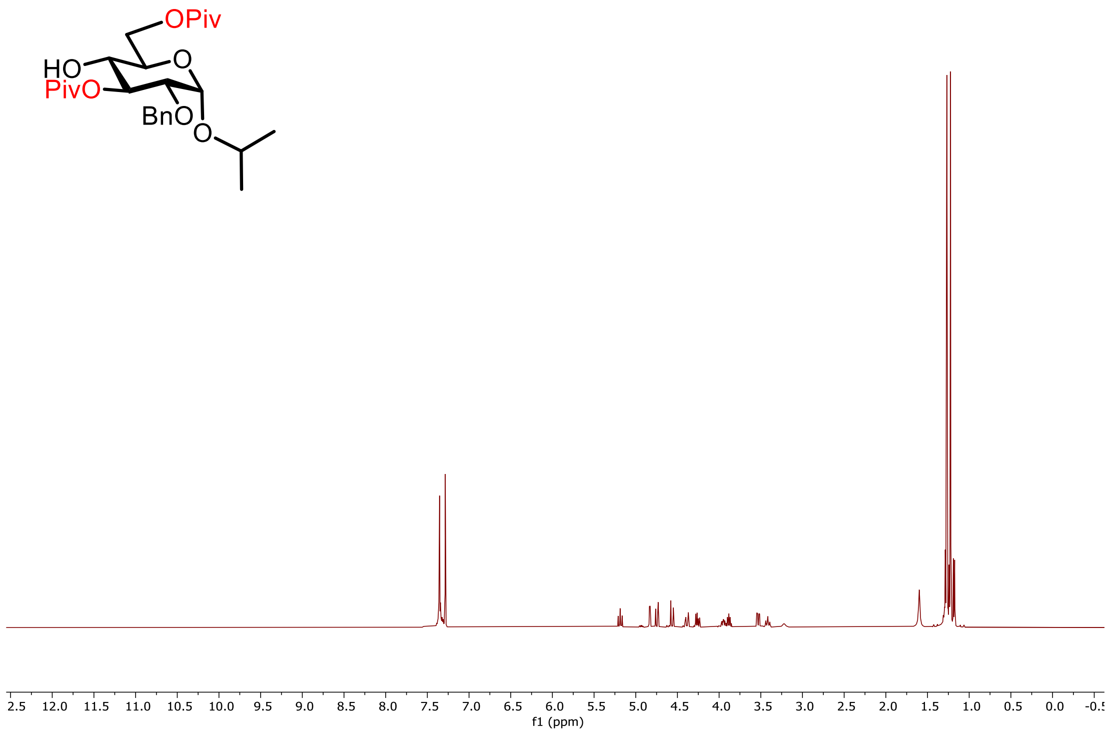

^{13}C NMR of 89a (101 MHz, $CDCl_3$)

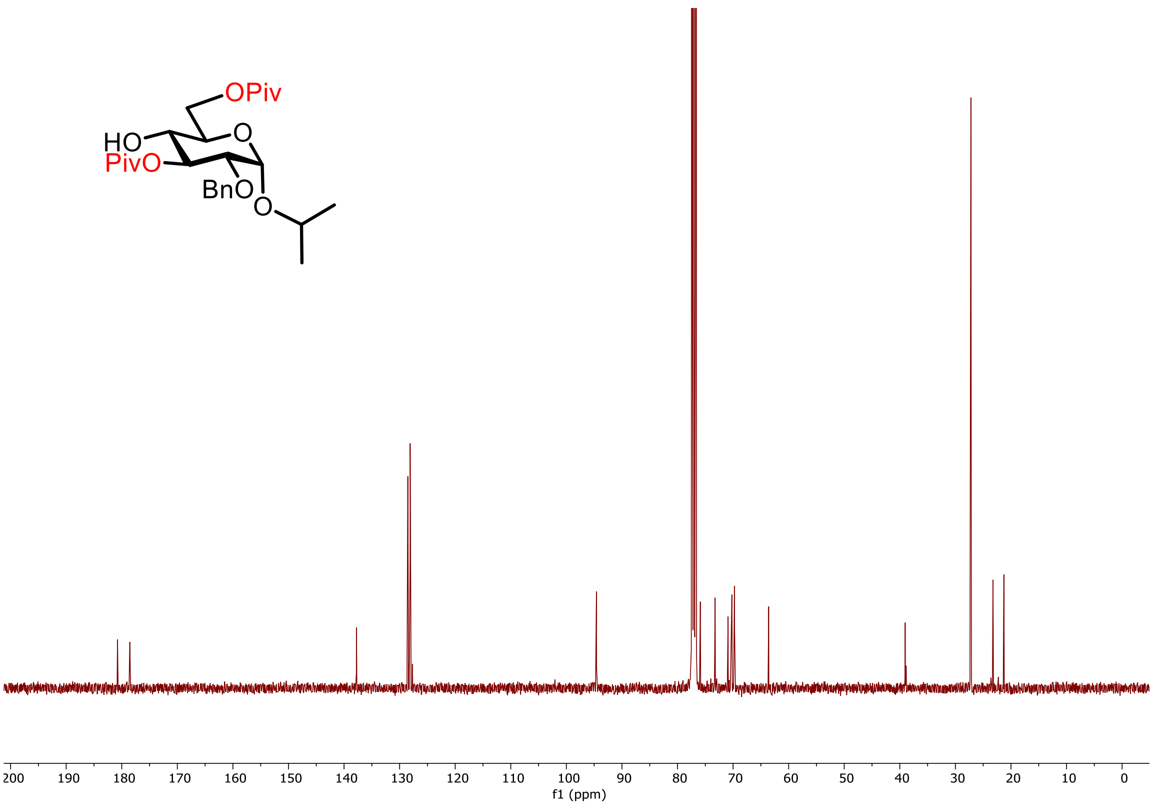

COSY NMR of 89a ($CDCl_3$)

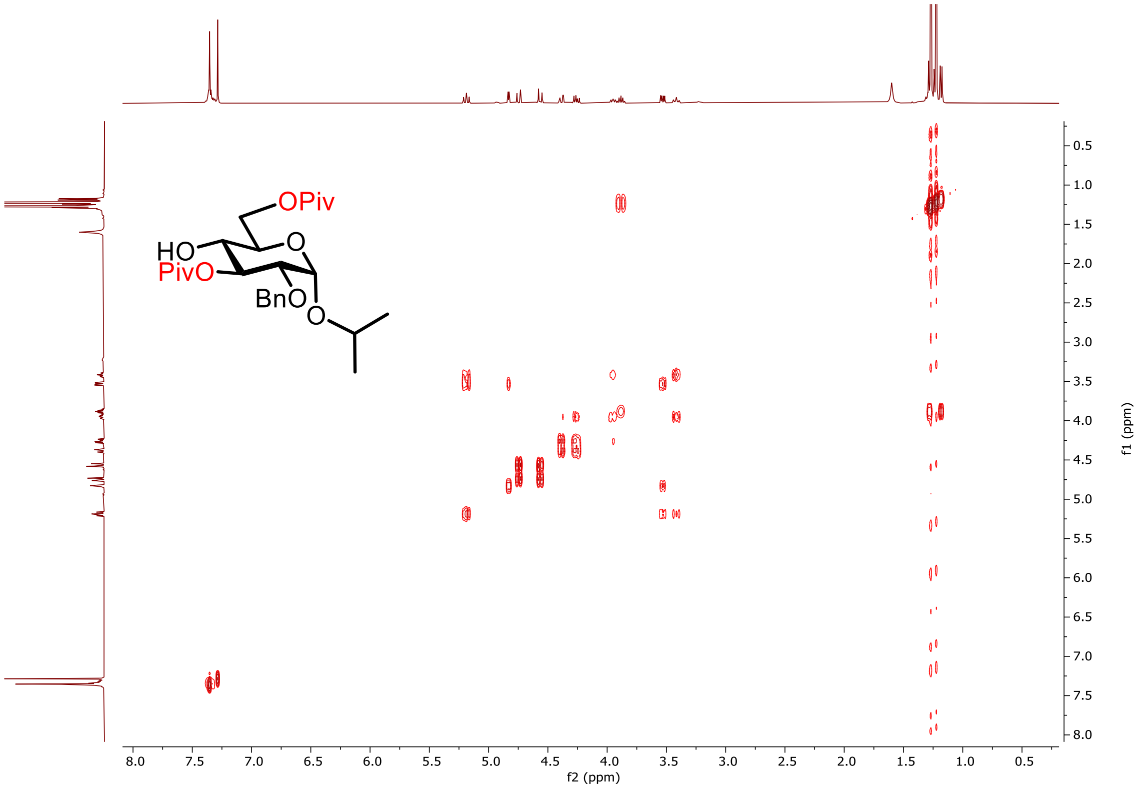

HSQC NMR of 89a ($CDCl_3$)

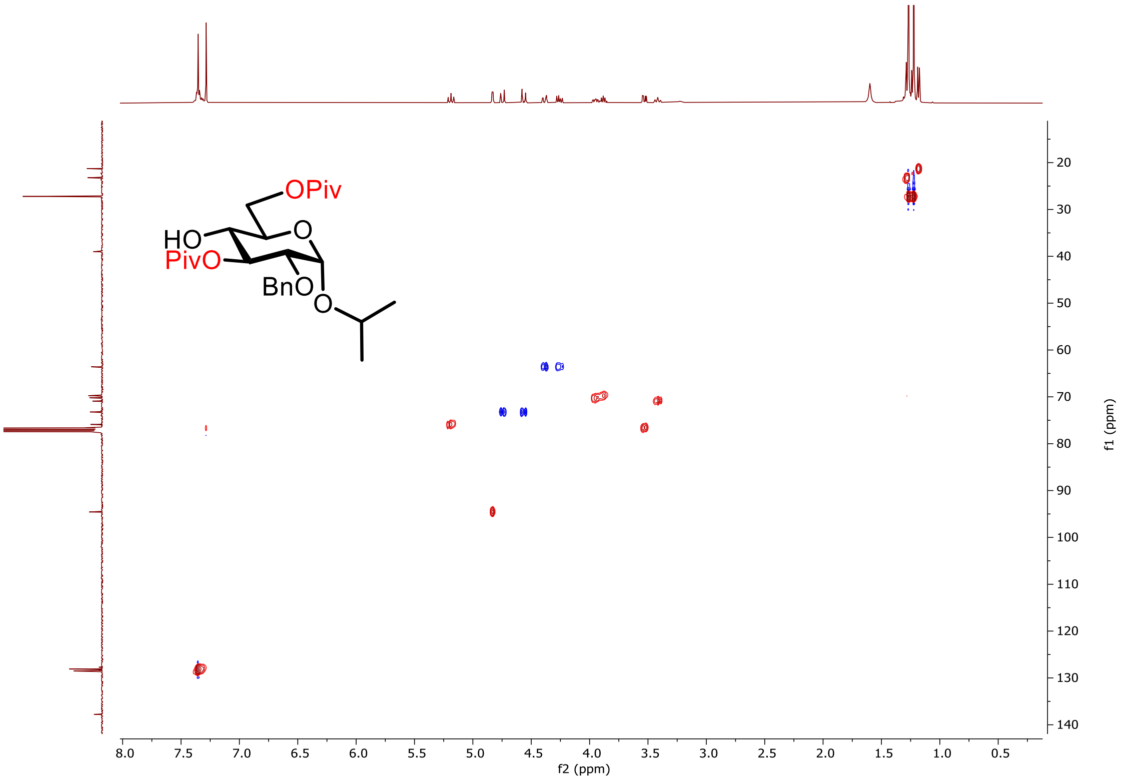

4.17 Isopropyl 2-*O*-benzyl-3,6-di-*O*-benzoyl-D-glucopyranoside, 90

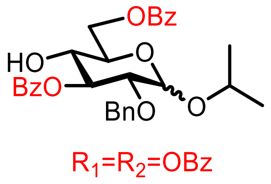

Total yield: 87% (45.3 mg). Ratio of anomer α : β = 4.8 : 1.

Spectrum data for isopropyl 2-*O*-benzyl-3,6-di-*O*-benzoyl-α-D-glucopyranoside **90a**: ^{1}H NMR (400 MHz, $CDCl_3$) δ 8.13 – 8.04 (m, 4H), 7.67 – 7.55 (m, 2H), 7.48 (q, *J* = 8.0 Hz, 4H), 7.29 (s, 5H), 5.55 (appt, *J* = 9.5 Hz, 1H), 5.01 (d, *J* = 3.7 Hz, 1H), 4.75 – 4.56 (m, 4H), 4.16 (ddd, *J* = 10.0, 5.2, 2.3 Hz, 1H), 3.94 (hept, *J* = 6.2 Hz, 1H), 3.74 – 3.65 (m, 2H), 3.39 – 3.28 (br. s, 1H), 1.32 (d, *J* = 6.3 Hz, 3H), 1.23 (d, *J* = 6.1 Hz, 3H); ^{13}C NMR (101 MHz, $CDCl_3$) δ 167.92, 166.81, 137.76, 133.42, 133.19, 129.98, 129.82, 129.79, 129.65, 128.46, 128.40, 128.00, 127.98, 94.95, 76.44, 76.17, 72.65, 70.48, 70.32, 70.16, 63.82, 23.27, 21.51; $[\alpha]_D^{25}$ 88.28 (c = 1, $CHCl_3$); IR (neat) ν_{max} = 3490, 2974, 1721, 1270, 1064, 711 cm^{-1}; m/z (HRMS$^+$) $[M + Na]^+$ 543.1992 ($C_{30}H_{32}O_8Na^+$ requires 543.1989).

^{1}H NMR of crude 90 (600 MHz, $CDCl_3$)

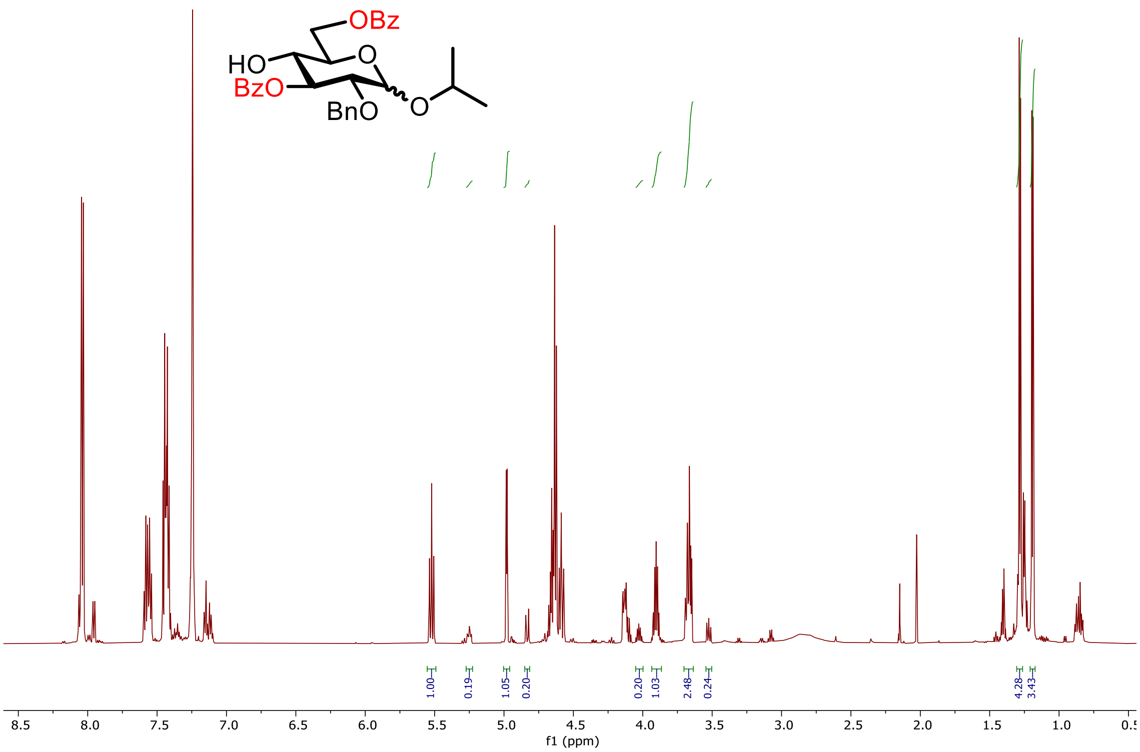

^{13}C NMR of crude 90 (151 MHz, $CDCl_3$)

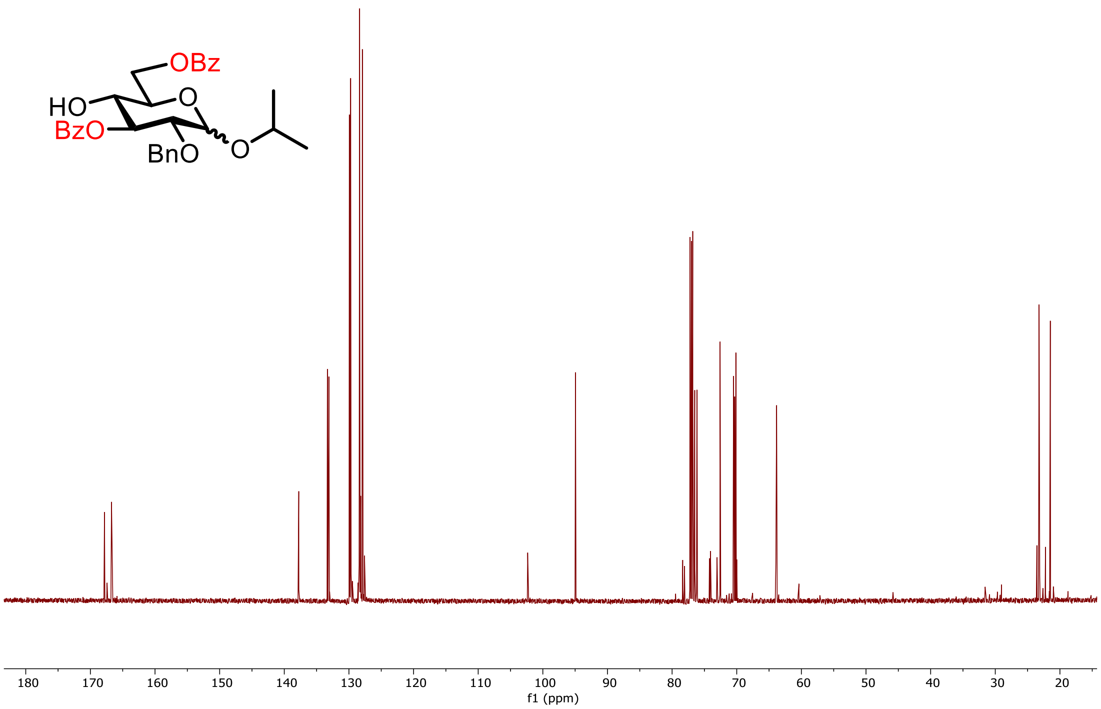

HSQC NMR of crude 90 ($CDCl_3$)

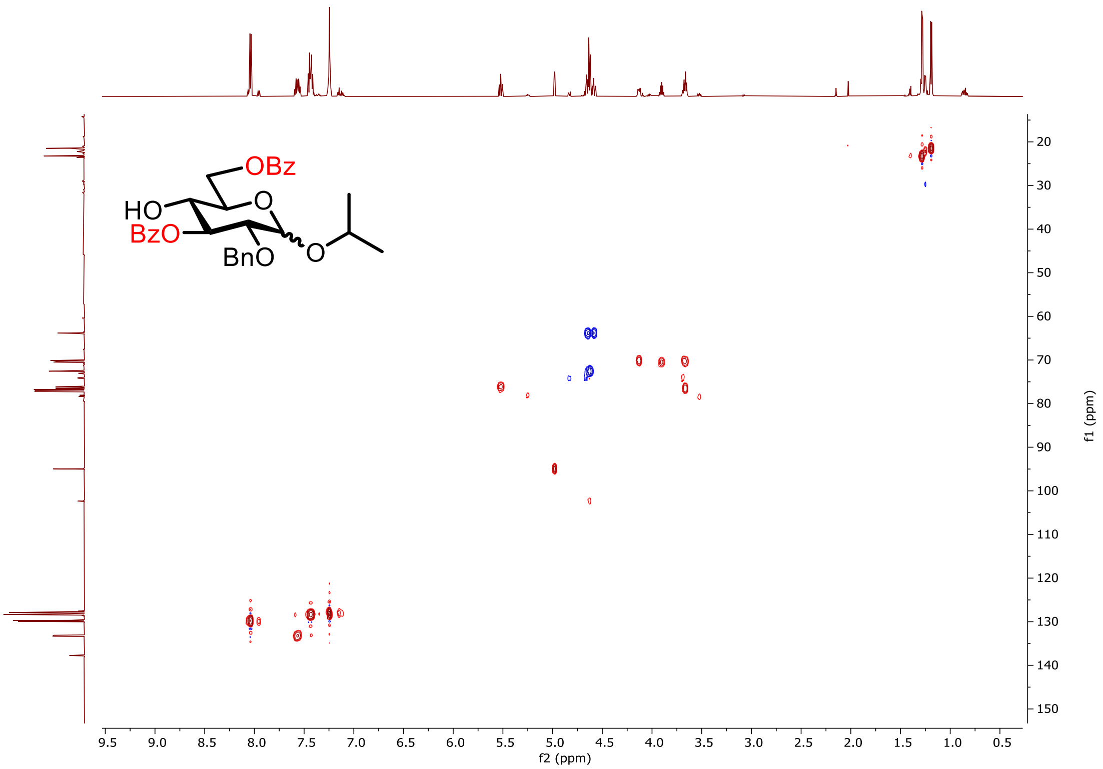

Coupled HSQC NMR of crude 90 ($CDCl_3$)

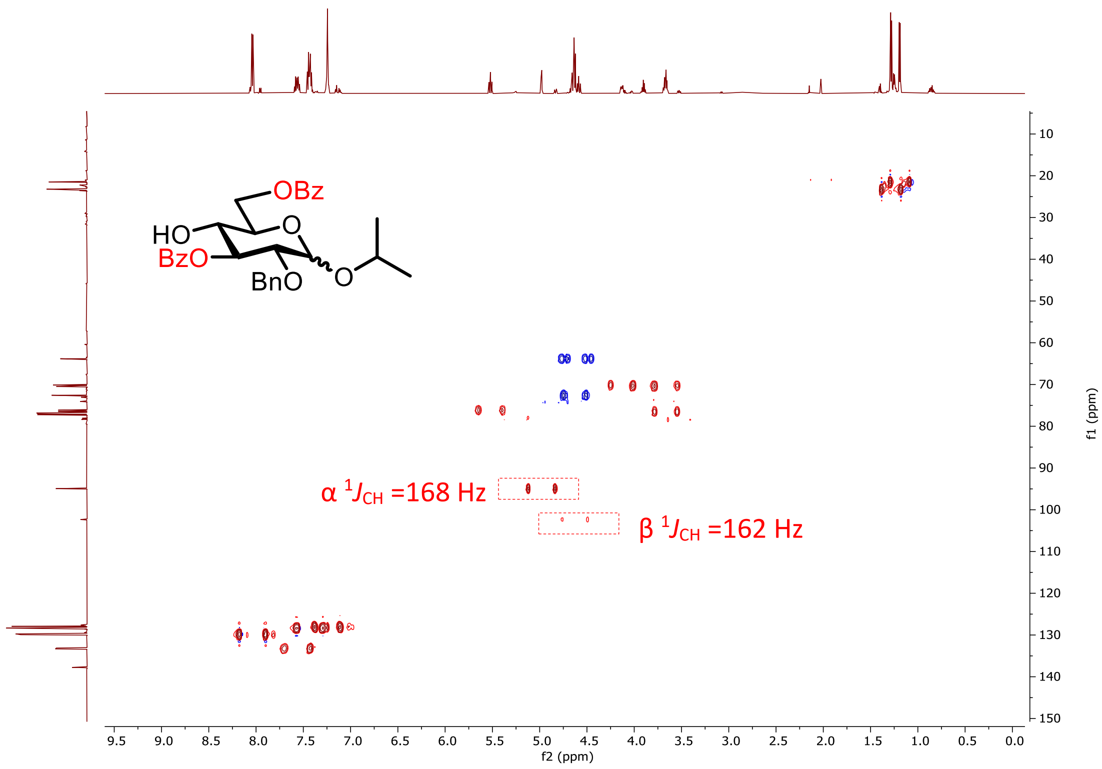

^{1}H NMR of 90a (400 MHz, $CDCl_3$)

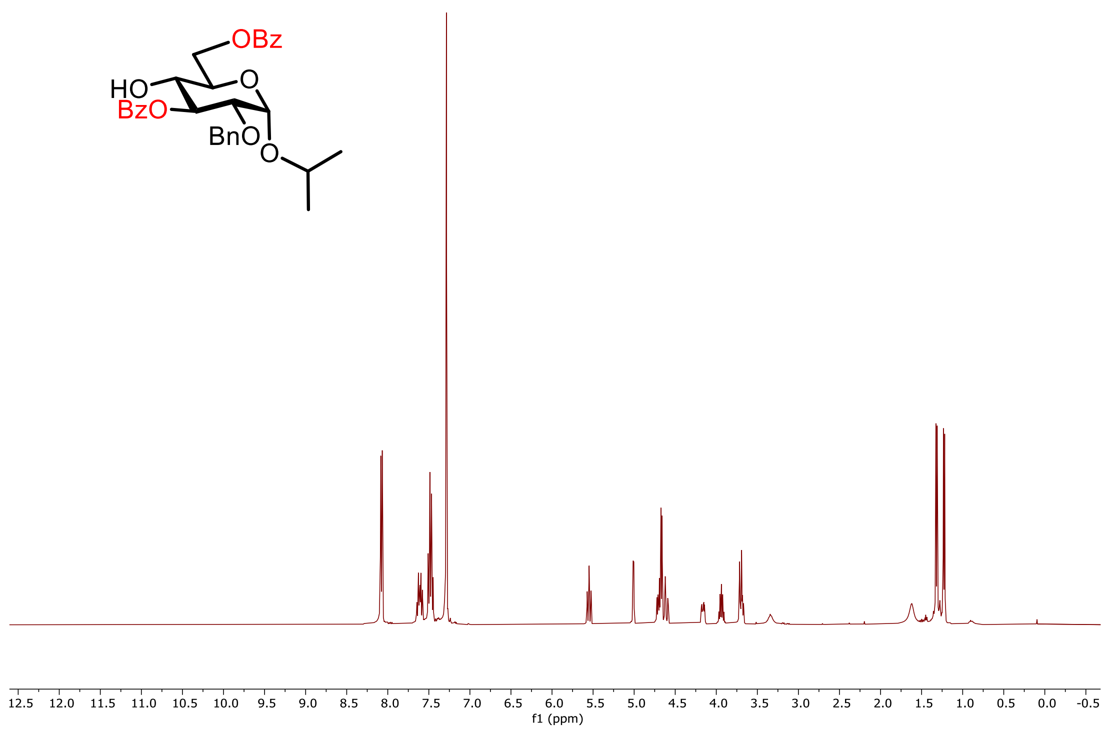

^{13}C NMR of 90a (101 MHz, $CDCl_3$)

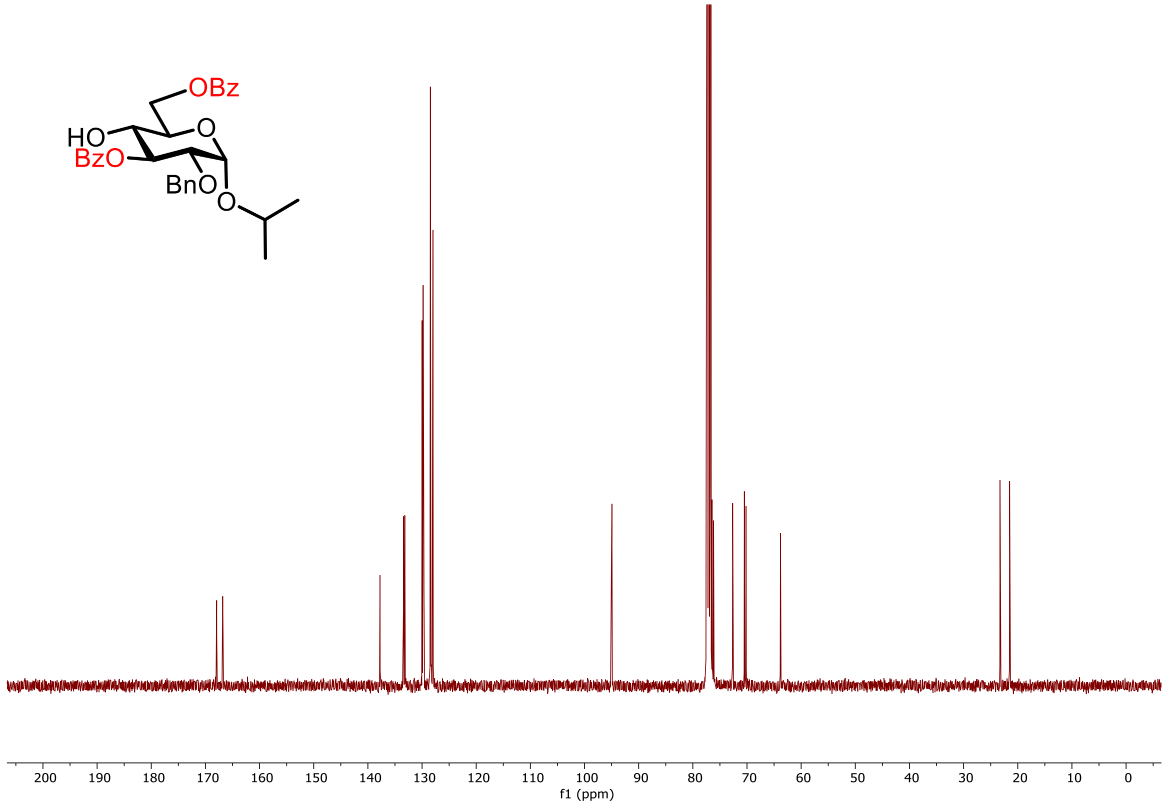

COSY NMR of 90a ($CDCl_3$)

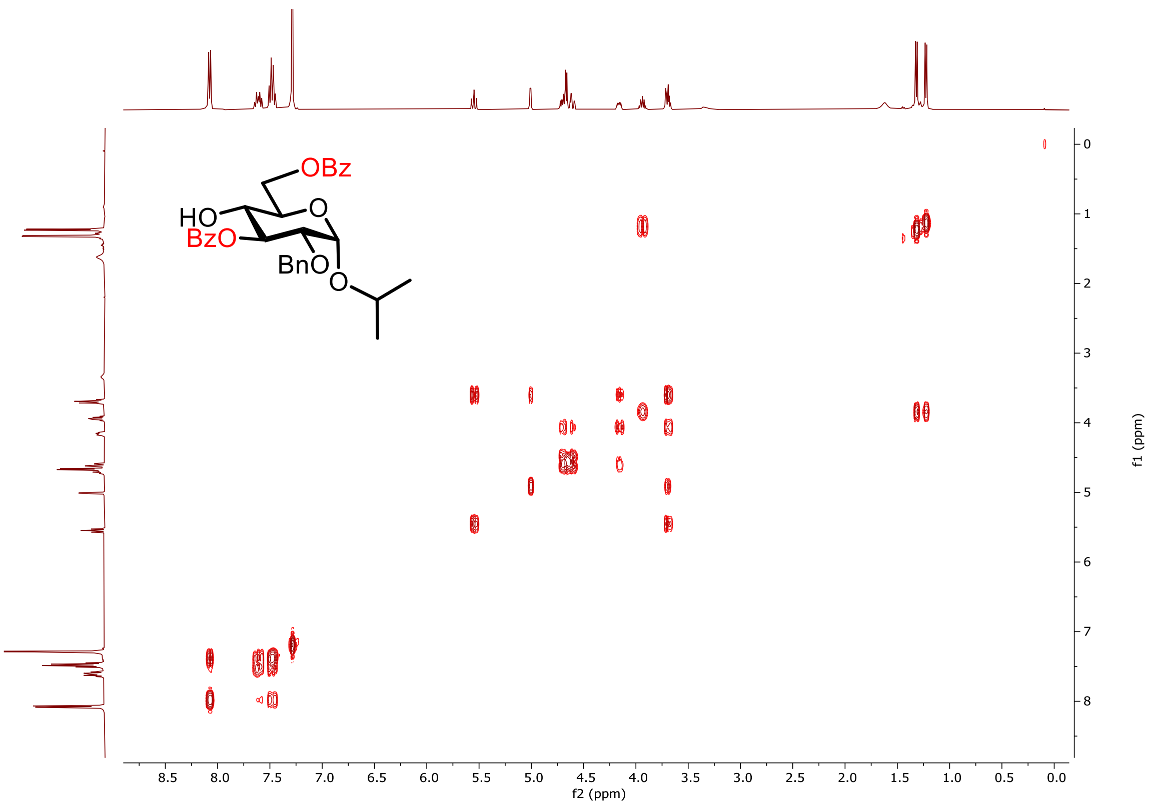

HSQC NMR of 90a ($CDCl_3$)

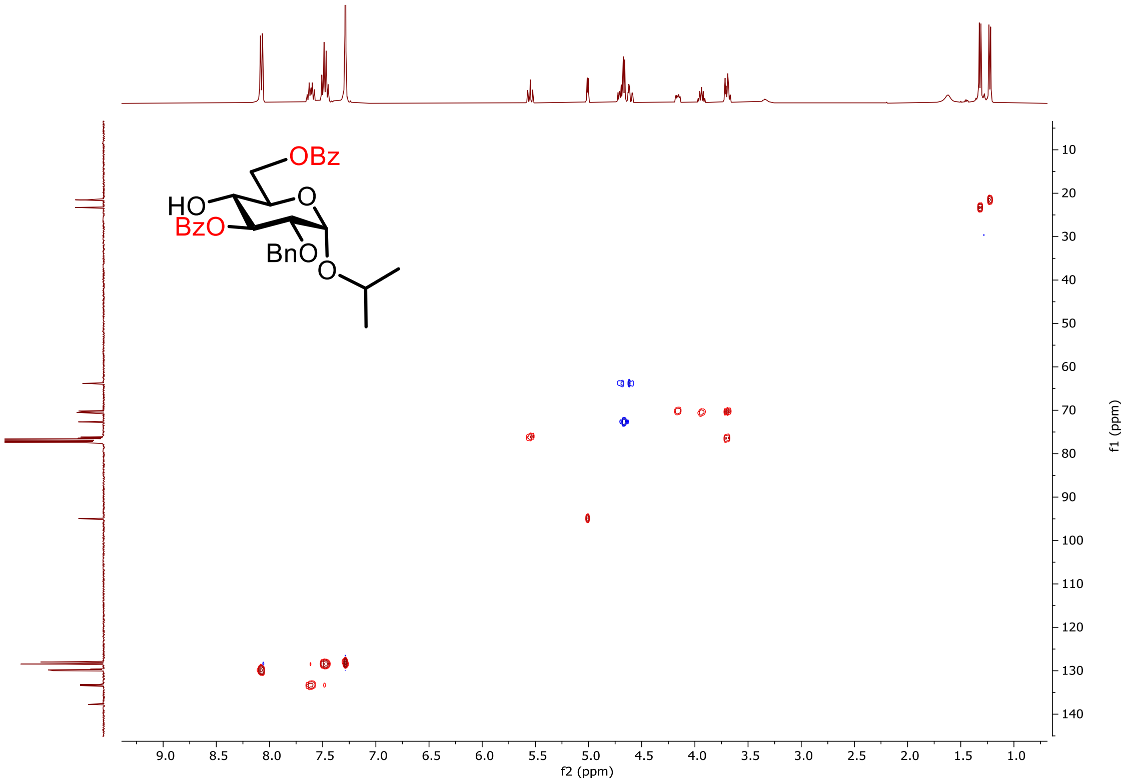

4.18 Isopropyl 2-*O*-benzyl-3,6-di-*O*-(4-nitrobenzoyl)-D-glucopyranoside, 91

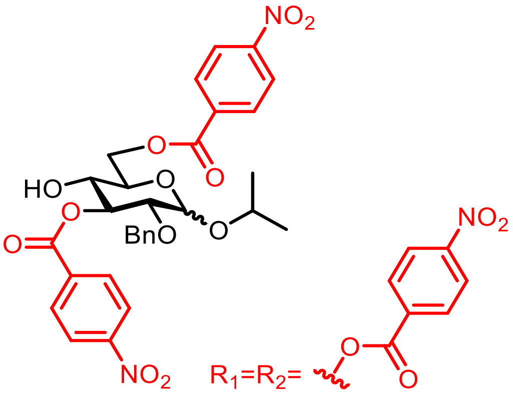

Total yield: 74% (45.2 mg). Ratio of anomer α : β = 7.1 : 1.

Spectrum data for isopropyl 2-*O*-benzyl-3,6-di-*O*-(4-nitrobenzoyl)-α-D-glucopyranoside **91a**: ^{1}H NMR (400 MHz, $CDCl_3$) δ 8.36 – 8.29 (m, 4H), 8.27 – 8.23 (m, 2H), 8.21 – 8.15 (m, 2H), 7.32 – 7.22 (m, 5H), 5.57 (appt, *J* = 9.5 Hz, 1H), 5.06 (d, *J* = 3.7 Hz, 1H), 4.75 (dd, *J* = 12.0, 5.2 Hz, 1H), 4.72 – 4.56 (m, 3H), 4.18 (ddd, *J* = 10.0, 5.2, 2.4 Hz, 1H), 3.93 (p, *J* = 6.2 Hz, 1H), 3.74 – 3.63 (m, 2H), 3.00 (br. s, 1H), 1.32 (d, *J* = 6.2 Hz, 3H), 1.25 (d, *J* = 6.1 Hz, 3H); ^{13}C NMR (101 MHz, $CDCl_3$) δ 165.73, 164.92, 150.75, 137.43, 135.08, 134.96, 131.04, 130.90, 128.55, 128.20, 128.00, 123.65, 123.62, 94.85, 76.26, 72.46, 70.97, 70.18, 69.95, 64.60, 23.28, 21.60; $[\alpha]_D^{25}$ 90.20 (c = 0.25, $CHCl_3$); IR (neat) ν_{max} = 3506, 2926, 1728, 1526, 1269, 719 cm^{-1}; m/z (HRMS$^+$) [M + Na]$^+$ 633.1724 ($C_{30}H_{30}N_2O_{12}Na^+$ requires 633.1691).

^{1}H NMR of crude 91 (600 MHz, $CDCl_3$)

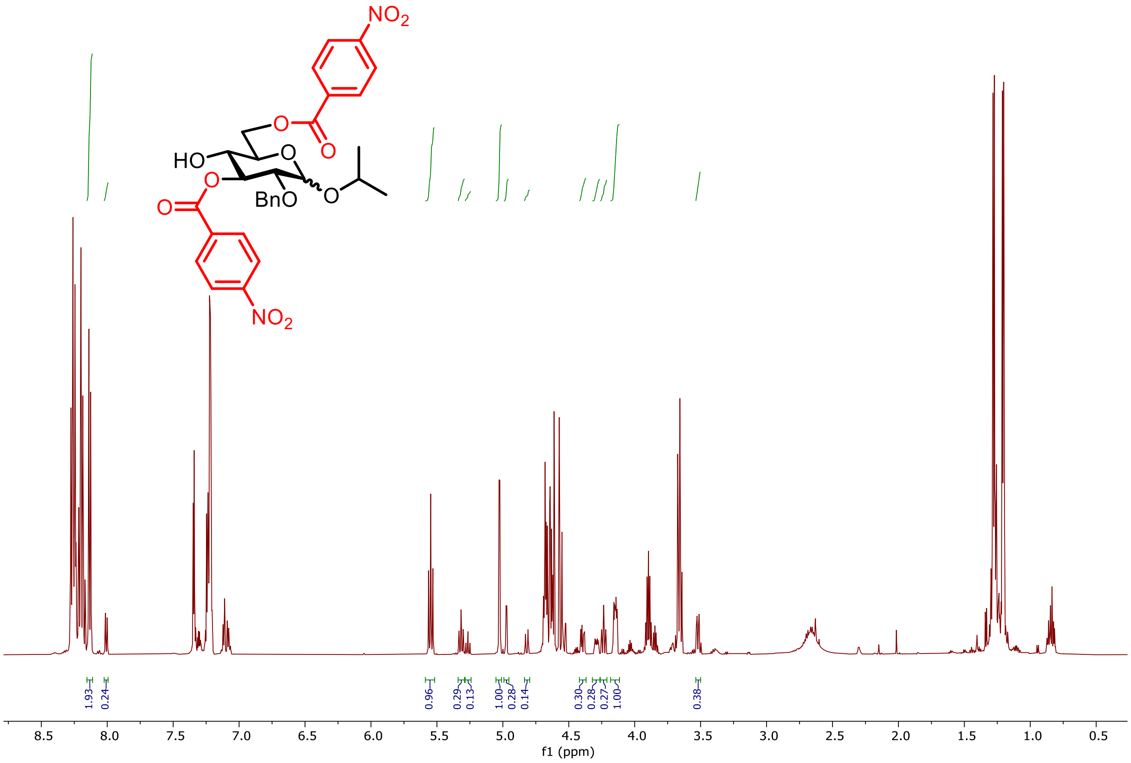

^{13}C NMR of crude 91 (151 MHz, $CDCl_3$)

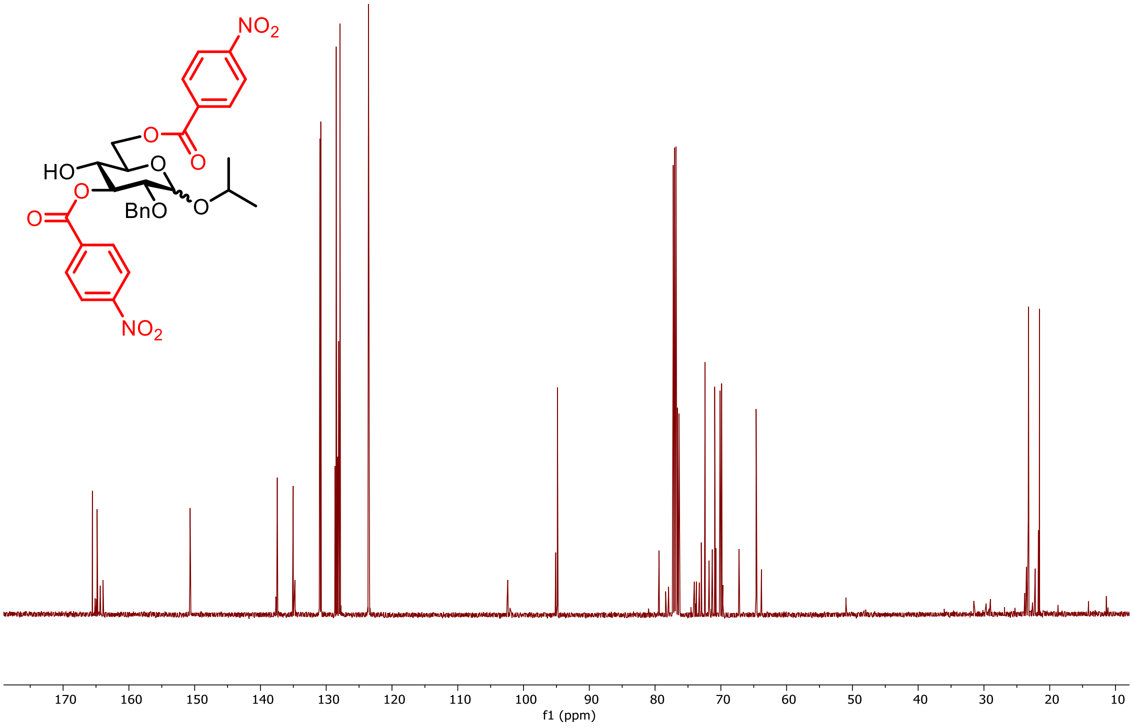

HSQC NMR of crude 91 ($CDCl_3$)

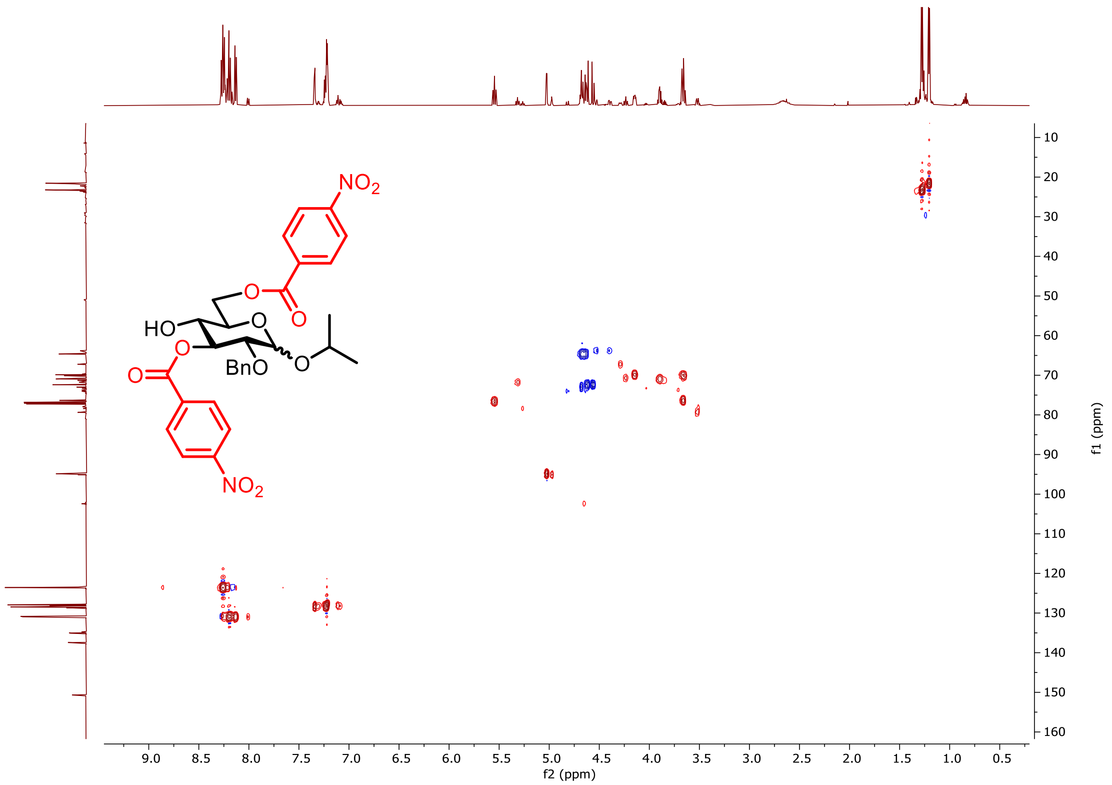

Coupled HSQC NMR of crude 91 ($CDCl_3$)

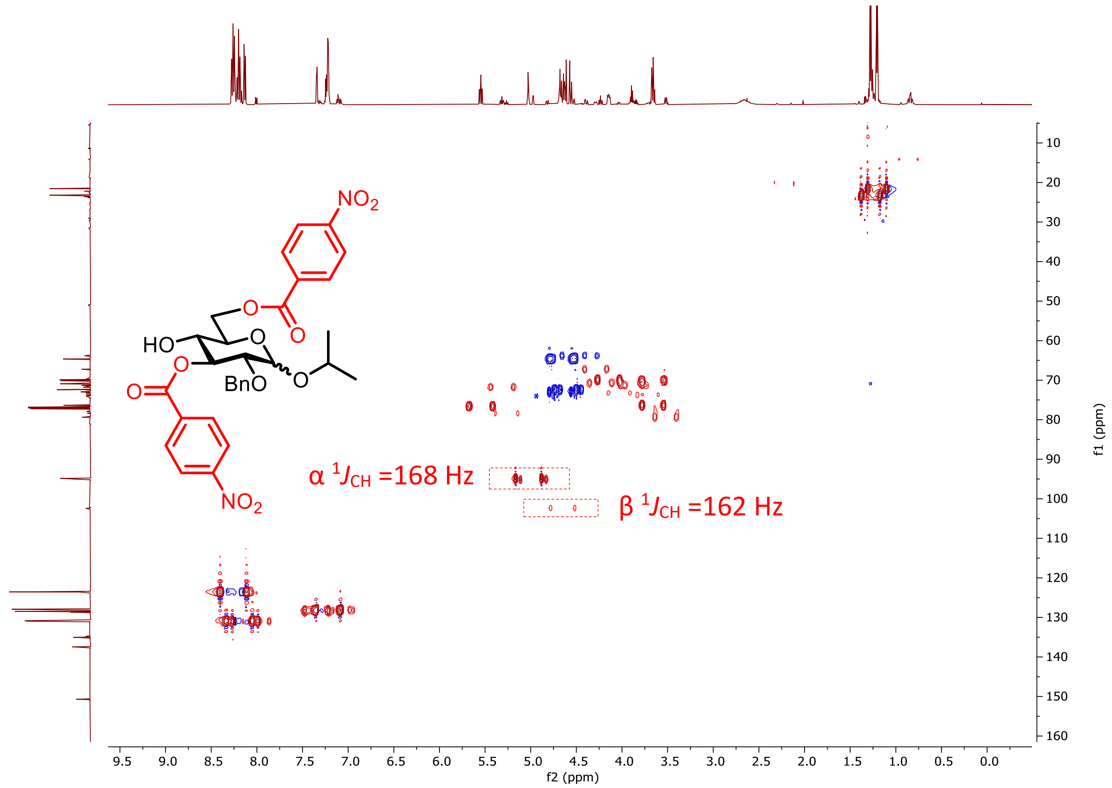

^{1}H NMR of 91a (400 MHz, $CDCl_3$)

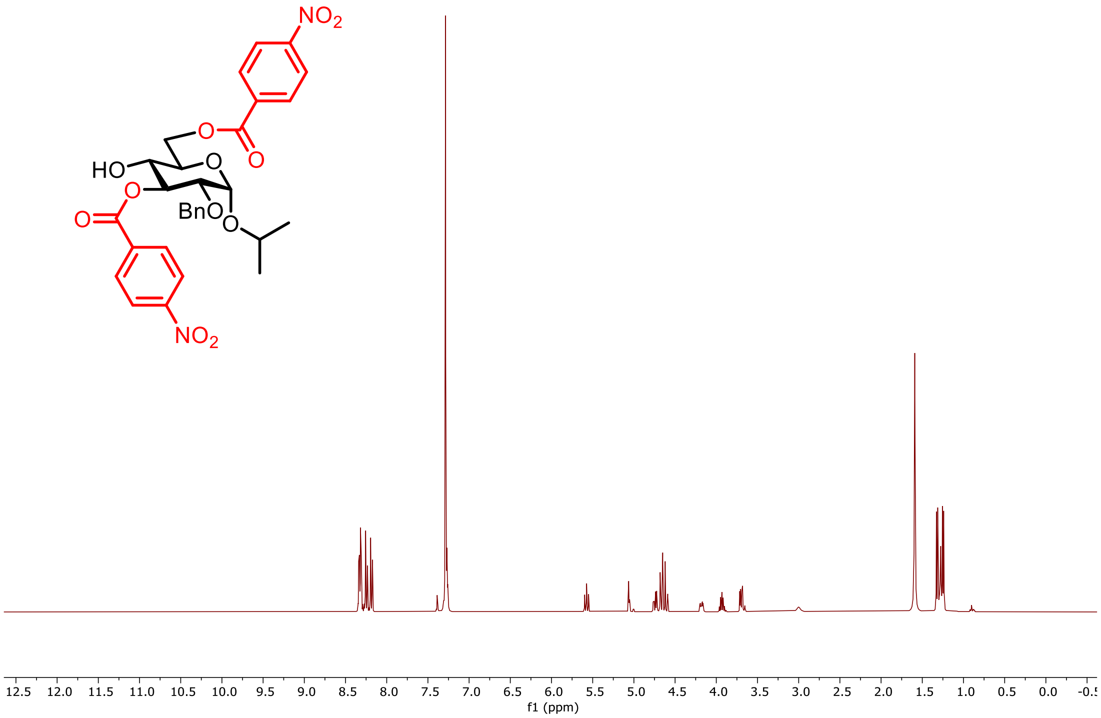

^{13}C NMR of 91a (101 MHz, $CDCl_3$)

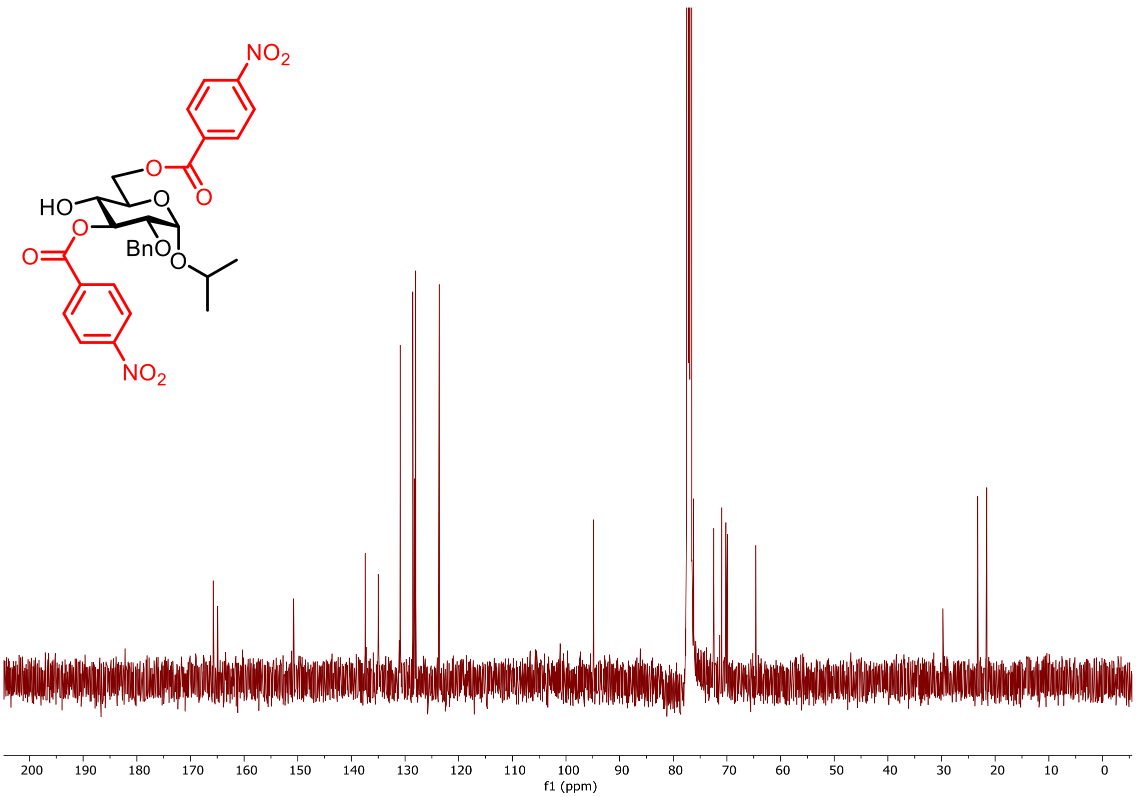

COSY NMR of 91a ($CDCl_3$)

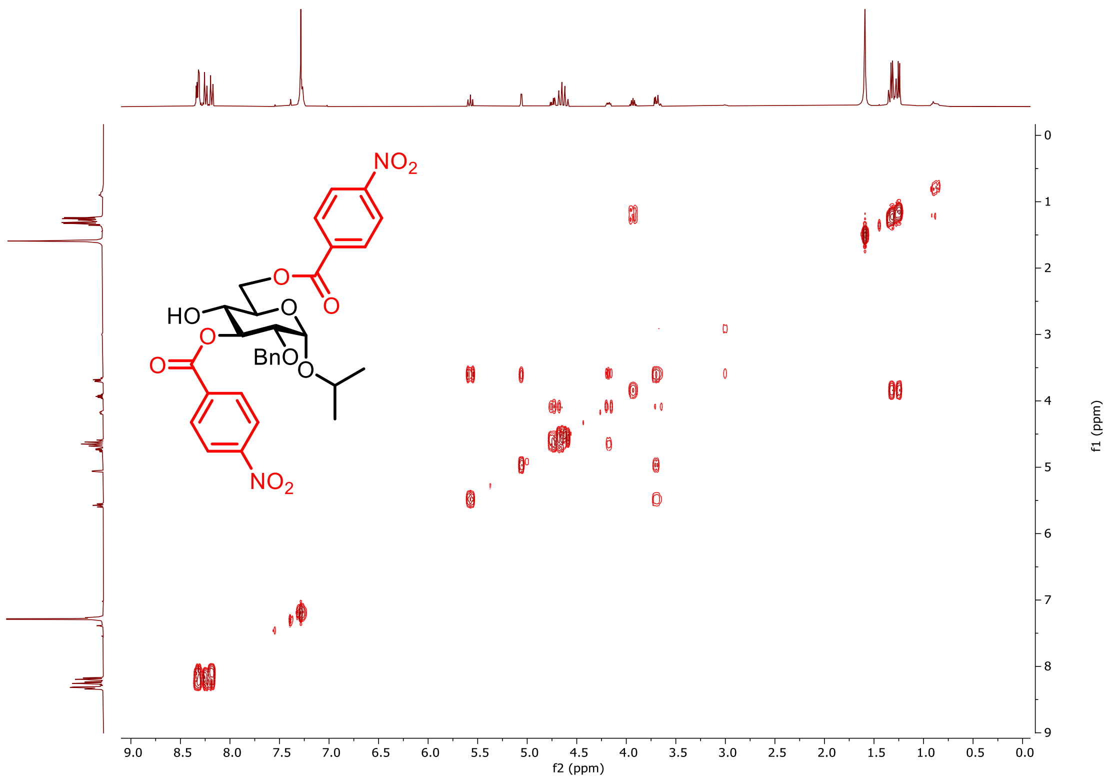

HSQC NMR of 91a ($CDCl_3$)

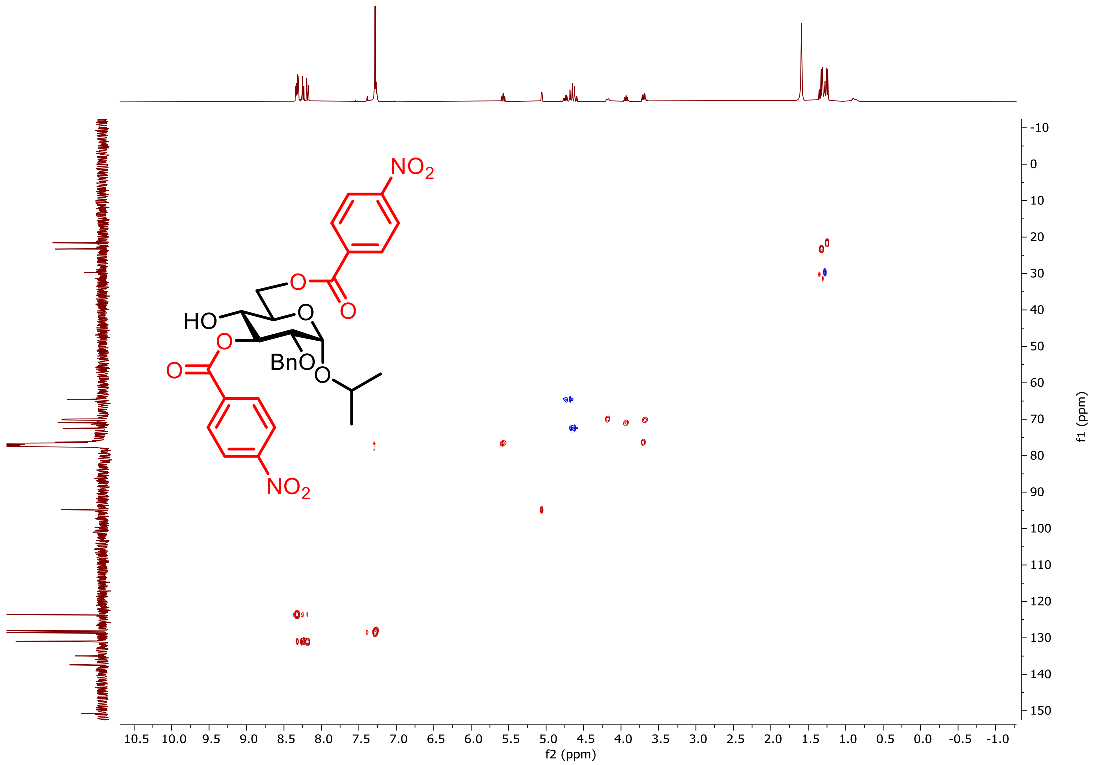

4.19 Isopropyl 2-*O*-benzyl-3-*O*-benzoyl-6-*O*-levulinoyl-D-glucopyranoside, 92

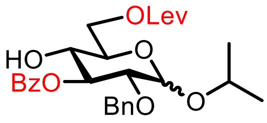

R_1=OLev, R_2=OBz

Total yield: 76% (39.1 mg). Ratio of anomer α : β = 2.4 : 1.

Spectrum data for isopropyl 2-*O*-benzyl-3-*O*-benzoyl-6-*O*-levulinoyl-α-D-glucopyranoside **92a**: ^{1}H NMR (400 MHz, $CDCl_3$) δ 8.11 – 8.04 (m, 2H), 7.67 – 7.58 (m, 1H), 7.53 – 7.44 (m, 2H), 7.29 (s, 5H), 5.52 (appt, *J* = 9.5 Hz, 1H), 4.99 (d, *J* = 3.7 Hz, 1H), 4.72 – 4.60 (m, 2H), 4.51 (dd, *J* = 12.1, 4.8 Hz, 1H), 4.30 (dd, *J* = 12.1, 2.3 Hz, 1H), 4.00 (ddd, *J* = 9.9, 4.7, 2.2 Hz, 1H), 3.92 (p, *J* = 6.1 Hz, 1H), 3.71 – 3.60 (m, 2H), 3.20 (br. s, 1H), 2.82 – 2.75 (m, 2H), 2.69 – 2.60 (m, 2H), 2.20 (s, 3H), 1.31 (d, *J* = 6.3 Hz, 3H), 1.23 (d, *J* = 6.1 Hz, 3H); ^{13}C NMR (151 MHz, $CDCl_3$) δ 206.08, 173.09, 167.69, 133.35, 129.97, 129.74, 128.45, 128.43, 128.01, 127.95, 94.95, 76.41, 75.99, 72.64, 70.42, 69.94, 69.92, 63.40, 37.94, 29.89, 27.82, 23.23, 21.46; $[\alpha]_D^{25}$ 60.80 (c = 0.5, $CHCl_3$); IR (neat) ν_{max} = 3500, 2925, 1722, 1272, 1066, 713 cm^{-1}; m/z ($HRMS^+$) $[M + Na]^+$ 537.2098 ($C_{28}H_{34}O_9Na^+$ requires 537.2095).

¹H NMR of crude 92 (400 MHz, CDCl$_3$)

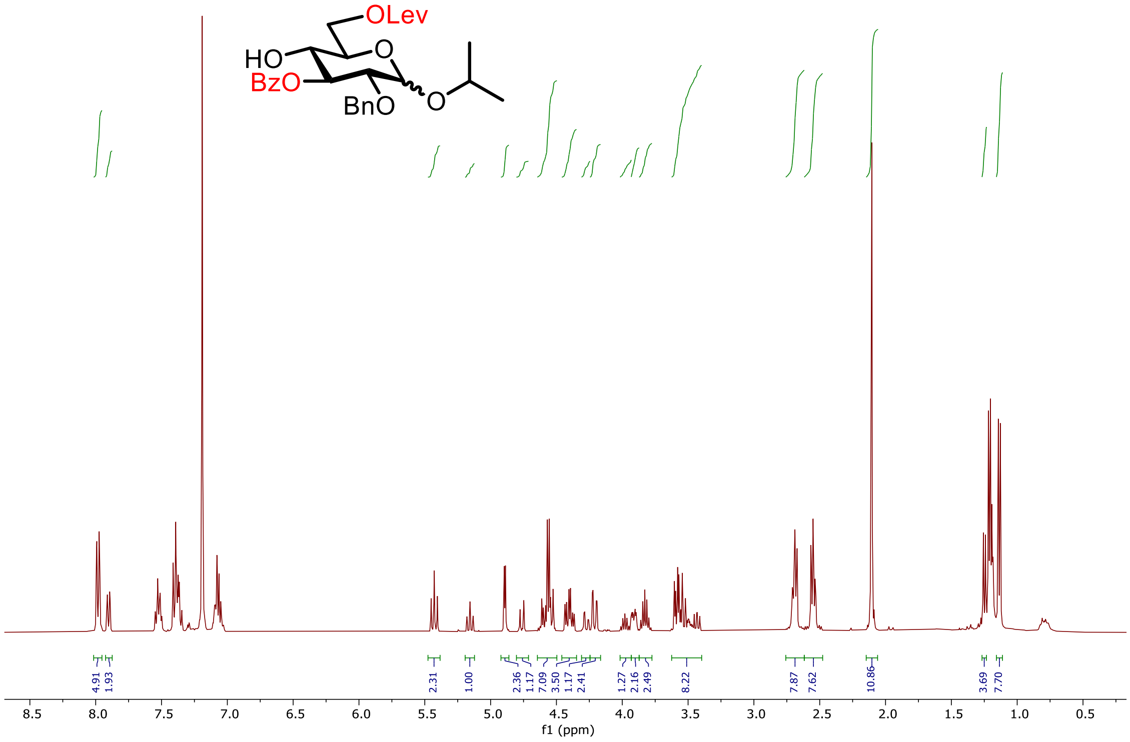

¹³C NMR of crude 92 (101 MHz, CDCl$_3$)

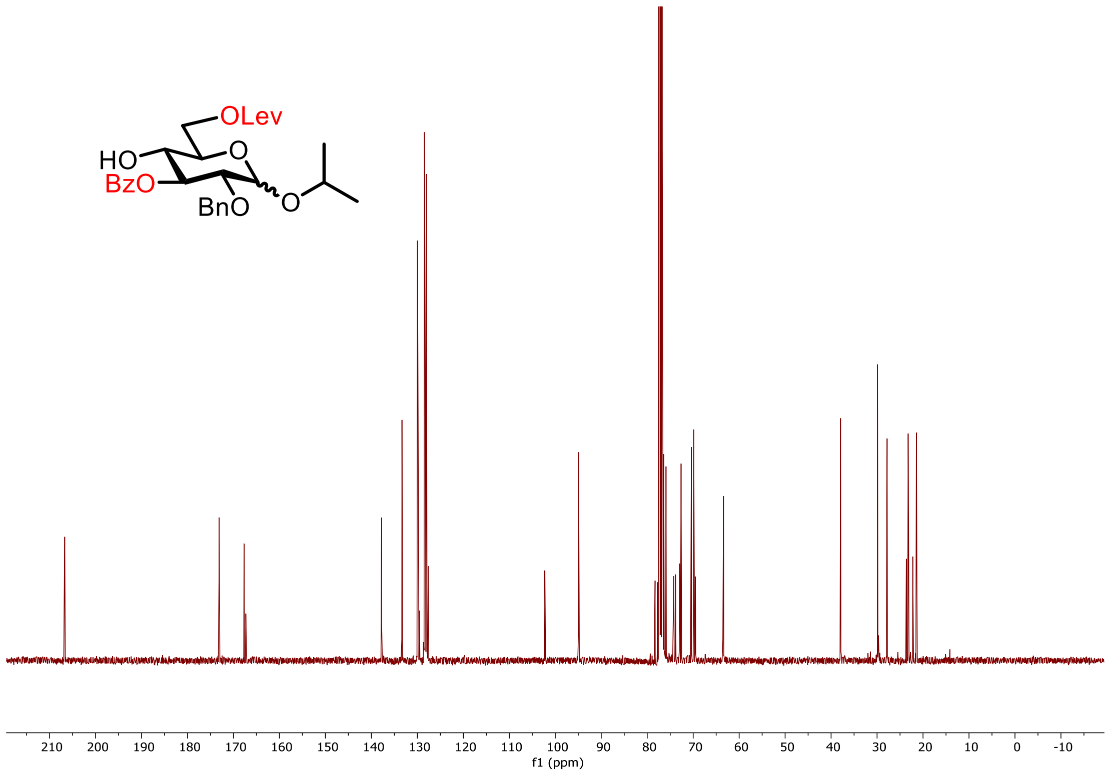

HSQC NMR of crude 92 ($CDCl_3$)

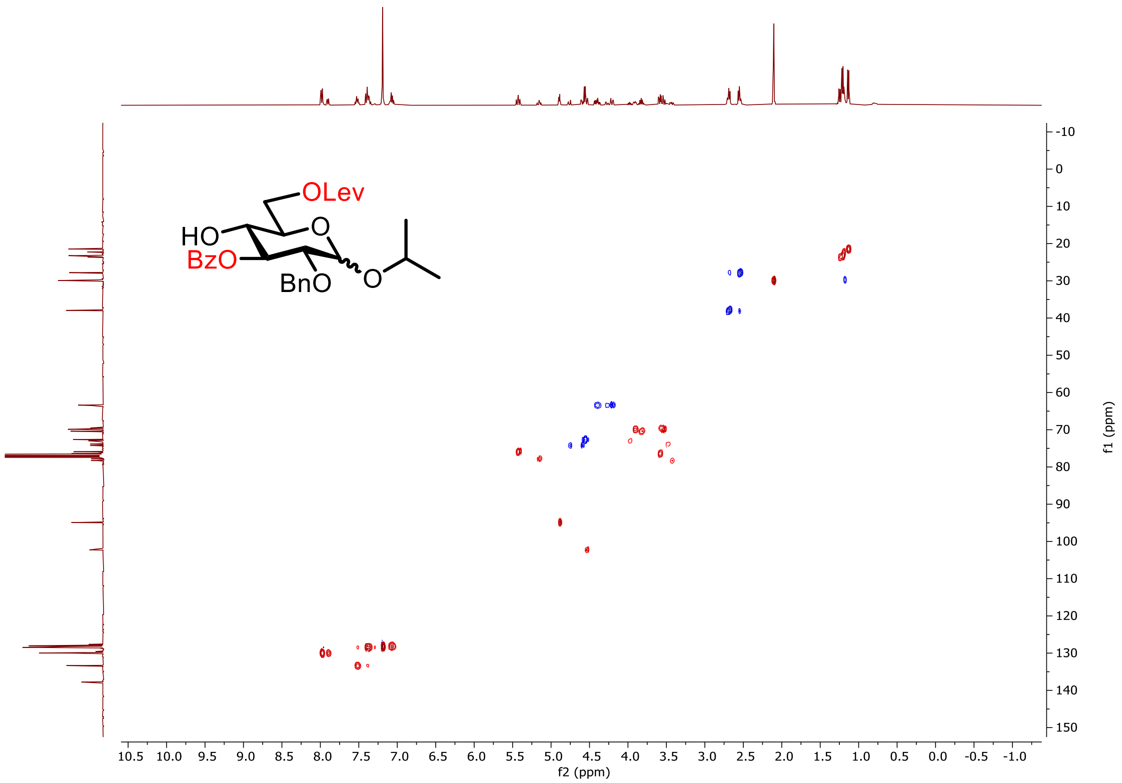

Coupled HSQC NMR of crude 92 ($CDCl_3$)

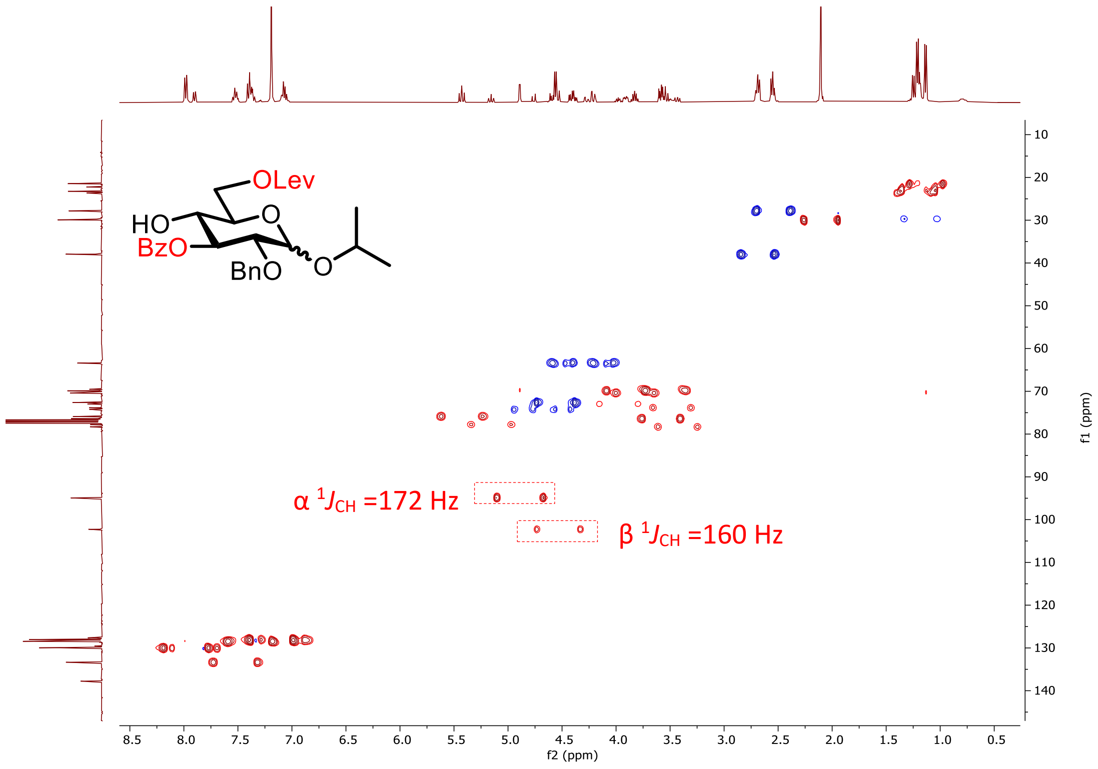

^{1}H NMR of 92a (400 MHz, $CDCl_3$)

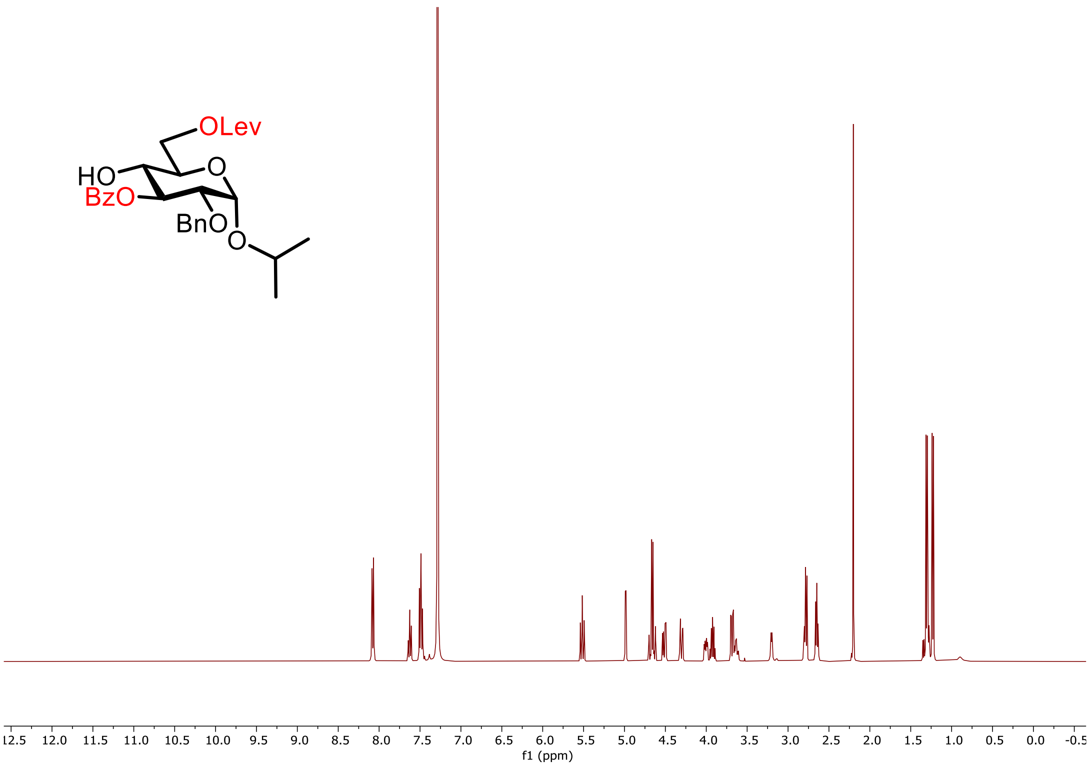

^{13}C NMR of 92a (151 MHz, $CDCl_3$)

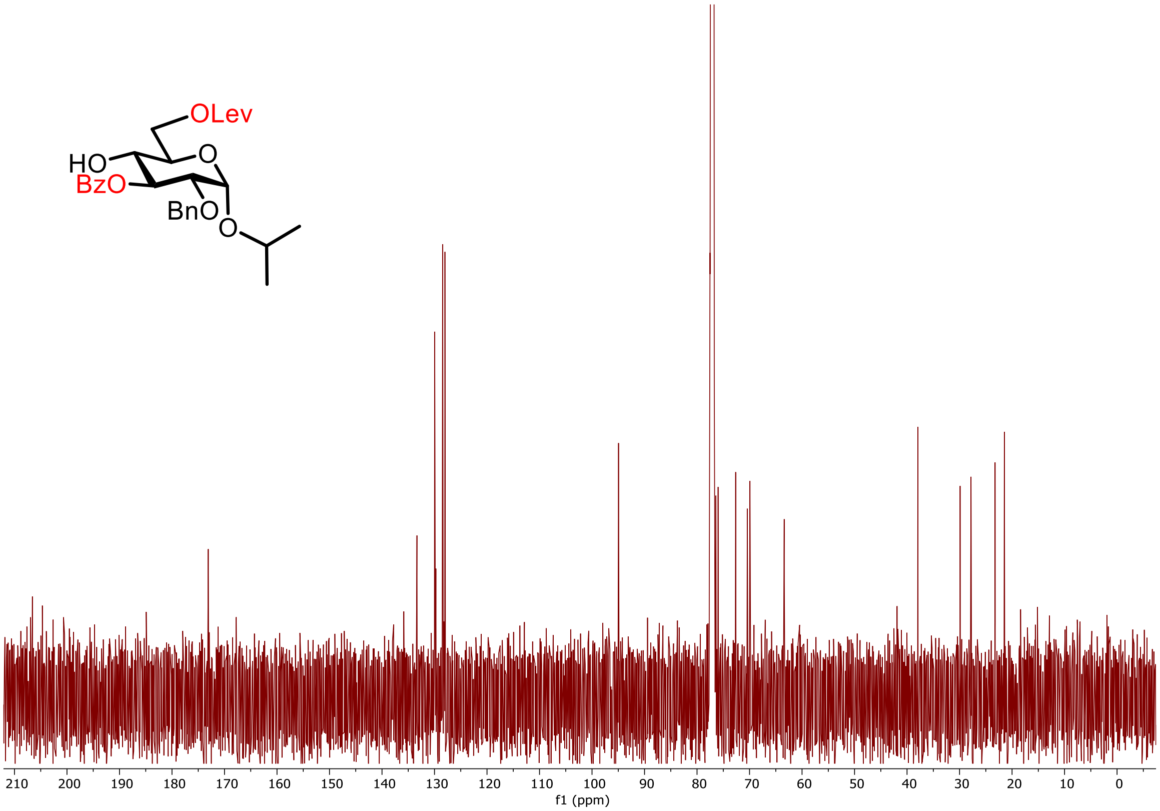

COSY NMR of 92a ($CDCl_3$)

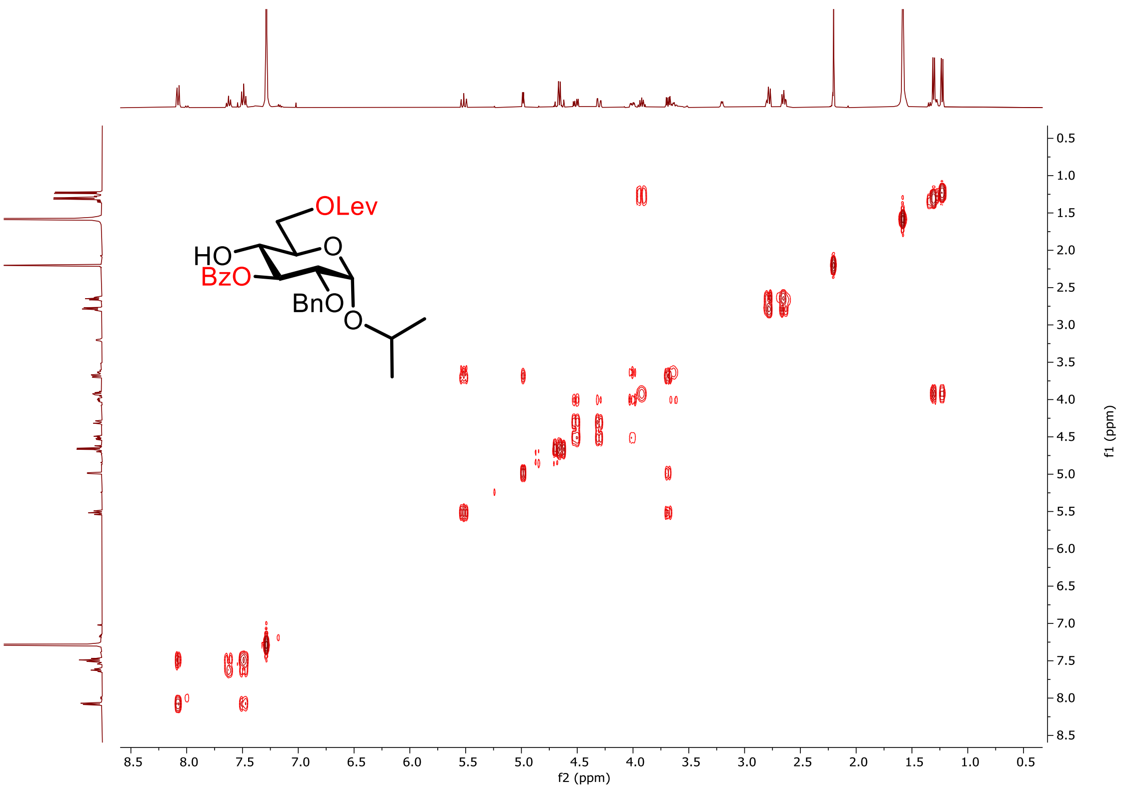

HSQC NMR of 92a ($CDCl_3$)

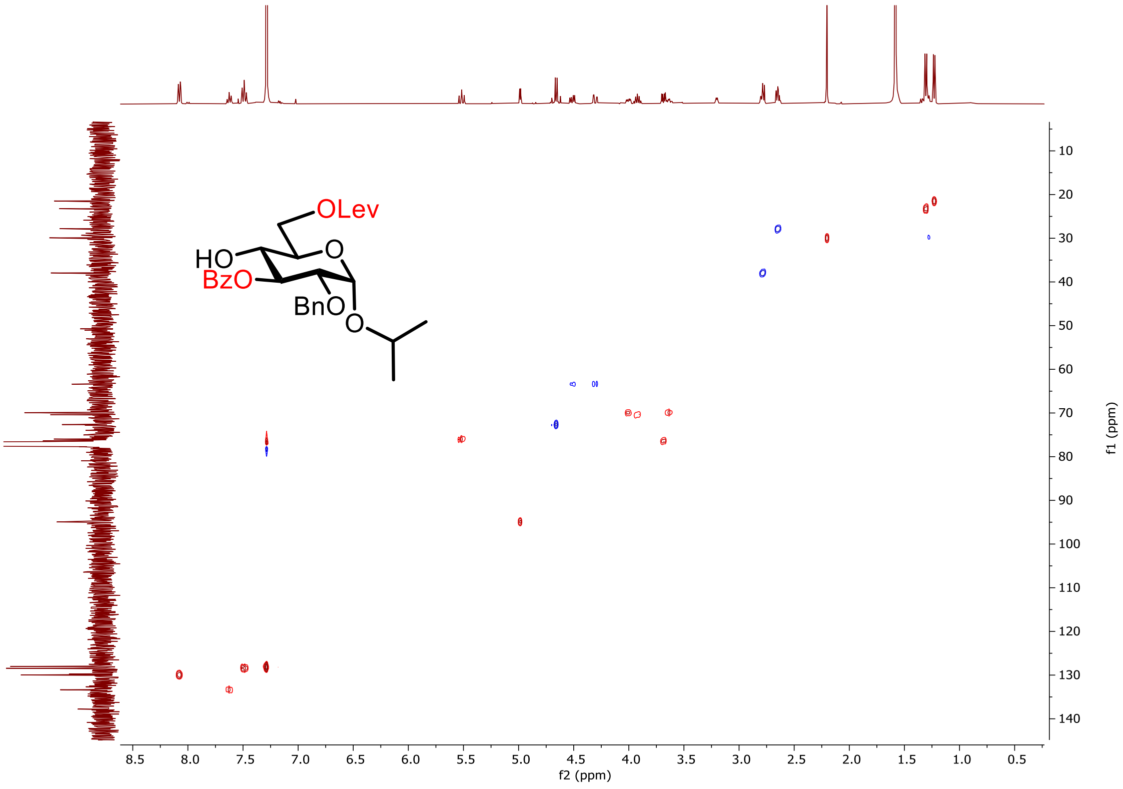

4.20 Isopropyl 2,3-di-*O*-benzyl-6-deoxy-6-fluoro-D-glucopyranoside, 93

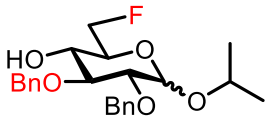

R_1=F; R_2=OBn

Total yield: 96% (38.8 mg). Ratio of anomer α : β = 1 : 1.1.

Spectrum data for isopropyl 2,3-di-*O*-benzyl-6-deoxy-6-fluoro-β-D-glucopyranoside **93b**: ^{1}H NMR (400 MHz, $CDCl_3$) δ 7.44 – 7.30 (m, 10H), 5.09 (d, *J* = 11.5 Hz, 1H), 4.93 (d, *J* = 3.6 Hz, 1H), 4.79 – 4.62 (m, 4H), 4.58 (dd, *J* = 10.1, 4.0 Hz, 0.5H), 4.53 (dd, *J* = 10.1, 2.0 Hz, 0.5H), 3.99 – 3.77 (m, 3H), 3.61 – 3.51 (m, 2H), 2.26 (br. s, 1H), 1.27 (d, *J* = 6.2 Hz, 3H), 1.22 (d, *J* = 6.1 Hz, 3H); ^{13}C NMR (151 MHz, $CDCl_3$) δ 138.80, 137.95, 128.69, 128.54, 128.20, 128.04, 127.95, 127.89, 94.85, 82.18 (d, *J* = 172.1 Hz), 81.44, 79.62, 75.36, 72.91, 69.93 (d, *J* = 17.7 Hz), 69.47, 68.96 (d, *J* = 7.0 Hz), 23.20, 21.21; ^{19}F NMR (564 MHz, $CDCl_3$) δ -235.48 (td, *J* = 48.4, 47.9, 26.7 Hz); $[\alpha]_D^{25}$ 31.80 (c = 1, $CHCl_3$); IR (neat) ν_{max} = 3486, 2926, 1455, 1058, 698 cm^{-1}; m/z (HRMS$^+$) $[M + Na]^+$ 427.1865 ($C_{23}H_{29}FO_5Na^+$ requires 427.1891).

^{1}H NMR of crude 93 (600 MHz, CDCl$_3$)

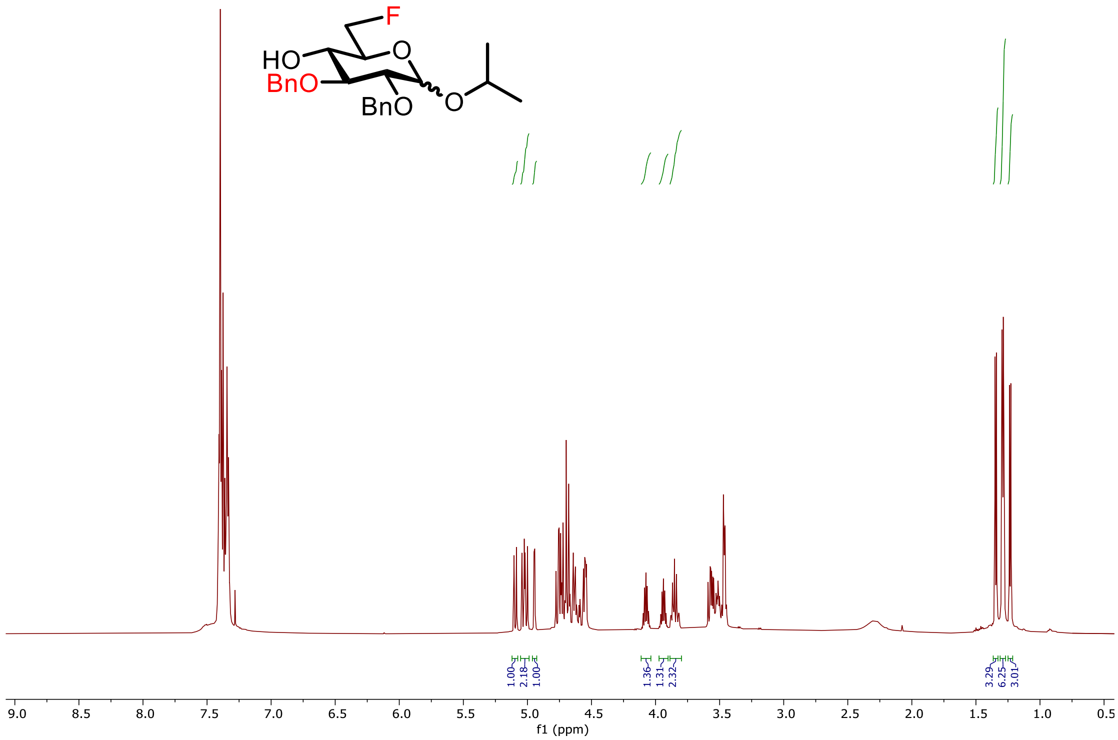

^{13}C NMR of crude 93 (151 MHz, CDCl$_3$)

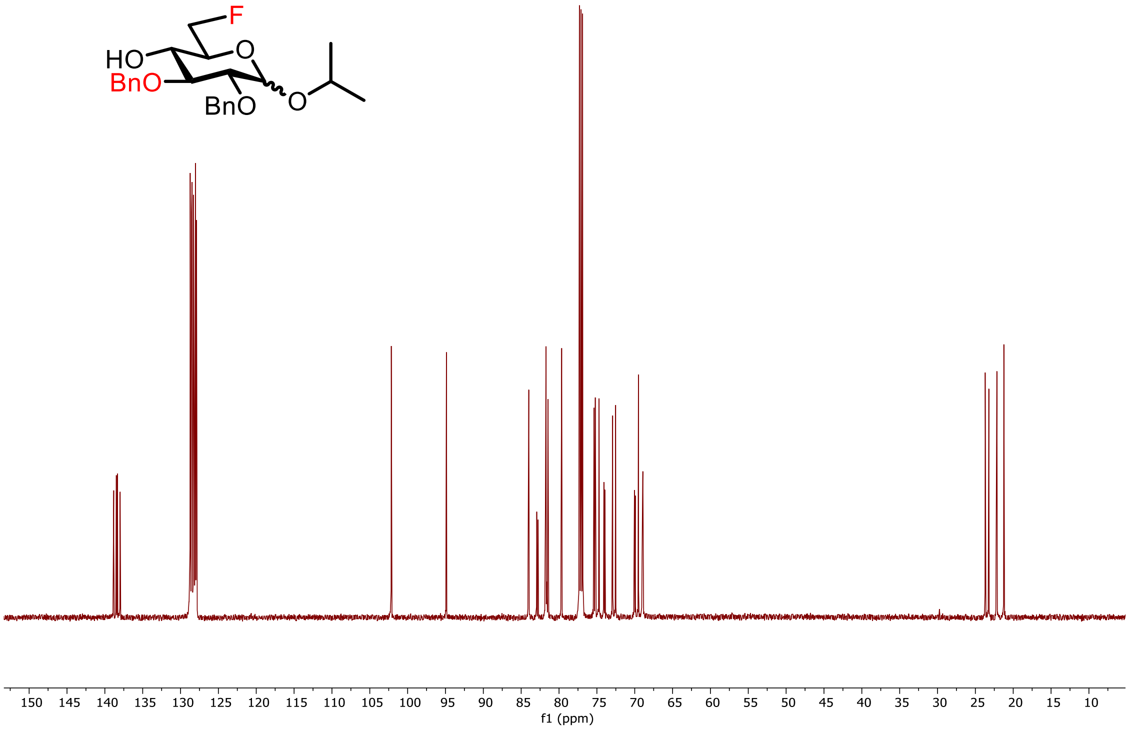

HSQC NMR of crude 93 ($CDCl_3$)

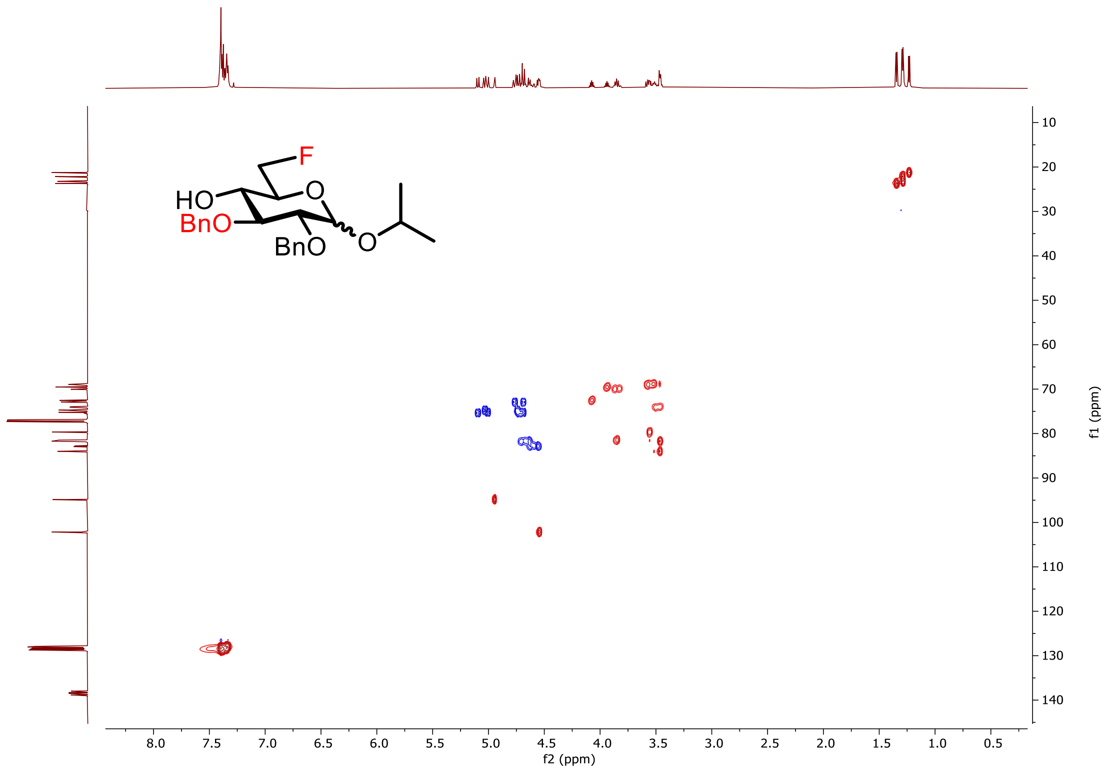

Coupled HSQC NMR of crude 93 ($CDCl_3$)

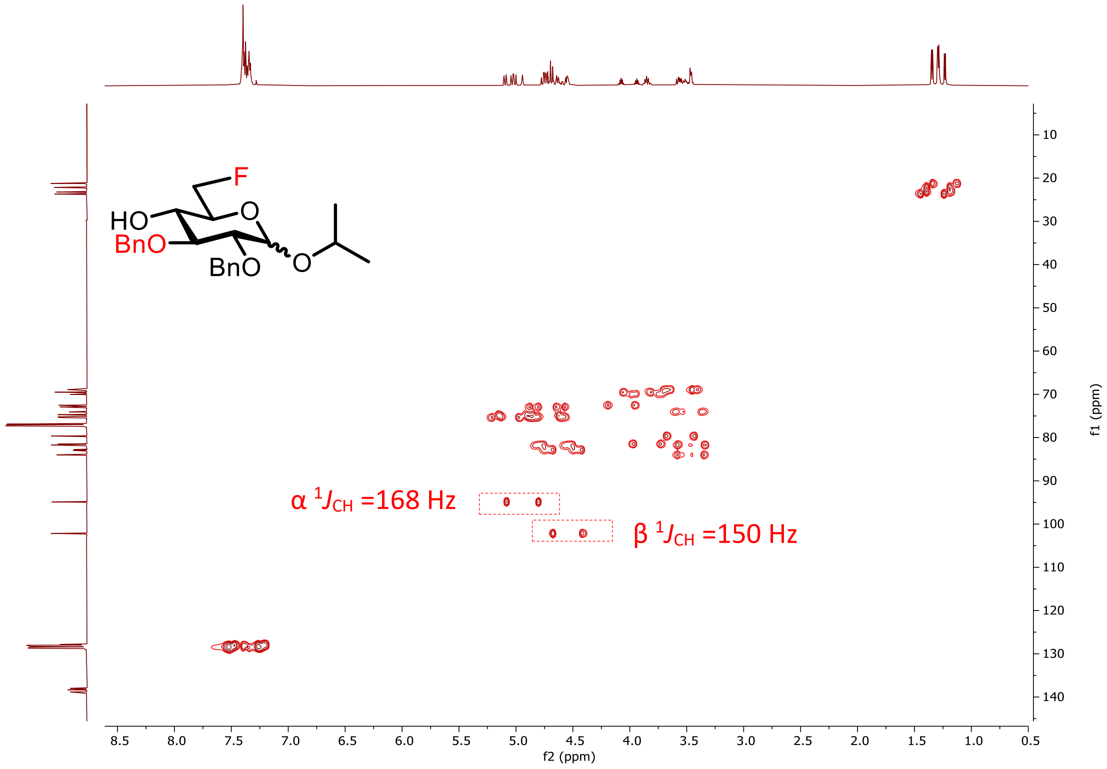

^{1}H NMR of 93b (400 MHz, CDCl$_3$)

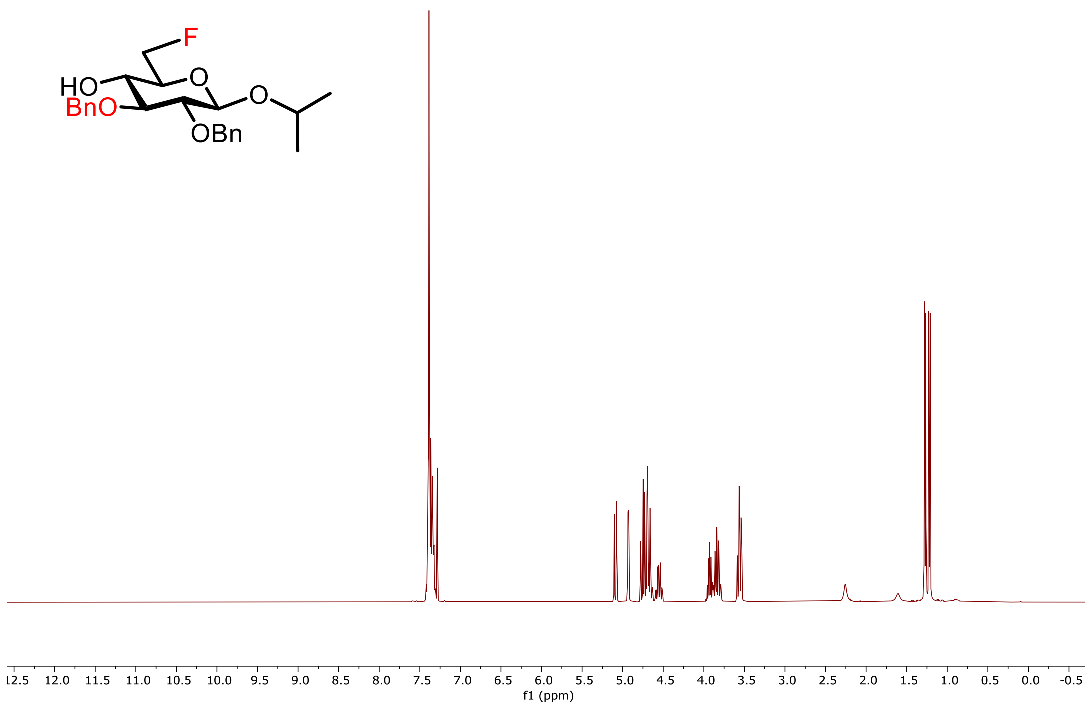

^{13}C NMR of 93b (151 MHz, CDCl$_3$)

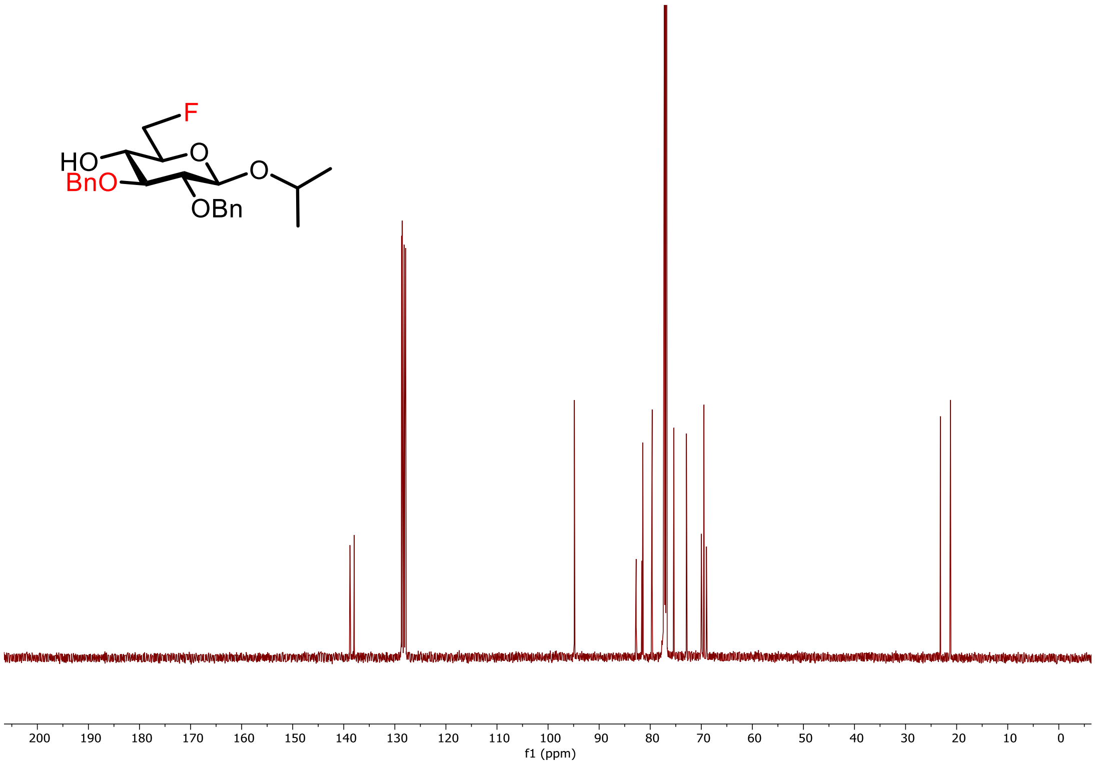

^{19}F NMR of 93b (564 MHz, CDCl$_3$)

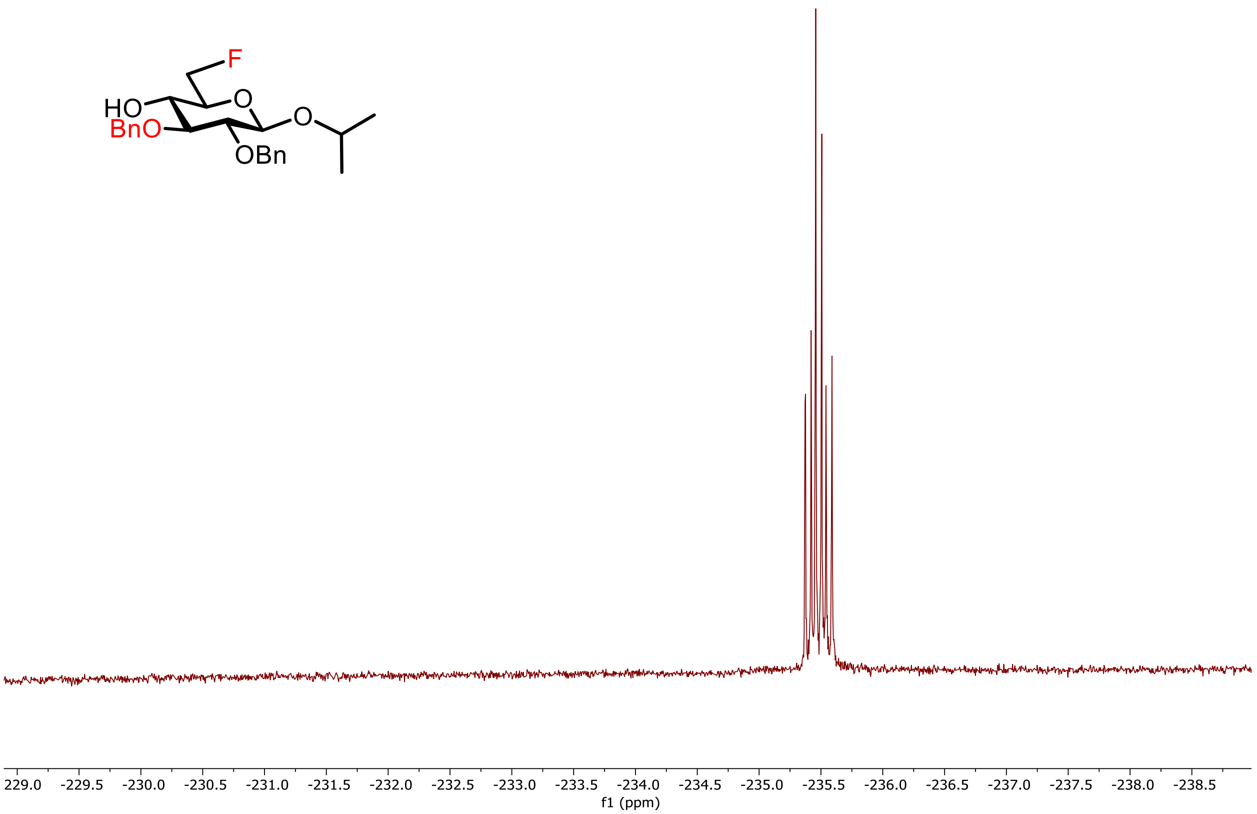

COSY NMR of 93b ($CDCl_3$)

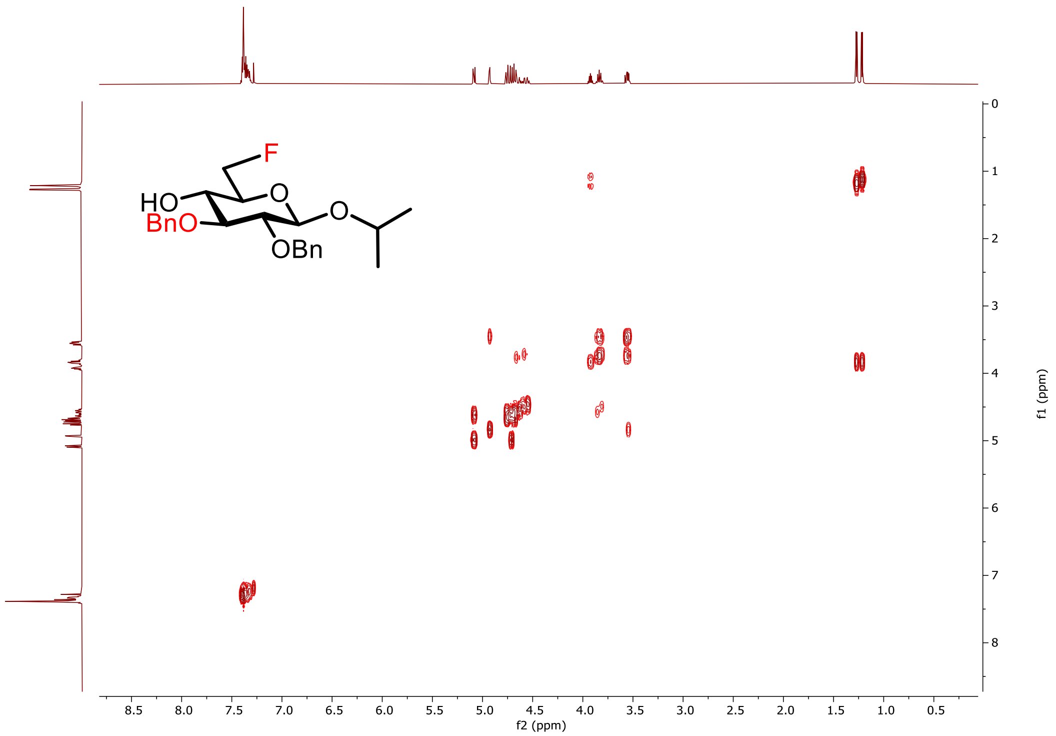

HSQC NMR of 93b ($CDCl_3$)

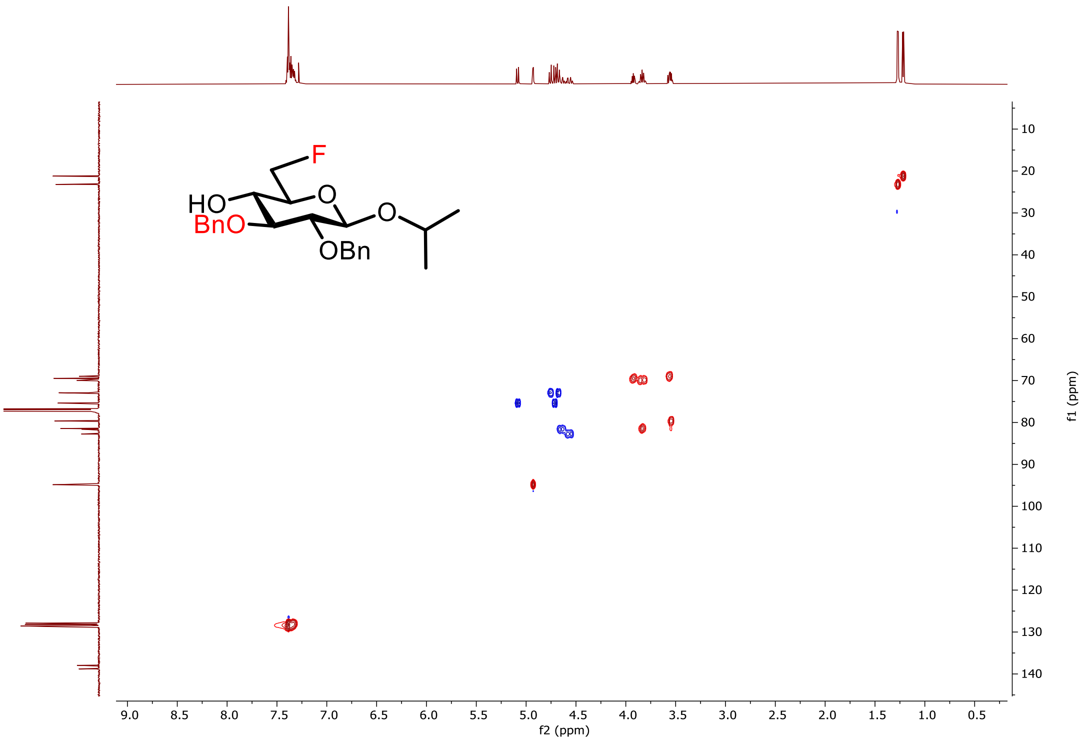

4.21 Isopropyl 2,3-di-*O*-benzyl-6-*O*-methyl-D-glucopyranoside, 94

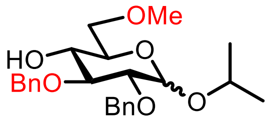

R$_1$=OMe; R$_2$=OBn

Total yield: 94% (39.1 mg). Ratio of anomer α : β = 1 : 2.6.

Spectrum data for isopropyl 2,3-di-*O*-benzyl-6-*O*-methyl-β-D-glucopyranoside **94b**: ^{1}H NMR (600 MHz, $CDCl_3$) δ 7.47 – 7.22 (m, 10H), 5.00 (d, *J* = 10.9 Hz, 1H), 4.96 (d, *J* = 11.5 Hz, 1H), 4.74 (d, *J* = 8.1 Hz, 1H), 4.72 (d, *J* = 7.5 Hz, 1H), 4.51 (d, *J* = 7.5 Hz, 1H), 4.04 (hept, *J* = 6.2 Hz, 1H), 3.69 (dd, *J* = 10.4, 3.9 Hz, 1H), 3.63 (dd, *J* = 10.4, 5.4 Hz, 1H), 3.57 (dd, *J* = 9.6, 8.5 Hz, 1H), 3.49 – 3.38 (m, 6H), 2.54 (br. s, 1H), 1.31 (d, *J* = 6.2 Hz, 3H), 1.26 (d, *J* = 6.1 Hz, 3H); ^{13}C NMR (151 MHz, $CDCl_3$) δ 138.70, 138.46, 128.58, 128.41, 128.25, 127.98, 127.86, 127.73, 102.11, 84.16, 81.71, 75.22, 74.76, 73.79, 72.90, 72.27, 71.50, 59.60, 23.70, 22.15; $[\alpha]_D^{25}$ -18.01 (c = 1, $CHCl_3$); IR (neat) ν_{max} = 3455, 2905, 1455, 1060, 698 cm^{-1}; m/z (HRMS$^+$) [M + Na]$^+$ 439.2070 ($C_{24}H_{32}O_6Na^+$ requires 439.2091).

^{1}H NMR of crude 94 (600 MHz, $CDCl_3$)

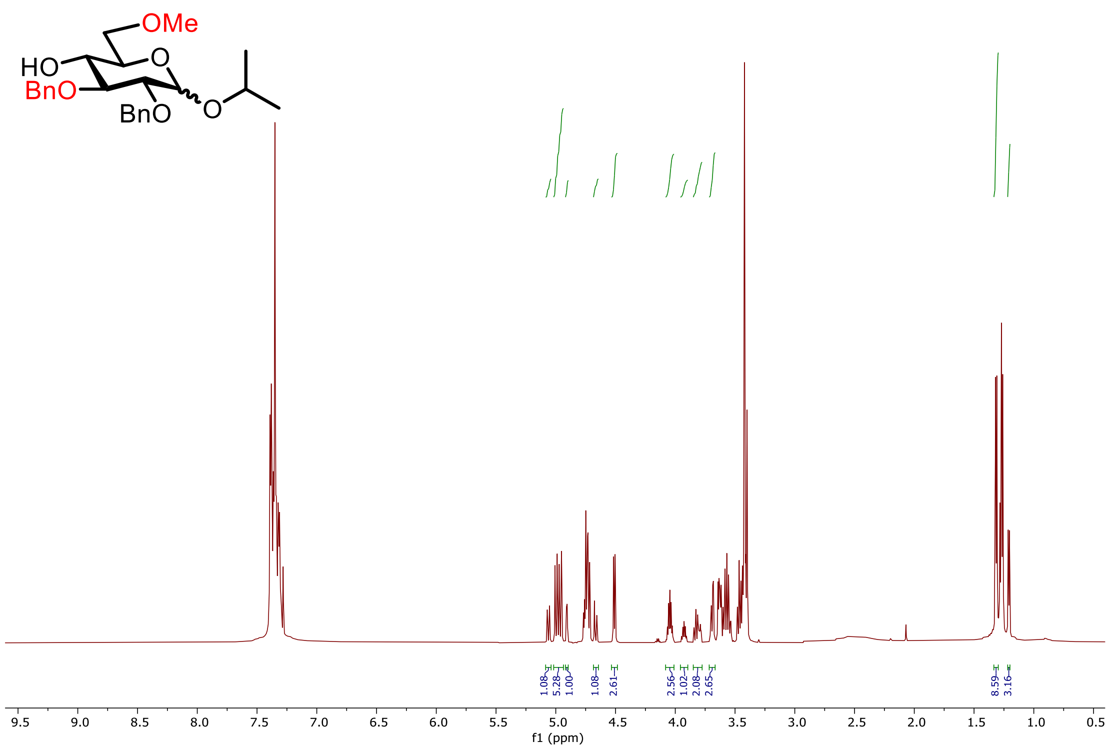

^{13}C NMR of crude 94 (151 MHz, $CDCl_3$)

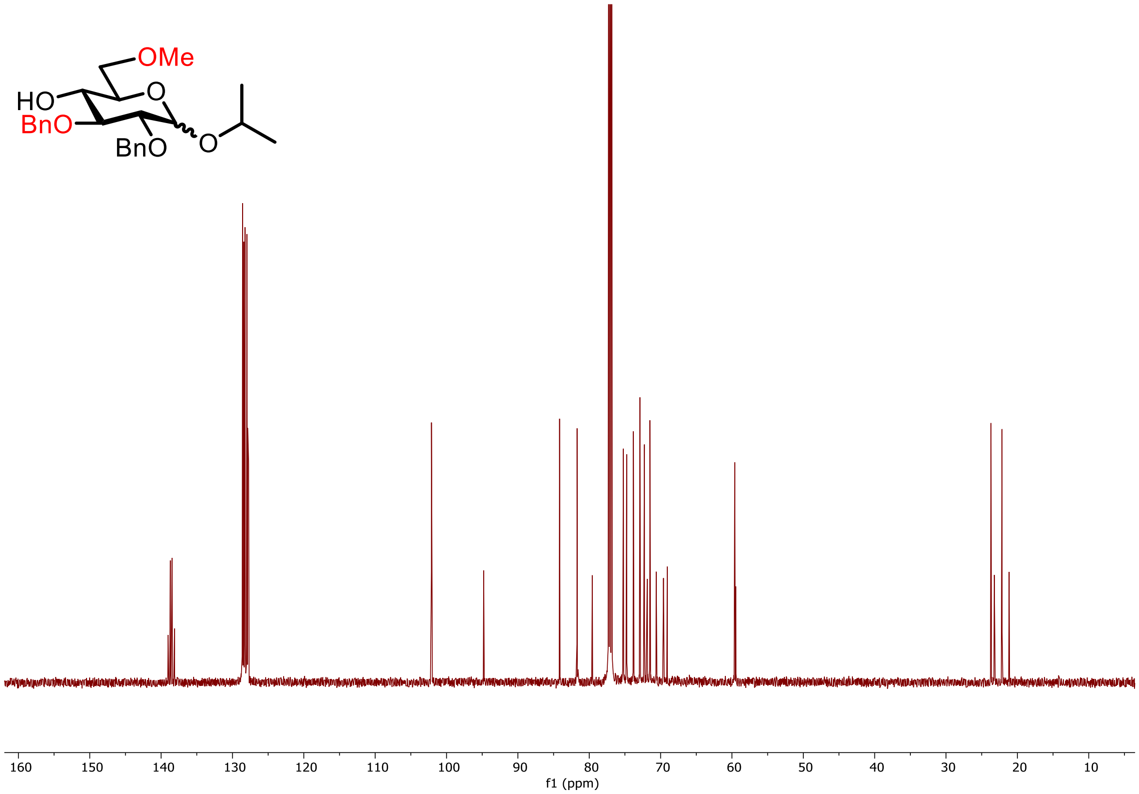

HSQC NMR of crude 94 ($CDCl_3$)

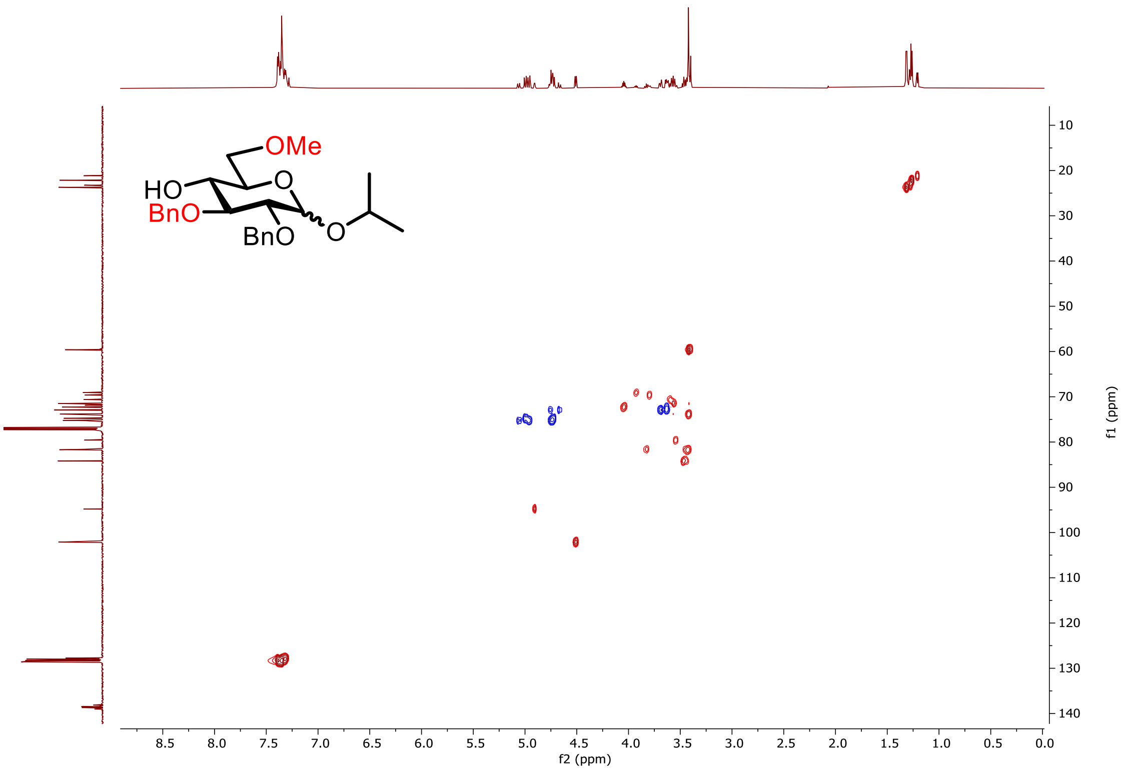

Coupled HSQC NMR of crude 94 ($CDCl_3$)

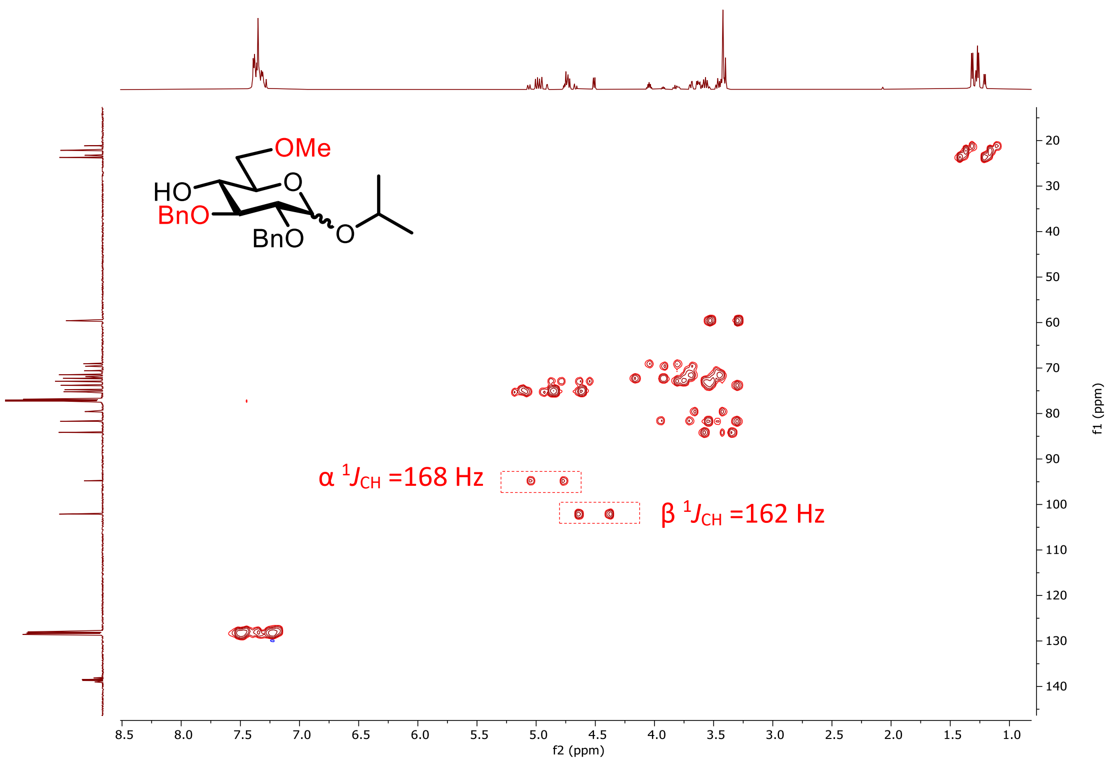

^{1}H NMR of 94b (400 MHz, $CDCl_3$)

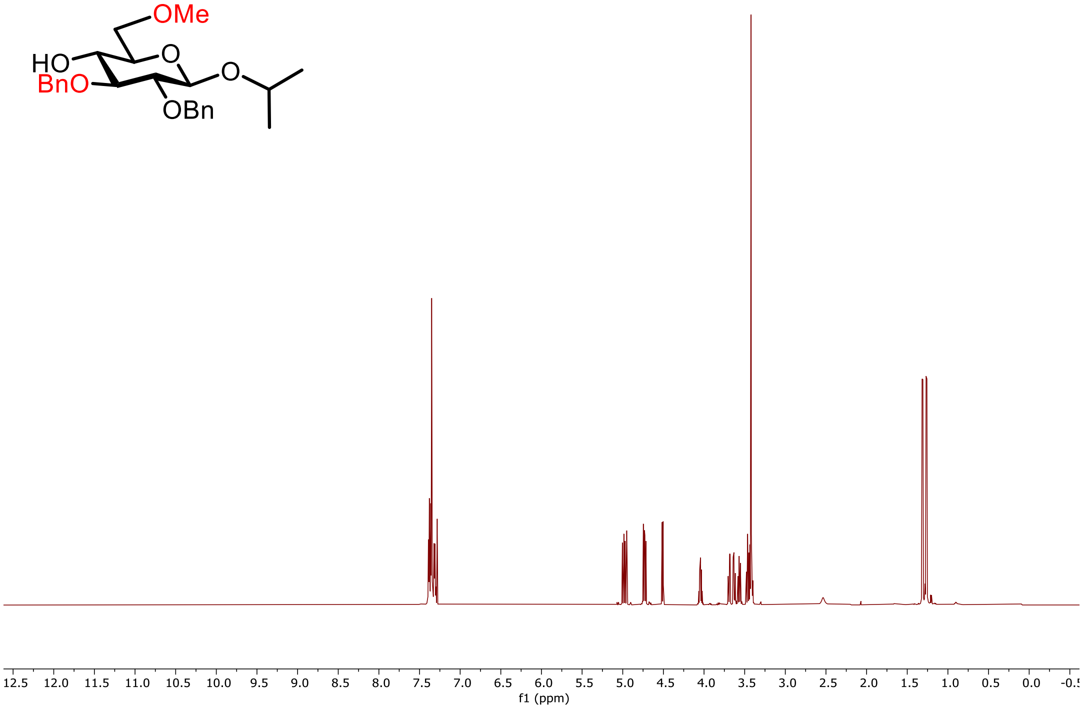

^{13}C NMR of 94b (151 MHz, $CDCl_3$)

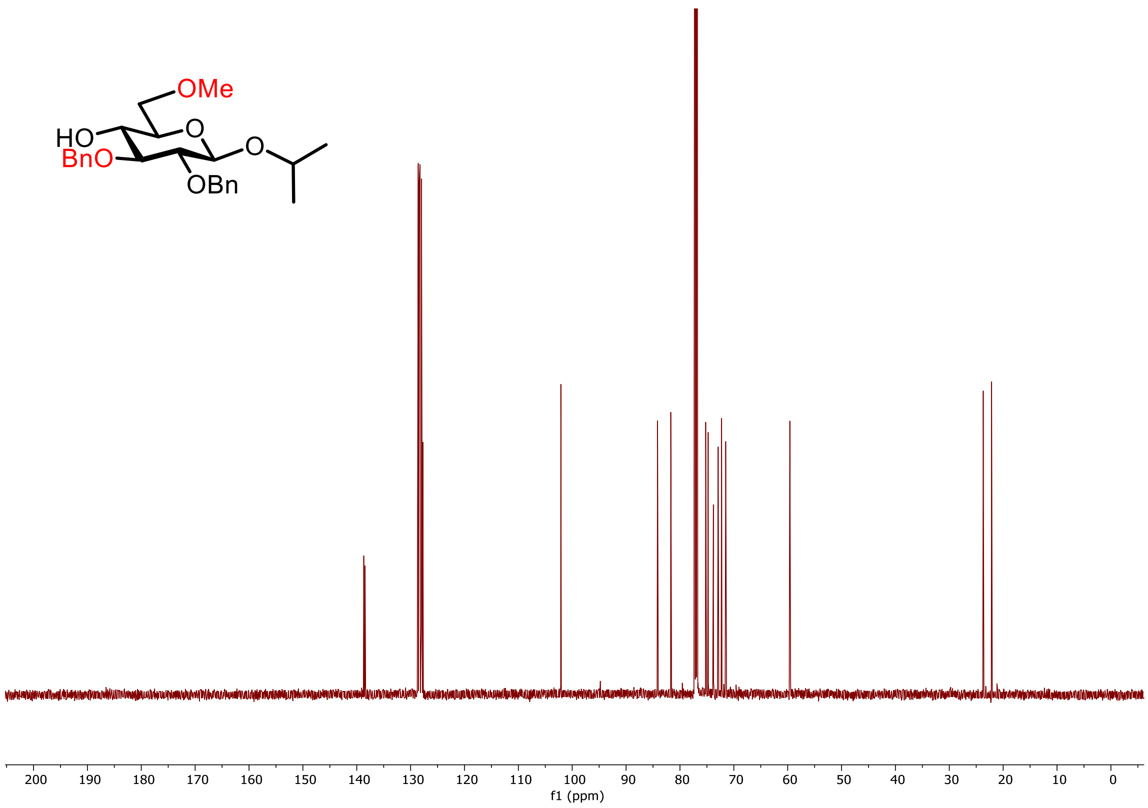

COSY NMR of 94b ($CDCl_3$)

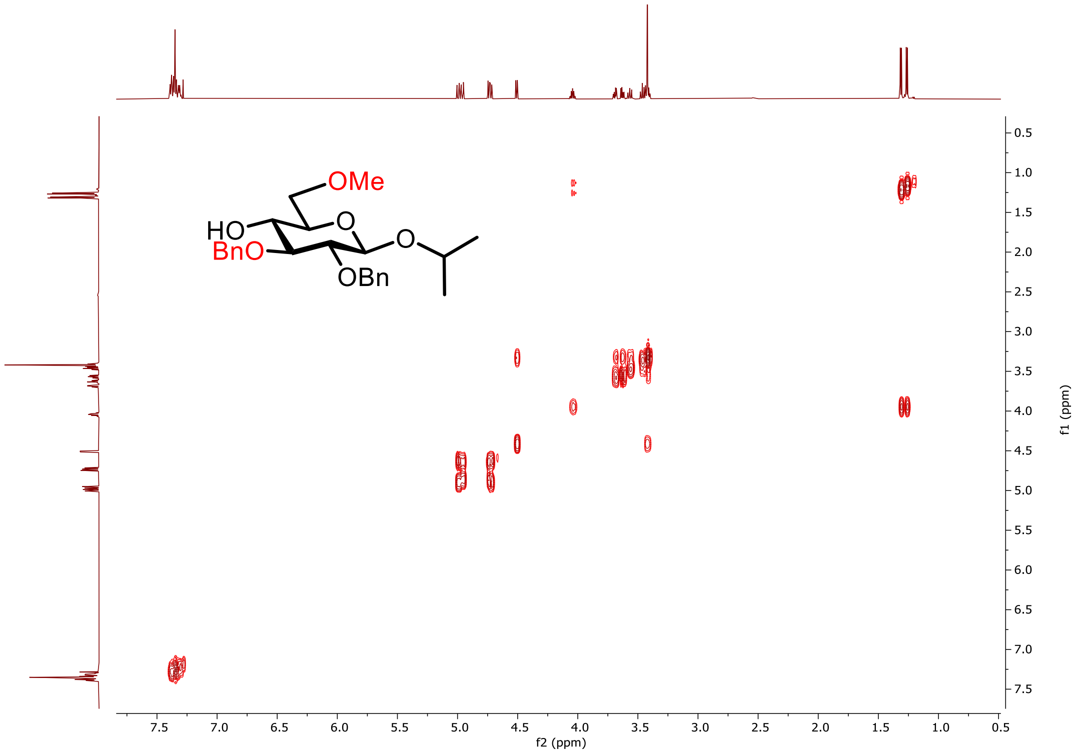

HSQC NMR of 94b ($CDCl_3$)

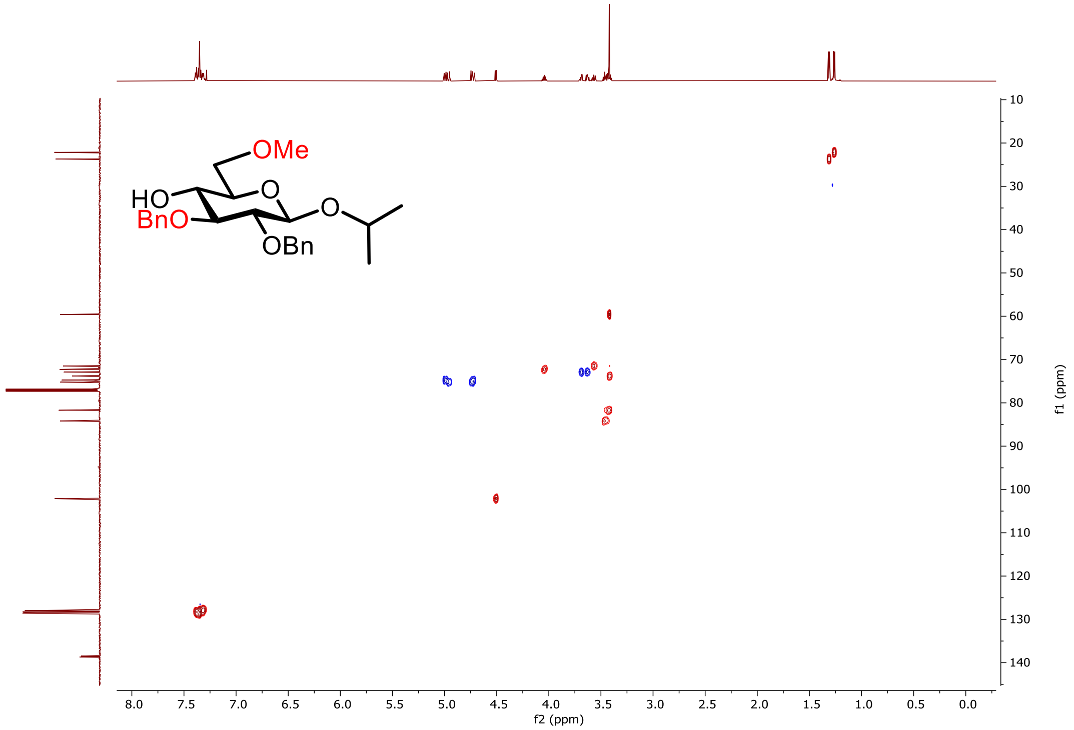

4.22 Benzyl 2,3,6-tri-*O*-benzyl-D-glucopyranosyl-(1→4)-2-*O*-benzoyl-3,6-di-*O*-benzyl-β-D-glucopyranoside, 95

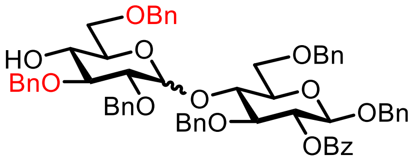

R_1=R_2=OBn

Total yield: 83% (81.9 mg). Ratio of anomer α : β = 1.5 : 1.

Spectrum data for benzyl 2,3,6-tri-*O*-benzyl-α-D-glucopyranosyl-(1→4)-2-*O*-benzoyl-3,6-di-*O*-benzyl-β-D-glucopyranoside **95a**: ^{1}H NMR (400 MHz, $CDCl_3$) δ 8.01 (d, *J* = 7.7 Hz, 2H), 7.61 (appt, *J* = 7.4 Hz, 1H), 7.47 (appt, *J* = 7.6 Hz, 2H), 7.42 – 7.28 (m, 15H), 7.23 (d, *J* = 5.3 Hz, 10H), 7.17 – 7.09 (m, 5H), 5.58 (d, *J* = 3.6 Hz, 1H), 5.50 (appt, *J* = 8.2 Hz, 1H), 4.93 (appt, *J* = 11.0 Hz, 2H), 4.79 – 4.51 (m, 10H), 4.44 (d, *J* = 12.1 Hz, 1H), 4.29 (appt, *J* = 8.8 Hz, 1H), 4.02 (appt, *J* = 8.6 Hz, 1H), 3.96 (dd, *J* = 11.1, 4.4 Hz, 1H), 3.90 – 3.74 (m, 3H), 3.73 – 3.65 (m, 2H), 3.61 (dd, *J* = 10.3, 4.1 Hz, 1H), 3.54 (dd, *J* = 10.3, 3.8 Hz, 1H), 3.49 (dd, *J* = 9.6, 3.6 Hz, 1H), 2.53 (br. s, 1H); ^{13}C NMR (101 MHz, $CDCl_3$) δ 165.25, 138.72, 138.36, 137.94, 137.83, 137.16, 133.16, 129.90, 129.87, 128.56, 128.43, 128.37, 128.32, 128.23, 127.95, 127.92, 127.82, 127.78, 127.74, 127.71, 127.69, 127.61, 127.55, 127.43, 127.40, 99.16, 96.78, 82.98, 81.15, 78.92, 75.34, 75.00, 73.59, 73.38, 73.07, 72.89, 72.85, 72.60, 71.23, 70.70, 69.99, 69.67, 69.04; $[\alpha]_D^{25}$ 19.52 (c = 1, $CHCl_3$); IR (neat) ν_{max} = 2867, 1729, 1453, 1055, 695 cm^{-1}; m/z (HRMS$^+$) $[M + Na]^+$ 1009.431 ($C_{61}H_{62}O_{12}Na^+$ requires 1009.413).

^{1}H NMR of crude 95 (400 MHz, $CDCl_3$)

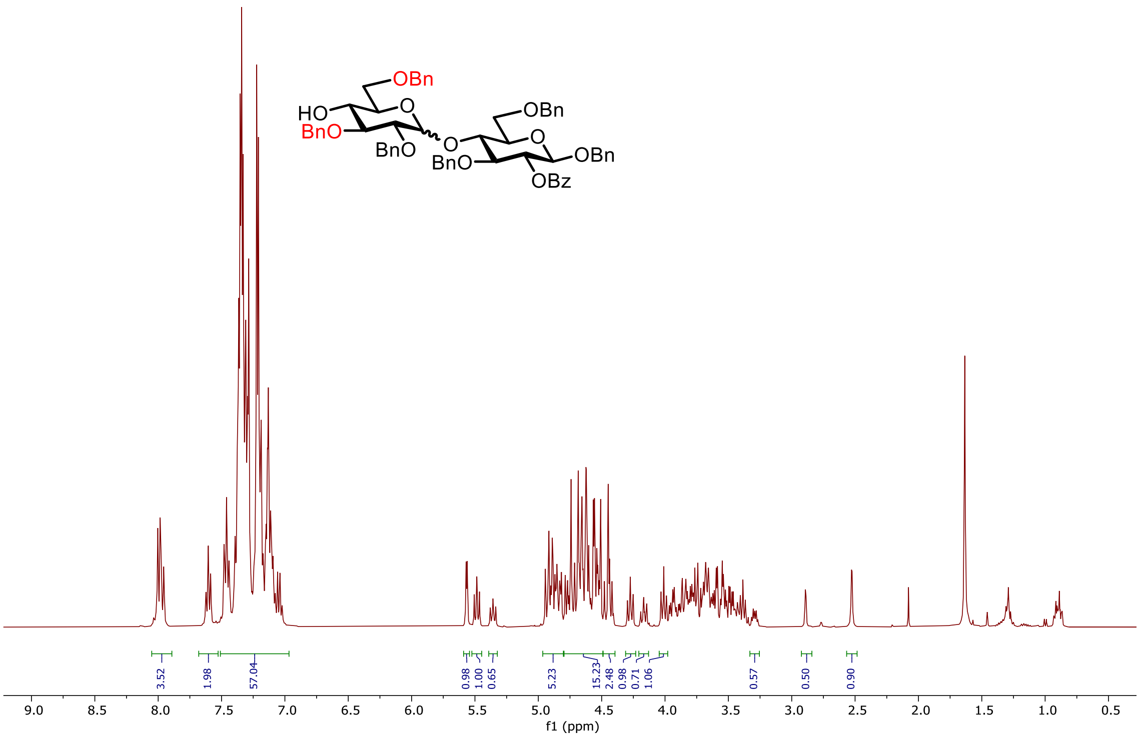

^{13}C NMR of crude 95 (101 MHz, $CDCl_3$)

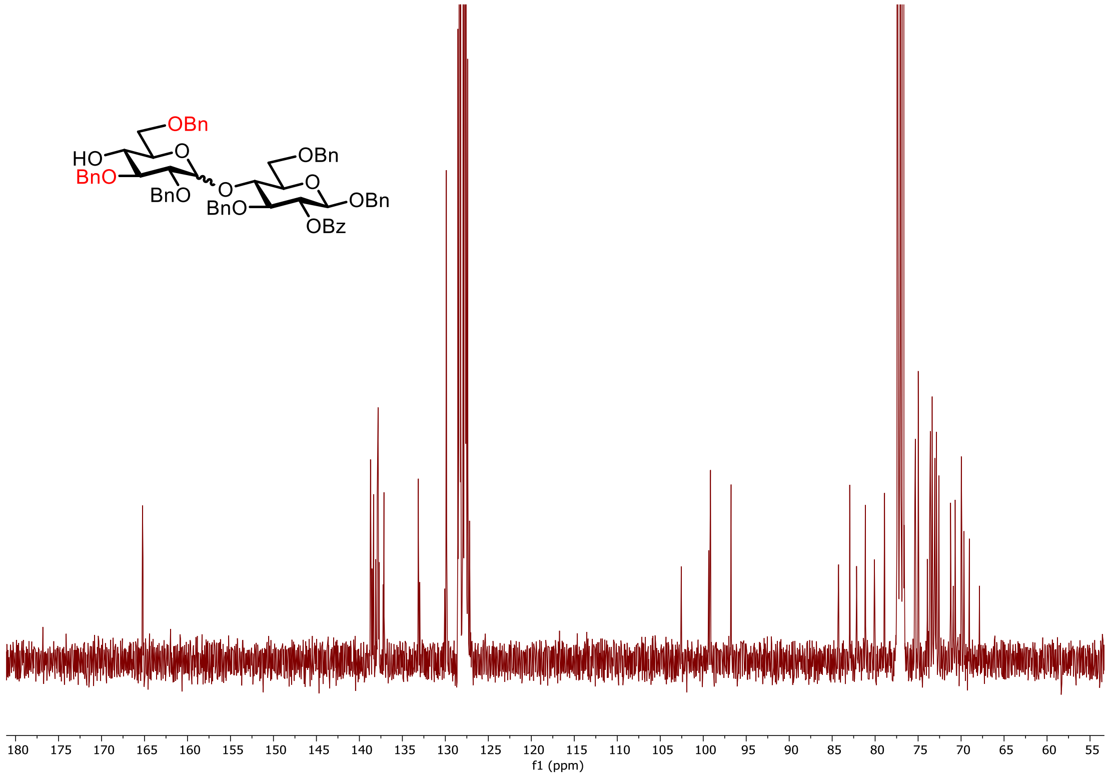

HSQC NMR of crude 95 ($CDCl_3$)

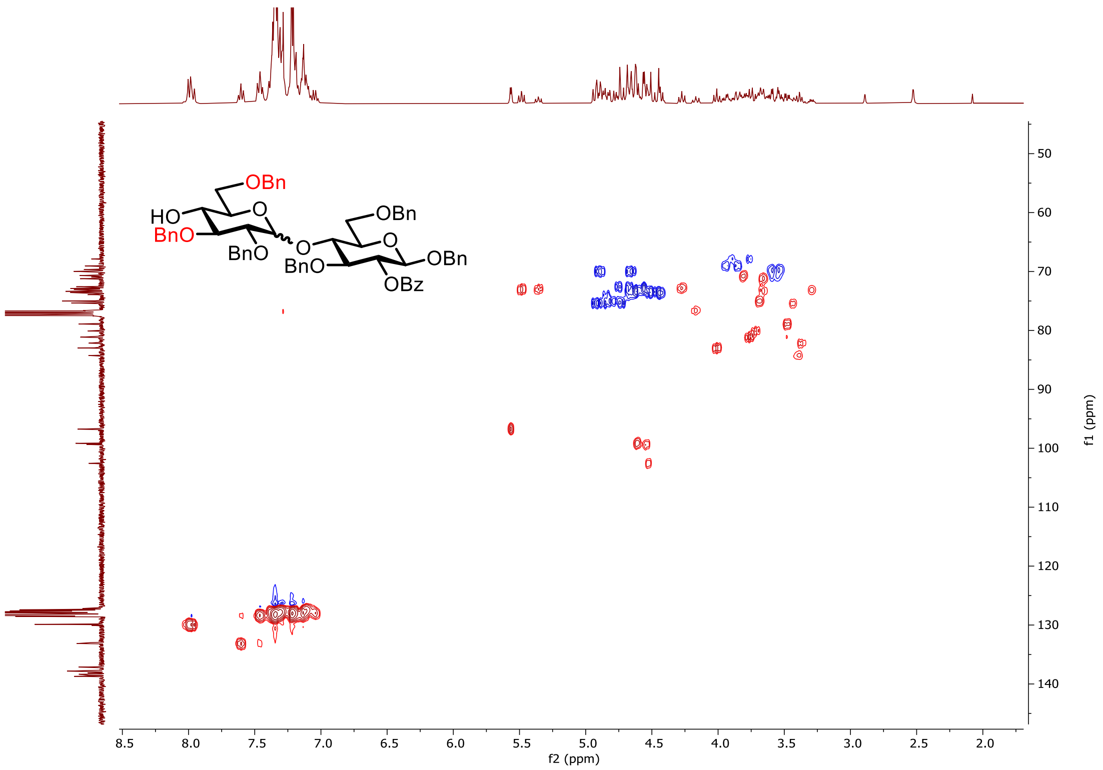

Coupled HSQC NMR of crude 95 ($CDCl_3$)

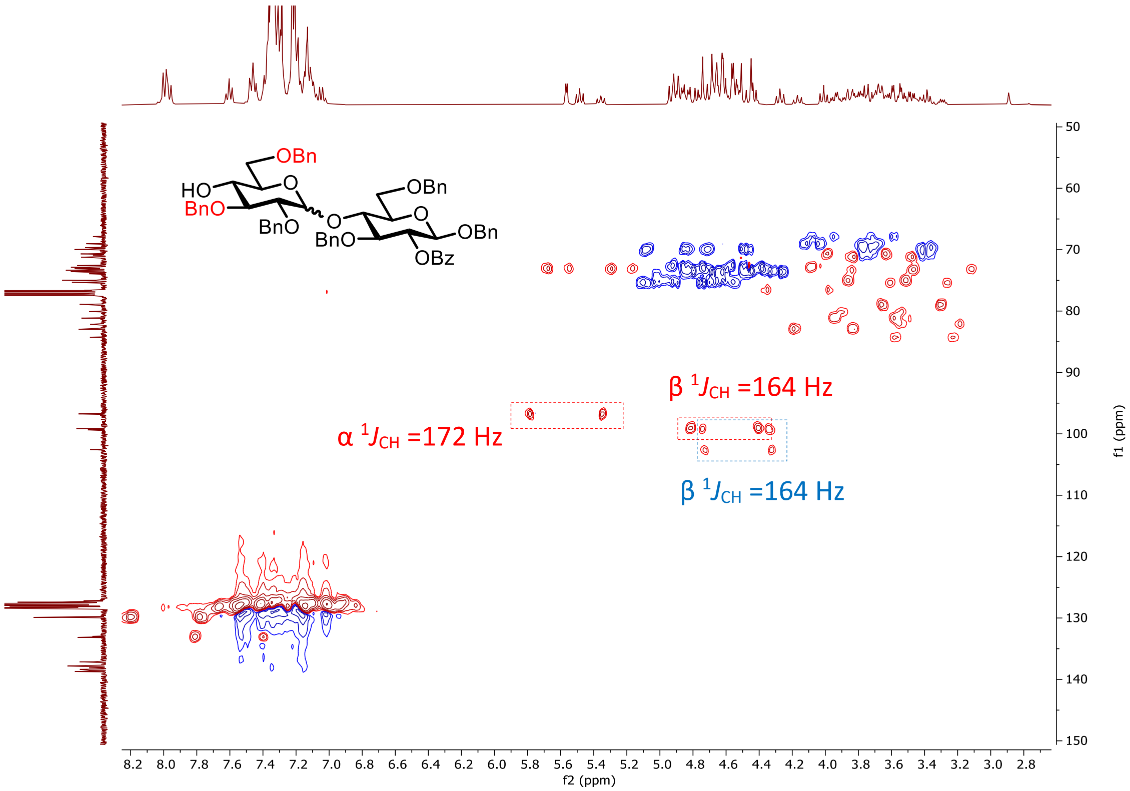

^{1}H NMR of 95a (400 MHz, $CDCl_3$)

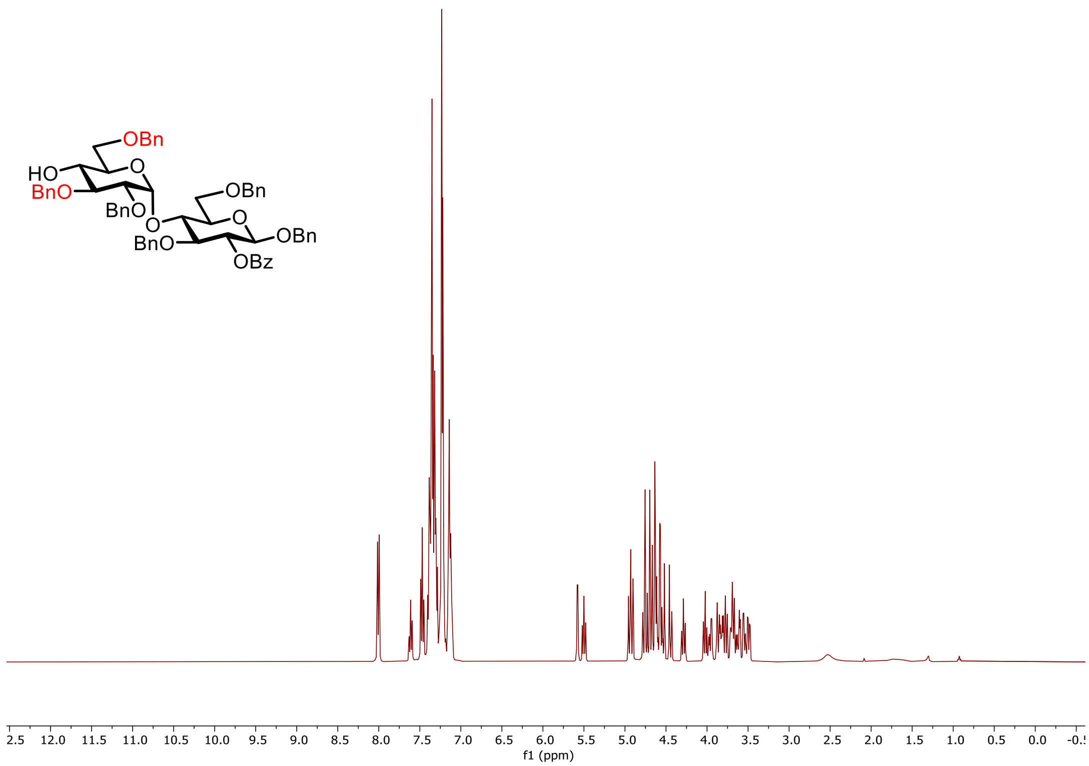

^{13}C NMR of 95a (101 MHz, $CDCl_3$)

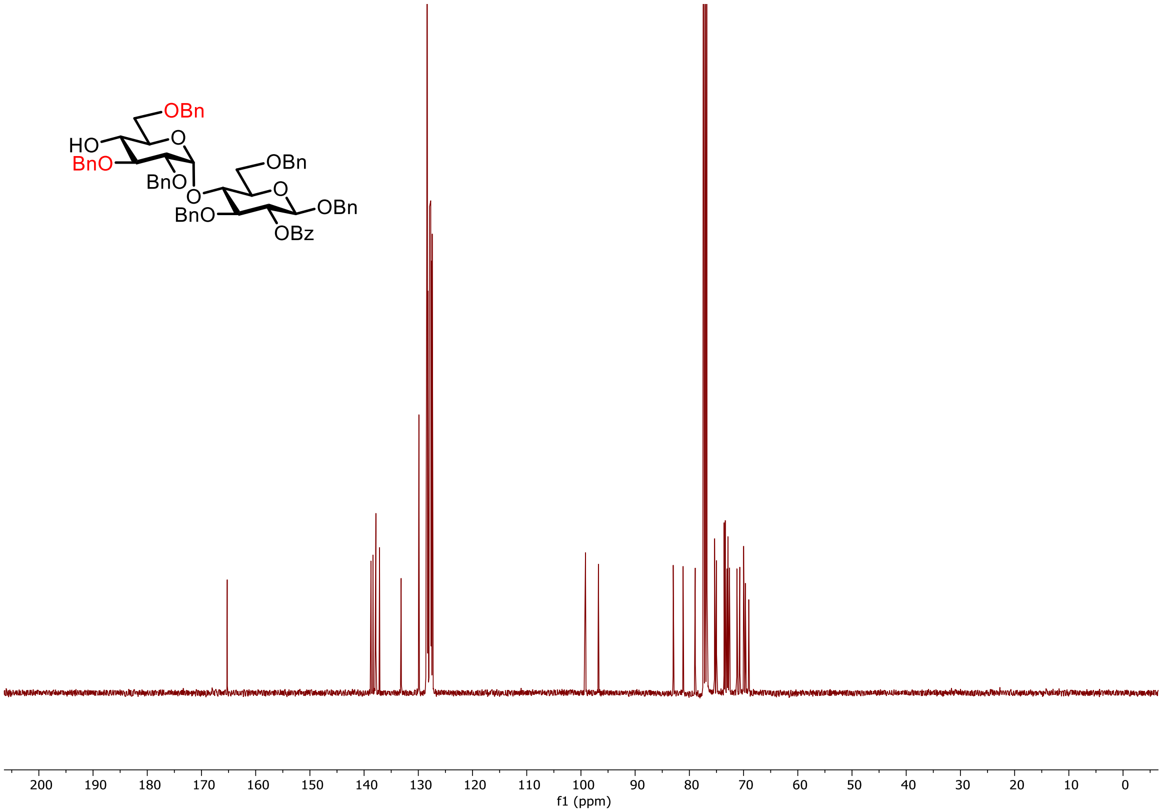

COSY NMR of 95a ($CDCl_3$)

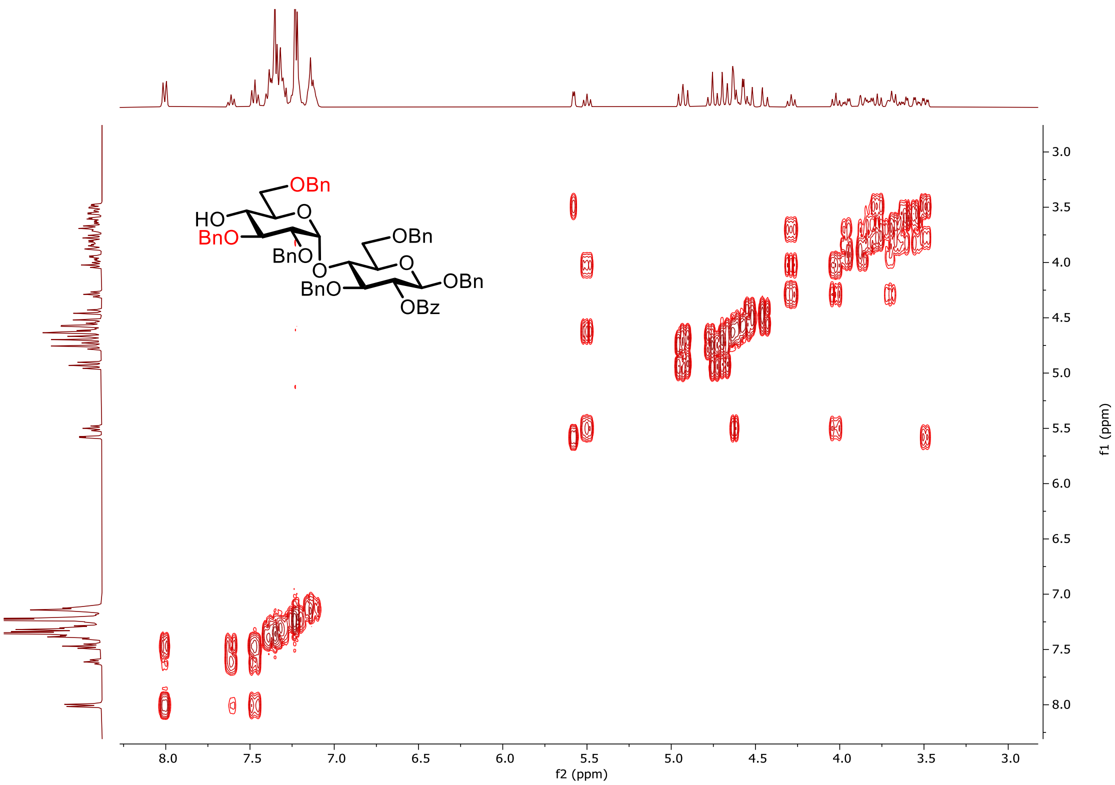

HSQC NMR of 95a ($CDCl_3$)

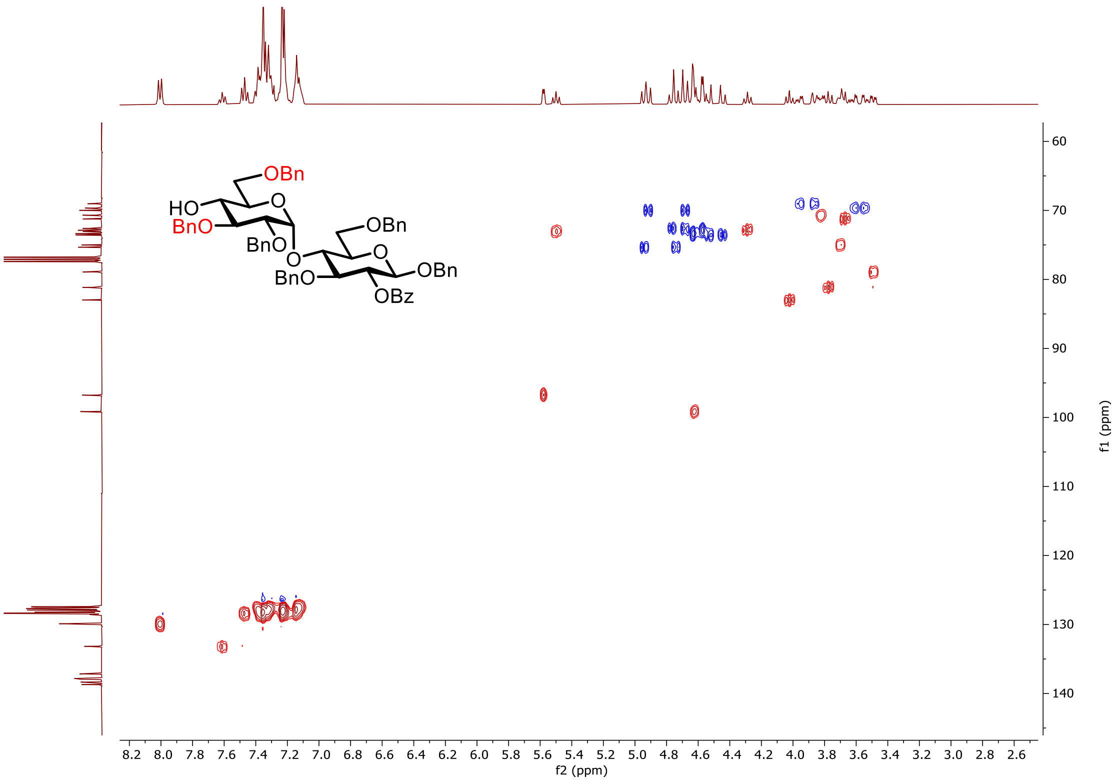

4.23 Benzyl 2,3-di-*O*-benzyl-6-*O*-acetyl-D-glucopyranosyl-(1→4)-2-*O*-benzoyl-3,6-di-*O*-benzyl-β-D-glucopyranoside, 96

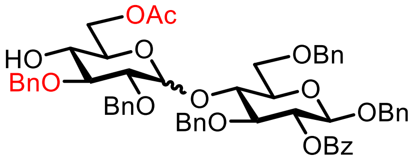

Total yield: 75% (70.4 mg). Ratio of anomer α : β = 3.8 : 1.

Spectrum data for benzyl 2,3-di-*O*-benzyl-6-*O*-acetyl-α-D-glucopyranosyl-(1→4)-2-*O*-benzoyl-3,6-di-*O*-benzyl-β-D-glucopyranoside **96a**: ^{1}H NMR (400 MHz, $CDCl_3$) δ 8.02 – 7.97 (m, 2H), 7.66 – 7.57 (m, 1H), 7.51 – 7.30 (m, 12H), 7.28 – 7.20 (m, 10H), 7.17 – 7.09 (m, 5H), 5.57 (d, *J* = 3.6 Hz, 1H), 5.50 (dd, *J* = 8.8, 7.6 Hz, 1H), 4.96 – 4.90 (m, 2H), 4.76 (appt, *J* = 10.6 Hz, 2H), 4.72 – 4.65 (m, 4H), 4.64 (d, *J* = 7.5 Hz, 1H), 4.62 – 4.54 (m, 2H), 4.40 (dd, *J* = 12.2, 4.3 Hz, 1H), 4.27 (dd, *J* = 9.3, 8.4 Hz, 1H), 4.10 – 3.98 (m, 2H), 3.94 (dd, *J* = 11.2, 4.4 Hz, 1H), 3.91 – 3.83 (m, 2H), 3.78 (appt, *J* = 9.3 Hz, 1H), 3.70 (ddd, *J* = 9.5, 4.4, 2.2 Hz, 1H), 3.46 (dd, *J* = 9.8, 3.7 Hz, 1H), 3.41 (dd, *J* = 10.0, 8.8 Hz, 1H), 2.67 (br. s, 1H), 2.08 (s, 3H); ^{13}C NMR (101 MHz, $CDCl_3$) δ 171.73, 165.25, 138.51, 138.15, 137.82, 137.77, 137.11, 133.19, 129.90, 129.82, 128.61, 128.46, 128.41, 128.38, 128.34, 128.23, 128.03, 127.94, 127.92, 127.80, 127.77, 127.73, 127.71, 127.41, 127.39, 99.19, 96.78, 82.85, 80.78, 78.90, 75.55, 74.97, 73.53, 73.05, 73.02, 72.94, 72.58, 70.44, 70.04, 69.90, 68.90, 63.24, 20.95; $[\alpha]_D^{25}$ 16.02 (c = 1, $CHCl_3$); IR (neat) ν_{max} = 3503, 2874, 1732, 1454, 1268, 1060, 698 cm^{-1}; m/z (HRMS$^+$) [M + Na]$^+$ 961.3948 ($C_{56}H_{58}O_{13}Na^+$ requires 961.3770).

^{1}H NMR of crude 96 (600 MHz, $CDCl_3$)

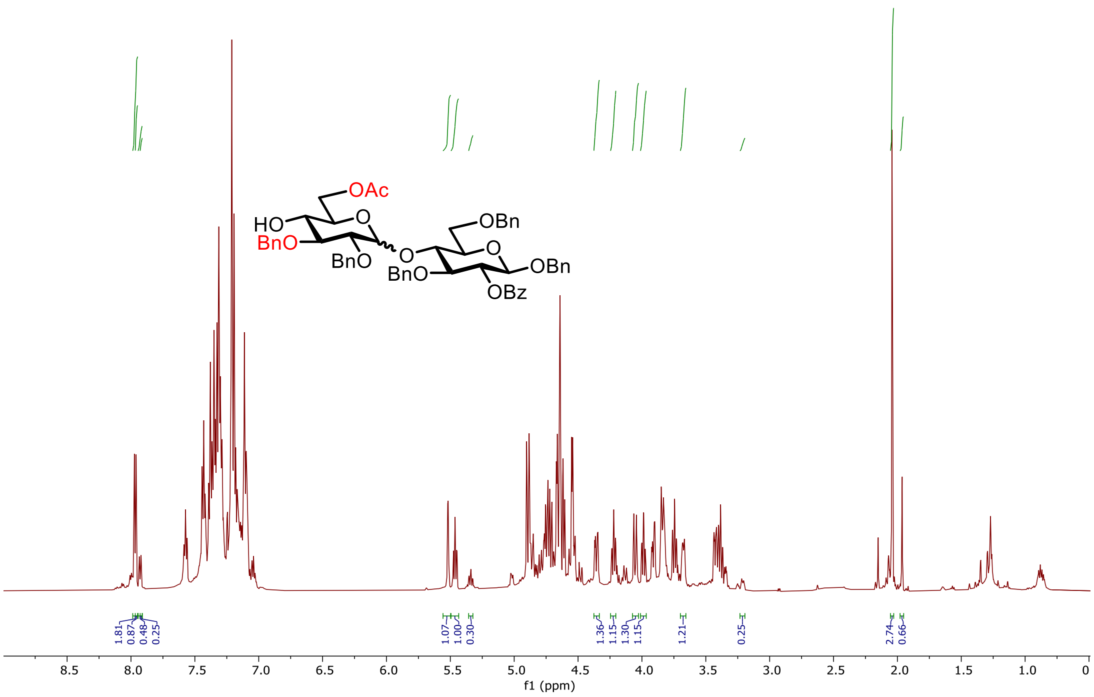

^{13}C NMR of crude 96 (151 MHz, $CDCl_3$)

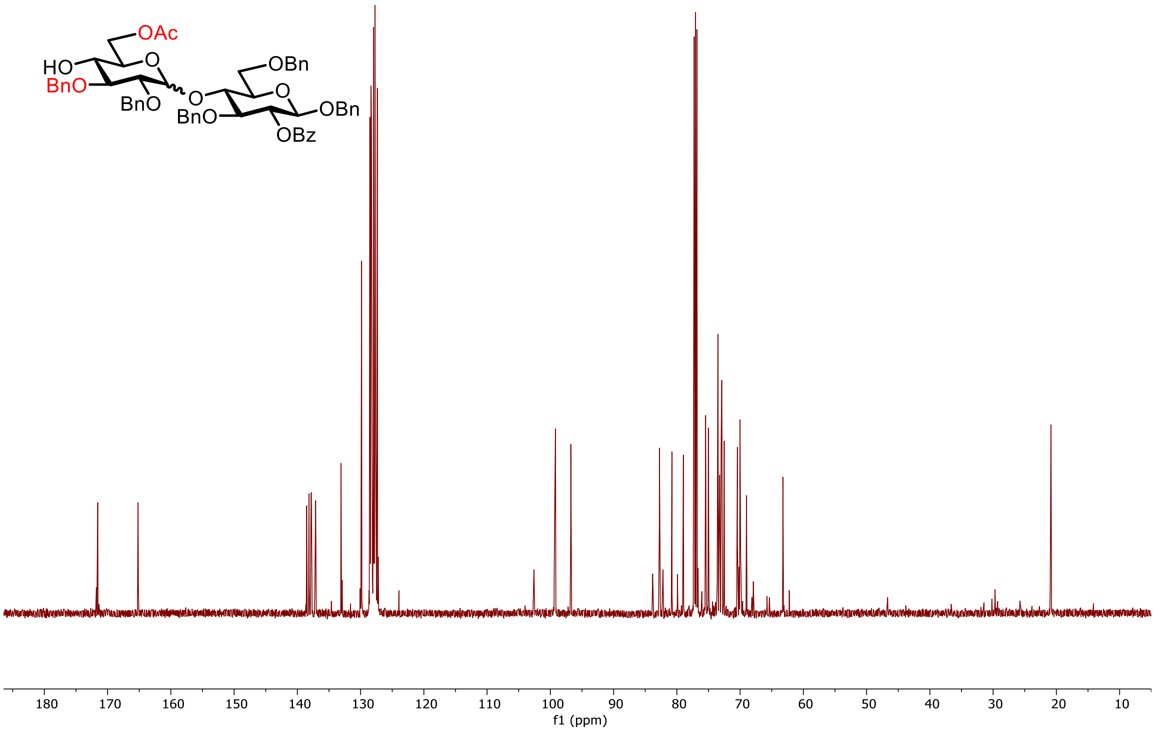

HSQC NMR of crude 96 ($CDCl_3$)

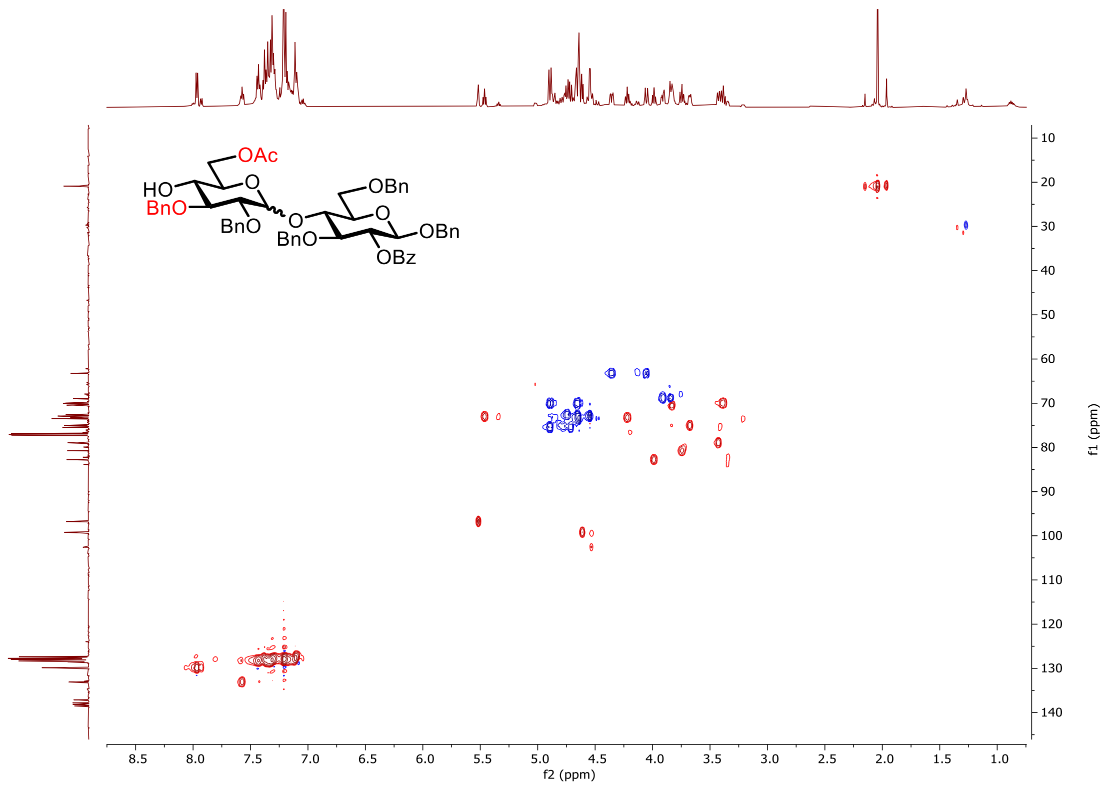

Coupled HSQC NMR of crude 96 ($CDCl_3$)

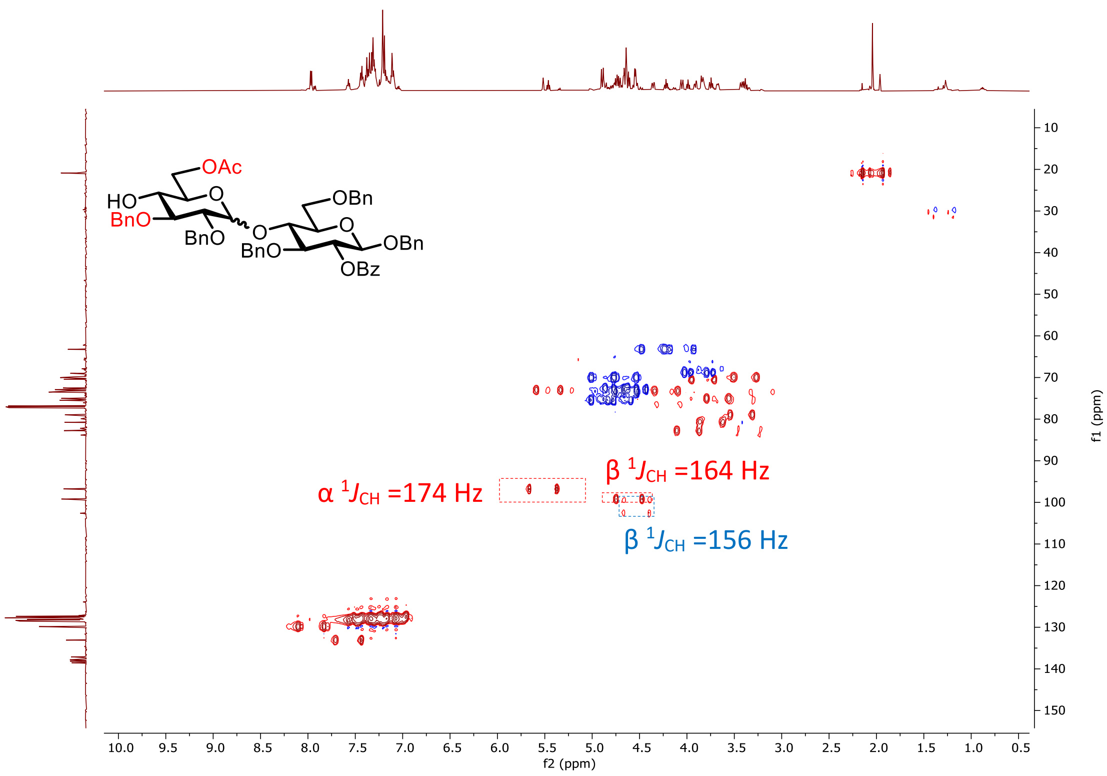

^{1}H NMR of 96a (400 MHz, $CDCl_3$)

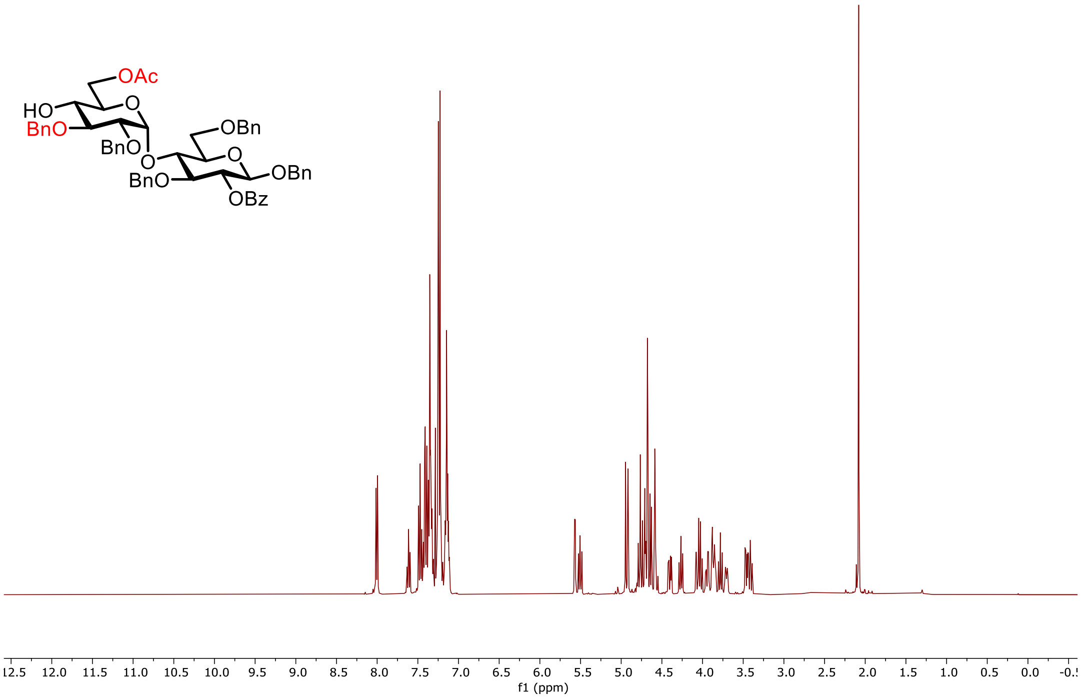

^{13}C NMR of 96a (101 MHz, $CDCl_3$)

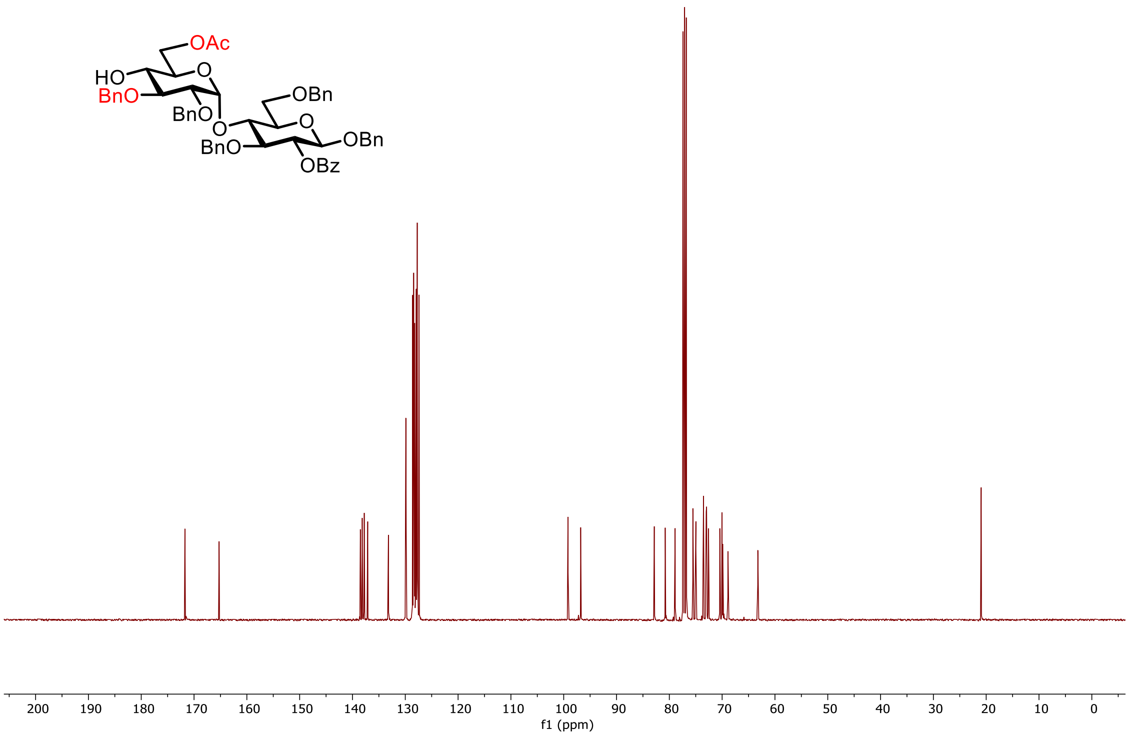

COSY NMR of 96a ($CDCl_3$)

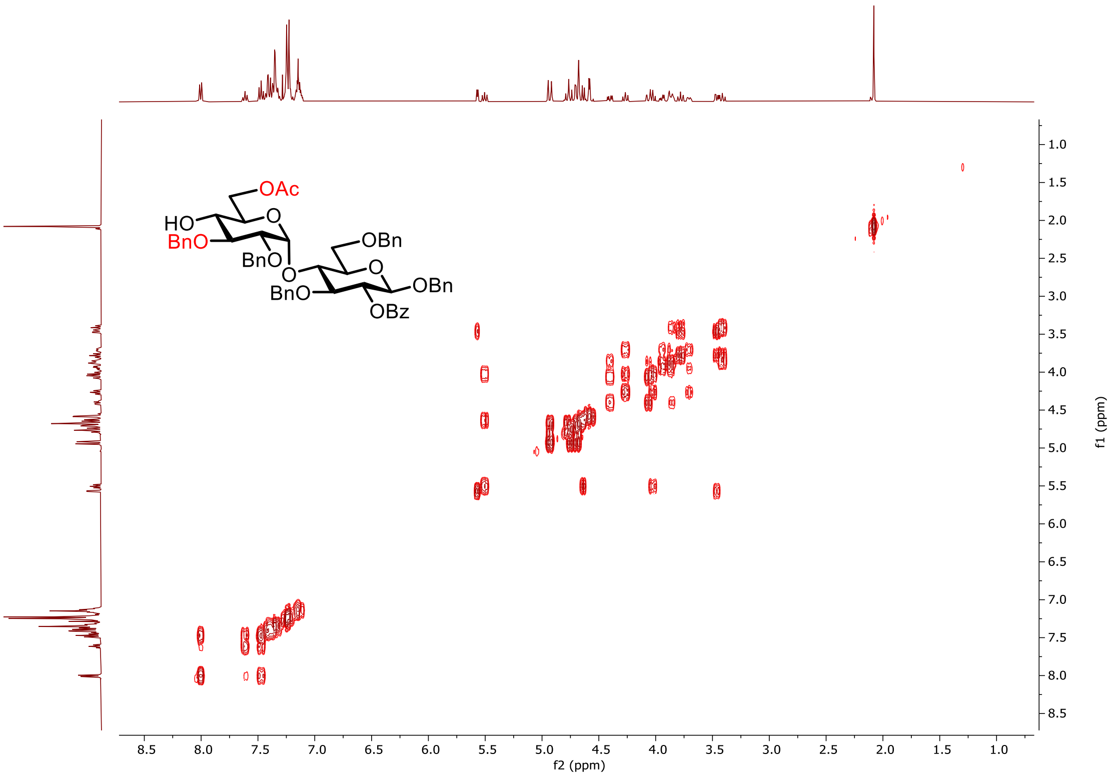

HSQC NMR of 96a ($CDCl_3$)

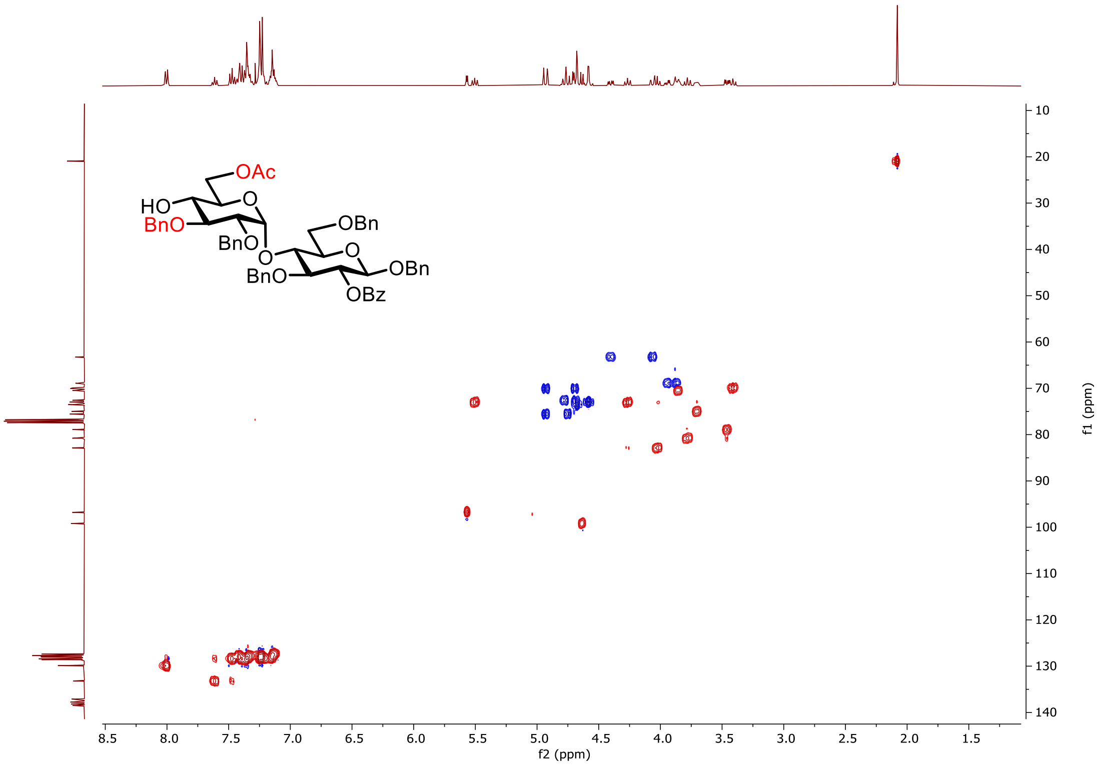

4.24 Benzyl 2,3-di-*O*-benzyl-6-*O*-pivaloyl-D-glucopyranosyl-(1→4)-2-*O*-benzoyl-3,6-di-*O*-benzyl-β-D-glucopyranoside, 97

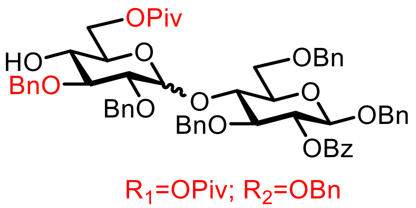

Total yield: 69% (67.7 mg). Ratio of anomer α : β = 5.5 : 1.

Spectrum data for benzyl 2,3-di-*O*-benzyl-6-*O*-pivaloyl-α-D-glucopyranosyl-(1→4)-2-*O*-benzoyl-3,6-di-*O*-benzyl-β-D-glucopyranoside **97a**: ^{1}H NMR (400 MHz, $CDCl_3$) δ 8.02 – 7.96 (m, 2H), 7.62 – 7.57 (m, 1H), 7.46 (appt, *J* = 7.8 Hz, 2H), 7.43 – 7.27 (m, 10H), 7.26 – 7.18 (m, 10H), 7.16 – 7.07 (m, 5H), 5.55 (d, *J* = 3.6 Hz, 1H), 5.47 (dd, *J* = 8.9, 7.6 Hz, 1H), 4.91 (dd, *J* = 11.8, 2.6 Hz, 2H), 4.79 – 4.71 (m, 2H), 4.71 – 4.59 (m, 5H), 4.57 (d, *J* = 1.9 Hz, 2H), 4.32 (dd, *J* = 12.2, 4.5 Hz, 1H), 4.26 (dd, *J* = 9.4, 8.4 Hz, 1H), 4.15 (dd, *J* = 12.2, 2.2 Hz, 1H), 4.00 (appt, *J* = 8.7 Hz, 1H), 3.93 (dd, *J* = 11.0, 4.4 Hz, 1H), 3.89 – 3.80 (m, 2H), 3.76 (dd, *J* = 9.7, 8.8 Hz, 1H), 3.68 (ddd, *J* = 9.4, 4.4, 2.2 Hz, 1H), 3.44 – 3.34 (m, 2H), 2.64 (br. s, 1H), 1.22 (s, 9H); ^{13}C NMR (101 MHz, $CDCl_3$) δ 179.15, 165.23, 138.50, 138.14, 137.82, 137.79, 137.10, 133.14, 129.88, 129.85, 128.59, 128.43, 128.38, 128.34, 128.31, 128.18, 128.05, 127.93, 127.86, 127.77, 127.75, 127.70, 127.68, 127.47, 127.39, 99.18, 96.37, 82.79, 80.70, 79.19, 75.51, 74.97, 73.49, 72.90, 72.78, 72.63, 72.20, 70.63, 70.12, 70.01, 69.03, 63.21, 38.96, 27.27; $[\alpha]_D^{25}$ 15.30 (c = 1, $CHCl_3$); IR (neat) ν_{max} = 3488, 2925, 1729, 1455, 1268, 1062, 698 cm^{-1}; m/z ($HRMS^+$) $[M + Na]^+$ 1003.440 ($C_{59}H_{64}O_{13}Na^+$ requires 1003.423).

^{1}H NMR of crude 97 (600 MHz, $CDCl_3$)

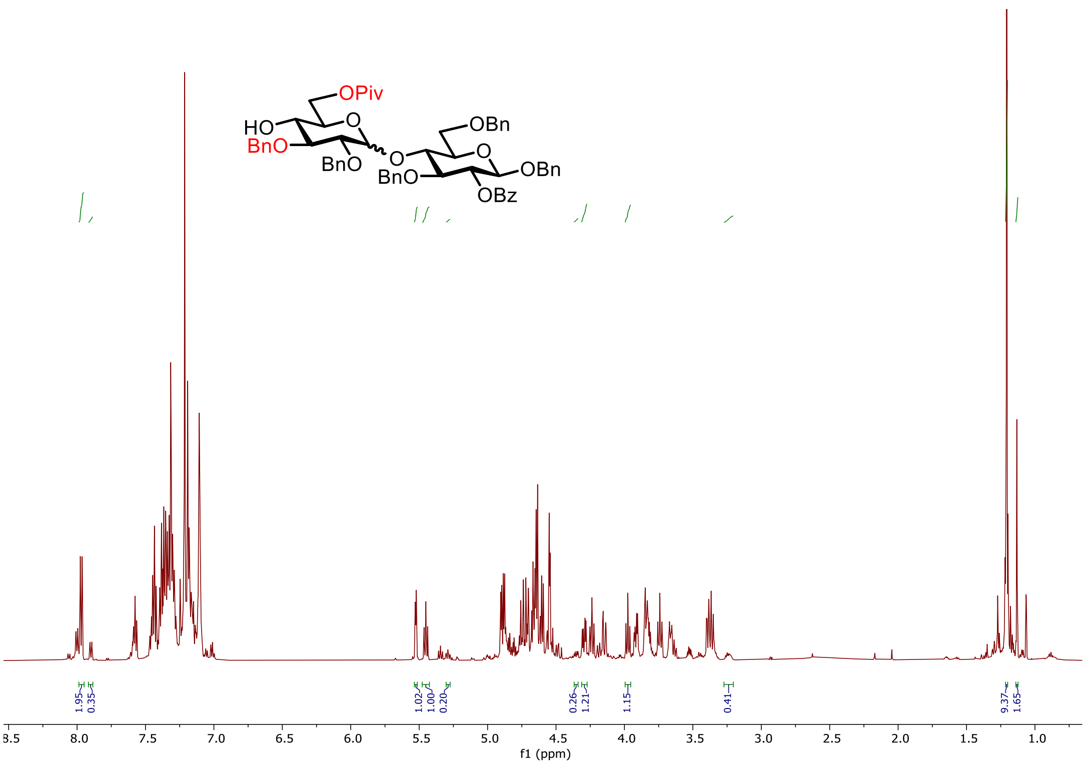

^{13}C NMR of crude 97 (151 MHz, $CDCl_3$)

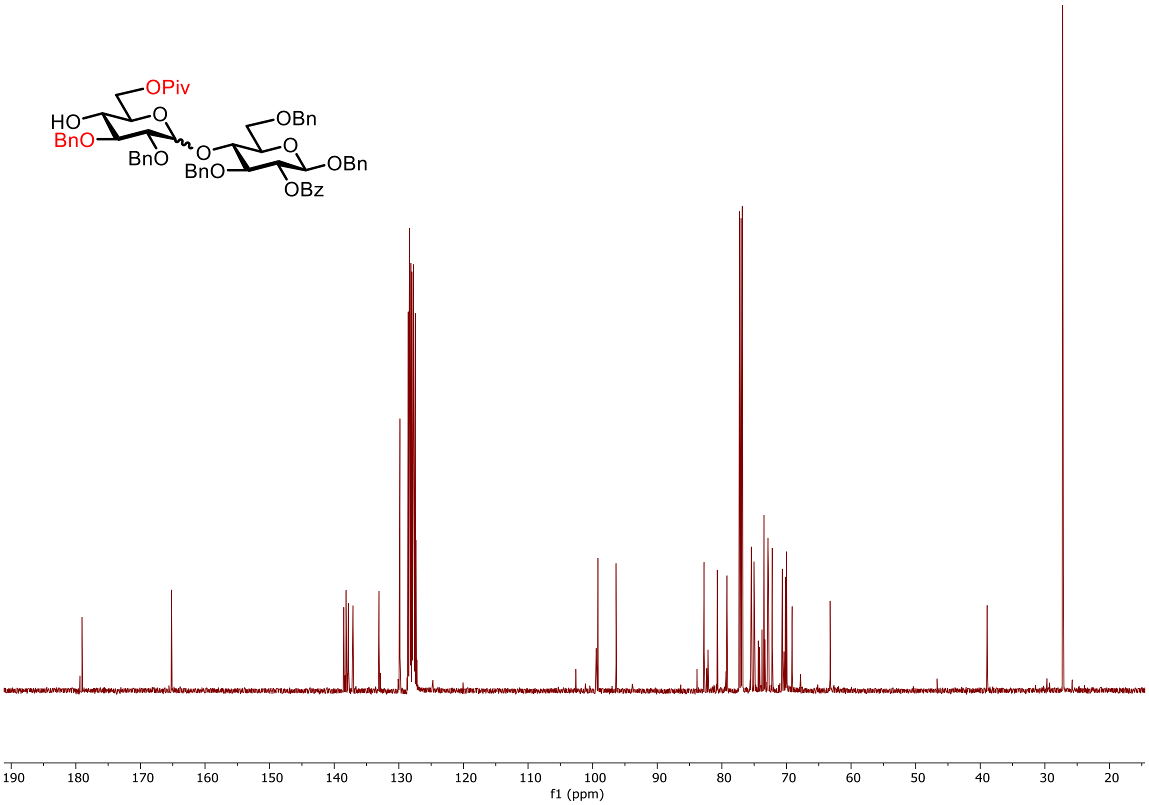

HSQC NMR of crude 97 ($CDCl_3$)

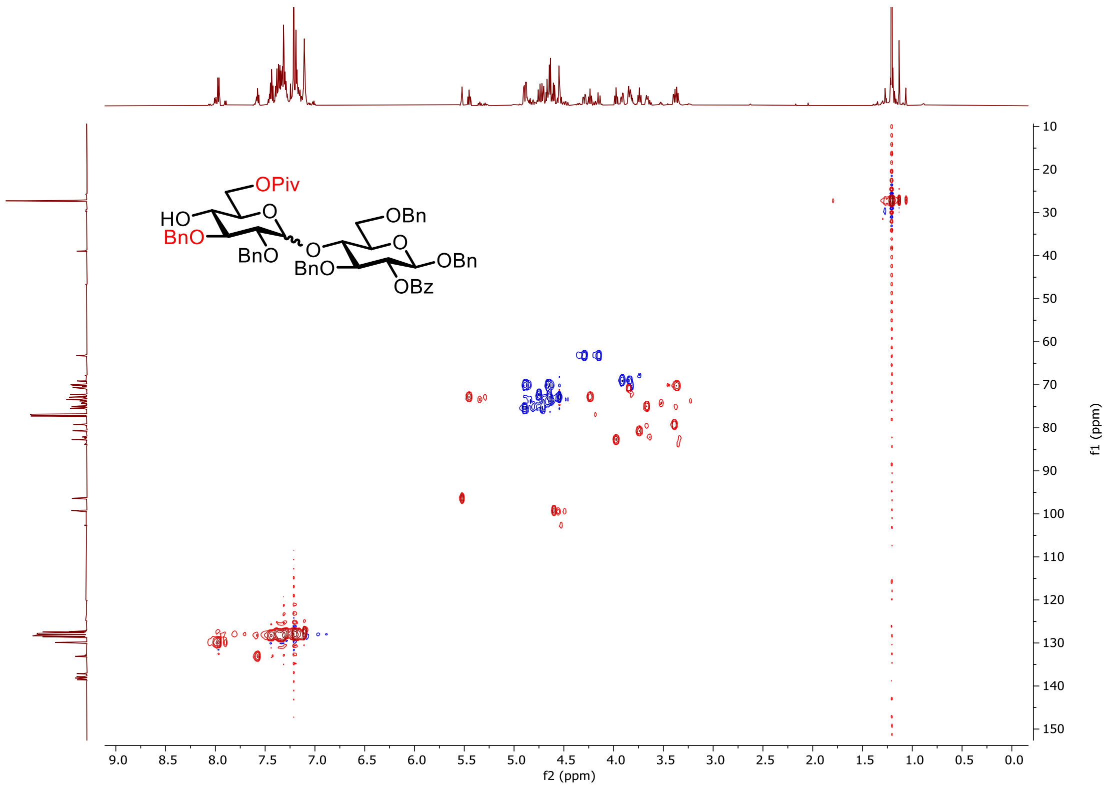

Coupled HSQC NMR of crude 97 ($CDCl_3$)

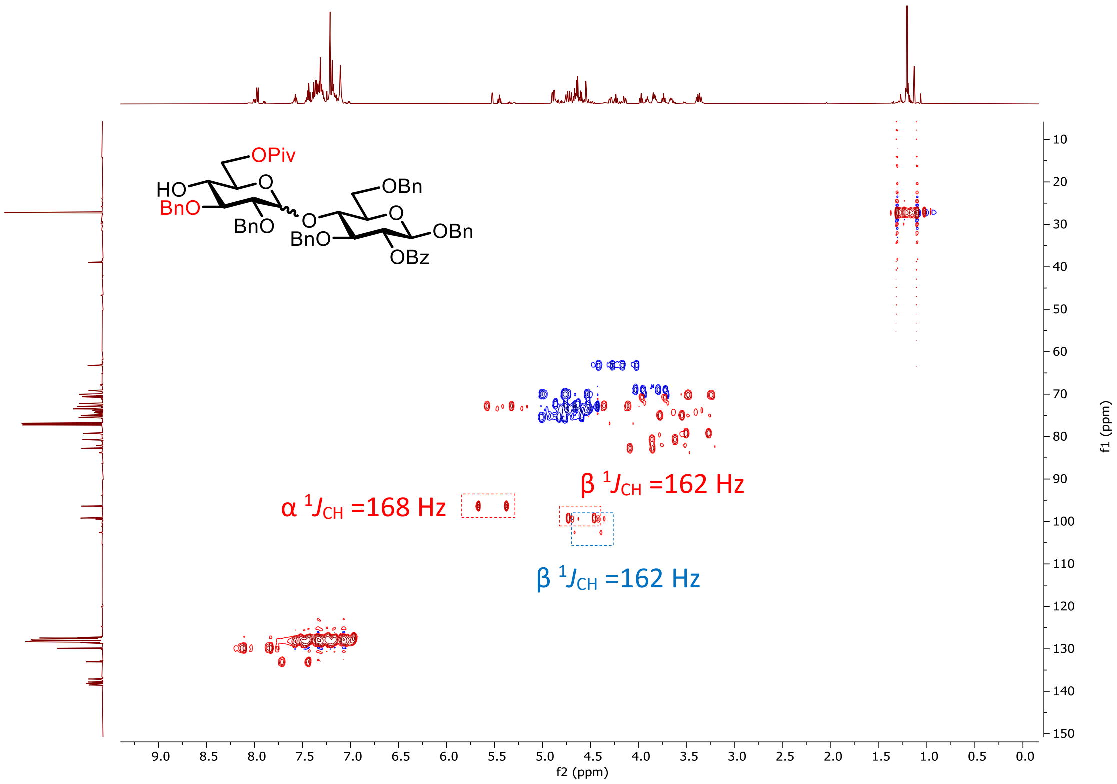

^{1}H NMR of 97a (400 MHz, $CDCl_3$)

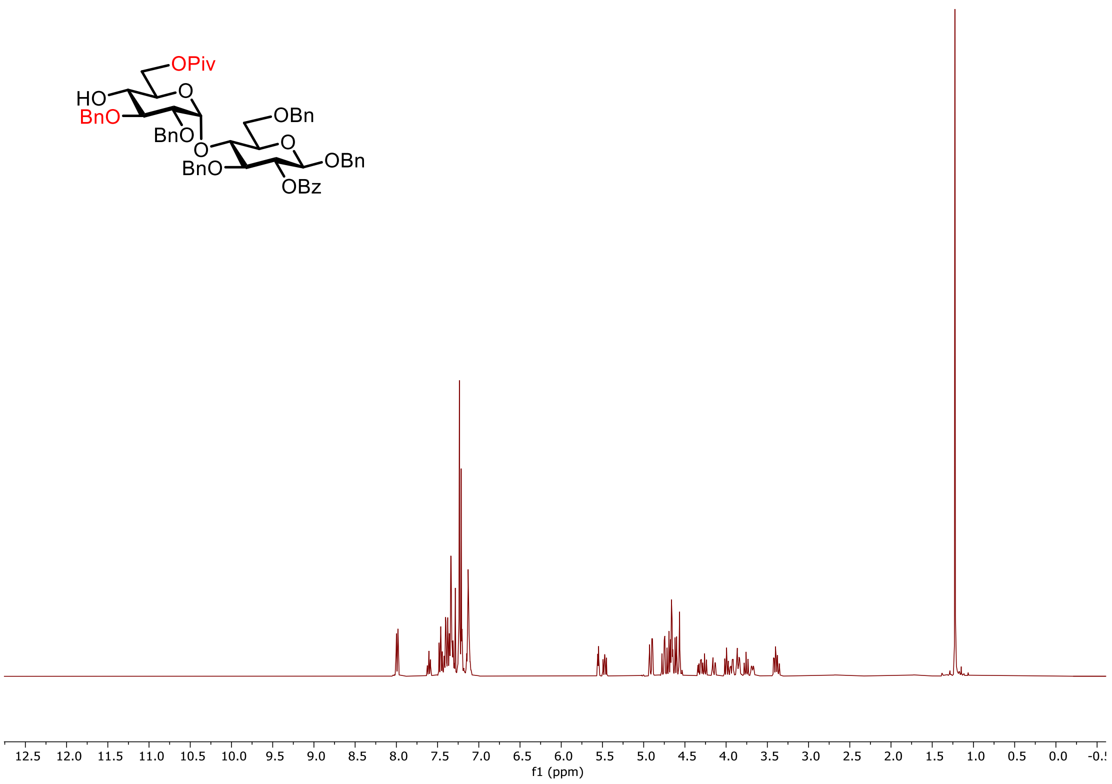

^{13}C NMR of 97a (101 MHz, $CDCl_3$)

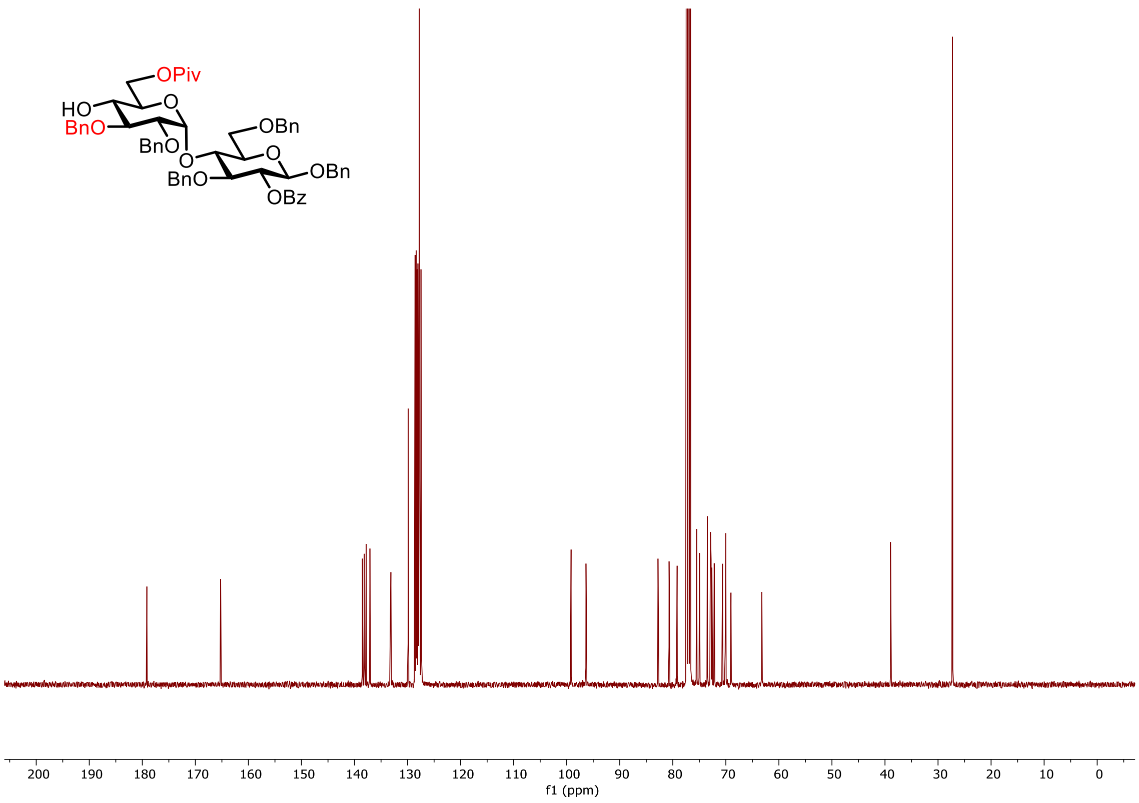

COSY NMR of 97a ($CDCl_3$)

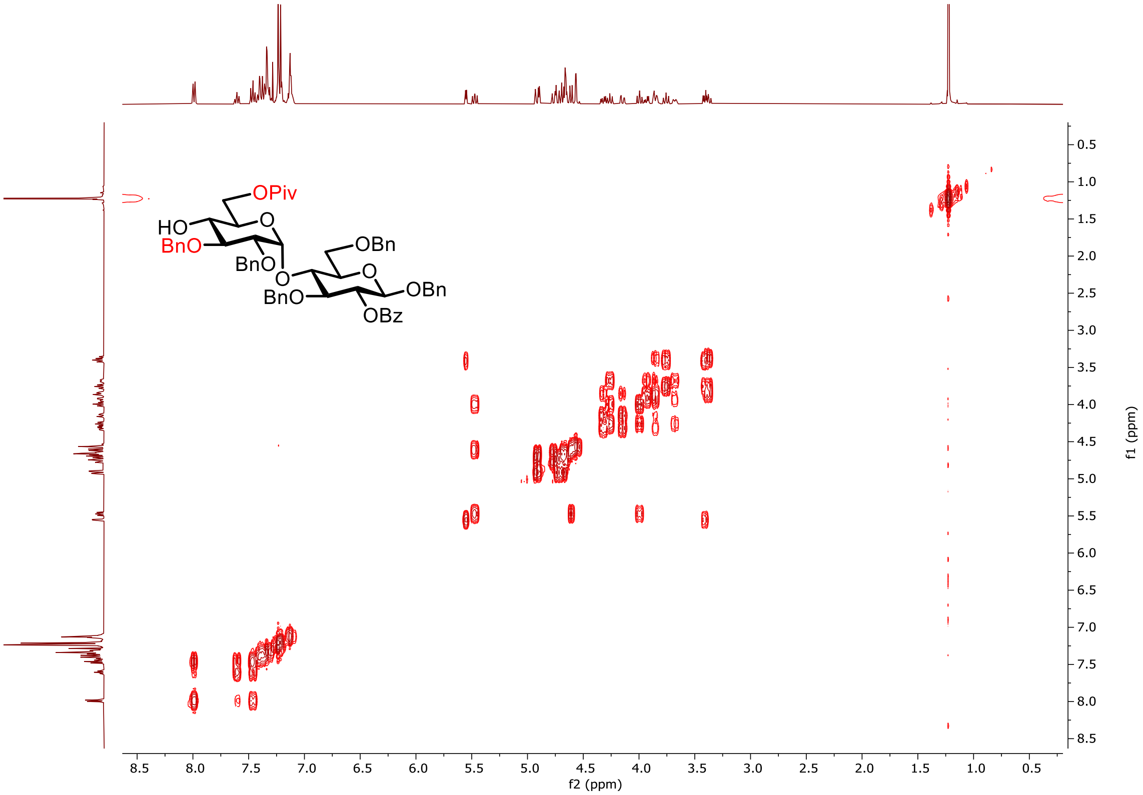

HSQC NMR of 97a ($CDCl_3$)

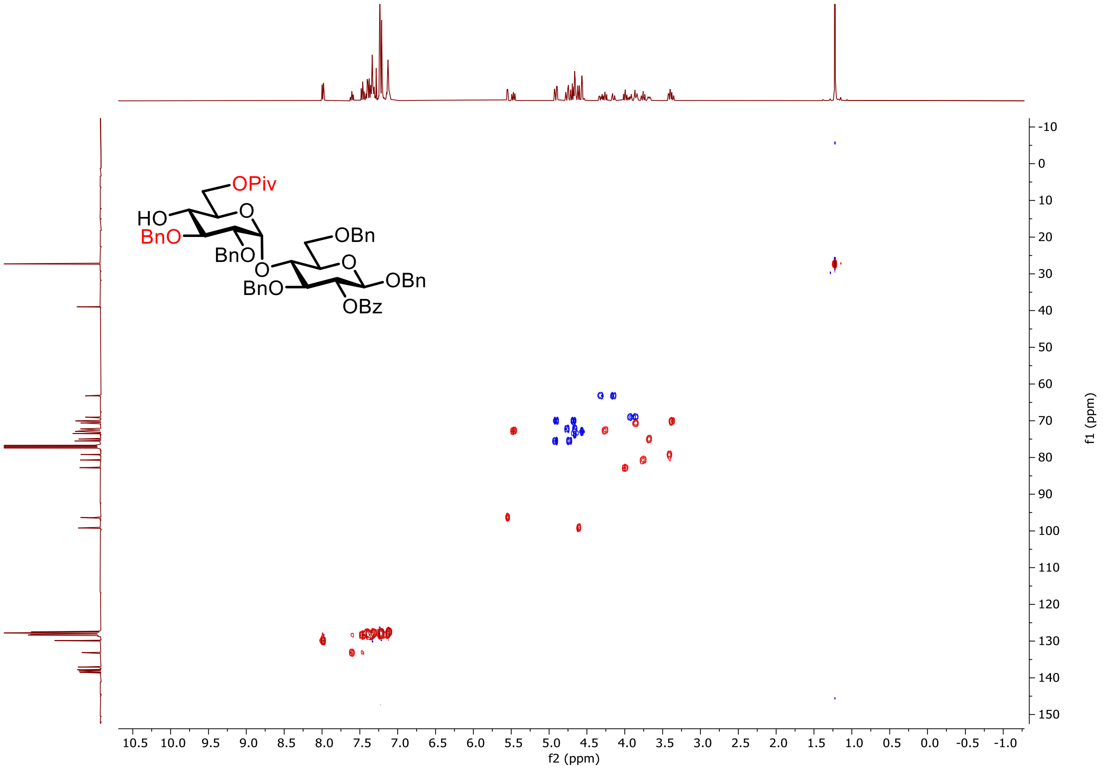

4.25 Benzyl 2,3-di-*O*-benzyl-6-*O*-benzoyl-D-glucopyranosyl-(1→4)-2-*O*-benzoyl-3,6-di-*O*-benzyl-β-D-glucopyranoside, 98

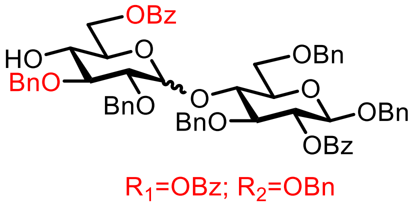

Total yield: 72% (72.1 mg). Ratio of anomer α : β = 6.8 : 1.

Spectrum data for benzyl 2,3-di-*O*-benzyl-6-*O*-benzoyl-α-D-glucopyranosyl-(1→4)-2-*O*-benzoyl-3,6-di-*O*-benzyl-β-D-glucopyranoside **98a**: ^{1}H NMR (400 MHz, $CDCl_3$) δ 8.11 – 8.06 (m, 2H), 8.05 – 7.99 (m, 2H), 7.65 – 7.58 (m, 2H), 7.48 (td, *J* = 7.8, 2.0 Hz, 4H), 7.44 – 7.31 (m, 10H), 7.31 – 7.21 (m, 10H), 7.17 – 7.10 (m, 5H), 5.60 (d, *J* = 3.6 Hz, 1H), 5.51 (dd, *J* = 8.9, 7.6 Hz, 1H), 4.97 (d, *J* = 8.4 Hz, 1H), 4.94 (d, *J* = 9.7 Hz, 1H), 4.78 (d, *J* = 11.1 Hz, 2H), 4.73 – 4.58 (m, 8H), 4.44 – 4.30 (m, 2H), 4.09 – 3.96 (m, 3H), 3.91 – 3.80 (m, 2H), 3.72 (ddd, *J* = 9.3, 4.3, 2.1 Hz, 1H), 3.57 – 3.45 (m, 2H), 2.84 – 2.27 (br. s, 1H); ^{13}C NMR (101 MHz, $CDCl_3$) δ 167.10, 165.27, 138.53, 138.14, 137.85, 137.81, 137.15, 133.35, 133.20, 129.92, 129.90, 129.86, 129.70, 128.62, 128.49, 128.45, 128.42, 128.39, 128.36, 128.23, 128.06, 127.95, 127.93, 127.80, 127.79, 127.75, 127.71, 127.50, 127.41, 99.22, 96.61, 82.78, 80.84, 79.15, 75.58, 75.00, 73.52, 72.93, 72.84, 72.32, 70.69, 70.13, 70.03, 68.92, 63.74; $[\alpha]_D^{25}$ 26.65 (c = 1, $CHCl_3$); IR (neat) ν_{max} = 3491, 2873, 1723, 1454, 1271, 1062, 698 cm^{-1}; m/z ($HRMS^+$) $[M + Na]^+$ 1023.409 ($C_{61}H_{60}O_{13}Na^+$ requires 1023.392).

^{1}H NMR of crude 98 (600 MHz, $CDCl_3$)

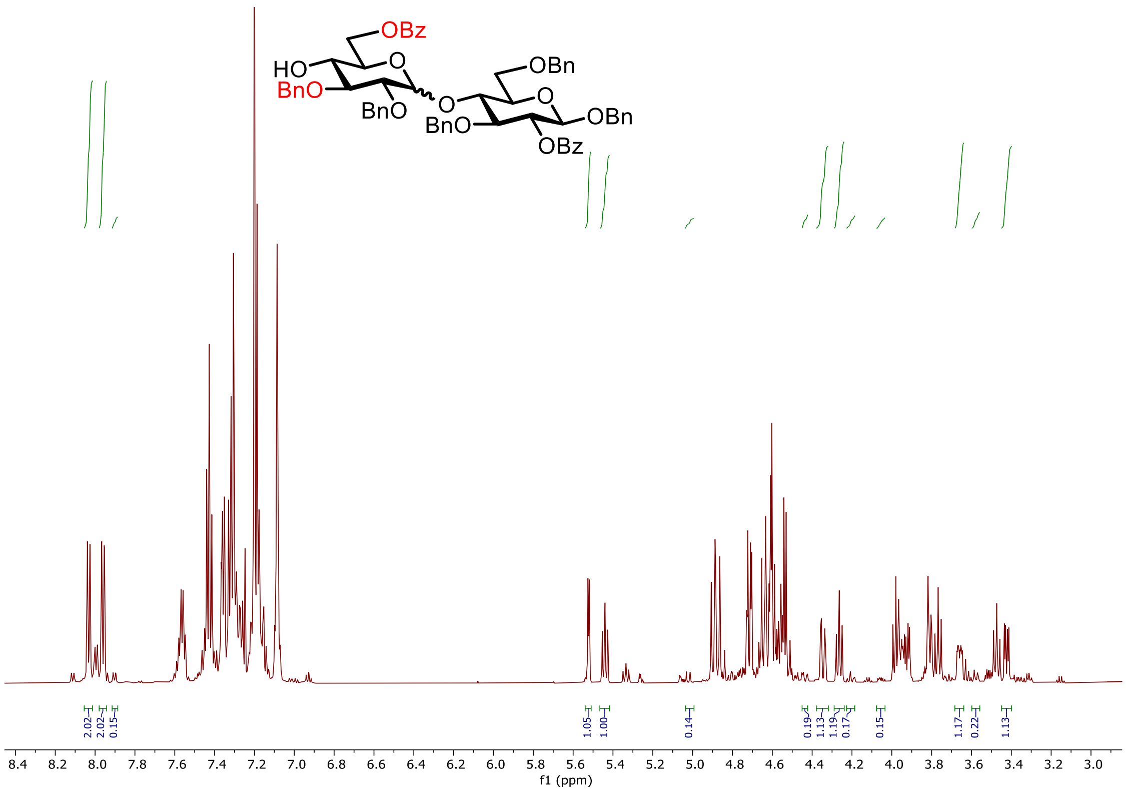

^{13}C NMR of crude 98 (151 MHz, $CDCl_3$)

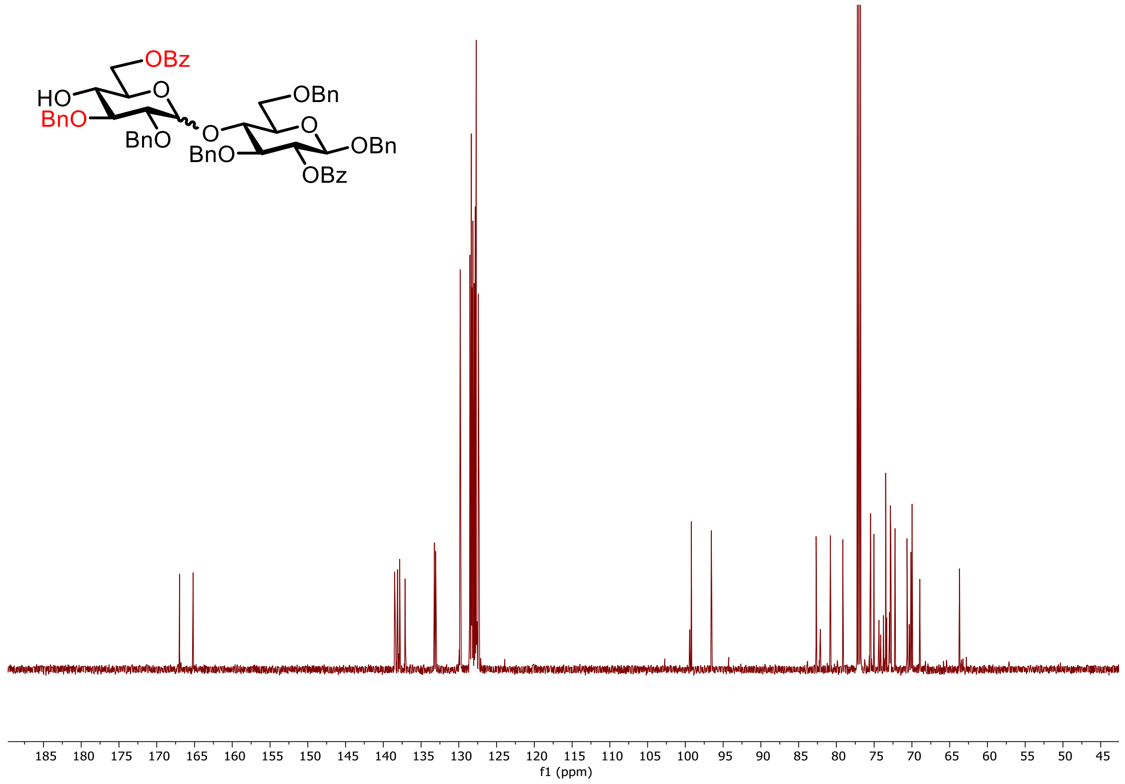

HSQC NMR of crude 98 ($CDCl_3$)

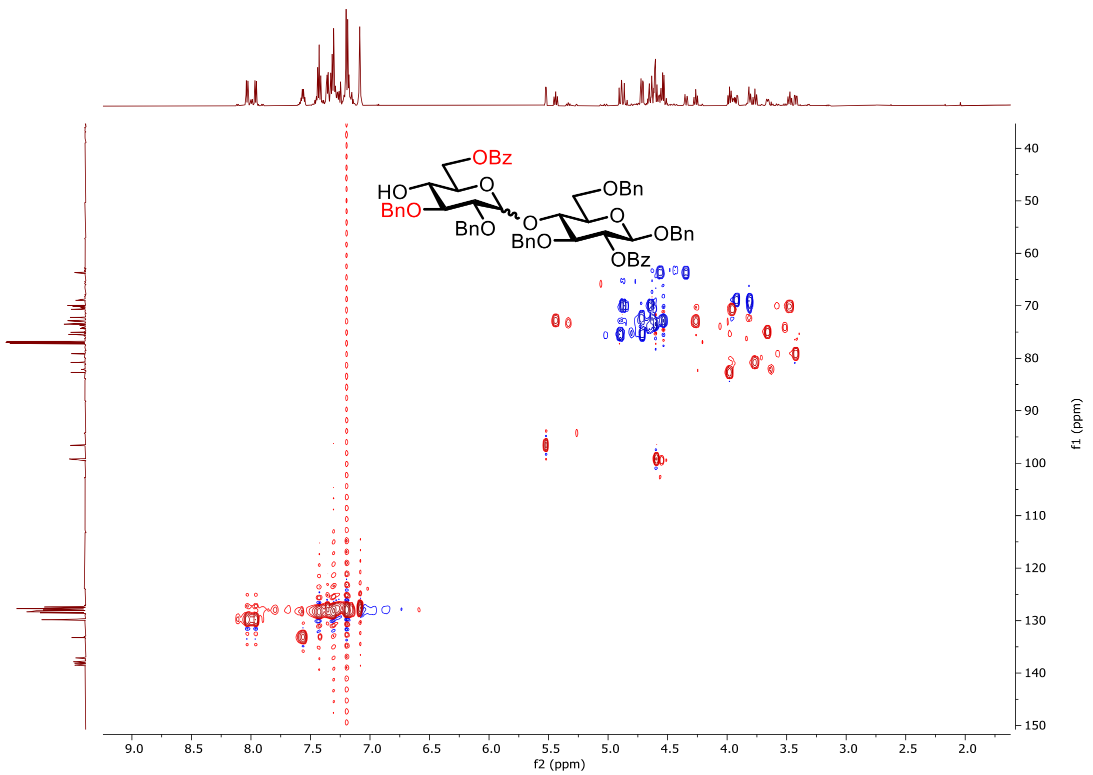

Coupled HSQC NMR of crude 98 ($CDCl_3$)

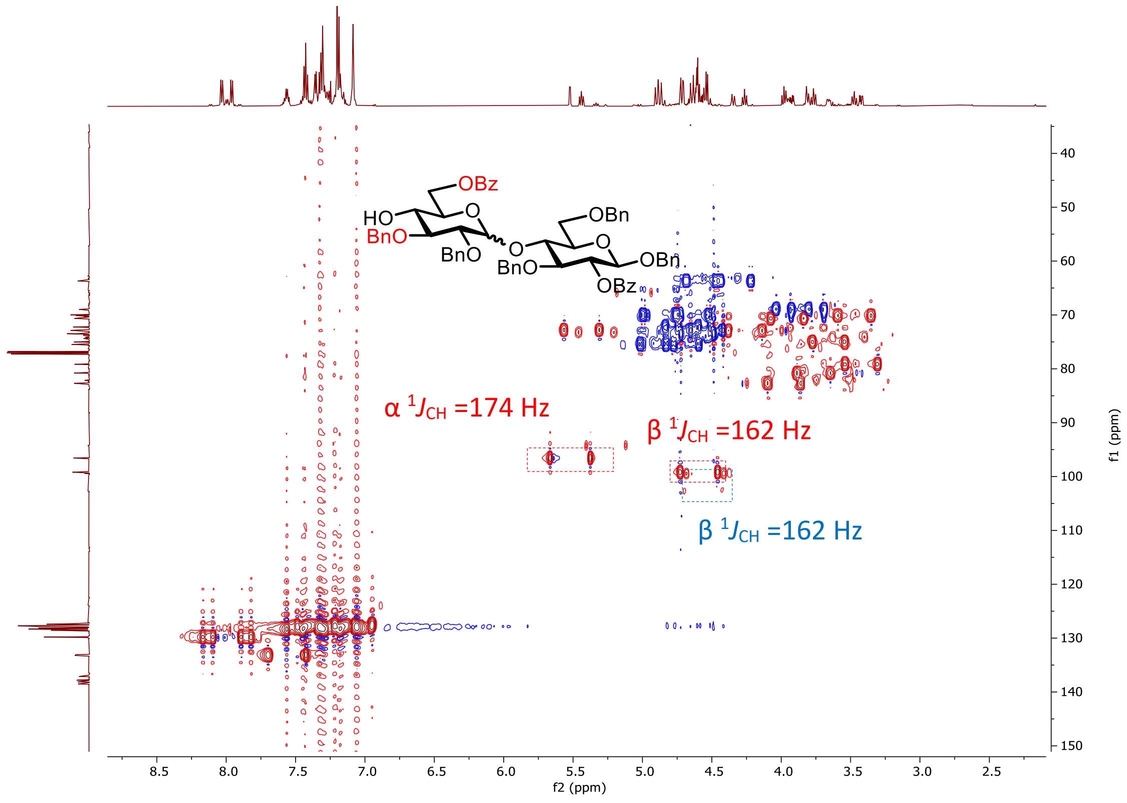

^{1}H NMR of 98a (400 MHz, $CDCl_3$)

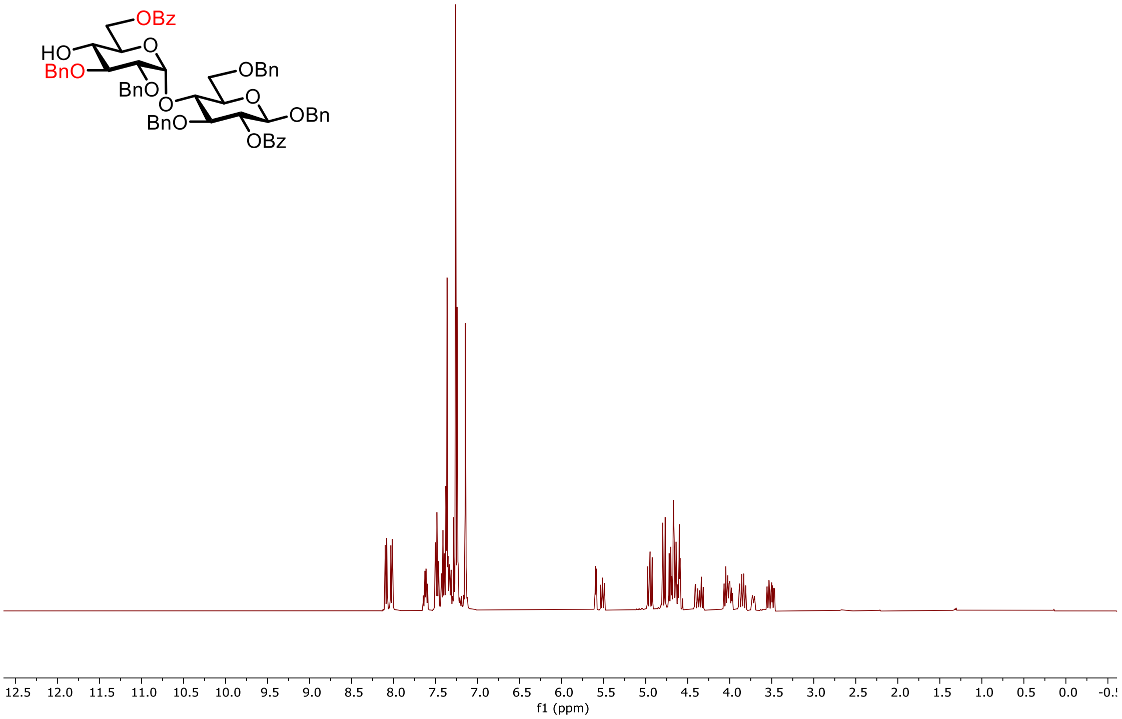

^{13}C NMR of 98a (101 MHz, $CDCl_3$)

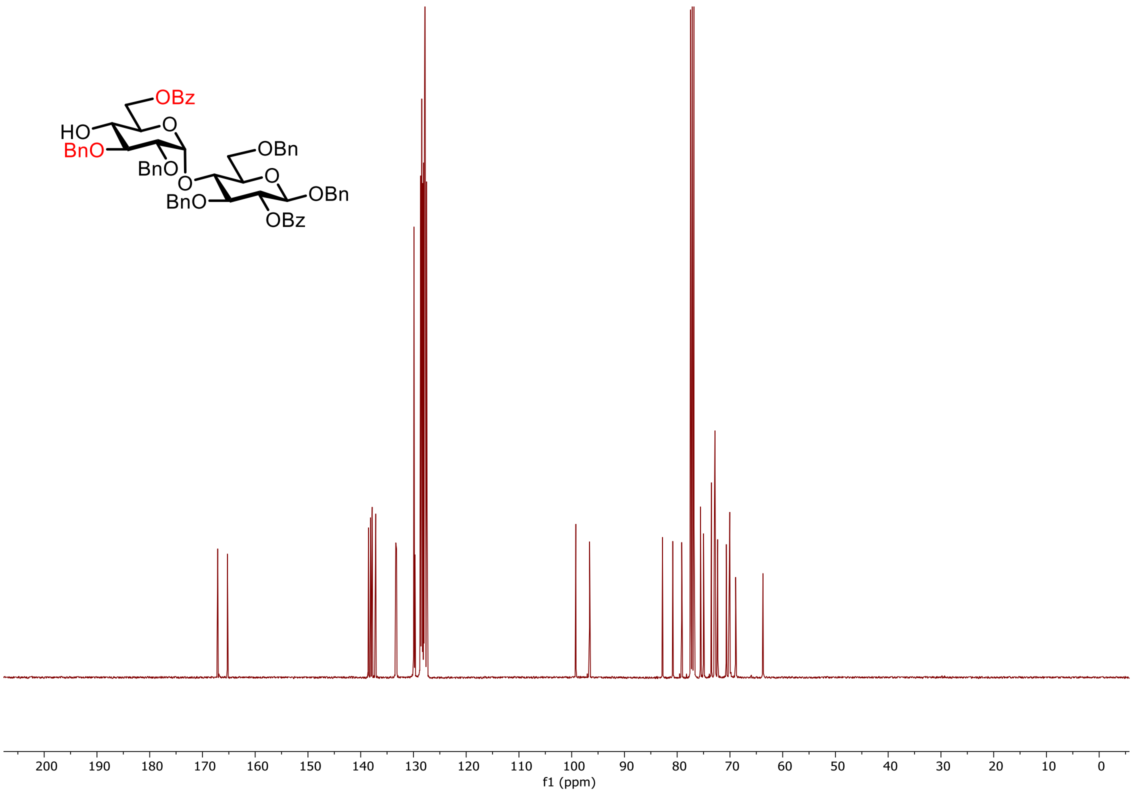

COSY NMR of 98a ($CDCl_3$)

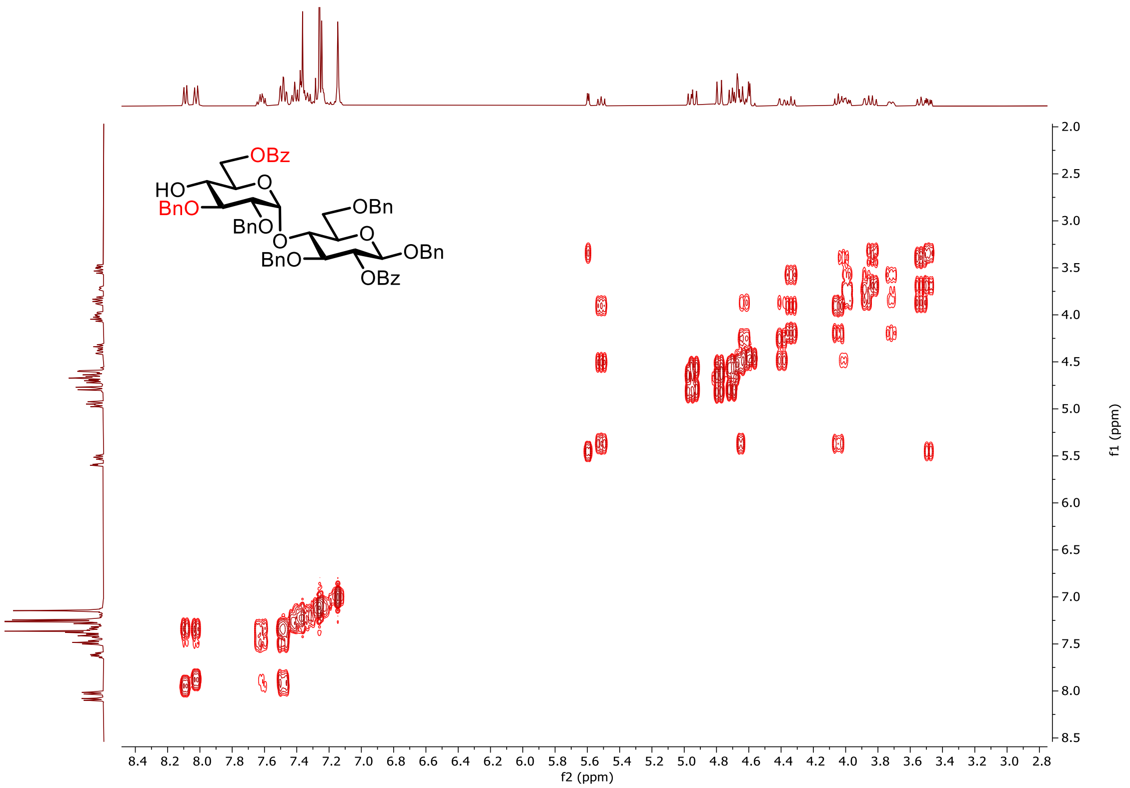

HSQC NMR of 98a ($CDCl_3$)

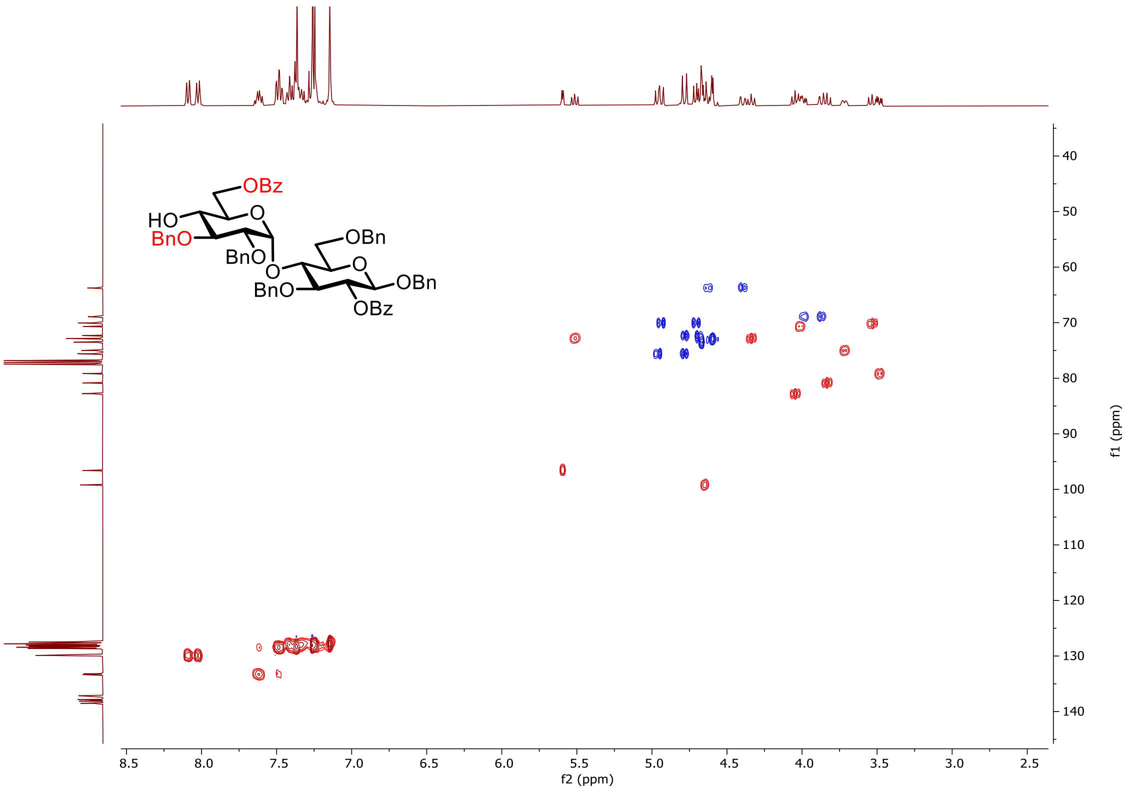

4.26 Benzyl 2,3-di-*O*-benzyl-6-*O*-(4-methoxybenzoyl)-D-glucopyranosyl-(1→4)-2-*O*-benzoyl-3,6-di-*O*-benzyl-β-D-glucopyranoside, 99

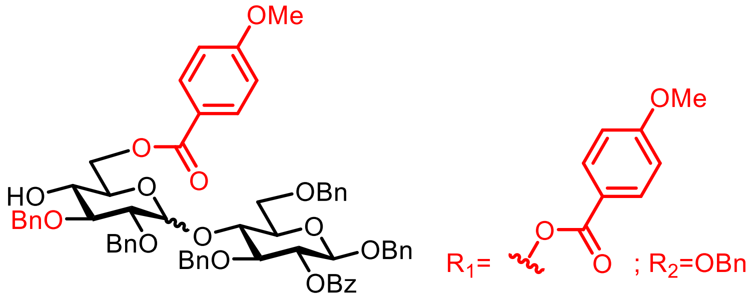

Total yield: 68% (70.1 mg). Ratio of anomer α : β = 5.6 : 1.

Spectrum data for benzyl 2,3-di-*O*-benzyl-6-*O*-(4-methoxybenzoyl)-α-D-glucopyranosyl-(1→4)-2-*O*-benzoyl-3,6-di-*O*-benzyl-β-D-glucopyranoside **99a**: ^{1}H NMR (400 MHz, $CDCl_3$) δ 8.08 – 8.01 (m, 4H), 7.65 – 7.61 (m, 1H), 7.49 (appt, *J* = 7.8 Hz, 2H), 7.46 – 7.32 (m, 12H), 7.31 – 7.22 (m, 10H), 7.21 – 7.12 (m, 5H), 6.99 – 6.94 (m, 2H), 5.61 (d, *J* = 3.6 Hz, 1H), 5.53 (dd, *J* = 8.9, 7.6 Hz, 1H), 4.96 (dd, *J* = 11.9, 6.3 Hz, 2H), 4.85 – 4.77 (m, 2H), 4.74 – 4.61 (m, 6H), 4.40 – 4.32 (m, 2H), 4.10 – 3.98 (m, 3H), 3.93 – 3.82 (m, 5H), 3.74 (ddd, *J* = 9.4, 4.3, 2.2 Hz, 1H), 3.57 – 3.47 (m, 2H), 2.97 (br. s, 1H); ^{13}C NMR (101 MHz, $CDCl_3$) δ 166.95, 165.27, 163.69, 138.60, 138.19, 137.89, 137.88, 137.17, 133.21, 132.02, 129.92, 129.89, 128.61, 128.46, 128.43, 128.40, 128.37, 128.24, 128.07, 127.94, 127.92, 127.81, 127.79, 127.76, 127.71, 127.51, 127.42, 122.02, 113.76, 99.29, 96.64, 82.83, 80.87, 79.15, 75.60, 75.02, 73.51, 72.98, 72.87, 72.82, 72.34, 70.88, 70.23, 70.09, 68.95, 63.54, 55.52; $[\alpha]_D^{25}$ 25.58 (c = 1, $CHCl_3$); IR (neat) ν_{max} = 3468, 2916, 1719, 1454, 1260, 1061, 698 cm^{-1}; m/z (HRMS$^+$) $[M + Na]^+$ 1053.420 ($C_{62}H_{62}O_{14}Na^+$ requires 1053.403).

^{1}H NMR of crude 99 (600 MHz, $CDCl_3$)

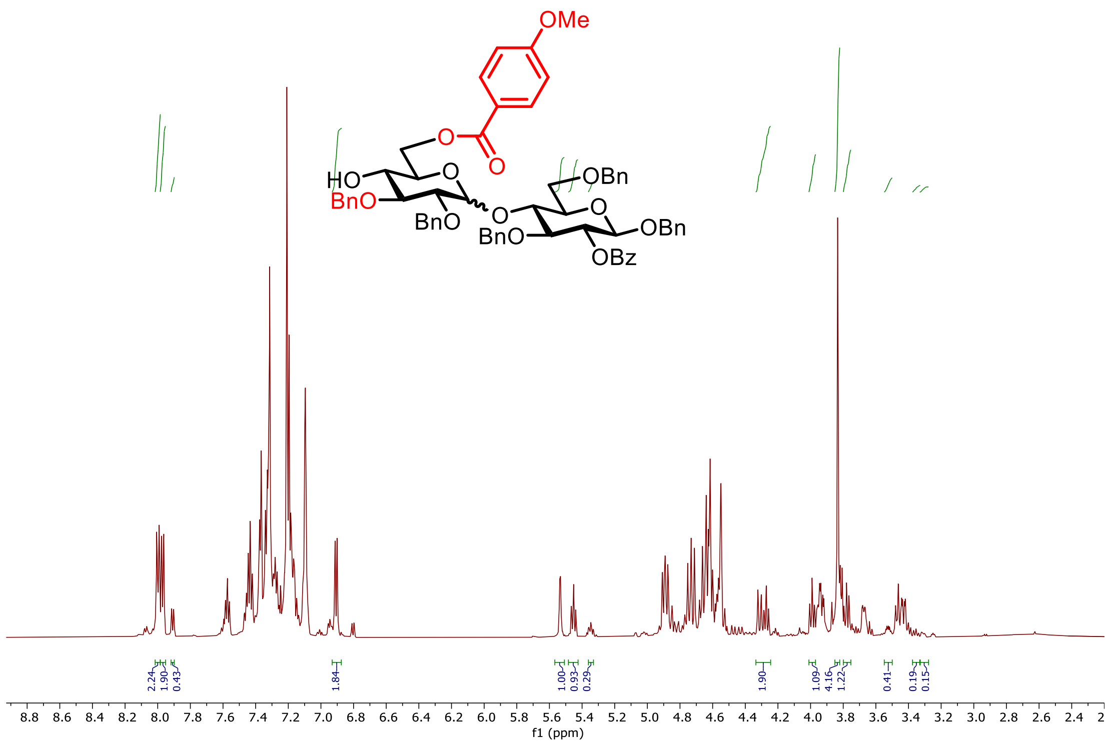

^{13}C NMR of crude 99 (151 MHz, $CDCl_3$)

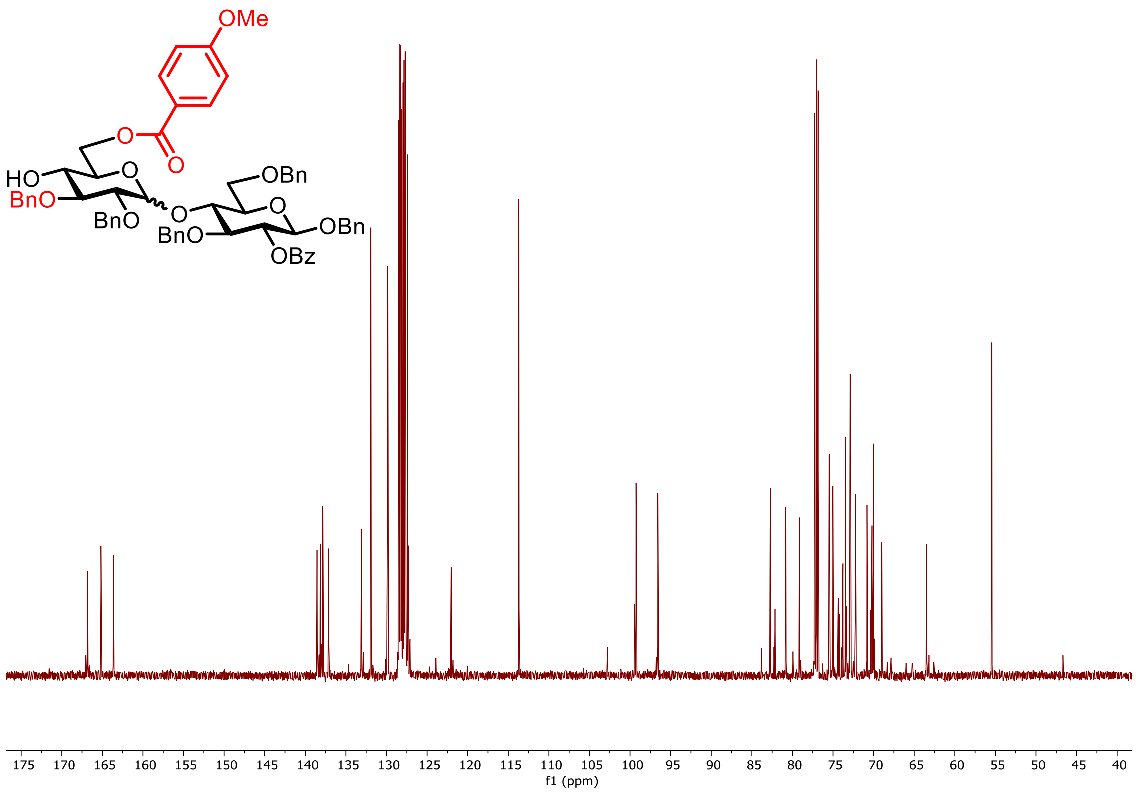

HSQC NMR of crude 99 ($CDCl_3$)

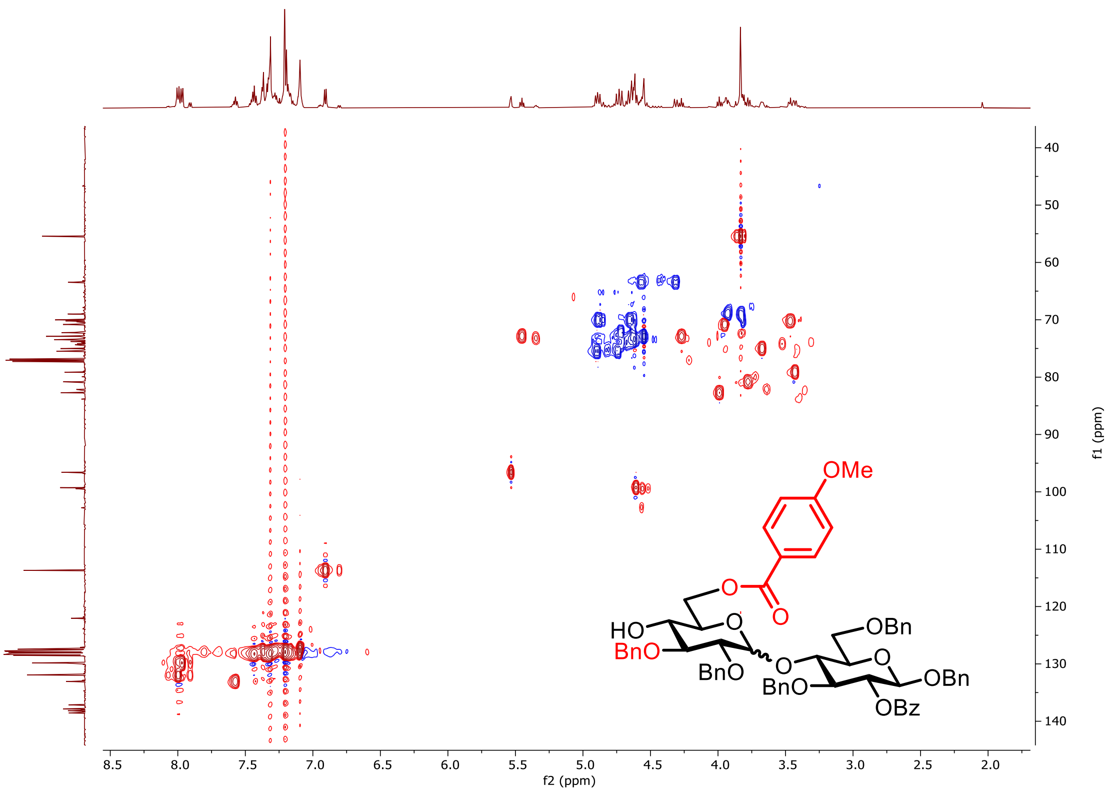

Coupled HSQC NMR of crude 99 ($CDCl_3$)

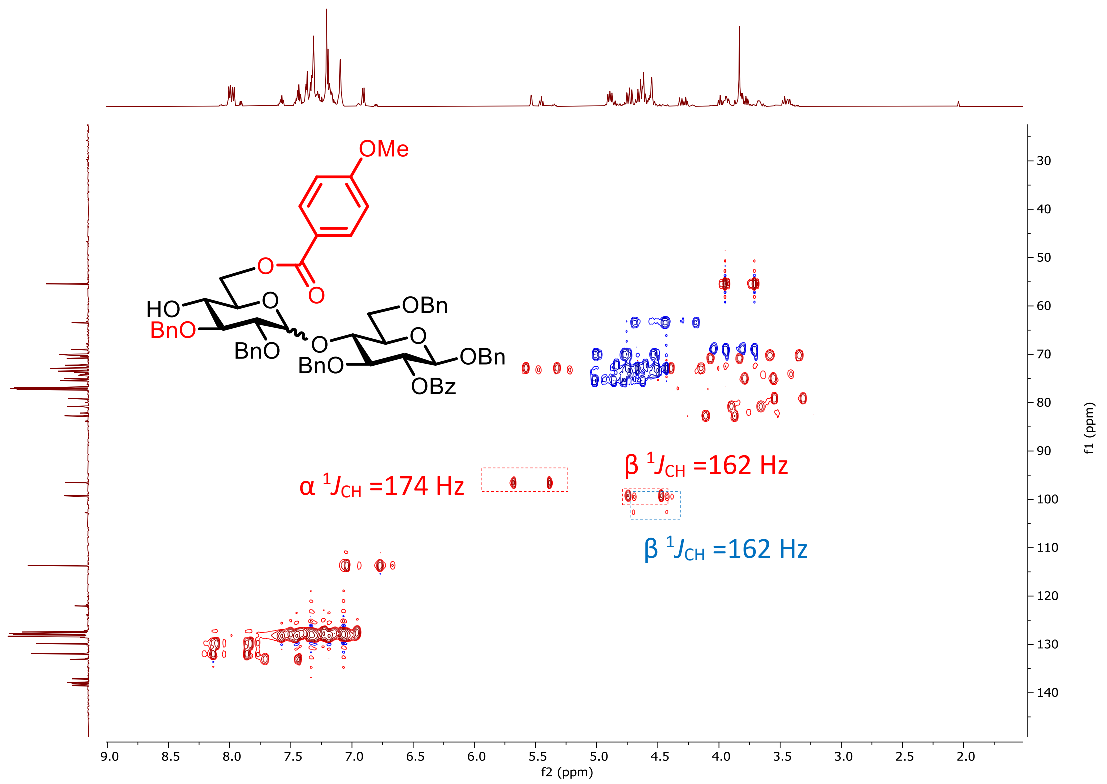

^{1}H NMR of 99a (400 MHz, $CDCl_3$)

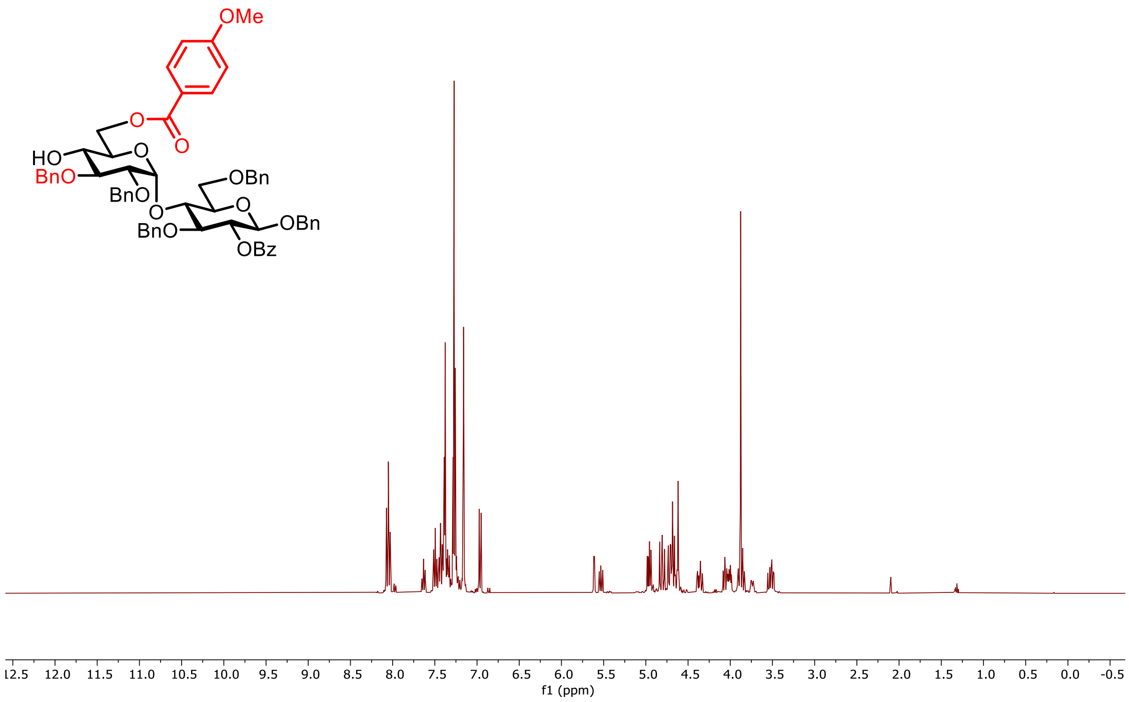

^{13}C NMR of 99a (101 MHz, $CDCl_3$)

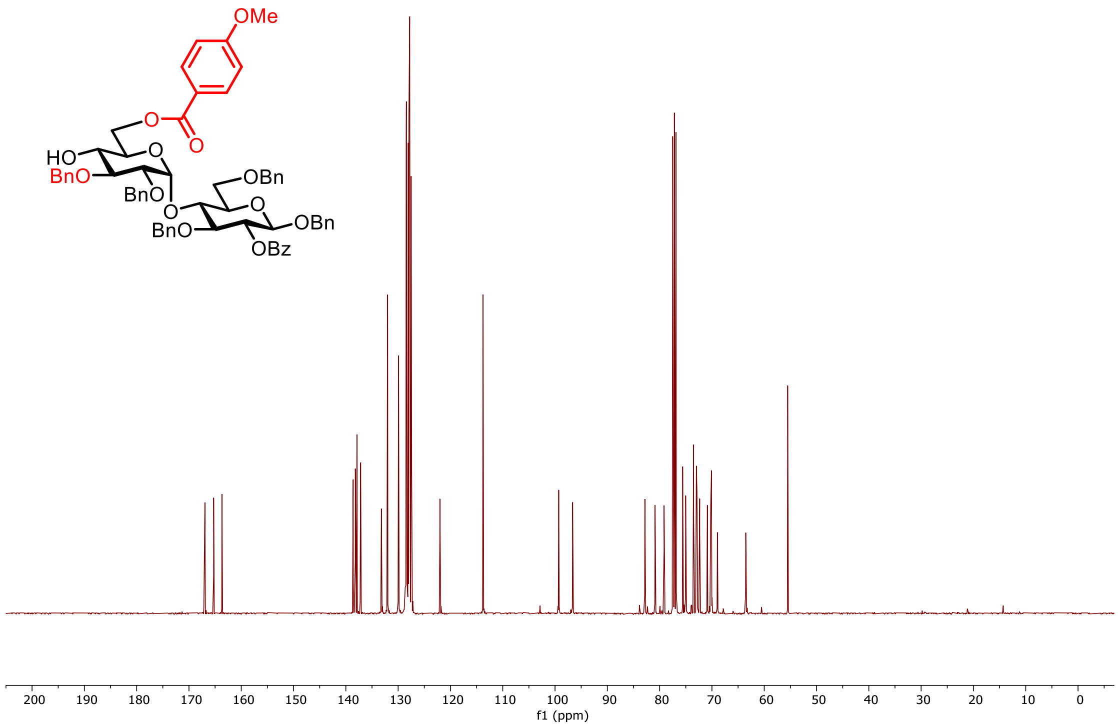

COSY NMR of 99a ($CDCl_3$)

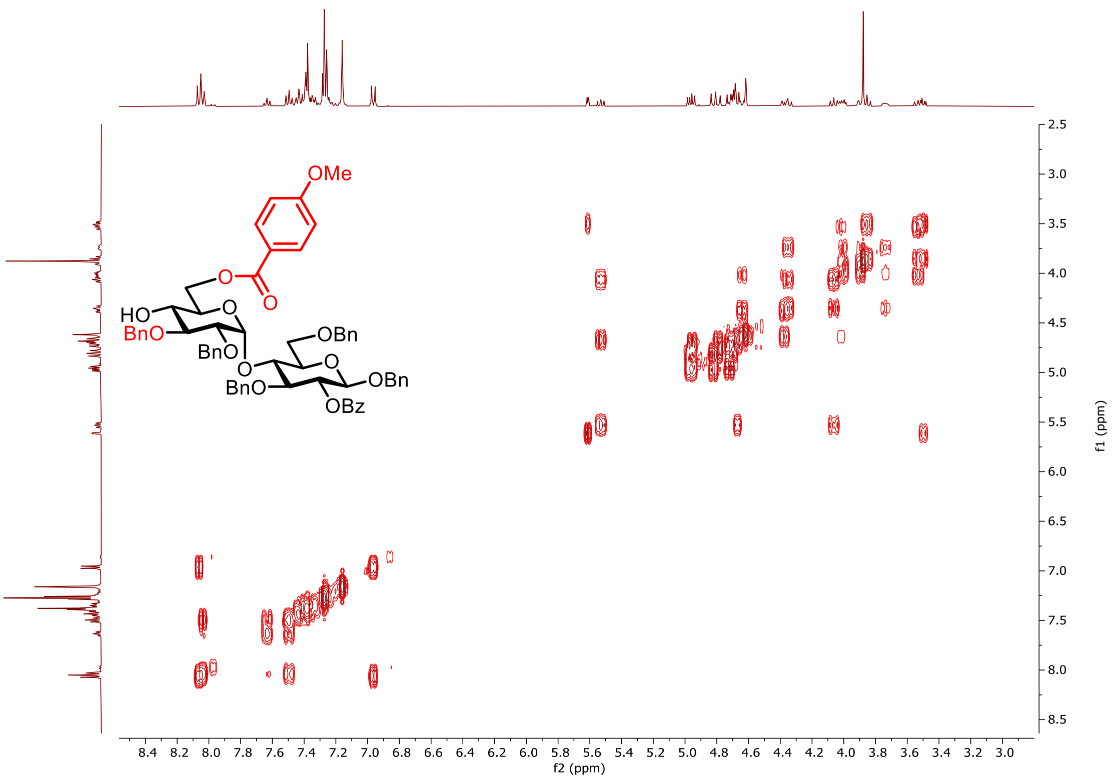

HSQC NMR of 99a ($CDCl_3$)

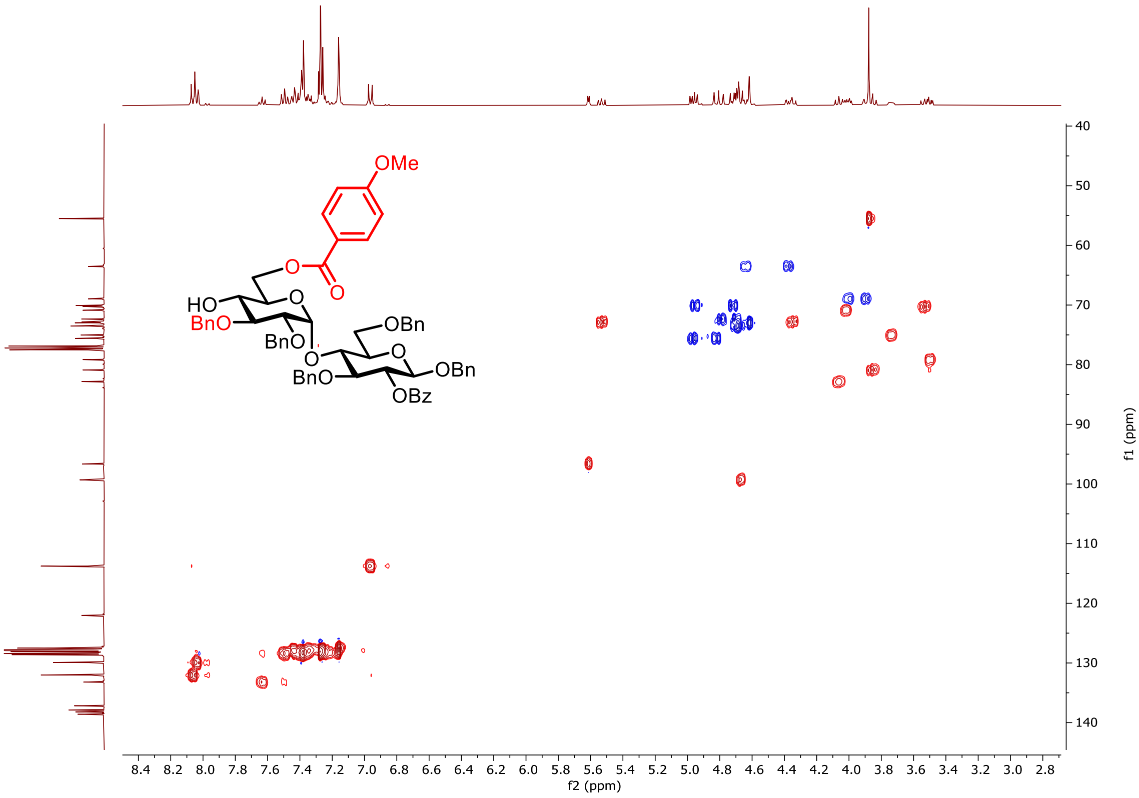

4.27 Benzyl 2,3-di-*O*-benzyl-6-*O*-(4-nitrobenzoyl)-D-glucopyranosyl-(1→4)-2-*O*-benzoyl-3,6-di-*O*-benzyl-β-D-glucopyranoside, 100

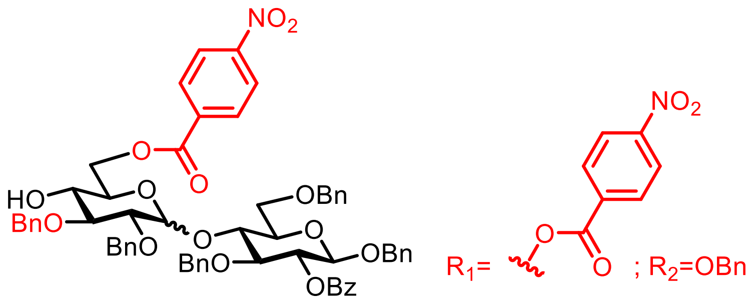

Total yield: 67% (70.1 mg). Ratio of anomer α : β = 6.2 : 1.

Spectrum data for benzyl 2,3-di-*O*-benzyl-6-*O*-(4-nitrobenzoyl)-α-D-glucopyranosyl-(1→4)-2-*O*-benzoyl-3,6-di-*O*-benzyl-β-D-glucopyranoside **100a**: ^{1}H NMR (400 MHz, $CDCl_3$) δ 8.30 – 8.26 (m, 2H), 8.22 – 8.17 (m, 2H), 8.05 – 8.00 (m, 2H), 7.67 – 7.59 (m, 1H), 7.48 (appt, *J* = 7.8 Hz, 2H), 7.43 – 7.31 (m, 10H), 7.30 – 7.20 (m, 10H), 7.19 – 7.10 (m, 5H), 5.61 (d, *J* = 3.6 Hz, 1H), 5.49 (dd, *J* = 8.9, 7.5 Hz, 1H), 4.99 (d, *J* = 11.2 Hz, 1H), 4.95 (d, *J* = 12.6 Hz, 1H), 4.78 – 4.65 (m, 7H), 4.62 – 4.55 (m, 3H), 4.44 (dd, *J* = 12.0, 2.2 Hz, 1H), 4.33 (appt, *J* = 8.8 Hz, 1H), 4.10 – 4.01 (m, 2H), 3.97 (dd, *J* = 11.1, 4.1 Hz, 1H), 3.88 (dd, *J* = 11.0, 2.2 Hz, 1H), 3.83 (appt, *J* = 9.2 Hz, 1H), 3.74 (ddd, *J* = 9.4, 4.1, 2.2 Hz, 1H), 3.56 – 3.46 (m, 2H), 2.52 (br. s, 1H); ^{13}C NMR (101 MHz, $CDCl_3$) δ 165.23, 164.93, 150.62, 138.39, 138.05, 137.73, 137.69, 137.10, 135.13, 133.24, 130.95, 129.91, 129.80, 128.70, 128.47, 128.44, 128.41, 128.37, 128.25, 128.08, 128.06, 127.91, 127.89, 127.77, 127.73, 127.49, 127.46, 123.62, 99.23, 96.29, 82.78, 80.82, 79.14, 75.57, 74.89, 73.58, 72.85, 72.22, 70.24, 70.08, 70.01, 68.87, 64.60; $[\alpha]_D^{25}$ 27.41 (c = 1, $CHCl_3$); IR (neat) ν_{max} = 3535, 2872, 1728, 1528, 1270, 1060, 698 cm^{-1}; m/z (HRMS$^+$) $[M + Na]^+$ 1068.397 ($C_{61}H_{59}NO_{15}Na^+$ requires 1068.377).

^{1}H NMR of crude 100 (600 MHz, $CDCl_3$)

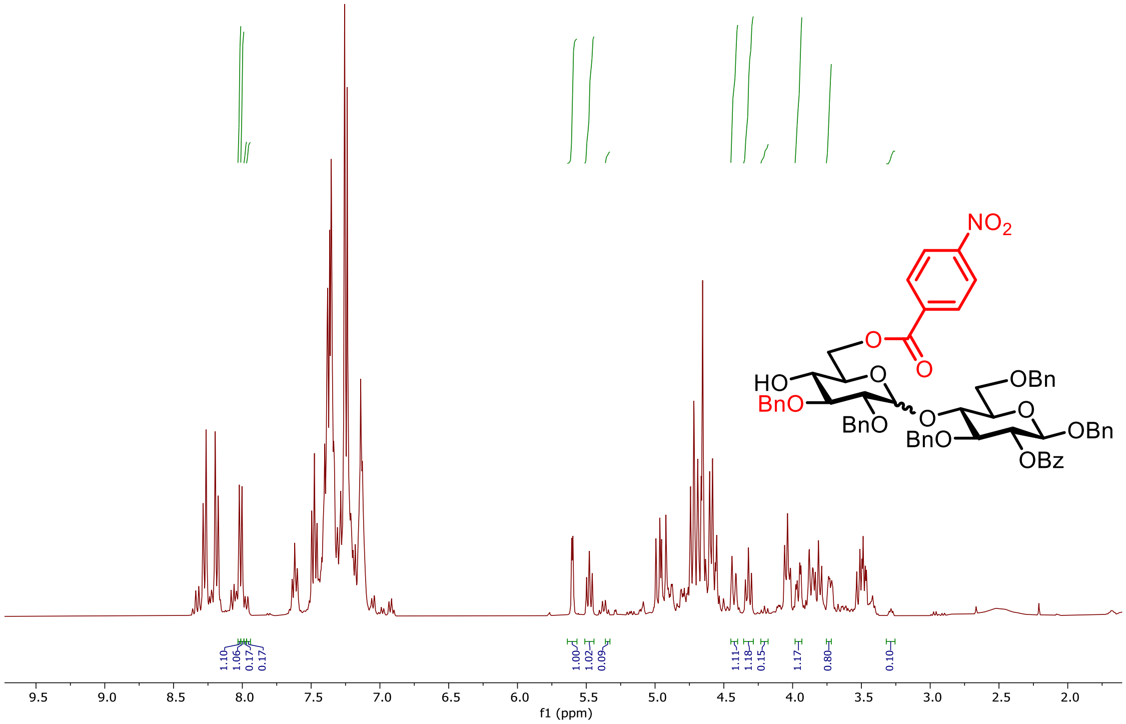

^{13}C NMR of crude 100 (151 MHz, $CDCl_3$)

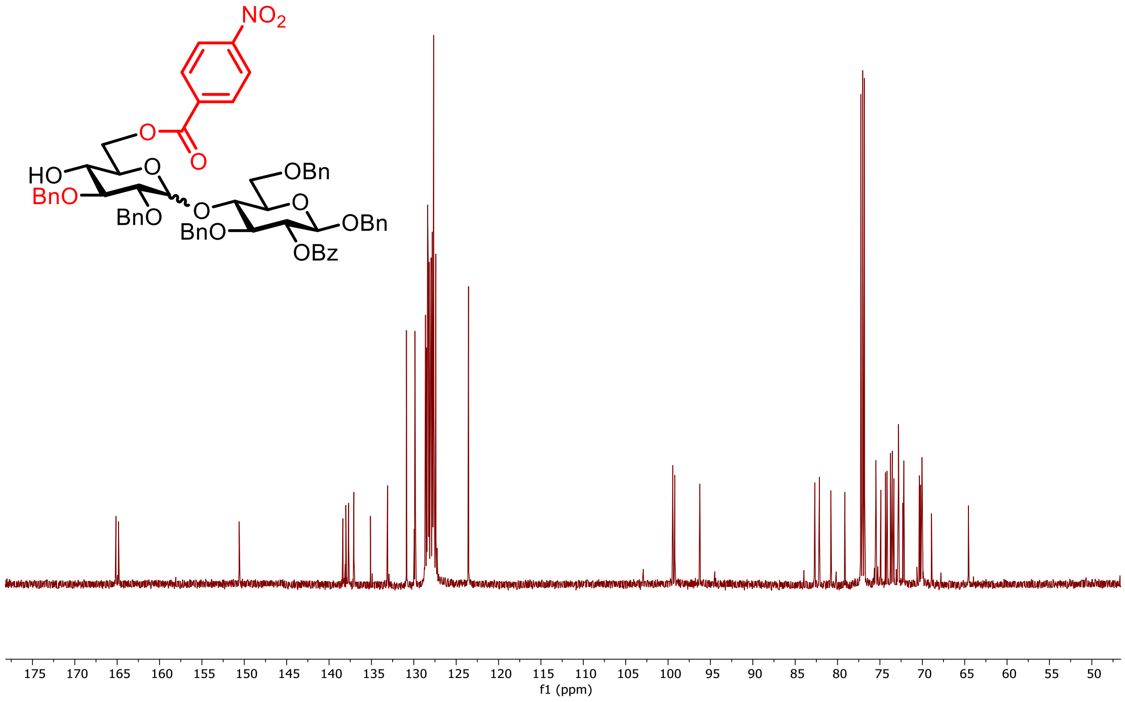

HSQC NMR of crude 100 ($CDCl_3$)

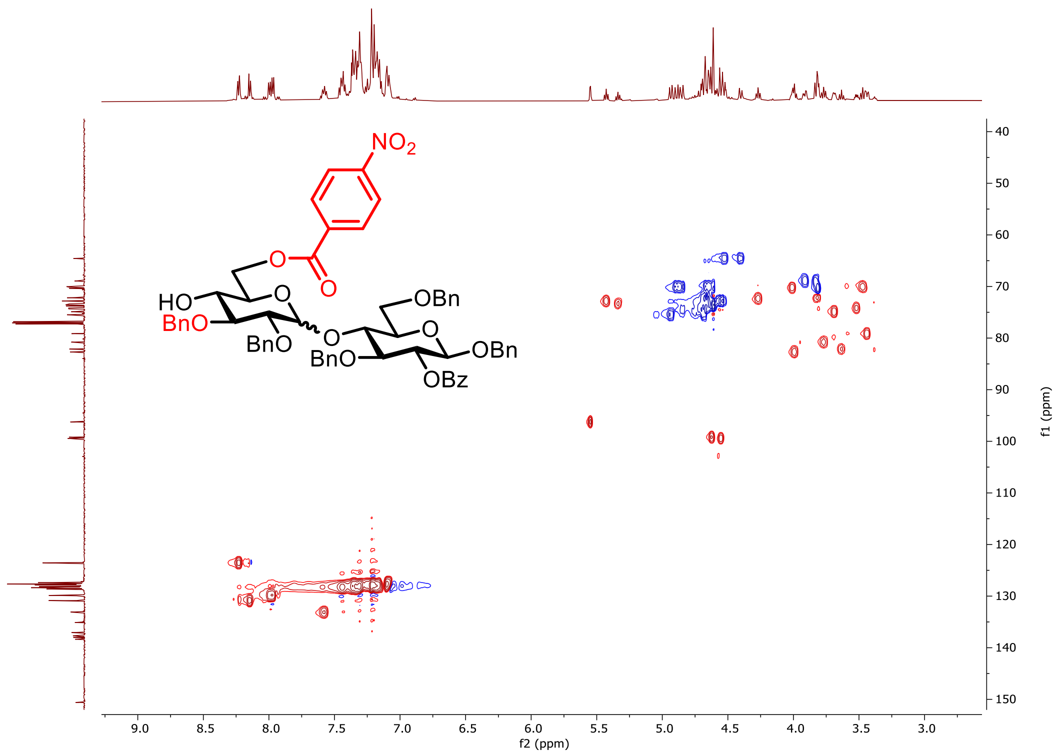

Coupled HSQC NMR of crude 100 ($CDCl_3$)

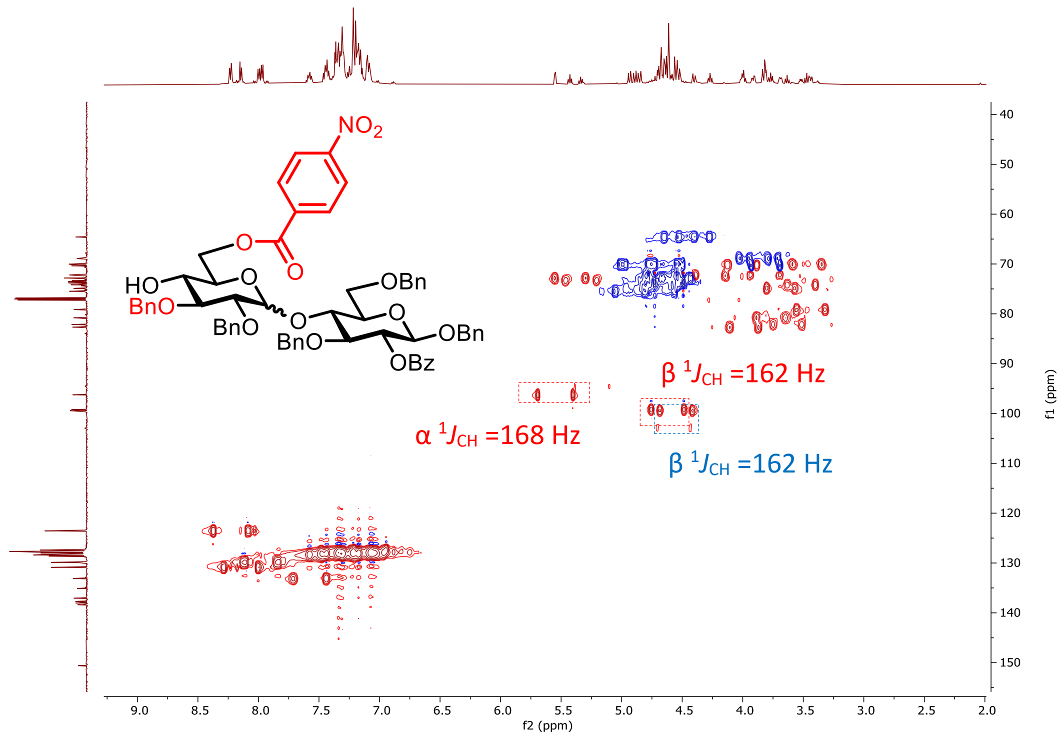

^{1}H NMR of 100a (400 MHz, $CDCl_3$)

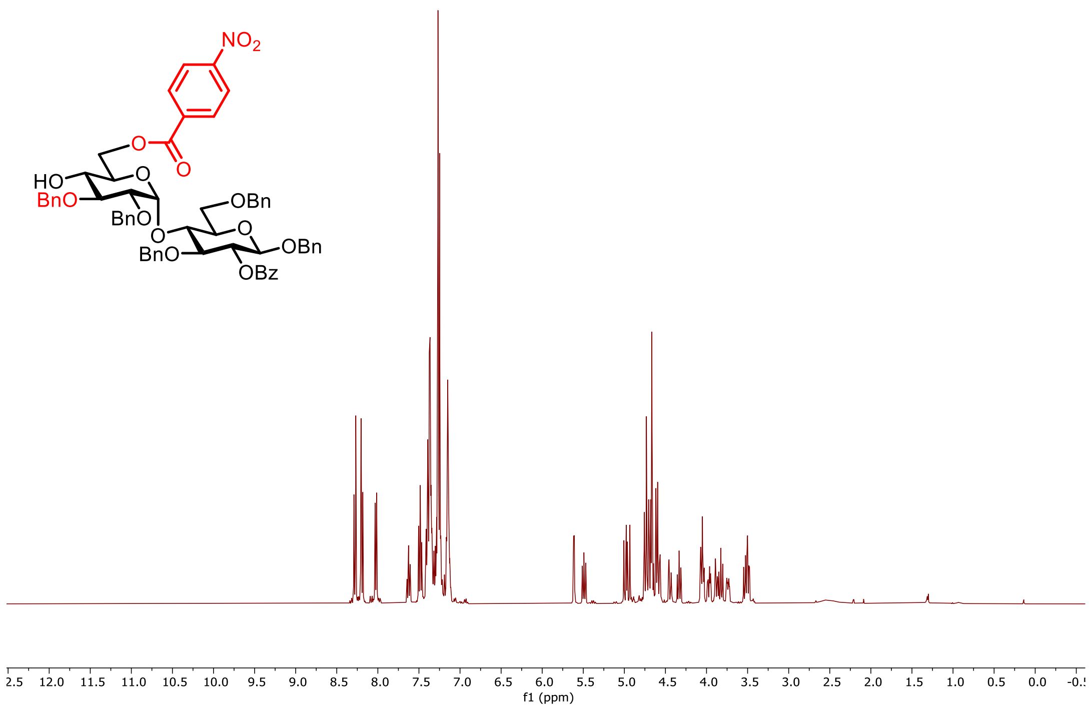

^{13}C NMR of 100a (101 MHz, $CDCl_3$)

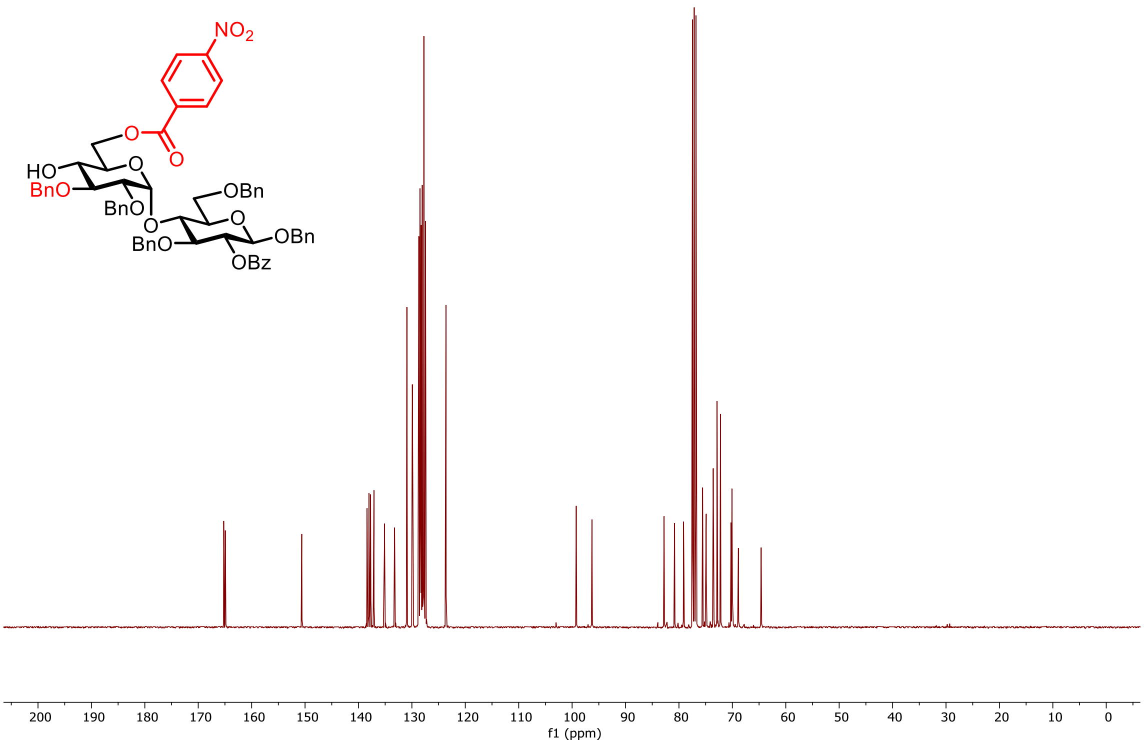

COSY NMR of 100a ($CDCl_3$)

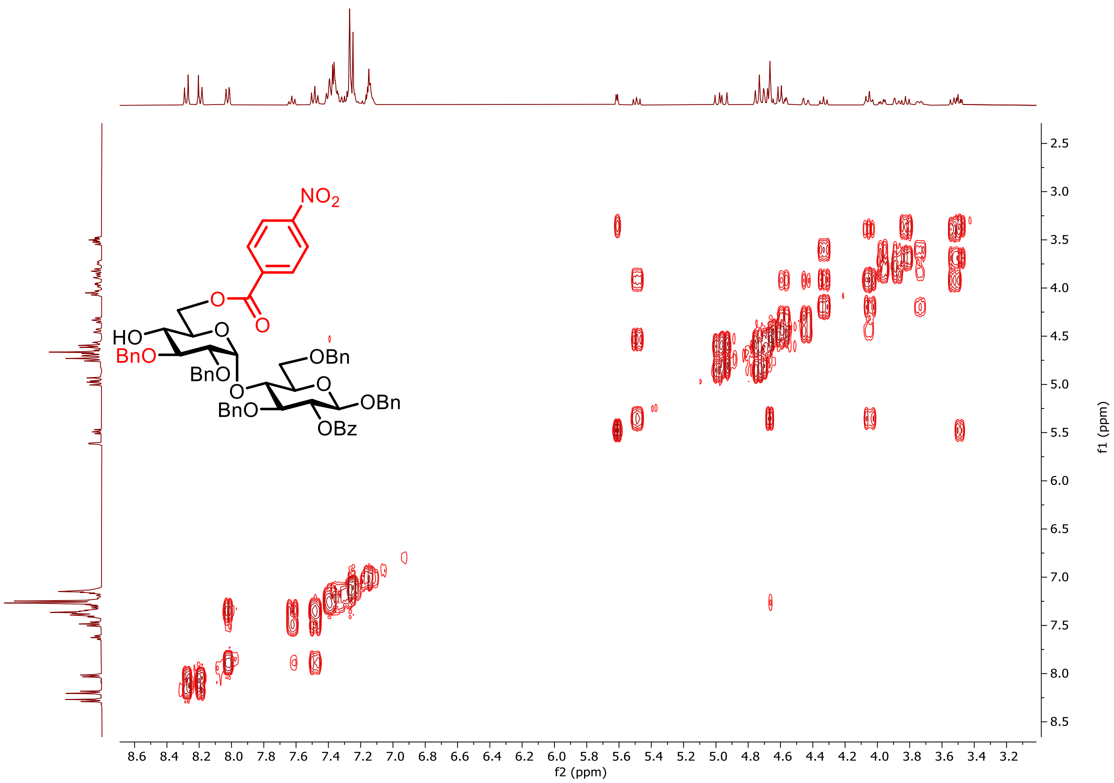

HSQC NMR of 100a ($CDCl_3$)

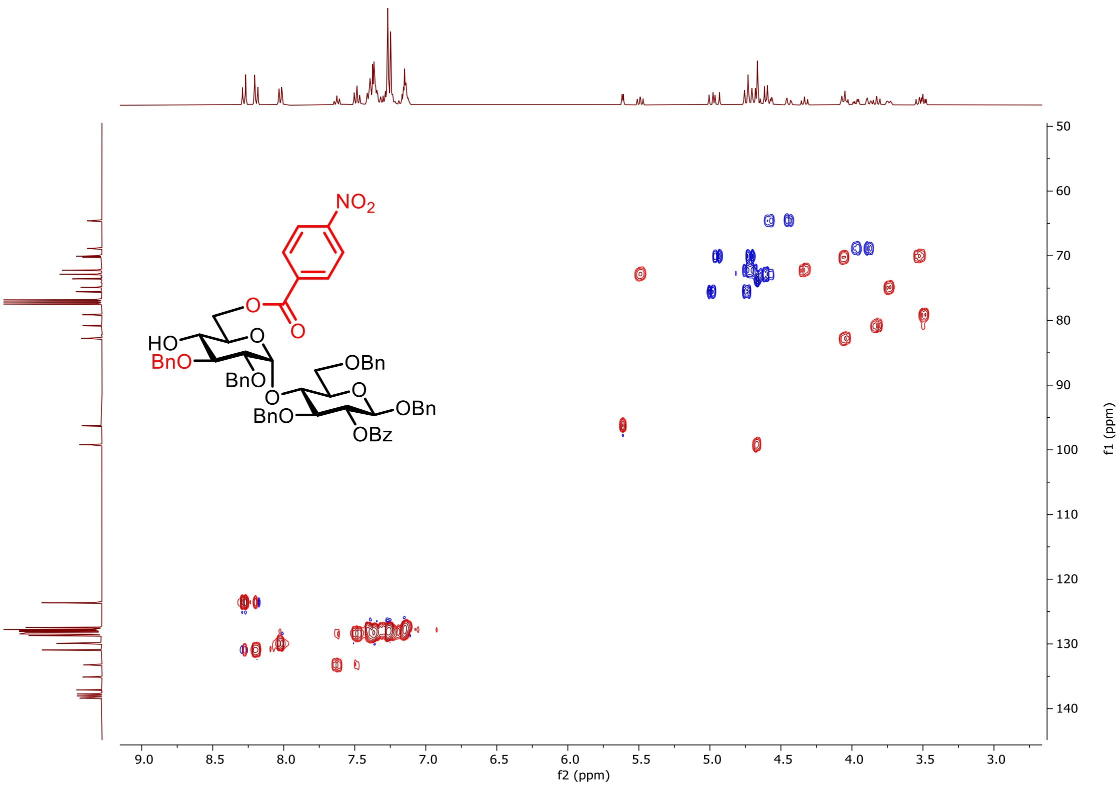

4.28 Benzyl 2,3-di-*O*-benzyl-6-*O*-triphenylacetyl-D-glucopyranosyl-(1→4)-2-*O*-benzoyl-3,6-di-*O*-benzyl-β-D-glucopyranoside, 101

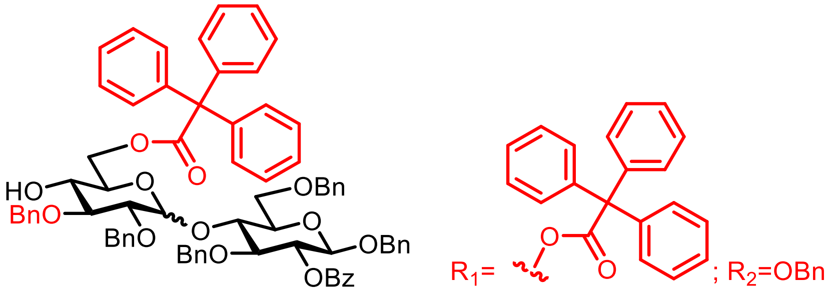

Total yield: 44% (51.3 mg). Ratio of anomer α : β = 7.5 : 1.

Spectrum data for benzyl 2,3-di-*O*-benzyl-6-*O*-triphenylacetyl-α-D-glucopyranosyl-(1→4)-2-*O*-benzoyl-3,6-di-*O*-benzyl-β-D-glucopyranoside **101a**: ^{1}H NMR (400 MHz, $CDCl_3$) δ 8.05 – 7.97 (m, 2H), 7.65 – 7.58 (m, 1H), 7.47 (appt, *J* = 7.7 Hz, 2H), 7.40 – 7.27 (m, 11H), 7.27 – 7.09 (m, 29H), 5.53 (d, *J* = 3.5 Hz, 1H), 5.47 (dd, *J* = 9.1, 7.7 Hz, 1H), 4.90 (appt, *J* = 11.6 Hz, 2H), 4.71 – 4.62 (m, 3H), 4.57 (dd, *J* = 12.1, 3.7 Hz, 5H), 4.48 (d, *J* = 12.1 Hz, 1H), 4.38 (dd, *J* = 12.1, 2.4 Hz, 1H), 4.33 (dd, *J* = 12.1, 4.2 Hz, 1H), 4.14 (appt, *J* = 9.0 Hz, 1H), 3.97 (appt, *J* = 8.8 Hz, 1H), 3.81 – 3.73 (m, 2H), 3.70 (dd, *J* = 11.1, 2.2 Hz, 1H), 3.65 (appt, *J* = 9.3 Hz, 1H), 3.55 (ddd, *J* = 9.5, 4.3, 2.2 Hz, 1H), 3.18 (dd, *J* = 9.7, 3.6 Hz, 1H), 3.02 (appt, *J* = 9.4 Hz, 1H), 2.29 (br. s, 1H); ^{13}C NMR (101 MHz, $CDCl_3$) δ 173.95, 165.26, 142.76, 138.69, 138.15, 137.81, 137.70, 137.17, 133.15, 130.27, 129.90, 129.87, 128.53, 128.42, 128.40, 128.36, 128.32, 128.26, 127.88, 127.78, 127.73, 127.69, 127.66, 127.59, 127.44, 126.98, 99.27, 95.97, 82.88, 80.38, 78.39, 75.04, 74.82, 73.45, 72.46, 72.37, 71.57, 71.48, 70.30, 69.98, 69.76, 68.97, 67.57, 64.13; $[\alpha]_D^{25}$ 9.30 (c = 1, $CHCl_3$); IR (neat) ν_{max} = 3528, 2874, 1729, 1268, 1060, 698 cm^{-1}; m/z ($HRMS^+$) $[M + Na]^+$ 1189.495 ($C_{74}H_{70}O_{13}Na^+$ requires 1189.471).

^{1}H NMR of crude 101 (600 MHz, $CDCl_3$)

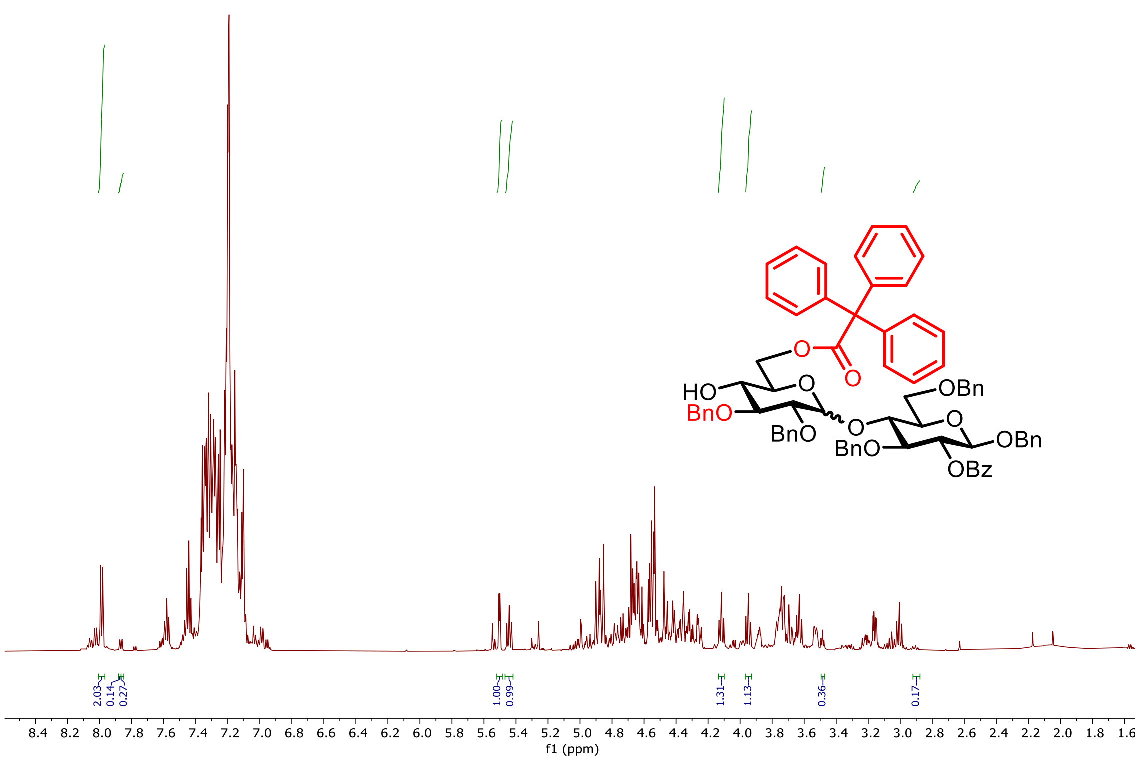

^{13}C NMR of crude 101 (151 MHz, $CDCl_3$)

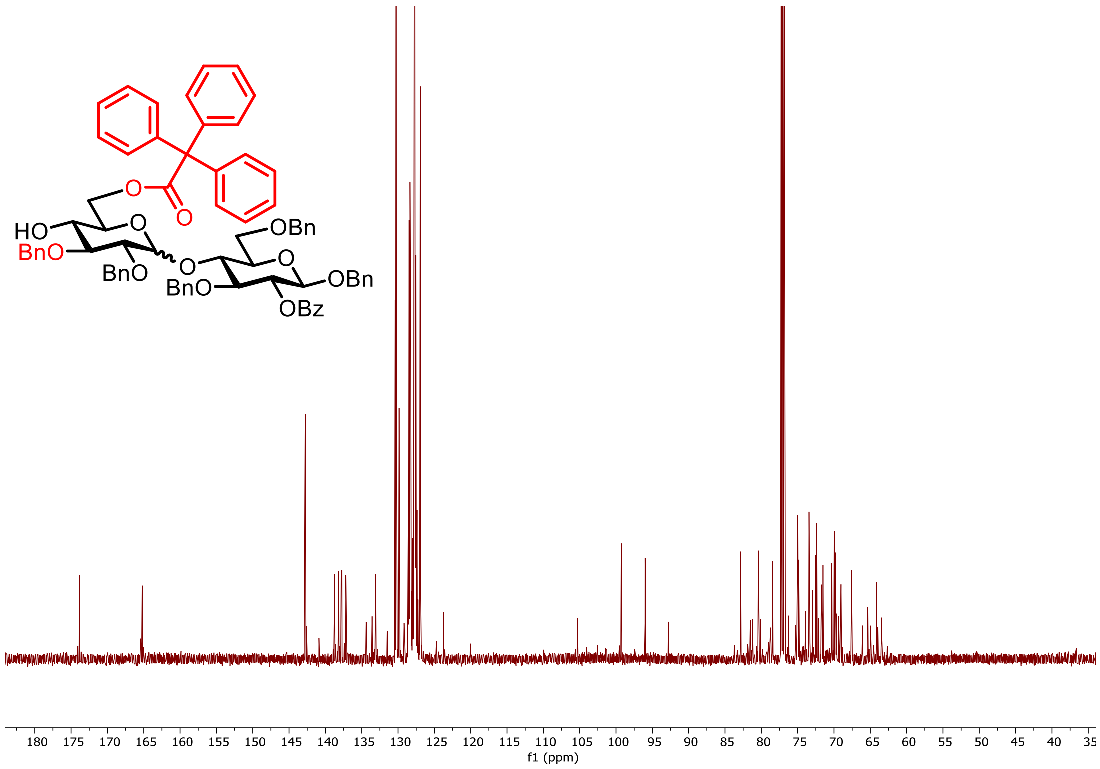

HSQC NMR of crude 101 ($CDCl_3$)

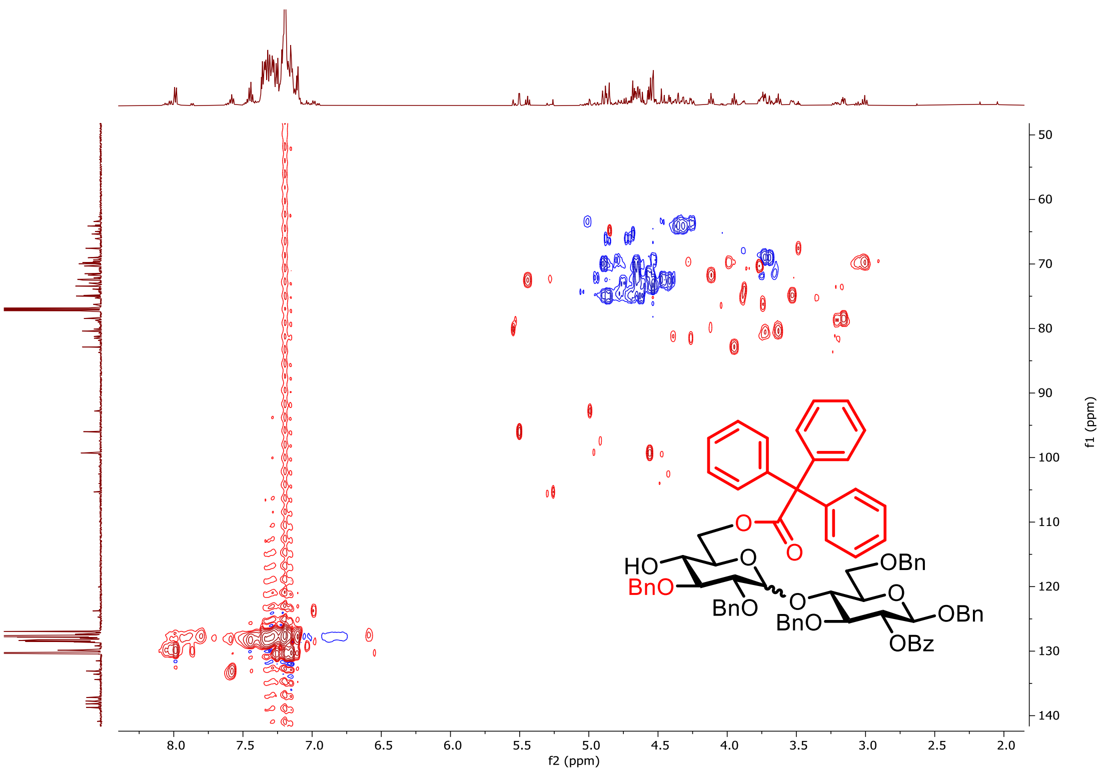

Coupled HSQC NMR of crude 101 ($CDCl_3$)

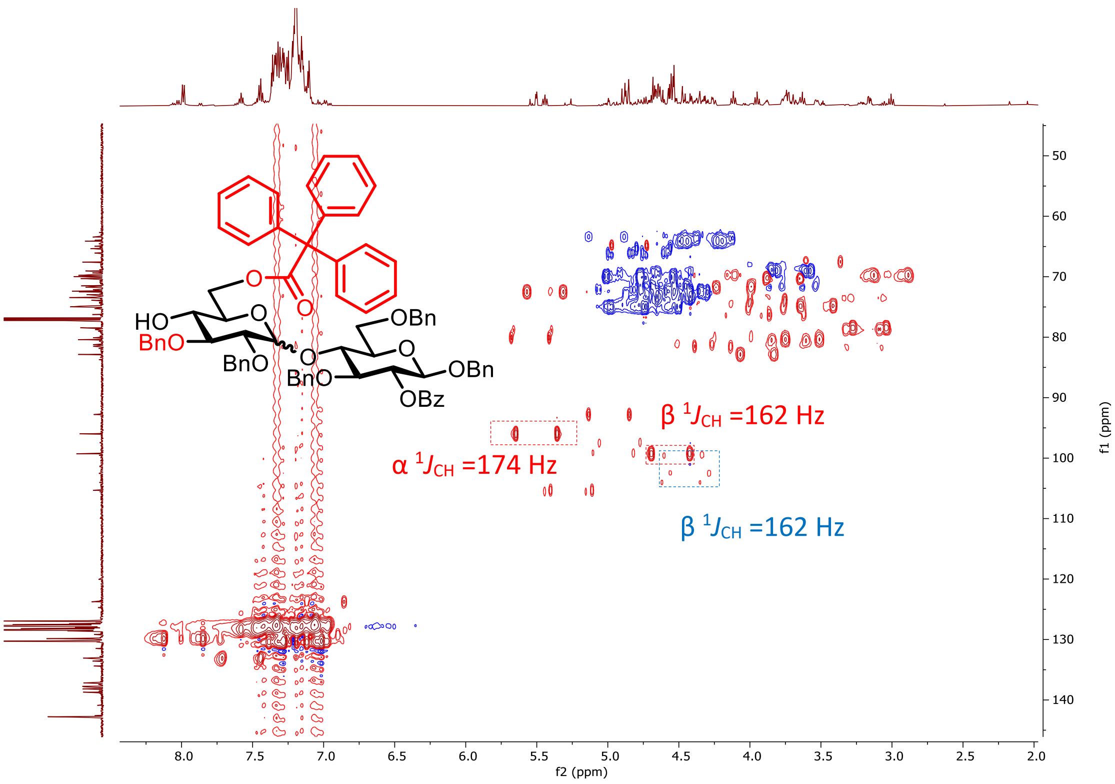

^1H NMR of 101a (400 MHz, $CDCl_3$)

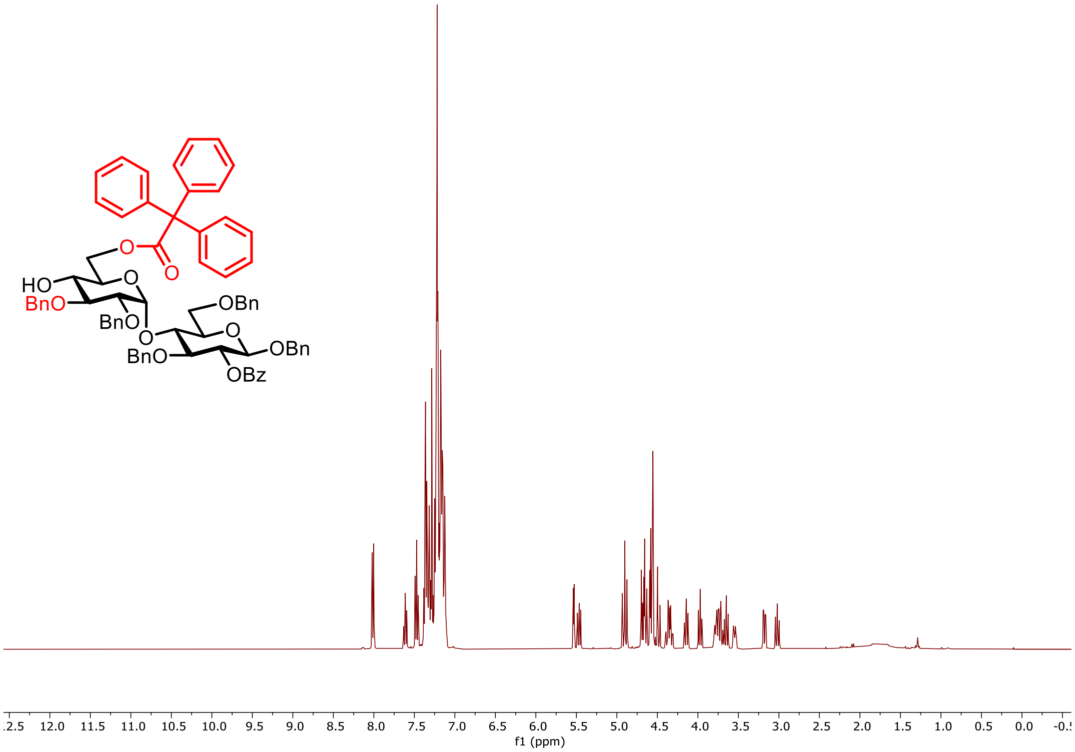

^{13}C NMR of 101a (101 MHz, $CDCl_3$)

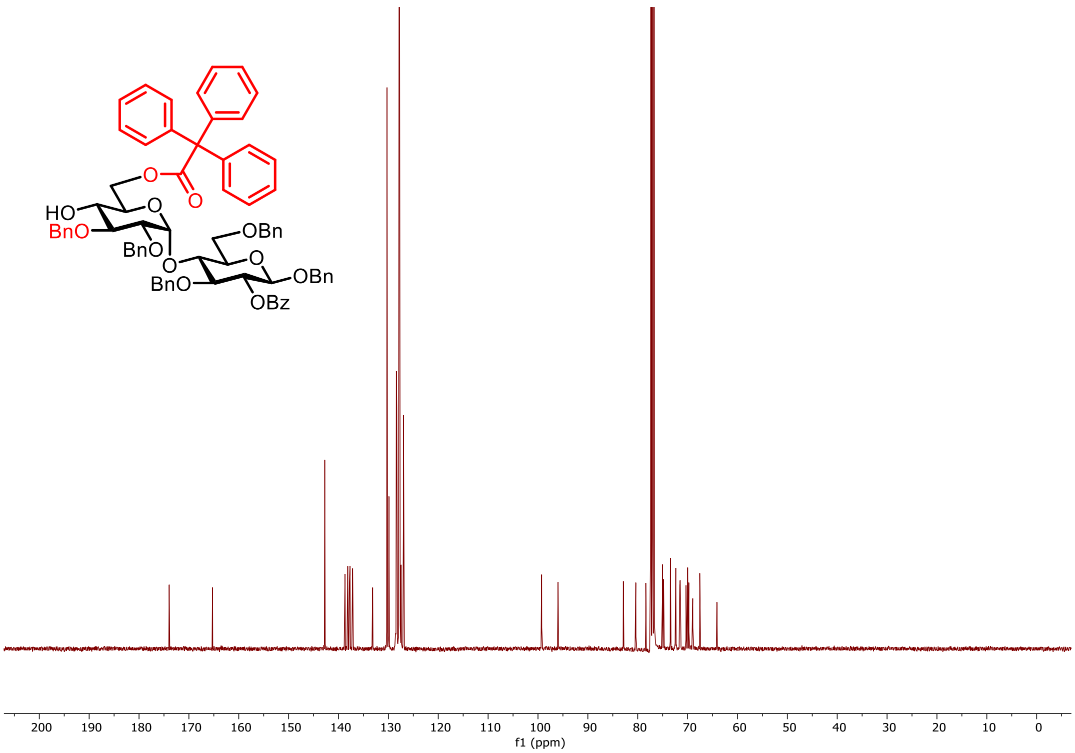

COSY NMR of 101a ($CDCl_3$)

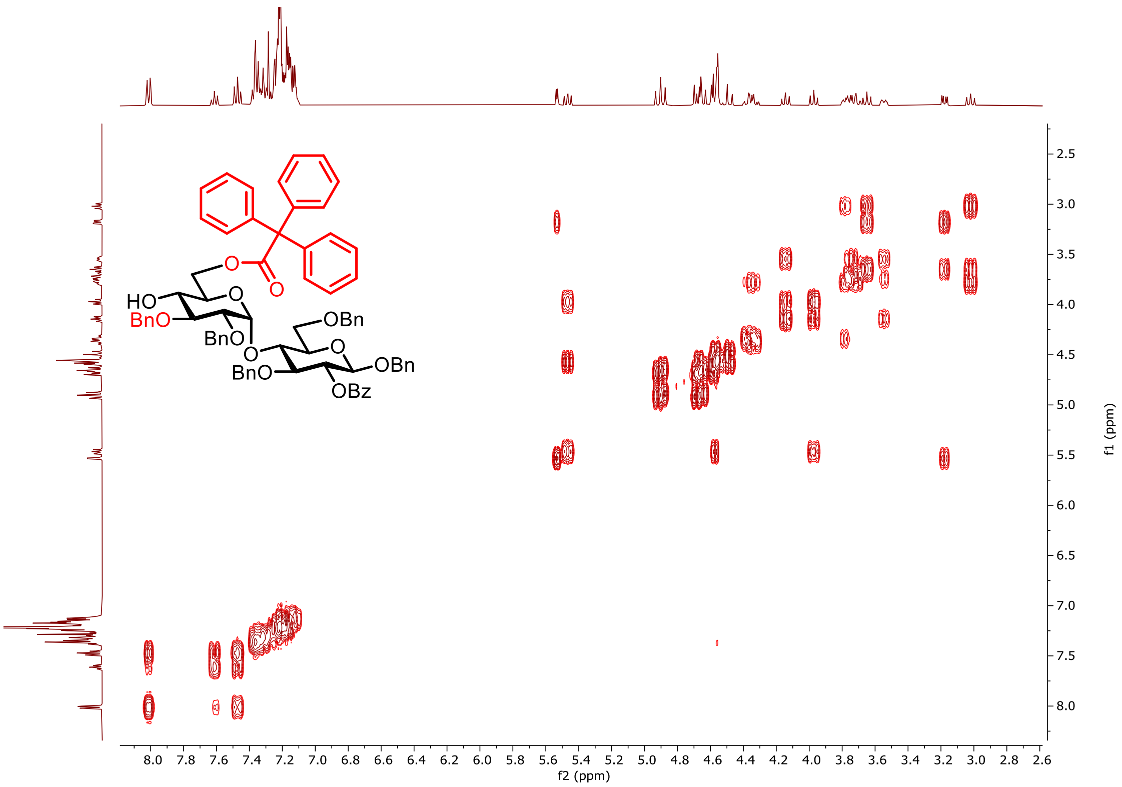

HSQC NMR of 101a ($CDCl_3$)

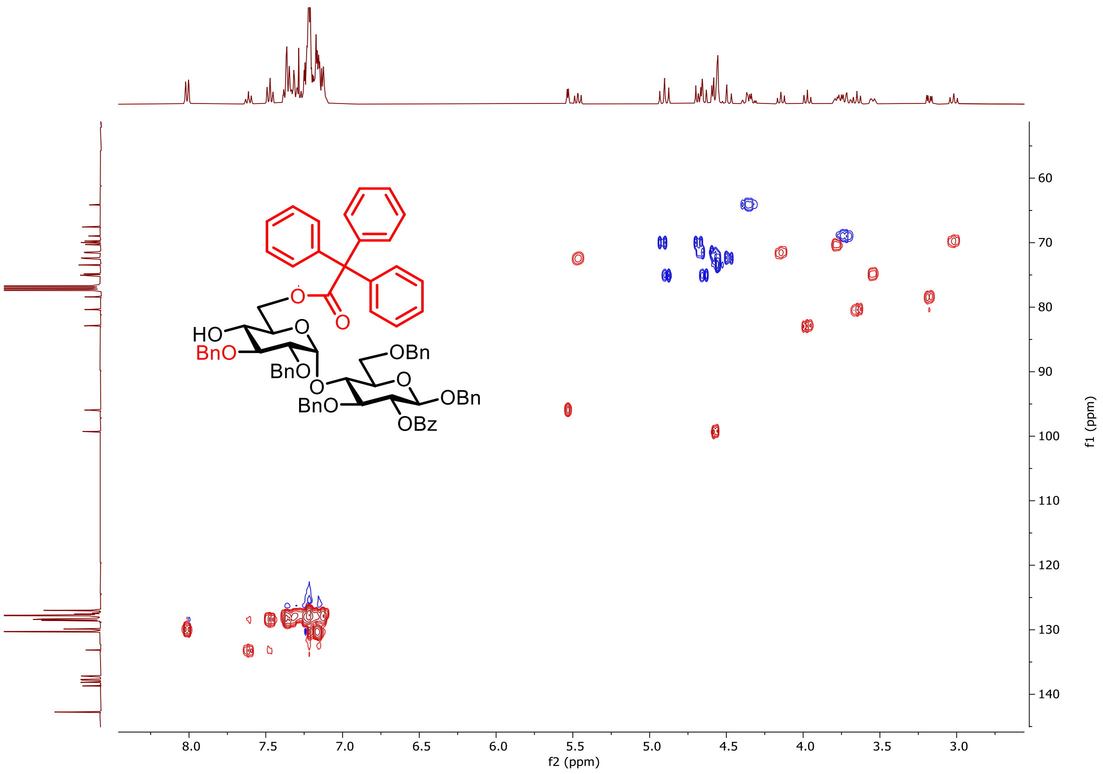

4.29 Benzyl 2-*O*-benzyl-3,6-di-*O*-acetyl-D-glucopyranosyl-(1→4)-2-*O*-benzoyl-3,6-di-*O*-benzyl-β-D-glucopyranoside, 102

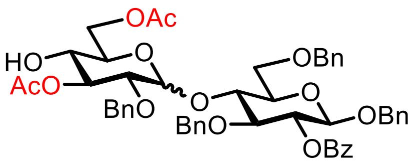

R_1=R_2=OAc

Total yield: 78% (69.5 mg). Ratio of anomer α : β = 9.8 : 1.

Spectrum data for benzyl 2-*O*-benzyl-3,6-di-*O*-acetyl-α-D-glucopyranosyl-(1→4)-2-*O*-benzoyl-3,6-di-*O*-benzyl-β-D-glucopyranoside **102a**: ^{1}H NMR (400 MHz, $CDCl_3$) δ 8.02 – 7.96 (m, 2H), 7.64 – 7.56 (m, 1H), 7.49 – 7.37 (m, 6H), 7.35 – 7.31 (m, 1H), 7.29 – 7.24 (m, 3H), 7.24 – 7.19 (m, 5H), 7.18 – 7.09 (m, 7H), 5.60 (d, *J* = 3.7 Hz, 1H), 5.48 (dd, *J* = 9.0, 7.6 Hz, 1H), 5.26 (appt, *J* = 9.6 Hz, 1H), 4.91 (d, *J* = 12.6 Hz, 1H), 4.75 – 4.63 (m, 5H), 4.61 (d, *J* = 7.6 Hz, 1H), 4.50 (d, *J* = 12.3 Hz, 1H), 4.37 (td, *J* = 8.5, 4.3 Hz, 2H), 4.24 (dd, *J* = 9.5, 8.4 Hz, 1H), 4.06 (dd, *J* = 12.3, 2.2 Hz, 1H), 4.02 – 3.92 (m, 2H), 3.90 – 3.81 (m, 2H), 3.67 (ddd, *J* = 9.5, 4.3, 2.1 Hz, 1H), 3.45 – 3.34 (m, 2H), 3.04 – 2.84 (br. s, 1H), 2.09 (s, 3H), 2.05 (s, 3H); ^{13}C NMR (101 MHz, $CDCl_3$) δ 171.73, 165.25, 138.15, 137.93, 137.67, 137.07, 133.18, 129.88, 129.78, 128.48, 128.37, 128.32, 128.19, 127.82, 127.74, 127.72, 127.69, 127.68, 127.33, 127.26, 99.22, 96.61, 82.84, 76.36, 74.87, 74.25, 73.50, 73.47, 73.24, 72.81, 72.77, 70.70, 70.11, 69.18, 68.83, 63.08, 21.07, 20.93; $[\alpha]_D^{25}$ 32.57 (c = 1, $CHCl_3$); IR (neat) ν_{max} = 3493, 2873, 1732, 1454, 1267, 1233, 1064, 698 cm^{-1}; m/z (HRMS$^+$) $[M + Na]^+$ 913.3550 ($C_{51}H_{54}O_{14}Na^+$ requires 913.3406).

^{1}H NMR of crude 102 (600 MHz, $CDCl_3$)

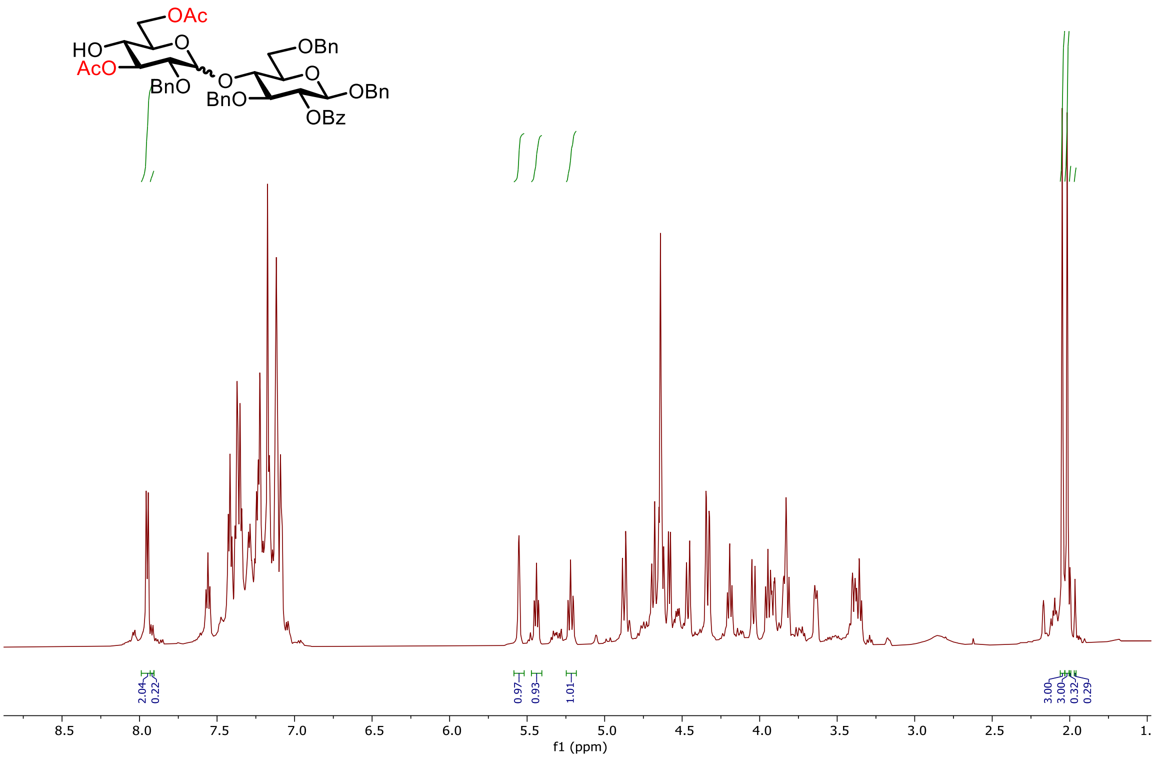

^{13}C NMR of crude 102 (151 MHz, $CDCl_3$)

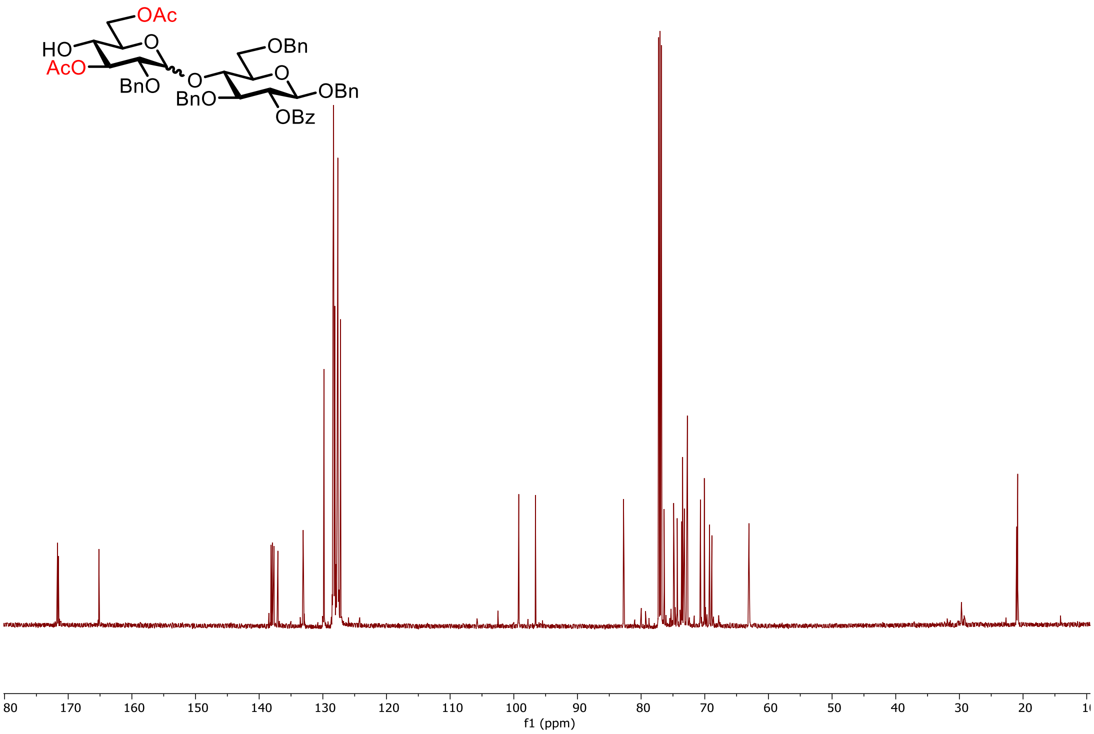

HSQC NMR of crude 102 ($CDCl_3$)

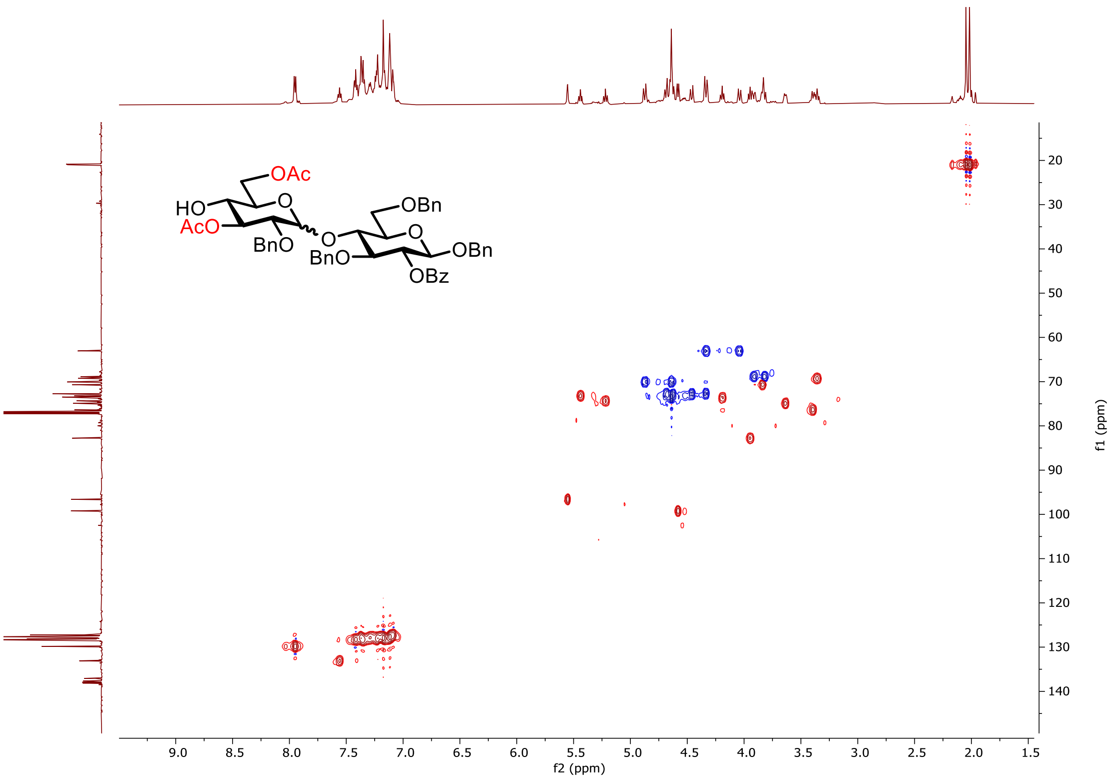

Coupled HSQC NMR of crude 102 ($CDCl_3$)

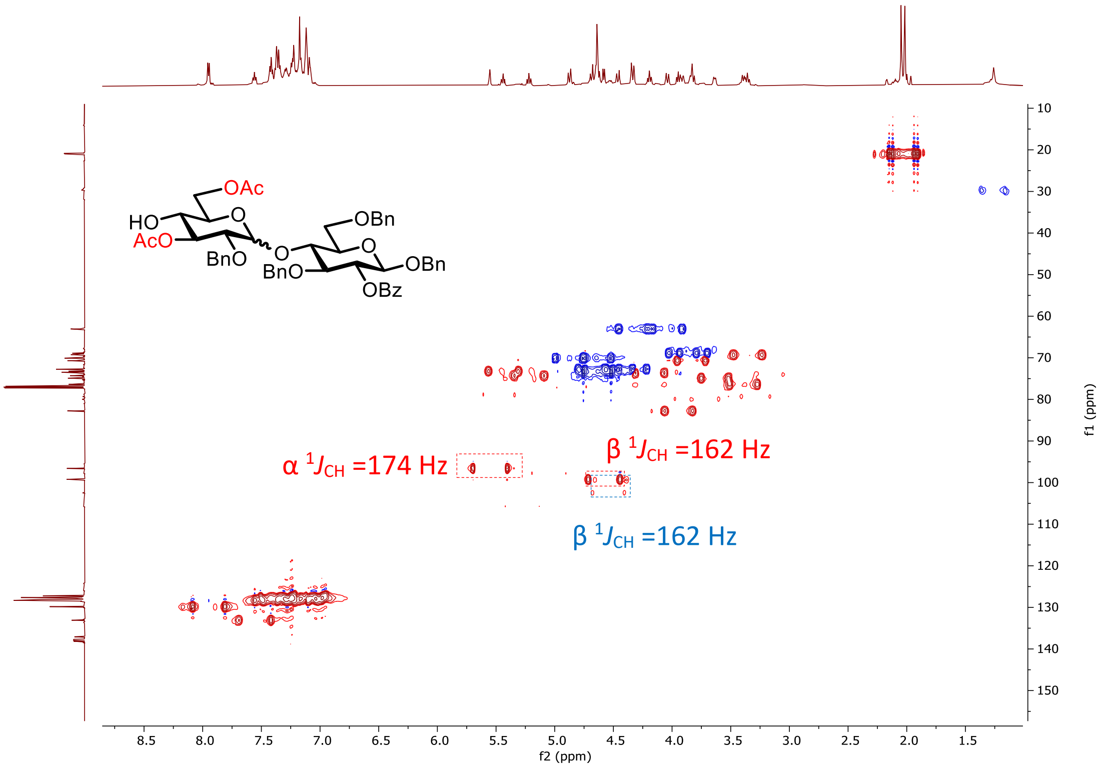

^{1}H NMR of 102a (400 MHz, $CDCl_3$)

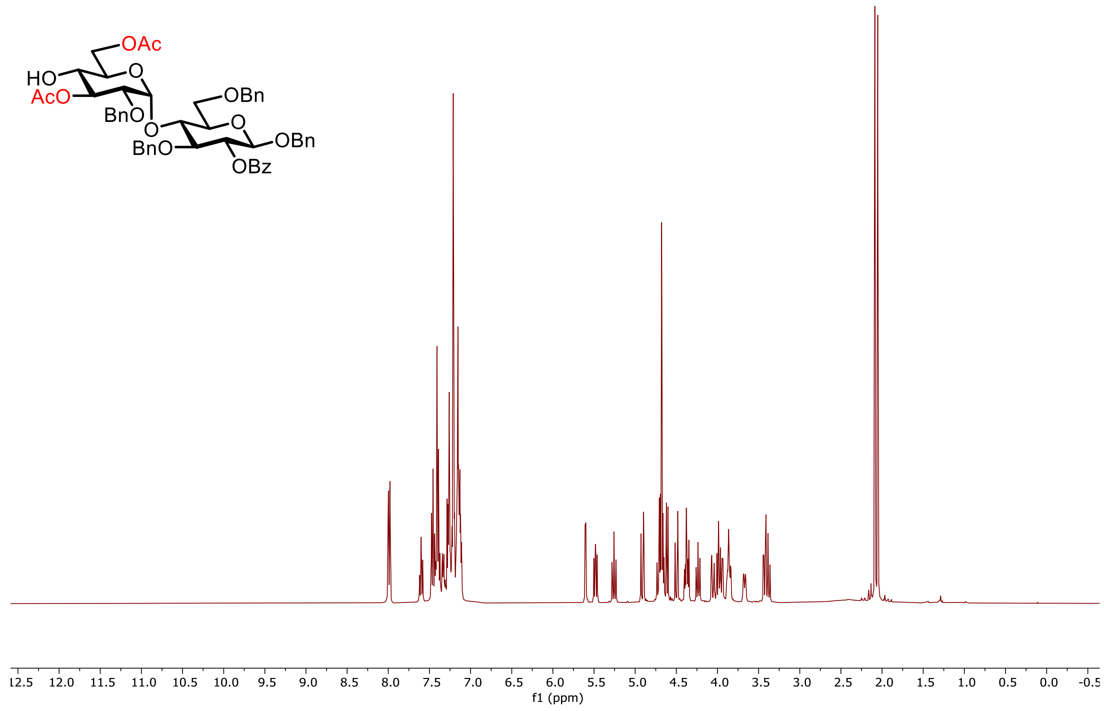

^{13}C NMR of 102a (101 MHz, $CDCl_3$)

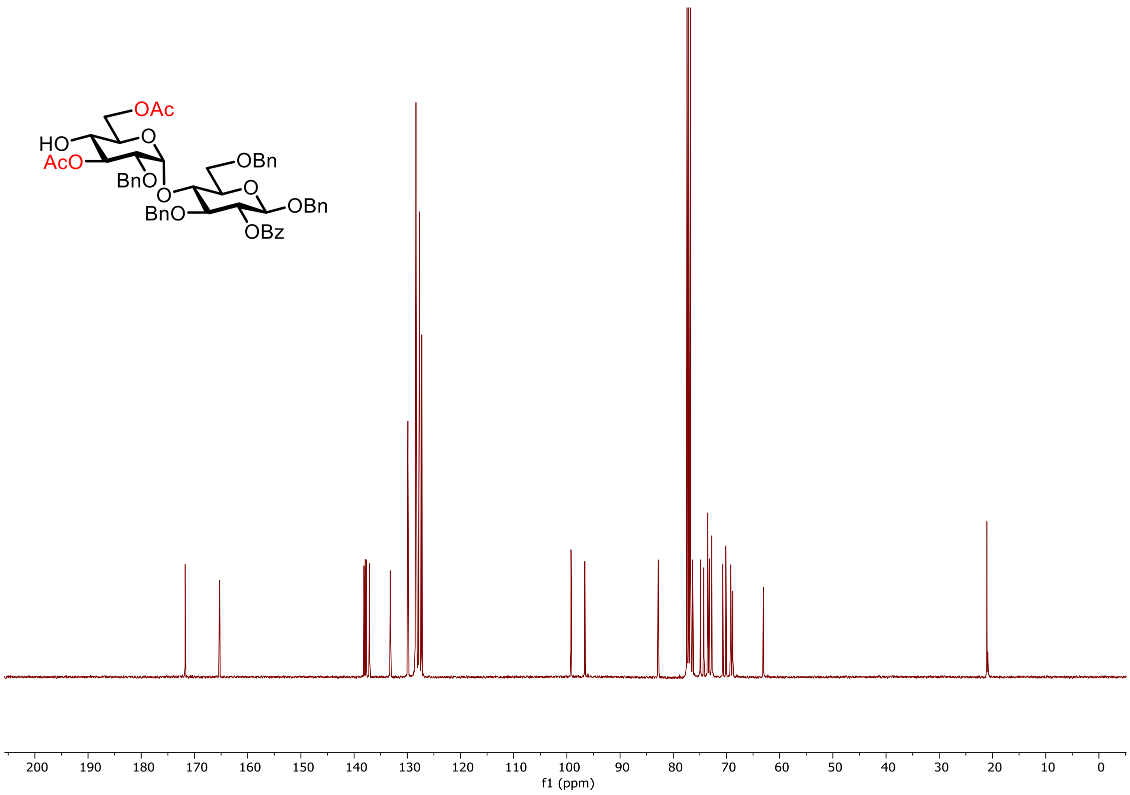

COSY NMR of 102a ($CDCl_3$)

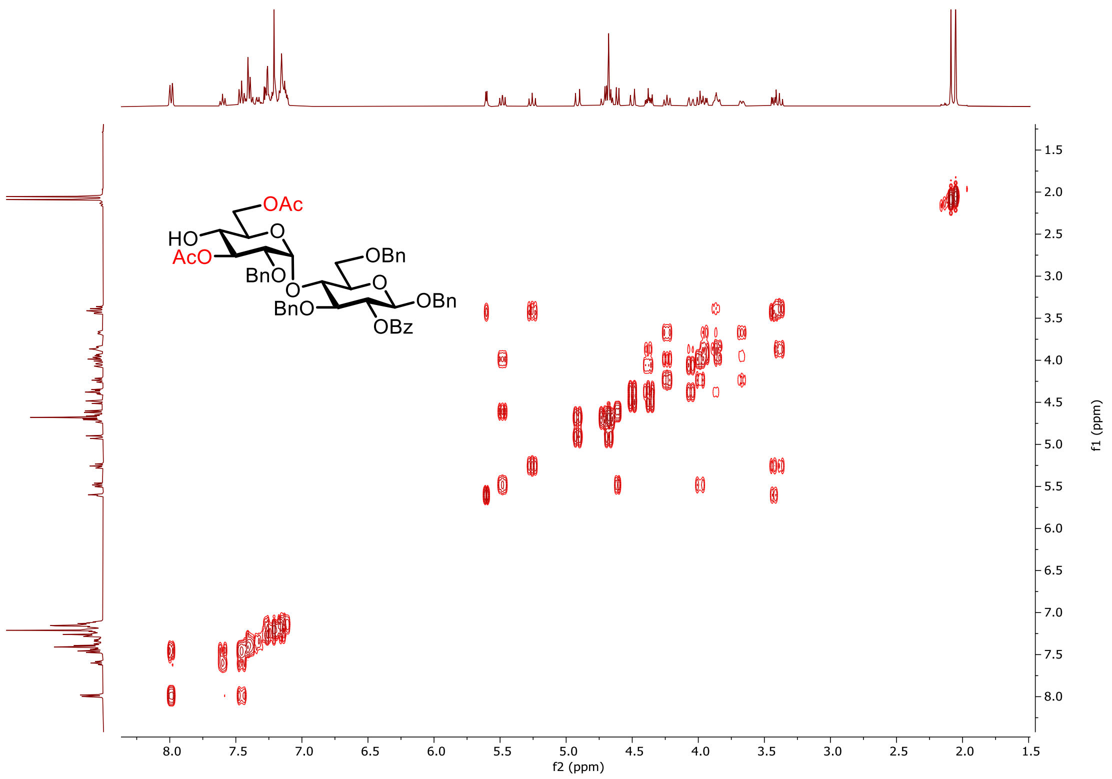

HSQC NMR of 102a ($CDCl_3$)

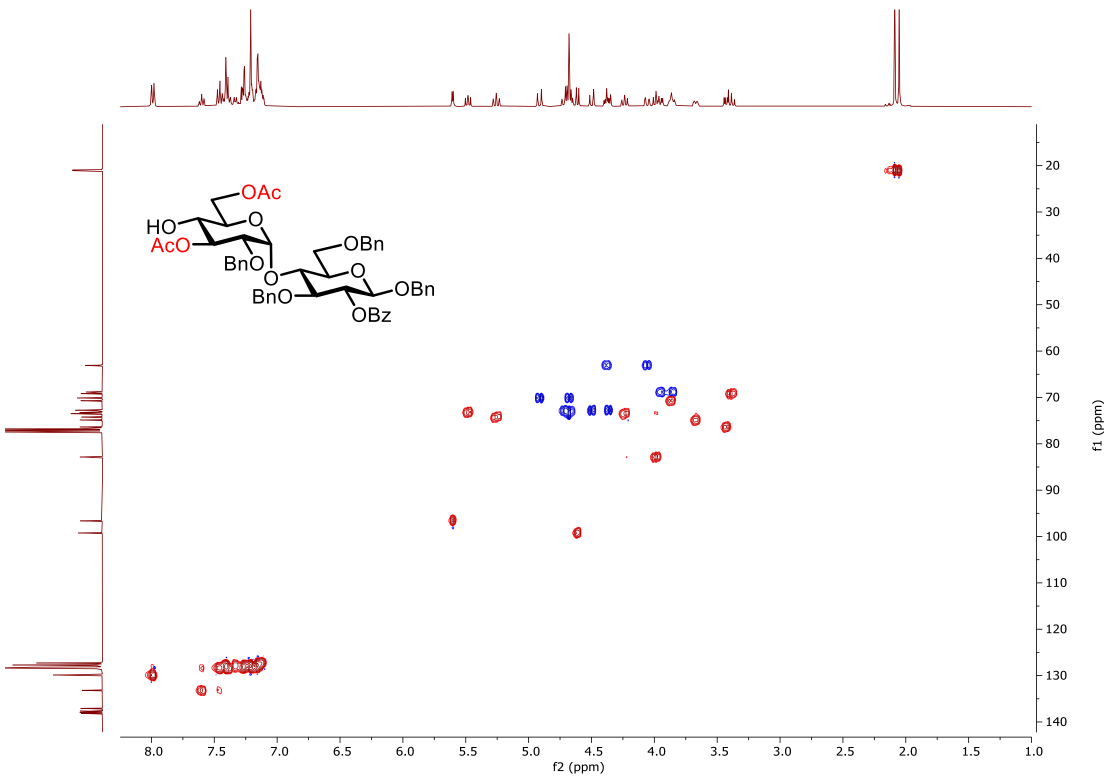

4.30 Benzyl 2-*O*-benzyl-3,6-di-*O*-pivaloyl-D-glucopyranosyl-(1→4)-2-*O*-benzoyl-3,6-di-*O*-benzyl-β-D-glucopyranoside, 103

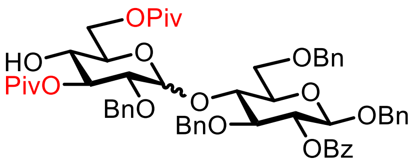

R_1=R_2=OPiv

Total yield: 62% (60.4 mg). Ratio of anomer α : β > 10 : 1.

Spectrum data for benzyl 2-*O*-benzyl-3,6-di-*O*-pivaloyl-α-D-glucopyranosyl-(1→4)-2-*O*-benzoyl-3,6-di-*O*-benzyl-β-D-glucopyranoside **103a**: ^{1}H NMR (400 MHz, $CDCl_3$) δ 8.01 – 7.96 (m, 2H), 7.64 – 7.56 (m, 1H), 7.48 – 7.43 (m, 2H), 7.42 – 7.30 (m, 6H), 7.26 – 7.17 (m, 8H), 7.17 – 7.08 (m, 6H), 5.52 (d, *J* = 3.6 Hz, 1H), 5.46 (dd, *J* = 9.0, 7.7 Hz, 1H), 5.25 (dd, *J* = 10.0, 9.2 Hz, 1H), 4.90 (d, *J* = 12.6 Hz, 1H), 4.75 – 4.56 (m, 6H), 4.43 (s, 2H), 4.31 – 4.21 (m, 2H), 4.18 (dd, *J* = 12.1, 2.3 Hz, 1H), 3.98 – 3.87 (m, 3H), 3.86 – 3.82 (m, 1H), 3.63 (ddd, *J* = 9.5, 4.3, 2.2 Hz, 1H), 3.42 (dd, *J* = 10.0, 3.6 Hz, 1H), 3.36 (appt, *J* = 9.6 Hz, 1H), 2.92 (br. s, 1H), 1.23 (s, 9H), 1.22 (s, 9H); ^{13}C NMR (101 MHz, $CDCl_3$) δ 179.87, 178.96, 165.25, 138.12, 137.92, 137.45, 137.07, 133.15, 129.88, 129.81, 128.44, 128.35, 128.31, 128.10, 127.76, 127.72, 127.69, 127.67, 127.43, 127.28, 99.21, 96.11, 82.69, 76.42, 74.86, 74.41, 73.41, 72.96, 72.83, 72.71, 72.26, 71.02, 70.05, 70.01, 68.87, 63.16, 38.99, 38.95, 27.28, 27.16; $[\alpha]_D^{25}$ 26.81 (c = 1, $CHCl_3$); IR (neat) ν_{max} = 3485, 2873, 1730, 1455, 1269, 1233, 1062, 698 cm^{-1}; m/z (HRMS$^+$) [M + Na]$^+$ 997.4512 ($C_{57}H_{66}O_{14}Na^+$ requires 997.4345).

^{1}H NMR of crude 103 (600 MHz, $CDCl_3$)

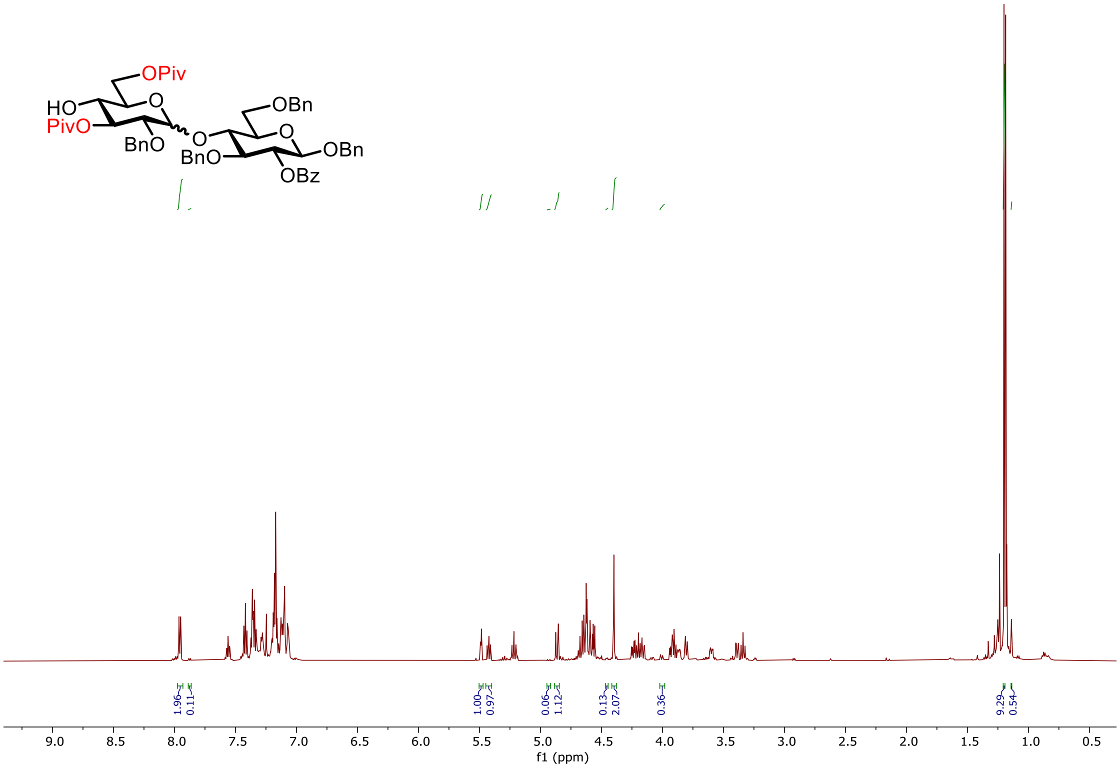

^{13}C NMR of crude 103 (151 MHz, $CDCl_3$)

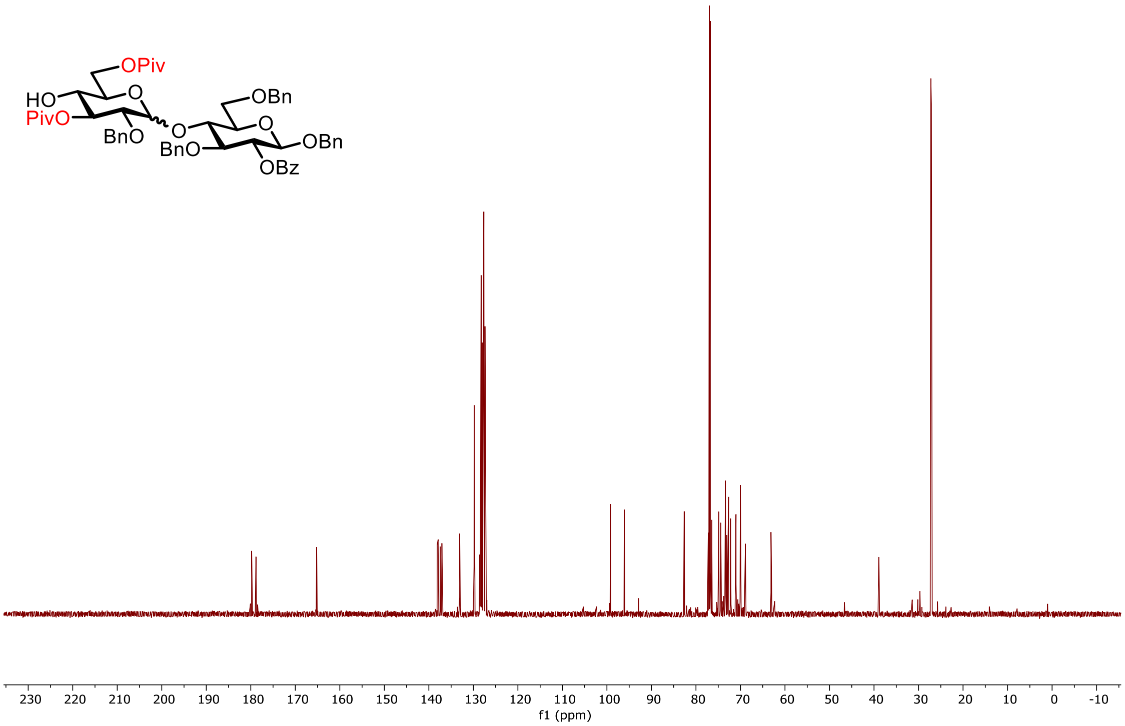

HSQC NMR of crude 103 ($CDCl_3$)

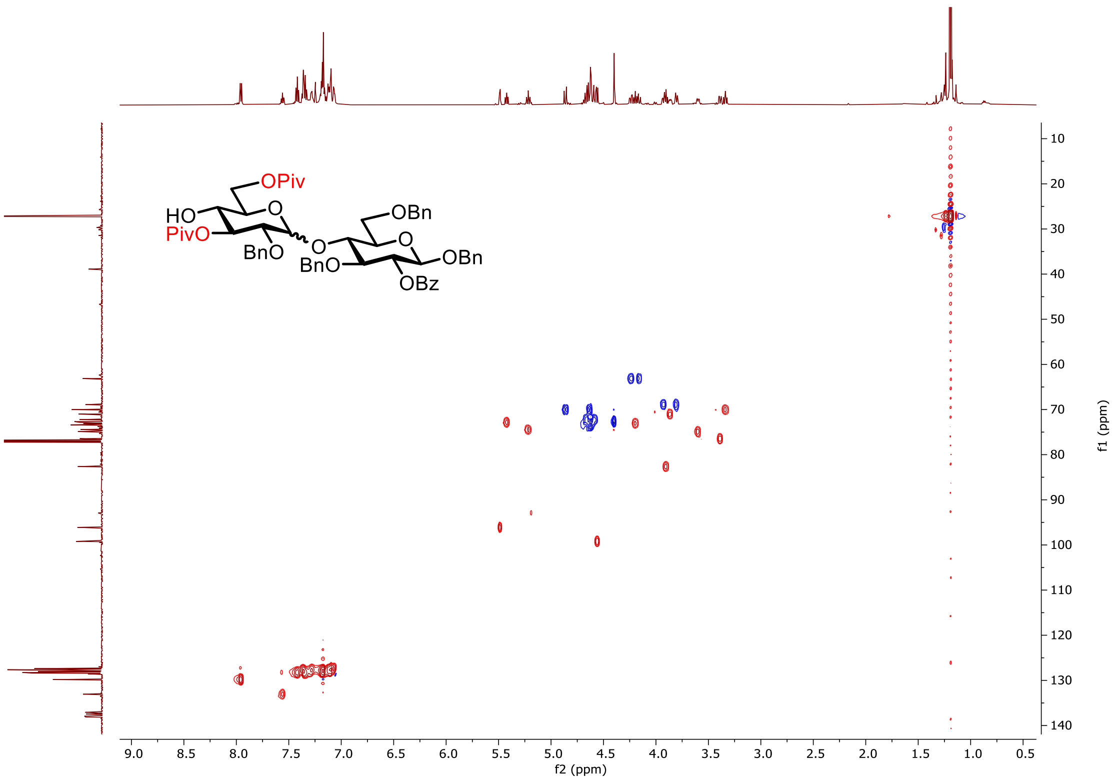

Coupled HSQC NMR of crude 103 ($CDCl_3$)

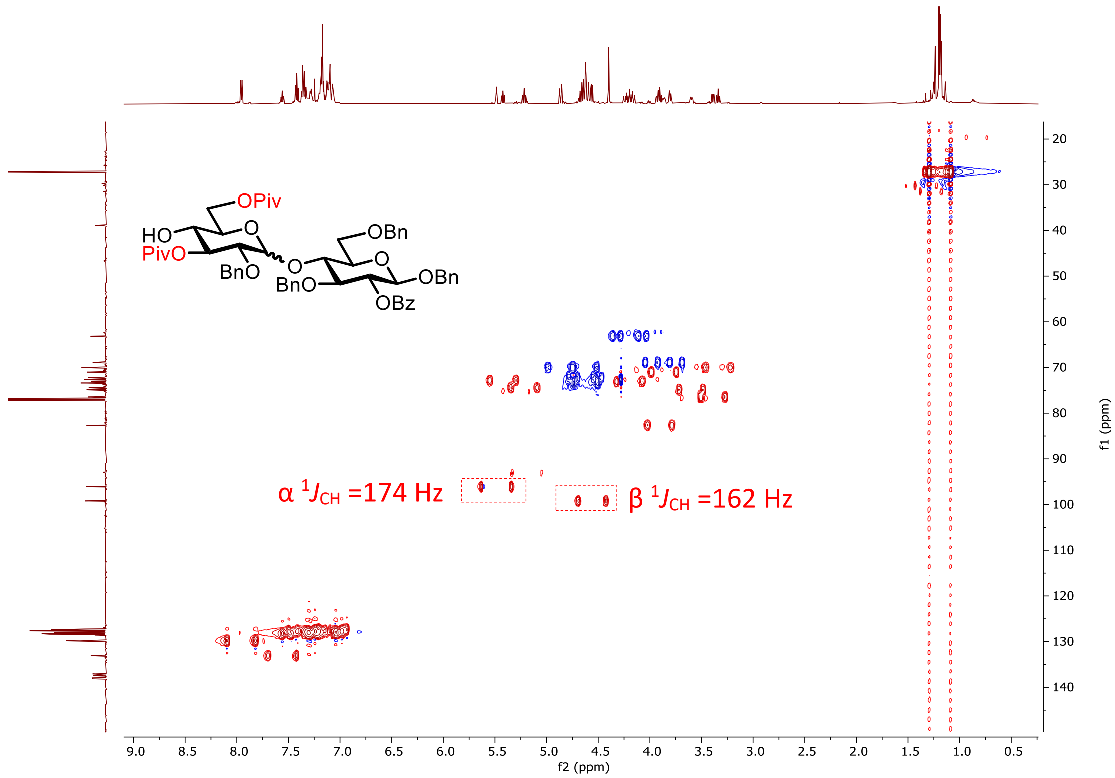

^{1}H NMR of 103a (400 MHz, $CDCl_3$)

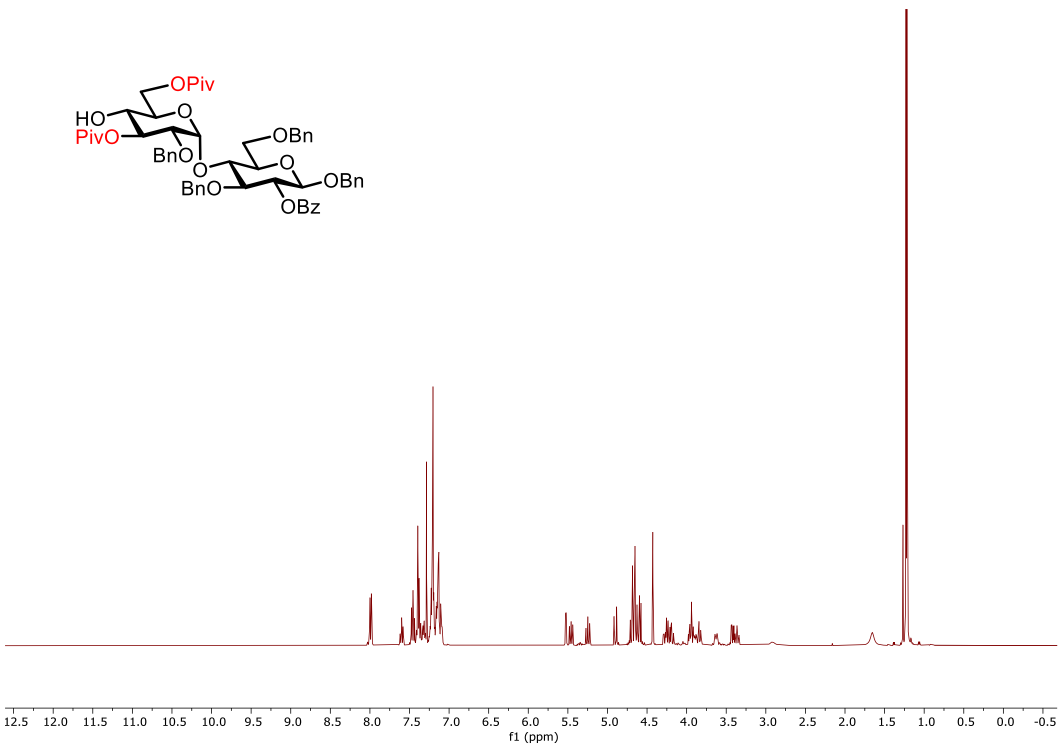

^{13}C NMR of 103a (101 MHz, $CDCl_3$)

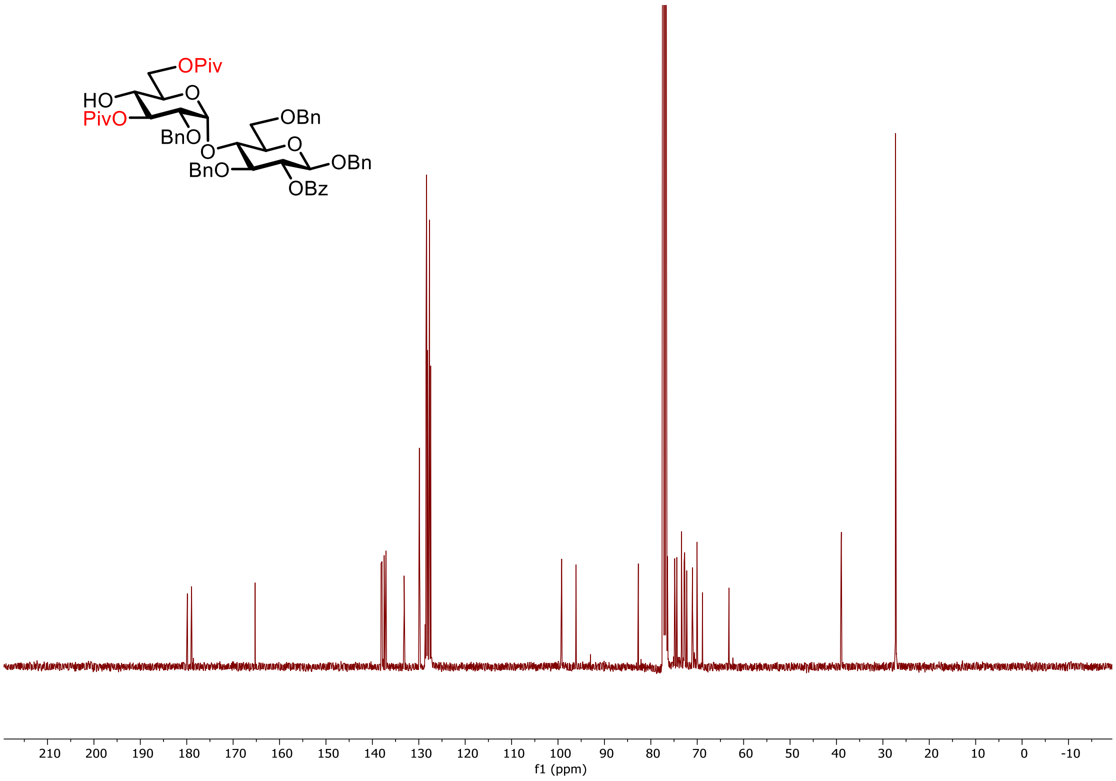

COSY NMR of 103a ($CDCl_3$)

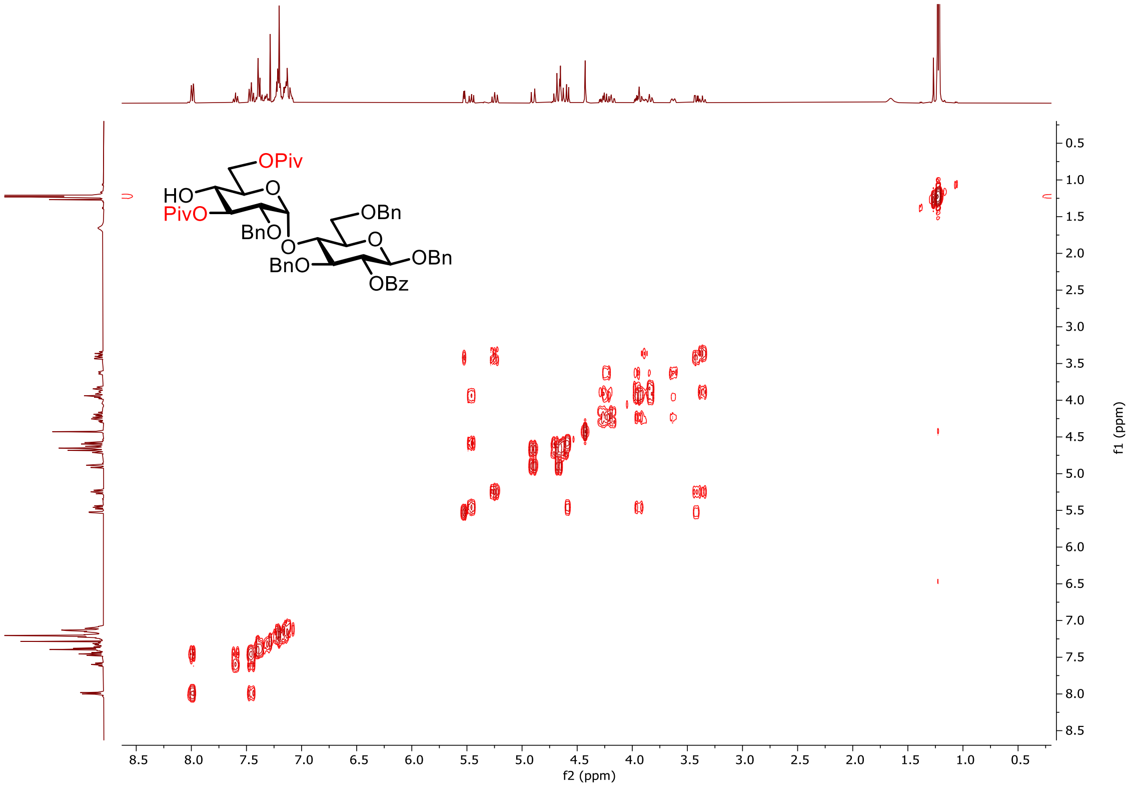

HSQC NMR of 103a ($CDCl_3$)

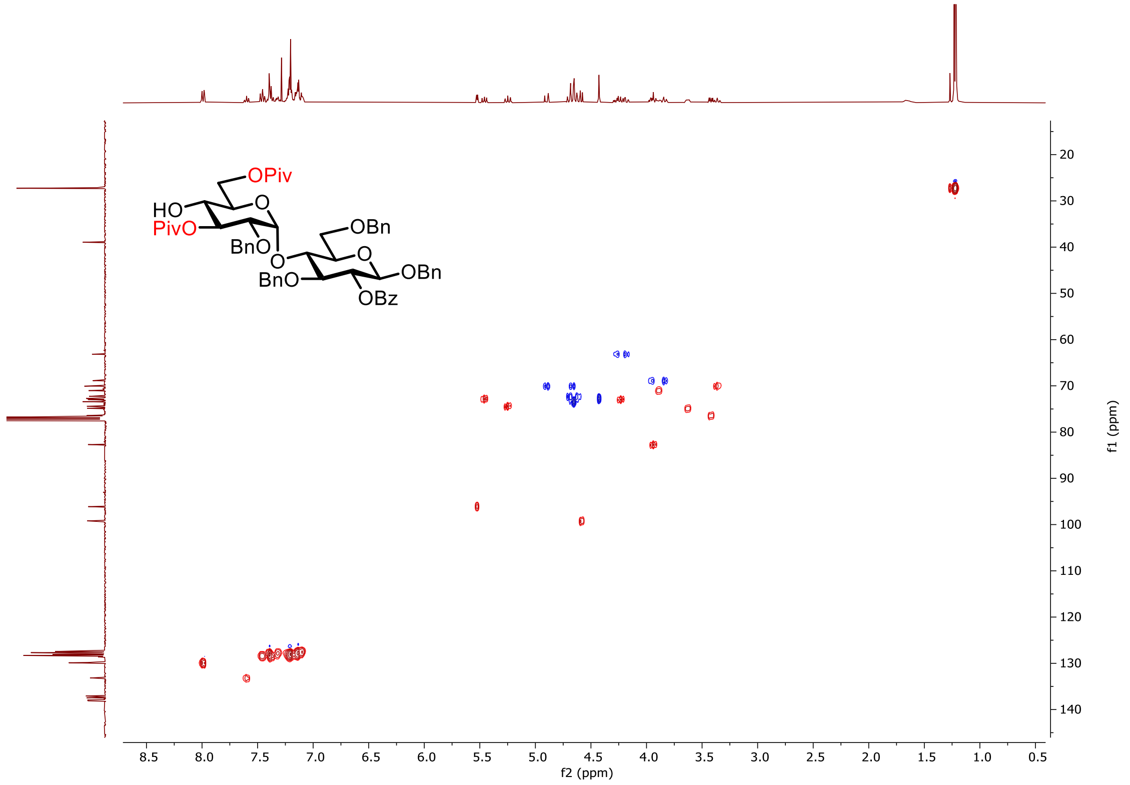

4.31 Benzyl 2-*O*-benzyl-3,6-di-*O*-benzoyl-D-glucopyranosyl-(1→4)-2-*O*-benzoyl-3,6-di-*O*-benzyl-β-D-glucopyranoside, 104

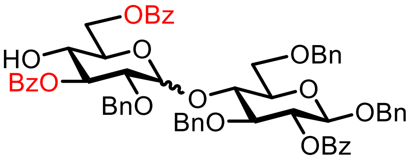

R_1=R_2=OBz

Total yield: 83% (84.2 mg). Ratio of anomer α : β > 10 : 1.

Spectrum data for benzyl 2-*O*-benzyl-3,6-di-*O*-benzoyl-α-D-glucopyranosyl-(1→4)-2-*O*-benzoyl-3,6-di-*O*-benzyl-β-D-glucopyranoside **104a**: ^{1}H NMR (400 MHz, $CDCl_3$) δ 8.13 – 8.07 (m, 2H), 8.03 (td, *J* = 7.8, 7.3, 1.4 Hz, 4H), 7.66 – 7.57 (m, 3H), 7.52 – 7.36 (m, 10H), 7.34 – 7.19 (m, 6H), 7.18 – 7.07 (m, 8H), 7.03 (dt, *J* = 6.9, 1.5 Hz, 2H), 5.67 (d, *J* = 3.6 Hz, 1H), 5.60 (appt, *J* = 9.6 Hz, 1H), 5.51 (dd, *J* = 9.0, 7.7 Hz, 1H), 4.93 (d, *J* = 12.6 Hz, 1H), 4.80 – 4.63 (m, 7H), 4.49 (d, *J* = 12.3 Hz, 1H), 4.43 – 4.31 (m, 3H), 4.11 (ddd, *J* = 10.1, 4.5, 2.3 Hz, 1H), 4.09 – 3.98 (m, 2H), 3.88 (dd, *J* = 11.2, 2.1 Hz, 1H), 3.70 (ddd, *J* = 9.6, 4.1, 2.1 Hz, 1H), 3.66 – 3.55 (m, 2H), 2.88 (br. s, 1H); ^{13}C NMR (101 MHz, $CDCl_3$) δ 167.14, 167.13, 165.29, 138.16, 137.95, 137.39, 137.13, 133.40, 133.34, 133.18, 129.96, 129.93, 129.91, 129.83, 129.70, 129.64, 128.51, 128.46, 128.43, 128.39, 128.35, 128.28, 128.17, 127.89, 127.77, 127.75, 127.73, 127.70, 127.49, 127.32, 99.31, 96.36, 82.75, 76.24, 74.95, 74.78, 73.49, 73.21, 73.00, 72.49, 72.41, 71.04, 70.11, 69.51, 68.80, 63.67; $[\alpha]_D^{25}$ 54.62 (c = 1, $CHCl_3$); IR (neat) ν_{max} = 3494, 2873, 1723, 1453, 1269, 1063, 711 cm^{-1}; m/z (HRMS$^+$) $[M + Na]^+$ 1037.390 ($C_{61}H_{58}O_{14}Na^+$ requires 1037.371).

^{1}H NMR of crude 104 (600 MHz, $CDCl_3$)

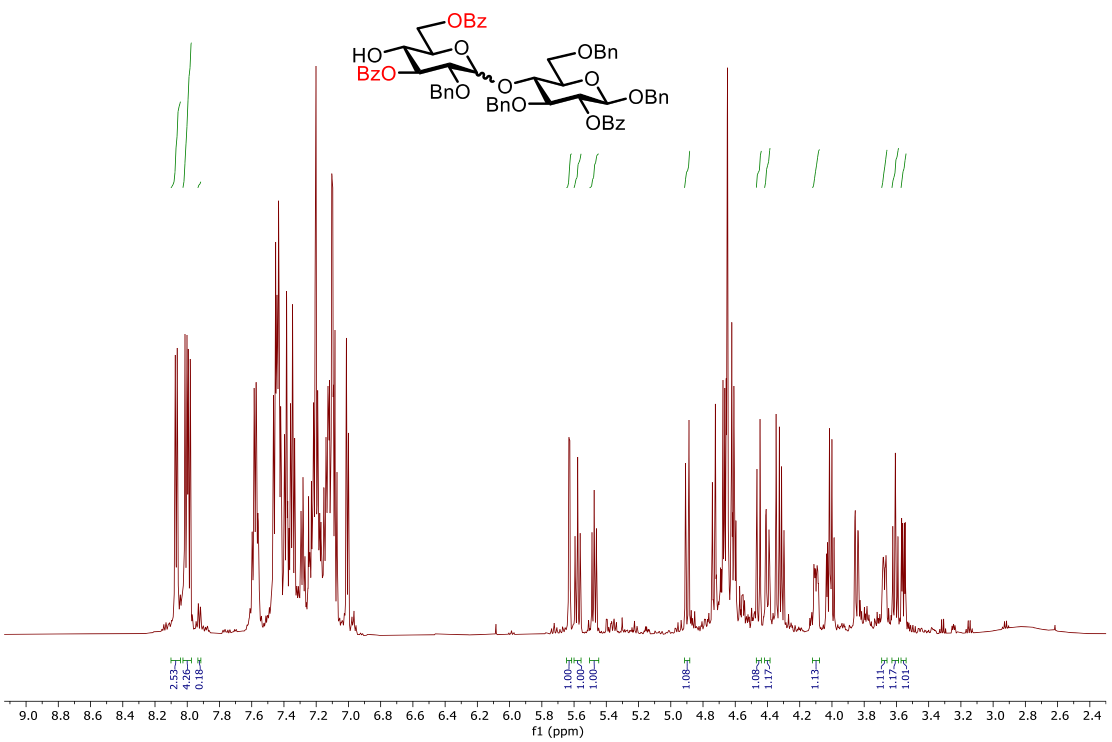

^{13}C NMR of crude 104 (151 MHz, $CDCl_3$)

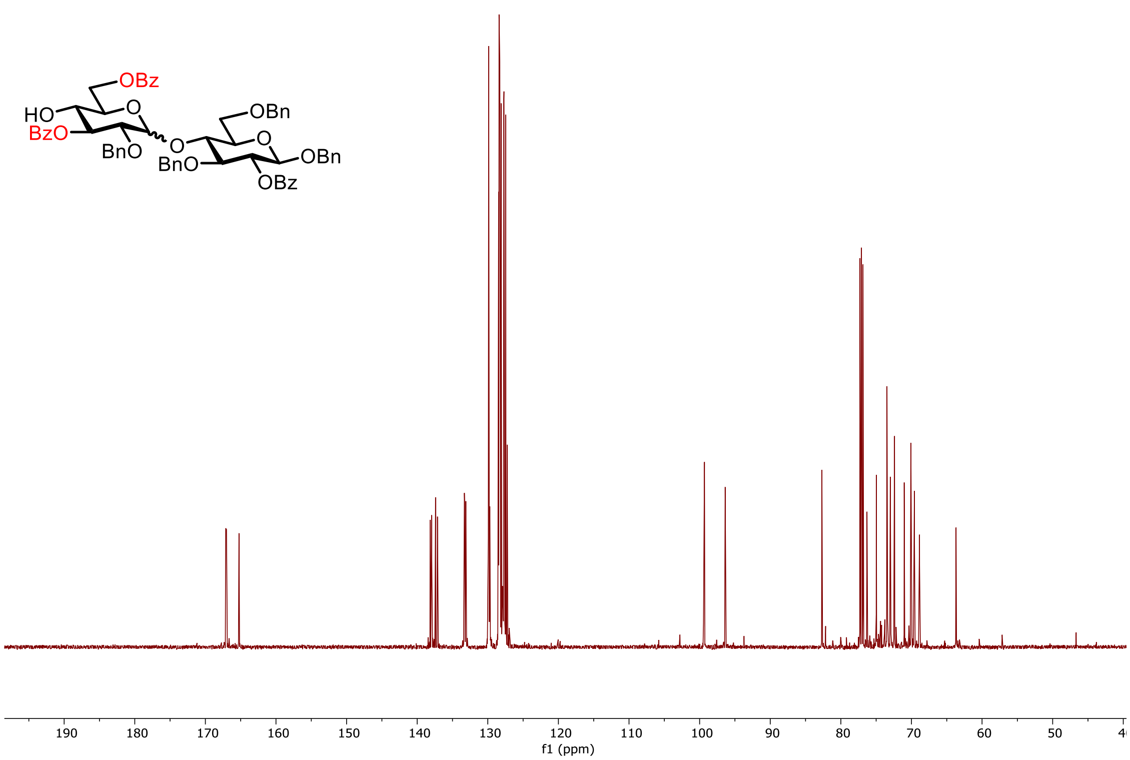

HSQC NMR of crude 104 ($CDCl_3$)

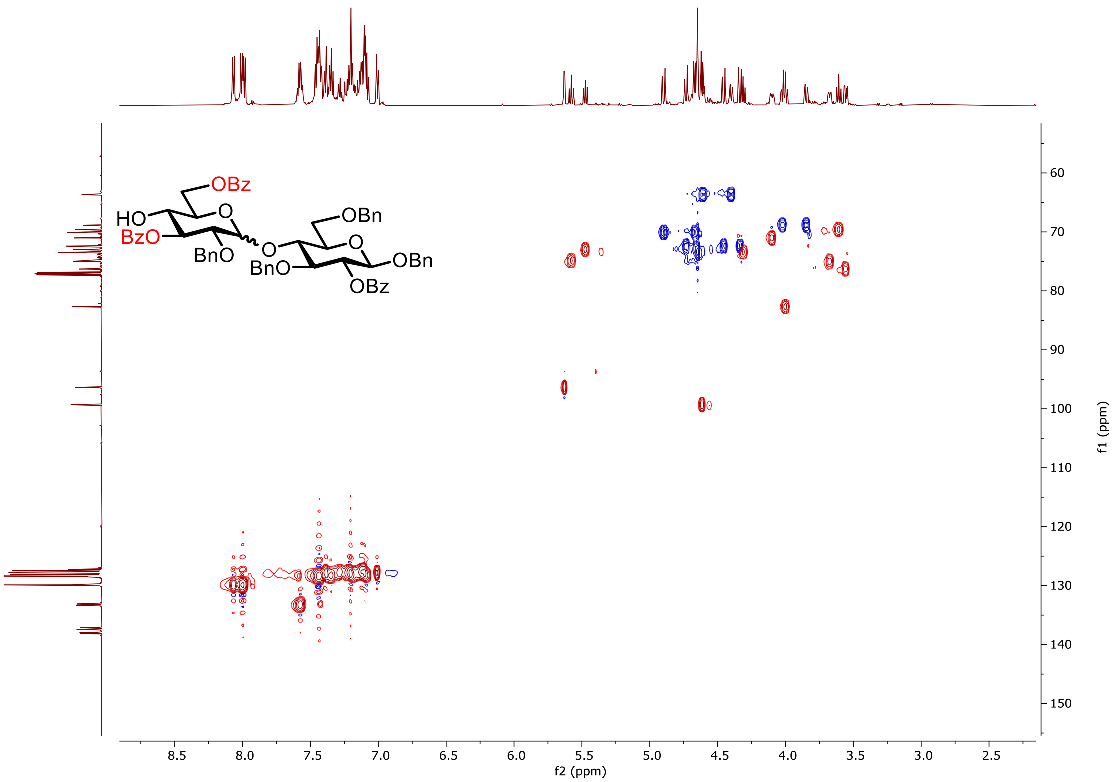

Coupled HSQC NMR of crude 104 ($CDCl_3$)

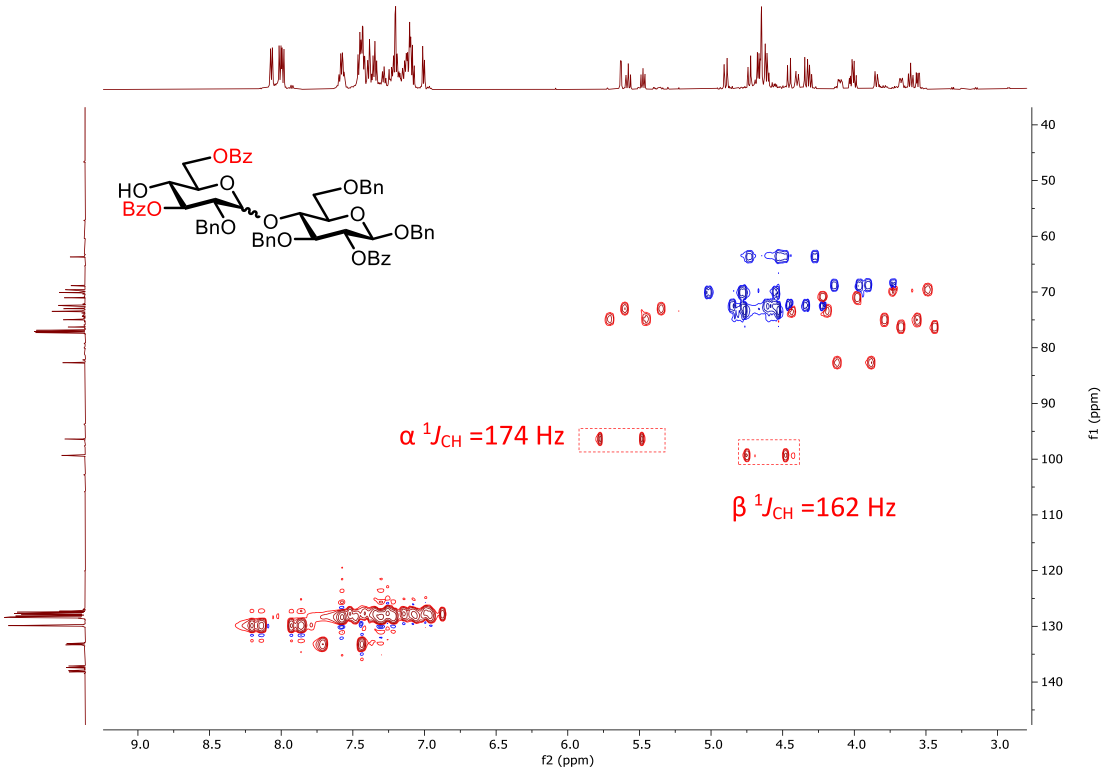

^{1}H NMR of 104a (400 MHz, $CDCl_3$)

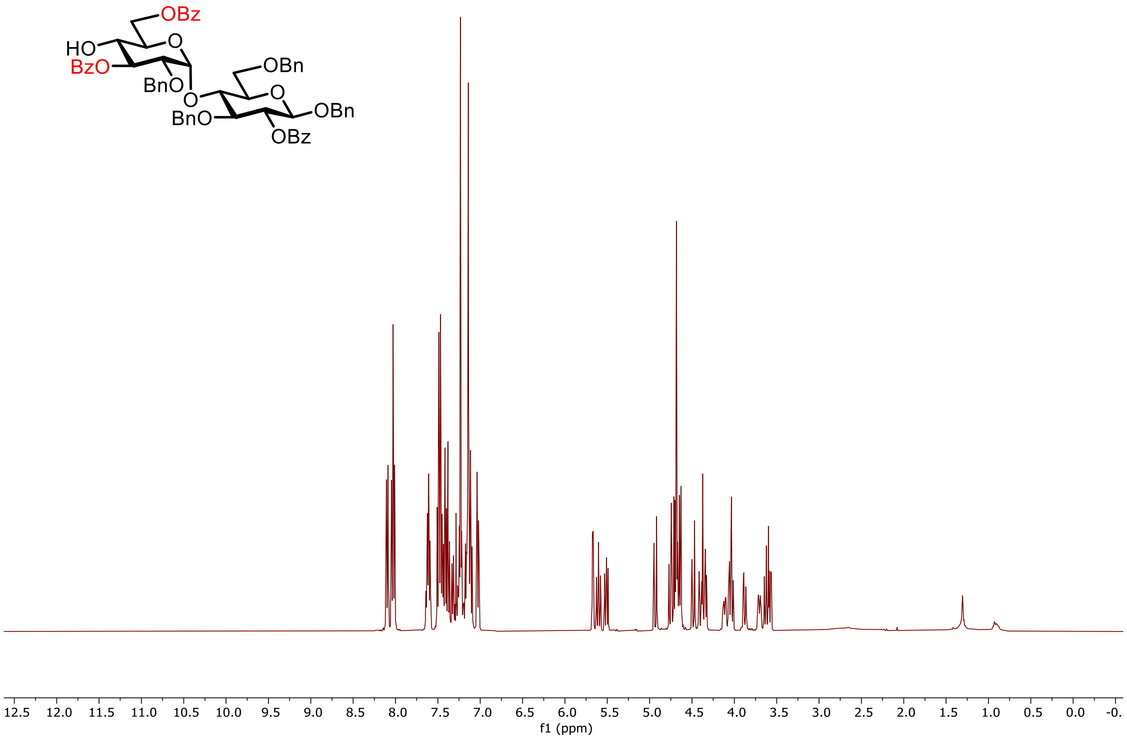

^{13}C NMR of 104a (101 MHz, $CDCl_3$)

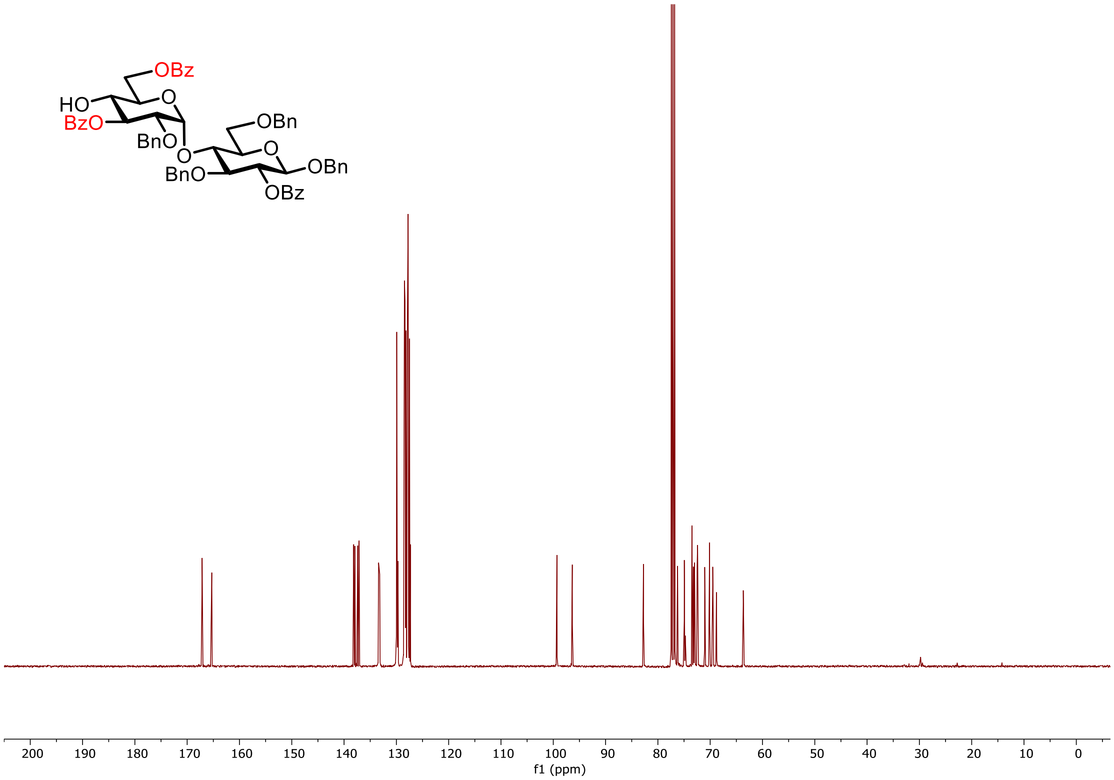

COSY NMR of 104a ($CDCl_3$)

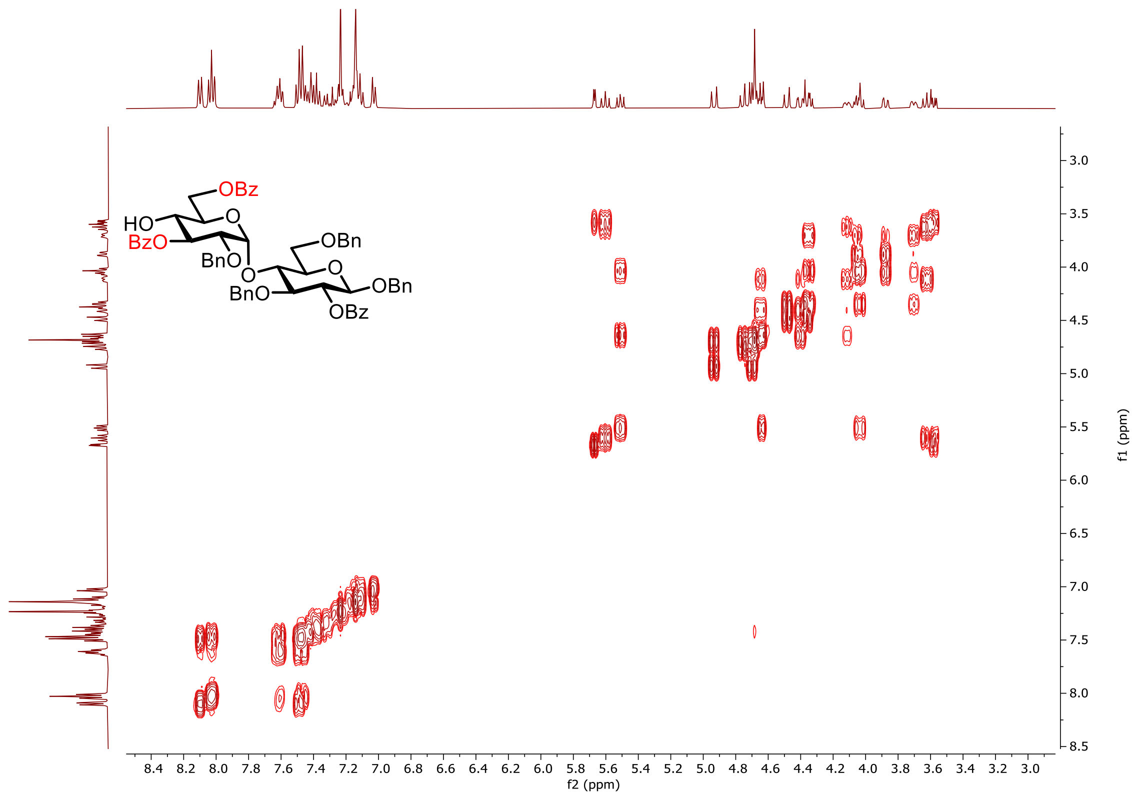

HSQC NMR of 104a ($CDCl_3$)

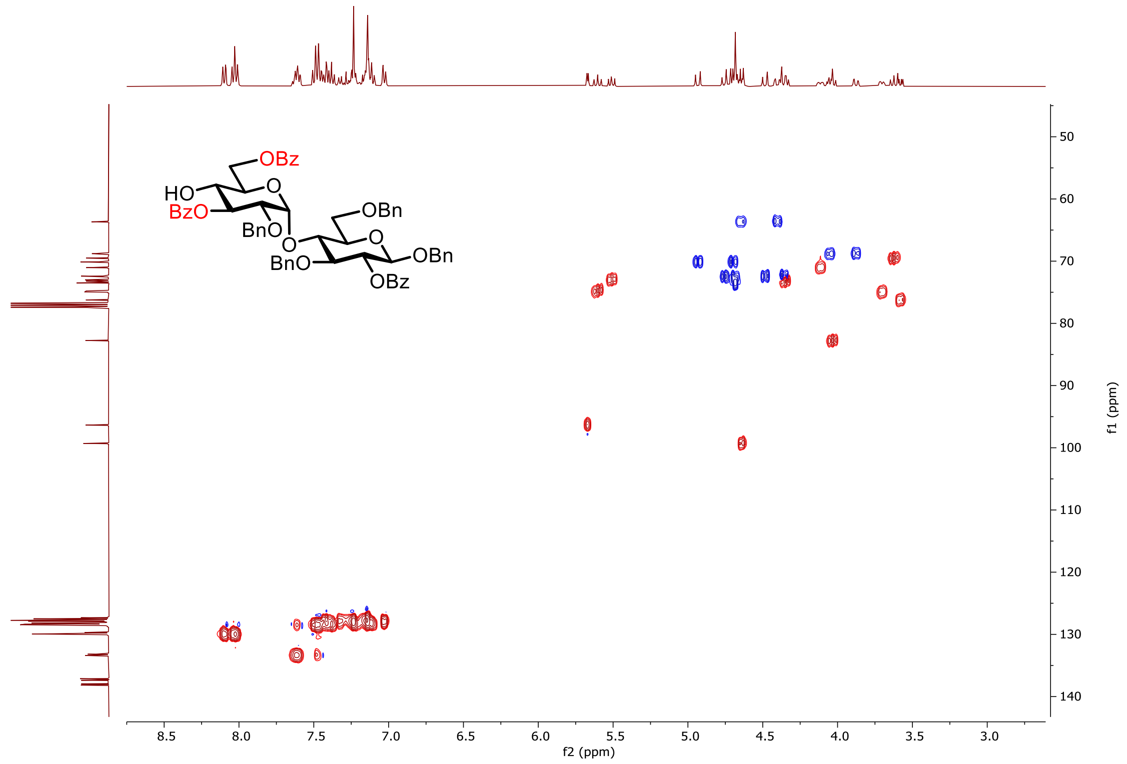

4.32 Benzyl 2-*O*-benzyl-3,6-di-*O*-(4-nitrobenzoyl)-D-glucopyranosyl-(1→4)-2-*O*-benzoyl-3,6-di-*O*-benzyl-β-D-glucopyranoside, 105

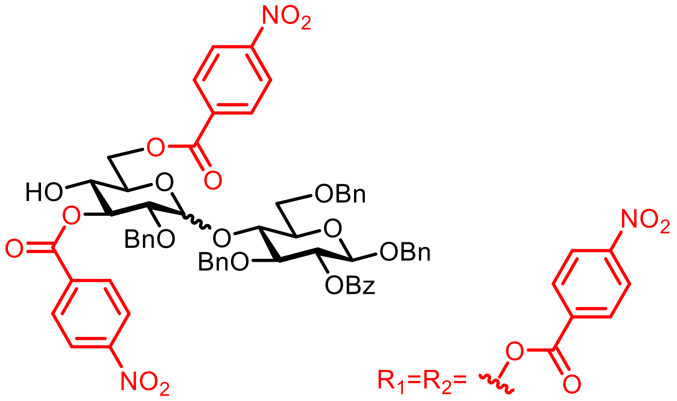

Total yield: 64% (70.7 mg). Ratio of anomer α : β > 10 : 1.

Spectrum data for benzyl 2-*O*-benzyl-3,6-di-*O*-(4-nitrobenzoyl)-α-D-glucopyranosyl-(1→4)-2-*O*-benzoyl-3,6-di-*O*-benzyl-β-D-glucopyranoside **105a**: ^{1}H NMR (400 MHz, $CDCl_3$) δ 8.32 – 8.24 (m, 4H), 8.24 – 8.18 (m, 2H), 8.14 – 8.07 (m, 2H), 8.05 – 7.98 (m, 2H), 7.64 – 7.58 (m, 1H), 7.47 (appt, *J* = 7.8 Hz, 2H), 7.43 – 7.34 (m, 4H), 7.33 – 7.14 (m, 12H), 7.13 – 7.07 (m, 2H), 7.00 – 6.94 (m, 2H), 5.74 (d, *J* = 3.6 Hz, 1H), 5.59 (appt, *J* = 9.6 Hz, 1H), 5.50 (dd, *J* = 8.9, 7.6 Hz, 1H), 4.93 (d, *J* = 12.6 Hz, 1H), 4.76 – 4.68 (m, 4H), 4.68 – 4.60 (m, 2H), 4.51 (d, *J* = 12.4 Hz, 1H), 4.44 – 4.33 (m, 2H), 4.26 (d, *J* = 12.4 Hz, 1H), 4.14 (ddd, *J* = 10.0, 4.5, 2.3 Hz, 1H), 4.10 – 4.01 (m, 2H), 3.89 (dd, *J* = 11.3, 2.0 Hz, 1H), 3.72 (ddd, *J* = 9.4, 3.7, 2.1 Hz, 1H), 3.64 – 3.53 (m, 2H), 2.97 (br. s, 1H); ^{13}C NMR (101 MHz, $CDCl_3$) δ 165.26, 165.24, 164.99, 150.72, 150.64, 138.04, 137.77, 137.15, 137.02, 135.00, 134.87, 133.29, 131.02, 130.97, 129.89, 129.69, 128.49, 128.43, 128.37, 128.34, 128.25, 127.93, 127.82, 127.78, 127.73, 127.49, 127.37, 123.67, 123.54, 99.30, 95.81, 82.82, 76.12, 75.42, 74.81, 73.66, 73.02, 72.46, 72.37, 72.24, 70.64, 70.18, 69.32, 68.75, 64.39; $[\alpha]_D^{25}$ 66.36 (c = 1, $CHCl_3$); IR (neat) ν_{max} = 3505, 2873, 1728, 1526, 1267, 1060, 718 cm^{-1}; m/z (HRMS$^+$) $[M + Na]^+$ 1127.362 ($C_{61}H_{56}N_2O_{18}Na^+$ requires 1127.342).

^{1}H NMR of crude 105 (600 MHz, $CDCl_3$)

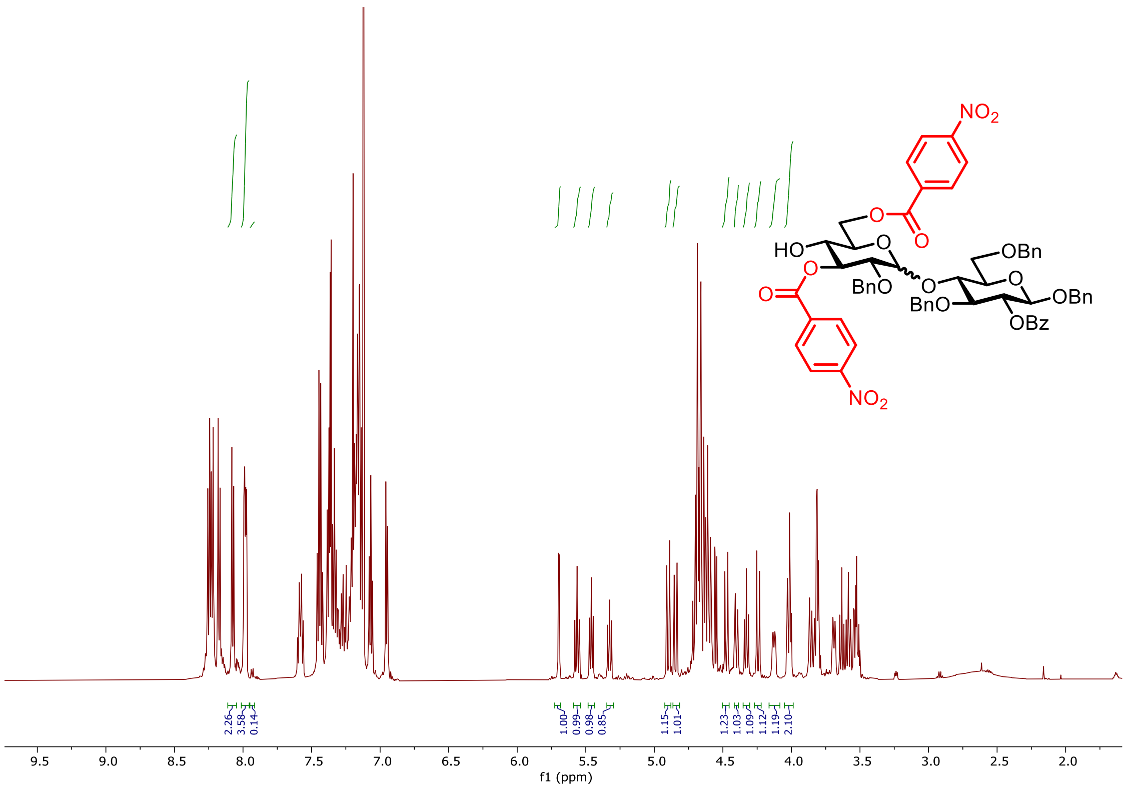

^{13}C NMR of crude 105 (151 MHz, $CDCl_3$)

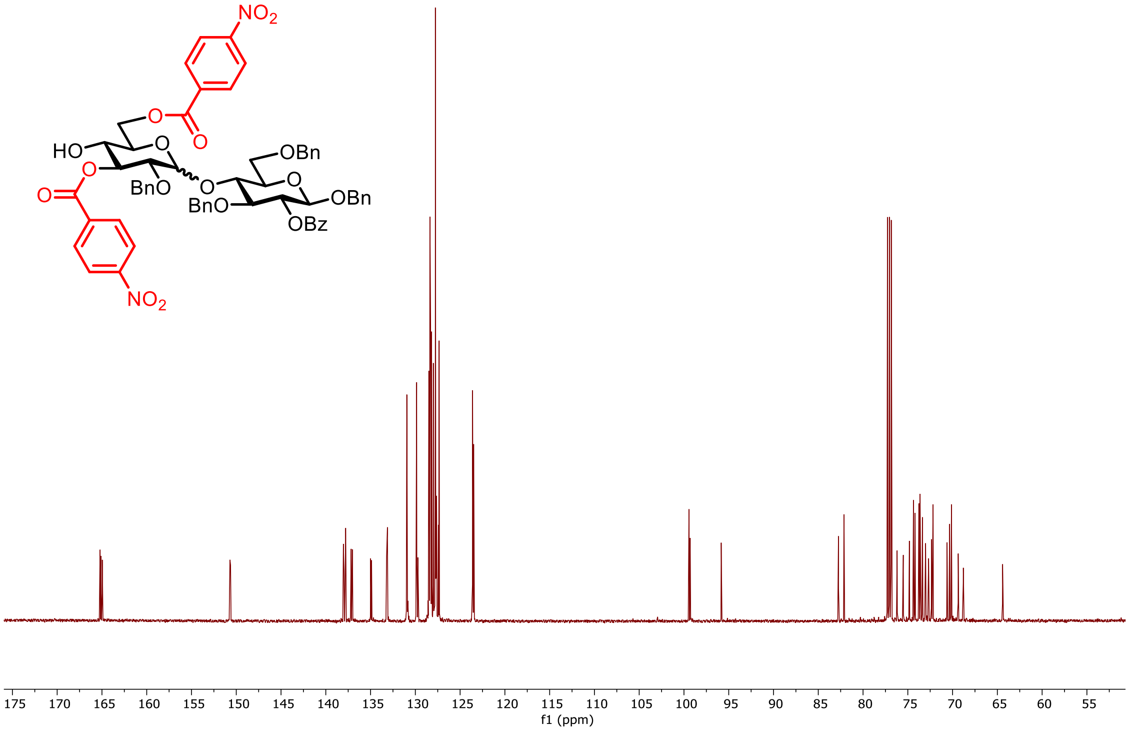

HSQC NMR of crude 105 ($CDCl_3$)

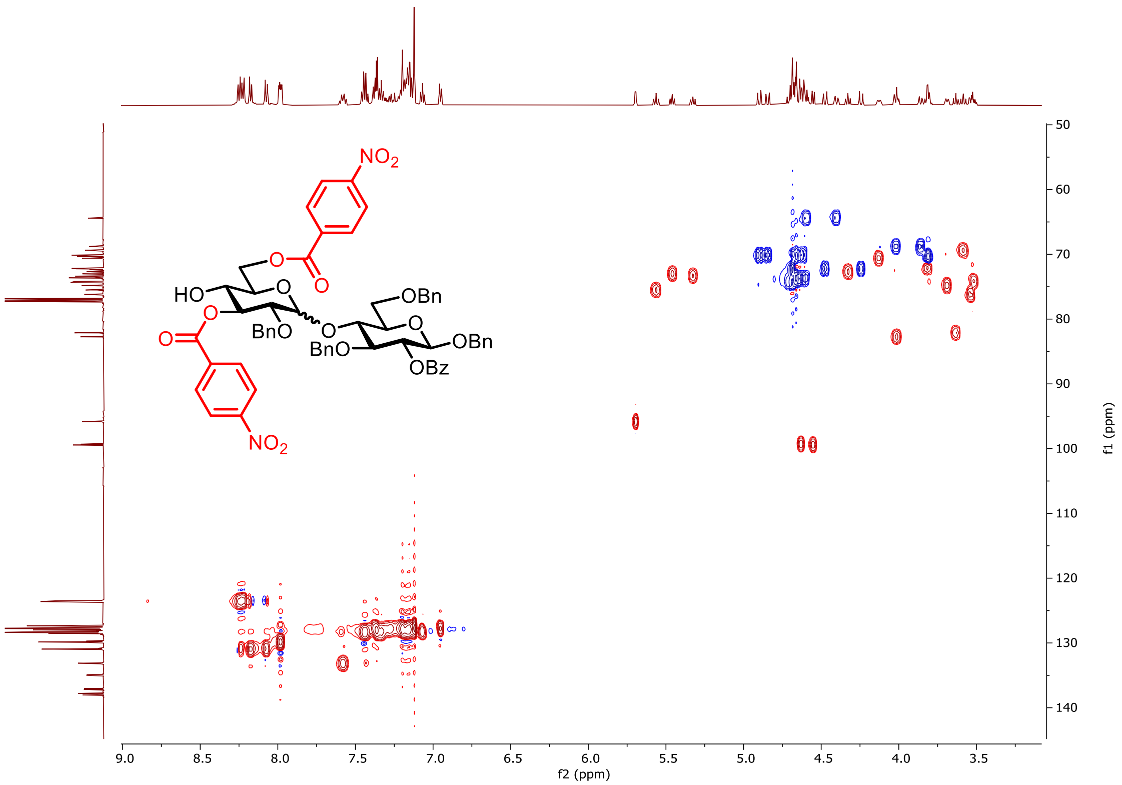

Coupled HSQC NMR of crude 105 ($CDCl_3$)

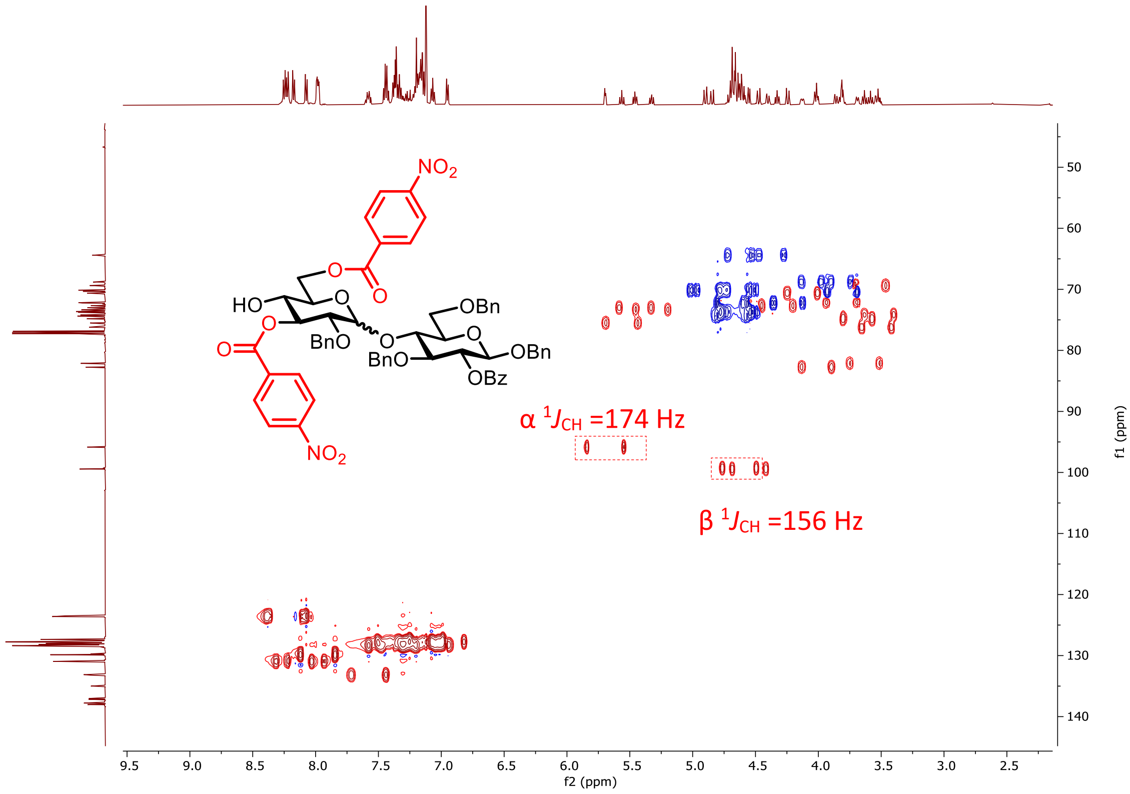

^1H NMR of 105a (400 MHz, $CDCl_3$)

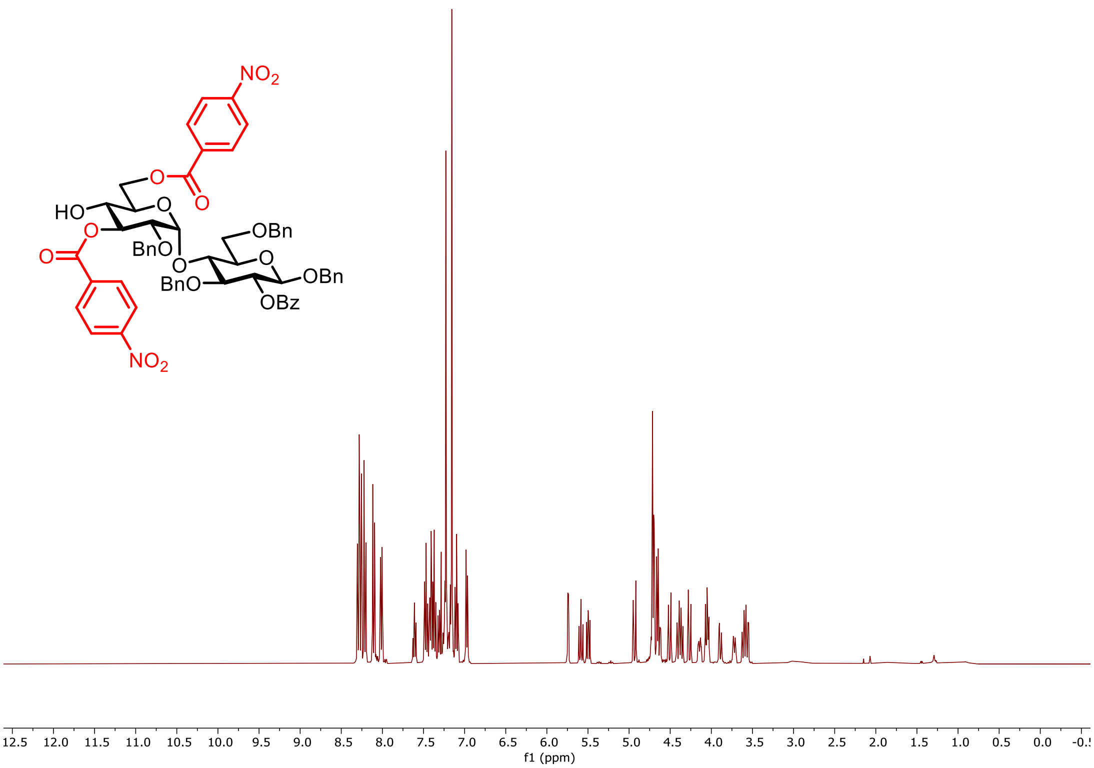

^{13}C NMR of 105a (101 MHz, $CDCl_3$)

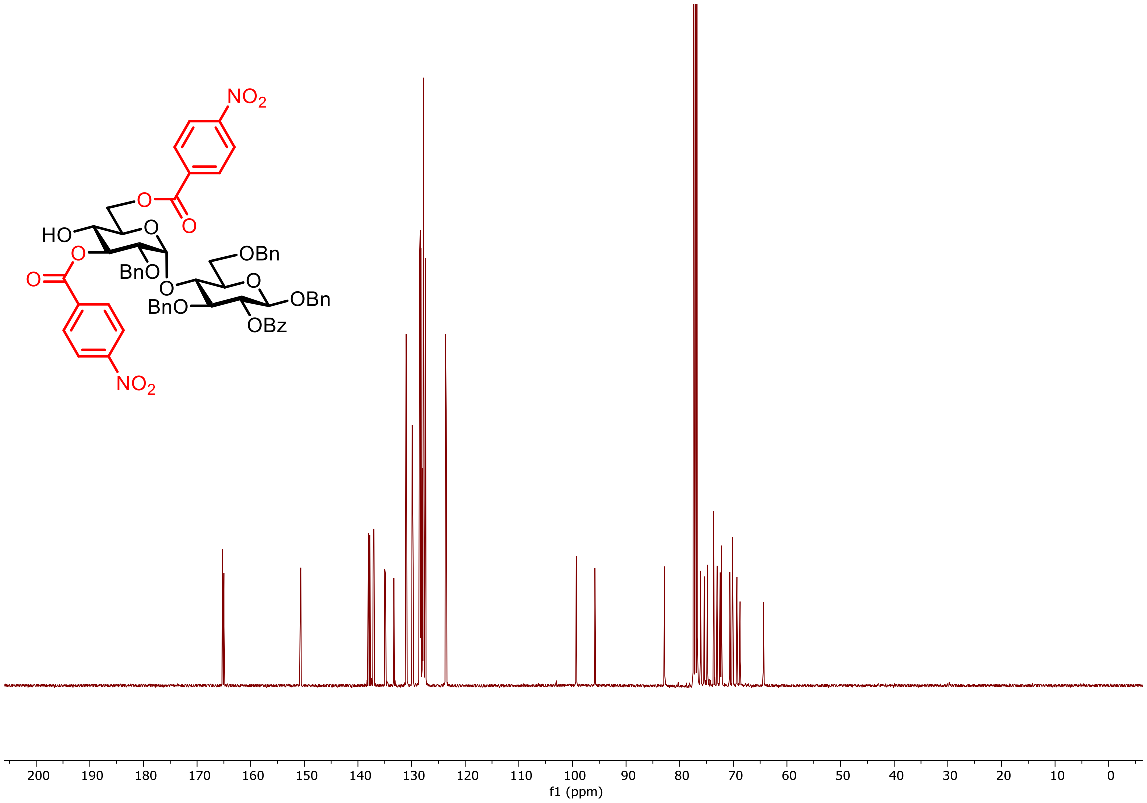

COSY NMR of 105a ($CDCl_3$)

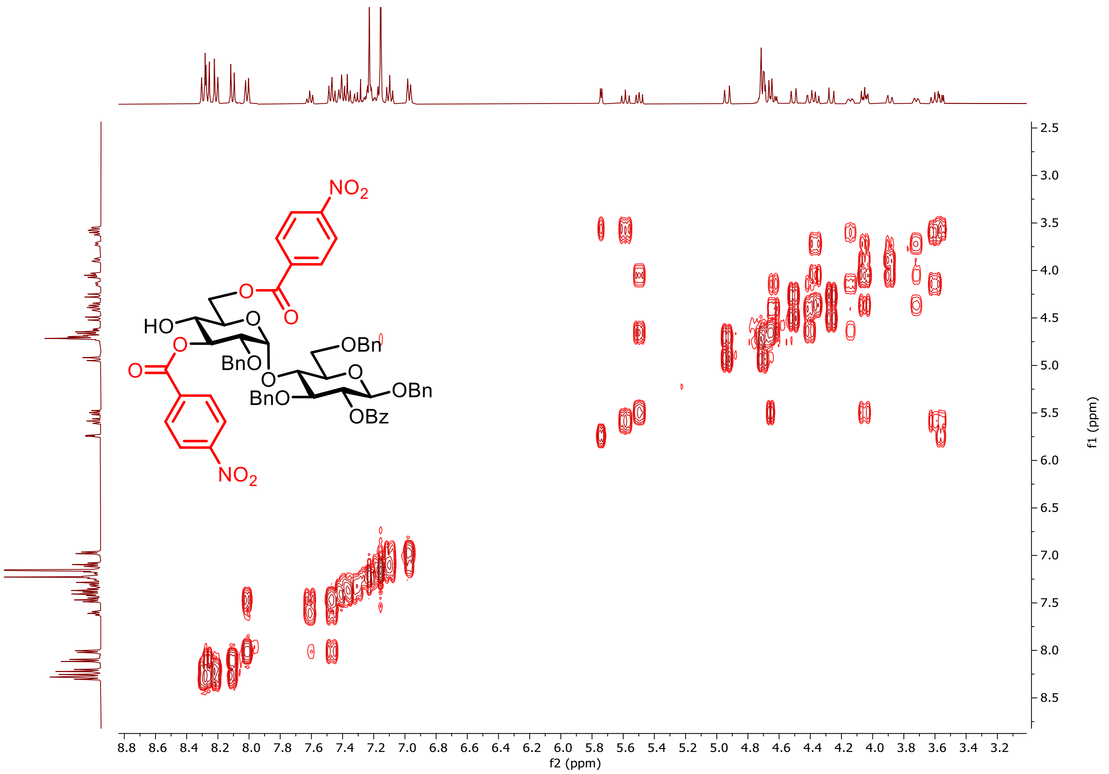

HSQC NMR of 105a ($CDCl_3$)

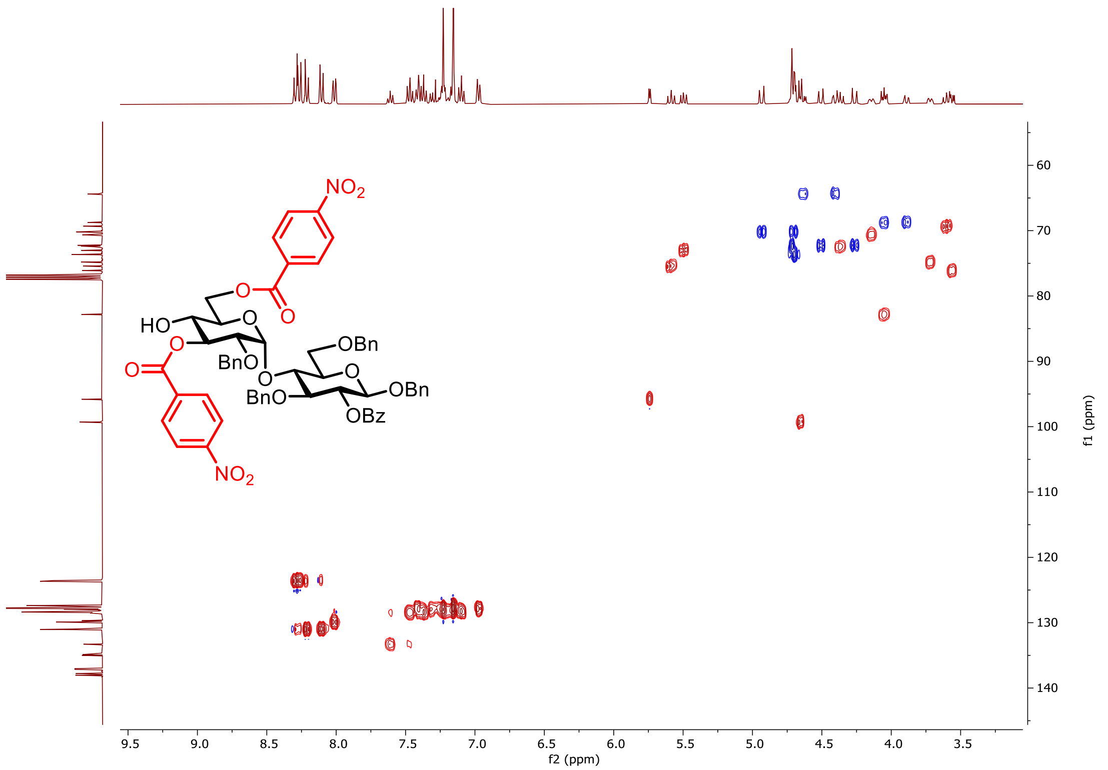

4.33 Benzyl 2-*O*-benzyl-3-*O*-benzoyl-6-*O*-levulinoyl-D-glucopyranosyl-(1→4)-2-*O*-benzoyl-3,6-di-*O*-benzyl-β-D-glucopyranoside, 106

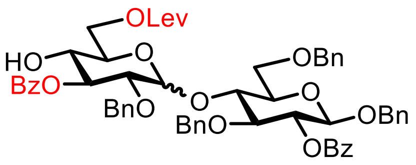

R_1=OLev; R_2=OBz

Total yield: 80% (81.0 mg). Ratio of anomer α : β > 10 : 1.

Spectrum data for benzyl 2-*O*-benzyl-3-*O*-benzoyl-6-*O*-levulinoyl-α-D-glucopyranosyl-(1→4)-2-*O*-benzoyl-3,6-di-*O*-benzyl-β-D-glucopyranoside **106a**: ^{1}H NMR (400 MHz, CDCl$_3$) δ 8.06 – 7.98 (m, 4H), 7.64 – 7.56 (m, 2H), 7.50 – 7.38 (m, 8H), 7.36 – 7.30 (m, 1H), 7.27 – 7.07 (m, 13H), 7.02 (dt, *J* = 6.9, 1.5 Hz, 2H), 5.64 (d, *J* = 3.6 Hz, 1H), 5.56 (appt, *J* = 9.6 Hz, 1H), 5.50 (dd, *J* = 9.0, 7.7 Hz, 1H), 4.92 (d, *J* = 12.6 Hz, 1H), 4.76 (d, *J* = 11.1 Hz, 1H), 4.72 – 4.66 (m, 4H), 4.63 (d, *J* = 7.7 Hz, 1H), 4.46 (td, *J* = 8.5, 4.3 Hz, 2H), 4.35 (d, *J* = 12.3 Hz, 1H), 4.29 (dd, *J* = 9.4, 8.5 Hz, 1H), 4.09 (dd, *J* = 12.2, 2.2 Hz, 1H), 4.03 – 3.93 (m, 3H), 3.88 (dd, *J* = 11.2, 2.0 Hz, 1H), 3.70 (ddd, *J* = 9.5, 4.1, 2.1 Hz, 1H), 3.63 – 3.56 (m, 2H), 2.99 (br. s, 1H), 2.76 (dd, *J* = 7.2, 5.7 Hz, 2H), 2.60 (dd, *J* = 8.0, 5.7 Hz, 2H), 2.17 (s, 3H); ^{13}C NMR (101 MHz, CDCl$_3$) δ 206.76, 173.35, 167.00, 165.27, 138.22, 137.95, 137.40, 137.12, 133.28, 133.17, 129.95, 129.89, 129.81, 129.78, 128.46, 128.40, 128.37, 128.32, 128.25, 128.17, 127.91, 127.74, 127.71, 127.68, 127.41, 127.32, 99.29, 96.55, 82.84, 76.22, 74.92, 74.68, 73.47, 73.44, 73.21, 72.83, 72.48, 70.81, 70.11, 69.06, 68.77, 63.20, 37.94, 29.88, 27.86; $[\alpha]_D^{25}$ 29.33 (c = 1, CHCl$_3$); IR (neat) ν_{max} = 3491, 2924, 1725, 1453, 1270, 1064, 712 cm^{-1}; m/z (HRMS$^+$) [M + Na]$^+$ 1031.384 ($C_{59}H_{60}O_{15}Na^+$ requires 1031.382).

^{1}H NMR of crude 106 (400 MHz, $CDCl_3$)

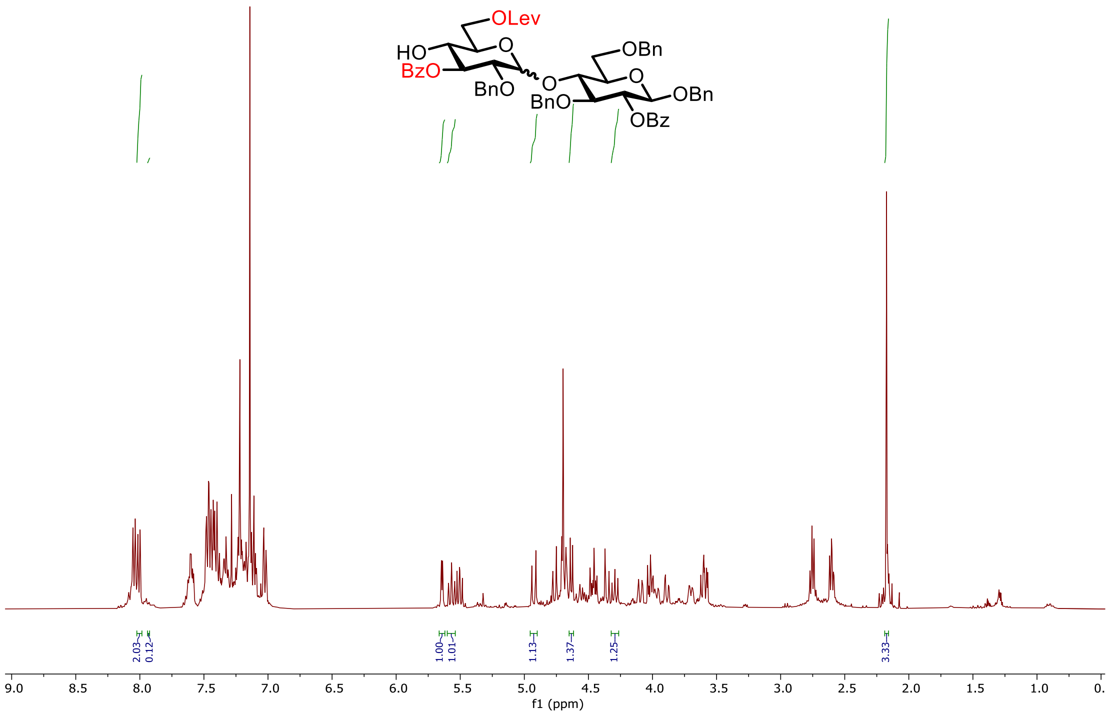

^{13}C NMR of crude 106 (101 MHz, $CDCl_3$)

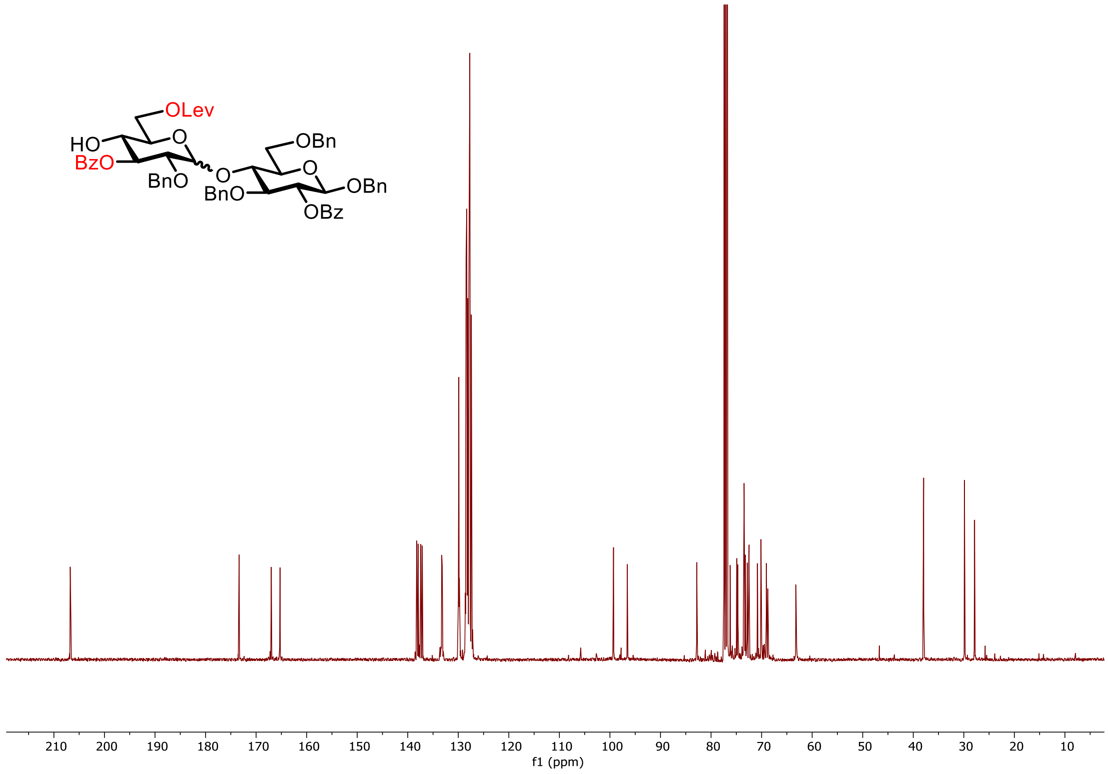

HSQC NMR of crude 106 ($CDCl_3$)

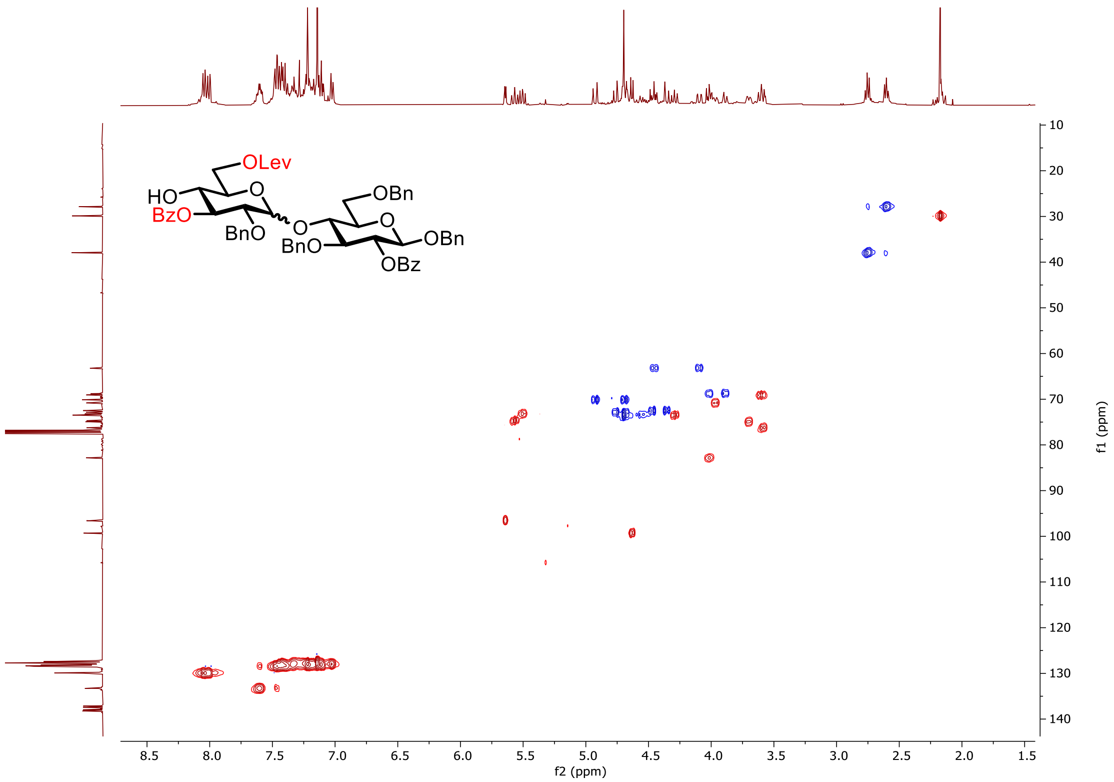

Coupled HSQC NMR of crude 106 ($CDCl_3$)

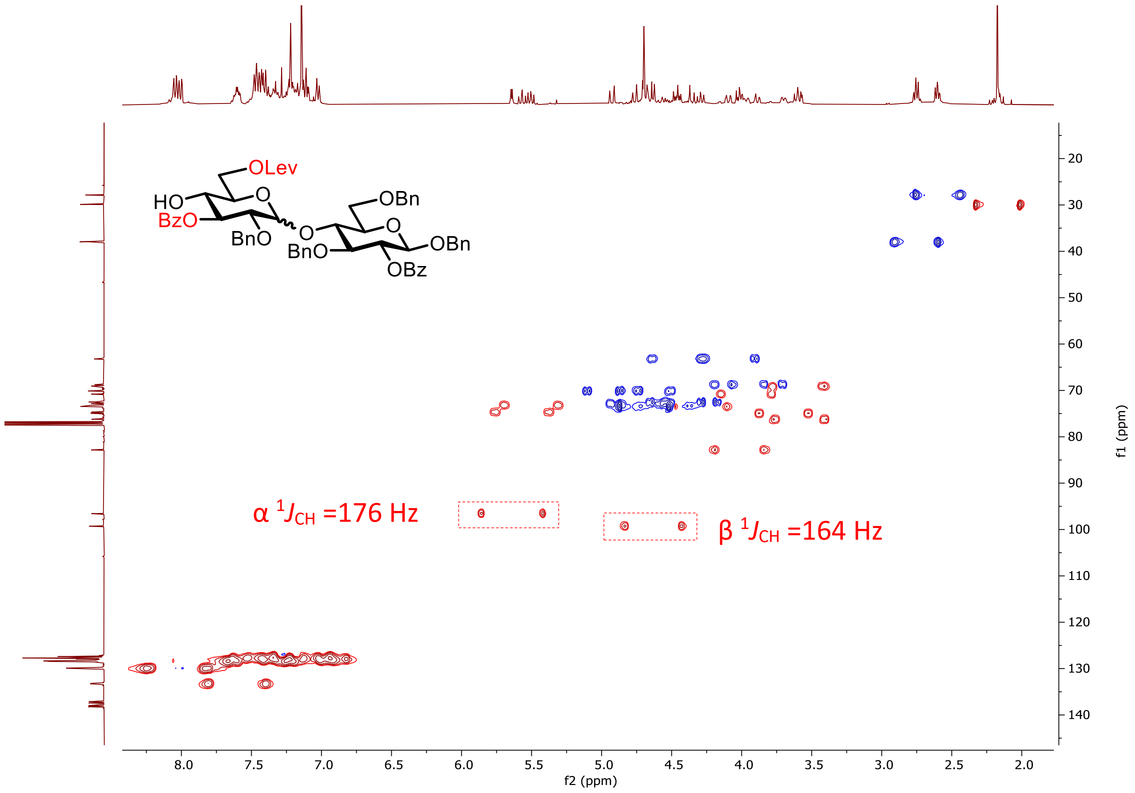

^{1}H NMR of 106a (400 MHz, $CDCl_3$)

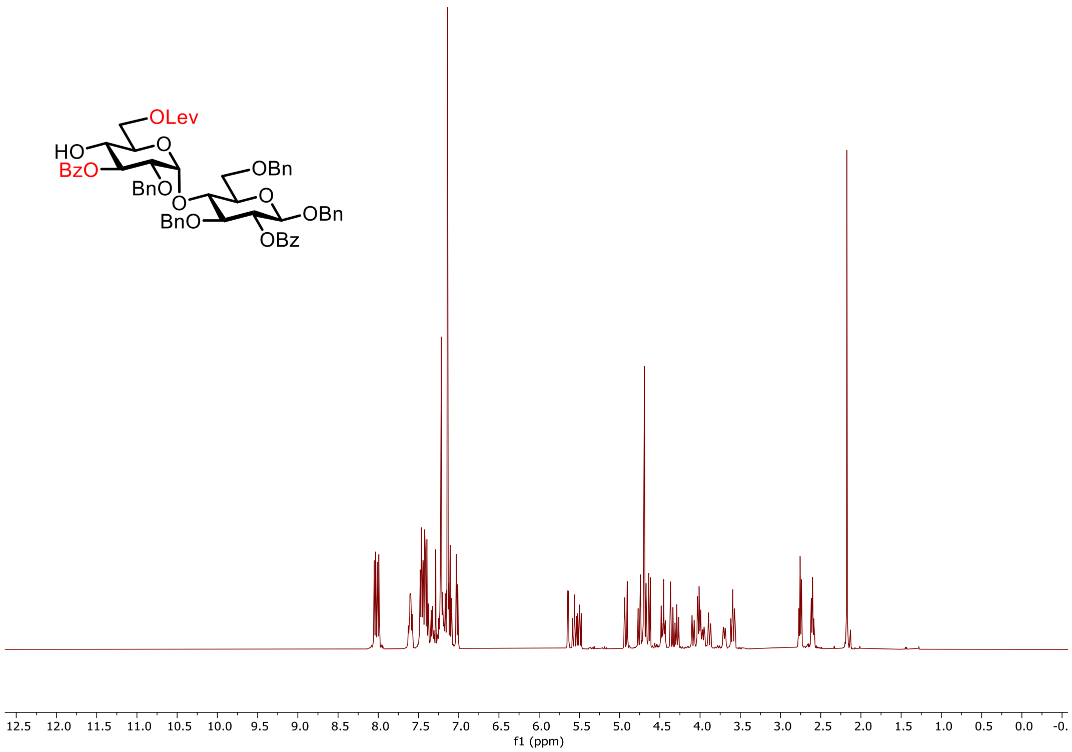

^{13}C NMR of 106a (101 MHz, $CDCl_3$)

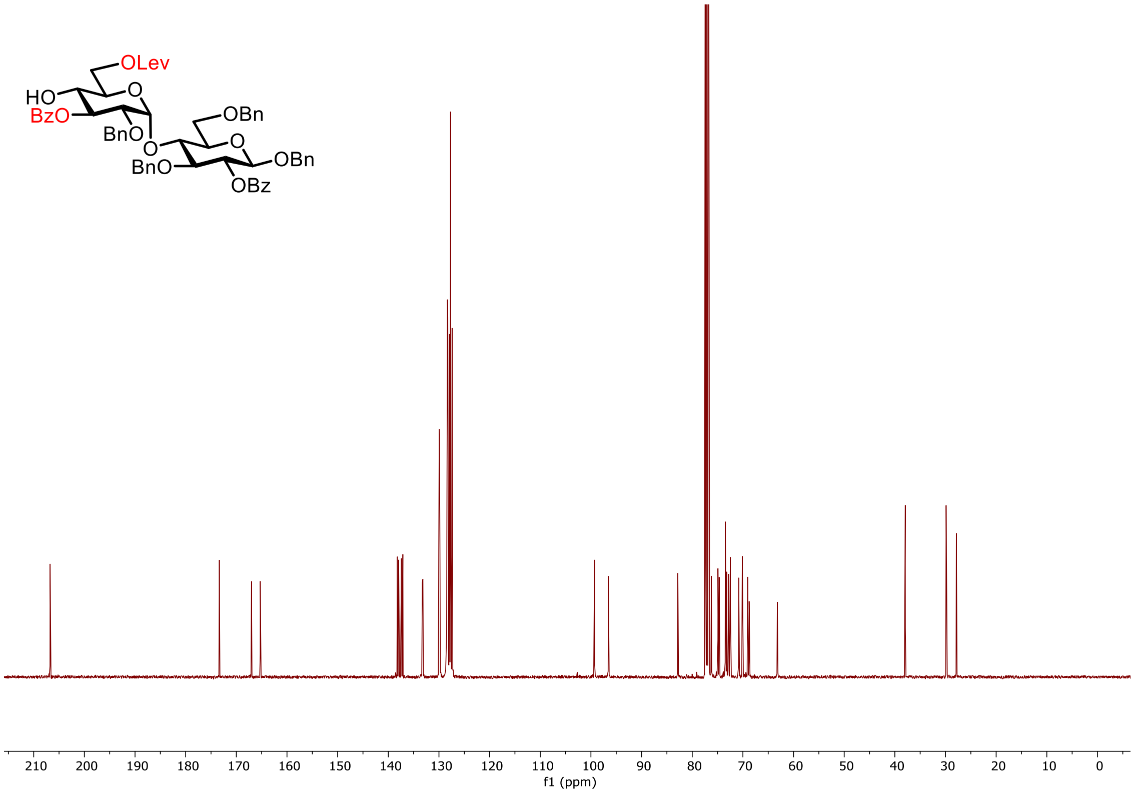

COSY NMR of 106a ($CDCl_3$)

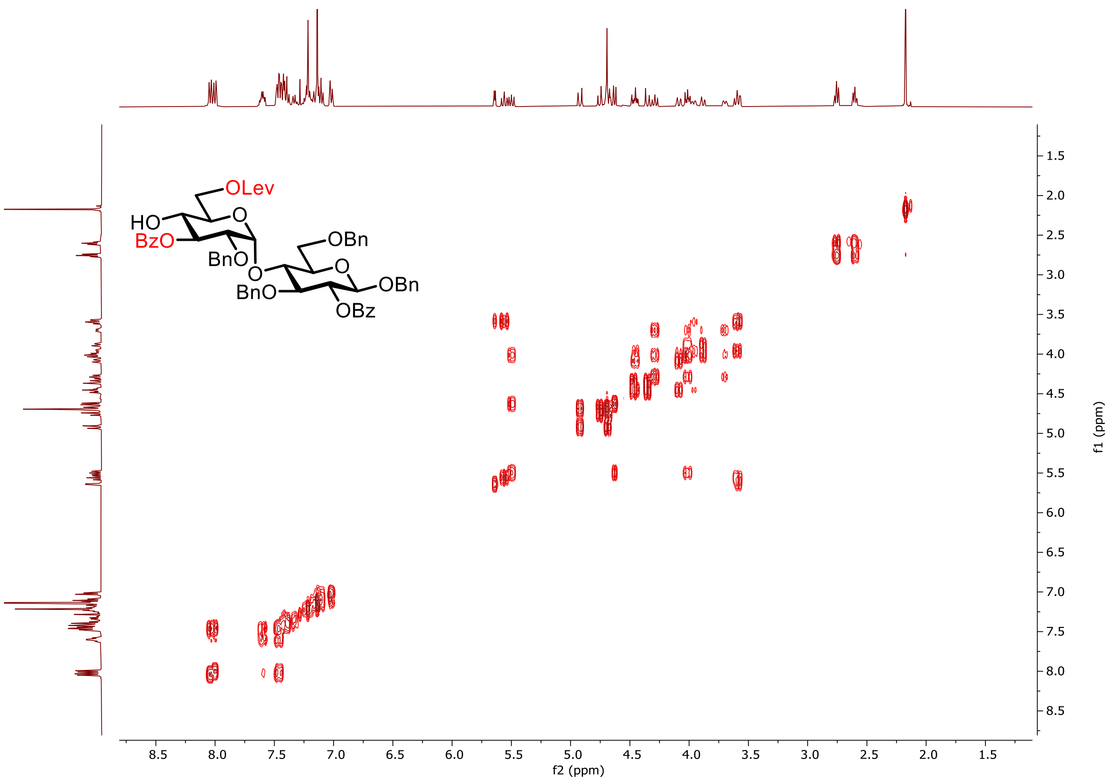

HSQC NMR of 106a ($CDCl_3$)

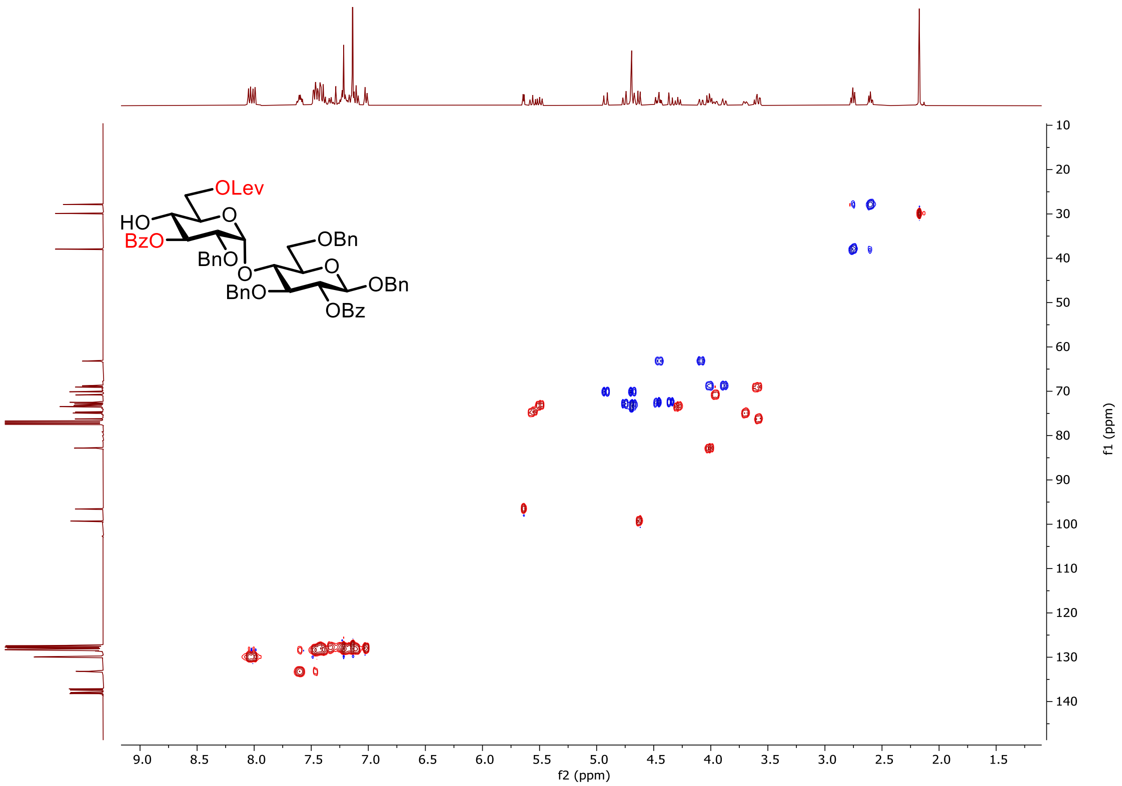

4.34 Benzyl 2,3-di-*O*-benzyl-6-deoxy-6-fluoro-D-glucopyranosyl-(1→4)-2-*O*-benzoyl-3,6-di-*O*-benzyl-β-D-glucopyranoside, 107

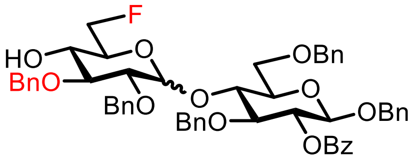

R_1=F; R_2=OBn

Total yield: 75% (67.4 mg). Ratio of anomer α : β = 3.9 : 1.

Spectrum data for benzyl 2,3-di-*O*-benzyl-6-deoxy-6-fluoro-α-D-glucopyranosyl-(1→4)-2-*O*-benzoyl-3,6-di-*O*-benzyl-β-D-glucopyranoside **107a**: ^{1}H NMR (400 MHz, $CDCl_3$) δ 8.03 – 7.97 (m, 2H), 7.64 – 7.58 (m, 1H), 7.47 (appt, *J* = 7.8 Hz, 2H), 7.42 – 7.29 (m, 10H), 7.27 – 7.18 (m, 10H), 7.17 – 7.10 (m, 5H), 5.58 (d, *J* = 3.5 Hz, 1H), 5.50 (dd, *J* = 8.8, 7.6 Hz, 1H), 4.98 (d, *J* = 11.4 Hz, 1H), 4.92 (d, *J* = 12.6 Hz, 1H), 4.77 (d, *J* = 11.1 Hz, 1H), 4.70 (d, *J* = 3.9 Hz, 1H), 4.68 – 4.40 (m, 8.5H), 4.37 – 4.27 (m, 1.5H), 4.02 (appt, *J* = 8.6 Hz, 1H), 3.94 (dd, *J* = 11.2, 4.2 Hz, 1H), 3.84 (dt, *J* = 11.1, 2.6 Hz, 2H), 3.75 (appt, *J* = 9.3 Hz, 1H), 3.68 (dt, *J* = 7.3, 2.2 Hz, 1H), 3.56 (dd, *J* = 10.1, 8.8 Hz, 1H), 3.46 (dd, *J* = 9.7, 3.6 Hz, 1H), 2.21 (br. s, 1H); ^{13}C NMR (151 MHz, $CDCl_3$) δ 165.20, 138.52, 138.18, 137.78, 137.63, 137.13, 133.15, 129.88, 129.86, 128.66, 128.40, 128.35, 128.30, 128.24, 127.97, 127.95, 127.84, 127.82, 127.76, 127.69, 127.67, 127.43, 127.40, 99.19, 96.70, 82.83, 81.94 (d, *J* = 172.6 Hz), 80.99, 79.02, 75.24, 75.00, 73.51, 72.98, 72.95, 72.73, 72.48, 70.97 (d, *J* = 17.5 Hz), 70.00, 68.89, 68.66 (d, *J* = 7.0 Hz); ^{19}F NMR (564 MHz, $CDCl_3$) δ -235.61 (td, *J* = 47.8, 47.1, 27.5 Hz); $[\alpha]_D^{25}$ 15.03 (c = 1, $CHCl_3$); IR (neat) ν_{max} = 3477, 2875, 1729, 1454, 1268, 1061, 698 cm^{-1}; m/z ($HRMS^+$) $[M + K]^+$ 937.3502 ($C_{54}H_{55}FO_{11}K^+$ requires 937.3360).

^{1}H NMR of crude 107 (600 MHz, $CDCl_3$)

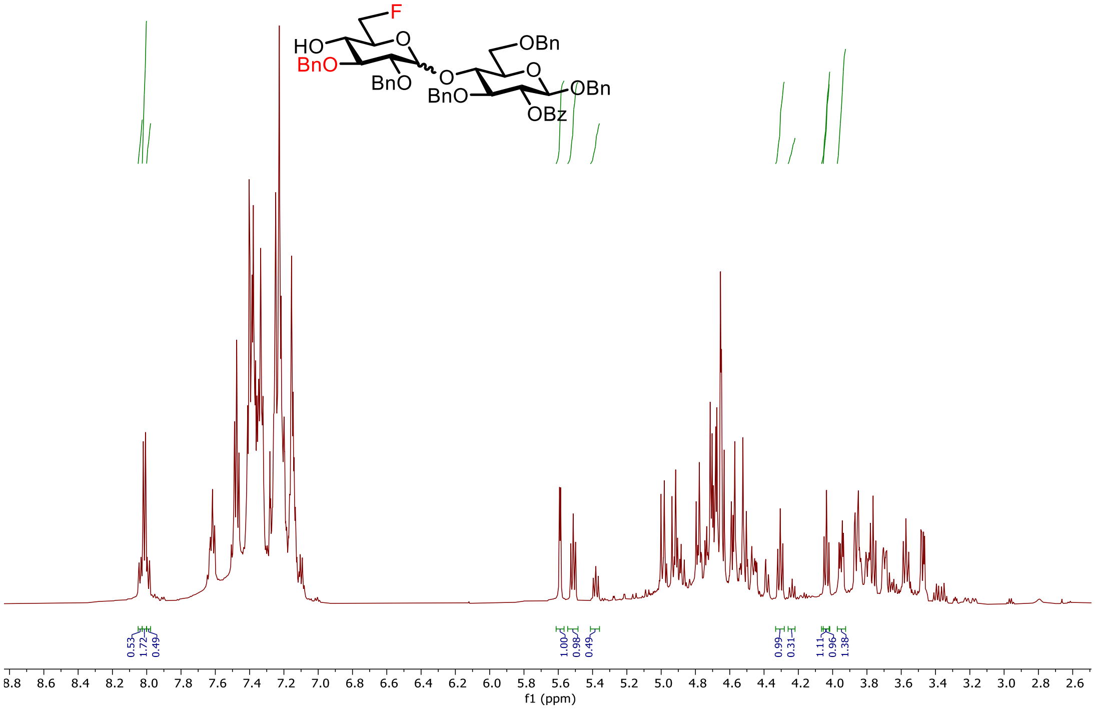

^{13}C NMR of crude 107 (151 MHz, $CDCl_3$)

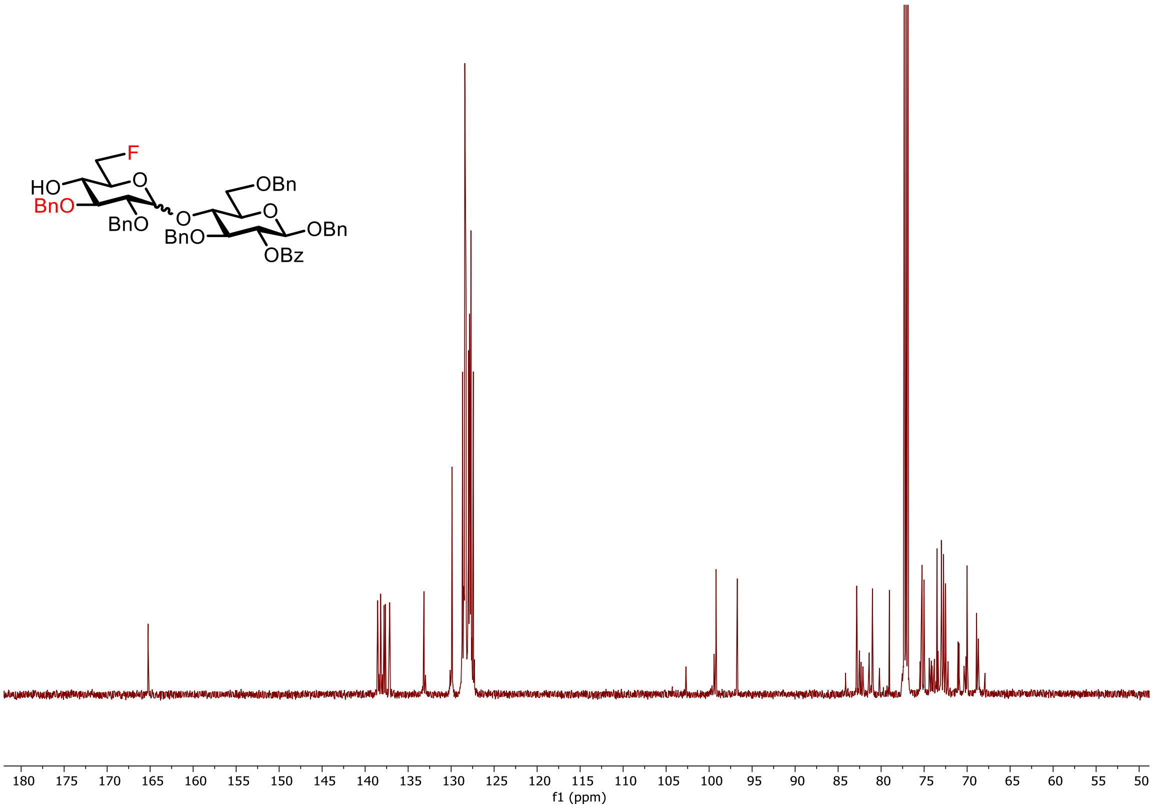

HSQC NMR of crude 107 ($CDCl_3$)

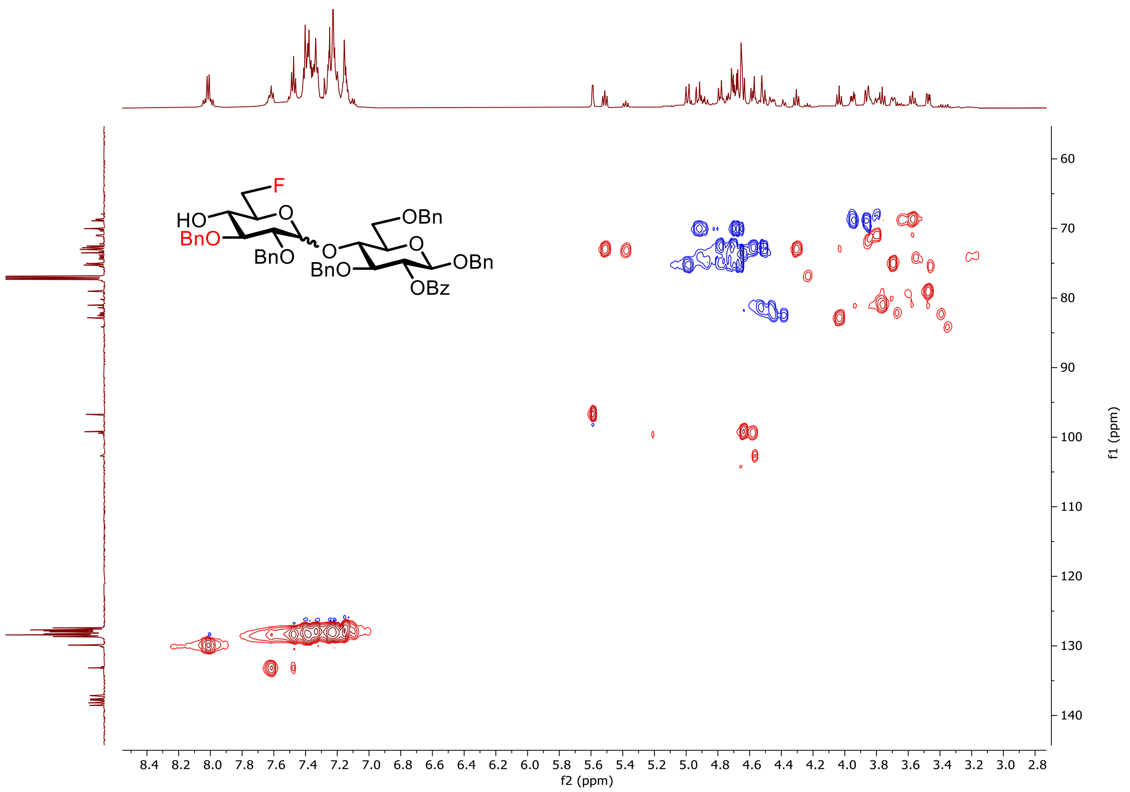

Coupled HSQC NMR of crude 107 ($CDCl_3$)

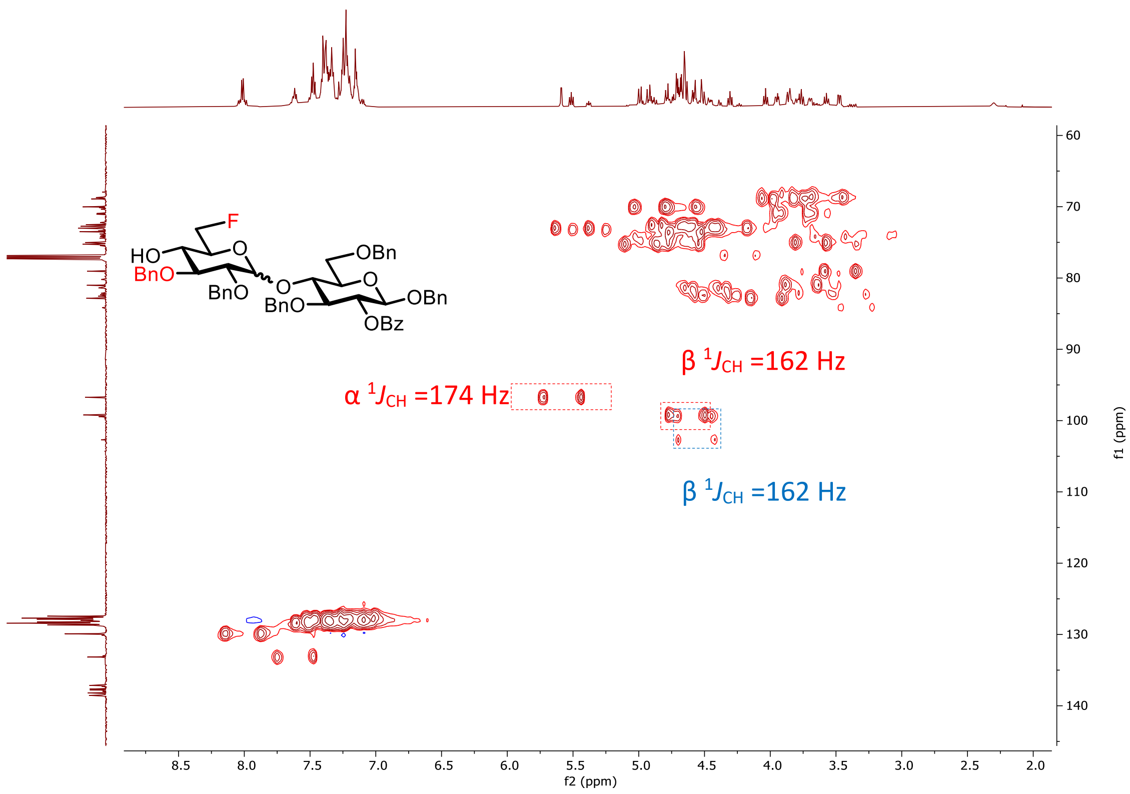

^{1}H NMR of 107a (600 MHz, $CDCl_3$)

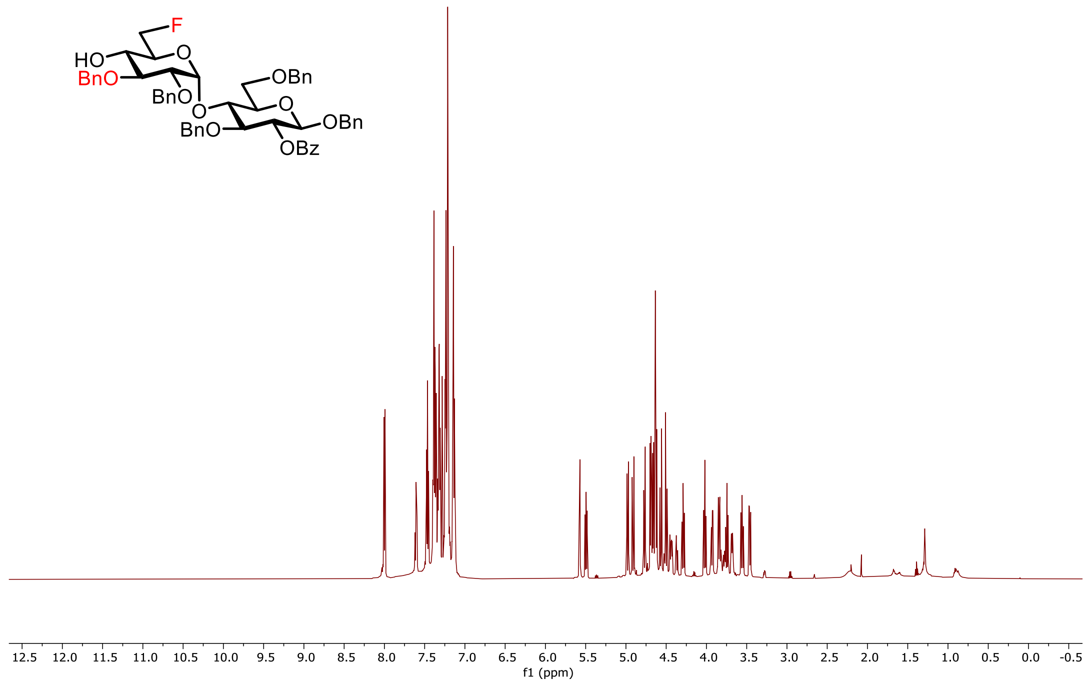

^{13}C NMR of 107a (151 MHz, $CDCl_3$)

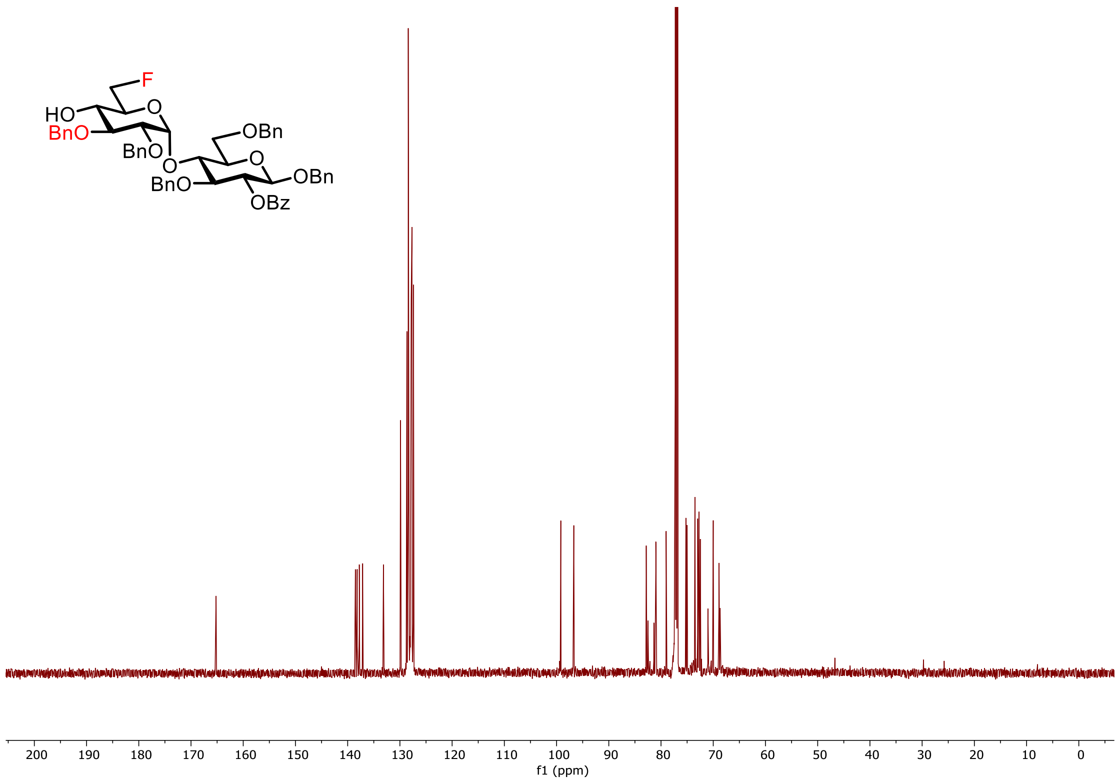

^{19}F NMR of 107a (564 MHz, $CDCl_3$)

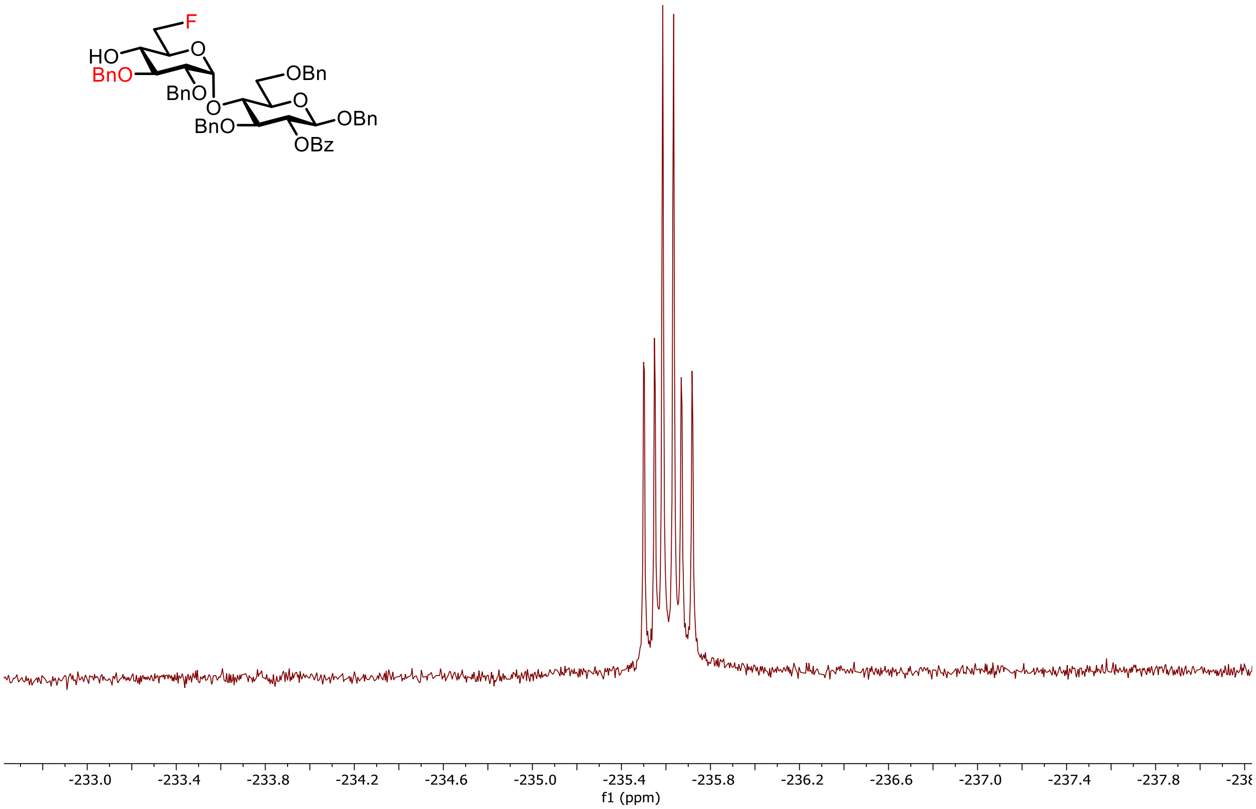

COSY NMR of 107a ($CDCl_3$)

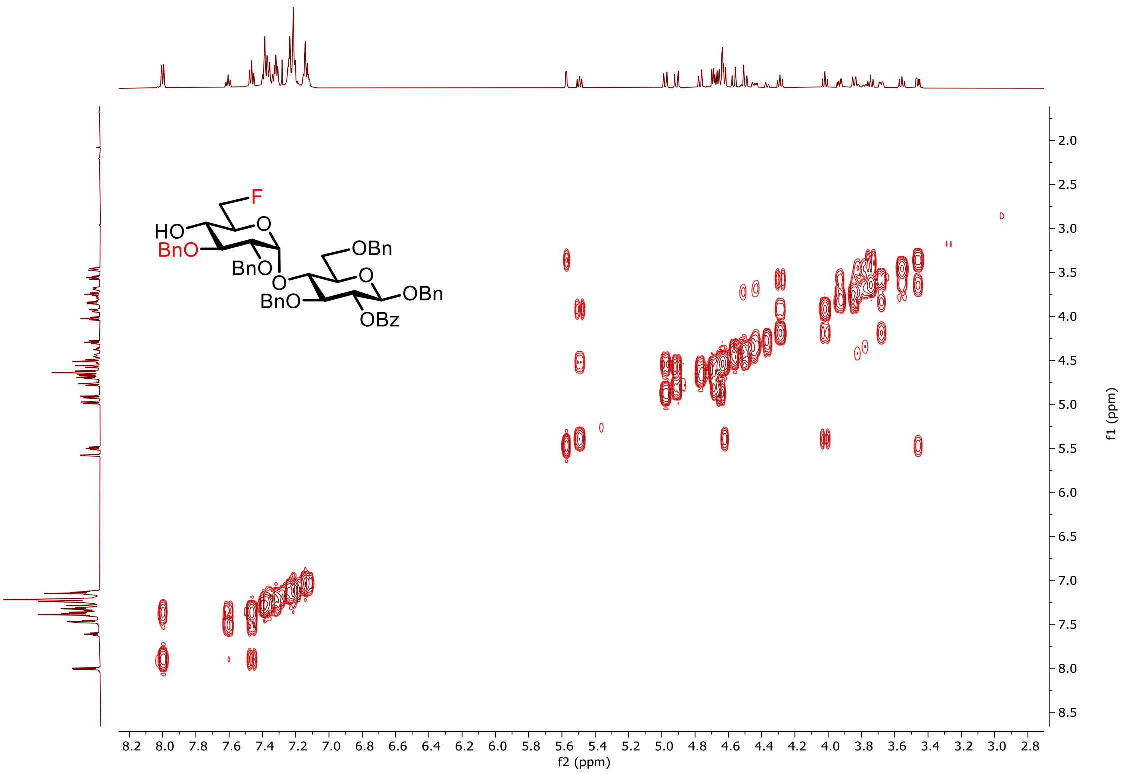

HSQC NMR of 107a ($CDCl_3$)

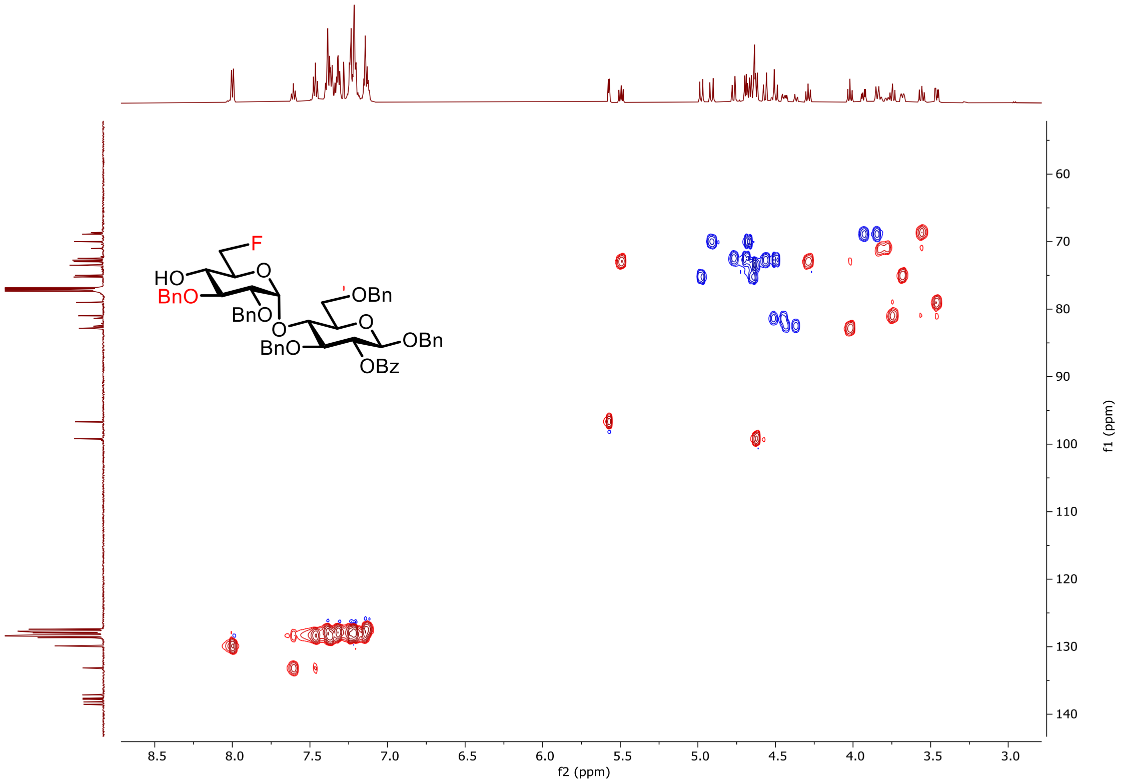

4.35 Benzyl 2,3-di-*O*-benzyl-6-*O*-methyl-D-glucopyranosyl-(1→4)-2-*O*-benzoyl-3,6-di-*O*-benzyl-β-D-glucopyranoside, 108

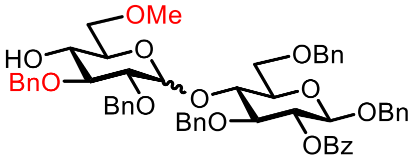

R_1=OMe; R_2=OBn

Total yield: 81% (73.8 mg). Ratio of anomer α : β = 2.7 : 1.

Spectrum data for benzyl 2,3-di-*O*-benzyl-6-*O*-methyl-α-D-glucopyranosyl-(1→4)-2-*O*-benzoyl-3,6-di-*O*-benzyl-β-D-glucopyranoside **108a**: ^{1}H NMR (600 MHz, $CDCl_3$) δ 8.02 – 7.98 (m, 2H), 7.60 (td, *J* = 7.3, 1.3 Hz, 1H), 7.46 (appt, *J* = 7.8 Hz, 2H), 7.43 – 7.29 (m, 10H), 7.27 – 7.18 (m, 10H), 7.16 – 7.09 (m, 5H), 5.56 (d, *J* = 3.6 Hz, 1H), 5.49 (dd, *J* = 8.8, 7.6 Hz, 1H), 4.94 (d, *J* = 11.4 Hz, 1H), 4.91 (d, *J* = 12.7 Hz, 1H), 4.77 – 4.61 (m, 7H), 4.59 – 4.52 (m, 2H), 4.29 – 4.24 (m, 1H), 4.02 (appt, *J* = 8.5 Hz, 1H), 3.94 (dd, *J* = 11.1, 4.5 Hz, 1H), 3.88 (dd, *J* = 11.1, 2.2 Hz, 1H), 3.79 – 3.73 (m, 2H), 3.72 – 3.68 (m, 1H), 3.62 (appt, *J* = 9.3 Hz, 1H), 3.53 (dd, *J* = 10.2, 3.8 Hz, 1H), 3.48 (dd, *J* = 9.7, 3.6 Hz, 1H), 3.43 (dd, *J* = 10.2, 3.6 Hz, 1H), 3.31 (s, 3H), 2.49 (br. s, 1H); ^{13}C NMR (151 MHz, $CDCl_3$) δ 165.21, 138.77, 138.38, 137.83, 137.82, 137.17, 133.13, 129.88, 128.55, 128.36, 128.34, 128.29, 128.20, 128.03, 127.92, 127.82, 127.79, 127.76, 127.71, 127.66, 127.63, 127.55, 127.37, 127.36, 99.18, 96.86, 83.00, 81.20, 78.92, 75.20, 75.03, 73.42, 73.15, 72.89, 72.63, 71.98, 70.91, 70.54, 69.99, 69.08, 59.45; $[\alpha]_D^{25}$ 15.59 (c = 1, $CHCl_3$); IR (neat) ν_{max} = 3478, 2923, 1730, 1454, 1268, 1059, 698 cm^{-1}; m/z (HRMS$^+$) [M + Na]$^+$ 933.3966 ($C_{55}H_{58}O_{12}Na^+$ requires 933.3820).

^{1}H NMR of crude 108 (600 MHz, $CDCl_3$)

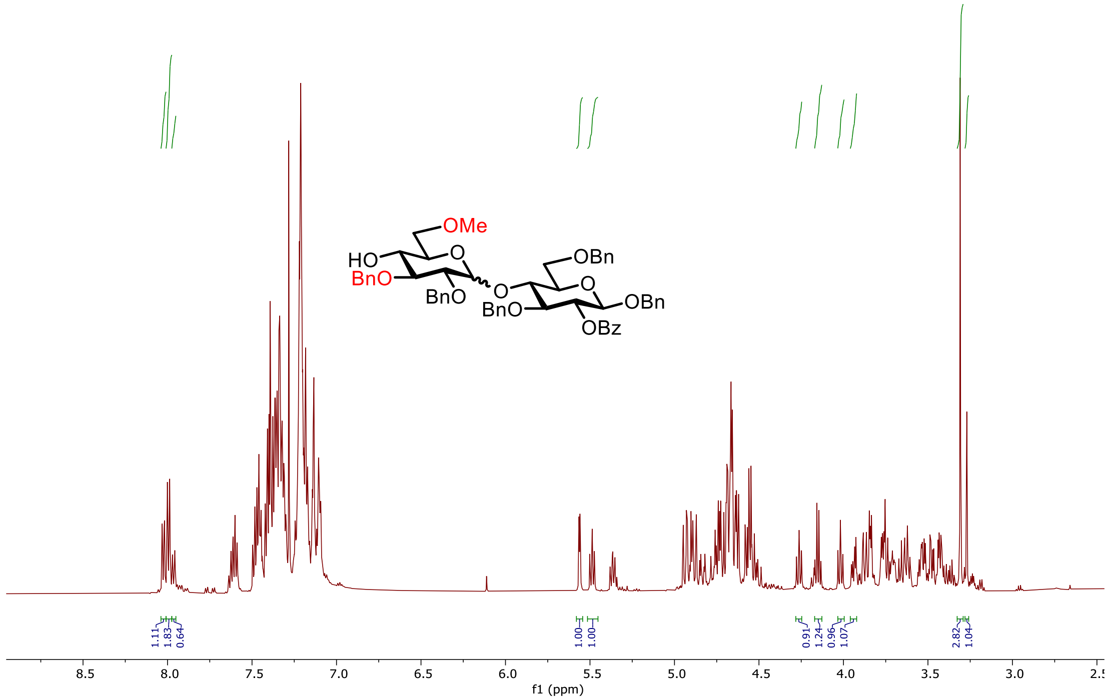

^{13}C NMR of crude 108 (151 MHz, $CDCl_3$)

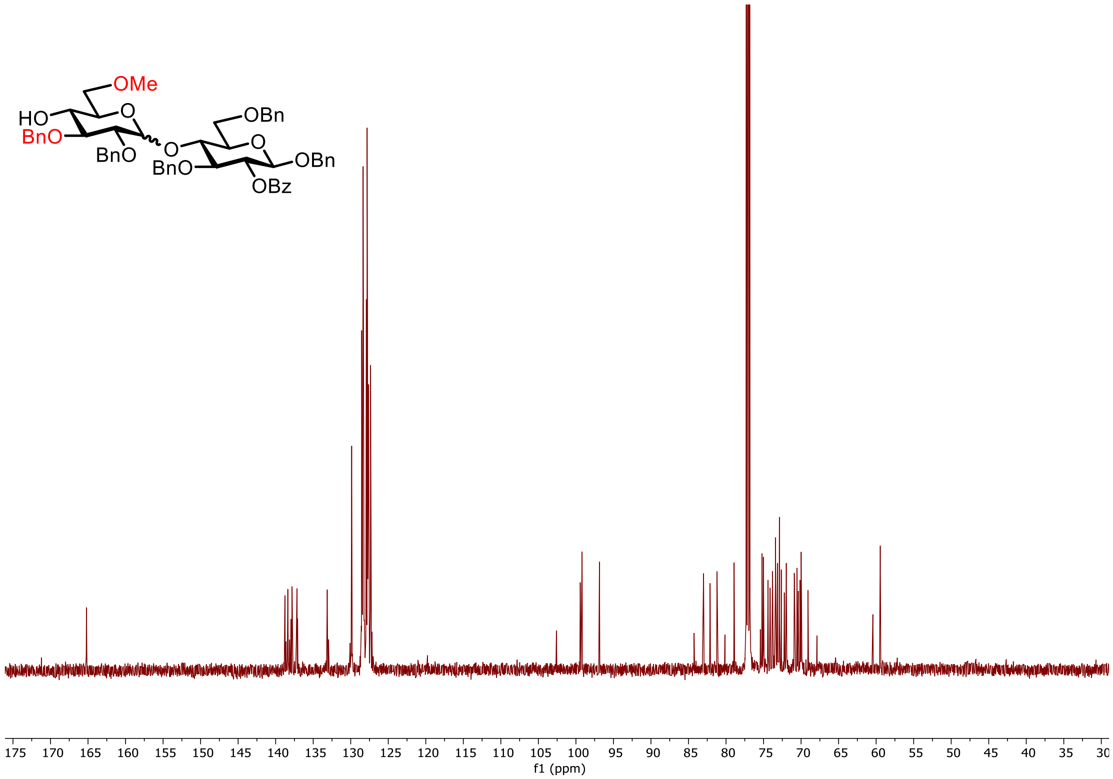

HSQC NMR of crude 108 ($CDCl_3$)

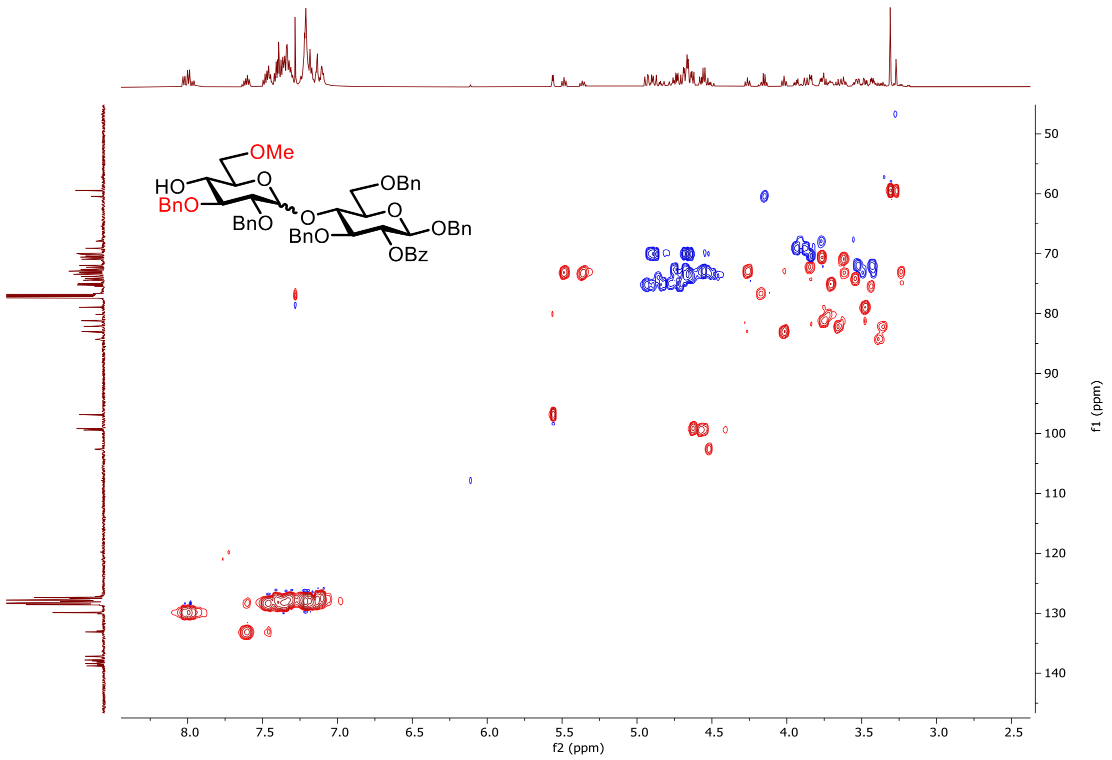

Coupled HSQC NMR of crude 108 ($CDCl_3$)

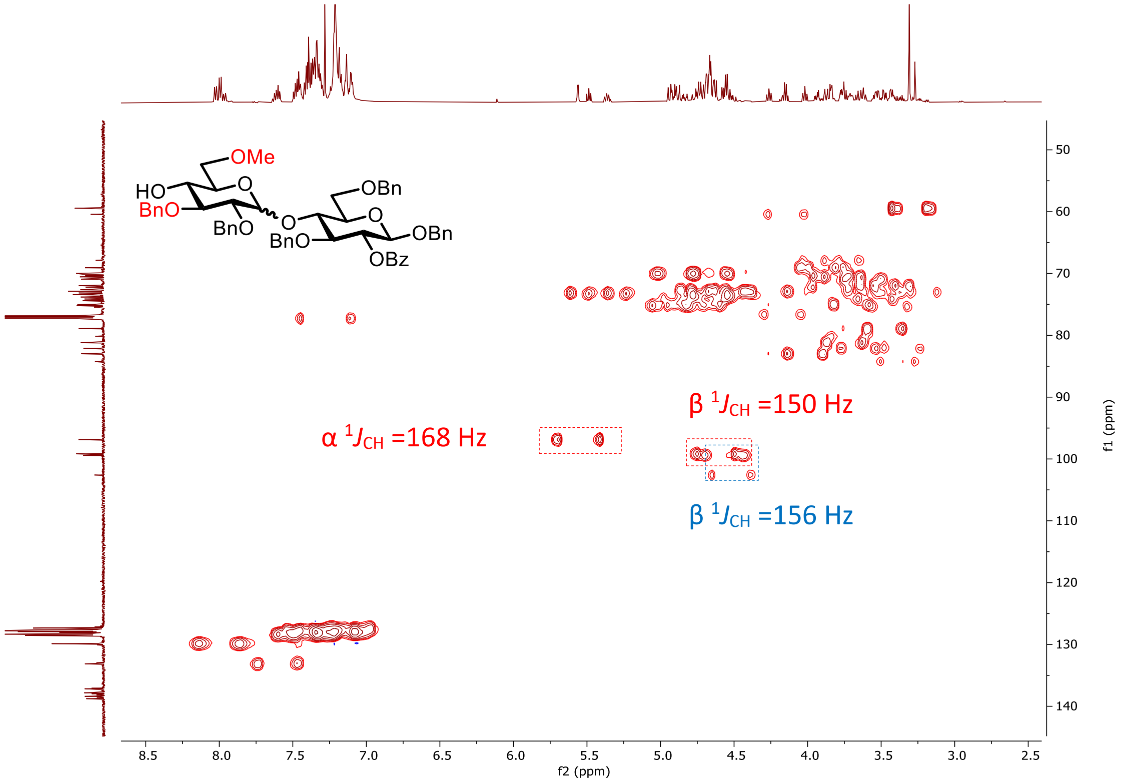

^{1}H NMR of 108a (600 MHz, CDCl$_3$)

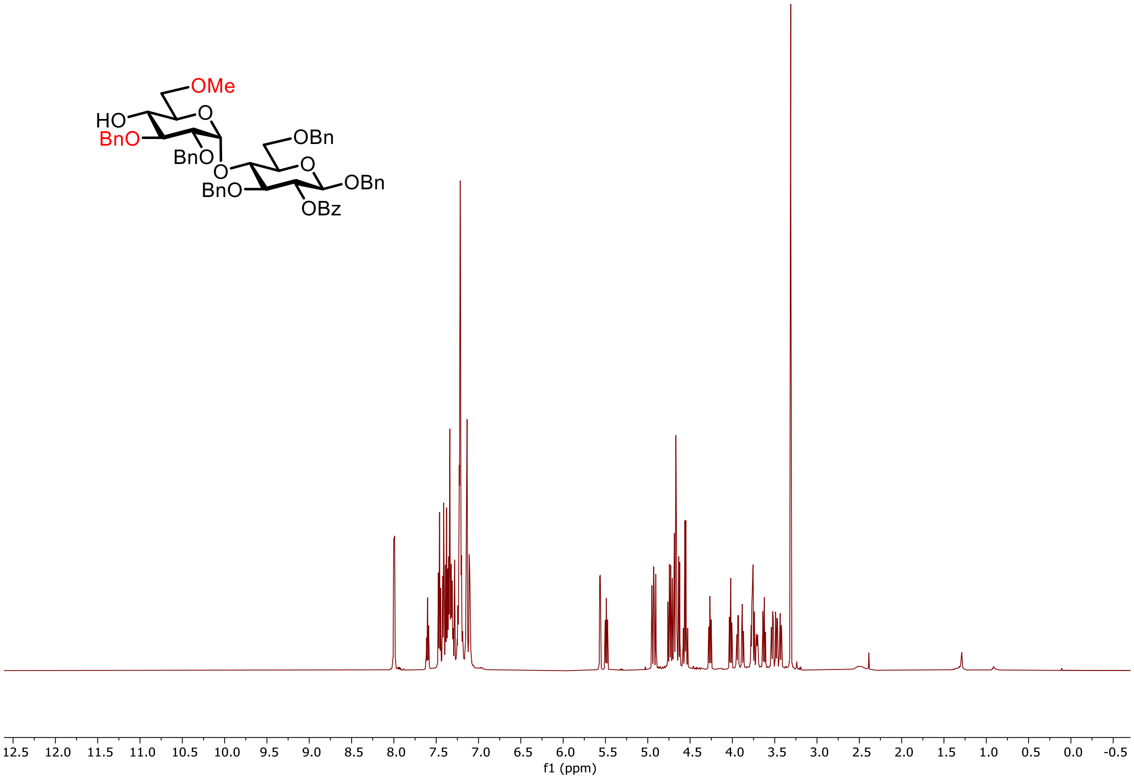

^{13}C NMR of 108a (151 MHz, CDCl$_3$)

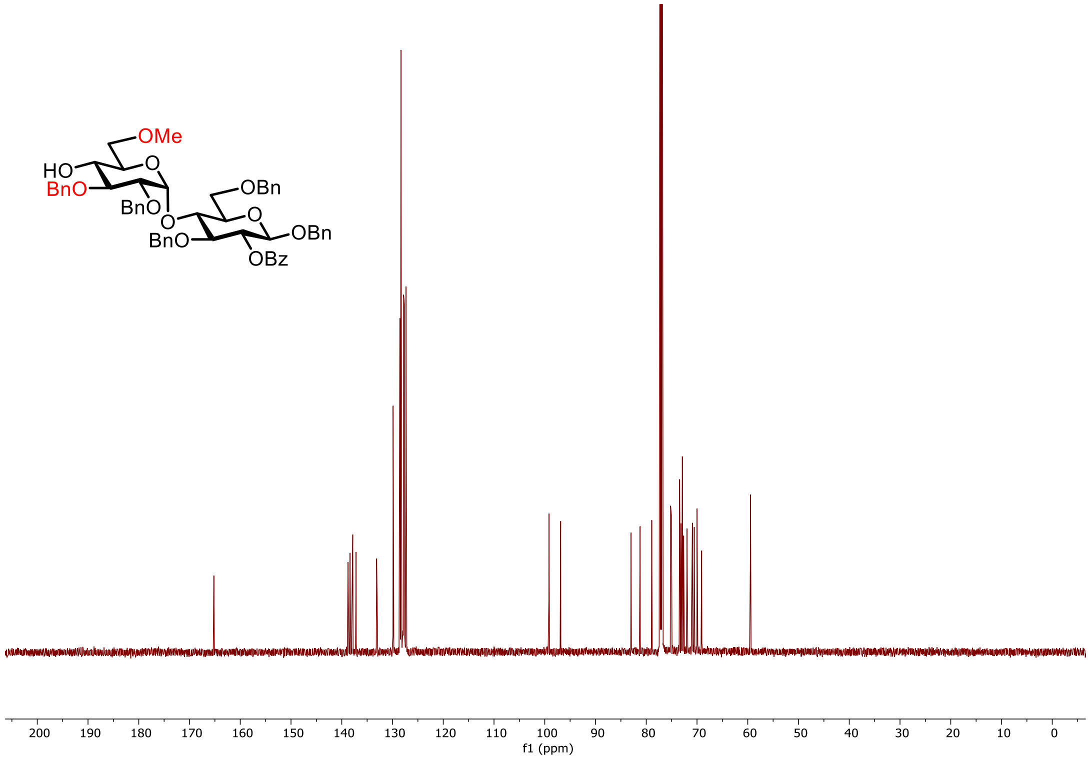

COSY NMR of 108a ($CDCl_3$)

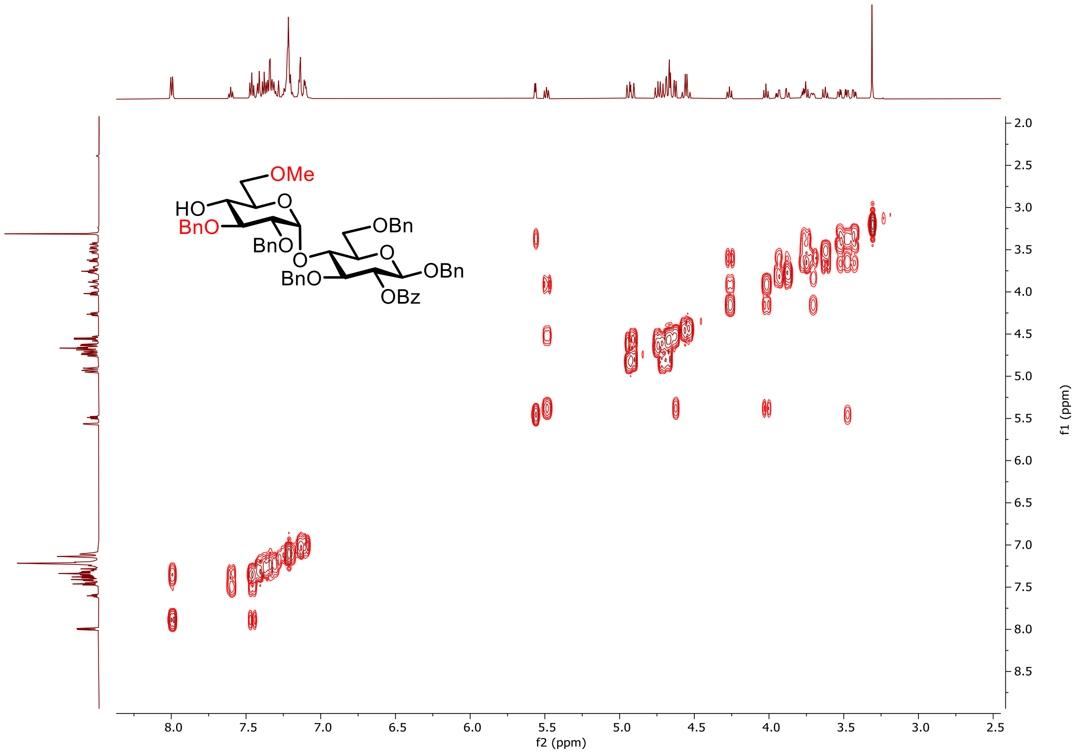

HSQC NMR of 108a ($CDCl_3$)

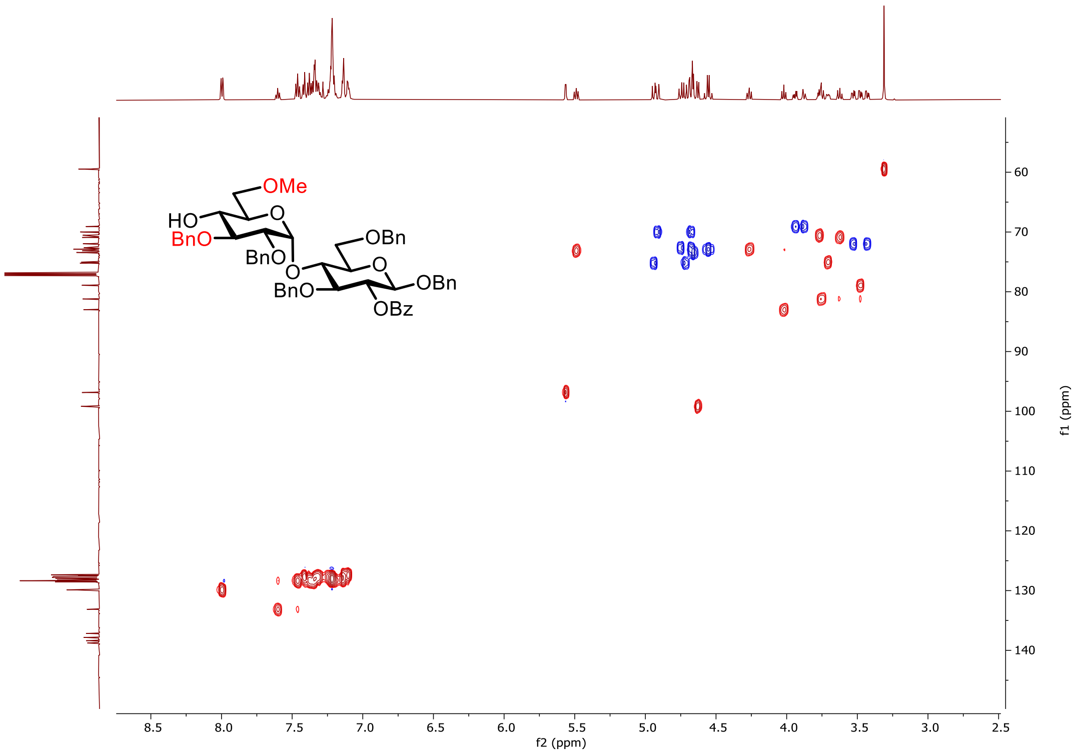

4.36 Isopropyl 2,6-di-*O*-benzyl-3-*O*-acetyl-D-glucopyranoside, 117

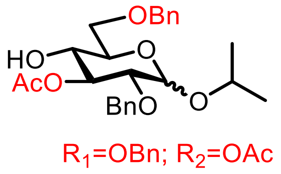

Total yield: 79% (35.0 mg). Ratio of anomer α : β = 1 : 1.4.

Spectrum data for isopropyl 2,6-di-*O*-benzyl-3-*O*-acetyl-α-D-glucopyranoside **117a**: ^{1}H NMR (400 MHz, $CDCl_3$) δ 7.39 – 7.29 (m, 10H), 5.25 (appt, *J* = 9.5 Hz, 1H), 4.92 (d, *J* = 3.7 Hz, 1H), 4.70 – 4.56 (m, 4H), 3.94 – 3.84 (m, 2H), 3.75 (dd, *J* = 10.4, 4.1 Hz, 1H), 3.71 – 3.65 (m, 2H), 3.54 (dd, *J* = 9.9, 3.7 Hz, 1H), 2.87 (d, *J* = 4.6 Hz, 1H), 2.14 (s, 3H), 1.26 (d, *J* = 6.3 Hz, 3H), 1.20 (d, *J* = 6.1 Hz, 3H); ^{13}C NMR (101 MHz, $CDCl_3$) δ 172.53, 138.03, 128.46, 128.40, 127.95, 127.93, 127.70, 127.65, 94.85, 76.36, 75.86, 73.61, 72.72, 70.63, 70.35, 69.91, 69.20, 23.26, 21.35, 21.16; $[\alpha]_D^{25}$ 67.91 (c = 1, $CHCl_3$); IR (neat) ν_{max} = 2925, 1749, 1234, 1052, 698 cm^{-1}; m/z (HRMS$^+$) [M + Na]$^+$ 467.2032 ($C_{25}H_{32}O_7Na^+$ requires 467.2040).

^{1}H NMR of crude 117 (600 MHz, $CDCl_3$)

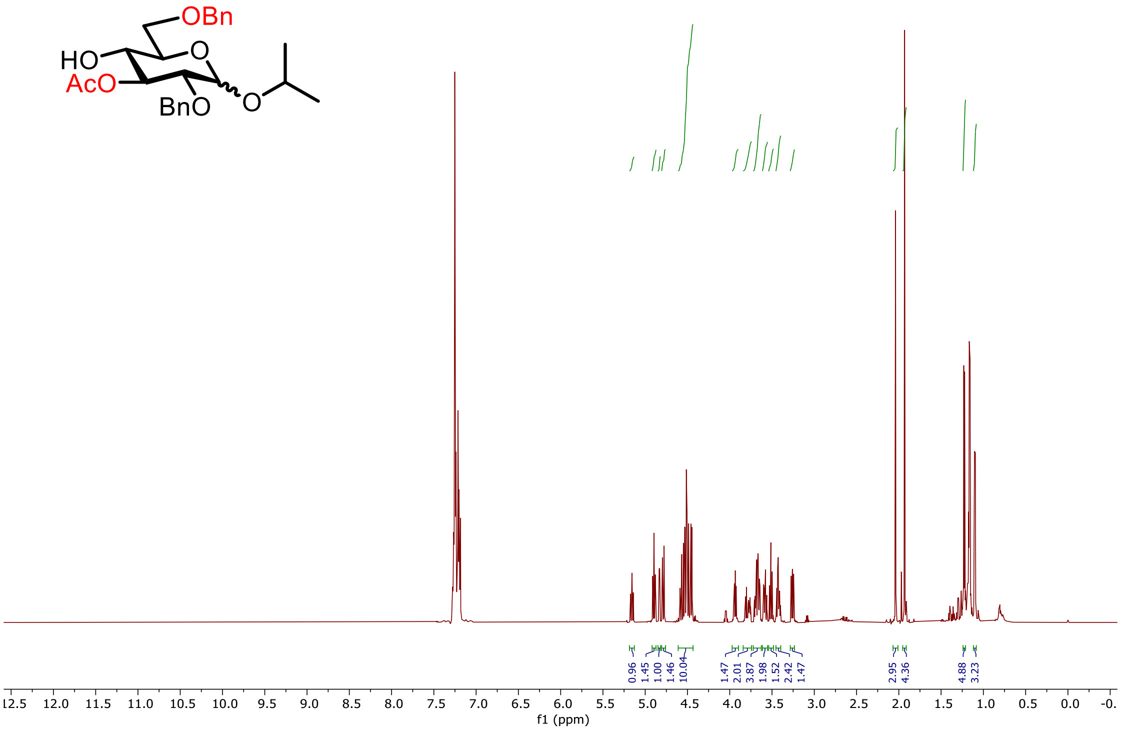

^{13}C NMR of crude 117 (151 MHz, $CDCl_3$)

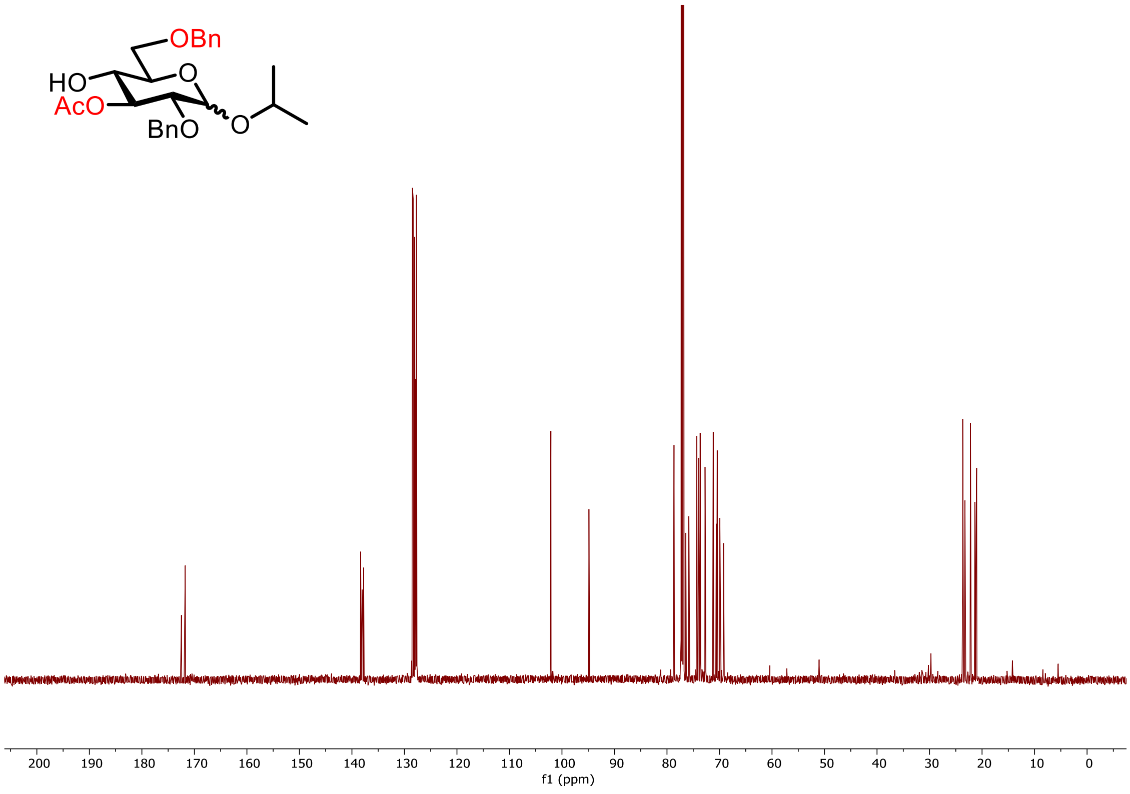

HSQC NMR of crude 117 ($CDCl_3$)

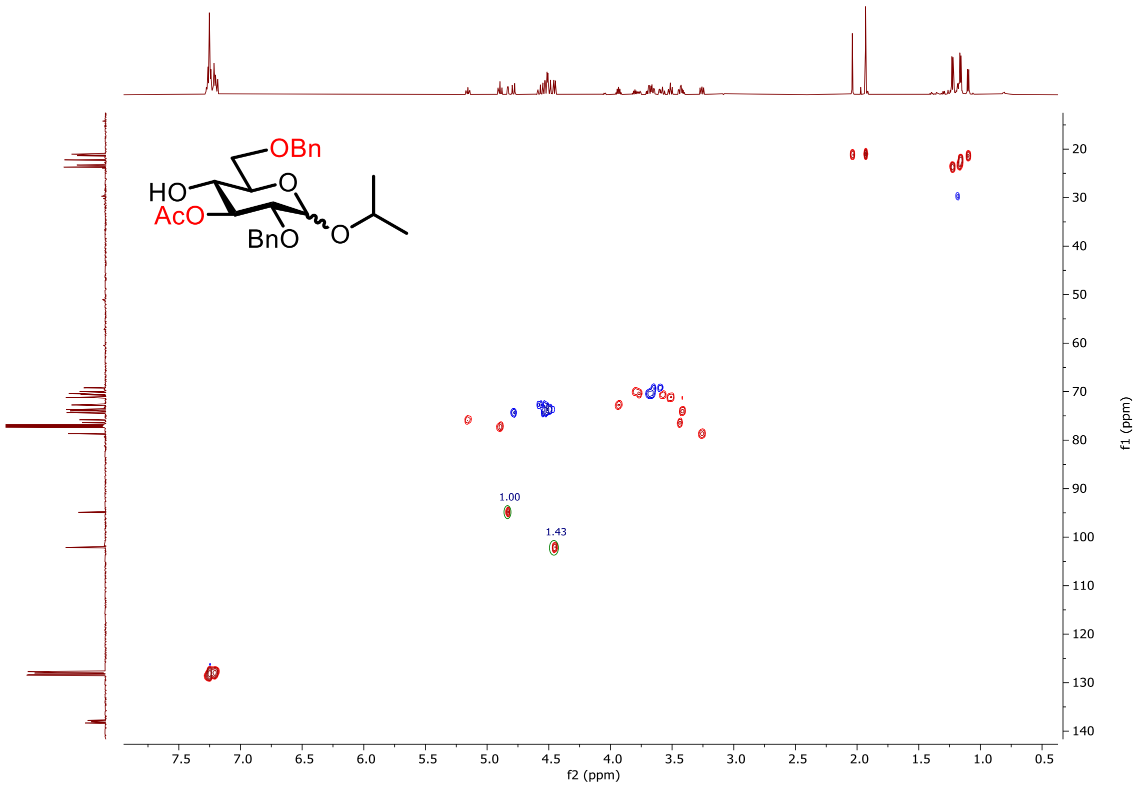

Coupled HSQC NMR of crude 117 ($CDCl_3$)

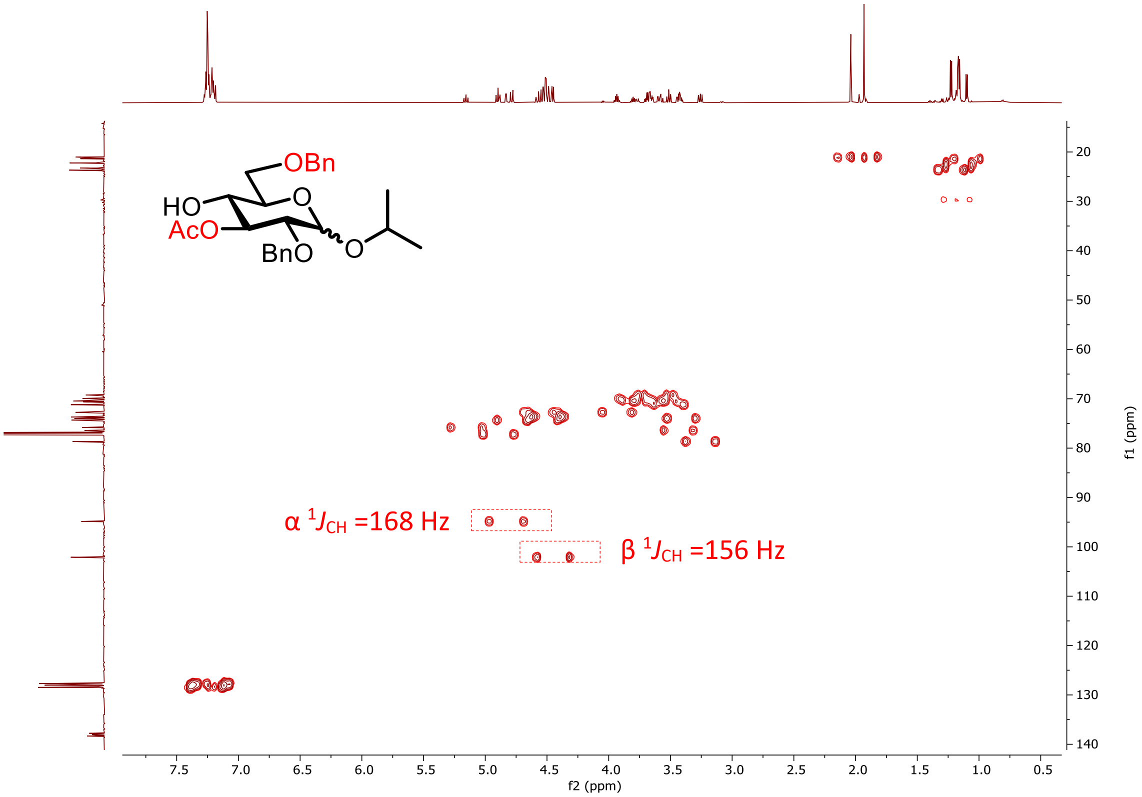

^{1}H NMR of 117a (400 MHz, $CDCl_3$)

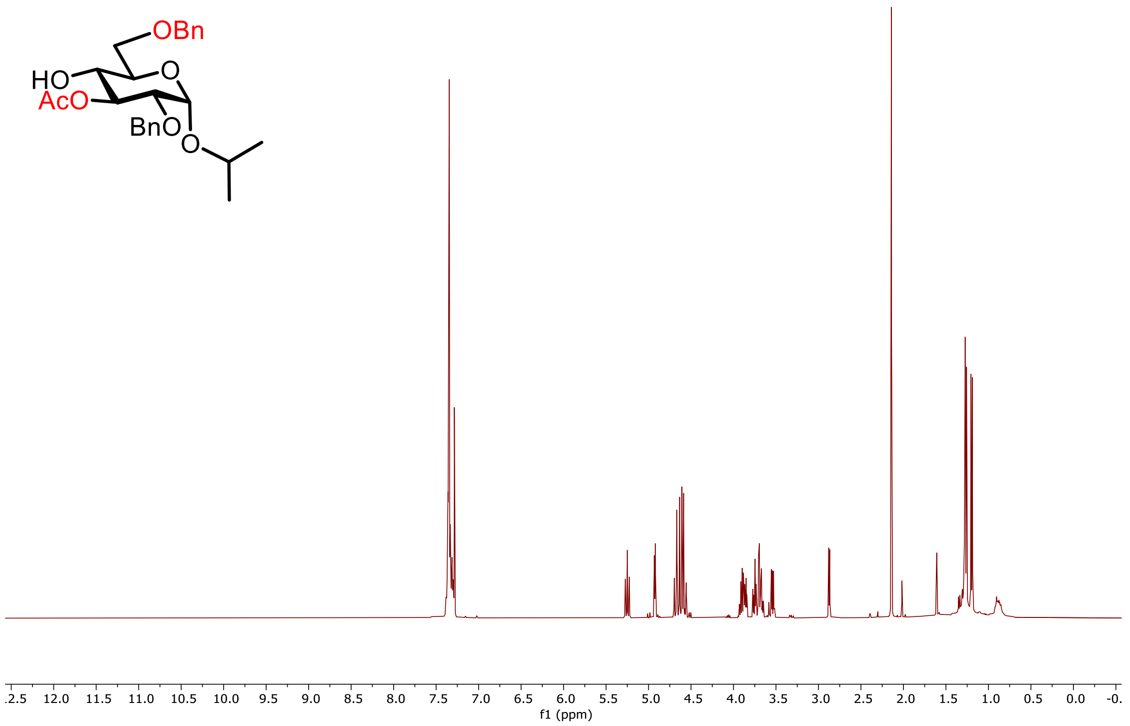

^{13}C NMR of 117a (101 MHz, $CDCl_3$)

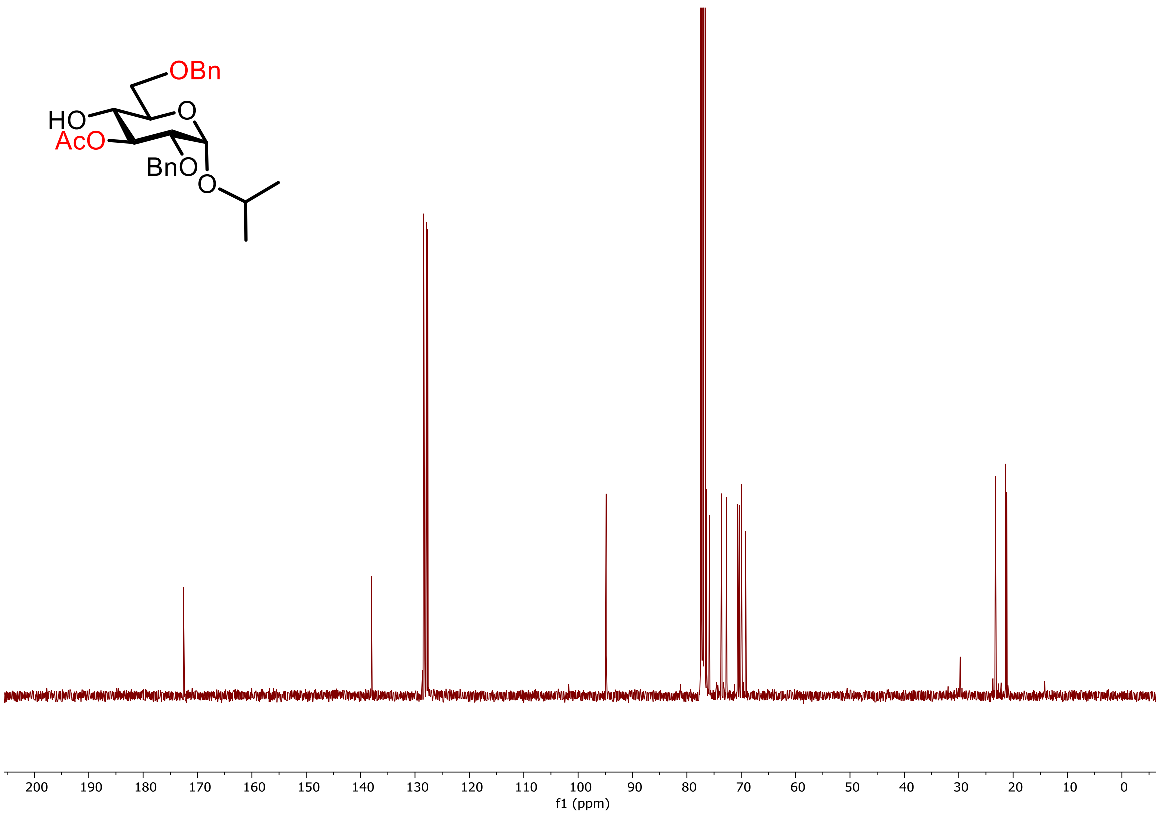

COSY NMR of 117a ($CDCl_3$)

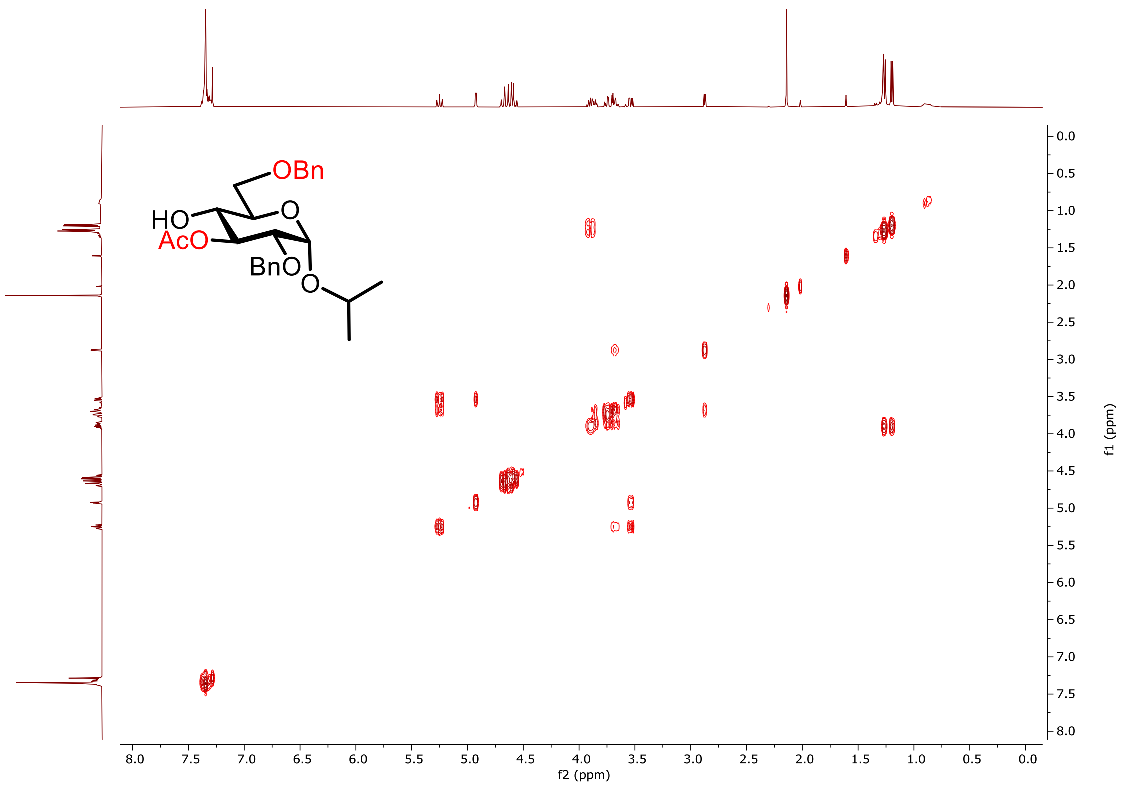

HSQC NMR of 117a ($CDCl_3$)

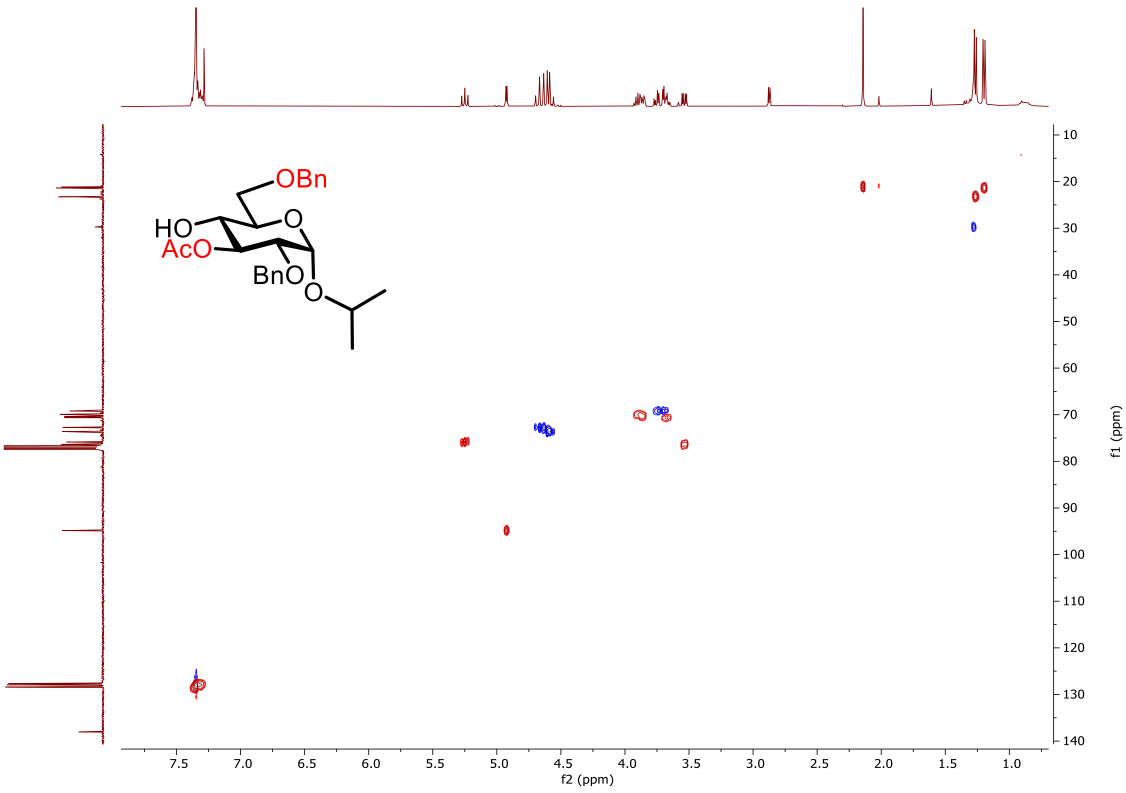

4.37 Isopropyl 2,6-di-*O*-benzyl-3-*O*-benzoyl-D-glucopyranoside, 118

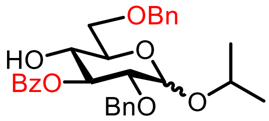

R_1=OBn; R_2=OBz

Total yield: 95% (48.0 mg). Ratio of anomer α : β = 1 : 1.1.

Spectrum data for isopropyl 2,6-di-*O*-benzyl-3-*O*-benzoyl-α-D-glucopyranoside **118a**: ^{1}H NMR (400 MHz, $CDCl_3$) δ 8.12 – 8.04 (m, 2H), 7.67 – 7.58 (m, 1H), 7.49 (appt, *J* = 7.8 Hz, 2H), 7.36 (appd, *J* = 4.4 Hz, 4H), 7.32 – 7.21 (m, 6H), 5.52 (appt, *J* = 9.5 Hz, 1H), 5.01 (d, *J* = 3.6 Hz, 1H), 4.72 – 4.55 (m, 4H), 3.98 – 3.92 (m, 2H), 3.85 (dd, *J* = 9.4, 4.3 Hz, 1H), 3.80 (dd, *J* = 10.5, 4.1 Hz, 1H), 3.72 (ddd, *J* = 10.0, 7.1, 3.4 Hz, 2H), 3.05 (d, *J* = 4.3 Hz, 1H), 1.30 (d, *J* = 6.2 Hz, 3H), 1.23 (d, *J* = 6.1 Hz, 3H); ^{13}C NMR (101 MHz, $CDCl_3$) δ 167.85, 137.97, 137.85, 133.32, 129.97, 129.81, 128.43, 128.40, 128.06, 127.93, 127.69, 127.67, 94.83, 76.47, 76.35, 73.63, 72.59, 70.56, 70.46, 70.01, 69.17, 23.30, 21.40; $[\alpha]_D^{25}$ 70.09 (c = 1, $CHCl_3$); IR (neat) ν_{max} = 2925, 1725, 1271, 1056, 711 cm^{-1}; m/z (HRMS$^+$) $[M + Na]^+$ 529.2197 ($C_{30}H_{34}O_7Na^+$ requires 529.2197).

^{1}H NMR of crude 118 (600 MHz, $CDCl_3$)

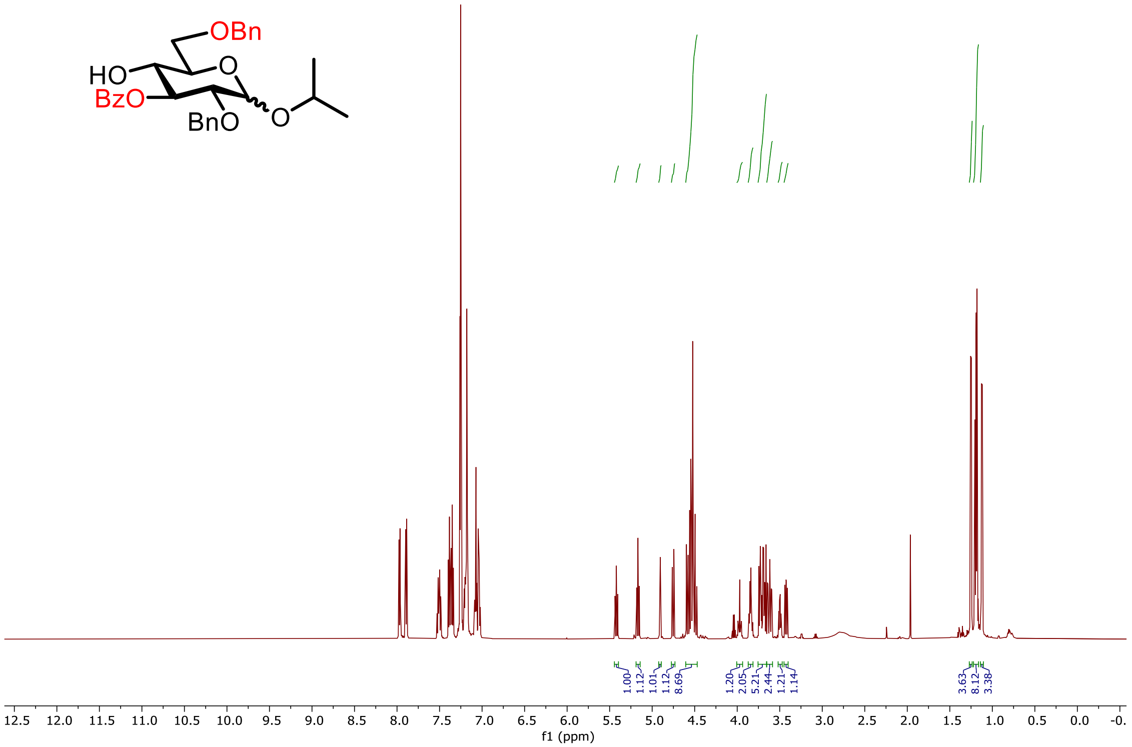

^{13}C NMR of crude 118 (151 MHz, $CDCl_3$)

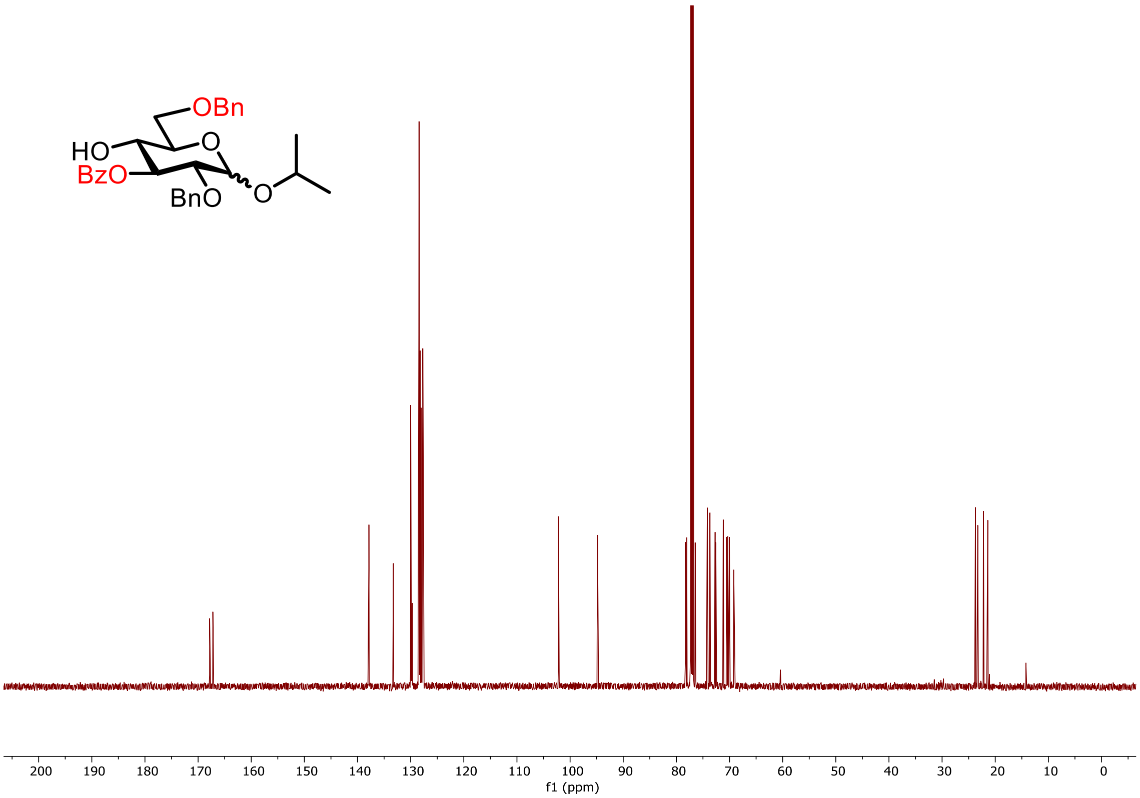

HSQC NMR of crude 118 ($CDCl_3$)

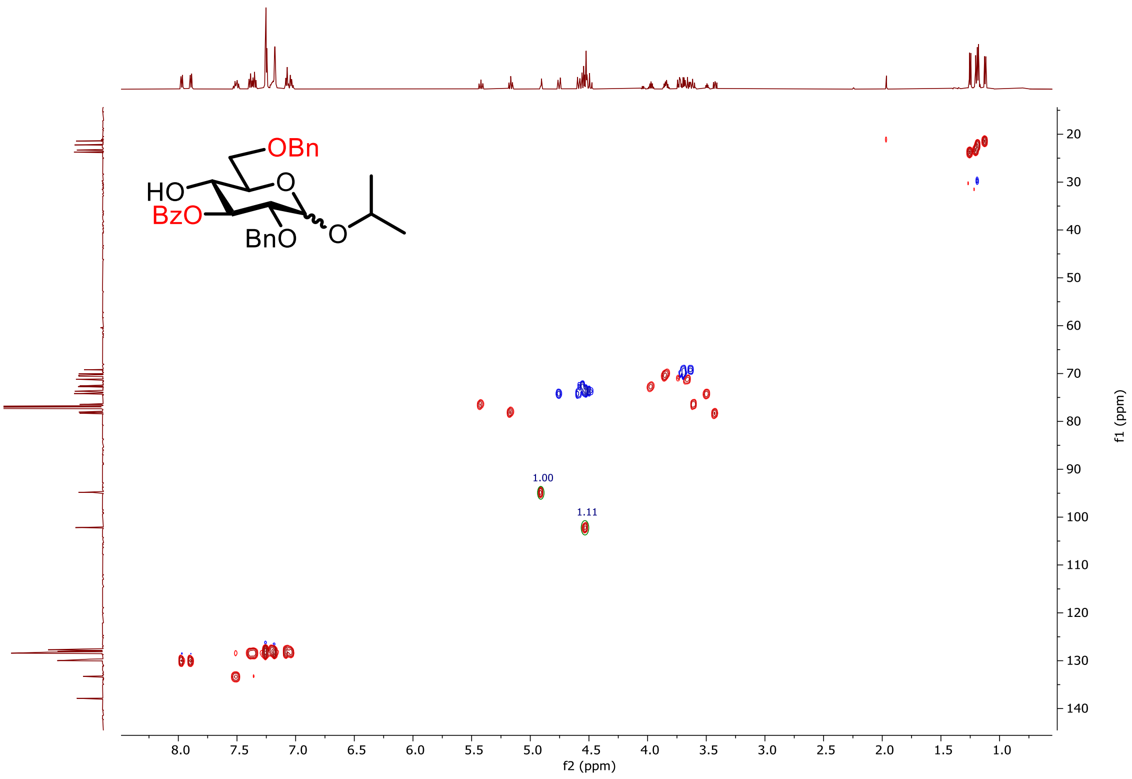

Coupled HSQC NMR of crude 118 ($CDCl_3$)

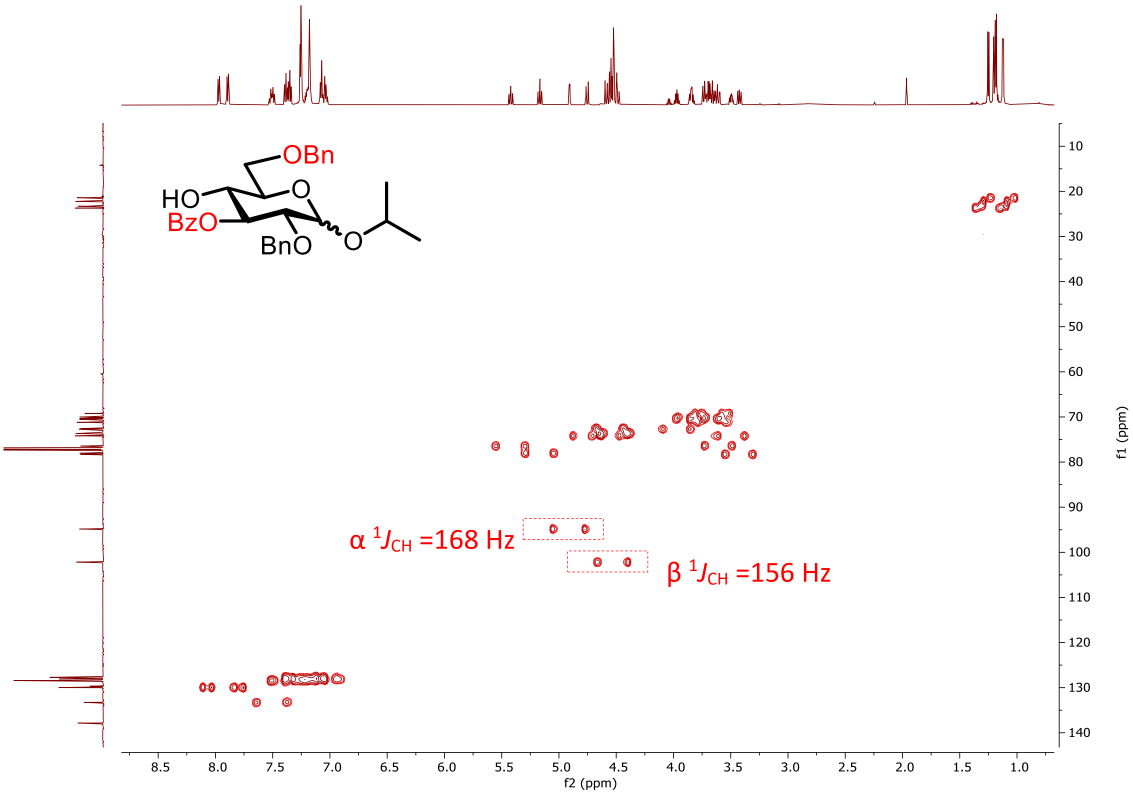

^{1}H NMR of 118a (400 MHz, $CDCl_3$)

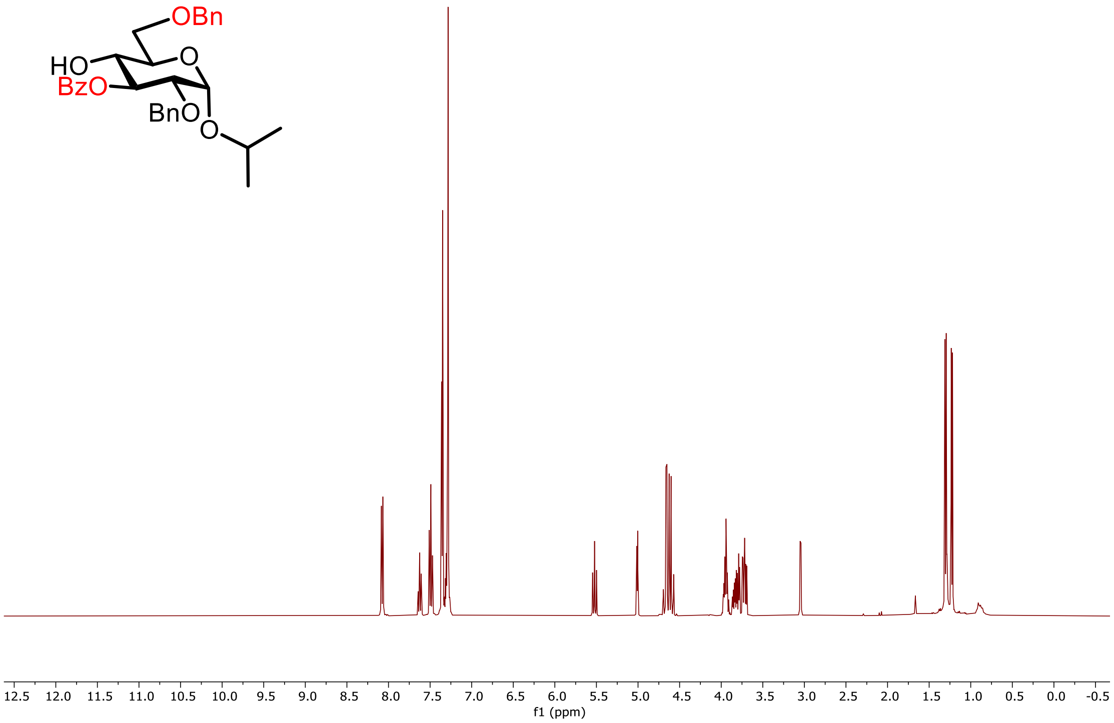

^{13}C NMR of 118a (101 MHz, $CDCl_3$)

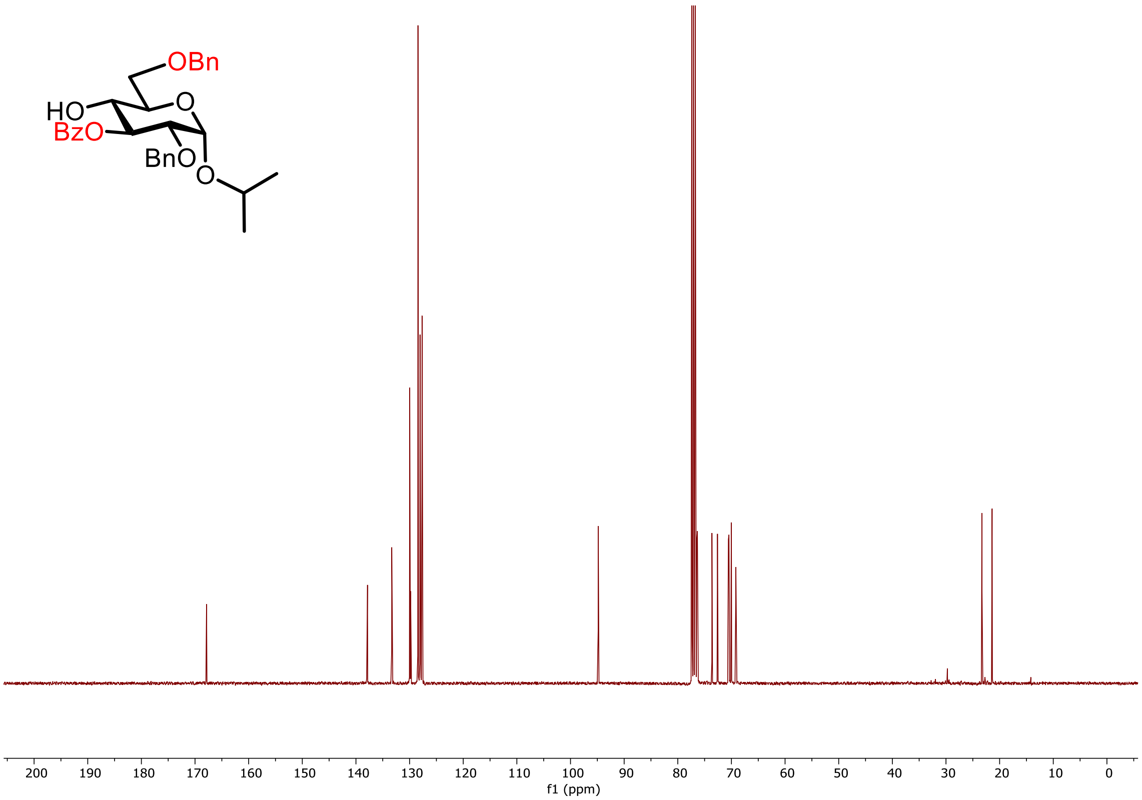

COSY NMR of 118a ($CDCl_3$)

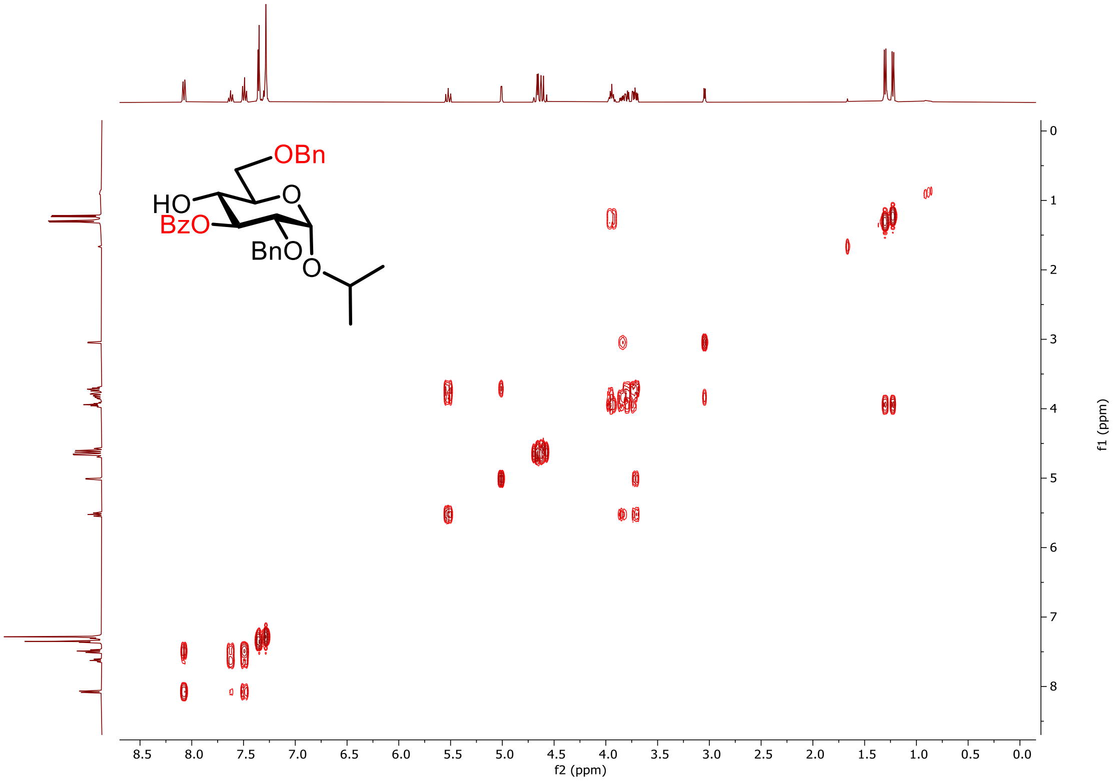

HSQC NMR of 118a ($CDCl_3$)

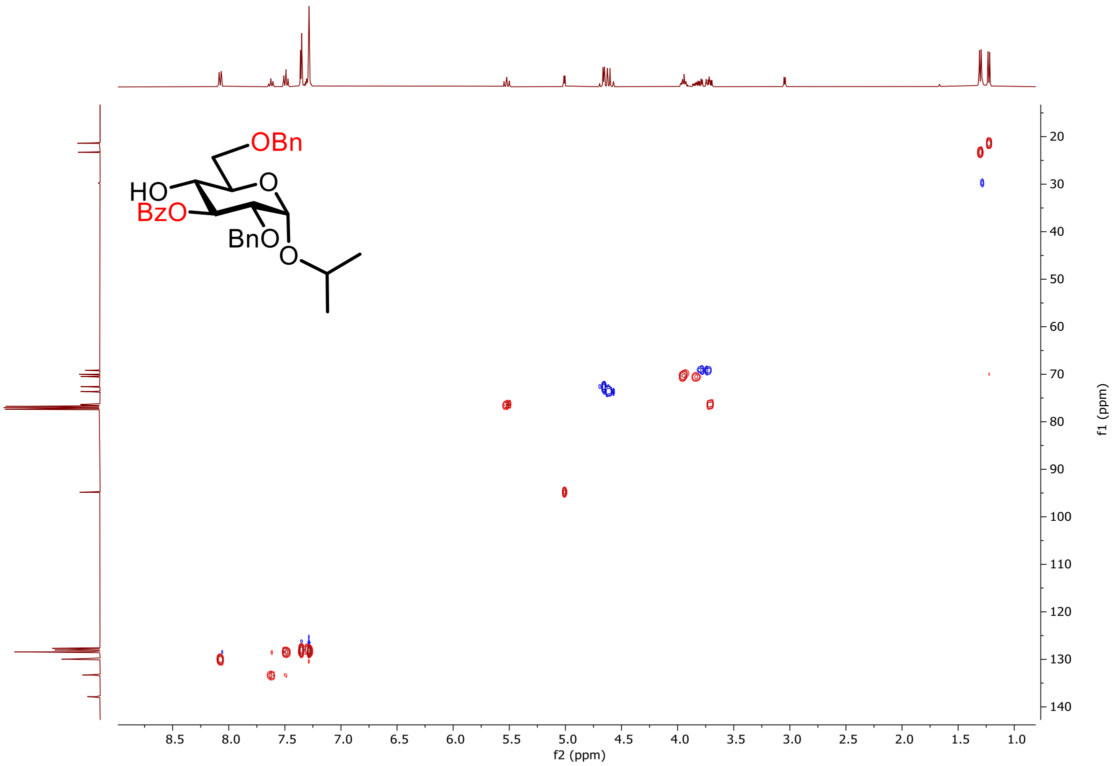

4.38 Isopropyl 2,6-di-*O*-benzyl-3-*O*-pivaloyl-D-glucopyranoside, 119

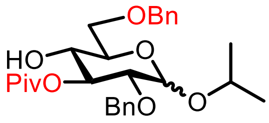

R_1=OBn; R_2=OPiv

Total yield: 73% (35.6 mg). Ratio of anomer α : β = 1 : 1.3.

Spectrum data for isopropyl 2,6-di-*O*-benzyl-3-*O*-pivaloyl-α-D-glucopyranoside **119a**: ^{1}H NMR (400 MHz, $CDCl_3$) δ 7.40 – 7.25 (m, 10H), 5.20 (appt, *J* = 9.5 Hz, 1H), 4.86 (d, *J* = 3.7 Hz, 1H), 4.74 (d, *J* = 12.1 Hz, 1H), 4.63 – 4.53 (m, 3H), 3.92 – 3.82 (m, 2H), 3.78 – 3.62 (m, 3H), 3.58 (dd, *J* = 9.9, 3.7 Hz, 1H), 3.08 (d, *J* = 4.5 Hz, 1H), 1.32 – 1.22 (m, 12H), 1.18 (d, *J* = 6.1 Hz, 3H); ^{13}C NMR (101 MHz, $CDCl_3$) δ 180.63, 138.05, 137.89, 128.49, 128.37, 128.14, 128.05, 127.67, 127.64, 94.83, 76.58, 76.16, 73.62, 73.16, 70.67, 70.65, 69.73, 69.10, 39.02, 27.24, 23.25, 21.32; $[\alpha]_D^{25}$ 57.44 (c = 1, $CHCl_3$); IR (neat) ν_{max} = 2925, 1736, 1056, 698 cm^{-1}; m/z (HRMS$^+$) $[M + Na]^+$ 509.2503 ($C_{28}H_{38}O_7Na^+$ requires 509.2510).

^{1}H NMR of crude 119 (600 MHz, $CDCl_3$)

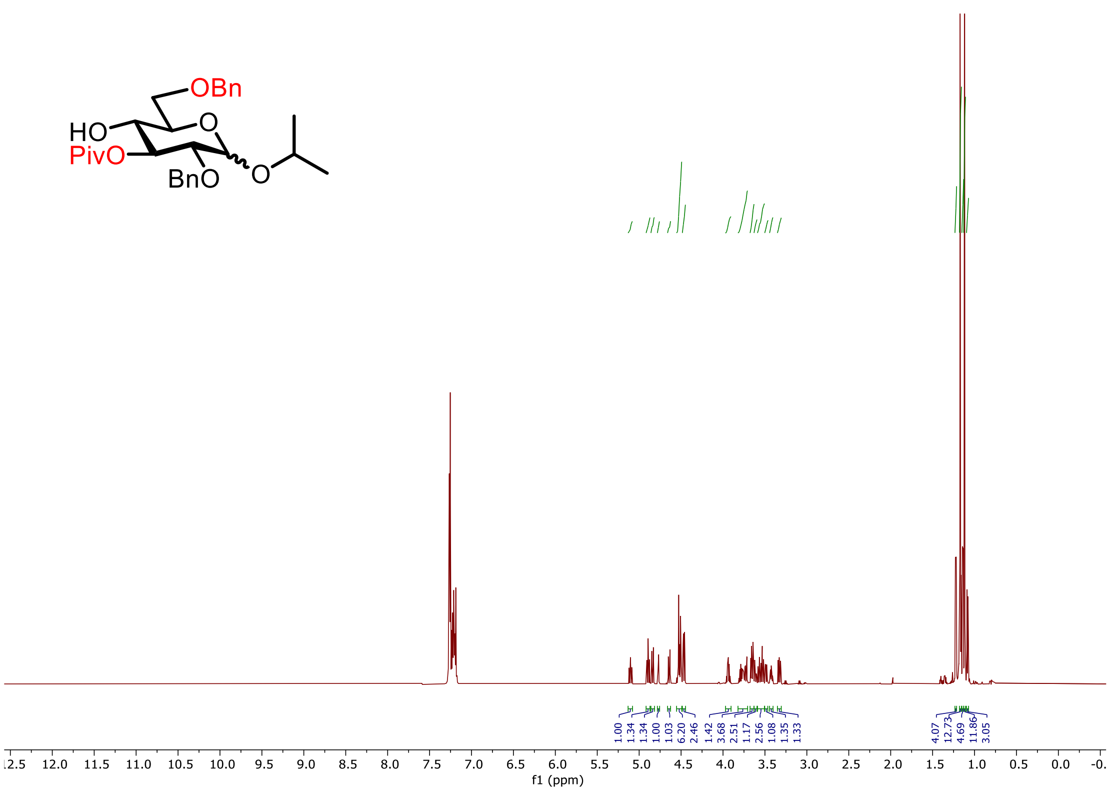

^{13}C NMR of crude 119 (151 MHz, $CDCl_3$)

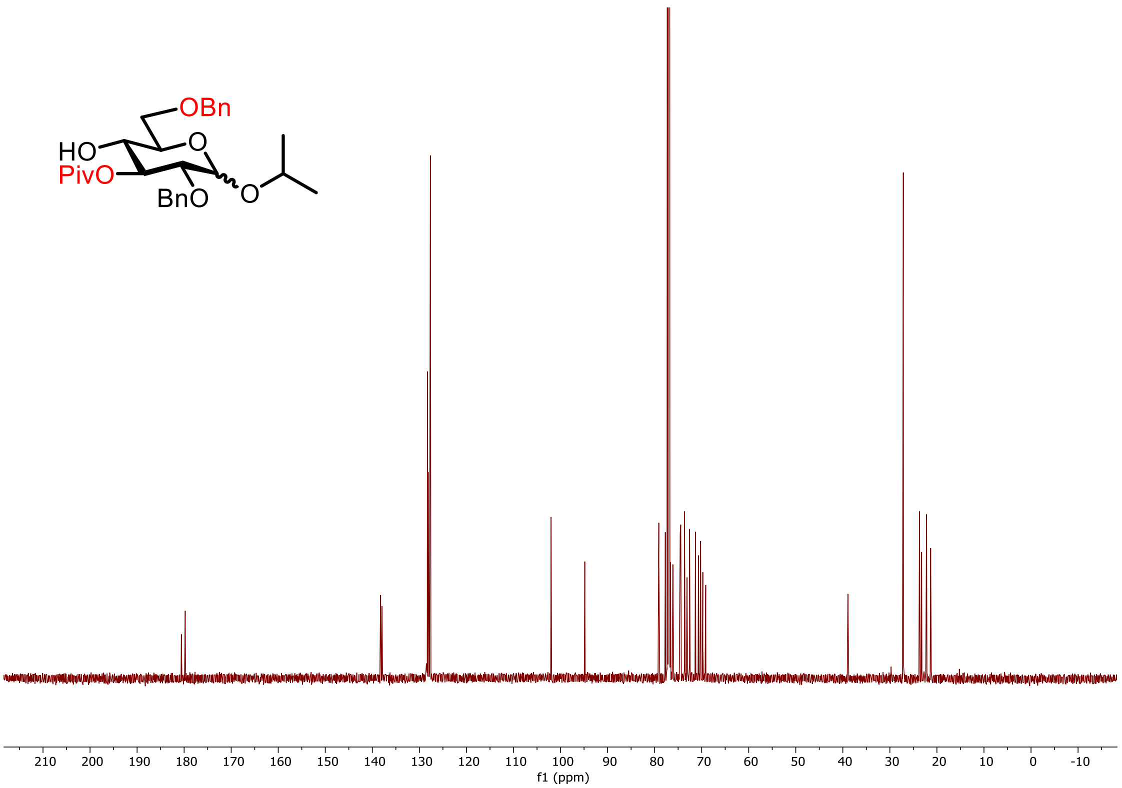

HSQC NMR of crude 119 ($CDCl_3$)

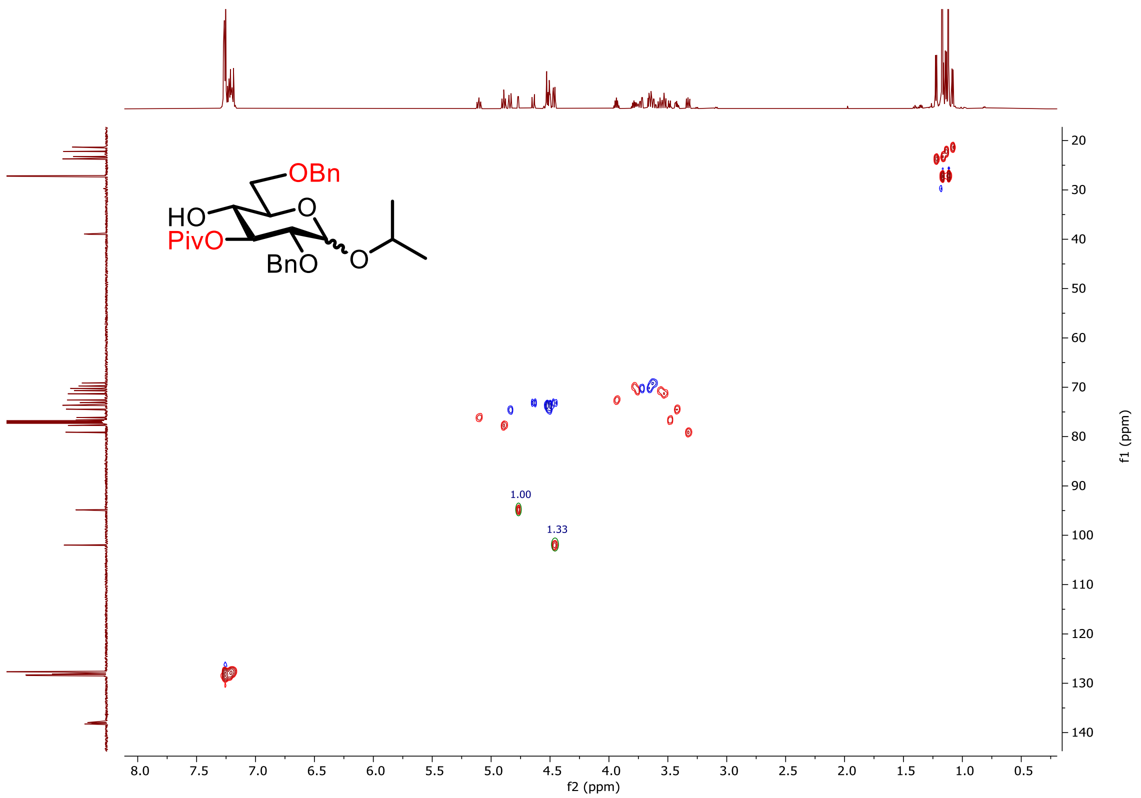

Coupled HSQC NMR of crude 119 ($CDCl_3$)

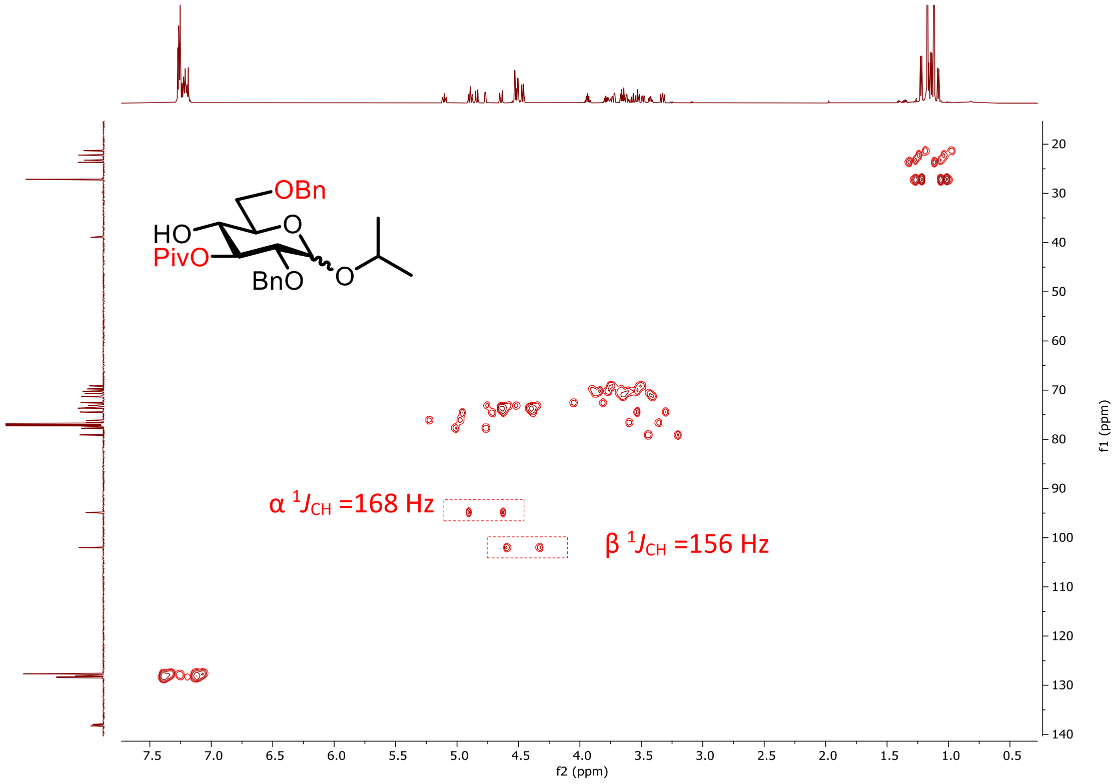

^{1}H NMR of 119a (400 MHz, $CDCl_3$)

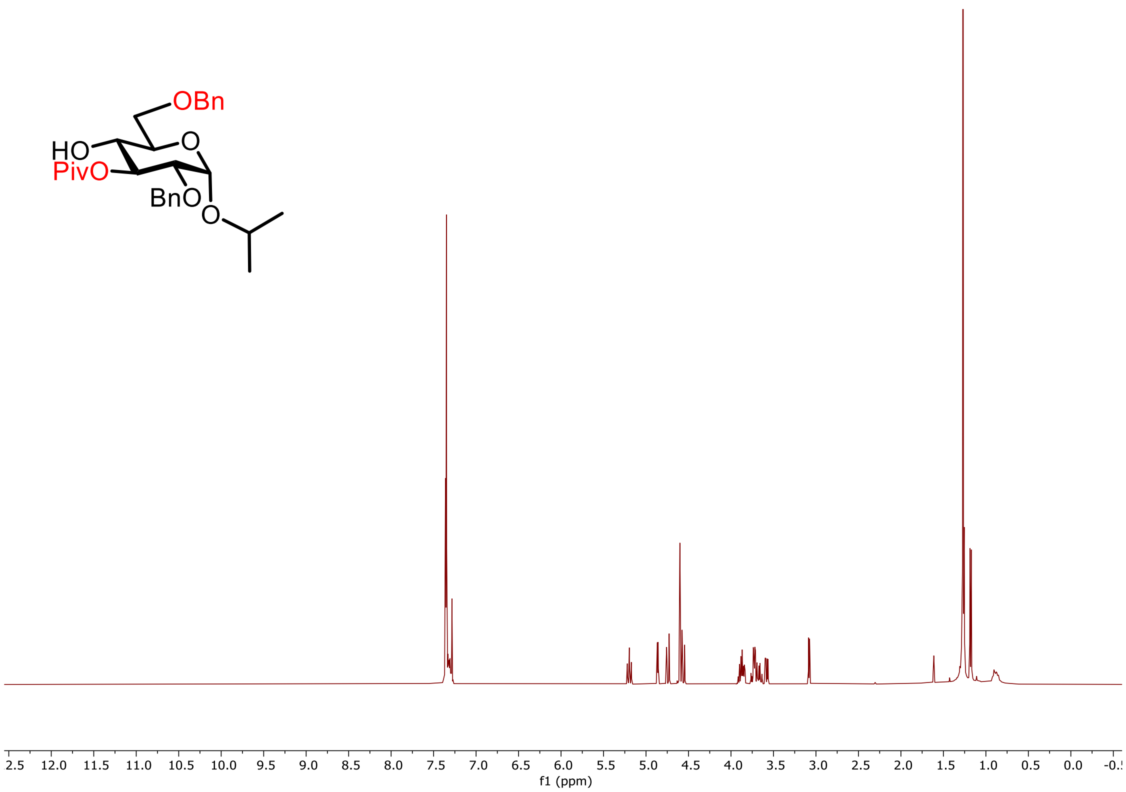

^{13}C NMR of 119a (101 MHz, $CDCl_3$)

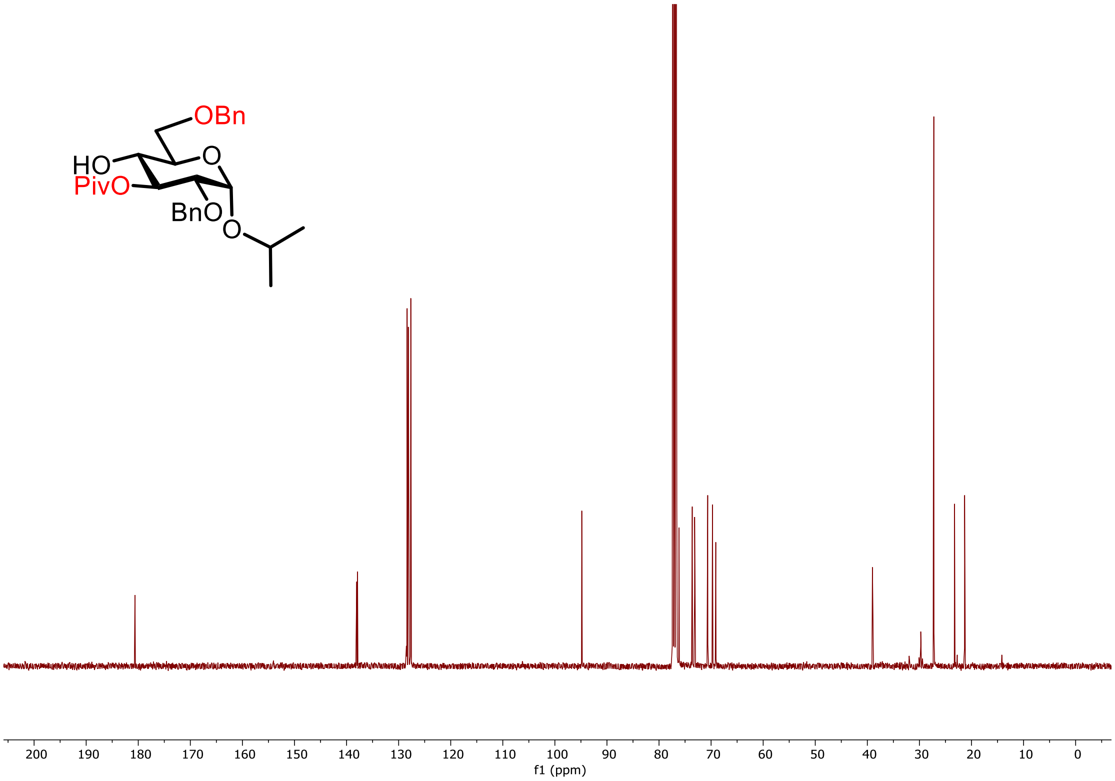

COSY NMR of 119a ($CDCl_3$)

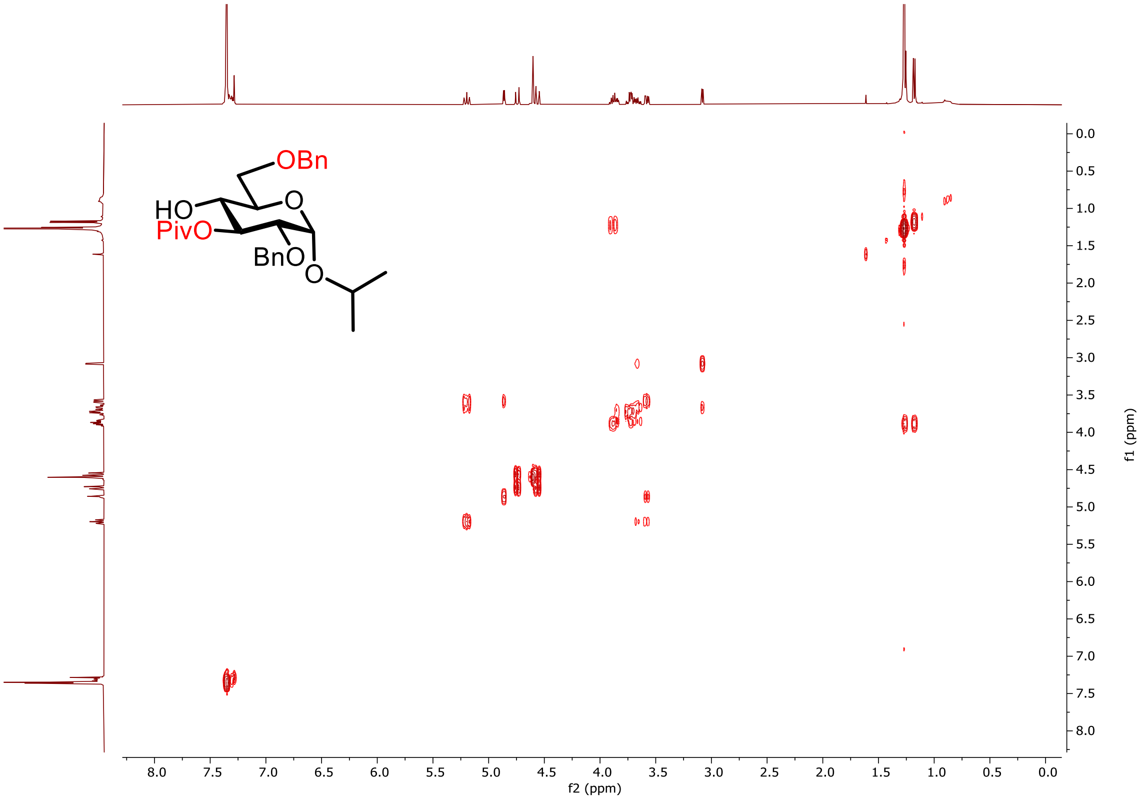

HSQC NMR of 119a ($CDCl_3$)

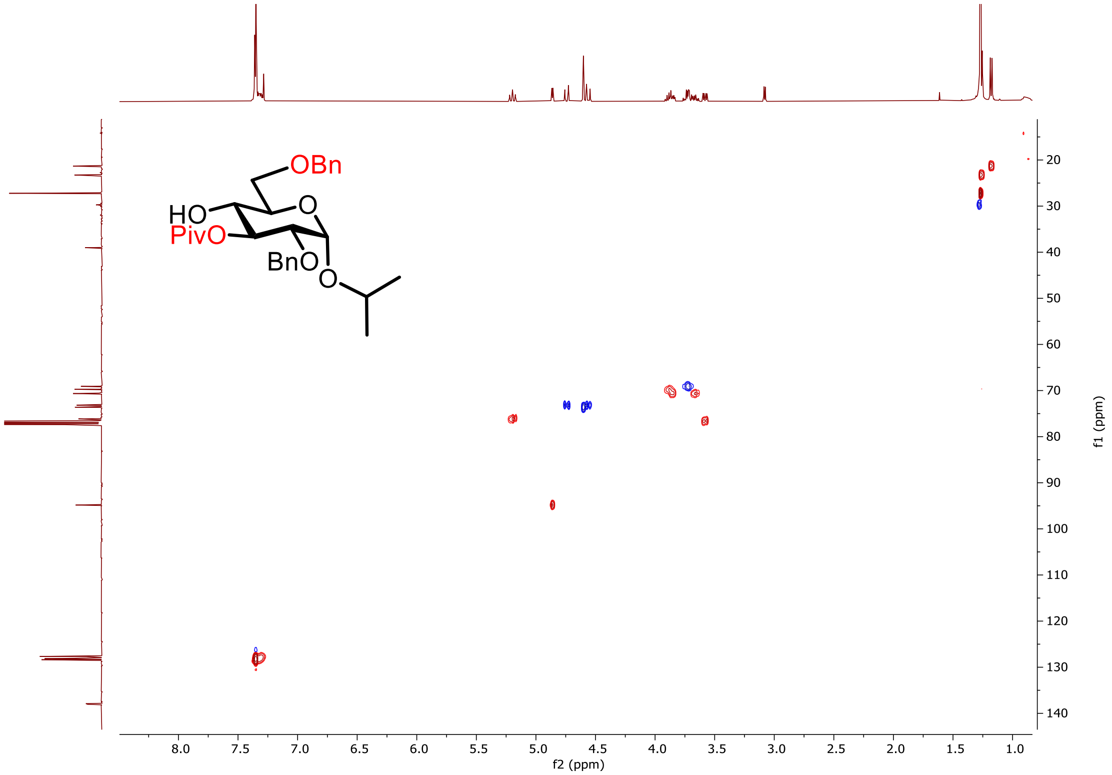

4.39 Benzyl 2,6-di-*O*-benzyl-3-*O*-acetyl-D-glucopyranosyl-(1→4)-2-*O*-benzoyl-3,6-di-*O*-benzyl-β-D-glucopyranoside, 120

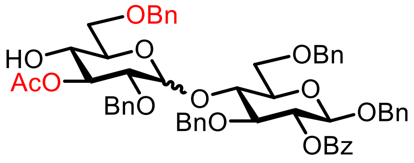

R_1=OBn; R_2=OAc

Total yield: 65% (61.4 mg). Ratio of anomer α : β = 2.9 : 1.

Spectrum data for benzyl 2,6-di-*O*-benzyl-3-*O*-acetyl-D-glucopyranosyl-(1→4)-2-*O*-benzoyl-3,6-di-*O*-benzyl-β-D-glucopyranoside **120a**: ^{1}H NMR (400 MHz, $CDCl_3$) δ 8.01 – 7.96 (m, 2H), 7.62 – 7.57 (m, 1H), 7.45 (t, *J* = 7.8 Hz, 2H), 7.39 – 7.18 (m, 18H), 7.17 – 7.08 (m, 7H), 5.61 (d, *J* = 3.7 Hz, 1H), 5.47 (dd, *J* = 9.0, 7.7 Hz, 1H), 5.25 (appt, *J* = 9.6 Hz, 1H), 4.90 (d, *J* = 12.6 Hz, 1H), 4.73 – 4.57 (m, 6H), 4.49 (dd, *J* = 12.2, 7.7 Hz, 2H), 4.42 (d, *J* = 12.2 Hz, 1H), 4.35 (d, *J* = 12.3 Hz, 1H), 4.26 (dd, *J* = 9.5, 8.4 Hz, 1H), 4.01 – 3.93 (m, 2H), 3.86 – 3.80 (m, 2H), 3.69 – 3.58 (m, 3H), 3.50 (dd, *J* = 10.2, 4.0 Hz, 1H), 3.44 (dd, *J* = 10.1, 3.7 Hz, 1H), 2.71 (d, *J* = 4.3 Hz, 1H), 2.05 (s, 3H); ^{13}C NMR (101 MHz, $CDCl_3$) δ 171.78, 165.23, 138.35, 137.94, 137.76, 137.73, 137.11, 133.14, 129.88, 129.83, 128.45, 128.36, 128.34, 128.32, 128.30, 128.18, 127.80, 127.74, 127.72, 127.68, 127.55, 127.31, 127.26, 99.20, 96.60, 82.98, 76.31, 74.87, 74.72, 73.62, 73.34, 73.28, 73.20, 72.84, 72.72, 70.76, 70.55, 70.04, 69.44, 68.93, 21.11; $[\alpha]_D^{25}$ 41.58 (c = 1, $CHCl_3$); IR (neat) ν_{max} = 3474, 2872, 1729, 1454, 1267, 1060, 698 cm^{-1}; m/z (HRMS$^+$) $[M + Na]^+$ 961.3794 ($C_{56}H_{58}O_{13}Na^+$ requires 961.3770).

^{1}H NMR of crude 120 (600 MHz, $CDCl_3$)

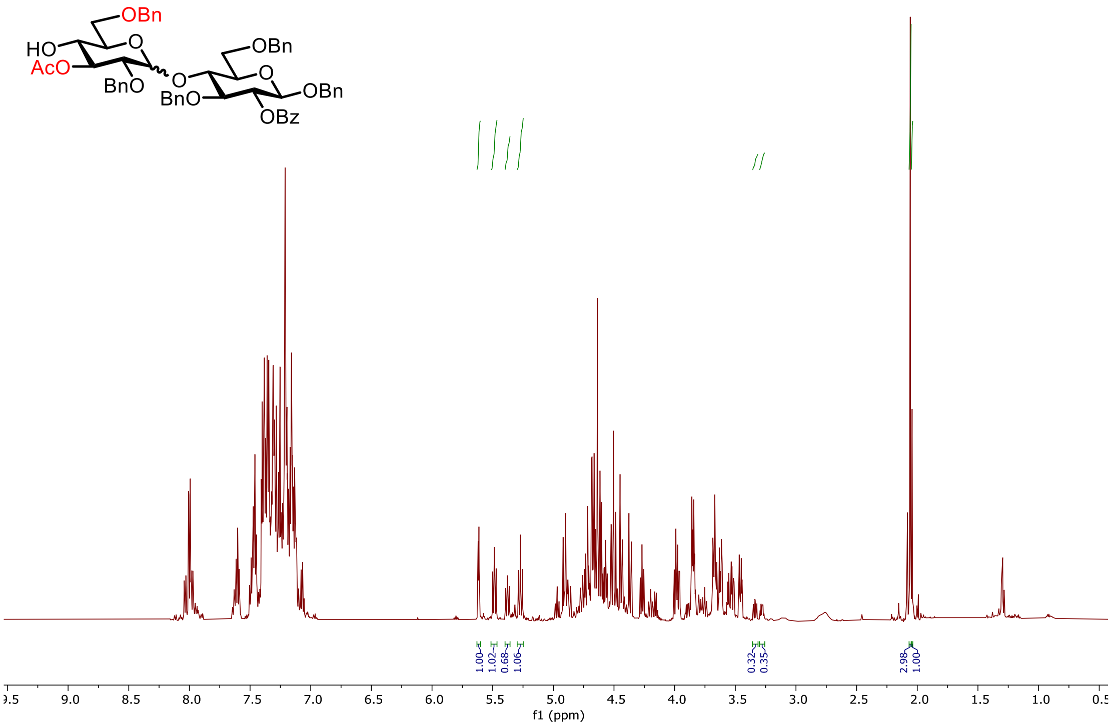

^{13}C NMR of crude 120 (151 MHz, $CDCl_3$)

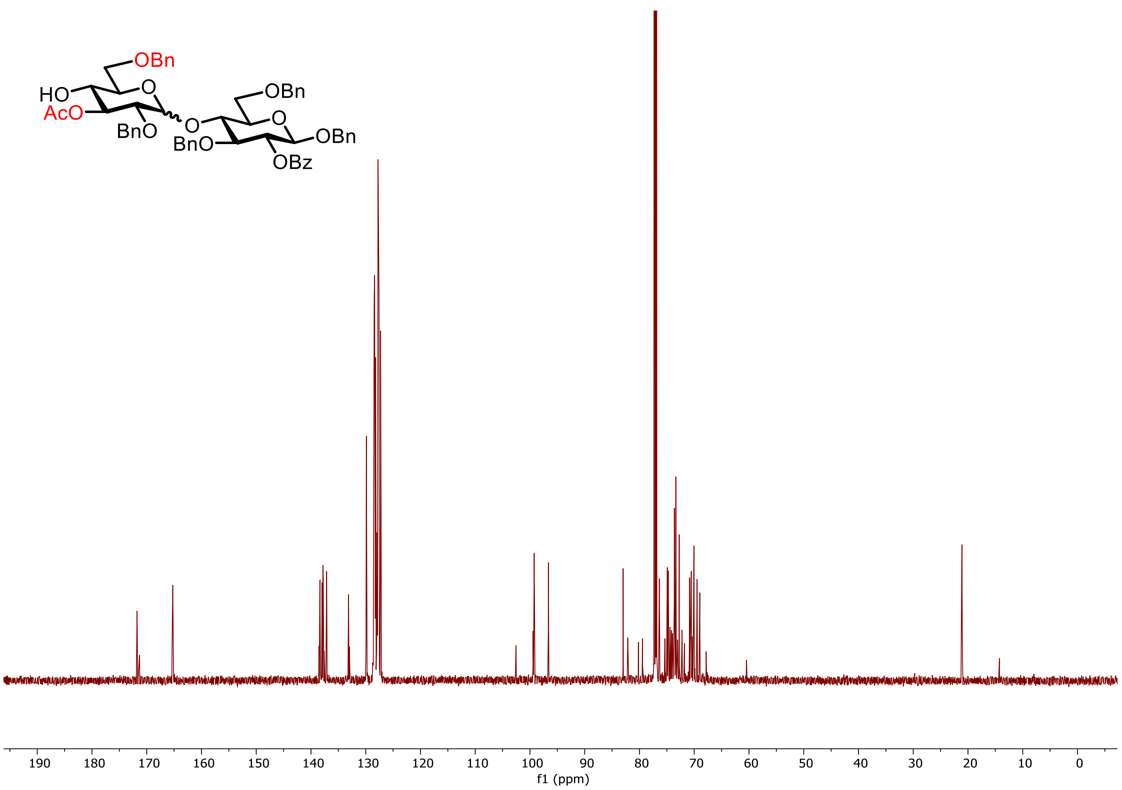

HSQC NMR of crude 120 ($CDCl_3$)

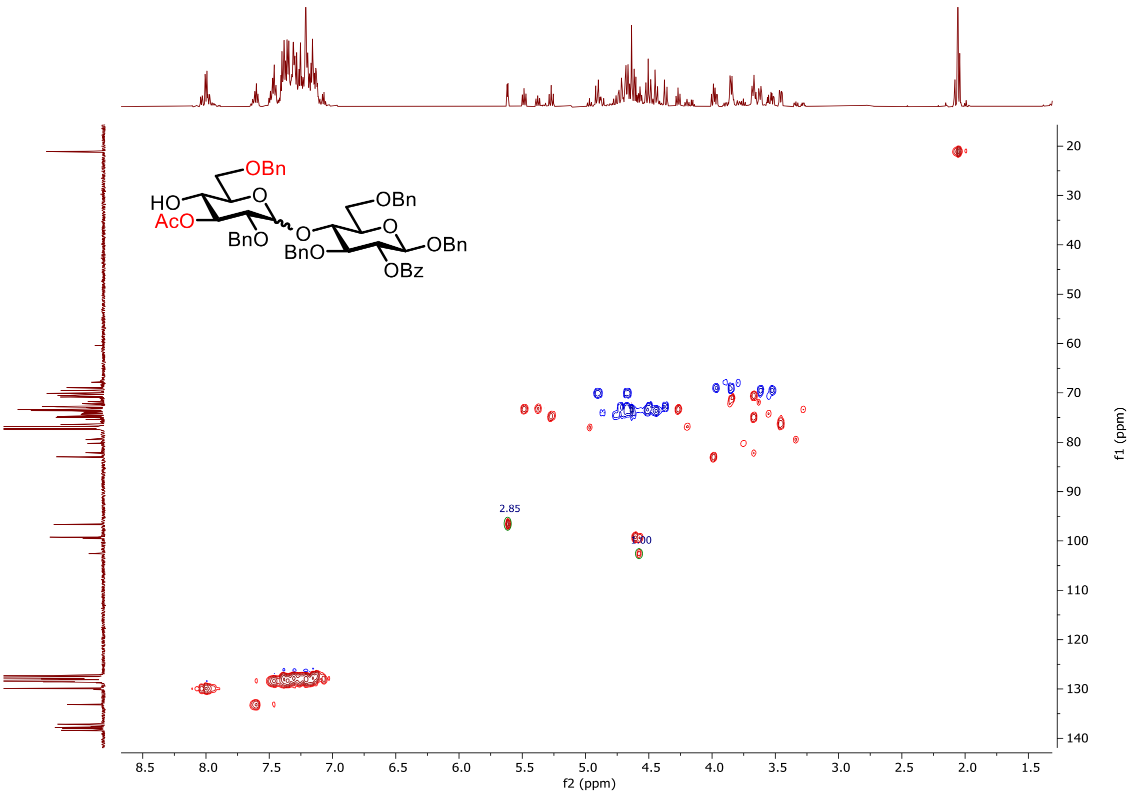

Coupled HSQC NMR of crude 120 ($CDCl_3$)

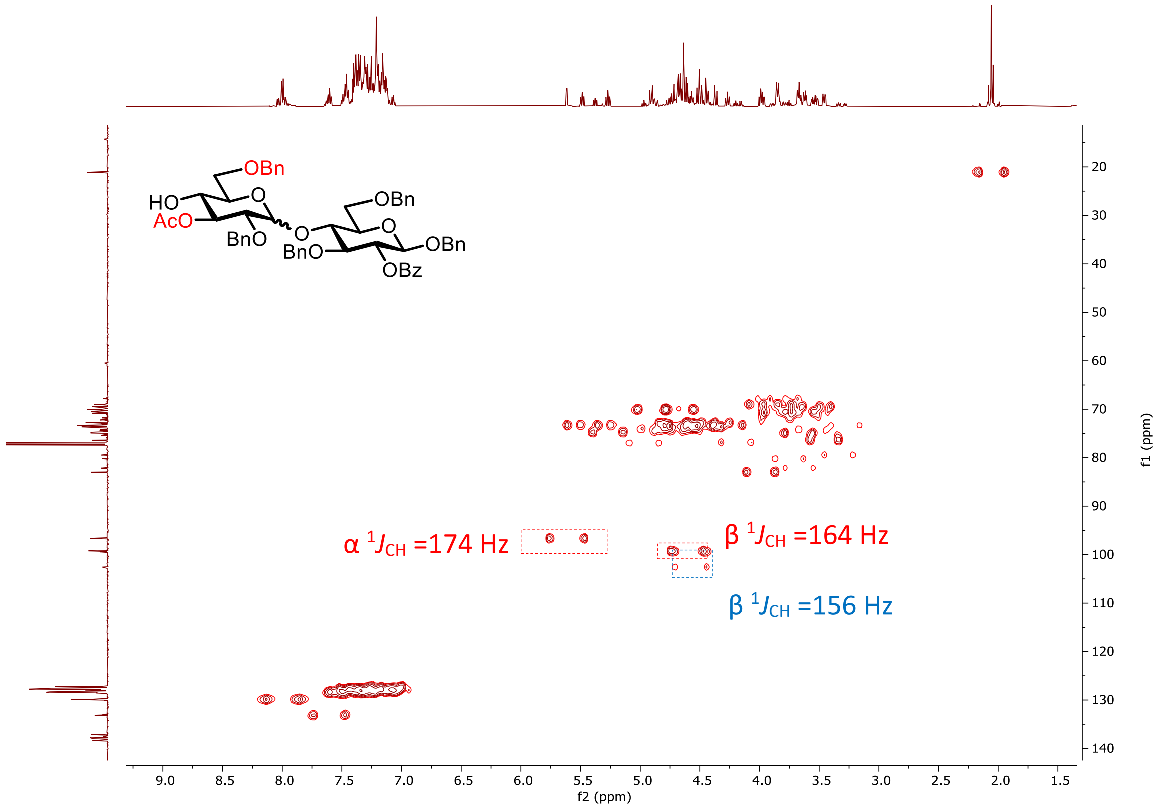

^{1}H NMR of 120a (400 MHz, $CDCl_3$)

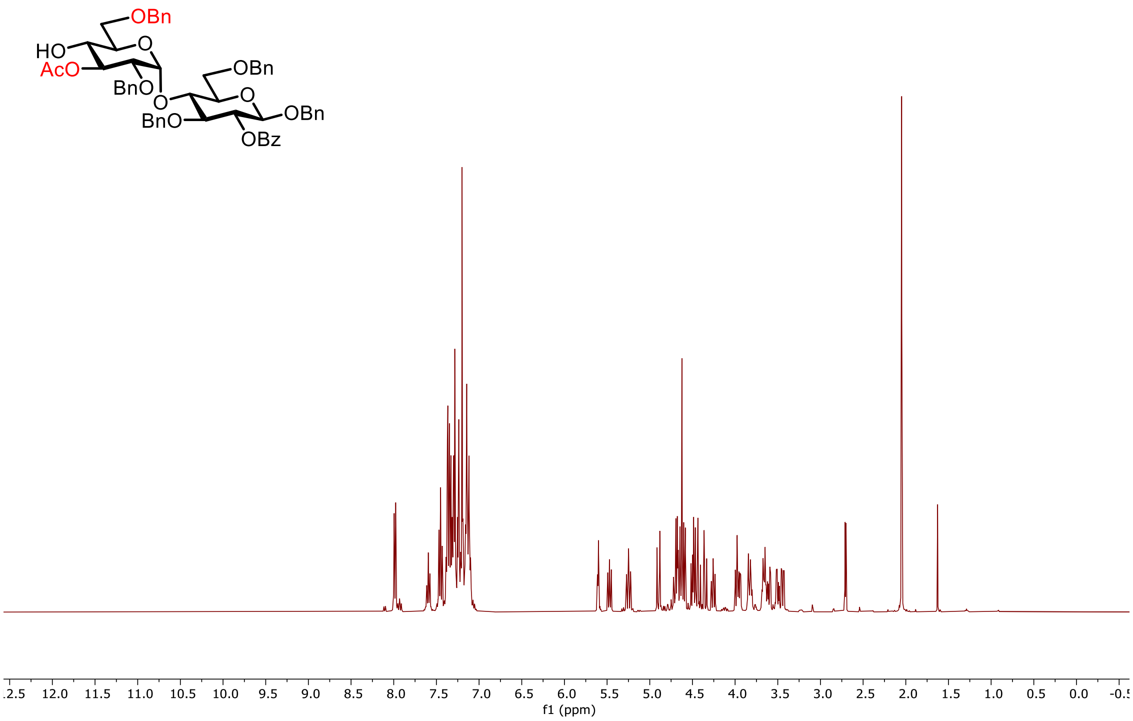

^{13}C NMR of 120a (101 MHz, $CDCl_3$)

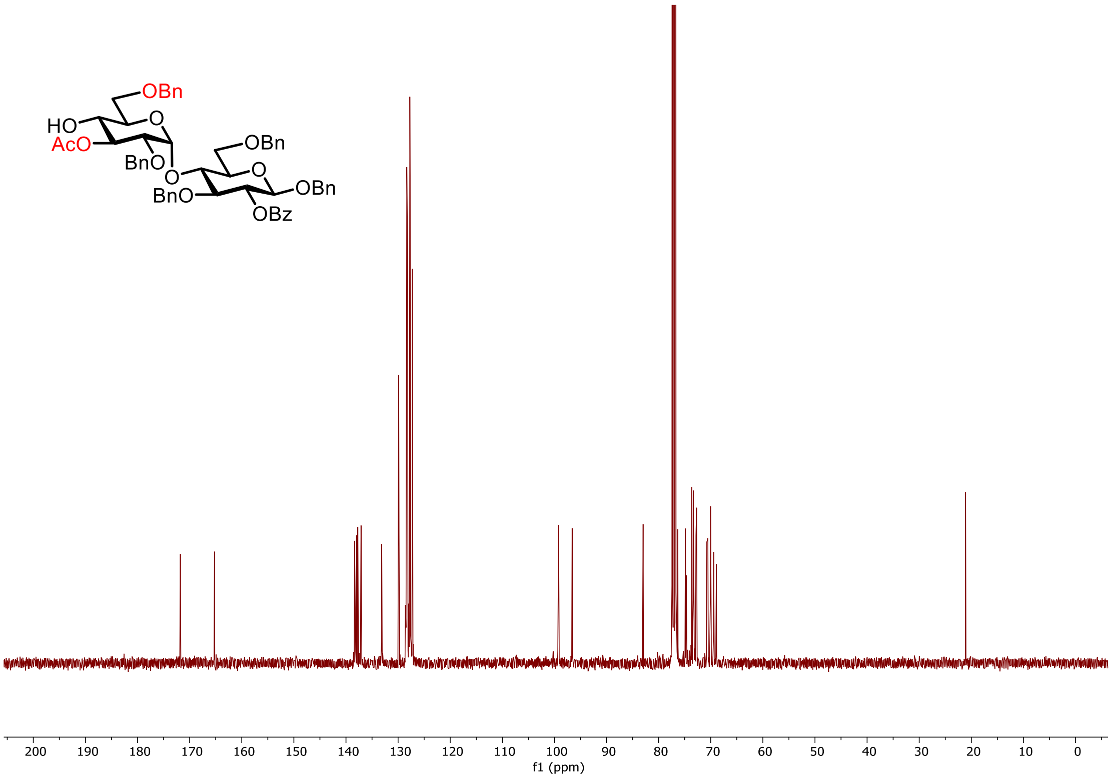

COSY NMR of 120a ($CDCl_3$)

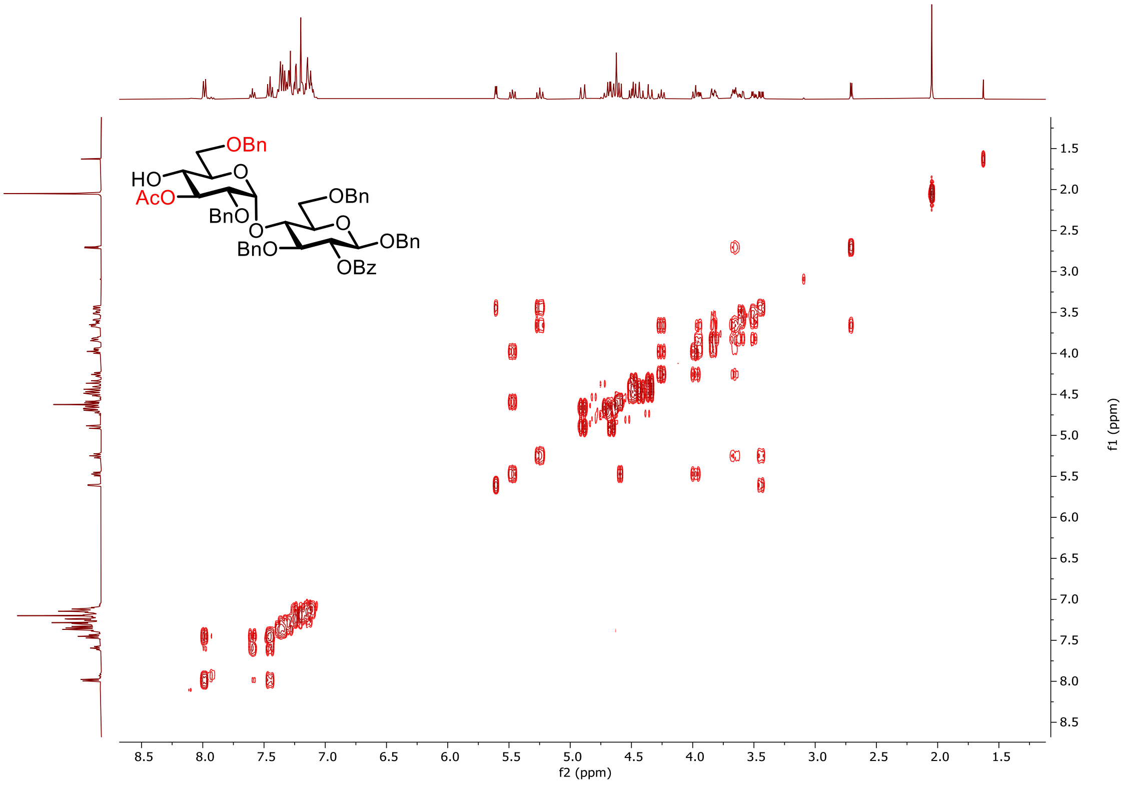

HSQC NMR of 120a ($CDCl_3$)

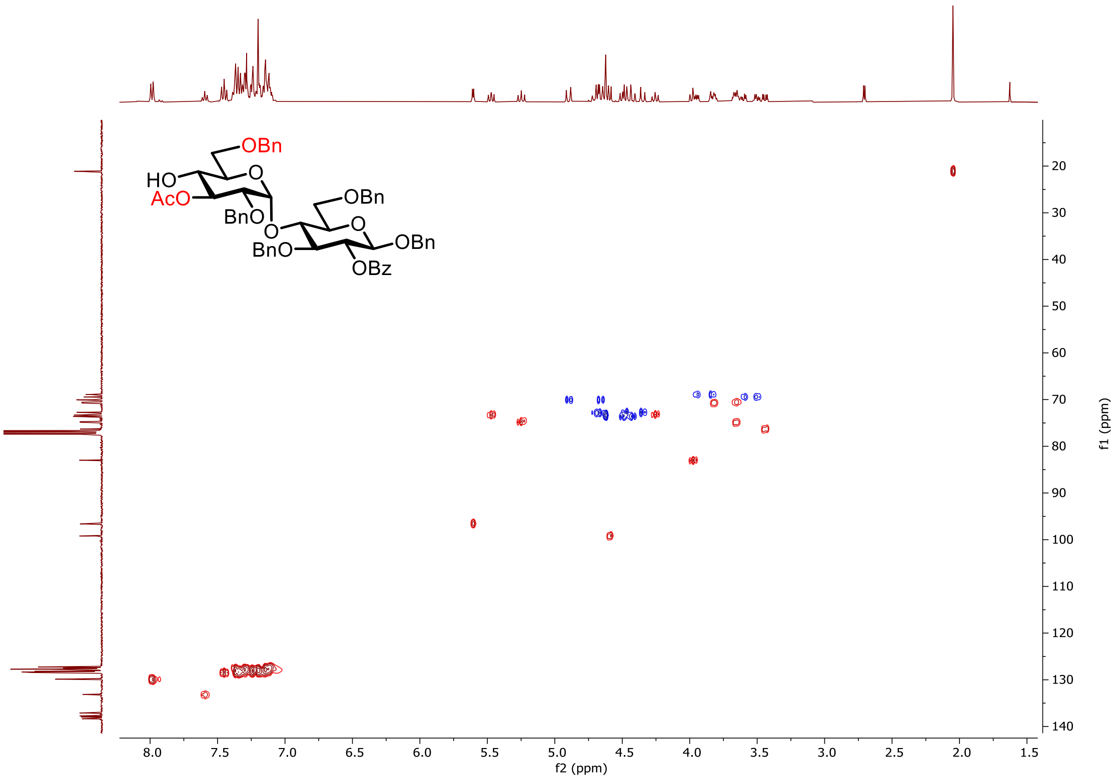

4.40 Benzyl 2,6-di-*O*-benzyl-3-*O*-benzoyl-D-glucopyranosyl-(1→4)-2-*O*-benzoyl-3,6-di-*O*-benzyl-β-D-glucopyranoside, 121

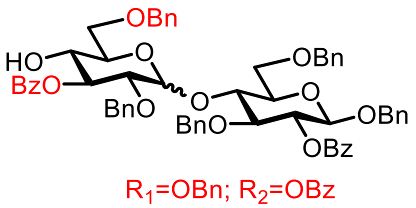

Total yield: 64% (63.7 mg). Ratio of anomer α : β = 4.9 : 1.

Spectrum data for benzyl 2,6-di-*O*-benzyl-3-*O*-benzoyl-α-D-glucopyranosyl-(1→4)-2-*O*-benzoyl-3,6-di-*O*-benzyl-β-D-glucopyranoside **121a**: ^{1}H NMR (400 MHz, $CDCl_3$) δ 8.05 – 7.96 (m, 4H), 7.63 – 7.57 (m, 2H), 7.50 – 7.42 (m, 4H), 7.42 – 7.27 (m, 10H), 7.26 – 7.06 (m, 13H), 7.02 – 6.98 (m, 2H), 5.66 (d, *J* = 3.6 Hz, 1H), 5.58 – 5.46 (m, 2H), 4.91 (d, *J* = 12.6 Hz, 1H), 4.78 – 4.59 (m, 6H), 4.56 – 4.41 (m, 3H), 4.37 – 4.28 (m, 2H), 4.06 – 3.98 (m, 2H), 3.95 – 3.78 (m, 3H), 3.73 – 3.51 (m, 4H), 2.79 (d, *J* = 4.1 Hz, 1H); ^{13}C NMR (101 MHz, $CDCl_3$) δ 167.13, 165.26, 138.37, 137.94, 137.78, 137.40, 137.14, 133.26, 133.14, 129.93, 129.89, 129.83, 129.78, 128.45, 128.39, 128.37, 128.30, 128.23, 128.18, 127.92, 127.80, 127.74, 127.68, 127.56, 127.54, 127.39, 127.32, 99.25, 96.52, 82.96, 76.03, 75.23, 74.93, 73.64, 73.34, 73.22, 73.17, 72.78, 72.40, 70.85, 70.50, 70.06, 69.42, 68.89; $[\alpha]_D^{25}$ 39.04 (c = 1, $CHCl_3$); IR (neat) ν_{max} = 3480, 2924, 1727, 1453, 1270, 1066, 698 cm^{-1}; m/z (HRMS$^+$) $[M + Na]^+$ 1023.391 ($C_{61}H_{60}O_{13}Na^+$ requires 1023.392).

^{1}H NMR of crude 121 (600 MHz, $CDCl_3$)

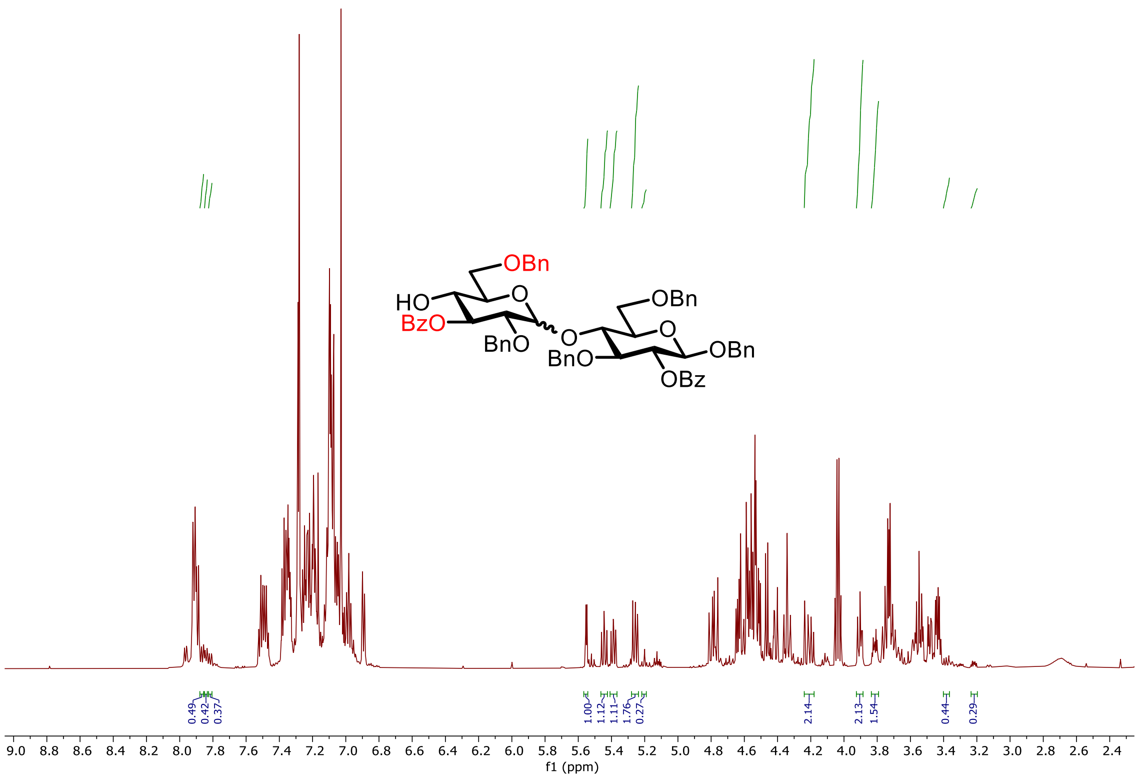

^{13}C NMR of crude 121 (151 MHz, $CDCl_3$)

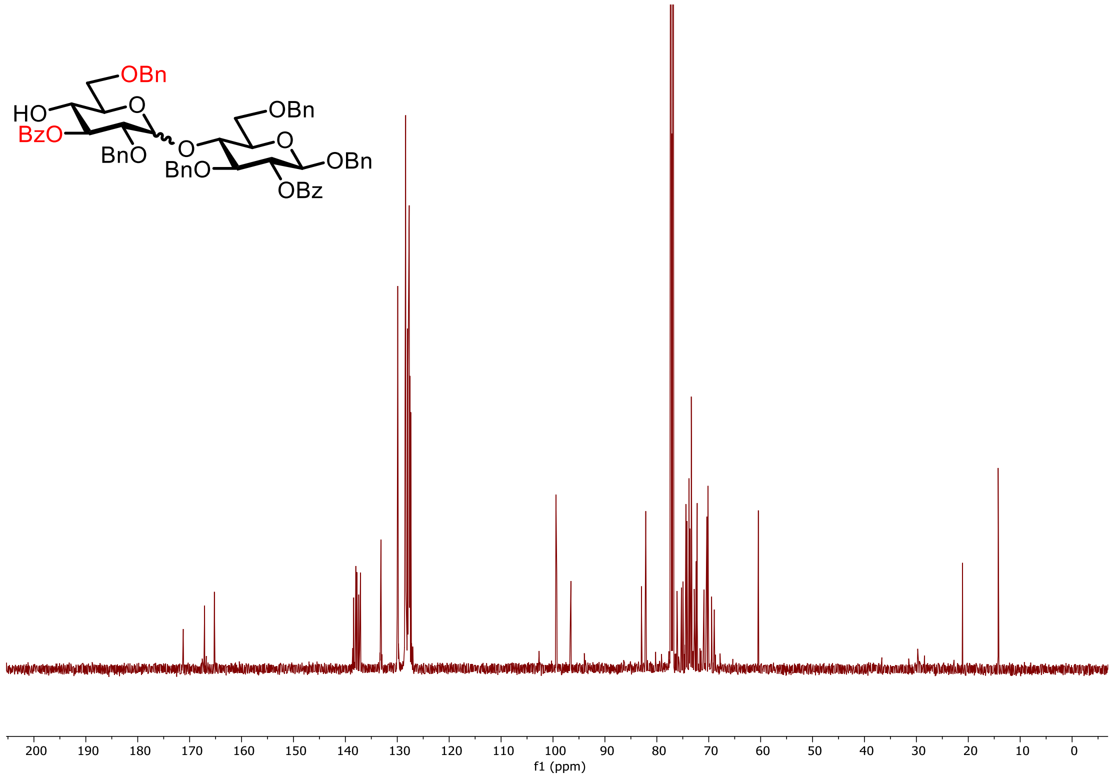

HSQC NMR of crude 121 ($CDCl_3$)

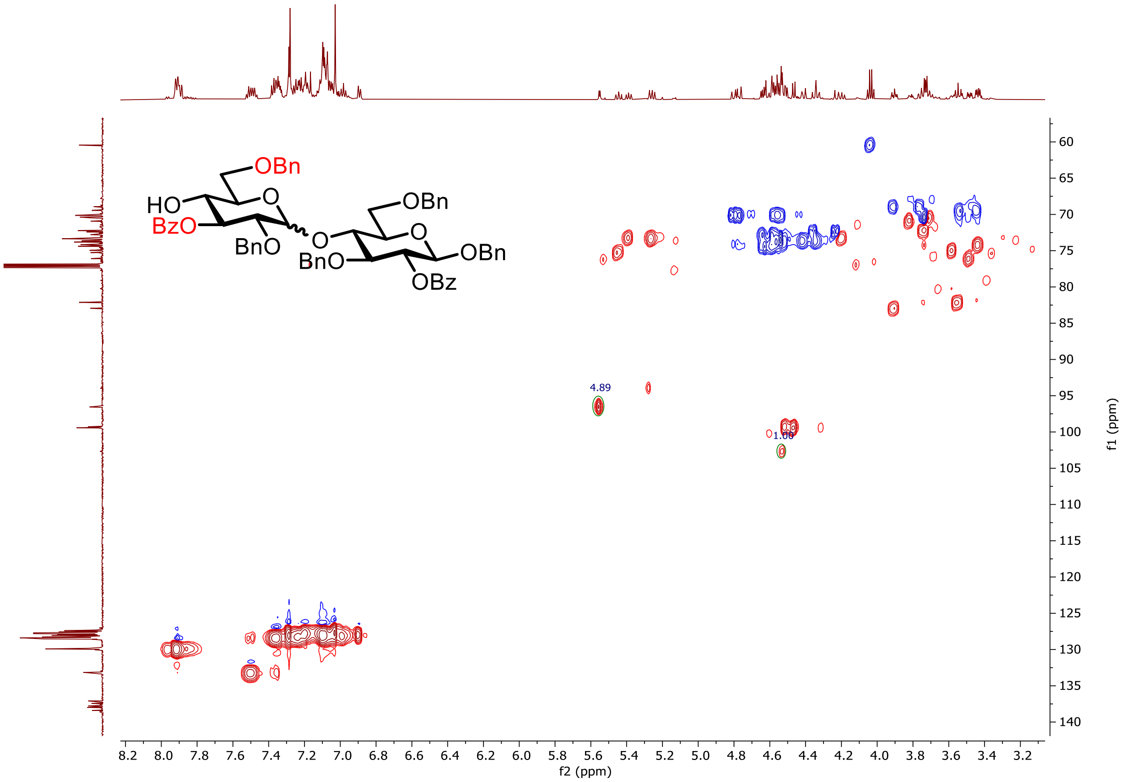

Coupled HSQC NMR of crude 121 ($CDCl_3$)

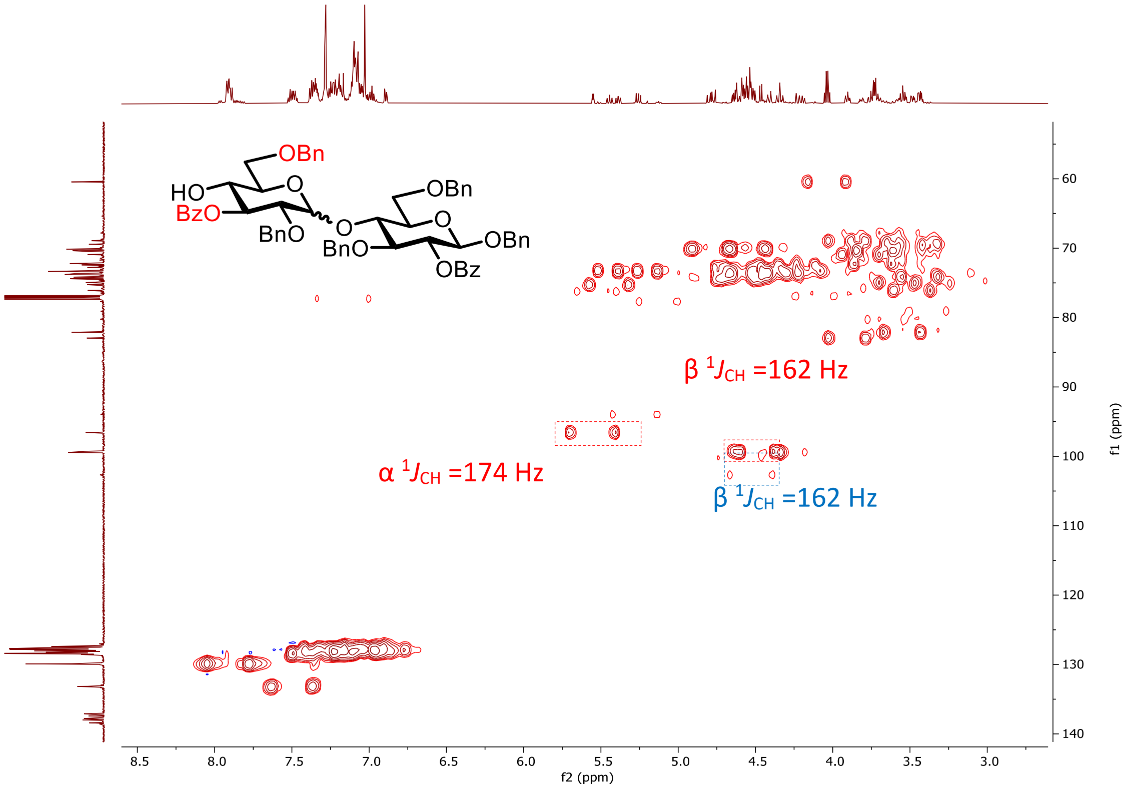

^{1}H NMR of 121a (400 MHz, $CDCl_3$)

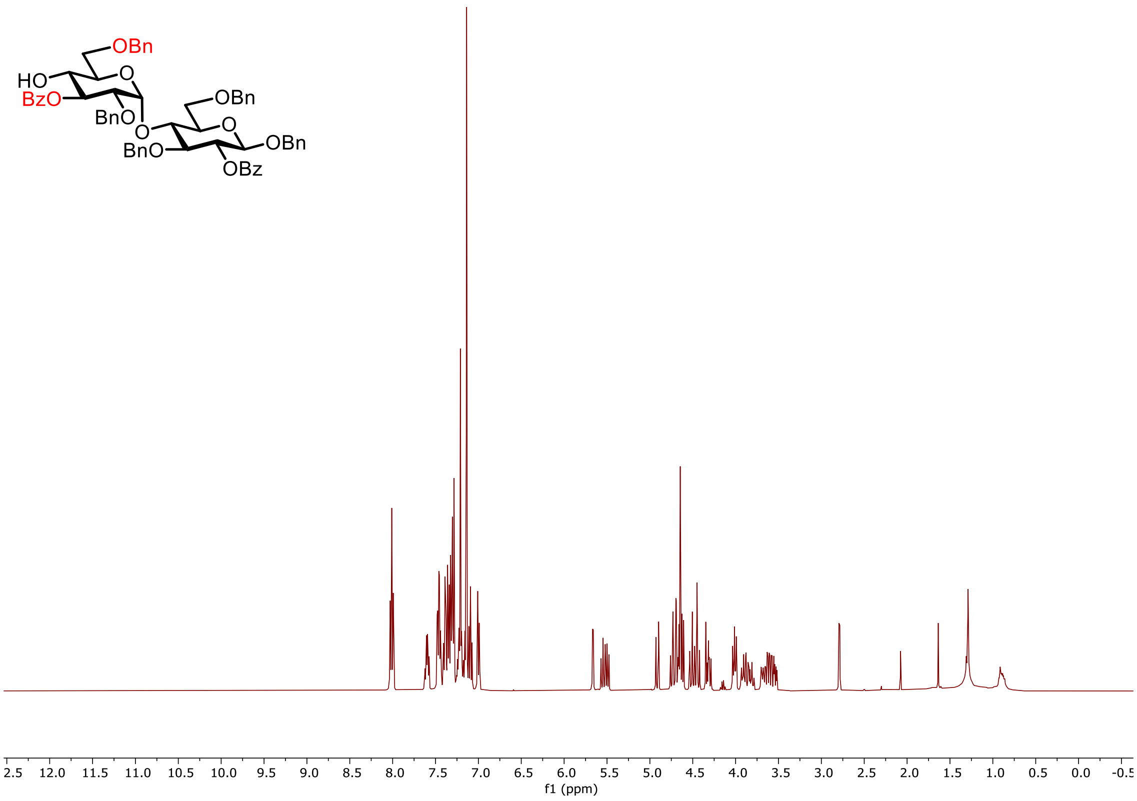

^{13}C NMR of 121a (101 MHz, $CDCl_3$)

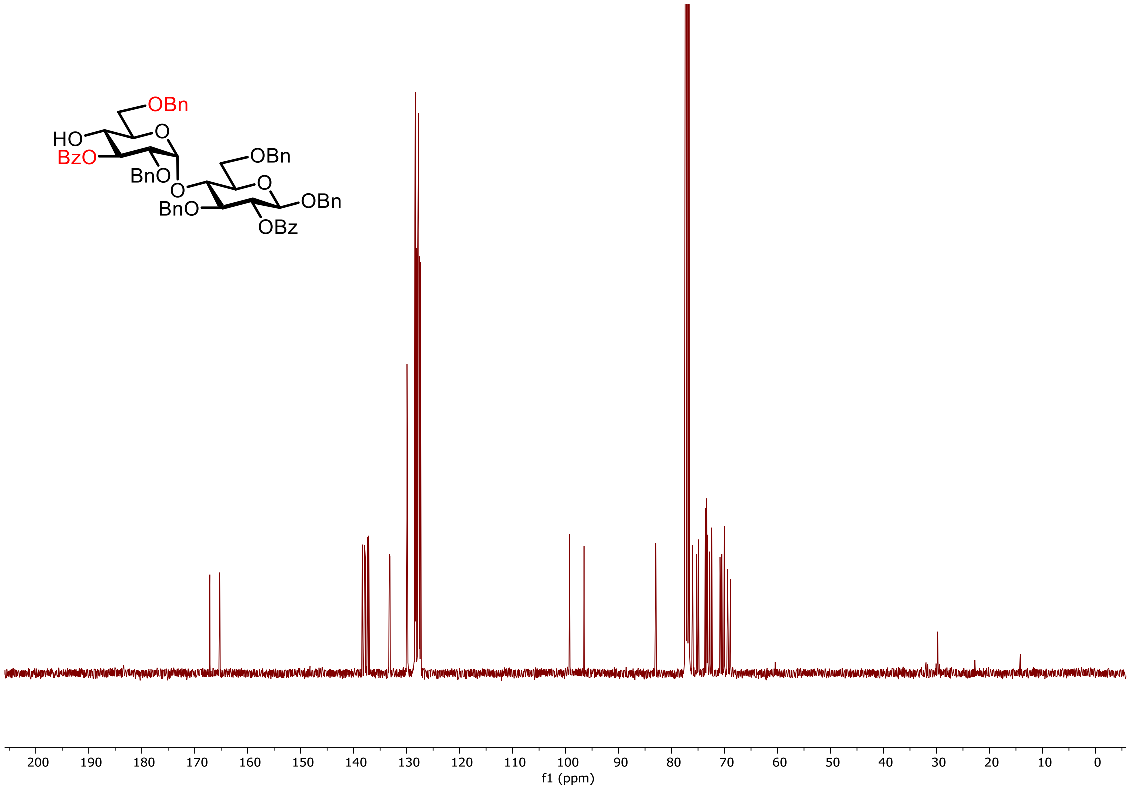

COSY NMR of 121a ($CDCl_3$)

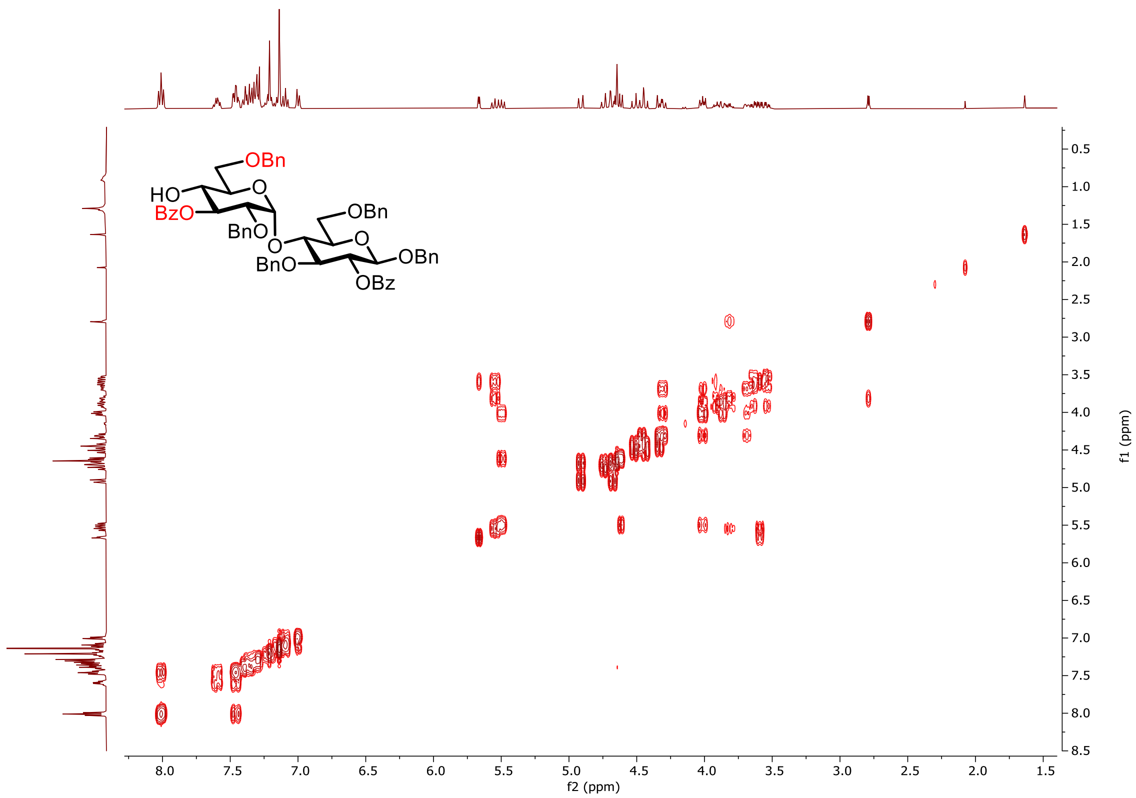

HSQC NMR of 121a ($CDCl_3$)

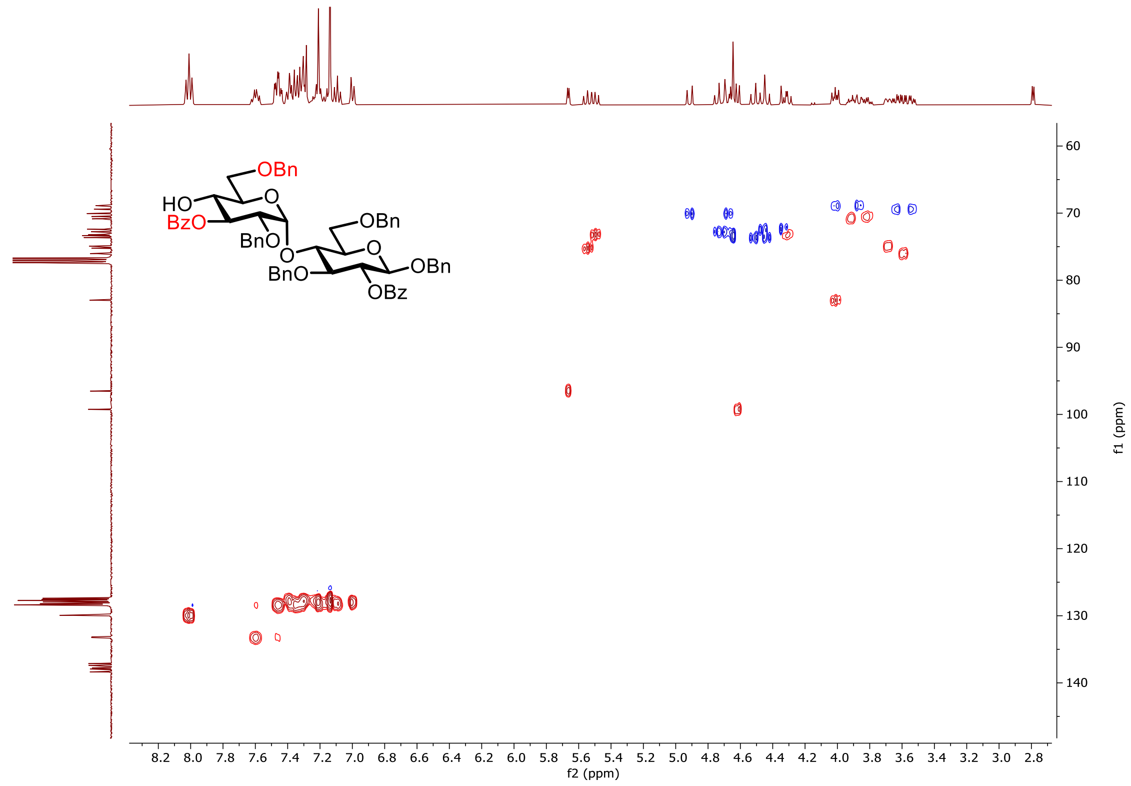

4.41 Benzyl 2,6-di-*O*-benzyl-3-*O*-pivaloyl-D-glucopyranosyl-(1→4)-2-*O*-benzoyl-3,6-di-*O*-benzyl-β-D-glucopyranoside, 122

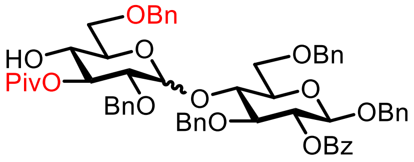

R_1=OBn; R_2=OPiv

Total yield: 65% (63.6 mg). Ratio of anomer α : β = 4.7 : 1.

Spectrum data for benzyl 2,6-di-*O*-benzyl-3-*O*-pivaloyl-α-D-glucopyranosyl-(1→4)-2-*O*-benzoyl-3,6-di-*O*-benzyl-β-D-glucopyranoside **122a**: ^{1}H NMR (400 MHz, $CDCl_3$) δ 8.02 – 7.97 (m, 2H), 7.62 – 7.57 (m, 1H), 7.45 (appt, *J* = 7.8 Hz, 2H), 7.41 – 7.26 (m, 11H), 7.26 – 7.05 (m, 14H), 5.54 (d, *J* = 3.6 Hz, 1H), 5.47 (dd, *J* = 9.0, 7.6 Hz, 1H), 5.26 (appt, *J* = 9.6 Hz, 1H), 4.90 (d, *J* = 12.6 Hz, 1H), 4.74 – 4.56 (m, 6H), 4.53 – 4.40 (m, 4H), 4.27 – 4.21 (m, 1H), 3.98 – 3.89 (m, 2H), 3.86 – 3.79 (m, 2H), 3.70 – 3.57 (m, 3H), 3.51 (ddd, *J* = 13.6, 10.2, 3.6 Hz, 2H), 2.74 (d, *J* = 4.5 Hz, 1H), 1.21 (s, 9H); ^{13}C NMR (101 MHz, $CDCl_3$) δ 179.78, 165.24, 138.34, 137.95, 137.87, 137.56, 137.13, 133.12, 129.87, 129.84, 128.42, 128.35, 128.29, 128.11, 127.75, 127.73, 127.72, 127.70, 127.66, 127.54, 127.50, 127.38, 127.26, 99.19, 96.58, 82.86, 76.28, 74.91, 74.84, 73.63, 73.28, 73.19, 73.07, 72.68, 72.62, 71.11, 70.65, 70.01, 69.34, 68.86, 38.99, 27.18; $[\alpha]_D^{25}$ 37.16 (c = 1, $CHCl_3$); IR (neat) ν_{max} = 3339, 2972, 1731, 1455, 1379, 1160, 952, 698 cm^{-1}; m/z (HRMS$^+$) [M + Na]$^+$ 1003.426 ($C_{59}H_{64}O_{13}Na^+$ requires 1003.423).

^{1}H NMR of crude 122 (600 MHz, $CDCl_3$)

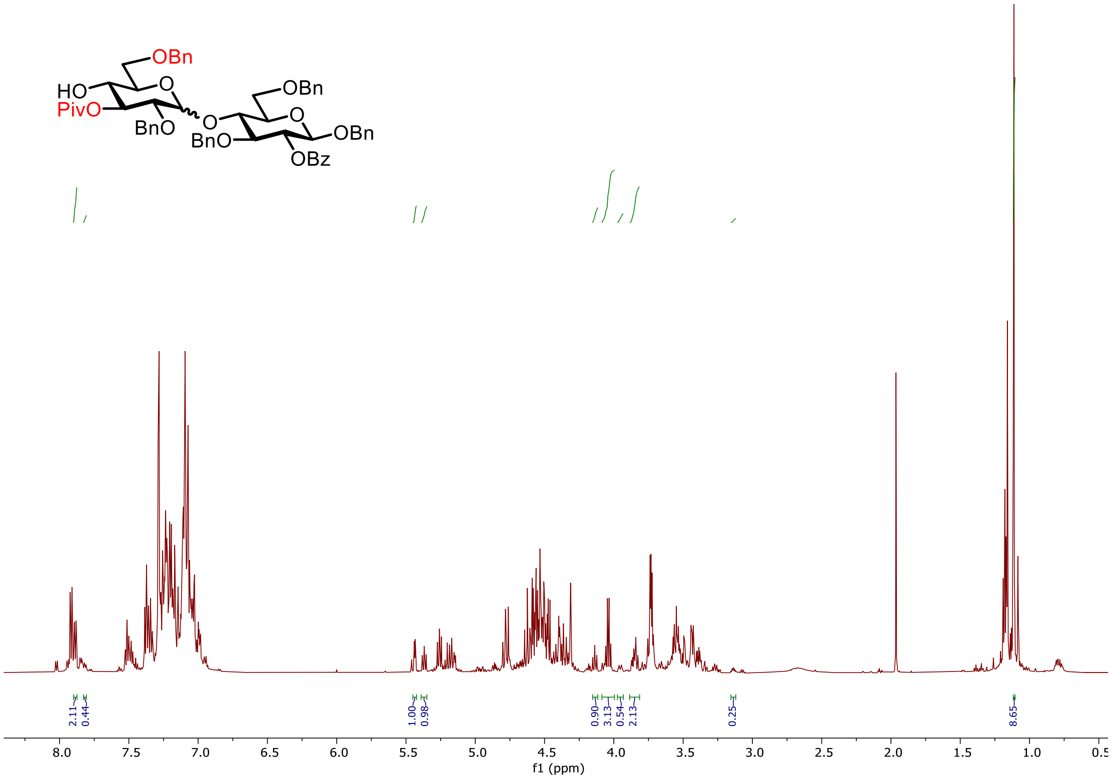

^{13}C NMR of crude 122 (151 MHz, $CDCl_3$)

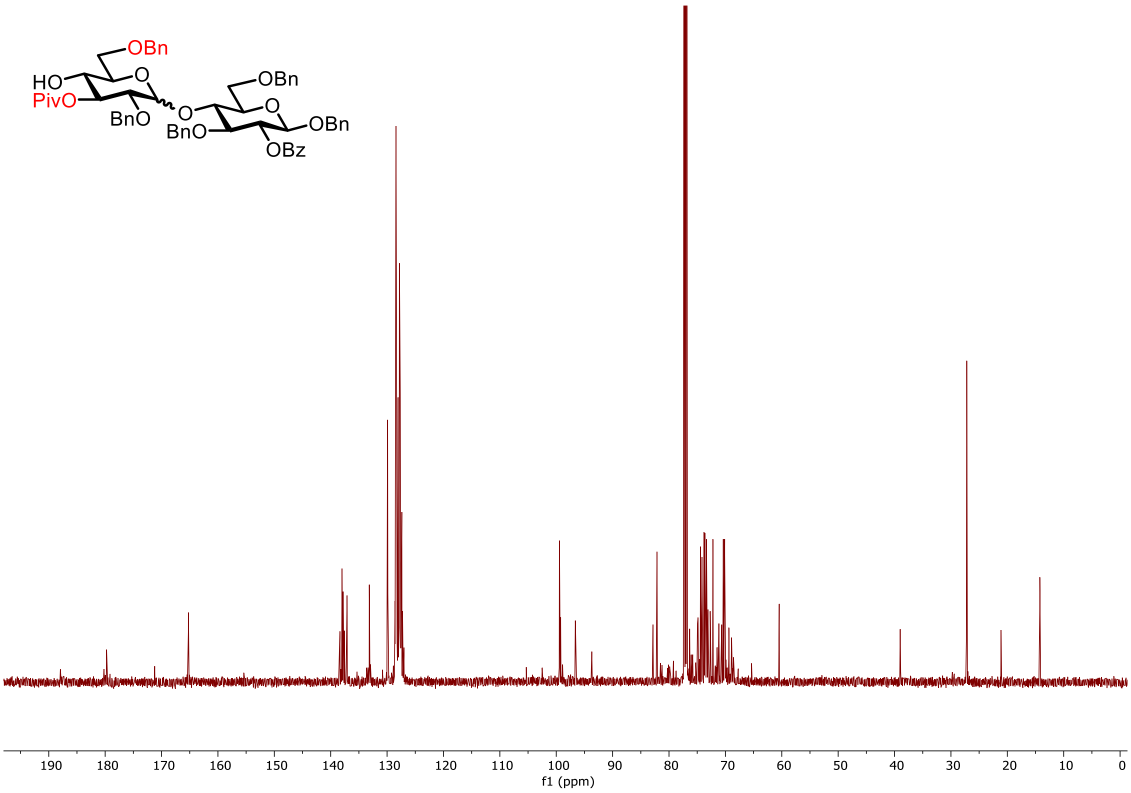

HSQC NMR of crude 122 ($CDCl_3$)

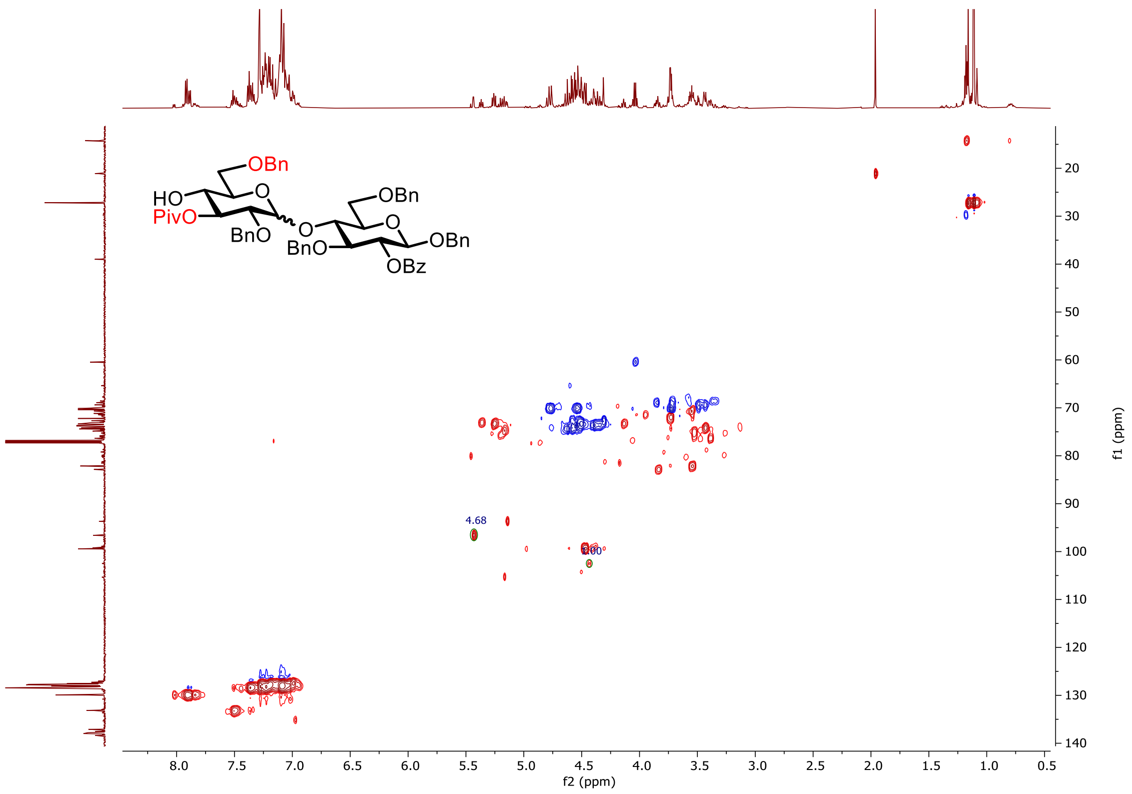

Coupled HSQC NMR of crude 122 ($CDCl_3$)

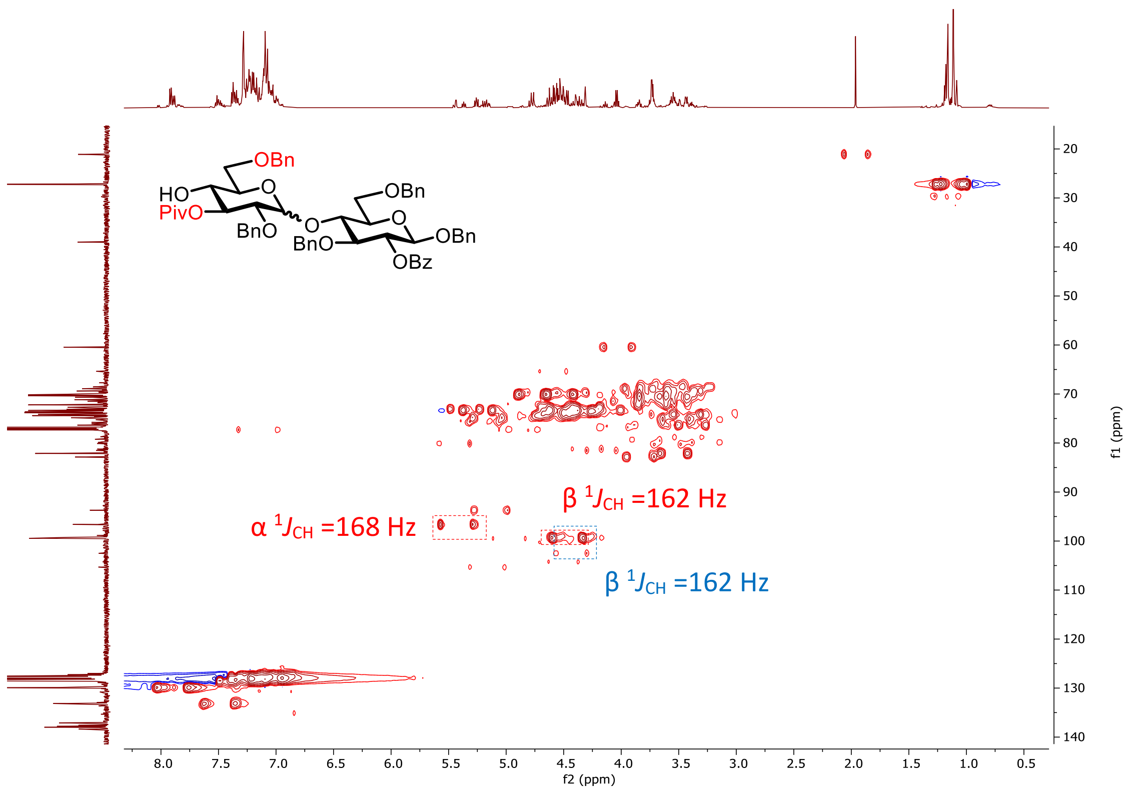

^{1}H NMR of 122a (400 MHz, $CDCl_3$)

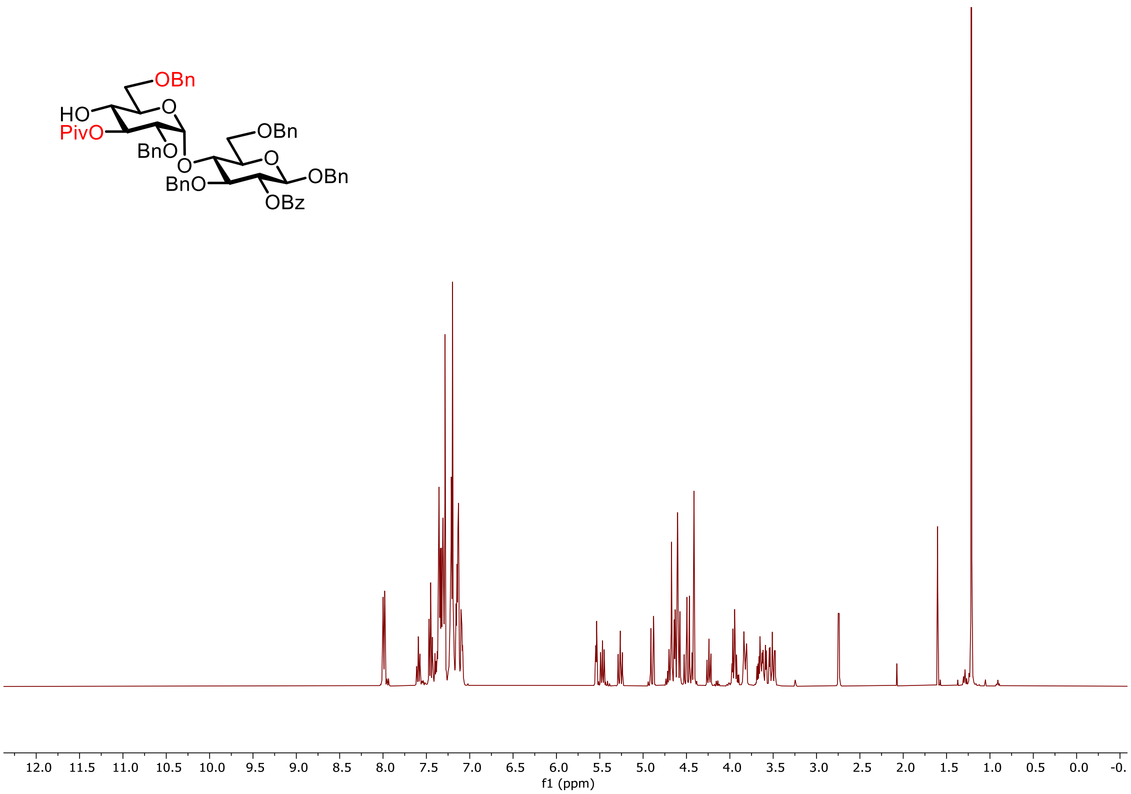

^{13}C NMR of 122a (101 MHz, $CDCl_3$)

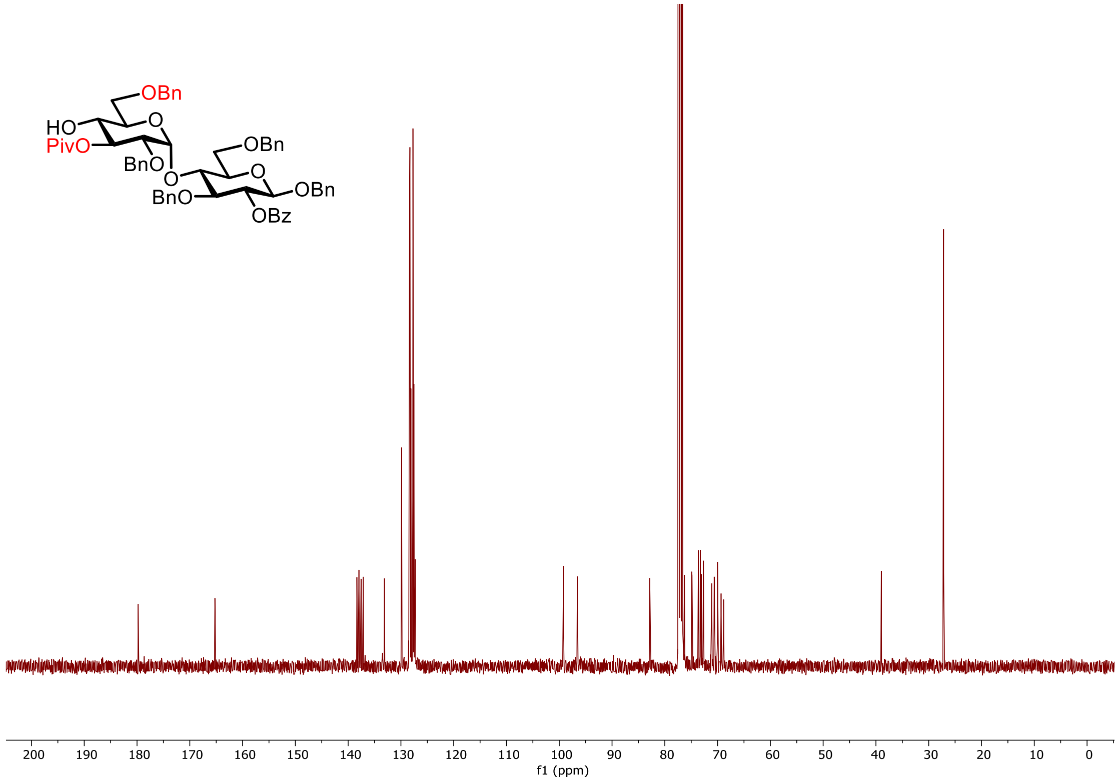

COSY NMR of 122a ($CDCl_3$)

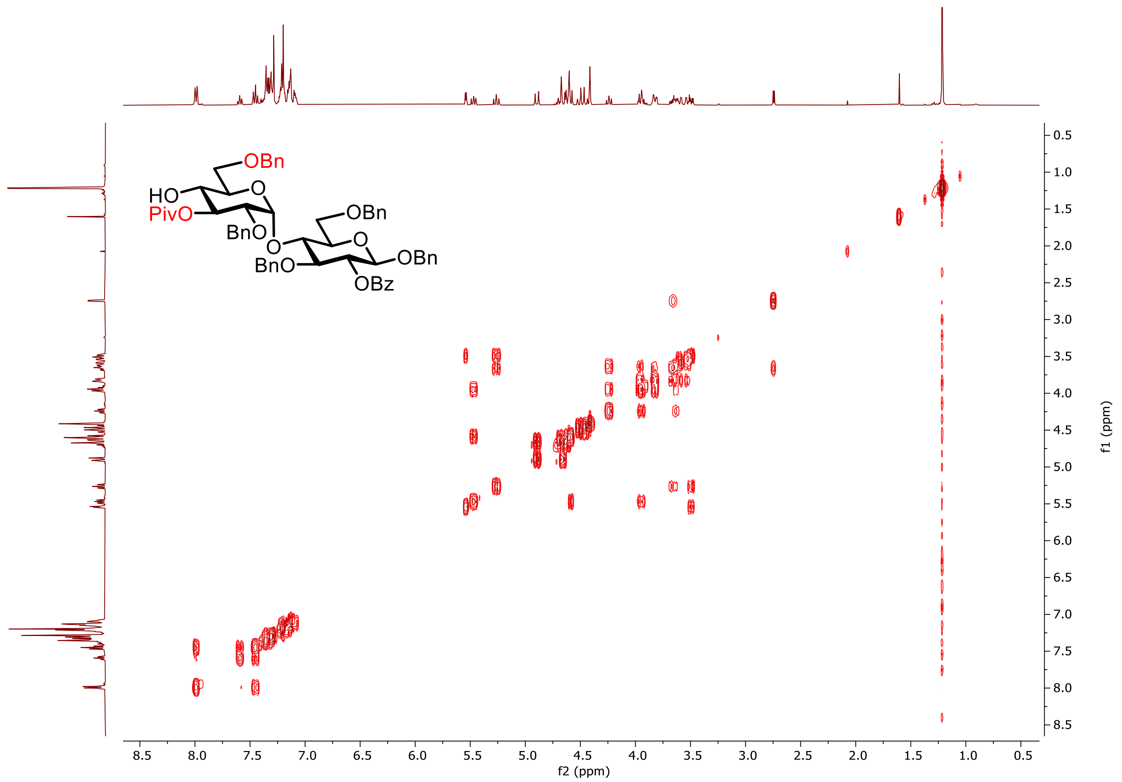

HSQC NMR of 122a ($CDCl_3$)

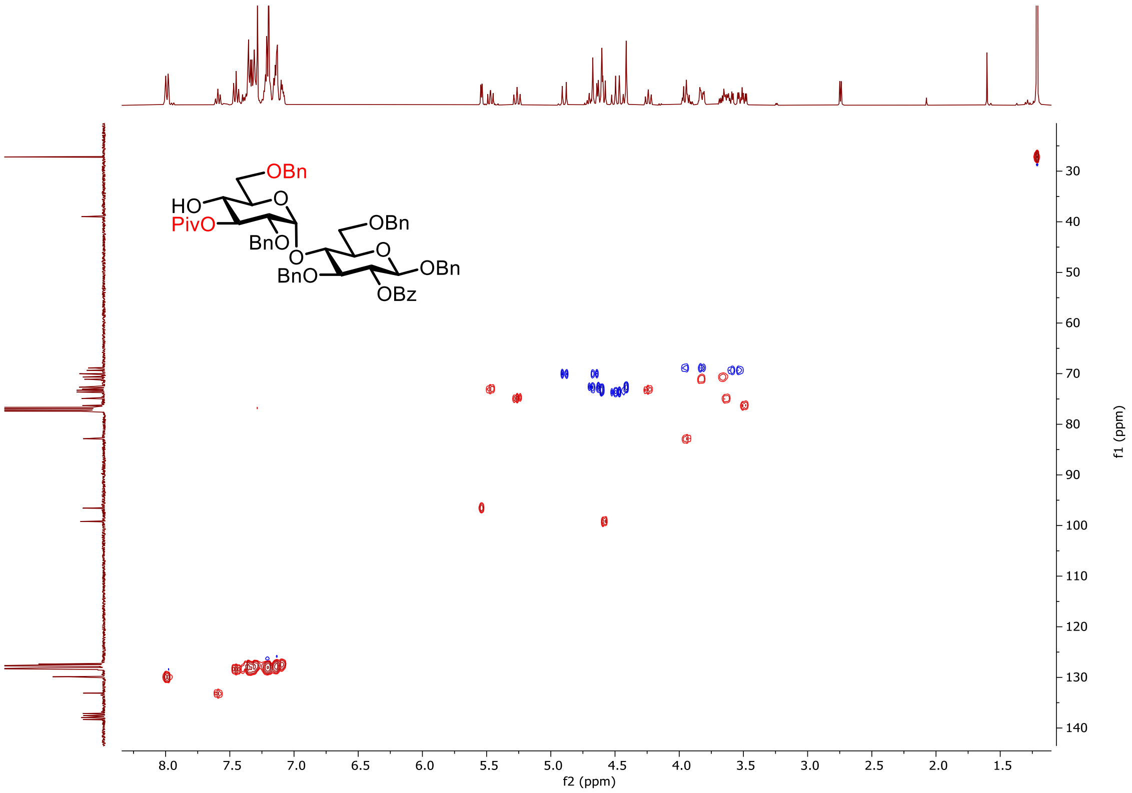

5. Temperature and Solvent Control Study

5.1 Temperature control study

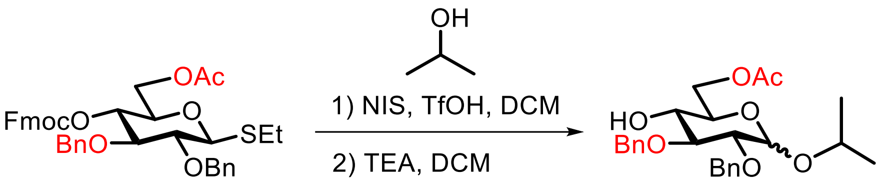

13 **75**

^{1}H NMR study of crude 75 at different temperatures (400 MHz, $CDCl_3$):

1. T= -45 °C, 60 min: Total yield: 17% (80% starting material remaining). Ratio of anomer α : β = 1.3 : 1.

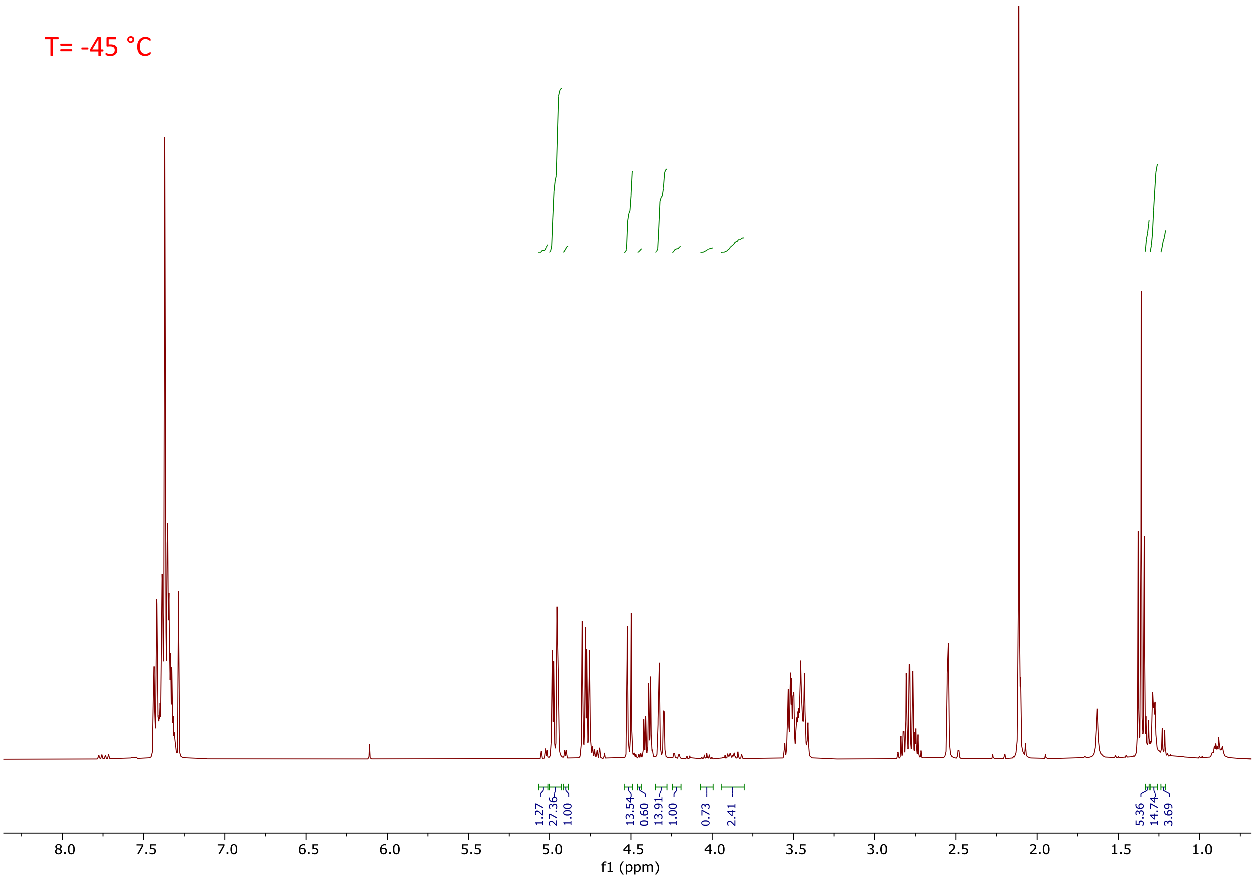

2. T= -20 °C, 60 min: Total yield: 84%. Ratio of anomer α : β=1.8 : 1.

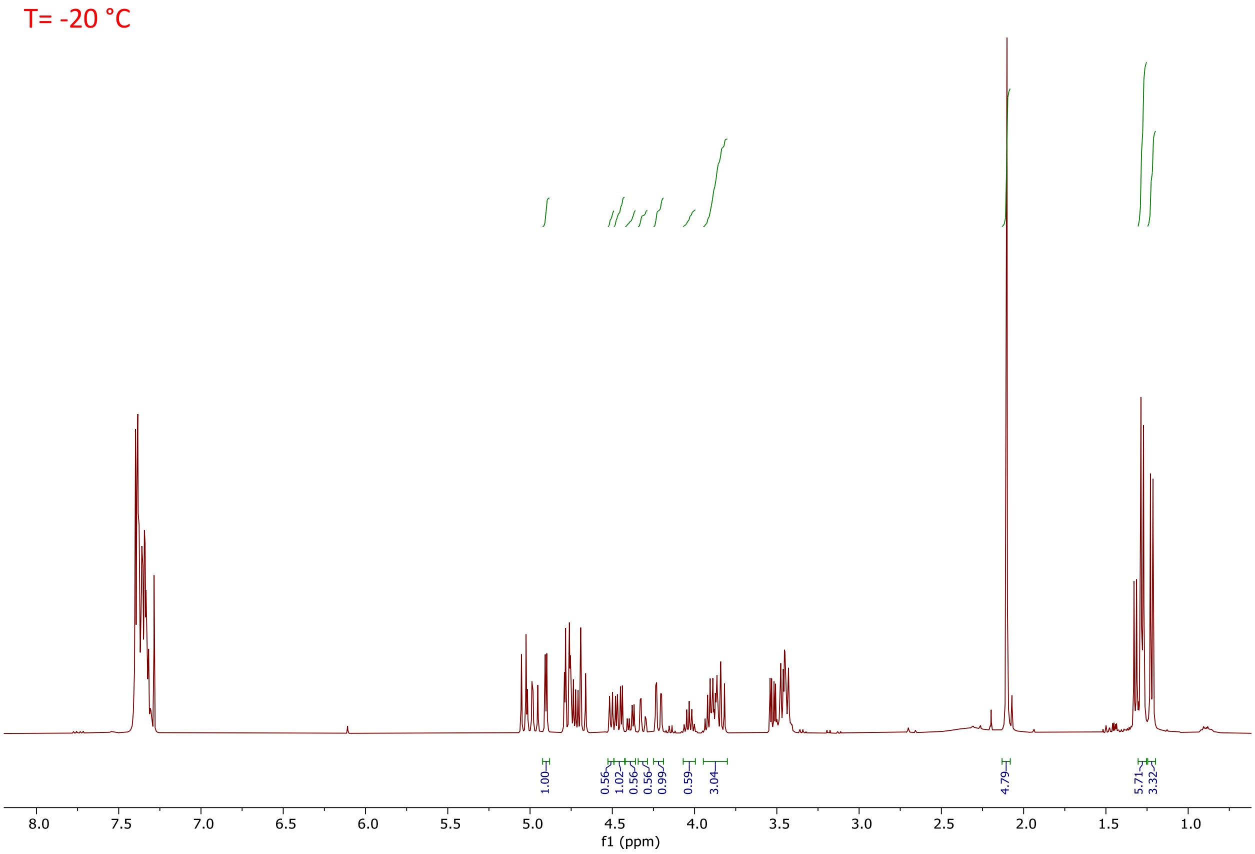

3. T= -15 °C for 5 min, then T= 0 °C for 60 min: Total yield: 95%. Ratio of anomer α : β = 2.3 : 1.

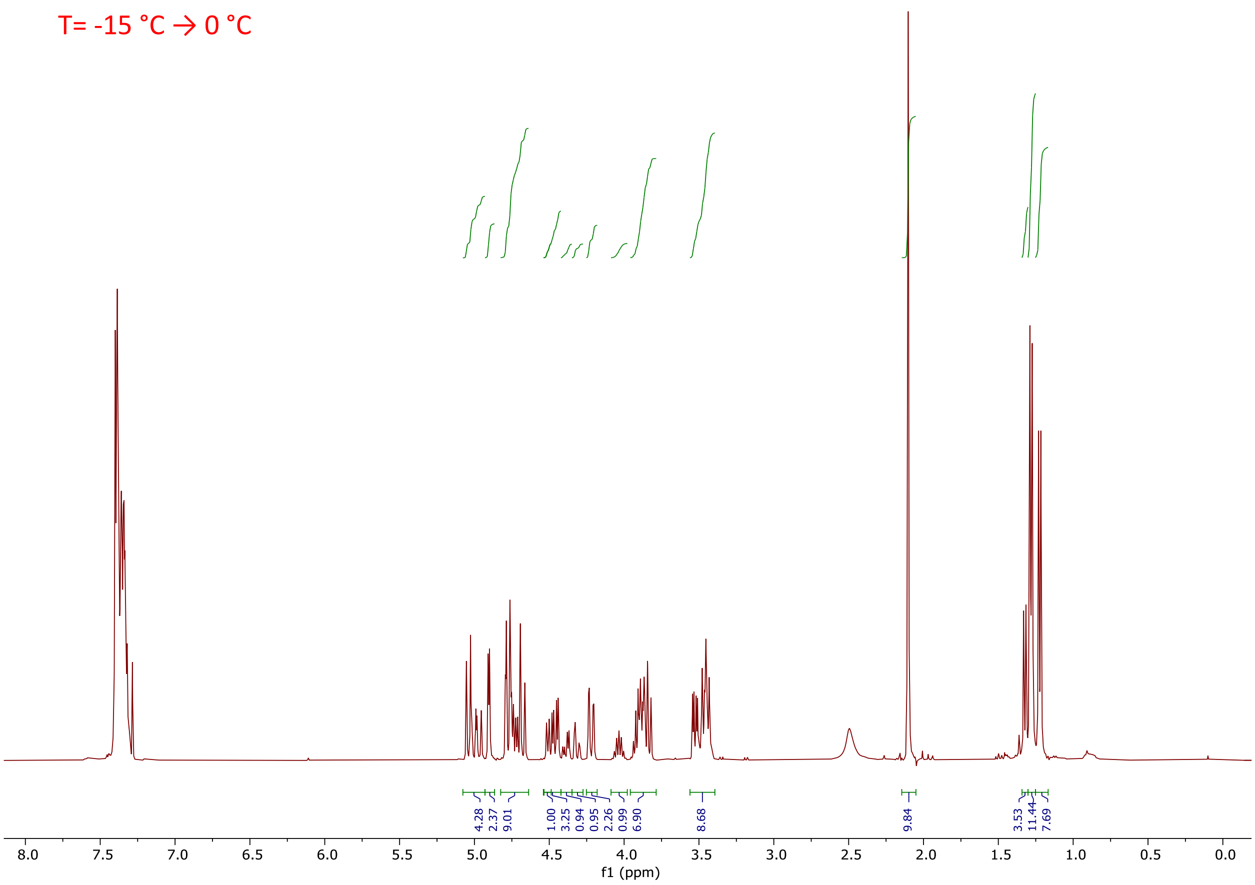

4. T= 0 °C, 60 min: Total yield: 90%. Ratio of anomer α : β = 2.9 : 1.

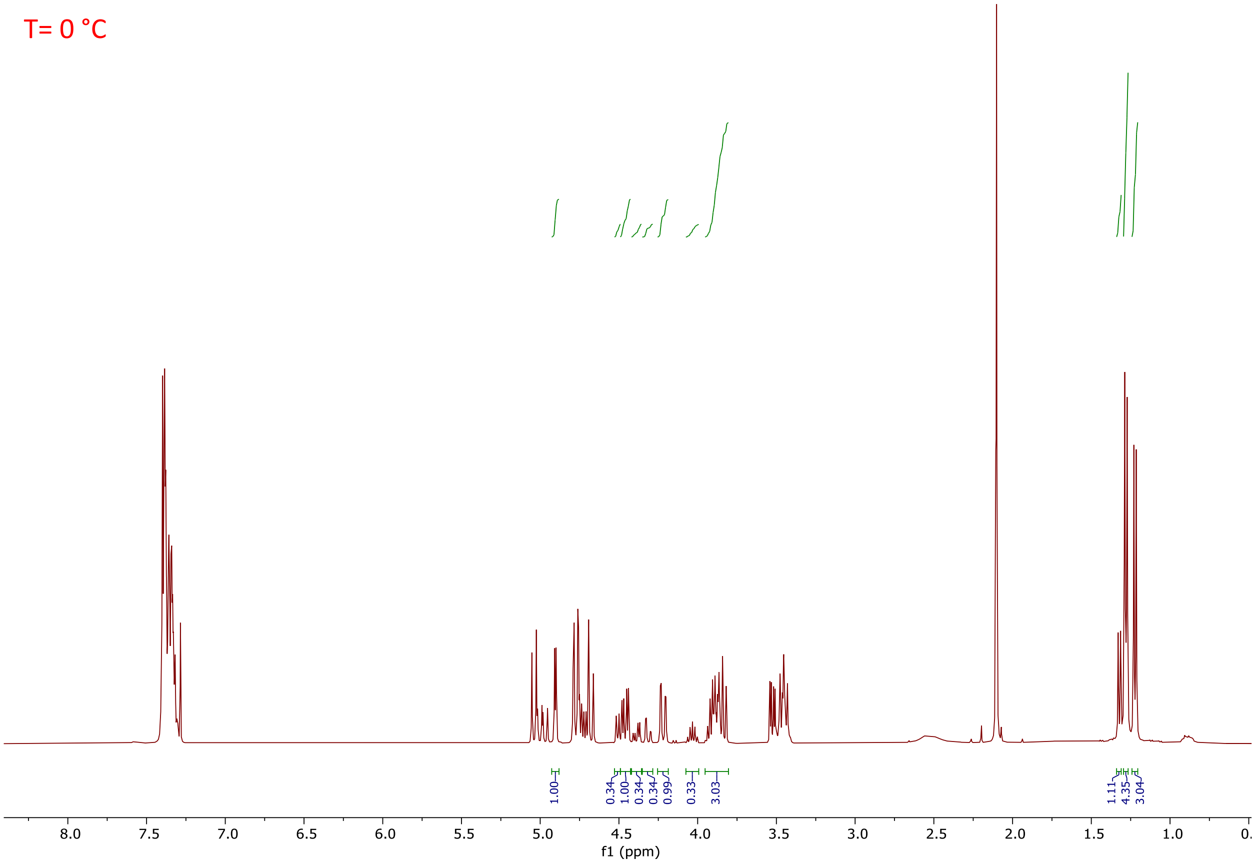

5.2 Glycosylation study in different ethers

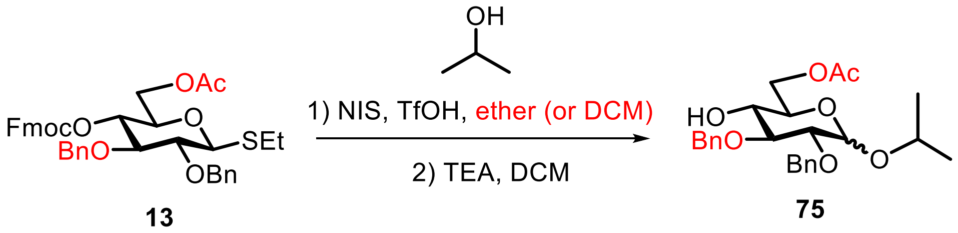

¹H NMR study of crude 75 after glycosylation in different solvents (400 MHz, $CDCl_3$):

1. DCM: Total yield: 95%. Ratio of anomer α : β = 2.3 : 1.

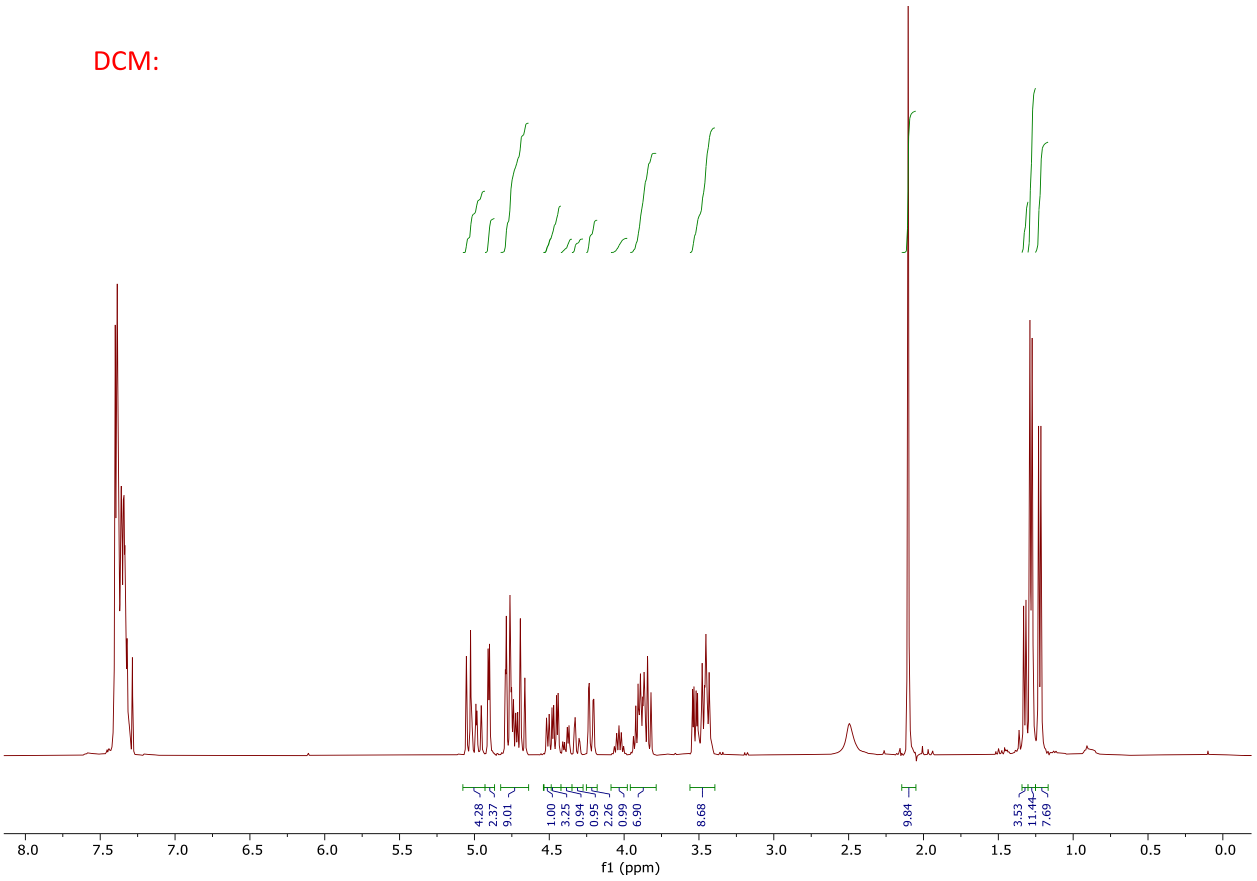

2. Diethyl ether (Et_2O): Total yield: 87%. Ratio of anomer α : β = 4.1 : 1. Poor solubility of BB+NIS.

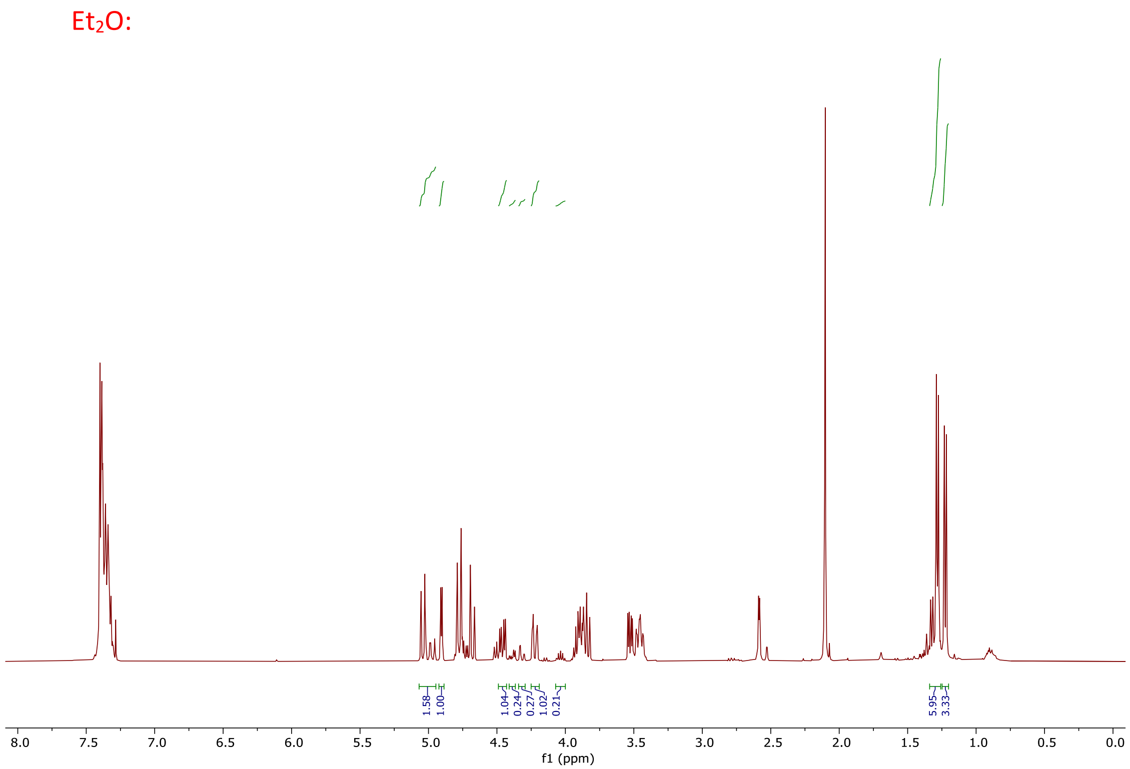

3. Tetrahydrofuran (THF): Total yield: 83%. Ratio of anomer α : β = 2.2 : 1.

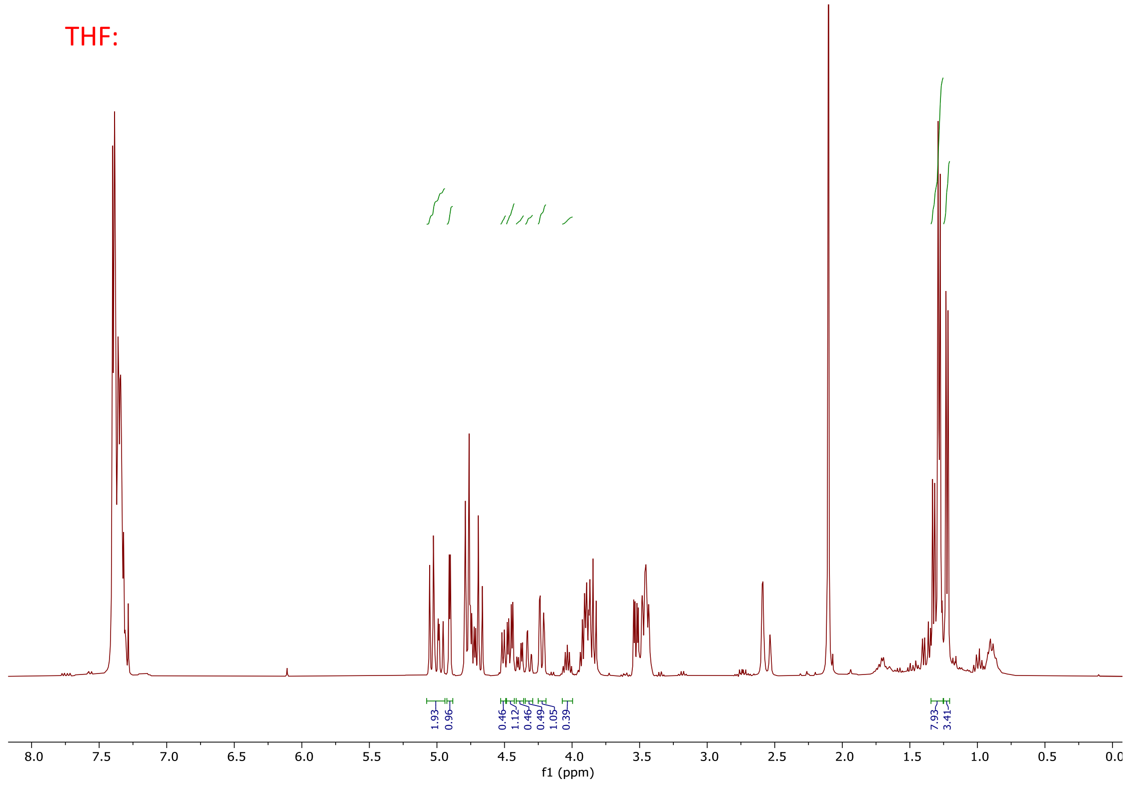

4. Dioxane: Total yield: 92%. Ratio of anomer α : β = 4.3 : 1. Solvent frozen during reaction.

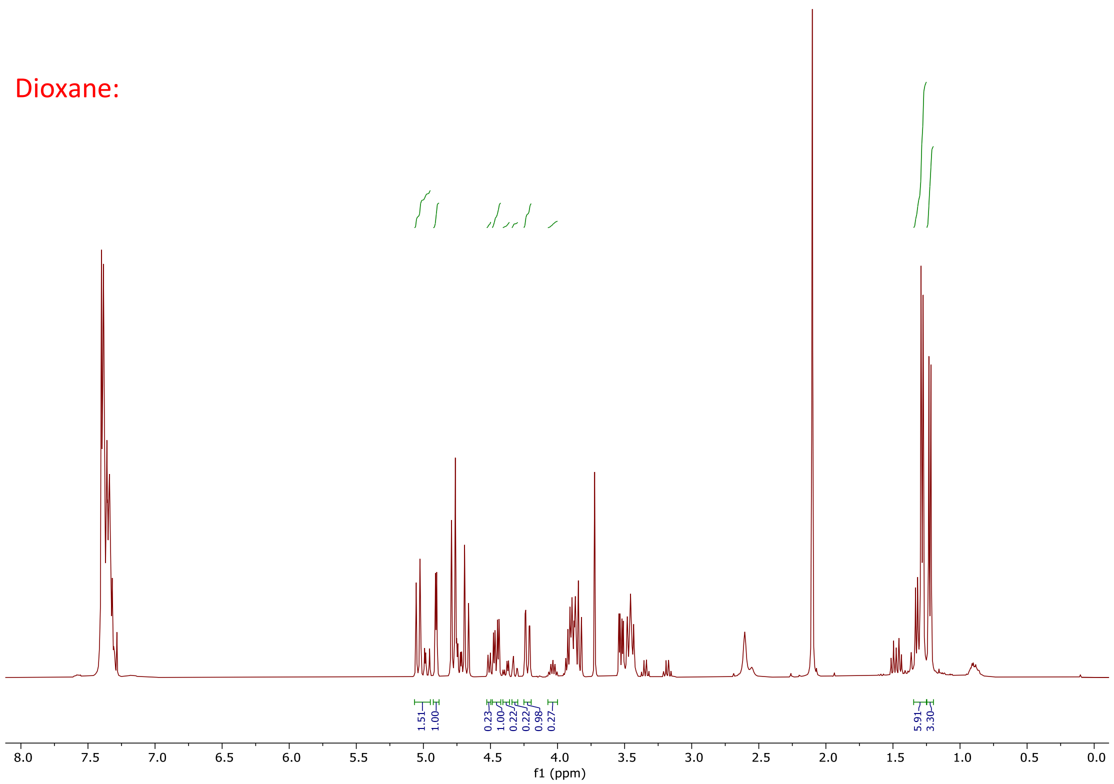

5. Methyl *tert*-butyl ether (MTBE): Total yield: 70%. Ratio of anomer α : β = 5.0 : 1. Poor solubility of BB+NIS.

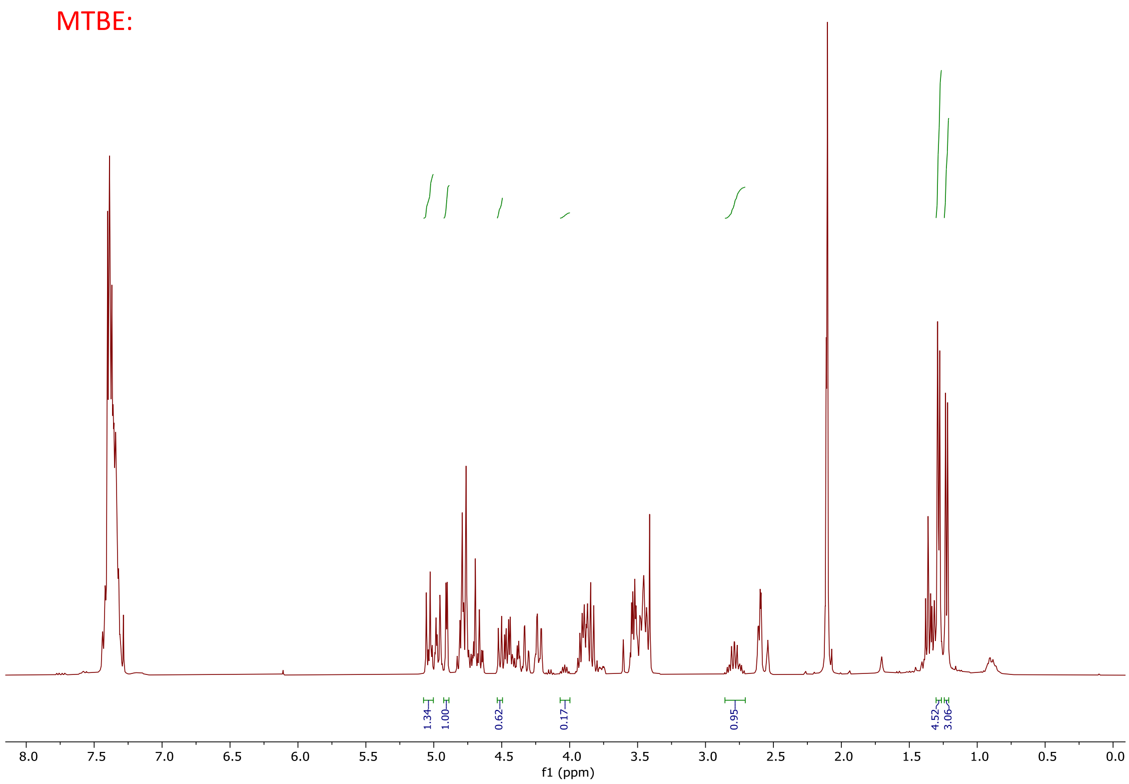

5.3 Glycosylation study in anhydrous DCM-dioxane (5:1)

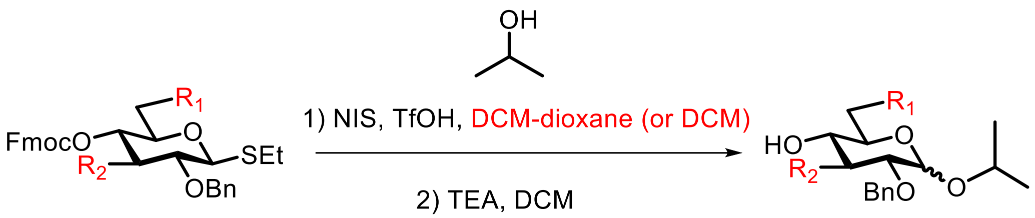

12,13,20,111,28 **74,75,82,118,90**

^{1}H NMR study of crude glycosylation products (400 MHz, $CDCl_3$):

1. BB**12**:

DCM: Total yield: 93%. Ratio of anomer α : β = 1 : 2.9.

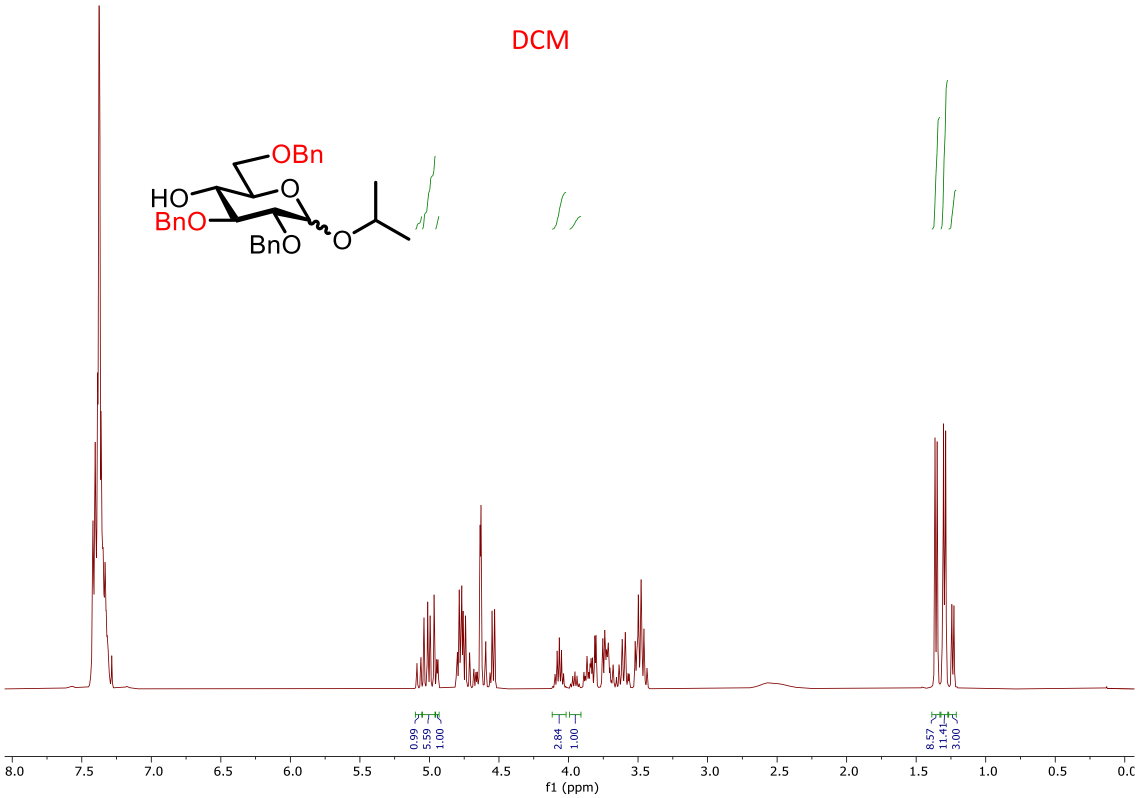

DCM-dioxane: Total yield: 92%. Ratio of anomer α : β = 1 : 1.4.

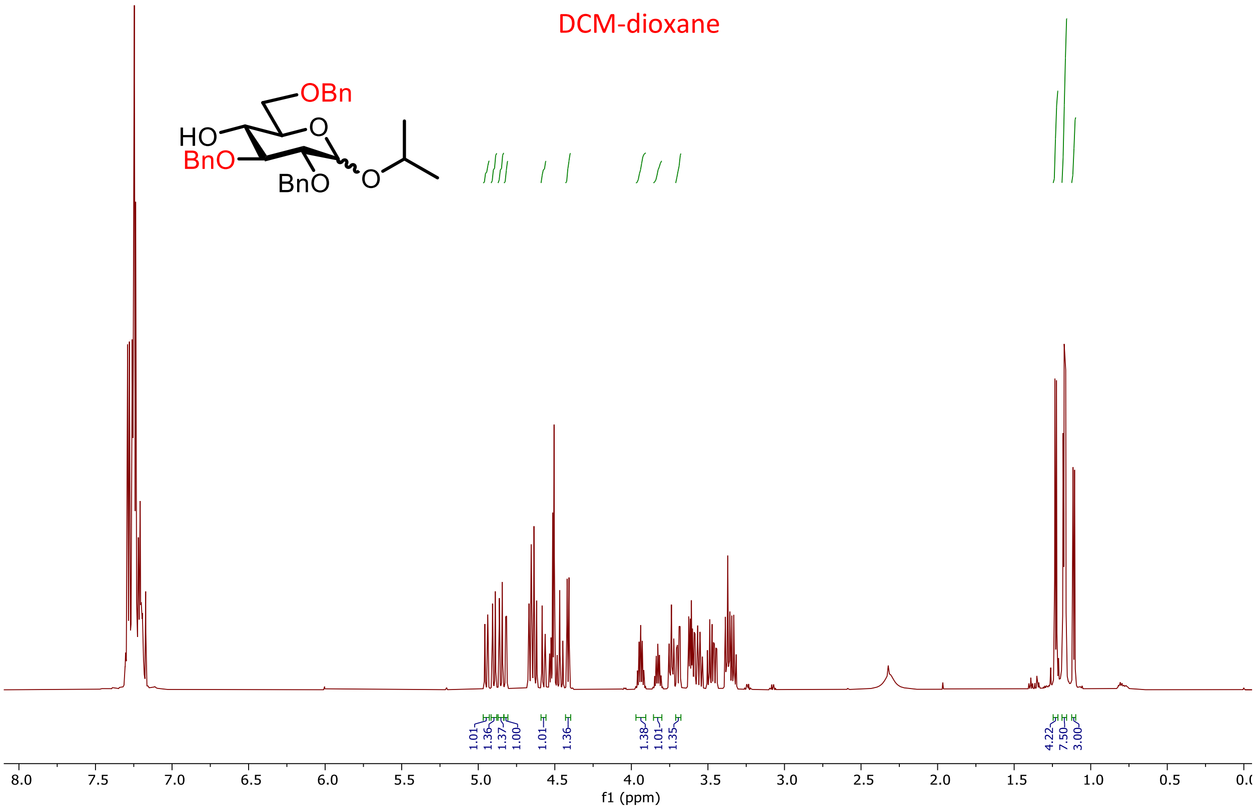

2. BB**13**:

DCM: Total yield: 95%. Ratio of anomer α : β = 2.3 : 1.

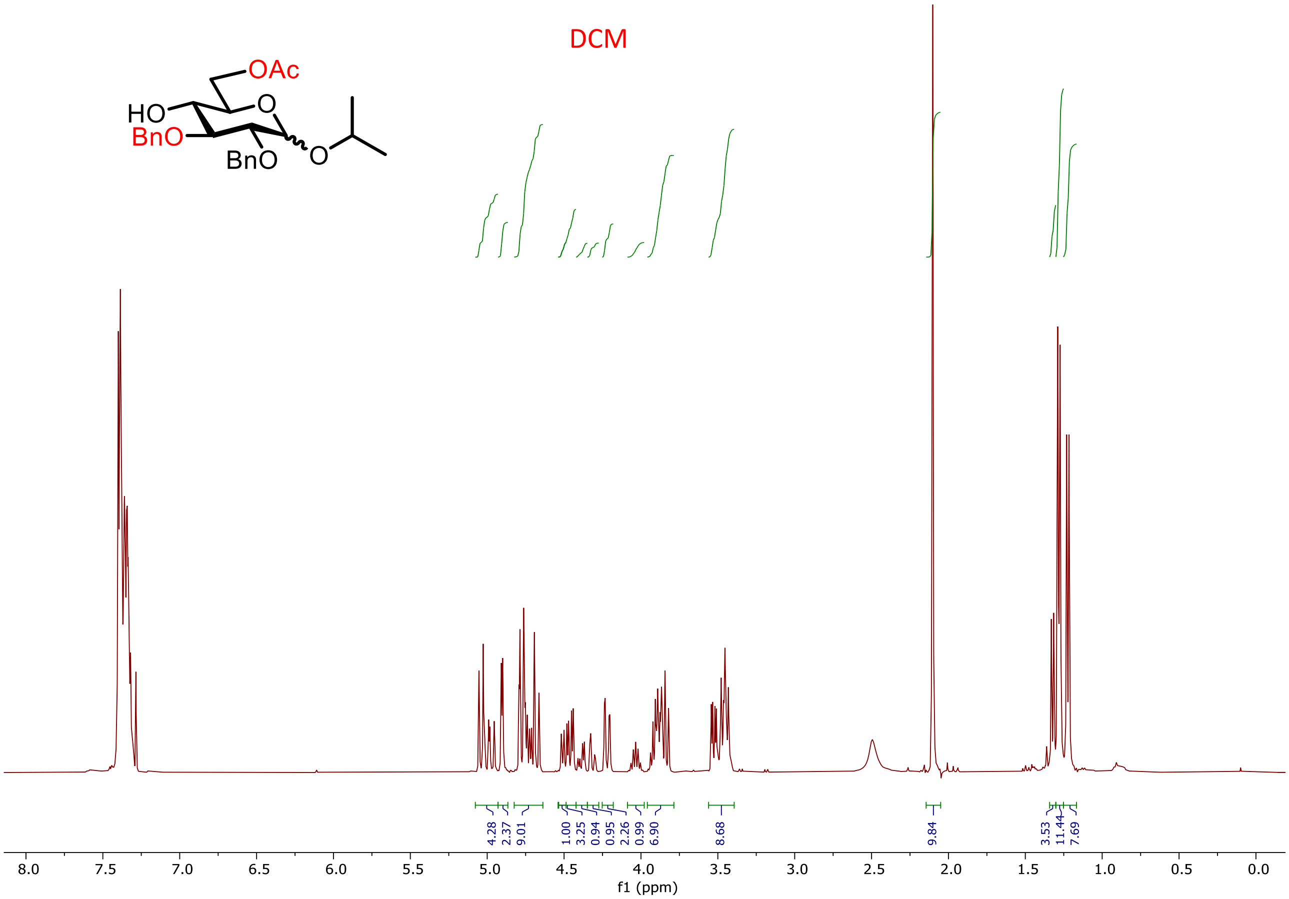

DCM-dioxane: Total yield: 90%. Ratio of anomer α : β = 2.8 : 1.

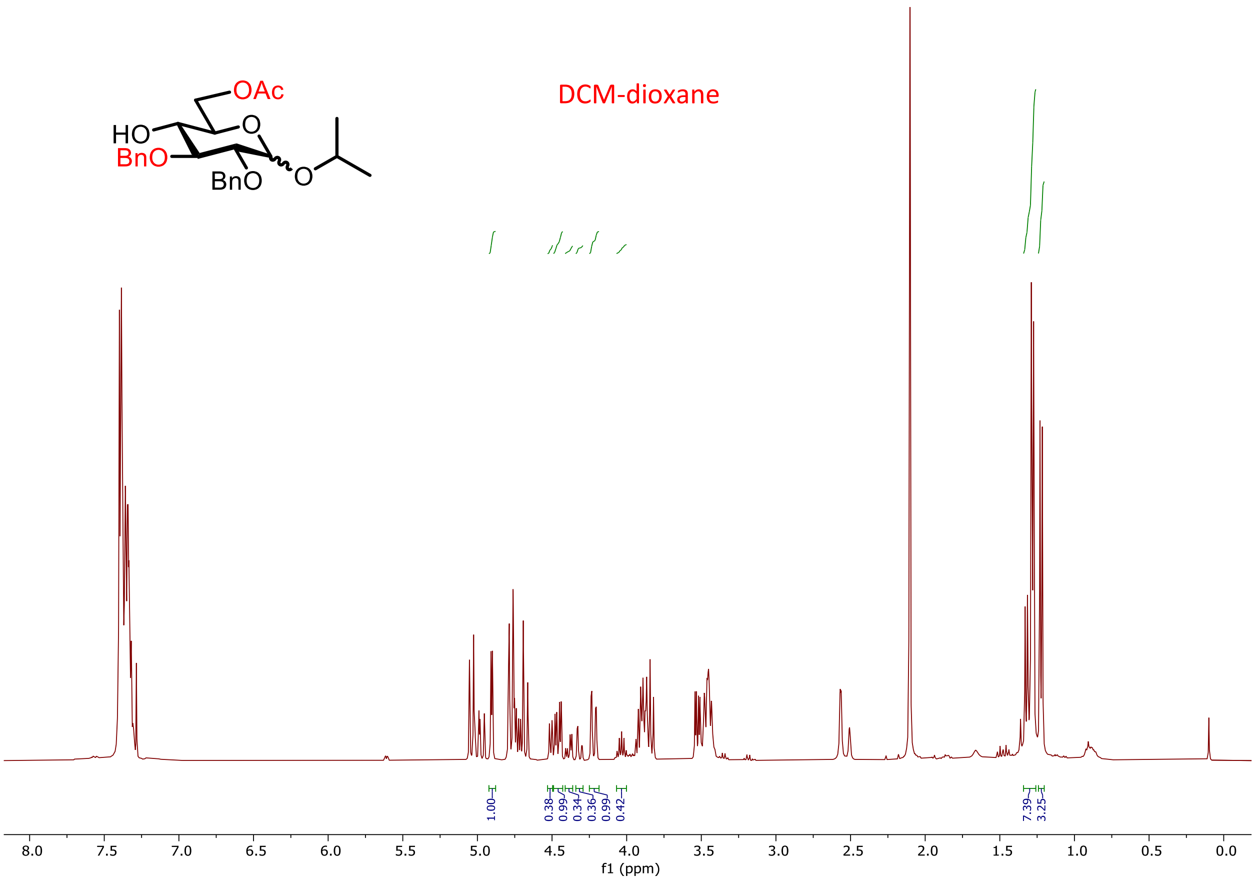

3. BB**20**:

DCM: Total yield: 93%. Ratio of anomer α : β = 1.7 : 1.

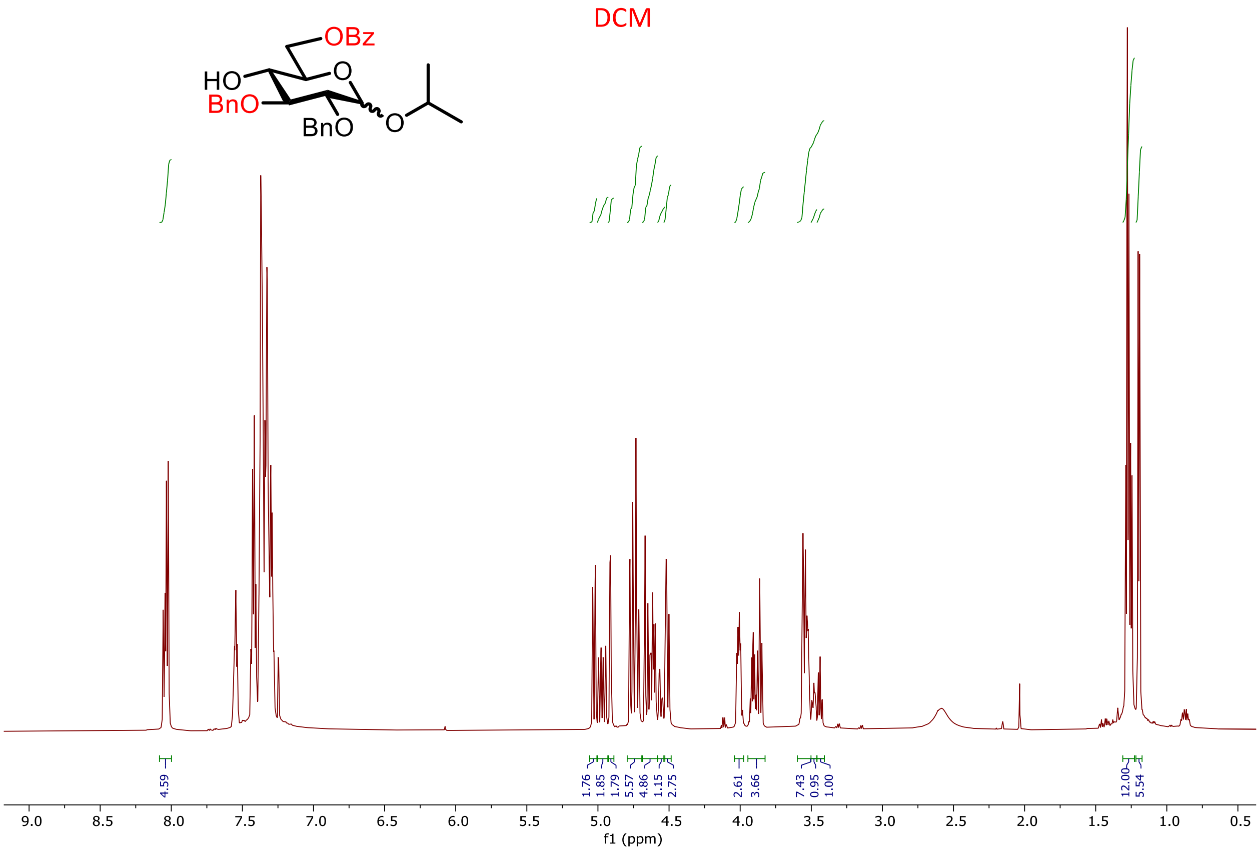

DCM-dioxane: Total yield: 94%. Ratio of anomer α : β = 2.2 : 1.

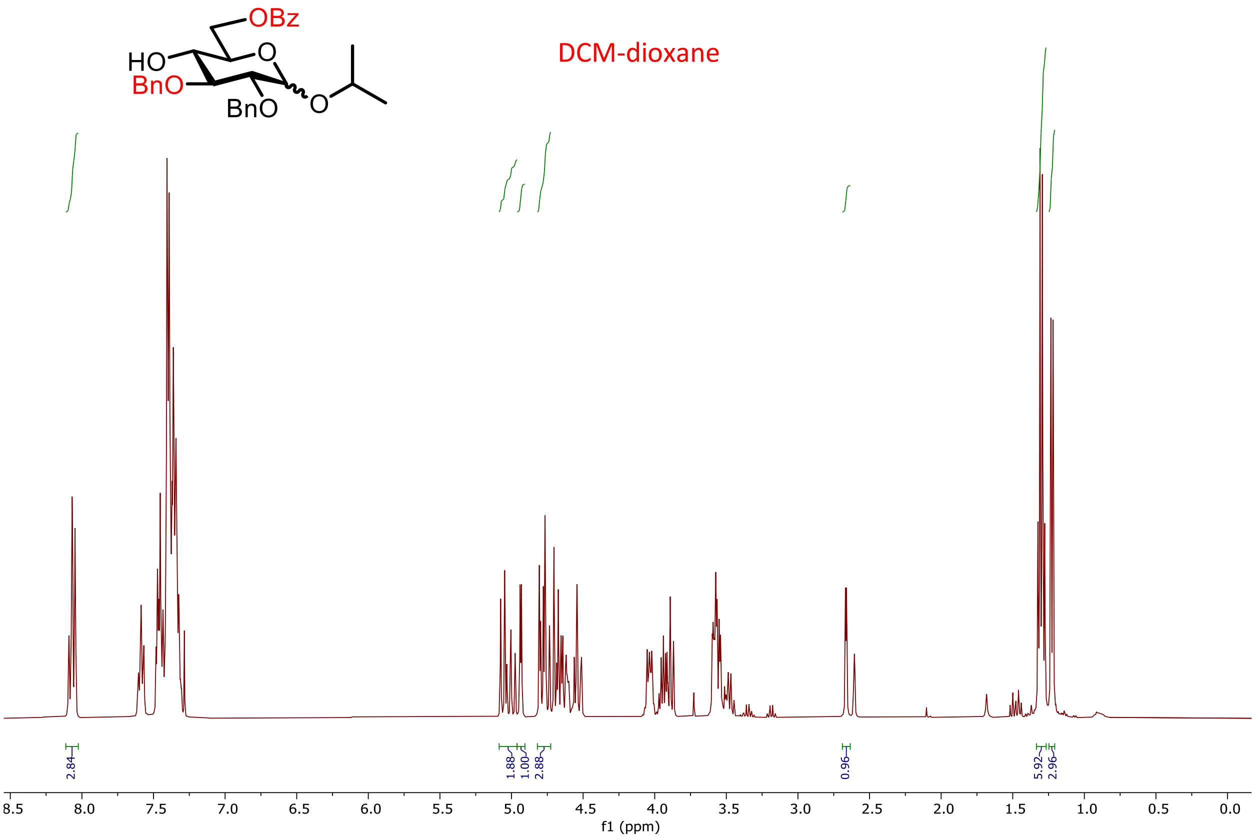

4. BB**111**:

DCM: Total yield: 95%. Ratio of anomer α : β = 1 : 1.1.

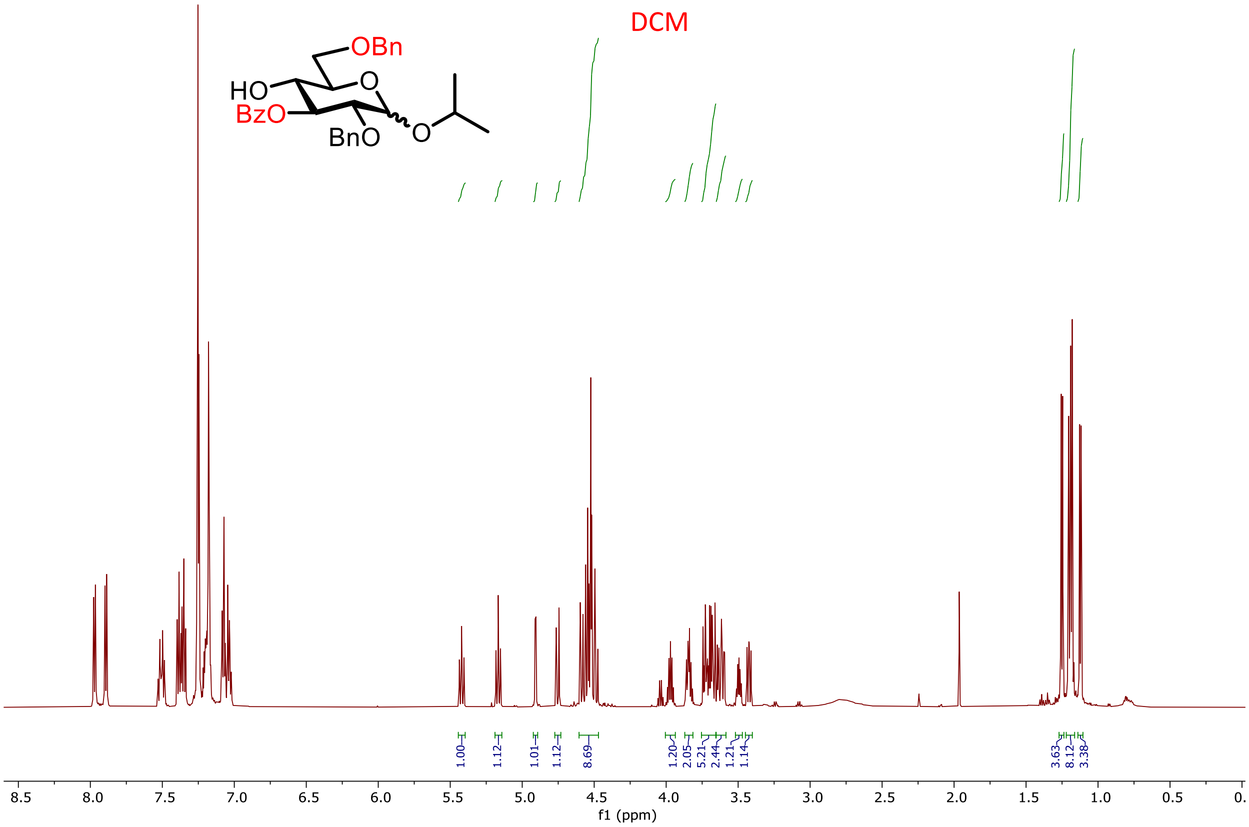

DCM-dioxane: Total yield: 93%. Ratio of anomer α : β = 2.2 : 1.

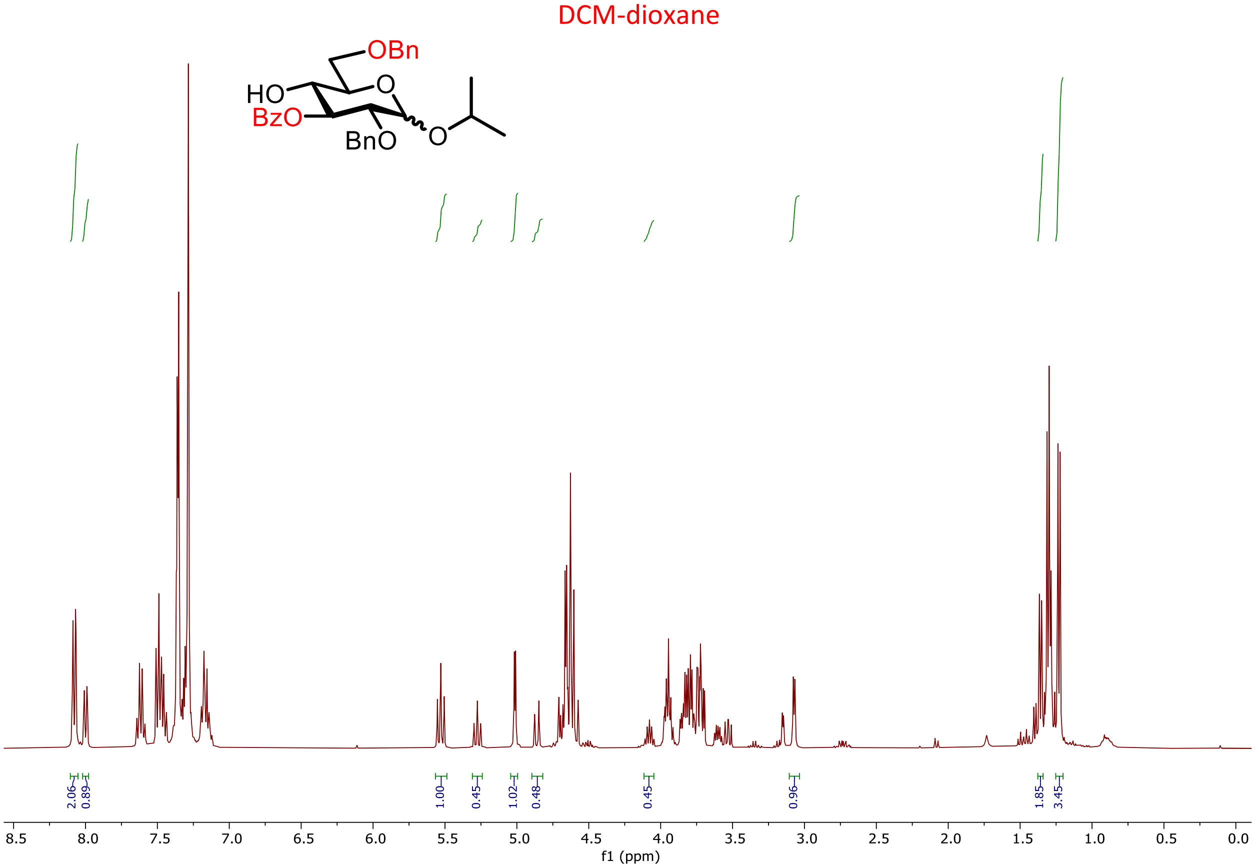

5. BB**28**:

DCM: Total yield: 87%. Ratio of anomer α : β = 4.8 : 1.

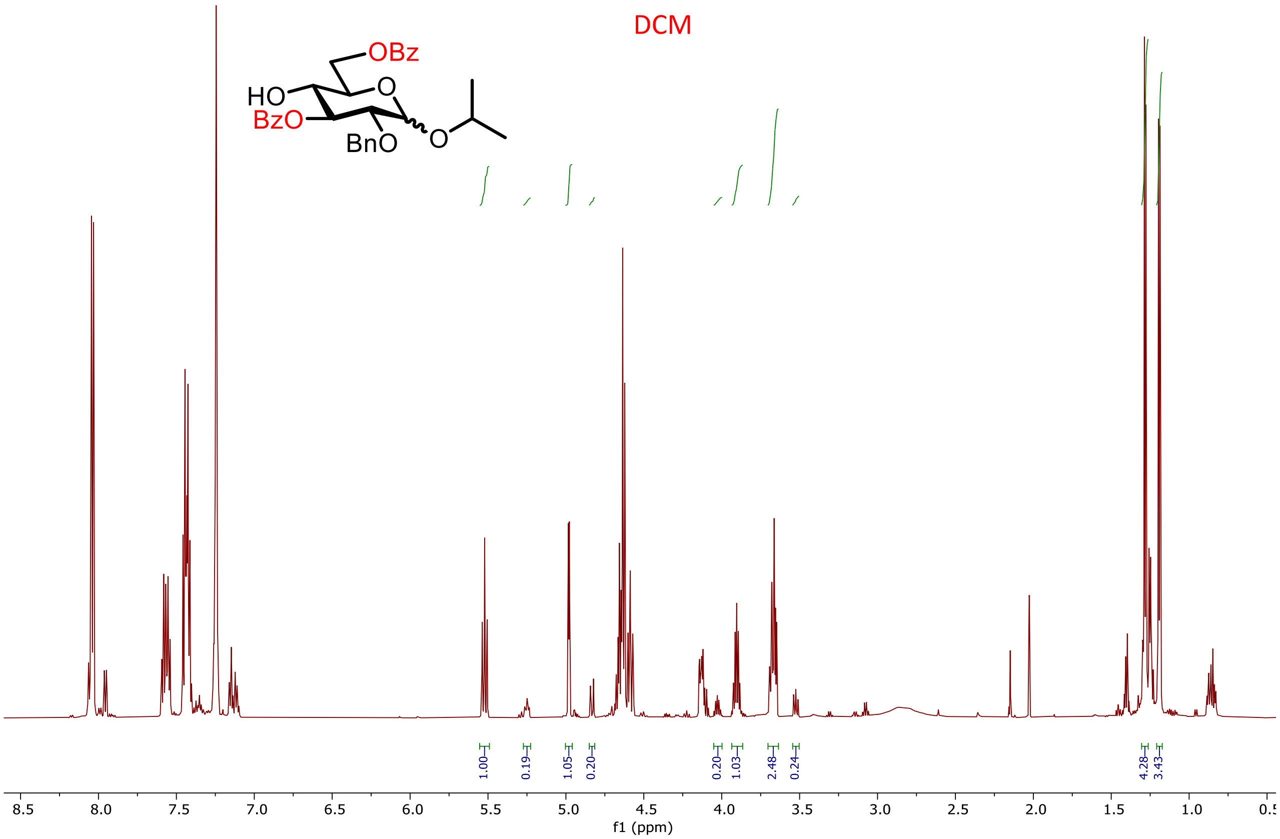

DCM-dioxane: Total yield: 84%. Ratio of anomer α : β = 5.1 : 1.

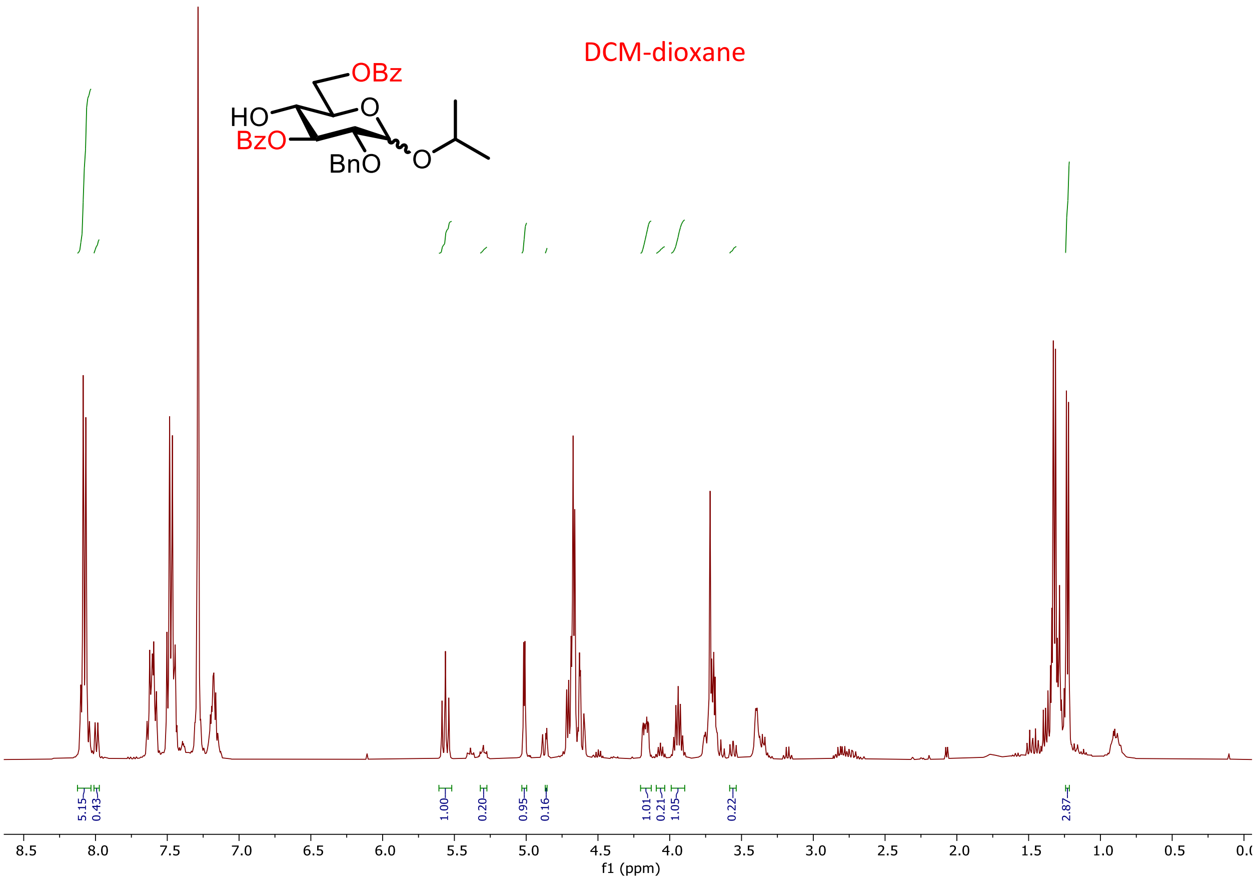

6. Automated Glycan Assembly

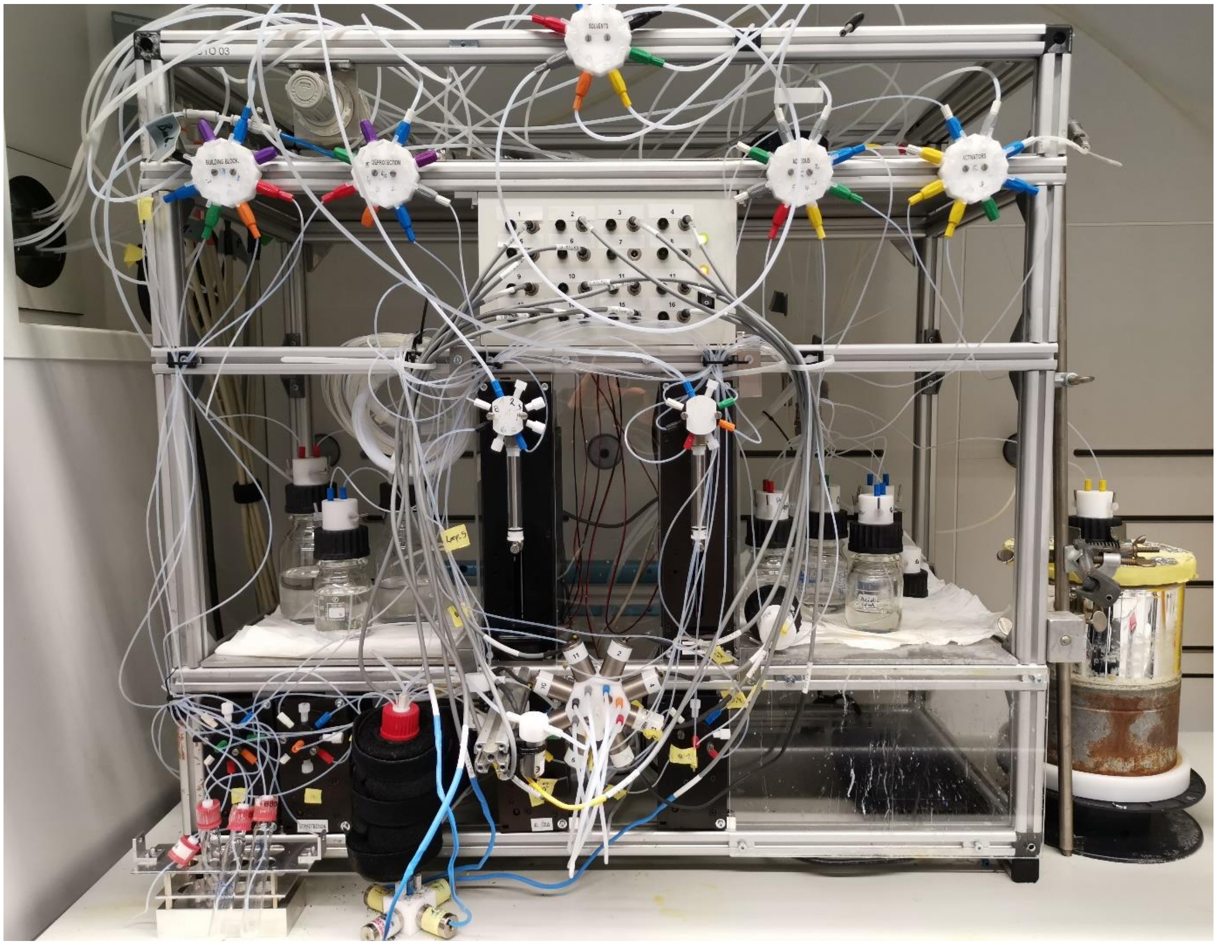

Picture of the home-built synthesizer used in this study.

6.1 General Materials and Methods

All solvents used were HPLC-grade. The solvents used for the building block, activator, TMSOTf and capping solutions were taken from an anhydrous solvent system (jcmeyer-solvent systems). The building blocks were co-evaporated three times with toluene and dried for 1 h under high vacuum before use. Activator, capping, deprotection, acidic wash and building block solutions were freshly prepared and kept under argon during the automation run. All yields of products obtained by AGA were calculated on the basis of resin loading. Resin loading was determined following previously established procedures.[5]

(1) Preparation of stock solutions

Building Block: between 0.1 mmol of building block was dissolved in DCM (1 mL).

TMSOTf solution: TMSOTf (0.45 mL) was added to DCM (40 mL).

Activator solution A (for thioglycoside): 1.50 g of recrystallized NIS was dissolved in 40 mL of a 2:1 mixture of anhydrous DCM and anhydrous dioxane. Then triflic acid (100 µL) was added. The solution was kept at 0°C for the duration of the automation run.

Activator solution B (for phosphate donor): TMSOTf (0.90 mL) was added to DCM (40 mL).

Capping solution: A solution of 10% acetic anhydride and 2% methanesulfunic acid in DCM (v/v) was prepared.

Fmoc deprotection solution: A solution of 20% triethylamine in DMF (v/v) was prepared.

Lev deprotection solution: A solution of hydrazine acetate (725 mg) in the mixture of pyridine (40 mL), acetic acid (10 mL) and water (2.5 mL) was prepared.

(2) Modules for automated synthesis

Module A: Resin Preparation for Synthesis (20 min)

The automated syntheses were performed on 40 mg resin scale:

1. Conjugation-ready linker with a loading of 0.3 mmol/g **35** for solid-phase glycosylation study.

2. Traceless linker with a loading of 0.4 mmol/g **42** for the synthesis of starch/glycogen α-glucan.

Resin was placed in the reaction vessel and swollen in DCM for 20 min at room temperature prior to synthesis. During this time, all reagent lines needed for the synthesis were washed and primed. Before the first glycosylation, the resin was washed with the DMF, THF, and DCM (three times each with 2 mL for 25 s).

Module B: Acidic Wash with TMSOTf Solution (20 min)

The resin was swollen in 2 mL DCM and the temperature of the reaction vessel was adjusted to -20°C. Upon reaching the low temperature, TMSOTf solution (1 mL) was added drop wise to the reaction vessel. After bubbling for 3 min, the acidic solution was drained and the resin was washed with 2 mL DCM for 25 s.

Action	Cycles	Solution	Amount	T (°C)	Incubation time
**Cooling**	-	-	-	-20	(15 min)*
**Deliver**	1	DCM	2 mL	-20	-
**Deliver**	1	TMSOTf solution	1 mL	-20	3 min
**Wash**	1	DCM	2 mL	-20	25 sec

*Time required to reach the desired temperature.

Module C1: Thioglycoside Glycosylation × 1 Cycle (75 min)

The building block solution (0.1 mmol of BB in 1 mL of DCM per glycosylation) was delivered to the reaction vessel. After the set temperature was reached, the reaction was started by drop wise addition of the activator solution (1.0 mL, excess). The glycosylation conditions are building block dependent (we report the most common set of conditions). After completion of the reaction, the solution is drained and the resin was washed with DCM, DCM : dioxane (1:2, 3 mL for 20 s) and DCM (two times, each with 2 mL for 25 s). The temperature of the reaction vessel is increased to 25°C for the next module.

Action	Cycles	Solution	Amount	T (°C)	Incubation time
**Cooling**	-	-	-	-20	-
**Deliver**	1	BB solution	1 mL	-20	-
**Deliver**	1	Activator solution A	1 mL	-20	-
**Reaction time**	1			-20<br>to 0	5 min<br>60 min
**Wash**	1	DCM	2 mL	0	5 sec
**Wash**	1	DCM : Dioxane (1:2)	2 mL	0	20 sec
**Heating**	-	-	-	25	-
**Wash**	2	DCM	2 mL	> 0	25 sec

Module C2: Thioglycoside Glycosylation × 2 Cycle (145 min)

The building block solution (0.1 mmol of BB in 1 mL of DCM per glycosylation) was delivered to the reaction vessel. After the set temperature was reached, the reaction was started by drop wise addition of the activator solution (1.0 mL, excess). The glycosylation conditions are building block dependent (we report the most common set of conditions). After completion of the first glycosylation, the previous steps were repeated again. The solution is drained and the resin was washed with DCM, DCM : dioxane (1:2, 3 mL for

20 s) and DCM (two times, each with 2 mL for 25 s). The temperature of the reaction vessel is increased to 25°C for the next module.

Action	Cycles	Solution	Amount	T (°C)	Incubation time
**Cooling**	-	-	-	-20	-
**Deliver**	1	BB solution	1 mL	-20	-
**Deliver**	1	Activator solution A	1 mL	-20	-
**Reaction time**	1			-20<br>to 0	5 min<br>60 min
**Deliver**	1	BB solution	1 mL	-20	-
**Deliver**	1	Activator solution A	1 mL	-20	-
**Reaction time**	1			-20<br>to 0	5 min<br>60 min
**Wash**	1	DCM	2 mL	0	5 sec
**Wash**	1	DCM : Dioxane (1:2)	2 mL	0	20 sec
**Heating**	-	-	-	25	-
**Wash**	2	DCM	2 mL	> 0	25 sec

Module C3: Phosphate Glycosylation × 2 Cycle (105 min)

The building block solution (0.1 mmol of BB in 1 mL of DCM per glycosylation) was delivered to the reaction vessel. After the set temperature was reached, the reaction was started by drop wise addition of the activator solution (1.0 mL, excess). The glycosylation conditions are building block dependent (we report the most common set of conditions). After completion of the first glycosylation, the previous steps were repeated again. The solution is drained and the resin was washed with DCM, DCM : dioxane (1:2, 3 mL for 20 s) and DCM (two times, each with 2 mL for 25 s). The temperature of the reaction vessel is increased to 25°C for the next module.

Action	Cycles	Solution	Amount	T (°C)	Incubation time
**Cooling**	-	-	-	-20	-
**Deliver**	1	BB solution	1 mL	-20	-
**Deliver**	1	Activator solution B	1 mL	-20	-
**Reaction time**	1			-20<br>to 0	5 min<br>40 min
**Deliver**	1	BB solution	1 mL	-20	-
**Deliver**	1	Activator solution B	1 mL	-20	-
**Reaction time**	1			-20<br>to 0	5 min<br>40 min

**Wash**	1	DCM	2 mL	0	5 sec
**Wash**	1	DCM : Dioxane (1:2)	2 mL	0	20 sec
**Heating**	-	-	-	25	-
**Wash**	2	DCM	2 mL	> 0	25 sec

Module D: Capping (30 min)

The resin was washed with DMF (two times with 2 mL for 25 s) and the temperature of the reaction vessel was adjusted to 25°C. Pyridine solution (2 mL, 10% in DMF) was delivered into the reaction vessel. After 1 min, the reaction solution was drained and the resin washed with DCM (three times with 3 mL for 25 s). Capping solution (4 mL) was delivered into the reaction vessel. After 20 min, the reaction solution was drained and the resin washed with DCM (three times with 3 mL for 25 s).

Action	Cycles	Solution	Amount	T (°C)	Incubation time
**Heating**	-	-	-	25	(5 min)*
**Wash**	2	DMF	2 mL	25	25 sec
**Deliver**	1	10% Pyridine in DMF	2 mL	25	1 min
**Wash**	3	DCM	2 mL	25	25 sec
**Deliver**	1	Capping Solution	4 mL	25	20 min
**Wash**	3	DCM	2 mL	25	25 sec

*Time required to reach the desired temperature.

Module E1: Fmoc Deprotection (20 min)

The resin was washed with DMF (three times with 2 mL for 25 s) and the temperature of the reaction vessel was adjusted to 25°C. 2 mL of Fmoc deprotection solution was delivered to the reaction vessel. After 5 min, the reaction solution was drained. The deprotection process was repeated three times. The resin was washed with DMF (three times with 3 mL for 25 s) and DCM (five times each with 2 mL for 25 s). The temperature of the reaction vessel is decreased to -20°C for the next module.

Action	Cycles	Solution	Amount	T (°C)	Incubation time
**Wash**	3	DMF	2 mL	25	25 sec
**Deliver**	3	Fmoc depr. Solution	2 mL	25	5 min
**Wash**	1	DMF	2 mL	25	25 sec
**Cooling**	-	-	-	-20	-
**Wash**	3	DMF	2 mL	< 25	25 sec

**Wash**	5	DCM	2 mL	< 25	25 sec

Module E2: Lev Deprotection (80 min)

The resin was washed with DCM (three times with 2 mL for 25 s) and the temperature of the reaction vessel was adjusted to 25°C. 2 mL of Lev deprotection solution was delivered to the reaction vessel. After 30 min, the reaction solution was drained. After washing with DCM (six times with 2 mL for 25 s), the deprotection process was repeated again. When the deprotection was finished, the resin was washed with DMF, THF and DCM (six times with 2 mL for 25 s each). The temperature of the reaction vessel is decreased to -20°C for the next module.

Action	Cycles	Solution	Amount	T (°C)	Incubation time
**Heating**	-	-	-	25	(5 min)*
**Wash**	3	DCM	2 mL	25	25 sec
**Deliver**	1	Lev depr. Solution	2 mL	25	30 min
**Wash**	6	DCM	2 mL	25	25 sec
**Deliver**	1	Lev depr. Solution	2 mL	25	30 min
**Wash**	6	DCM	2 mL	25	25 sec
**Cooling**	-	-	-	-20	-
**Wash**	6	DMF	2 mL	< 25	25 sec
**Wash**	6	THF	2 mL	< 25	25 sec
**Wash**	6	DCM	2 mL	< 25	25 sec

*Time required to reach the desired temperature.

(3) Post-synthesizer manipulations

Module F: Cleavage from Solid Support

The oligosaccharides were cleaved from the solid support using a continuous-flow photoreactor as described previously.[6]

Module G: Solution-phase Methanolysis

The protected oligosaccharide was dissolved in THF (1.0 mL). NaOMe in MeOH (0.5 M, 3 equiv. per benzoyl ester) was added and the solution was stirred at room temperature for 24 h. THF was removed and MeOH (1.0 mL) was added. After another 24 h, the reaction was neutralized with HOAc and concentrated *in vacuo.* The crude compound was used for hydrogenolysis without further purification.

Module H: Hydrogenolysis at Ambient Pressure

The crude compound was dissolved in 2 mL of EA: *t*BuOH: H_2O (1:0.5:0.5). 100% by weight Pd-C (10%) was added and the reaction was stirred under H_2–atmosphere for 6 h. The reaction was filtered through celite and washed with *t*BuOH and H_2O. The filtrates were concentrated *in vacuo*, and dissolved in 3.5 mL water.

Module I: Purification

Purification was conducted at different stages of the synthesis as reported for the individual procedures. The products were analyzed using analytical HPLC (Agilent 1200 Series spectrometer). The purification was conducted using preparative HPLC (Agilent 1200 Series spectrometer).

Method A1: (YMC-Diol-300 column, 150 x 4.6 mm) flow rate of 1.0 mL / min with Hex – 20% EtOAc as eluent [isocratic 20% EtOAc (5 min), linear gradient to 70% EtOAc (35 min), linear gradient to 100% EtOAc (5 min)].

Method A2: (YMC-Diol-300 column, 150 x 4.6 mm) flow rate of 1.0 mL / min with Hex – 20% EtOAc as eluent [isocratic 20% EtOAc (5 min), linear gradient to 70% EtOAc (45 min), linear gradient to 100% EtOAc (5 min)].

Method A3: (YMC-Diol-300 column, 150 x 4.6 mm) flow rate of 1.0 mL / min with Hex – 20% EtOAc as eluent [isocratic 20% EtOAc (5 min), linear gradient to 70% EtOAc (60 min), linear gradient to 100% EtOAc (5 min)].

Method B1: (YMC-Diol-300 column, 150 x 20 mm) flow rate of 15 mL / min with Hex – 20% EtOAc as eluents [isocratic 20% EtOAc (5 min), linear gradient to 70% EtOAc (35 min), linear gradient to 100% EtOAc (5 min)].

Method B2: (YMC-Diol-300 column, 150 x 20 mm) flow rate of 15 mL / min with Hex – 20% EtOAc as eluents [isocratic 20% EtOAc (5 min), linear gradient to 70% EtOAc (45 min), linear gradient to 100% EtOAc (5 min)].

Method B3: (YMC-Diol-300 column, 150 x 20 mm) flow rate of 15 mL / min with Hex – 20% EtOAc as eluents [isocratic 20% EtOAc (5 min), linear gradient to 70% EtOAc (60 min), linear gradient to 100% EtOAc (5 min)].

Method C: (Synergi Hydro RP18 column, 250 x 4.6 mm) flow rate of 1.0 mL / min with H_2O (0.1% formic acid) as eluents [isocratic (5 min), linear gradient to 20% ACN (30 min), linear gradient to 100% ACN (5 min)].

Method D: (Synergi Hydro RP18 column, 250 x 10 mm) flow rate of 4.0 mL / min with H_2O (0.1% formic acid) as eluents [isocratic (5 min), linear gradient to 20% ACN (30 min), linear gradient to 100% ACN (5 min)].

Method E: (Manual normal phase silica gel column chromatography): Hexanes: EtOAc = 2:1 to Hexanes: EtOAc = 1:3.

Following final purification, all deprotected products were lyophilized on a Christ Alpha 2-4 LD plus freeze dryer prior to characterization.

6.2 Solid-phase Glycosylation Study

The selectivity is calculated as the ratio of the amount of desired product containing four α linkages to all pentamer products (peaks are identified by MALDI test), as measured by NMR spectroscopy.

6.2.1 Synthesis of pentasaccharide 36

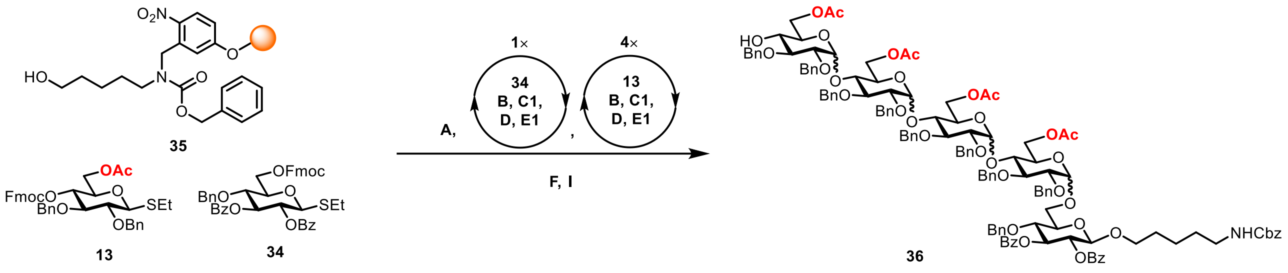

	Module	Conditions
	**A**: Resin Preparation for Synthesis	
	**B**: Acidic Wash with TMSOTf Solution	
	**C1**: Thioglycoside Glycosylation × 1 Cycle	Building block **34**, 6.5 equiv. (-20°C for 5 min, 0°C for 60 min)
	**D**: Capping	
	**E1**: Fmoc Deprotection	
4	**B**: Acidic Wash with TMSOTf Solution	
	**C1**: Thioglycoside Glycosylation × 1 Cycle	Building block **13**, 6.5 equiv. (-20°C for 5 min, 0°C for 60 min)
	**D**: Capping	
	**E1**: Fmoc Deprotection	
	**F:** Cleavage from Solid Support	
	**I**: Purification	**Method E and B1**

Automated synthesis and purification afforded **36** as a white solid (11 mg, 40% for crude pentamer; 4.0 mg, 14% for α only product **36a**[2]). Selectivity 0.35 (by isolated yield).

NP-HPLC of crude 36 after AGA (ELSD trace, Method A1, t_R = 22.6-25.0 min)

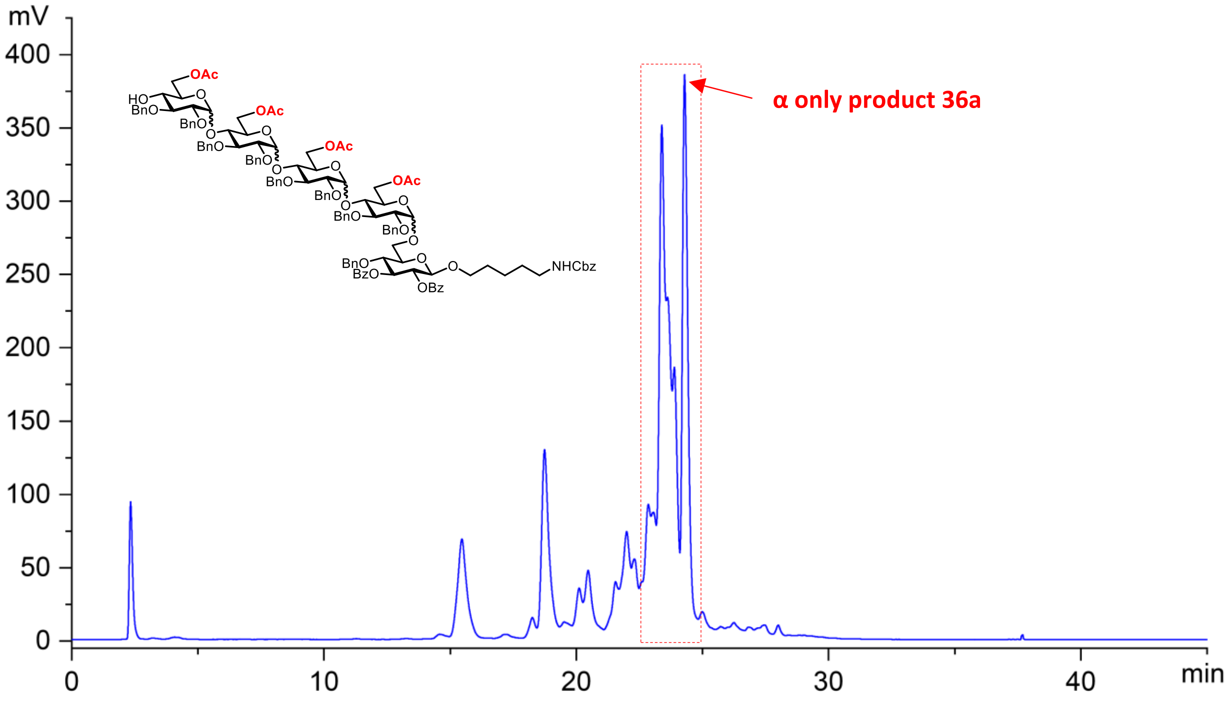

HSQC NMR of 36a ($CDCl_3$)

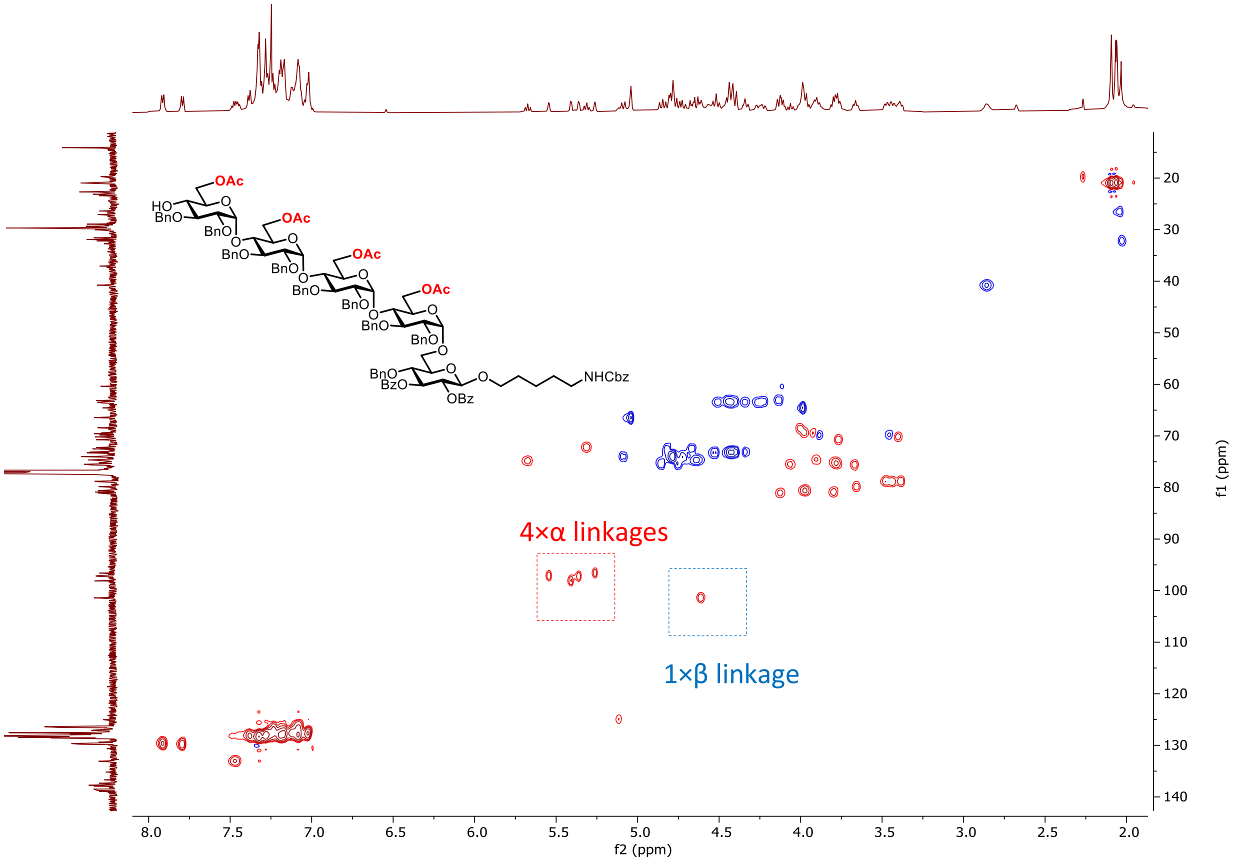

Coupled HSQC NMR of 36a ($CDCl_3$)

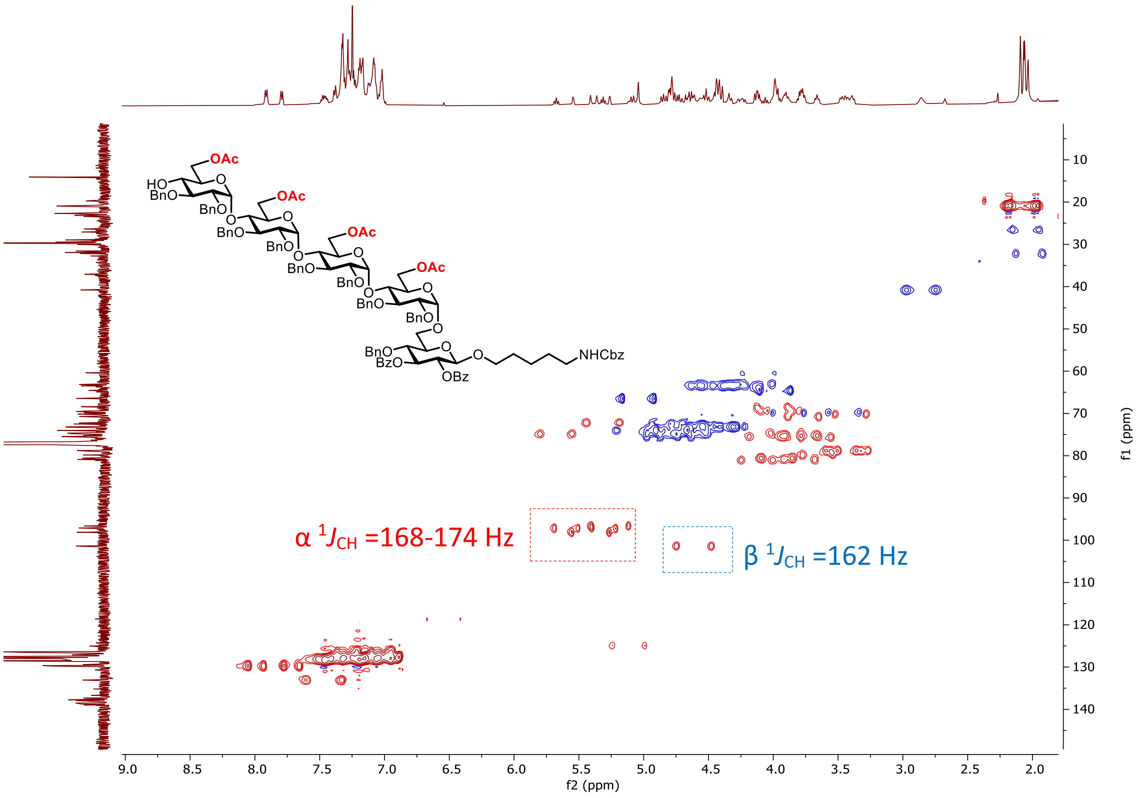

HSQC NMR of crude 36 ($CDCl_3$)

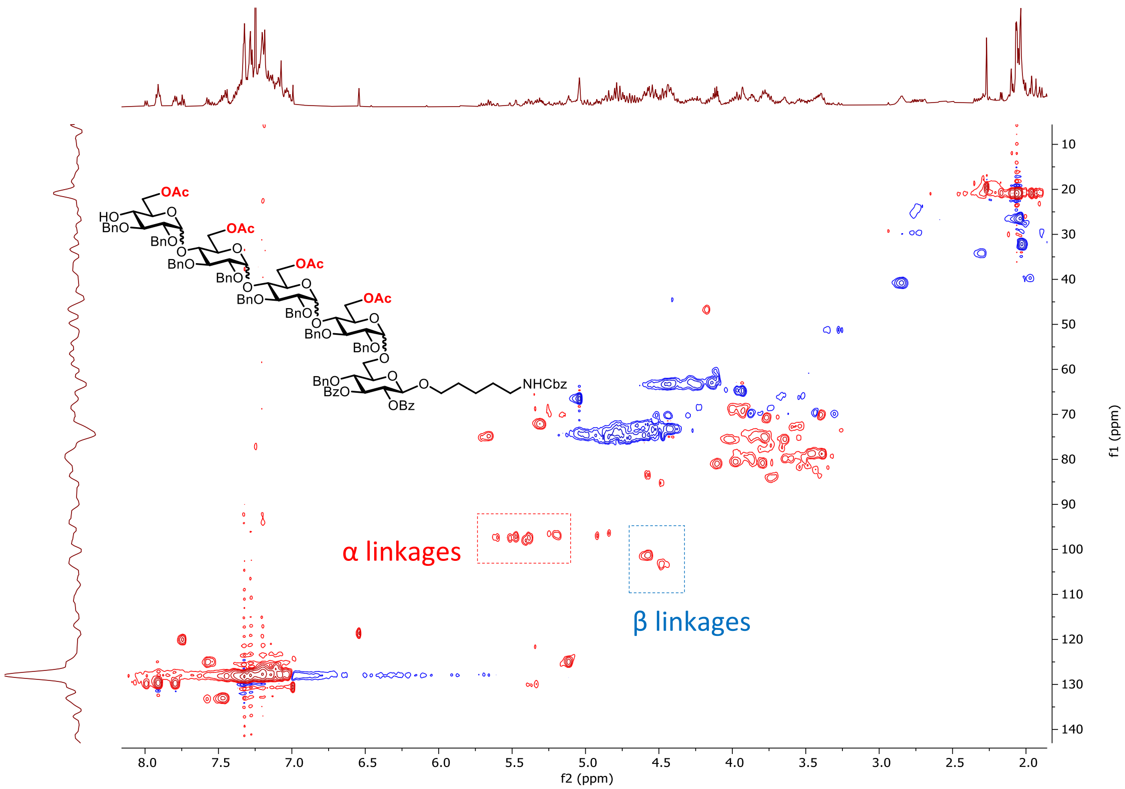

6.2.2 Synthesis of pentasaccharide 37

Thioglycoside:

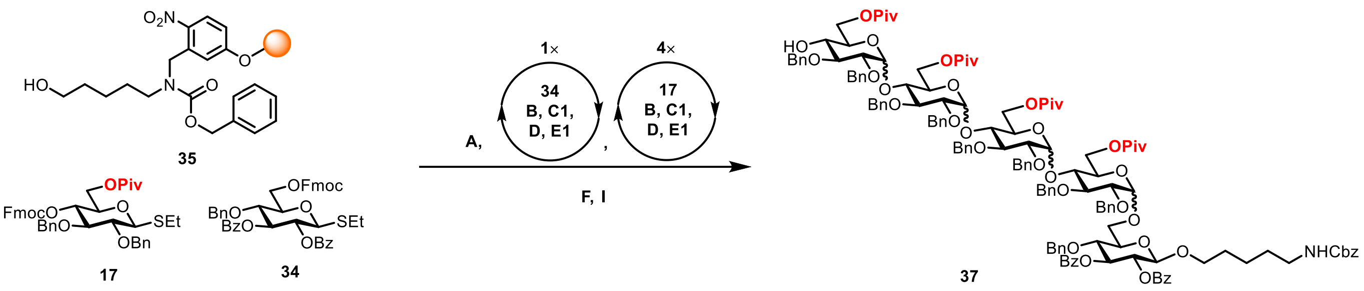

	Module	Conditions
	**A**: Resin Preparation for Synthesis	
	**B**: Acidic Wash with TMSOTf Solution	
	**C1**: Thioglycoside Glycosylation × 1 Cycle	Building block **34**, 6.5 equiv. (-20°C for 5 min, 0°C for 60 min)
	**D**: Capping	
	**E1**: Fmoc Deprotection	
	**B**: Acidic Wash with TMSOTf Solution	
**4**	**C1**: Thioglycoside Glycosylation × 1 Cycle	Building block **17**, 6.5 equiv. (-20°C for 5 min, 0°C for 60 min)
	**D**: Capping	
	**E1**: Fmoc Deprotection	
	**F:** Cleavage from Solid Support	
	**I**: Purification	**Method E and B1**

Automated synthesis and purification afforded **37** as a light yellow oil (1.2 mg, 4% for crude pentamer); m/z (HRMS$^+$) $[M + Na]^+$ 2425.050 ($C_{140}H_{163}O_{34}NNa^+$ requires 2425.094). Selectivity 0.93.

NP-HPLC of crude 37 after AGA (ELSD trace, Method A1, t_R = 17.2-17.6 min)

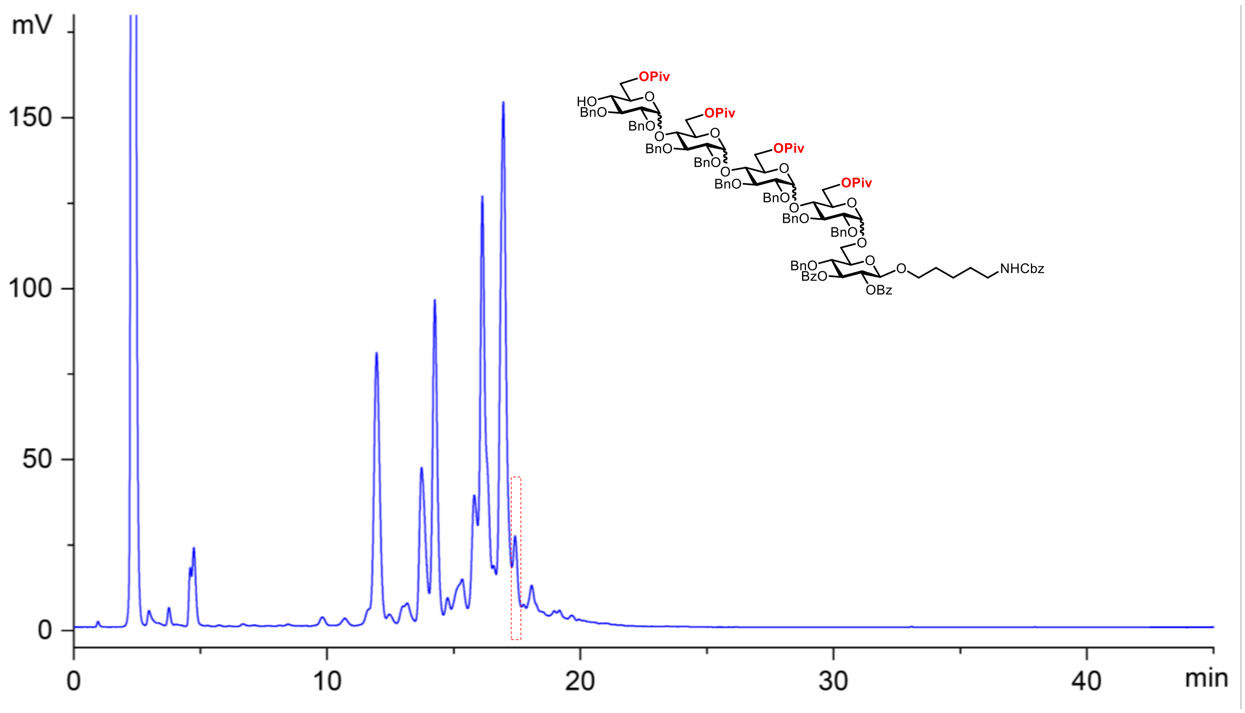

HSQC NMR of crude 37 ($CDCl_3$)

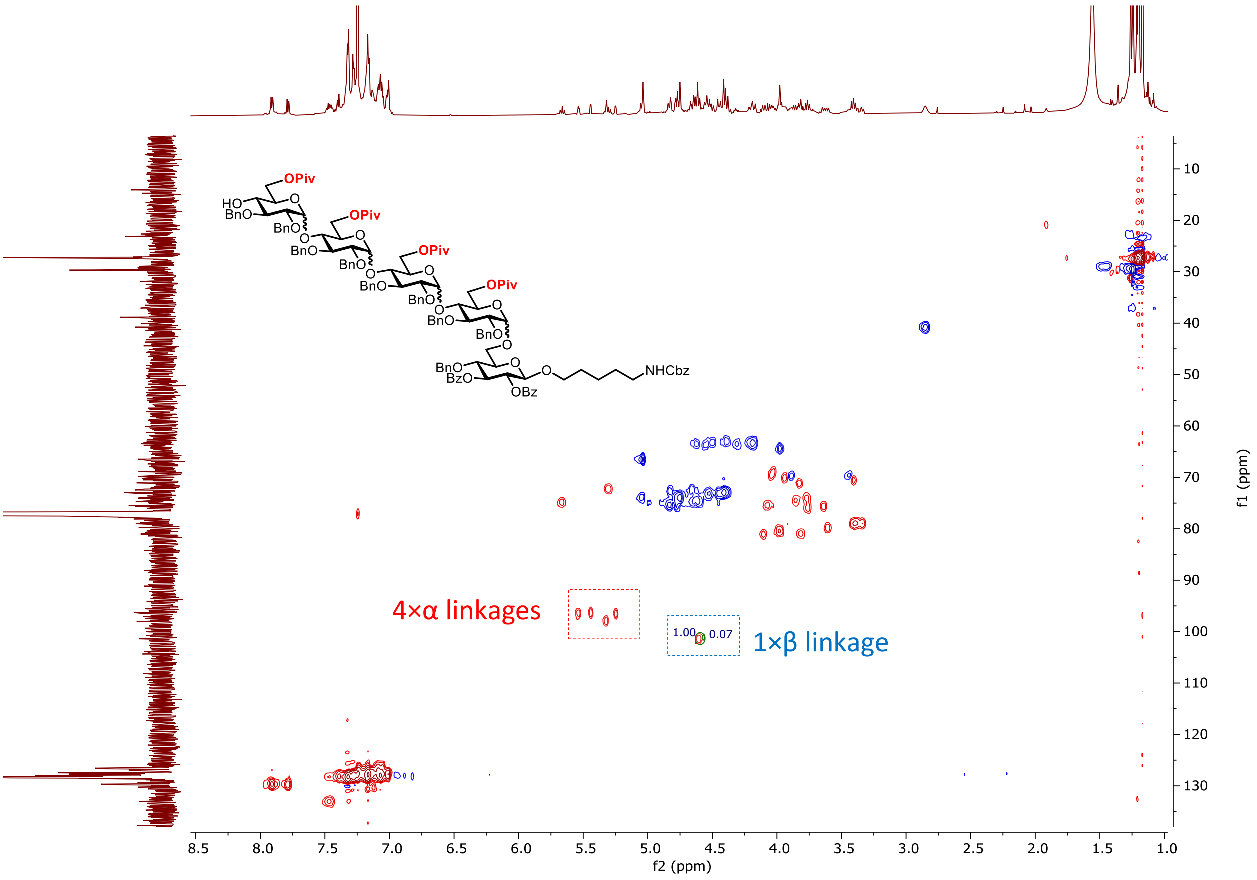

Coupled HSQC NMR of crude 37 ($CDCl_3$)

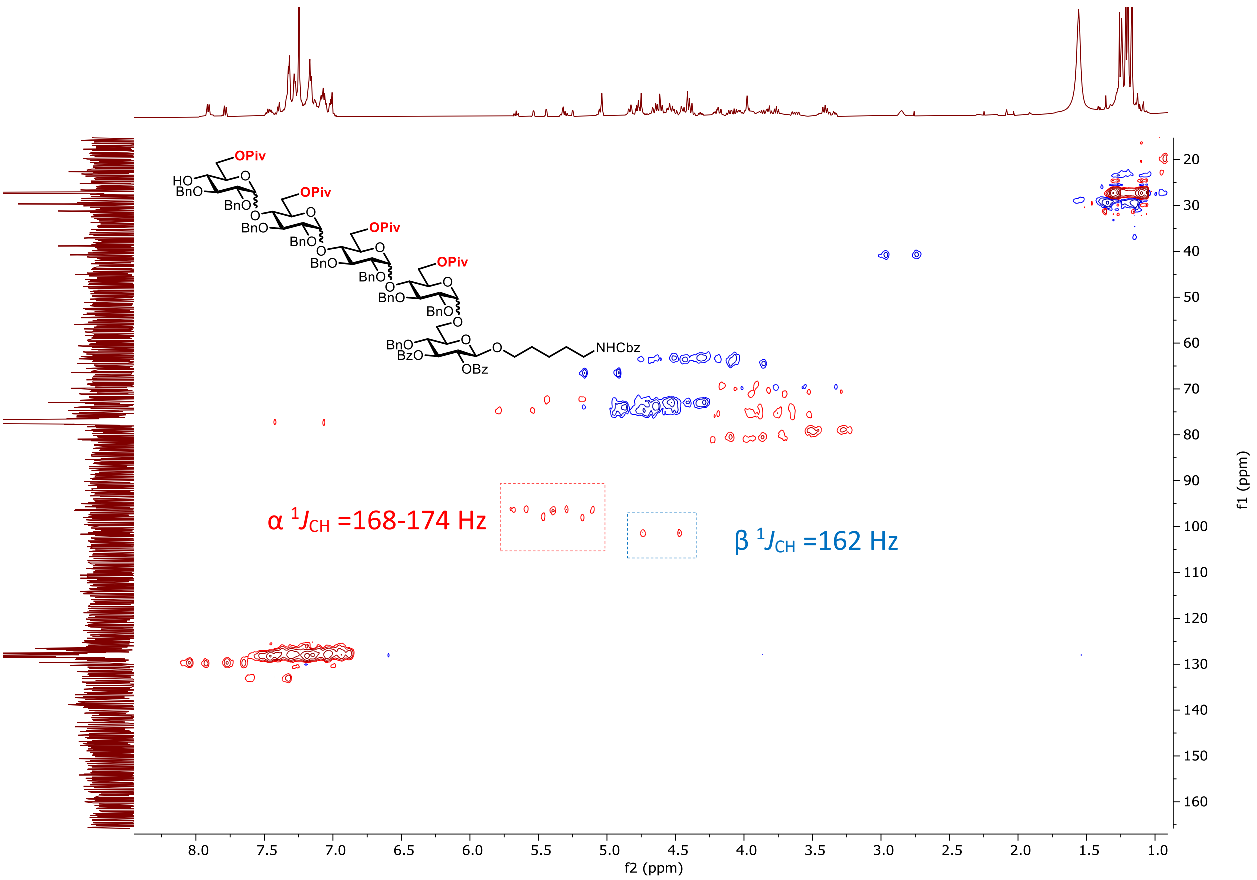

Glycosyl phosphate:

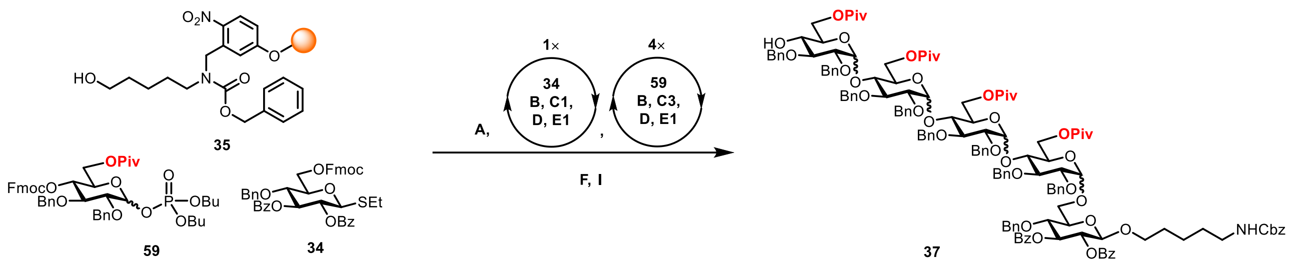

	Module	Conditions
	**A**: Resin Preparation for Synthesis	
	**B**: Acidic Wash with TMSOTf Solution	
	**C1**: Thioglycoside Glycosylation × 1 Cycle	Building block **34**, 6.5 equiv. (-20°C for 5 min, 0°C for 60 min)
	**D**: Capping	
	**E1**: Fmoc Deprotection	
**4**	**B**: Acidic Wash with TMSOTf Solution	
**4**	**C3**: Phosphate Glycosylation × 2 Cycle	Building block **59**, 6.5 equiv. (-20°C for 5 min, 0°C for 40 min)
**4**	**D**: Capping	
**4**	**E1**: Fmoc Deprotection	
	**F:** Cleavage from Solid Support	
	**I**: Purification	**Method E and B1**

Automated synthesis using glycosyl phosphate and purification afforded **37** as a light yellow oil (1.8 mg, 6% for crude pentamer).

NP-HPLC of crude 37 after AGA (ELSD trace, Method A1, t_R = 17.2-17.6 min)

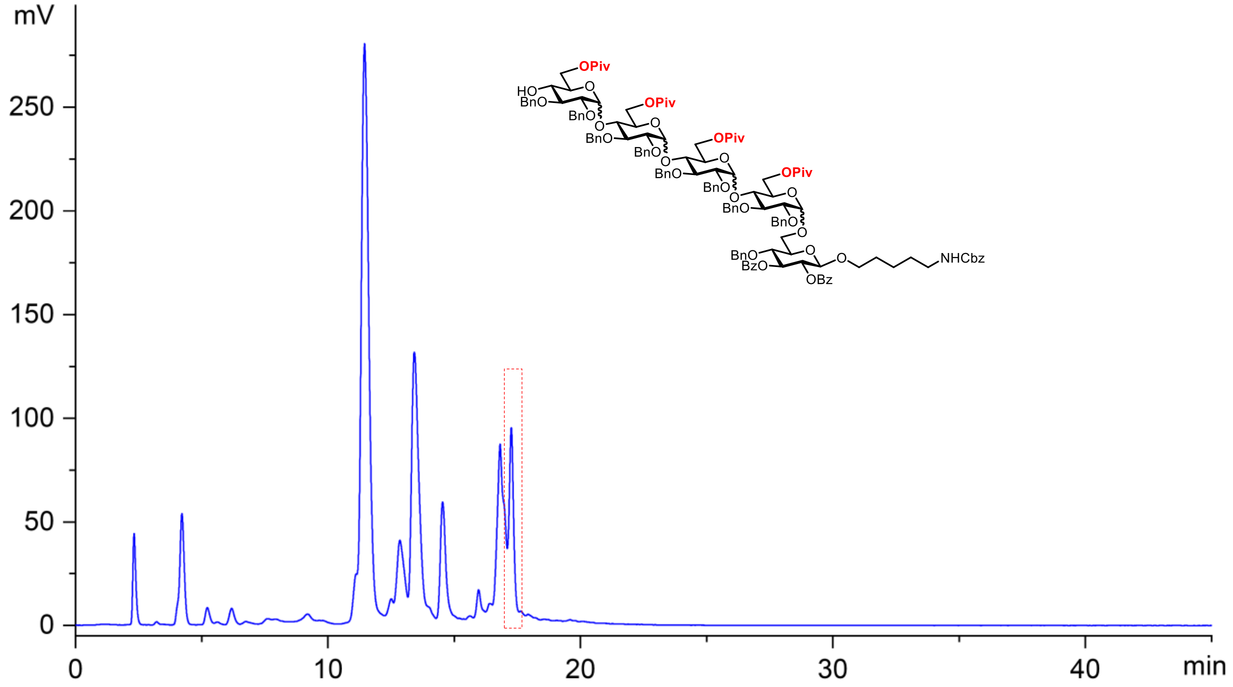

6.2.3 Synthesis of pentasaccharide 38

Thioglycoside:

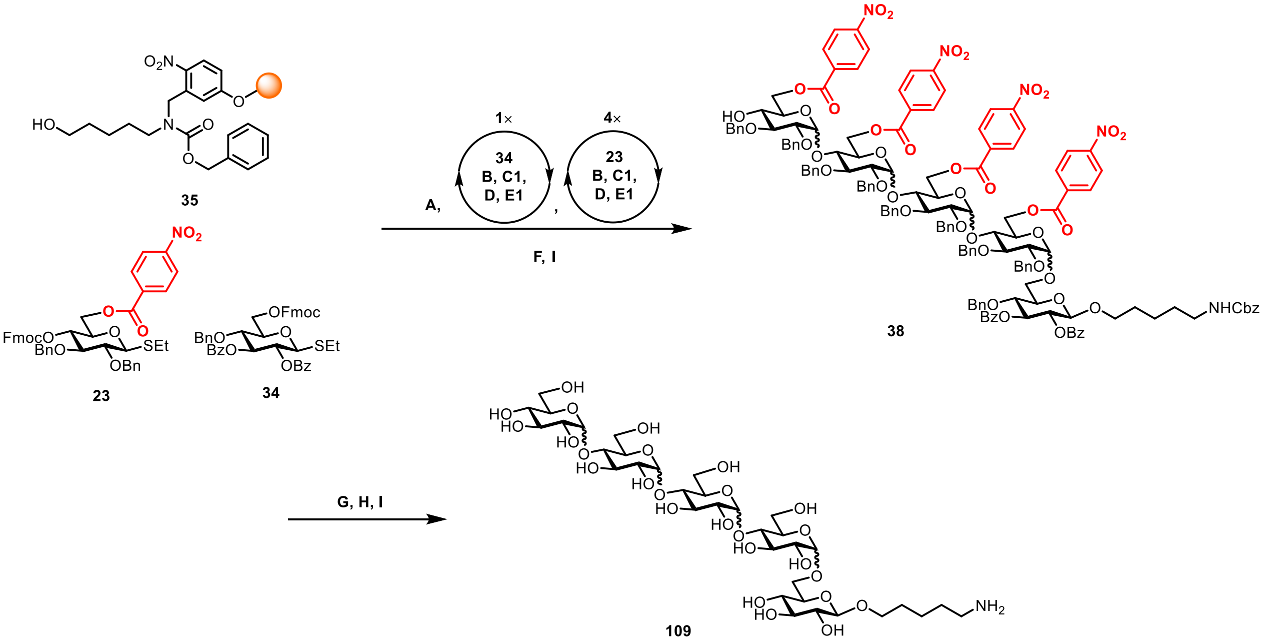

Module	Conditions
**A**: Resin Preparation for Synthesis	
**B**: Acidic Wash with TMSOTf Solution	
**C1**: Thioglycoside Glycosylation × 1 Cycle	Building block **34**, 6.5 equiv. (-20°C for 5 min, 0°C for 60 min)
**D**: Capping	
**E1**: Fmoc Deprotection	
**4** { **B**: Acidic Wash with TMSOTf Solution	
**C1**: Thioglycoside Glycosylation × 1 Cycle	Building block **23**, 6.5 equiv. (-20°C for 5 min, 0°C for 60 min)
**D**: Capping	
**E1**: Fmoc Deprotection }	
**F: Cleavage from Solid Support**	
**I**: Purification	**Method E and B1**
**G**: Solution-phase Methanolysis	
**H**: Hydrogenolysis at Ambient Pressure	
**I**: Purification	**Method D**

Automated synthesis and purification afforded **38** as a light yellow solid (4.7 mg, 14% for crude pentamer); m/z (HRMS$^+$) [M + Na]$^+$ 2684.976 ($C_{148}H_{143}O_{42}N_5Na^+$ requires 2684.910). Deprotection and purification afforded **109**[2] as a white solid (1.0 mg, 9% overall yield for crude pentamer); Selectivity 0.68.

NP-HPLC of crude 38 after AGA (ELSD trace, Method A1, t_R = 21.4-22.0 min)

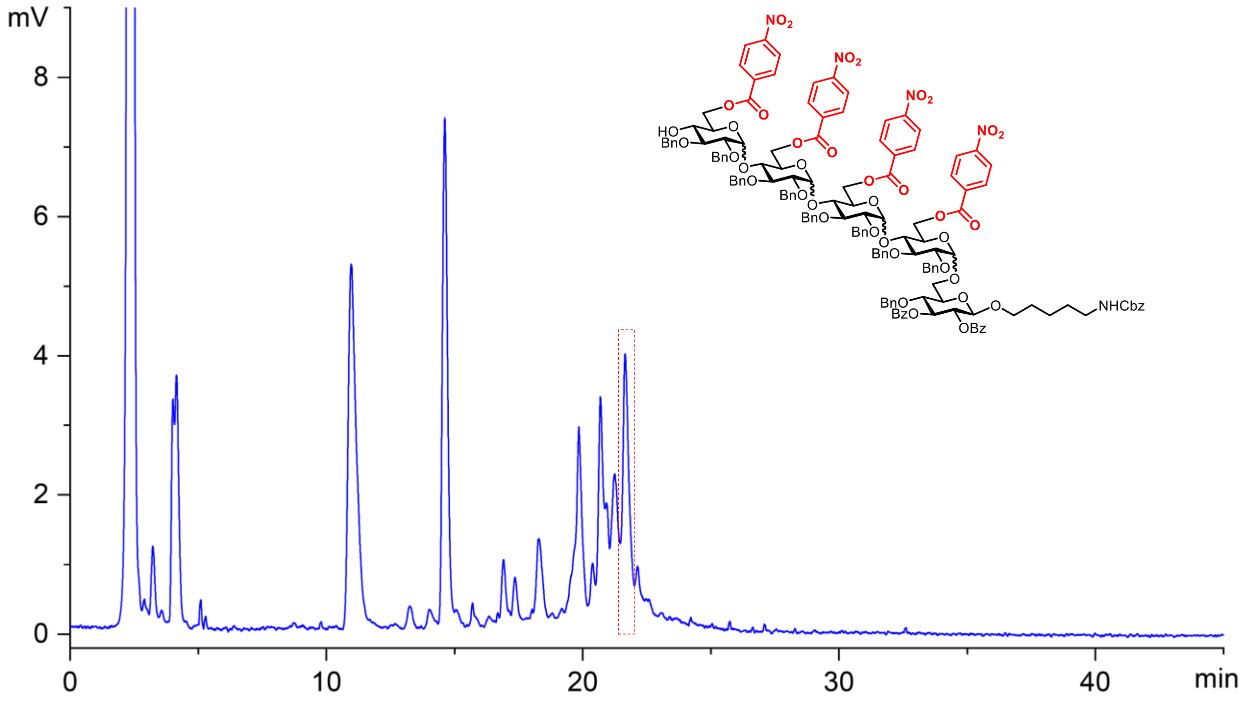

HSQC NMR of crude 109 (from 38) (D_2O)

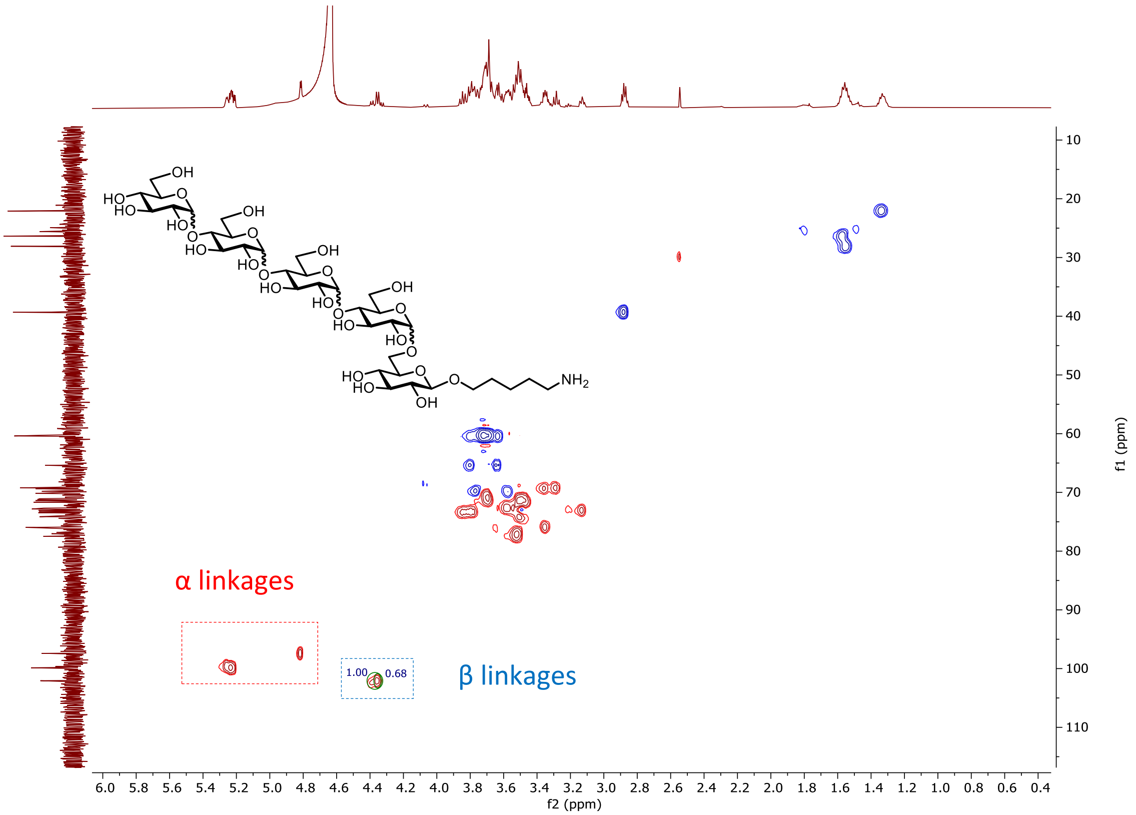

Coupled HSQC NMR of crude 109 (from 38) (D_2O)

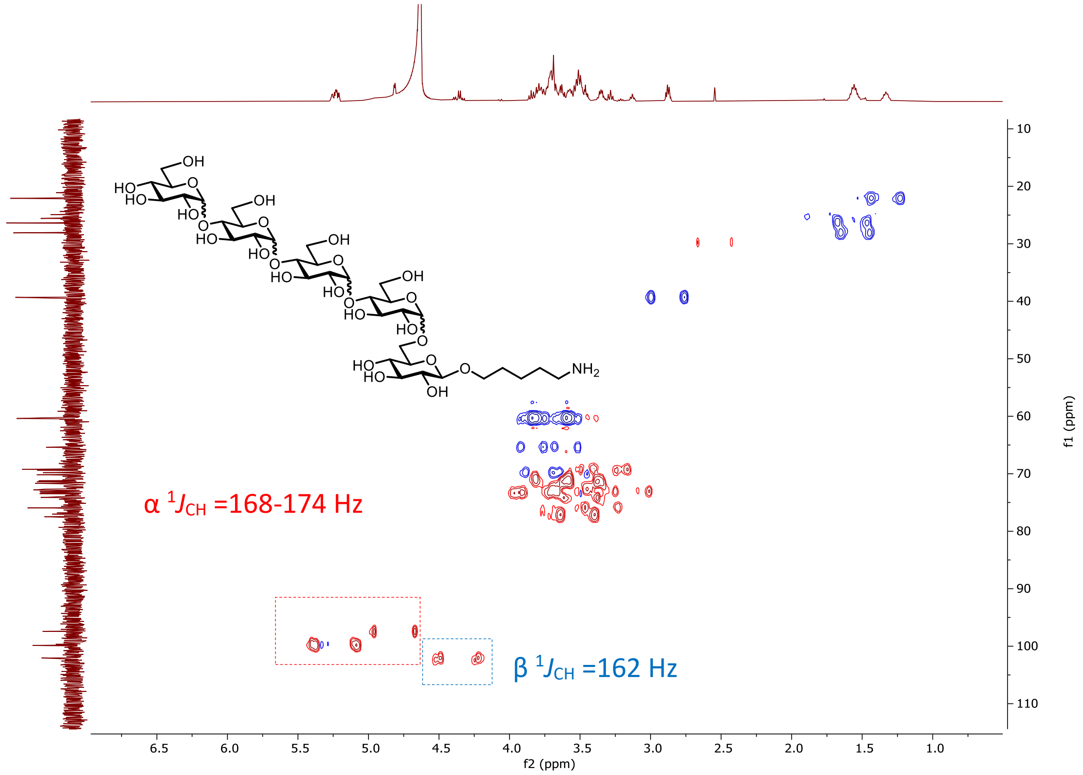

Glycosyl phosphate:

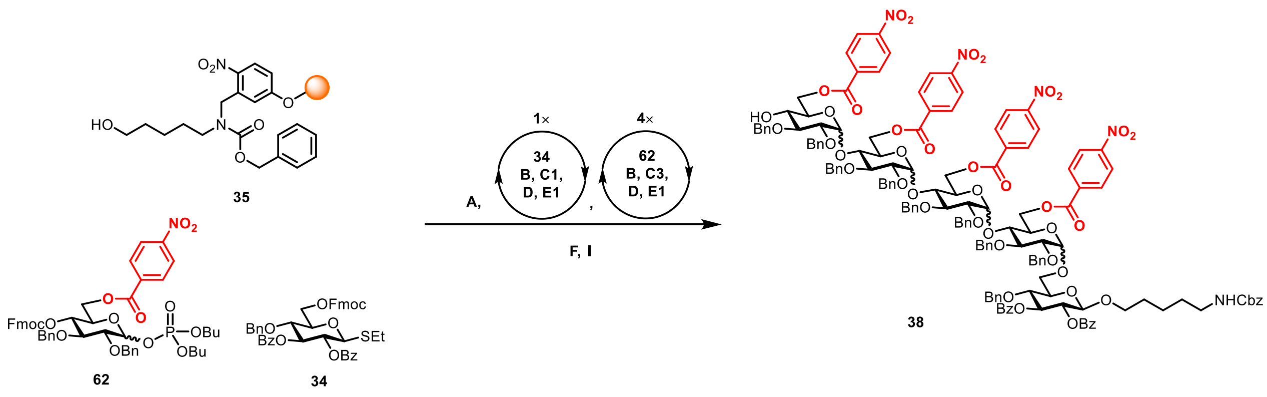

	Module	Conditions
	**A**: Resin Preparation for Synthesis	
	**B**: Acidic Wash with TMSOTf Solution	
	**C1**: Thioglycoside Glycosylation × 1 Cycle	Building block **34**, 6.5 equiv. (-20°C for 5 min, 0°C for 60 min)
	**D**: Capping	
	**E1**: Fmoc Deprotection	
4	**B**: Acidic Wash with TMSOTf Solution	
4	**C3**: Phosphate Glycosylation × 2 Cycle	Building block **62**, 6.5 equiv. (-20°C for 5 min, 0°C for 40 min)
4	**D**: Capping	
	**E1**: Fmoc Deprotection	
	**F:** Cleavage from Solid Support	
	**I**: Purification	**Method E and B1**

Automated synthesis using glycosyl phosphate and purification afforded **38** as a light yellow solid (6.7 mg, 20% for crude pentamer).

NP-HPLC of crude 38 after AGA (ELSD trace, Method A1, t_R = 21.4-22.0 min)

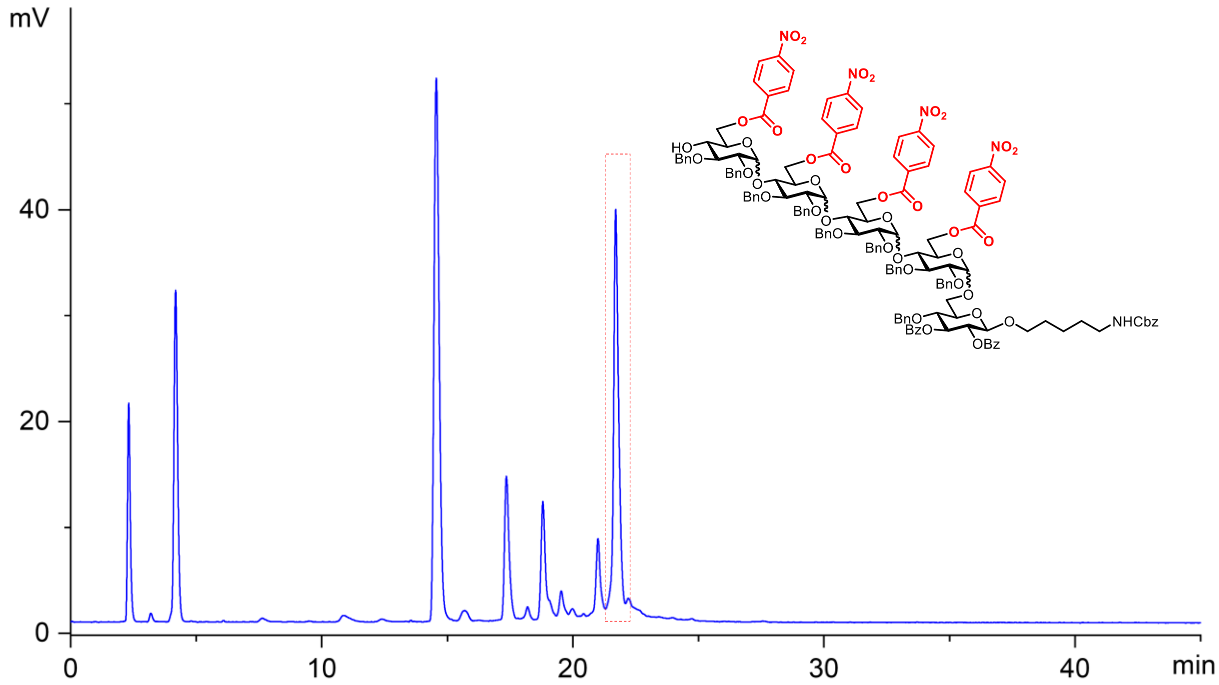

6.2.4 Synthesis of pentasaccharide 39

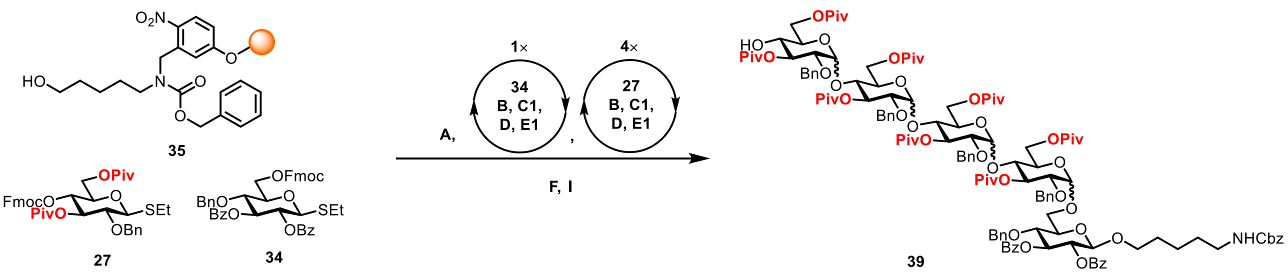

	Module	Conditions
	**A**: Resin Preparation for Synthesis	
	**B**: Acidic Wash with TMSOTf Solution	
	**C1**: Thioglycoside Glycosylation × 1 Cycle	Building block **34**, 6.5 equiv. (-20°C for 5 min, 0°C for 60 min)
	**D**: Capping	
	**E1**: Fmoc Deprotection	
4	**B**: Acidic Wash with TMSOTf Solution	
4	**C1**: Thioglycoside Glycosylation × 1 Cycle	Building block **27**, 6.5 equiv. (-20°C for 5 min, 0°C for 60 min)
4	**D**: Capping	
4	**E1**: Fmoc Deprotection	
	**F:** Cleavage from Solid Support	
	**I**: Purification	**Method E and B1**

Automated synthesis and purification afforded **39** as a colorless oil (0.9 mg, 3% for crude pentamer); m/z (HRMS$^+$) $[M + Na]^+$ 2401.178 ($C_{132}H_{171}O_{38}NNa^+$ requires 2401.137). Selectivity >0.95.

NP-HPLC of crude 39 after AGA (ELSD trace, Method A1, t_R = 14.7-15.2 min)

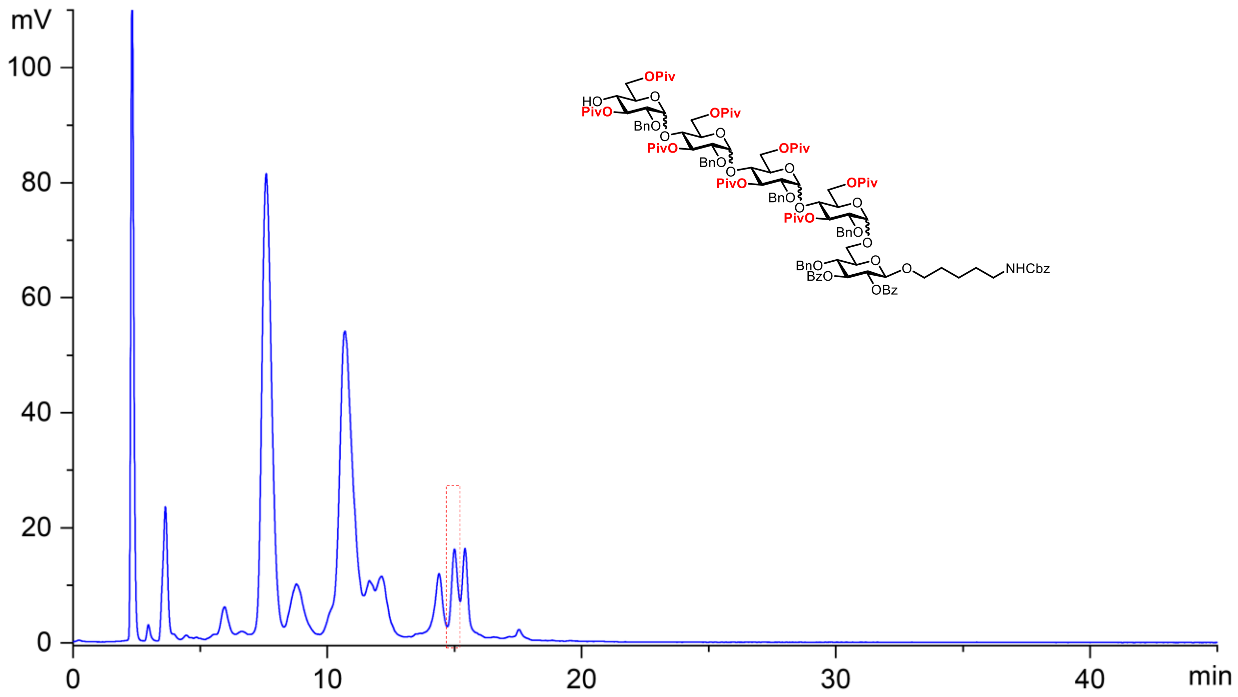

HSQC NMR of crude 39 (CDCl₃)

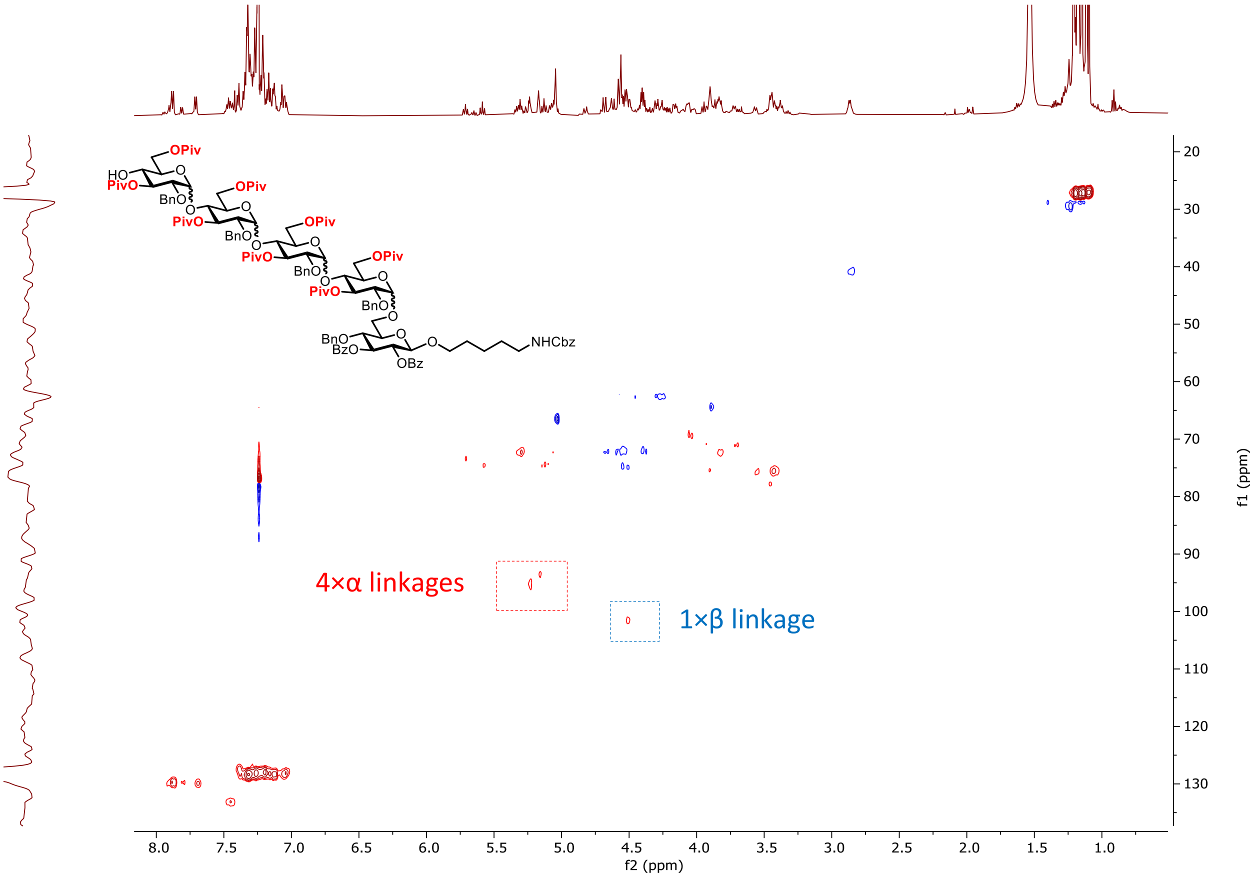

Coupled HSQC NMR of crude 39 ($CDCl_3$)

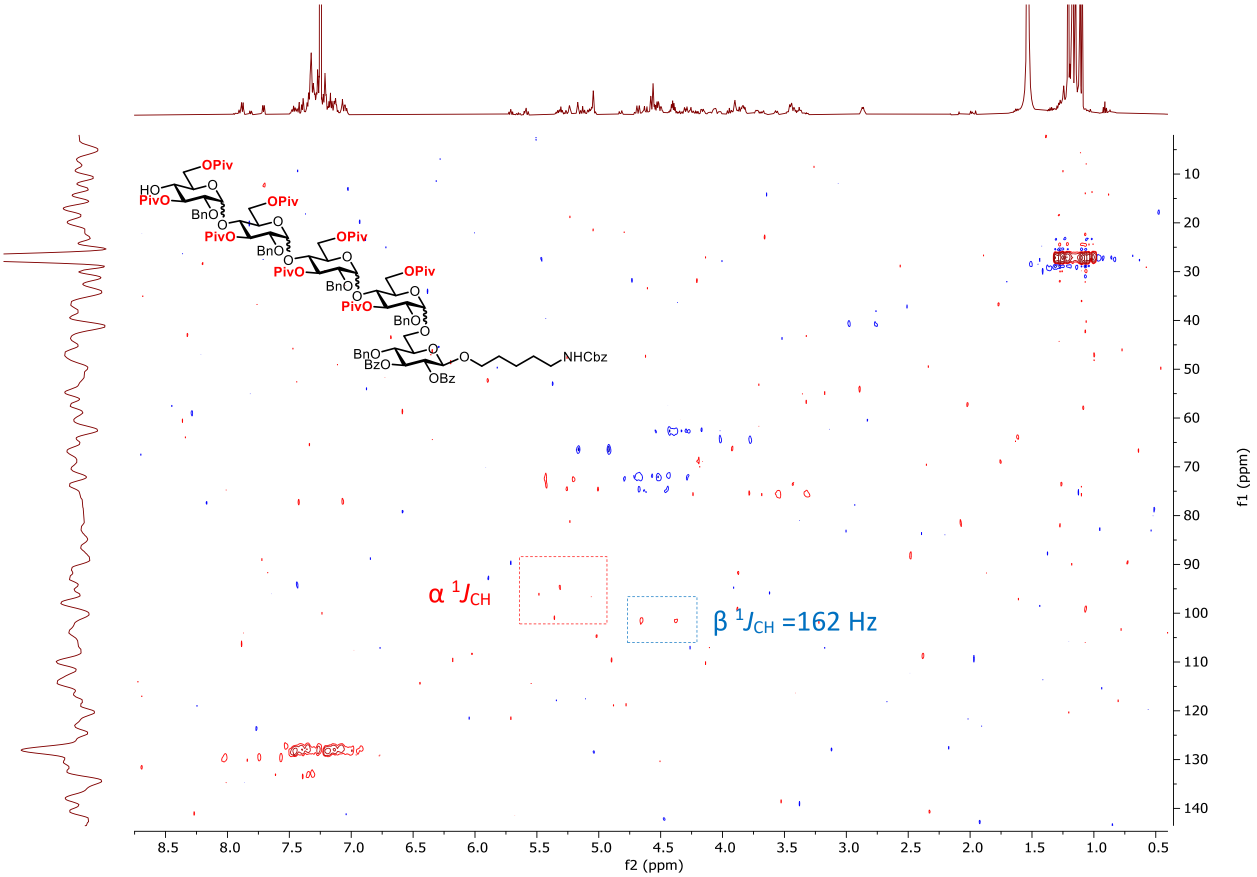

6.2.5 Synthesis of pentasaccharide 40

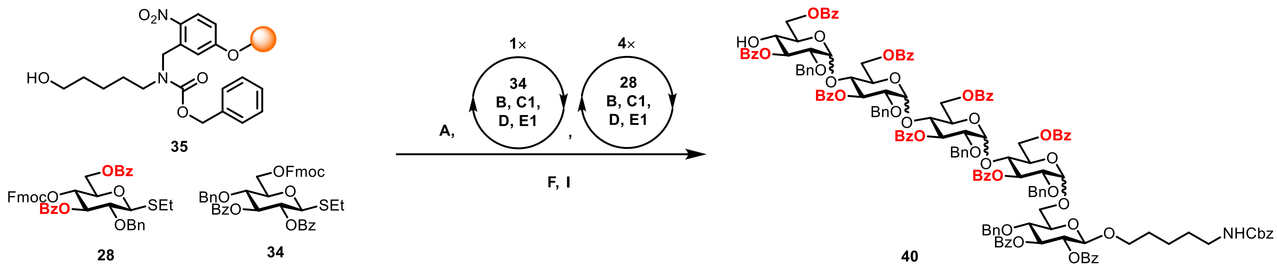

	Module	Conditions
	**A**: Resin Preparation for Synthesis	
	**B**: Acidic Wash with TMSOTf Solution	
	**C1**: Thioglycoside Glycosylation × 1 Cycle	Building block **34**, 6.5 equiv. (-20°C for 5 min, 0°C for 60 min)
	**D**: Capping	
	**E1**: Fmoc Deprotection	
4	**B**: Acidic Wash with TMSOTf Solution	
	**C1**: Thioglycoside Glycosylation × 1 Cycle	Building block **28**, 6.5 equiv. (-20°C for 5 min, 0°C for 60 min)
	**D**: Capping	
	**E1**: Fmoc Deprotection	
	**F:** Cleavage from Solid Support	
	**I**: Purification	**Method E and B1**

Automated synthesis and purification afforded **40** as a white solid (5.2 mg, 17% for crude pentamer); m/z (HRMS$^+$) [M + Na]$^+$ 2560.952 ($C_{148}H_{139}O_{38}NNa^+$ requires 2560.886). Selectivity 0.74.

NP-HPLC of crude 40 after AGA (ELSD trace, Method A1, t_R = 24.4-25.1 min)

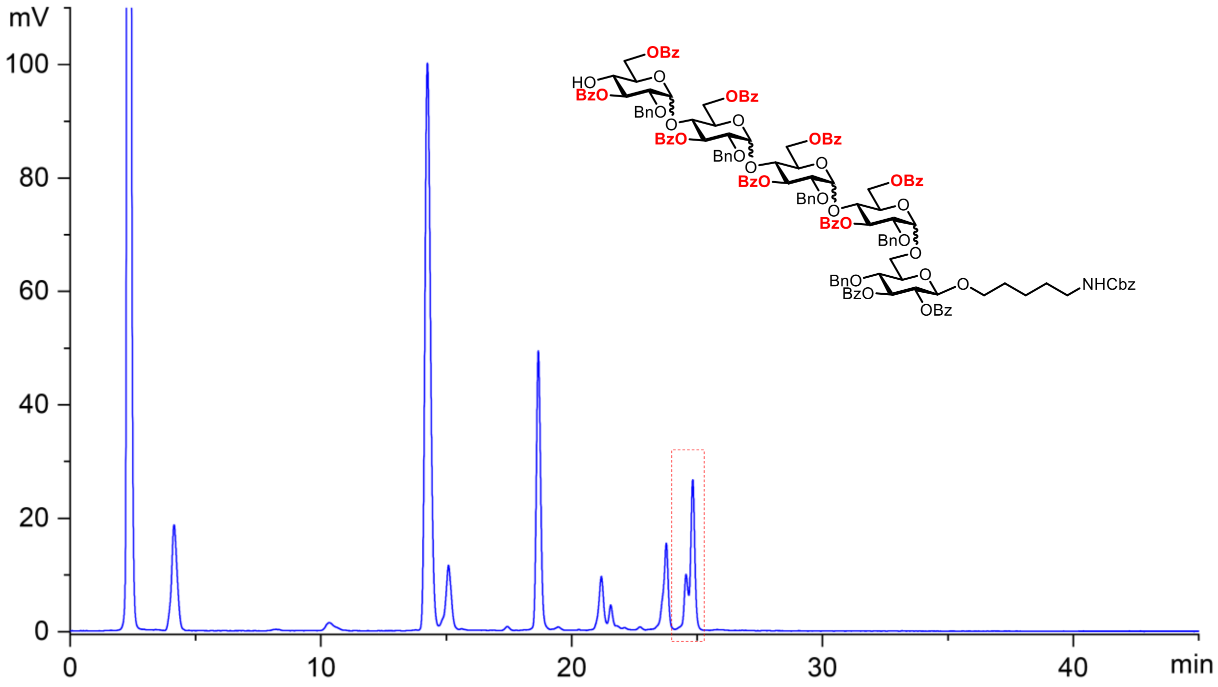

HSQC NMR of crude 40 ($CDCl_3$)

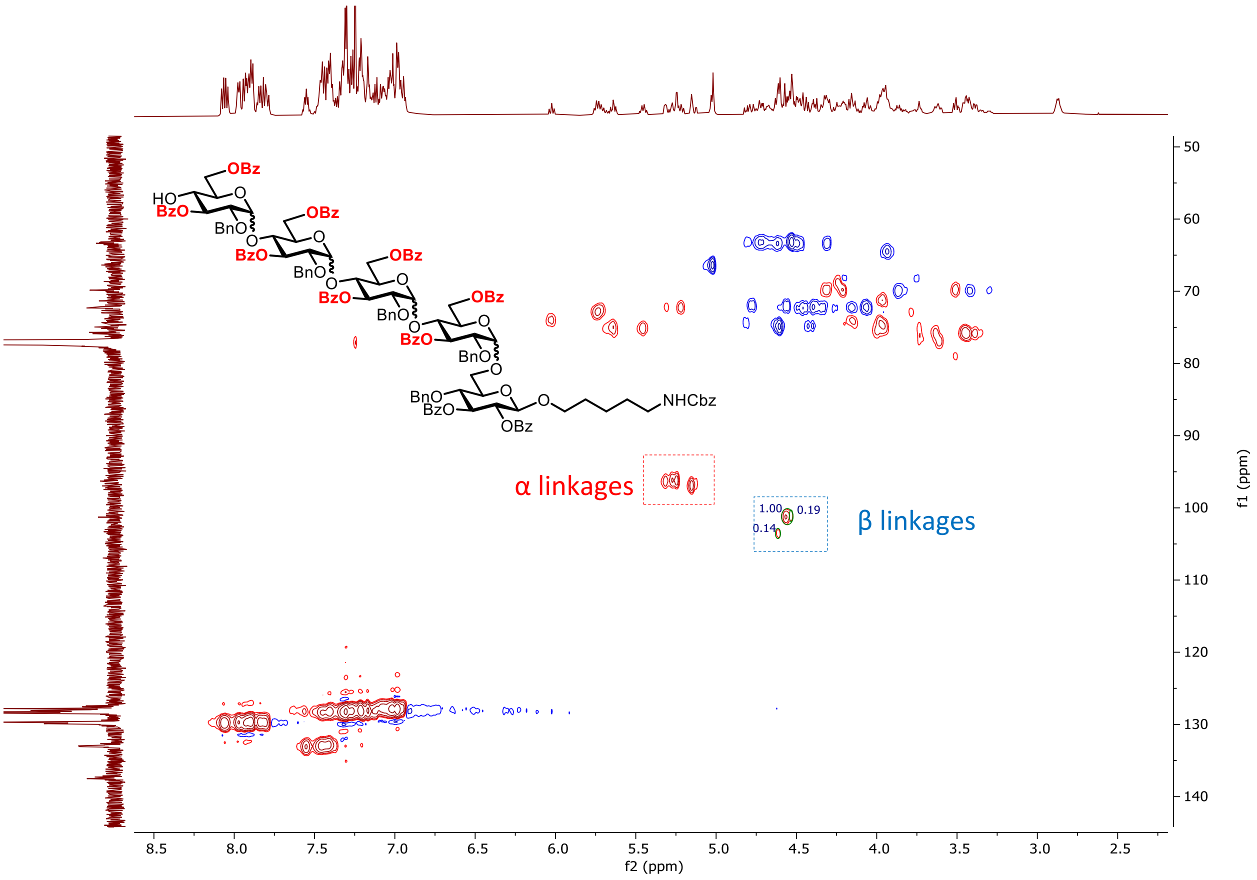

Coupled HSQC NMR of crude 40 ($CDCl_3$)

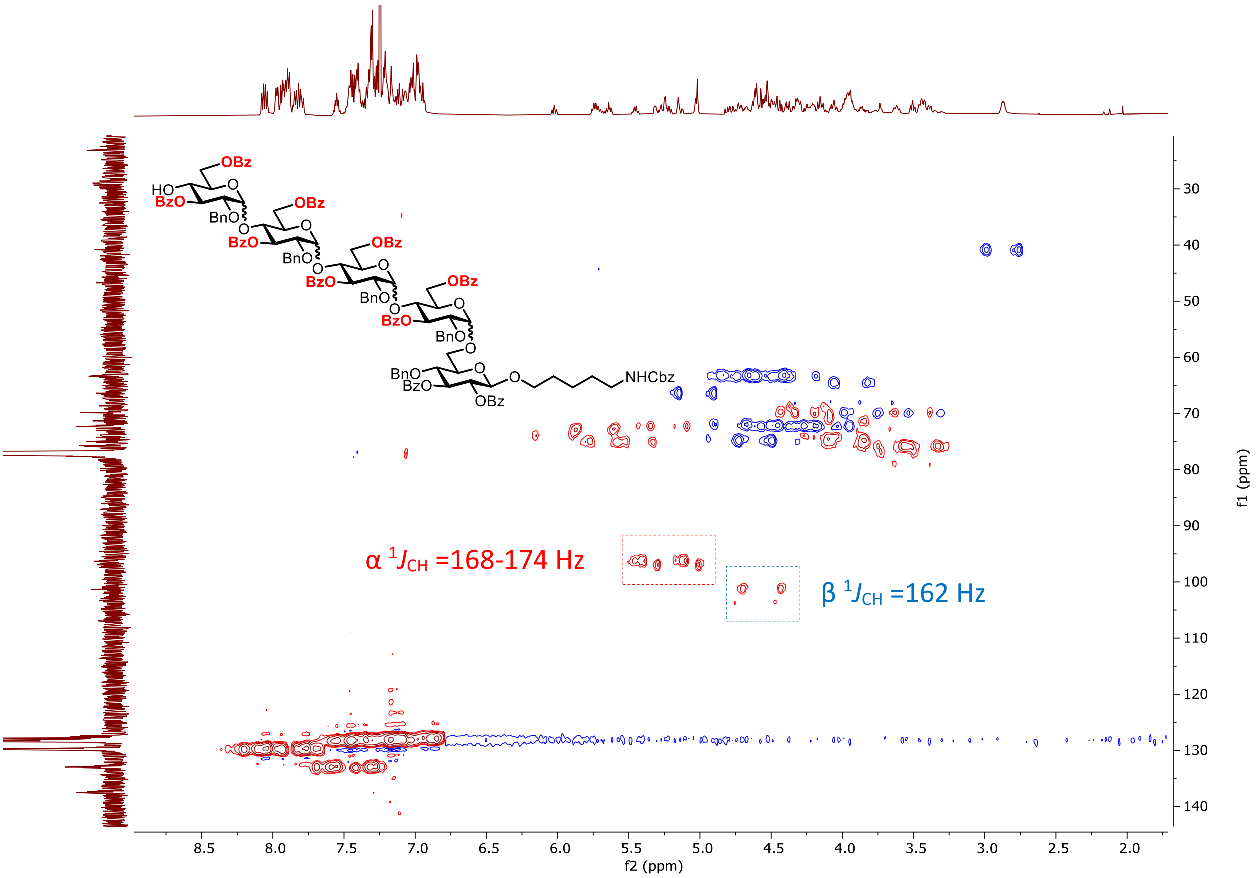

6.2.6 Synthesis of pentasaccharide 41

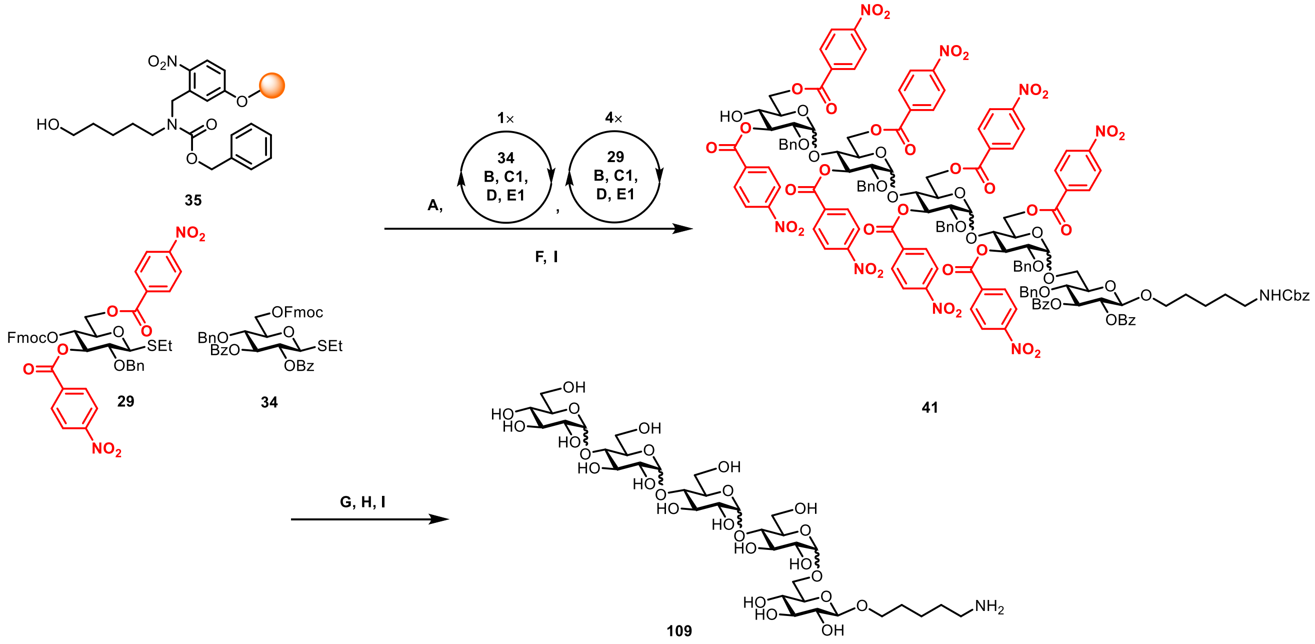

	Module	Conditions
	**A**: Resin Preparation for Synthesis	
	**B**: Acidic Wash with TMSOTf Solution	
	**C1**: Thioglycoside Glycosylation × 1 Cycle	Building block **34**, 6.5 equiv. (-20°C for 5 min, 0°C for 60 min)
	**D**: Capping	
	**E1**: Fmoc Deprotection	
4	**B**: Acidic Wash with TMSOTf Solution	
	**C1**: Thioglycoside Glycosylation × 1 Cycle	Building block **29**, 6.5 equiv. (-20°C for 5 min, 0°C for 60 min)
	**D**: Capping	
	**E1**: Fmoc Deprotection	
	**F:** Cleavage from Solid Support	
	**I**: Purification	**Method E and B1**
	**G**: Solution-phase Methanolysis	
	**H**: Hydrogenolysis at Ambient Pressure	
	**I**: Purification	**Method D**

Automated synthesis and purification afforded **41** as a light yellow solid (6.7 mg, 19% for crude pentamer); m/z (HRMS$^+$) [M + Na]$^+$ 2920.702 ($C_{148}H_{131}O_{54}N_9Na^+$ requires 2920.767). Deprotection and purification afforded **109**[2] as white solid (1.1 mg, 10% overall yield for crude pentamer); Selectivity 0.45.

NP-HPLC of crude 41 after AGA (ELSD trace, Method A1, t_R = 25.1-26.1 min)

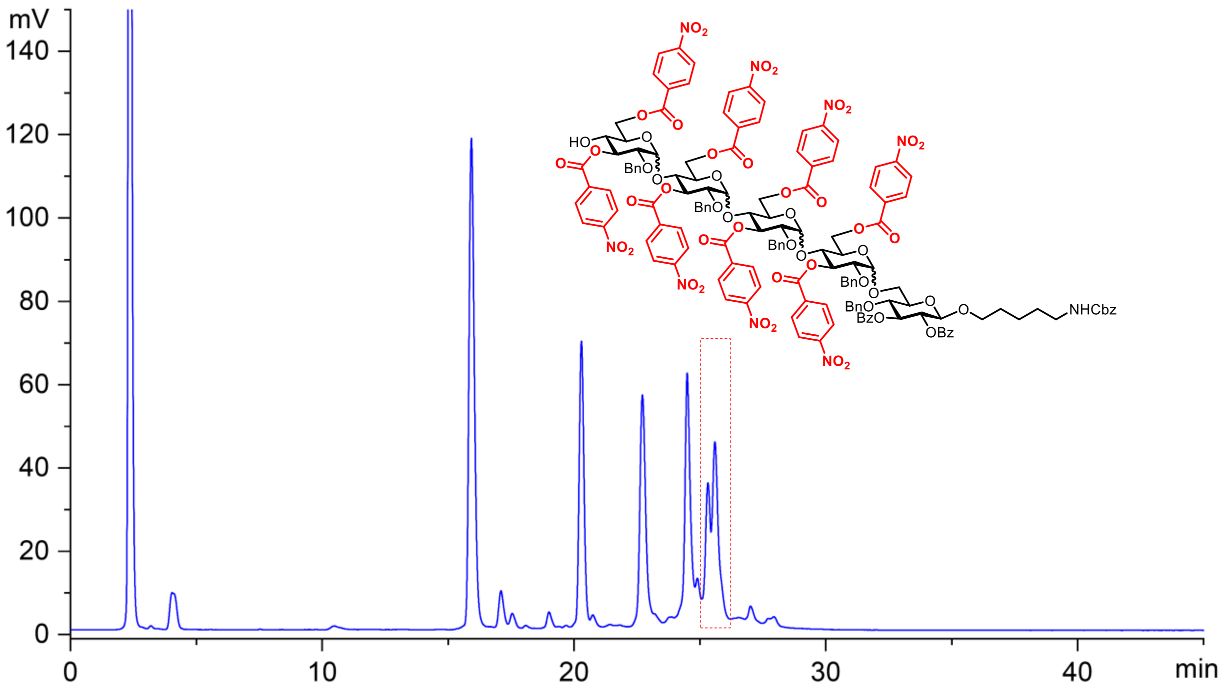

HSQC NMR of crude 109 (from 41) (D_2O)

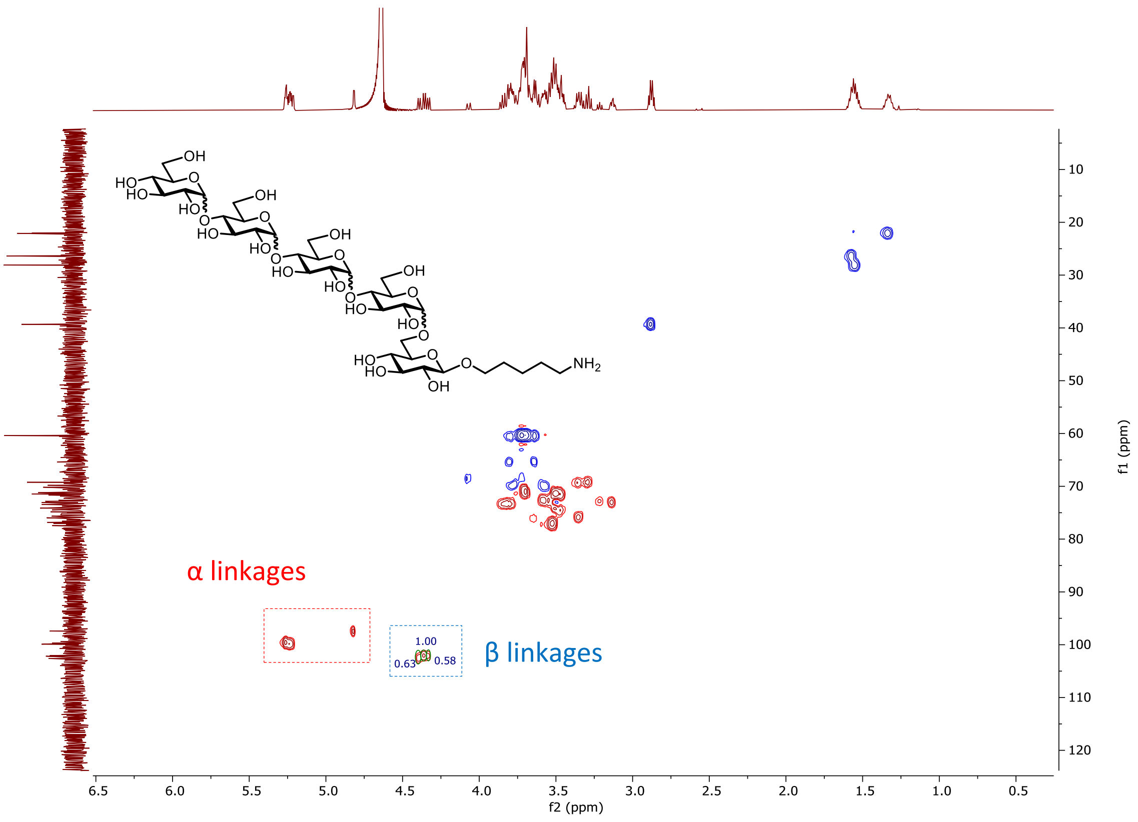

Coupled HSQC NMR of crude 109 (from 41) (D_2O)

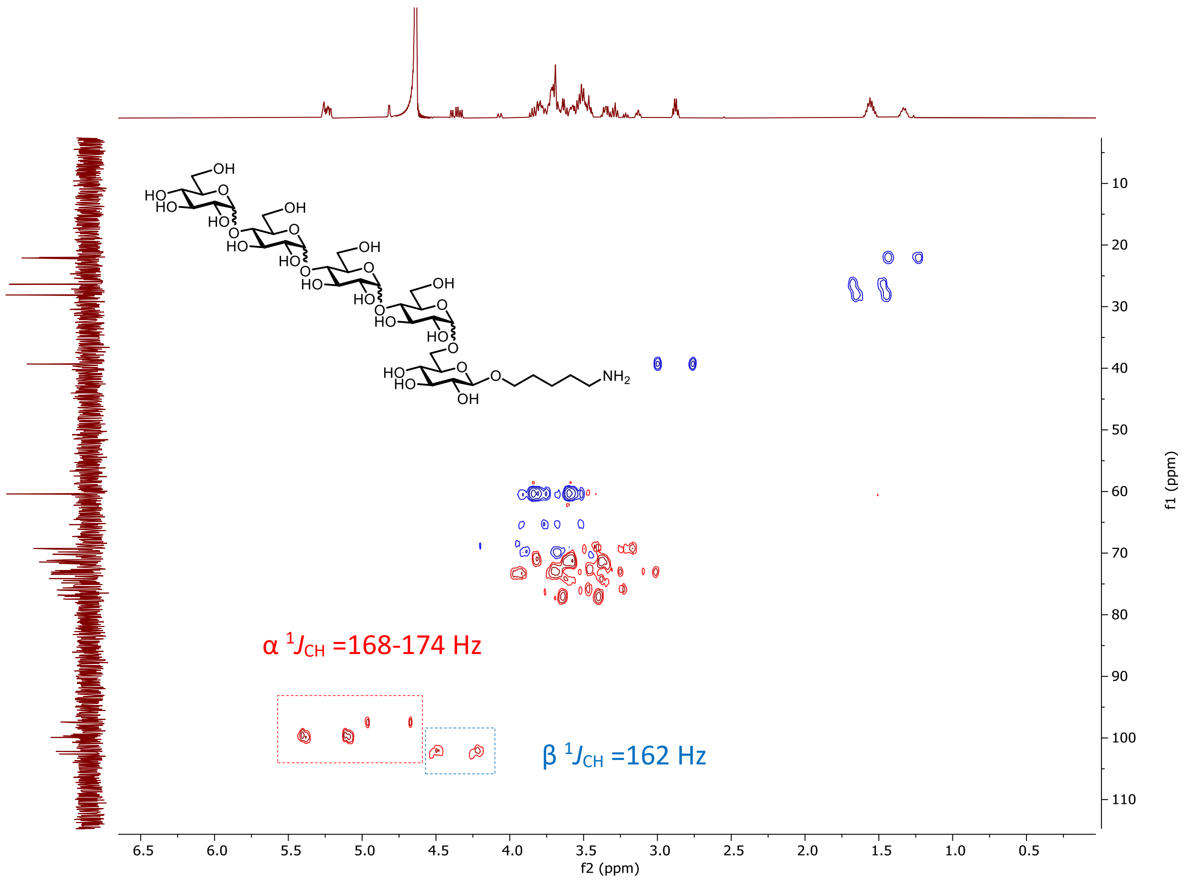

7. Synthesis of Starch/Glycogen α-Glucan

7.1 Synthesis of amylose tetramer 5

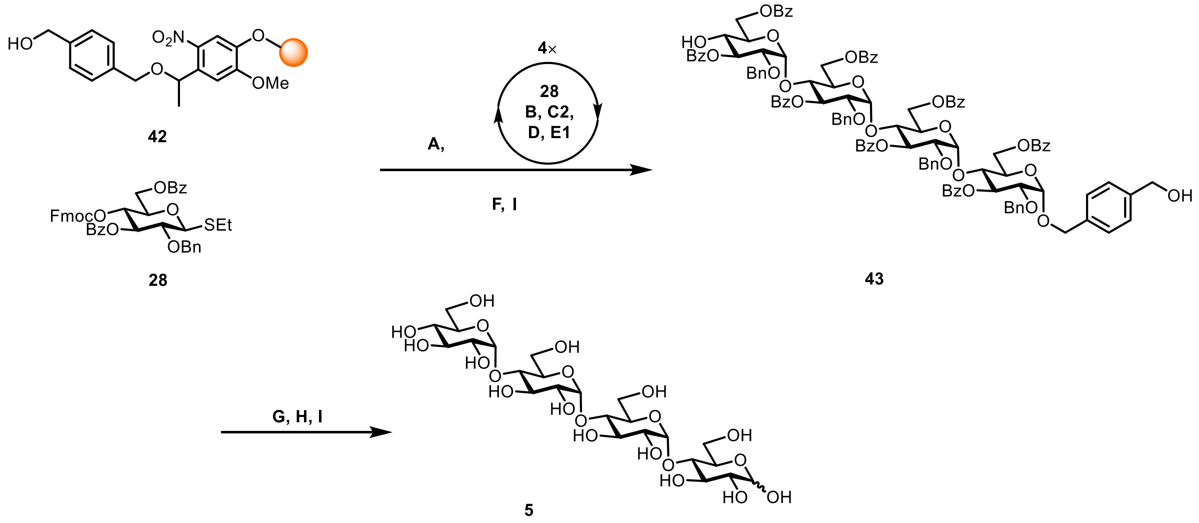

	Module	Conditions
	**A**: Resin Preparation for Synthesis	
	**B**: Acidic Wash with TMSOTf Solution	
4	**C2**: Thioglycoside Glycosylation × 2 Cycle	Building block **28**, 6.5 equiv. (-20°C for 5 min, 0°C for 60 min)
	**D**: Capping	
	**E1**: Fmoc Deprotection	
	**F:** Cleavage from Solid Support	
	**I**: Purification	**Method E and B1**
	**G**: Solution-phase Methanolysis	
	**H**: Hydrogenolysis at Ambient Pressure	
	**I**: Purification	**Method D**

Automated synthesis and purification afforded protected tetramer **43** as a white solid (17 mg, 54%).

Analytical data for **43**: ^{1}H NMR (600 MHz, $CDCl_3$) δ 8.13 – 8.04 (m, 4H), 8.03 – 7.85 (m, 12H), 7.64 – 7.56 (m, 2H), 7.54 – 7.22 (m, 28H), 7.21 – 6.94 (m, 18H), 6.05 (dd, *J* = 9.9, 8.4 Hz, 1H), 5.83 – 5.76 (m, 2H), 5.52 (appt, *J* = 9.5 Hz, 1H), 5.26 (d, *J* = 3.7 Hz, 1H), 5.24 (d, *J* = 3.7 Hz, 1H), 5.15 (d, *J* = 3.5 Hz, 1H), 4.97 (d, *J* = 3.5 Hz, 1H), 4.77 – 4.44 (m, 14H), 4.39 – 4.29 (m, 4H), 4.26 – 4.11 (m, 4H), 4.09 – 3.93 (m, 5H), 3.64 – 3.52 (m, 2H), 3.49 – 3.38 (m, 3H); ^{13}C NMR (151 MHz, $CDCl_3$) δ 167.06, 166.46, 165.99, 165.91, 165.32, 165.05, 164.97, 140.72, 137.61, 137.54, 137.49, 137.37, 136.36, 133.13, 133.09, 132.97, 132.94, 132.91, 132.85,

132.66, 130.34, 130.23, 130.02, 129.94, 129.91, 129.83, 129.81, 129.79, 129.77, 129.72, 129.70, 129.69, 129.62, 129.04, 128.46, 128.44, 128.36, 128.34, 128.28, 128.26, 128.22, 128.18, 128.12, 128.10, 127.84, 127.80, 127.71, 127.56, 127.47, 127.42, 127.11, 97.05, 96.21, 96.15, 94.98, 77.28, 75.91, 75.86, 75.69, 75.11, 74.33, 74.20, 73.97, 73.07, 72.69, 72.31, 72.25, 72.12, 71.34, 69.83, 69.81, 69.20, 68.83, 65.08, 63.41, 63.39, 63.22, 63.18; m/z (HRMS$^+$) $[M + NH_4]^+$ 1996.722 ($C_{116}H_{110}NO_{30}^+$ requires 1996.710).

NP-HPLC of crude 43 after AGA (ELSD trace, Method A1)

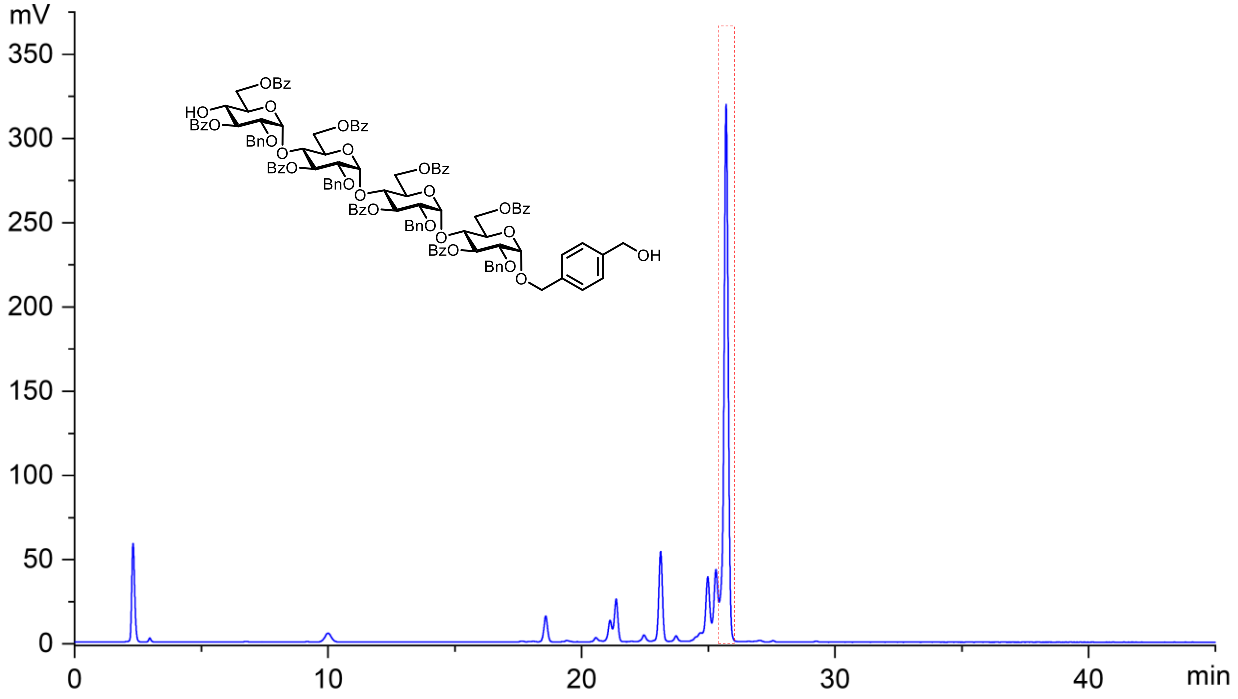

NP-HPLC of pure 43 (ELSD trace, Method A1, t_R = 27.4 min)

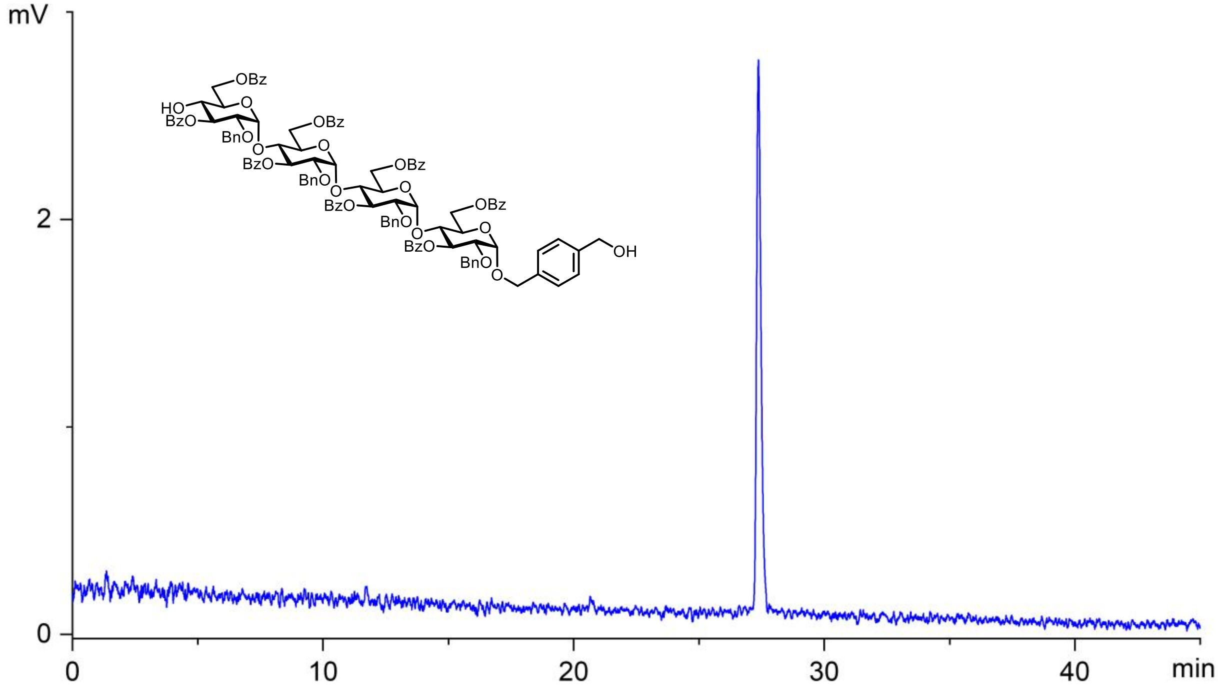

MALDI spectrum of 43

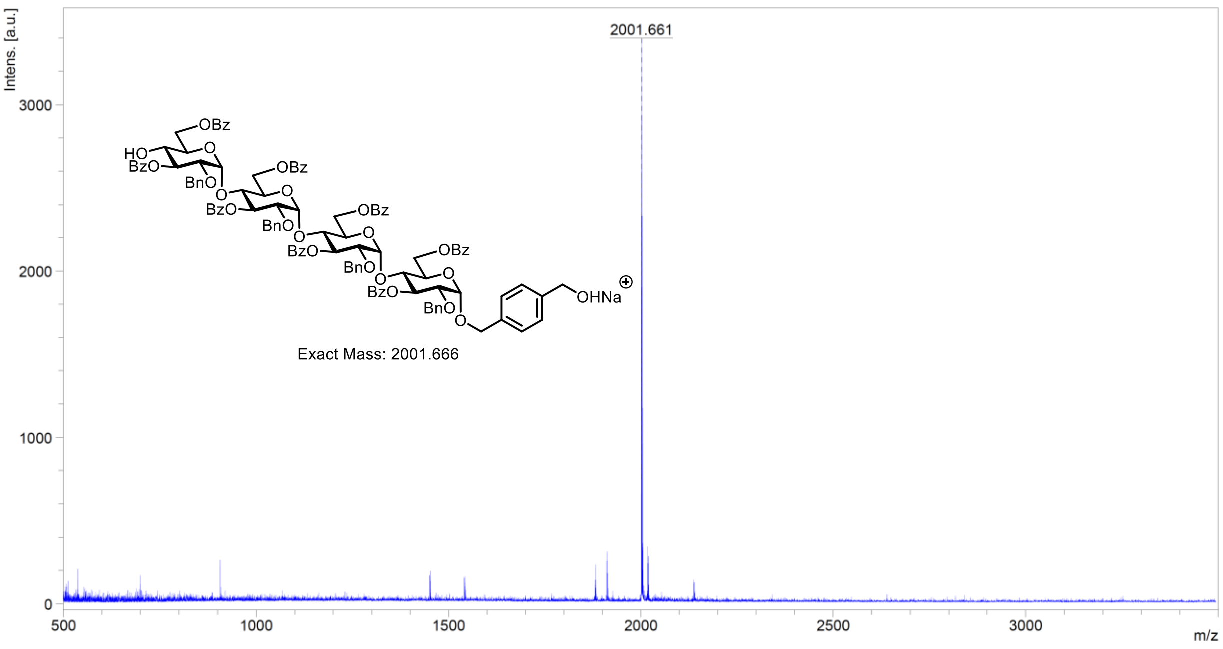

^{1}H NMR of 43 (600 MHz, $CDCl_3$)

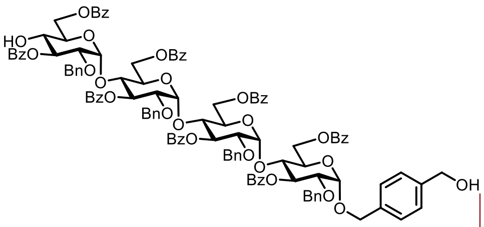

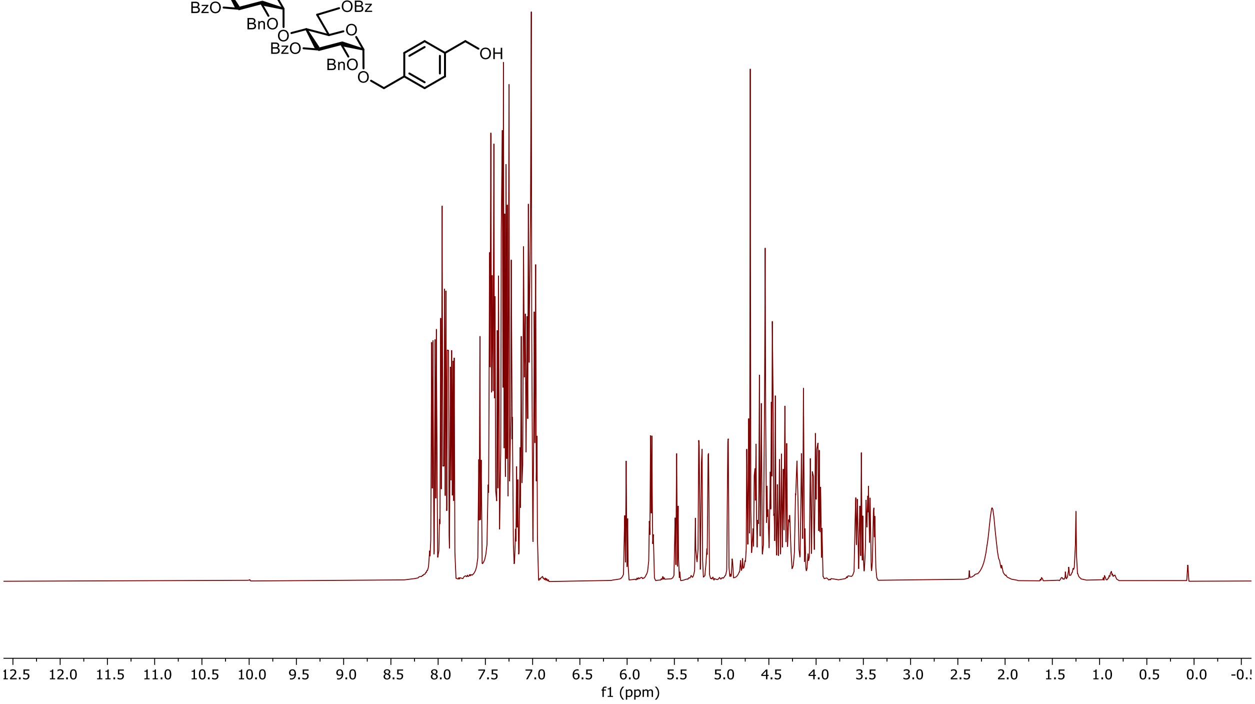

^{13}C NMR of 43 (151 MHz, $CDCl_3$)

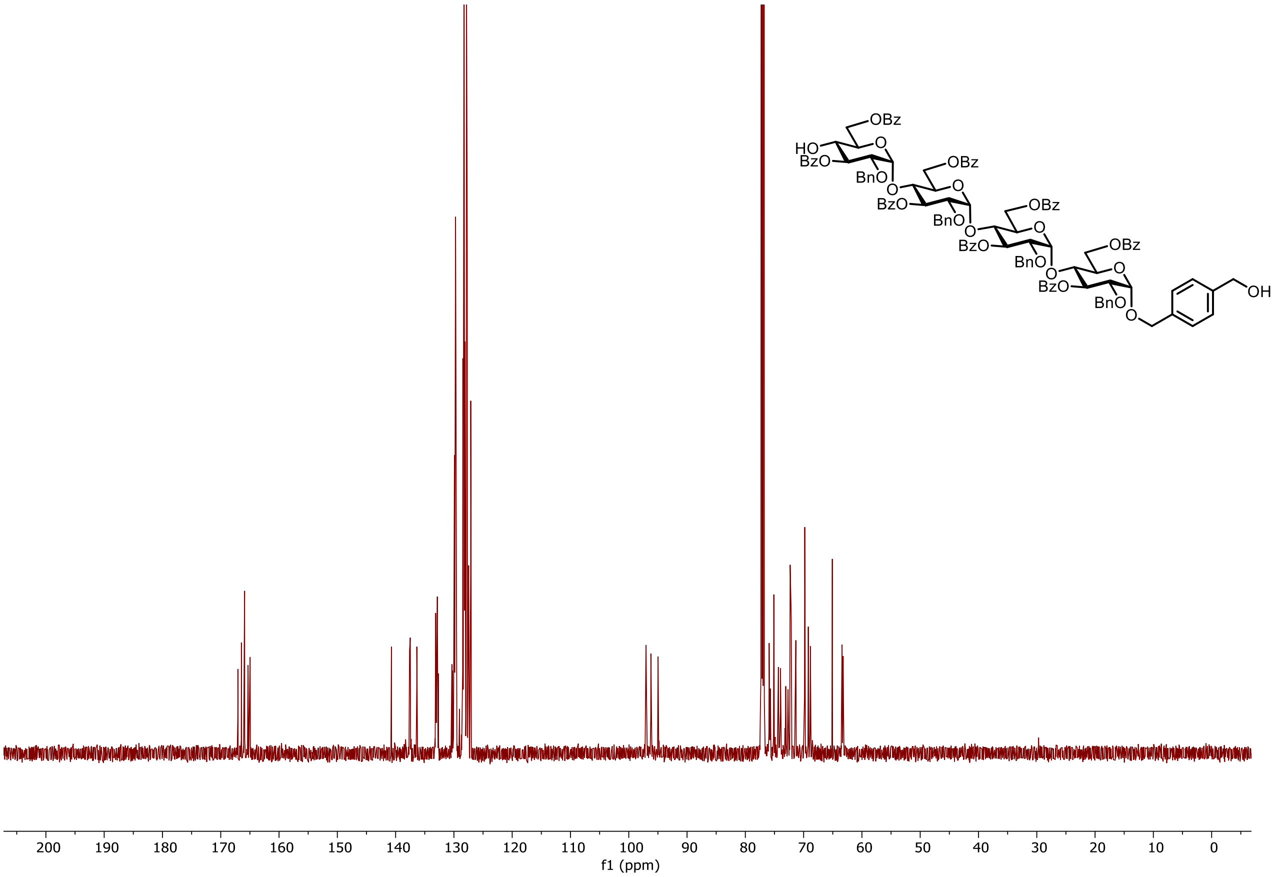

HSQC NMR of 43 ($CDCl_3$)

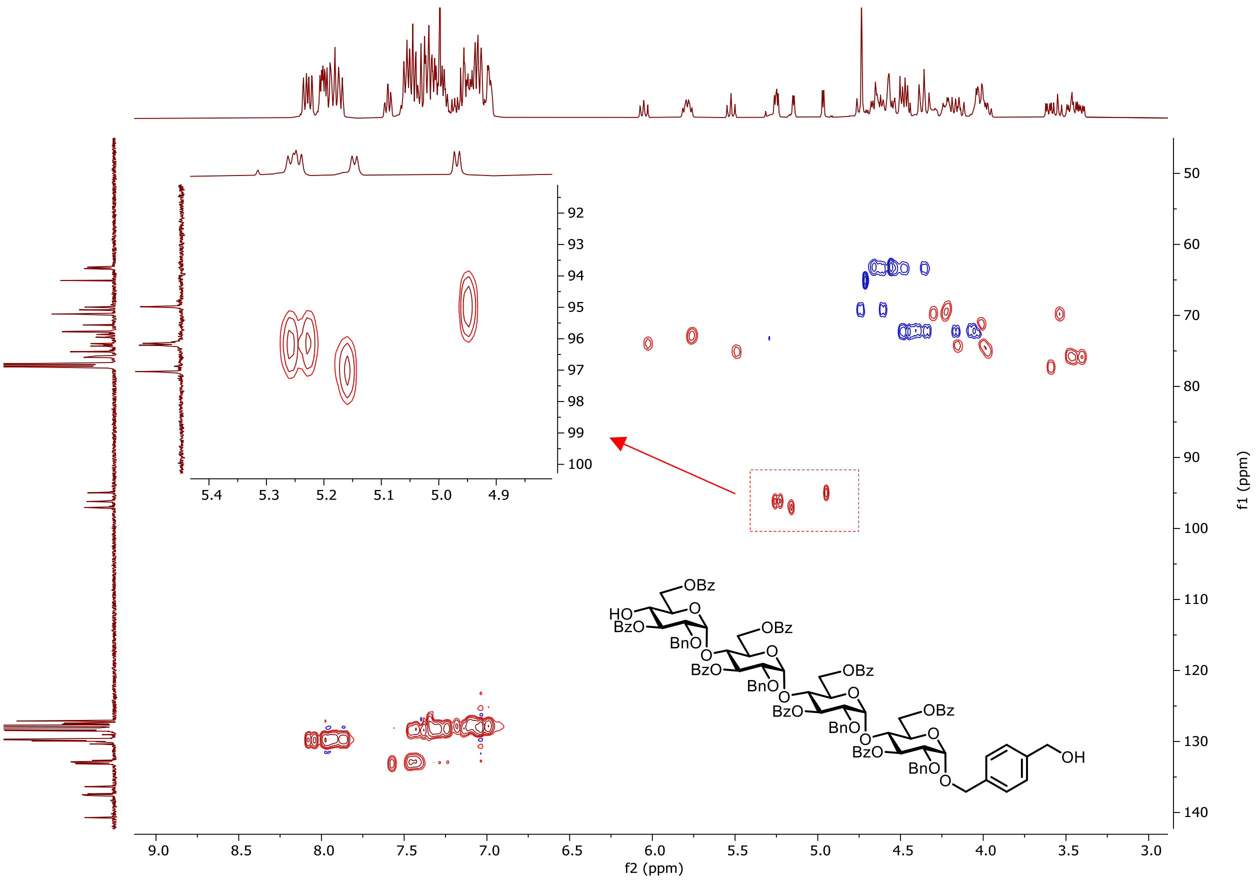

Coupled HSQC NMR of 43 ($CDCl_3$)

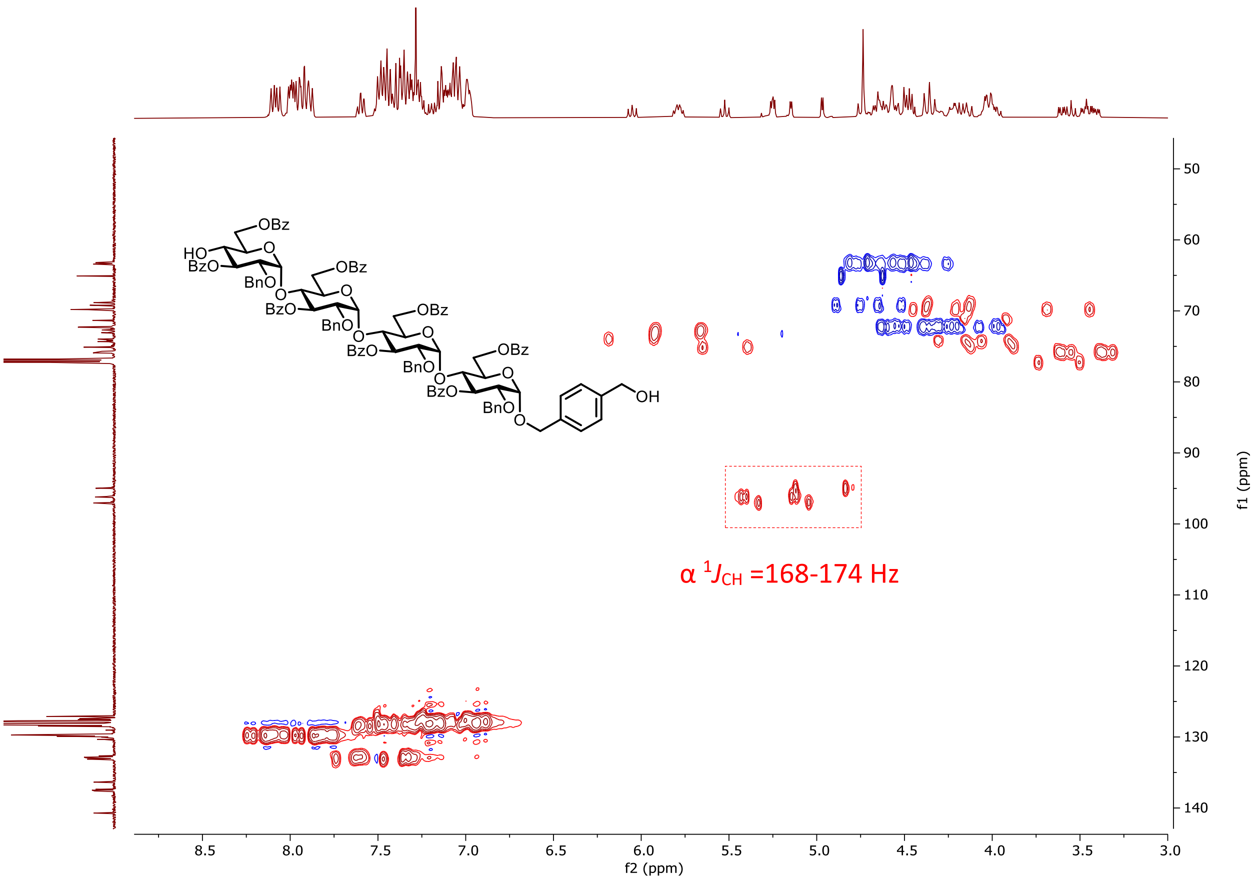

Global deprotection and purification afforded deprotected tetramer **5** as a white solid (3.2 mg, 30% overall).

Analytical data for **5**: ^{1}H NMR (600 MHz, D_2O) δ 4.45 – 4.37 (m, 3H, 3×α(1→4) anomeric H), 5.24 (d, *J* = 3.8 Hz, 0.32H, α-H1), 4.67 (d, *J* = 8.0 Hz, 0.68H, β-H1), 4.04 – 3.56 (m, 22.32H), 3.46 – 3.40 (m, 1H), 3.29 (dd, *J* = 9.5, 8.0 Hz, 0.68H); ^{13}C NMR (151 MHz, D_2O) δ 99.65, 99.55, 99.39, 99.31, 95.69 (β-C1), 91.82 (α-C1), 76.89, 76.86, 76.83, 76.68, 76.60, 76.11, 74.46, 73.92, 73.27, 73.22, 73.20, 73.13, 72.78, 72.63, 71.65, 71.48, 71.38, 71.22, 71.13, 71.08, 71.06, 69.86, 69.24, 60.60, 60.46, 60.39, 60.33; m/z (HRMS^{+}) [M + Na]$^{+}$ 689.2151 ($C_{24}H_{42}O_{21}Na^{+}$ requires 689.2111).

RP-HPLC of 5 (ELSD trace, Method C, t_R = 3.0 min)

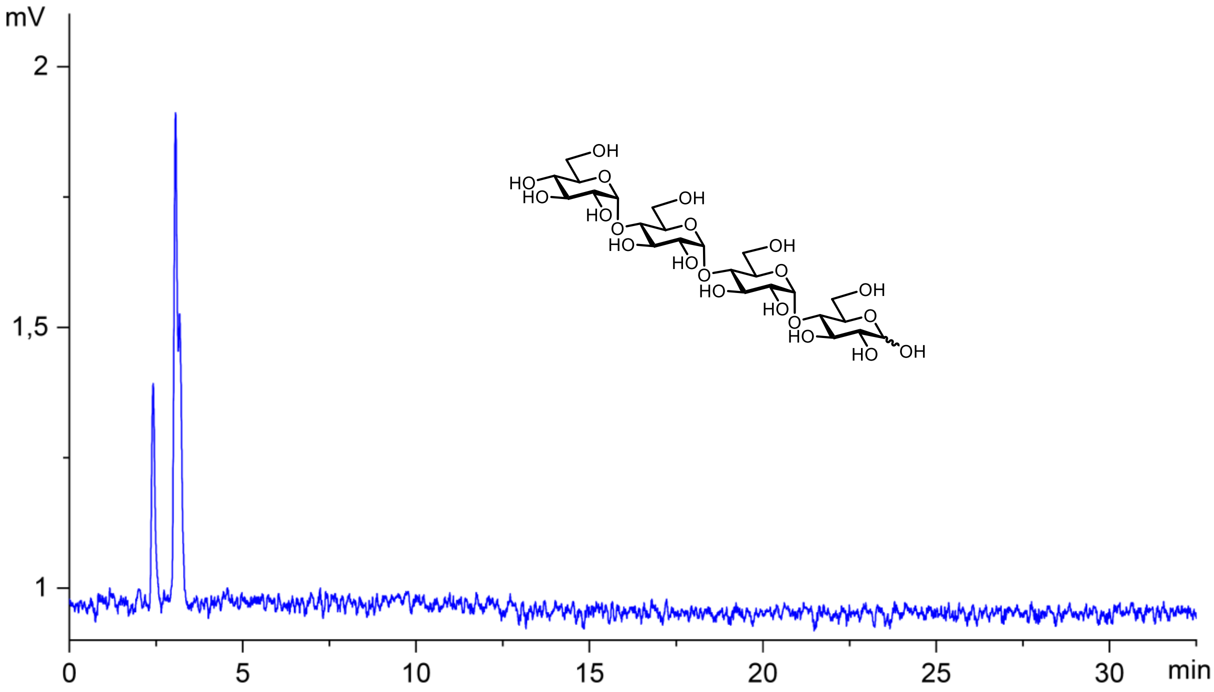

MALDI spectrum of 5

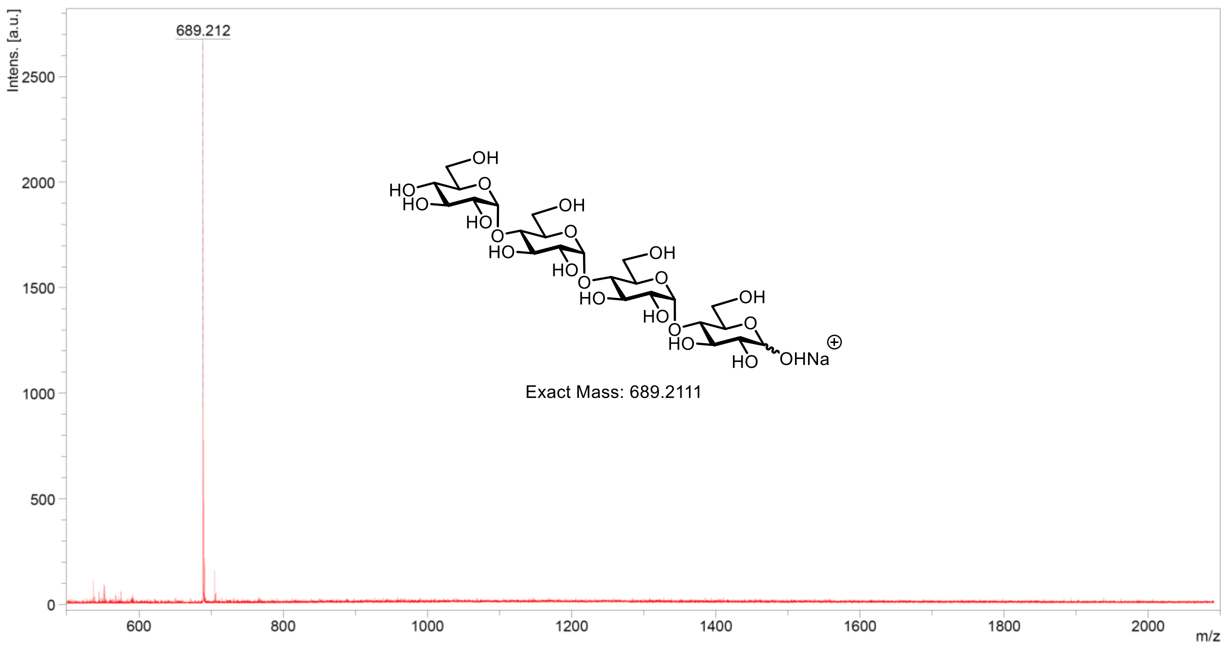

^1H NMR of 5 (600 MHz, D_2O)

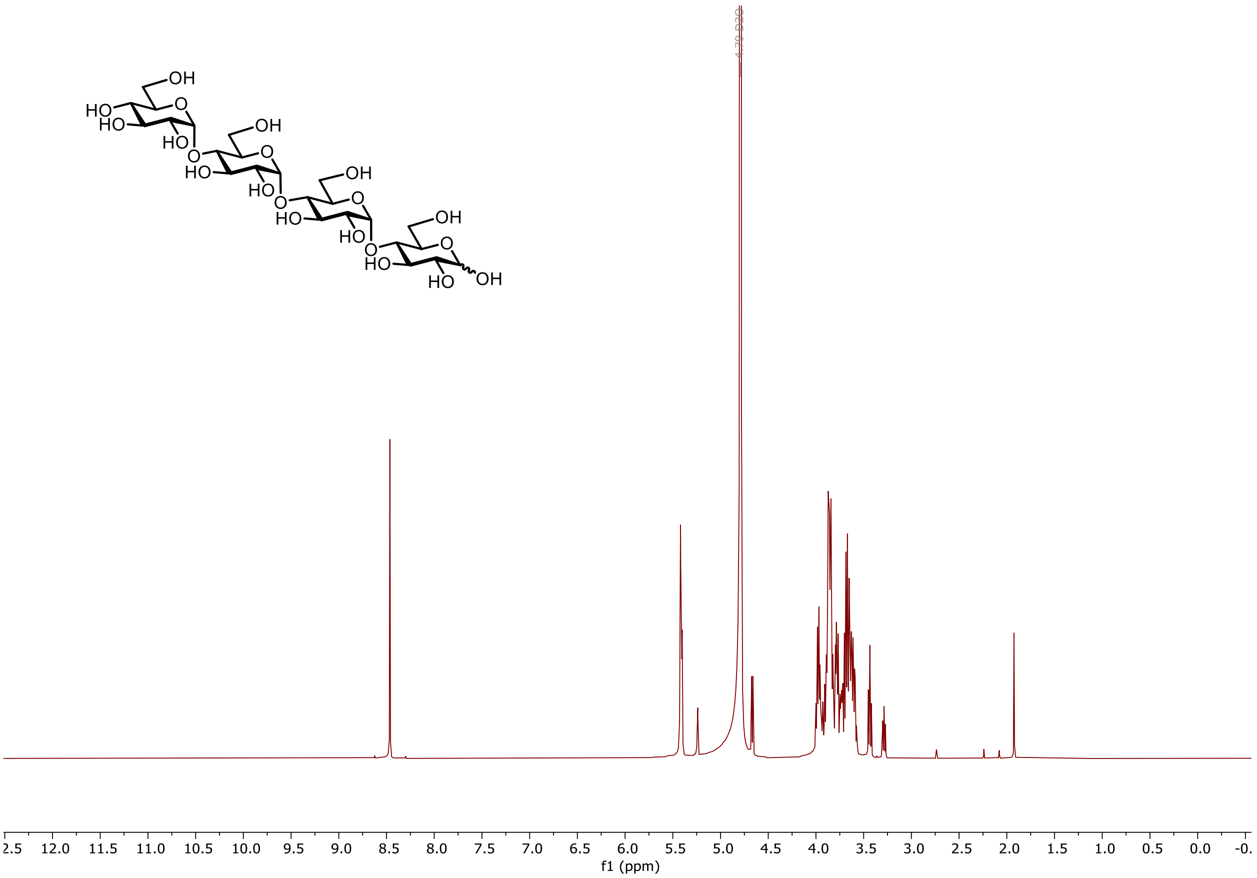

^{13}C NMR of 5 (151 MHz, D_2O)

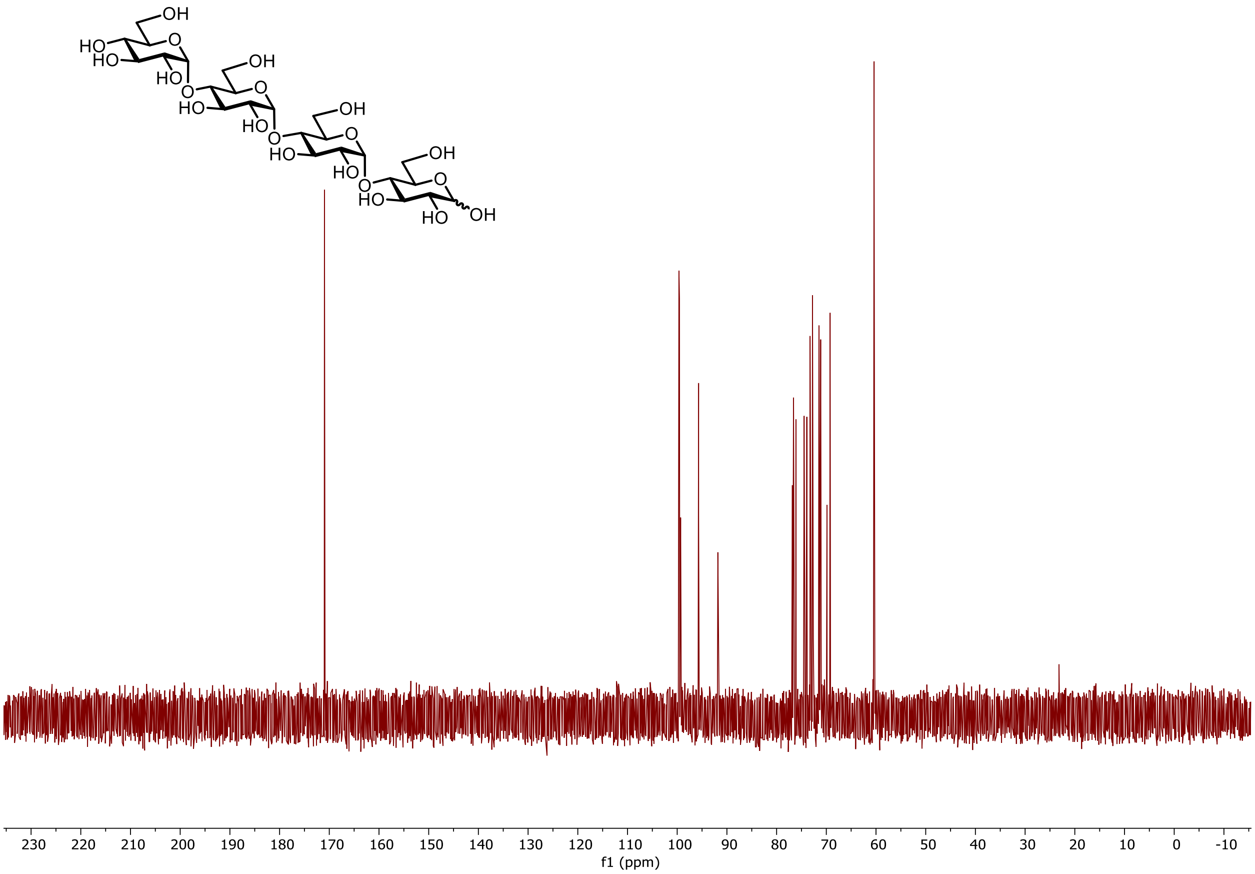

HSQC NMR of 5 (D_2O)

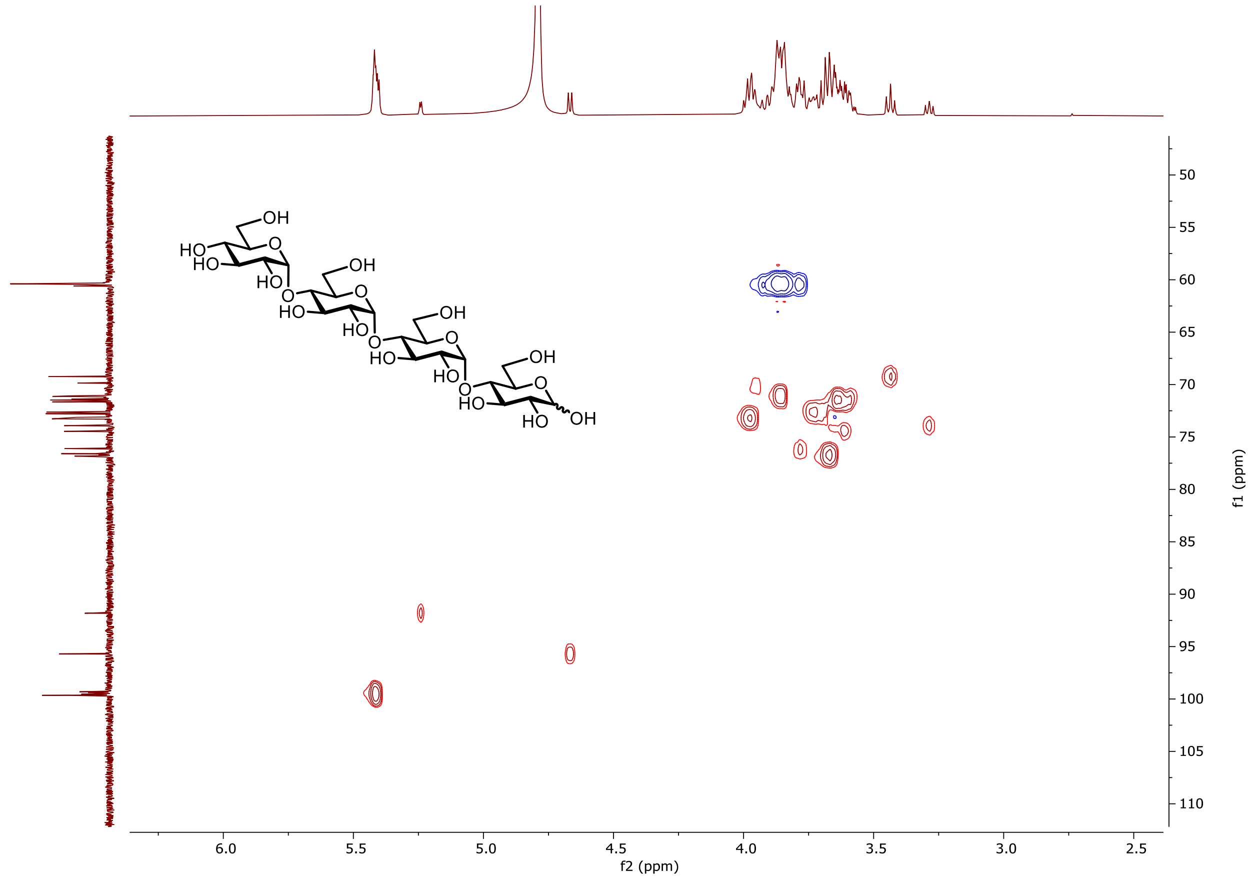

Coupled HSQC NMR of 5 (D_2O)

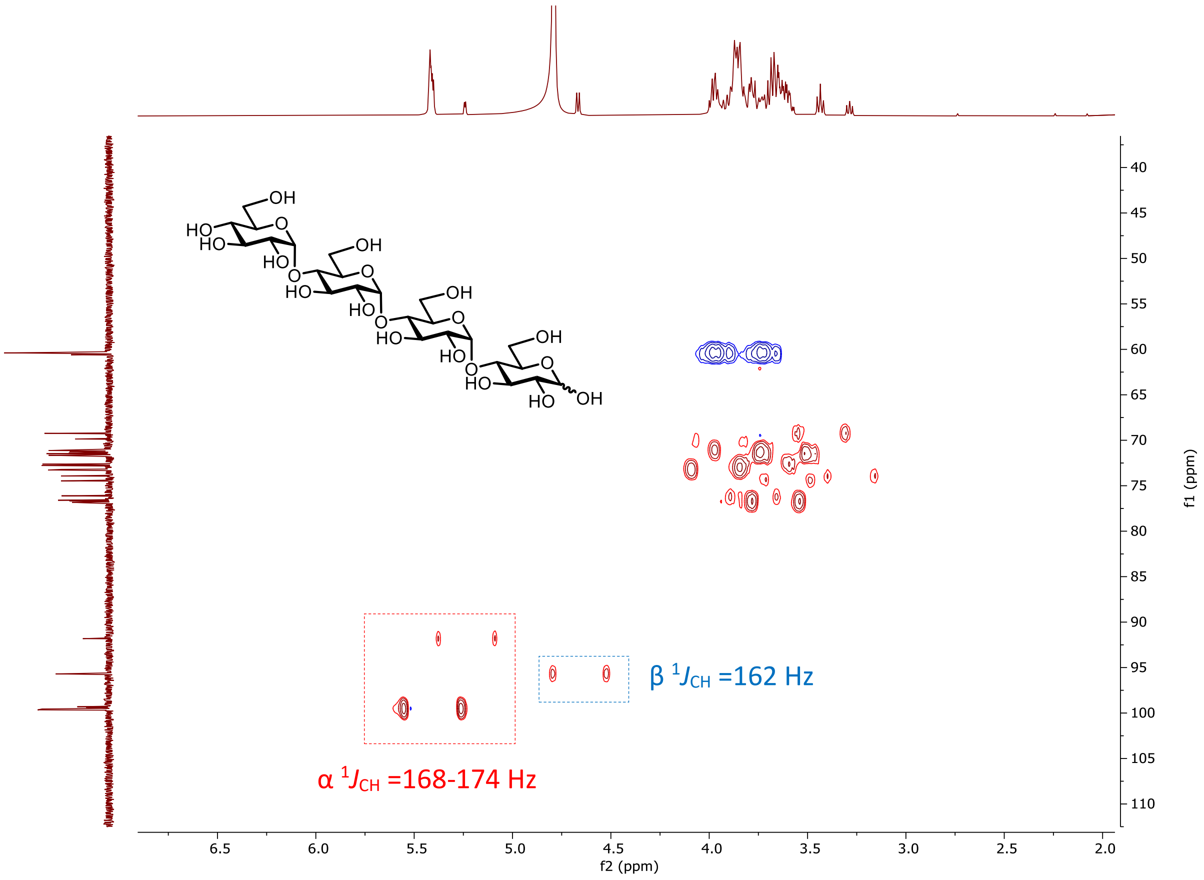

7.2 Synthesis of amylose octamer 6

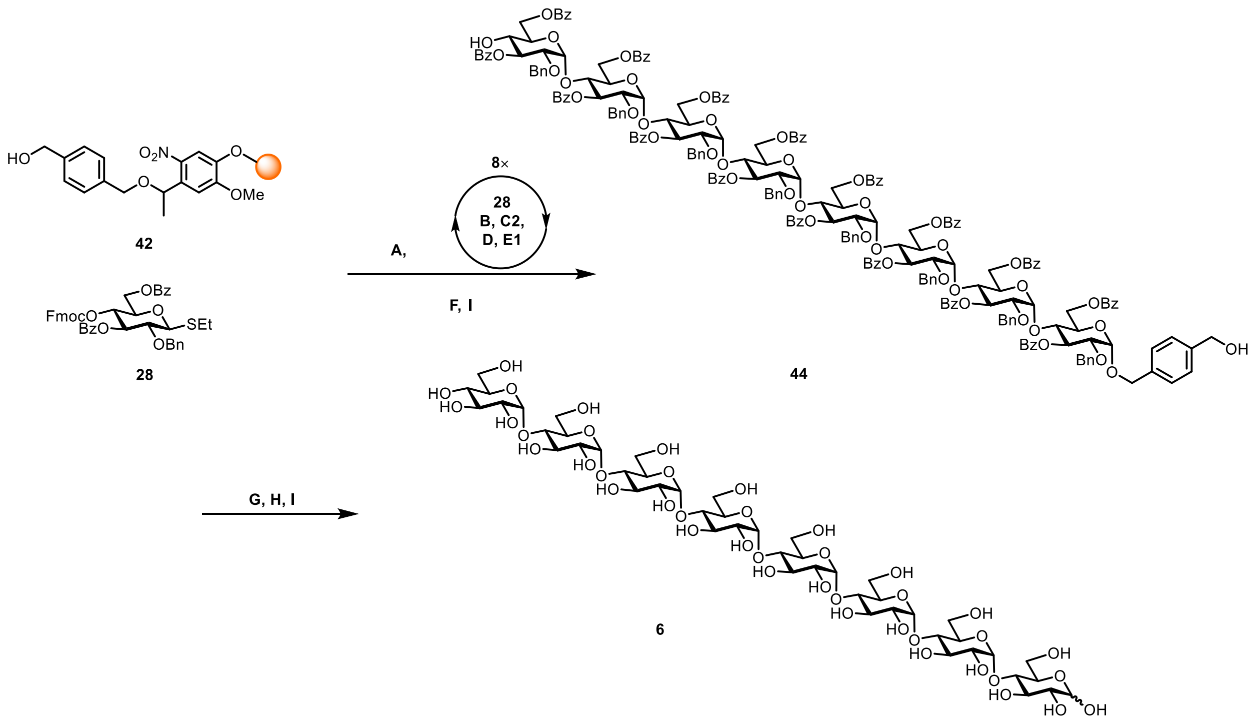

	Module	Conditions
	**A**: Resin Preparation for Synthesis	
8	**B**: Acidic Wash with TMSOTf Solution	
	**C2**: Thioglycoside Glycosylation × 2 Cycle	Building block **28**, 6.5 equiv. (-20°C for 5 min, 0°C for 60 min)
	**D**: Capping	
	**E1**: Fmoc Deprotection	
	**F:** Cleavage from Solid Support	
	**I**: Purification	**Method E and B1**
	**G**: Solution-phase Methanolysis	
	**H**: Hydrogenolysis at Ambient Pressure	
	**I**: Purification	**Method D**

Automated synthesis and purification afforded protected octamer **44** as a white solid (28 mg, 45%).

Analytical data for **44**: ^{1}H NMR (600 MHz, $CDCl_3$) δ 8.07 (d, *J* = 7.7 Hz, 2H), 8.02 (d, *J* = 7.7 Hz, 2H), 7.99 (d, *J* = 7.7 Hz, 2H), 7.89 (m, 26H), 7.55 (dt, *J* = 12.0, 7.4 Hz, 2H), 7.49 – 7.14 (m, 53H), 7.12 – 6.91 (m, 37H),

5.99 (appt, J = 9.3 Hz, 1H), 5.78 – 5.68 (m, 6H), 5.46 (appt, J = 9.5 Hz, 1H), 5.27 (d, J = 3.8 Hz, 3H), 5.25 (d, J = 3.9 Hz, 1H), 5.22 (d, J = 3.8 Hz, 1H), 5.21 (d, J = 3.9 Hz, 1H), 5.13 (d, J = 3.5 Hz, 1H), 4.90 (d, J = 3.5 Hz, 1H), 4.73 (d, J = 12.4 Hz, 1H), 4.67 (d, J = 9.2 Hz, 4H), 4.65 – 4.60 (m, 1H), 4.59 – 4.41 (m, 20H), 4.40 – 4.34 (m, 2H), 4.34 – 4.25 (m, 2H), 4.24 – 4.09 (m, 11H), 4.06 (d, J = 12.5 Hz, 1H), 4.03 – 3.91 (m, 9H), 3.54 – 3.48 (m, 2H), 3.47 – 3.40 (m, 3H), 3.39 (dd, J = 8.7, 3.7 Hz, 1H), 3.34 (dd, J = 8.6, 3.7 Hz, 2H), 3.30 (dd, J = 8.9, 3.7 Hz, 1H); ^{13}C NMR (151 MHz, $CDCl_3$) δ 167.04, 166.44, 165.97, 165.91, 165.87, 165.76, 165.72, 165.70, 165.67, 165.32, 165.03, 165.00, 164.99, 164.96, 164.94, 140.68, 137.61, 137.54, 137.43, 137.42, 137.40, 137.38, 137.34, 136.35, 133.12, 133.08, 132.92, 132.88, 132.84, 132.80, 132.65, 130.29, 130.25, 129.98, 129.91, 129.85, 129.80, 129.78, 129.73, 129.68, 129.66, 129.64, 129.61, 128.50, 128.46, 128.33, 128.32, 128.26, 128.24, 128.17, 128.12, 128.08, 127.93, 127.92, 127.89, 127.85, 127.84, 127.80, 127.73, 127.70, 127.55, 127.46, 127.43, 127.41, 127.39, 127.07, 96.92, 95.99, 95.85, 95.81, 95.72, 95.65, 94.83, 77.35, 76.06, 75.91, 75.85, 75.74, 75.67, 75.13, 74.96, 74.09, 73.98, 73.79, 73.59, 73.49, 73.17, 73.08, 72.72, 72.33, 72.28, 72.24, 72.07, 71.33, 69.88, 69.80, 69.75, 69.71, 69.64, 69.59, 69.53, 69.12, 68.81, 65.10, 63.37, 63.14, 63.07, 63.06, 63.01; m/z (HRMS$^+$) [M + NH_4]$^+$ 3837.585 ($C_{224}H_{206}NO_{58}^+$ requires 3837.319).

NP-HPLC of crude 44 after AGA (ELSD trace, Method A1)

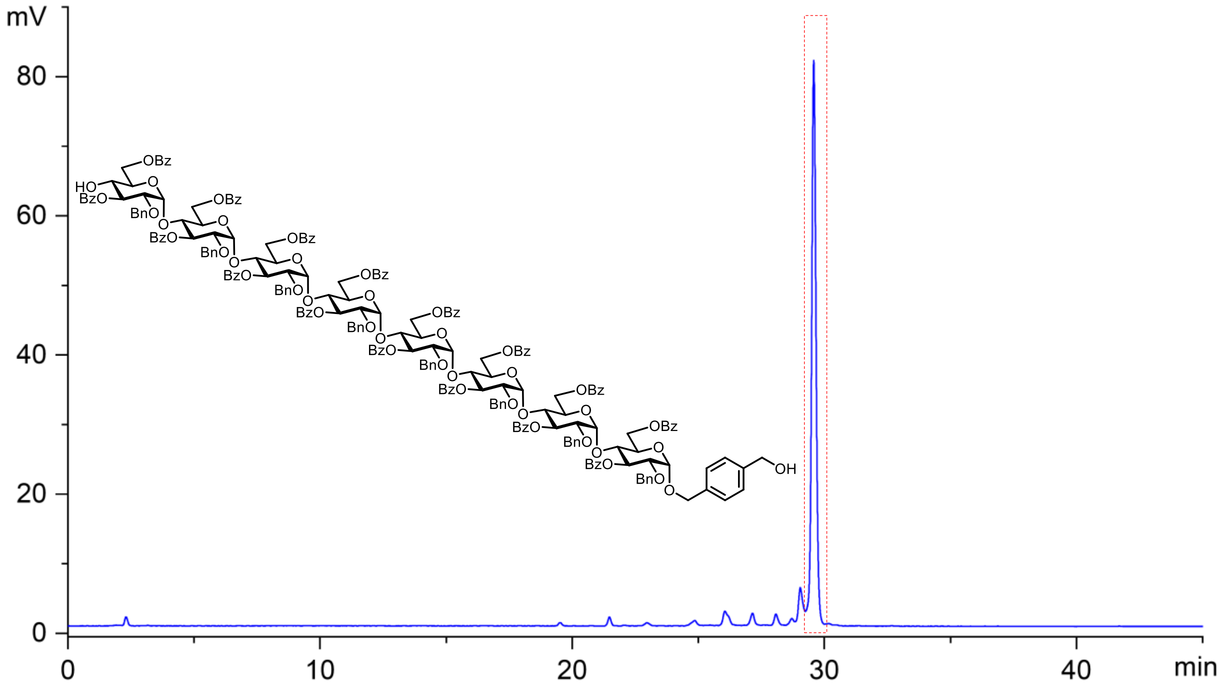

NP-HPLC of pure 44 (ELSD trace, Method A1, t_R = 30.9 min)

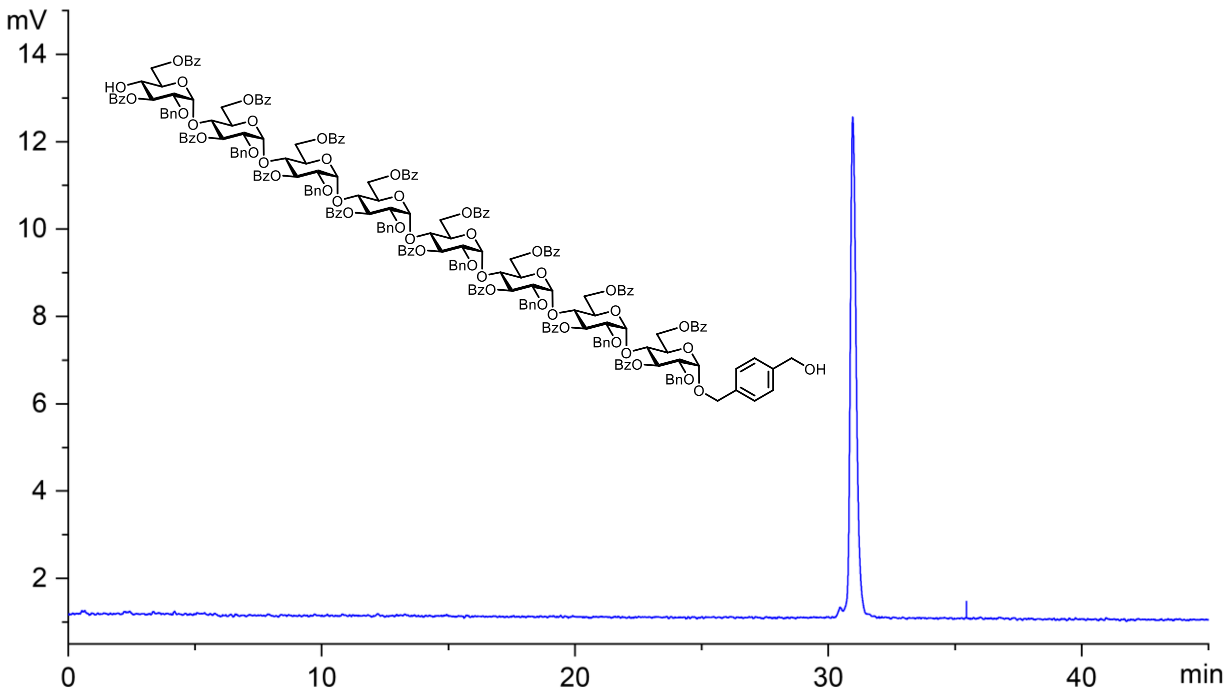

MALDI spectrum of 44

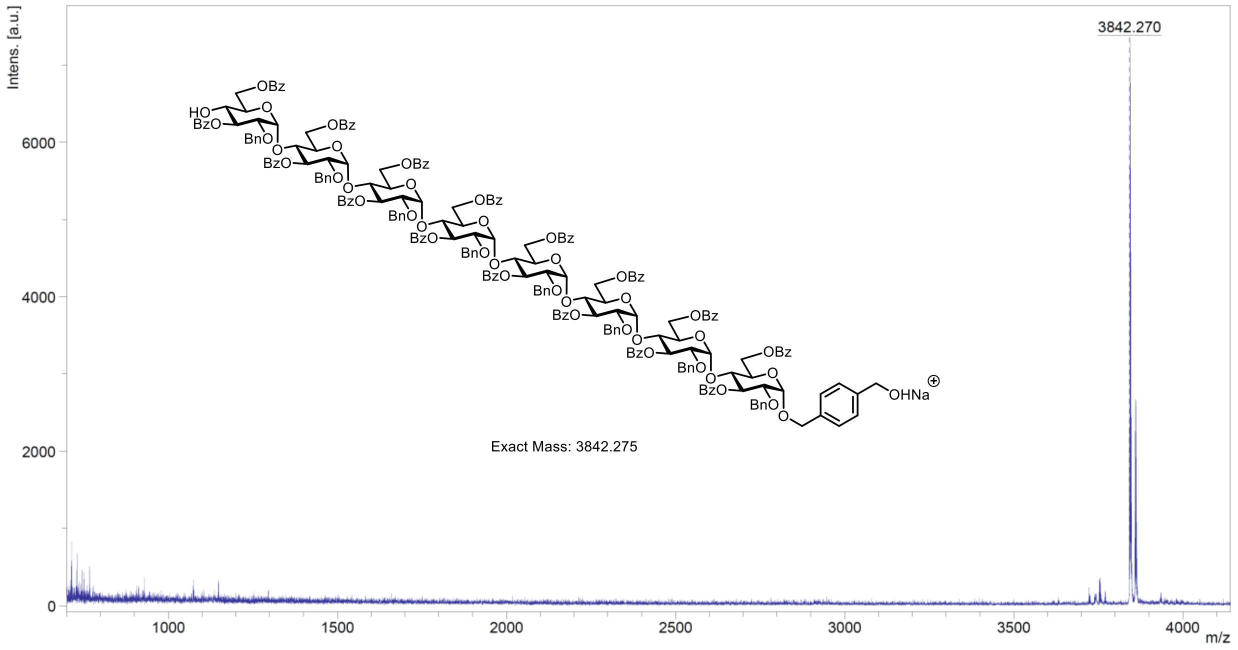

^{1}H NMR of 44 (600 MHz, $CDCl_3$)

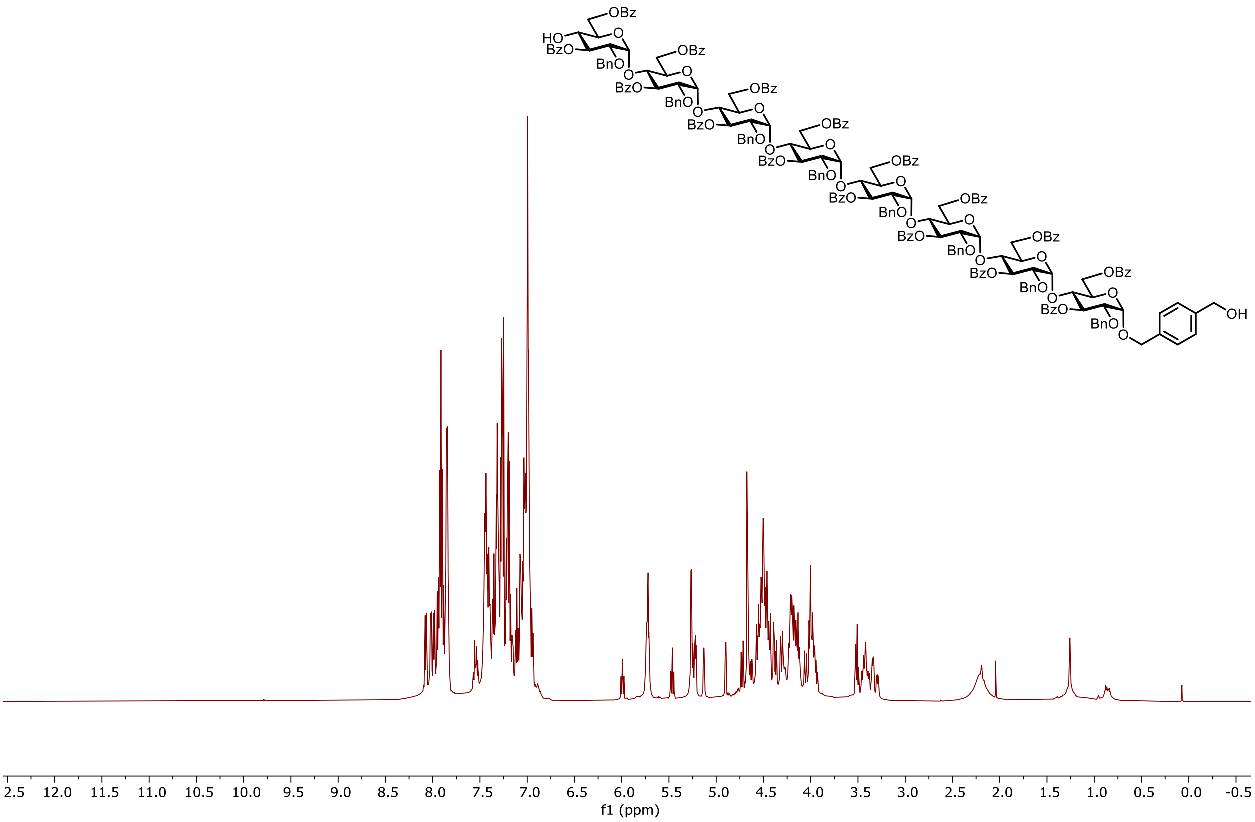

^{13}C NMR of 44 (151 MHz, $CDCl_3$)

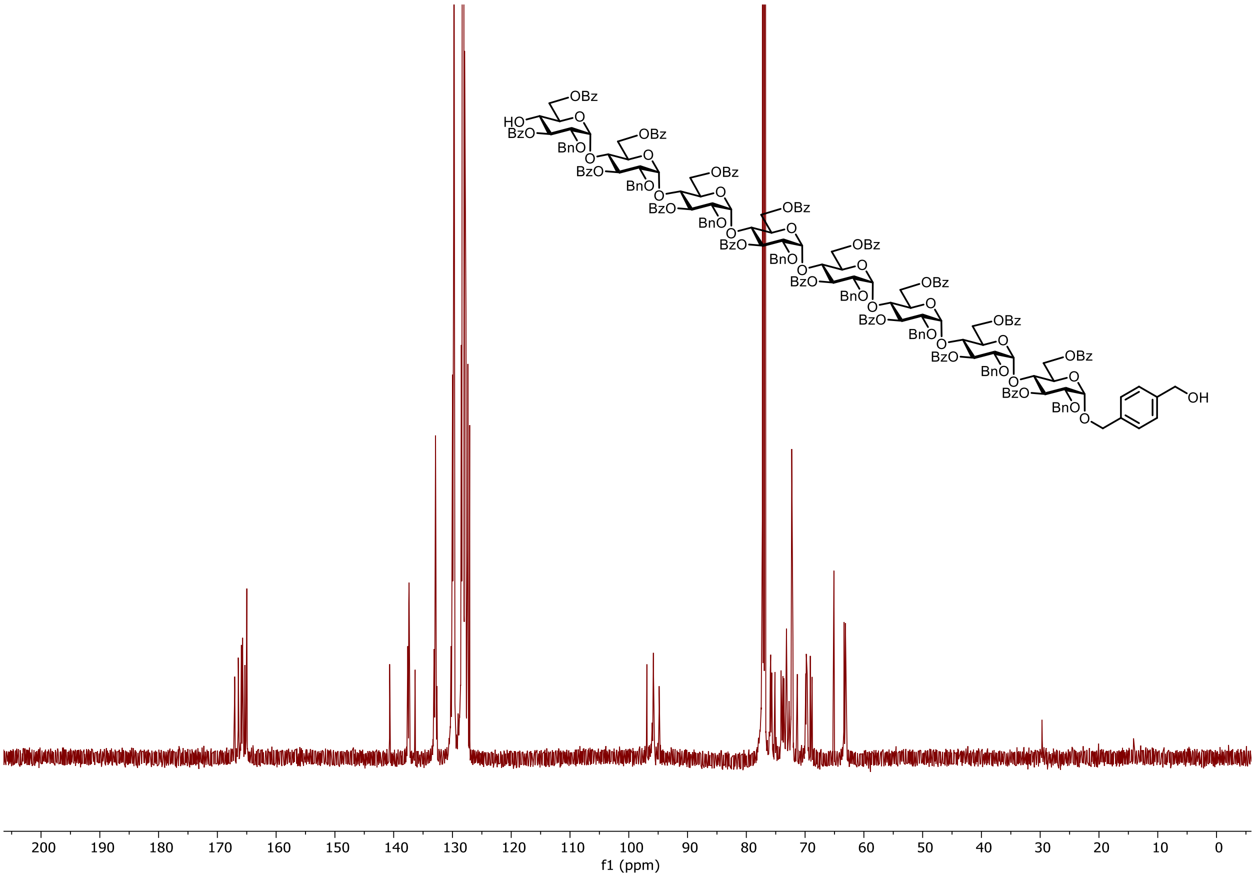

HSQC NMR of 44 ($CDCl_3$)

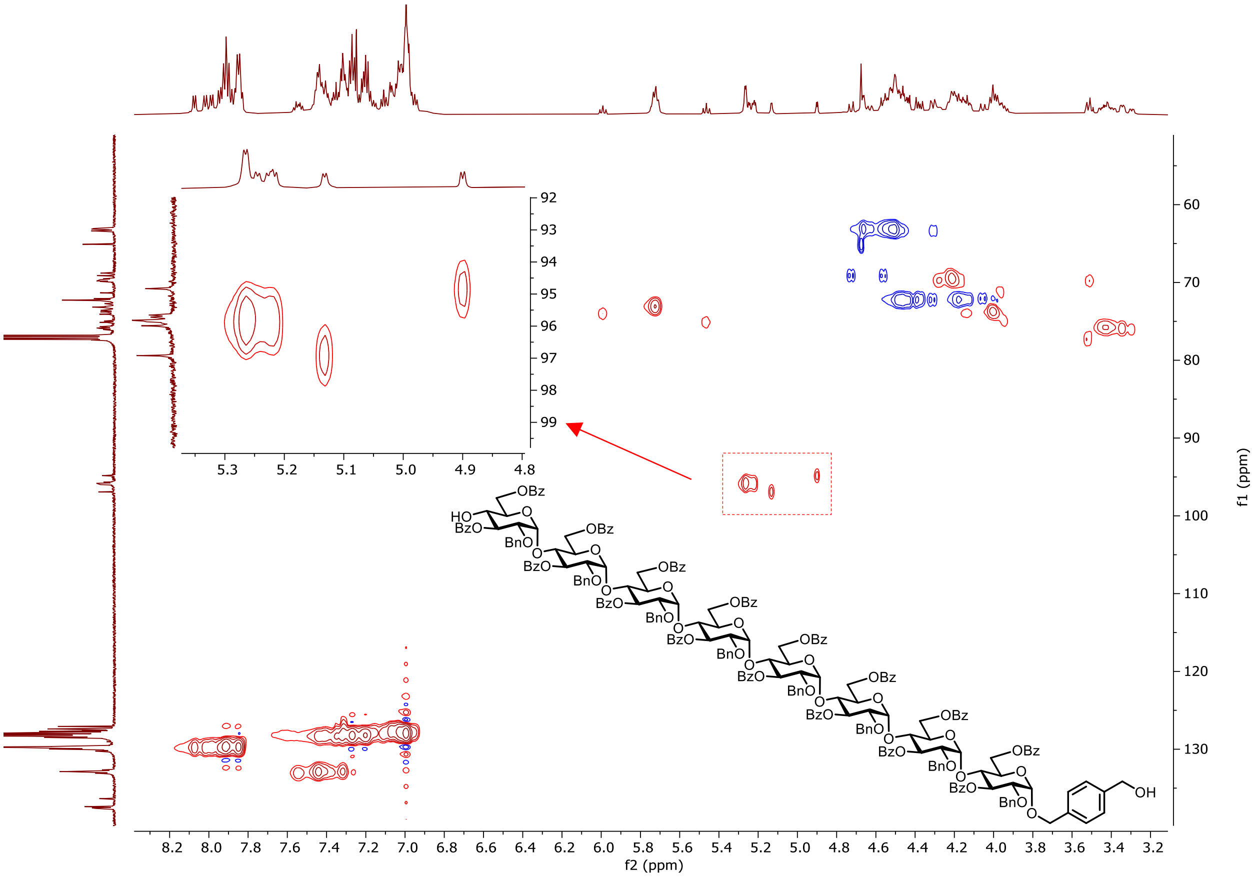

Coupled HSQC NMR of 44 ($CDCl_3$)

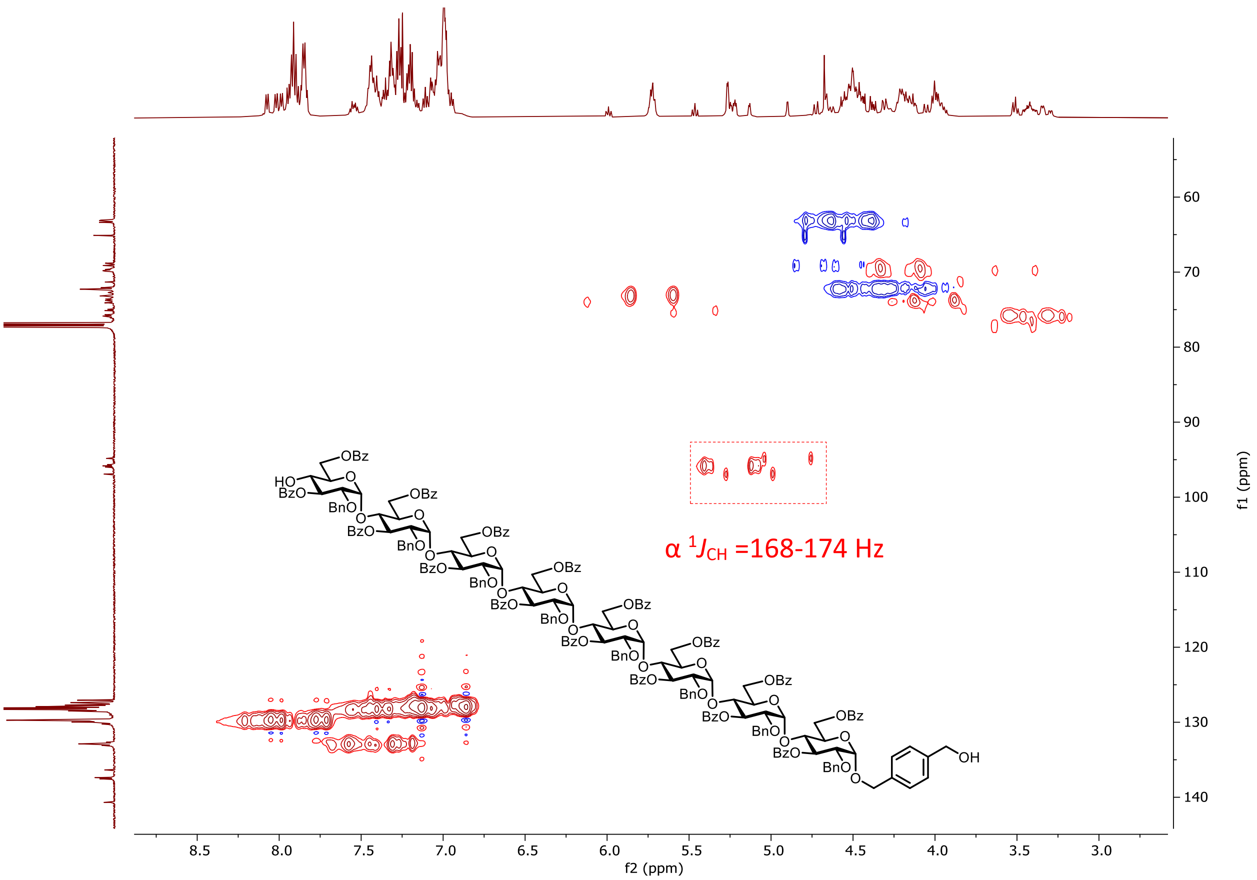

Global deprotection and purification afforded deprotected octamer **6** as a white solid (6.4 mg, 30% overall).

Analytical data for **6**: ^{1}H NMR (600 MHz, D_2O) δ 5.45 – 5.37 (m, 7H, 7×α(1→4) anomeric H), 5.24 (d, *J* = 3.9 Hz, 0.34H, α-H1), 4.67 (d, *J* = 7.9 Hz, 0.66H, β-H1), 4.01 – 3.56 (m, 46.34H), 3.46 – 3.40 (m, 1H), 3.29 (dd, *J* = 9.5, 8.0 Hz, 0.66H); ^{13}C NMR (151 MHz, D_2O) δ 99.65, 99.52, 99.49, 99.47, 99.39, 99.30, 95.70 (β-C1), 91.83 (α-C1), 76.88, 76.75, 76.71, 76.68, 76.65, 76.62, 76.11, 74.46, 73.93, 73.27, 73.25, 73.23, 73.13, 72.79, 72.63, 71.65, 71.49, 71.46, 71.44, 71.39, 71.22, 71.13, 71.09, 71.05, 69.86, 69.24, 60.61, 60.47, 60.39, 60.33; m/z (HRMS$^+$) [M + Na]$^+$ 1337.312 ($C_{48}H_{82}O_{41}Na^+$ requires 1337.422).

RP-HPLC of 6 (ELSD trace, Method C, t_R = 4.7 min)

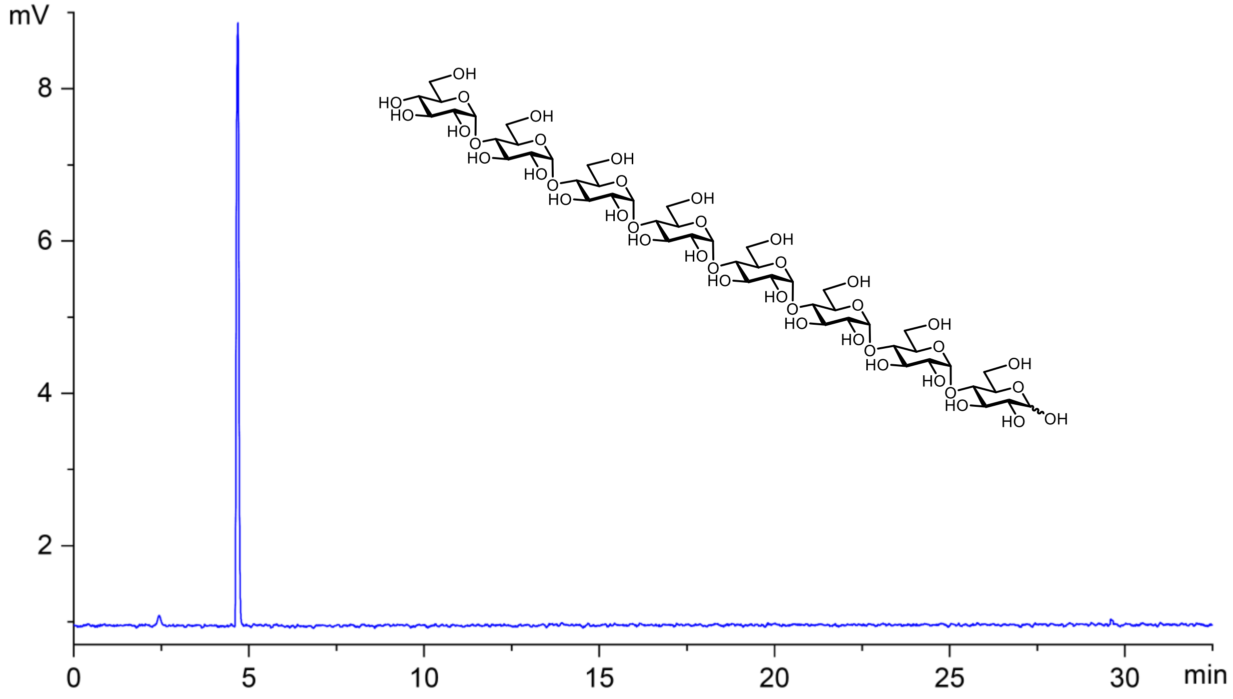

MALDI spectrum of 6

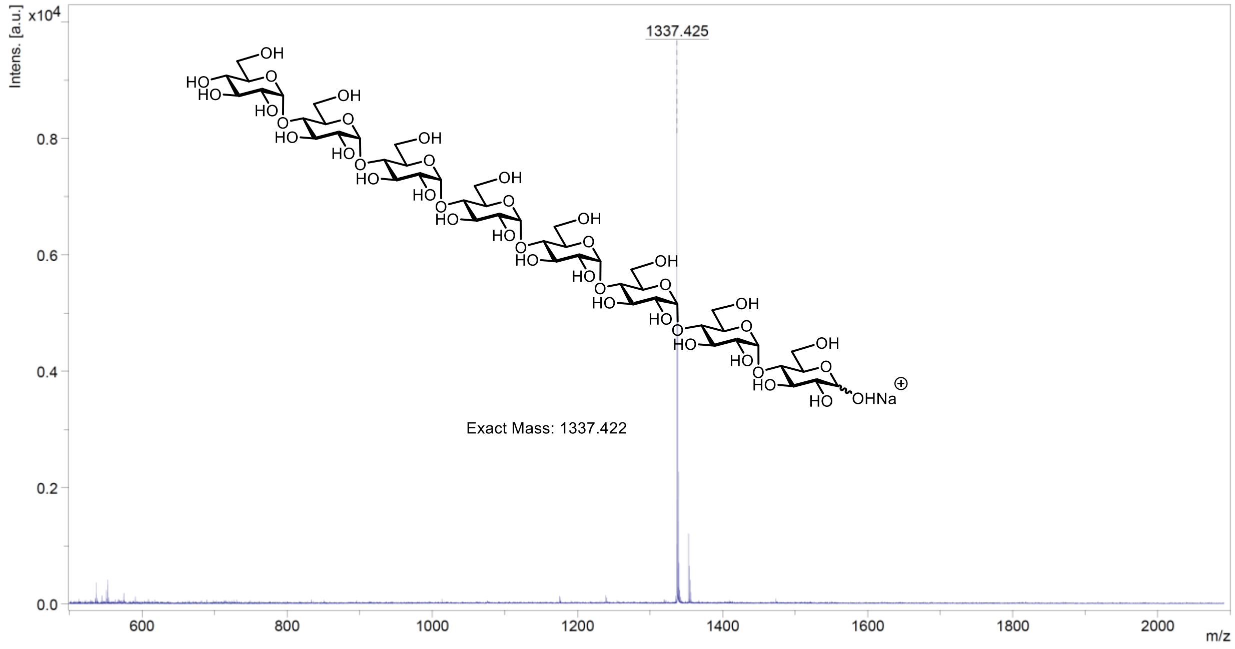

^{1}H NMR of 6 (600 MHz, D_2O)

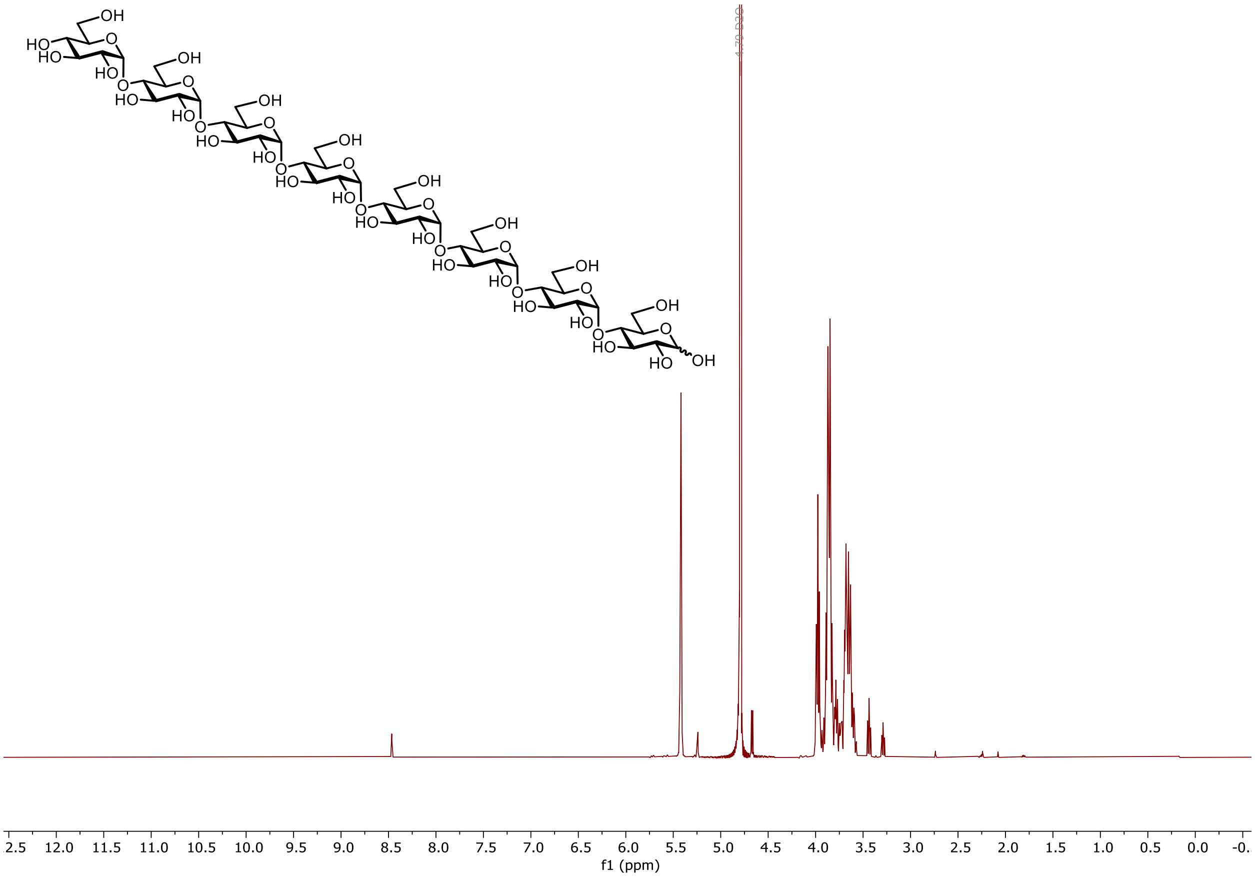

^{13}C NMR of 6 (151 MHz, D_2O)

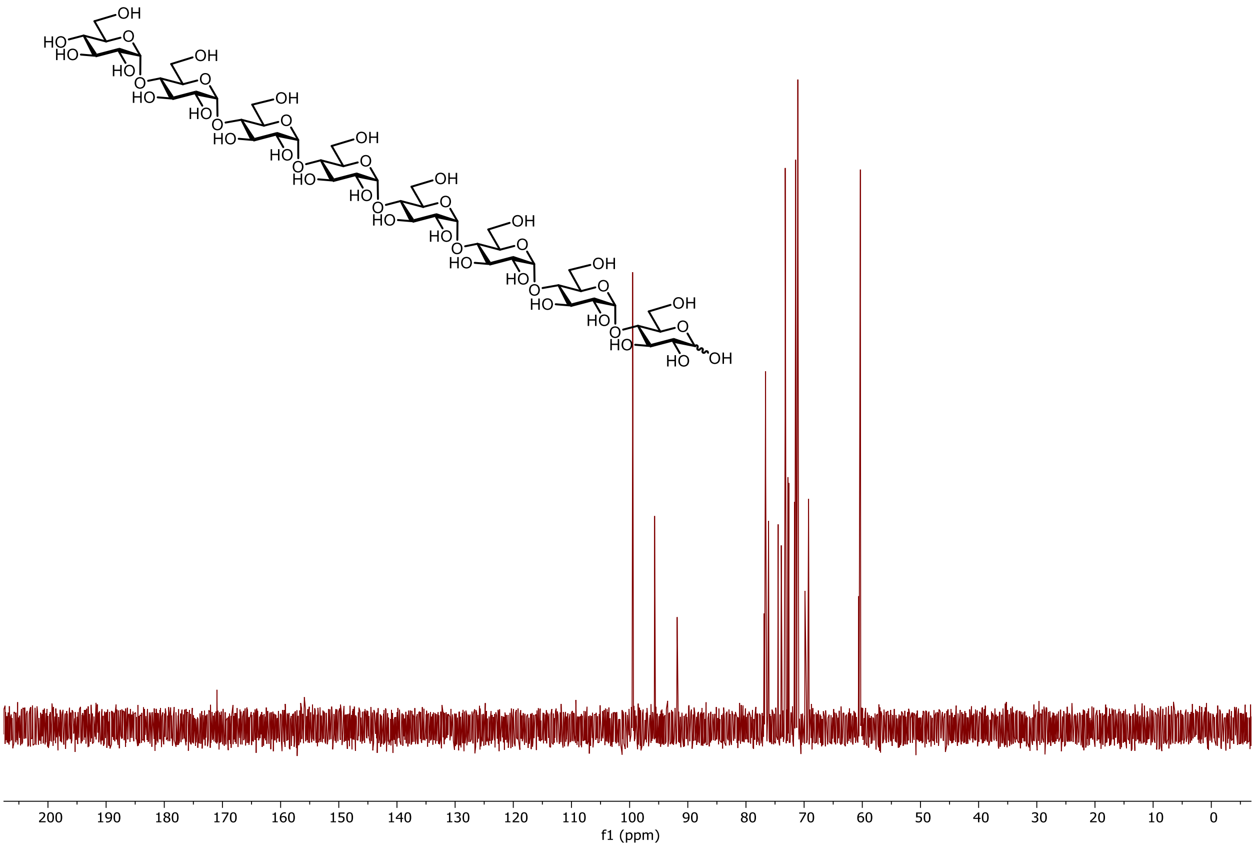

HSQC NMR of 6 (D_2O)

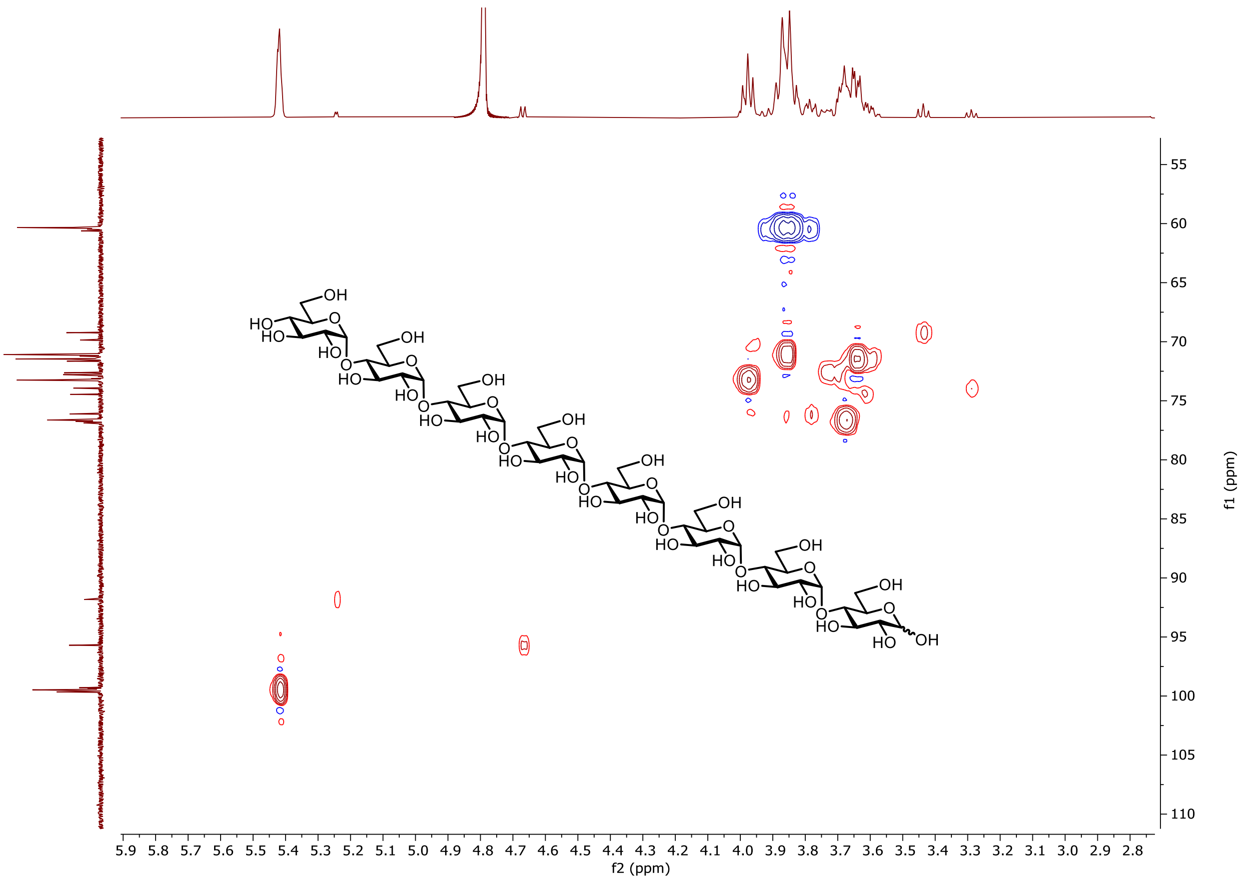

Coupled HSQC NMR of 6 (D_2O)

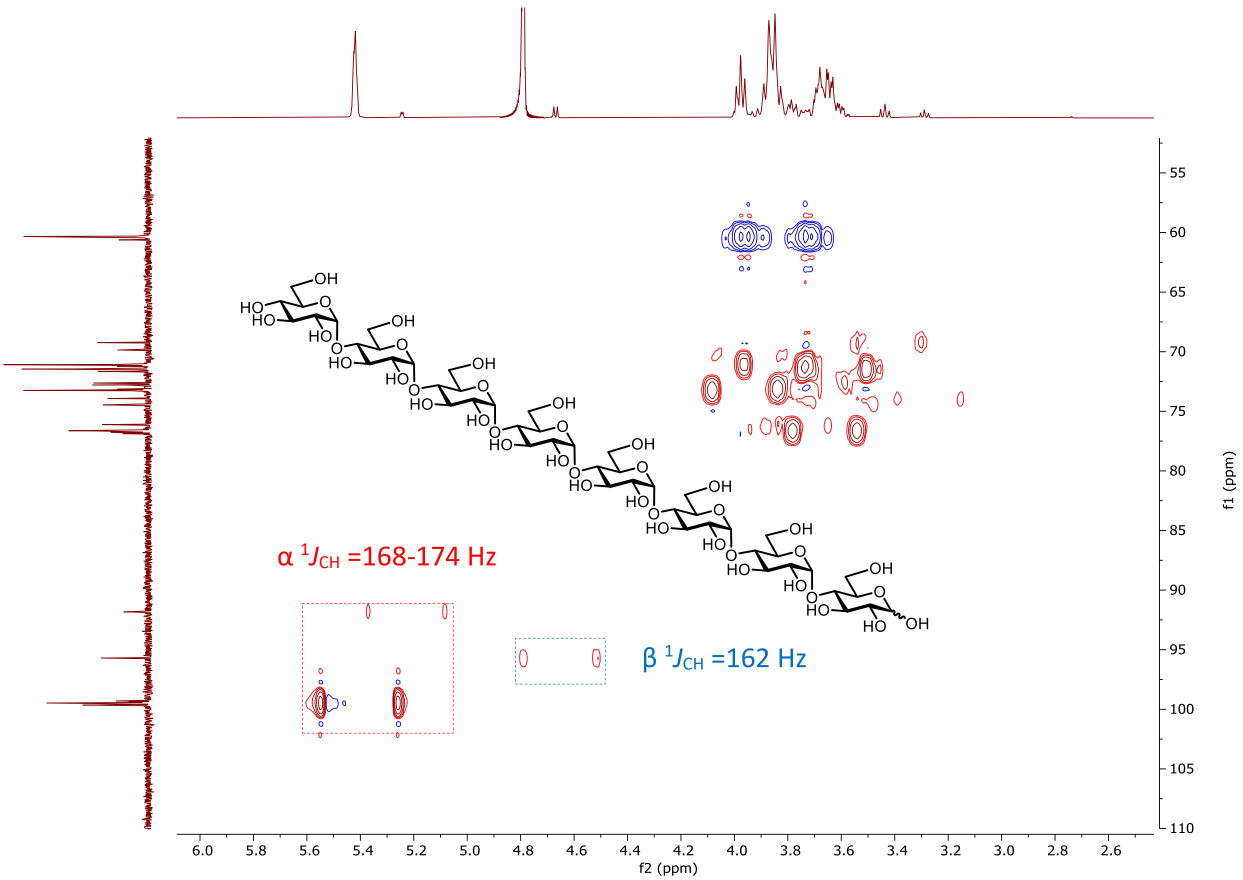

7.3 Synthesis of amylose 16-mer 7

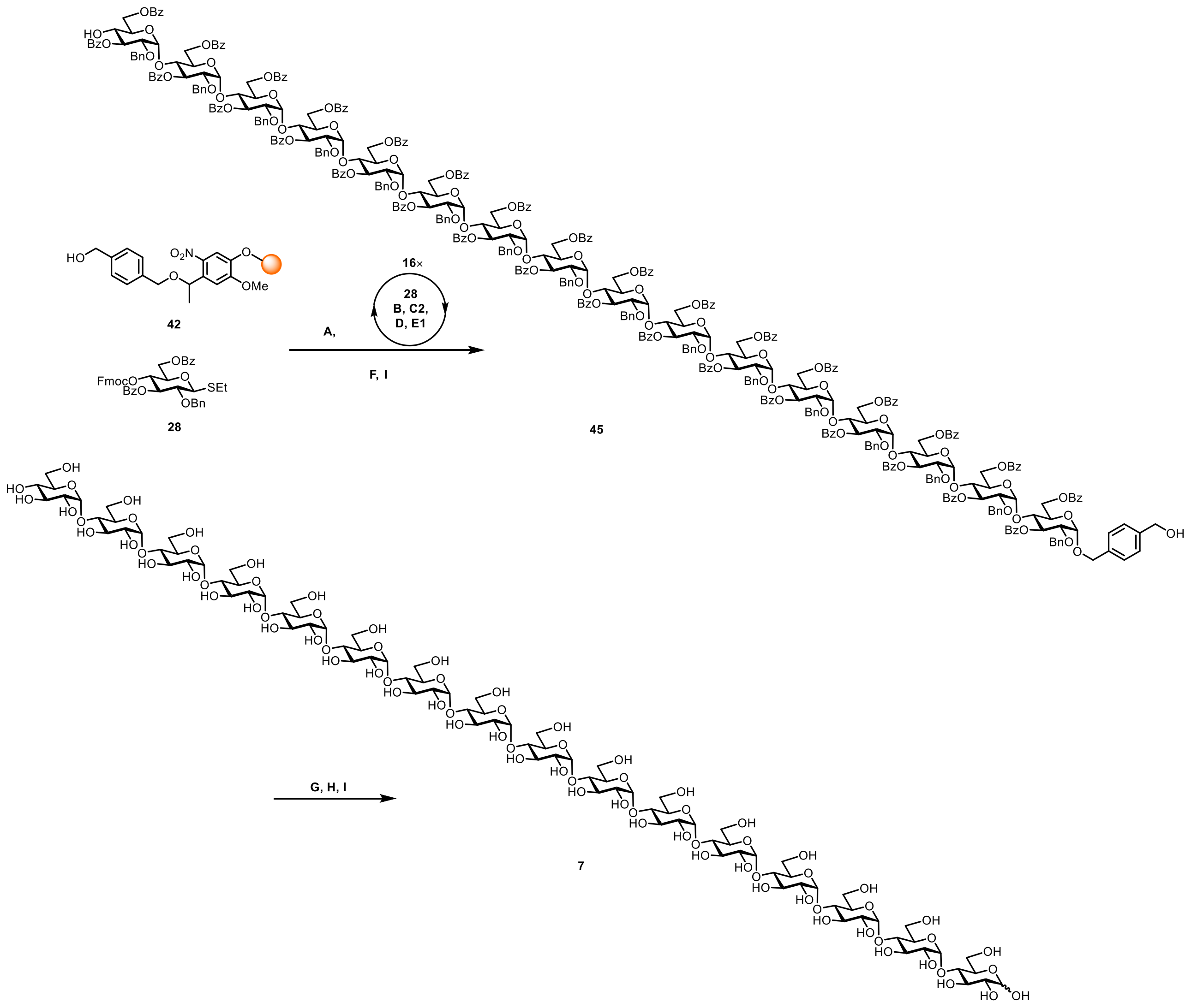

	Module	Conditions
	**A**: Resin Preparation for Synthesis	
16	**B**: Acidic Wash with TMSOTf Solution	
	**C2**: Thioglycoside Glycosylation × 2 Cycle	Building block **28**, 6.5 equiv. (-20°C for 5 min, 0°C for 60 min)
	**D**: Capping	
	**E1**: Fmoc Deprotection	

**F:** Cleavage from Solid Support	
**I**: Purification	**Method E and B2**
**G**: Solution-phase Methanolysis	
**H**: Hydrogenolysis at Ambient Pressure	
**I**: Purification	**Method D**

Automated synthesis and purification afforded protected 16-mer **45** as a white solid (23 mg, 19%).

Analytical data for **45**: ^{1}H NMR (600 MHz, $CDCl_3$) δ 8.11 (d, *J* = 7.6 Hz, 2H), 8.05 (d, *J* = 7.8 Hz, 2H), 8.02 (d, *J* = 7.7 Hz, 2H), 8.00 – 7.80 (m, 58H), 7.58 (dt, *J* = 15.6, 7.5 Hz, 2H), 7.53 – 7.41 (m, 20H), 7.41 – 7.17 (m, 74H), 7.16 – 6.85 (m, 84H), 6.02 (appt, *J* = 9.4 Hz, 1H), 5.86 – 5.63 (m, 14H), 5.48 (appt, *J* = 9.5 Hz, 1H), 5.32 – 5.22 (m, 14H), 5.17 (d, *J* = 3.5 Hz, 1H), 4.93 (d, *J* = 3.5 Hz, 1H), 4.75 (d, *J* = 13.9 Hz, 1H), 4.71 – 4.63 (m, 3H), 4.63 – 4.39 (m, 46H), 4.36 – 4.08 (m, 32H), 4.07 – 3.94 (m, 18H), 3.56 – 3.51 (m, 2H), 3.49 – 3.42 (m, 3H), 3.41 (dd, *J* = 8.6, 3.5 Hz, 1H), 3.37 – 3.27 (m, 10H); ^{13}C NMR (151 MHz, $CDCl_3$) δ 167.13, 166.49, 165.98, 165.92, 165.85, 165.75, 165.69, 165.34, 164.98, 140.69, 137.64, 137.56, 137.40, 136.41, 133.14, 132.94, 132.90, 132.80, 132.67, 130.27, 129.97, 129.83, 129.77, 129.71, 128.53, 128.49, 128.28, 128.10, 128.06, 127.95, 127.93, 127.73, 127.42, 127.37, 127.10, 96.88, 96.02, 95.82, 95.66, 95.53, 94.84, 75.88, 75.72, 74.90, 73.24, 72.75, 72.34, 72.25, 72.11, 71.35, 69.82, 69.63, 69.13, 68.83, 65.16, 63.37, 63.12, 63.01; m/z (HRMS$^+$) $[M + Na]^{3+}$ 2525.556 ($C_{440}H_{394}NO_{114}Na_3^{3+}$ requires 2525.498).

NP-HPLC of crude 45 after AGA (ELSD trace, Method A2)

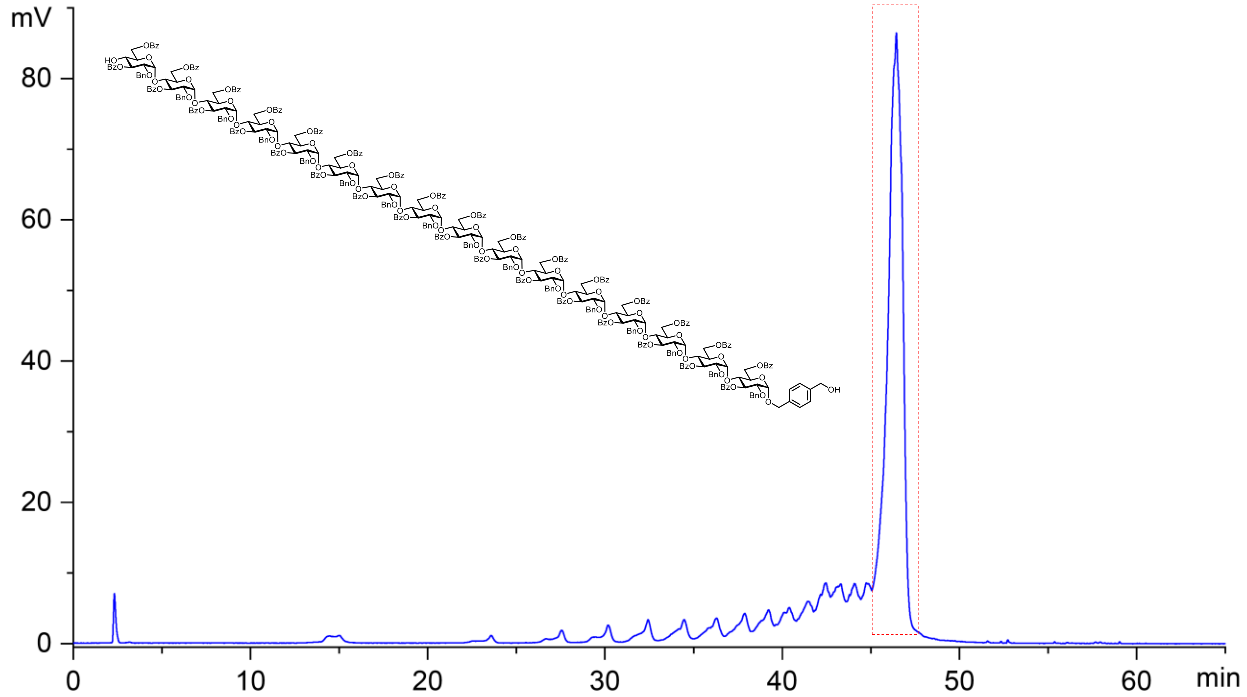

NP-HPLC of pure 45 (ELSD trace, Method A2, t_R = 46.8 min)

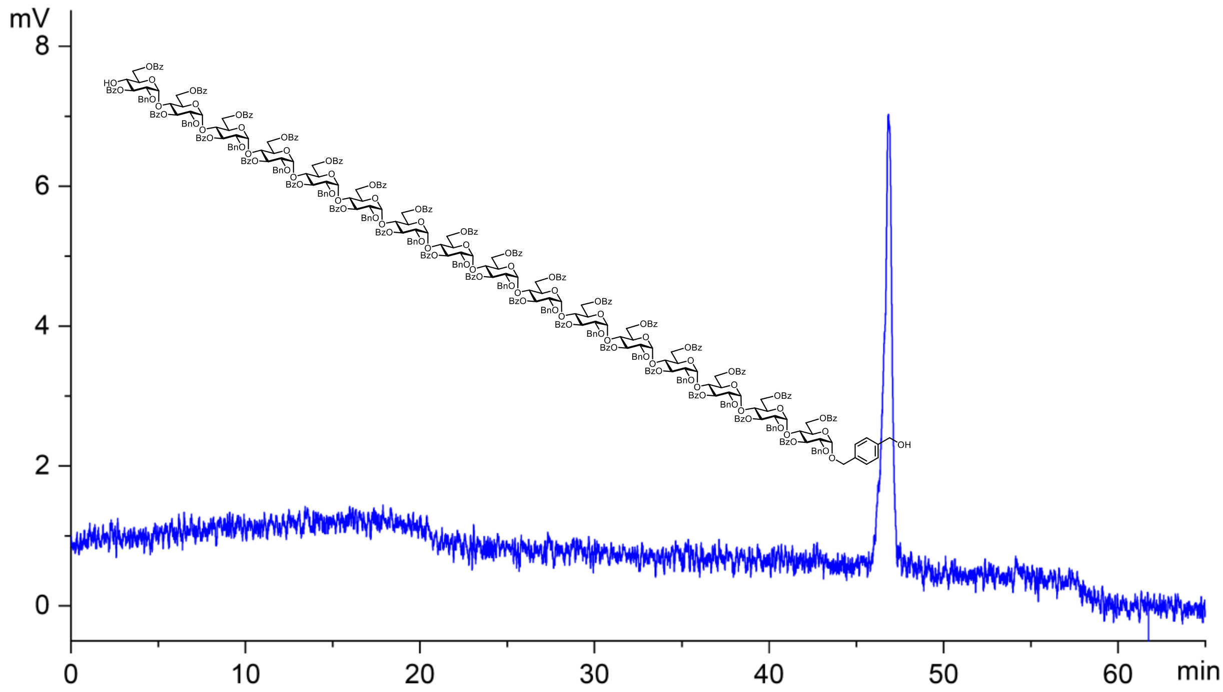

MALDI spectrum of 45

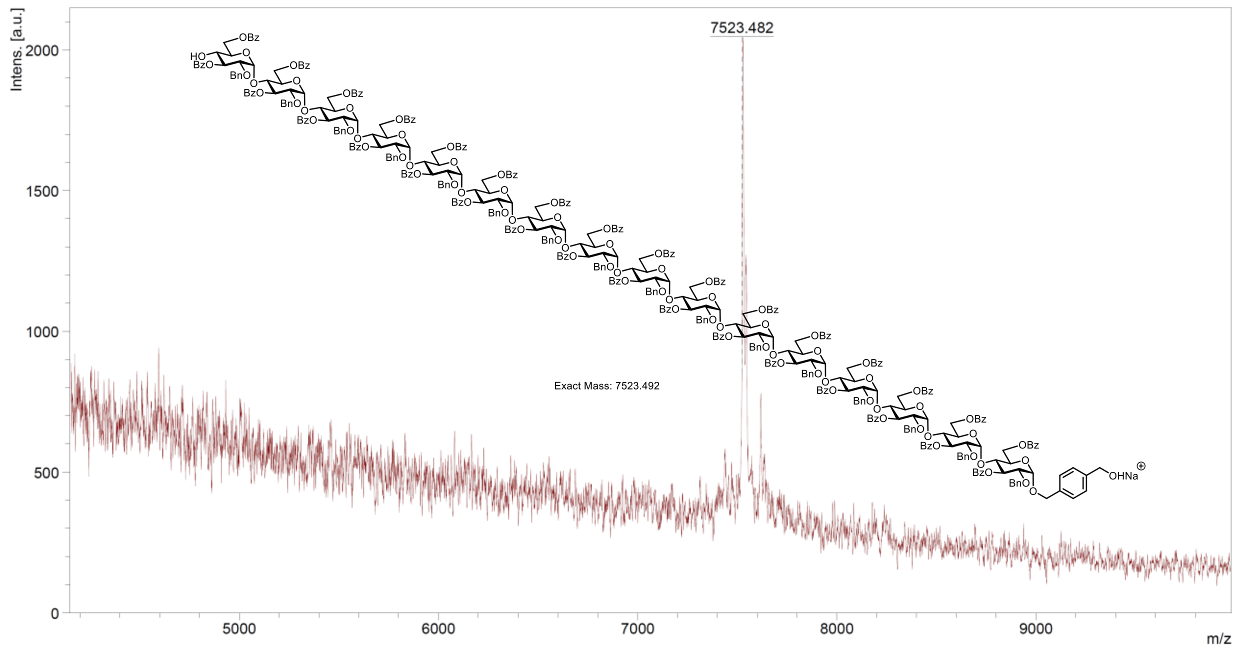

^{1}H NMR of 45 (600 MHz, $CDCl_3$)

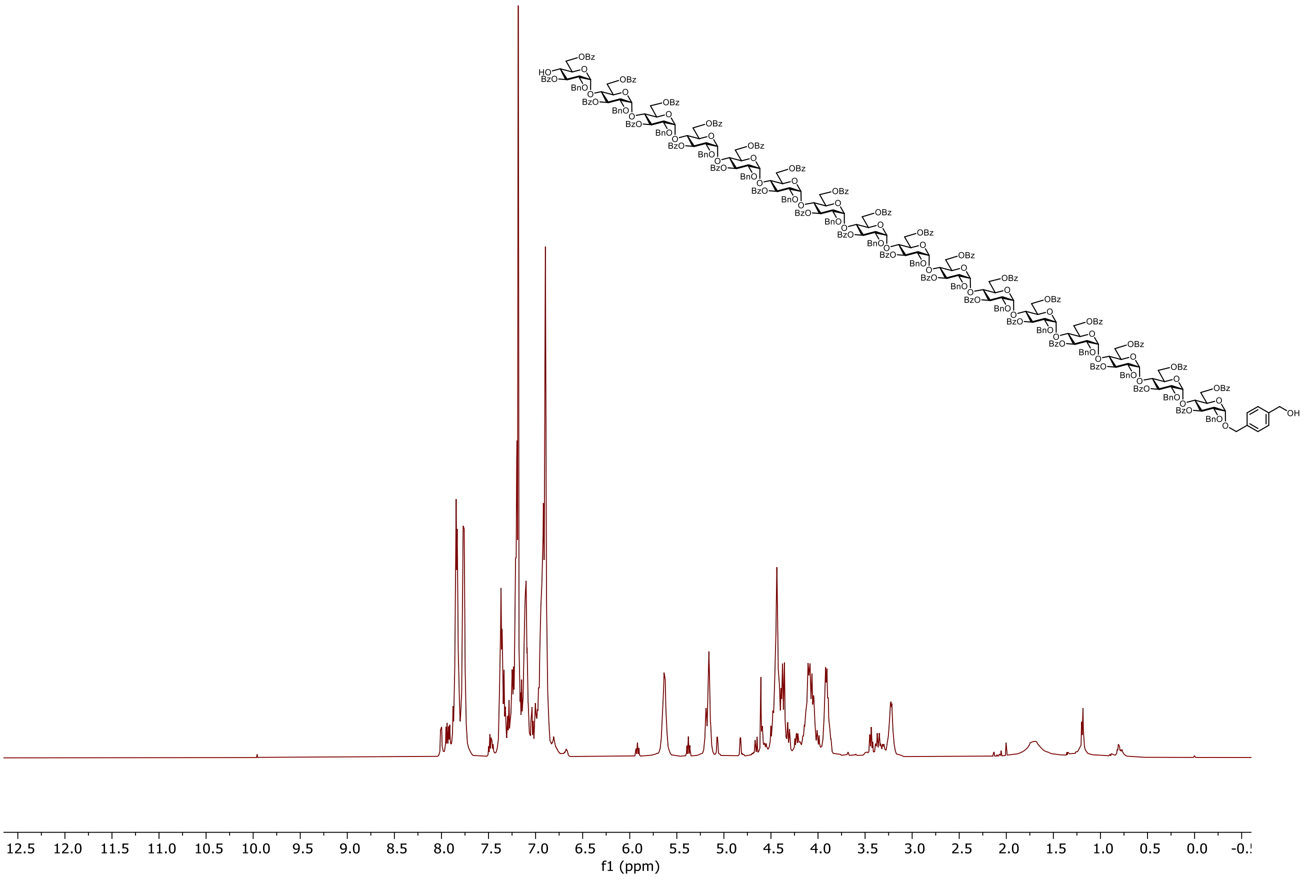

^{13}C NMR of 45 (151 MHz, $CDCl_3$)

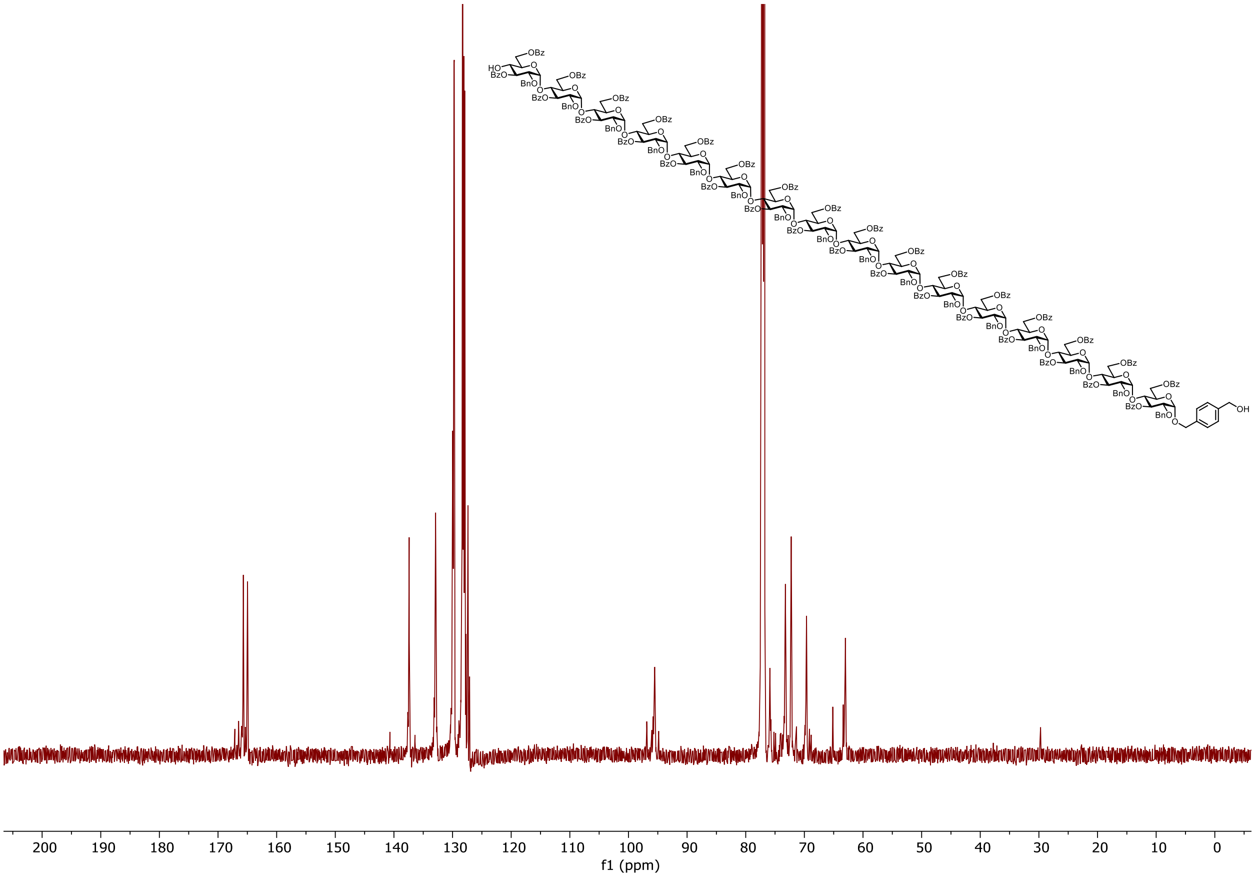

HSQC NMR of 45 ($CDCl_3$)

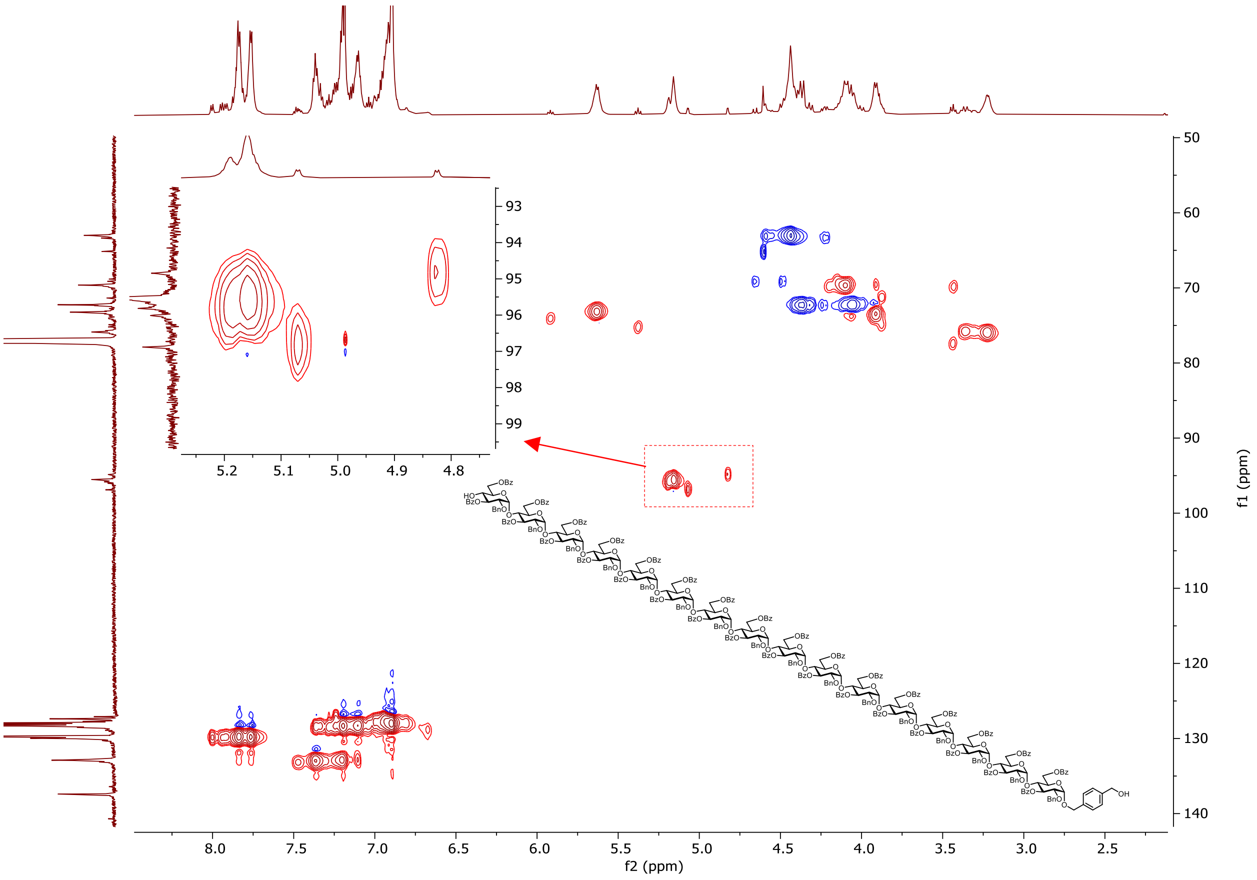

Coupled HSQC NMR of 45 ($CDCl_3$)

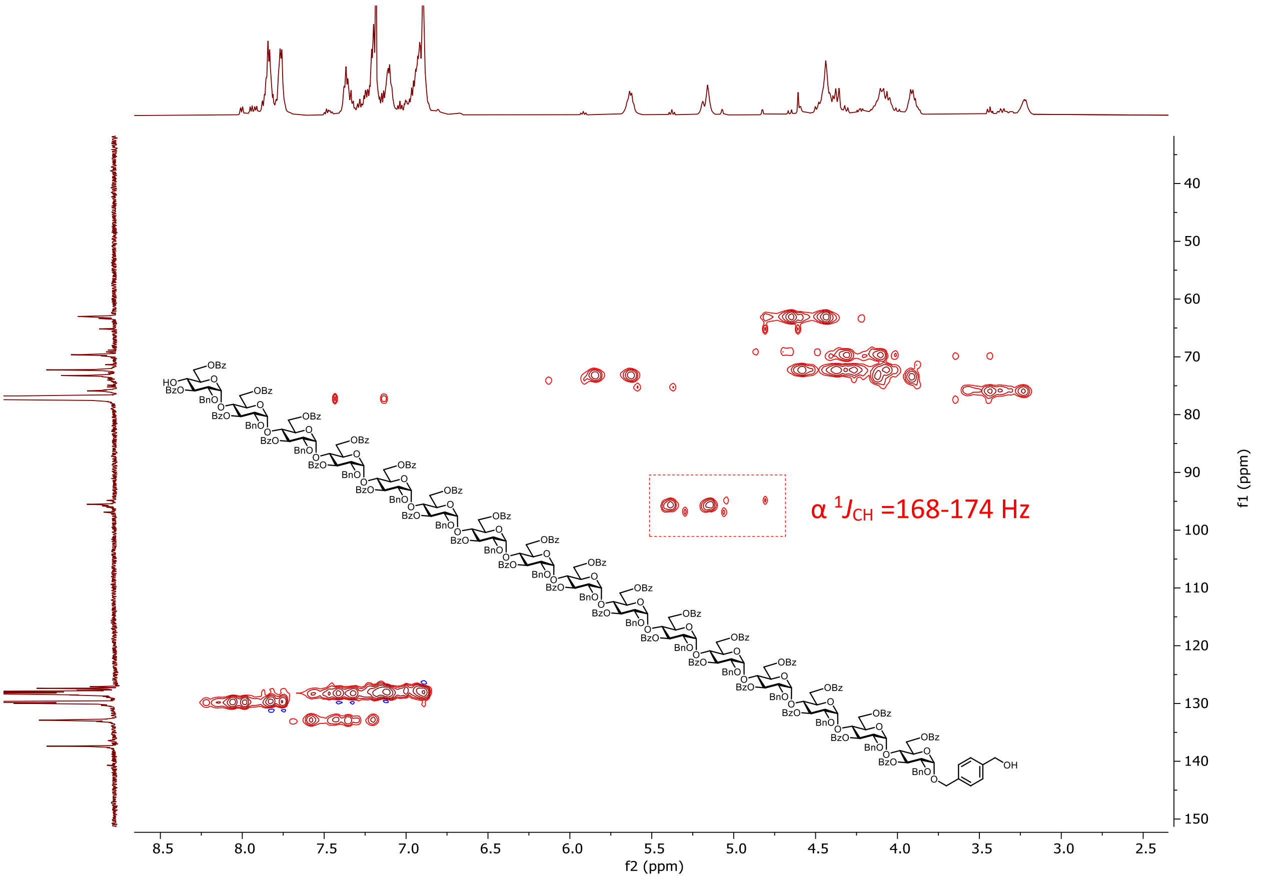

Global deprotection and purification afforded deprotected 16-mer **7** as white a solid (4.3 mg, 10% overall).

Analytical data for **7**: ^{1}H NMR (700 MHz, D_2O) δ 5.47 – 5.37 (m, 15H, 15×α(1→4) anomeric H), 5.24 (d, *J* = 3.8 Hz, 0.44H, α-H1), 4.66 (d, *J* = 8.0 Hz, 0.56H, β-H1), 4.04 – 3.56 (m, 94.44H), 3.43 (appt, *J* = 9.5 Hz, 1H), 3.28 (dd, *J* = 9.5, 8.0 Hz, 0.56H); ^{13}C NMR (176 MHz, D_2O) δ 99.74, 99.62, 99.57, 99.55, 99.38, 95.78 (β-C1), 91.91 (α-C1), 76.98, 76.87, 76.81, 76.78, 76.75, 76.71, 76.68, 76.19, 74.54, 74.01, 73.33, 73.21, 72.87, 72.71, 71.73, 71.56, 71.53, 71.30, 71.22, 71.17, 69.94, 69.32, 60.70, 60.56, 60.48, 60.43; m/z (HRMS$^+$) [M + Na]$^+$ 2633.886 ($C_{96}H_{162}O_{81}Na^+$ requires 2633.845).

RP-HPLC of 7 (ELSD trace, Method C, t_R = 5.2 min)

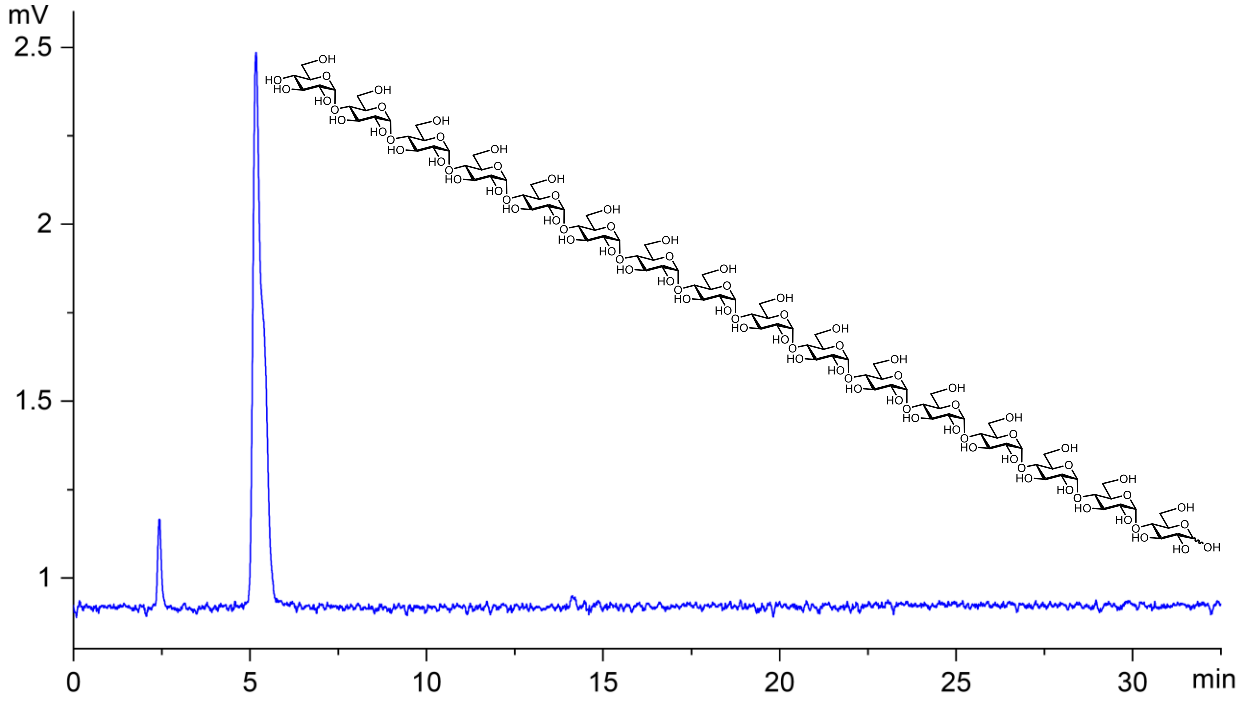

MALDI spectrum of 7

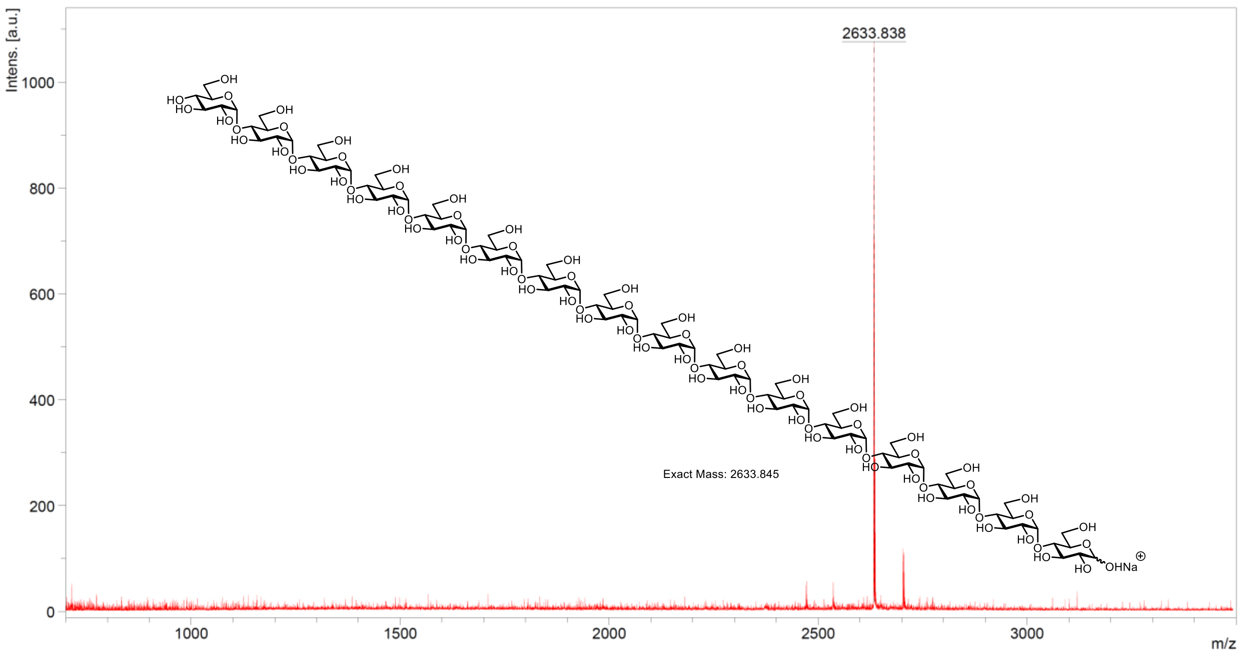

^{1}H NMR of 7 (700 MHz, D_2O)

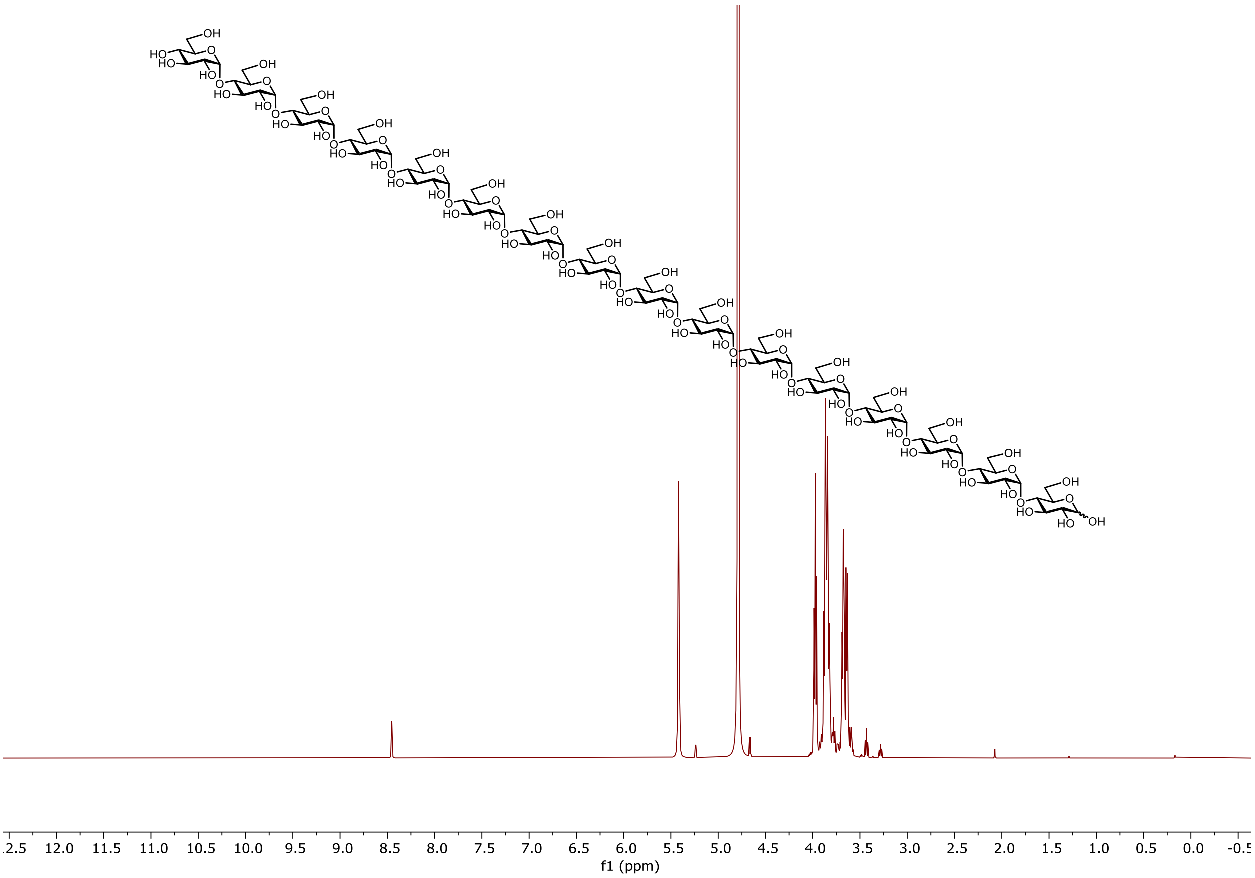

^{13}C NMR of 7 (176 MHz, D_2O)

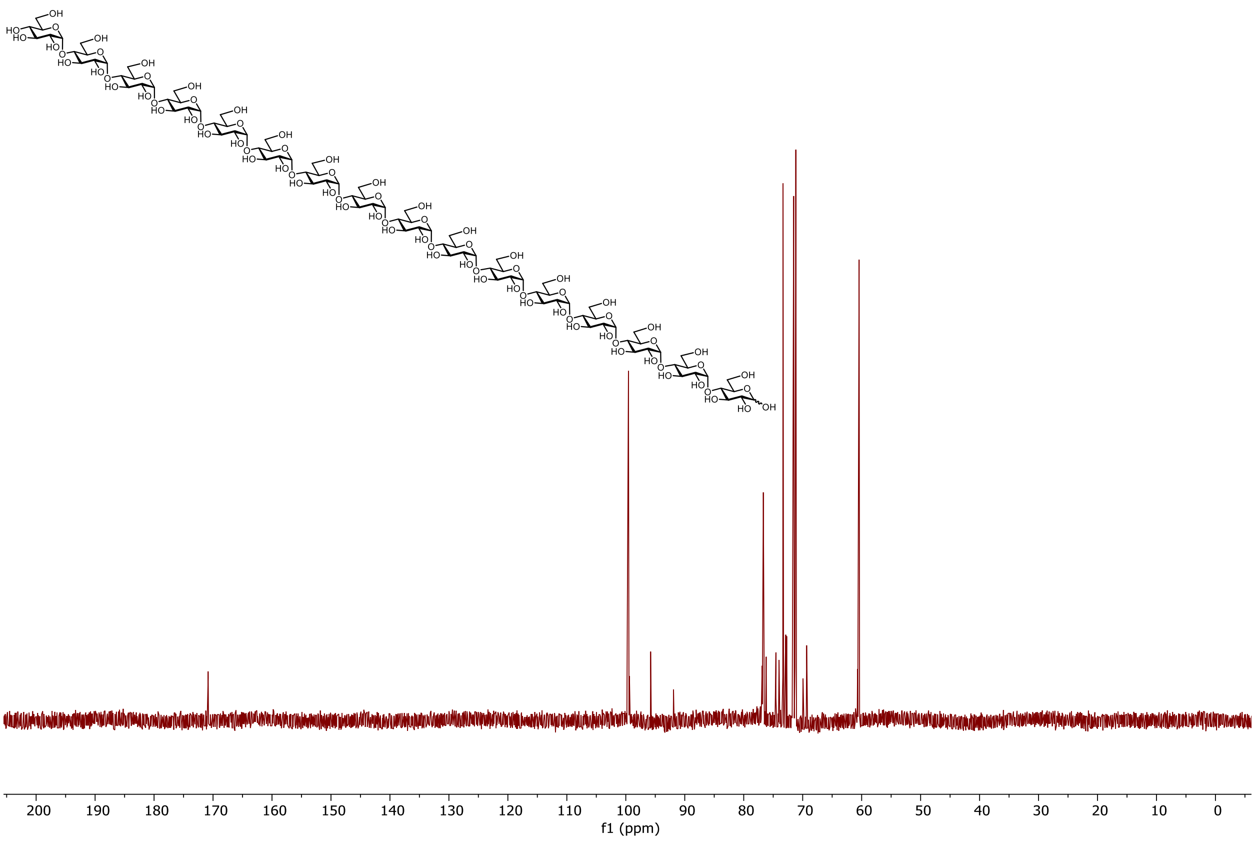

HSQC NMR of 7 (D_2O)

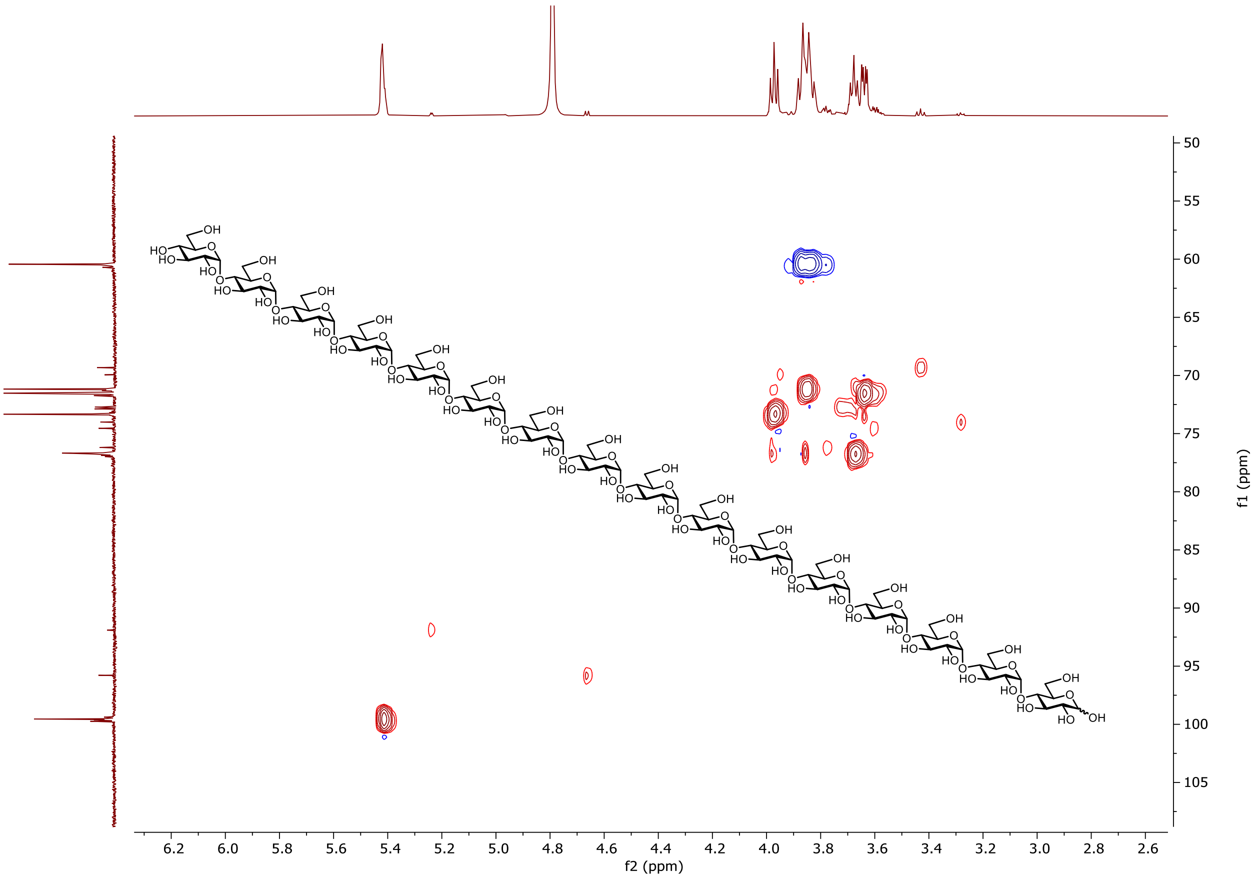

Coupled HSQC NMR of 7 (D_2O)

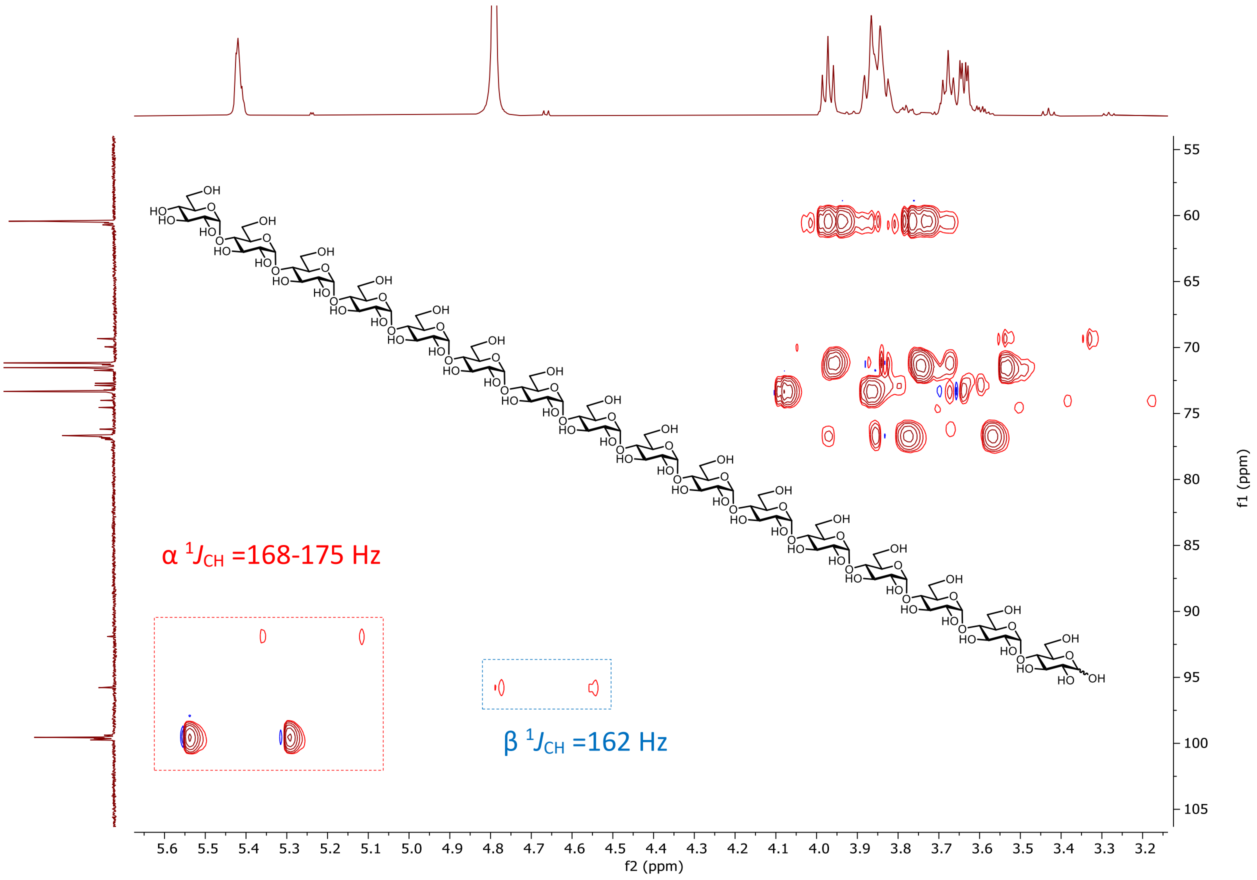

7.4 Synthesis of trimer 50

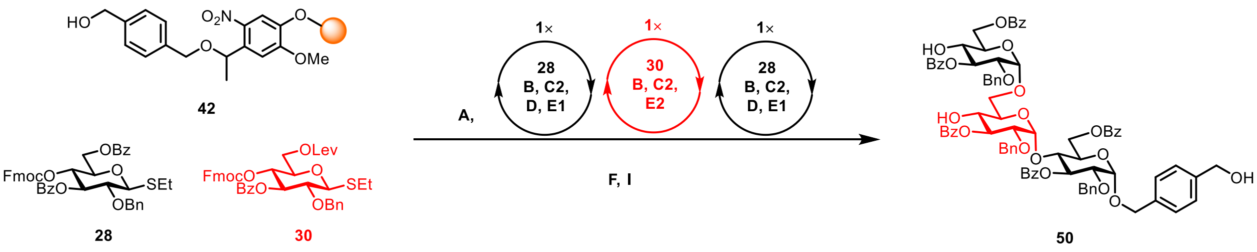

Module	Conditions
**A**: Resin Preparation for Synthesis	
**B**: Acidic Wash with TMSOTf Solution	
**C2**: Thioglycoside Glycosylation × 2 Cycle	Building block **28**, 6.5 equiv. (-20°C for 5 min, 0°C for 60 min)
**D**: Capping	
**E1**: Fmoc Deprotection	
**B**: Acidic Wash with TMSOTf Solution	
**C2**: Thioglycoside Glycosylation × 2 Cycle	Building block **30**, 6.5 equiv. (-20°C for 5 min, 0°C for 60 min)
**E2**: Lev Deprotection	
**B**: Acidic Wash with TMSOTf Solution	
**C2**: Thioglycoside Glycosylation × 2 Cycle	Building block **28**, 6.5 equiv. (-20°C for 5 min, 0°C for 60 min)
**D**: Capping	
**E1**: Fmoc Deprotection	
**F:** Cleavage from Solid Support	
**I**: Purification	**Method E and B2**

Automated synthesis and purification afforded protected trimer **50** as a white solid (7.4 mg, 33%).

Analytical data for **50**: ^{1}H NMR (600 MHz, $CDCl_3$) δ 8.06 – 8.01 (m, 8H), 7.98 – 7.95 (m, 2H), 7.65 – 7.56 (m, 3H), 7.56 – 7.52 (m, 1H), 7.48 – 7.42 (m, 8H), 7.39 (appt, *J* = 8.0 Hz, 3H), 7.33 (d, *J* = 7.7 Hz, 2H), 7.25 – 7.17 (m, 12H), 7.09 – 7.03 (m, 3H), 6.91 – 6.88 (m, 2H), 6.04 (appt, *J* = 9.6 Hz, 1H), 5.76 (appt, *J* = 9.5 Hz, 1H), 5.40 (appt, *J* = 9.6 Hz, 1H), 5.06 – 5.01 (m, 2H), 4.99 – 4.95 (m, 1H), 4.93 (d, *J* = 3.4 Hz, 1H), 4.89 (d, *J* = 3.6 Hz, 1H), 4.86 (d, *J* = 12.3 Hz, 1H), 4.71 – 4.51 (m, 8H), 4.36 – 4.32 (m, 1H), 4.16 – 4.06 (m, 5H), 3.83 (dd, *J* = 10.0, 3.6 Hz, 1H), 3.79 – 3.71 (m, 2H), 3.67 – 3.63 (m, 2H), 3.60 (d, *J* = 10.4 Hz, 1H), 3.38 (appt, *J* = 9.6 Hz, 1H), 3.25 (dd, *J* = 9.9, 3.3 Hz, 1H); ^{13}C NMR (151 MHz, $CDCl_3$) δ 167.80, 166.87, 166.74, 166.56, 165.58, 140.58, 137.99, 137.90, 137.62, 136.45, 133.38, 133.31, 133.05, 133.01, 132.41, 131.01, 130.10, 130.00, 129.96, 129.91, 129.83, 129.79, 129.75, 129.71, 129.64, 129.59, 128.75, 128.57, 128.52, 128.44, 128.36, 128.33, 128.30, 128.22, 128.07, 127.77, 127.75, 127.69, 127.63, 127.15, 127.12, 98.23, 97.11, 94.89, 76.04,

75.97, 75.11, 73.20, 72.46, 72.32, 72.13, 72.04, 70.40, 70.04, 69.84, 68.89, 68.56, 67.38, 65.18, 64.61, 63.62; m/z (HRMS$^+$) $[M + K]^+$ 1453.483 ($C_{82}H_{78}O_{22}K^+$ requires 1453.461).

NP-HPLC of 50 after AGA (ELSD trace, Method A2)

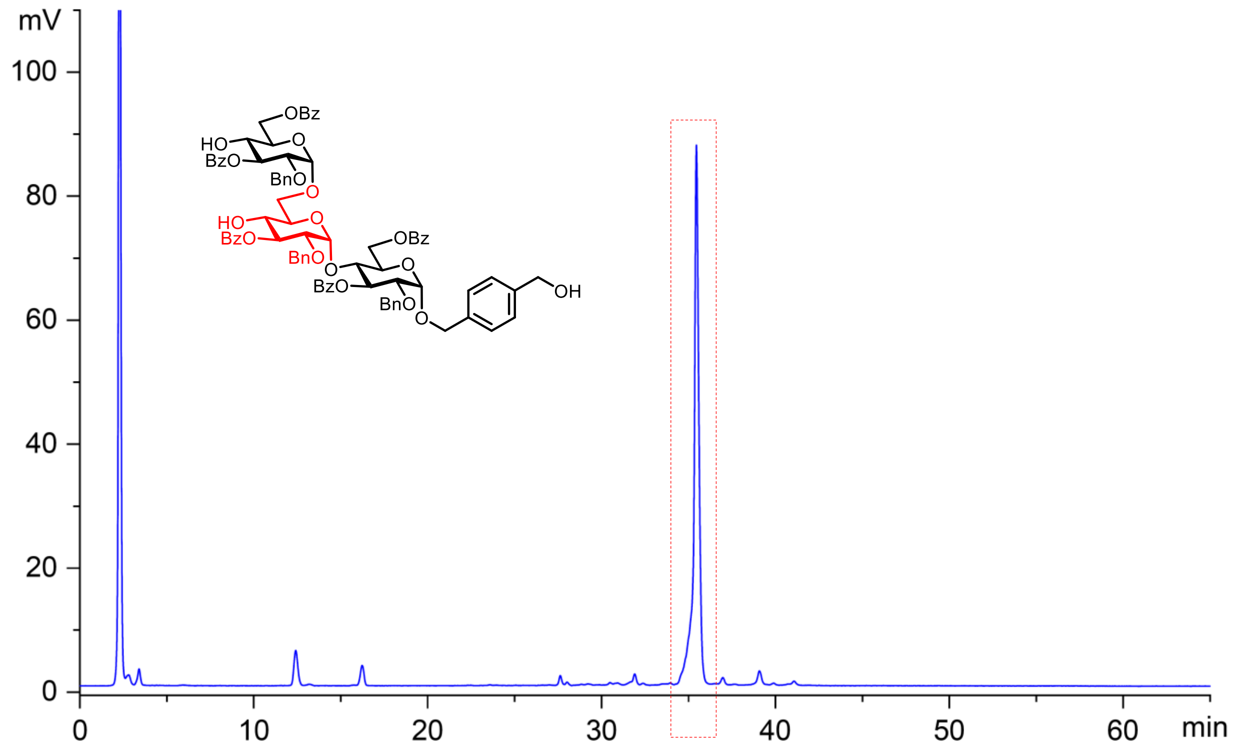

NP-HPLC of pure 50 (ELSD trace, Method A2, t_R = 35.1 min)

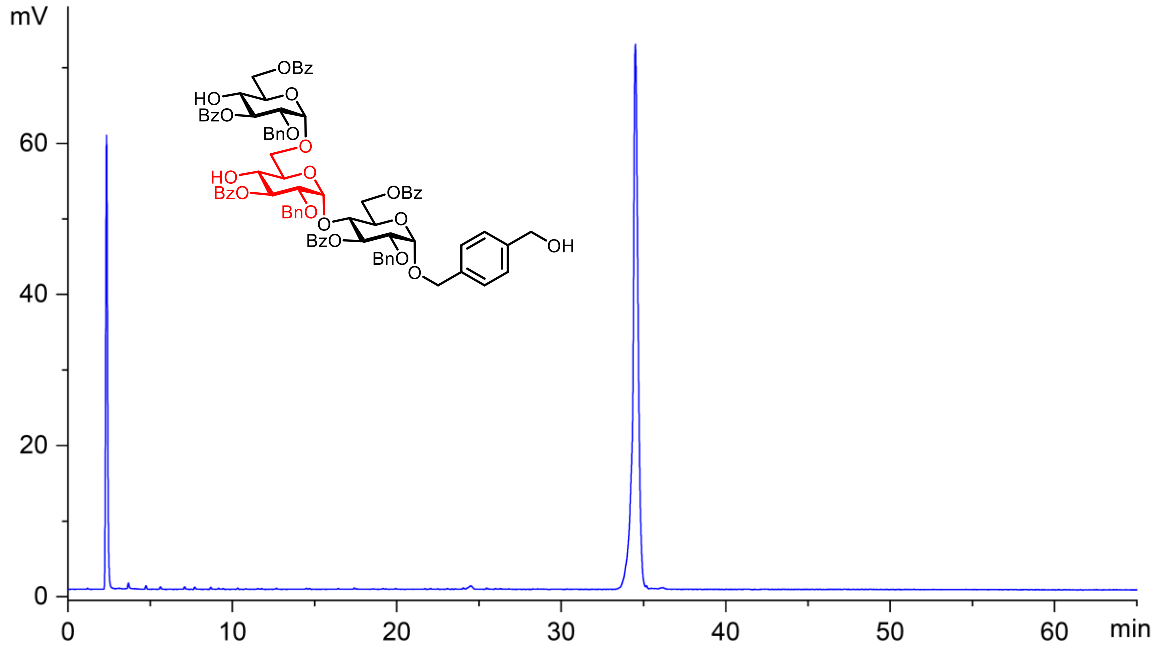

MALDI spectrum of 50

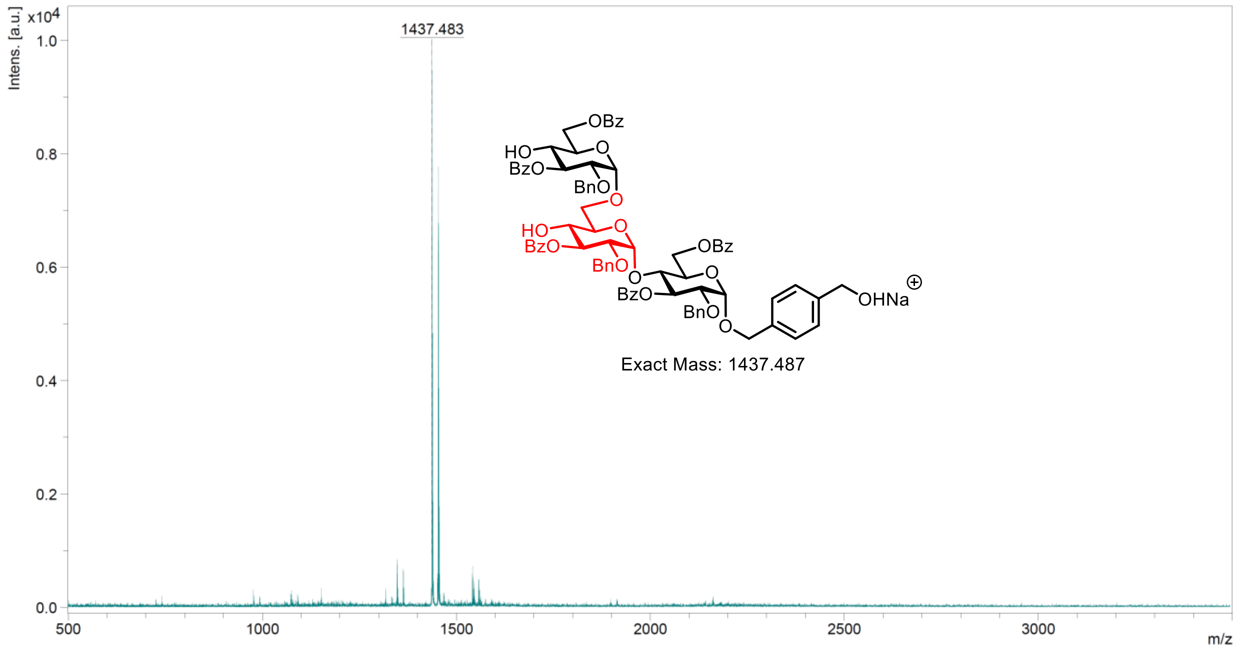

^{1}H NMR of 50 (600 MHz, $CDCl_3$)

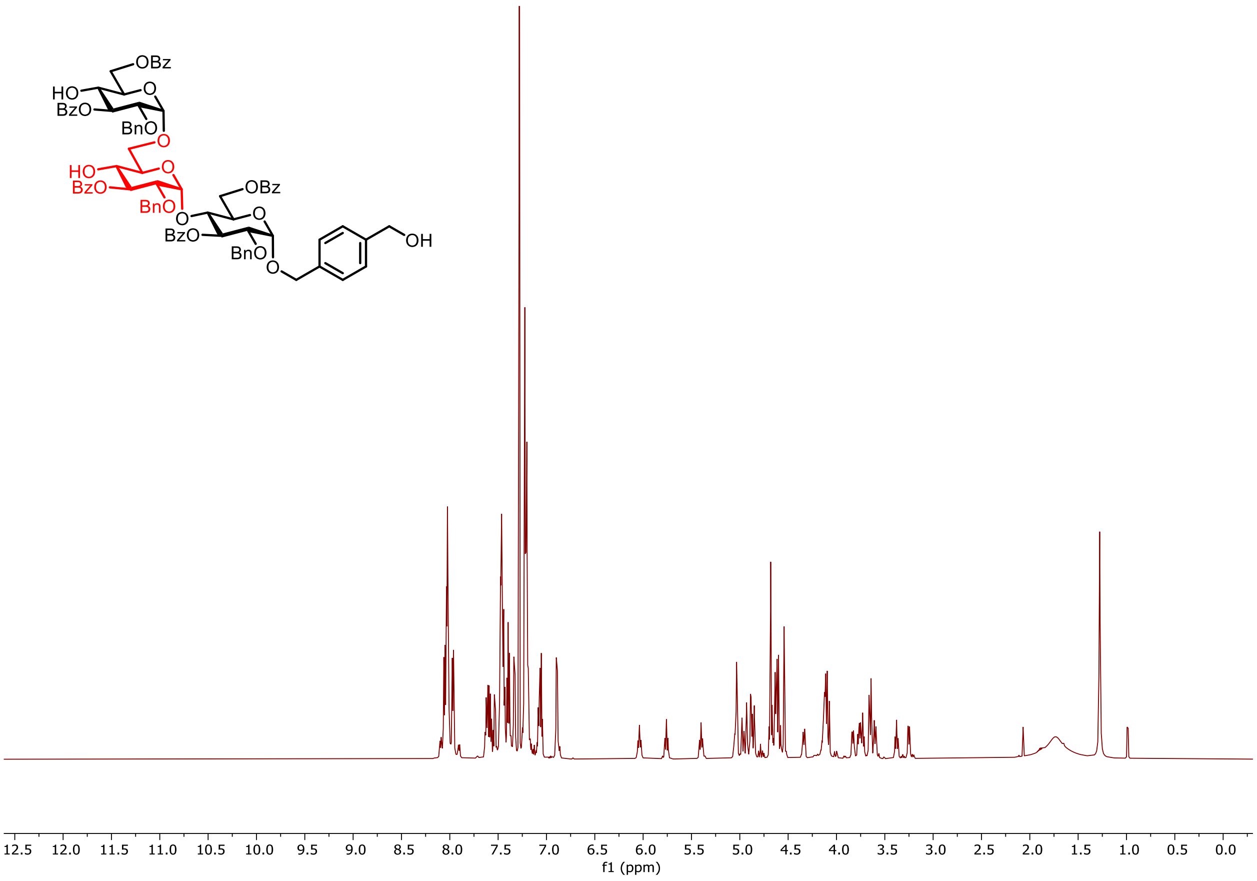

^{13}C NMR of 50 (151 MHz, $CDCl_3$)

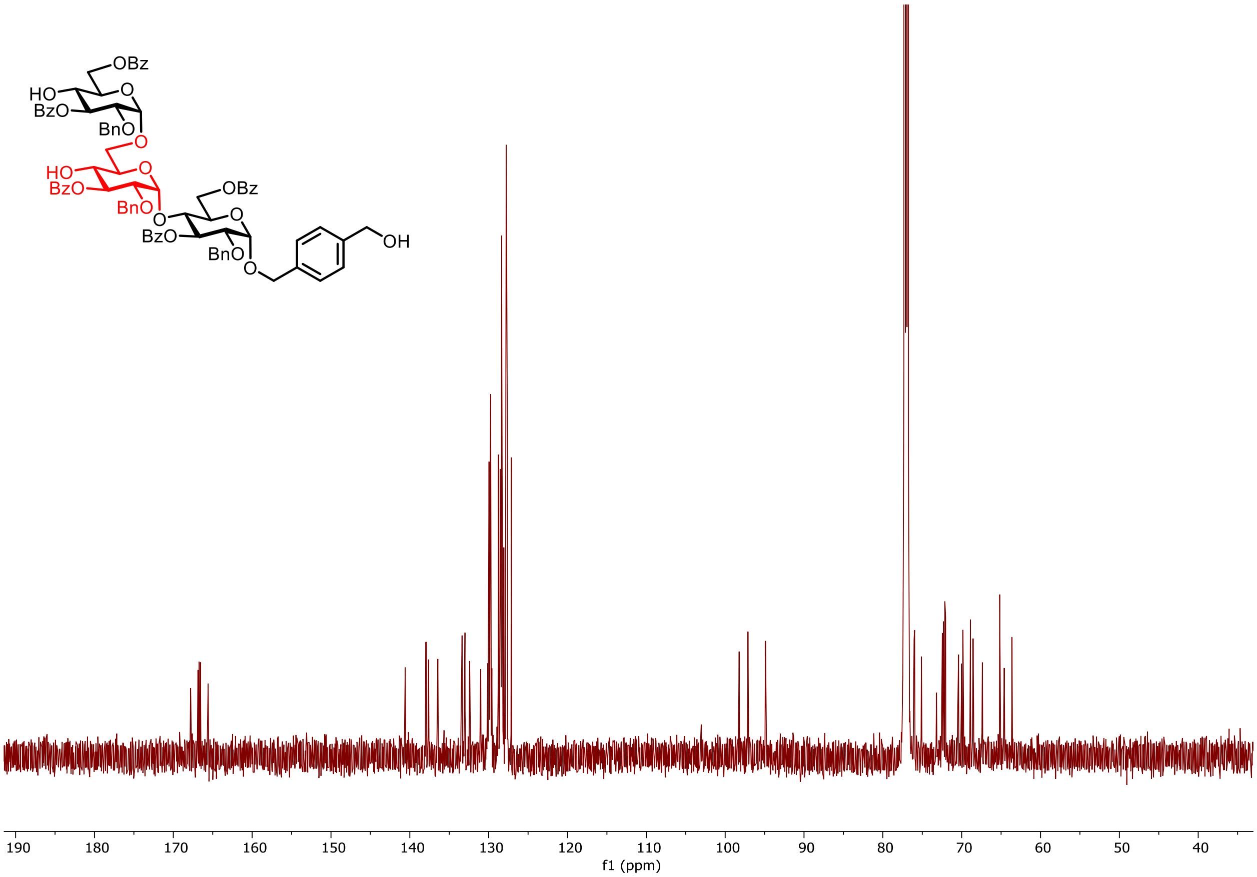

HSQC NMR of 50 ($CDCl_3$)

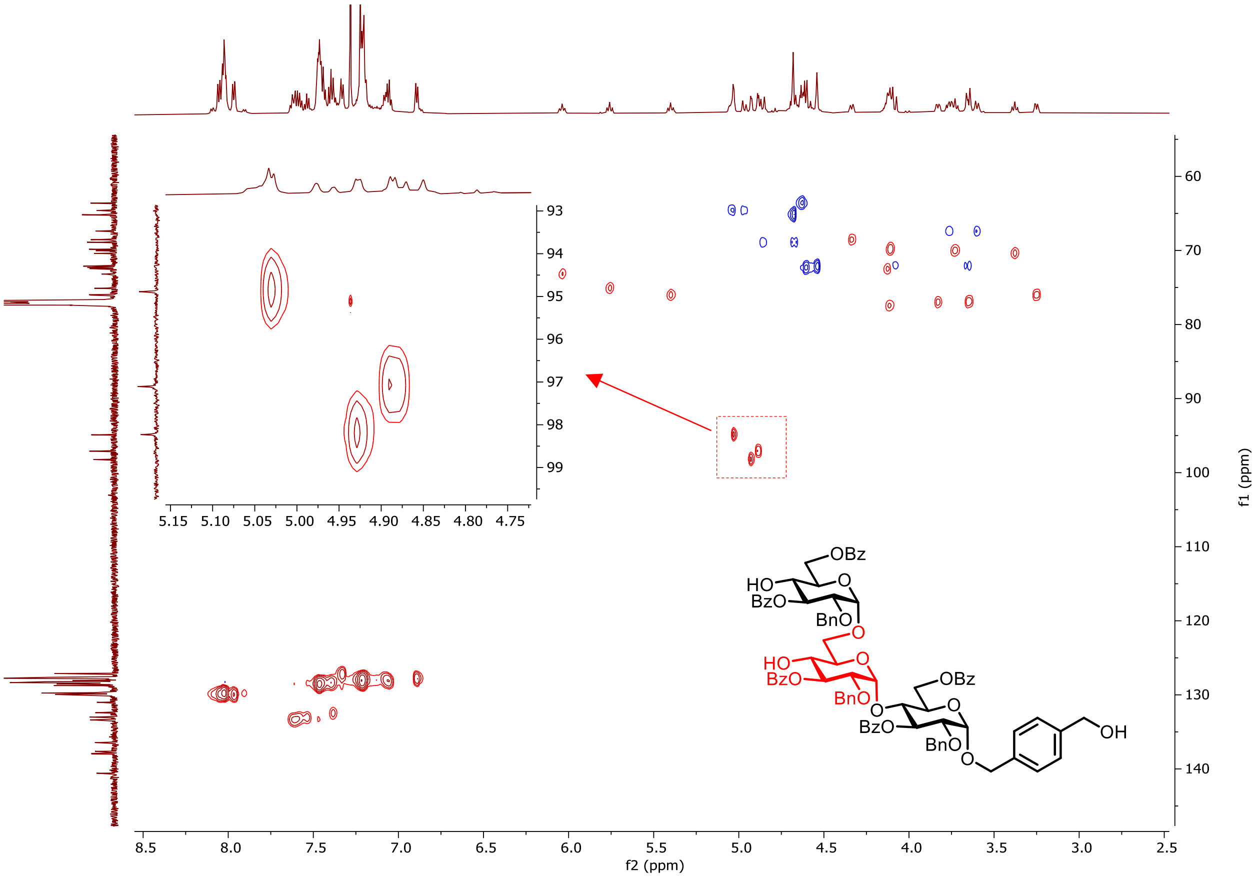

Coupled HSQC NMR of 50 ($CDCl_3$)

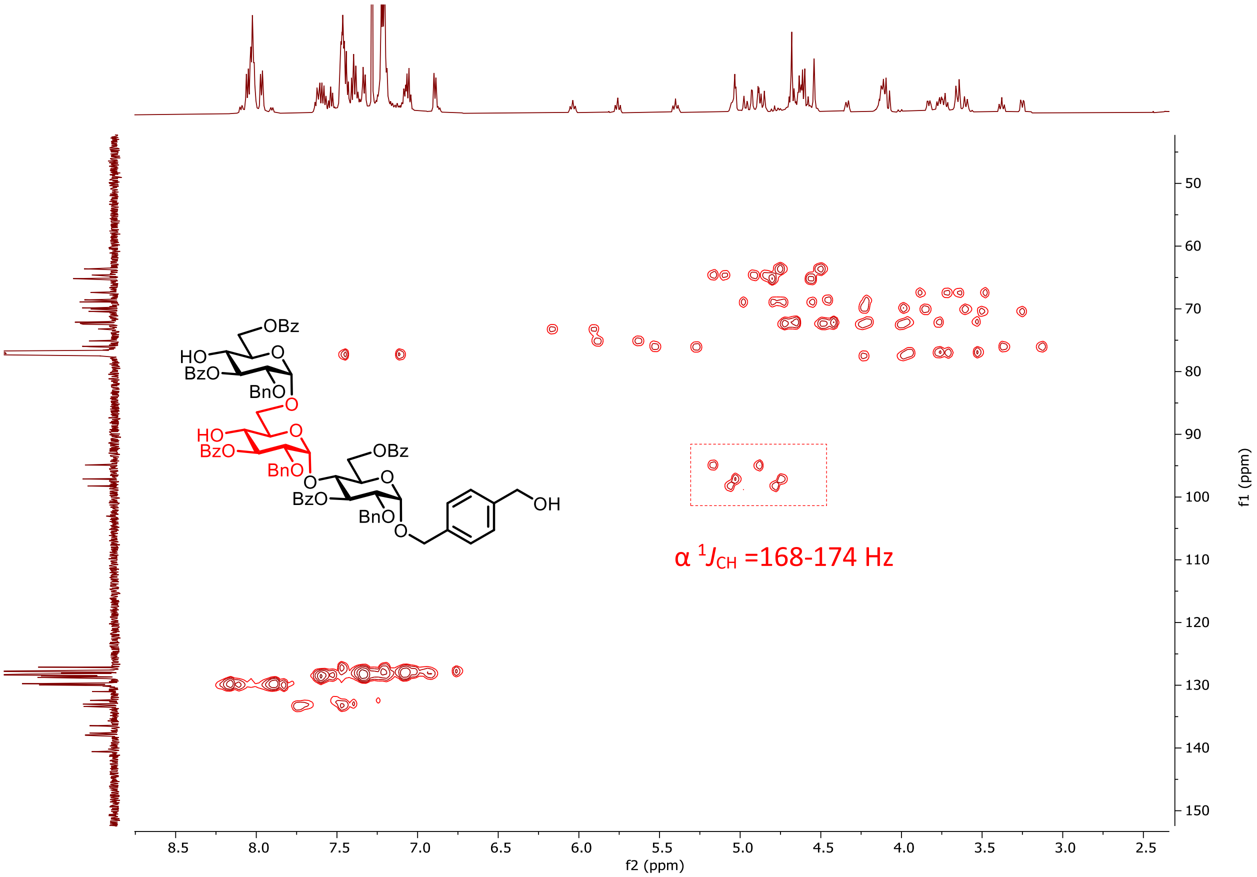

7.5 Synthesis of amylopectin tetramer 8

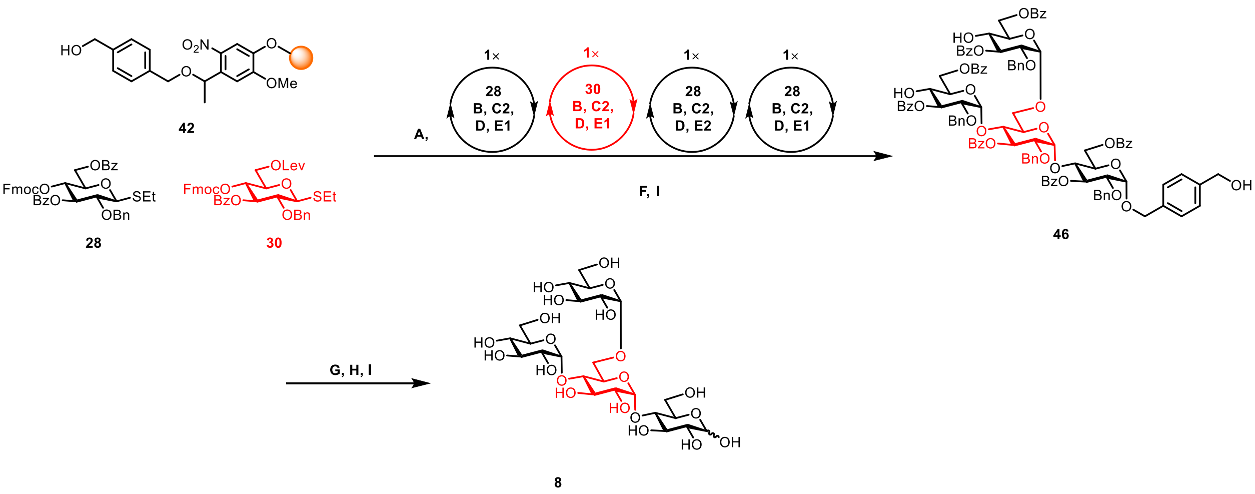

Module	Conditions
**A**: Resin Preparation for Synthesis	
**B**: Acidic Wash with TMSOTf Solution	
**C2**: Thioglycoside Glycosylation × 2 Cycle	Building block **28**, 6.5 equiv. (-20°C for 5 min, 0°C for 60 min)
**D**: Capping	
**E1**: Fmoc Deprotection	
**B**: Acidic Wash with TMSOTf Solution	
**C2**: Thioglycoside Glycosylation × 2 Cycle	Building block **30**, 6.5 equiv. (-20°C for 5 min, 0°C for 60 min)
**D**: Capping	
**E1**: Fmoc Deprotection	
**B**: Acidic Wash with TMSOTf Solution	
**C2**: Thioglycoside Glycosylation × 2 Cycle	Building block **28**, 6.5 equiv. (-20°C for 5 min, 0°C for 60 min)
**D**: Capping	
**E2**: Lev Deprotection	
**B**: Acidic Wash with TMSOTf Solution	
**C2**: Thioglycoside Glycosylation × 2 Cycle	Building block **28**, 6.5 equiv. (-20°C for 5 min, 0°C for 60 min)
**D**: Capping	
**E1**: Fmoc Deprotection	
**F:** Cleavage from Solid Support	
**I**: Purification	**Method E and B2**
**G**: Solution-phase Methanolysis	

H: Hydrogenolysis at Ambient Pressure

I: Purification **Method D**

Automated synthesis and purification afforded protected tetramer **46** as a white solid (7.0 mg, 23%).

Analytical data for **46**: ^{1}H NMR (700 MHz, $CDCl_3$) δ 8.14 (d, *J* = 7.7 Hz, 2H), 8.10 (2×d, *J* = 7.7, 4H), 8.04 (d, *J* = 7.8 Hz, 2H), 7.97 (d, *J* = 7.8 Hz, 2H), 7.91 (d, *J* = 7.7 Hz, 2H), 7.85 (d, *J* = 7.7 Hz, 2H), 7.62 (appt, *J* = 7.5 Hz, 1H), 7.57 (dt, *J* = 14.6, 7.4 Hz, 3H), 7.53 – 7.32 (m, 20H), 7.23 – 7.14 (m, 7H), 7.14 – 7.08 (m, 2H), 7.05 (q, *J* = 7.4 Hz, 3H), 7.03 – 6.97 (m, 3H), 6.94 (appt, *J* = 7.7 Hz, 4H), 6.86 (d, *J* = 7.6 Hz, 2H), 6.07 (appt, *J* = 9.4 Hz, 1H), 5.74 (appt, *J* = 8.9 Hz, 1H), 5.64 (appt, *J* = 9.5 Hz, 1H), 5.42 (appt, *J* = 9.5 Hz, 1H), 5.24 (d, *J* = 3.4 Hz, 1H), 5.20 (d, *J* = 3.7 Hz, 1H), 5.18 (d, *J* = 3.6 Hz, 1H), 5.01 (d, *J* = 3.6 Hz, 1H), 4.80 (d, *J* = 12.3 Hz, 1H), 4.78 – 4.68 (m, 6H), 4.66 (d, *J* = 12.3 Hz, 1H), 4.61 (dd, *J* = 12.4, 2.3 Hz, 1H), 4.59 – 4.49 (m, 3H), 4.45 (d, *J* = 12.5 Hz, 1H), 4.34 (appt, *J* = 12.7 Hz, 2H), 4.27 (dt, *J* = 9.4, 3.1 Hz, 1H), 4.20 (appt, *J* = 9.5 Hz, 1H), 4.18 – 4.12 (m, 3H), 4.08 – 4.01 (m, 4H), 3.81 – 3.72 (m, 3H), 3.70 (appt, *J* = 9.6 Hz, 1H), 3.65 (appt, *J* = 9.6 Hz, 1H), 3.58 (dd, *J* = 9.9, 3.5 Hz, 1H), 3.46 (dd, *J* = 9.8, 3.4 Hz, 1H), 3.16 (dd, *J* = 9.8, 3.6 Hz, 1H); ^{13}C NMR (176 MHz, $CDCl_3$) δ 167.29, 166.84, 166.80, 166.72, 166.27, 165.27, 140.73, 137.79, 137.68, 137.67, 137.33, 136.43, 133.31, 133.11, 133.05, 132.93, 132.40, 130.53, 130.49, 130.00, 129.96, 129.94, 129.91, 129.86, 129.84, 129.81, 129.77, 128.59, 128.41, 128.36, 128.32, 128.28, 128.27, 128.24, 128.19, 128.09, 127.96, 127.91, 127.81, 127.76, 127.68, 127.53, 127.49, 127.38, 127.22, 127.14, 97.31, 96.22, 96.14, 95.01, 76.61, 76.54, 75.92, 75.11, 75.01, 74.27, 73.74, 72.83, 72.35, 72.31, 71.92, 71.39, 70.16, 69.71, 69.60, 69.26, 68.52, 65.85, 65.15, 63.91, 63.39, 63.22; m/z (HRMS^{+}) [M + Na]$^{+}$ 1897.651 ($C_{109}H_{102}O_{29}Na^{+}$ requires 1897.639).

NP-HPLC of crude 46 after AGA (ELSD trace, Method A2)

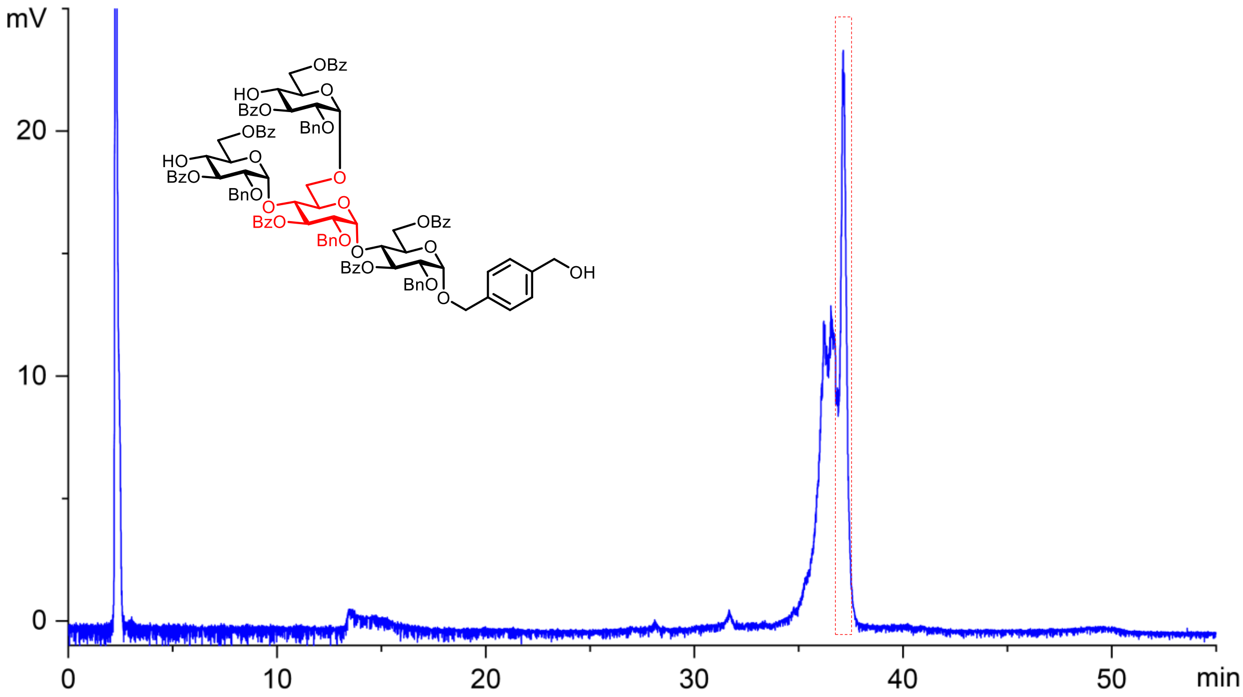

NP-HPLC of pure 46 (ELSD trace, Method A2, t_R = 37.2 min)

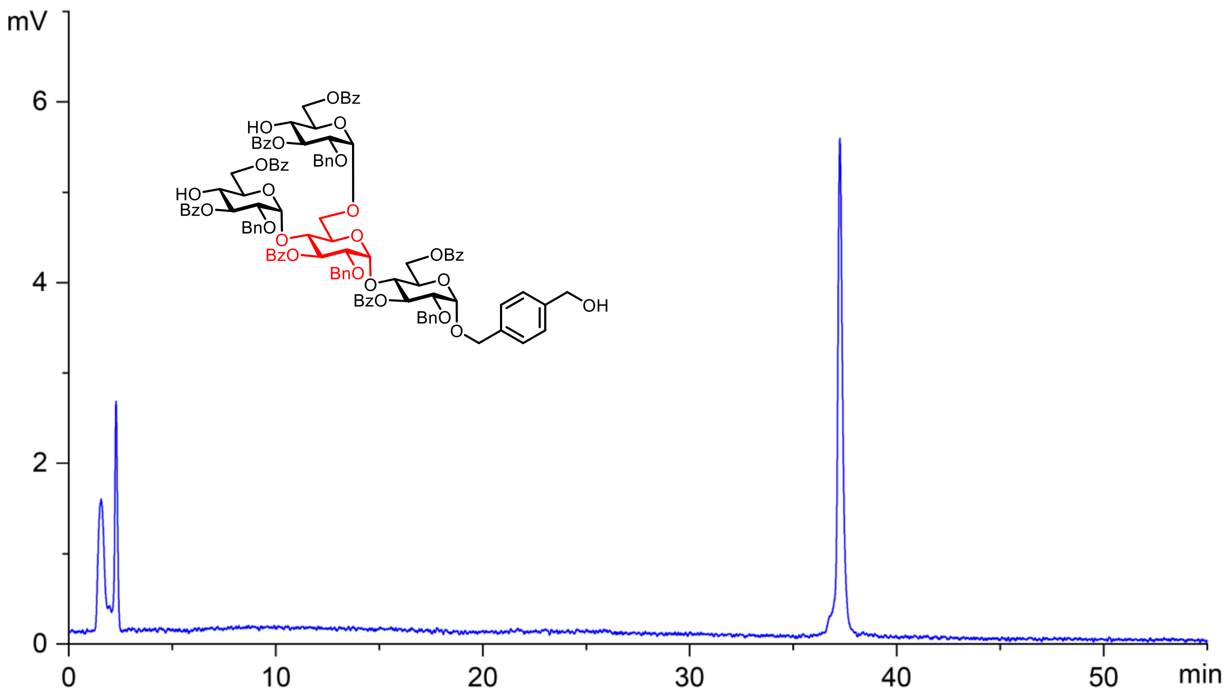

MALDI spectrum of 46

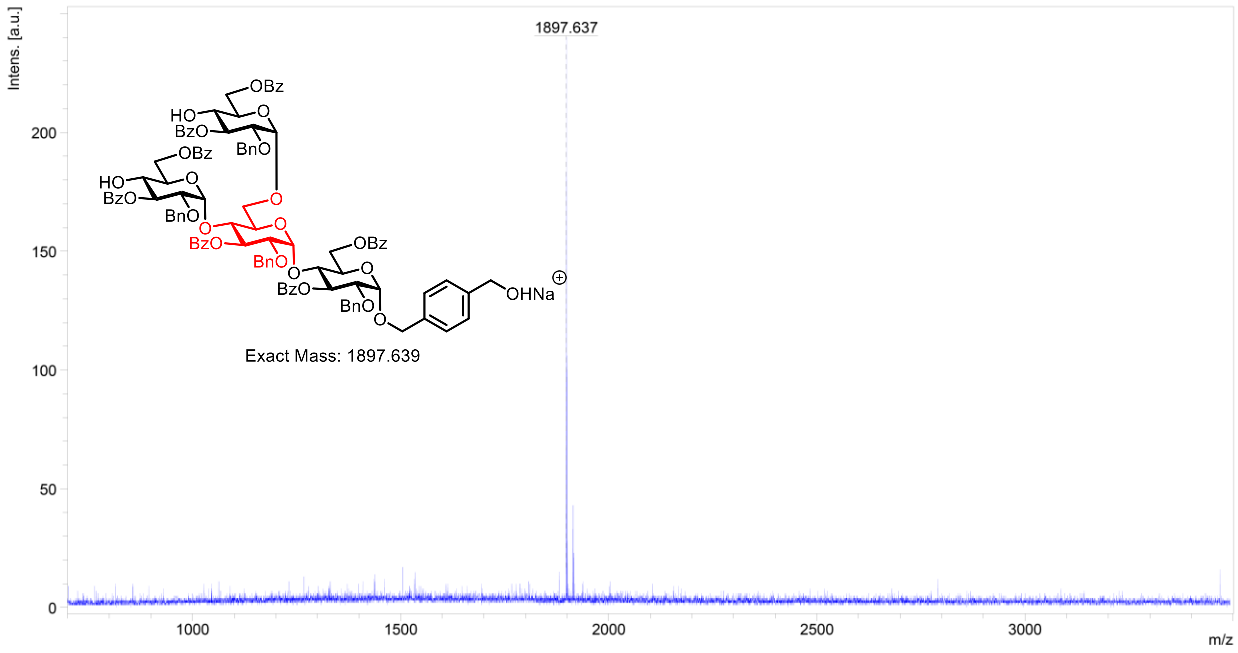

^{1}H NMR of 46 (700 MHz, $CDCl_3$)

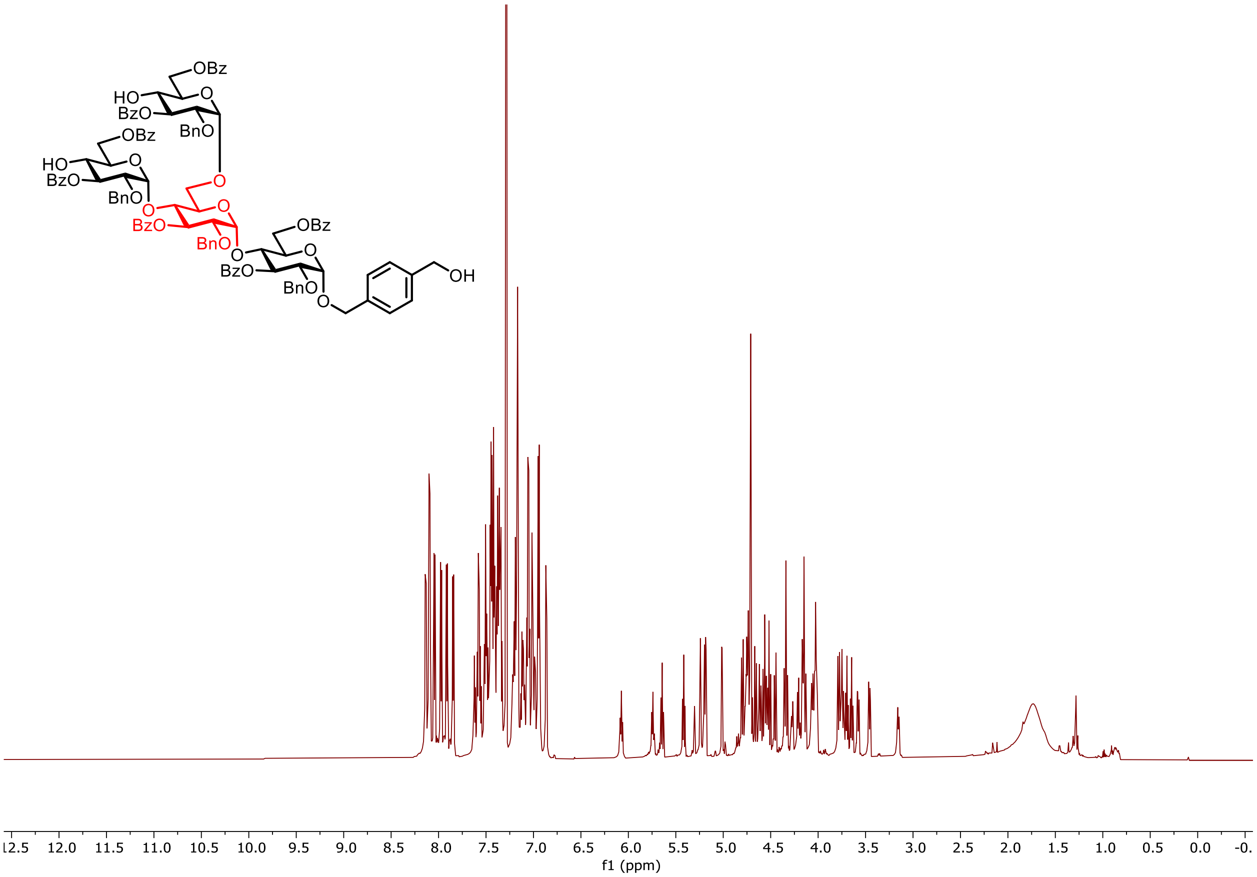

^{13}C NMR of 46 (176 MHz, CDCl$_3$)

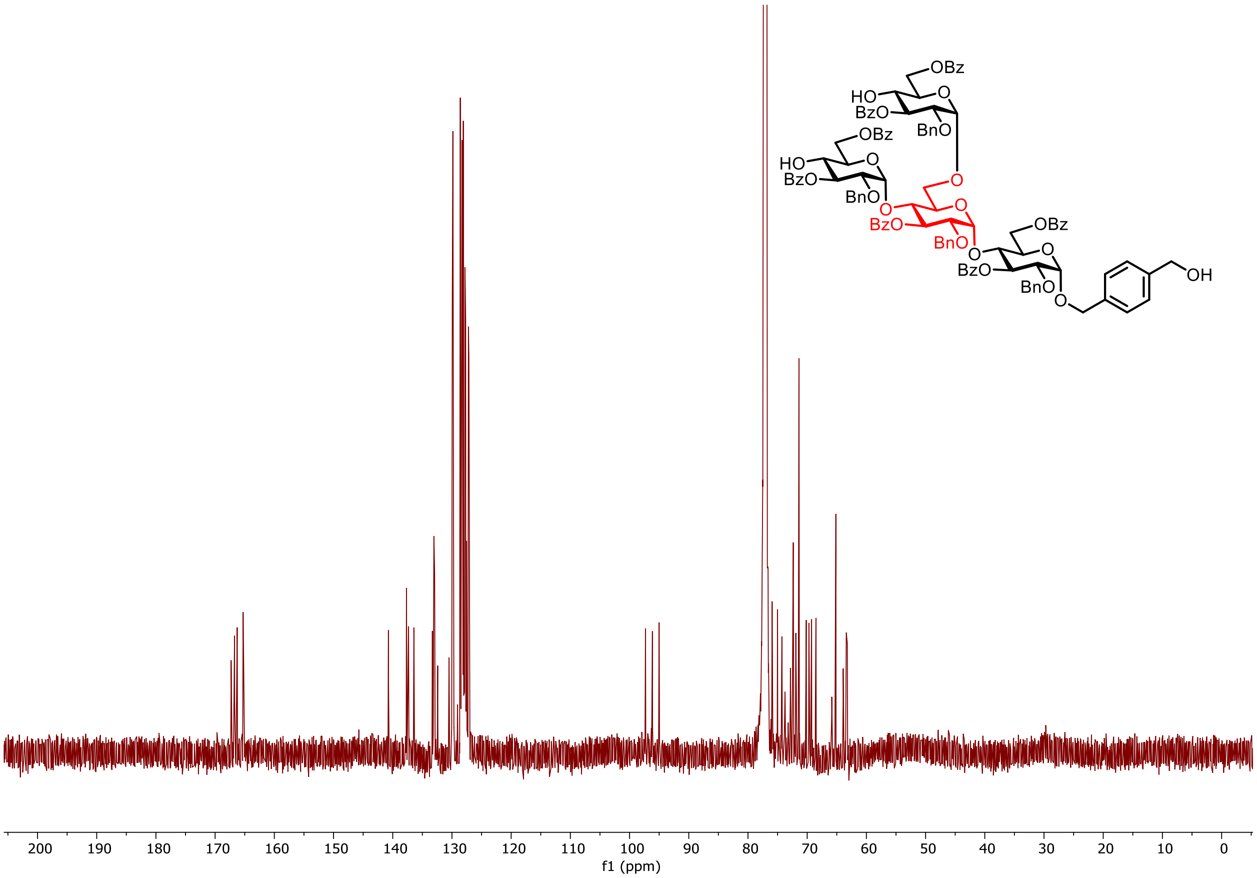

HSQC NMR of 46 ($CDCl_3$)

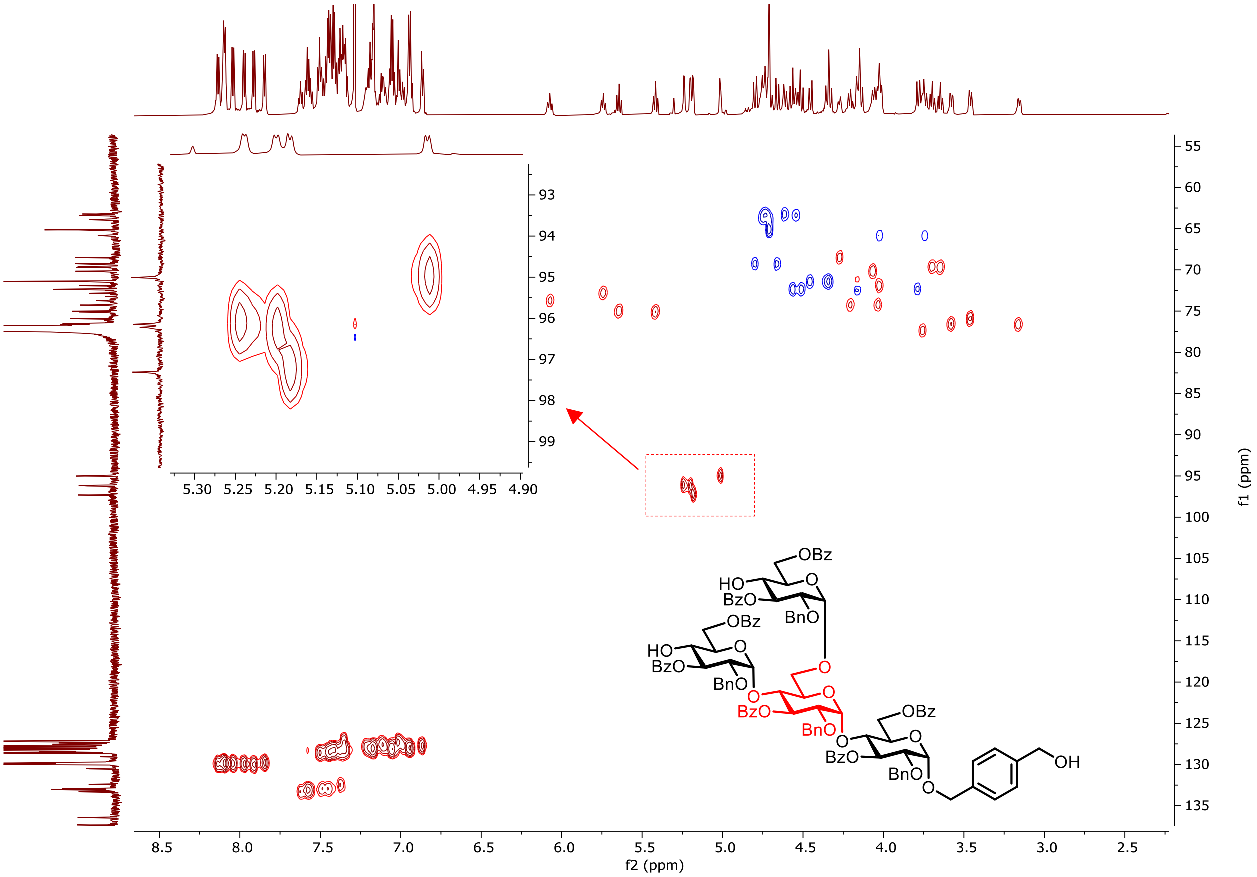

Coupled HSQC NMR of 46 ($CDCl_3$)

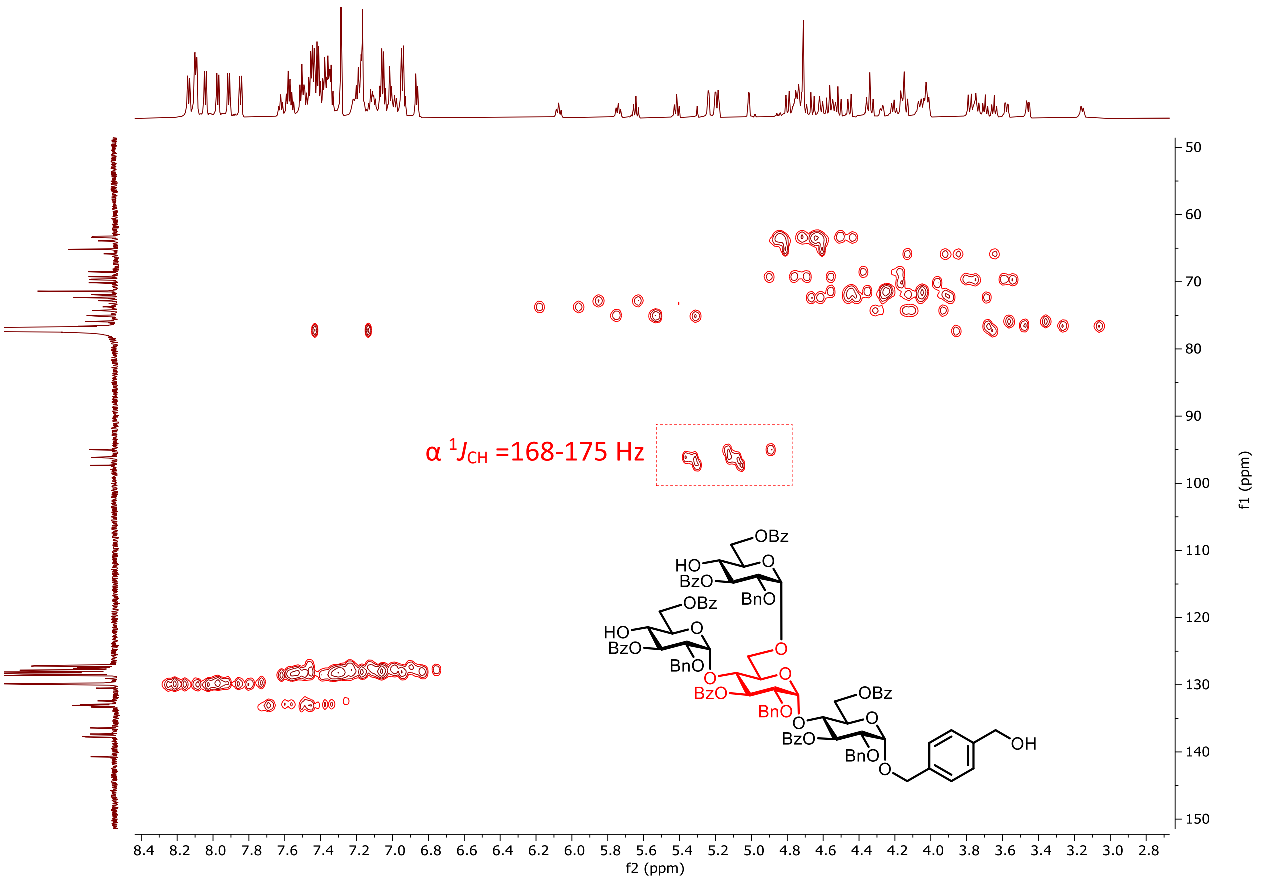

Global deprotection and purification afforded deprotected tetramer **8** as a white solid (1.2 mg, 11% overall).

Analytical data for **8**: ^{1}H NMR (700 MHz, D_2O) δ 5.38 (d, J = 3.5 Hz, 1H, α(1→4) anomeric H), 5.35 (d, J = 3.8 Hz, 1H, α(1→4) anomeric H), 5.24 (d, J = 3.8 Hz, 0.37H, α-H1), 4.99 (d, J = 3.4 Hz, α(1→6) anomeric H), 4.66 (d, J = 8.0 Hz, 0.63H, β-H1), 4.08 – 3.54 (m, 21.37H), 3.46 – 3.40 (m, 2H), 3.29 (dd, J = 9.6, 8.0 Hz, 0.63H); ^{13}C NMR (176 MHz, D_2O) δ 99.92, 99.63, 99.54, 98.76, 98.73, 95.77 (β-C1), 91.91 (α-C1), 78.17, 78.13, 77.78, 77.76, 77.51, 76.12, 74.61, 73.93, 73.12, 72.99, 72.89, 72.79, 71.89, 71.88, 71.81, 71.46, 71.45, 71.38, 71.24, 70.21, 70.00, 69.51, 69.36, 66.97, 66.95, 60.82, 60.68, 60.58, 60.42; m/z (HRMS$^+$) [M + Na]$^+$ 689.2158 ($C_{24}H_{42}O_{21}Na^+$ requires 689.2111).

RP-HPLC of 8 (ELSD trace, Method C, t_R = 5.4 min)

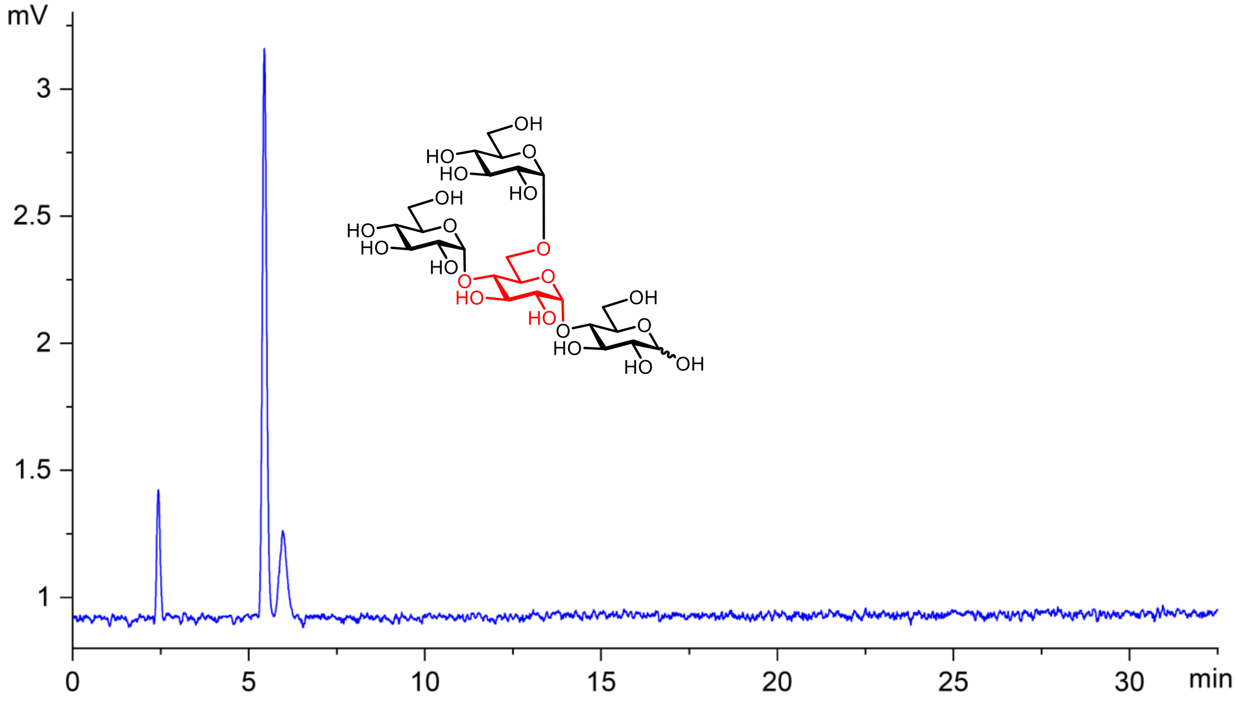

MALDI spectrum of 8

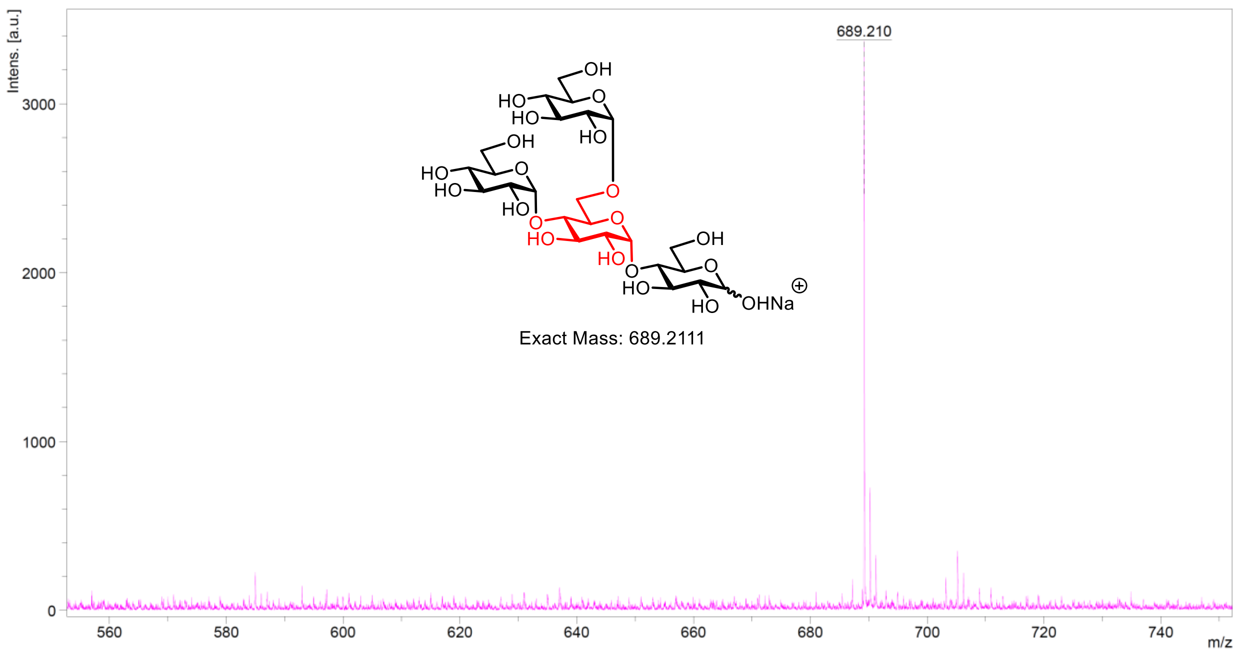

¹H NMR of 8 (700 MHz, D_2O)

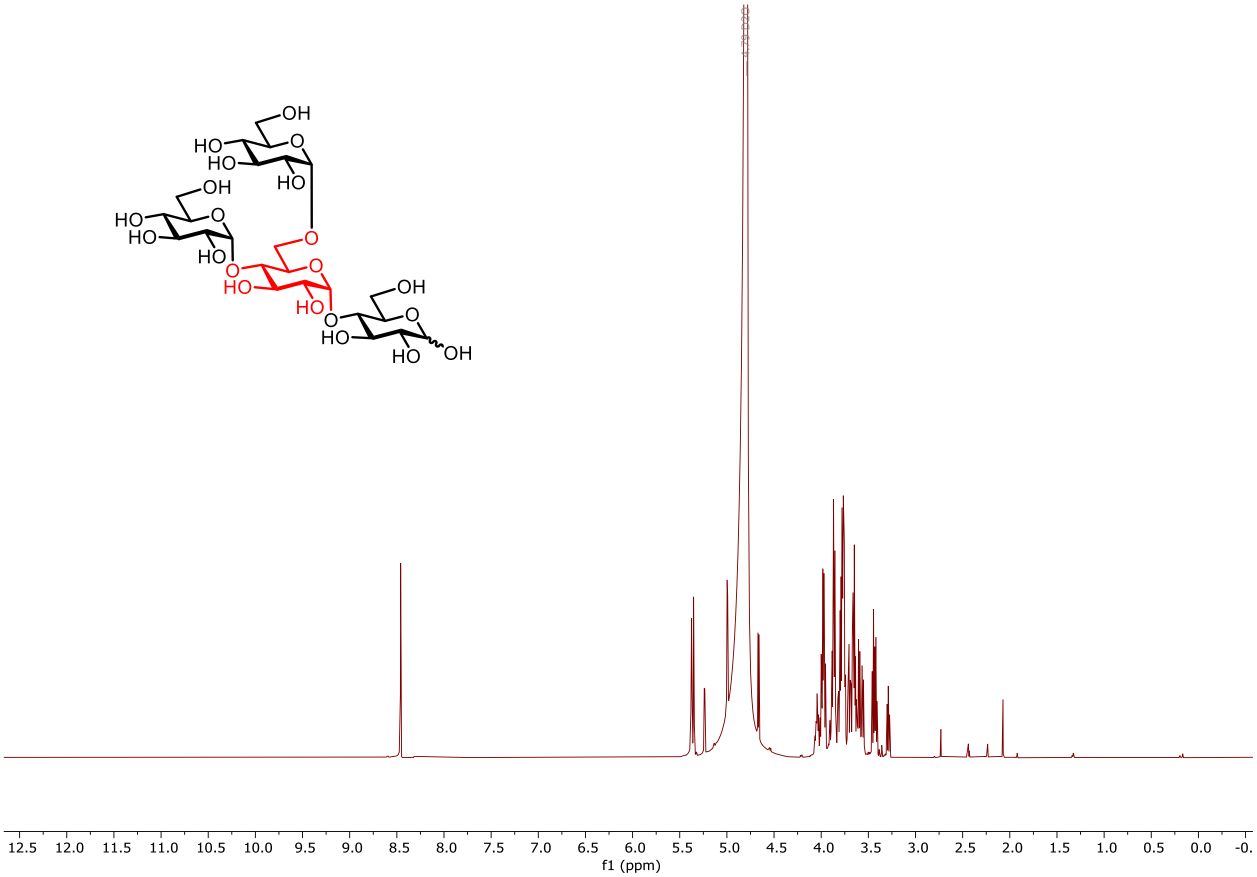

^{13}C NMR of 8 (176 MHz, D_2O)

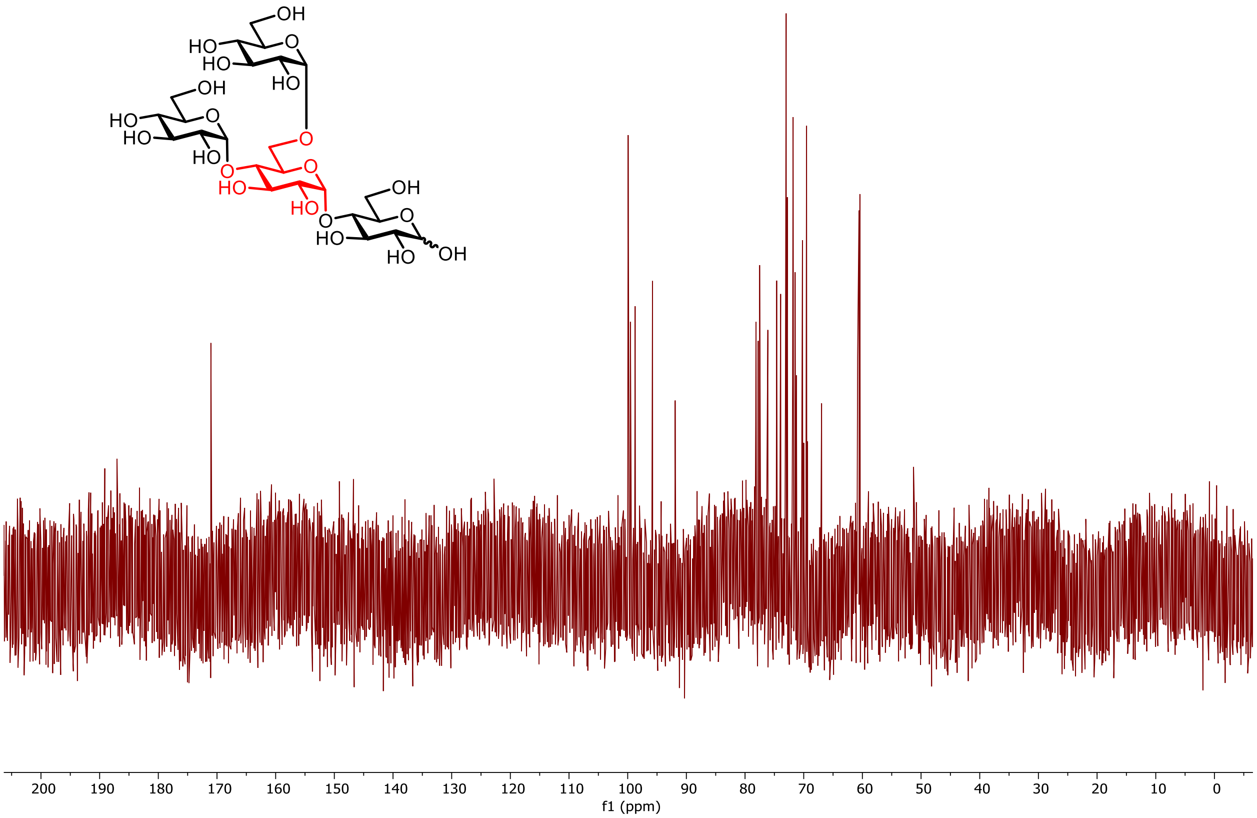

HSQC NMR of 8 (D_2O)

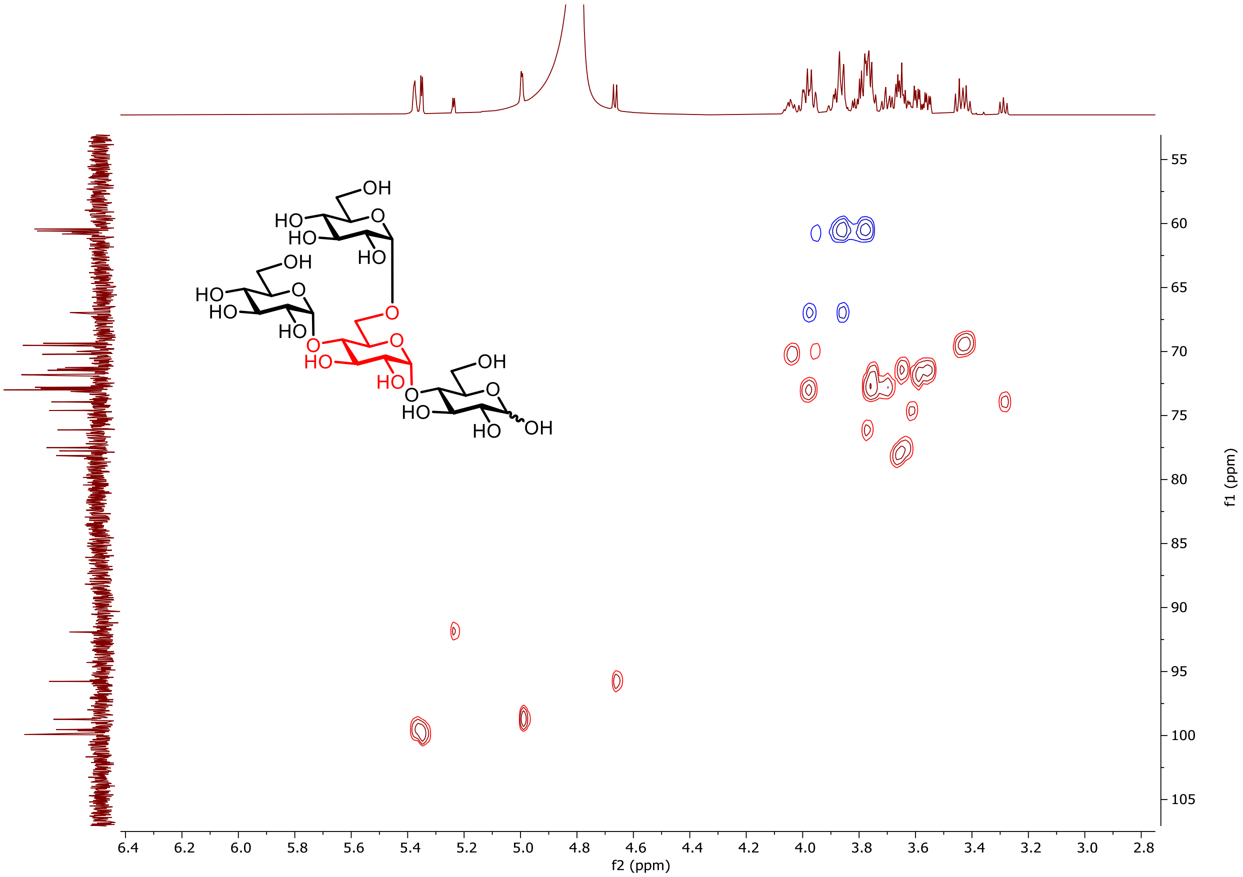

Coupled HSQC NMR of 8 (D_2O)

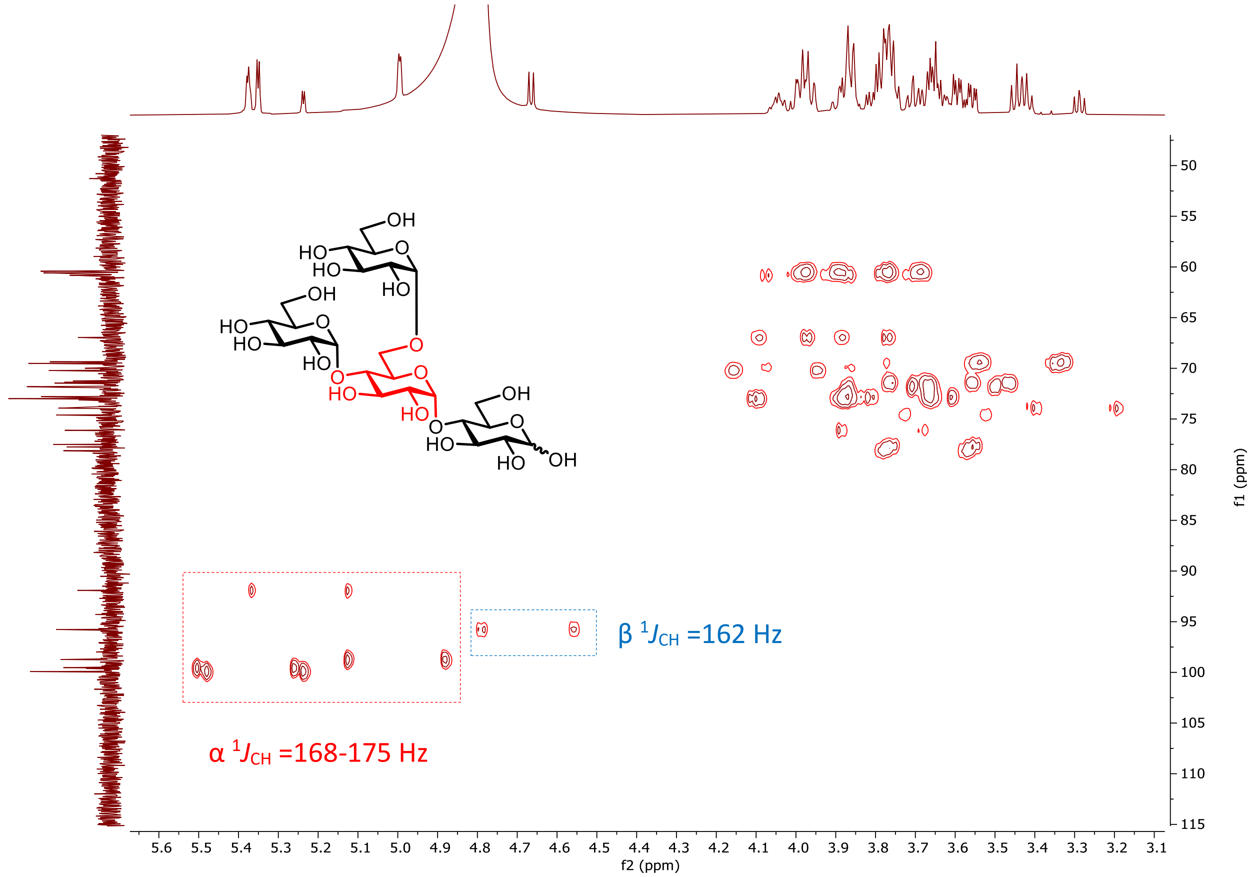

7.6 Synthesis of amylopectin heptamer 9

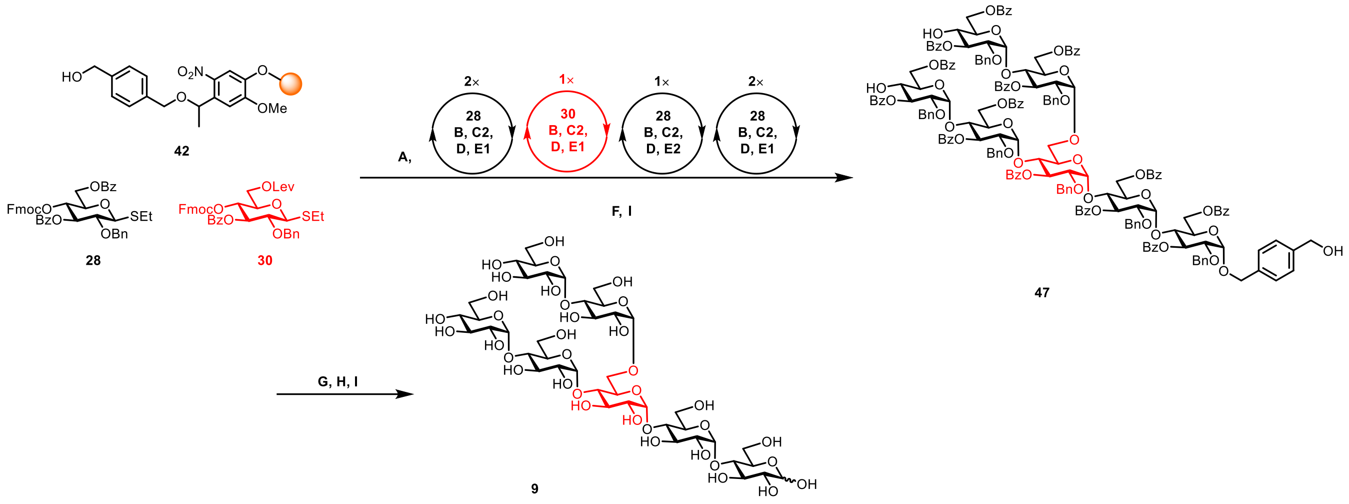

	Module	Conditions
	**A**: Resin Preparation for Synthesis	
2	**B**: Acidic Wash with TMSOTf Solution	
	**C2**: Thioglycoside Glycosylation × 2 Cycle	Building block **28**, 6.5 equiv. (-20°C for 5 min, 0°C for 60 min)
	**D**: Capping	
	**E1**: Fmoc Deprotection	
	**B**: Acidic Wash with TMSOTf Solution	
	**C2**: Thioglycoside Glycosylation × 2 Cycle	Building block **30**, 6.5 equiv. (-20°C for 5 min, 0°C for 60 min)
	**D**: Capping	
	**E1**: Fmoc Deprotection	
	**B**: Acidic Wash with TMSOTf Solution	
	**C2**: Thioglycoside Glycosylation × 2 Cycle	Building block **28**, 6.5 equiv. (-20°C for 5 min, 0°C for 60 min)
	**D**: Capping	
	**E2**: Lev Deprotection	
2	**B**: Acidic Wash with TMSOTf Solution	
	**C2**: Thioglycoside Glycosylation × 2 Cycle	Building block **28**, 6.5 equiv. (-20°C for 5 min, 0°C for 60 min)
	**D**: Capping	
	**E1**: Fmoc Deprotection	
	**F:** Cleavage from Solid Support	
	**I**: Purification	**Method E and B2**

G: Solution-phase Methanolysis

H: Hydrogenolysis at Ambient Pressure

I: Purification **Method D**

Automated synthesis and purification afforded protected heptamer **47** as a white solid (7.4 mg, 14%).

Analytical data for **47**: ^{1}H NMR (700 MHz, $CDCl_3$) δ 8.14 – 8.04 (m, 8H), 8.01 – 7.90 (m, 10H), 7.84 (d, *J* = 7.8 Hz, 2H), 7.81 (d, *J* = 7.9 Hz, 2H), 7.79 (d, *J* = 8.0 Hz, 2H), 7.73 (d, *J* = 7.6 Hz, 2H), 7.61 (appt, *J* = 7.4 Hz, 1H), 7.57 (appt, *J* = 7.4 Hz, 1H), 7.54 – 7.47 (m, 7H), 7.47 – 7.27 (m, 28H), 7.26 – 7.09 (m, 18H), 7.09 – 6.92 (m, 11H), 6.90 – 6.79 (m, 10H), 6.60 (d, *J* = 7.6 Hz, 2H), 6.06 – 5.96 (m, 3H), 5.85 – 5.78 (m, 4H), 5.57 (d, *J* = 3.3 Hz, 1H), 5.44 (d, *J* = 4.0 Hz, 1H), 5.41 (d, *J* = 3.5 Hz, 1H), 5.26 (d, *J* = 3.4 Hz, 1H), 5.24 (d, *J* = 3.6 Hz, 1H), 5.22 – 5.18 (m, 2H), 5.10 (d, *J* = 12.4 Hz, 1H), 4.93 (d, *J* = 3.5 Hz, 1H), 4.82 (d, *J* = 12.3 Hz, 1H), 4.77 (appt, *J* = 12.8 Hz, 3H), 4.71 (s, 2H), 4.68 – 4.31 (m, 21H), 4.30 – 4.21 (m, 4H), 4.17 (appt, *J* = 10.9 Hz, 2H), 4.14 – 4.08 (m, 2H), 4.05 (dd, *J* = 13.3, 8.8 Hz, 3H), 3.98 (d, *J* = 11.9 Hz, 1H), 3.83 (d, *J* = 11.9 Hz, 1H), 3.79 (d, *J* = 13.4 Hz, 1H), 3.55 (dq, *J* = 12.9, 9.0, 8.6 Hz, 6H), 3.47 (dd, *J* = 8.7, 3.6 Hz, 1H), 3.38 (dd, *J* = 9.3, 3.4 Hz, 1H), 3.32 (dd, *J* = 10.0, 3.5 Hz, 1H), 3.19 (dd, *J* = 9.9, 3.5 Hz, 1H); ^{13}C NMR (176 MHz, $CDCl_3$) δ 167.75, 167.13, 166.30, 166.26, 165.90, 165.74, 165.59, 165.54, 165.51, 165.45, 165.35, 165.25, 164.76, 140.69, 138.18, 137.65, 137.35, 137.33, 137.31, 137.06, 136.37, 133.09, 133.04, 133.03, 132.93, 132.90, 132.87, 132.84, 132.78, 132.58, 132.55, 132.27, 130.62, 130.36, 130.27, 130.18, 130.16, 130.13, 130.04, 130.00, 129.98, 129.94, 129.90, 129.86, 129.77, 129.72, 129.70, 129.67, 129.61, 129.50, 128.52, 128.47, 128.42, 128.40, 128.35, 128.33, 128.29, 128.26, 128.23, 128.18, 128.12, 128.03, 127.96, 127.93, 127.90, 127.87, 127.74, 127.71, 127.47, 127.45, 127.31, 127.18, 127.13, 127.09, 97.31, 96.85, 96.27, 95.54, 94.91, 94.08, 93.36, 76.28, 75.50, 75.25, 74.90, 74.59, 74.45, 74.35, 74.29, 73.91, 72.80, 72.35, 72.25, 71.90, 71.65, 71.43, 71.32, 71.09, 70.73, 70.66, 70.59, 70.15, 70.02, 69.68, 69.41, 69.16, 68.93, 68.67, 65.12, 64.13, 63.22, 63.12, 62.90; m/z (HRMS^{+}) $[M + 2Na]^{2+}$ 1651.584 ($C_{190}H_{174}O_{50}Na_2^{2+}$ requires 1651.546).

NP-HPLC of crude 47 after AGA (ELSD trace, Method A2)

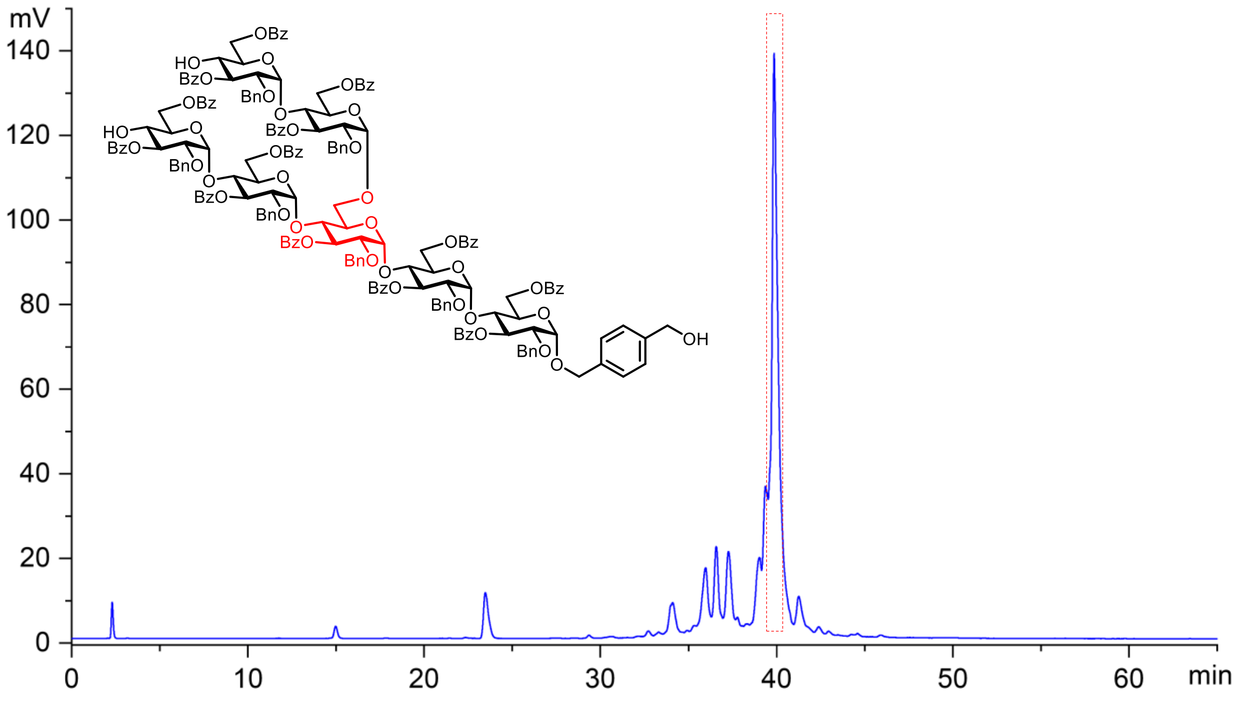

NP-HPLC of pure 47 (ELSD trace, Method A2, t_R = 37.2 min)

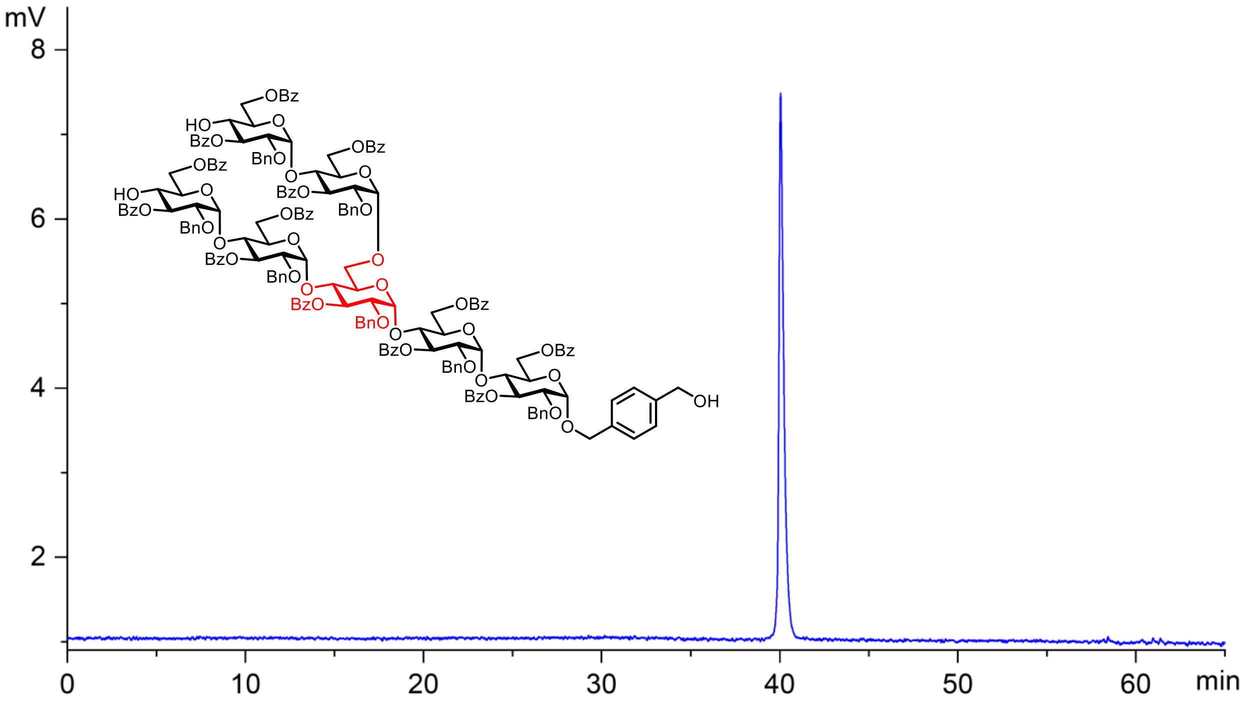

MALDI spectrum of 47

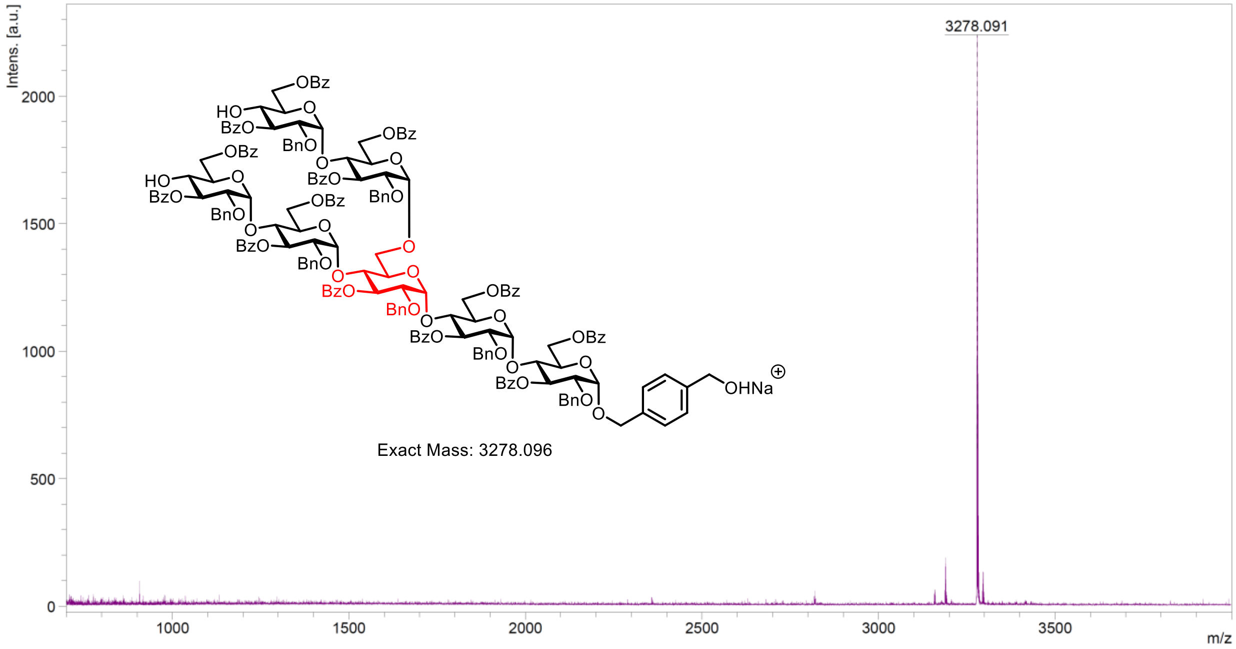

^1H NMR of 47 (700 MHz, $CDCl_3$)

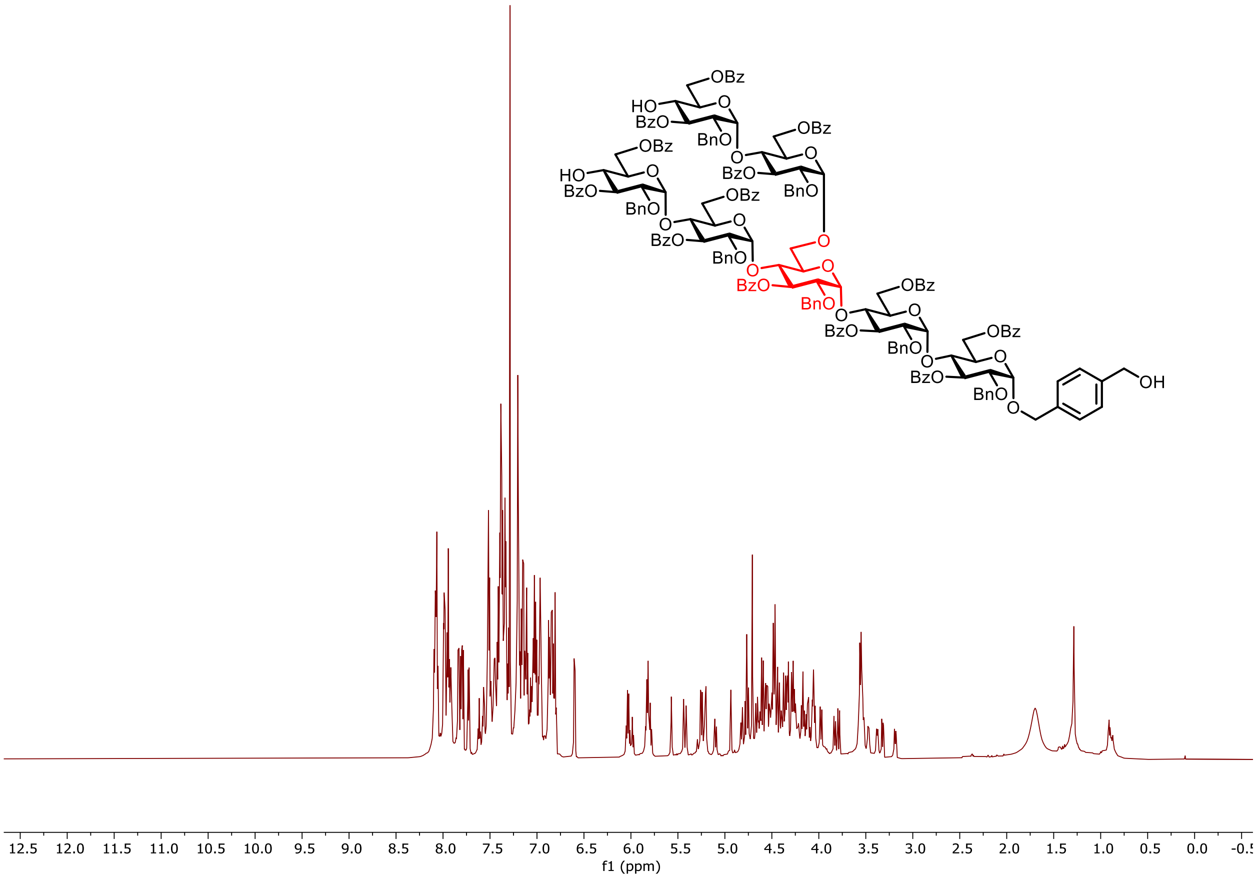

^{13}C NMR of 47 (176 MHz, $CDCl_3$)

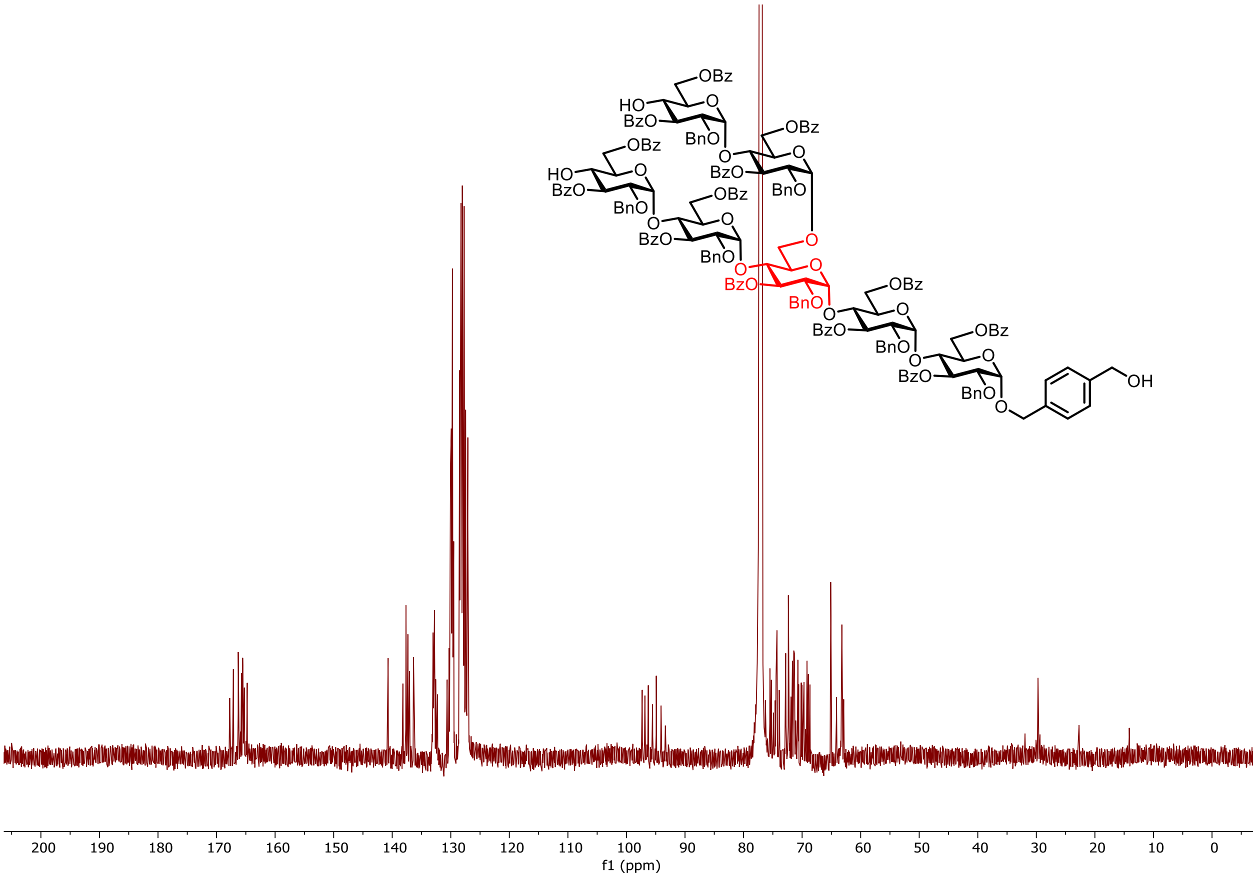

HSQC NMR of 47 ($CDCl_3$)

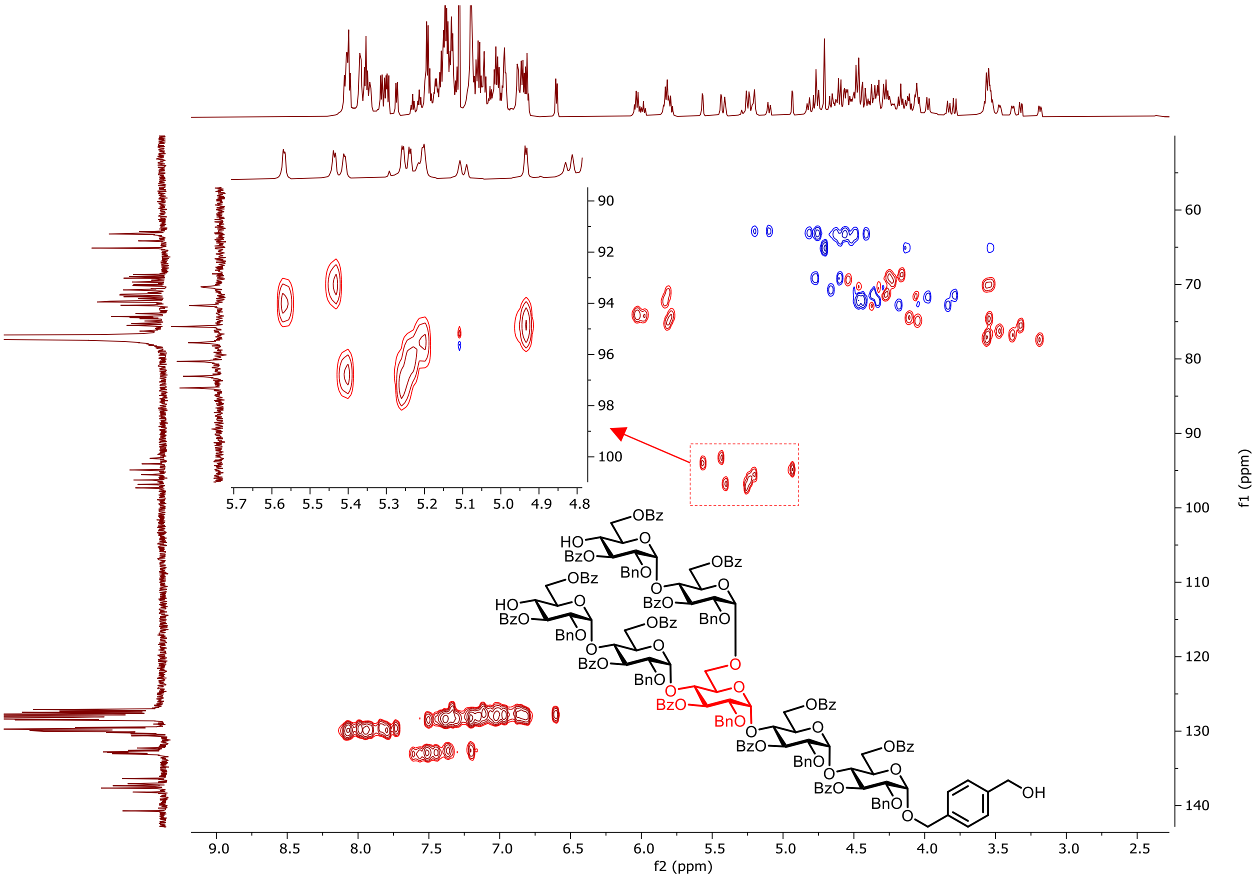

Coupled HSQC NMR of 47 ($CDCl_3$)

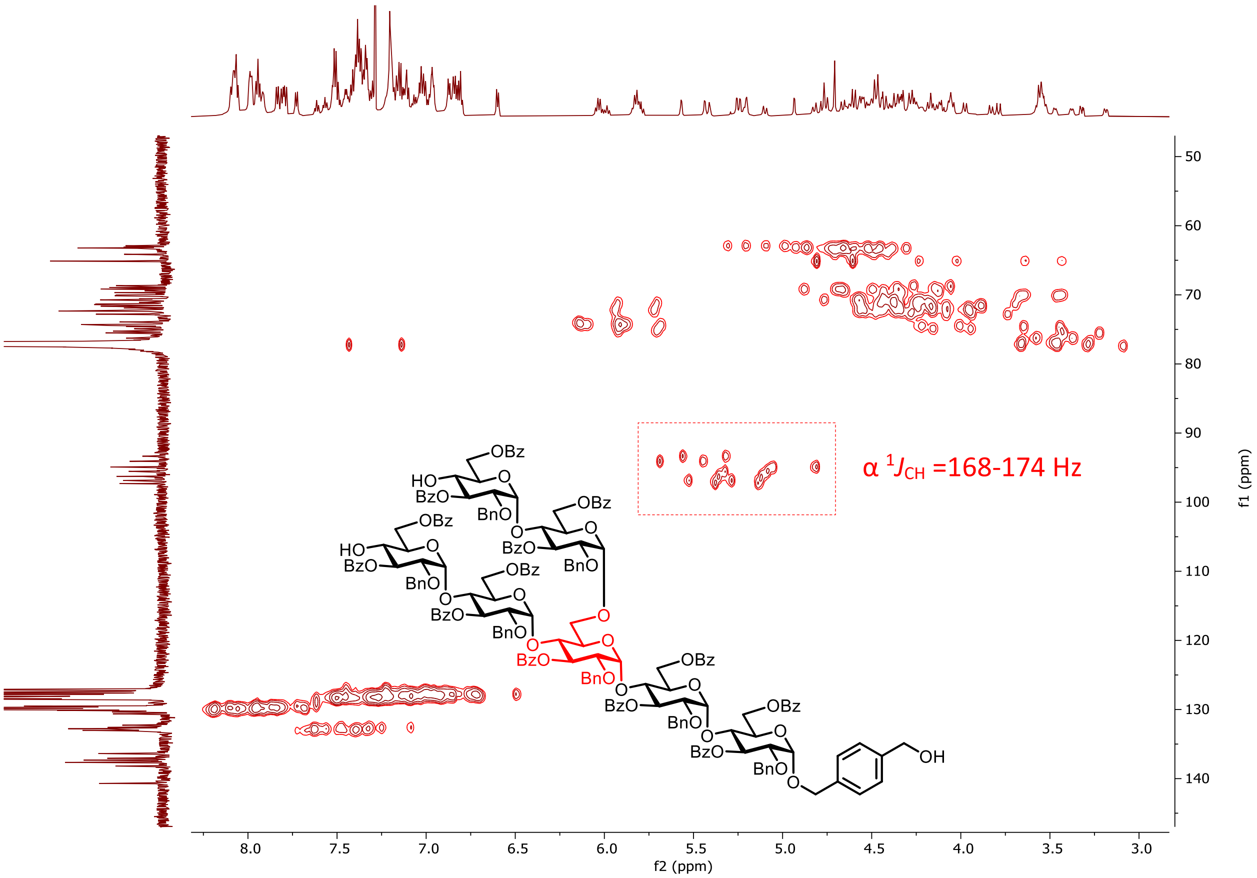

HSQC NMR analysis of impurity (showed in HPLC purple square) during synthesis of 47 ($CDCl_3$)

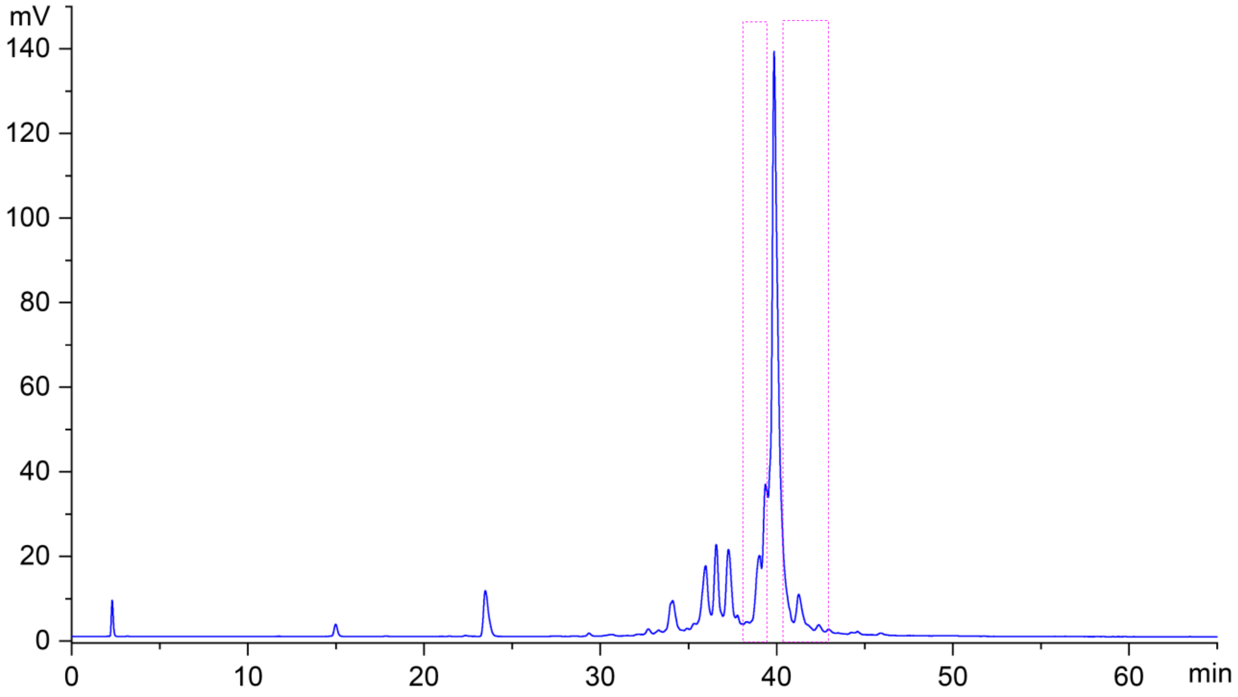

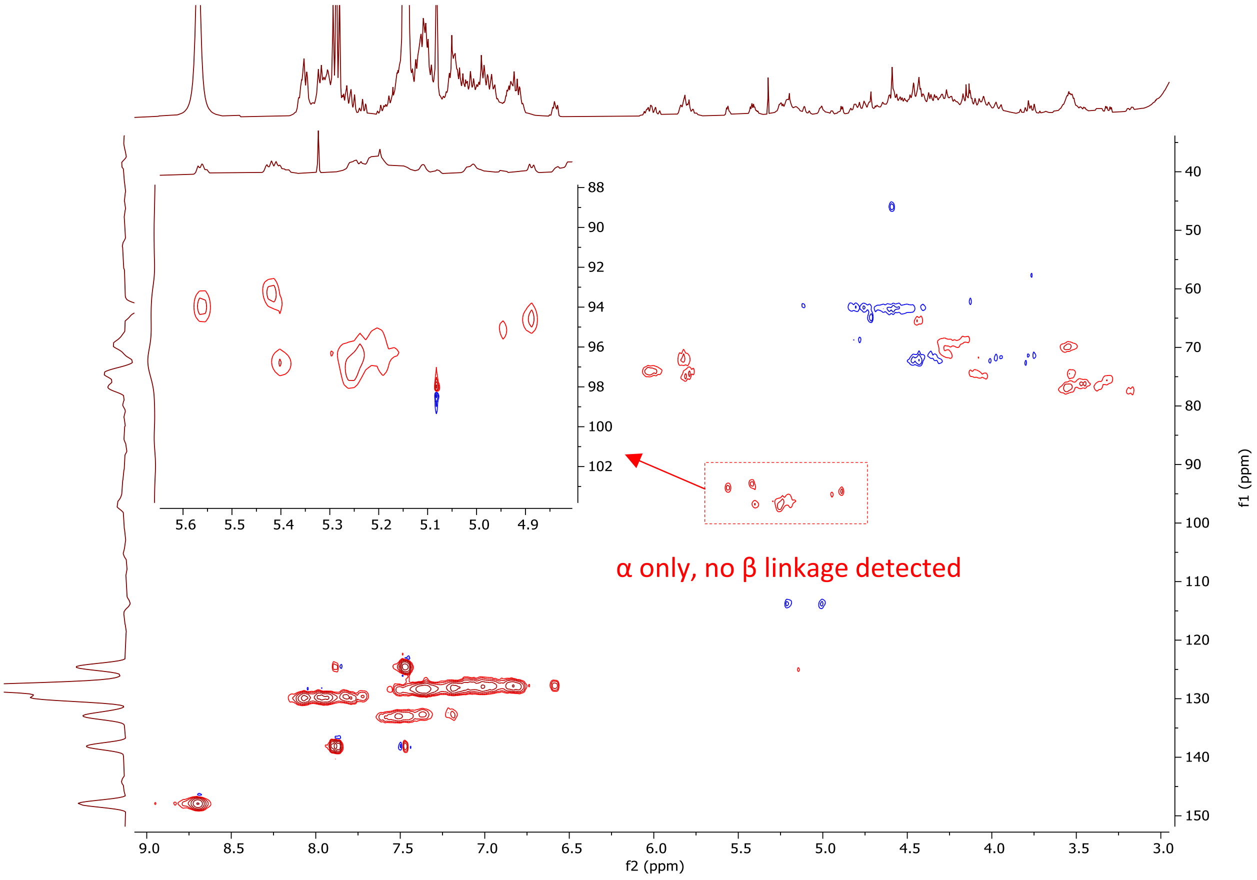

Global deprotection and purification afforded deprotected heptamer **9** as a white solid (1.9 mg, 10% overall). Analytical data for **9**: ^{1}H NMR (700 MHz, D_2O) δ 5.42 (d, *J* = 4.0 Hz, 1H, α(1→4) anomeric H), 5.41 (d, *J* = 3.9 Hz, 1H, α(1→4) anomeric H), 5.38 (d, *J* = 3.9 Hz, 1H, α(1→4) anomeric H), 5.35 (d, *J* = 3.9 Hz, 1H, α(1→4) anomeric H), 5.34 (d, *J* = 3.9 Hz, 1H, α(1→4) anomeric H), 5.24 (d, *J* = 3.8 Hz, 0.35H, α-H1), 4.98 (d, *J* = 3.7 Hz, 1H, α(1→6) anomeric H), 4.67 (d, *J* = 8.0 Hz, 0.65H, β-H1), 4.07 – 3.55 (m, 39.35H), 3.46 – 3.40 (m, 2H), 3.28 (dd, *J* = 9.6, 8.0 Hz, 0.65H); ^{13}C NMR (176 MHz, D_2O) δ 99.99, 99.97, 99.90, 99.72, 99.47, 99.38, 98.53, 95.77 (β-C1), 91.90 (α-C1), 78.74, 77.88, 77.90, 77.46, 77.01, 76.79, 76.77, 76.18, 74.55, 74.00, 73.35, 73.34, 73.23, 73.20, 73.00, 72.94, 72.86, 72.74, 72.73, 71.85, 71.73, 71.69, 71.53, 71.43, 71.32, 71.30, 71.17, 70.39, 70.27, 69.95, 69.33, 69.31, 67.48, 61.48, 60.70, 60.69, 60.58, 60.56, 60.50, 60.49, 60.40; m/z (HRMS$^+$) [M + Na]$^+$ 1175.383 ($C_{42}H_{72}O_{36}Na^+$ requires 1175.369).

RP-HPLC of 9 (ELSD trace, Method C, t_R = 14.3 min)

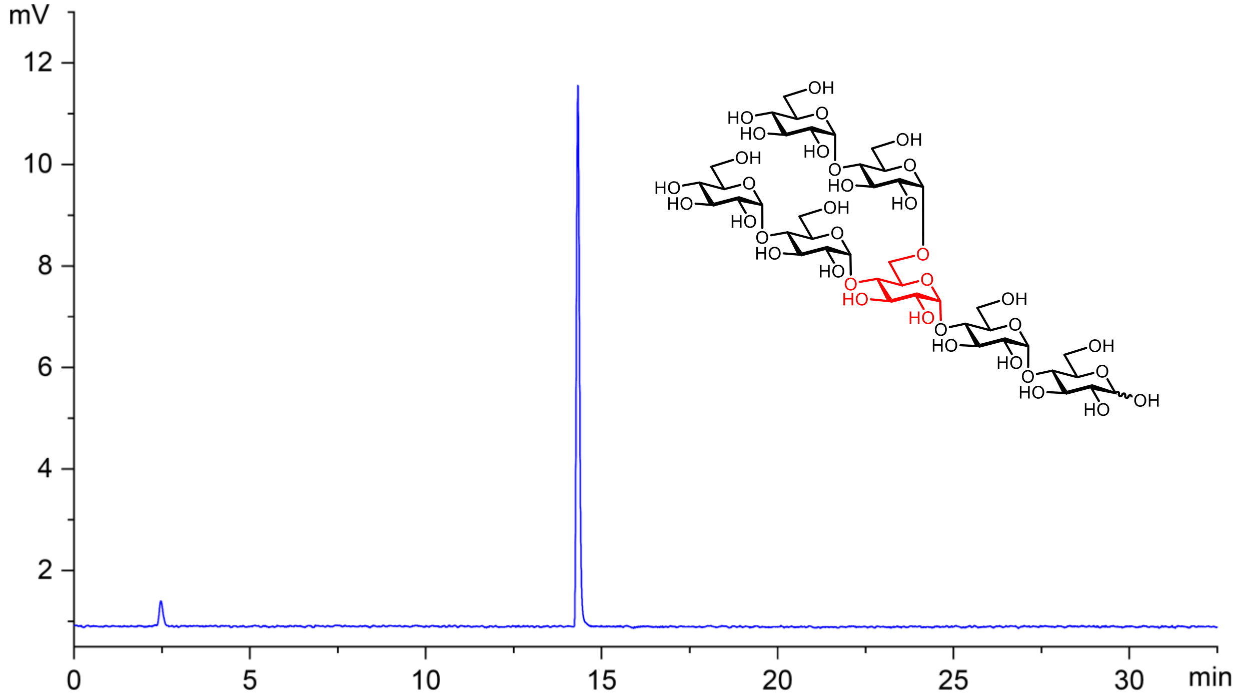

MALDI spectrum of 9

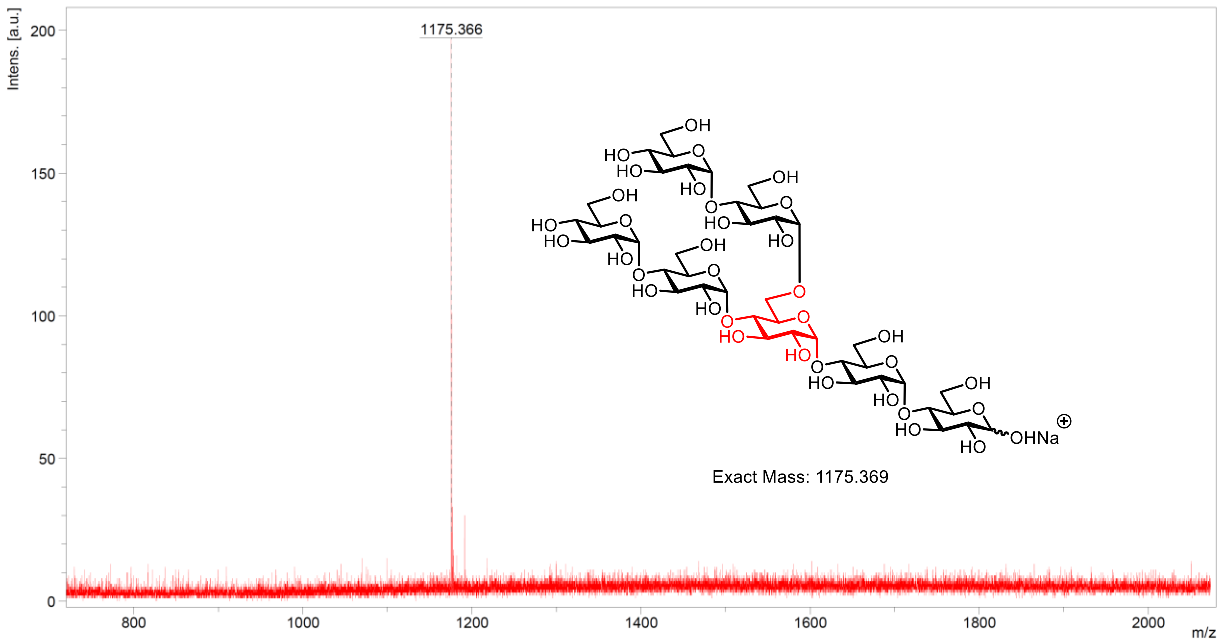

^{1}H NMR of 9 (700 MHz, D_2O)

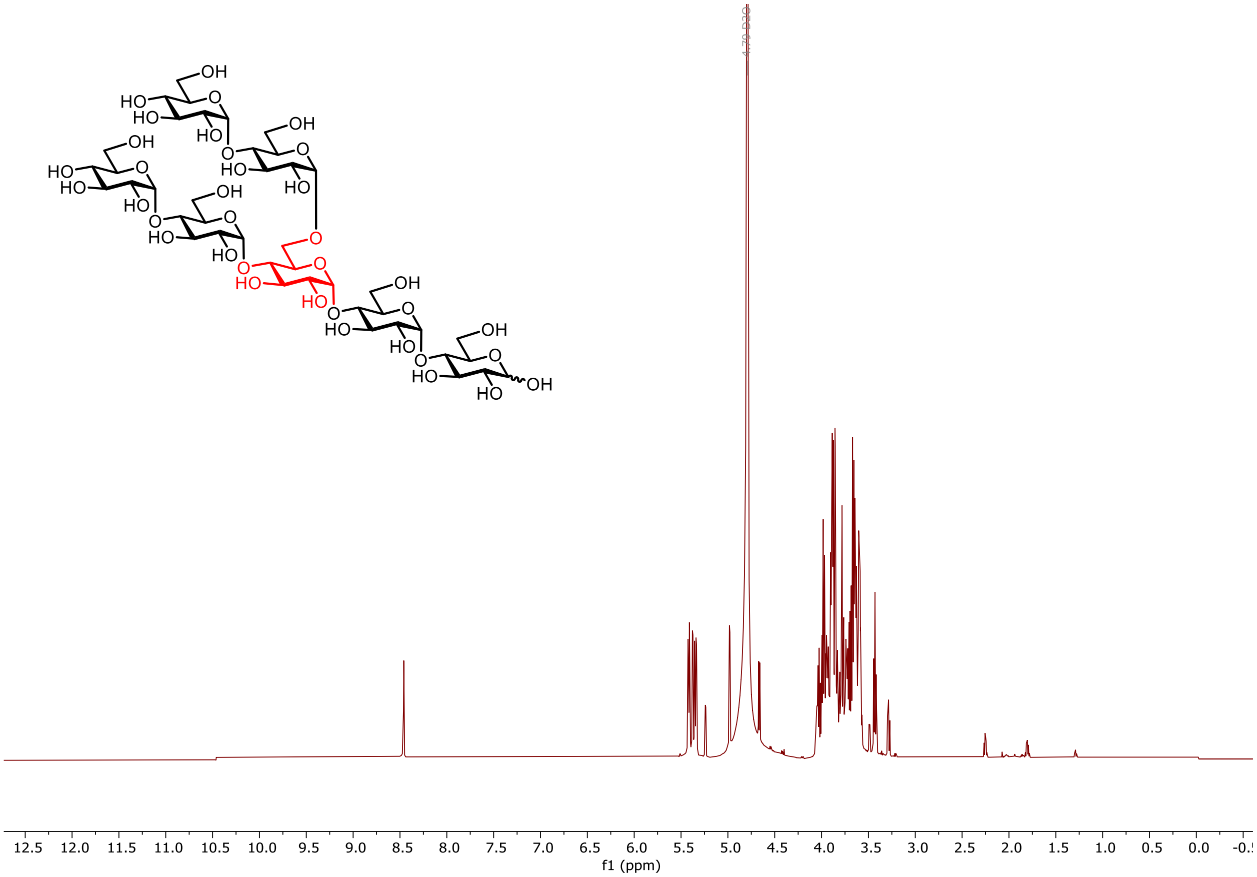

^{13}C NMR of 9 (176 MHz, D_2O)

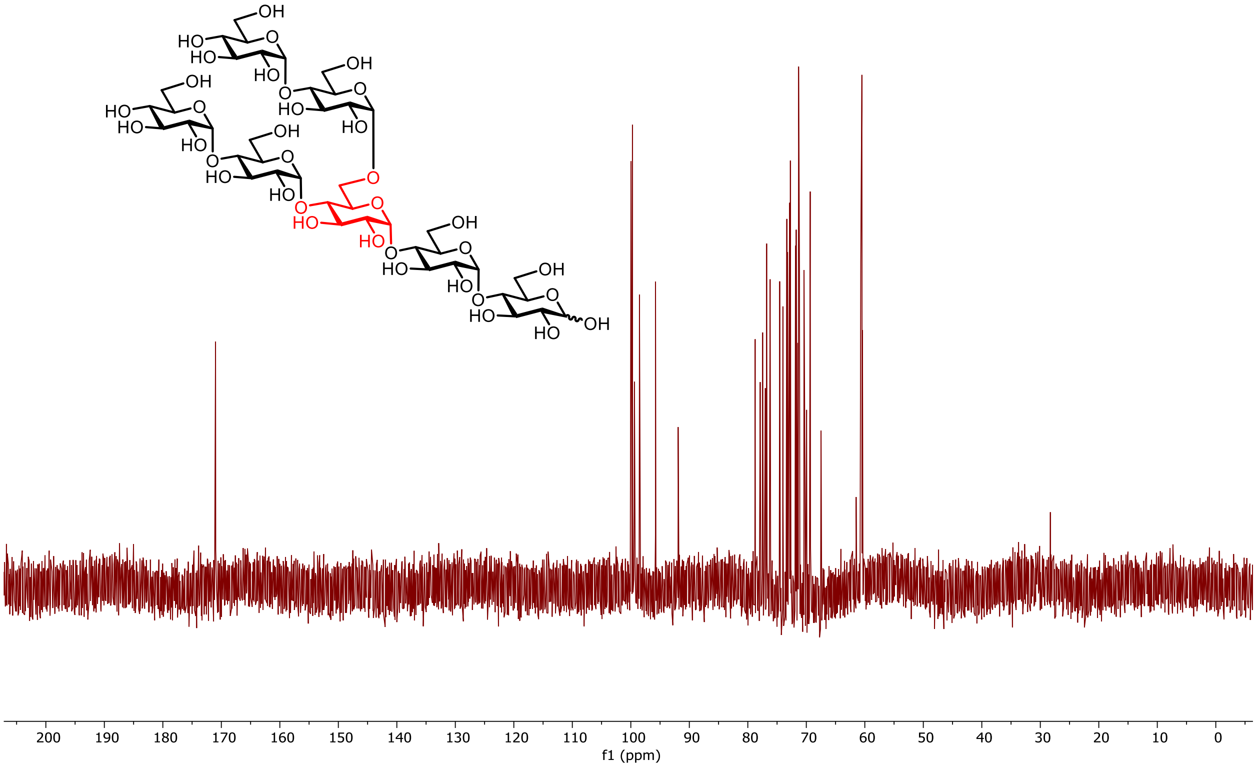

HSQC NMR of 9 (D_2O)

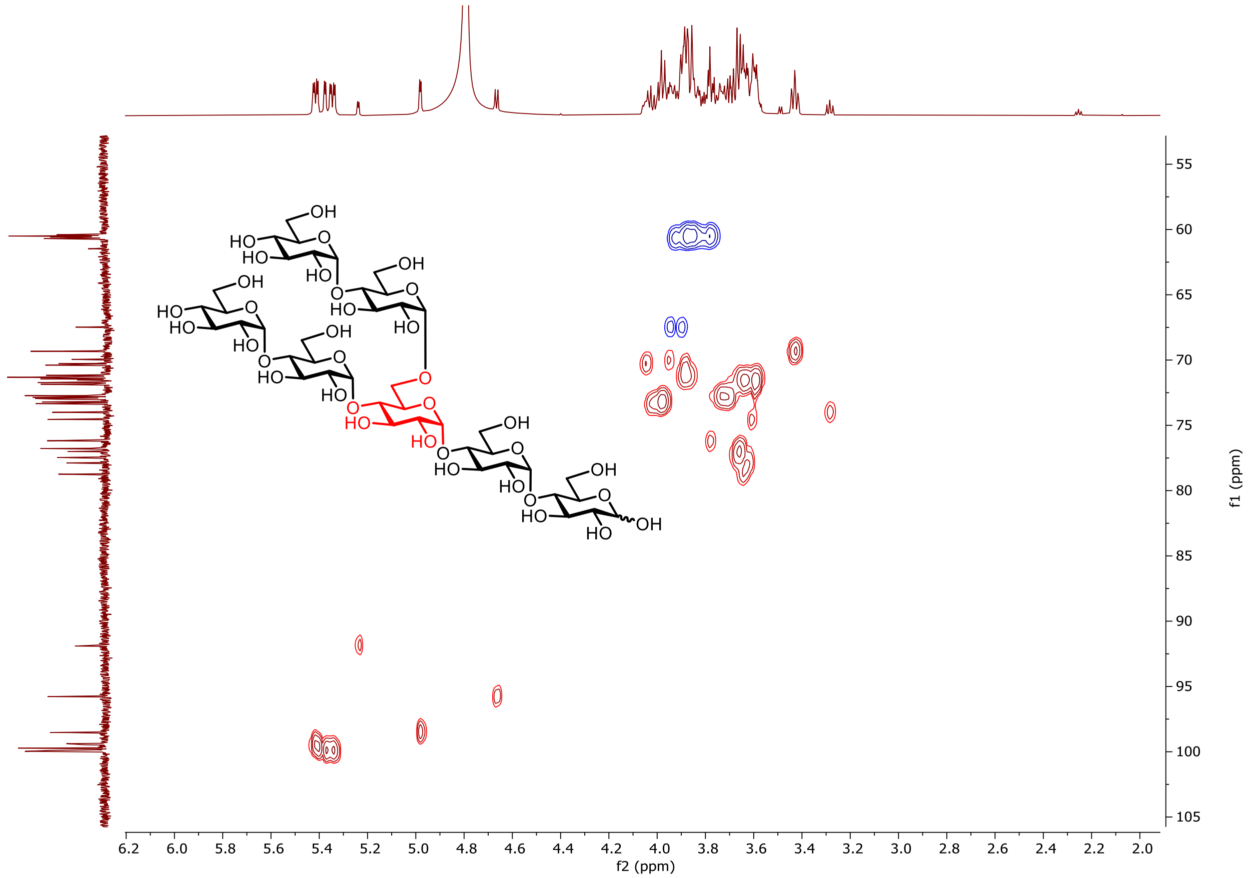

Coupled HSQC NMR of 9 (D_2O)

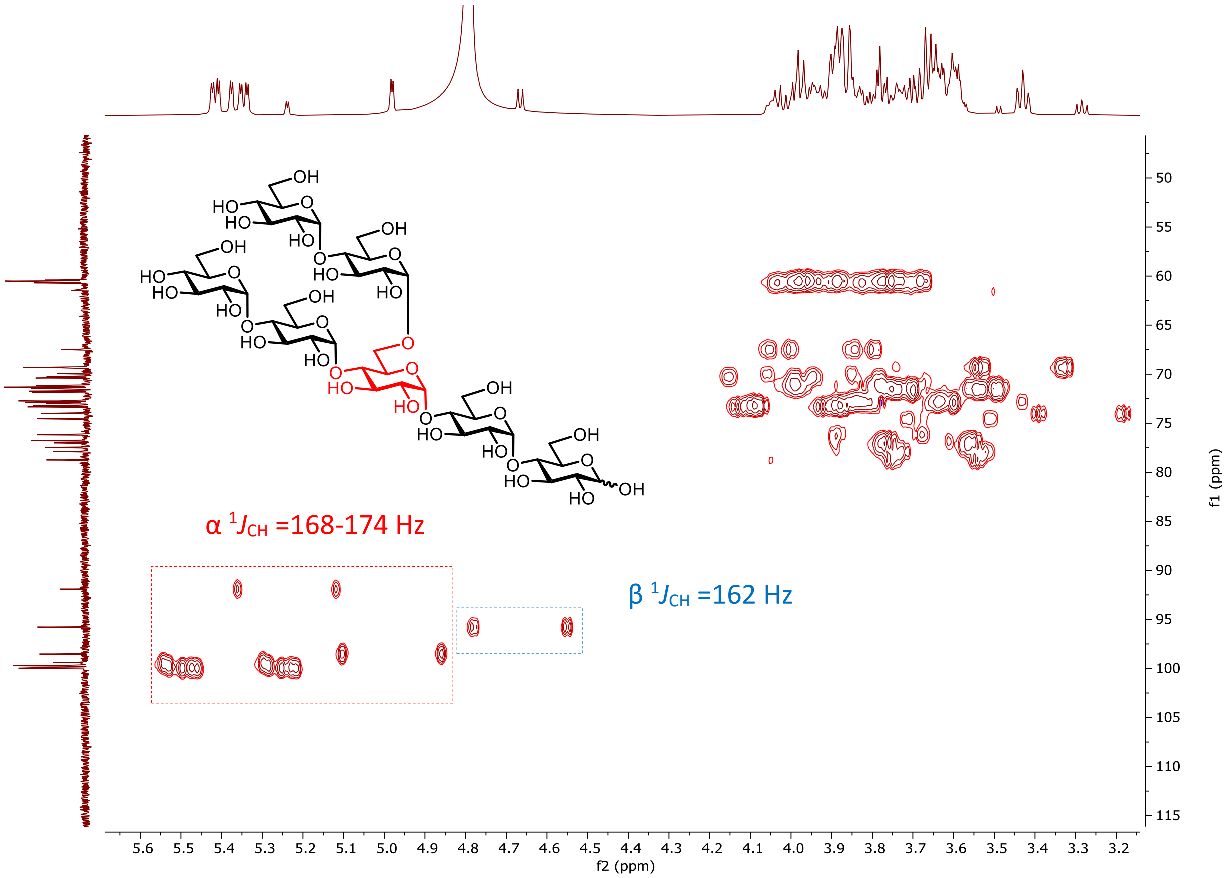

7.7 Synthesis of amylopectin 20-mer 10

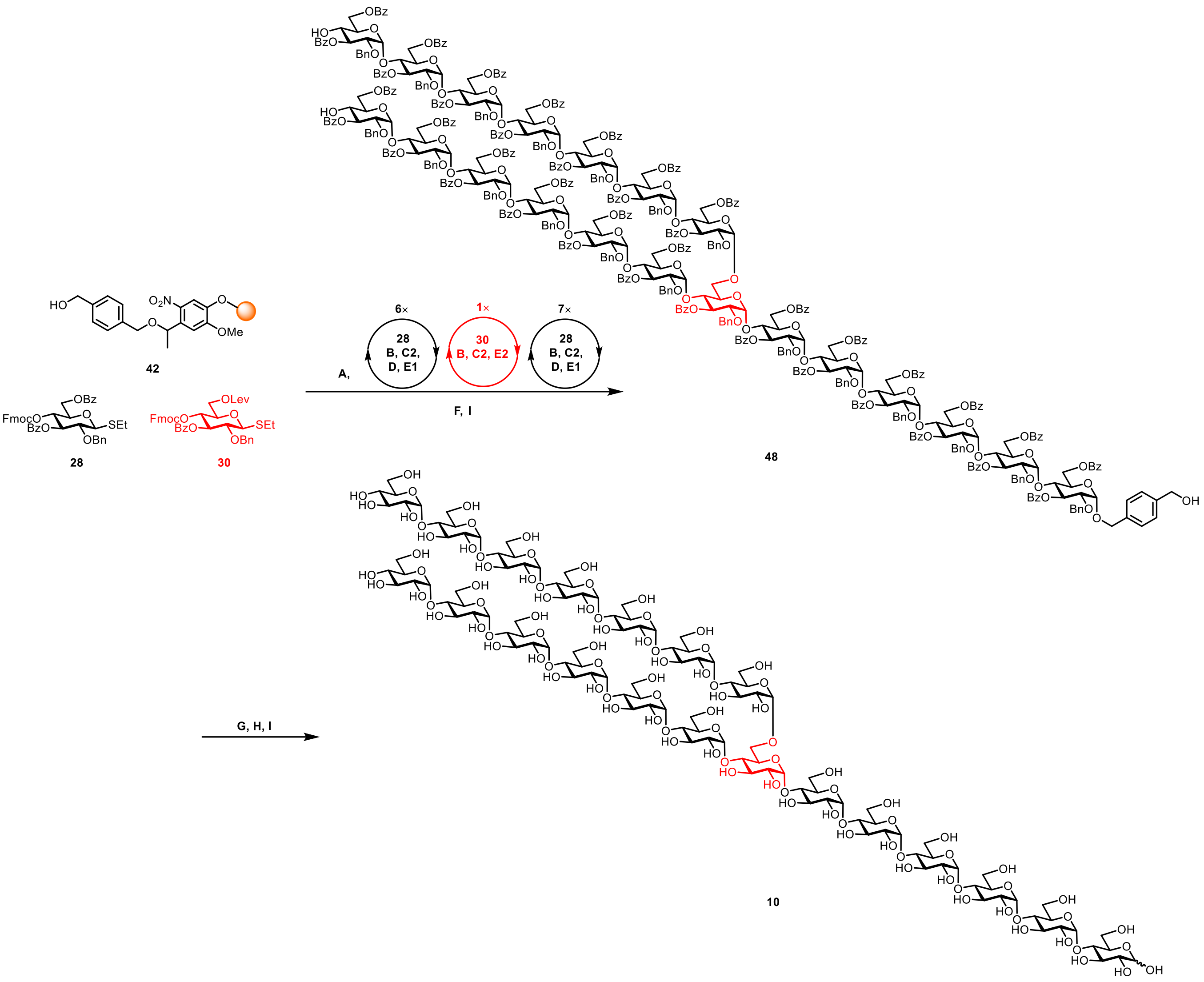

	Module	Conditions
	**A**: Resin Preparation for Synthesis	
6	**B**: Acidic Wash with TMSOTf Solution	
6	**C2**: Thioglycoside Glycosylation × 2 Cycle	Building block **28**, 6.5 equiv. (-20°C for 5 min, 0°C for 60 min)
6	**D**: Capping	
6	**E1**: Fmoc Deprotection	
	**B**: Acidic Wash with TMSOTf Solution	
	**C2**: Thioglycoside Glycosylation × 2 Cycle	Building block **30**, 6.5 equiv. (-20°C for 5 min, 0°C for 60 min)
	**E2**: Lev Deprotection	

7	**B**: Acidic Wash with TMSOTf Solution	
	**C2**: Thioglycoside Glycosylation × 2 Cycle	Building block **28**, 6.5 equiv. (-20°C for 5 min, 0°C for 60 min)
	**D**: Capping	
	**E1**: Fmoc Deprotection	
	**F:** Cleavage from Solid Support	
	**I**: Purification	**Method E and B3**
	**G**: Solution-phase Methanolysis	
	**H**: Hydrogenolysis at Ambient Pressure	
	**I**: Purification	**Method D**

Automated synthesis and purification afforded protected 20-mer **48** as a white solid (36 mg, 24%).

Analytical data for **48**: ^{1}H NMR (700 MHz, $CDCl_3$) δ 8.15 (d, *J* = 7.7 Hz, 2H), 8.11 (d, *J* = 7.6 Hz, 2H), 8.05 (d×2, *J* = 7.6 Hz, 4H), 8.02 (d, *J* = 7.7 Hz, 2H), 8.01 – 7.80 (m, 62H), 7.72 (d, *J* = 7.8 Hz, 2H), 7.62 – 7.55 (m, 4H), 7.54 – 7.41 (m, 32H), 7.41 – 7.17 (m, 60H), 7.16 – 6.81 (m, 123H), 6.78 – 6.68 (m, 6H), 6.05 – 5.96 (m, 2H), 5.81 – 5.67 (m, 16H), 5.48 (appt, *J* = 9.5 Hz, 2H), 5.38 – 5.18 (m, 19H), 4.93 (d, *J* = 3.4 Hz, 1H), 4.82 – 4.40 (m, 44H), 4.41 – 4.29 (m, 9H), 4.28 – 4.09 (m, 38H), 4.09 – 3.92 (m, 30H), 3.74 – 3.65 (m, 2H), 3.54 (appt, *J* = 9.4 Hz, 3H), 3.52 – 3.38 (m, 8H), 3.38 – 3.24 (m, 9H), 3.17 – 3.13 (m, 1H); ^{13}C NMR (176 MHz, $CDCl_3$) δ 167.17, 166.50, 166.00, 165.98, 165.93, 165.91, 165.87, 165.84, 165.76, 165.72, 165.69, 165.63, 165.54, 165.34, 165.27, 165.19, 165.06, 165.00, 164.95, 164.90, 164.71, 164.63, 140.69, 138.16, 137.66, 137.58, 137.55, 137.50, 137.48, 137.44, 137.41, 137.38, 137.28, 137.24, 136.42, 133.13, 132.94, 132.89, 132.85, 132.67, 130.33, 130.29, 130.04, 130.00, 129.97, 129.94, 129.90, 129.87, 129.83, 129.80, 129.77, 129.73, 129.68, 129.65, 128.58, 128.53, 128.49, 128.36, 128.28, 128.26, 128.21, 128.16, 128.11, 128.07, 128.01, 127.96, 127.92, 127.88, 127.85, 127.77, 127.74, 127.72, 127.69, 127.59, 127.44, 127.42, 127.38, 127.24, 127.19, 127.10, 126.84, 126.67, 97.19, 96.90, 96.77, 96.22, 96.11, 95.91, 95.86, 95.82, 95.77, 95.68, 95.64, 95.55, 95.43, 95.20, 95.06, 94.88, 94.84, 94.75, 77.65, 77.45, 76.11, 76.02, 75.92, 75.85, 75.70, 75.66, 75.26, 74.94, 74.83, 74.14, 74.00, 73.93, 73.71, 73.50, 73.46, 73.43, 73.20, 73.10, 72.80, 72.36, 72.30, 72.26, 72.21, 72.14, 72.04, 71.93, 71.49, 71.36, 70.85, 69.92, 69.87, 69.85, 69.77, 69.70, 69.59, 69.44, 69.33, 69.11, 68.85, 68.50, 65.16, 63.38, 63.15, 63.07, 62.97, 62.88, 62.82, 62.72; m/z (HRMS^{+}) $[M + 3Na]^{3+}$ 3103.152 ($C_{541}H_{486}O_{141}Na_3^{3+}$ requires 3103.021).

NP-HPLC of crude 48 after AGA (ELSD trace, Method A3)

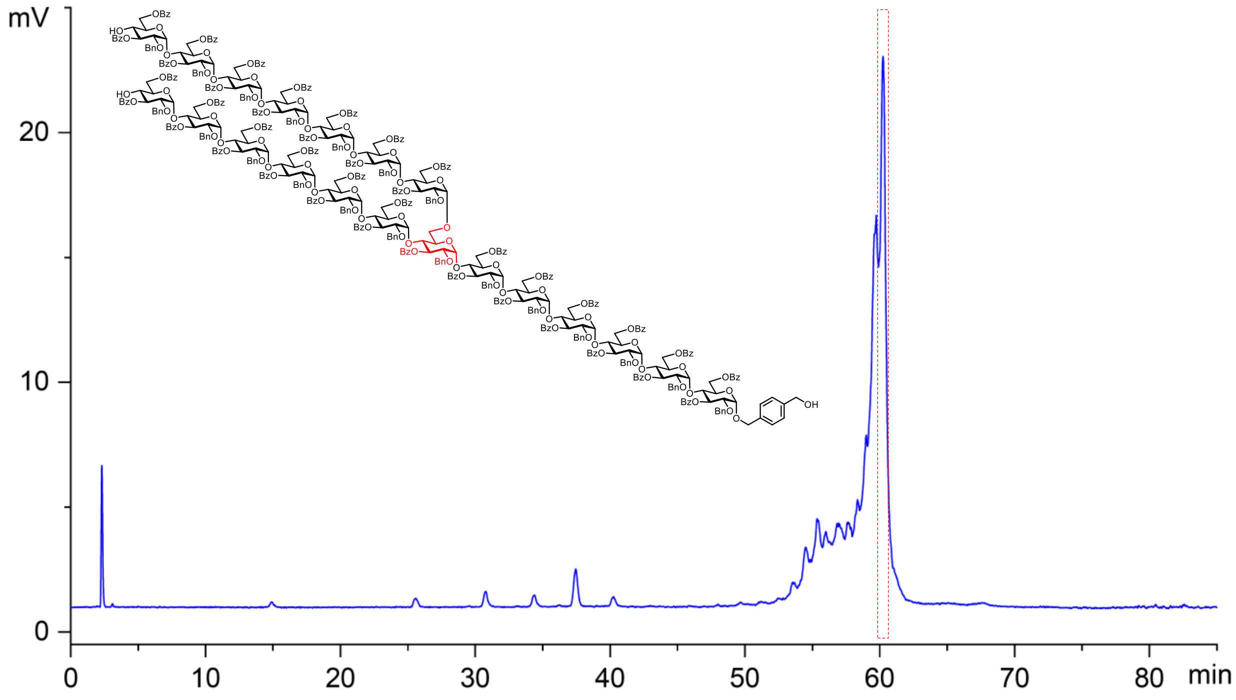

NP-HPLC of pure 48 (ELSD trace, Method A3, t_R = 60.7 min)

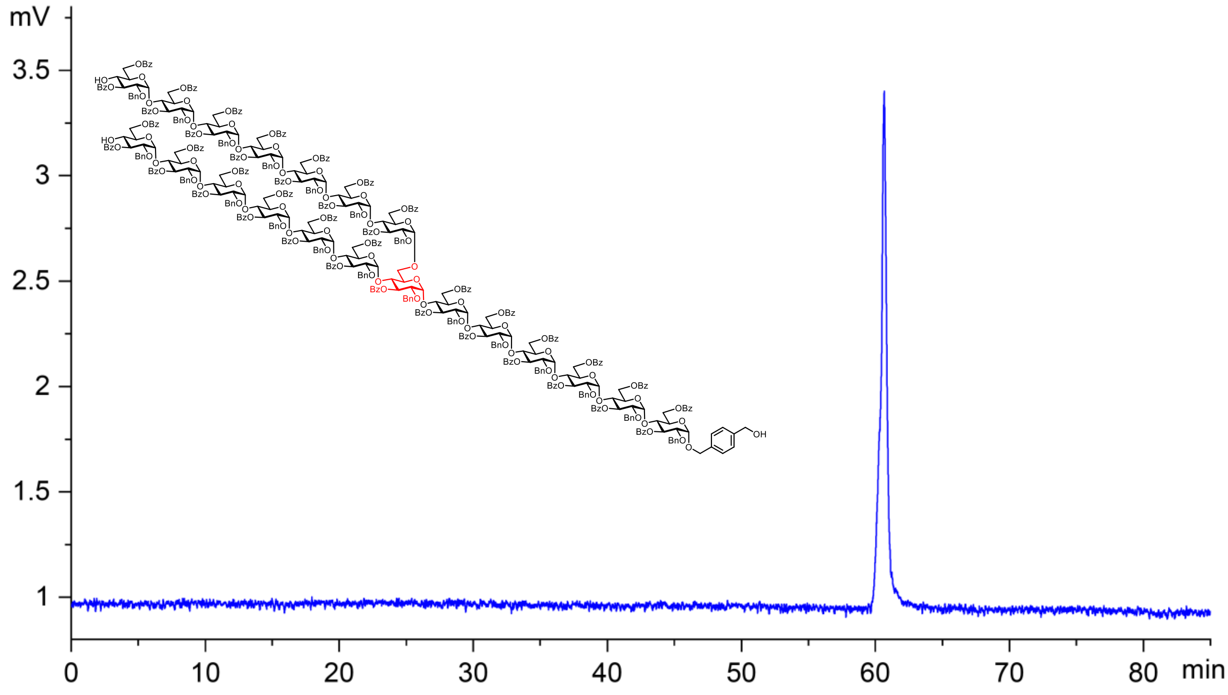

MALDI spectrum of 48

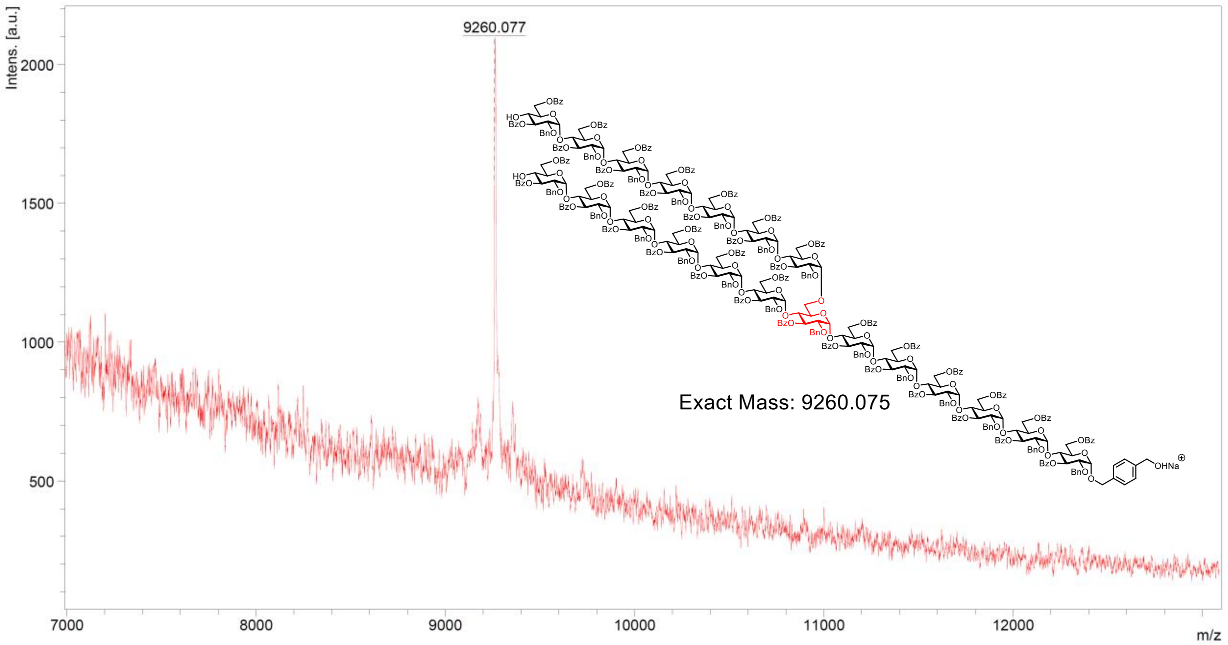

^{1}H NMR of 48 (700 MHz, $CDCl_3$)

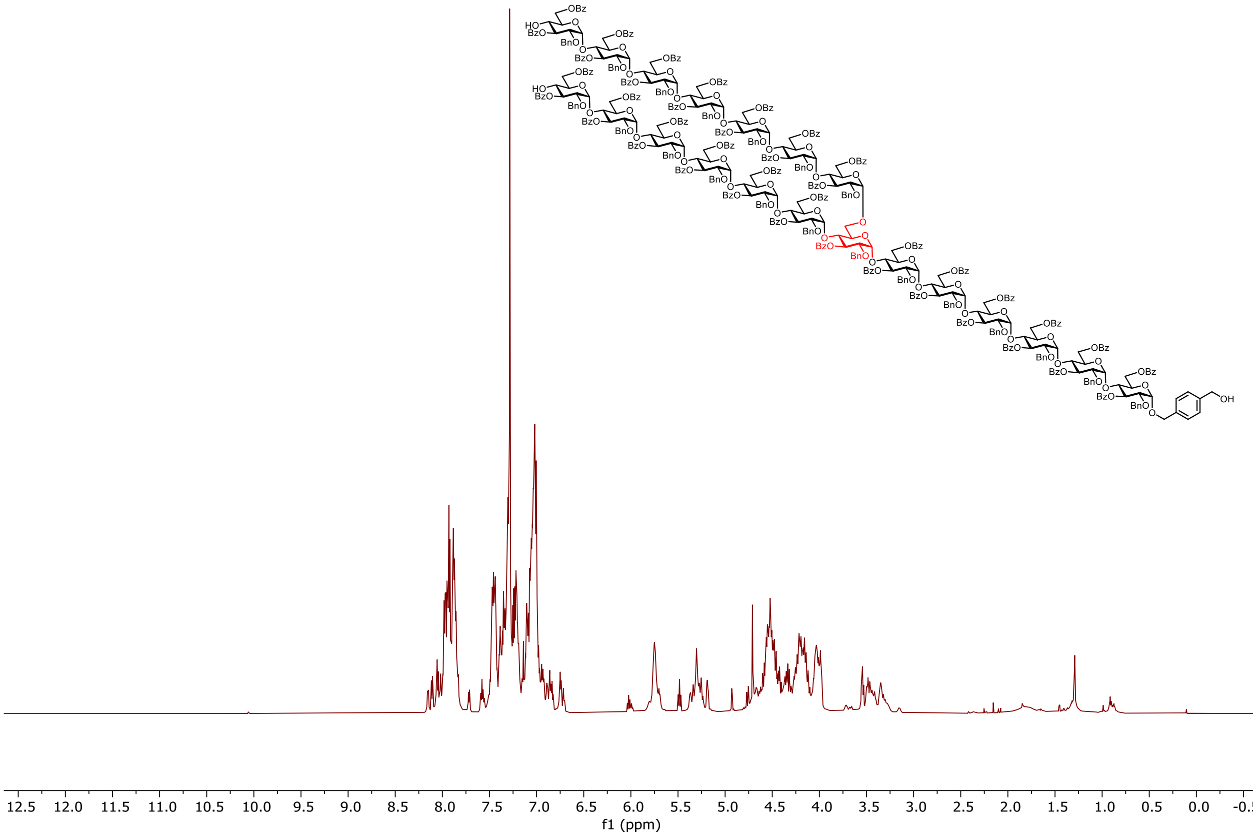

^{13}C NMR of 48 (176 MHz, $CDCl_3$)

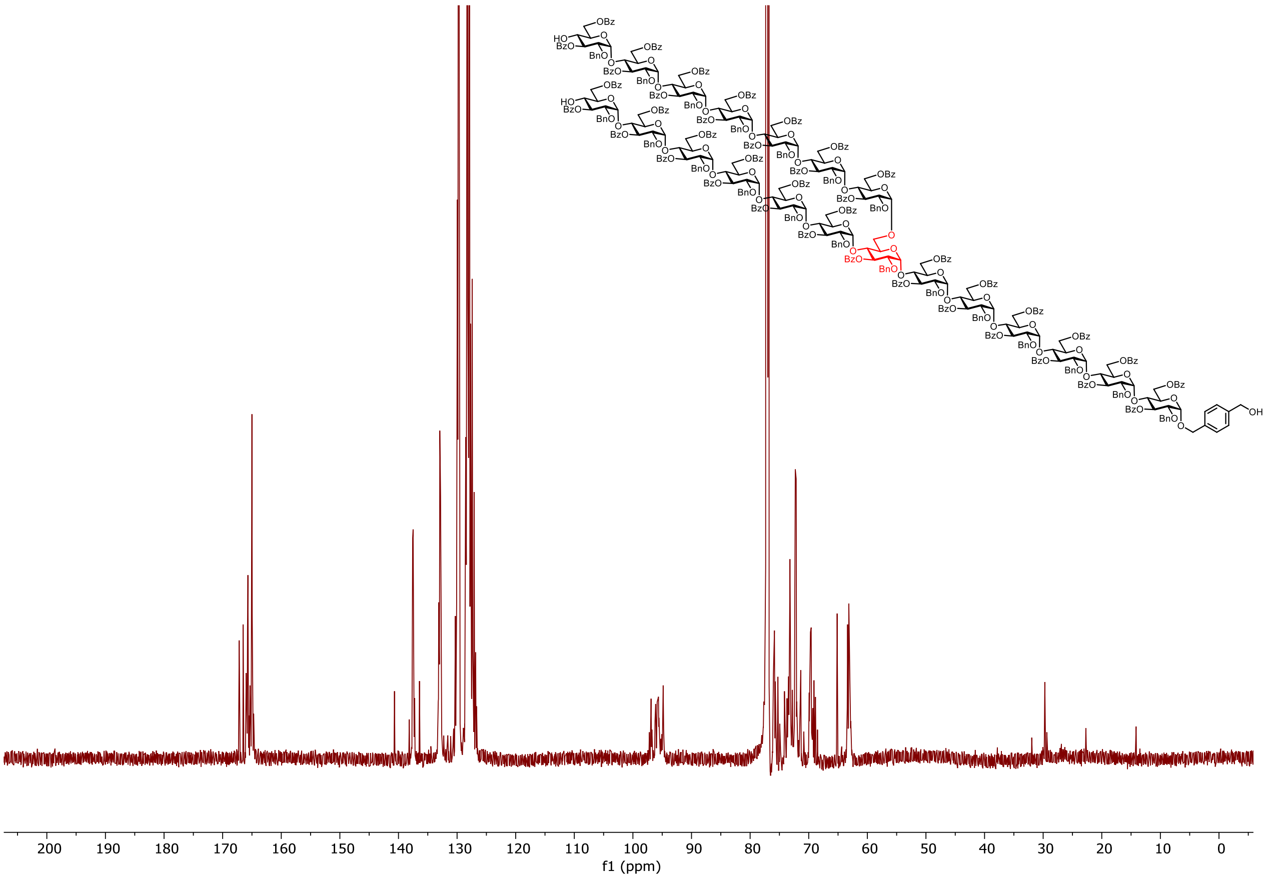

HSQC NMR of 48 ($CDCl_3$)

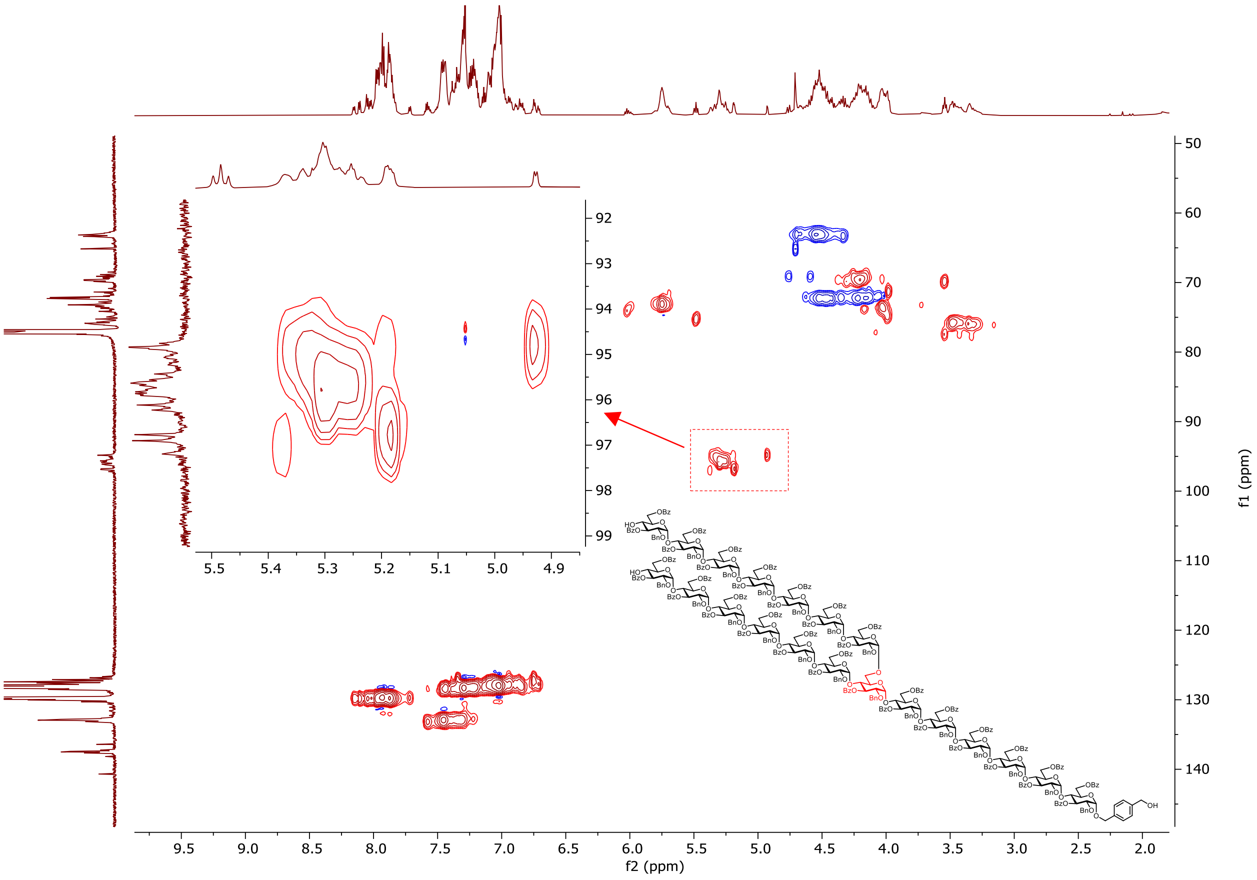

Coupled HSQC NMR of 48 ($CDCl_3$)

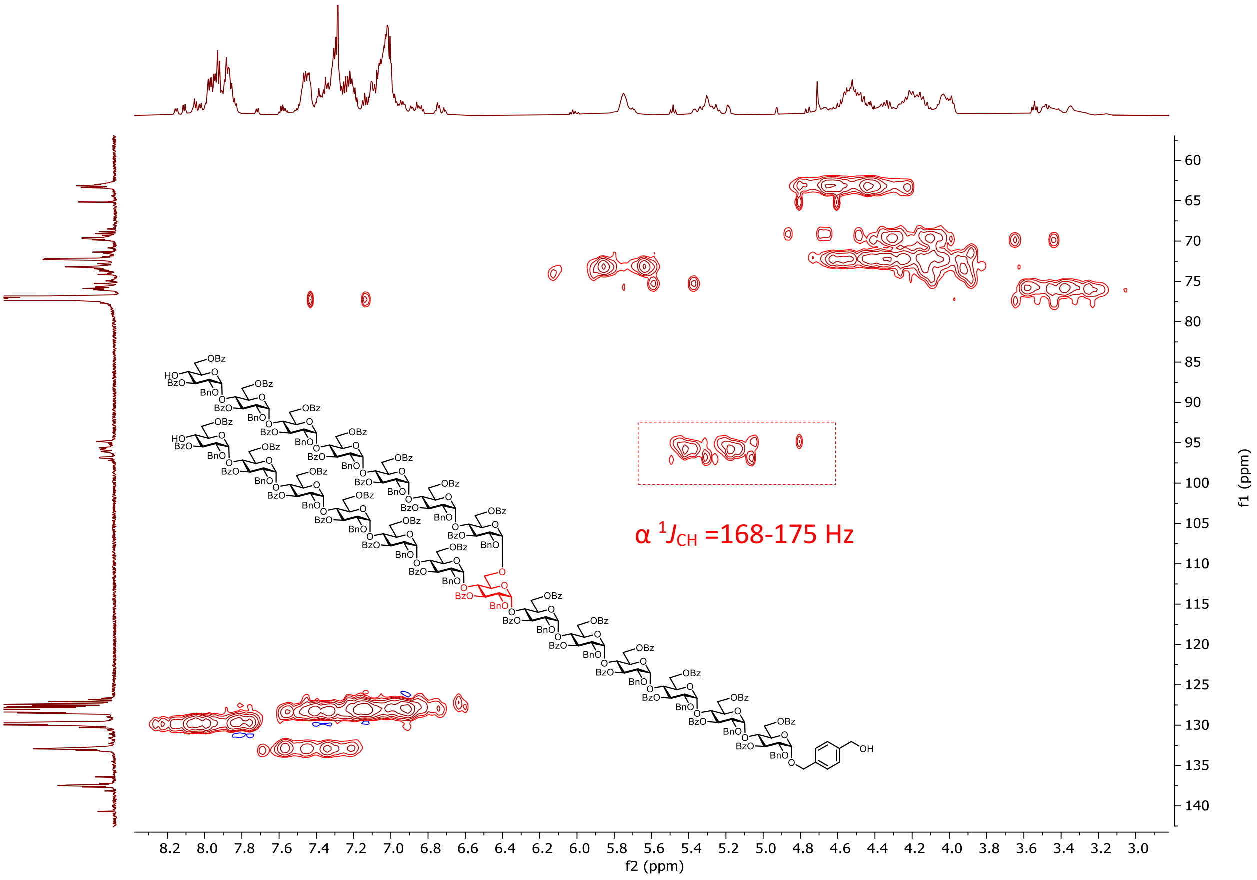

Global deprotection and purification afforded deprotected 20-mer **10** as a white solid (7.3 mg, 14% overall). Analytical data for **10**: ^{1}H NMR (700 MHz, D_2O) δ 5.47 – 5.38 (m, 15H, α(1→4) anomeric H), 5.37 (d, J = 4.0 Hz, 1H, α(1→4) anomeric H), 5.36 (d, J = 3.9 Hz, 1H, α(1→4) anomeric H), 5.35 (d, J = 3.9 Hz, 1H, α(1→4) anomeric H), 5.24 (d, J = 3.8 Hz, 0.33 H, α-H1), 4.98 (d, J = 3.6 Hz, 1H, α(1→6) anomeric H), 4.66 (d, J = 8.0 Hz, 0.67 H, β-H1), 4.09 – 3.53 (m, 117.33 H), 3.43 (appt, J = 9.6 Hz, 2H), 3.28 (dd, J = 9.5, 7.9 Hz, 0.67 H); ^{13}C NMR (176 MHz, D_2O) δ 100.00, 99.95, 99.86, 99.78, 99.71, 99.67, 99.64, 99.63, 99.58, 99.56, 99.53, 99.51, 99.49, 99.47, 99.38, 98.63, 95.78 (β-C1), 91.90 (α-C1), 77.91, 77.72, 76.97, 76.93, 76.90, 76.85, 76.79, 76.75, 76.66, 76.65, 76.62, 76.19, 74.54, 74.01, 73.41, 73.34, 73.31, 73.21, 73.02, 72.88, 72.71, 71.74, 71.67, 71.53, 71.52, 71.47, 71.43, 71.33, 71.30, 71.26, 71.22, 71.18, 70.38, 69.94, 69.32, 60.74, 60.69, 60.56, 60.54, 60.48, 60.44; m/z (HRMS^{+}) $[M + Na]^{+}$ 3282.014 ($C_{120}H_{202}O_{101}Na^{+}$ requires 3282.056).

RP-HPLC of 10 (ELSD trace, Method C, t_R = 15.2 min)

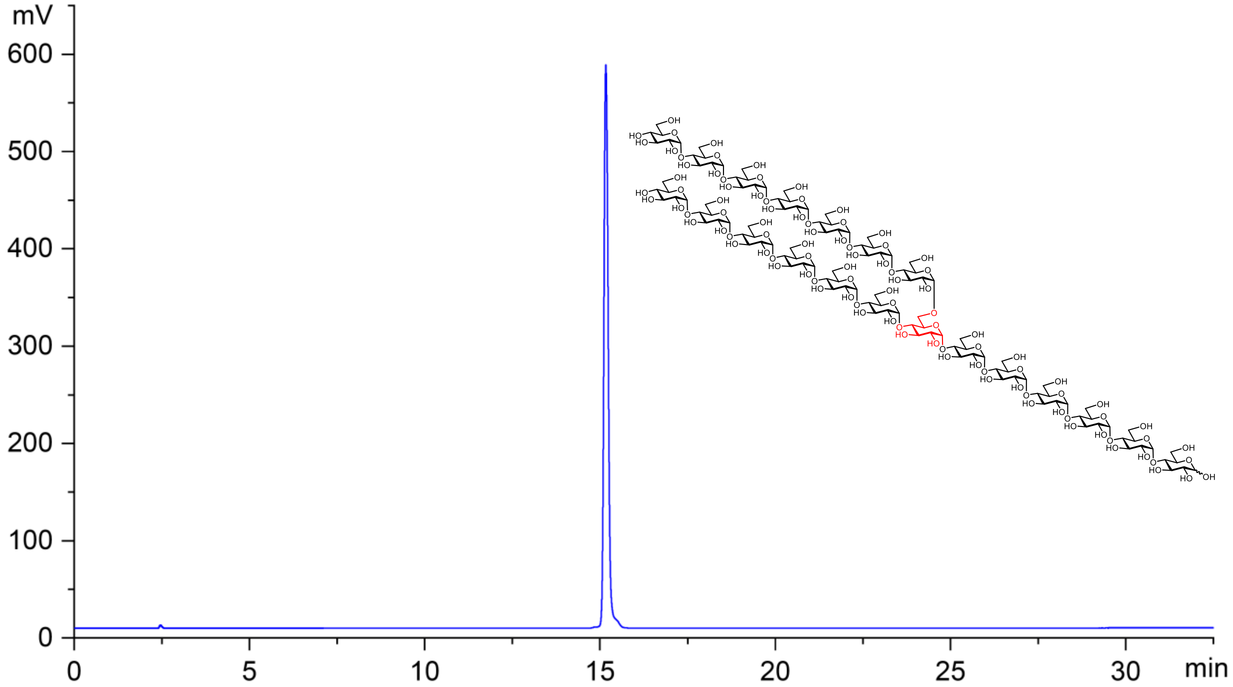

MALDI spectrum of 10

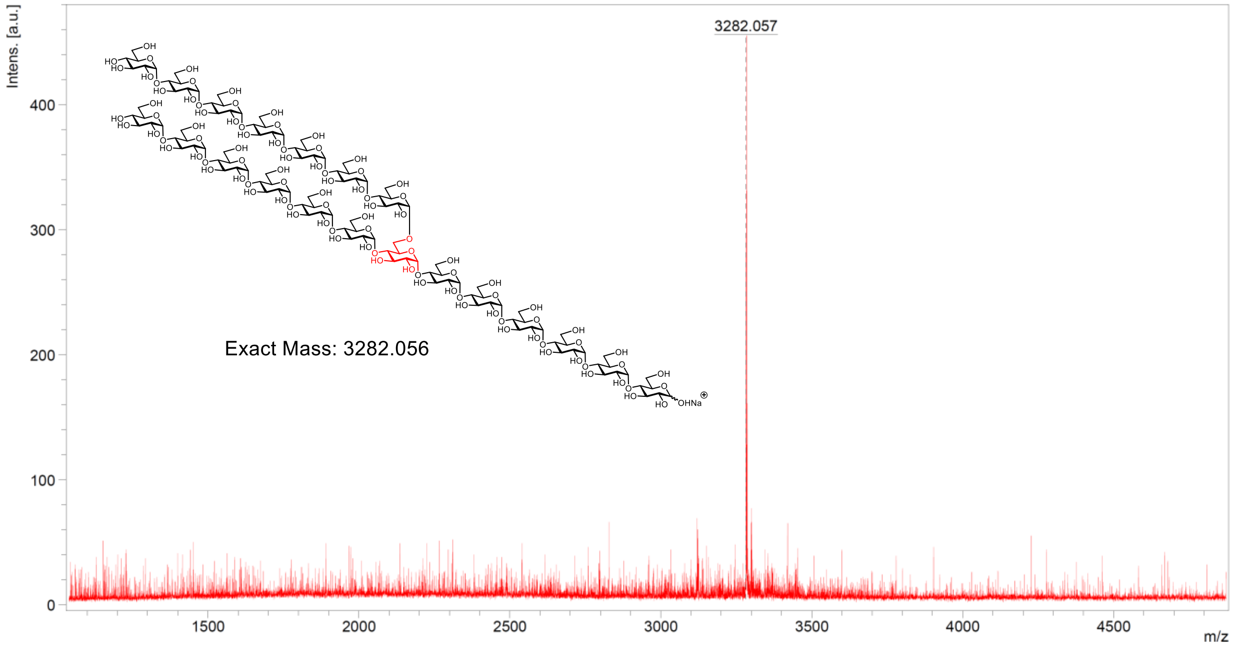

^{1}H NMR of 10 (700 MHz, D_2O)

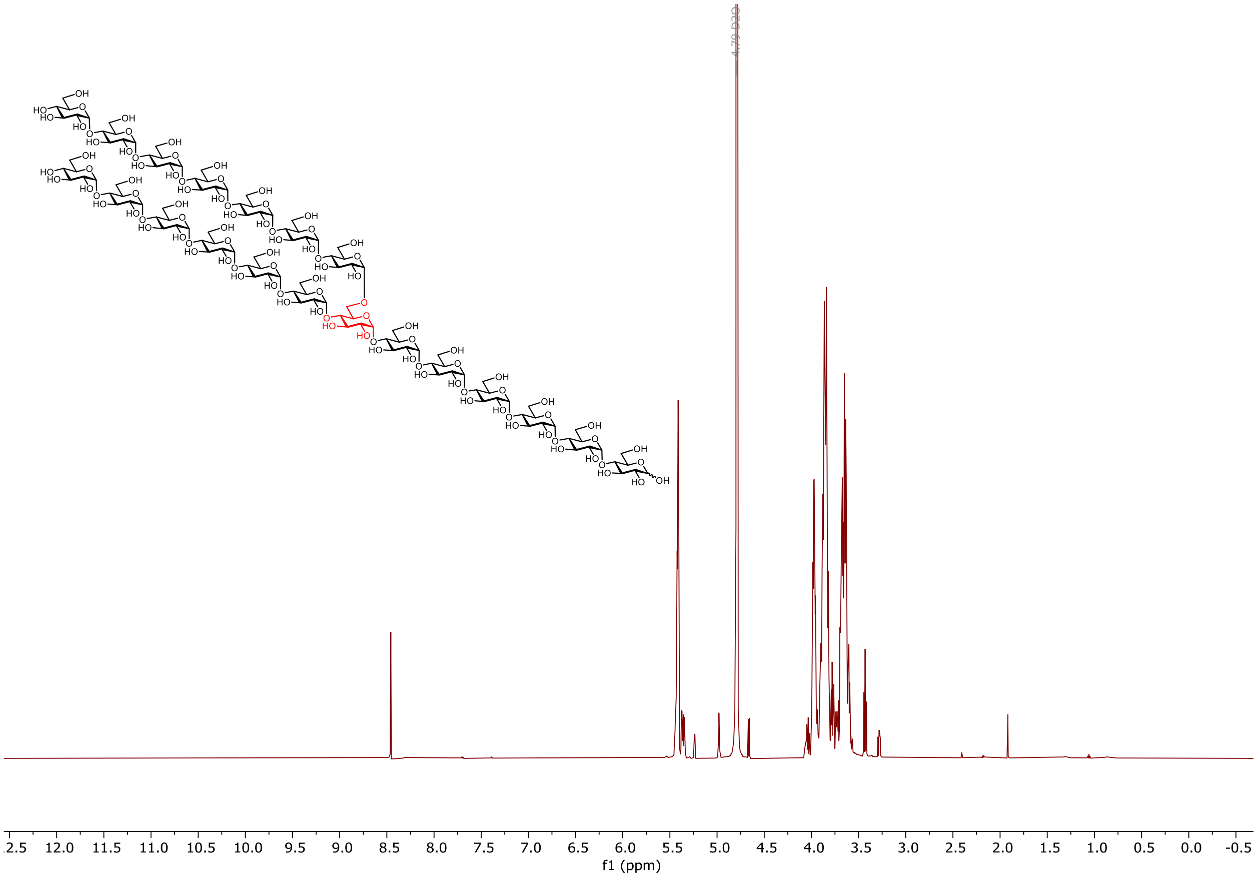

^{13}C NMR of 10 (176 MHz, D_2O)

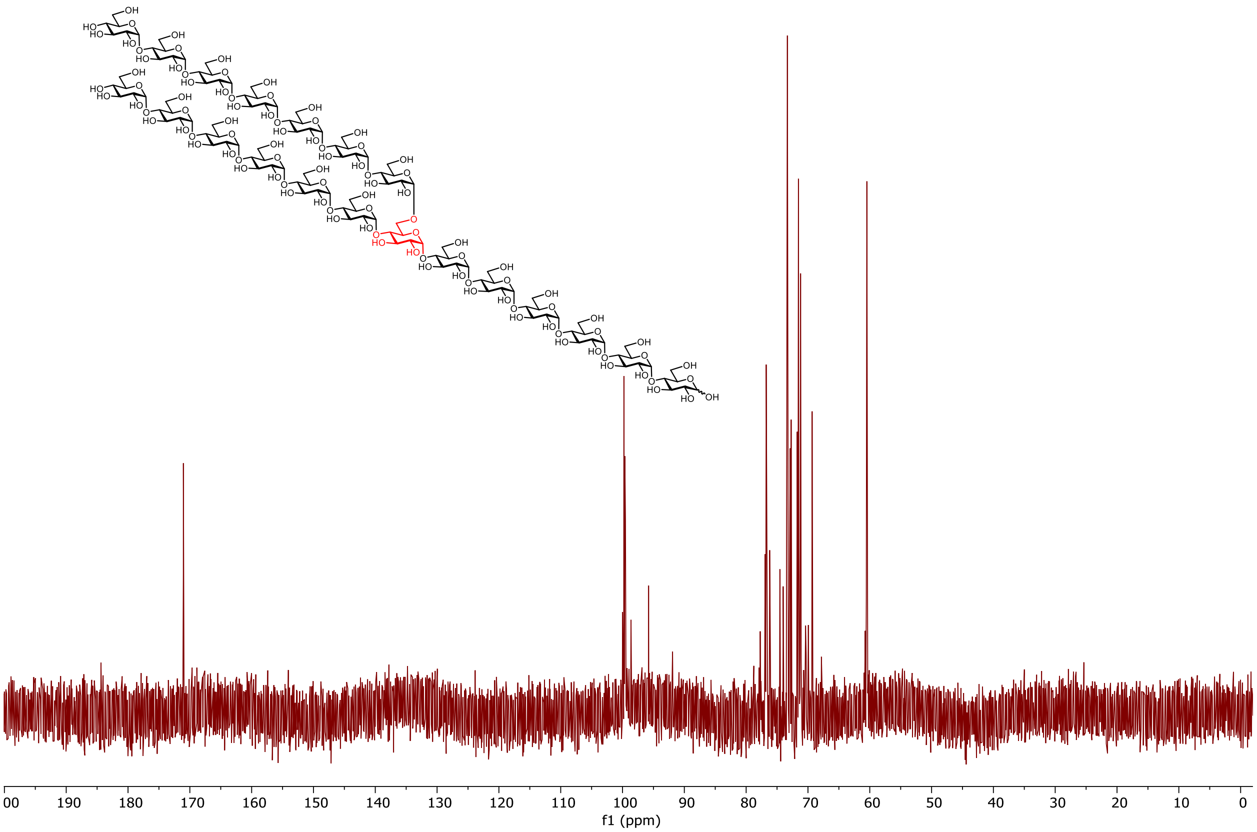

HSQC NMR of 10 (D_2O)

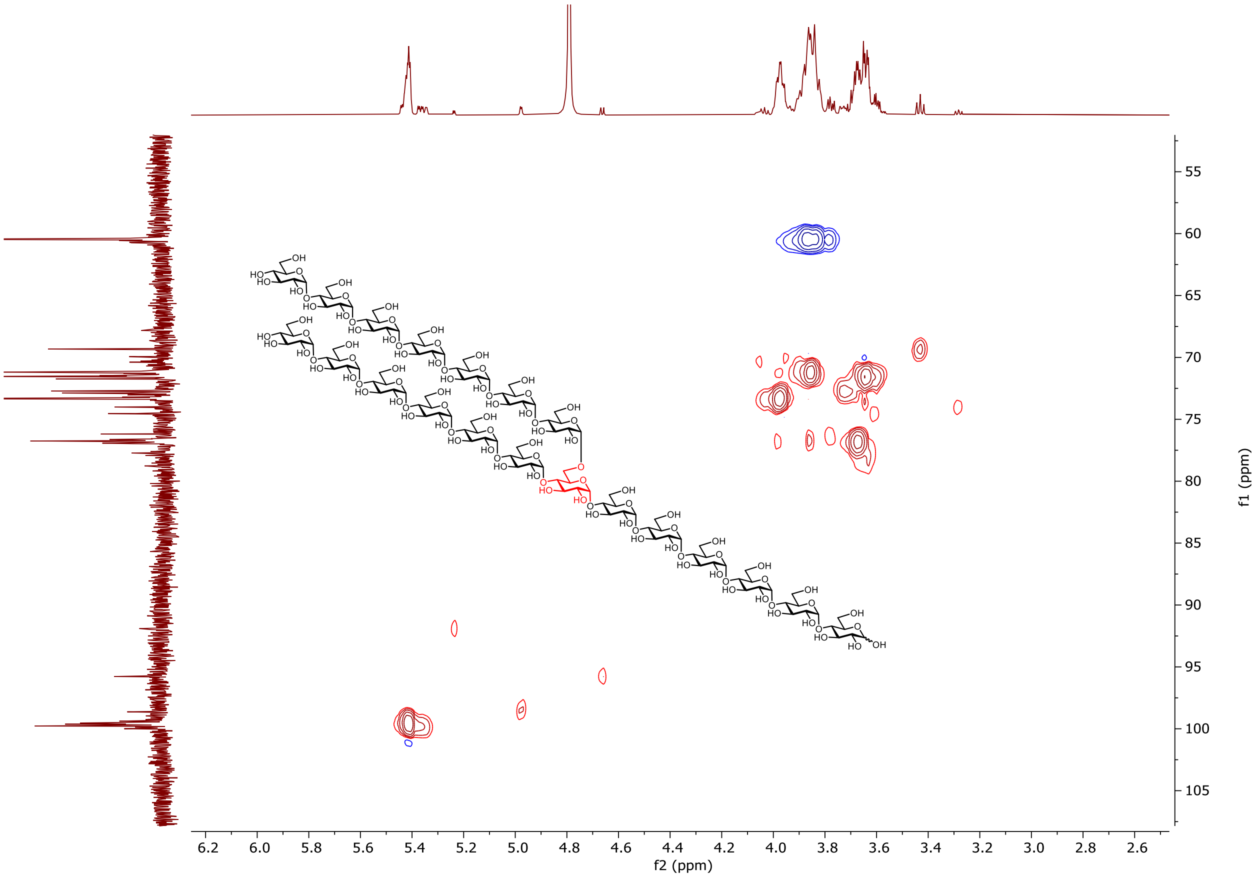

Coupled HSQC NMR of 10 (D_2O)

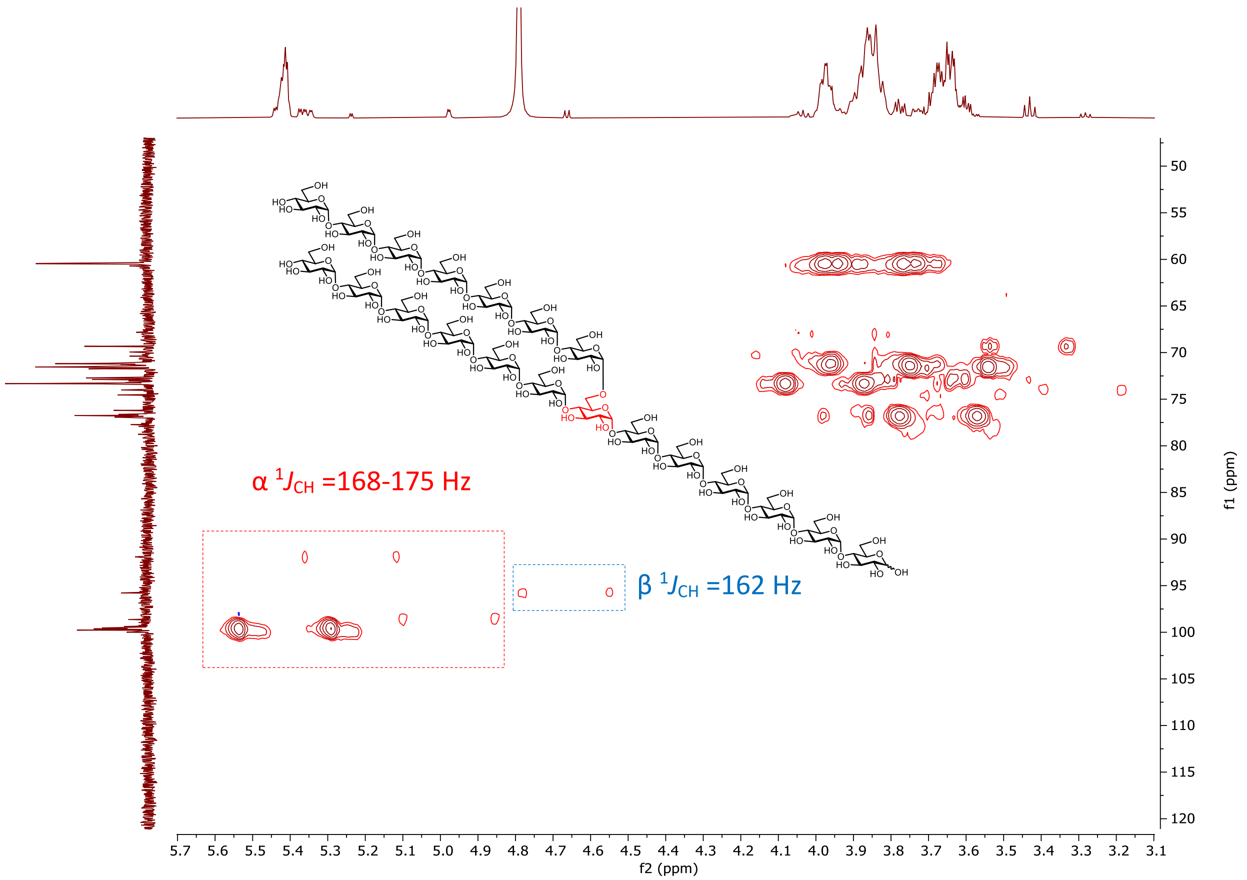

7.8 Synthesis of glycogen 14-mer 11

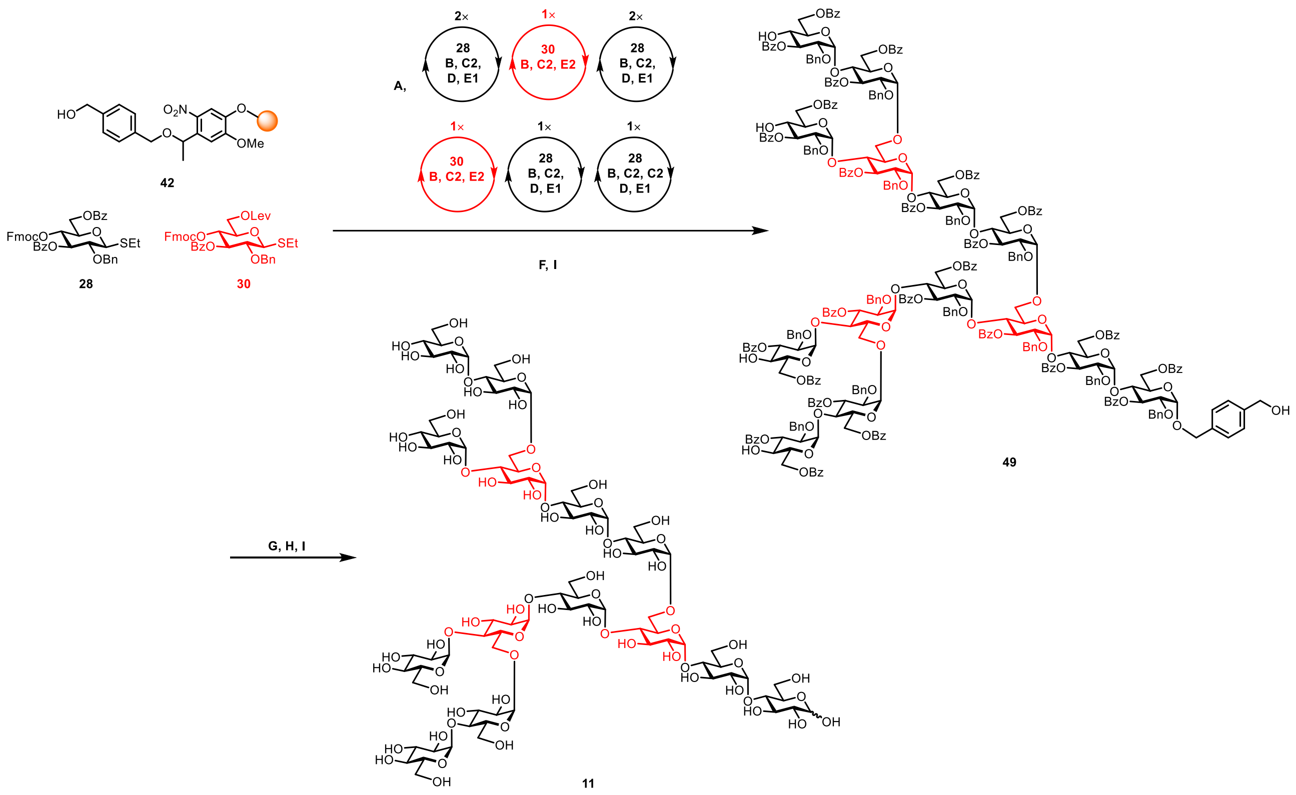

	Module	Conditions
	**A**: Resin Preparation for Synthesis	
2	**B**: Acidic Wash with TMSOTf Solution	
	**C2**: Thioglycoside Glycosylation × 2 Cycle	Building block **28**, 6.5 equiv. (-20°C for 5 min, 0°C for 60 min)
	**D**: Capping	
	**E1**: Fmoc Deprotection	
	**B**: Acidic Wash with TMSOTf Solution	
	**C2**: Thioglycoside Glycosylation × 2 Cycle	Building block **30**, 6.5 equiv. (-20°C for 5 min, 0°C for 60 min)
	**E2**: Lev Deprotection	
2	**B**: Acidic Wash with TMSOTf Solution	
	**C2**: Thioglycoside Glycosylation × 2 Cycle	Building block **28**, 6.5 equiv. (-20°C for 5 min, 0°C for 60 min)
	**D**: Capping	
	**E1**: Fmoc Deprotection	

**B**: Acidic Wash with TMSOTf Solution	
**C2**: Thioglycoside Glycosylation × 2 Cycle	Building block **30**, 6.5 equiv. (-20°C for 5 min, 0°C for 60 min)
**E2**: Lev Deprotection	
**B**: Acidic Wash with TMSOTf Solution	
**C2**: Thioglycoside Glycosylation × 2 Cycle	Building block **28**, 6.5 equiv. (-20°C for 5 min, 0°C for 60 min)
**D**: Capping	
**E1**: Fmoc Deprotection	
**B**: Acidic Wash with TMSOTf Solution	
**C2**: Thioglycoside Glycosylation × 2 Cycle	Building block **28**, 6.5 equiv. (-20°C for 5 min, 0°C for 60 min)
**C2**: Thioglycoside Glycosylation × 2 Cycle	Building block **28**, 6.5 equiv. (-20°C for 5 min, 0°C for 60 min)
**D**: Capping	
**E1**: Fmoc Deprotection	
**F:** Cleavage from Solid Support	
**I**: Purification	**Method E and B3**
**G**: Solution-phase Methanolysis	
**H**: Hydrogenolysis at Ambient Pressure	
**I**: Purification	**Method D**

Automated synthesis and purification afforded protected 14-mer **49** as a white solid (10 mg, 10%).

Analytical data for **49**: ^{1}H NMR (700 MHz, $CDCl_3$) δ 8.17 – 7.78 (m, 50H), 7.62 – 7.17 (m, 79H), 7.17 – 6.68 (m, 70H), 6.06 – 5.98 (m, 2H), 5.91 – 5.85 (m, 2H), 5.84 – 5.70 (m, 6H), 5.57 – 5.45 (m, 4H), 5.45 – 5.40 (m, 3H), 5.39 (d, *J* = 3.6 Hz, 1H), 5.36 (d, *J* = 3.3 Hz, 1H), 5.31 (d, *J* = 3.8 Hz, 1H), 5.29 (d, *J* = 3.6 Hz, 1H), 5.17 (d, *J* = 3.5 Hz, 1H), 5.16 – 5.07 (m, 4H), 5.06 (d, *J* = 3.3 Hz, 1H), 5.04 (d, *J* = 3.3 Hz, 1H), 4.95 (d, *J* = 3.3 Hz, 1H), 4.84 – 3.94 (m, 67H), 3.93 – 3.71 (m, 8H), 3.71 – 3.39 (m, 21H), 3.33 (dd, *J* = 9.8, 3.3 Hz, 2H), 3.26 – 3.20 (m, 1H), 3.15 – 3.08 (m, 1H), 3.08 – 2.99 (m, 1H); ^{13}C NMR (176 MHz, $CDCl_3$) δ 167.33, 167.23, 166.88, 166.78, 166.55, 166.50, 166.07, 166.05, 165.96, 165.68, 165.56, 165.41, 165.33, 165.25, 164.84, 164.71, 164.31, 140.74, 138.14, 138.02, 137.86, 137.76, 137.70, 137.64, 137.61, 137.48, 137.47, 137.43, 137.39, 137.25, 136.37, 133.28, 133.11, 133.00, 132.89, 132.42, 132.24, 131.65, 131.11, 130.87, 130.72, 130.51, 130.32, 130.20, 130.11 (d, *J* = 8.5 Hz), 130.05, 130.02, 129.84, 129.77, 129.72, 128.55, 128.49, 128.33, 128.29, 128.23, 128.14, 128.04, 128.01, 127.97, 127.95, 127.90, 127.75, 127.69, 127.54, 127.49, 127.46, 127.37, 127.24, 127.15, 127.02, 126.99, 126.72, 97.91, 97.64, 97.30, 97.02, 96.84, 96.58, 96.23, 96.14, 95.62, 95.42, 95.25, 95.12, 94.97, 76.40, 76.35, 76.16, 76.08, 75.90, 75.64, 74.74, 74.62, 74.33, 74.14, 74.02, 73.38, 73.27, 73.13, 72.36, 72.30, 72.20, 72.16, 72.03, 72.01, 71.94, 71.76, 71.58, 71.52, 71.04, 70.96, 70.82, 70.62, 70.31, 70.19, 69.60, 69.48, 69.37, 69.20, 69.09, 68.84, 68.69, 68.55, 68.42, 65.08, 64.94, 64.91, 64.50, 63.97, 63.73, 63.38, 63.20, 63.09, 62.92; m/z (HRMS$^+$) $[M + 3Na]^{3+}$ 2113.424 ($C_{365}H_{334}O_{97}Na_3^{3+}$ requires 2113.366).

NP-HPLC of crude 49 after AGA (ELSD trace, Method A3)

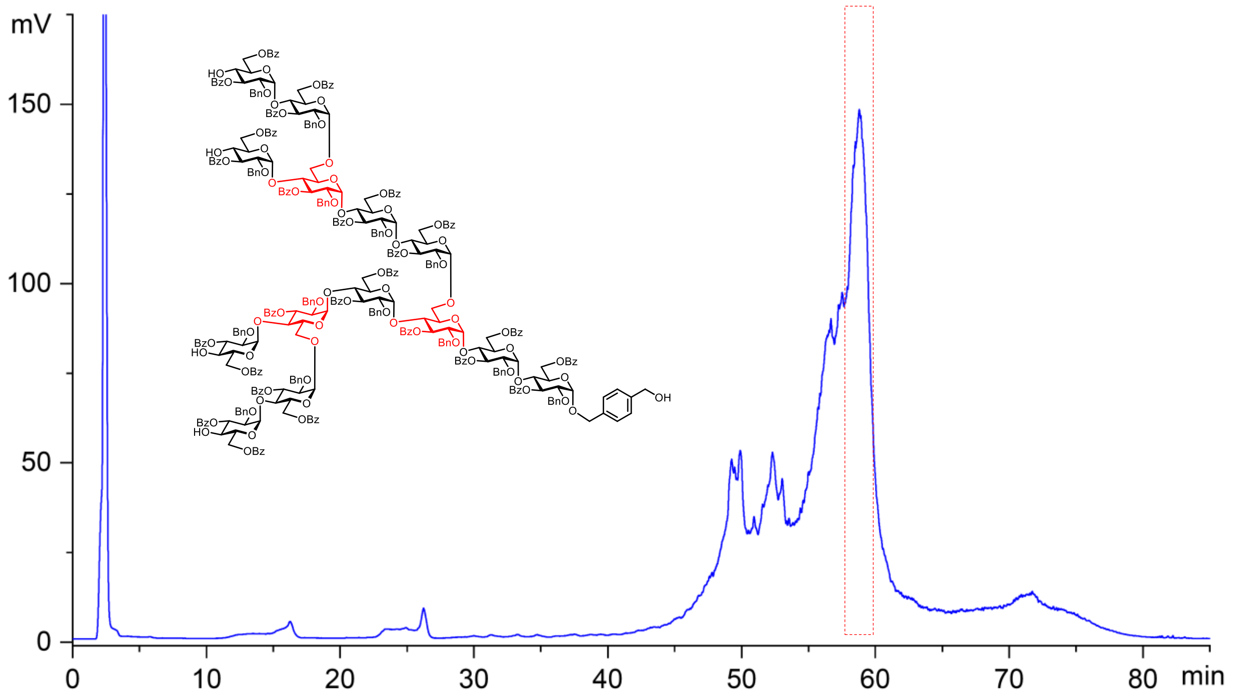

NP-HPLC of pure 49 (ELSD trace, Method A3, t_R = 60.2 min)

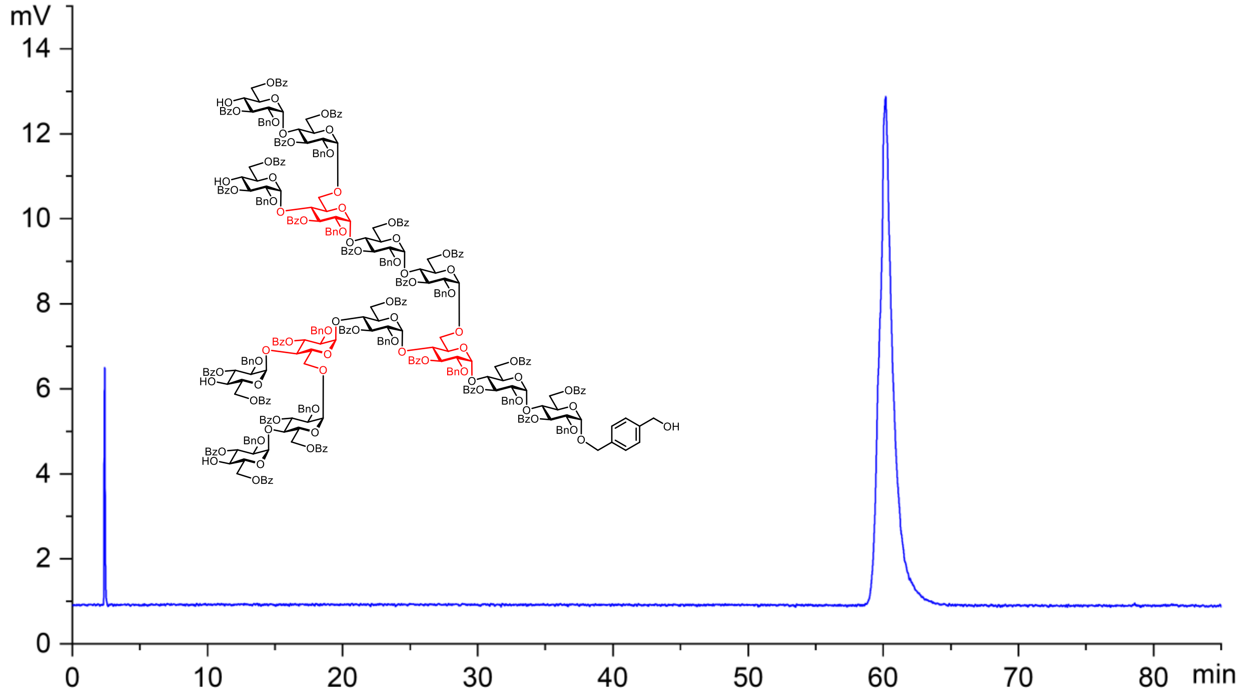

MALDI spectrum of 49

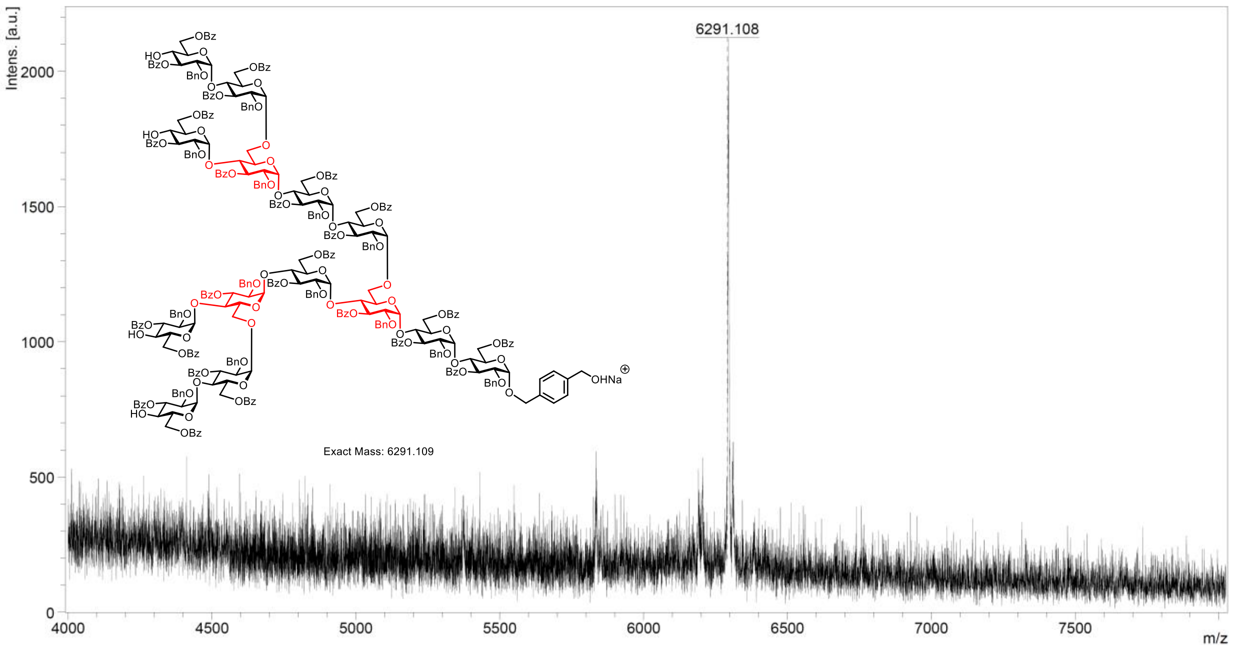

^{1}H NMR of 49 (700 MHz, $CDCl_3$)

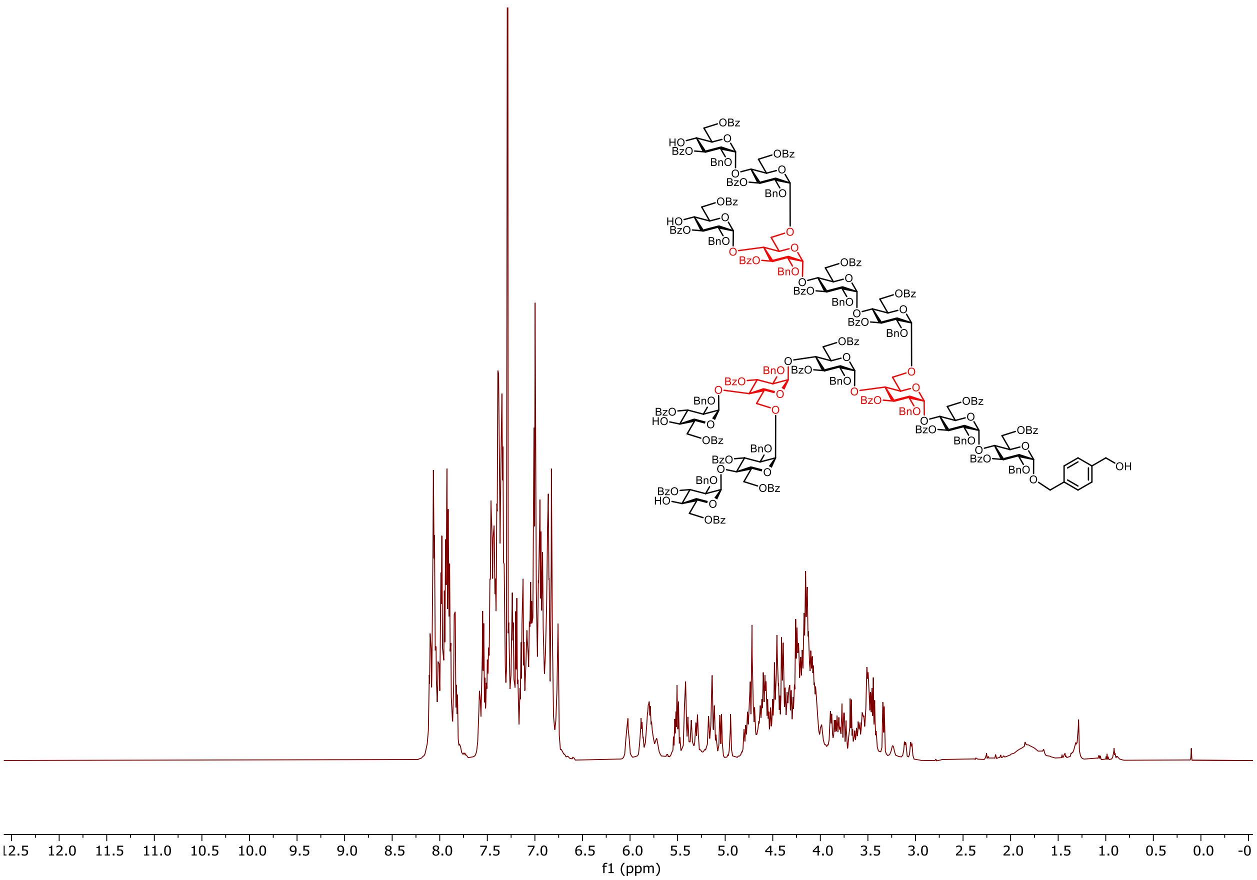

^{13}C NMR of 49 (176 MHz, $CDCl_3$)

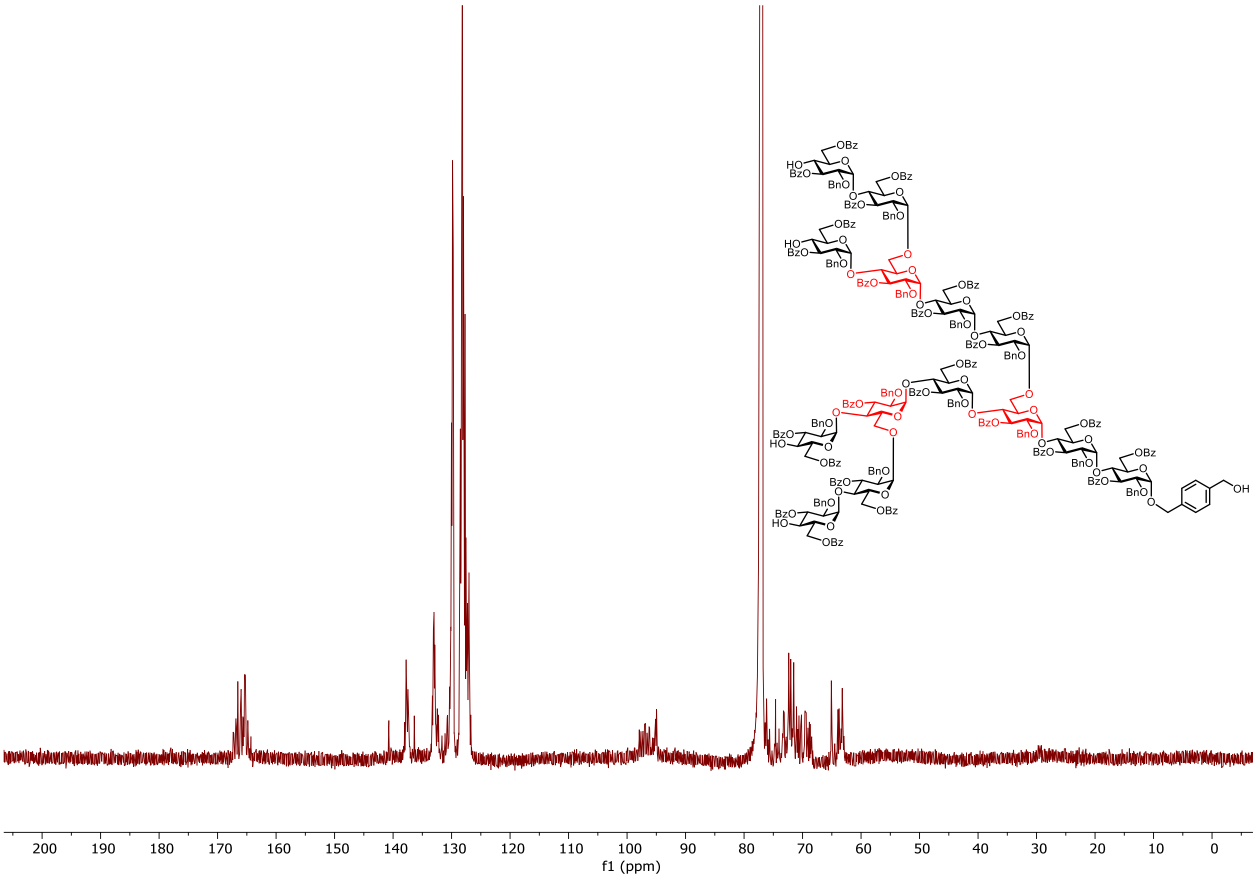

HSQC NMR of 49 ($CDCl_3$)

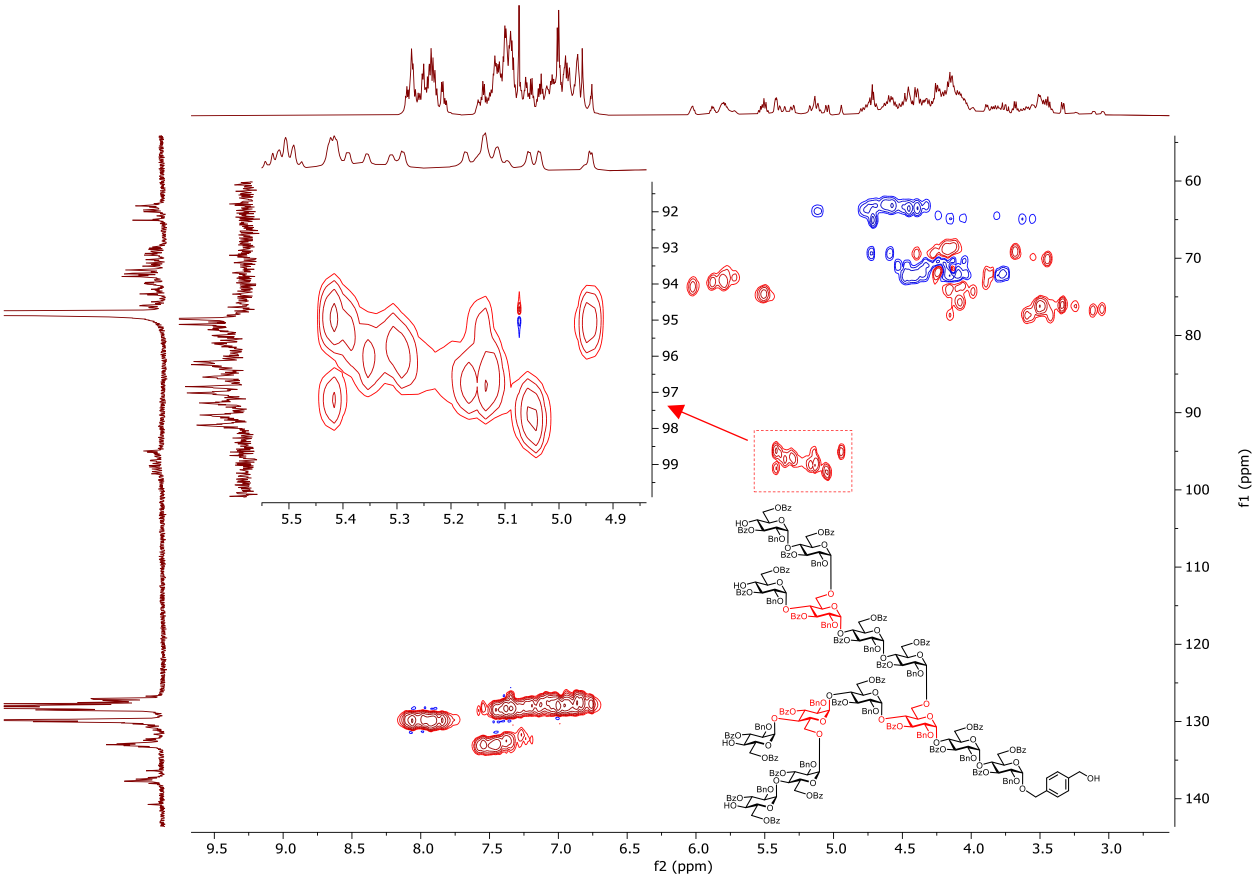

Coupled HSQC NMR of 49 ($CDCl_3$)

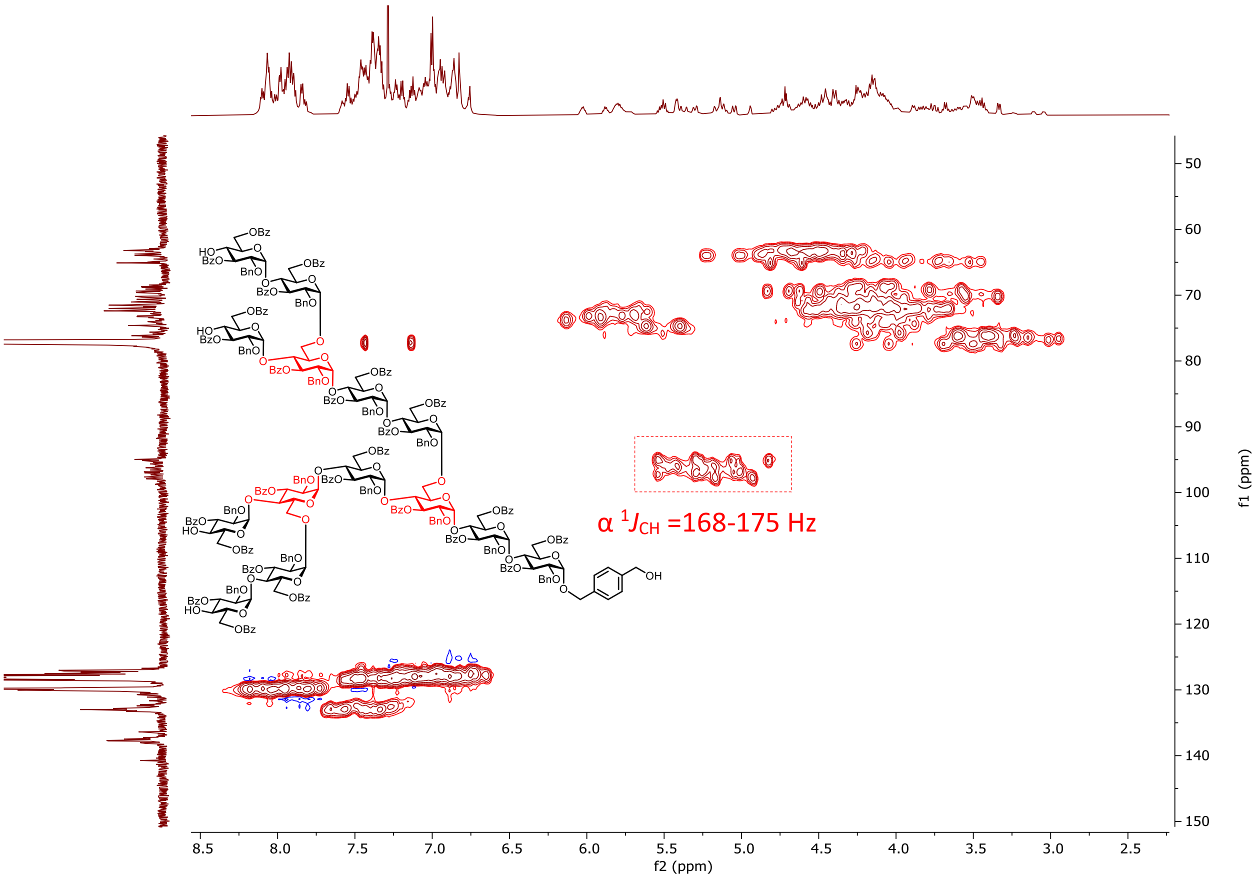

Global deprotection and purification afforded deprotected 14-mer **11** as a white solid (2.5 mg, 7% overall). Analytical data for **11**: ^{1}H NMR (700 MHz, D_2O) δ 5.43 (d, J = 3.9 Hz, 1H, α(1→4) anomeric H), 5.40 – 5.36 (m, 5H, α(1→4) anomeric H), 5.35 – 5.32 (m, 4H, α(1→4) anomeric H), 5.24 (d, J = 3.8 Hz, 0.42H, α-H1), 4.99 – 4.96 (m, 3H, α(1→6) anomeric H), 4.67 (d, J = 8.0 Hz, 0.58H, β-H1), 4.12 – 3.55 (m, 79.42H), 3.45 – 3.39 (m, 4H), 3.29 (appt, J = 8.7 Hz, 0.58H); ^{13}C NMR (176 MHz, D_2O) δ 100.04, 100.00, 99.79, 99.74, 99.40, 98.55, 98.60, 95.76 (β-C1), 91.90 (α-C1), 79.09, 78.28, 78.12, 78.03, 77.76, 77.62, 77.58, 77.52, 77.10, 76.88, 76.18, 74.57, 73.99, 73.33, 73.22, 73.11, 73.07, 72.97, 72.85, 72.81, 72.74, 71.85, 71.62, 71.50, 71.44, 71.36, 71.18, 71.11, 70.40, 70.33, 70.30, 69.37, 69.34, 68.05, 67.31, 67.27, 60.75, 60.63, 60.50, 60.42; m/z (HRMS$^+$) $[M + Na]^+$ 2309.792 ($C_{84}H_{142}O_{71}Na^+$ requires 2309.739).

RP-HPLC of 11 (ELSD trace, Method C, t_R = 15.0 min)

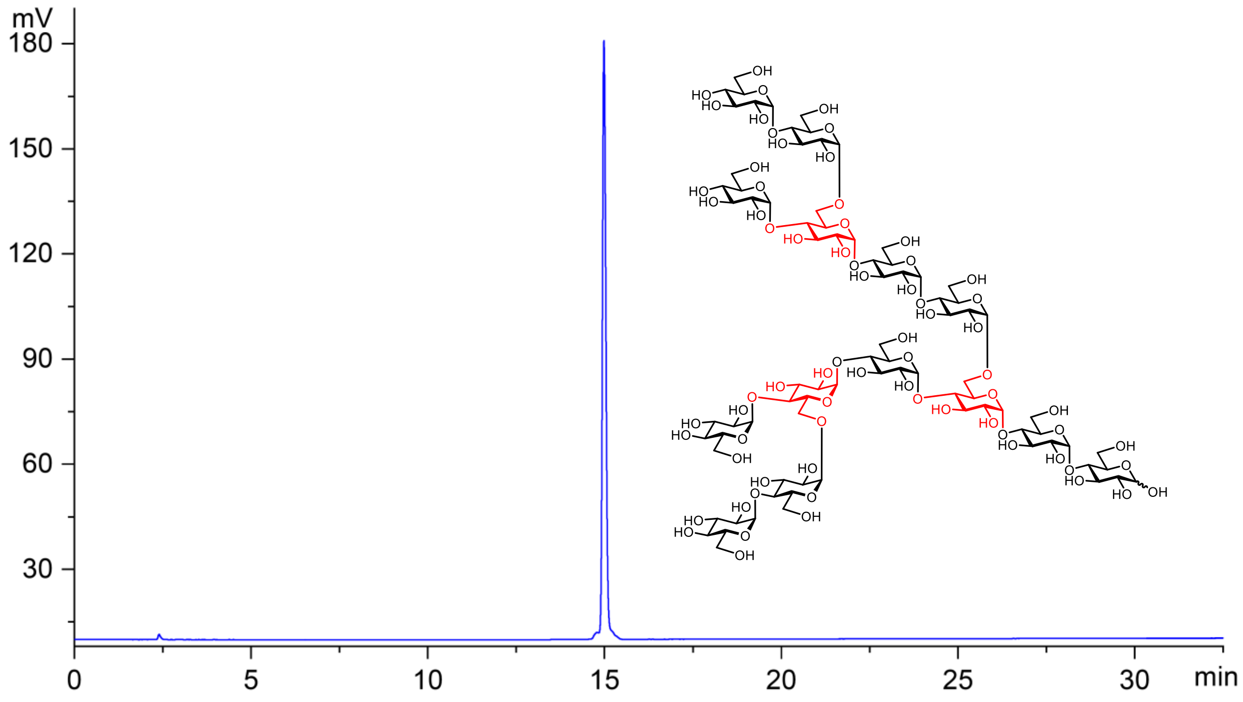

MALDI spectrum of 11

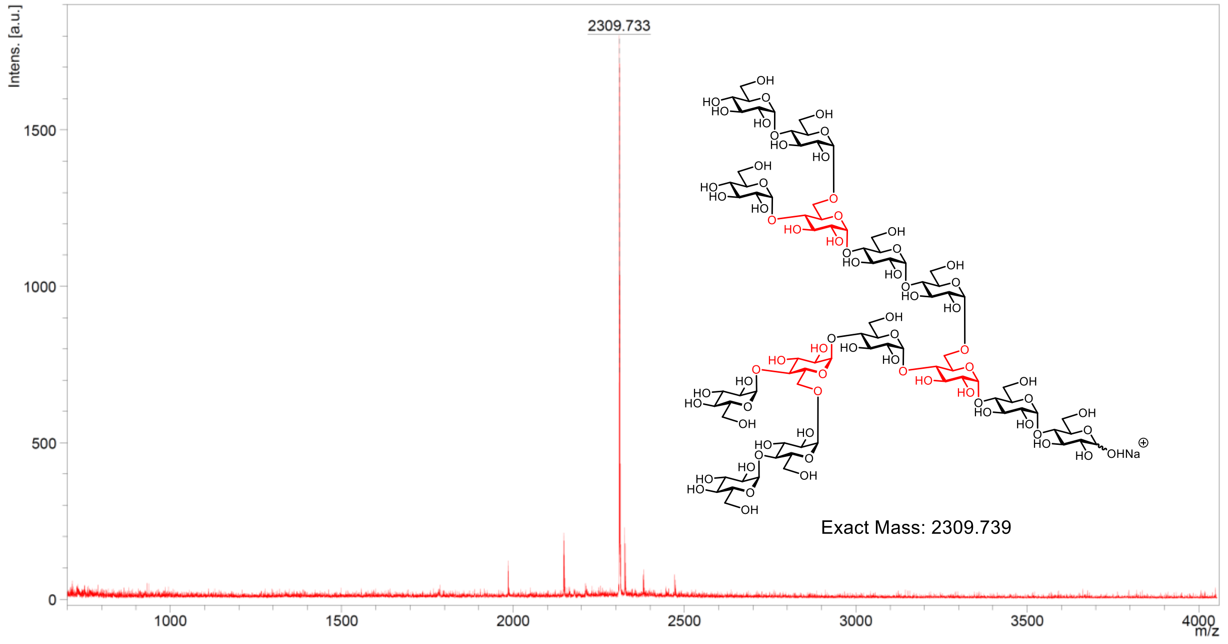

^{1}H NMR of 11 (700 MHz, D_2O)

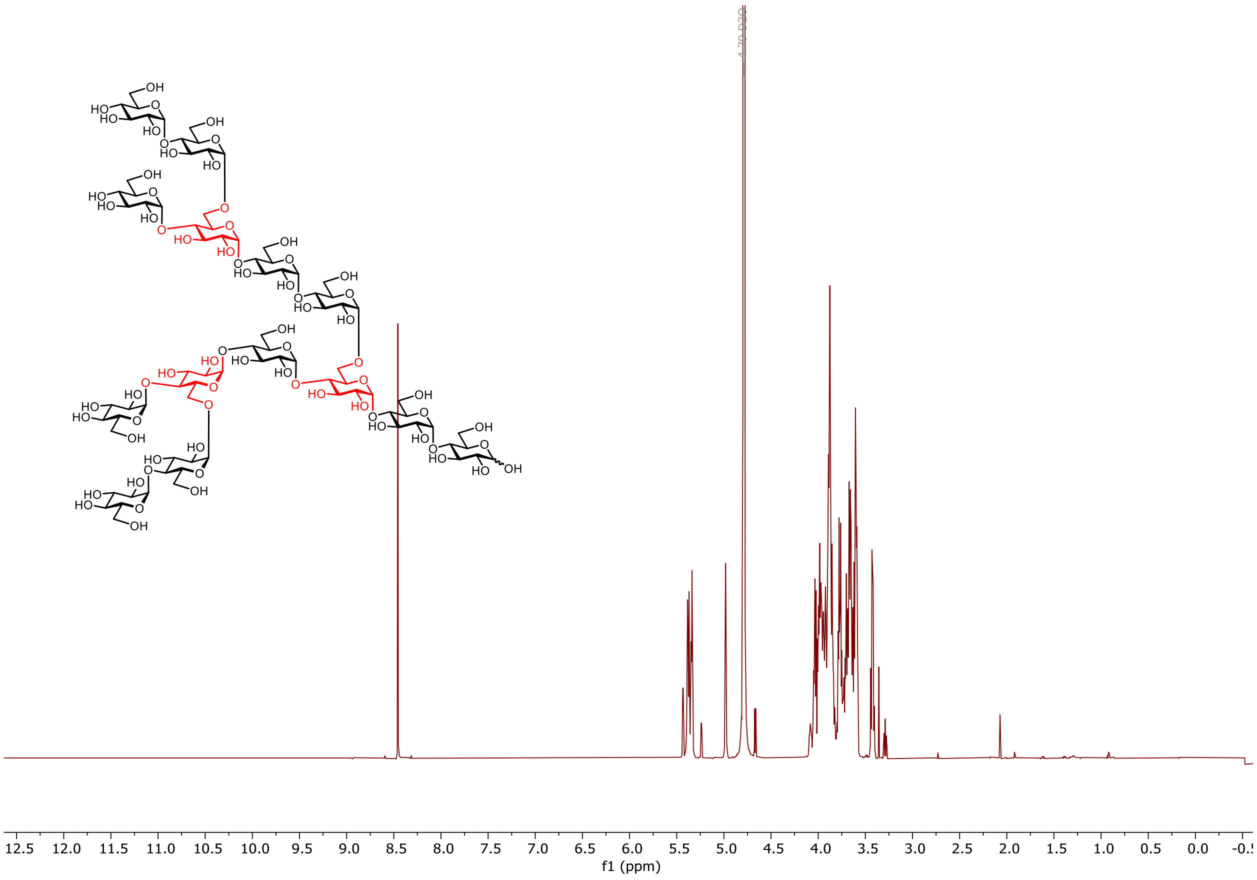

^{13}C NMR of 11 (176 MHz, D_2O)

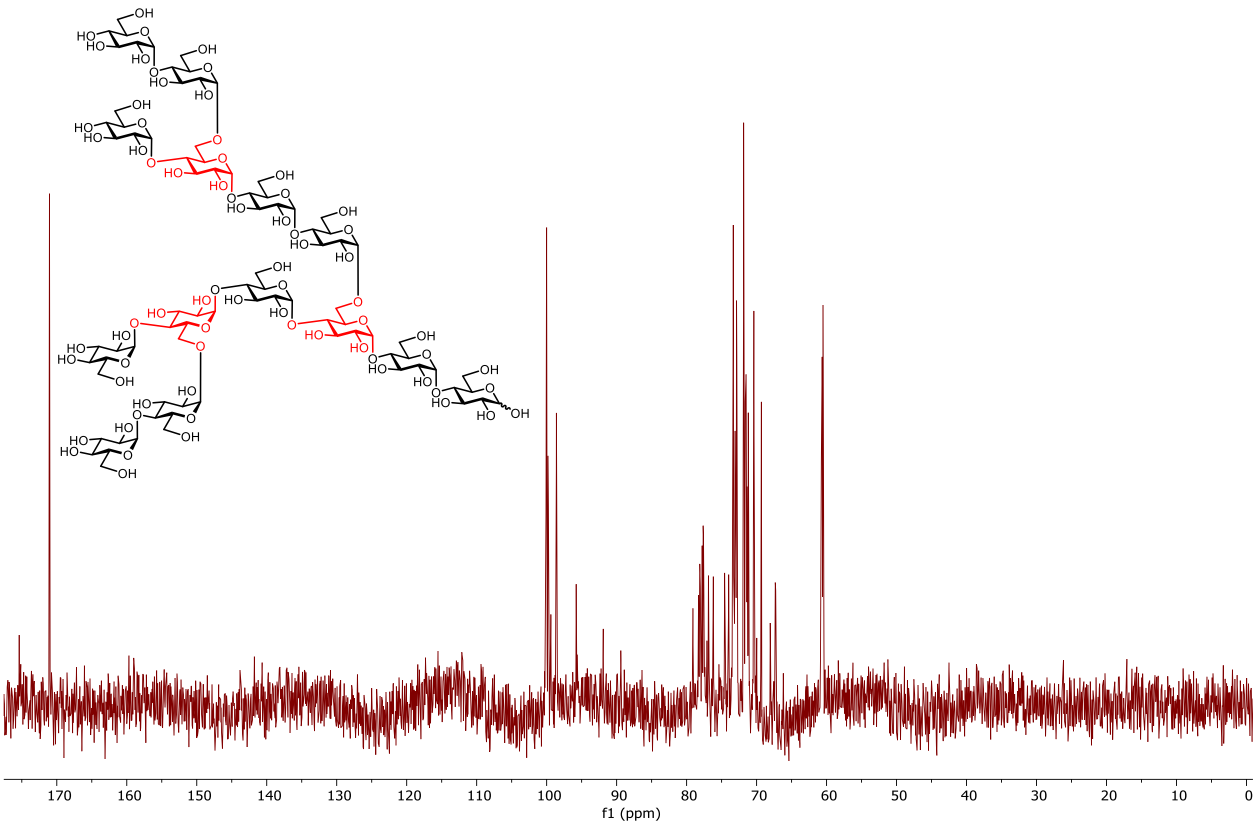

HSQC NMR of 11 (D_2O)

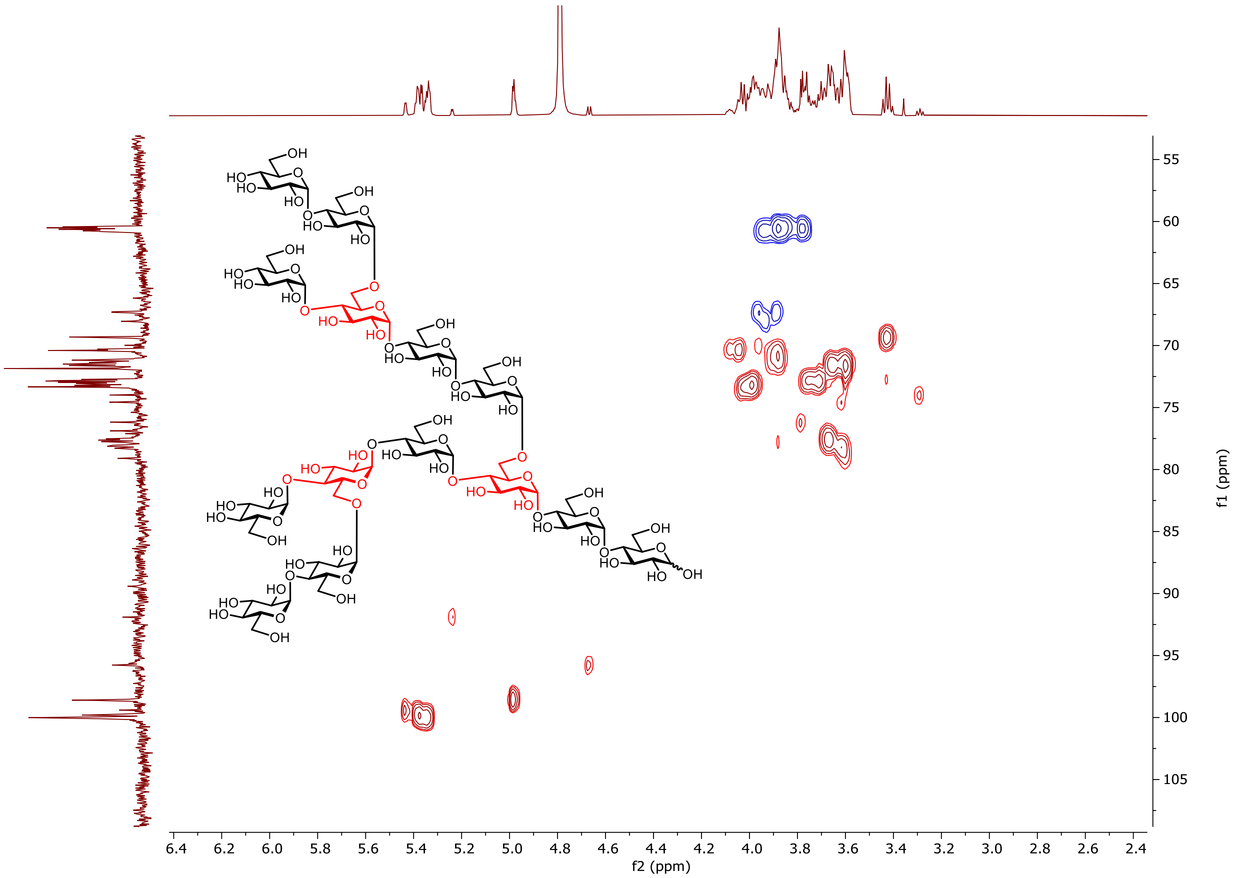

Coupled HSQC NMR of 11 (D_2O)

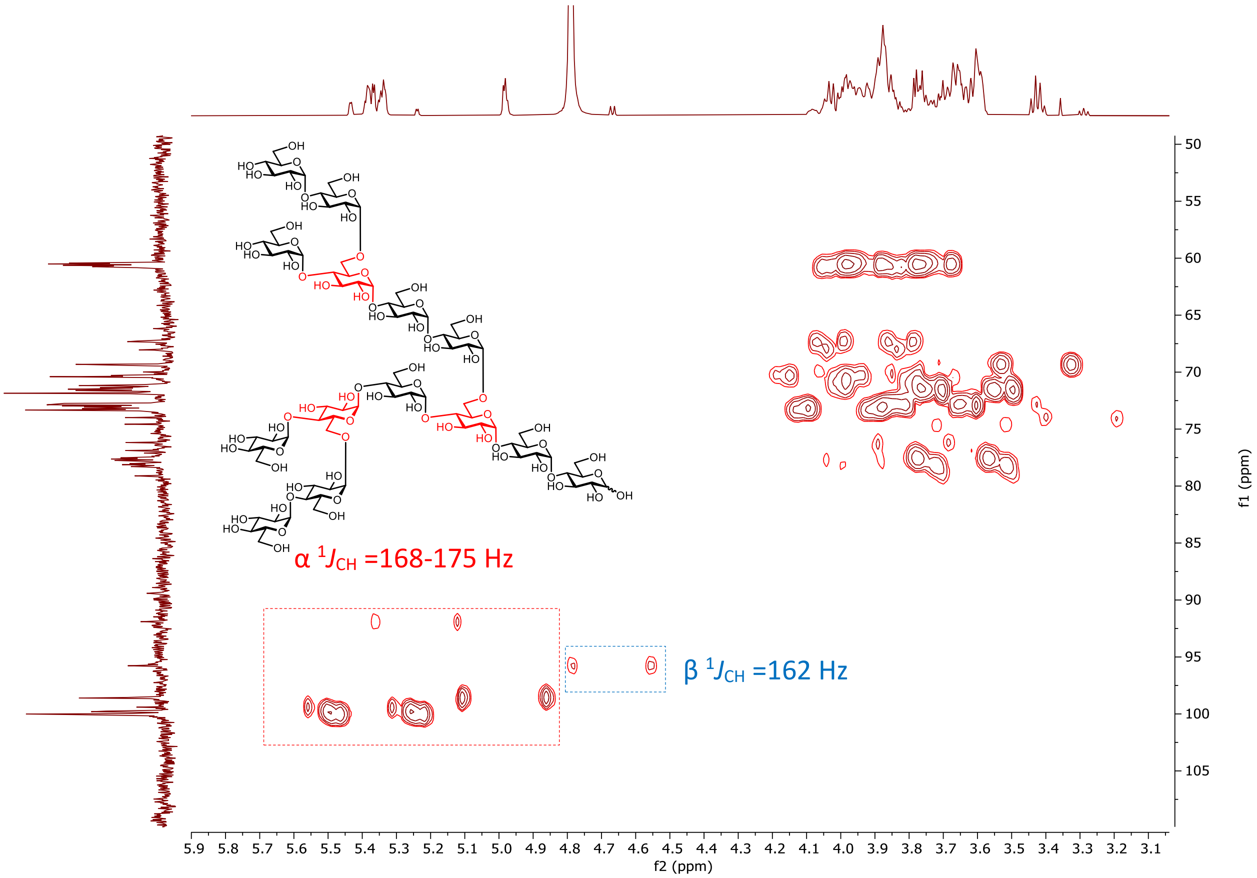

8. I_2-KI Stain Test

Iodine (52 mg, 0.20 mmol) was dissolved in EtOH (10 mL) to obtain the solution A. KI (520 mg, 3.1 mmol) was dissolved in water (10 mL) to make the solution B. Synthetic α-glucan, glucose or commercial starch (CAS: 9005-84-9)/glycogen (CAS: 9005-79-2) (0.5 mg) was dissolved in solution B (50 μL), and solution A (5 μL) was added. The mixture was shaken for 30 min before taking the picture.

9. References

1 Sureshan, K. M. *et al.* Contribution of phosphates and adenine to the potency of adenophostins at the IP3 receptor: synthesis of all possible bisphosphates of adenophostin A. *J. Med. Chem.* **55**, 1706-1720 (2012).

2 Hahm, H. S., Hurevich, M. & Seeberger, P. H. Automated assembly of oligosaccharides containing multiple cis-glycosidic linkages. *Nat. Commun.* **7**, 1-8 (2016).

3 Mannino, M. P. & Demchenko, A. V. Synthesis of β-glucosides with 3-*O*-picoloyl-protected glycosyl donors in the presence of excess triflic acid: a mechanistic study. *Chem. Eur. J.* **26**, 2927-2937 (2020).

4 Yu, Y. *et al.* Oligosaccharides Self-Assemble and Show Intrinsic Optical Properties. *J. Am. Chem. Soc.* **141**, 4833-4838 (2019).

5 Delbianco, M. *et al.* Well-defined oligo-and polysaccharides as ideal probes for structural studies. *J. Am. Chem. Soc.* **140**, 5421-5426 (2018).

6 Krock, L. *et al.* Streamlined access to conjugation-ready glycans by automated synthesis. *Chem. Sci.* **3**, 1617-1622 (2012).
